# Landscape Analysis of Existing Policies, Laws and Regulations on Antimicrobial Stewardship in 10 Low- and Middle-Income Countries

**DOI:** 10.3390/antibiotics15080770

**Published:** 2026-08-10

**Authors:** J. P. Waswa, Mohan P. Joshi, Reuben Kiggundu, Francis Kakooza, Dathan M. Byonanebye, Rodgers Rodriguez Ayebare, Kate Kikule, Hassan Kasujja, Niranjan Konduri, Emmanuel Nfor

**Affiliations:** 1 USAID Medicines, Technologies, and Pharmaceutical Services (MTaPS) Program, Management Sciences for Health (MSH), Kampala P.O. Box 920102, Uganda; rkiggundu@idi.co.ug (R.K.); hkasujja@ciu.ac.ug (H.K.); 2 Department of Global Health Security, Infectious Diseases Institute, College of Health Sciences, Makerere University, Kampala P.O. Box 22418, Uganda; fkakooza@idi.co.ug (F.K.); rayebare@idi.co.ug (R.R.A.); 3 USAID Medicines, Technologies, and Pharmaceutical Services (MTaPS) Program, Management Sciences for Health (MSH), Arlington, VA 22203, USA; mohan.p.joshi@gmail.com (M.P.J.); kkikule@msh.org (K.K.); yuniwo@gmail.com (E.N.); 4 Department of Community Health and Behavioural Sciences, School of Public Health, Makerere University, Kampala P.O. Box 7072, Uganda; 5 Department of Microbiology, School of Nursing and Midwifery, Clarke International University, Kampala P.O. Box 7782, Uganda; 6 Department of Medical Laboratory Technology, Habib Medical School, Islamic University in Uganda, Mbale P.O. Box 2555, Uganda

**Keywords:** antimicrobial stewardship, antimicrobial use, policies, regulation, laws, antimicrobial resistance, regulatory framework, global health security

## Abstract

**Background/Objectives:** Effective antimicrobial stewardship (AMS) is vital for containing antimicrobial resistance (AMR), yet evidence regarding existing regulatory frameworks in low- and middle-income countries (LMICs) remains scarce. This study conducted a landscape analysis of policies, laws, and regulations governing AMS across human and animal health sectors in ten LMICs to identify gaps and inform national action plans. **Methods:** Between October 2019 and September 2022, data were collected through desk reviews of national documents and a standardized, structured questionnaire administered to key national informants in ten countries: Bangladesh, Burkina Faso, Cameroon, Côte d’Ivoire, Mali, Mozambique, Nigeria, Senegal, Tanzania, and Uganda. **Results:** Findings were analyzed across five core regulatory domains. While all countries maintained national medicine policies and regulatory authorities, antimicrobial-specific measures were often insufficient, particularly in the animal health sector. Human health frameworks were generally more advanced; however, overarching regulations for marketing authorization, post-marketing surveillance, and pharmacovigilance frequently lacked the necessary specificity for antimicrobials. **Conclusions:** Addressing these gaps requires strengthening One Health legal frameworks, particularly through targeted animal health policies and multisectoral coordination. Enhanced investments in capacity-building and systematic enforcement are essential to optimize antimicrobial use and strengthen the AMR response.

## 1. Introduction

The overuse and misuse of antimicrobials in human and animal sectors drives antimicrobial resistance (AMR) [1]. Antimicrobial stewardship (AMS) aims to optimize antimicrobial use through effective systems and mechanisms regulating indiscriminate consumption and use, particularly in human and animal health [2], where the overuse and misuse are more pronounced [3]. This is achieved through balancing regulation and persuasion [4].

The inadequate implementation of AMS in low- and middle-income countries (LMICs), particularly in animal health and agriculture, hampers global AMR containment efforts [5,6]. Challenges include limited resources, weak regulatory systems, and inadequate surveillance systems for AMR and antimicrobial consumption (AMC) and use (AMU), requiring a comprehensive One Health approach to address them effectively [7]. Behavior change interventions, implemented through persuasion, have improved AMU in some countries with deficient, insufficient, or poorly enforced AMS regulations [8], but they rely heavily on individual motivations and face sustainability challenges [9]. Moreover, their impact on AMU in the community is limited. To ensure AMU, implementing and enforcing robust regulatory frameworks is crucial. These frameworks, core to national AMS programs, complement persuasion strategies like awareness and training by providing policy guidance for effective AMS strategy implementation across facilities and communities [2].

A functional regulatory system ensures public health protection through legal frameworks that guarantee safe, effective, and quality-assured medical products [10]. To sustain AMS initiatives, countries need regulatory frameworks on AMC/U, developed with stakeholder input and ratified into laws to ensure effective enforcement [11]. The World Health Organization’s (WHO) global action plan on AMR advocates for enforceable AMS regulations [12]. The WHO’s second Joint External Evaluation (JEE-2) (2018) and the 2019 International Health Regulations (IHR) benchmarks tools recommended national AMS regulatory frameworks for human and animal health to achieve sustainable AMS capacity [13,14]. Undertaking assessment of AMS regulatory framework completes benchmark 3.4 capacity level 2 action—“Undertake an assessment of stewardship policies and activities, including regulatory framework and supply chain management of antimicrobials, using a multisectoral approach”—on the 2019 WHO benchmarks for IHR tool [14]. Despite these recommendations, information on AMS regulatory frameworks, particularly in LMICs, is limited. A comprehensive analysis of existing AMS policies, laws, and regulations in human and animal health was undertaken in Bangladesh, Burkina Faso, Cameroon, Côte d’Ivoire, Mali, Mozambique, Nigeria, Senegal, Tanzania, and Uganda as part of programmatic operationalization of country NAPs-AMR. This paper presents the findings of this multicounty assessment and proposes recommendations for strengthening AMS regulatory frameworks in these countries.

## 2. Results

### 2.1. Overarching National Policies, Laws, and Regulations

The existing overarching national regulatory measures across 11 indicators for AMS in the human and animal health sectors at the time of the assessment are shown in Table 1 (See Box 1 for the specific legal and regulatory definitions used in this assessment). Laws establishing National Medicines Regulatory Authorities (NMRAs) were universally present in all ten study countries across both the human and animal health sectors, with all countries having enacted legislation to provide legal mandates for these authorities. These NMRAs oversee registration systems and ensure that medicines are safe, effective, and quality-assured before market introduction. While the internal structures of these authorities varied, with five countries utilizing composite NMRAs to handle both sectors (Bangladesh, Burkina Faso, Nigeria, Tanzania, and Uganda), the existence of an established legal basis for these bodies was a consistent finding across the entire assessment.

All ten countries had a national medicines policy for the human health sector, and nine countries (excluding Mozambique) had an equivalent policy for the animal health sector. However, only two countries (Bangladesh and Nigeria) had a dedicated antimicrobial policy for human health, while four countries (Bangladesh, Burkina Faso, Cameroon, and Côte d’Ivoire) had established these policies for the animal health sector.

Regulations for medicines registration were universally established across both human and animal health sectors in all ten countries, with every nation requiring authorization certificates for market access. While regulations for the registration of pharmaceutical premises and outlets were widely present for human health, the animal health sector exhibited gaps, such as in Burkina Faso, which lacked these specific registration requirements. While personnel registration is mandatory for the human health sector in all countries, nine countries have also established equivalent regulatory requirements for personnel dispensing veterinary medicines, with gaps identified only in Burkina Faso. While all NMRAs maintained tools for antimicrobial registration and quality control in human health, corresponding tools were absent in the animal health sectors in Côte d’Ivoire and Tanzania, highlighting that regulatory frameworks for human health are generally more advanced than those for animal health.

All study countries had enforcement mechanisms and conducted regular inspections to ensure compliance with regulations. However, these mechanisms were not operational in the animal health sectors in Bangladesh, Cameroon, and Nigeria. The majority of countries lacked systems for monitoring antimicrobial consumption and use with only four countries (Bangladesh, Mozambique, Nigeria, and Uganda) with such systems for both sectors, while in Senegal, monitoring was limited to the human health sector. While seven countries restricted antimicrobials to prescription-only status in the human health sector, with gaps identified in Cameroon, Mali, and Nigeria, nine countries had established such regulations for the animal health sector, with Mozambique being the only exception. There was universal presence of regulations for the inspection and monitoring of pharmaceutical outlets for compliance across both human and animal health sectors, though significant gaps exist in their operationalization. Specifically, despite the existence of these legal frameworks, the assessment found that enforcement mechanisms are missing or not operational in the animal health sectors of Bangladesh, Cameroon, and Nigeria.

### 2.2. Marketing Authorization, Licensing, and Inspection

**Figure 1:**
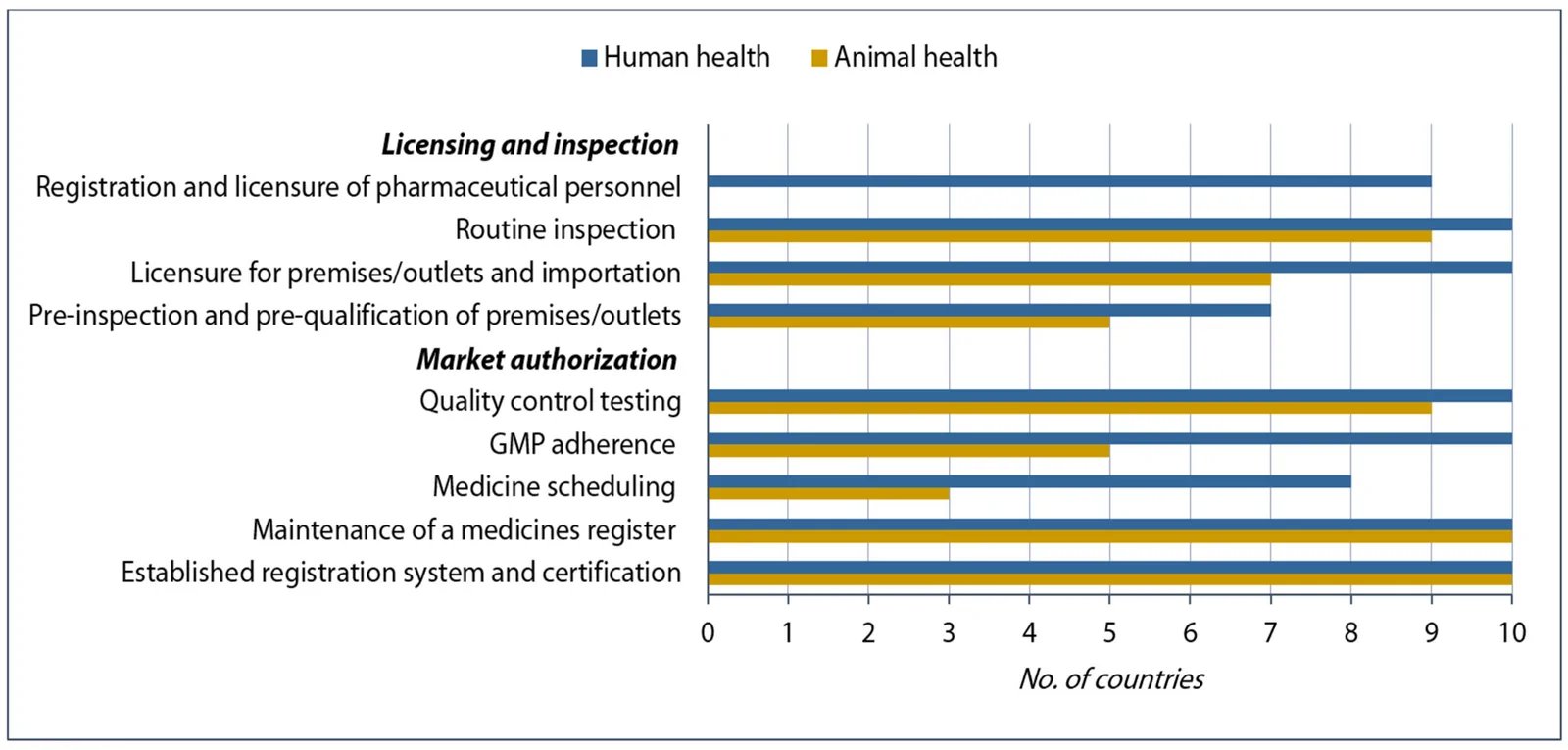
Existing regulations on marketing authorization, licensing, and inspection in the human health and animal health sectors in the ten study countries. The results show the status during the period of the assessment.

Each of the ten study countries implemented systems to ensure the safety, efficacy, and quality of medicines before market introduction, although to varying extents (Figure 1). All countries had a registration system for medicines and required authorization certificates for market access. NMRAs, established through government legislation, oversaw these systems, but their structures differed between the human and animal health sectors. Only six countries (Bangladesh, Burkina Faso, Côte d’Ivoire, Senegal, Tanzania, and Uganda) have established scheduling systems or prescription-only classifications for both human and veterinary medicines. While all countries assessed Good Manufacturing Practice (GMP) adherence in the human sector, five countries (Burkina Faso, Mali, Nigeria, Tanzania, and Uganda) applied the same standards to veterinary medicines. Quality control tests were conducted for all antimicrobials, though Burkina Faso did not extend this to veterinary antimicrobials.

All ten countries required inspections to pre-qualify pharmaceutical premises and outlets for human medicines. However, three countries (Bangladesh, Cameroon, and Nigeria) lacked similar mandatory pre-qualification inspections for the animal health sector. All ten countries mandated licenses for pharmaceutical premises and outlets in the human health sector. In the animal health sector, only Burkina Faso lacked these specific registration requirements, while the remaining nine countries maintained equivalent veterinary extensions. The NMRAs in all study countries conducted routine inspections, though Côte d’Ivoire did not conduct inspections for veterinary medicines. All countries required registration and licensure for pharmaceutical personnel in the human sector, but none had such regulations for veterinary medicine personnel.

### 2.3. Post-Marketing Surveillance (PMS) and Pharmacovigilance (PV)

**Figure 2:**
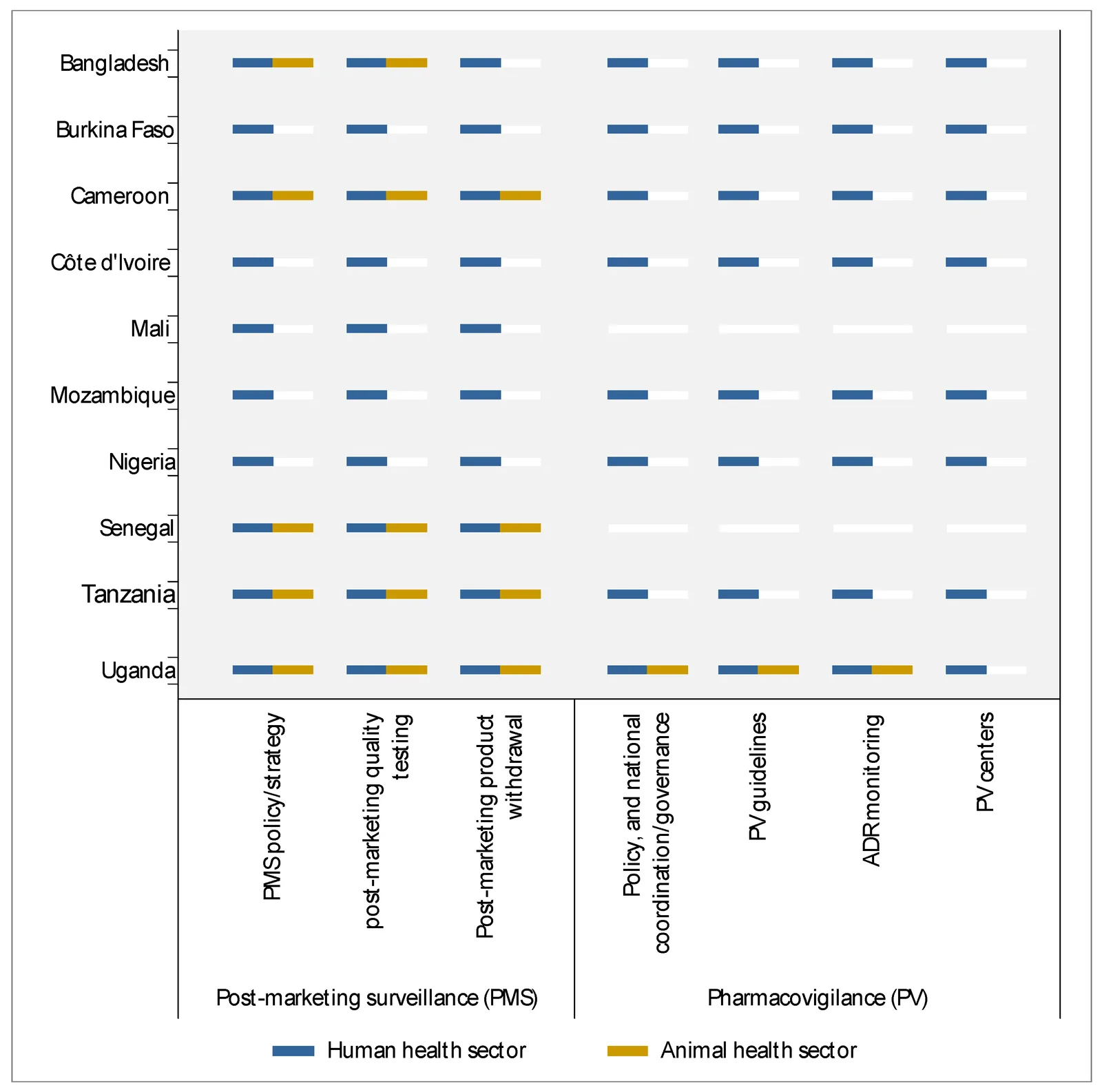
Existing policies/regulation on post-marketing surveillance and pharmacovigilance in the human health and animal health sectors in the ten study countries. The results for each country show the status during the period of the assessment. 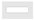 Means regulation was absent.

All study countries implemented a PMS policy or strategy for the human health sector, while only five countries, i.e., Bangladesh, Cameroon, Senegal, Tanzania, and Uganda, had a similar approach for the animal health sector (Figure 2). All study countries conducted post-marketing quality testing for human medicines, but only five- Bangladesh, Cameroon, Senegal, Tanzania, and Uganda—extended this to animal health medicines. Except for Nigeria, all countries conducted post-marketing product withdrawals for human medicines. However, despite NMRAs being required to implement this measure, only four countries, Cameroon, Senegal, Tanzania, and Uganda, applied it to animal health medicines.

The assessment also evaluated the availability of national systems for overseeing and coordinating PV activities, including PV guidelines, centers, and adverse drug reactions (ADR) monitoring. Eight countries, except Mali and Senegal, had established national governance/coordinating structures, implemented PV guidelines, and conducted routine ADR monitoring in the human health sector. Only Uganda had similar structures for the animal health sector. Additionally, all but Mali and Senegal had PV centers (both national and regional) for human health, but none had dedicated PV centers for animal health.

### 2.4. Supply Chain Management (SCM) Policies and Guidelines Relevant to Antimicrobials

**Figure 3:**
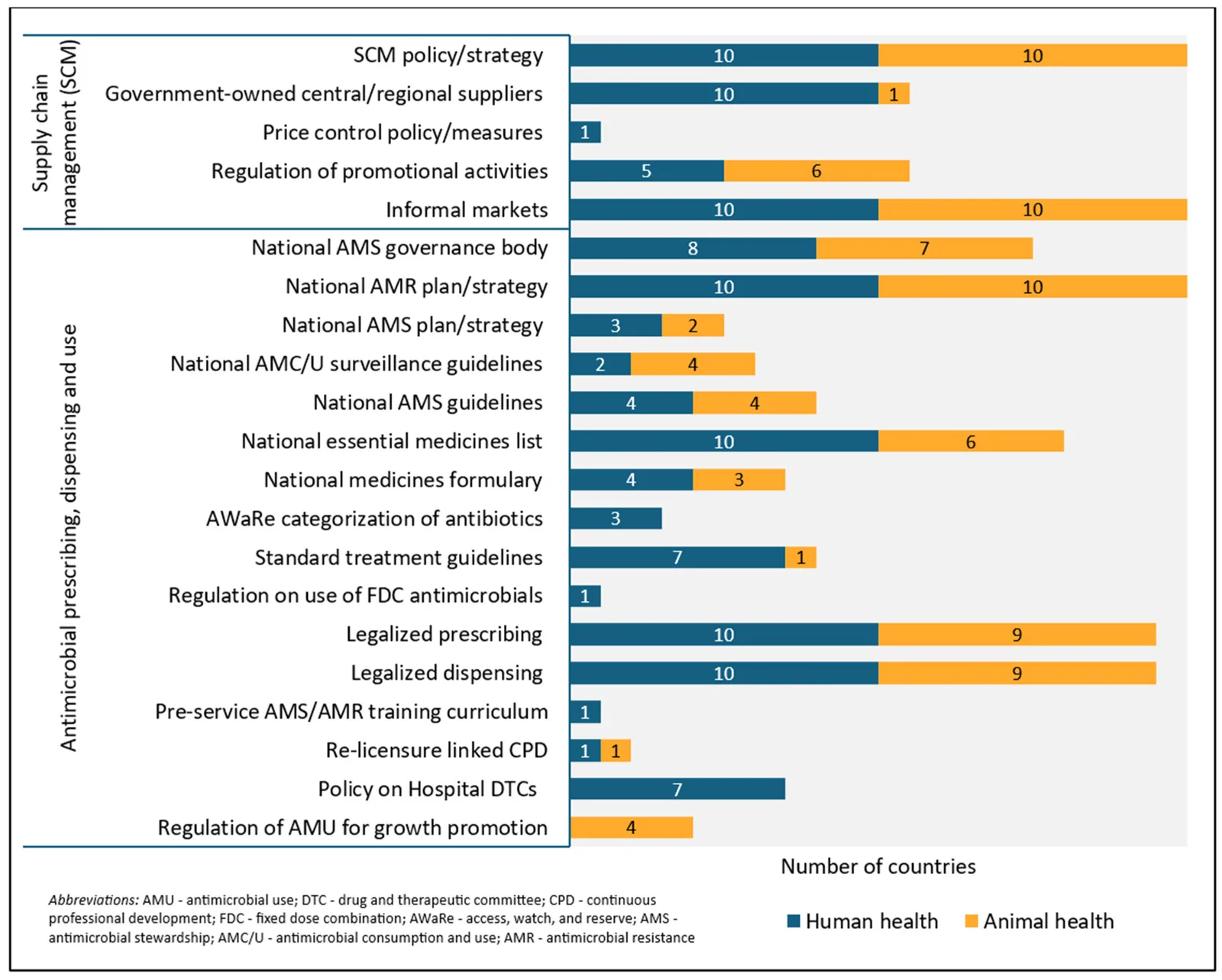
Existing regulation/guidelines on supply chain management and antimicrobial prescribing, dispensing, and use in human health and animal health sectors in the ten study countries. The results show the status during the period of the assessment.

All study countries implemented various actions, including SCM policies, government-owned suppliers, price control measures, and regulation of marketing activities, as shown in Figure 3. All study countries had government-controlled suppliers/stores for human medicines for use in public healthcare facilities, with Nigeria and Senegal having both central and regional suppliers. Nigeria was the only country with government-owned suppliers for animal health medicines. Medicines outside the public system were imported or locally procured and distributed by private wholesalers and retailers. Price control policies were largely absent, with Mozambique the only country to have one for human medicines. Five countries—Bangladesh, Burkina Faso, Mali, Nigeria, and Uganda had regulations on promotion, advertising, and marketing of human medicines, and six countries- Bangladesh, Burkina Faso, Mali, Mozambique, Nigeria, and Uganda had similar regulations for animal health. All study countries recognized the presence and risks of informal pharmaceutical markets. Detailed findings are available in Supplemental Material S4A.

### 2.5. Antimicrobial Prescribing, Dispensing, and Use

Figure 3 summarizes the regulatory measures governing antimicrobial prescribing, dispensing, and use in the human and animal health sectors across the ten study countries. Country-specific findings are provided in Supplementary Material S4B. All study countries had developed NAPs-AMR with One Health-oriented AMS objectives. Legalized prescribing and dispensing were mandated in the human health sector in all countries, with specific health providers authorized to prescribe and dispense accordingly. In both sectors, the sale and dispensing of certain antimicrobials were generally prohibited without a valid prescription, with exceptions clearly defined, but dispensing veterinary medicines did not always require licensed personnel. Mozambique is the only country that lacked these regulations for the animal health sector.

All study countries had national essential medicines lists (NEMLs) for human health, but only four, Bangladesh, Burkina Faso, Mozambique, and Nigeria, had developed national medicines formularies, lacking essential guidance for the selection and use of medicines, including antimicrobials. Despite being a critical WHO recommendation, only three countries, Bangladesh, Mali, and Tanzania, incorporated the access, watch, and reserve (AWaRe) classification of antibiotics into their NEMLs. For the animal health sector, only six countries—Bangladesh, Côte d’Ivoire, Mali, Mozambique, Nigeria, and Uganda had NEMLs, and just three- Bangladesh, Mozambique, and Nigeria developed national medicines formularies reflecting the slower progress in AMS implementation for animal health.

Most countries established national AMS governance bodies and structures for both human and animal health sectors (Figure 3), though their functionality varied across mandate, membership, plans, and funding. Dedicated AMS plans were present in only three countries: Bangladesh, Côte d’Ivoire, and Mali for human health, and two: Cameroon and Côte d’Ivoire for animal health. In the human health sector, all but three countries, Cameroon, Mozambique, and Senegal, implemented standard treatment guidelines (STGs), with only Burkina Faso, Côte d’Ivoire, Mali, Nigeria, Tanzania, and Uganda having universal STGs, and Bangladesh having dedicated STGs for common infectious diseases. Only Bangladesh and Tanzania incorporated the WHO AWaRe classification of antibiotics into their STGs. To ensure the effective implementation of STGs and other AMS initiatives in healthcare facilities, all but three countries—Burkina Faso, Nigeria, and Senegal had developed government policies mandating the establishment of functional drug and therapeutic committees (DTCs). There was a paucity of mechanisms for enforcing STG adherence across the board with only Tanzania using its National Health Insurance Fund to link reimbursement to compliance with STGs. The animal health sector showed even more significant gaps, with only Tanzania having developed STGs for the sector.

The assessment identified additional critical gaps in the existing regulatory measures for optimizing AMU in both sectors, including limited availability of national guidelines for AMS and AMC/U surveillance and lack of regulation on the use of fixed-dose combination (FDC) antimicrobials. Furthermore, most countries had not mandated comprehensive AMS/AMR curricula for pre-service training or linked continuous professional development (CPD) on AMS/AMR topics to health practitioners’ re-licensure.

Finally, regulatory frameworks governing the use of antimicrobials as growth promoters in livestock production were absent in the majority of the study countries, with only four countries (Bangladesh, Mozambique, Senegal, and Tanzania) had established formal regulations for this purpose in the animal health sector.

## 3. Discussion

To our knowledge, this is one of the first comprehensive multisectoral analyses of AMS policies, laws, and regulations across multiple LMICs. Our multisectoral assessment with partner countries complete WHO benchmark 3.4 capacity level 2 recommended action—“Undertake an assessment of stewardship policies and activities, including regulatory framework and supply chain management of antimicrobials, using a multisectoral approach”, facilitating countries in advancing to higher capacity levels and strengthening health security [14]. The multisectoral approach facilitated the adoption of best practices, identified existing gaps and opportunities across sectors, and promoted One Health.

The findings of our study mirror the gaps identified in WHO baseline JEEs, demonstrating that despite progress in AMS interventions across many LMICs [5,6], addressing these gaps and strengthening AMS regulatory frameworks remains a low priority. Countries should use the WHO Global Benchmarking Tool, specifically its indicators for legal provisions, regulations, and guidelines (Category 1), organization and governance (Category 2), and human, financial, and infrastructure resources (Category 8), to systematically strengthen regulatory and build NMRA capacity [10].

Our study revealed considerable variations in regulatory measures across countries and sectors in all ten countries. These differences may explain the varying progress in AMS and AMR containment interventions across sectors and LMICs [5,6].

Regulatory measures in the human health sector were generally more advanced than those in the animal health sector, reflecting the common prioritization of human health within One Health systems [5,8]. While this imbalance is understandable given the immediate imperative to protect human health, it may inadvertently constrain the effectiveness and sustainability of One Health approaches. Addressing these gaps requires deliberate actions to strengthen AMS regulations in the animal health sector, harmonization with the human health sector, as recommended by WHO and FAO [15,16], and enhance investments in One Health-focused capacity-building as part of NAP-AMR implementation.

Persuasive strategies like behavior change approaches have been effective in enhancing AMS practices in LMICs [17,18], but their sustainability is uncertain [17]. Enforced regulation, by providing appropriate incentives and disincentives, offers a more reliable strategy to sustain AMS initiatives, ensuring accountability and compliance [19]. While effective regulation can enforce practices, it is often slow and resource-intensive, and thus remain underprioritized in overburdened health systems [20]. These gaps, along with poor enforcement, lead to limited oversight of pharmaceutical systems and threatens AMS and AMR containment.

### 3.1. Overarching Policies, Laws and Regulations

Our assessment revealed that all study countries had developed and were actively implementing country-specific national medicines policies, consistent with findings from other studies conducted in LMICs [21,22]. Furthermore, our assessment identified the existence of dedicated national antimicrobial medicines policies in some countries, with some being sector-specific. These antimicrobial-specific medicine policies complement the broader national medicines policies and highlight the significant progress in addressing suboptimal AMU and AMR in LMICs [5,6].

The lack of regulations restricting antimicrobials to prescription-only status in the human health sectors of Cameroon, Mali, and Nigeria is concerning. Furthermore, while the animal health sector generally led in this indicator, the absence of such regulations in Mozambique presents a significant risk to AMS given the widespread use of antimicrobials in animal production [23]. While some progress has been made to monitor AMC at the national level, most countries lack systems to track the in-country sale of antimicrobials, especially for the animal health sector, mirroring findings from other studies [24], and underscoring a significant risk to AMS.

### 3.2. Marketing Authorization, Licensing, and Inspection

All study countries had systems for registering antimicrobials before market access, along with licensing requirements for their sale, prescription, and dispensing, although with significant disparities between sectors, undermining One Health efforts. While only licensed veterinary practitioners could prescribe antimicrobials in all the study countries, no such requirement for those dispensing them, reflecting gaps commonly observed in LMICs [8].

Inspections for pre-qualifying animal health pharmaceutical premises and outlets were less common than those for human health. Previous research highlights inadequate inspection capacity in LMICs, largely due to resource constraints [25,26], leading to inconsistent enforcement and limited oversight. This has resulted in a high prevalence of substandard and falsified antimicrobials in LMICs [27], compromising AMS and AMR containment.

### 3.3. Post-Marketing Surveillance and Pharmacovigilance

Regulatory measures to ensure quality, safe, and effective medicines, including antimicrobials, were uncommon in the animal health sector, posing risks to food safety and public health. Strengthening post-market withdrawal procedures for poor-quality antimicrobials in animal health is critical for AMS and AMR containment efforts [28]. Despite such mechanisms in the human health sector, product withdrawals in Africa remain lower than in other continents, highlighting enforcement gaps [29].

Pharmacovigilance is crucial for assessing the effectiveness and safety of antimicrobials [30]. In our study, PV systems were largely lacking in the animal health sector, consistent with findings from other studies [31,32], and emphasizing the need to address these discrepancies.

### 3.4. Supply Chain Management

Informal pharmaceutical markets were prevalent in both human and animal health sectors across all study countries, aligning with previous research findings [33], and reflecting gaps in regulatory enforcement. These gaps are increasing burden of substandard and falsified antimicrobials [27], as well as uncontrolled community use [34]. The inadequate regulation of pharmaceutical promotional activities in both sectors is threatening AMS [35,36]. Informal pharmaceutical markets arise when gaps in medicine regulation, enforcement capacity, supply chain oversight, and access to formal healthcare services allow unauthorized or unregulated actors to supply antimicrobials outside established regulatory systems [37]. Addressing this requires strengthening regulatory frameworks through comprehensive measures, including mandatory licensing, registration, and inspection of all pharmaceutical outlets; extending licensure and accountability requirements to personnel dispensing antimicrobials, especially within veterinary services; implementing regulatory incentives and disincentives, such as linking reimbursement mechanisms to adherence with STGs or requiring AMS training for professional renewal; and strengthening post-marketing surveillance systems to detect, withdraw, and prevent circulation of poor-quality or illegally supplied antimicrobials [38,39]. The absence of price control policies and measures in most study countries leaves the population vulnerable to high financial burdens, undermining efforts to achieve universal health coverage and Sustainable Development Goals [40].

### 3.5. Antimicrobial Prescribing, Dispensing and Use

Our study found widespread existence of various AMU-related regulatory measures across countries, consistent with findings from other studies [41]. Despite this, progress in optimizing AMU in both sectors remains limited in many LMICs [3], largely due to enforcement gaps [25], as seen during the COVID-19 pandemic [42]. While national multisectoral governance bodies and NAPs-AMR represent early progress in the One Health approach to AMR containment, many countries continue to lack dedicated AMS action plans and guidelines, as well as mandatory AMS/AMR training for health practitioners. These gaps leave practitioners and implementers without standardized local guidance and skills needed for effective AMS interventions.

The study revealed insufficient adoption of the WHO AWaRe categorization in NEMLs and STGs across countries, undermining AMS efforts [43]. This finding is particularly concerning, given the widespread use of Watch and Reserve antibiotics in these countries [44], and the need to adhere to the AWaRe categorization as one of the global approaches to combating AMR [43]. Structurally, many LMICs grapple with resource-constrained regulatory systems where updating national frameworks is a slow, resource-intensive process that often remains underprioritized amid competing health system demands [45,46]. Consequently, AWaRe categorization may not be fully incorporated into national essential medicines lists, treatment guidelines, prescribing policies, and routine monitoring systems. The enforcement gaps identified across the study countries suggest that, even where relevant policies exist, the administrative capacity to operationalize and monitor adherence to AWaRe-informed guidance remains limited. Without dedicated national AMS action plans and the institutional mechanisms needed to translate global frameworks into locally applicable policies and implementation tools, AWaRe risks remaining an underutilized resource in efforts to optimize antimicrobial use and contain AMR.

Furthermore, the widespread lack of regulation on the availability and use of non-recommended FDC antibiotics in both sectors is further exacerbating suboptimal AMU in the study countries, a persistent problem in LMICs [47]. Moreover, despite regulations relating to legalized prescribing and dispensing, dispensing antimicrobials without prescriptions and self-medication remains significantly prevalent in LMICs [34,48], further highlighting enforcement gaps. The regulatory gap concerning the use of antimicrobials for livestock growth promotion represents a significant challenge, given the widespread practice of antimicrobial use in livestock production across LMICs, with potential risks of accelerating AMR and contributing to the accumulation of antimicrobial residues in the food chain [49,50]. Lastly, the lack of mandated CPD, particularly on AMS and AMR topics, such as linkages to re-licensure of health practitioners in both sectors, has led to a general deficiency in knowledge, as well as suboptimal attitudes and practices related to AMS and AMU [51].

### 3.6. Study Limitations

A major limitation of our study was the lack of an assessment of the functionality, operationalization, implementation, and enforcement aspects of the regulatory frameworks, which limited our ability to fully understand the impact of these regulations. Additionally, incorporating a qualitative assessment of implementers’ and health practitioners’ attitudes toward these regulations would have further strengthened the study’s findings. A further limitation is that the findings presented in this study reflect the regulatory status only during the specific period of the assessment for each country. Because data collection was conducted between October 2019 and September 2022, the results represent a cross-sectional snapshot of the regulatory landscape at that time. Consequently, these findings may not account for recent policy updates, legislative amendments, or regulatory developments that have occurred in the participating countries since the conclusion of their respective assessment windows. However, the study had several strengths, including the large number of countries assessed, the use of a standardized tool, and the analysis of results and findings validated within each country.

### 3.7. Recommendations for Strengthening AMS Regulatory Frameworks

Our findings highlight that strengthening AMS regulatory frameworks in LMICs requires context-specific approaches due to demonstrated variations in regulatory environments, institutional capacities, and implementation challenges; therefore, progress will depend on identifying and adapting successful approaches within existing health system structures. For example, Bangladesh demonstrates the potential of integrated policy approaches within a composite regulatory authority, Tanzania provides an example of using financing mechanisms to promote compliance with treatment guidelines, and Uganda illustrates the value of establishing veterinary pharmacovigilance systems. These country experiences provide practical models that can be adapted to similar contexts s.

A key opportunity identified is the role of composite NMRAs, where human and veterinary medicines regulation are managed within a shared institutional framework. Countries with composite NMRAs, including Bangladesh, Burkina Faso, Nigeria, Tanzania, and Uganda, may be better positioned to advance One Health regulatory approaches by leveraging common administrative systems, technical expertise, and regulatory processes. These authorities should prioritize harmonizing regulatory standards across human and animal health sectors, including applying established approaches for medicine registration, quality assurance, inspection, and post-marketing surveillance to veterinary medicines. Rather than creating parallel regulatory systems, shared regulatory platforms may facilitate cross-sectoral learning and accelerate improvements in areas where animal health regulation remains less developed.

Priority actions should be tailored to country-specific regulatory gaps and implementation readiness (Table 2). Countries where policies and legislation exist but enforcement remains limited should focus on strengthening inspectorate capacity, licensing systems, and accountability mechanisms. Countries with gaps in veterinary medicines regulation should prioritize registration of veterinary pharmaceutical outlets, regulation of antimicrobial dispensing personnel, and development of sector-specific treatment guidelines. In addition, countries should explore sustainable incentive mechanisms that encourage compliance, including linking reimbursement systems to adherence with Standard Treatment Guidelines, integrating AMS competencies into professional accreditation and continuing education requirements, and strengthening post-marketing surveillance systems to ensure the quality and appropriate use of antimicrobials across sectors.

## 4. Materials and Methods

### 4.1. Setting

Between September 2018 and February 2025, the US Agency for International Development (USAID)-funded Medicines, Technologies, and Pharmaceutical Services program (henceforth referred to as “the program”), led by Management Sciences for Health (MSH), supported 13 countries in combating AMR and strengthening health security capacities [52]. The program helped implement actions from countries’ national action plans on AMR (NAPs-AMR) and WHO’s IHR benchmarks (2019), focusing on multisectoral coordination for AMR, infection prevention and control, and AMS.

Although the program operated in 13 countries, the study was limited to 10 countries (Bangladesh, Burkina Faso, Cameroon, Côte d’Ivoire, Mali, Mozambique, Nigeria, Senegal, Tanzania, and Uganda) because the remaining three countries had inaccessible policy and regulatory documentation, incomplete stakeholder validation processes, or country-specific contextual and operational constraints that limited the collection and verification of data within the study period. Baseline JEE assessments in the 10 study countries revealed significant gaps in AMS regulation, particularly related to weak laws and inadequate enforcement (detailed findings are provided in the Supplementary Material S1). The data for this study were generated through original assessment activities led by the program’s technical team (authors) in collaboration with country teams and national stakeholders. While baseline JEEs informed the initial identification of regulatory gaps, the findings presented here are the result of primary data collection conducted specifically to support the operationalization of NAPs-AMR.

### 4.2. Assessments

Assessments were carried out between October 2019 and September 2022 to gather data on countries’ policies, laws and regulations related to AMS to inform countries’ decision-making processes and actions for NAP-AMR implementation and dedicated national AMS strategies and action plans. These assessments focused on identifying the existence and details of AMS-related policies, laws and regulations, and in general did not include the evaluation of enforcement levels and practices.

### 4.3. Assessment Tool

The assessment tool, based on the 2017 coalition-building guide on AMR developed by MSH, was used to collect data on country’s policies, laws, regulations, processes, and systems associated with AMS in human and animal health sectors [53]. The structured questionnaire, designed with adaptable question sets for country-specific and multisectoral application, consisted of four sections and a total of 88 indicators—37 main questions and 51 sub-questions. The sections included: (1) national regulations, legislation, and policies; (2) medicine use and AMR-related policies/regulations; (3) supply management policies; and (4) appropriate use by prescribers, dispensers, and patients (see Supplementary Materials S2 for the detailed tool). The tool included factual questions requiring dichotomous responses (yes/no) and open questions requiring detailed responses. The tool’s definitions of key terms (Box 1) were based on standard and global literature. In all countries except Mozambique, the MSH tool was used for data collection. In Mozambique, the tool developed by the Food and Agriculture Organization (FAO) was used to collect data in the animal health sector [54]. Despite structural differences, the FAO tool provided comparable data, which was collated with MSH tool findings for analysis and comparison.

Box 1 **Definitions of key terms in the assessment tool.**

  Legislation: The first state of the legislative process, in which laws are passed by the legislative body of government with regard to a subject matter, e.g., control of pharmaceuticals.

  Laws define the roles, rights, and obligations of all parties involved in the subject matter in general terms [55].

  Regulation: The second stage of the legislative process (the first stage being legislation) in which regulations are specifically designed to provide the legal machinery to achieve the administrative and technical goals of legislation [55].

  Policy: Principles, rules, guidelines, or actions adopted by governments or institutions. In public health, policy development involves creating and implementing laws, regulations, or practices that drive system development, organizational change, and individual behavior to improve health outcomes [56].

  Guidelines: Non-statutory advisory publications intended to assist those parties affected by legislation to interpret requirements for ease in implementation [55].

  Regulatory framework: A regulatory framework comprises laws, regulations, guidelines, and instruments used by a government to oversee an industry or sector. It establishes operational standards, compliance requirements, and oversight protocols for individuals and institutions within the sector [57].

### 4.4. Data Collection

In each country, the program country teams conducted data collection through desk reviews and key informant interviews, using the assessment tool. Desk reviews gathered existing information on policies, laws and regulations, while interviews provided context-specific insights. Desk reviews analyzed public literature, reports, and official documents. Preliminary content analyses identified information gaps, new stakeholders, and follow-up plans.

Key informants were purposively selected to ensure representation of stakeholders directly involved in AMR containment and pharmaceutical systems within each country. Selection was based on technical expertise or decision-making responsibility in AMS, medicines regulation, and related policy areas. Informants represented key institutions across the human and animal health sectors, including Ministries of Health, Ministries responsible for agriculture, livestock, and fisheries, National Medicines Regulatory Authorities, academic and research institutions, national One Health platforms and AMR coordinating committees, as well as professional bodies and private sector organizations, including national veterinary associations and pharmaceutical wholesalers. Key informant interviews clarified evidence, addressed information gaps, and validated findings. In total, seven countries produced combined reports for human and animal health, while three countries developed separate sector reports.

To achieve cross-sectoral consensus and ensure the accuracy of the findings, a multi-stage validation process was employed. Following the initial desk reviews and interviews, preliminary findings were synthesized into draft country reports. These reports were then subjected to formal validation by national stakeholders through multidisciplinary workshops. This process allowed representatives from both the human and animal health sectors to verify the data, resolve any conflicting information between sectors, and reach a collective agreement on the status of the regulatory indicators. Detailed country reports are included in Supplementary Materials S3).

### 4.5. Data Analysis

The multi-country cross-sectional study analyzed validated country reports in detail in a structured way using content analysis to extract information on the existing policies, laws and regulations supporting AMS in the ten countries. The analysis covered five analytical areas reflecting the tool sections: (1) national policies, laws and regulations; (2) marketing authorization, licensing establishments and regulatory inspections; (3) post-marketing surveillance and pharmacovigilance; (4) supply chain management policies and guidelines relevant for antimicrobials; and (5) antimicrobial prescribing, dispensing and use. Data were managed and analyzed using Microsoft 365 Excel (Microsoft Corporation, Redmond, WA, USA). 

The Mozambique FAO tool data was harmonized with the study framework through a structured technical mapping process that aligned thematic domains, fundamental indicators, and data definitions across the two approaches. The FAO assessment of sector-specific legal frameworks, including medicines regulation, authorization and licensing, supply chain management, and prescribing and dispensing requirements, corresponded directly with the core domains assessed using the MSH tool. Specific FAO findings were mapped to the study’s fundamental indicators, including regulatory authority mandates, registration of pharmaceutical premises, and prescription requirements for antimicrobials. Because both approaches assessed the presence or absence of defined legal mandates, the harmonization process focused on verifying that the legal instruments identified by the FAO analysis met the MSH framework’s definitions of laws and regulations.

## 5. Conclusions

Our study underscores the urgent need to strengthen AMS policies, laws, and regulations in both the human and animal health sectors, with a particular focus on addressing gaps in the animal health sector as part of One Health and NAP-AMR implementation. Insufficient antimicrobial-specific regulations needs to be addressed to enhance AMU and effectively contribute to AMR containment. Enhanced coordination between human and animal health sectors, is essential for achieving the needed improvements. Systematic approaches to strengthen enforcement, alongside compliance monitoring, mandated training, and awareness raising are essential for maximizing the impact of AMS policies, laws, and regulations.

## Figures and Tables

**Table 1:** Overarching national policies, laws and regulations on antimicrobial stewardship in the ten study countries *.

Country ^¥^ (Human/Animal)	Assessment Date (from/to)	Laws Establishing NMRAs	National Medicines Policy	Antimicrobial Medicines Policy	Regulation for Medicines Registration	Regulation for Registration of Pharmaceutical Premises/Outlets	Regulation for Registration of Pharmaceutical Personnel	Tools for Antimicrobial Registration, Quality Control and Assurance	Inspection and Monitoring of Pharmaceutical Outlets for Compliance	System for Monitoring Antimicrobial Sales	Regulation for Import, Export, Production, Distribution, Storage and Use of Antimicrobials and Ingredients	Regulation for Prescription-Only Medicines Status
Bangladesh	Mar 2022/Jul 2022	+/+	+/+	+/+	+/+	+/+	+/+	+/+	+/-	+/+	+/+	+/+
Burkina Faso	Mar 2020/Mar 2020	+/+	+/+	-/+	+/+	+/-	-/+	+/+	+/+	-/-	+/+	+/+
Cameroon	Aug 2020/Aug 2020	+/+	+/+	-/+	+/+	+/+	+/+	+/+	+/-	-/-	+/+	-/+
Côte d’Ivoire	Jun 2020/Jun 2020	+/+	+/+	-/+	+/+	+/+	+/+	+/-	+/+	-/-	+/+	+/+
Mali	Mar 2020/Mar 2020	+/+	+/+	-/-	+/+	+/+	+/+	+/+	+/+	-/-	+/+	-/+
Mozambique	Aug 2020/Nov 2020	+/+	+/-	-/-	+/+	+/+	+/+	+/+	+/+	+/+	+/+	+/-
Nigeria	Oct 2021/Dec 2021	+/+	+/+	+/-	+/+	+/+	+/+	+/+	+/-	+/+	+/+	-/+
Senegal	Aug 2020/Aug 2020	+/+	+/+	-/-	+/+	+/+	+/+	+/+	+/+	+/-	+/+	+/+
Tanzania	Sep 2022/Sep 2022	+/+	+/+	-/-	+/+	+/+	+/+	+/-	+/+	-/-	+/+	+/+
Uganda	Oct 2021/Oct 2021	+/+	+/+	-/-	+/+	+/+	+/+	+/+	+/+	+/+	+/+	+/+

+ means policy/regulation/guidance present; - means no policy/regulation/guidance. Abbreviation: NMRAs—national medicines regulatory authorities; * The results for each country show the status during the period of the assessment; ^¥^ Please see individual country reports for more details (Supplementary Material S3).

**Table 2:** Country-specific recommendations for strengthening AMS regulatory frameworks.

Country	Priority Recommendations
Bangladesh	Operationalize dormant animal health enforcement mechanisms. Expand current multisectoral antimicrobial policy to include community-level AMU monitoring. Mandate AWaRe-based prescribing in veterinary curricula. Implement post-marketing quality testing for all veterinary drugs.
Burkina Faso	Enact regulations for registering animal health pharmaceutical premises. Expand quality control testing to include veterinary antimicrobials. Develop sector-specific STGs for animal health. Link DTCs to sub-national hospitals.
Cameroon	Operationalize existing animal health enforcement and inspection mechanisms. Develop and implement STGs for the human health sector. Establish national AMS governance bodies with dedicated funding. Create a national PV coordinating center for human health.
Côte d’Ivoire	Institutionalize routine inspections for veterinary medicine outlets. Establish quality control and registration tools for the animal sector. Implement a national essential medicines list specifically for livestock. Pilot AMC monitoring in major urban centers.
Mali	Establish national PV guidelines and centers for the human sector. Enact legal mandates for human pharmaceutical premises registration. Develop AWaRe-aligned STGs for common human infectious diseases. Expand PMS strategies to the veterinary sector.
Mozambique	Ratify the national medicines policy for the animal health sector. Implement a prescription-only status for veterinary medicines. Develop national medicines formularies for both sectors. Establish a national system for adverse drug reaction monitoring.
Nigeria	Establish legal mandates for registration of personnel dispensing veterinary medicines. Activate non-functional animal health enforcement mechanisms. Harmonize regional and central supplier regulations for veterinary products. Develop a dedicated national veterinary antimicrobial policy.
Senegal	Harmonize pharmaceutical personnel registration requirements across both sectors. Mandate government policies for functional hospital DTCs. Expand post-marketing quality testing to the animal health sector. Pilot price control measures to reduce informal market reliance.
Tanzania	Pilot the national health insurance fund reimbursement model for veterinary STG compliance. Strengthen animal health PMS guidelines. Integrate AWaRe into all sub-national pharmacy monitoring tools. Formalize cross-border regulatory collaboration with East African partners.
Uganda	Scale the established veterinary PV coordinating structure to full national operationalization. Enforce marketing and advertising regulations in the animal sector. Link veterinary practitioner re-licensure to mandatory AMS continuing professional development. Digitalize AMC/AMU tracking for both sectors.

## Data Availability

The original data presented in the study has been provided in the Supplementary Materials.

## Supplementary Materials

The following supporting information can be downloaded at: https://www.mdpi.com/article/10.3390/antibiotics15080770/s1; S1: Baseline AMS_regulation status per country JEEs; S2: Assessment tool; S3: Country Reports; S4: SCM regulations. References [58,59,60,61,62,63,64,65,66,67,68] are cited in the Supplementary Materials.
