# Supplementary material for: Landscape Analysis of Existing Policies, Laws and Regulations on Antimicrobial Stewardship in 10 Low- and Middle-Income Countries
Source: Antibiotics (Basel). 2026 Aug 10;15(8):770. doi: 10.3390/antibiotics15080770 (PMC13510074; doi:10.3390/antibiotics15080770)
Supplement: Supplementary file 1 [file antibiotics-15-00770-s001.zip › S3 Country Reports/S3A-1_Bangladesh Human health).pdf]

USAID MEDICINES, TECHNOLOGIES, AND PHARMACEUTICAL SERVICES (MTaPS) PROGRAM

Improved Access. Improved Services. Better Health Outcomes.

ASSESSMENT REPORT OF NATIONAL LEVEL ANTIMICROBIAL STEWARDSHIP POLICIES, REGULATIONS, LEGISLATIONS AND PRACTICES CONDUCTED IN HUMAN HEALTH SECTORS

N- H- Salsabil

Dr. Najia Amin Salsabil
2022.11.08
23:28:49 +06'00'

USAID
FROM THE AMERICAN PEOPLE

This document is made possible by the generous support of the American people through the US Agency for International Development (USAID) contract no. 7200AA18C00074. The contents are the responsibility of Management Sciences for Health and do not necessarily reflect the views of USAID or the United States Government

About the USAID MTaPS Program

The USAID Medicines, Technologies, and Pharmaceutical Services (MTaPS) Program enables low- and middle-income countries to strengthen their pharmaceutical systems, which is pivotal to higher-performing health systems. MTaPS focuses on improving access to essential medical products and related services and on the appropriate use of medicines to ensure better health outcomes for all populations. The program brings expertise honed over decades of seminal pharmaceutical systems experience across more than 40 countries. The MTaPS approach builds sustainable gains in countries by including all actors in health care—government, civil society, the private sector, and academia. The program is implemented by a consortium of global and local partners and led by Management Sciences for Health (MSH), a global health nonprofit.

The MTaPS Consortium

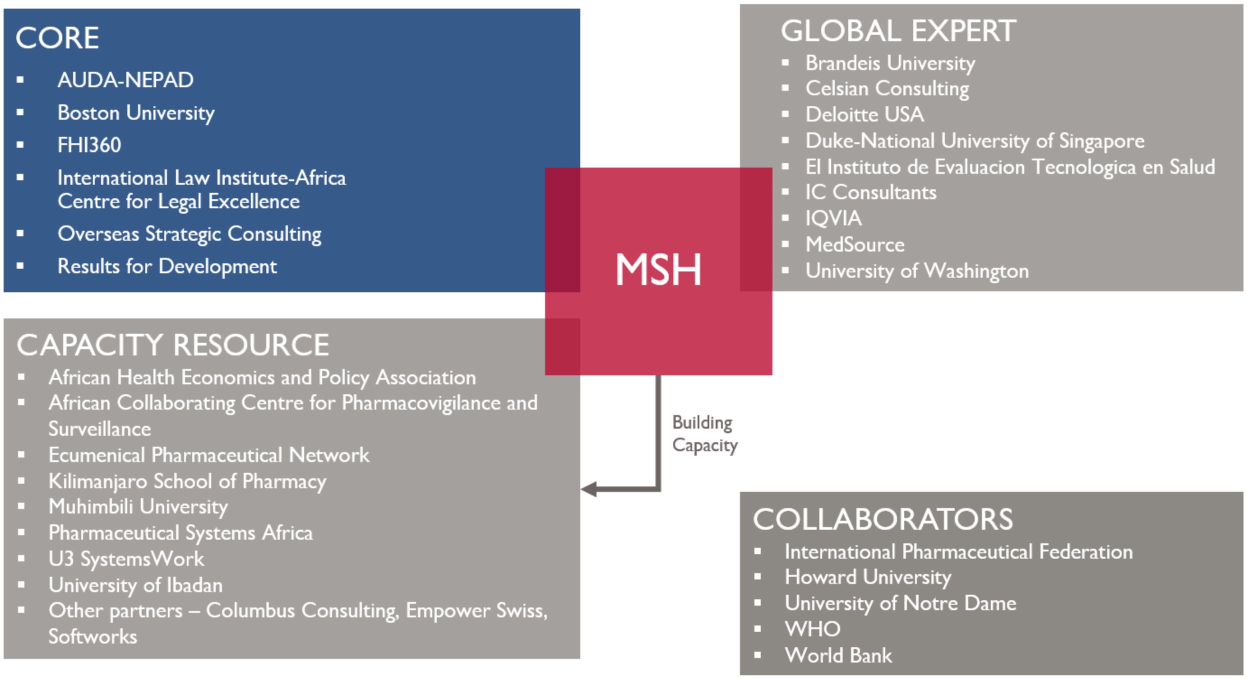

This document is submitted by:

Dr. Najia Amin Salsabil

Consultant, USAID MTaPS Program

najia.amin.salsabil@gmail.com

ACRONYMS AND ABBREVIATIONS

ADE	Adverse Drug Event
ADRAC	Adverse Drug Reaction Advisory Committee
AMC	Antimicrobial Consumption
AMR	Antimicrobial Resistance
AMS	Antimicrobial Stewardship
AMU	Antimicrobial Usage
ARC	Antimicrobial Resistance Containment
ASP	Antimicrobial Stewardship Program
AST	Antimicrobial Susceptibility Testing
AWaRe	Access, Watch, Reserve
BCC	Behavior Change Communication
BDS	Bachelor of Dental Surgery
BFRI	Bangladesh Fisheries Research Institute
BLRI	Bangladesh Livestock Research Institute
BP	British Pharmacopeia
CAPTURA	Capturing Data on Antimicrobial Resistance Patterns and Trends in Use in Regions of Asia
CDC	Communicable Disease Control
CHCP	Community Health Care Provider
CMSD	Central Medical Stores Depot
COPP	Certificate of Pharmaceutical Product
COVID	Corona Virus Disease
CPTU	Central Procurement Technical Unit
CRO	Clinical Research Organization
CVH	Central Veterinary Hospital
DAR	Drug Administration Registration

DCC	Drug Control Committee
DD	Deputy Director
DDD	Defined Daily Dose
DGDA	Directorate General of Drug Administration
DGHS	Directorate General of Health Services
DGME	Directorate General of Medical Education
DLS	Department of Livestock Services
DoF	Department of Fisheries
DVH	District Veterinary Hospital
EDCL	Essential Drug Company Limited
EDL	Essential Drug List
EQA	External Quality Assessment
FAO	Food and Agriculture Organization (FAO)
FDA	Food and Drug Administration
GHSA	Global Health Security Agenda
GLASS	Global Antimicrobial Resistance and Use Surveillance System
GMP	Good Manufacturing Practice
HAI	Healthcare Associated Infection
HIV	Human Immunodeficiency Virus
ICDDRB	International Centre for Diarrheal Disease Research, Bangladesh
IEDCR	Institute of Epidemiology, Disease Control and Research
IFPMA	International Federation of Pharmaceutical Manufacturers & Associations
IHR	International Health Regulation
INN	International Nonproprietary Names
IPC	Infection Prevention and Control
IVI	International Vaccine Institute

JEE	Joint External Evaluation
LMICs	Low- and Middle-Income Country
M&E	Monitoring and Evaluation
MA	Marketing Authorization
MDR	Multi-Drug Resistance
MO	Medical Officer
MoE	Ministry of Education
MoFL	Ministry of Fisheries and Livestock
MoHFW	Ministry of Health and Family Welfare
MSH	Management Sciences for Health
MTaPS	Medicines, Technologies, and Pharmaceutical Services
NAP	National Action Plan
NGO	Non-Government Organization
NRA	National Regulatory Authority
NTC	National Technical Committee
OPs	Operational Plans
OTC	Over the Counter
PHC	Primary Health Care
PV	Pharmacovigilance
QI	Quality Improvement
QIS	Quality Improvement Secretariate
QR code	Quick Response Code
RB-PMS	Risk-Based Post-Marketing Surveillance
RFQ	Request for Quotation
RMO	Resident Medical Officer
SDG	Sustainable Development Goal

SF	Substandard and Falsified
SOP	Standard Operating Procedure
STGs	Standard Treatment Guidelines
TB	Tuberculosis
TOR	Terms of Reference
TOT	Training of Trainers
TWG	Technical Working Group
UH&FPO	Upazilla Health and Family Planning Officer
UHC	Universal Health Coverage
USAID	United States Agency for International Development
USP	United States Pharmacopeia
UVH	Upazilla Veterinary Hospital
VS	Veterinary Schools
WAAW	World Antibiotic Awareness Week
WASH	Water, Sanitation and Hygiene
WHO	World Health Organization
WHO-UMC	WHO-Upsala Monitoring Centre
XDR	Extensive Drug Resistance

GLOSSARY

Antibiotic: The term "antibiotic" refers to an antimicrobial agent with the ability to kill or inhibit bacterial growth.

Antimicrobial: An agent or substance derived from any source (microorganisms, plants, animals, synthetic or semi-synthetic) that acts against any type of microorganism, such as bacteria (antibacterial), mycobacteria (anti-mycobacterial), fungi (antifungal), parasite (anti-parasitic) and viruses (antiviral). All antibiotics are antimicrobials, but not all antimicrobials are antibiotics.

Antimicrobial Resistance (AMR): Microorganisms such as bacteria, fungi, viruses and parasites change when exposed to antimicrobial drugs such as antibiotics (= antibacterial), antifungals, antivirals, antimalarials and anthelmintics. As a result, the medicines become ineffective.

Antimicrobial Stewardship (AMS): A coherent set of actions which promote the responsible use of antimicrobials. This definition can be applied to actions at the individual level as well as the national and global level, and across human health, animal health and the environment.

Antimicrobial Stewardship Program (AMS program): An organizational or system-wide health-care strategy to promote appropriate use of antimicrobials through the implementation of evidence-based interventions.

Legislations: The first stage of the legislative process, in which laws are passed by the legislative body of government with regard to a subject matter, e.g., control of pharmaceuticals. Laws define the roles, rights, and obligations of all parties involved in the subject matter in general terms.

Regulations: The second stage of the legislative process (the first stage being legislation) in which regulations are specifically designed to provide the legal machinery to achieve the administrative and technical goals of legislation.

Guidelines: Non-statutory advisory publications intended to assist those parties affected by legislation to interpret requirements.

Policy: Policy is a law, regulation, procedure, administrative action, incentive, or voluntary practice of governments and other institutions. Within the context of public health, policy development includes the advancement and implementation of public health law, regulations, or voluntary practices that influence systems development, organizational change, and individual behavior to promote improvements in health.

TABLE OF CONTENTS

Acronyms and Abbreviations iii

Glossary vii

Project Summary 1

Executive Summary 2

Introduction 4

Background 5

Objectives of the Assessment 6

Methodology 7

Summary of Data Collection and Analysis 10
- Essential national core elements for AMS programming 10
  - I. National Plan and Strategies: 10
  - II. Regulation and Guidelines: 13
  - III. Awareness, Training and Education: 17
  - IV. Supporting Technologies and Data: 19
- Other Elements 20
  - I. Supply Management Policies: 20
  - II. Reports/Studies on the Level of Appropriate Use by Prescribers, Dispensers and Patients . 23

Recommendations 25

Limitations 28

Stakeholders' Workshop 28

Conclusion 32

Appendix1: GO Letter (Assessment) 33

Appendix 2: Rapid Assessment Tool (used for both desktop review and stakeholder interviews) 34

Appendix 03: GO Letter (Workshop) 70

Appendix 04: Schedule of Workshop 71

Appendix 05: Participants List (Workshop)* 72

Appendix 06: Presentation on the Assessment– Human Health 74

Appendix 07: Presentation on the Assessment– Animal Health 74

Appendix 08: Presentation on the Overview of AMS Guidelines for Human Health 74

Appendix 09: National Multisectoral AMS Plan (Human Health and Animal Health); 2022-2026 75

PROJECT SUMMARY

**Program Name:**		USAID Medicines, Technologies, and Pharmaceutical Services (MTaPS) Program
**Activity Start Date and End Date:**		September 20, 2018–September 19, 2023
**Name of Prime Implementing Partner:**		Management Sciences for Health
**Contract Number:**		7200AA18C00074
**MTaPS Partners**	**Core Partners**	Boston University, FHI 360, Overseas Strategic Consulting, Results for Development, International Law Institute-Africa Centre for Legal Excellence, NEPAD
	**Global Expert Partners**	Brandeis University, Deloitte USA, Duke-National University of Singapore, El Instituto de Evaluacion Technologica en Salud, IC Consultants, Imperial Health Sciences, MedSource, QuintilesIMS, University of Washington
	**Capacity Resource Partners**	African Health Economics and Policy Association, Ecumenical Pharmaceutical Network, U3 SystemsWork, University of Ibadan, University of Ghana's World Health Organizations (WHO) Pharmacovigilance Collaborating Center, Kilimanjaro School of Pharmacy, Muhimbili University, Pharmaceutical Systems Africa
	**Collaborators**	International Pharmaceutical Federation, Howard University, University of Notre Dame, WHO, World Bank

EXECUTIVE SUMMARY

Antimicrobial resistance (AMR) is a global health and development threat. Antimicrobial stewardship (AMS) is a technical capacity area for a country to contain AMR and it includes a coherent set of actions which promote the responsible use of antimicrobials. These actions applied to individual level as well as the national and global level and across human health, animal health and the environment. In alignment with the global and WHO initiatives, the recently updated NAP-ARC (2021-2026) explicitly states AMS is a priority area for the Ministry of Health and Family Welfare (MoH&FW), Bangladesh. The objectives of this assessment of national AMS policies, regulations, legislation and practices conducted in human health sector are –

1. To gather data on existing national AMS policies, regulations, legislation and practices conducted in human health sector.
2. To identify the gaps and challenges.
3. To produce a set of recommendations to strengthen AMS governance.

Although the levels of viral, fungal and parasite resistance to antimicrobials are also increasing, the focus of this assessment was bacterial resistance to antibiotics.

This was a rapid assessment using a national protocol and checklist covering AMS core elements from WHO AMS practical toolkit. The methodology and the rapid assessment tool (Appendix-2) were contextualized after discussing with CDC, DGHS. Before starting the formal assessment process, a GO letter (Appendix-1) was issued from CDC, DGHS. The major stakeholders for this assessment were CDC, DGHS; IEDCR, DGDA and QIS.

The AMS related data were collected under the following four major areas -

1. National plan and strategies
2. Regulation and guidelines
3. Awareness, education and training
4. Supporting technologies and data

There are 20 core elements under the above broad areas that were assessed and the findings were –

Status of WHO National Core Elements	No. of core elements (%) (Total 20)
Fully implemented	09 (45%)
Partially implemented/initiated	08 (40%)
Not implemented	03 (15%)

After this exercise, the 'partially implemented' and 'not implemented' core elements were prioritized based on the country context and the recommendations were made as short term and long-term interventions –

1. National Plan and Strategies

Short Term

i. Establish a TWG or AMS subcommittee (National Multisectoral AMS Committee) with clear TOR which is linked to AMC and AMR surveillance and IPC TWG
ii. Develop an achievable national implementation plan for AMS
iii. Allocate dedicated funding for AMS activities in all relevant OPs
iv. Develop national antibiotic policy

Long Term

i. Establish Monitoring and Evaluation mechanism emphasizing capacity building of concerned DGHS officials both at the national and sub-national level and alignment of the indicators with DHIS-II
ii. Establish and Strengthen IPC and WASH program
iii. Establish integrated AMS activities within the action plan of other programs such as IPC, WASH, TB, malaria, HIV, UHC and Primary Health Care (PHC)

2. Regulation and Guidelines

Short Term

i. Update the Essential Drug List (EDL) by incorporating WHO EML AWaRe and maintaining coherence between EDL and developed STGs
ii. Ensure STGs are followed at facility level and develop specific revision plan for the developed STGs
iii. Monitor health care providers who are authorized to prescribe limited antimicrobials in specific clinical condition (eg. CHCP, midwives etc.)
iv. Strengthen monitoring of 'Model Pharmacy' and 'Pharmacy Shop'
v. Strengthen capacity building measures on pharmacovigilance reporting
vi. Update 'Code of Pharmaceutical Marketing Practices' with strict regulations on promotional activities of pharmaceutical companies. For creating transparency regarding financial relationship between doctors and pharmaceutical companies, labs- the country can adopt a monitoring platform similar to 'Physicians Payment Sunshine Act' followed in some countries.

Long Term

i. Provide full course antibiotics free of cost from government hospitals provided that the prescriptions are supported by C/S reports
ii. Make laws on proper disposal of expired/unused antibiotics at the consumer/client level
iii. Reform pharmacy education and expand pharmacy profession in the area of clinical pharmacy and community pharmacy

3. Awareness, Education and Training

Short Term

i. Strengthen measures to complete the process to form AMS committee and AMS team at all health care facilities up to Upazilla level
ii. Utilize the initial experiences of AMS implementation at some facilities in scaling up
iii. Introduce national level awards/recognition for high performing health facility in AMS program implementation

iv. Facilitate access to and/or support pre- and in-service training on how to optimize antibiotic prescribing, dispensing and administration for all relevant health-care professional groups (e.g., doctors, pharmacists, nurses) with involvement of professional societies
v. Train and create a pool of journalists for AMR related news coverage through media fellowships

Long Term

i. Develop structured and targeted BCC strategy and campaign based on formative research and behavioral analysis including plan for impact study
ii. Continue education on basic IPC principles including hand hygiene, which gets momentum during COVID-19 pandemic, at all educational institutes starting from primary schools and back up this practice by setting up functional hand washing blocks.
iii. Ensure practice of STGs and hence AMS at the beginning of the professional career of health care professional (medical, pharmacy, nursing, community health workers) and introduce mandatory professional development activities throughout the professional career
iv. Set criteria to secure specific government funding for AMS in all facilities

4. Supporting Technologies and Data

Short Term

i. Collaboration with private sectors to include private lab data in national AMR data base

Long Term

i. Create post for Consultant Microbiologist at district level hospitals and beyond, and establish AST facility
ii. Establish AMU surveillance system
iii. Establish HAI surveillance system
iv. Establish an advanced AMR surveillance system incorporating molecular and genetic sequencing

This assessment using WHO AMS Practical Toolkit was to support establishment of a national AMS program to enable facility level AMS where access to health care is not an issue. Thus, establishing AMS in areas with limited access to health care, was not under the scope of this assessment. To produce and sustain the achievements of the national AMS program, it is important to create an enabling environment. Some of the measures that can indirectly help to retain the output of national AMS program - the status of the informal providers and their scope of practice should be clearly defined and monitored, plan to overcome health workforce shortage to meet the WHO recommended appropriate skill mix of doctors, nurses and medical technologists with a ratio of 1:3:5, strengthen non-communicable diseases prevention programs by addressing lifestyle issues, the concept of antimicrobial stewardship programs should be included in the country's disaster planning or emergency response preparedness efforts etc.

AMS is not an option; it is an integral part of the quality of care. We all should play our role.

INTRODUCTION

Sir Alexander Flemming discovered penicillin in 1928 and immediately after winning the Nobel prize in 1945, he warned about the possibility of emergence of resistance as a consequence of misuse of this

'miracle drug'. The term 'Antimicrobial Stewardship' was coined in 1996 - a gap sufficient to blur the benefits of antibiotics and to put the major achievements of modern medical science at risk[1]. If there is no effective antibiotics in hand, it will not only make it impossible to treat any infectious diseases, it will also challenge different life saving procedures like major surgery, chemotherapy, organ transplantation. A recent study published in Lancet highlighted that an estimated 4.95 million deaths associated with bacterial AMR in 2019, including 1.27 million deaths attributable to bacterial AMR. This study is considered as the first comprehensive assessment of global burden of AMR.[2]

BACKGROUND

Antimicrobial Resistance (AMR) is a global health and development threat.[3] In 2015, World Health Assembly's endorsement of the Global Action Plan (GAP) on Antimicrobial Resistance (AMR), with five strategic objectives as key outlines for countries in developing national action plans against AMR, is one of the major steps in the fight against AMR. The fourth objective of GAP states "Optimize use of antimicrobial medicine in human and animal health".[4] This optimization of antimicrobial use is possible through Antimicrobial Stewardship Programs (AMS/ASP) which ultimately improves patient outcomes, reduce AMR and health-care-associated infections and save health-care costs.[5]

AMS is an AMR technical capacity area in the Joint External Evaluation (JEE) which was published by WHO in 2016 as an effort to facilitate International Health Regulation (IHR) reporting to World Health Assembly.[6] This is also reflected at WHO's Benchmark for IHR Capacities as the section 3.4 states- "Optimize use of antimicrobial medicines in human and animal health and agriculture."[7]

Considering the magnitude of its importance in this ongoing COVID-19 pandemic period, WHO in 2021 has compelled to pick "Combat drug resistance" as one of the top 10 global health issues to track.[8]

In alignment with the GAP, the Ministry of Health and Family Welfare (MoH&FW) of Bangladesh has developed the National Action Plan (2017-2022) based on the National Strategy for ARC (Antimicrobial Resistance Containment) through a multisectoral working group representing human health, animal health and drug administration.[9] Recently, National AMR Focal Point, Communicable Disease Control (CDC), DGHS has updated the NSP and NAP with support from USAID MTaPS program in Bangladesh. The updated NAP-ARC (2021-2026) has adopted a comprehensive One Health approach including aquatic

---

[1] https://en.wikipedia.org/wiki/Antimicrobial_stewardship

[2] https://www.thelancet.com/journals/lancet/article/PIIS0140-6736(21)02724-0/fulltext

[3] https://www.who.int/en/news-room/fact-sheets/detail/antimicrobial-resistance

[4] https://www.who.int/publications/i/item/9789241509763

[5] https://pubmed.ncbi.nlm.nih.gov/26947617/

[6] https://apps.who.int/iris/bitstream/handle/10665/259961/9789241550222-eng.pdf

[7] https://ghsagenda.org/2020/07/16/who-benchmarks-for-international-health-regulations-ihr-capacities-2019/

[8] https://www.who.int/news-room/spotlight/10-global-health-issues-to-track-in-2021

[9] National Action Plan on Antimicrobial Resistance Containment in Bangladesh; 2017-2022, CDC, DGHS, Ministry of Health and Family Welfare

health and environmental perspective of AMR which was lacking in the previous NSP and ARC.[10] Here, among the eight key strategic objectives, objective five states "promoting rational use of antimicrobials" which explicitly states AMS is a priority area for action.

On the other hand, the constitution of Bangladesh commits to address inequalities in access to health care and the country joined the global community in committing to achieve UHC by 2030. The SDG target 3.8 states that "Achieve universal health coverage, including financial risk protection, access to quality essential health-care services and access to safe, effective, quality and affordable essential medicines and vaccines for all".[11, 12] Achieving Universal Health Coverage requires addressing the quality-of-care agenda. The government of Bangladesh has planned to improve the quality of health care service delivery. The quality improvement secretariat (QIS) of MOHFW is playing a crucial role in overseeing the nationwide quality improvement in health care service delivery areas. The major mandate of QIS is to guide & oversee the quality improvement activities nationwide through:

1. M&E of Quality of Care for Heath Service Delivery
2. Quality of Care related protocols, guidelines, SOP, tools
3. Ensuring attainment of National Health Care Standards
4. Ensuring Coordination mechanism among GO, NGO, DP and autonomous body [13]

USAID MTaPS program has been working with the Government stakeholders in different domain of the health system strengthening, knowledge management and governance. The antimicrobial resistance containment portfolio is one of the major areas of this evolving collaboration. The Program's GHSA work focuses on contributing to antimicrobial resistance (AMR) containment, specifically by supporting infection prevention and control (IPC), antimicrobial stewardship (AMS), and multisectoral coordination on AMR.[14] This MTaPS/Bangladesh supported national level assessment of policies, regulations, legislation and guidelines, including supply chain management that can impact AMS practices in the country, will facilitate to develop a robust AMS program in the country.

OBJECTIVES OF THE ASSESSMENT

The objectives of the assessment of national AMS policies, regulations, legislation and practices conducted in human health sector are –

1. To gather data on existing national AMS policies, regulations, legislation and practices conducted in human health sector.

---

[10] National Strategy and Action Plan for Antimicrobial Resistance Containment in Bangladesh; 2021-2026, CDC, DGHS, Ministry of Health and Family Welfare

[11] https://bangladesh.savethechildren.net/sites/bangladesh.savethechildren.net/files/library/UHC%20Report%20Bangladesh_Final_7%20Feb%202018.pdf

[12] https://www.who.int/data/gho/data/themes/topics/indicator-groups/indicator-group-details/GHO/sdg-target-3.8-achieve-universal-health-coverage-(uhc)-including-financial-risk-protection

[13] http://qis.gov.bd/wp-content/uploads/2019/11/About-QIS-min.pdf

[14] https://msh.org/projects/mtaps/

2. To identify the gaps and challenges.
3. To produce a set of recommendations to strengthen AMS governance.

Although the levels of viral, fungal and parasite resistance to antimicrobials are also increasing, the focus of this rapid assessment is bacterial resistance to antibiotics.

METHODOLOGY

This was a rapid assessment using a national protocol adapted from the standard protocol developed by MTaPS[15] and using the checklist of national core elements from WHO AMS practical toolkit.[16]

The methodology and the rapid assessment tool (Appendix-2) were contextualized after discussing with CDC, DGHS. Before starting the formal assessment process, a GO (Appendix-1) letter was issued from CDC, DGHS.

Description of the Rapid Assessment Tool

The checklist of national core elements from WHO AMS practical toolkit was used for this national level rapid assessment with some additional components for assessment, for better understanding of Bangladesh's current AMS scenario.

WHO developed the checklist of national core elements with an aim to guide countries in identifying the most critical elements for their national context supporting the implementation of the AMS program.

The four broad core elements of national AMS programs as identified by WHO are –

1. National plan and strategies
2. Regulation and guidelines
3. Awareness, education and training
4. Supporting technologies and data

Each four core elements are subdivided into some related components. WHO stratified these components of the core elements into two types – Basic (which may require fewer resources) and Advanced (which may require more resources).

To use the national core elements list, first it is identified what is already in place and the level of implementation, which core elements require accelerated implementation (partially implemented) and what is missing (Figure-1).

[15] Tool to Support Conduct of Rapid Situational Analysis of Antimicrobial Stewardship Policies in MTaPS Program Countries, MSH, 2019
[16] https://apps.who.int/iris/handle/10665/329404

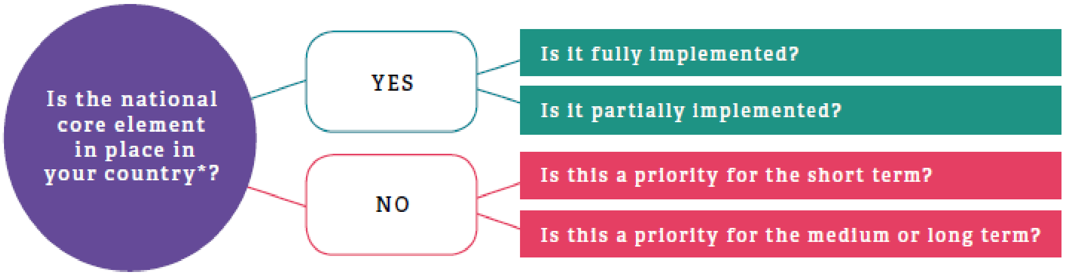

Figure 1: Guide for Navigating the National Core Elements Checklist to Identify, Prioritize and Develop a Stepwise Implementation Plan

In addition to the core elements of WHO (Section 1-4 of Rapid Assessment Tool), information was also collected more elaborately on supply management policies (Section 5 of Rapid Assessment Tool) and on the level of appropriate use of antibiotics by prescribers, dispensers and patients through data on related studies/reports (Section 6 of Rapid Assessment Tool) as they are relevant to this assessment.

In summary all the responses/findings were recorded in this Rapid Assessment Tool as –

**Fully implemented**	Yes
**Partially implemented**	Yes
**Not implemented**	No

There is a comment section in the assessment tool, where the assessor needed to include qualitative explanation in support of the responses to make the overall report close to the realities on the ground.

To strengthen the reliability of the information, some documents (TOR, meeting minutes, official e-mail copy etc.) were collected as verifiers.

After this exercise, the 'partially implemented' and 'not implemented' core elements were prioritized based on the country context and the recommendations were developed accordingly.

Data Collection Method

- Desktop review and
- Stakeholder's interviews: direct and virtual (if necessary, considering the pandemic situation)

The desktop review was an important step of this rapid assessment for providing a foundation upon which the subsequent step of stakeholders' interview took place. On the other hand, the stakeholders' interviews were mainly used for filling up the gaps of information and clarify any complex issue. Also, sometimes it was the stakeholders' interview which guided the desktop review to make efficient use of the limited time period of this rapid assessment.

Duration of Data Collection

The desktop review started just after preparing a draft outline of the Rapid Assessment Tool on March 14, 2022. Stakeholder's interviews were conducted from March16- June 30, 2022. After this formal data collection period, data were analyzed to prepare the assessment report.

Major Stakeholders

Major Stakeholders	Assigned Roles and Responsibilities related to AMS Bangladesh Perspective	Part of Relevant Section in the Rapid Assessment Tool
Communicable Disease Control (CDC), DGHS.<br>https://www.cdc.gov/	Antimicrobial Resistance Containment in Bangladesh 2017-2022 program is coordinated by the Disease Control Unit of CDC, DGHS. The National Strategy and Action Plan of AMR containment for the duration of 2021-2026 was updated and revised recently where CDC, DGHS played the key role.	• Section 1(National Plan and Strategies);<br>• Section 2 (Regulation and Guidelines);<br>• Section 3 (Awareness, Training and Education)<br>• As needed
Directorate General of Drug Administration (DGDA).<br>https://dgda.portal.gov.bd/	The Directorate General of Drug Administration (DGDA) under the Ministry of Health & Family Welfare is the Drug Regulatory Authority of the country.	• Section1(National Plan and Strategies);<br>• Section 2 (Regulation and Guidelines);<br>• Section 3 (Awareness, Training and Education)<br>• Section 5 (Supply Management Policies)<br>• As needed
Institute of Epidemiology, Disease Control and Research (IEDCR).<br>https://iedcr.gov.bd/	Acts as the National Referral Laboratory for Human Health for National AMR Surveillance.	• Section 4 (Supporting Technologies and Data)<br>• Section 6 (Report or Studies on The Level of Appropriate Use by Prescribers, Dispensers and Patients)<br>• As needed
Quality Improvement Secretariate (QIS).<br>http://qis.gov.bd/about-us/	Established in 2015, the Quality Improvement Secretariate is playing an important role in overseeing the nationwide activities of the quality improvement of health care service delivery areas. As part of its mandate the QIS has developed the Antimicrobial Resistance QI Framework to be used in the facility level.	• Section 6 (Report or Studies on The Level of Appropriate Use by Prescribers, Dispensers and Patients)<br>• As needed
International Centre for Diarrheal Disease and Research, Bangladesh (icddr,b)<br>https://www.icddrb.org/	International health research organization based in Dhaka, Bangladesh. Emerging and re-emerging infections, Universal Health Coverage are some of its major research themes.	• Section 6 (Report or Studies on The Level of Appropriate Use by Prescribers, Dispensers and Patients)<br>• As needed
Central Medical Store Depot (CMSD)<br>http://cmsd.gov.bd/	It's a Bangladesh government agency under the Ministry of Health and Family Welfare that is responsible for procuring medical equipment for public hospitals.	• Section 05 (Supply Management Policies)<br>• As needed

Major Stakeholders	Assigned Roles and Responsibilities related to AMS Bangladesh Perspective	Part of Relevant Section in the Rapid Assessment Tool
World Health Organization (WHO)<br>https://www.who.int/bangladesh/about-us/our-work	WHO is the directing and coordinating authority for health within the United Nations System. One of the strategic priorities of the WHO is- Implementing the provisions of the International Health Regulations.	• As needed

Data Cleaning and Data Analysis: After data collection, data cleaning and data analysis was done as per objectives of the rapid assessment.

SUMMARY OF DATA COLLECTION AND ANALYSIS

The findings using the Rapid Assessment Tool (Ref. to Appendix- 2) are described below separately for essential national core elements for AMS programming as suggested by WHO and other additional elements related with AMS programming. To know the context, additional information also gathered through this assessment tool by adding relevant items for assessment.

ESSENTIAL NATIONAL CORE ELEMENTS FOR AMS PROGRAMMING

WHO identified 20 essential national core elements (Originally 19 core elements; here for ease of analysis, 9a and 9b counted separately thus totaling to 20) under four broad area for AMS programming in the low-and-middle income countries. Current status of these core elements as found through this assessment is summarized in the following table –

Status of WHO National Core Elements	No. of core elements (%) (Total 20)
Fully implemented	09 (45%)
Partially implemented/initiated	08 (40%)
Not implemented	03 (15%)

As per four categories, the findings of this assessment are summarized in Figure-2.

*I. **National Plan and Strategies:***

WHO identifies five national core elements for AMS programming under "National Plan and Strategies'. These include –

1. National Action Plan on AMR that states AMS is a priority
2. Dedicated funding for the national action plan on AMR
3. Technical Working Group on AMS established with clear terms of reference
4. National AMS implementation plan or policy endorsement

5. Monitoring and evaluation mechanism in place for the national action plan on AMR (WHO Essential National Core Elements for AMS Programming in LMICs -advanced)

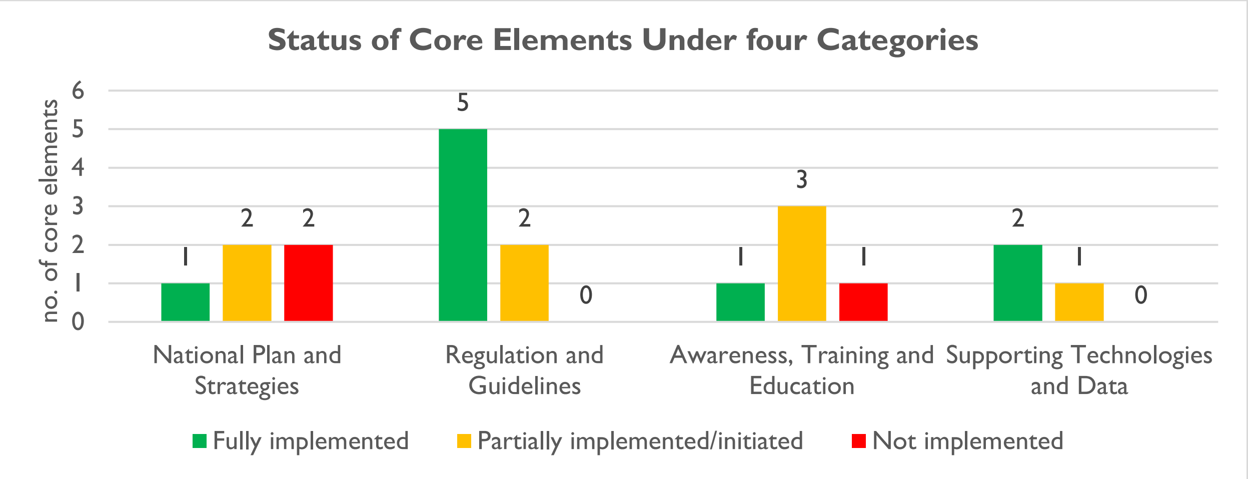

Figure 2: Status of Core Elements Under Four Categories

Among the total five core elements under 'National Plan and Strategy', one core element was found to be fully implemented (Ref. to Appendix 2: Rapid Assessment Tool Item for assessment # 1.3.1), two core elements were partially implemented or initiated (Ref. to Appendix 2: Rapid Assessment Tool Item for assessment # 1.4.1,1.7.1) and two were not implemented or initiated (Ref. to Appendix 2: Rapid Assessment Tool Item for assessment # 1.5.1, 1.6.1) during this assessment period.

The Ministry of Health and Family Welfare (MoH&FW) formulated The National Drug Policy 2016 that offers guidance on the overall pharmaceutical management and regulation. It was in alignment with National Health Policy 2011 and the National Population Policy 2012. The Objectives of the National Drug Policy 2016 were –

i. To ensure people can have easy access to safe, effective and good quality drugs at affordable prices.

ii. To ensure rational and safe use of drugs and proper dispensing.

iii. To achieve self-sufficiency in the manufacture of drugs and raw materials by providing services and facilities on a priority basis to all local drug manufacturing industries.

iv. To expand the export of drugs that manufactured in the country.

v. To establish effective surveillance system of medicines.

In concordance with the global and WHO activities on ARC, the MoH&FW in Bangladesh has taken initiative to conduct program for containment of antimicrobial resistance in Bangladesh. Director, Disease Control and Line Director, Communicable Disease Control, DGHS was selected as a national focal point to coordinate the national program. The National Action Plan (2017-2022) for Antimicrobial Resistance Containment (ARC) in Bangladesh was developed based on the national strategy through a multisectoral

working group. In early 2022, the Directorate General of Health Services (DGHS) with the technical support of the USAID MTaPs Program has revised and updated the National Strategy and Action Plan for ARC in Bangladesh which has been set out for the next five-years (2021-2026). During the revision process, One Health Approach was followed giving aquatic health and environment their due priority alongside human health and terrestrial animals' issues. Among the eight key strategic objectives, the key strategy # 05 explicitly mentioned about AMS as a priority. Other strategies are also important and interlinked to establish AMS in the country. The eight key objectives are –

- Key Strategy 1: Strengthen multi-sectoral coordination;
- Key Strategy 2: Increasing stakeholder awareness and engagement;
- Key Strategy 3: Establish integrated surveillance and strengthen laboratory capacity;
- Key Strategy 4: Enabling good practices;
- Key Strategy 5: Promoting responsible use of antimicrobials;
- Key Strategy 6: Promote innovation and research on AMR;
- Key Strategy 7: Reduce environmental spread of AMR;
- Key Strategy 8: Strengthen global collaboration and partnerships.

On the other hand, the Quality Improvement Secretariat (QIS) of Ministry of Health and Family Welfare (MoH&FW) developed National Anti-microbial Stewardship Quality Improvement (QI) Framework as part of its mandate of overseeing the nationwide activities of the quality improvement of health care service delivery areas. Published in 2019, the framework mainly focuses on facility level initiative to prevent antibiotics resistance and thus ensuring quality of care. It emphasizes on developing the culture of prescribing antibiotics according to clinical protocol and hence describes eight clinical standards. The document also outlines AMS implementation plan; roles and responsibilities of AMS team members, quality indicators for AMS QI framework; and monitoring and evaluation. On January 2022, a formal letter was issued by QIS to form AMS committee and AMS team at all district hospitals of the country.

In this assessment, it was found that some core elements under 'National Plan and Strategy' are just initiated/ partially implemented. These are summarized below.

Dedicated funding for the national action plan on AMR is one of the core elements of AMS programming at the national level. After, the National Strategy and Action Plan for ARC in Bangladesh [2021-2026] was prepared and approved, the costed national action plan on AMR has been drafted through engaging all relevant stakeholders.

An established monitoring and evaluation mechanism for the national action plan on AMR is recognized by WHO as an advanced level essential element for AMS Programming in LMICs. The NTC endorsed Monitoring and Evaluation (M&E) Framework for NAP for ARC 2017-2022 on early 2021 and the revised NSP & NAP of Antimicrobial Resistance Containment (2021-2026) also has in-built section on M&E mechanism. Thus, the M&E framework (the guiding document) was developed to measure the progress of implementation; but the mechanism or total system for M&E is not yet properly established.

The essential core elements that are found to be not implemented or initiated are- there is no subcommittee on AMS/National Multisectoral AMS committee/ AMS technical working group, National IPC committee not yet developed (although an ad-hoc IPC committee worked at the national level to

meet the IPC requirements of the COVID-19 pandemic). National IPC policy, national AMS policy or practice guideline and national antibiotic policy yet to be developed.

II. Regulation and Guidelines:

WHO identifies seven core elements for AMS programming under "Regulation and Guidelines'. These include –

1. Integration of the AWaRe classification of antibiotics in the national EML and formulary
2. Up-to-date clinical guidelines that include AMS principles and integrate the AWaRe classification of antibiotics
3. Regulations on fixed-dose combinations of antibiotics
4. Regulations on prescriptions-only sale of antibiotics;
5. Regulation and enforcement of prescription-only dispensing of antibiotics (WHO Essential National Core Elements for AMS Programming in LMICs -advanced)
6. Measures in place to ensure continued availability of quality-assured antibiotics.
7. Measures in place to ensure affordability of essential antibiotics

Among the total seven core elements under 'Regulation and Guidelines', five core elements are found to be fully implemented (Ref. to Appendix 2: Rapid Assessment Tool Item for assessment # 2.2.1, 2.3.1, 2.4a.1, 2.5.1, 2.6.1) and two core elements are partially implemented or initiated (Ref. to Appendix 2: Rapid Assessment Tool Item for assessment #2.1.1, 2.4b.1) during this assessment period.

Under the initiative of CDC, DGHS Standard Treatment Guidelines (STG) on Antibiotic Use in Common Infectious Diseases of Bangladesh was prepared and got approval from the NTC on 20 January 2022. USAID, MTaPS closely collaborated with CDC throughout the process. The objectives of STG are –

i. To recommend treatment for common clinical infections requiring antibiotic therapy.
ii. To promote "AWaRe" classification and approach at all level before prescribing an antibiotic.
iii. To promote rational use of antibiotics.
iv. To serve as a tool of antimicrobial stewardship in the hospitals.

The whole process of developing the guidelines was accomplished through a working group with representation from different disciplines like Infectious Disease, Internal Medicine, Critical Care Medicine, Pediatrics, Surgery, Otolaryngology, Ophthalmology, Orthopedics, Obstetrics & Gynecology, Microbiology, Pharmacology and Epidemiology. All standard procedures like literature search and summarization of the evidence, listing commonly occurring diseases of Bangladesh, documenting microbiology sensitivity pattern from available authentic sources were followed. A template of summary tables with preferred and alternative antibiotics for empirical treatment incorporating the AWaRe classification of antibiotics for the likely causative agents was constructed. Following necessary steps, the final version was approved by NTC with the suggestion of continuous updating by incorporating the National AMR Surveillance data and recent research on AMR pattern. This is recommended that the developed STG is going to be used for empirical antimicrobial treatment before getting culture sensitivity

result or for using in hospitals where regular microbiology testing is not available. The hospitals where microbiology testing is available should develop their own protocol for empirical antibiotic therapy using their institutional antibiogram. For infectious diseases, where separate national guidelines are available, those guidelines should be preferred over this guideline. Thus, in general this national guideline meant to be used by all HCF up to district level. Medical college hospitals and specialized hospitals should develop their own treatment guideline based on their antibiogram opportunity. The coherence between the STG and the EML will be ensured in the next revision of EML for which a committee has been formed.

STG Mobile application has also developed which will be available at Google Play Store once finalized. Any medical practitioner (MBBS and BDS) can use this app to look for appropriate antibiotic combinations according to WHO AWaRe classification for a particular infectious disease before prescribing.

TOT on Standard Treatment Guideline (STG) on Antibiotic Use and Antimicrobial Stewardship (AMS) was organized by CDC, DGHS in collaboration with USAID MTaPS to develop trainers for cascade training. A total of 420 participants (Female 60 and male 360) received TOT in February-March, 2022. The participants comprised of resident medical officers (RMOs), junior consultants, resident physicians (RPs), medical officers (MOs) & Upazilla Health and Family Planning Officers (UHFPOs) from districts hospitals, Upazilla Health Complex including one private hospital. The major topics covered through the TOT were Antimicrobial Resistance: Role of Antimicrobial stewardship and IPC, AMR: Mechanism & Beyond, Pharmacology of Antibiotics, Principles of Antibiotic Prescribing for Clinicians, STG App introduction & orientation.

In Bangladesh, prescribing right is not only limited to registered physicians it also includes a wider group of health professionals like –

1. In national disease control programs, antimicrobials may be allowed to be prescribed by the trained health workers, in particular cases such as Acute Respiratory Infection (ARI).
2. Community Health Care Provider (CHCP) having 3-6 months of basic training on the management and treatment of common diseases are allowed to prescribe a list of antibiotics based on clinical signs and symptoms of patient.
3. Before referral, midwives in specific emergency situation can prescribe antibiotics within their Scope of Practice and as per National Guidelines for Midwives.

The Directorate General of Drug Administration (DGDA) under the Ministry of Health and Family Welfare is the drug regulatory authority of the country. As per guideline of WHO, DGDA performs the following 09 functions through 09 functional unit or cell.

1. National Regulatory System (RS)
2. Registration and Marketing Authorization (MA)
3. Vigilance (VL)
4. Market Surveillance and Control (MC)
5. Licensing Establishment (LI)
6. Regulatory Inspection (RI)

7. Laboratory Testing (LT)
8. Clinical Trial's Oversight (CT)
9. NRA Lot Release (LR)

The Drug Control Committee (DCC) of DGDA was formed according to the direction of section 4 of the Drugs (Control) Ordinance. As per section 6, this committee is entrusted to evaluate all medicines registered in Bangladesh to ensure safety, efficacy and usefulness. It discourages the use of fixed-dose antibiotic combination.

There are 39 items labeled as OTC, for rest of the items drug sellers cannot sell without prescription. Other than OTC drugs, rest are prescription only medicine. There is also a high court verdict that states that without prescription from a registered doctor, antibiotics cannot be sold.

DGDA as the drug control authority of the country acts to ensure that available antibiotics are of suitable quality and substandard or falsified drugs are not being sold based on the 1940 Drug Act, Drug Control Ordinance 1982. Regulatory, administrative, and legal action taken for Good Manufacturing Practice (GMP) noncompliance, substandard and counterfeit manufacturers, distributors, and sellers. Recently, to make the law more appropriate to meet the existing challenges, a new law (Drug Act 2022) has been submitted to the cabinet division combining Drug Act of 1940 and Drug Control Ordinance 1982. Here, all offenses are divided into two types- cognizable offence and non-cognizable offence.

Pharmacies are inspected through divisional office, district office. Inspection reports are submitted on daily basis. In 2020-2021, total 36,441pharmacies were inspected. Legal actions were taken through drug court, magistrate court and mobile court. In some cases, Special Power Act can also be applied. From July'20-June'21, different legal actions taken as follows –

Through drug court	13 law suits
Through magistrate court	92 law suits
Through mobile court	1,715 law suits
Total fine through mobile court	BDT 7,58,00,100
Imprisonment of different duration	49 persons
Substandard or falsified drugs seized (equivalent in BDT)	BDT 7,75,30,153

The pharmaceutical companies have been regularly inspected to monitor GMP compliance. The law enforces that if a company do not follow the specified criteria, their license will be cancelled. To date, 21 pharmaceutical companies have been suspended for not maintaining the standard. Post-marketing surveillance is also strictly followed.

Drug Administration Registration number or DAR number and Marketing Authorization Number or MA number at the medicines' packages of all medicines including antibiotics signifies authenticity of the drug. There is a DGDA drug verification apps (developed during COVID-19) by which drugs can be verified through scanning of QR code. Drug related query or complaints can also be submitted through this apps.

To promote public health by ensuring the safety, efficacy, and quality of medicines and other health products, MOH&FW declared the ADRM cell of DGDA as the National Pharmacovigilance Centre (NPC) for Bangladesh in 2013. An Adverse Drug Reaction Advisory Committee (ADRAC) formed by MOH&FW

support the ADRM cell to provide technical guidance for implementing PV activities, evaluating adverse drug events (ADE) reports, and recommending regulatory decisions and actions to ensure medicine safety.

Bangladesh has gained international recognition and access to early worldwide information about potential safety risks by becoming the 120th member of the World Health Organization's International Drug Monitoring Centre, known as the Uppsala Monitoring Centre (WHO-UMC). DGDA developed the National Guideline on Pharmacovigilance System in Bangladesh in 2018. As per DGDA annual report 2020-2021, 32 hospitals and 30 pharmaceutical companies are submitting online ADR reports to National ADR cell till date. Once submitted, the reports are analyzed through WHO Causality Assessment Scale. ADRAC review the reports for making needful regulatory recommendations to the Licensing Authority (Drugs) for regulatory actions to protect public health from medicine's harm and then reports are uploaded in VigiFlow.

National Pharmacovigilance Centre of Bangladesh received total 968 reports between January, 2020- June, 2021for primary evaluation by ADRM cell. Out of these 968 reports, 381reports (about 40%) were found incomplete for lacking of minimum information. During this period, 11th and 12th ADRAC meetings were held where total 106 reports were evaluated and 49 reports were classified as likely/probable and 57 reports were classified as possible as per WHO Causality Assessment Scale. At present, MTaPS project of USAID is providing support to PV activities at DGDA.

The DGDA started the Model Pharmacy and Model Medicine Shop accreditation program to help ensure good pharmacy, dispensing, and storage practices. Model pharmacies are supervised by a graduate pharmacist, and 668 model pharmacies and 41,009 model medicine shops are entering the program.

DGDA ensures the affordability of all drugs including the antibiotics through Price Fixation Committee and Price Fixation Technical Subcommittee of DGDA. There are representation of Ministry of Health and Family Welfare, DGDA, DGHS, Bangladesh Pharmaceutical Society, Bangladesh Consumers' Association as members of that committee. There are separate defined process and formula of price fixation for drugs included in the primary health care, drugs other than those that are needed for primary health care and drugs that are imported. Suitable dosage forms (including Pediatrics formulations when appropriate) are available in the country. Allocation of all types of medicines to government health facilities based on the ratio of bed versus patients and consumption of medicines in the previous year; whereas in private sector monitoring of antimicrobial sales throughout the country internally analyzed for their marketing strategy department. The pharmaceuticals companies have established extensive sales and distribution networks. Hundreds of Medical Promotion Officer (MPO) or Medical Representatives (MR) visit retail pharmacies and doctors' chamber to promote their products.

The system for regulating the promotional activities of pharmaceuticals companies not yet established in the country. 'Code of Pharmaceutical Marketing Practices' was developed by Directorate of Drug Administration on 1994 based on Ethical Criteria for Medicinal Drug Promotion, WHO, Geneva; IFPMA Code of Pharmaceutical Marketing Practices, Code of Practice for the Pharmaceutical Industry, Association of British Pharmaceutical Industries, London after due consideration to the socio-economic, cultural and health care context of Bangladesh. The main objective of the Code of Pharmaceutical Marketing Practices is to support and encourage the improvement of health care through the rational use of medical substance. Now DGDA has taken the initiative to update this document. DGDA review and

approve the promotional literature of the pharmaceutical product, but does not monitor the 'gift' offered/given to medical professionals as part of the promotional activities by the pharmaceuticals.

The pharmaceutical companies of Bangladesh meet almost 98% demand of the local market. Now, Bangladesh is exporting medicines to 157 countries including UK, USA, Canada.

In this assessment, two important essential core elements under 'Regulations and Guidelines' are found to be 'partially implemented.'

Firstly, the sale of drugs without prescription is prohibited in Bangladesh, but not punishable under the National Drug Policy 2016. For this, a new act with the provision of penalty is now at the cabinet division for final approval. In proposed Drug Act it is incorporated that selling antibiotic without prescription is a punishable offence and monetary punishment will be up to 20,000 BDT.

Secondly, although Bangladesh has adopted WHO AWaRe categorization at Bangladesh National Formulary (BDNF), 5th version, 2019, still the Essential Drug List (EDL) not yet updated. It was known from DGDA that WHO AWaRE categorization will be incorporated in the next version of Essential Drug List.

Bangladesh runs a pluralistic health system with five parallel systems of medicine- allopathic, ayurvedic, herbal, homeopathic and unani. Regulatory oversight of all systems is provided by the National Regulatory Authority, the Directorate General of Drug Administration. As per annual report of DGDA (2020-2021) there were 323 persons employed at the DGDA, whereas the no. of vacant post was 397 at that time. In July 2021, 'WHO Benchmarking of the National Regulatory Authority (NRA) through Global Benchmarking Tool (GBT)' assessed nine functions of DGDA and classify DGDA as a non-stringent regulatory agency. Through this assessment, two functional areas of DGDA were found to be at the maturity level 02 (Registration and Marketing Authorization; Laboratory Testing) and other functional areas were found to be at maturity level 01.

III. Awareness, Training and Education:

WHO identifies five national core elements for AMS programming under "Awareness, Training and Education'. These include –

1. Regular public antibiotic awareness campaigns
2. Education in schools on basic infection prevention and control (IPC) principles
3. Training on AMS competencies for AMS team members
4. Education and training for all health-care professionals on AMS
5. Incentives to support implementation of AMS programmes in all health-care facilities, including staffing standards, training and accreditation (WHO Essential National Core Elements for AMS Programming in LMICs -advanced)

Among the total five core elements under 'Awareness, Training and Education' 01 core component is 'fully implemented' (Ref. to Appendix 2: Rapid Assessment Tool Item for assessment # 3.1.1), three core components are 'partially implemented' or initiated (Ref. to Appendix 2: Rapid Assessment Tool Item for

assessment # 3.2.1,3.3.1,3.4.1) and one core component is found to be not implemented during this assessment period (Ref. to Appendix 2: Rapid Assessment Tool Item for assessment # 3.5.1)

Regular public antibiotic awareness campaigns like WAAW celebrated every year on 18-24 November since 2015 in a coordinated way by different stakeholders. The theme of 2021 WAAW was 'Spread awareness, stop resistance'. CDC, DGHS, DGDA, FAO organized different programs both at the national and upazilla level. Some of the highlights of WAAW 2021 were- advocacy workshop at DGDA, a roundtable discussion on AMR covered by one of the most circulated national daily '*Kalerkantha*'. SMS or Short Message Service was circulated nation-wide through Bangladesh Telecommunication Regulatory Authority (BRTA) to raise awareness on AMR. Awareness programs for medical college students were organized. In addition, the CDC held advocacy meetings in every upazila with active participation from human and animal health sectors. Different IEC materials highlighted the day's theme.

Several AMR awareness meetings in different districts took place during model pharmacy inauguration sessions organized by DGDA with participation of honorable health minister, state ministers, local leaders and leaders of the Bangladesh Association of Pharmaceutical Industries and Bangladesh Chemist and Druggist Samity. To sensitize people on AMR issues, different messages displayed through TV channels. There is an AMR Web platform for show-casing all sorts of knowledge products with a one health approach. Till date, 03 issues of AMR newsletters, published with support of Fleming Fund country grant, uploaded in the web platform.

A regulatory decision was endorsed by the Drug Control Committee on 20th March, 2022 that "The manufacturers will put the red identification mark with the text 'Antibiotic' and the message 'Do not use without prescription of registered physician'. This decision was based on the findings of two prior studies- "Baseline survey on the level of awareness about antimicrobials & AMR and its impacts on self-medication in Bangladesh" and the key informant interview "Rapid situation analysis of present labeling of antibiotics in Bangladesh and preference for label change patterns for incorporation of identification mark on Antimicrobial products and implementation challenges." DGDA has a plan to do an impact study of this regulatory measures.

IPC and AMS are interrelated and for this, basic IPC principles, including hand hygiene should be included at early schools' education and school should provide an enabling environment to practice it. Education on basic IPC principles including hand hygiene become evident and strengthened in the country during COVID pandemic when schools were preparing to open. In government primary schools, around 71% schools have hand washing blocks.

Only theoretical aspect of AMR is included at the curricula of the health care professional (medical, pharmacy, nursing). The concept of STGs and hence AMS and their importance are missing in the curricula.

Training on AMS competencies for AMS team members is fundamental for establishing AMS programming in the country which was found to be initiated ('partially implemented') in the country during this evaluation. GO letter from QIS to form AMS committee and AMS team at all district hospitals of the country was issued in January, 2022.

CDC is planning to develop a national AMS guideline and AMS training module. A draft has been circulated to all relevant stakeholders to review, modify and improve towards standard.

Incentives to support implementation of AMS programmes in all health-care facilities, including staffing standards, training and accreditation is an advanced level Core Elements for AMS Programming in LMICs as identified by WHO which was not implemented during this assessment. AMS programming in the country is at early stage and at this moment, there is no formal incentives from the government to support implementation of AMS programs in all health-care facilities.

IV. Supporting Technologies and Data:

WHO identifies three national core elements for AMS programming under 'Supporting Technologies and Data'. These include –

1. National surveillance system on AMC in place
2. National surveillance system on AMR in place with laboratory capacity to guide optimal use of antibiotics in clinical practice and update clinical guidelines
3. Diagnostic tests available and capacity building undertaken to optimize antibiotic use (WHO Essential National Core Elements for AMS Programming in LMICs -advanced)

Among the total three core elements under 'Supporting Technologies and Data', two core components are 'fully implemented' (Ref. to Appendix 2: Rapid Assessment Tool Item for assessment # 4.1.1, 4.2.1), one core components are 'partially implemented' or initiated (Ref. to Appendix 2: Rapid Assessment Tool Item for assessment # 4.3.1) as found during this assessment period.

DGDA is the national authority for Antimicrobial Consumption (AMC) and Antimicrobial Use (AMU) surveillance. To monitor Antimicrobial Consumption (AMC)/ Antimicrobial Use (AMU) a taskforce titled "Taskforce to Monitor Antimicrobial Consumption/Use in Bangladesh" has been formed on March 2021. Chaired by the Director General of DGDA, this taskforce with one health approach has representation from human health, animal health, fisheries and environment.

National Focal Point and Alternate National Focal Point for GLASS-AMC reporting have been nominated. The National Guideline on Antimicrobial Consumption Surveillance in Bangladesh has been approved in the taskforce meeting held on 12 June 2022. In that meeting, 2019-2020 AMC surveillance data was presented.

The national AMR surveillance strategy for 2020-2025 has been developed recently with support of the Fleming Fund country grant. Institute of Epidemiology, Disease Control and Research (IEDCR) and Department of Livestock Services (DLS) has been identified as the focal institute to monitor and coordinate the AMR surveillance in the human health and animal health sector in the country respectively.

Since 2017, the IEDCR has been conducting countrywide AMR surveillance, beginning with five sentinel sites, then scaled up to nine, with the support of the Government of Bangladesh (GoB), the Global Health Security Agenda (GHSA), and the World Health Organization (WHO). There is a trained team at IEDCR with representation from ARC for AMR surveillance which is a standardized active prospective surveillance. There are designated staffs at all sites who use a form to collect case-based sample. There are pre-defined priority samples and priority pathogens. The samples are tested for AST (Antibiotic Susceptibility Testing) in the local attached lab. Some samples are being tested at IEDCR for external quality assessment (EQA). This national data of AMR surveillance is entered into the data base of IEDCR.

There is yearly dissemination of this data to all stakeholders (e.g., Public Health Specialists, Clinicians, Development Partners). There is also a web-based real time data sharing from IEDCR. Internationally, as a signatory, Bangladesh is providing data to GLASS (Global Antimicrobial Resistance and Use Surveillance System). In future IEDCR plans to increase the number of sentinel sites from 09 to 11 for a better representation of the country.

There are Sectoral Working Groups to coordinate the activities of surveillance. Each Sectoral Working Group (SWG) will work as a forum of the surveillance sites, laboratory networks, and reference laboratories within the sector. They will work closely to facilitate joint-learning and problem solving. A state-of-the-art National Reference Laboratory has been established at IEDCR premises with support from Fleming Fund which participated in 03 rounds of External Quality Assessment Systems (EQAS) in 2021.

Although, Bangladesh's health system's development is commendable; there is lack of facilities for some essential investigations like culture sensitivity test. At present, AST facility is available at all medical college hospitals and only at a few district hospitals.

Most of the district hospitals and upazilla level health facilities do not have AST facilities.

OTHER ELEMENTS

I. Supply Management Policies:

For the purpose of this assessment, part of supply management policies or guideline those are related to pharmaceutical product quality and the utilization of the EML were explored (Ref to Appendix- 2: Rapid Assessment Tool, Section # 05).

To ensure the quality of antimicrobials marketed in the country, no separate mechanisms or policies beyond common policies for all medicines. The government sector source antibiotics mainly from the state-owned Essential Drugs Company Limited (EDCL). Government can also source from private pharmaceutical companies through competitive bids. After MoH&FW approval, requisitions from all the line directors are forwarded to CMSD to be filled and distributed to health facilities at various level of health system countrywide. In the RFQ, for vendor selection and evaluation, there is requirement of certain prequalification of suppliers. The total procurement policy follows Public Procurement Act, 2006 and Public Procurement Guidelines, 2008 (updated on February 2021). In this guideline, there are many in-built standard procurement documents with specific criteria which guide to select qualified suppliers. Some of these pre-requisites are- the pharmaceutical company and the product must be registered in the country with DGDA (at their own country in case of international procurement), must have Certificate of Pharmaceutical Product (COPP), must have GMP certification as per WHO, have some lab analysis report as required. There are specific procurement forms coded as PG1, PG2 and PG3 available from Central Procurement Technical Unit (CPTU) of Ministry of Planning which are used for procurement of product below 5 lacs, below 25 lacs and above 25 lacs respectively.

After receiving goods at the warehouse following standard operating procedures, DD CMSD serves notification to Head of Survey Committee and other members scheduling a date for the survey. Survey

committee issues Survey Report checking against invoice, forwarding docs and technical specs. Then Survey Report sent to Director CMSD for approval.

For quality assurance of medicine, visual inspection of packaging, labelling and physical appearance (at time of receipt at all levels of supply chain), basic rapid quality tests using portable minilab systems, post-purchase testing at Drug Testing Laboratory done as required. Some sophisticated tests are performed through CRO.

Monitoring of the marketed product is done by DGDA.

In private sector, no separate mechanisms for antimicrobials to ensure the quality of the marketed products in the country; common mechanisms followed like all other medicines. The supplier or importers need to be registered through DGDA. The product also needs to be registered. Two types of drug inclusion in the DGDA list.

1. New generic drug that is the first time in Bangladesh
   - If the product is INN, in the registration process, need to submit the drug sample for testing in the drug testing laboratory,
   - If the product is BP, USP or FDA approved, the drug sample will be provided for registration but it is not necessary to test in the drug testing laboratory
2. New brand of existing generic
   - Just provide quality analysis data along with the registration form
   - No need to test

National Control Laboratory received the ISO17025:2005 certification by Bangladesh Accreditation Board in 2017 and ISO/IEC 17025:2017 certification by American National Accreditation Board. When some sensitive drug test like bioequivalence, required for the export market, it's done from outside through third party (CRO) as it's not possible to perform within the capacity of DGDA. In Bangladesh, only BP, USP, INN or FDA included products are approved for registration. Thus, if a drug has been registered in the DGDA or has a DAR no., no need to check its pharmacopoeia standard.

Every pharmaceutical company possesses Good Manufacturing Practice (GMP) certification. As per the regulation, without GMP certification, no pharmaceutical company will manufacture drugs. Thus, every company in the pharmaceuticals sector has this certification. Usually, every year DGDA visit pharmaceutical factory to check this. If the company has GMP certification but does not follow the instruction in the GMP, the DGDA authority has right to close the factory.

In the production process, one or two strip (10/20 tablets) from each batch (10,000 tablets) has been preserved in the quality assurance department of each pharmaceutical. If there is a complaint of any drug in the market, they will collect the sample from the market, match the batch number with their preserved products and then make a quality test of it; so that they can provide explanation to the complaint and take necessary action to overcome it.

In November 2018 DGDA started implementation of Risk-Based Post-Marketing Surveillance (RB-PMS) with technical support from USAID funded program USP-PQM. RB-PMS focuses on sampling considering the most vulnerable risk factors i.e., top selling sample, geographical area, hard to reach area, new product, product having stability issue, costly products, disease, season etc. that may affect the medicine quality and prioritize testing those products to find out SF products and subsequent legal action to ensure quality products in the market with the limited resource in a cost-effective way.

As per DGDA Annual Report, from July 2020 to June 2021, total 353 tests performed and among them 08 were substandard. (Mini Lab Report for all medicines).

The EML of Bangladesh was last updated on 2016.The EML list contains only few medicines and most of the pharmaceuticals don't want to promote these antimicrobials due to the controlled price. Supply chain activities within DGHS involve a mix of 'push' activities managed centrally or 'pull' activities based on customer demands.

From National AMC Surveillance Data –

Year	AMC data
2016	16.65 DDD per 1000 inhabitants/day
2017	19.87 DDD per 1000 inhabitants/day
2018	21.78 DDD per 1000 inhabitants/day
2019	24.74 DDD per 1000 inhabitants/day
2020	25.37 DDD per 1000 inhabitants/day

With the support of Fleming Fund country grant, the Communicable Disease Control program under the Directorate General of Health Services, Ministry of Health, and Family Welfare (MOH&FW) of Bangladesh shared key findings from the Capturing Data on Antimicrobial Resistance Patterns and Trends in Use in Regions of Asia (CAPTURA) project on May 18, 2022.

The project resulted in the following key accomplishments:

- Identification of historical and current data on AMR, AMC, and AMU
- Collation and analysis of 4 years of retrospective AMR/U data from 34 laboratories (11 public and 23 private) and 5 pharmacies (all private model pharmacies)
- Comprehensive capacity-building activities to optimize local data management practices and encourage data sharing at the national, regional, and global levels
- More than one million records of AST data were identified and digitized, where necessary, collected, and analyzed
- *e. coli*, *Klebsiella*, *Pseudomonas*, *staphylococcus aureus*, *Enterococcus*, *salmonella typhi* and *Acinetobacter spp*. were the most frequently isolated bacteria

- No major change in susceptibility patterns were observed in E. coli and Staphylococcus aureus in the country over the last 4 years
- Multidrug Resistance (MDR), possible Extensive Drug Resistance (XDR), and Pan-Drug Resistance (PDR) was prevalent and needs close monitoring to prevent potential spread
- WHO Global Priority List of Antibiotic-resistant Bacteria were also observed to be prevalent in some settings in the country
- A gap in internal and external quality assessment has been observed in majority of the labs, which needs to be addressed to ensure validity of AMR data being generated in the country
- The use of WATCH group and RESERVE group of antibiotics was found to be much higher (70%-88%) than the recommended use (within 40%) which is alarming.

II. Reports/Studies on the Level of Appropriate Use by Prescribers, Dispensers and Patients

Policies should be formulated based on the reality on the ground. So, it's important to have research or studies in-built within the system while implementing any program. Acknowledging the importance of linking research activities with policy level decision making, an invitation for submitting research proposal on AMR has been circulated on March 2022 by the Communicable Disease Control (CDC) program of Directorate General of Health services (DGHS), Ministry of Health and Family Welfare (MOH&FW). Through this competitive process research grant will be awarded to the nominated researchers or research organizations.

In this section, some studies conducted by different research organizations including icddr,b are presented to highlight the current scenario of antibiotic use at prescribers', dispensers' and patients' level (Ref to Appendix- 2: Rapid Assessment Tool, Section # 06).

Reports or studies on the level of appropriateness of antimicrobial prescribing:

A point prevalence survey was conducted in four acute care government hospitals (two tertiary and two secondary level hospitals) selected purposively in 2 administrative divisions of Bangladesh from 18 February to 6 April, 2021. The survey was conducted among inpatients of medicine, surgery, gynae and obstetrics, and pediatric wards including intensive care units. Total 1417 patients were enrolled-52% were female and 63% were from the 15–64 years age group. Nearly 78% of patients received at least one antibiotic during the survey period. Third-generation cephalosporins (44.6%), penicillins (12.3%), imidazoles (11.8%), aminoglycosides (7.2%), and macrolides (5.8%) were found to be highly used antibiotics. Overall, 64.0% of Watch, 35.6% of Access, and 0.1% of Reserve group antibiotics were used for treatment. The use of Watch group antibiotics was high in medicine wards (78.7%) and overall high use of Watch antibiotics was observed at secondary hospitals (71.5%) compared to tertiary hospitals (60.2%) (p-value of 0.000). Findings of this PPS, underscore the need for an urgent nationwide antibiotic stewardship program for physicians including the development and implementation of local guidelines and in-service training on antibiotic use.

To know the prescribing pattern of private practitioners, a study was conducted by collecting samples of prescription of private practitioners from patients randomly. Mode of collection was copying of

prescriptions by photocopy or by digital camera. Patients consent was taken. All the prescriptions were analyzed according to International Network of Rational Use of Drugs (INRUD) indicators. Total 500 prescriptions were analyzed using WHO formula to study prescriptions and the findings were –

1. Average number of drugs per prescription 4.40%
2. Percentage of drugs prescribed by generic name 0.30%
3. Percentage of prescriptions with an antibiotic prescribed 72 %
4. Percentage of prescriptions with an injection prescribed 10%
5. Percentage of drug prescribed from essential drug list 48%
6. Whether prescription is complete with respect to –
   a) Format 40%
   b) Dosage and duration 74 %
   c) Patient medical information 23%

From this study, it was revealed that prescribing pattern of the private practitioners were irrational regarding polypharmacy, generic name, use of antibiotics, drug selection from EDL and providing medical information.

Reports or studies on the level of appropriateness of antimicrobial dispensing:

DGDA conducted a study on "Baseline survey on level of awareness about antimicrobials and AMR and its impacts on self-medication in Bangladesh" in 08 divisions of Bangladesh. This study found that 67.3 % of pharmacy retailers do not have the knowledge about antimicrobial drugs and it's not easy for them to identify the antibiotics.

To explore antibiotic dispensing pattern in pharmacies according to the WHO AWaRe classification, drug-sellers from randomly selected pharmacies and randomly selected customers attending the pharmacies were interviewed, from January to July 2021. Data were collected on demographics and medicines purchased. The purchased antibiotics were classified into the Access, Watch, and Reserve groups. Among 128 pharmacies surveyed, 98 (76.6%) were licensed; 61 (47.7%) drug-sellers had pharmacy training. Out of 2686 customers, the study interviewed 580 (21.6%) persons who purchased antibiotics. Among the 580 customers, 523 purchased one, 52 purchased two, and 5 purchased three courses of antibiotics (total 642 courses). Of the antibiotic courses, the Watch group accounted for the majority (344, 53.6%), followed by the Access (234, 36.4%) and Reserve (64, 10.0%) groups. Approximately half of the antibiotics (327/642, 50.9%) were purchased without a registered physician's prescription. Dispensing of non-prescribed antibiotics was higher in the Access group (139/234, 59.4%), followed by Watch (160/344, 46.5%) and Reserve (28/64, 43.8%) groups. These findings highlight the need to implement strict policies and enforce existing laws, and pharmacy-targeted antimicrobial stewardship focusing on proper dispensing practices to mitigate antimicrobial resistance in Bangladesh.

Reports or studies on the level of appropriateness of antimicrobial usage by the public:

A survey project was designed to explore the pattern of antibiotics use at the primary health care level in Bangladesh using carefully constructed questionnaires containing questions covering both antibiotic prescription habit of the physicians and patients' response to antibiotic use. In the first phase of this effort,

20 Upazila Health Complexes and the Union Health Centers of Dhaka and Chittagong divisions were randomly surveyed. It was evident from patient survey that cold, fever and acute respiratory infection (ARI) were prevalent (39.78%) causes that brought the patients to physician. 60.1 % of the patients reported that they took essential antibiotics from hospital free of cost while 24.5% did not get antibiotics from hospital. 34.76% of the patients reported that they completed the course of antibiotic therapy by buying antibiotics from the market whereas 56.14% of them did not buy the full course and stop taking medicine (53.46%) as soon as symptoms subside. The results of the present survey indicate that antibiotics are widely and inappropriately used without following standard guidelines or based on any rationality. This is an alarming situation, which should be properly taken care of by the relevant authority to save the people from growing antibiotic resistance.

A mobile phone survey among a general population across eight administrative divisions of Bangladesh was conducted during January–March 2021. Reported illness episodes irrespective of COVID-19 in the preceding four weeks of the interview, use of antibiotics for the illnesses, and knowledge on antibiotics among the general population were recorded. Descriptive analyses were performed. Total 1854 participants were randomly interviewed, with a mean age of 28.5 years (range: 18–75 years); 60.6% were male. Among all participants, 86.3% (95% CI: 84.7–87.8) heard names of antibiotics (which was only 48% as per a study finding before the pandemic), but only 12.1% reported unspecified harmful effects, and 3.5% reported antimicrobial resistance when antibiotics were taken without a physician's prescription. Among 257 (13.9%) participants, who consumed medicines for their recent illness episode, 32.7% (95% CI: 27.2–38.6) reported using antibiotics. Of those who could recall the names of antibiotics prescribed (n = 36), the most frequently used was azithromycin (22.2%) followed by cefixime (11.1%) and ciprofloxacin (5.6%). This study exposed an increased antibiotic use for illnesses reported in the preceding four weeks and an elevated knowledge at the community level during the COVID-19 pandemic compared with the pre-pandemic period.

RECOMMENDATIONS

National plan and strategies

Short Term

I. Establish a TWG or subcommittee on AMS (National Multisectoral AMS Committee) with clear TOR which is linked to AMC and AMR surveillance and IPC TWG
II. Develop an achievable national implementation plan for AMS
III. Allocate dedicated funding for AMS activities in all relevant OPs
IV. Develop national antibiotic policy

Long Term

I. Establish Monitoring and Evaluation mechanism emphasizing capacity building of concerned DGHS officials both at the national and sub-national level and alignment of indicators with DHIS-II
II. Establish and Strengthen IPC and WASH program

III. Establish integrated AMS activities within the action plan of other programs such as IPC, WASH, TB, malaria, HIV, UHC and Primary Health Care (PHC)

Regulation and Guidelines

Short Term

I. Update the Essential Drug List (EDL) by incorporating WHO EML AWaRe and maintaining coherence between EDL and developed STGs

II. Ensure STGs are followed at facilities and develop specific revision plan for the developed STGs

vii. Monitor health care providers who have authority to prescribe limited antimicrobials (eg. CHCP, midwives etc.)

III. Strengthen monitoring of model pharmacy and pharmacy shop[17]

IV. Update the curriculum of pharmacists' certificate course for pharmacy retailers by Bangladesh Pharmacy Council giving due importance to AMR and AMS and arrange refresher training for those pharmacists who have completed their course a long time ago.

V. Strengthen capacity building measures on pharmacovigilance reporting

VI. Update 'Code of Pharmaceutical Marketing Practices' with strict regulations on promotional activities of pharmaceutical companies. For creating transparency regarding financial relationship between doctors and pharmaceutical companies, labs; the country can adopt a monitoring platform similar to 'Physicians Payment Sunshine Act' followed in some countries.[18]

Long Term

I. Provide full course antibiotics free of cost from government hospitals provided that the prescription is supported by C/S report

II. Make laws on proper disposal of expired/unused antibiotics at the community level.

III. Reform Pharmacy education and explore pharmacy profession in the area of clinical pharmacy and community pharmacy

Awareness, Education and Training

Short Term

I. Strengthen measures to complete the process to form AMS committee and AMS team at all health care facilities up to Upazilla level

II. Utilize the initial experience of AMS implementation at some facilities in scaling up

III. Introduce national level awards/recognition for high performing health facility in AMS program implementation

IV. Facilitate access to and/or support pre- and in-service training on how to optimize antibiotic prescribing, dispensing and administration for all relevant health-care professional groups (e.g., doctors, pharmacists, nurses) with involvement of professional societies

V. Train and create a pool of journalists for AMR related news coverage through media fellowships

[17] https://fb.watch/dcPtMfoS8i/

[18] https://www.hopkinsmedicine.org/office-of-johns-hopkins-physicians/resources/sunshine-act.html

Long Term

I. Develop structured and targeted BCC strategy and campaign based on formative research and behavioral analysis including plan for impact study
II. Continue education on basic IPC principles including hand hygiene, which gets importance during COVID pandemic, at all educational institute starting from primary schools and back up this practice by setting up functional hand washing blocks.
III. Install the concept of STGs and hence AMS at the beginning of the professional career of health care professional (medical, pharmacy, nursing, community health workers) and introduce mandatory continued professional development activities throughout the professional career
IV. Set criteria to secure specific government funding for AMS in all facilities

Supporting Technologies and Data

Short Term

I. Collaboration with private sectors to include private lab data in national AMR data base

Long Term

I. Create post for Consultant Microbiologist at district level hospitals and establish AST facility
II. Establish AMU surveillance system
III. Establish HAI surveillance system
IV. Establish an advanced AMR surveillance system incorporating molecular and genetic sequencing

Others

Short Term

i. Encourage research activities focused on different aspect of AMR, AMS, IPC at medical colleges with active participation of undergraduate medical students.

Long Term

i. The status of the informal providers and their scope of practice should be clearly defined and monitored
ii. Set up modern storage facility at upazilla health complexes with proper storekeeping by a medical store keeper using digital technology
iii. Overcome health workforce shortage to meet the WHO recommended appropriate skill mix of doctors, nurses and medical technologists with a ratio of 1:3:5
iv. Strengthen non-communicable diseases prevention programs by addressing lifestyle issues[19]
v. The concept of antimicrobial stewardship programs should be included in the country's disaster planning or emergency response preparedness efforts

[19]https://www.researchgate.net/publication/348527870_Antibiotic_Prescribing_Patterns_at_COVID-19_Dedicated_Wards_in_Bangladesh_A_Single_Center_Point-Prevalence_Survey

LIMITATIONS

1. This assessment using WHO AMS Practical Toolkit is for supporting establishment of a national AMS program to enable facility level AMS, where access to health care is not an issue. Thus, establishing AMS in areas with limited access to health care, was not under the scope of this assessment.
2. Although the levels of viral, fungal and parasite resistance to antimicrobials are also increasing, the focus of this assessment is bacterial resistance to antibiotics.
3. There was no focal person at CMSD for Antimicrobial Resistance Containment Program which causes difficulty in data collection from CMSD.
4. Due to the busy schedule of the national level stakeholders and due to the need for more than one interviews in some cases, it took much time to complete this assessment.

STAKEHOLDERS' WORKSHOP

After the data collection and analysis of national level AMS assessment (Human and Animal Health Sectors) was completed, a workshop was organized by Centre for Disease Control, DGHS on 16th August 2022 at the conference room of Old DGHS building, DGHS, Mohakhali (Appendix-03,04). The workshop was facilitated by USAID MTaPS Program.

Objectives of the Workshop

The objectives of this national level multisectoral workshop on AMS were-

1. To share the findings of national level assessment of AMS policies and practices (Human Health Sectors)
2. To share the findings of national level assessment of AMS policies and practices (Animal Health Sectors)
3. To share an overview of AMS guidelines for human health sector
4. To share the draft outline of national multisectoral AMS plan

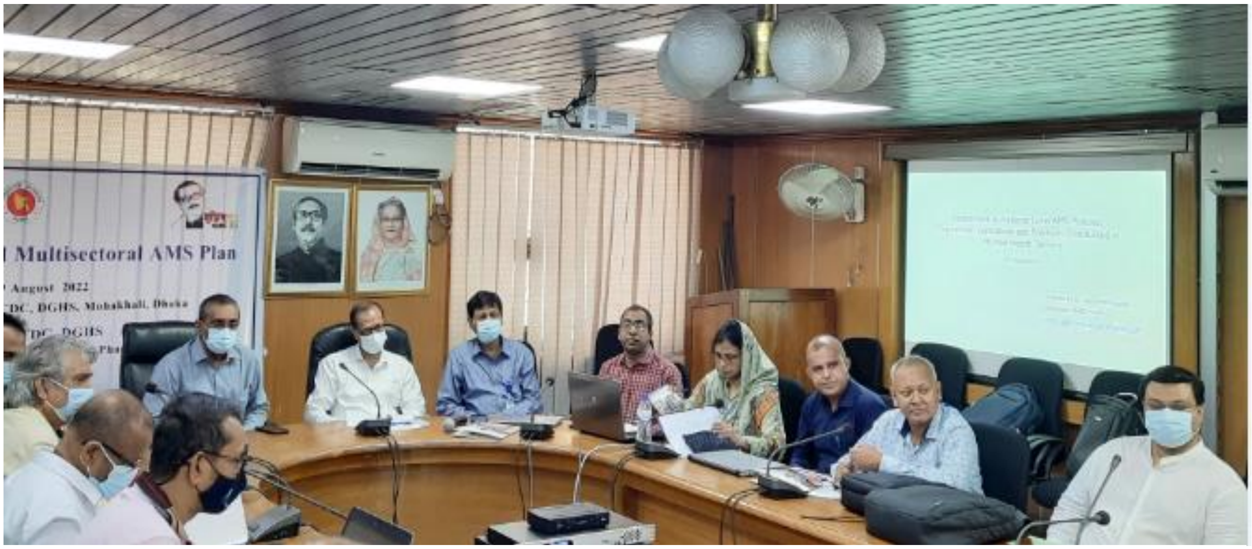

Picture 1: Opening of the AMS Workshop

Participants of the Workshop

There were about 30 participants at the workshop (Appendix-05). There were participations from the government sector, development partners, research and academic institutions. Among the government side, there were representative from CDC-DGHS, DGDA, DLS, BLRI, BARC, DOF, IEDCR, NIPSOM and among the development partners there were representative from WHO, FAO, USAID MTaPS, DAI.

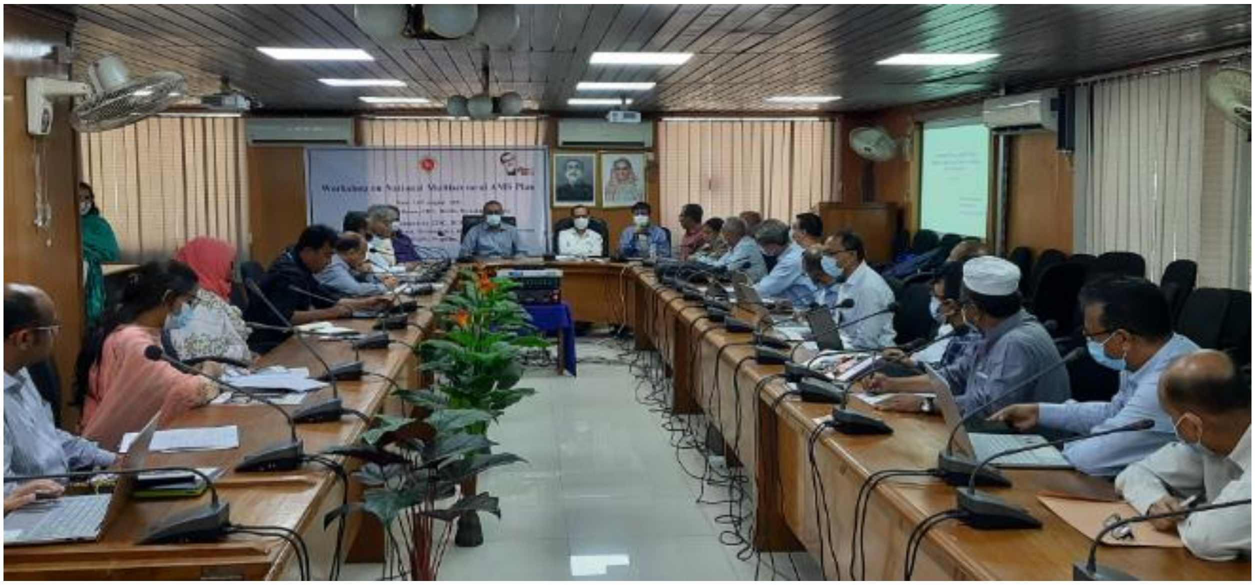
Picture 2: Participants in the AMS Workshop

Academic institutions were represented by Imperial Hospital, Uttara Adhunik Medical College Hospital (UAMCH).

Opening of the Workshop

At the beginning of the workshop, all the participants gave self-introduction. Then Dr. Aninda Rahman, DPM, CDC-DGHS mentioned about the objectives of the workshop. Prof. Dr. Md. Nazmul Islam; Director, Disease Control and Line Director, Communicable Disease Control, DGHS formally inaugurated the workshop and gave his welcome speech. In his welcome remark, he said that AMS is now a reality. The morbidity and mortality, due to the ever-increasing AMR, could be more than those due to COVID-19 if AMR remain un-challenged, he mentioned. He also added that we know what to do to tackle AMR in a country like Bangladesh and whom to collaborate with; thus, now it's time to take these agenda forward. He also highlighted that we want to move forward with a One Health Approach and if we can strengthen this approach, we can reach the expected destination of controlling AMR.

Sharing of the draft report on 'Assessment of National Level AMS Policies, Regulations, Legislations and Practices conducted in Human Health Sectors'

Dr. Najia Amin Salsabil, consultant of USAID, MTaPS program shared the draft report on 'Assessment of National Level AMS Policies, Regulations, Legislations and Practices Conducted in Human Health Sectors'. The purpose of assessment was to review the baseline/existing resources, identifying the gaps and challenges and to provide a set of recommendations. During her presentation, besides objectives she covered the methodology, description of the rapid assessment tool, data analysis, summary of findings and analysis and limitations of the assessment. She concluded her presentation with some recommendations. (Appendix-06)

Sharing of the draft report on 'National Stewardship Policies, Regulations and Practices conducted in Animal Health Sectors'

Dr. Nure Alam Siddiky, consultant of USAID, MTaPS program shared the draft report on 'National Stewardship Policies, Regulations, and Practices Conducted in Animal Health Sector'. During his presentation, he covered the objectives, milestones, methodology, tools, composition of the AMS assessment report, salient features of essential core elements for AMS policies, guidelines and practices. He concluded his presentation with some relevant recommendations. (Appendix-07)

Sharing of the draft report on 'Antimicrobial Stewardship (AMS): Guideline for Human Health Sector-An Overview'

Dr. Javedur Rahman, consultant of USAID MTaPS program, shared an overview of 'AMS Guideline for Human Health Sector'. In his presentation, he highlighted the background, steps of development of the guideline, national strategy documents /international AMS guidelines consulted, key components and purpose of the AMS guideline. (Appendix-08)

Open Discussion

After the presentations, there was an interactive discussion.

- Human Health Sectors

- From the findings of the national AMS assessment of human health sectors, there were some findings to be hopeful for and also there are findings which reminded us to be very cautious. Completion of the 'partially implemented core components' would be challenging.
- We have gained a thorough insight through this assessment, but sometimes we need to go beyond what is prescribed by WHO. For coordination purpose, if we need to form a separate technical working group for AMS in addition to the existing AMR committee; we may need to consider the practicality of this approach, as same group of people will be there. We may form one committee for overall AMR covering AMS, IPC and diagnostic stewardship. Now NAP has been costed which will positively impact implementation and we can consider AMS as part of the AMR.
- We are proposing here separate core working group for AMS, in addition to the existing Technical Working Group of AMR. It's also a requirement of WHO to have a separate committee for AMS. If we form a single committee covering all, then there will be limited discussion time in meetings. So, even some of the persons are same in different committee, it is essential to have separate committee for the sake of speedy implementation.
- The purpose of separate committee is to form separate leadership body for different agenda.
- Too many committees sometimes become detrimental. We may form different committee when we are developing some documents, some methodology. But when we are going to implement that, some sort of integrated approach is needed. For example, IPC or stewardship program or diagnostic stewardship- all these are highly interlinked and when we are going to implement them at the facility level too many committees will become non-functional. But when we are doing some assessment, then high level consultation will be helpful. For example, in the assessment done in human health sector, 'partially implemented core components' can be subdivided into some small groups to give some in-depth insights- (5-10) % implemented, (10-20) % implemented etc.
- For 'partially implemented core components', there is a column explaining the current status of each component like why it is called 'partially implemented' and what have been done so far. These explanations are included in the report.
- In the workshop it was informed that at the hospital level, besides the IPC and AMS committee, there is another committee called 'Quality Improvement Committee' with overlapping agenda and similar membership pattern.
- We all are working as per the strategy. IPC, AMS and surveillance are the three challenging issues. We have proposed for a separate committee for surveillance and there is already such a committee at the national level. At present at national level, no separate committee for IPC and no separate committee for AMS. At national level, we need separate committee undoubtedly and for coordination there is a Core Working Group.
- For facility level, IPC and AMS, cannot be all the same. For IPC, we need nurse, cleaners, patient party in addition to doctors. On the other hand, for AMS, doctors are the main stakeholders and in some cases the pharmacists (if they are placed in that hospital). So, for facilities we should try to form separate committee for IPC and AMS.
- About the assessment result, it was mentioned that the high percentage of 'fully implemented status' is due to the presence/availability of policies, guidelines, SOP etc. as per assessment tool.

- To follow the WHO AWaRe classification is important; but sometimes it is more important to save the patient. We become fearful, if we found that 'Watch' group of drugs are more used in the hospital; but it can happen as in the hospital more serious patient are admitted. 'Access' group will be found more used when the whole country is considered.
- In AMS committee, representatives of different professional societies should be included- Medicine, Surgery, Gynae etc. All committees should be liable to core committee.
- The goal of all committees at the facility level should be for reducing the use of antibiotics.
- AMR and AMS committee can be merged. But IPC and Quality Management Committee should be separate at the facility level.

After presentations of AMS assessment of Animal Health Sectors and AMS Guideline for Human Health Sectors, some open discussion followed. Among different issues, an important discussion focusing on developing National Antibiotic Policy took place. It was informed in the workshop that CDC should initiate the process of developing National Antibiotic Policy where CDC will focus on Human Health and DLS will focus on animal health and the approval should be from the Ministry of Health to term it as a policy.

Also, it was recommended to include representatives from pharmaceutical industries in different committees.

To establish antibiotics audit system and feedback mechanisms, formulary restrictions, establishing HAI infections surveillance, developing antibiotics order tool were some important components to establish AMS at facility level which we need to implement considering our country perspective. For this, we need to develop SOP. Engaging the physicians, through different professional societies, in the whole process is important. CDC has planned discussion meetings initially with physicians of Medicine, Surgery, Gynae and Pediatrics. We may do piloting of AMS at some selected facilities. PPS, AMU surveillance should be done by the hospital for better credibility and acceptance.

Closing of the workshop

It was decided in the workshop that, the presentations and the draft outline of the multisectoral AMS plan will be shared with the participants of the workshop through e-mails for their feedback and after 07 working days it will be finalized based on the feedback. (Appendix-09)

CONCLUSION

To be successful in implementing AMS and thus reducing AMR, the country needs to follow "One Health" approach. Hence, a multisectoral national AMS plan could be a game changer. Establishing AMS program at national and facility level is important and fundamental; but it will only be sustainable, if we can engage the community. Coordination between IPC, WASH and AMS activities needed at the national and facility level. The political commitment, which is the main driving force to bring about any change, is already there- as Bangladesh's PM is leading the Global Leaders Group on Antimicrobial Resistance as the Co-Chair. We have to take the ownership of the problem and should play our role to stop the next pandemic. No time to waste. Act Now!

APPENDIX1: GO LETTER (ASSESSMENT)

Government of People's Republic of Bangladesh
Disease Control Division
Directorate General of Health Services
Mohakhali, Dhaka-1212

Memo No: DGHS/CDC/ARC/2022 - 982 Date: 30.03.2022

To

……………………………………………

…………………………………………………………………………

Subject: Request to extend cooperation on the assessment of national AMS policies and practices conducted in human health sector

As you are aware that Antimicrobial Resistance (AMR) has become one of the most serious and growing threats to public health globally. National Strategy and National Action Plan for Antimicrobial Resistance Containment [2021-2026] has been updated in which antimicrobial stewardship (AMS) is a key priority area for intervention. There is a growing need for more specific guidance on how to establish, implement and evaluate effective AMS program at the national and facility level.

USAID MTaPS program is closely working with DGHS in different domain of health. MTaPS/Bangladesh has planned to support the national core working group of AMR to review and assess policies, regulations, legislation and guidelines, including supply chain issues. MTaPS/Bangladesh will conduct an assessment using a national protocol adapted from the standard protocol developed by MTaPS and using the national-level checklist in the WHO AMS Practical Toolkit to gather data on the status of the national AMS program. Assigned consultant will collect data as part of the assessment. Data gathered through this exercise will be analyzed to produce a set of recommendations for AMS programming in the country.

You are therefore requested to extend your support and cooperation during the process of assessment including data collection at your department.

30/03/2022

Prof. Dr. Md. Nazmul Islam
Director, Disease Control
and Line Director, Communicable Disease Control (CDC),
Director General of Health Services

Distribution for information and necessary action (not according to seniority)

- Director General, Directorate General of Health Services, Mohakhali, Dhaka-1212 (Attn: A/D Coordination)
- Director General, Directorate General of Drug Administration, Mohakhali, Dhaka-1212
- Director, IEDCR
- Program Manager, QIS
- Dr. Jebunnesa Rahman, Country Project Director, USAID MTaPS Program
- Dr. Abu Zahid, Principal Technical Advisor, USAID MTaPS Program
- Deputy Program Manager (ARC, Viral Hepatitis), CDC, DGHS
- Office Copy.

APPENDIX 2: RAPID ASSESSMENT TOOL (USED FOR BOTH DESKTOP REVIEW AND STAKEHOLDER INTERVIEWS)

	Item for assessment	Yes / No	Comments	Source of Information/ Verifiers Used (Websites/ Interviews/ Reports etc.)
**I**	**NATIONAL PLAN AND STRATEGIES***			
**1.1**	**National Drug Policy that offers guidance on pharmaceutical management and regulation**			
**1.1.1**	There is a national drug policy that offers guidance on pharmaceutical management and regulation.	Yes	• Bangladesh has **National Drug Policy 2016** that offers guidance on pharmaceutical management and regulation.<br>• The MoH&FW formulated The National Drug Policy 2016 on December 2016 upon review of National Drug Policy,1982 and National Drug Policy,2005.<br>• It was in alignment with National Health Policy 2011 and the National Population Policy 2012.<br>• The Objectives of the National Drug Policy 2016 are –<br>– To ensure people can have easy access to safe, effective and good quality drugs at affordable prices.<br>– To ensure rational and safe use of drugs and proper dispensing.<br>– To achieve self-sufficiency in the manufacture of drugs and raw materials by providing services and facilities on a priority basis to all local drug manufacturing industries.<br>– To expand the export of drugs that manufactured in the country.<br>– To establish effective surveillance system of medicines.<br>• The main features of the National Drug Policy 2016 for AMR containment are –	• Interview: S. M. Sabrina Yesmin, Assistant Director, Directorate General of Drug Administration<br>• Updated National drug Policy 2016 http://dgdagov.info/index.php/information-center/guidance-documents/1136-national-drug-policy-2016-with-essential-drug-list-and-otc-list<br>• DGDA Annual Report 2020-2021 http://dgdagov.info/index.php/about-dgda/annual-report<br>• Antibiotic Use and Resistance in Bangladesh: Situation Analysis and Recommendations on Antibiotic Resistance; The GARP- Bangladesh National Working Group, GARP-Bangladesh & CDDEP https://cddep.org/publications/bangladesh-situation-analysis-amr/<br>• Effective Multisectoral Coordination on Antimicrobial Resistance in Bangladesh: Current Status and Recommendations; November 2019, USAID MTaPS.

	Item for assessment	Yes / No	Comments	Source of Information/ Verifiers Used (Websites/ Interviews/ Reports etc.)
			– good pharmacy, storage, and distribution practices; STGs; government and private hospitals with 100 or more than 100 beds to have their own "Antibiotic user guidelines'; retail sale of drugs without prescription prohibited except OTC ones; DTCs in public and private hospitals to promote rational use of medicines; regular updating of essential medicines lists etc.	
**1.2**	**Policies that support the regulation of medicines with specific focus to antimicrobials**			
**1.2.1**	There are other policies that support the regulation of medicines with specific focus to antimicrobials	Yes	• Quality Improvement Secretariat (QIS) of Ministry of Health and Family Welfare (MOH&FW) has developed National Anti-microbial Stewardship Quality Improvement (QI) Framework as part of its mandate of overseeing the nationwide activities of the quality improvement of health care service delivery areas.<br>• This document mainly focuses on facility level initiative to prevent antibiotics resistance and thus ensuring quality of care.<br>• It talks about developing the culture of prescribing antibiotics according to clinical protocol and hence describes eight clinical standards.<br>• This document also outlines AMS QI framework implementation plan; roles and responsibilities of AMS team members, quality indicators for AMS QI framework; and monitoring and evaluation.<br>• A formal letter was issued by QIS to form AMS committee and AMS team at all district hospitals of the country on January 2022.	• Interview: Dr. Md. Akhteruzzaman, Program Manager (Joint Secretary), Health Economics Unit (HEU), Health Services Division, Ministry of Health and Family Welfare.<br>• National Antimicrobial Quality Improvement Framework, QIS 2019 http://qis.gov.bd/wp-content/uploads/2019/05/National-Anti-Microbial-Stewardship-AMS-QI-Framework.pdf<br>• Supporting Documents –<br>– GO letter from QIS to form AMS committee and AMS team at all district hospitals of the country
**1.3**	**National Action Plan on AMR that states AMS is a priority**			

	Item for assessment	Yes / No	Comments	Source of Information/ Verifiers Used (Websites/ Interviews/ Reports etc.)
1.3.1	The government endorses a National Action Plan on AMR explicitly stating that AMS is a national priority. (WHO NATIONAL CORE ELEMENT # 01)	Yes	• With the initiative of Directorate General of Health Services (DGHS), Bangladesh and with the technical support of the USAID MTaPs Program of Management Science for Health (MSH), National Strategy and Action Plan has been revised and updated.<br>• The National strategy and action plan has been set out for the next five-years (2021-2026) for containing AMR.<br>• In this strategy document, comprehensive One Health Approach is emphasized.<br>• Aquatic health and environment are given their due priority along side human health and terrestrial animals issues.<br>• There are eight key strategic objectives and among them, the key strategy # 05 explicitly mentioned about AMS as a priority. Other strategies are also important to establish AMS in the country.<br>• The eight key objectives are –<br>– **Key Strategy 1:** Strengthen multi-sectoral coordination;<br>– **Key Strategy 2:** Increasing stakeholder awareness and engagement;<br>– **Key Strategy 3:** Establish integrated surveillance and strengthen laboratory capacity;<br>– **Key Strategy 4:** Enabling good practices;<br>– **Key Strategy 5:** Promoting responsible use of antimicrobials;<br>– **Key Strategy 6:** Promote innovation and research on AMR;	• Interview: Dr. Aninda Rahman, DPM, CDC, DGHS<br>• National Strategy and Action Plan for Antimicrobial Resistance Containment in Bangladesh [2021-2026]

	Item for assessment	Yes / No	Comments	Source of Information/ Verifiers Used (Websites/ Interviews/ Reports etc.)
			– **Key Strategy 7:** Reduce environmental spread of AMR;<br>– **Key Strategy 8:** Strengthen global collaboration and partnerships.	
**1.4**	**Dedicated funding for the national action plan on AMR**			
1.4.1	The national action plan on AMR has been costed and includes national activities for implementing AMS activities in the short to medium term (1–3 years) and/or long term (5 years). (WHO NATIONAL CORE ELEMENT # 02)	Yes (partial-initiated)	• National Strategy and Action Plan for Antimicrobial Resistance Containment in Bangladesh [2021-2026] has been prepared and approved.<br>• A consultant has been appointed with support of the USAID MTaPS program for the development of the costed national action plan on AMR.<br>• To support this, a 03-days training on AMR costing tool of WHO was held at CDC, DGHS with technical support from WHO Head Quarter and WHO Bangladesh Team.<br>• All relevant stakeholders participated in the training.	• Interview: Dr. Aninda Rahman, DPM, CDC, DGHS<br>• National Strategy and Action Plan for Antimicrobial Resistance Containment in Bangladesh [2021-2026]<br>• Supporting Documents –<br>– Invitation letter for training on AMR costing tool from WHO<br>– Presentation at a meeting on June 06 at MSH office
**1.5**	**Technical working group on AMS established with clear terms of reference**			
1.5.1	The national multisectoral coordination group has established a TWG or subcommittee on AMS that includes at least one ministry of health focal point and is linked to the AMC and AMR surveillance and IPC technical working groups. (WHO NATIONAL CORE ELEMENT # 03)	No	• No subcommittee on AMS – there is no National AMS Committee / AMS technical working group. Draft of TOR for the committee is prepared for this.<br>• National IPC committee not yet developed; although a National IPC Committee developed during the COVID pandemic to meet the emergency.	• Interview: Dr. Aninda Rahman, DPM, CDC, DGHS<br>• National Strategy and Action Plan for Antimicrobial Resistance Containment in Bangladesh [2021-2026]<br>• Supporting Documents –<br>– Draft TOR of National AMS committee
**1.6**	**National AMS implementation plan or policy endorsement**			

	Item for assessment	Yes / No	Comments	Source of Information/ Verifiers Used (Websites/ Interviews/ Reports etc.)
1.6.1	An achievable national implementation plan for AMS with defined goals, outcomes, timelines, structures (national and hospital core elements) and responsibilities has been developed. It is linked to the national IPC plan or policy if it exists and is integrated into the government's annual action plan as appropriate.<br>(WHO NATIONAL CORE ELEMENT # 04)	No	• Bangladesh has no national AMS policy or practice guideline.<br>• No antibiotic policy yet developed.<br>• Also, there is no national infection prevention and control (IPC) policy, although an ad-hoc IPC committee worked at the national level to meet the IPC requirements of the COVID pandemic.	• Interview: Dr. Aninda Rahman, DPM, CDC, DGHS<br>• National Strategy and Action Plan for Antimicrobial Resistance Containment in Bangladesh [2021-2026]
**1.7**	**Monitoring and evaluation mechanism in place for the national action plan on AMR (WHO Essential National Core Elements for AMS Programming in LMICs -advanced)**			
1.7.1	A mechanism is in place to monitor and evaluate progress on implementing the national action plan on AMR with the explicit inclusion of AMS and IPC activities.<br>(WHO NATIONAL CORE ELEMENT # 05)	Yes (partial)	• Monitoring and evaluation framework (the guiding document) has been developed to measure the progress of implementation; but the mechanism or system for monitoring and evaluation is not yet developed.<br>• Although NTC endorsed Monitoring and Evaluation Framework for NAP for ARC 2017-2022 on early 2021; the total monitoring and evaluation framework still not get functional.<br>• To ensure objective review and monitoring of the implementation progress, the revised NSP & NAP of Antimicrobial Resistance Containment (2021-2026) also has in-built section on monitoring and evaluation mechanism.	• Interview: CDC, DGHS<br>• National Strategy and Action Plan for Antimicrobial Resistance Containment in Bangladesh [2021-2026]<br>• National Action Plan for Antimicrobial Resistance Containment in Bangladesh: Monitoring and Evaluation Framework

	Item for assessment	Yes / No	Comments	Source of Information/ Verifiers Used (Websites/ Interviews/ Reports etc.)
** Please refer to page 08 of the Antimicrobial stewardship programmes in health-care facilities in low- and middle-income countries. A practical toolkit. Geneva: World Health Organization; 2019.Licence: CC BY-NC-SA 3.0 IGO. https://apps.who.int/iris/handle/10665/329404 and page 09 of the Tool to Support Conduct of Rapid Situational Analysis of Antimicrobial Stewardship Policies in MTaPS Program Countries, MSH, 2019*				
**2**	**REGULATION AND GUIDELINES***			
**2.1**	**Integration of the AWaRe classification of antibiotics in the national EML and formulary**			
2.1.1	Develop or review and adapt the antibiotics contained in the national EML and the national formulary with reference to the WHO EML AWaRe groups of antibiotics and outline AMS strategies for each group. (WHO NATIONAL CORE ELEMENT # 06)	Yes (partial)	• Bangladesh has adopted WHO AWaRe categorization at Bangladesh National Formulary (BDNF), 5th version, 2019.<br>• The Essential Drug List (EDL) still not updated (last updated on 2016). WHO EML AWaRe will be incorporated in the next version of EDL.	• Interview: S. M. Sabrina Yesmin, Assistant Director, Directorate General of Drug Administration (DGDA)<br>• Bangladesh National Formulary (BDNF); 5th Version, 2019<br>• Essential drug List (EDL) 2016 http://dgdagov.info/index.php/information-center/guidance-documents/1136-national-drug-policy-2016-with-essential-drug-list-and-otc-list
**2.2**	**Up-to-date clinical guidelines that include AMS principles and integrate the AWaRe classification of antibiotics**			
2.2.1	The government endorses and makes available up-to-date standard treatment guidelines for infection management, based on national susceptibility surveillance data (where possible) to assist with antibiotic selection for common clinical conditions. These guidelines should be based on and explicitly include stewardship principles. Incorporate the WHO EML AWaRe classification of antibiotics into the next update of the	Yes	• Under the initiative of CDC, DGHS Standard Treatment Guidelines (STG) on Antibiotic Use in Common Infectious Diseases of Bangladesh was prepared and got approval by NTC on January 2022. USAID, MTaPs was closely collaborated with CDC throughout the process.<br>• The objectives of this national standard treatment guideline are –<br>1. To recommend treatment for common clinical infections requiring antibiotic therapy.<br>2. To promote "AWaRe" classification and approach at all level before prescribing an antibiotic.<br>3. To promote rational use of antibiotics.	• Interview: Dr. Aninda Rahman, DPM, CDC, DGHS<br>• Standard Treatment Guidelines (STG) on Antibiotic Use in Common Infectious Diseases of Bangladesh; Version:1.0; December 2021; CDC, DGHS http://amr.cdc.gov.bd/stg-guideline/<br>• Meeting minutes of NTC meeting of 20 January 2022<br>• STG Apps presentation by the consultant, USAID MTaPS<br>• Standard Treatment Guideline (STG) for appropriate use of antibiotic in sub-districts<br>• BSMMU Antibiotic Guideline 2016<br>• Antibiotic Protocol for BIRDEM

	Item for assessment	Yes / No	Comments	Source of Information/ Verifiers Used (Websites/ Interviews/ Reports etc.)
	guidelines. Where guidelines exist, a first step is to review them and to identify missing guidelines with an initial focus on empirical treatment. Where guidelines do not exist, the government provides human and financial resources to support the development of such national standard treatment guidelines and their dissemination as a priority activity. Coherence between guidelines and EMLs should be ensured. (WHO NATIONAL CORE ELEMENT # 07)<br><br>(Additional information:<br>– the process of their development<br>– revision process<br>– last revision<br>– STGs for different levels of health care practice)		4. To serve as a tool of antimicrobial stewardship in the hospitals.<br>• The whole process of developing the guidelines was accomplished through a working group with representation from different disciplines like infectious disease, internal medicine, critical care medicine, Pediatrics, Surgery, Otolaryngology, Ophthalmology, Orthopedics, Obstetrics & Gynaecology, Microbiology, Pharmacology and Epidemiology.<br>• All standard procedures like literature search and summarization of the evidence-listing commonly occurring diseases of Bangladesh, documenting microbiology sensitivity pattern from available authentic sources were followed.<br>• A template of summary tables with preferred and alternative antibiotics for empirical treatment incorporating the AWaRe classification of antibiotics for the likely causative agents was constructed.<br>• Then through two consultative workshops participated by all stakeholders including the core working group members STG was finalized. Before finalization, the draft version of STG was shared with the professional association for their opinion.<br>• There is no mention about the specific next revision plan in the STG although it got approval from the NTC with the suggestion of continuous updating by incorporating the National AMR Surveillance data and recent research on AMR pattern.<br>• This national guideline meant to be used by all HCF up to District level. Medical college and specialized	

	Item for assessment	Yes / No	Comments	Source of Information/ Verifiers Used (Websites/ Interviews/ Reports etc.)
			hospitals should develop their own treatment guideline based on local antibiogram.<br>• STG Mobile application was developed by "JBR SOFT" supported by the USAID MTaPS and it will be available at Google Play Store once finalized. Any medical practitioner (MBBS and BDS) can use this app to look for appropriate antibiotic combinations according to WHO AWaRe classification for a particular infectious disease before prescribing those to patients.<br>• The coherence between the STG and the EML will be ensured in the next revision of EML for which a committee has been formed.<br>• TOT on Standard Treatment Guideline (STG) on Antibiotic Use and Antimicrobial Stewardship (AMS) was organized by CDC, DGHS in collaboration with USAID MTaPS.<br>• This one- day training session was attended by 420 participants (Female 60 and male 360). The participants comprised of resident medical officers (RMOs), junior consultants, resident physicians (RPs), medical officers (MOs) & Upazilla Health and Family Planning Officers (UHFPOs) from districts hospitals, Upazilla Health Complex including one private hospital. The major topics covered through the TOT were Antimicrobial Resistance: Role of Antimicrobial stewardship and IPC, AMR: Mechanism & Beyond, Pharmacology of Antibiotics, Principles of Antibiotic Prescribing for Clinicians, STG App introduction & orientation. Thus total 17 training sessions was held between February 22 to March 22, 2022.	
2.2.2	There is a policy/regulation guiding the use of STG.	Yes	• This is recommended that the developed STG is going to be used for empirical antimicrobial	• Interview: Dr. Aninda Rahman, DPM, CDC, DGHS

	Item for assessment	Yes / No	Comments	Source of Information/ Verifiers Used (Websites/ Interviews/ Reports etc.)
	(Additional information: – Description of the policies/recommendations (e.g., mandatory or voluntary adherence to STGs in a hospital setup, availability of those medicines recommended in the STG in the hospital pharmacy)		treatment before getting culture sensitivity result or in hospitals where regular microbiology testing is not available. • The hospitals where microbiology testing is available should develop their own protocol for empirical antibiotic therapy using their institutional antibiogram. • For infectious diseases where separate national guidelines are available, those guidelines should be preferred over this guideline.	• Standard Treatment Guidelines (STG) on Antibiotic Use in Common Infectious Diseases of Bangladesh; Version:1.0; December 2021; CDC, DGHS
**2.3**	**Regulations on fixed-dose combinations of antibiotics**			
2.3.1	The government puts in place regulations that ban fixed-dose antibiotic combinations not approved by national or international guidelines. (WHO NATIONAL CORE ELEMENT # 08)	Yes	• The Drug Control Committee (DCC)of DGDA was formed according to the direction of section 4 of the Drugs (Control) Ordinance. As per section 6, this committee is entrusted to evaluate all medicines registered in Bangladesh to ensure safety, efficacy and usefulness. It discourages the use of fixed-dose antibiotic combination.	• Interview: S. M. Sabrina Yesmin, Assistant Director, Directorate General of Drug Administration (DGDA)
**2.4a**	**Regulations on prescriptions-only sale of antibiotics**			
2.4a.1	The government puts in place legislation or regulations that require antibiotics to be dispensed only on prescription by a qualified health-care professional (where access to health care is not an issue). (WHO NATIONAL CORE ELEMENT # 9a)	Yes	• There are 39 items labeled as OTC, for rest of the items drug sellers cannot sell without prescription. Other than OTC drugs, rest are prescription only medicine. • There is also a high court verdict that states that without prescription from a registered doctor, antibiotics cannot be sold.	• Interview: S. M. Sabrina Yesmin, Assistant Director, Directorate General of Drug Administration (DGDA)

	Item for assessment	Yes / No	Comments	Source of Information/ Verifiers Used (Websites/ Interviews/ Reports etc.)
	(Additional information:<br>– levels of prescribers who can prescribe antimicrobials<br>– level of implementations of this legislation or regulation<br>– data available on the impact of this legislation or regulation)			
2.4a.2	There is a new legislation or regulation being developed to allow prescribing rights to a wider group of health professionals.<br>(Additional information:<br>– the level of implementations of this legislation or regulation,<br>– data available on the impact of this legislation or regulation)	Yes	• In national disease control programs, antimicrobials may be allowed to be prescribed by the trained health workers, in particular cases such as Acute Respiratory Infection (ARI).<br>• Community Health Care Provider (CHCP) having 3-6 months of basic training on the management and treatment of common diseases are allowed to prescribe a list of antibiotics based on clinical signs and symptoms of patient.<br>• Before referral, midwives in specific emergency situation can prescribe antibiotics within their Scope of Practice and as per National Guidelines for Midwives.	• Antibiotic Use and Resistance in Bangladesh- Situation Analysis & Recommendations on Antibiotic Resistance, 2018, GARP-Bangladesh https://cddep.org/wp-content/uploads/2018/08/antibiotic-use-and-resistance-in-bangladesh.pdf<br>• National Guidelines for Midwives 2017, Directorate General of Nursing and Midwifery (DGNM) http://dgnm.portal.gov.bd/sites/default/files/files/dgnm.portal.gov.bd/page/18c15f9c_9267_44a7_ad2b_65affc9d43b3/2021-06-24-11-25-23141d2949e9295a21b4564983984047.pdf
**2.4b**	**Regulation and enforcement of prescription-only dispensing of antibiotics (WHO Essential National Core Elements for AMS Programming in LMICs -advanced)**			
2.4b.1	Legislation or regulation is actively implemented and enforced that requires antibiotics to be dispensed only on prescription by a qualified health-care professional (where access	Yes (partial)	• The sale of drugs without prescription is prohibited in Bangladesh, but not punishable under the National Drug Policy 2016.<br>• For this, a new act with the provision of penalty is now at the cabinet division for final approval.	• Interview: S. M. Sabrina Yesmin, Assistant Director, Directorate General of Drug Administration (DGDA)<br>• The Daily Start report "Germs winning over drugs" March 27.2022.

	Item for assessment	Yes / No	Comments	Source of Information/ Verifiers Used (Websites/ Interviews/ Reports etc.)
	to health care is not an issue).<br>(WHO NATIONAL CORE ELEMENT # 9b)<br>(Additional information:<br>– the basis of this enforcement: law/policy/ regulation/guideline)		• In proposed Drug Act it has been incorporated that selling antibiotic without prescription is a punishable offence and punishment is 20,000 BDT.	
**2.5**	**Measures in place to ensure continued availability of quality-assured antibiotics**			
2.5.1	The government acts to ensure that available antibiotics are of suitable quality and that substandard or falsified drugs are not being sold.<br>(WHO NATIONAL CORE ELEMENT # 10)<br><br>(Also, see section 5)	Yes	• DGDA as the Drug Control authority of the country acts to ensure that available antibiotics are of suitable quality and that substandard or falsified drugs are not being sold based on the 1940 Drug Act, Drug Control Ordinance 1982.<br>• Recently, to make the law more appropriate to meet the demand of time, a new law (Drug Act 2019) has been submitted to the cabinet division combining Drug Act of 1940 and Drug Control Ordinance 1982. Here, all offenses are divided into two types- cognizable offence and non-cognizable offence.<br>• Regulatory, administrative, and legal action taken for Good Manufacturing Practice (GMP) noncompliance, substandard and counterfeit manufacturers distributors, and sellers.<br>• Legal actions are taken through Drug court, magistrate court and mobile court. Special Power Act can also be applied in these types of offenses.<br>• The pharmaceuticals are regularly inspected for GMP compliance and post-marketing surveillance strictly followed. If any pharmaceuticals do not follow the specified criteria, there license is	• Interview: S. M. Sabrina Yesmin, Assistant Director, Directorate General of Drug Administration (DGDA)<br>• Interview: https://www.facebook.com/dgda.gov.bd/videos/atn/278810496693274/<br>• (Interview at ATN) Reports: DGDA Annual Report 2020-2021 http://dgdagov.info/index.php/about-dgda/annual-report<br>• World Antibiotics Awareness Week Report 202, USAID MTaPS

	Item for assessment	Yes / No	Comments	Source of Information/ Verifiers Used (Websites/ Interviews/ Reports etc.)
			cancelled. To date about 21 pharmaceuticals are suspended for not maintaining the standard.<br>• Besides these measures, people can sure of the authenticity of the drug by looking for the (Drug Administration Registration number) or DAR number and Marketing Authorization Number or MA number at the medicines' packages. This is applicable for all medicines including the antibiotics.<br>• The DGDA started the Model Pharmacy and Model Medicine Shop accreditation program to help ensure good pharmacy, dispensing, and storage practices. Model pharmacies are supervised by a graduate pharmacist, and 668 model pharmacies and 41,009 model medicine shops are entering the program.<br>• The pharmaceuticals companies of Bangladesh meet almost 98% demand of the local market. Now, Bangladesh is exporting to 157 countries of the world including UK, USA, Canada.	
2.5.2	The national medicines regulatory authority has tools for quality control/quality assurance and registration of antimicrobials in place.	Yes	• Quality ensured through WHO guidelines for 09 functions. These are –<br>– National Regulatory System (RS)<br>– Registration and Marketing Authorization (MA)<br>– Pharmacovigilance (PV)<br>– Market Surveillance and Control (MC)<br>– Licensing Premises (LI)<br>– Regulatory Inspection (RI)<br>– Laboratory Access and Testing (LA)<br>– Clinical Trial's Oversight (CTO)<br>– NRA Lot Release (LR)	• Interview: S. M. Sabrina Yesmin, Assistant Director, Directorate General of Drug Administration (DGDA)<br>• DGDA Annual report http://dgdagov.info/index.php/about-dgda/annual-report

	Item for assessment	Yes / No	Comments	Source of Information/ Verifiers Used (Websites/ Interviews/ Reports etc.)
2.5.3	Pharmacies are routinely being inspected and monitored to ensure compliance to laws and regulations with enforcement mechanisms in place.	Yes	• Pharmacies are inspected through divisional office, district office. Inspection report are submitted on daily basis.<br>• In 2020-2021, total 36,441pharmacies are inspected.	• Interview: S. M. Sabrina Yesmin, Assistant Director, Directorate General of Drug Administration (DGDA)<br>• DGDA Annual report http://dgdagov.info/index.php/about-dgda/annual-report
2.5.4	Data are available on the level of enforcement of the regulations.	Yes	• In 2020-2021, total law suits submitted through different courts as follows –<br>– Through drug court- 13<br>– Through magistrate court- 92<br>– Through mobile court- 1,715<br>– Total fine- BDT 7,58,00,100.00	• Interview: S. M. Sabrina Yesmin, Assistant Director, Directorate General of Drug Administration (DGDA)<br>• DGDA Annual report http://dgdagov.info/index.php/about-dgda/annual-report
2.5.5	Data are available on the impact of the regulations.	Yes	• 'Under control- this is the impact for a country like Bangladesh.'	• Interview: S. M. Sabrina Yesmin, Assistant Director, Directorate General of Drug Administration (DGDA)<br>• DGDA Annual report http://dgdagov.info/index.php/about-dgda/annual-report
**2.6**	**Measures in place to ensure affordability of essential antibiotics**			
2.6.1	The government acts to ensure that antibiotics are made available in suitable dosages (including Pediatrics formulations when appropriate) at a reasonable price to the public.<br>(WHO NATIONAL CORE ELEMENT # 11)	Yes	• DGDA ensures the affordability of all drugs including the antibiotics through Price Fixation Committee and Price Fixation Technical Subcommittee of DGDA.<br>• There are representation of Ministry of Health and Family Welfare, DGDA, DGHS, Bangladesh Pharmaceutical Society, Bangladesh Consumers' Association as members in these committee.<br>• There are separate defined process and formula of price fixation for drugs included in the primary health care, drugs other than those that are needed for primary health care and drugs that are imported.	• Interview: S. M. Sabrina Yesmin, Assistant Director, Directorate General of Drug Administration (DGDA)<br>• Price Fixation Committee http://dgdagov.info/index.php/about-dgda/committees/drug-pricing-committee/630-price-fixation-committee<br>• Price Fixation Technical Subcommittee http://dgdagov.info/index.php/about-dgda/committees/drug-pricing-committee/629-price-fixation-technical-sub-committee<br>• Updated National drug Policy 2016 http://dgdagov.info/index.php/information-

	Item for assessment	Yes / No	Comments	Source of Information/ Verifiers Used (Websites/ Interviews/ Reports etc.)
			• Suitable dosage forms (including Pediatrics formulations when appropriate) are available in the country.	center/guidance-documents/1136-national-drug-policy-2016-with-essential-drug-list-and-otc-list<br>• Supporting Documents –<br>➢ Process of price fixation
2.6.2	There is a system of monitoring antimicrobial sales throughout the country.<br>(Additional information:<br>– If yes, what guides the system?)	Yes	• Allocation of all types of medicines to government health facilities based on the ratio of bed versus patients and consumption of medicines in the previous year.<br>• In private sector monitoring of antimicrobial sales throughout the country internally analyzed for their marketing strategy department.	• Antibiotic Use and Resistance in Bangladesh- Situation Analysis & Recommendations on Antibiotic Resistance, 2018, GARP-Bangladesh https://cddep.org/wp-content/uploads/2018/08/antibiotic-use-and-resistance-in-bangladesh.pdf<br>• Interview: Maruf Hassan, Ex Senior Manager, Marketing Strategy Department; Incepta Pharmaceuticals
**2.7**	**Measures in place to monitor adverse drug reactions of essential antibiotics**			
2.7.1	A National Pharmacovigilance system is established to monitor adverse drug reactions of essential antibiotics<br>(Additional information:<br>– If yes, describe its activities over the past 12 months.<br>– If no, is there any plan to start to such system.)	Yes	• To promote public health by ensuring the safety, efficacy, and quality of medicines and other health products, MOH&FW declared the ADRM cell of DGDA as the National Pharmacovigilance Centre (NPC) for Bangladesh in 2013.<br>• An Adverse Drug Reaction Advisory Committee (ADRAC) formed by MOH&FW support the ADRM cell to provide technical guidance for implementing PV activities, evaluating adverse drug events (ADE) reports, and recommending regulatory decisions and actions to ensure medicine safety.<br>• Bangladesh has gained international recognition and access to early worldwide information about potential safety risks by becoming the 120th member of the World Health Organization's International Drug Monitoring Centre, known as the Uppsala Monitoring Centre (WHO-UMC).<br>• DGDA developed the National Guideline on Pharmacovigilance System in Bangladesh on 2018 with the assistance from USAID-funded SIAPS program, implemented by MSH.	• Interview: S. M. Sabrina Yesmin, Assistant Director, Directorate General of Drug Administration (DGDA)<br>• DGDA Annual Report 2020-2021 http://dgdagov.info/index.php/about-dgda/annual-report<br>• Updated National Drug Policy 2016 http://dgdagov.info/index.php/information-center/guidance-documents/1136-national-drug-policy-2016-with-essential-drug-list-and-otc-list<br>• National Guideline on Pharmacovigilance System in Bangladesh, Directorate General of Drug Administration, January 2018. http://dgdagov.info/index.php/publications/8-national-guideline-on-the-pharmacovigilance-system-in-bangladesh

	Item for assessment	Yes / No	Comments	Source of Information/ Verifiers Used (Websites/ Interviews/ Reports etc.)
			• As per DGDA annual report 2020-2021, 32 hospitals and 30 pharmaceuticals are submitting online ADR reports to National ADR cell till date.<br>• Once submitted, the reports are analyzed through WHO Causality Assessment Scale.<br>• ADRAC review the reports for making needful regulatory recommendations to the Licensing Authority (Drugs) for regulatory actions to protect public health from medicine's harm and then reports are uploaded in VigiFlow.<br>• There were two meetings of ADRAC held between January, 2020- June, 2021 where total 106 reports were evaluated and 49 reports were classified as likely/probable and 57 reports were classified as possible as per WHO Causality Assessment Scale.<br>• At present, MTaPS project of USAID is providing support to PV activities at DGDA.	
**2.8**	**A system of national independent drug information service**			
2.8.1	There is a system of national independent drug information service to reply to different queries related to antibiotics.<br>(Additional information:<br>– If yes, describe its activities over the past 12 months<br>– If no, is there any plan to start to such service.)	Yes	• There is a DGDA drug verification apps (developed during COVID-19) by which drugs can be verified through scanning of QR code. Drug related query or complains can also be submitted through this apps.	• Interview: S. M. Sabrina Yesmin, Assistant Director, Directorate General of Drug Administration (DGDA)<br>• National drug Policy 2016 http://dgdagov.info/index.php/information-center/guidance-documents/1136-national-drug-policy-2016-with-essential-drug-list-and-otc-list
**2.9**	**A system of regulating the promotional activities of pharmaceuticals companies**			

	Item for assessment	Yes / No	Comments	Source of Information/ Verifiers Used (Websites/ Interviews/ Reports etc.)
2.9.1	There are regulations and guidelines to regulate the promotional activities of pharmaceutical companies.<br>(Additional information:<br>– If yes, what regulations are in place<br>– Are any data available on the level of enforcement of the regulations?<br>– Are any data available on the impact of the regulations)	Yes (partial)	• Directorate of Drug Administration developed 'Code of Pharmaceutical Marketing Practices' on 1994.<br>• This document was developed based on Ethical Criteria for Medicinal Drug Promotion, WHO, Geneva; IFPMA Code of Pharmaceutical Marketing Practices, Code of Practice for the Pharmaceutical Industry, Association of British Pharmaceutical Industries, London after due consideration to the socio-economic, cultural and health care context of Bangladesh.<br>• 'The main objective of the Code of Pharmaceutical Marketing Practices is to support and encourage the improvement of health care through the rational use of medical substance.'<br>• 'The Code, therefore, represents an act of self-discipline and appeals to pharmaceutical manufacturer and distributors, the promotion industry, health personal involved in the prescription, dispensing, supply and distribution of drugs, universities and other teaching institutions, professional associations, patient and consumer groups, the professional and general media (including publishers and editors of medical journals and related publications), and the public.<br>• To use these criteria as appropriate to their spheres of competence, activity and responsibility.<br>• To adopt measures based on these criteria as appropriate, and monitor and enforce their standards.'<br>• Now DGDA has taken the initiative to update this document. A committee has been formed.	• Interview: S. M. Sabrina Yesmin, Assistant Director, Directorate General of Drug Administration (DGDA)<br>• Updated National drug Policy 2016 http://dgdagov.info/index.php/information-center/guidance-documents/1136-national-drug-policy-2016-with-essential-drug-list-and-otc-list<br>• Code of Pharmaceutical Marketing Practices, Directorate of Drug Administration, April 1994 https://www.researchgate.net/publication/207423861_Code_of_Pharmaceutical_Marketing_Practices

	Item for assessment	Yes / No	Comments	Source of Information/ Verifiers Used (Websites/ Interviews/ Reports etc.)
* Please refer to page 08 of the Antimicrobial stewardship in health-care facilities in low- and middle-income countries. A practical toolkit. Geneva: World Health Organization; 2019.Licence: CC BY-NC-SA 3.0 IGO https://apps.who.int/iris/handle/10665/329404 and pages 09-11 of the Tool to Support Conduct of Rapid Situational Analysis of Antimicrobial Stewardship Policies in MTaPS Program Countries, MSH, 2019				
3	**AWARENESS, TRAINING AND EDUCATION ***			
**3.1**	**Regular Public antibiotic awareness campaigns**			
3.1.1	Antibiotic awareness campaigns such as World Antibiotic Awareness Week and other targeted campaigns are regularly organized to address specific national or local issues and communities.<br>(WHO NATIONAL CORE ELEMENT # 12)<br><br>(Additional information:<br>– what kind of campaigns have been conducted and how frequently,<br>– who conducted these campaigns,<br>– are there education campaigns relating to infection prevention (e.g., hand hygiene, food hygiene, vector control, immunizations),<br>– are there education campaigns relating to appropriate use of antimicrobials/antibiotic	Yes	• WAAW celebrated every year on 18-24 November since 2015 in a coordinated way by different stakeholders.<br>• The theme of 2021 WAAW is 'Spread awareness, stop resistance'. CDC, DGHS, DGDA, FAO organized different programs covering both at the national level and also at the upazilla level.<br>• Some of the highlights of WAAW 2021 were advocacy workshop at DGDA, a roundtable discussion on AMR covered by one of the most circulated national daily *'Kalerkantha'*.<br>• SMS or Short Message Service was circulated nation-wide through Bangladesh Telecommunication Regulatory Authority (BRTA) to raise awareness on AMR.<br>• Awareness programs for medical college students were organized.<br>• In addition, the CDC held advocacy meetings in every upazila with active participation from human and animal health sectors<br>• Different IEC materials highlighting the day's theme were also developed.<br>• Several AMR awareness meetings in different districts took place during model pharmacy inauguration sessions organized by DGDA with participation of honorable health minister, state	• Interview: Dr. Aninda Rahman, DPM, CDC, DGHS<br>• Interview: S. M. Sabrina Yesmin, Assistant Director, Directorate General of Drug Administration (DGDA)<br>• National AMR Web Portal http://amr.cdc.gov.bd/<br>• Reports: https://youtu.be/UA-c934hTL4, https://www.edailysun.com/home/next_page/2#tab1, https://www.kalerkantho.com/online/national/2021/11/24/1095081, https://banglanews24.com/health/news/bd/894582.details<br>• Supporting Documents –<br>– WAAW report 2021, USAID MTaPS

	Item for assessment	Yes / No	Comments	Source of Information/ Verifiers Used (Websites/ Interviews/ Reports etc.)
	s and sensitization of AMR issues,<br>– are there any data on the impact of these measures)		ministers, local leaders and leaders of the Bangladesh Association of Pharmaceutical Industries and Bangladesh Chemist and Druggist Samity.<br>• To sensitize people on AMR issues, appropriate messages are scrolling on some TV channels.<br>• DGDA officers are taking part in different government development programs/fairs where they promote AMR containment and the appropriate use of antimicrobials by displaying posters.<br>• Rally organized at all field offices of DGDA on WAAW.<br>• Besides, as an FF fellow the concerned DGDA official already arranged awareness meeting in 08 divisions. Now a survey is conducted to know the level of understanding of antimicrobial dispensing and disposal<br>• AMR Web platform for show-casing all sorts of knowledge products with<br>• a one health approach developed with support from USIAD MTaPS program of MSH.<br>• AMR newsletter- 03 issues of AMR newsletter were published with one health approach with Fleming Fund funding.<br>• A regulatory decision was endorsed by the Drug Control Committee on 20th March, 2022 that "The manufacturers will put the red identification mark with the text 'Antibiotic' and the message 'Do not use without prescription of registered physician'. This decision was based on the findings of two prior studies-"Baseline survey on the level of awareness about antimicrobials & AMR and its impacts on self-medication in Bangladesh" and the	

	Item for assessment	Yes / No	Comments	Source of Information/ Verifiers Used (Websites/ Interviews/ Reports etc.)
			key informant interview "Rapid situation analysis of present labeling of antibiotics in Bangladesh and preference for label change patterns for incorporation of identification mark on Antimicrobial products and implementation challenges." DGDA has a plan to do an impact study of this regulatory measures.	
**3.2**	**Education in schools on basic infection principles**			
3.2.1	The government ensures that schools provide education on basic IPC principles, including hand hygiene.<br>(WHO NATIONAL CORE ELEMENT # 13)	Yes (Partial)	• Education on basic IPC principles including hand hygiene become evident and strengthened during COVID pandemic when schools were preparing to open. In government primary schools, around 71% schools have hand washing blocks.	• COVID-19 Response and Recovery Plan, Education Sector, Government of the People's Republic of Bangladesh, Ministry of Primary and Mass Education, May 2020. http://www.mopme.gov.bd/sites/default/files/files/mopme.portal.gov.bd/page/e2b6ac9f_d032_4634_835d_22b477eff4a2/COVID%2019%20Response%20and%20Recovery%20Plan_Govt.pdf
**3.3**	**Training on AMS competencies for AMS team members**			
3.3.1	The government and/or health-care facilities facilitate access to in-service training in antimicrobial prescribing and stewardship for AMS team members in facilities. Use existing core competencies and set standards or adapt curricula.<br><br>(WHO NATIONAL CORE ELEMENT # 14)	Yes (Partial)	• GO letter from QIS to form AMS committee and AMS team at all district hospitals of the country was issued on January, 2022 which yet to be fully implemented.<br>• Introductory training on the concept of AMS given to doctors of health facilities other than medical colleges of the country during dissemination of STG.<br>• USAID MTaPS program of MSH is supporting AMS program along with IPC program in 10 facilities. These are –<br>1. Comilla Medical College.<br>2. Munshiganj District Hoapital.<br>3. Nilphamari District Hospital.<br>4. Taraganj Upazilla Health Complex.	• Interview: Dr. Aninda Rahman, DPM, CDC, DGHS<br>• Supporting Documents –<br>– GO letter from QIS to form AMS committee and AMS team at all district hospitals of the country<br>– GO letter from CDC, DGHS for ToT on Standard Treatment Guideline (STG) on Antibiotic Use and Antimicrobial Stewardship (AMS)<br>– Schedule of the ToT on Standard Treatment Guideline (STG) on Antibiotic Use and Antimicrobial Stewardship (AMS)<br>– GO letter from CDC, DGHS for approving USAID MTaPS program for providing technical assistance for facility level IPC and AMS activities.

	Item for assessment	Yes / No	Comments	Source of Information/ Verifiers Used (Websites/ Interviews/ Reports etc.)
			5. Sher-e-Bangla Medical College<br>6. Jhalokati Sadar Hospital<br>7. Jhenaidah Sadar hospital<br>8. Satkhira Sadar Hospital<br>9. Lohagara Upazilla Health Complex<br>10. Impulse Hospital, Dhaka<br>AMS activities are at different level of implementation at these facilities.	
**3.4**	**Education and training for all health-care professionals on AMS**			
3.4.1	The government and/or other relevant bodies (e.g., professional societies) facilitate access to and/or support pre- and in-service training on how to optimize antibiotic prescribing, dispensing and administration for all relevant health-care professional groups (e.g., doctors, pharmacists, nurses). Use existing core competencies and set standards or adapt curricula (e.g., adaptation of the WHO core competencies and the AMR education and training curriculum guide).<br>(WHO NATIONAL CORE ELEMENT # 15)<br>(Additional information:<br>the frequency of trainings being conducted	Yes (partial)	• CDC is planning to develop a national guideline for AMS implementation. A consultant is appointed with support of USAID MTaPS and a working group is formed to facilitate the process with representation from QIS, Hospitals and clinics section of DGHS, Upazila Health Care OP of DGHS, DGDA, Bangladesh Society of Medical Microbiologists, USAID MTaPS, Flemming Fund Country Grant. CDC, the national focal point for ARC is coordinating the whole process.	• Interview: Dr. Aninda Rahman, DPM, CDC, DGHS<br>• GO letter for Working Group formation for developing National Guideline for AMS implementations

	Item for assessment	Yes / No	Comments	Source of Information/ Verifiers Used (Websites/ Interviews/ Reports etc.)
	availability of STGs in health facilities)			
3.4.2	It is very important to install the concept of STGs and hence AMS at the beginning of the professional career of health care professional. So, health students (medical, pharmacy, nursing, community health workers) trained on the importance of STG.	No	• Only theoretical aspect of AMR is included at the curricula of the health care professional.<br>• The concept of STGs and hence AMS and their importance are missing in the curricula.	• Interview: Dr. Aninda Rahman, DPM, CDC, DGHS
**3.5**	**Incentives to support implementation of AMS programmes in all health-care facilities, including staffing standards, training and accreditation (WHO Essential National Core Elements for AMS Programming in LMICs -advanced)**			
3.5.1	The government sets staffing standards for the AMS programme, makes implementation of AMS programmes in all facilities (government and private) a requirement, ensures that the health-care facility core elements are in place (e.g. by requiring certification/accreditation) and sets criteria to secure specific government funding for AMS in all facilities.<br>(WHO NATIONAL CORE ELEMENT # 16)	No	• AMS programming in the country now at too early stage. At this moment, there is no formal incentives from the government to support implementation of AMS programs in all health-care facilities.	• Interview: Dr. Aninda Rahman, DPM, CDC, DGHS<br>• Interview: Dr. Md. Akhteruzzaman, Program Manager (Joint Secretary), Health Economics Unit (HEU), Health Services Division, Ministry of Health and Family Welfare.

* Please refer to page 09 of the Antimicrobial stewardship programmes in health-care facilities in low- and middle-income countries. A practical toolkit. Geneva: World Health Organization; 2019.Licence: CC BY-NC-SA 3.0 IGO. https://apps.who.int/iris/handle/10665/329404 and pages 13 of the Tool to Support Conduct of Rapid Situational Analysis of Antimicrobial Stewardship Policies in MTaPS Program Countries, MSH, 2019

	Item for assessment	Yes / No	Comments	Source of Information/ Verifiers Used (Websites/ Interviews/ Reports etc.)
**4**	**SUPPORTING TECHNOLOGIES AND DATA***			
**4.1**	**National surveillance system on AMC in place**			
4.1.1	The government supports programs to compile and analyze appropriate data on the quantity and types of antibiotics purchased or distributed in the country (distinguishing between the health-care facility and community sector, if possible), following the WHO methodology on surveillance of AMC.<br>(WHO NATIONAL CORE ELEMENT # 17)	Yes	• DGDA is the national centre for Antimicrobial Consumption (AMC) and Antimicrobial Use (AMU) surveillance of Bangladesh.<br>• To monitor Antimicrobial Consumption (AMC)/ Antimicrobial Use (AMU)a taskforce titled "Taskforce to Monitor Antimicrobial Consumption/Use in Bangladesh" has been formed on March 2021. Chaired by the Director General of DGDA this taskforce with one health approach has representation from human health, animal health, fisheries and environment<br>• National Focal Point and Alternate National Focal Point for GLASS-AMC reporting have been nominated.<br>• The National Guideline on Antimicrobial Consumption Surveillance in Bangladesh has been approved in the taskforce meeting held on 12 June 2022. In this meeting 2019-2020 AMC surveillance data was presented.<br>• Prof. Sayedur Rahman and Dr. Kibria collected AMC data during 2016-2018. It was funded by WHO. This study was not disseminated and published. It was a base line survey. After that, DGDA started collecting AMC data with support of FF for 2019 and 2020 and is continued.	• Interview: S. M. Sabrina Yesmin, Assistant Director, Directorate General of Drug Administration (DGDA)<br>• Interview: Prof. Dr. Zakir Hossain Habib, Principal Scientific Officer, IEDCR<br>• Supporting Documents –<br>– Notification: Taskforce to Monitor Antimicrobial Consumption/Use in Bangladesh<br>– Official letter for nomination of National Focal Point and Alternate National Focal Point for GLASS-AMC reporting
**4.2**	**National surveillance system on AMR in place with laboratory capacity to guide optimal use of antibiotics in clinical practice and update clinical guidelines**			
4.2.1	Laboratory capacity is in place at the health-care facility or off-site (reference	Yes	• The national AMR surveillance strategy for 2020-2025 has been developed recently with support of The Fleming Fund grant. Institute of Epidemiology,	• Interview: Prof. Dr. Zakir Hossain Habib, Principal Scientific Officer, IEDCR

	Item for assessment	Yes / No	Comments	Source of Information/ Verifiers Used (Websites/ Interviews/ Reports etc.)
	laboratory) to identify pathogens and their antibiotic susceptibility, to guide optimal use of antibiotics in clinical practice and to update guidelines. The laboratory further supports identification of key pathogens or syndromes to target AMS interventions. The government supports programmes to collate, compile and compare data from different facilities to identify trends over time and possibly to identify facilities that are outliers and might warrant investigation and assistance.<br>(WHO NATIONAL CORE ELEMENT # 18)		Disease Control and Research (IEDCR) and Department of Livestock Services (DLS) has been identified as the focal institute to monitor and coordinate the AMR surveillance in the human health and animal health sector in the country respectively.<br>• Since 2017, the IEDCR has been conducting countrywide AMR surveillance, beginning with five sentinel sites, then scaling up to nine, with the support of the Government of Bangladesh (GoB), the Global Health Security Agenda (GHSA), and the World Health Organization (WHO).<br>• There is a trained team at IEDCR with representation from ARC for AMR surveillance which is a standardized active prospective surveillance. There are designated staffs at all sites who use a form to collect case-based sample. There are pre-defined priority samples and priority pathogens. The samples are tested for AST (Anti-biotic Susceptibility Testing) in the local attached lab. Some samples also tested at IEDCR for external quality assessment (EQA).<br>• This national data of AMR surveillance is entered into the data base of IEDCR. There is yearly dissemination of this data to all stakeholders (For example, Public Health Specialists, Clinicians, Development Partners). There is also a web-based real time data sharing from IEDCR.<br>• Internationally, as a signatory, Bangladesh is providing data to GLASS (Global Antimicrobial Resistance and Use Surveillance System).<br>• In future IEDCR plans to increase the number of sentinel sites from 09 to 11 for a better representation of the country.	• National Strategy and Action Plan for Antimicrobial Resistance Containment in Bangladesh [2021-2026]<br>• The National Antimicrobial Resistance (AMR) Surveillance Strategy of Bangladesh [2020-2025]<br>• Findings from AMR surveillance in Bangladesh, a presentation by Dr. Zakir Hossain Habib; Principal Scientific Officer (Associate Professor), Department of Microbiology, Member Secretary, AMR Surveillance Coordination Sub-Committee; Institute of Epidemiology, Disease control & Research (IEDCR).<br>• Global Anti-microbial Resistance and Use Surveillance System (GLASS) Report, Early Implementation 2020, WHO https://apps.who.int/iris/bitstream/handle/10665/332081/9789240005587-eng.pdf<br>• Disease Surveillance System of Bangladesh: Combating Public Health Emergencies ISDS 2019 Conference Abstract https://www.ncbi.nlm.nih.gov/pmc/articles/pmc6606310/<br>• Website: https://iedcr.gov.bd/pages/amr<br>• Supporting Documents –<br>– Government notification of NRL for AMR surveillance program.<br>– Epi-data Collection Form of AMR surveillance

	Item for assessment	Yes / No	Comments	Source of Information/ Verifiers Used (Websites/ Interviews/ Reports etc.)
			• There are Sectoral Working Groups to coordinate the activities of surveillance. Each Sectoral Working Group (SWG) will work as a forum of the surveillance sites, laboratory networks, and reference laboratories within the sector. They will work closely to facilitate joint-learning and problem solving.<br>• A state-of-the-art National Reference Laboratory has been established at IEDCR premises with support from Fleming Fund which participated in 03 rounds of EQAsia External Quality Assessment Systems (EQAS) in 2021.	
**4.3**	**Diagnostic tests available and capacity building undertaken to optimize antibiotic use (WHO Essential National Core Elements for AMS Programming in LMICs -advanced)**			
4.3.1	Governments encouraged to procure and promote the use of relevant diagnostic tests to optimize antibiotic use. The government acts to ensure that relevant and essential investigations (e.g. biology, microbiology, imaging) are available for all health-care facilities (either on-site, or with available access off-site).<br>(WHO NATIONAL CORE ELEMENT # 19)	Yes (Partial)	• Although, Bangladesh's health system's infrastructure is commendable; there is lack of facilities for some essential investigations for AMS like culture sensitivity test. At present, AST facility available at all medical college hospitals and only a few district hospitals.<br>• Most of the district hospitals and upazilla level health facilities do not have AST facilities.	• Seminar organized by Bangladesh Society of Medical Microbiologists (BSMM), BSMMU, May 27, 2022.
* Please refer to page 09 of the Antimicrobial stewardship programmes in health-care facilities in low- and middle-income countries. A practical toolkit. Geneva: World Health Organization; 2019.Licence: CC BY-NC-SA 3.0 IGO. https://apps.who.int/iris/handle/10665/329404 and pages 09-13 of the Tool to Support Conduct of Rapid Situational Analysis of Antimicrobial Stewardship Policies in MTaPS Program Countries, MSH, 2019				
**5**	**SUPPLY MANAGEMENT POLICIES***			
**5.1**	**Antimicrobials (Antibiotics) quality assurance system**			

	Item for assessment	Yes / No	Comments	Source of Information/ Verifiers Used (Websites/ Interviews/ Reports etc.)
5.1.1	*Standard Quality Assurance Mechanisms*** are in place in the government sector to ensure the quality of antimicrobials marketed in the country.<br>(Additional information:<br>– Percentage of government laboratories that are capable of testing antimicrobial quality,<br>– types of quality assurance mechanisms that are in place<br>– the extent of enforcement of the above mechanisms/regulations)	Yes	• To ensure the quality of antimicrobials marketed in the country no separate mechanisms for antimicrobials; common policy for all medicines.<br>• The government sector source antibiotics mainly from the state-owned Essential drugs Company Limited (EDCL). Government can also source from private pharmaceutical companies through competitive bids.<br>• After MoH&FW approval, requisitions from all the line directors are forwarded to CMSD to be filled and distributed to health facilities at various level of health system countrywide.<br>• In the RFQ, for vendor selection and evaluation, there is requirement of certain prequalification of suppliers. The total procurement policy follows Public Procurement Act, 2006 and Public Procurement Guidelines, 2008 (updated on February 2021). In this guideline, there are many in-built standard procurement documents with specific criteria which guide to select qualified suppliers. Some of these pre-requisites are- the pharmaceutical company and the product must be registered in the country with DGDA (at their own country in case of international procurement), must have Certificate of Pharmaceutical Product (COPP), must have GMP certification as per WHO, have some lab analysis report as required. There are specific procurement forms coded as PG1, PG2 and PG3 available from Central Procurement Technical Unit (CPTU) of Ministry of Planning which are used for procurement of product below 5 lacs, below 25 lacs and above 25 lacs respectively.<br>• After receiving goods at the warehouse following standard operating procedures, DD CMSD serves	• Interview: Md. Anwarul Islam, Senior Technical Advisor-Procurement, USAID MTaPS<br>• Central Medical Stores Depot http://cmsd.gov.bd/<br>• Central Procurement Technical Unit https://cptu.gov.bd/<br>• Warehouse Operating Procedures, CMSD, DGHS, MOH&FW, October 2015. https://siapsprogram.org/wp-content/uploads/2018/03/15-317-CMSD_SOPs.final2_24-10-16.pdf<br>• Antibiotic Use and Resistance in Bangladesh- Situation Analysis & Recommendations on Antibiotic Resistance, 2018,The GARP-Bangladesh National Working Group https://cddep.org/wp-content/uploads/2018/08/antibiotic-use-and-resistance-in-bangladesh.pdf

	Item for assessment	Yes / No	Comments	Source of Information/ Verifiers Used (Websites/ Interviews/ Reports etc.)
			notification to Head of Survey Committee and other members scheduling a date for the survey. Survey committee issues Survey Report checking against Invoice, forwarding docs and technical specs. Then Survey Report sent to Director CMSD for approval.<br>• For quality assurance of medicine, visual inspection of packaging, labelling and physical appearance (at time of receipt at all levels of supply chain), basic rapid quality tests using portable minilab systems, post-purchase testing at Drug Testing Laboratory also done as required. Some sophisticated tests are done through CRO.<br>• Monitoring of the marketed product is done by DGDA.	
5.1.2	*Standard Quality Assurance Mechanisms*** are in place in the private sectors to ensure the quality of antimicrobials marketed in the country.<br>(Additional information:<br>– Percentage of private laboratories that are capable of testing antimicrobial quality,<br>– types of quality assurance mechanisms that are in place,<br>– the extent of enforcement of the above mechanisms/ regulations)	Yes	• In private sector, no separate mechanisms for antimicrobials to ensure the quality of the marketed products in the country; common mechanisms followed like all other medicines.<br>• The supplier or importers need to be registered through DGDA. The product also needs to be registered. Two types of drug inclusion in the DGDA list.<br>1. New generic drug that is the first time in Bangladesh<br>– If the product is INN, in the registration process, need to submit the drug sample for testing in the drug testing laboratory<br>– If the product is BP, USP or FDA approved, the drug sample will be provided for registration but it is not necessary to test in the drug testing laboratory<br>2. New brand of existing generic	• Interview: Maruf Hassan, Ex Senior Manager, Marketing Strategy Department; Incepta Pharmaceuticals

	Item for assessment	Yes / No	Comments	Source of Information/ Verifiers Used (Websites/ Interviews/ Reports etc.)
			– Just provide quality analysis data along with the registration form<br>– No need to test<br>• Specific drug reports requested, such as bioavailability<br>• Not possible by the govt./DGDA<br>• None in the private sector has done this<br>• If someone wants to do bioequivalence test for their export market, they will do it outside from Bangladesh through third party<br>• In Bangladesh, only BP, USP, INN or FDA included products are approved for registration. Thus, if a drug has been registered in the DGDA or has a DAR no., no need to check its pharmacopoeia standard.<br>• Every pharmaceutical company possesses Good Manufacturing Practice (GMP) certification. As per the regulation, without GMP certification, no pharmaceutical company will manufacture drugs. Thus, every company in the pharmaceuticals sector has this certification. Usually, every year DGDA visit pharmaceutical factory to check this. If the company has GMP certification but does not follow the instruction in the GMP, the DGDA authority has right to close the factory<br>• In the production process, one or two strip (10/20 tablets) from each batch (10,000 tablets) has been preserved in the quality assurance department of each pharmaceutical. If there is a complain of any drug in the market, they will collect the sample from the market, match the batch number with their preserved products and then make a quality test of it so that they can provide explanation to	

	Item for assessment	Yes / No	Comments	Source of Information/ Verifiers Used (Websites/ Interviews/ Reports etc.)
			the complaint and take necessary action to overcome it.<br>• In November 2018 DGDA started implementation of Risk-Based Post-Marketing Surveillance (RB-PMS) with technical support from USAID funded program USP-PQM. RB-PMS focuses on sampling considering the most vulnerable risk factors i.e. top selling sample, geographical area, hard to reach area, new product, product having stability issue, costly products, disease, season etc. that may affect the medicine quality and prioritize testing those products to find out SF products and subsequent legal action to ensure quality products in the market with the limited resource in a cost-effective way.	
5.1.3	Data on the proportion of antimicrobials that were substandard in the last two years are available.	Yes	In DGDA Annual Report, from July 2020 to June 2021, total 353 tests performed and among them 08 were substandard. (Mini Lab Report for all medicines).	• DGDA Annual report http://dgdagov.info/index.php/about-dgda/annual-report
5.1.4	Documented reports of falsified antimicrobials are available.	Yes	Documented reports of falsified antimicrobials are available with the DGDA.	• Interview: S. M. Sabrina Yesmin, Assistant Director, Directorate General of Drug Administration (DGDA)
**5.2**	**Utilization of the Essential Medicine List (EML)**			
5.2.1	There is a policy of using the EML antimicrobial list for procurement of antimicrobials. (If yes, ask the follow-up questions listed below starting from 5.2.1 A to 5.2.1 D.	No	• The EML of Bangladesh was last updated on 2016.<br>• The EML list contains only few medicines and most of the pharmaceuticals don't want to promote these antimicrobials due to controlled price.<br>• Supply chain activities within DGHS involve a mix of 'push' activities managed centrally or 'pull' activities based on customer demands.	• Interview: Maruf Hassan, Ex Senior Manager, Marketing Strategy Department; Incepta Pharmaceuticals<br>• Comprehensive Assessment for the Government of Bangladesh, Directorate General of Health Services, Central Medical Stores Depot, December 2010. https://scmpbd.org/index.php/documents/scip/technical-reports/62-comprehensive-assessment-for-the-cmsd-dghs/file

	Item for assessment	Yes / No	Comments	Source of Information/ Verifiers Used (Websites/ Interviews/ Reports etc.)
5.2.1. A	This policy exists in the government sector	N/A	• N/A	• N/A
5.2.1. B	This policy exists in the private sector	N/A	• N/A	• N/A
5.2.1.C	There is data on the level of adherence to the policies at the government sector	N/A	• N/A	• N/A
5.2.1 D	There is data on the level of adherence to the policies at the private sector	N/A	• N/A	• N/A
5.2.2	There is data on antimicrobial consumption by value and volume at country level	Yes	From National AMC Surveillance Data –<br>– 2016 (16.65 DDD per 1000 inhabitants/day),<br>– 2017 (19.87 DDD per 1000 inhabitants/day),<br>– 2018 (21.78 DDD per 1000 inhabitants/day),<br>– 2019 (24.74 DDD per 1000 inhabitants/day),<br>– 2020 (25.37 DDD per 1000 inhabitants/day.)	• Interview: S. M. Sabrina Yesmin, Assistant Director, Directorate General of Drug Administration (DGDA)
5.2.3	There is data on antimicrobial consumption by value and volume in a national hospital level	Yes (Partial)	• The International Vaccine Institute (IVI) and the Communicable Disease Control program under the Directorate General of Health Services, Ministry of Health, and Family Welfare (MOH&FW) of Bangladesh shared key findings from the Capturing Data on Antimicrobial Resistance Patterns and Trends in Use in Regions of Asia (CAPTURA) project on May 18, 2022.<br>• CAPTURA is an IVI-led initiative to increase the volume and quality of data on antimicrobial resistance (AMR), consumption (AMC), and use (AMU) in South and Southeast Asia - funded by The Fleming Fund, a UK Aid programme working in partnership with Mott MacDonald.	• Press release on the dissemination event by CDC, DGHS

	Item for assessment	Yes / No	Comments	Source of Information/ Verifiers Used (Websites/ Interviews/ Reports etc.)
			• The project resulted in the following key accomplishments:<br>– Identification of historical and current data on AMR, AMC, and AMU<br>– Collation and analysis of 4 years of retrospective AMR/U data from 34 laboratories (11 public and 23 private) and 5 pharmacies (all private model pharmacies)<br>– Comprehensive capacity-building activities to optimize local data management practices and encourage data sharing at the national, regional, and global levels<br>– More than one million records of AST data were identified and digitized, where necessary, collected, and analyzed<br>– E. coli, Klebsiella, Pseudomonas, Staphylococcus aureus, Enterococcus, Salmonella Typhi and Acinetobacter spp. were the most frequently isolated bacteria<br>– No major change in susceptibility patterns were observed in E. coli and Staphylococcus aureus in the country over the last 4 years<br>– Multidrug Resistance (MDR), possible Extensive Drug Resistance (XDR), and Pan-Drug<br>– Resistance (PDR) is prevalent and needs close monitoring to prevent potential spread<br>– WHO Global Priority List of Antibiotic-resistant Bacteria were also observed to be prevalent in some settings in the country<br>– A gap in internal and external quality assessment has been observed in majority of the labs, which needs to be addressed to	

	Item for assessment	Yes / No	Comments	Source of Information/ Verifiers Used (Websites/ Interviews/ Reports etc.)
			ensure validity of AMR data being generated in the country<br>– The use of WATCH group and RESERVE group of antibiotics is found to be much higher (70%-88%) than the recommended use (within 40%) which is alarming	
*Please refer to page 12 of the Tool to Support Conduct of Rapid Situational Analysis of Antimicrobial Stewardship Policies in MTaPS Program Countries, MSH, 2019<br>** Standard Quality Assurance Mechanisms** include –<br>a. Prequalification of suppliers<br>b. Supplier submits registration form<br>c. Physical inspection of drug samples<br>d. Laboratory analysis of drug samples<br>e. Specific drug reports requested, such as bioavailability<br>f. Informal information gathered from other procurement programs<br>g. Tender/order documents specify which pharmacopeia standards are acceptable<br>h. Good Manufacturing Practice certification<br>i. Monitoring of quality of the marketed product<br>j. Inspection at the point of importation<br>k. Inspection at retail/wholesale pharmacies<br>l. Lab testing of the samples obtained from drug outlets<br>m. Supplier performance M&E<br>n. Other (describe)				
**6**	**REPORT/STUDIES ON THE LEVEL OF APPROPRIATE USE BY PRESCRIBERS, DISPENSERS AND PATIENTS***			
**6.1**	**Report or studies on the level of appropriateness of antimicrobial prescribing**			
6.1.1	Report or studies exists on the level of appropriateness of antimicrobial prescribing at the national level.	Yes	• A point prevalence survey was conducted in four acute care government hospitals (two tertiary and two secondary level hospitals) selected purposively in 2 administrative divisions of Bangladesh from 18 February to 6 April 2021. This survey on AMU among inpatients was the part of a larger project	• Pattern of Antibiotic Use among Hospitalized Patients according to WHO Access, Watch, Reserve (AWaRe) Classification: Findings from a Point Prevalence Survey in Bangladesh. https://www.mdpi.com/2079-6382/11/6/810/htm

	Item for assessment	Yes / No	Comments	Source of Information/ Verifiers Used (Websites/ Interviews/ Reports etc.)
			having the objectives of understanding antimicrobial use in humans (at hospitals, communities, and pharmacies), commercial chicken, and aquaculture in Bangladesh through the 'One Health' approach. To assess the extent of antimicrobial prescribing patterns among the hospitalized patients at different wards of survey hospitals, a structured survey questionnaire was adapted from WHO and Global-PPS design. The survey was conducted among inpatients of medicine, surgery, gynae and obstetrics, and pediatric wards including intensive care units. Total 1417 patients were enrolled-52% were female and 63% were from the 15–64 years age group. Nearly 78% of patients received at least one antibiotic during the survey period. Third-generation cephalosporins (44.6%), penicillins (12.3%), imidazoles (11.8%), aminoglycosides (7.2%), and macrolides (5.8%) were found to be highly used antibiotics. Overall, 64.0% of Watch, 35.6% of Access, and 0.1% of Reserve group antibiotics were used for treatment. The use of Watch group antibiotics was high in medicine wards (78.7%) and overall high use of Watch antibiotics was observed at secondary hospitals (71.5%) compared to tertiary hospitals (60.2%) (p-value of 0.000). Findings of this PPS, underscore the need for an urgent nationwide antibiotic steward- ship program for physicians including the development and implementation of local guidelines and in-service training on antibiotic use.	
6.1.2	Report or studies exists on the level of appropriateness of antimicrobial prescribing at private level.	Yes	• A study (published on 2017) was conducted by collecting samples of prescription of private practitioners from patients randomly. Mode of collection was copying of prescriptions by photocopy or by digital camera. Patients consent was taken. All the prescriptions were analyzed	• Pattern of Prescription of Private Practitioners in Bangladesh<br>• https://doi.org/10.3329/jdmc.v26i1.34001<br>•

	Item for assessment	Yes / No	Comments	Source of Information/ Verifiers Used (Websites/ Interviews/ Reports etc.)
			according to International Network of Rational Use of Drugs (INRUD) indicators. Total 500 prescriptions were analyzed using WHO formula to study prescriptions.<br>1. Average number of drugs per prescription 4.40%<br>2. Percentage of drugs prescribed by generic name 0.30%<br>3. Percentage of prescriptions with an antibiotic prescribed 72 %<br>4. Percentage of prescriptions with an injection prescribed 10%<br>5. Percentage of drug prescribed from essential drug list 48%<br>6. Whether prescription is complete with respect to –<br>a. Format 40%<br>b. Dosage and duration 74 %<br>c. Patient medical information 23%<br>• From this study it was revealed that prescribing pattern of the private practitioners were irrational regarding polypharmacy, generic name, use of antibiotics, drug selection from EDL and providing medical information.	
**6.2**	**Report or studies on the level of appropriateness of antimicrobial dispensing**			
6.2.1	There are reports or studies on the level of appropriateness of antimicrobial dispensing at the national level	Yes	• DGDA conducted a study on "Baseline survey on level of awareness about antimicrobials and AMR and its impacts on self-medication in Bangladesh" on 08 divisions of Bangladesh. This study found that 67.3 % of pharmacy retailers do not have the knowledge about antimicrobial drugs and it's not	• Interview: S. M. Sabrina Yesmin, Assistant Director, Directorate General of Drug Administration (DGDA)

	Item for assessment	Yes / No	Comments	Source of Information/ Verifiers Used (Websites/ Interviews/ Reports etc.)
			easy for them to identify the antibiotics. The findings of this study shared on taskforce meeting held on 12 June,2022.	
6.2.2	Report or studies exists on the level of appropriateness of antimicrobial dispensing at the private level	Yes	• This study aimed to explore antibiotic dispensing pattern in pharmacies according to the WHO AWaRe classification. From January to July 2021, Drug-sellers from randomly selected pharmacies and randomly selected customers attending the pharmacies were interviewed. Data were collected on demographics and medicines purchased. The purchased antibiotics were classified into the Access, Watch, and Reserve groups. Among 128 pharmacies surveyed, 98 (76.6%) were licensed; 61 (47.7%) drug-sellers had pharmacy training. Of 2686 customers interviewed 580 (21.6%) purchased antibiotics. Among the 580 customers, 523 purchased one, 52 purchased two, and 5 purchased three courses of antibiotics (total 642 courses). Of the antibiotic courses, the Watch group accounted for the majority (344, 53.6%), followed by the Access (234, 36.4%) and Reserve (64, 10.0%) groups. Approximately half of the antibiotics (327/642, 50.9%) were purchased without a registered physician's prescription. Dispensing of non-prescribed antibiotics was higher in the Access group (139/234, 59.4%), followed by Watch (160/344, 46.5%) and Reserve (28/64, 43.8%) groups. These findings highlight the need to implement strict policies and enforce existing laws, and pharmacy-targeted antimicrobial stewardship focusing on proper dispensing practices to mitigate antimicrobial resistance in Bangladesh.	• Pattern of Antibiotic Dispensing at Pharmacies According to the WHO Access, Watch, Reserve (AWaRe) Classification in Bangladesh. https://www.mdpi.com/2079-6382/11/2/247
**6.3**	• **Report or studies on the level of appropriateness of antimicrobial usage by the public**			
6.3.1	There are reports or studies on the level of	Yes	• A survey project has been designed to conduct nationwide to explore the pattern of antibiotics use	• Pattern of Antibiotics use at the Primary Health Care Level of Bangladesh

	Item for assessment	Yes / No	Comments	Source of Information/ Verifiers Used (Websites/ Interviews/ Reports etc.)
	appropriateness of antimicrobial usage by the public at the national level		at the primary health care level in Bangladesh using carefully constructed questionnaires containing questions covering both antibiotic prescription habit of the physicians and patients' response to antibiotic use. In the first phase of this effort, 20 Upazila Health Complexes and the Union Health Centres thereunder each of Dhaka and Chittagong division were randomly surveyed. It was evident from patient survey that cold, fever and acute respiratory infection (ARI) were prevalent (39.78%) causes that brought the patients to physician. 60.1 % of the patients reported that they get essential antibiotics from hospital free of cost while 24.5% of them mentioned that they do not get antibiotics from hospital. 34.76% of the patients reported that they complete the course of antibiotic therapy by buying antibiotics from the market whereas 56.14% of them do not buy the full course and stop taking medicine (53.46%) as soon as symptoms subside. The results of the present survey indicate that antibiotics are widely and inappropriately used without following standard guidelines or based on any rationality. This is an alarming situation, which should be properly taken care of by the relevant authority to save the people from growing antibiotic resistance.<br>• The first phase of this survey findings was published on 2009.	https://www.banglajol.info/index.php/SJPS/article/view/5809
6.3.2	Report or studies exists on the level of appropriateness of antimicrobial usage by the public at the private level	Yes	• A mobile phone survey among a general population across eight administrative divisions of Bangladesh was conducted during January–March 2021. Reported illness episodes irrespective of COVID-19 in the preceding four weeks of the interview, use of antibiotics for the illnesses, and knowledge on antibiotics among the general population were recorded. Descriptive analyses were performed.	• Antibiotics Use and Its Knowledge in the Community: A Mobile Phone Survey during the COVID-19 Pandemic in Bangladesh. https://www.mdpi.com/2079-6382/10/9/1052

	Item for assessment	Yes / No	Comments	Source of Information/ Verifiers Used (Websites/ Interviews/ Reports etc.)
			Total 1854 participants were randomly interviewed, with a mean age of 28.5 years (range: 18–75 years); 60.6% were male. Among all participants, 86.3% (95% CI: 84.7–87.8) heard names of antibiotics (which was only 48% as per a study finding before the pandemic), but only 12.1% reported unspecified harmful effects, and 3.5% reported antimicrobial resistance when antibiotics were taken without a physician's prescription. Among 257 (13.9%) participants, who consumed medicines for their recent illness episode, 32.7% (95% CI: 27.2–38.6) reported using antibiotics. Of those who could recall the names of antibiotics prescribed (n = 36), the most frequently used was azithromycin (22.2%) followed by cefixime (11.1%) and ciprofloxacin (5.6%). This study exposed an increased antibiotic use for illnesses reported in the preceding four weeks and an elevated knowledge at the community level during the COVID-19 pandemic compared with the pre-pandemic period.	

* Please refer to page 12 of the Tool to Support Conduct of Rapid Situational Analysis of Antimicrobial Stewardship Policies in MTaPS Program Countries, MSH, 2019

APPENDIX 03: GO LETTER (WORKSHOP)

গণপ্রজাতন্ত্রী বাংলাদেশ সরকার
রোগ নিয়ন্ত্রণ শাখা
স্বাস্থ্য অধিদপ্তর
মহাখালী, ঢাকা-১২১২

স্মারক নং- স্বাঃঅধিঃ/রোঃনিঃ/এআরসি/২০২২/ ২৮৬৫

তারিখঃ ১১ আগস্ট, ২০২২ইং

বরাবর
....
....

বিষয়ঃ এন্টিমাইক্রোবিয়াল স্টুয়ার্ডশিপ বিষয়ক কর্মশালায় অংশগ্রহণ প্রসঙ্গে।

উপরোক্ত বিষয়ের প্রেক্ষিতে জানানো যাচ্ছে যে, সারা বিশ্বে এন্টিমাইক্রোবিয়াল রেজিস্ট্যান্স একটি গুরুতর জনস্বাস্থ্য সমস্যা হিসেবে দেখা দিয়েছে। জীবাণুসমূহ এন্টিবায়োটিকের প্রতি তাদের প্রতিরোধ ক্ষমতা বৃদ্ধি করেই চলেছে। উপরন্তু, এন্টিবায়োটিকের যথেচ্ছ, অনিয়ন্ত্রিত ও অযৌক্তিক ব্যবহার বিভিন্ন ধরণের এন্টিবায়োটিক রেজিস্ট্যান্স এর জন্য দায়ী। আপনারা অবগত আছেন যে, বিশ্ব স্বাস্থ্য সংস্থা বিশ্বব্যাপী জনসচেতনতা বৃদ্ধিসহ এন্টিবায়োটিকের যথাযথ ব্যবহার নিশ্চিতকরনের লক্ষ্যে পলিসি ও নির্দেশিকা প্রনয়ণসহ বিভিন্ন ধরনের কার্যক্রম চালিয়ে যাচ্ছে এবং টেকসই উন্নয়ন লক্ষ্যমাত্রা'২০১৬ (Sustainable Development Goal'2016) এ এন্টিমাইক্রোবিয়াল রেজিস্ট্যান্স সম্পর্কিত লক্ষ্যমাত্রা নির্ধারণ করা হয়েছে। স্বাস্থ্য অধিদপ্তরের রোগ নিয়ন্ত্রণ শাখা বিষয়টি গুরুত্বের সাথে গ্রহণ করে ইতিমধ্যেই এ বিষয়ে কৌশলপত্র, কর্মপরিকল্পনা, চিকিৎসা গাইডলাইন, আইপিসি প্রোগ্রাম, প্রশিক্ষণ, এন্টিমাইক্রোবিয়াল স্টুয়ার্ডশিপ সহ অন্যান্য কার্যক্রম পরিচালনা করছে। এরই ধারাবাহিকতায়, এন্টিমাইক্রোবিয়াল স্টুয়ার্ডশিপ বিষয়ক National assessment রিপোর্ট পর্যালোচনা এবং দেশের হাসপাতালগুলোর জন্য এন্টিমাইক্রোবিয়াল স্টুয়ার্ডশিপ গাইডলাইন প্রণয়নের লক্ষ্যে এন্টিমাইক্রোবিয়াল রেজিস্ট্যান্স কন্টেইনমেন্ট এর কোর ওয়ার্কিং গ্রুপের সদস্যদের অংশগ্রহণে আগামী ১৬/০৮/২০২২ ইং তারিখে একটি কর্মশালা আহ্বান করা হয়েছে। উক্ত কর্মশালায় উপস্থিত থেকে আপনার মূল্যবান মতামত প্রদানের জন্য অনুরোধ জানানো যাচ্ছে।
উক্ত কর্মশালায় USAID MTaPS কারিগরি সহায়তা প্রদান করবে ।
উল্লেখ্য, অংশগ্রহণকারীগণ HPNSP অনুযায়ী ভাতাদি প্রাপ্য হবেন।

ভেন্যুঃ কনফারেন্স রুম (২ তলা),স্বাস্থ্য অধিদপ্তর (পুরাতন ভবন), মহাখালী, ঢাকা।
তারিখঃ ১৬/০৮/২০২২ ইং **সময়ঃ** সকাল ১০.০০ – দুপুর ২.০০ ঘটিকা

প্রয়োজনে যোগাযোগঃ
ডাঃ অনিন্দ্য রহমান,
ডিপিএম, এআরসি, ভাইরাল হেপাটাইটিস ও ডায়রিয়া নিয়ন্ত্রণ প্রোগ্রাম।
মোবাইলঃ ০১৮১৭৫৪১৭৯৭ ইমেইলঃ dr.turjossmc@gmail.com

অধ্যাপক ডা. মো: নাজমুল ইসলাম
পরিচালক, রোগ নিয়ন্ত্রণ ও
লাইন ডাইরেক্টর, কমিউনিকেবল ডিজিজ কন্ট্রোল
স্বাস্থ্য অধিদপ্তর, মহাখালী, ঢাকা
ফোন নংঃ ৯৮৮০৯৪৮, ফ্যাক্সঃ ৯৮৯৯০৮৫

সদয় অবগতির জন্য প্রেরণ করা হলঃ (জ্যেষ্ঠতার ক্রমানুসারে নয়)

১. মহাপরিচালক, স্বাস্থ্য অধিদপ্তর, মহাখালী, ঢাকা। (দৃষ্টি আকর্ষণঃ সহকারী পরিচালক (সমন্বয়)।
২.মহাপরিচালক, স্বাস্থ্য শিক্ষা অধিদপ্তর, মহাখালী, ঢাকা।
৩. অতিরিক্ত মহাপরিচালক (প্রশাসন), স্বাস্থ্য অধিদপ্তর, মহাখালী, ঢাকা।
৪. অতিরিক্ত মহাপরিচালক (পরিকল্পনা ও উন্নয়ন), স্বাস্থ্য অধিদপ্তর, মহাখালী, ঢাকা।
৫. উপ-পরিচালক, সিডিসি ,স্বাস্থ্য অধিদপ্তর ,মহাখালী ,ঢাকা।
৬. পরিচালক/অধ্যক্ষ /তত্ত্বাবধায়ক.......................................
৭. ডিপিএম, এআরসি,ভিএইচ ও ডায়রিয়া প্রোগ্রাম ,সিডিসি ,স্বাস্থ্য অধিদপ্তর ,মহাখালী ,ঢাকা।
৮. অফিস কপি।

APPENDIX 04: SCHEDULE OF WORKSHOP

Government of the People's Republic of Bangladesh

Workshop on National Multisectoral AMS Plan

Venue: Conference room, 1st floor, Old DGHS building, DGHS, Mohakhali.

Date: 16./08/2022

Time	Topics/activities	Resource person
09:30-10:00 am	Registration	
10:00-10:20 am	Welcome Speech	Prof Dr Md Nazmul Islam, Director, Disease Control & Line Director, CDC, DGHS.<br><br>Dr. Md Shafiqul Islam Deputy Director, CDC, DGHS.
10.20-10.30 am	Self –Introduction	
10:30 -11:00am	Sharing objectives of the Workshop	Dr. Aninda Rahman/ Dr. S M Shahriar Rizvi/Dr. Piash Kumer Deb/ Dr. Hurul jannat
11.00-11:15am	Snacks Break	
11:15-11:30	Presentation on "Assessment of National Level AMS Policies and Practices in Human Health"	Dr. Nazia Amin Salsabil , Consultant USAID-MTaPS ,GHSA
11:30-11:45 am	Presentation on "Assess National Stewardship Policies, Regulations, and Practices Conducted in Animal Health Sectors "	Dr. Nur e Alam Siddiky , Consultant USAID-MTaPS ,GHSA
11:45-12:00 pm	Presentation on AMS Guideline	Dr. Javedur Rahman, Consultant USAID-MTaPS ,GHSA
12:00-13.30pm	Open Discussion	All Participants
	Lunch	

DGHS.MOH&FW BANGLADESH

USAID FROM THE AMERICAN PEOPLE

APPENDIX 05: PARTICIPANTS LIST (WORKSHOP)*

Serial No.	Name of the Participants	Designation and Organization	E-mail
1	Prof. Dr. Md. Nazmul Islam	Director, Disease Control andLine Director, CDC, DGHS	cdc@ld.dghs.gov.bd
2	Prof. (Dr.) Sanya Tahmina	Director, Disease Control andLine Director, CDC, DGHS. (retired)	sanya.tahmina@gmail.com
3	Dr. Md. Shafiqul Islam	Deputy Director, CDC, DGHS	shafikislam67@yahoo.com
4	Prof. Dr. M A Faiz	Director General (retired), DGHS	drmafaiz@gmail.com
5	Prof. Dr. Nitish Debnath	Country Lead, Fleming Fund, DAI	nitish_debnath@dai.com
6	Zobaidul Haque Khan	Deputy Team Lead, Fleming Fund, Fleming Fund, DAI	khanzhaq@gmail.com
7	Dr. Aninda Rahman	DPM, CDC, DGHS	dr.aninda@mis.dghs.gov.bd
8	Md. Habibur Rahman	Consultant, FAO	md.habibur@fao.org
9	Dr. Hurul Jannat	MO, CDC, DGHS	shimussmc@gmail.com
10	Paritosh Chakma	National Consultant-EDM, WHO BAN	chakmap@who.int
11	S. M. Sabrina Yesmin	Assistant Director, DGDA	sabrinadgda22@gmail.com
12	Dr. Mahfuza Nasrin	Associate Professor, UAMCH	doctormahfuza@yahoo.com
13	Dr. Md. Jamal Uddin	Assistant Professor,NIPSOM	mohammadjamaluddin_1980@yahoo.com
14	Dr. Piash Kumer Deb	MO, CDC, DGHS	pkpiash@gmail.com
15	Dr. S. M. Shariar Rizvi	Evaluator, CDC, DGHS	shahriarrizvi@gmail.com
16	Prof. Dr. Zakir H. Habib	CSO, IEDCR	parashhabib@gmail.com
17	Prof. Dr. Md. Akram	Senior Consultant, Imperial Hospital	akram.prg@gmail.com
18	Dr. Md. Abul Kalam Azad	Deputy Director, CDC, DGHS	
19	Dr. Md. Abu Sufian	Director, DLS	sufian04@gmail.com
20	Dr. Md. Golam Azam Chowdhury	PSO; CDIL, DLS	psocdil@gmail.com
21	Dr. TABM Muzaffar Goni Osmani	Deputy Director, DLS	drmosmani@yahoo.com

Serial No.	Name of the Participants	Designation and Organization	E-mail
22	Dr. Mohammad Sadekuzzaman	ULO, CDIL, DLS	numanmicro@gmail.com
23	Dr. Mohammed A. Samad	BLRI	samad_blri@yahoo.co.nz
24	Dr. Md Samun Sarker	BLRI	samuncvasu@gmail.com
25	Dr. Md. Rafiqul Islam	CSO, BARC	mrislam210@hotmail.com
26	Kbd Manik Mia	Quality Manager, FIQC, DoF	manikfiqc.govt@yahoo.com
27	Dr. Md. Abu Sayem	Senior Technical Advisor, USAID MTaPS	msayem@mtapsprogram.org
28	Dr. Javedur Rahman	Consultant, USAID MTaPS	drjavedrahman@gmail.com
29	Dr. Md. Nure Alam Siddiky	Consultant, USAID MTaPS	nasiddiky.saarc@gmail.com
30	Dr. Najia Amin Salsabil	Consultant, USAID MTaPS	najia.amin.salsabil@gmail.com

** Not according to seniority*

APPENDIX 06: PRESENTATION ON THE ASSESSMENT– HUMAN HEALTH

AMS
Assessment_HH.pdf

APPENDIX 07: PRESENTATION ON THE ASSESSMENT– ANIMAL HEALTH

AMS
Assessment_AH.pdf

APPENDIX 08: PRESENTATION ON THE OVERVIEW OF AMS GUIDELINES FOR HUMAN HEALTH

AMS Guideline
Outline.pdf

APPENDIX 09: NATIONAL MULTISECTORAL AMS PLAN (HUMAN HEALTH AND ANIMAL HEALTH); 2022-2026

S/N	Recommendations/Actions Required	Lead Organization	Implementing/ Collaborating Partners	Timeline
**Major Core Element 1: National Plan and Strategies**				
**Human Health and Animal Health**				
1	National Multisectoral AMS Policy and Implementation Plan	CDC, DGHS	DGDA, DLS, DoF	2022-2023
2	National Multisectoral Technical Working Group on AMS with clear terms of reference	CDC, DGHS	DGDA, DLS, DoF	2022
3	Dedicated funding for the implementation of national action plan on AMR	CDC, DGHS	DGDA, DoE, DLS, DoF, BLRI, VS	2022-2026
4	Monitoring and Evaluation of the implementation of national action plan on AMR	CDC, DGHS	DGDA, DOE, DLS, DOF	2023-2026
**Major Core Element 2: Regulation and Guidelines**				
**Human Health**				
1	National antibiotic policy and user guidelines in human health	CDC, DGHS	DGDA	2023-2024
2	Integration of the AWaRe classification of antibiotics in the national EML and formulary	DGDA	-	2022-2023
3	Up-to-date clinical guidelines that include AMS principles and integrate the AWaRe classification of antibiotic	CDC, DGHS	Professional societies	2022
4	Enforcement of the regulations on fixed-dose combinations of antibiotics	DGDA	-	2023
5	Enforcement of regulations on prescription-only sale of antibiotics	DGDA	-	2023
6	Measures to ensure the continued availability of quality-assured antibiotics	DGDA	-	2022
7	Measures to ensure the affordability of essential antibiotics	DGDA	-	2022
8	Licensing of all antimicrobial premises/drug shops	DGDA	-	2023-2024
9	Strengthen monitoring of ‘model pharmacy’ and ‘pharmacy shop’	DGDA	-	2023
10	Strengthen post marketing quality control of the antimicrobials	DGDA	-	2024
11	Strengthen capacity building measures on pharmacovigilance reporting	DGDA	Hospitals, pharmaceutical companies, pharmacies	2023
12	Strengthen inspection of pharmacies to identify falsified, expired, banned and poor-quality antimicrobials	DGDA	-	2023
13	Regulations on proper disposal of unused, expired antibiotics by the users/clients/patients receiving care at the community level	DGDA	Pharmacies, pharmaceutical companies, media	2023-2026

S/N	Recommendations/Actions Required	Lead Organization	Implementing/ Collaborating Partners	Timeline
14	Update 'Code of Pharmaceutical Marketing Practices' with strict regulations on promotional activities of pharmaceutical companies.	DGDA	BMDC, BAPI	2023
15	Strengthen the regulations to monitor different categories of health care provider (e.g. CHCP, 'village doctors') to increase their accountability.	CDC, DGHS	DGME, CBHC	2023-2024
	**Animal Health**			
1	Antimicrobial policy and user guidelines in animal health	CDC, DGHS	DLS, DoF, DGDA	2023-2024
2	Develop and enact legislation on the use of antimicrobials in the farm animals	DLS	MoFL	2023
3	Develop and enact legislation on the use of antimicrobials in the aquaculture	DoF	MoFL	2023
4	Development of Antimicrobial Use (AMU) surveillance and monitoring guideline	1. DGDA	DLS	2022
5	Strict enforcement of fish feed and animal feed act, 2010	DLS	DoF	2022-2025
6	Integration of the AWaRe classification of antibiotics in the national EML and formulary	DGDA	DLS, DoF, CDC DGHS	2023-2024
7	Up-to-date clinical guidelines that include AMS principles and integrate the AWaRe classification of antibiotic	DLS	DGDA, CDC DGHS	2023-2024
8	Enforcement of the regulations on fixed-dose combinations of antibiotics	DGDA	DLS, DoF	2023-2026
9	Enforcement of regulations on prescription-only sale of antibiotics	DGDA	DLS, DoF	2023-2026
10	Measures to ensure the continued availability of quality-assured antibiotics	DGDA	DLS, DoF	2023-2026
11	Measures to ensure the affordability of essential antibiotics	DGDA	DLS, DoF	2022-2026
12	Licensing of all antimicrobial premises/drug shops	DGDA	DLS, DoF	2023-2024
13	Strengthen monitoring of 'model pharmacy' and 'pharmacy shop'	DGDA	DLS, DoF	2023-2026
14	Strengthen post marketing quality control of the antimicrobials	DGDA	DLS, DoF	2023-2026
15	Strengthen capacity building measures on pharmacovigilance reporting	DGDA	Hospitals, pharmaceutical companies, pharmacies	2023-2026
16	Strengthen inspection of pharmacies to identify falsified, expire, banned and sub- standard (poor quality) antimicrobials	DGDA	DLS, DoF	2023-2026
	**Major Core Component 3: Awareness, Training and Education**			
	**Human Health**			
1	Regular public antibiotic awareness campaigns and impact study	CDC, DGHS	DGDA, DLS, DoF, DOE	2022-2026
2	Education and practice in schools on basic infection principles	CDC, DGHS	MOE	2023-2024
3	Training on AMS competencies for AMS team members	CDC, DLS	All health care settings	2023-2024
4	Education and training for all health-care professionals on AMS	CDC, DLS	All health care settings	2023-2025

S/N	Recommendations/Actions Required	Lead Organization	Implementing/ Collaborating Partners	Timeline
5	Support the implementation of AMS programmes in all health-care facilities	CDC, DGHS	All health care settings	2024-2025
6	Introduce national level awards for high performing health facility in AMS program implementation	CDC, DGHS	All health care settings	2024-2025
7	Reform pharmacy education by giving emphasis on clinical pharmacy and community pharmacy	CDC, DGHS	UGC; DGDA, BPC, BAPI	2024-2025
8	Train and create a pool of journalists for AMR related news coverage through media fellowships (Media Fellowship for Reporting on AMR)	CDC, DGHS	DGDA, IEDCR, academic institutes	2023-2026
**Animal Health**				
1	Regular public antimicrobial awareness campaigns	DLS	DoF	2022-2026
2	Include AMR in veterinary education syllabus	DLS, BVC	MoE	2023-2024
3	Training on AMS competencies for field veterinarians	DLS	DGDA	2023-2024
4	Support to implementation of AMS programmes in animal health-care facilities	DLS	CVH, DVH, UVH	2024-2025
**Major Core Component 4: Surveillance and Data Management**				
**Human Health**				
1	Establishing HAI surveillance	CDC, DGHS	Hospitals, IEDCR	2023-2026
2	Establishing regular PPS	CDC, DGHS	Hospitals	2023-2026
3	National surveillance of Antimicrobial Consumption (AMC) at regular interval	DGDA	CDC, DGDS	2023-2026
4	National surveillance of Antimicrobial Use (AMU) at regular interval	CDC, DGDS	All health care settings	2023-2026
5	Annual release of national AMC and AMU surveillance data	DGDA	All stakeholders	2024-2026
6	National surveillance of AMR at regular interval	IEDCR	Medical colleges/ hospitals	2025
7	Strengthen the AMR surveillance capacity of the laboratories	CDC, DGDS	IEDCR	2025
8	Collaboration with private sectors to include private lab data in national AMR data base	CDC, DGHS	All leading private labs	2023
9	Annual release of national AMR surveillance data and policy recommendations	CDC	IEDCR	2023-2026
**Animal Health**				
1	AMR and AMU surveillance at regular interval	DLS, DoF	BLRI, BFRI, VS	2023-2026
2	Strengthen the AMR surveillance capacity of the laboratories in the animal and aquatic health care settings	DLS, DoF	BLRI, BFRI and Academic and Private labs	2023-2025
3	Collaboration with private sectors to include private lab data in national AMR data base	DLS, DoF	All leading private labs	2023-2026
4	Annual release of AMU and AMR surveillance data	DGDA	DGHS, DLS, DoF	2023-2026
**Miscellaneous**				

S/N	Recommendations/Actions Required	Lead Organization	Implementing/ Collaborating Partners	Timeline
**Human Health**				
1	Promote research activities on AMR involving undergraduate students of different related disciplines (medical, pharmacy, dental, nursing etc.)	CDC, DGHS	Medical, nursing and dental colleges, universities	2022-2026
2	Develop SOP for establishing antibiotics audit system and feedback mechanisms, formulary restrictions	CDC, DGHS	Different professional societies, DGDA, hospitals, pharmaceutical companies	2022-2023
3	Fill-up the vacant posts of DGDA	DGDA	-	2022-2024
4	Create posts for consultant microbiologists at district level hospital	CDC, DGHS	HSM, BSMM	2024
**Animal Health**				
1	Strengthen the capacity of veterinary cell at DGDA with deployment of adequate human resources	DGDA	DLS	2023-2024
2	Deployment of more veterinarian as a drug inspector under DGDA	DGDA	DLS	2023-2024
3	Formation of joint taskforce of DGDA and DLS to monitor the quality of antimicrobials at the production and marketing outlet	DGDA	DLS	2023
4	Strengthen the capacity of DLS to monitor the rational uses of antimicrobials at the farm production	DLS	-	2023-2025
