# Supplementary material for: Landscape Analysis of Existing Policies, Laws and Regulations on Antimicrobial Stewardship in 10 Low- and Middle-Income Countries
Source: Antibiotics (Basel). 2026 Aug 10;15(8):770. doi: 10.3390/antibiotics15080770 (PMC13510074; doi:10.3390/antibiotics15080770)
Supplement: Supplementary file 1 [file antibiotics-15-00770-s001.zip › S3 Country Reports/S3A-2_Bangladesh (Animal health).pdf]

USAID MEDICINES, TECHNOLOGIES, AND PHARMACEUTICAL SERVICES (MTaPS) PROGRAM

Improved Access. Improved Services. Better Health Outcomes.

Report on Deliverable 4

Deliverable 4: Draft assessment report of national stewardship policies, regulations, legislations and practices conducted in animal health sectors, including methodology, findings and recommendations

The report on deliverable 4 is submitted by

(Md. Nure Alam Siddiky)
Consultant
Cell: +88 01716475486
Email: nasiddiky.saarc@gmail.com

Report title: To Assess National Antimicrobial Stewardship Policies, Regulations and Practices Conducted in Animal Health Sector of Bangladesh

This document is made possible by the generous support of the American people through the US Agency for International Development (USAID) contract no. 7200AA18C00074. The contents are the responsibility of Management Sciences for Health and do not necessarily reflect the views of USAID or the United States Government

About the USAID MTaPS Program

The USAID Medicines, Technologies, and Pharmaceutical Services (MTaPS) Program enables low- and middle-income countries to strengthen their pharmaceutical systems, which is pivotal to higher-performing health systems. MTaPS focuses on improving access to essential medical products and related services and on the appropriate use of medicines to ensure better health outcomes for all populations. The program brings expertise honed over decades of seminal pharmaceutical systems experience across more than 40 countries. The MTaPS approach builds sustainable gains in countries by including all actors in health care—government, civil society, the private sector, and academia. The program is implemented by a consortium of global and local partners and led by Management Sciences for Health (MSH), a global health nonprofit.

The MTaPS Consortium

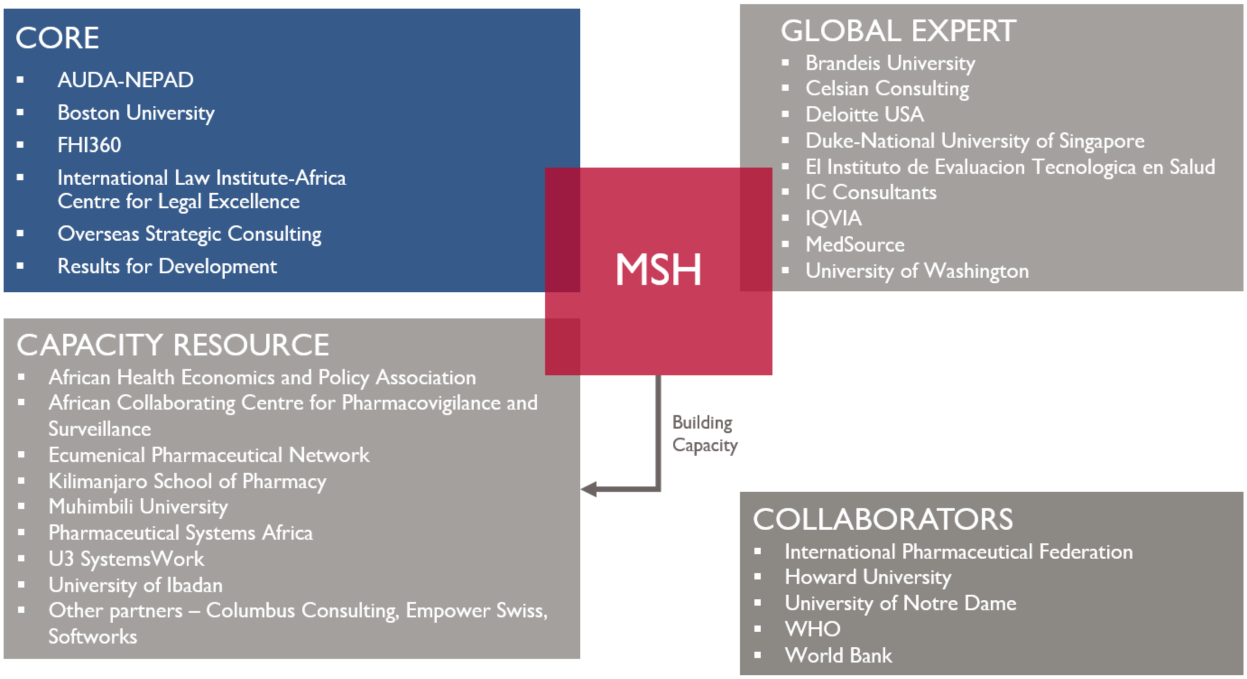

This document is submitted by: Nure Alam Siddiky, Consultant

Email: nasiddiky.saarc@gmail.com

ACRONYMS AND ABBREVIATIONS

ADR	Adverse Drug Reaction
ADRAC	Adverse Drug Reaction Advisory Committee
ADRM	Adverse Drug Reaction Monitoring
AMC	Antimicrobial Consumption
AMR	Antimicrobial Resistance
AMS	Antimicrobial Stewardship
AMs	Antimicrobials
AMU	Antimicrobial Use
ARC	Antimicrobial Resistance Containment
AST	Antimicrobial Susceptibility Testing
AWaRe	Access Watch and Reserve
BAHIS	Bangladesh Animal Health intelligence System
BARC	Bangladesh Agricultural Research Council
BDNF	Bangladesh National Formulary
BFSA	Bangladesh Food Safety Authority
BLRI	Bangladesh Livestock Research Institute
BVA	Bangladesh Veterinary Association
BVC	Bangladesh Veterinary Council
CDC	Communicable Disease Control
CDIL	Central Disease Investigation Laboratory
CLSI	Clinical and Laboratory Standards Institute
CPE	Continuous Professional Education
DCC	Drug Control Committee
DGDA	Directorate General of Drug Administration
DGHS	Directorate General of Health services
DLS	Department of Livestock Services
DVH	District Veterinary Hospital
EML	Essential Medicine List
FAO	Food and Agriculture Organisation
FDIL	Field Disease Investigation Laboratory
GAP	Global Action Plan
HC	High Court
IPC	Infection Prevention and Control
KII	Key Informant Interview
LDDP	Livestock and Dairy Development Project
MoFL	Ministry of Fisheries and Livestock

MOH&FW	Ministry of Health and Family Welfare
MTaPS	Medicines, Technologies, and Pharmaceutical Services
NCL	National Control Laboratory
NPC	National Pharmacovigilance Centre
OTC	Over the Counter
PV	Pharmacovigilance
STG	Standard Treatment Guidelines
SWOT	Strength, Weakness, Opportunity and Threats
TWG	Technical Working Group
UVH	Upazilla Veterinary Hospital
VH	Viral Hepatitis
VMP	Veterinary Medicinal Products
WHO	World Health Organization
WOAH	World Organization for Animal Health
WTO SPS	World Trade Organisation- Sanitary and Phytosanitary
GLASS	Global Antimicrobial Resistance and Use Surveillance System

CONTENTS

S. N.	Contents	Page No.
	Acronyms and Abbreviations	
	Project Summary	10
1	Introduction- background with objectives	11-12
2	Methodology/ assessment tools	12
2.1	Desk review of WHO, OIE, MTaPS and different national guidelines	12-13
2.2	Desk review of rules, regulation, policies, guidelines, reports, peer reviewed journals	13
2.3	Key informant interviews, focus group discussion and in person communication	13
2.4	Strength, Weakness, Opportunity and Threats (SWOT) analysis	13-14
3	National and global organization involved with AMS policies, guidelines and practices	15
3.1	Department of Livestock Services (DLS)	15
3.2	Communicable Disease Control (CDC), DGHS	15
3.3	Directorate General of Drug Administration (DGDA)	16
3.4	Bangladesh Food Safety Authority (BFSA)	16
3.5	World Organization for Animal Health (WOAH)	16
3.6	World Health Organization (WHO)	17
4	National core elements of antimicrobial stewardship	17
4.1	National plan and strategies	17
4.1.1	National action plan on AMR that states AMS is a priority	17
4.1.2	National AMU and AMR surveillance strategy in place	17
4.1.3	Technical Working Group (TWG) on AMS with clear terms of reference	18
4.1.4	National AMS implementation plan or policy	18
4.1.5	Monitoring and evaluation mechanism in place for the national action plan on AMR	18
4.2	Leadership and commitment	18

4.2.1	Institutionalization of AMR in animal health sector	18
4.2.2	Deployment of human resources to coordinate AMR	18
4.2.3	Dedicated funding for the national action plan on AMR	19
4.3	Policies, regulations and guidelines on antimicrobial stewardship	19
4.3.1	Drug Act, 1940 and Subsequent Govt. gazette of 2020	19
4.3.2	The Drug (Control) Ordinance 1982	19
4.3.3	National Drug Policy, 2016	20-21
4.3.4	Fish Feed and Animal Feed Act, 2010	21
4.3.5	National Livestock Extension Policy, 2013	22
4.3.6	National Livestock Development Policy, 2007	22
4.3.7	Veterinary Practitioners Ordinance, 1982	22
4.3.8	Food Safety Act, 2013	22-23
4.3.9	Policies that support the regulation of medicines with specific focus to antimicrobials	23
4.3.10	Integration of the AWaRe classification of antibiotics in the national essential medical list (EML) and formulary	23-24
4.3.11	Up-to-date clinical guidelines that include AMS principles and integrate the AWaRe classification of antibiotics	24
4.3.12	Regulations on fixed-dose combinations of antimicrobials	24
4.3.13	Regulations on prescription of sale of antimicrobials	24
4.3.14	Regulation and enforcement of prescription-only dispensing of antimicrobials	24
4.3.15	Regulations for continued availability of quality antimicrobials	24
4.3.16	Regulations in place to ensure affordability of essential antimicrobials	25
4.3.17	A system of regulating the promotional activities of pharmaceuticals companies	25
4.4	Marketing authorization, licensing and inspection of antimicrobials	25
4.4.1	Marketing authorization of the antimicrobials	25
4.4.2	Licensing of antimicrobial premises/ drug shops	25
4.4.3	Regulatory inspection	26

4.5	Post market surveillance and pharmacovigilance	26
4.5.1	Post marketing quality control	26
4.5.2	Pharmacovigilance	26-27
4.6	Prescription, dispensing and use of antimicrobials	27
4.6.1	Pattern of antibiotic dispensing at pharmacies according to the WHO Access, Watch, Reserve (AWaRe) classification	27
4.6.2	Antibiotic prescribing and dispensing in accordance with national drug policies	27
4.6.3	Mapping the antimicrobial supply chain in animal health sector	27
4.7	Awareness, training and education	28
4.7.1	Regular public antimicrobial awareness campaigns	28
4.7.2	Education in schools on basic infection principles	28
4.7.3	Training on AMR competencies for animal health physicians	28
4.7.4	Continuous education for all health-care professionals on AMS	28
4.7.5	Incentives to support implementation of AMS programmes in all animal health-care facilities, including staffing standards, training and accreditation	28
4.8	AMR surveillance capacities and data sharing mechanism	28
4.8.1	National regular surveillance on antimicrobial use (AMU) in place	28-29
4.8.2	National surveillance system on AMR in place with laboratory capacity to guide optimal use of antibiotics in clinical practice and update clinical guideline	29
4.8.3	Facilities of antimicrobial susceptibility testing (AST) at health care and diagnostic settings	29
4.8.4	AMR data sharing platform in animal health	29
4.8.5	Diagnostic tests available and capacity building undertaken to optimize antibiotic use	29-30
5	World Organization for Animal Health (WOAH) guidelines for responsible and prudent use of antimicrobial agents in veterinary medicine	29-30

5.1	WOAH Guiding principles of responsible and prudent use of antimicrobials	30
5.2	Marketing authorization guidelines	30
5.3	Quality control of antimicrobial agents	30
5.4	Responsibilities of the veterinary pharmaceutical industry with regards to VMP containing antimicrobial agents	31
5.4.1	Marketing authorization	31
5.4.2	Marketing and export	31
5.4.3	Advertising	31
5.5	Responsibilities of wholesale and retail distributors	31-32
5.6	Responsibilities of veterinarians	32
5.6.1	Use of antimicrobial agents	32
5.6.2	Choosing antimicrobial agents	32-33
5.6.3	Recording of data	33
5.6.4	Training and continued professional development	33
5.7	Responsibilities of food animal producers	33-34
5.8	Responsibilities of animal feed manufacturers	35
6	Recommendations	35-36
7	Conclusions	37-38
8	Glossary	38-39
9	References	39-40
10	Annexure 1: Assessment Tool for Antimicrobial Stewardship Policies, Guidelines and Practices in Animal Health Sector	41-59

PROJECT SUMMARY

<table>
<tr><td colspan="2">Program Name:</td><td>USAID Medicines, Technologies, and Pharmaceutical Services (MTaPS) Program</td></tr>
<tr><td colspan="2">Activity Start Date and End Date:</td><td>February 18, 2022–July 31, 2022</td></tr>
<tr><td colspan="2">Name of Prime Implementing Partner:</td><td>Management Sciences for Health</td></tr>
<tr><td colspan="2">Contract Number:</td><td>7200AA18C00074</td></tr>
<tr><td rowspan="4">MTaPS Partners</td><td>Core Partners</td><td>Boston University, FHI 360, Overseas Strategic Consulting, Results for Development, International Law Institute-Africa Centre for Legal Excellence, NEPAD</td></tr>
<tr><td>Global Expert Partners</td><td>Brandeis University, Deloitte USA, Duke-National University of Singapore, El Instituto de Evaluacion Technologica en Salud, IC Consultants, Imperial Health Sciences, MedSource, QuintilesIMS, University of Washington</td></tr>
<tr><td>Capacity Resource Partners</td><td>African Health Economics and Policy Association, Ecumenical Pharmaceutical Network, U3 SystemsWork, University of Ibadan, University of Ghana's World Health Organizations (WHO) Pharmacovigilance Collaborating Center, Kilimanjaro School of Pharmacy, Muhimbili University, Pharmaceutical Systems Africa</td></tr>
<tr><td>Collaborators</td><td>International Pharmaceutical Federation, Howard University, University of Notre Dame, WHO, World Bank</td></tr>
</table>

Executive Summary

The regulatory aspect of antimicrobial stewardship (AMS) is a very important strategic focus for curbing the emergence of antimicrobial resistance (AMR). The assessment of AMS policies, regulations and practices conducted in animal health is carried out under the Medicines, Technology, and Pharmaceutical Services (MTaPS) program using the WHO toolkit along with various national and international guidelines. This assessment showed that the Directorate General of Drug Administration (DGDA) is the principal authority to develop policies and guidelines for the responsible use of antibiotics in the animal health sector. DGDA is responsible for the monitoring of production, importation, quality control, marketing authorisation, post marketing surveillance, pharmacovigilance, dispensing and prescription auditing of the antimicrobials (AMs). Though DGDA has time befitting policies and regulations related to AMS, but enforcement is very weak due to inadequate resources at the field conditions. In addition, lack of national policies and guidelines for antimicrobial use is a major gap.

The Department of Livestock Services (DLS) is the leading authority to coordinate and monitor AMR and antimicrobial use (AMU) surveillance in the food producing animals and foods of animal origin. There is no national database on AMR and AMU due to inconsistent integrated surveillance. The rational use of AMs in livestock is the responsibility of DLS, but there are no guidelines or regulations regarding this. The DLS should develop guidelines on the use of AMs in the farming practices for therapeutic, preventive and growth promotion purposes. Further, DLS should take lead to develop standard treatment guidelines for the field veterinarians to ensure the responsible use of AMs. DLS must lead the implementation of national AMU/AMR surveillance and the collection and compilation of data for decision making. However, specifically, it is noted that there is a deficiency in the regulation of antimicrobial management in the animal health sector.

According to the assessment, the animal health sector is far behind and needs further improvement in AMS stewardship through regulation for consistent development of AMR and AMS situation in Bangladesh. The Bangladesh Veterinary Council can also take initiative to develop ethics and guidelines for the registered veterinarian for animal welfare and protection of human health through rational use of AMs. To achieve a stronger AMS, it is highly recommended to build stronger and more effective coordination and collaboration amongst the regulatory authorities, DGDA, DLS and BFSA.

1. Introduction

Antimicrobial stewardship (AMS) refers to the actions veterinarians take individually and as a profession to preserve the effectiveness and availability of antimicrobial drugs through conscientious oversight and responsible medical decision-making while safeguarding animal, public, and environmental health**.** Stewardship is defined as "the careful and responsible management of something entrusted to one's care". It was originally applied in the health-care setting as a tool for optimizing antimicrobial use, termed "antimicrobial stewardship" (AMS). Stewardship has since been applied in the context of governance of the health sector as a whole, taking responsibility for the health and well-being of the population and guiding health systems at the national and global level.

The AMS is imperative in terms to optimize the use of antibiotics; to promote behaviour change in antibiotic prescribing and dispensing practices; to improve quality of care and patient outcomes; to save on unnecessary treatment costs; to reduce further emergence, selection and spread of AMR; to prolong the lifespan of existing antibiotics; to limit the adverse economic impact of AMR; and to build the best-practices capacity of health-care professionals regarding the rational use of antibiotics.

The stewardship policy provides the framework for how the antimicrobial stewardship program operates in veterinary practices. This program aims to promote the optimal use of antimicrobial agents by veterinarians in order to maximize effectiveness and minimize the potential for drug resistance. The primary goal of AMS is to optimize clinical outcomes while minimizing unintended consequences of antimicrobial use, including the emergence of resistance. Given the association between antimicrobial use and the selection of resistant pathogens, the frequency of inappropriate antimicrobial use is often used as a surrogate marker for the avoidable impact on antimicrobial resistance. The combination of effective AMS with a comprehensive infection control program has been shown to limit the emergence and transmission of antimicrobial-resistant bacteria. AMS is important to both animal health and food safety. Just like humans, animals get infections that require treatment with antibiotics. Responsible antibiotic use is essential to prevent emergence of antibiotic-resistant bacteria, protect patients from adverse drug effects, and avoid antibiotic residue in animal products. The use of "best practices" for animal management, including vaccination, parasite control, stress reduction, and optimization of environment and nutrition, serves to maintain animal health and reduce the need for antibiotics.

Many countries around the world including Bangladesh have developed and are implementing their national action plan (NAP) on AMR, in which AMS is a key priority. Although there is a scientific evidence base for AMS, and national, regional and global guidance documents exist, there is a growing need for more specific guidance on how to establish, implement and evaluate effective AMS programmes at the national level. To meet this need, WHO, in collaboration with global AMS experts, has developed a practical toolkit for assessing the national policies and guidelines for AMS programmes.

The study on the assessment of AMS policies, guidelines and practices in animal health sector of Bangladesh is conducted in alignment of the **following objectives**:

- To assess the existing national policies and guidelines developed related to AMS in animal health sector;
- To identify the gaps in the national AMS policies and guidelines to optimize the use of antibiotics;
- To recommend the possible synergies and collaborations in building stronger national AMS programmes.

2. Methodology/ Assessment Tools

2.1 Desk review of WHO, WOAH and MTaPS guidelines

A WHO practical toolkit on antimicrobial stewardship programmes in health care facilities in low and middle income countries has been consulted. The toolkit provides guidance on where to get started, including the structures and resources that should be put in place at the national and health-care facility level, through a stepwise approach in low-resource settings. As the ultimate goal of an AMS programme is sustainable behaviour change in physicians' antibiotic prescribing practices, the toolkit also provides detailed guidance on how to plan, perform and assess AMS interventions-including feedback on antibiotic use over time. Finally, the toolkit provides an overview of the competencies an AMS team needs to guide health-care professionals in changing their antibiotic prescribing behaviours.

WOAH Standards, Guidelines and Resolution on antimicrobial resistance and the use of antimicrobial agents was consulted and reviewed. WOAH guidelines on "responsible and prudent use of antimicrobial agents in veterinary medicine" provides guidance for the responsible and prudent use of antimicrobial agents in veterinary medicine, with the aim of protecting both animal

and human health as well as the environment. It defines the respective responsibilities of the Competent Authority and stakeholders such as the veterinary pharmaceutical industry, veterinarians, animal feed manufacturers, distributors and food animal producers who are involved in the authorisation, production, control, importation, exportation, distribution and use of veterinary medicinal products (VMP) containing antimicrobial agents. Responsible and prudent use is determined taking into account the specifications detailed in the marketing authorisation and their implementation when antimicrobial agents are administered to animals and is part of good veterinary and good agricultural practice.

A national report of Burkina Faso on "rapid assessment of antimicrobial stewardship policies and regulatory framework" implemented by USAID Medicines, Technologies, and Pharmaceutical Services (MTaPS) program was consulted to identify the potential synergy.

2.2 Desk review of rules, regulation, policies, guidelines, reports and peer reviewed journals

The relevant laws, regulations and guidelines has been consulted which was developed over the time by the AMR regulatory authorities, i.e., Directorate General of Drug Administration (DGDA), Department of Livestock Services (DLS) and Bangladesh Food Safety Authority (BFSA). Alongside, published review manuscripts is also consulted and studied to harness the useful information related to AMS in animal health.

2.3 Key informant interviews, focus group discussion and personal communication

A series of key informant interviews were carried out with the responsible officials of DGDA and DLS during data collection using self-developed assessment tools. Sometimes, data was also collected through focus group discussion and personal communication.

2.4. Strength, Weakness, Opportunity and Threats (SWOT) analysis

A SWOT analysis was done to assess the competencies of the AMS in the animal health.

2.4.1 Strengths

- AMS is the priority area in the AMR national action plan;
- AMR surveillance strategy in place;
- AMU strategy in place;
- National competent authority has been established to develop AMS policies and strategies;
- AMS policies and strategies are in place to promote rational use of antimicrobials (AMs);

- Continuous professional development of the state veterinarian is prioritized;
- Increasing public awareness and stakeholder involvement.

2.4.2 Weakness

- Weak institutional capacity to address AMR issues across the country;
- Weak implementation and monitoring of AMS policies and strategies;
- Inadequate surveillance on the quality assessment of AMs;
- Insufficient monitoring the uses of AMs in the livestock and poultry farming practices;
- Lack of sufficient dedicated drug inspectors at the field level;
- No regular audit on the prescription of the veterinarian;
- Weak post marketing surveillance and pharmacovigilance;
- No Technical Working Group (TWG) on AMS with clear terms of reference in the animal health;
- Poor marketing authorization of the AMs.

2.4.3 Opportunities

- Improving institutional commitment and ownership;
- Increasing capacity in terms of laboratory and human resources;
- Multisectoral collaboration and coordination;
- Enforcement of AMs policies and regulations;
- Active implementation of the NAP on AMR;
- Increasing national awareness of AMR and its consequences for health;
- Development of AMR and AMU national database;
- Increasing funding opportunity for AMR containment.

2.4.4 Threats

- Easy access to the essential AMs;
- No control on the uses of AMs in the farm production;
- Lack of accurate AMs import and local production data;
- Increasing trends of AMR in the foods of animal origin;
- Non therapeutic uses of AMs in the farm practices;
- Poor quality AMs available in the animal health sector.

3 National and Global Organization Involved with AMS Policies, Guidelines and Practices

There is a number of national and global organizations are solely involved for the development of regulatory policies, guidelines and practices for antimicrobial stewardship. The name of the organizations are as follows

- Department of Livestock Services (DLS) (http://www.dls.gov.bd)
- Communicable Disease Control (CDC), DGHS. (https://dghs.gov.bd)
- Directorate General of Drug Administration (DGDA) (https://dgda.portal.gov.bd)
- Bangladesh Food Safety Authority (BFSA) (http://www.bfsa.gov.bd)
- World Organization for Animal Health (WOAH) (https://www.woah.int/en/home/)
- World Health Organization (WHO) (https://www.who.int/bangladesh/about-us/our-work)

3.1 Department of Livestock Services (DLS)

Within the Ministry of Fisheries and Livestock (MoFL), the DLS is the principal organisation with responsibility for the delivery of services to livestock farmers and for ensuring animal health, welfare and veterinary public health, including the safety of food of animal origin destined for human consumption. DLS has mandate for monitoring and surveillance of AMs in the animal production system. DLS is the authority who can impose laws and regulations for the prudent use of AMs in the animal production system.

3.2 Communicable Disease Control (CDC), DGHS

Antimicrobial Resistance Containment component has been introduced into Communicable Disease Control (CDC) of Directorate General of Health services (DGHS), Ministry of Health and Family Welfare (MOH&FW) in the fourth Sector wide program as a sub-component of Antimicrobial Resistance Containment (ARC), Viral hepatitis (VH) & Diarrhea. CDC is the national focal institute to coordinate the implementation of AMR in the animal health, aquatic health and environmental sector. CDC is also responsible to review and update the NAP of AMR in a regular interval. CDC hosts and coordinate the governance of AMR, i.e. sectoral working group, national technical committee and national steering committee. CDC plays vital role in implementing AMS in the health care facilities.

3.3 Directorate General of Drug Administration (DGDA)

The Directorate General of Drug Administration (DGDA) under the Ministry of Health & Family Welfare, Government of the People's Republic of Bangladesh, is the Drug Regulatory Authority of the country. This DGDA supervises and implements all prevailing drug regulations in the country and regulates all activities related to import, procurement of raw and packing materials, production and import of finished drugs, export, sales, pricing, etc. of all kinds of medicines including AMs. At present, there are 47 district offices under the DGDA in the country. All the officers of the DGDA are designated as "Drug Inspector" in pursuant to the drug laws and assist the licensing authority to discharging his responsibilities properly. Besides, a number of committees, such as Drug Control Committee (DCC), Standing Committee for imports of raw materials and finished drugs, Pricing Committee and a number of other relevant committees, which comprise of experts of different fields, are there to advice licensing authority and recommend about the matters related to drugs and medicines.

3.4 Bangladesh Food Safety Authority (BFSA)

The BFSA was established in 2015 as an entity of the Ministry of Food (MoF) under the authority of the Food Safety Act (2013). The BFSA continues to grow in assuming its mandate. The Act establishes the Bangladesh Food Safety Authority (BFSA) itself, and under Section 13 gives the Authority the power to, "*regulate and monitor the activities related to manufacture, import, processing, storage, distribution and sale of food so as to ensure access to safe food through exercise of appropriate scientific methods, and coordinate the activities of all the organizations concerned with food safety coordination management.*"

3.5 World Organization for Animal Health (WOAH)

The WOAH is an intergovernmental organization responsible for improving animal health worldwide. The WOAH is recognized as a reference organization by the World Trade Organization. WOAH's primary objective is to protect the health of animals and to ensure a safe and fair trade in animals and animal products worldwide, by ensuring transparency in the global animal disease situation and by publishing health standards for international trade. It also sets guidelines for animal welfare although this mandate does not fall under the WTO SPS agreement. The WOAH promotes the responsible and prudent use of antimicrobial agents in terrestrial and aquatic animals, so as to preserve their therapeutic efficacy and prolong their use in both animals and humans.

3.6 World Health Organization (WHO)

The WHO supports in the development of strategies to contain AMR globally, promotes Member State commitment to local, national and global surveillance, and contributes to building technical and human capacity. WHO has developed Global Action Plan (GAP) to guide the country how to develop national action plan. In addition, WHO has developed the toolkit to assess the antimicrobial stewardship policies, strategies and practices at the institutional level as well as health care facilities.

4 National Core Elements of Antimicrobial Stewardship

4.1 National Plan and Strategies

A national AMR strategy and action plan is a state policy document which includes detailed guidelines, activities and implementation plan of surveillance of AMU and AMR along with intervention for a specific period of time. Bangladesh has developed national antimicrobial resistance containment (ARC) plan along with road map of national action plan of ARC for the duration of 2017-2021. Further, Bangladesh has recently revised and updated the AMR action plan for the duration of 2021-2026. However, this AMR action plan has not yet been approved by the National Steering Committee. The AMR action plan is a multisectoral one health strategic document with the participation and partnership of human health, animal health, aquatic health and environmental health. The AMR action plan of Bangladesh principally owned by human health department. The Communicable Disease Control (CDC) unit of Directorate General of Health Services (DGHS) is the lead agency to coordinate the update and revise the AMR national strategy and its operation plan. The national AMR strategy specified the activities for the animal health sector. So, animal health sector doesn't have separate national AMR strategy and action plan alone.

4.1.1 National action plan on AMR that states AMS is a priority

AMS has been considered as a national priority at the animal health in the existing AMR national action plan.

4.1.2 National AMU and AMR surveillance strategy

Bangladesh recently developed an AMR surveillance strategy with the technical support of Fleming Fund grant for the duration of 2020-2025. The national AMR strategy is a multisectoral document and animal health sector is one of its important section. However, DGDA has recently developed

on July, 2022, a draft guideline on Antimicrobial Consumption (AMC) surveillance in Bangladesh for both human and animal health sectors.

4.1.3 Technical Working Group (TWG) on AMS with clear terms of reference

The technical working group on AMR has not yet been formed in animal health. The overall aim of the TWG is to optimize the use of existing AMs and prevent the spread of resistant infections. So, animal health sector has to immediately develop technical working group with the involvement of multidisciplinary teams including experienced veterinarian, pharmacists, microbiologists, epidemiologists, and medicine specialist. But recently, AMR sectoral working group is formed under the leadership of DLS to coordinate the AMR surveillance and data sharing through a common platform.

4.1.4 National AMS implementation plan or policy

There is no formal AMS implementation plan or policy in the animal health sector. But some activities are directly or indirectly linked with AMS. The animal health sector has to develop AMS implementation plan to oversee the different uses of antimicrobials at the livestock production system.

4.1.5 Monitoring and evaluation mechanism in place for the national action plan on AMR

Multisectoral monitoring and evaluation mechanism in place but no sectoral monitoring and evaluation mechanism has been developed specific for animal health. A multisectoral core working group on AMR has been established to oversee the monitoring of progress in implementation of AMR action plan.

4.2 Leadership and Commitment

The state animal health sector must have commitment and acquire ownership to tackle AMR as a priority food safety issue. The following ownership and commitment must be considered for better stewardship

4.2.1 Institutionalization of AMR in animal health sector

There is no designated desk/ wing at DLS to coordinate the AMR issues in animal health. The state animal health sector doesn't have institutional framework to oversee, coordinate and implement the AMR issues. Therefore, institutionalization of AMR is a demand of time to uphold the AMR as one of the food safety priority issue.

4.2.2 Deployment of dedicated human resources

There is few legitimate staff to deal with AMR issues at animal health sector. A team should be formed by the DLS who will solely be involved with AMR issues. A dedicated team is essential to conduct and coordinate the AMU and AMR surveillance regularly.

4.2.3 Dedicated funding for the implementation of national action plan on AMR

There is no regular revenue budget to implement the NAP of AMR. Current AMR funding are either from project based or overseas resource or grant. Regular revenue head budget allocation is needed to conduct the AMR surveillance and intervention activities smoothly and effectively in a regular pace. DLS has taken important initiatives in continuous professional development as well as in the development of policy framework for integrated AMR surveillance, risk analysis, risk mitigation in the animal health sector with the support of world bank funded Livestock and Dairy Development Project (LDDP). Besides, development partners (FAO, Fleming Fund, USAID-MTaPS) are supporting to animal health sector for AMR containment in different capacities.

4.3 Policies, Regulations and Guidelines Related to Antimicrobial Stewardship

4.3.1 Drug Act, 1940 and Subsequent Govt. gazette of 2020

The drug act, 1940 has been enacted in the British regime to regulate the import, export, manufacture, distribution and sale of drugs including AMs. The main features of the drug act, 1940 are (i) guidelines for the import and export of drugs, and (ii) manufacture, sales and distribution of drugs. The important milestones related to AMS found in the drug act, 1940 are:

- there is a prohibition of manufacture and sale of drugs which is not a standard quality and misbrand;
- license is required before importation of any drugs including AMs;
- require the date of manufacture and the date of expiry of potency to be clearly and truly stated on the label of drugs;
- every manufacturer must approve their promotional materials from the competent authority prior to distribution;
- every manufacture must take marketing authorization from the competent authority.

4.3.2 The Drug (Control) Ordinance 1982

The drug (control) ordinance 1982 has been enacted in 1984 to control manufacture, import, distribution and sale of drugs. The core components of the drug control ordinance are (i) formation of drug control committee, (ii) registration of medicines before sale, (iii) prohibition of manufacture of certain medicines, (iv) restriction on import of certain pharmaceutical raw

material; (v) fixation of price of drugs, (vi) deployment of registered pharmacist in the pharmaceutical manufacturing industry, (vii) control of advertisement and claims in respect of drugs, (viii) prohibition of prescription of unregistered medicine, (ix) good practices in the manufacture and quality control of drugs, (x) penalty for manufacture of certain banned drugs, (xi) penalty for manufacture, stock or sale of sub-standard drugs, (xii) penalty for unauthorised import of drugs, (xiii) penalty for illegal advertisement and claims, (xiv) formation of drug courts, (xv) formation of national drug advisory council, etc.

4.3.3 National Drug Policy, 2016

The National Drug Policy 2016 updates and replaces the National Drug Policy from 1982 and 2005. The National Drug Policy 2016 includes well-defined directives for drug registration, production, marketing, distribution, storage, sale, use, import, export and quality control. The Government of Bangladesh has enacted National Drug Policy, 2016 with aiming to ensure people can have easy access to safe, effective and good quality drugs at affordable prices. The main directives of national drug policy, 2016 with regards to antimicrobial stewardship are:

- stringent adherence to the WHO-recommended GMP guidelines in drug production and quality control must be ensured by the pharmaceutical sector;
- each locally produced or imported drug must be registered by the licensing authority (DGDA) based on the recommendation of Drug Control Committee (DCC);
- the manufacture, sale and distribution of fake, adulterated, harmful, un-registered, counterfeit, misbranded and substandard drugs including AMs be forbidding and exemplary punishment bestowed upon people responsible for such offences;
- to regulate all sorts of advertisements and promotion of AMs in public media and prevent unethical marketing and multi-level marketing of all recognized system of AMs to ensure safe, rational and effective use;
- ensure accessibility to AMs at affordable price and fix drug prices by transparent and rational methods;
- to ensure pharmacovigilance and appropriate monitoring of Adverse Drug Reactions (ADR) through motivation for all concerned people to be accurately informed about adverse drug events;
- to prohibit sales and distribution of AMs without prescription from registered physician to ensure rational use of drugs;

- rational and safe use of drugs will be assured through pursuance of Standard Treatment Guidelines (STG);
- to assure rational use of antibiotics at hospitals with their own 'Antibiotic user guidelines';
- sale and dispensing of AMs will be conducted under direct supervision of professional pharmacists for providing counseling to patients on the appropriate use and storage of drugs;
- retail sales of drugs are prohibited without prescription by registered physicians/ veterinarians other than the OTC drugs;
- prescription and dispensing systems must be regularly monitored by DGDA to ensure rational use of drugs;
- drugs and therapeutic committee will be formed in all public and private hospitals to ensure rational use of drugs including AMs;
- essential Medicine List (EML) will be updated regularly based on essential medicine concept;
- all AMs that are produced in different dosage forms, imported, distributed or marketed or used have to be registered by licensing authority based on the recommendation of Drug Control Committee (DCC). Regular meetings of the DCC will convene to make faster availability of new life-saving drugs for the public;
- the list of essential veterinary drug will be prepared and published having opinion of the veterinary specialists, and the list will be updated accordingly. The production and distribution of the essential drugs will be ensured as per the need;
- a list of Over the Counter (OTC) drugs for veterinary medicine will be prepared and published on the basis of the opinion of veterinary specialists.

4.3.4 Fish Feed and Animal Feed Act, 2010

Banning of the use of antibiotic, growth hormone, insecticides etc. in fish feed and animal feed is strictly prohibited. Under this act, any harmful chemicals including **antibiotic**, growth hormone, steroid and insecticides cannot be used in fish feed and animal feed. Anybody violating the law will come under the purview of crime. If any person commits any crime under this law, that person will undergo imprisonment of one year or penalty up to 50,000 Bangladeshi Taka or will be convicted in both the cases simultaneously.

4.3.5 National Livestock Extension Policy, 2013

The National Livestock Extension Policy 2013 touches upon AMU and drug residue issues only marginally, in the section for "Emerging livestock development issues", as follows:

- **Drug residues** in food products of animal origin is a **major food safety concern** of present time, and effective control should be implemented following OIE (WOAH) guidelines;
- AMU is strictly regulated in **organic farming** and bio-rational-based management systems should be disseminated in the farms to ensure productivity and products' safety and quality.

4.3.6 National Livestock Development Policy, 2007

The National Livestock Development Policy 2007 touches upon AMU only marginally, in the "Poultry development" and "Veterinary services and animal health" sections, by stating the following:

- The **use of antibiotics in feeds is thought to be common** and a cause of public health concern;
- **No registration is required for feed additives** such as toxins binder, antibiotics, and vitamin/mineral premixes, animal protein, many of which are potentially detrimental to human health. Most of the drugs traders and shop keepers have **no formal training on drug handling**, transportation, storing and dispensing, and readily sell drugs such as antibiotics, hormones, and sedatives across the counter without prescription.

4.3.7 Veterinary Practitioners Ordinance, 1982

This ordinance makes provision for the regulation, control and registration of veterinary practitioners and establishes that veterinary medicine and surgery should be practised only by **registered veterinary practitioners**. It also includes the constitution of a **Veterinary Council** that, among other tasks, reviews and approves applications for registration of veterinary practitioners.

4.3.8 Food Safety Act, 2013

The Government of Bangladesh enacted food safety act, 2013 to ensure the safe and hygienic food for the nation including animal derived foods. The following points of the Act are of particular relevance in the AMU/AMR and food safety perspective:

- provide the concerned authority or organization with necessary support in updating or upgrading the permissible limit at its highest safety level of veterinary drug residues, antibiotics, etc. as determined by any other authority or organization under the existing laws, and monitor implementation thereof;
- determine, through exercise of scientific methods, permissible limit of veterinary drug residues, antibiotics, etc. where the permissible limit is not determined under any existing law;
- it is prohibited to use or include in food or food ingredients veterinary drug residues, antibiotic or growth promoter residues, active ingredients of drugs, microbes or parasites in excess to the maximum residue limit prescribed by regulations or by any other law for the time being in force;
- it is prohibited to store, market and sell food or food ingredient containing drug residues in excess to the maximum residue limit.

4.3.9 Policies that support the regulation of medicines with specific focus to antimicrobials

There is a multisectoral Drug Control Committee (DCC) headed by Secretary, Ministry of Health and Family Welfare (MOHFW) to provide the approval for exportation and importation of drugs including AMs. There is a technical committee in place to evaluate every medicine that may be manufactured or imported in order to determine its safety, efficacy and usefulness. With recommendations by the technical committee, the list of AMs is sent to DCC for approval. Recently, the DCC recommended few aspects of AMs in its 253rd board meeting that antibiotic manufacturers must put a red identification mark on the outside of the box before marketing with indication of a highlights “cannot be used without a prescription from a qualified doctor”.

4.3.10 Integration of the AWaRe classification of antibiotics in the national EML and BDNF Bangladesh National Formulary (BDNF)

There is no guideline on AWaRE classification of antibiotics in the animal health sector. The essential medicine list (EML) and over the counter (OTC) drug list is place in National Drug Policy 2016. Latest version of Bangladesh National Formulary (BDNF) was introduced by DGDA in 2019. BDNF is an online pharma indexing information providing tailored solutions for Bangladesh market that comprises >30,000 prescription drugs, over-the-counter medicines and natural products. The AWaRe classification of antibiotics was developed in 2017 by the WHO

expert committee on selection and use of essential medicines as a tool to support antibiotic stewardship efforts at local, national and global levels, Antibiotics are classified into three groups, Access, Watch and Reserve, taking into account the impact of different antibiotics and antibiotic classes on antimicrobial resistance, to emphasize the importance of their appropriate use. In 2021, AWaRe classification was updated and included an additional 78 antibiotics and brought total 258.

4.3.11 Up-to-date clinical guidelines that include AMS principles and integrate the AWaRe classification of antibiotics

There is no clinical guideline in place that include AMS principles and integrate the AWaRE classification of antibiotics in animal health sector.

4.3.12 Regulations on fixed-dose combinations of antibiotics

There are no regulations on fixed-dose combinations of antibiotics but it is discouraged in the national drug policy 2016. The drug control committee (DCC) also discouraged the producer and importer regarding fixed dose combinations of antibiotics.

4.3.13 Regulations on prescription of sale of antimicrobials

As per National Drug Policy 2016, there is a provision of regulations for sale of AMs. In addition, prescription of sale of AMs is also discussed in new drug act, 2022 which is now in cabinet division for final approval from the competent authority. The sale of antibiotic is prohibited without the prescription of a registered veterinarian. Selling of antibiotics without a prescription is a criminal offense and the penalty is 20,000 Bangladeshi Taka. Besides, The High Court (HC) division of Bangladesh directed the Government to take necessary steps to stop the sale of antibiotics without registered doctors' prescriptions (25th April, 2019, High Court Directives).

4.3.14 Regulation and enforcement of prescription-only dispensing of antimicrobials

There is a guideline in the national drug policy, 2016 on prescription-only dispending of AMs. The sale of antibiotic is prohibited without the prescription of a registered veterinarian.

4.3.15 Regulations for continued availability of quality antimicrobials

As per drug act, 1940 and national drug policy 2016, there is a provision of regulations for continued availability of quality antimicrobials for human and animals. The DGDA examine the quality of AMs at the National Control Laboratory (NCL) to identify falsified, counterfeit and poor quality. DGDA has two central NCLs in Dhaka and Chittagong and eight minilabs are located across the country to identify the quality of AMs. The NCL has issued a comprehensive quality manual that undergoes periodic revisions (7 revisions has been done in the period 2016-

2021) and is aligned with internationally recognized ISO 17025:2017 and WHO standards. The quality manual describes the quality system and the procedures to assess quality, efficacy, potency and toxicity of any drugs, vaccines and biological products produced or imported in the country.

4.3.16 Regulations in place to ensure affordability of essential antimicrobials

The national drug policy, 2016 directed to producer and importer to ensure affordability of essential AMs. The DGDA regularly sets prices for essential medicines and AMs.

4.3.17 A system of regulating the promotional activities of pharmaceuticals companies

There is a regulation in place on promotional activities of pharmaceutical companies. Following the guidelines of drug act 1940, drug control ordinance 1982 and national drug policy 2016, every manufacturer must approve their promotional materials from the DGDA prior to distribution. Promotional activities of pharmaceutical industry are strictly controlled and regulated by DGDA. There is a guideline in national drug policy about the promotional activities of the companies.

4.4 Marketing Authorization, Licensing and Inspection of Antimicrobials

4.4.1 Marketing authorization of the antimicrobials

Multisectoral guideline of marketing authorization for both human and animal health are in place. Drug act, 1940 and subsequent Govt. gazette of 2020, and the drug (control) ordinance 1982 guided every manufacture must take marketing authorization from DGDA. The guideline for post marketing authorization of AMs was developed by DGDA in 2018. The guideline for post marketing authorization variations is a very important document to control and maintain the quality, safety and efficacy of registered AMs. There is a core working committee who look after the marketing authorization of AMs. Antibacterial agents are only allowed access to the market after quality and safety assessments;

4.4.2 Licensing of antimicrobial premises/ drug shops

The DGDA is the competent national authority responsible to provide license to drug shops. A retail drug license is mandatory for operating a medicine selling business throughout the country. According to the national drug policy, 2016 licensed drug shops can only be permitted to dispense medicines but antibiotics and other prescription drugs can only be dispensed against a registered physician’s prescription, with full course.

4.4.3 Pharmacy guidelines

The drug act, 1940 instructed the AMs must be sold under the supervision of a Pharmacist. Following the model pharmacies guidelines, model pharmacies are allowed to stock and sell all prescription-only medicines registered by DGDA. Model pharmacies are allowed to stock and sell all non-prescription/ OTC medicines registered by DGDA. The aim of the model pharmacy is to reinforce regulations in the retail pharmacy business and achieve service uniformity among pharmacies and medicine shops, thereby maximizing patient care while minimizing health hazards. Model pharmacy is a community-based pharmacist whose responsibilities include: checking and dispensing of prescription drugs, providing advice on drug selection and usage to doctors and other health professionals and counseling patients in health promotion, disease prevention and the proper use of medicines. Model pharmacies are subject to periodic regulatory inspection. Any person who violate any provision of these standards shall be liable upon conviction to a warning, fine, imprisonment as specified under the existing acts, ordinance, and rules.

4.5 Post Marketing Surveillance and Pharmacovigilance

4.5.1 Post marketing quality control

There is a post marketing quality control system of AMs for both human and animal consumption. As per drug act, 1940 there is a provision of post marketing quality control of AMs. During post marketing quality control, antimicrobial sampling is performed at various levels of the national drug distribution chain for quality evaluation. The National Control Laboratory (NCL) of DGDA is the referral laboratory for quality testing of AMs. The NCL got the World Health Organization's accreditation of prequalification for ensuring quality, safety and efficacy of AMs.

4.5.2 Pharmacovigilance (PV)

Pharmacovigilance promotes public health by ensuring the safety, efficacy and quality of AMs and other health products. In 2013, the Ministry of Health and Family Welfare (MOHFW) declared the Adverse Drug Reaction Monitoring (ADRM) cell of the Directorate General of Drug Administration (DGDA) as the National Pharmacovigilance Centre (NPC) for Bangladesh. The MOHFW formed an Adverse Drug Reaction Advisory Committee (ADRAC) to work in collaboration with ADRM cell to provide technical guidance for implementing PV activities, evaluating Adverse Drug Event (ADE) reports, recommending regulatory decisions and actions to ensure AMs safety.

The PV system in Bangladesh is focused on detecting, evaluating and preventing adverse events related to AMs and other pharmaceutical products by managing and mitigating the risk pose to animals. ADR emphasizes reporting on medication error as well as product quality issue.

The objectives of national PV system in Bangladesh are:

- monitoring and evaluation of antimicrobial safety, effectiveness and quality problems through passive and active surveillance of adverse events;
- assessing the safety quality, effectiveness, risk/ benefit of pharmaceutical products;
- disseminating information on the safety and appropriate use of antimicrobials to the animal health professionals to mitigate risk;
- monitoring and engaging pharmaceuticals manufacturers and other supplier in the pharmaceutical industry to ensure that marketed AMs are high quality and safe for animal consumption;

4.6 Prescription, Dispensing and Use of Antimicrobials

4.6.1 Pattern of antibiotic dispensing at pharmacies according to the WHO Access, Watch, Reserve (AWaRe) Classification

There is no national policy on dispensing of AMs according to WHO classification (AWaRe). The WHO Essential Medicines List Access, Watch, and Reserve (AWaRe) classification could facilitate antibiotic stewardship and optimal use. In Bangladesh, data on antibiotic dispensing in pharmacies according to the AWaRe classification are scarce. A survey report predicted that consumer purchased mostly watch group antimicrobials (53.6%), followed by access (36.4%) and reserve (10%) groups. These findings highlight the need to have policies and its enforcement.

4.6.2 Antibiotic prescribing and dispensing in accordance with national drug policies

National drug policy, 2016 guided about the antibiotic prescribing and dispensing. Without prescription of a registered veterinarian antibiotic dispensing is prohibited. A DGDA survey is going on dispensing and disposal practices of pharmacy retailer across the country. A recent study jointly conducted by DGDA and WHO reported that antibiotic sales increased by 31% in 2021 and most antibiotics were sold without a doctor's prescription.

4.6.3 Antimicrobial supply chain in animal health

There 818 AMs were registered by DGDA for veterinary uses. The most common antibiotic classes, comprising over half (55%) of all licensed antibiotics, were: fluoroquinolone (18%), tetracycline (17%), penicillin (10%), and sulfonamide (10%).

4.7 Awareness, Training and Education

4.7.1 Regular public antibiotic awareness campaigns

Antibiotic awareness campaigns such as World Antimicrobial Awareness Week and other targeted campaigns are regularly organized to build awareness among the public.

4.7.2 Education in schools on basic infection principles

The existing school education curriculum contains few ideas on basic infection prevention and control (IPC) principles, such as hand hygiene, water hygiene, and mass vaccination.

4.7.3 Training on AMR competencies for AMS team members

There is no such training on AMR competencies for AMS team members in animal health sector.

4.7.4 Continuous education for all health-care professionals on AMS

The government has limited in-service training opportunities for the state veterinarian in antimicrobial prescribing and stewardship. Under the Livestock and Dairy Development Project (LDDP) more than thousands public veterinarian has been trained up on the basic concepts and intervention measures of AMR and AMS. In addition, BARC, BLRI, FAO and Fleming Fund also offers training programs for the veterinarian on different cross cutting issues of the AMR.

4.7.5 Incentives to support implementation of AMS programmes in all animal health-care facilities, including staffing standards, training and accreditation

There are no incentives to support of AMS programmes in animal health care facilities.

4.8 AMR Surveillance Capacities and Data Sharing Mechanism

4.8.1 National surveillance on antimicrobial use/ consumption (AMU/AMC) in place

The national AMU surveillance strategy for human and animal health has been drafted recently by the DGDA. In 2017 Directorate General of Drug Administration formed the "Task Force to Monitor Antimicrobial Consumption in Bangladesh". The main responsibilities of this Task Force was approval of the methodology to study antimicrobial consumption in Bangladesh with appropriate modification of the WHO methodology. A series of meeting has been conducted to develop the methodology to conduct AMC surveillance in Bangladesh. In 2021 Ministry of Health and Family Welfare reformed the "Task Force" to monitor antimicrobial consumption and antimicrobial use surveillance in Bangladesh.

Directorate General of Drug Administration has developed draft guideline on Antimicrobial Consumption (AMC) surveillance in Bangladesh on June 2022. DGDA plays the role as the National center for Antimicrobial Consumption (AMC) surveillance in Bangladesh based on

official nomination of Ministry of Health and Family Welfare. The nominated national focal and an alternate national focal person coordinate the overall surveillance activities including data sharing with global antimicrobial resistance and use surveillance system, the collaboration with World Health Organization and other relevant developments partners. Ultimately the "AMR Cell" of DGDA involve to conduct the AMC surveillance every year.

4.8.2 National surveillance system on AMR in place

There is no regular national surveillance of antimicrobial resistance in animal health sector. Though there is a multisectoral national AMR surveillance strategy (2020-2025) in place. Nevertheless, there is a sporadic AMR surveillance conducted by the research and academic institutes for their research and academic interest.

4.8.3 Facilities of antimicrobial susceptibility testing (AST)

Limited facilities for antimicrobial susceptibility testing are available in the field of animal health. A central (CDIL) and two sentinel laboratories (FDILs) of DLS is designated and has capacity to perform AST. Upazila Veterinary Hospital (UVH) and District Veterinary Hospital (DVH) has no capacity to perform AST. Bangladesh Livestock Research Institute (BLRI) has state of art facilities for AST. In addition, few academic institutes, veterinary schools and microbiology departments of state universities has AST facilities.

4.8.4 AMR data sharing platform in animal health

DLS has developed Bangladesh Animal Health Intelligence System (BAHIS) digital platform as a AMU and AMR data sharing platform. There is a AMR sectoral working group is place in animal health. The focal person of AMR sectoral group is designated to upload the AMR data to BAHIS.

4.8.5 Diagnostic tests available and capacity building undertaken to optimize antibiotic use

Disease diagnostic facilities are not available for animal health at the field conditions. Disease diagnosis of animal health mostly depends on symptomatic and post mortem examination (poultry). Rather, the molecular disease diagnostic capacity is limited in animal health.

5 World Organization for Animal Health (WOAH) guidelines for responsible and prudent use of antimicrobial agents in veterinary medicine

The WOAH provides guidance for the responsible and prudent use of antimicrobial agents in veterinary medicine, with the aim of protecting both animal and human health as well as the environment. It defines the respective responsibilities of the Competent Authority and

stakeholders such as the veterinary pharmaceutical industry, veterinarians, animal feed manufacturers, distributors and food animal producers who are involved in the authorisation, production, control, importation, exportation, distribution and use of veterinary medicinal products (VMP) containing antimicrobial agents.

5.1 WOAH Guiding principles of responsible and prudent use of antimicrobials

- ensuring the rational use of antimicrobial agents in animals with the purpose of optimising both their efficacy and safety;
- complying with the ethical obligation and economic need to keep animals in good health;
- preventing or reducing the transfer of resistant micro-organisms or resistance determinants within animal populations, the environment and between animals and humans;
- contributing to the maintenance of the efficacy and usefulness of antimicrobial agents used in animal and human medicine;
- protecting consumer health by ensuring the safety of food of animal origin with respect to residues of antimicrobial agents.

5.2 Marketing authorization guidelines

All Member Countries should combat the unauthorised manufacture, compounding, importation, advertisement, trade, distribution, storage and use of unlicensed, adulterated and counterfeit products, including bulk active ingredients, through appropriate regulatory controls and other measures.

5.3 Quality control of antimicrobial agents

Quality controls should be performed:

- in compliance with the provisions of good manufacturing practices;
- to ensure that analysis specifications of AMs used as active ingredients comply with the provisions of registration documentations (such as monographs) approved by the relevant Competent Authority;
- to ensure that the quality of AMs in the marketed dosage forms is maintained until the expiry date, established under the recommended storage conditions;
- to ensure the stability of AMs when mixed with feed or drinking water;

- to ensure that all AMs and the VMP containing them are manufactured to the appropriate quality and purity in order to guarantee their safety and efficacy.

5.4 Responsibilities of the veterinary pharmaceutical industry with regards to VMP containing antimicrobial agents

5.4.1 Marketing authorization

The veterinary pharmaceutical industry has responsibilities to:

- supply all the information requested by the national Competent Authority;
- guarantee the quality of this information in compliance with the provisions of good manufacturing, laboratory and clinical practices;
- implement a pharmacovigilance programme and on request, specific surveillance for bacterial susceptibility and resistance data.

5.4.2 Marketing and export

For the marketing and export of VMP containing antimicrobial agents:

- only licensed and officially approved VMP containing AMs should be sold and supplied, and then only through licensed/authorised distribution systems;
- the pharmaceutical industry should provide quality certificates prepared by the Competent Authority of the exporting and manufacturing countries to the importing country;
- the national regulatory authority should be provided with the information necessary to evaluate the amount of AMs marketed.

5.4.3 Advertising

The veterinary pharmaceutical industry should respect principles of responsible and prudent use and should comply with established codes of advertising standards, including to:

- distribute information in compliance with the provisions of the granted authorisation;
- not advertise VMP containing AMs directly to the food animal producer.

5.5 Responsibilities of wholesale and retail distributors

- distributors of VMP containing AMs should only do so on the prescription of a veterinarian or other suitably trained person authorised to prescribe VMP containing AMs in accordance with the national legislation and under the supervision of a veterinarian. All products should be appropriately labelled;

- recommendations on the responsible and prudent use of VMP containing AMs should be reinforced by retail distributors who should keep detailed records of:

  a) date of supply,

  b) name of prescriber,

  c) name of user,

  d) name of product,

  e) batch number,

  f) expiration date,

  g) quantity supplied,

  h) copy of prescription.

- Distributors should also be involved in training programmes on the responsible and prudent use of VMP containing AMs.

5.6 Responsibilities of veterinarians

The veterinarian's responsibility is to promote public health, animal health and animal welfare, including identification, prevention and treatment of animal diseases. The promotion of sound animal husbandry methods, hygiene procedures, biosecurity and vaccination strategies can help to minimise the need for AMs use in food-producing animals. Veterinarians should only prescribe AMs for animals under their care.

5.6.1 Use of antimicrobial agents

The responsibilities of veterinarians are to carry out a proper clinical examination of the animal(s) and then:

- administer or prescribe AMs only when necessary and taking into consideration the WOAH list of AMs of veterinary importance;
- make an appropriate choice of AMs based on clinical experience and diagnostic laboratory information (pathogen isolation, identification and antibiogram) where possible;
- provide a detailed treatment protocol, including precautions and withdrawal times, especially when prescribing extra-label or off-label use.

5.6.2 Choosing antimicrobial agents

A. The expected efficacy of the treatment is based on:

- the clinical experience of the veterinarians, their diagnostic insight and therapeutic judgement;

- diagnostic laboratory information (pathogen isolation, identification and antibiogram);
- pharmacodynamics including the activity towards the pathogens involved;
- the appropriate dosage regimen and route of administration;
- pharmacokinetics and tissue distribution to ensure that the selected therapeutic agent is effective at the site of infection;
- the epidemiological history of the rearing unit, particularly in relation to the antimicrobial resistance profiles of the pathogens involved.

B. Use of combinations of AMs should be scientifically supported. Combinations of AMs may be used for their synergistic effect to increase therapeutic efficacy or to broaden the spectrum of activity.

5.6.3 Recording of data

Records on VMP containing AMs should be kept in conformity with the national legislation. Information records should include the following:

- quantities of VMP used per animal species;
- a list of all VMP supplied to each food-producing animal holding;
- treatment schedules including animal identification and withdrawal period;
- antimicrobial susceptibility data;
- comments concerning the response of animals to treatment;
- the investigation of adverse reactions to antimicrobial treatment, including lack of response due to possible antimicrobial resistance. Suspected adverse reactions should be reported to the appropriate regulatory authorities.

5.6.4 Training and continued professional development

Veterinary professional organisations should participate in the training programmes. It is recommended that veterinary professional organisations develop for their members' species specific clinical practice recommendations on the responsible and prudent use of VMP containing AMs.

5.7 Responsibilities of Food Animal Producers

5.7.1 Food animal producers, with the assistance and guidance of a veterinarian, are responsible for implementing animal health and animal welfare programmes on their farms in order to promote animal health and food safety.

5.7.2 Food animal producers should:

a) draw up a health plan with the attending veterinarian that outlines preventive measures (e.g. feedlot health plans, mastitis control plans, endo- and ectoparasite control, vaccination programmes and biosecurity measures);

b) use VMP containing AMs only on the prescription of a veterinarian or other suitably trained person authorised to prescribe VMP containing AMs in accordance with the national legislation and under the supervision of a veterinarian;

c) use VMP containing AMs in accordance with product label instructions, including storage conditions, or the instructions of the attending veterinarian;

d) isolate sick animals, when appropriate, to avoid the transfer of pathogens, dispose of dead or dying animals promptly under conditions approved by the relevant authorities;

e) address on-farm biosecurity measures and take basic hygiene precautions as appropriate;

f) comply with and record the recommended withdrawal periods to ensure that residue levels in animal-derived food do not present a risk for the consumer;

g) use VMP containing AMs within the expiry date and dispose of unused and expired surplus VMP containing AMs under conditions safe for the environment;

h) maintain all the laboratory records of bacteriological and susceptibility tests, these data should be made available to the veterinarian responsible for treating the animals;

i) keep adequate records of all VMP containing AMs used, including the following:

- ✓ name of the product and active substance, batch number and expiry date;
- ✓ name of prescriber and the supplier;
- ✓ date of administration;
- ✓ identification of the animal or group of animals to which the antimicrobial agent was administered;
- ✓ clinical conditions treated;
- ✓ dosage;
- ✓ withdrawal periods including the end-date of the withdrawal periods;
- ✓ result of laboratory tests;
- ✓ effectiveness of therapy;

j) inform the responsible veterinarian of recurrent disease problems.

5.8 Responsibilities of Animal Feed Manufacturers

- The supply of medicated feed containing AMs to farmers keeping food-producing animals by animal feed manufacturers should be allowed only on the prescription of a veterinarian. Alternatively, such medicated feed may be prescribed by other suitably trained persons authorised to prescribe VMP containing AMs in accordance with the national legislation and under the supervision of a veterinarian;
- Animal feed manufacturers preparing medicated feed should do so following rules put in place by the Competent Authority in accordance with the national legislation. All medicated feed and medicated premixes should be appropriately labelled;
- The regulations and recommendations on the responsible and prudent use of VMP containing AMs should be reinforced by animal feed manufacturers who should keep detailed records;
- Animal feed manufacturers preparing medicated feed should ensure that only approved sources of medications are added to feed at a level, and for a species and purpose as permitted by the drug premix label or a veterinary prescription;
- Animal feed manufacturers preparing medicated feed should ensure that medicated animal feed is labelled with the appropriate information (e.g. level of medication, approved claim, intended species, directions for use, warning, cautions) so as to ensure effective and safe use by the producer;
- Animal feed manufacturers preparing medicated feed should implement appropriate production practices to avoid unnecessary carry over and unsafe cross contamination of unmedicated feed.

6. Recommendations

The assessment of antimicrobial stewardship policies and strategies in animal health revealed a lack of adequate regulatory regime. Most of the stewardship regulatory policies and guidelines are governed by the Directorate General of Drug Administration (DGDA). There is an inadequate coordination between the DGDA and DLS. The DGDA is mainly involved with their activities in human health. The DGDA is the Drug Regulatory Authority of the country. They supervise and implements all prevailing Drug Regulations in the country and regulates all activities related to import, procurement of raw and packing materials, production and import of finished

drugs, export, sales, pricing, etc. of all kinds of medicines including antimicrobials. The following recommendations can be considered to promote and strengthen antimicrobial stewardship policies and guidelines in animal health sector:

- development of antibiotic policy and user guidelines;
- strengthen the capacity of veterinary cell of DGDA with deployment of adequate human resources;
- deployment of more veterinarian as a drug inspector under DGDA;
- DGDA and DLS form joint task force to monitor the quality of AMs at the production and marketing outlet;
- strengthen the capacity of DLS to monitor the rational uses of AMs at the farm production;
- develop and enact legislation and regulations on the use of AMs in the farm animals;
- strengthen AMU and AMR surveillance in the animal health sector;
- building stronger collaboration and coordination between DGDA and DLS;
- DLS can be empowered to marketing authorization of the AMs used in animal health;
- DLS need to develop standard treatment guidelines for the infectious diseases for the practicing veterinarian;
- strengthen surveillance of antimicrobial use, prescription and consumption;
- strengthen integrated multi-sectoral surveillance of antimicrobial resistance;
- formation of sectoral technical working groups to regulate AMS;
- development of AMS policy and guidelines in animal health sector;
- dedicated funding for AMR and AMU surveillance;
- continuous professional development program for the veterinarian;
- increasing awareness and stakeholder engagement;
- promote integrated AMR monitoring and monitoring capabilities;
- creating an environment for accountability and transparency in the use of AMs;
- operationalize the One Health national multisectoral coordination platform to address the AMR and AMS issue in both sectors.

7. Conclusions

AMS is a coordinated program that promotes the appropriate use of antimicrobials (including antibiotics), improves patient outcomes, reduces microbial resistance, and decreases the spread of infections caused by multidrug-resistant organisms. The antimicrobial stewardship programs are greatly enhanced by interdepartmental communication and coordination. The regulatory aspect of AMS is a very important strategic focus for stemming the emergence of AMR. The assessment of antimicrobial policies and the regulatory framework is conducted under the Medicines, Technologies and Pharmaceutical Services Program, using the WHO toolkit along with different national and international guidelines. This assessment showed that Directorate General of Drug Administration (DGDA) is the prime competent authority for the development of AMS policies and guidelines in the animal health sectors. The production, importation, quality control, marketing authorization, post marketing surveillance, pharmacovigilance, dispensing and prescription auditing of the AMs are the responsibility of DGDA. The DGDA has demand driven policies and guidelines related to AMS but enforcement and surveillance is not up to the mark due to inadequate resource mobilizations. The lack of national policies and guidelines on the use of antibiotics is a major problem.

The Department of Livestock Services (DLS) is the national AMU and AMR surveillance authority. The DLS is the leading authority to coordinate and monitor AMR surveillance in the food producing animals and foods of animal origin. The rational use of AMs in the farm animals is the responsibility of the DLS. There is a lack of policies and regulations of DLS to monitor AMU and AMR in the animal health. The DLS should develop guidelines on the use of AMs in the farming practices for therapeutic, preventive and growth promotion purposes. DLS also take lead to develop standard treatment guidelines for the field veterinarians to ensure responsible use of AMs. DLS needs to take the lead in conducting AMU/ AMR surveillance nationwide and collate and compile AMU/ AMR national data for decision making. However, specifically, it is noted that there is a deficiency in the regulation of antimicrobial management in the animal health sector.

According to the assessment, the animal health sector is far behind and needs further improvement in AMS stewardship. The animal health sector is very weak in terms of regulation and it needs to be improved as soon as possible for consistent development of AMR and AMS situation in Bangladesh. The Bangladesh Veterinary Council can take initiative to develop ethics and regulations to confirm prudent use of AMs for animal welfare and protection of human health.

The DLS should develop marketing authorization guidelines of the veterinary AMs along with quality assessment system. Holistic approach of the regulations, governance and institutional capacity is essential to building a better AMS program.

8. Glossary

Antibiotic: The term "antibiotic" refers to an antimicrobial agent with the ability to kill or inhibit bacterial growth.

Antimicrobial: An agent or substance derived from any source (microorganisms, plants, animals, synthetic or semi-synthetic) that acts against any type of microorganism, such as bacteria (antibacterial), mycobacteria (anti-mycobacterial), fungi (antifungal), parasite (anti-parasitic) and viruses (antiviral). All antibiotics are antimicrobials, but not all antimicrobials are antibiotics.

Antimicrobial Resistance (AMR): Microorganisms such as bacteria, fungi, viruses and parasites change when exposed to antimicrobial drugs such as antibiotics (antibacterial), antifungals, antivirals, antimalarials and anthelmintics. As a result, the medicines become ineffective.

Antimicrobial Stewardship (AMS): A coherent set of actions which promote the responsible use of antimicrobials. This definition can be applied to actions at the individual level as well as the national and global level, and across human health, animal health and the environment.

Antimicrobial Stewardship Program (AMS program): An organizational or system-wide healthcare strategy to promote appropriate use of antimicrobials through the implementation of evidence based interventions.

Veterinary Medicinal Products: It means medicinal products which are manufactured, sold, supplied, imported or exported for the purpose of being administered to animals, but not for the purpose of being administered to human beings.

Essential Medicines List: The WHO essential medicines list contains a selection of medications based on disease prevalence and public health relevance that are considered to be most effective, safe and cost effective. The EML is intended to guide countries and institutions to develop their own list of essential medicines.

Legislations: The first stage of the legislative process, in which laws are passed by the legislative body of government with regard to a subject matter, e.g., control of pharmaceuticals. Laws define the roles, rights, and obligations of all parties involved in the subject matter in general terms.

Regulations: The second stage of the legislative process (the first stage being legislation) in which regulations are specifically designed to provide the legal machinery to achieve the administrative and technical goals of legislation.

Guidelines: Non-statutory advisory publications intended to assist those parties affected by legislation to interpret requirements.

Policy: Policy is a law, regulation, procedure, administrative action, incentive, or voluntary practice of governments and other institutions. Within the context of public health, policy development includes the advancement and implementation of public health law, regulations, or voluntary practices that influence systems development, organizational change, and individual behavior to promote improvements in health.

9. References

Antimicrobial stewardship programmes in health-care facilities in low- and middle-income countries. A practical toolkit. Geneva: World Health Organization; 2019. License: CC BY-NC-SA 3.0 IGO.

Bangladesh Food Safety Authority (BFSA) (http://www.bfsa.gov.bd)

Communicable Disease Control (CDC), DGHS. (https://dghs.gov.bd)

Department of Livestock Services (http://www.dls.gov.bd/)

Directorate General of Drug Administration (DGDA) (https://dgda.portal.gov.bd)

Directorate General of Drug Administration (https://dgda.portal.gov.bd/)

Drug Control Ordinance, 1982. Available at https://dgda.portal.gov.bd/site/view/law/Act-and-Rules (accessed on 16 July 2022)

Drug control ordnance – 2006. Available at https://dgda.portal.gov.bd/site/view/law/Act-and-Rules (accessed on 16 July 2022)

Drugs Act, 1940. Available at https://dgda.portal.gov.bd/site/view/law/Act-and-Rules (accessed on 16 July 2022)

Fish Feed and Animal Feed Act, 2010. Available at https://mofl.gov.bd/site/view/law/ (accessed on 16 July 2022)

Guideline on Antimicrobial Consumption (AMC) Surveillance in Bangladesh. Version. 01. July, 2022. Available at http://dgdagov.info/index.php/information-center/guidance-documents/2340-amc-surveillance-guideline-for-comments/file (accessed on 16 July 2016)

Guidelines for post marketing authorization pf variations, 2018. Available at https://dgda.portal.gov.bd/ (accessed on 16 July 2016)

Murshid, M.E. and Haque, M., 2019. Bangladesh national drug policy 1982-2016 and recommendations in policy aspects. Eurasian Journal of Emergency Medicine, 18(2), p.104.

National Drug Policy, 2016. Available at https://dgda.portal.gov.bd/site/view/law/Act-and-Rules (accessed on 16 July 2022)

OIE Standards, Guidelines and Resolution on antimicrobial resistance and the use of antimicrobial agent. World Organisation for Animal Health; 2105, 12, rue de Prony, 75017 Paris, France

Orubu, E.S.F., Samad, M.A., Rahman, M.T., Zaman, M.H. and Wirtz, V.J., 2021. Mapping the Antimicrobial Supply Chain in Bangladesh: A Scoping-Review-Based Ecological Assessment Approach. Global Health: Science and Practice, 9(3), pp.532-547.

Rahman, M.A., Hossain, M.R., Hossain, M.A. and Amran, M.S., 2021. A Study on the National Drug Policies of Bangladesh to Ensure Health for All. Dhaka University Journal of Pharmaceutical Sciences, 20(1), pp.41-48.

Rapid assessment of antimicrobial stewardship policies and regulatory framework in Burkina Faso. Published in March 2020

standards for the establishment and operations of model pharmacies and model medicine shops. Available at http://www.drugsellerinitiatives.org/publication/altview/standards-for-the-establishment-and-operation-of-model-pharmacies-and-model-medicine-shops-2016/PDF/ (accessed on 16 July 2022)

Tool to Support Conduct of Rapid Situational Analysis of Antimicrobial Stewardship Policies in MTaPS Program Countries

World Health Organization (WHO) (https://www.who.int/bangladesh/about-us/our-work)

World Organization for Animal Health (WOAH) (https://www.woah.int/en/home/)

Annexure 1: Assessment Tool for Antimicrobial Stewardship Policies, Guidelines and Practices in Animal Health Sector

Tools used: desk reviews of policies, guidelines and manuscript in peer review journal; stakeholder consultations, KII, FGD, personal communication and digital source

Sl. No	Assessment Elements	(Assessed) Yes/ No	Existence/ Adherence in National Animal Health Sector	Main Features in Alignment with AMS/ AMR/ AMU	Source of Information
1	National Plan and Strategies				
1.1	National Action Plan (NAP) on AMR that states AMS is a priority	Yes	AMS has been considered as a national priority at the animal health in the existing AMR national action plan	• The national action plan of AMR is the highest AMR state policy document;<br>• The current AMR strategy along with roadmap is effective for the duration of 2017-2021;<br>• Recently new AMR strategy and action plan has been developed for 2021-2026;	Desk review of national action plan (NAP) of AMR containment in Bangladesh. Available at (https://dghs.gov.bd); (https://www.who.int/publications/m/item/bangladesh-antimicrobial-resistance-containment-in-bangladesh-2017-2022
1.2	National AMU and AMR surveillance strategy	Yes	A multi-sectoral AMR surveillance strategy, including animal health, is in operation. AMU surveillance guideline also has been drafted..	• Multisectoral national AMR surveillance strategy (2020-2025) is in operation;<br>• National AMU surveillance guideline also has been drafted (June 2022);	Desk review of AMR surveillance strategy for 2020-2025. The strategy document was collected from Communicable Disease Control, DGHS. AMU surveillance guidelines was found in DGDA website.
1.3	Technical Working Group (TWG) on AMS with clear terms of reference	Yes	No TWG on AMS in animal health sector	• TWG on AMS has not yet been formed in animal health;	Review of documents and interviewed/ consulted with DLS

Sl. No	Assessment Elements	(Assessed) Yes/ No	Existence/ Adherence in National Animal Health Sector	Main Features in Alignment with AMS/ AMR/ AMU	Source of Information
1.4	National AMS implementation plan or policy	Yes	There is no national AMS implementation plan or policy in animal health	• National AMS implementation plan or policy has not yet been developed in animal health	Interviewed/ consulted with DLS
1.5	Monitoring and evaluation mechanism in place for the national action plan on AMR	Yes	Multisectoral monitoring and evaluation mechanism in place but no sectoral monitoring and evaluation mechanism specific for animal health	• A multisectoral core working group on AMR has been established;<br>• Multisectoral core working group monitor and evaluate the progress of national action plan on AMR;	Desk review of documents and consulted with CDC, DGHS and DLS
2	Leadership and Commitment				
2.1	Institutionalization of AMR issue	Yes	There is no organizational structure on AMR in animal health	• Yet to establish organizational set up at DLS on AMR	Consulted with DLS
2.2	Deployment of dedicated human resources	Yes	There is few legitimate staff to deal with AMR issues at animal health	• Shortage of dedicated professional staff to deal with AMR	Interviewed with DLS
2.3	Dedicated funding for the implementation of national action plan on AMR	Yes	There is no regular revenue budget to implement the NAP of AMR. Current AMR findings are	• No regular revenue budget in place<br>• Currently, the Livestock and Dairy Development Project (LDDP) is providing support for AMR policy interventions;	Personal communication and interview

Sl. No	Assessment Elements	(Assessed) Yes/ No	Existence/ Adherence in National Animal Health Sector	Main Features in Alignment with AMS/ AMR/ AMU	Source of Information
			either from project based or overseas resource or grant.	• Overseas resources (Fleming Fund, FAO and MTaPS) are currently providing support;	
3	Policies, Regulations and Guidelines on Antimicrobial Stewardship				
3.1	Drug Act, 1940 and Subsequent Govt. gazette of 2020	Yes	The drug act, 1940 has been enacted in the British regime to regulate the import, export, manufacture, distribution and sale of drugs including AMs. This is applicable for both sectors.	The main features of the drug act, 1940 are (i) guidelines for the import and export of drugs, and (ii) manufacture, sales and distribution of drugs. The important milestones related to AMS found in the drug act, 1940 are:<br>• There is a prohibition of manufacture and sale of drugs which is not a standard quality and misbrand;<br>• License is required before importation of any drugs including AMs;<br>• Require the date of manufacture and the date of expiry of potency to be clearly and truly stated on the label of drugs;<br>• Every manufacturer must approve their promotional materials from the competent authority prior to distribution;<br>• Every manufacture must take marketing authorization from the competent authority.	Review of drugs act, 1940. Available at DGDA website https://dgda.portal.gov.bd/site/view/law/Act-and-Rules

Sl. No	Assessment Elements	(Assessed) Yes/ No	Existence/ Adherence in National Animal Health Sector	Main Features in Alignment with AMS/ AMR/ AMU	Source of Information
3.2	The Drug (Control) Ordinance, 1982	Yes	The drug (control) ordinance 1982 has been enacted in 1984 to control manufacture, import, distribution and sale of drugs. The ordinance addressed both human and animal.	The core components of the drug control ordinance are<br>• Formation of drug control committee;<br>• Registration of medicines before sale;<br>• Prohibition of manufacture of certain medicines;<br>• Restriction on import of certain pharmaceutical raw material;<br>• Fixation of price of drugs;<br>• Deployment of registered pharmacist in the pharmaceutical manufacturing industry;<br>• Control of advertisement and claims in respect of drugs;<br>• Prohibition of prescription of unregistered medicine;<br>• Good practices in the manufacture and quality control of drugs;<br>• Penalty for manufacture of certain banned drugs;<br>• Penalty for manufacture, stock or sale of sub-standard drugs;<br>• Penalty for unauthorised import of drugs;<br>• Penalty for illegal advertisement and claims;	Review of the drug control ordinance, 1982. Available at DGDA website: https://dgda.portal.gov.bd/site/view/law/Act-and-Rules

Sl. No	Assessment Elements	(Assessed) Yes/ No	Existence/ Adherence in National Animal Health Sector	Main Features in Alignment with AMS/ AMR/ AMU	Source of Information
				• Formation of drug courts;<br>• Formation of national drug advisory council, etc.	
3.3	National Drug Policy, 2016	Yes	A combined drug policy for human and animal health was developed by DGDA	National Drug Policy, 2016 addressed the following issues<br>• Good Manufacturing Practices of antimicrobials (AMs) to be ensured;<br>• To ensure rational and safe use of AMs through standard treatment guidelines;<br>• Each public and private health care settings must have antibiotic uses guidelines;<br>• Selling of antibiotics is prohibited without the prescription of registered physician and veterinarian;<br>• Monitoring of prescription and dispensing after certain intervals to ensure rational use assessment of AMs;<br>• To ensure the prudent use of AMs, each hospital should have "drugs and therapeutic committee"<br>• Each local or imported AMs must have licensing after the concurrence of drug control committee (DCC);	The national drug policy is available at DGDA website, (https://dgda.portal.gov.bd)

Sl. No	Assessment Elements	(Assessed) Yes/ No	Existence/ Adherence in National Animal Health Sector	Main Features in Alignment with AMS/ AMR/ AMU	Source of Information
				• Pharmacovigilance and adverse drug reaction (ADR) to be addressed;	
3.4	Fish Feed and Animal Feed Act, 2010	Yes	Specialized act for animal health	• The act deals for fish feed and animal feed processing, quality control, import, export, marketing, sale, distribution, transportation and pertaining ancillary matters;<br>• Any harmful chemicals including antibiotic, growth hormone, steroid and insecticides cannot be used in fish feed and animal feed;<br>• Anybody violating the provision of law will come under the purview of crime under this law;	Fish Feed and Animal Feed Act, 2010 is available in the following link (www.bangladeshtradeportal.gov.bd)
3.5	National Livestock Extension Policy, 2013	Yes	The National Livestock Extension Policy 2013 touches upon AMU and drug residue issues only marginally.	• The National Livestock Extension Policy 2013 touches upon AMU and drug residue issues only marginally, in the section for "emerging livestock development issues", as follows:<br>• Drug residues in food products of animal origin is a major food safety concern of present time, and effective control should be implemented following WOAH guidelines;<br>• AMU is strictly regulated in organic farming and bio-rational-based management systems	Review of national livestock extension policy, 2013, available at http://old.dls.gov.bd

Sl. No	Assessment Elements	(Assessed) Yes/ No	Existence/ Adherence in National Animal Health Sector	Main Features in Alignment with AMS/ AMR/ AMU	Source of Information
				should be disseminated in the farms to ensure productivity and products' safety and quality.	
3.6	National Livestock Development Policy, 2007	Yes	The National Livestock Development Policy 2007 touches upon AMU only marginally, in the "Poultry development" and "Veterinary services and animal health" sections	• The National Livestock Development Policy 2007 touches upon AMU only marginally, in the "Poultry development" and "Veterinary services and animal health" sections, by stating the following:<br>• The use of antibiotics in feeds is thought to be common and a cause of public health concern;<br>• No registration is required for feed additives such as toxins binder, antibiotics, and vitamin/mineral premixes, animal protein, many of which are potentially detrimental to human health;<br>• Most of the drugs traders and shop keepers have no formal training on drug handling, transportation, storing and dispensing, and readily sell drugs such as antibiotics, hormones, and sedatives across the counter without prescription.	Review of national livestock development policy, 2007 (http://old.dls.gov.bd/files/Livestock_Policy_Final.pdf)
3.7	Veterinary Practitioners Ordinance, 1982	Yes	Guidelines for the veterinarian	• This ordinance makes provision for the regulation, control and registration of veterinary practitioners and establishes that	• Review of veterinary practitioners ordinance, 1982 (dls.gov.bd)

Sl. No	Assessment Elements	(Assessed) Yes/ No	Existence/ Adherence in National Animal Health Sector	Main Features in Alignment with AMS/ AMR/ AMU	Source of Information
				veterinary medicine and surgery should be practised only by registered veterinary practitioners;<br>• It also includes the constitution of a Veterinary Council that, among other tasks, reviews and approves applications for registration of veterinary practitioners.	
3.8	Food Safety Act, 2013	Yes	General food safety act covers the food of animal origin	• This act may be called the Food Safety Act, 2013;<br>• The presence of veterinary drug residue beyond the permissible limit is not allowed in animal derived foods;	The food safety act is available in the following link http://www.bfsa.gov.bd/site/view/law/Food-Safety-Act,-2013
3.9	Policies that support the regulation of medicines with specific focus to antimicrobials	Yes	Drug Control Committee (DCC) is a multisectoral committee headed by Secretary, Ministry of Health and Family Welfare (MOHFW). There is another multisectoral technical subcommittee. The technical committee screening the AMs in	• The technical committee evaluate every medicine that may be manufactured or imported in order to determine its safety, efficacy and usefulness;<br>• With approval by the technical committee, the list of AMs will be sent to DCC for approval;<br>• The DCC decided few aspects of AMs in its 253rd board meeting. Antibiotic manufacturers must put a red identification mark on the outside of the box before marketing. Also indicates that it	The report of 253 sessions of DCC meeting. It is available in the following link (https://dgda.portal.gov.bd)

Sl. No	Assessment Elements	(Assessed) Yes/ No	Existence/ Adherence in National Animal Health Sector	Main Features in Alignment with AMS/ AMR/ AMU	Source of Information
			terms of quality and finally they recommend the list of AMs to the DCC for approval.	cannot be used without a prescription from a qualified doctor.	
3.10	Integration of the AWaRe classification of antibiotics in the national EML and BDNF Bangladesh National Formulary (BDNF)	Yes	There is no guideline on AWaRE classification of antibiotics in the animal health sector. The essential medicine list (EML) and over the counter (OTC) drug list is place in National Drug Policy 2016. Bangladesh National Formulary (BDNF) was introduced by DGDA in 2019	• The AWaRe classification of antibiotics was developed in 2017 by the WHO expert committee on selection and use of essential medicines as a tool to support antibiotic stewardship efforts at local, national and global levels, Antibiotics are classified into three groups, Access, Watch and Reserve, taking into account the impact of different antibiotics and antibiotic classes on antimicrobial resistance, to emphasize the importance of their appropriate use;<br>• In 2021, AWaRe classification was updated and included an additional 78 antibiotics and brought total 258;	The information is extracted from WHO in the following website (https://www.who.int/publications/i/item/2021-aware-classification)
3.11	Up-to-date clinical guidelines that include AMS principles and integrate the AWaRe classification of antibiotics	Yes	There is no clinical guideline in place that include AMS principles and integrate the AWaRE classification of	• There is no clinical guideline in place that include AMS principles and integrate the AWaRE classification of antibiotics in animal health sector	Personal communication and Key Informant Interview with DGDA and DLS

Sl. No	Assessment Elements	(Assessed) Yes/ No	Existence/ Adherence in National Animal Health Sector	Main Features in Alignment with AMS/ AMR/ AMU	Source of Information
			antibiotics in animal health sector		
3.12	Regulations on fixed-dose combinations of antibiotics	Yes	There is no standard guideline on dose combination of antibiotics in livestock even in human health sector;	• There is no regulations on fixed-dose combinations of antibiotics but it is discouraged by the national drug policy 2016 as well as DCC to the producer and importer;	Review of literature and policy documents. Consulted with DGDA official (KII)
3.13	Regulations on prescription of sale of antimicrobials	Yes	There is a guideline in the national drug policy, 2016 on prescription of sale of antimicrobials. In addition, the issue of prescription of sale of AMs is discussed in new drug act, 2022 which is now in cabinet division for necessary concurrence	• The sale of antibiotic is prohibited without the prescription of a registered veterinarian;<br>• Selling of antibiotics without a prescription is a criminal offense and the penalty is Tk. 20,000;<br>• The High Court (HC) division of Bangladesh directed the Government to take necessary steps to stop sale of antibiotics without registered doctors' prescriptions (25th April, 2019, High Court Directives)	This guideline is available in National Drug Policy, 2016 (https://dgda.portal.gov.bd); personal communication with DGDA official (KII); The high court directive is available in the following link (https://www.thedailystar.net/city/take-steps-to-stop-sale-of-antibiotics-without-prescriptions-1734625)
3.14	Regulation and enforcement of prescription-only dispensing of antimicrobials	Yes	There is a guideline in the national drug policy, 2016 on prescription-only dispending of antimicrobials	• The sale of antibiotic is prohibited without the prescription of a registered veterinarian;	This guideline is available in National Drug Policy, 2016 (https://dgda.portal.gov.bd); desk review and interviewed DGDA official.

Sl. No	Assessment Elements	(Assessed) Yes/ No	Existence/ Adherence in National Animal Health Sector	Main Features in Alignment with AMS/ AMR/ AMU	Source of Information
				• Selling of antibiotics without a prescription is a criminal offense and the penalty is Tk. 20,000;<br>• The High Court (HC) division of Bangladesh directed the Government to take necessary steps to stop sale of antibiotics without registered doctors' prescriptions (25th April, 2019, High Court Directives)	
3.14	Regulations for continued availability of quality antimicrobials	Yes	National drug policy, 2016 guided to ensure continued availability of quality AMs in animal health.	• As per national drug policy, 2016, there is a provision of regulations for continued availability of quality AMS for human and animals;<br>• The DGDA examine the quality of AMs at the National Control Laboratory (NCL) to identify falsified, counterfeit and poor quality;<br>• DGDA has two central NCLs in Dhaka and Chittagong and eight minilabs nationwide;	This guideline is available in National Drug Policy, 2016 (https://dgda.portal.gov.bd) and Key Informant Interview (KII)
3.16	Regulations in place to ensure affordability of essential antimicrobials	Yes	National drug policy, 2016 ensured affordability of essential AMs	• The national drug policy, 2016 directed to producer and importer to ensure affordability of essential AMs	This guideline is available in National Drug Policy, 2016 (https://dgda.portal.gov.bd)

Sl. No	Assessment Elements	(Assessed) Yes/ No	Existence/ Adherence in National Animal Health Sector	Main Features in Alignment with AMS/ AMR/ AMU	Source of Information
				• DGDA regularly sets prices for essential medicines;	
3.17	A system of regulating the promotional activities of pharmaceuticals companies	Yes	There is a regulation in place on promotional activities of pharmaceutical companies	• As per drug control ordinance 1982 and drug act 1940, every manufacturer must approve their promotional materials from DGDA prior to distribution;<br>• Promotional activities of pharmaceutical industry were strictly controlled and regulated by DGDA;<br>• There is a direction in national drug policy about the promotional activities of the companies;	This guideline is available in National Drug Policy, 2016 (https://dgda.portal.gov.bd) and interviewed DGDA official (KII)
4	Marketing Authorization, Licensing and Inspection of Antimicrobials				
4.1	Marketing authorization of the antimicrobials	Yes	Multisectoral guideline for marketing authorization for both human and animal health in place	• Drug act, 1940 and subsequent Govt. gazette of 2020, every manufacture must take marketing authorization from DGDA;<br>• The guideline for post marketing authorization of AMs was developed by DGDA in 2018;<br>• The guideline for post marketing authorization variations is a very important document to control and maintain the quality, safety and efficacy of registered AMs;<br>• There is a core working committee who look after the marketing authorization of AMs;	Consulted the document of guideline for post marketing authorization variations 2018. The guideline is available in the following link (https://dgda.portal.gov.bd) and KII

Sl. No	Assessment Elements	(Assessed) Yes/ No	Existence/ Adherence in National Animal Health Sector	Main Features in Alignment with AMS/ AMR/ AMU	Source of Information
				• Antibacterial agents are only allowed access to the market after quality and safety assessments;	
4.2	Licensing of antimicrobial premises/ drug shops	Yes	Licensing of animal drug shops are mandatory as per provision of DGDA	• As per drug act, 1940 all drugstores must be licensed before opening;<br>• The DGDA is the competent national authority responsible to provide license to drug shops;<br>• A retail drug license is mandatory for operating a medicine selling business throughout the country;<br>• According to the national drug policy, licensed drug shops can only be permitted to dispense medicines but antibiotics and other prescription drugs can only be dispensed against a registered physician's prescription, as a full course	The guideline for applying of drugstore registration is available in the following link (https://dgda.portal.gov.bd) and interviewed DGDA official (KII)
4.3	Pharmacy guidelines	Yes	DGDA has made guideline on standards for the establishment and operations of model pharmacies and model medicine shop in 2016	• As per drug act, 1940 medicine must be sold under the supervision of a Pharmacist;<br>• Model pharmacies will be allowed to stock and sell all prescription-only medicines registered by DGDA;<br>• Model pharmacies will be allowed to stock and sell all non-prescription / OTC medicines registered by DGDA;	The guideline for applying of drugstore registration is available in the following link (https://dgda.portal.gov.bd) and interviewed DGDA official

Sl. No	Assessment Elements	(Assessed) Yes/ No	Existence/ Adherence in National Animal Health Sector	Main Features in Alignment with AMS/ AMR/ AMU	Source of Information
				• Model pharmacy is a community-based pharmacist whose responsibilities include: checking and dispensing of prescription drugs, providing advice on drug selection and usage to doctors and other health professionals and counseling patients in health promotion, disease prevention and the proper use of medicines;<br>• Model pharmacies are subject to periodic regulatory inspection. Any person who violate any provision of these standards shall be liable upon conviction to a warning, fine, imprisonment as specified under the existing acts, ordinance, and rules.	
5	Post Market Surveillance and Pharmacovigilance				
5.1	Post marketing quality control	Yes	There is a post marketing quality control system of antimicrobials are prevailing for both human and animal health	• As per drug act, 1940 there are provision of post marketing quality control of AMs;<br>• The process by which antimicrobial sampling is performed at various levels of the national drug distribution chain for quality evaluation;<br>• The National Control Laboratory (NCL) of DGDA is the referral laboratory for quality testing of AMs;	Desk review of the documents and in person communication with DGDA officials (KII)

Sl. No	Assessment Elements	(Assessed) Yes/ No	Existence/ Adherence in National Animal Health Sector	Main Features in Alignment with AMS/ AMR/ AMU	Source of Information
				• The NCL achieved the World Health Organization's accreditation of prequalification for ensuring quality, safety and efficacy of AMs;	
5.2	Pharmacovigilance	Yes	Bangladesh has a national pharmacovigilance policy and guideline as well as gazette related to both human and animal health	• Monitoring and detecting AMs safety, effectiveness and quality through passive and active surveillance of adverse effects;<br>• Assessing the safety, quality and efficacy and risk of antimicrobials;<br>• Disseminating information on safety and appropriate use of AMs to the animal health;<br>• To ensure the quality and safety of marketed AMs both local manufacturing and importing for animal consumption;	Consulted national policy and guideline on the pharmacovigilance system in Bangladesh. The document is available in the following link (https://dgda.portal.gov.bd)
6	Prescription, Dispensing and Use of Antimicrobials				
6.1	Pattern of antibiotic dispensing at pharmacies according to the WHO Access, Watch, Reserve (AWaRe) Classification	Yes	There is no national policy on dispensing of AMs according to WHO classification (AWaRe)	• The WHO Essential Medicines List Access, Watch, and Reserve (AWaRe) classification could facilitate antibiotic stewardship and optimal use;<br>• In Bangladesh, data on antibiotic dispensing in pharmacies according to the AWaRe classification are scarce;<br>• A survey report predicted that consumer purchased mostly watch group antimicrobials (53.6%), followed by access	Consulted the published manuscripts on "Pattern of Antibiotic Dispensing at Pharmacies According to the WHO Access, Watch, Reserve (AWaRe) Classification in Bangladesh, doi: 10.3390/antibiotics11020247

Sl. No	Assessment Elements	(Assessed) Yes/ No	Existence/ Adherence in National Animal Health Sector	Main Features in Alignment with AMS/ AMR/ AMU	Source of Information
				(36.4%) and reserve (64, 10.0%) groups;<br>• These findings highlight the need to have policies and its enforcement;	
6.2	Antibiotic prescribing and dispensing in accordance with national drug policies	Yes	Antibiotic prescribing and dispensing guideline is place in national drug policy, 2016.	• National drug policy, 2016 guided about the antibiotic prescribing and dispensing;<br>• Without prescription of a registered veterinarian antibiotic dispensing is prohibited;<br>• A DGDA survey is going on dispensing and disposal practices of pharmacy retailer across the country;<br>• A recent study jointly conducted by DGDA and WHO reported that antibiotic sales increased by 31% in 2021 and most antibiotics were sold without a doctor's prescription;	The review of national drug policy, 2016 which is available in the following link (https://dgda.portal.gov.bd)
6.3	Antimicrobial supply chain in animal health	Yes	Antimicrobials are either locally produced or imported for animal health	• There 818 AMs were registered by DGDA for veterinary uses;<br>• The most common antibiotic classes, comprising over half (55%) of all licensed antibiotics, were: fluoroquinolone (18%); tetracycline (17%); penicillin (10%); and sulfonamide (10%)	Consulted the publication on "mapping the antimicrobial supply chain in Bangladesh: A scoping-review-based ecological assessment approach (https://www.ghspjournal.org/content/9/3/532)
7	Awareness, Training and Education				

Sl. No	Assessment Elements	(Assessed) Yes/ No	Existence/ Adherence in National Animal Health Sector	Main Features in Alignment with AMS/ AMR/ AMU	Source of Information
7.1	Regular public antibiotic awareness campaigns	Yes	Antimicrobial awareness week is celebrated every year with festive mood in animal health	• Antibiotic awareness campaigns such as World Antimicrobial Awareness Week and other targeted campaigns are regularly organized to build awareness among the public	Consultation with Department of Livestock Services and KII
7.2	Education in schools on basic infection principles	Yes	-	• The existing school education curriculum contains few ideas on basic infection prevention and control (IPC) principles, such as hand hygiene, water hygiene, and mass vaccination.	Desk review of school curricula
7.3	Training on AMR competencies for AMS team members	Yes	There is no such training on AMR competencies for AMS team members in animal health sector	• There is no structural AMS team for animal health;	Interview with AMR sectoral focal person of DLS
7.4	Continuous education for all health-care professionals on AMS	Yes	There is limited scope of continuous education for health care professionals on AMS in animal health sector	• The government has limited in-service training opportunities for the state veterinarian in antimicrobial prescribing and stewardship;<br>• Under the Livestock and Dairy Development Project (LDDP) more than thousands public veterinarian has been trained up on the basic concepts and intervention measures of AMR and AMS;	Interview with AMR sectoral focal person of DLS

Sl. No	Assessment Elements	(Assessed) Yes/ No	Existence/ Adherence in National Animal Health Sector	Main Features in Alignment with AMS/ AMR/ AMU	Source of Information
				• In addition, BARC, BLRI, FAO and Fleming Fund also offers training programs for the veterinarian on different cross cutting issues of the AMR	
7.5	Incentives to support implementation of AMS programmes in all animal health-care facilities, including staffing standards, training and accreditation	Yes	There is no incentives to support of AMS programmes in animal health care facilities	• No such provision in animal health sector regarding incentives of AMS programmes	Interview with Interview with AMR sectoral focal person of DLS
8	AMR Surveillance Capacities and Data Sharing Mechanism				
8.1	National surveillance on Antimicrobial Use (AMU) in place	Yes	There is no national surveillance of AMU in place	• A national AMU surveillance strategy for animal health has not yet been developed;<br>• A task force was formed led by Director General, DGDA to monitor the AMU and AMC in animal and human health;	Interview with DLS and DGDA officials and desk review the documents
8.2	National surveillance system on AMR in place	Yes	There is no national surveillance of antimicrobial resistance in animal health led by the National Livestock Department	• There is a multisectoral national AMR surveillance strategy (2020-2025);<br>• Sporadic AMR surveillance is conducted by research and academic institutes;	Interview with DLS officials and desk review of the documents
8.3	Facilities of antimicrobial susceptibility testing (AST)	Yes	Limited facilities for antimicrobial susceptibility testing	• A central (CDIL) and two sentinel laboratories (FDILs) of DLS has capacity to perform AST;	Review of the documents and personal communications

Sl. No	Assessment Elements	(Assessed) Yes/ No	Existence/ Adherence in National Animal Health Sector	Main Features in Alignment with AMS/ AMR/ AMU	Source of Information
			are available in the field of animal health	• Upazila Veterinary Hospital (UVH) and District Veterinary Hospital (DVH) has no capacity to perform AST;<br>• Bangladesh Livestock Research Institute (BLRI) has state of art facilities for AST;<br>• Few academic institutes, veterinary schools and microbiology departments of state universities has AST facilities;	
8.4	AMR data sharing platform in animal health	Yes	DLS has developed Bangladesh Animal Health Intelligence System (BAHIS) digital platform as a AMR data sharing platform	• There is a AMR sectoral working group in place in animal health;<br>• The focal person of AMR sectoral group are responsible to upload the AMR data to BAHIS;	Interview with DLS officials
8.5	Diagnostic tests available and capacity building undertaken to optimize antibiotic use	Yes	Disease diagnostic facilities are not available for animal health under field conditions	• Disease diagnosis of animal health mostly depends on symptomatic and post mortem examination (poultry);<br>• The molecular disease diagnostic capacity are limited in animal health	Desk review of the reports and interview with the DLS officials

Abu Zahid

Digitally signed by Abu Zahid
DN: cn=Abu Zahid, o=MSH, ou=Team Lead, MTaPS, email=azahid@mtapsprogram.org, c=BD
Date: 2022.08.09 23:27:02 +06'00'
