# Supplementary material for: Landscape Analysis of Existing Policies, Laws and Regulations on Antimicrobial Stewardship in 10 Low- and Middle-Income Countries
Source: Antibiotics (Basel). 2026 Aug 10;15(8):770. doi: 10.3390/antibiotics15080770 (PMC13510074; doi:10.3390/antibiotics15080770)
Supplement: Supplementary file 1 [file antibiotics-15-00770-s001.zip › S3 Country Reports/S3B_Burkina Faso (Human & Animal Health).pdf]

USAID MEDICINES, TECHNOLOGIES, AND PHARMACEUTICAL SERVICES (MTaPS) PROGRAM

Improved Access. Improved Services. Better Health Outcomes.

RAPID ASSESSMENT OF ANTIMICROBIAL STEWARDSHIP POLICIES AND REGULATORY FRAMEWORK IN BURKINA FASO

Burkina Faso

March 2020

USAID FROM THE AMERICAN PEOPLE

This document is made possible by the generous support of the American people through the US Agency for International Development (USAID) contract no. 7200AA18C00074. The contents are the responsibility of Management Sciences for Health and do not necessarily reflect the views of USAID or the United States Government.

About the USAID MTaPS Program

The USAID Medicines, Technologies, and Pharmaceutical Services (MTaPS) Program enables low- and middle-income countries to strengthen their pharmaceutical systems, which is pivotal to higher-performing health systems. MTaPS focuses on improving access to essential medical products and related services and on the appropriate use of medicines to ensure better health outcomes for all populations. The program brings expertise honed over decades of seminal pharmaceutical systems experience across more than 40 countries. The MTaPS approach builds sustainable gains in countries by including all actors in health care—government, civil society, the private sector, and academia. The program is implemented by a consortium of global and local partners and led by Management Sciences for Health (MSH), a global health nonprofit.

The MTaPS Consortium

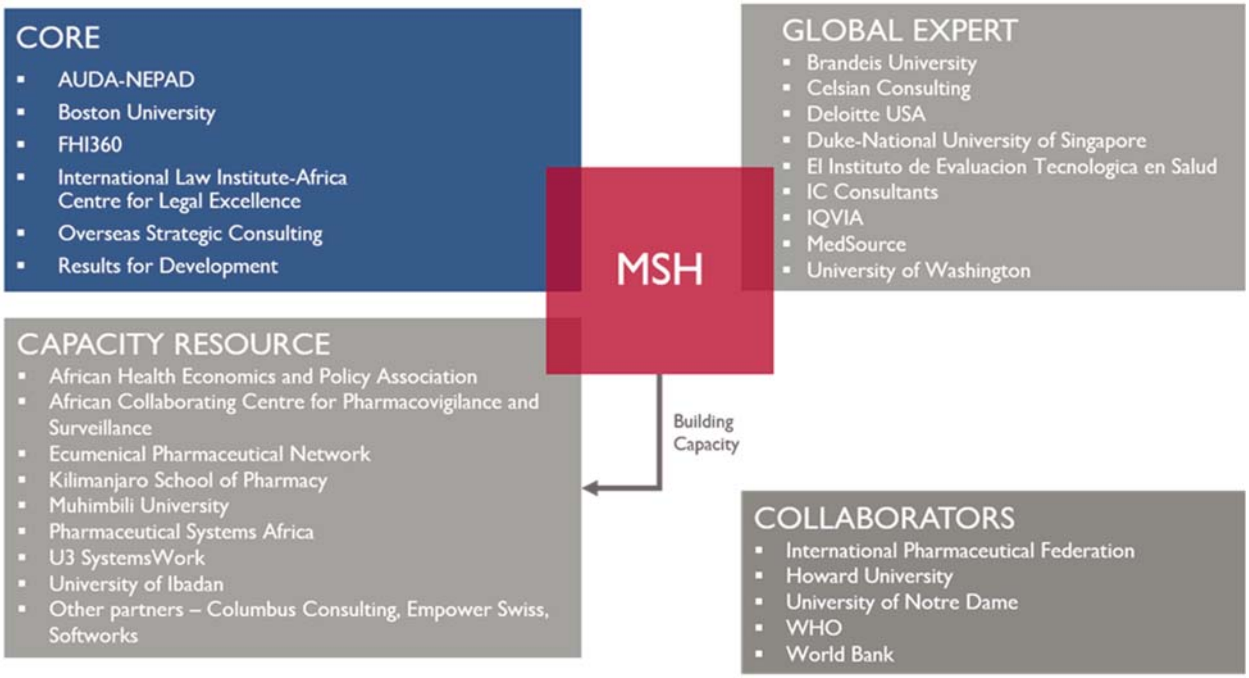

This report/document is submitted by:

Henri Kabore

Country Project Director

Medicines, Technologies & Pharmaceutical Services (MTaPS)
Program Delivery Group

Management Sciences for Health
Burkina Faso-Ouagadougou Burkina Faso
Direct: (+226) 72 72 23 15

Email: hkabore@mtapsprogram.org

Acronyms and Abbreviations

AMN	Autorisation de Mise sur le Marche
AMR	antimicrobial resistance
AMS	antimicrobial stewardship
ANRP	Autorité nationale de réglementation pharmaceutique (National Agency for Pharmaceutical Regulation)
CAMEG	Centrale d'Achat des Médicaments Essentiels Génériques et des Consommables Médicaux (Generic Medicines and Medical Consumables)
DGPML	Direction Générale de la Pharmacie, du Médicament et du Laboratoire
DSPVL	Directorate of Veterinary Public Health and Legislation
EML	essential medicines list
FAO	United Nations Food and Agriculture Organization
GHSA	Global Health Security Agenda (Action Program for Health Security)
IHR	International Health Regulations
LNE	Laboratoire National d'Elevage (National Livestock Laboratory)
LNSP	Laboratoire National de santé publique (National Public Health Laboratory)
LRE	Laboratoires Régionaux d'Elevage (Regional Livestock Laboratories)
MTaPS	Medicines, Technologies, and Pharmaceutical Services
OIE	Office International des Epizooties (World Organization for Animal Health)
VICH	International Cooperation on Harmonisation of Technical Requirements for Registration of Veterinary Medicinal Products
WAEMU	West African Economic and Monetary Union
WHO	World Health Organization
USAID	US Agency for International Development

CONTENTS

Acronyms and Abbreviations i
Project Summary iii
EXECUTIVE SUMMARY 1
BACKGROUND 1
METHODS 1
FINDINGS 1
CONCLUSION 2
INTRODUCTION 3
1. BACKGROUND 4
2. OBJECTIVES 5
3. METHODOLOGY 5
4. FINDINGS 5
4.1. SECTION 1: HUMAN HEALTH SECTOR 5
4.1.1. DATA SOURCES 5
4.1.2. THE NATIONAL AGENCY FOR PHARMACEUTICAL REGULATION 6
4.1.3. POLICIES, REGULATIONS, AND GUIDELINES ON ANTIMICROBIAL STEWARDSHIP. 8
4.1.4. MARKETING AUTHORIZATION, LICENSING, AND INSPECTION 9
4.1.5. POST-MARKET SURVEILLANCE AND PHARMACOVIGILANCE 12
4.1.6. SUPPLY MANAGEMENT POLICIES AND GUIDELINES FOR ANTIMICROBIALS 14
4.1.7. PRESCRIPTION, DISPENSING, AND USE OF ANTIMICROBIALS 14
4.2. SECTION II: ANIMAL HEALTH SECTOR 15
4.2.1. DATA SOURCES 15
4.2.2. VETERINARY MEDICINES POLICY AND REGULATORY FRAMEWORKS 16
4.2.3. MARKETING A VETERINARY MEDICINE IN WAEMU 18
4.2.4. MARKETING AUTHORIZATION, VETERINARY PHARMACEUTICAL INSPECTION, AND LICENSING 22
4.2.5. POST-MARKETING SURVEILLANCE AND PHARMACOVIGILANCE 22
4.5.6. GOOD SUPPLY MANAGEMENT POLICY AND DIRECTIVES CONCERNING ANTIMICROBIALS 23
4.2.7. PRESCRIPTION, DISPENSING, AND UTILIZATION 27
5. GENERAL DISCUSSION 27
5.1. HUMAN HEALTH SECTOR 27
5.2. ANIMAL HEALTH SECTOR 28
6. RECOMMENDATIONS 29
7. CONCLUSION 30
8. REFERENCES 31

PROJECT SUMMARY

**Program Name:**		USAID Medicines, Technologies, and Pharmaceutical Services (MTaPS) Program
**Activity Start Date And End Date:**		September 20, 2018–September 19, 2023
**Name of Prime Implementing Partner:**		Management Sciences for Health
**Contract Number:**		7200AA18C00074
**MTaPS Partners**	**Core Partners**	Boston University, FHI 360, Overseas Strategic Consulting, Results for Development, International Law Institute-Africa Centre for Legal Excellence, NEPAD
	**Global Expert Partners**	Brandeis University, Deloitte USA, Duke-National University of Singapore, El Instituto de Evaluacion Technologica en Salud, IC Consultants, Imperial Health Sciences, MedSource, QuintilesIMS, University of Washington
	**Capacity Resource Partners**	African Health Economics and Policy Association, Ecumenical Pharmaceutical Network, U3 SystemsWork, University of Ibadan, University of Ghana's World Health Organizations (WHO) Pharmacovigilance Collaborating Center, Kilimanjaro School of Pharmacy, Muhimbili University, Pharmaceutical Systems Africa
	**Collaborators**	International Pharmaceutical Federation, Howard University, University of Notre Dame, WHO, World Bank

EXECUTIVE SUMMARY

BACKGROUND

Antimicrobial resistance (AMR) is one of the most serious global public health problems. The emergence of AMR is a natural phenomenon resulting from the use of antimicrobial medicines over time, but it is accelerated by inappropriate use. In many developing countries, antimicrobials are purchased directly from the point of sale of medicines without a prescription or the advice of a qualified health professional. Antimicrobials are often used irrationally. It was necessary to assess the policy and regulatory environment in the context of management of antimicrobials to determine the gaps and recommend actions for improved control in Burkina Faso.

METHODS

As part of the support to the US Government's Global Health Security Agenda (GHSA) in Burkina Faso, the US Agency for International Development (USAID) Medicines, Technologies, and Pharmaceutical Services (MTaPS) project's country team conducted a literature review of the policy and regulatory environment for the rational management of antimicrobial use for both the human and animal health sectors. The World Health Organization (WHO) Benchmarks for International Health Regulations (IHR) Capacities and Joint External Evaluation was used as a reference. The review used the tool to support a rapid situational analysis of antimicrobial stewardship (AMS) policies to gather information from key stakeholders. The results were cross checked with public- and private-sector actors and analyzed. Finally, the document was validated through a multisectoral coordination workshop, ascertaining the findings highlighted and discussed.

FINDINGS

The evaluation showed the involvement of Ministries of Health and of Animal Resources and Fisheries in the management of AMR and AMS. It also highlighted the approaches used by the country to regulate medicines in general by using national pharmaceutical legislation for the human health sector. In the animal health sector, West African Economic and Monetary Union (WAEMU) regulations are primarily used to deal with veterinary medicines. The regulations established by WAEMU cover the region for both human and animal medicines and are filling gaps in member state regulations. However, the need for AMR regulation remains the same for both sectors, as the consequences affect both. The assessment reviewed policies, legislation, regulations, and guidelines governing the management and regulation of medicines in both the human and animal health sectors. Gaps and omissions, including the availability of national medicines policies and laws, were identified. Recommendations to strengthen AMS policies and regulations on market authorization, regulatory inspections, licensing of premises, post-market surveillance, pharmacovigilance, and prescribing and use of antimicrobials for promoting appropriate use were suggested. The existence of a system of registration, surveillance, supply, and dispensing of medicines in the human health sector was highlighted as well as the conduct of regulatory inspections for the establishment of pharmacies. Although the country is using regional regulations to manage the animal health sector, implementation and enforcement of these instruments are very weak.

Strengthening veterinary services, including capacity building (human resources and material), is necessary to address the weaknesses in this sector and will enable it to work with other Ministries to overcome the increasing threat of AMR in Burkina Faso.

CONCLUSION

The assessment revealed the absence of a specific national regulation for effective management of AMR. The lack of policies and guidelines for importation, monitoring of consumption, and prudent use of antimicrobials in both the human and animal health sectors limits better management of AMR. Despite developing an AMR strategic plan for 2017–2020 under the One Health approach, robust policies and regulations are still needed to strengthen the AMS regulatory framework for both sectors at the member state and regional levels to control AMR and improve AMS in Burkina Faso.

INTRODUCTION

Along with the massive use of antimicrobials in human and animal health in recent decades, the world is facing an acceleration in the emergence of AMR. However, the discovery of new treatments is not enough to fight bacteria—the organisms responsible for disease, sometimes serious, in humans and animals. It is therefore vital to ensure adequate access to effective antimicrobials to treat animal diseases, protect human health, and ensure food security. The globalization of the food trade and conventional and medical tourism allow existing or increasingly resistant bacteria to easily colonize the entire planet, regardless of the preventive measures applied. Locally, a country's risky behaviors are therefore likely to jeopardize the efficacy and availability of antibiotics worldwide.

The phenomenon of bacterial resistance became a concern in the early 1990s due to massive use of antibiotics, leading to the recommendation of the World Health Organization (WHO) that farmers no longer use antibiotics as growth factors and use them cautiously for therapeutic purposes. WHO estimates that more than half of all medicines are prescribed, distributed, or sold inappropriately.[1,2] Common types of irrational medicines use include using several medicines for one patient; inappropriate use of antimicrobials, often in inappropriate dosages, for the treatment of nonmicrobial infections; and inappropriate and frequent self-medication.

In line with the United Nations Food and Agriculture Organization (FAO), the World Organization for Animal Health (OIE) has been actively working for more than 10 years in the area of veterinary products such as antimicrobials, notably through international conferences. It has developed a global strategy on AMR in this area that is closely linked to its network of international experts and its 180 member countries and international partners.[3] The main objective of this initiative is to control the production, registration, importation, and distribution of antimicrobials and their use in animals through adequate regulation, qualified professionals, and a structured veterinary profession. It also ensures that support is provided to countries in need through international solidarity programs.

Since 2010, the OIE has also been engaged with WHO and the FAO in a tripartite alliance establishing the responsibilities of these three organizations in the fight against diseases with high health and economic impact, including zoonoses, through the implementation of the One Health concept. The fight against AMR is one of the three selected priorities. Tripartite actions consist of coordinating short-, medium-, and long-term strategies to combat AMR on a global scale and supporting their implementation on a national and regional scales. The objectives are:

- Ensuring that antimicrobial medicines remain effective
- Promoting the prudent and responsible use of these medicines
- Enabling everyone to access quality medicines

1. BACKGROUND

AMR is one of the most serious global public health problems. The emergence of AMR is a natural phenomenon resulting from use of these medicines over time. However, it is accelerated by inappropriate use.[4] As consumption levels increase, so do resistance levels. According to WHO, only half of all antibiotics are consumed by humans; the rest are mainly added to food for animals (pigs and poultry in particular) for mass treatment of infectious diseases or to promote growth.[5] In many developing countries, antimicrobials are purchased directly from the point of sale of medicines without a prescription or the advice of a qualified health professional. Antimicrobials are often used irrationally.[6] Causes of irrational use include insufficient knowledge, skills, or independent information; the absence of restrictions on the prescription of medicines; inappropriate promotion of medicines; and profit motivation for their sale. Regulation of the activities of all actors involved in the supply chain and use of medicines is essential to ensure rational use.[7,8]

In Burkina Faso, the regulatory framework for AMS for human health is coordinated by the National Agency for Pharmaceutical Regulation (Agence Nationale de Régulation Pharmaceutique [ANRP]). This agency was created on October 5, 2018, by Decree No. 2018-0861/PRES/PM/MINEFID/MS.[9] The ANRP is a public health institution with legal powers and administrative and financial autonomy. It coordinates the exercise of all regulatory functions in the pharmaceutical sector and can propose regulations governing the pharmaceutical sector, control and make decisions on health policy issue and injunctions, and impose sanctions to ensure the regulation of the sector. It has the power to issue legal and administrative acts in the form of notifications, decisions, opinions, and recommendations.

In the animal health sector, the regulation of the veterinary pharmaceutical sector is governed by provisions in Decree No. 2018-0729/PRES/PM/MRAH/MS/MATD/MSECU/MCIA. It describes provisions for import control, distribution, supply, marketing, monitoring of conditions for the opening and operation of manufacturing facilities, commercial storage, importation, and wholesale distribution of veterinary medicines. The provisions of this decree apply to veterinary medicines intended to be tested or marketed, presented in the form of pharmaceutical specialties, prefabricated veterinary medicines, or pre-medicine mixtures. There are also specific provisions for medicinal foods. The list of disinfection products used in livestock or prescribed in the fight against diseases deemed to cause transmission between organisms is set by decree of the Minister for Animal Resources and Fisheries. He determines by decree the maximum designs and conditions of use of complemented and supplemented foods.

For regulations to be effective, they must be enforced, and the regulatory body must be adequately funded and supported by the government. MTaPS supports improving health services in 11 countries around the world, including Burkina Faso, in the fight against AMR by implementing the GHSA.

As part of the containment of AMR in the context of inappropriate use of antimicrobials in Burkina Faso, it was necessary to assess the policy and regulatory environment for the regulation and management of antimicrobials to determine the gaps for better planning of control of antimicrobials and to develop a national strategy and national action plan for AMS as a means of adequately addressing the growing threat of AMR. This activity is in line with the WHO Benchmarks for IHR recommended actions to meet capacity level 2 by assessing current relevant legislation, laws, regulations, policies, and administrative requirements for IHR implementation and identify gaps.

2. OBJECTIVES

The general objective was to conduct a rapid situational analysis of AMS policies and regulations to uncover the gaps and challenges that perpetuate poor adherence to appropriate antimicrobial use in both the human and animal health sectors in Burkina Faso.

The specific objectives were to:

- Review policies, legislation, regulations, and guidelines governing the management and regulation of medicines in both the human and animal health sectors to determine the availability of national medicines policies and laws and identify gaps and omissions
- Suggest recommendations to strengthening AMS policies and regulations on market authorization, regulatory inspections, licensing of premises, post-market surveillance, pharmacovigilance, and prescribing and use of antimicrobials for promoting appropriate use
- Use the findings of the assessment to develop a draft national strategy and action plan for AMS

3. METHODOLOGY

The assessment of the AMS policies and regulatory environment for antimicrobial management and regulation was conducted by the MTaPS/Burkina Faso team in collaboration with the Ministry of Health and Ministry of Animal Resources and Fisheries. The assessment was conducted in two phases. During the first phase, a literature review of key documents was carried out to better understand of the structure and functioning of the pharmaceutical policies and regulatory system considering the greater human and animal health system. The second phase consisted of conducting interviews with key stakeholders for AMS policies and pharmaceutical regulation to clarify the evidence gathered on available and relevant policies, legislation, regulations, and guidelines.

An interview guide was developed in-house to help gather information on the current policies and regulatory environment for the management and regulation of antimicrobials. The interview guide comprised regulatory aspects of product registration, licensing of premises, regulatory inspections, post-marketing surveillance, pharmacovigilance systems, and policies and guidelines for the effective management of antimicrobials. Thematic content analysis was applied to the observations and findings. The aim was to describe the current policies and regulations for the effective management of antimicrobials and identify gaps and weaknesses. The WHO Benchmarks for IHR Capacities and Joint External Evaluation tool were used as references for this assessment.

4. FINDINGS

4.1. SECTION 1: HUMAN HEALTH SECTOR

4.1.1. DATA SOURCES

The following documents provided information on the laws, decisions, decrees, orders, directives, management policies, and regulatory frameworks for antimicrobials in Burkina Faso within the context of a rapid assessment.

- Annexes au règlement sur les procédures d'homologation des produits pharmaceutiques à usage humain dans les États membres de l'UEMOA
- Annuaire statistique 2018
- Décision No. 09/2010/CM/UEMOA adoptant le guide de bonnes pratiques pour la distribution et l'importation de produits pharmaceutiques à usage humain dans les États membres de l'UEMOA
- Décret No. 2017- 458 MS/CAB Le port de la publicité condition sur la drogue de produits pharmaceutiques et d'autres au Burkina Faso
- Loi No. 23/94/ADP portant Code de la Santé publique
- Loi No. 080-2015/CNT portant réglementation de la publicité au Burkina Faso
- Plan stratégique pharmaceutique du Burkina Faso 2012–2016
- Plan stratégique pharmaceutique du Burkina Faso 2019–2023
- Plan d'action national multisectoriel de lutte contre la résistance aux antimicrobiens, Octobre 2017–Septembre 2020
- Politique pharmaceutique du Burkina Faso 2012–2021
- Politique du secteur de la santé du Burkina Faso 2017–2026
- Profil pharmaceutique du Burkina Faso Edition 2011
- Note circulaire aux pharmacies privées: rappel réglementaire pour la distribution d'antibiotiques et de psychotropes
- Règlement No. 06/2010/CM/UEMOA sur les procédures d'enregistrement des produits pharmaceutiques à usage humain dans les États membres de l'UEMOA
- Rapport synthèse de la surveillance de la résistance aux antimicrobiens au laboratoire, Burkina Faso, 2018
- Rapport d'évaluation de la diffusion et de l'utilisation de la liste nationale des médicaments essentiels au Burkina Faso en 2018
- Recueil de textes réglementaires du secteur de la pharmacie, des médicaments et des laboratoires du Burkina Faso Volume I, Edition 2011
- Recueil de textes réglementaires du secteur de la pharmacie, du médicament et des laboratoires du Burkina Faso Volume II, Edition 2014

4.1.2. THE NATIONAL AGENCY FOR PHARMACEUTICAL REGULATION

To meet the new institutional challenges and enable the achievement of new national ambitions in terms of availability and access to quality health products for all, it was appropriate to move the pharmaceutical regulatory system toward an autonomous structure capable of guaranteeing the pharmaceutical quality and safety of health products.

The pharmaceutical regulatory system involves several related functions and activities. These include licensing of professionals, approval of facilities and practices, product evaluation and registration, inspection of establishments and good pharmaceutical practice, quality control of health products, provision of pharmaceutical and medical information, clinical trials, monitoring of adverse reactions, and control of medical promotion and advertising.

The various evaluations of Burkina Faso's national pharmaceutical regulatory system by WHO in 2006 and WAEMU in 2013 have revealed a fragmentation of regulatory responsibilities among the Directorate

General of Pharmacy, Medicines and Laboratories (DGPML), which includes almost all of the functions; the Technical Inspection of Health Services; the National Public Health Laboratory (LNSP); and the Directorate of Quality Control of Medicines and Other Health Products.

The process of developing the project to create the Pharmaceutical Regulatory Agency of Burkina Faso was carried out in several stages that were marked by studies and evaluations of the regulatory system by international (WHO 2006, UEMOA 2013, Consultants/Expertise France 2017) and national (DGPML 2014, National Reflection Committee 2016) structures.

Based on the results of the study conducted by WAEMU in 2013, Burkina Faso, along with Côte d'Ivoire, Mali, and Senegal, was classified at level 3 on a scale of 1 to 4 as one of the member states' pharmaceutical directorates with the capacity to evolve toward a national pharmaceutical regulatory agency implementing all regulatory functions. As a result, WAEMU, in line with the African Union's vision on the African Medicines Agency, recommended that countries adopt legislation to give the Directorates of Pharmacy management autonomy and a status of national regulatory agency. The ANRP brings together and coordinates all regulatory missions of the pharmaceutical sector.

The ANRP can propose regulations governing the pharmaceutical sector, control and make decisions on health policy issue and injunctions and impose sanctions to ensure the regulation of the sector. It has the power to issue legal and administrative acts in the form of notifications, decisions, opinions, and recommendations.

The ANRP is under the technical supervision of the Minister of Health and the financial supervision of the Minister of Finance. It is charged with:

- Granting authorizations and licenses for the creation, opening, and operation of pharmaceutical establishments, medical biology analysis laboratories, medical promotion agencies, and traditional medicines establishments
- Granting authorizations for clinical trials, traditional medicines, and medical sales representatives
- Granting marketing authorizations for health products
- Inspecting pharmaceutical establishments, including medical biology analysis laboratories, medical promotion agencies, traditional pharmacopoeia establishments, quality control laboratories, and clinical trial sites
- Ensuring quality auditing of pharmaceutical establishments, including biological testing laboratories, health promotion agencies, traditional medicines establishments, and clinical trial sites
- Ensuring quality control of health products
- Ensuring vigilance of health products
- Ensuring the control of advertising, promotion, and information on health products and pharmaceutical establishments
- Ensuring that health professionals and the general public are informed about health products
- Developing standards and regulations relating to health products and to the personnel and premises involved in the manufacture, import and export, storage, distribution, dispensing, promotion, use, and management of waste from these products
- Contributing to the development and implementation of pharmaceutical policy

- Contributing to monitoring of the availability of health products
- Issuing a technical opinion for the issuance of special import and export authorizations for health products
- Contributing to the regulation of the price of health products
- Participating in international cooperation and harmonization of pharmaceutical regulatory practices
- Ensuring the application of regulations on narcotic drugs, psychotropic substances, and their precursors
- Fighting against counterfeit health products and the illicit health product market

4.1.3. POLICIES, REGULATIONS, AND GUIDELINES ON ANTIMICROBIAL STEWARDSHIP

Appropriate medicines use is an important component of Burkina Faso's pharmaceutical policy, and its promotion is guided by the national essential medicines list (EML) and the national therapeutic formulary. The EML, which is regularly revised (most recently in 2016), lists essential medicines adapted to the health context of Burkina Faso for each level of care. The national therapeutic formulary, last revised in 2018, describes how to use all medicines on the EML.

In Burkina Faso, several ministerial departments are involved in the management of AMR and AMS, including health, animal resources and fisheries, agriculture, and the environment. At the Ministry of Health, apart from the 2013 order on the organization of the DGPML, which mentions the role of the national center for the vigilance of health products in the surveillance of AMR, the use of antimicrobials, AMR prevention, and surveillance are not provided for in any legislation. However, most antimicrobials belong to list I in the classification of poisonous substances and are subject to standard prescription and dispensing rules. Some national directives provide the basis for exemptions from these rules, such as dispensing antimalarial drugs without prescription or community distribution of amoxicillin.

Moreover, there is weak application of the rules on prescribing and dispensing list I medicines, including antimicrobials. In addition to legal texts, several normative documents are regularly developed and disseminated that contribute to the promotion of the appropriate use of antimicrobials. These include the EML, the national therapeutic formulary, and the CEDIM letter. All of these guidance documents are mainly for health professionals.

The Ministry of Health, through the national pharmaceutical regulatory agency, regularly reviews the EML and other health products in accordance with the recommendations of WHO to take better account of the needs of the population at different levels of health service delivery. This year the EML is being revised with a focus on the WHO AWaRe classification of antibiotics. There is a diagnosis and treatment guide for prescribers on therapeutic choice, the most recent version of which dates to 2008. A need for regular updating of the guide is required to quickly incorporate changes in treatment policies and new medicines in the national standard treatment guidelines.

Over the past decade, generic essential medicines prescriptions that comply with the EML have become almost systematic in first-level health facilities.[10] At the hospital level, the absence of a Drug and Therapeutics Committee and normative tools for appropriate use is detrimental to rational practices for prescribing and dispensing medicines within the framework of an organized hospital pharmacy.

The regulatory framework for AMS is coordinated by the ANRP.

4.1.4. MARKETING AUTHORIZATION, LICENSING, AND INSPECTION

A) MARKETING AUTHORIZATION

On July 4, 2005, in Dakar, the Council of Ministers of the WAEMU Commission adopted a regulation on the establishment of a structure responsible for the harmonization of the pharmaceutical regulation with the mission of implementing strict rules to ensure the safety, efficacy, innocuousness, and quality of pharmaceutical products.

A function for approving medicines for human use is a top priority. This is an essential function of pharmaceutical regulation that ensures the safe use of medicinal products authorized to be placed on the market. It is carried out through a meticulous dossier evaluation process that brings together all administrative and technical data on the medicine. The marketing authorization allows the regulatory authority to control the importation and distribution of drugs that have been assessed and authorized.

The annexes to the regulations on the procedures for the approval of pharmaceutical products for human use in WAEMU member states are part of the regulatory text and describe the referenced elements related to the content of the technical files and the procedure for the granting of marketing authorization.

These annexes are as follows:

- Annex I: Guidelines for the submission of application dossiers of homologation. This annex lists the formatting specifications for the submission of marketing authorization application dossiers.
- Annex II: The procedure for granting marketing authorizations, beginning with receipt of the dossier.
- Annex III: The standard format for the submission of a marketing authorization for a medicinal product, including all information that must be included in an authorization for placing a product on the market.

According to the Public Health Code, any health product manufactured, imported, held, distributed, or dispensed in Burkina Faso must have a marketing authorization issued by the Ministry of Health through the National Agency for Pharmaceutical Regulation.

The marketing authorization application files are evaluated by experts. An opinion is then given by the Technical Commission for the Registration of Medicines and other Pharmaceutical Products. When this opinion is favorable, the Ministry of Health authorizes the marketing of the product. By way of derogation, health products that do not have a marketing authorization in Burkina Faso but are deemed useful for public health or indispensable to the specialized health services for the care of patients may be subject to a special import authorization for a limited period of time. Registration procedures for drugs and other health products are governed by the WAEMU texts mentioned above.

The regulatory texts for marketing authorization are:

- Order 2013-243/MS/CAB/ of 13/03/2013 on the creation, attributions, composition, and functioning of the commission for the registration of health products for use in human medicine
- Order 2014-010/MS/CAB of 10/01/2014 appointing members of the expert committees for the evaluation of applications for registration of health products for human use
- Order 2013-245/MS/CAB of 13/03/2013 on the national nomenclature of pharmaceutical specialties and generic medicines authorized in Burkina Faso
- Order 2013-537/MS/CAB of 31/05/2013 on the regulation of in vitro diagnostic medical devices and medical consumables and annex

B) LICENSING OF PHARMACEUTICAL PREMISES

The creation and opening of a pharmaceutical and related establishment and the practice of the pharmacy profession are subject to obtaining licenses issued by the Ministry of Health through the ANRP. There are three types of licenses in Burkina Faso:

- Licenses for the creation and opening of pharmaceutical establishments (private pharmaceutical offices, hospital pharmacies, pharmaceutical wholesalers, and private pharmaceutical warehouses)

Regulatory texts:

- ✓ Decree No. 2014-047/PRES/PM/MS of 07/02/2014 on the code of ethics for pharmacists in Burkina Faso
- ✓ Order No. 2014-390/MS/CAB of 27/03/2014 on the internal regulations of the National Order of Pharmacists of Burkina Faso
- ✓ Order No. 2013-552/MS/CAB of 21/06/2013 on the modalities for the practice of traditional medicine in Burkina Faso
- ✓ Order No. 2010-360/MS/CAB authorizing the opening and operation of a private pharmaceutical dispensary
- ✓ Order No. 2013-549/MS/CAB of 21/06/2013 on the creation, attribution, composition, and functioning of the technical commission for the examination of applications for authorization to practice traditional medicine
- ✓ Order No. 2013-1341/MS/CAB of 23/12/2013 on the creation, attributions, composition and functioning of a commission for the examination of requests for authorization to create, open, extend, transform, transfer, and close health establishments

- Licenses for the opening and operation of promotion agencies—a laboratory for medical biology analysis

Regulatory text:

- ✓ Order No. 2017-451/MS/CAB, making it a condition for the opening and operation of a medical promotion agency or a representative office of a pharmaceutical preparation establishment

- Licenses to practice the pharmacy profession (sales representative and traditional healer)

Regulatory texts:

- ✓ Order No. 2013-549/MS/CAB of 21/06/2013 on the creation, attribution, composition, and functioning of the technical commission for the examination of applications for authorization to practice traditional medicine
- ✓ Decree No. 2012-1035/PRES/PM/PM/MS/MICA/MATDS/MEF of 28/12/2012 defining the definitions and conditions for opening and operating traditional medicine and pharmacopoeia establishments in Burkina Faso
- ✓ Order No. 2013-550/MS/CAB of 21/06/2013 on the modalities of opening and operating a traditional medicine and pharmacopoeia establishment in Burkina Faso
- ✓ Order No. 2013-551/MS/CAB of 21/06/2013 on the institution, organization, and functioning of a system of reference and recourse between traditional medicine and conventional medicine in Burkina Faso

These pharmaceutical licenses allow applicants to practice their profession in compliance with the regulatory texts. The National Agency for Pharmaceutical Regulation acts as the technical secretariat of the Ministry of Health for the examination of applications and issues an opinion on the granting of licenses.

In 2017, the private pharmaceutical distribution network consisted of 11 wholesale pharmaceutical establishments, 246 pharmacies, and more than 600 pharmaceutical depots. The geographical distribution of private pharmacies is inequitable, with a very high concentration in the cities of Ouagadougou and Bobo Dioulasso (81.56%). This limits access to quality medicines because several rural areas of the country lack pharmaceutical infrastructure and qualified personnel to dispense medicines. Licenses to practice are granted to medical sales representatives and traditional health practitioners.

Weaknesses include inadequacies in the granting of the medical sales representative professional card, the absence of a monitoring framework with services outside the agency, the absence of a regulatory framework for para-pharmacies, inadequacies in the control of advertising on social networks (internet), and the absence of an expert committee to evaluate applications for advertising and promotion.

C) REGULATORY INSPECTIONS

Before the creation of the ANRP, the main pharmaceutical regulatory functions were fragmented, and the lack of collaborative mechanisms among these structures led to shortcomings in the enforcement of regulatory texts. The ANRP's responsibility for coordinating key regulatory functions was not formally designated. It appears that the DGPML, which played the role of ANRP, did not perform all regulatory functions normally assigned to an ANRP.

Pharmaceutical inspection is carried out by the National Agency for Pharmaceutical Regulation through the Directorate of Pharmaceutical Inspection. Both are involved in inspections of pharmaceutical establishments (e.g., manufacturers, importers, wholesalers) and biomedical laboratories; the performance of routine and targeted investigations; the support for the destruction of pharmaceutical waste; and the review and use of standardized inspection tools.

The Directorate of Pharmaceutical Inspection is responsible for:

- Ensuring the application of regulations and standards in the fields of pharmacy, medical biology, traditional medicine, and pharmacopoeia through pharmaceutical inspection of pharmaceutical establishments, medical biology analysis laboratories, traditional medicine and pharmacopoeia establishments and clinical trial sites
- Monitoring the advertising and promotion of medicines and other pharmaceutical products
- Investigating and detecting fraud and illegal practices in pharmacy, medical laboratories, traditional medicine, and pharmacopoeia
- Monitoring the import and use of narcotic drugs, psychotropic substances, and precursors and making statistics available in accordance with the regulations in force
- Contributing to the design, organization, and monitoring of the implementation of programs to combat counterfeit medicines and other medical products and the illicit sale of those products
- Contributing to the control of the prices of medicines and other health products
- Monitoring the destruction of pharmaceutical waste
- Ensuring compliance with the texts in force relating to the quality assurance of pharmaceutical establishments and clinical trials

On February 9, 2019, the ARNP, in collaboration with the police, customs, and justice, organized a crackdown on the trafficking of falsified medicines. This operation, called HEERA 2, resulted in the seizure of approximately 28 tons of falsified medicines in the city of Ouagadougou.

4.1.5. POST-MARKET SURVEILLANCE AND PHARMACOVIGILANCE

A) POST-MARKET SURVEILLANCE

Import control

Burkina Faso imports 99% its medicines and other health products from foreign sources.[10] Import control function is therefore essential for monitoring the inflow of medicines in the country. Control at border entry points is carried out by customs services in collaboration with the ANRP and the LNSP. All invoices for health products are referred to the ANRP to verify their registration in the national product register or the existence of a special import authorization. The import permit issued by the ANRP allows an authorized importer to be exempt from customs duties.

However, there is inadequate control of imports due to access to the national medicine register to verify status of registration, the register not being disseminated to the public, no pharmacists or technical persons deployed at the border posts, and medicines imported without authorization from the ANRP. It is therefore difficult to monitor the volume of antimicrobial imports into Burkina Faso.

Exportation of pharmaceuticals

There is no legal provision of regulation of pharmaceutical exports in the country. For this reason, no data are available for the export of pharmaceuticals.

Post-marketing quality control

Laboratory testing of medicines is coordinated by the ARNP, which carries out sampling at different levels of the pharmaceutical distribution chain and has its compliance checked at the LNSP or in certified

international laboratories, depending on the sources of funding. Some donors require WHO prequalification or accreditation according to ISO: 17025 for the performance of quality control on the products for which they provide funding for procurement.

Market surveillance and control procedures set up by the ANRP consist of organizing post-marketing quality controls of medicines and other health products in all pharmaceutical establishments, hospitals, and health facilities in the country every six months. The purpose of this control is to verify the conformity or nonconformity of these products to the standard specifications.

The low financial resources devoted to quality control and laboratory testing, which has high operating costs, leads to very low quantities of samples picked and analyzed. Finally, the technical skills and capacity for detecting substandard and falsified health products are insufficient at all levels of the medicines supply chain in relation to the scale of the phenomenon observed at the global level.

To fight AMR, operations are carried out by the ANRP to detect illegal sale of medicines in liaison with enforcement agencies. During such operations in 2011, antimicrobials were seized during an operation referred to as "Operation Cobra" in Ouagadougou, where two tons of medicines were seized, including amoxicillin, ciprofloxacin, norfloxacin, azithromycin, cefalexin, chlortetracycline, and cotrimoxazole (25% of the amount seized). During the 2013 "Epic Operation" in Ouagadougou, amoxicillin (8,600 capsules); ciprofloxacin (10,250 tablets); and metronidazole (4,460 tablets) were seized.

B) PHARMACOVIGILANCE

In Burkina Faso, the ANRP is responsible for pharmacovigilance, which is integrated into a comprehensive national system for monitoring adverse reactions to health products. The purpose of this national health product vigilance system is to monitor adverse reactions and events related to the use of health products for human use, with the aim of guaranteeing their safe use. Its main goals are to:

- Detect undesirable effects
- Identify adverse reactions and collect information concerning them according to a defined circuit
- Record, evaluate, and use this information for prevention purposes
- Conduct studies regarding the safe use of health products for human use

The ANRP implements and coordinates the national system for monitoring adverse reactions to health products. This surveillance is carried out through the VIGIFLOW database. This system of vigilance makes it possible to manage problems related to medicine safety and quality, including the management of withdrawals of defective batches. Adverse reaction notification sheets are available, and a notification procedure has been established. In 2019, 5 of the 12 alerts received by the Pharmacovigilance Commission were related to antimicrobials: sulfadoxine-pyrimethamine and amodiaquine, praziquantel, dolutegravir, artemether + lumefantrine, and fluoroquinolone used in self-medication. However, deficiencies in the reporting information limit the detection of falsified medicines. In addition, the system encounters other difficulties related to insufficient reporting by health workers, such as a lack of awareness among the population on the reporting of incidents related to health products.

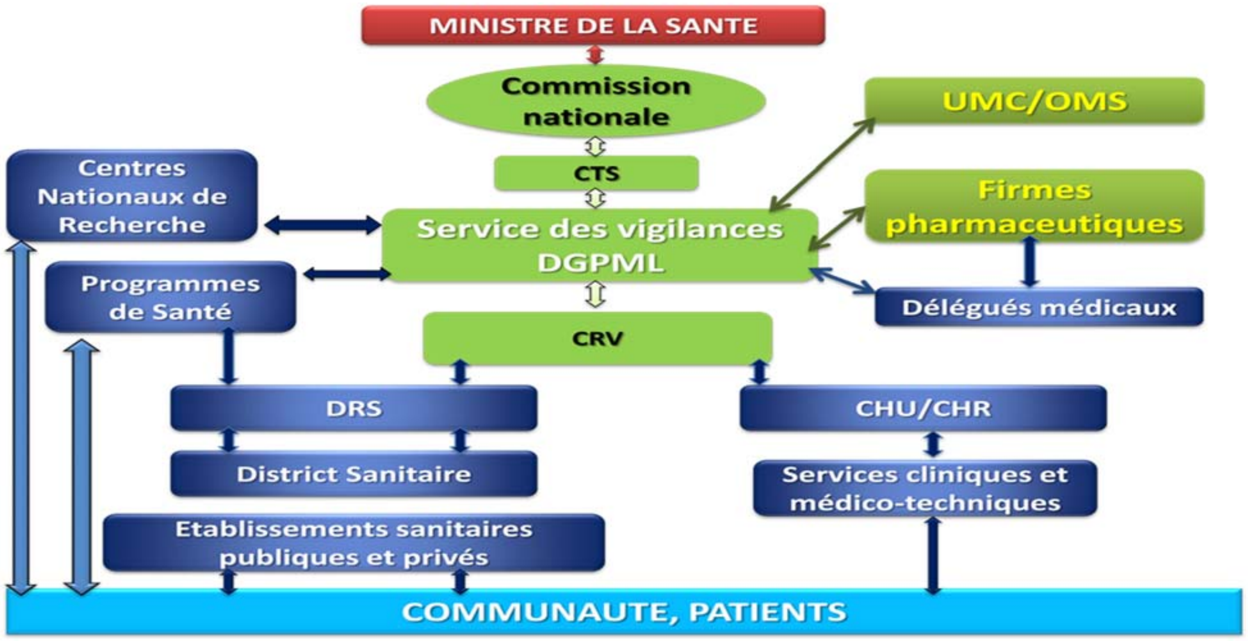

CHU/R: Centre hospitalier universitaire/régional. CRV: Comité régional de vigilance; DRS: Direction régionale de la santé; CTS: Comité technique spécialisé; UMC: Uppsala Monitoring Center

Figure 1. Organization of the National Heath Product Vigilance System

4.1.6. SUPPLY MANAGEMENT POLICIES AND GUIDELINES FOR ANTIMICROBIALS

Burkina Faso's supply of health products is 99% dependent on imports because local production of health products remains very low. In terms of supply and distribution, the system involves both public and private actors. The public sector is organized around the Central Purchasing Agency for Essential Generic Medicines and Medical Consumables (CAMEG) with support from several technical and financial partners. CAMEG ensures the supply of essential generic medicines and medical consumables to 70 district distribution depots via its 10 agencies in 8 regions. It also supplies private dispensaries and pharmacies of private clinics. The district distribution depots in turn supply the 1,992 Essential Generic Medicines depots of public and confessional health care facilities. Public hospitals (Centres Hospitaliers Universitaires and Centres Hospitaliers Régionaux) obtain their supplies primarily from CAMEG agencies but also from private structures according to the laws in force.

The private supply circuit is ensured by private wholesale distributors who supply private hospital pharmacies and private dispensaries, which in turn supply private drug depots. Several laws and regulations govern the supply of health products and the implementation of regulatory functions. Shortcomings are mainly found in local production, hospital pharmacy, and the fight against illicit drugs.

4.1.7. PRESCRIPTION, DISPENSING, AND USE OF ANTIMICROBIALS

The rational use of medicines and other health products contributes to better therapeutic management of populations, even in the context of limited resources. To achieve this objective, the government is committed to:

- Regularly updating the tools for promoting rational use, such as standard treatment regimens

- Providing health professionals and the general public with appropriate pharmaceutical information that promotes the proper use of medicines
- Regulating private medical promotion

Patient antimicrobial use data for Burkina Faso are not available. However, the available annual import statistics provide some insight into the extent of antimicrobial use. In 2015, Burkina Faso imported antimicrobials valued at 52.797 billion FCFA (nearly 50% of the total financial volume of pharmaceutical imports). Antibacterial and antiparasitic drugs accounted for 17.984 billion FCFA and 13.894 billion FCFA of these imports, respectively.

Studies have been conducted on the use, dispensing, and prescription of antibiotics in health services. A study carried out in 1999 on 2,040 women hospitalized in the gynecology/obstetrics department in a hospital in Ouagadougou found that 1,733 had received an antibiotic prescription, for a prescription rate of 85%; 15.8% of newborns (156/1,014) also received antibiotics. All hospitalized patients received initial antibiotic treatment for suspicion that was modified once in 95.9% of cases and twice in 24.1% of patients. Finally, 2.5% of patients underwent a third modification. Out of 276 indications for curative antibiotic therapy, 45 (16.3%) underwent a bacteriological examination, most of which were carried out after the first antibiotic prescriptions.[11]

An evaluation of the rational use of medicines carried out in 2015 by the Directorate General of Pharmacy, Medicines and Laboratories showed that out of 1,011 prescriptions, 53.91% contain at least one antibiotic. It also showed high use of antibiotics in children under 5 in gastroenteritis (33/48) and upper respiratory diseases (17/20).[12]

In 2016, as part of the implementation of free health care in Burkina Faso, a study conducted in Ouagadougou showed that among children aged 0 to 5 years in outpatient clinics, 83% of prescriptions contained at least one antibiotic This percentage of antibiotic prescriptions was higher than the WHO standard (≤ 30%). The health statistics of the central region in 2016 and 2017 showed that infections were less than 30% of the bacterial units of outpatient consultations in children. This study showed that prescribers in first-level contact health centers care do not take their diagnosis into account before prescribing antibiotics in children 0 to 5 years old.[6]

4.2. SECTION II: ANIMAL HEALTH SECTOR

4.2.1. DATA SOURCES

The review of the instruments governing veterinary medicines in Burkina were collected and classified according to their types. Six types were identified: Treaty and Additional Acts, Laws, Decrees, Directives, Regulations, and Action Plans. There is no policy related to veterinary medicines in general and particularly for antimicrobials. This review provided a good opportunity to question the situation, which needs to improve for better management of AMR and AMS.

Table 1. General instruments governing the veterinary medicines in Burkina Faso

Types of instruments	Focus of the instrument
**1. Treaty and Additional Acts**	▪ Le Traité de l'Union Economique et Monétaire Ouest Africaine, notamment en ses articles 4, 6, 16, 33,42 à 45, 101 et 102<br>▪ L'Acte additionnel n° 003/2001 du 19 décembre 2001 portant adoption de la politique agricole de l'UEMOA, notamment en son article 10
**2. Law**	▪ Loi n° 048-2017/AN du 16 novembre 2017 portant code de santé animale et de santé publique vétérinaire
**3. Decrees**	▪ Décret N°/2018 0729/PRES/PM/MRAH/MS/MATD/MSECU/MCIA Portant réglementation de la pharmacie vétérinaire au Burkina Faso<br>▪ Décret N° 2017- 1335/PESS/promulguant la loi N°048-2017/AN du 16 novembre 2017 portant code de santé animale et de santé publique vétérinaire
**4. Directives**	▪ Directive n° 07/2006/CM/UEMOA relative à la pharmacie vétérinaire
**5. Regulations**	▪ Règlement N° 02/2006/CM/UEMOA établissant des procédures communautaires pour l'autorisation de mise sur le marché et la surveillance des médicaments vétérinaires et instituant un comité régional du médicament vétérinaire<br>▪ Règlement N° 03/2006/CM/UEMOA instituant des redevances dans le domaine des médicaments vétérinaires au sein de l'UEMOA<br>▪ Le Règlement n° 001/2006/CM/UEMOA du 23 mars 2006 portant création et modalités de fonctionnement d'un Comité vétérinaire au sein de l'UEMOA du 23 mars 2006<br>▪ Le Règlement n° 002/2006/CM/UEMOA du 23 mars 2006 établissant des procédures communautaires pour l'autorisation de mise sur le marché et la surveillance des médicaments vétérinaires et instituant un Comité régional du médicament vétérinaire<br>▪ Le Règlement n° 004/2006/CM/UEMOA du 23 mars 2006 instituant un réseau de laboratoires chargés du contrôle de la qualité des médicaments vétérinaires dans la zone UEMOA
**6. Action Plans**	▪ Plan d'action national multisectoriel de lutte contre la résistance aux antimicrobiens octobre 2017- septembre 2020.

4.2.2. VETERINARY MEDICINES POLICY AND REGULATORY FRAMEWORKS

The Directorate General of Veterinary Services within the Ministry of Animal Resources and Fisheries is responsible for animal health and veterinary medicines. The Directorate General of Veterinary Services has three technical Directorates:

- The Directorate of Veterinary Public Health and Legislation is responsible for monitoring the importation, use, and statistics of veterinary medicines, the veterinary profession, and legislation
- The Directorate of the National Livestock Laboratory (LNE) is responsible for diagnosing animal diseases, with two services (medical bacteriology and food microbiology) involved in monitoring AMR
- The Directorate of Animal Health covers all animal health issue, including prevention (sanitary and medical), diagnostics, and treatment

The Directorate of Veterinary Public Health and Legislation is responsible for promoting, applying, and enforcing regulations and policies related to medicines and antimicrobials within the country. Decree No 2018-0729 /PRES/PM/MRAH/MS/MATD/MSECU/MCIA regulates veterinary pharmacy in Burkina Faso.

A) INTERNATIONAL ARRANGEMENTS

The international arrangement is a global approach that covers all initiatives at the country and regional levels to align policies, action plans, and issues that need to be addressed at any level of the hierarchy.

OIE: The OIE develops codes and guidelines for the veterinary medicines industry, practitioners, pharmacists, and breeders. The OIE's code of practice for the approval of veterinary medicines stipulates that countries must take the necessary measures to ensure the quality, safety, and efficacy of products that may be on the market.[14] For the particular use of antibiotics, a guideline has been developed that assigns a major role to the authorities responsible for the delivery of Authorization de Mise sur le Marche (AMM) of antimicrobial substances in view of the impact of misuse on public health and the environment.[15]

FAO: The FAO is also developing guidelines for the regulation of veterinary medicines. The guidelines relate to approval and granting marketing authorization, control of manufacturing, importation, distribution, and quality control.

Codex Alimentarius Commission: The commission was established by an agreement between the FAO and WHO in 1962. It is a joint FAO/WHO food standards program that is contained in a document called Codex Alimentarius, which includes the international code of practice recommended for the control and use of veterinary medicines. One of the aspects addressed by the Commission is the setting of the maximum residue limits of veterinary medicines in food.[16]

International Cooperation on Harmonization of Technical Requirements for Registration of Veterinary Medicinal Products (VICH): Originally created under the auspices of the OIE, the VICH was officially established in April 1996 at the first meeting of its Director's Committee. The VICH is a trilateral program (European Union, Japan, United States) charged with harmonizing the technical requirements for the approval of veterinary drugs.[17] Burkina Faso is part of this international cooperation though the OIE, of which it is a member, and participates in its annual general assembly. During the general assembly, issues related to the harmonization of technical requirements for veterinary medicines approval are discussed and agree to. The issues may be raised by the general assembly subsequent to a request of a country, group of countries, or the general assembly itself due to a general concern expressed by the member states of the organization.

B) REGIONAL VETERINARY PHARMACEUTICAL REGULATION IN WAEMU

Regional groupings are formed to encourage member states to create a vast economic area with freedom of trade. The regulation of veterinary medicines in the WAEMU zone is no exception to this principle.

Organization of WAEMU: WAEMU was created on January 10, 1994, in Dakar, Senegal, and comprised seven member states (Benin, Burkina, Côte d'Ivoire, Mali, Niger, Senegal, and Togo). On May 2, 1997, Guinea Bissau became the eighth member state.

WAEMU's legal system: WAEMU's legal system consists of primary and derived laws. The Treaty defines the fundamental rules on which the Union bases its actions. Derivative law is made up of all of WAEMU's legislative acts (directives, regulations, decisions, recommendations, and opinions) that derive

from the principles and objectives set out in the treaty of the Union. The Conference of Heads of State issues additional acts, and the Council of Ministers issues regulations, directives, and decisions. The Council of Ministers can make recommendations and/or express opinions to the heads of states to be considered. The WAEMU Commission sets regulations for the implementation of the actions of the Council of Ministers and issues decisions. The Commission also makes recommendations and/or expresses opinions for adoption. The Court of Justice ensures respect for the interpretation and application of the Treaty of the Union.

The regulations agreed upon in the Union are binding legislation and have a general scope. They are mandatory in all elements and are directly applicable in any Member State. The guidelines set targets for all WAEMU countries but leave it up to each country to determine how to achieve them.

Decisions are directly applicable only to the recipients to whom they are addressed (e.g., member country, company). Recommendations and opinions are not enforceable and nonbinding. Additional acts, directives, regulations, and decisions are published in the Official Bulletin of the Union.

Harmonization of veterinary pharmaceutical regulation: Regulation of veterinary medicines is subject to these different types of legislative acts adopted by the Union. Subsequent to the privatization of veterinary services in 1990 in Burkina Faso, several categories of unofficial actors were actively involved in the distribution of veterinary medicines.[18] The requirements and guidelines for importation of veterinary medicines by wholesalers and veterinary firms are often unclear.[19] At the retail level, most countries within the region do not have enough geographical coverage for veterinary outlets, which encourages the emergence of an illicit market. Gaps the supply chain of medicines create a vacuum leading importer to sell directly to farmers.

A review of the legislation showed a very high heterogeneity in the legal nature, content, and degree of implementation of these legislative and regulatory texts in the veterinary pharmaceutical sector at the country level and particularly in Burkina Faso.[20] In many cases at the country level, regional regulations have not been implemented due to a lack of human and financial resources required for to implement the regulatory provisions. Regulation of veterinary medicines is essential for protecting human health, animal welfare, and food security.[22] These concerns motivated WAEMU to initiate reform of harmonization of veterinary pharmaceutical legislation within the framework of the objectives outlined in its Agricultural Policy.

4.2.3. MARKETING A VETERINARY MEDICINE IN WAEMU

A) APPLICABLE REGULATIONS AND DECISIONS

These regulations and decisions are the foundation of marketing of medicines in Burkina Faso and are not specific to antimicrobials. They should be implanted in conjunction with other regulations.

REGULATIONS	DIRECTIVES
No.01/2006/CM/WAEMU<br><br>This regulation establishes the veterinary committee of WAEMU and specifies the essential provisions relating to its composition, its area of competence, and its	07/2006/CM/WAEMUUEMOA<br><br>This directive relates to veterinary pharmacies. It details the field application of Regulation 02/2006/CM/WAEMU. Its purpose is to describe

operation. The veterinary committee, comprising directors of veterinary services of Member States, is responsible for providing technical advisory advice on the decisions of the Marketing Authorization taken by the Commission.	provisions that Member States must implement in terms of import control; traffic within the union; marketing; and monitoring of opening conditions and operations of facilities for the manufacture, commercial detention, importation, and wholesale distribution of veterinary medicines.
No. 02/2006/CM/WAEMU<br><br>This community regulation is the basis of veterinary pharmaceutical legislation in WAEMU Member States. It defines the regulatory framework, procedures, and institutional arrangements necessary for the operation of the centralized authorization system for the marketing of medicines for veterinary use, the organization of their control, and surveillance of the veterinary medicine market.	No. 009/2009/COM/WAEMU<br><br>The purpose of this directive is to set the terms of the application for the community marketing authorization to with the Commission for any medicine, whether it is new or already in circulation in the area before the regional regulation. It describes the documents required for an AMM application, the language of the application, the composition of the AMM file, and the number of copies to be provided.
No. 03/2006/CM/WAEMU<br><br>This text establishes royalties and specifies the different types of authorizations and services for which a royalty is paid, as well as the respective amount of those royalties.	No. 011/2009/COM/WAEMU<br><br>This directive lists changes to marketing authorization files.
No. 04/2006/CM/WAEMU<br><br>This text establishes a network of laboratories responsible for monitoring the quality of veterinary medicines in the WEAMU area. It defines objectives, terms of operation, and organization.	

B) IMPLEMENTATION OF REGULATIONS AND PROCEDURES IN WAEMU

Enforcement regulation No. 007/2009/COM/WAEMU sets analytical, safety, preclinical, and clinical standards and protocols for veterinary medicine trials. It provides the outline that the information and documents must be presented in as part of an AMM application.

The procedures for authorizing the marketing of veterinary medicines in WAEMU are in accordance with the agreed upon regulations. With the exception of medicated foods prepared, no veterinary medicine may be issued to the public if it has not received a marketing authorization issued by the WAEMU Commission under the conditions referred to in Regulation No. 02/2006/CM/WAEMU establishing community procedures for marketing authorization and monitoring of veterinary medicines and establishing a Regional Committee for Veterinary Medicine. By derogation, a veterinary medicine may be used on national territory after having received an exceptional authorization for use issued by the national veterinary authority, under the conditions mentioned in this regulation.

Paragraph 1, Article 13 of Regulation No. 02/2006/CM/WAEMU states that "Except for medicinal foods, no veterinary medicine can be put on the market of a Member State free of charge or expensive without a marketing authorization having been issued by the Commission of WAEMU". The marketing authorization issued by the WAEMU Commission enables the Ministry of Animal Resources and Fishery

to issue a ministerial note approving the marketing and utilization of the medicine. The approved medicine can then be sold in Burkina Faso.

Medicinal foods may only be prepared from drug premixes that have been approved for marketing in accordance with this Regulation.

This article also states that: "authorization to market is granted only if the holder justifies:

- that it has a method of manufacture and control process to guarantee the quality of the drug at the mass production level;
- that it has verified the pharmacological properties and the safety of the drug vis-à-vis of animals, humans and the environment,
- that, for medicines for animals producing food for human food, a maximum residue limit based on the active substances that contain and their potentially hazardous residues in the food these animals are established and that the required waiting time to obtain it is justified and that it has a method of detecting these residues;
- that it has verified the efficacy of the drug in relation to the therapeutic indications it proposes"

The different parts of a veterinary drug file consider the three key criteria found in this article namely, quality, safety and efficacy when granting a marketing authorization. An AMM application to WAEMU includes:

- An introductory letter
- A completed AMM application form
- A file including nine parts for a chemical drug:

  Part I: Administrative information, the product characteristics summary (PCR), labeling, and expert reports. Submit the original document plus three copies and 13 CD-ROMs.

  Part II: Analytical trials of veterinary drugs other than immunological drugs. These tests provide information on composition, preparation method, raw material control, intermediate product control, finished product control, and stability. Submit the original document plus three copies and 13 CD-ROMs.

  Part III: Safety tests, which collect toxicological data, and residue studies, which present the pharmacokinetics of residues and their methods of analysis. Submit the original document plus two copies and 13 CD-ROMs.

  Part IV: Preclinical and clinical trials. This section examines preclinical trials (pharmacodynamics, pharmacokinetics, tolerance, resistance) carried out in the laboratory and clinical trials carried out in experimental trials and then in the field. Submit the original document plus two copies and 13 CD-ROMs.

  Part V: Same as Part I.

  Part VI: Data on the analytics of veterinary immunological drugs, including information on composition, manufacturing and production, control of raw materials and finished product,

stability tests and specific measures regarding the prevention of transmission of certain animal diseases. Submit the original document plus two copies and 13 CD-ROMs.

Part VII: Safety tests that reveal potential risks associated with veterinary immunological drugs under the conditions proposed in animals. Submit the original document plus two copies and 13 CD-ROMs.

Part VIII: Demonstrate or confirm the efficacy of the veterinary immunological drug. Submit the original document plus two copies and 13 CD-ROMs.

Part IX: Scientific information and documents relating to safety controls (Part VII) and efficacy trials (Part VIII) of veterinary immunological drugs that must be provided to locate the subject and clarify the controls carried out. Submit the original document plus two copies and 13 CD-ROMs.

As part of the implementation of the centralized AMM procedure at the WAEMU level, two main structures are involved in the evaluation of veterinary drug files: The Permanent Secretariat and the Regional Veterinary Medicine Committee. The List of Veterinary Drug Experts established by Decision No.0049/2010/PCOM/WAEMU also supports the Regional Committee as required. The Veterinary Committee advises on the proposed AMM decisions made by the Regional Committee.

C) THE PERMANENT SECRETARIAT

The AMM application file is filed with the Permanent Secretariat, which is responsible for:

- The study of the management of administrative procedures at the commission level
- The study of the administrative admissibility of the files
- Updating and disseminating the list of authorized veterinary drugs
- The management of pharmacovigilance
- Preparation of the meetings of the Regional Veterinary Medicine Committee

D) REGIONAL VETERINARY MEDICINE COMMITTEE

The Regional Veterinary Medicine Committee:

- Investigates all technical files required for granting of administrative authorizations (e.g., issue or refusal of AMM, renewal, modification, suspension, withdrawal)
- Submits to the WAEMU Commission draft administrative decisions resulting from the evaluation of veterinary drug files

The Regional Veterinary Medicine Committee comprises experts in galenic pharmacy/drug analysis, toxicology/pharmacology, veterinary clinics, and immunology.

E) STEPS AND DURATIONS OF THE AMM PROCEDURE

The application file is filed with the Permanent Secretariat, which reviews its admissibility in relation to regulations in force at the WAEMU level. The duration of the procedure for granting the AMM should not exceed 240 days from the time an application is made. Once the file is recognized as valid, the

Regional Committee conducts a technical evaluation of the file. An evaluation report is developed, and the PCR, notices, and labeling are validated. The documents are forwarded with a proposal for a decision to the WAEMU Commission president no more than 60 days before the end of the deadline. This 60-day period includes consultation with the Summer Committee.

The WAEMU Commission president will make a decision within 240 days of submission of a full application file, but this period can be extended if additional elements are requested.

The decision is conveyed to the applicant and to the authorities responsible for the breeding, trade, and customs of the member states and is published in the official union bulletin. The decision to grant marketing authorization is accompanied by the PCR and the draft notice and labeling.

F) THE AMM ISSUED BY THE COMMISSION

The procedure for authorizing the marketing of veterinary medicines in WAEMU has helped to improve safety requirements for veterinary medicines trade in member states. The reform allows the issuance of a regional AMM after a scientific evaluation process. This regional AMM replaces the national one. The WAEMU regulation goes beyond the AMM by covering another set of obligations for industry stakeholders before importing, distributing, or delivering veterinary medicines. This set of regulations gives WAEMU quality control of veterinary medicines through the network of established laboratories. A community inspection scheme for the manufacture and distribution of veterinary medicines will maintain quality control at the regional level.

4.2.4. MARKETING AUTHORIZATION, VETERINARY PHARMACEUTICAL INSPECTION, AND LICENSING

Veterinary medicine is essential to control the health condition of a herd and ensure quality and productivity. The possible presence of medicine residues in animal products or the environment requires a rigorous assessment of their safety. Therefore, their control, manufacture, marketing, and use must be monitored. To meet these objectives, international legal mechanisms are put in place imposing requirement such as obtaining an AMM for any veterinary medicine.[23] The weakness of the resources of WAEMU member states to ensure the quality, safety, and effectiveness of veterinary medicines necessitated an effort to harmonize veterinary pharmaceutical regulations within the Union. The result meets the objectives set out in the WAEMU Treaty and allows the WAEMU Commission to be the decision making authority over marketing authorization to market and control the quality of veterinary medicines.

Veterinary medicines that have received marketing authorization from WAEMU are listed in a register accessible to all member states. Based on the principle of supra nationality of the legal acts of WAEMU, the authorization granted is applicable within all member states of the Union without any restriction.

4.2.5. POST-MARKETING SURVEILLANCE AND PHARMACOVIGILANCE

In Burkina Faso and other many countries, antimicrobials are widely but illegally available to users, directly or indirectly, with little restriction. Of the 130 countries recently assessed by the OIE, 110 do not have legislation on appropriate conditions for importing, manufacturing, distributing, and using veterinary products, including antimicrobials. These products circulate unchecked like ordinary goods and are often not in good condition for use. Most of the countries in the WAEMU Commission have

not completed the translation of all regional regulations into their national instruments or do not have the necessary human and financial resources to apply, enforce, and control the regional regulations. There is no harmonized monitoring system for the use and circulation of antimicrobials worldwide. However, these data are needed to analyze and monitor the origin of medicines, obtain reliable import data, track circulation, and assess the quality of products.

It is in this context that the OIE was mandated by its member countries to collect this missing information and establish a global database for monitoring the use of antimicrobials in connection with OIE's global zoo sanitary information system. This mandate is also supported by the FAO and WHO as part of WHO's Comprehensive Action Plan against Antibiotic Resistance. The database will provide a solid basis for the work of the three organizations in their fight against antibiotic resistance. Many countries, including Burkina Faso, have been trained to populate the database to enable further analysis of post-marketing surveillance and pharmacovigilance. The process is ongoing, and software is being developed to meet this need.

4.5.6. GOOD SUPPLY MANAGEMENT POLICY AND DIRECTIVES CONCERNING ANTIMICROBIALS

Before the privatization of the veterinary profession in Burkina Faso, veterinary medicines were managed by public service. After privatization, medicines management was handed to private veterinarians. Within the same time, private veterinarians were mandated to cover the areas not covered by public service. Public service, in line with WAEMU Commission regulations, is putting efforts in place to control the importation, storage, distribution, and utilization of these medicines. There is no specific regulation for antibiotics leaving the domain without an appropriate management system. Nearly 10 years later, the OIE has published intergovernmental standards for the responsible and prudent use of antimicrobials in terrestrial and aquatic animals. These standards were fully updated in May 2015 and cover the use of antimicrobials, program monitoring, quantity monitoring, and assessment of risks linked to the emergence or spread of resistant bacteria resulting from their use in animals. The OIE also publishes intergovernmental standards on the list of antibiotics important in veterinary medicine to provide guidance in veterinary medicines management.

A) SITUATION ANALYSIS AND ASSESSMENT

Monitoring Antimicrobial Resistance

The LNE, through its medical bacteriology and food microbiology services, performs different types of research and analysis:

- The search for antibiotic residue in animal foodstuffs
- Microbiological analysis of animal-borne foodstuffs (e.g., milk and dairy products, eggs and egg products, meats and deli meats, preserves)
- Routine sensitivity tests to identify germs responsible for animal diseases and the most active antibiotics on these germs
- The implementation of active and passive surveillance campaigns of the main animal pathologies (viruses, bacteria, parasites)

Monitoring the Use of Antimicrobials

The DVPHL through the Direction Générale des services veterinaries (Directorate General of Veterinary Services):

- Organizes frameworks for consultation between private veterinarians (wholesalers, clinicians) to carry out an overview of the existence and increased development of the parallel market for veterinary medicines
- Organizes unannounced reports and checks on the ground to seize medicines that are on the market illegally
- Maps veterinary pharmacies every two years and grants approvals for imports of veterinary medicines into Burkina Faso

Laboratory Capabilities and Standards Available at the National Level to Deal with AMR

The LNE has human resources to carry out bacteriological analyses. Bacterial sensitivity tests are carried out only by the LNE because not all regional livestock laboratories (LREs) are functional and have no means of carrying out these tests. The lack of reagents (culture media and antibiotic discs) and have inadequacy capacity to run the test and LRE actors, and there is no national repository for germ identification and the production of antibiotics.

To identify and quantify drug residues (antibiotics) in animal foodstuffs and pesticide residues in honey, equipment has been acquired, but it remains nonfunctional due to a lack of reagents and the need to build the capacity of technicians.

Consumption Data/Distribution for Antimicrobials in Animal Health

Import records show that tetracyclines are one of the most widely used groups of antibiotics in veterinary medicine because of their broad spectrum, numerous indications, and low toxicity. The three known natural tetracyclines are used in veterinary medicine: tetracycline, oxytetracycline (Teramycin) and chlortetracycline (Aureomycin).

Table 2. Proportion of antibiotics within imported veterinary drugs

Years	Total cost of imported drugs (FCFA)	Cost of antibiotics (FCFA)	Percentage of antibiotics cost (%)
**2014**	2,707,689,894	139,909,500,	5.17
**2015**	2,669,504,180	413,754,585.5	15.50
**2016**	2,756,834,166.88	330,825,894	12.0

Source: DSPVL annual reports (2014 and 2016)

Monitoring Data at the LNE

At the LNE, data are routinely monitored but not analyzed. However, a 2016 study showed that of 20 samples from poultry, E. coli accounted for 70% of the identified germs followed by Proteus mirabilis (15%), Salmonella gallinarum and Staphylococcus. Spp (10%), and Shigella boydii (5%).

In 2014, qualitative analyses for the search for drug residues (antibiotics) in the eggs and kidneys of cattle marketed in the city of Ouagadougou showed the presence of drug residues in 25% of the 100 eggs analyzed and in 20% of the 50 cattle kidney samples analyzed at the LNE.[26]

B) GUIDELINES ON SURVEILLANCE AND CONTROL

The guidelines on surveillance and control developed by OIE were adopted by member states, including Burkina Faso, at the OIE 79th General Assembly in May 2011.

Guidelines on Legislation and Use of Veterinary Drugs and Risk Analysis

Veterinary legislation is an essential part of national provisions that enable veterinary authorities to fulfil their key functions, including surveillance, rapid response to health emergencies, and prevention and control in all areas of animal health. Thus, veterinary drugs are an essential component of the implementation of such legislation. At its 79th General Session in May 2011, the OIE adopted a guideline setting out recommendations for the development of a regulatory framework for member states to implement an effective animal health policy and to give the necessary legal expertise to control services. This guideline can be used to support countries to improve the animal health of their livestock and meet the standards set by the OIE. In addition to recommendations on the general legal framework, the criminal system, and the powers of the competent authority, this guideline sets out specific recommendations in each technical sector for minimum legislation to be put in place. A chapter is thus devoted to veterinary pharmacy and defines the main themes to be regulated throughout the veterinary medicines chain, including authorization of marketing, raw materials, manufacturing and distribution conditions, and quality control of medicines.

Harmonization of National Antibiotic Resistance Monitoring and Surveillance Program (Chapter 6.7. of the Land Code)

Regular monitoring of antibiotic resistance and its evolution in pathogenic and nonpathogenic bacteria, taken from animals, animal foodstuffs, or the environment is an essential component of any strategy to limit the spread of antibiotic resistance. Such monitoring also optimizes the choice of antibiotics in the treatment of animals and makes recommendations for prescribing veterinarians. As such, the OIE adopted a guideline in May 2003 to harmonize national antibiotic resistance monitoring and surveillance programs in animals and animal feed. The main objective of this guideline is to provide criteria for developing national antibiotic monitoring and monitoring programs or for harmonizing existing ones. Such monitoring tracks the evolution of resistance, detects the emergence of new mechanisms, and provides data needed to analyze risks to public health and animal health to support recommendations to prescribers for prudent use of antibiotics. The guideline defines a sampling strategy with recommendations for:

- Places of collection (animals, food, and animal feed)
- Categories of bacteria to follow
- Classes of antibiotics to be tested
- Information to be saved

It is important to carry out this monitoring over time to study developments and take appropriate management measures.

Laboratory Methods Used for Antibiotic Resistance Tests (Chapter 1.1.6. of the Earth Manual)

This guideline allows laboratories to define the method of in vitro testing of the sensitivity of pathogens to the antimicrobials to be used. It is essential that the different laboratories use a similar procedure to establish their method of analysis and harmonized and standardized modalities to interpret the results. Such standardization of methods of analysis and interpretation of results allows a direct comparison of the results between different countries or regions.

Control of the Quantities of Antimicrobials Used in Animal Production (Chapter 6.8. of the Land Code)

The main objective of this guideline is to define how to collect a certain amount of data (qualitative and quantitative information) to show how antimicrobials are used in animal production by species, animal, and antimicrobial class. The purpose is to assess exposure to antimicrobials. These data can contribute to the interpretation of the results of antibiotic resistance surveillance and enable a more accurate and targeted response by implementing reduction strategies tailored to the situations encountered. Data will also be used to judge the impact of the measures adopted. The basic information to be collected is the number of antimicrobials (in kg of active substance) used each year by species, the nature of their use, and the route of administration. Information on dosage and duration of administration are important elements in estimating the use of antimicrobials in animals.

Assessment of the Risks of Antibiotic Resistance Secondary to the Use of Antimicrobials in Animals (Chapter 6.10. of the Land Code)

This guideline sets out a methodology for OIE member states to establish a risk analysis applied to antibiotic resistance to assess and manage the health risks posed to humans' and animals' secondary resistance to antimicrobial use in animals. This analysis should consider the identification of the danger based on the results of the monitoring set up, the assessment of the spread of resistance, the assessment of exposure to that danger, and the potential consequences. All of this should help to estimate risk and help define the management measures to be adopted and the communication to be undertaken on risks.

Responsible and Prudent Use of Antimicrobials in Veterinary Medicine (Chapter 6.9. of the Land Code)

This guideline sets recommendations for all players in the veterinary drug chain with the aim of adopting prudent use of antimicrobials. The recommendations are aimed primarily at the competent authorities in charge of establishing the necessary regulatory framework for conditions for evaluating and authorizing antimicrobials, the control of their quality and advertising, the supervision of their distribution, and the modalities of their monitoring. Similarly, recommendations are also made to the pharmaceutical industry, pharmacists in charge of the delivery of antimicrobials at retail, prescribing veterinarians, and animal breeders. The entire chain of antibiotics, from authorization to administration to the animal, must

be the subject of special attention, with specific recommendations at each stage of the drug's life for each concerned actor.

4.2.7. PRESCRIPTION, DISPENSING, AND UTILIZATION

According to Article 230 of the Animal Health Code of Burkina Faso, the veterinarian must prescribe as a priority a licensed veterinary medicine for the animal species under consideration and for the intended therapeutic indication. Section 228 states that the list of drugs exempted from writing a prescription and the conditions of that exemption are set by regulation. This prescription can only prescribe the amount of medication needed for the treatment. For medicinal foods, the prescription can only prescribe one treatment lasting no more than three months. The success of the fight against antibiotic resistance depends on the implementation of global strategies by all countries on five continents (public, veterinary, and environmental health authorities). As far as animal health services are concerned, the implementation of the OIE's standards involves at the national level:

- The presence of adequate legislation on the use of antimicrobials
- Good governance of national animal health systems
- A well-trained veterinary profession regulated by law

The medicated food is regularly checked to ensure its homogeneity, stability, and good preservation and that it is kept during the period covered by the prescription.

The deliverance verifies compliance with the conditions of issuance provided by national regulations, such as date of manufacture, importation, transfer, or issuance. In facilities authorized to distribute medicinal food, the following information is needed: the date of acquisition, transfer, or issuance. Retail delivery is conditional on the presentation of a prescription from a veterinary doctor.

The Minister of Animal Resources and Fishery determines by decree the maximum concentrations and conditions of use of complemented and supplemented foods. By derogation from the provisions of Article 6 of this decree, a veterinary medicine may be used on national territory after having received an exceptional authorization for use by the national veterinary authority, under the conditions mentioned in Articles 22 and 23 of Regulation No. 02 /2006/CM/UEMOA as of March 23, 2006, establishing community procedures for marketing authorization and monitoring of veterinary medicines and a regional committee of veterinary medicine. Veterinary medicines imported into a WAEMU member state under an exceptional authorization for use can only be used within this country. If exploitation of a veterinary medicine is likely to pose a danger to public health, the Minister of Animal Resources and Fishery may, by reasoned decision, suspend the use of the product after informing the Commission of WAEMU.

5. GENERAL DISCUSSION

5.1. HUMAN HEALTH SECTOR

The review of indicators of policies and regulatory framework for antimicrobials in the human health sector revealed that Burkina Faso has a national pharmaceutical policy in which the rational use of

medicines is a very important focus. There are also regulatory texts and therapeutic guidelines for the proper use of medicines but no orientation guide or strategic plan specifically concerning the proper use of antimicrobials. The absence of such guidelines does not allow coordinating structures to promote rational use of antimicrobials, which could lead to inappropriate and inadequate use of antimicrobials in hospitals, health centers, and community settings. This rapid assessment also revealed marketing authorization, licensing, and inspection regulatory shortcomings, including the withdrawal of marketing authorizations, the failure to grant temporary use authorizations, and the absence of regulations governing penalties. This inadequacy could be explained by the fact that formally, the regulatory missions of the pharmaceutical sector were not coordinated by a single structure with financial and legal autonomy. The ANRP will help to avoid any misconduct with AMR and AMS. Regulation of the activities of all actors involved in the use of medicines is essential to ensure rational and appropriate use of antimicrobials. For regulations to be effective, they must be enforced and the regulatory body adequately funded and supported by the judicial structure.[24] The misuse of drugs, especially antimicrobials, can be explained by the absence of a strategic plan for rational management of antimicrobials, which could lead to an increase in antimicrobial resistance.

5.2. ANIMAL HEALTH SECTOR

The veterinary medicines policy and regulatory framework in Burkina Faso is based on the Decree No/2018/0729/PRES/PM/MRAH/MS/MATD/MSECU/MCIA Portant réglementation de la pharmacie vétérinaire au Burkina Faso. This decree outlined the provisions for importation and marketing and the control of conditions for opening, operating, manufacturing, commercializing, and wholesale distribution of veterinary medicines. The provisions of this decree apply to veterinary medicines intended to be tested and presented in the form of pharmaceutical specialties, prefabricated veterinary medicines, and medicated pre-mixes. Special provisions are made for medicinal foods. However, the decree issued in 2018 is not operationalized with subsequent ministerial orders allowing the decree to be fully operational. Therefore, the regulation of medicines in Burkina Faso is still weak. The regional veterinary pharmaceutical regulation in WAEMU, which superseding the decree, was put in place to fill the gap.

Despite the decree, the regional veterinary medicines policy and regulatory framework did not avail implementation of regulations that aligned with the provisions of the decree. The WAEMU Commission tasked itself with providing Burkina Faso and other countries in the region with key regulations related to marketing authorization of veterinary medicines. The regulatory framework established by WAEMU sets key steps to be followed by countries before receiving authorization that focus on safety and good utilization of medicines.

The marketing authorization of veterinary medicines in the WAEMU zone is an important tool for veterinary medicine users due to safety concerns that must be taken into account to avoid any adverse consequence. Nevertheless, the fraud related to these medicines is the main concern in Burkina Faso, as nonauthorized persons are importing and selling these medicines.

The marketing authorization, veterinary pharmaceutical inspection, and licensing is provided by the Ministry of Animal Resources and Fishery. The license is granted to veterinarians who have studied and understand the importance of these medicines.

Post-marketing surveillance and pharmacovigilance are almost nonexistent due to weakness in the system but are critical to controlling adverse effects of the medicines. A few supporting projects have enabled the Ministry of Animal Resources and Fishery to investigate the issue, particularly as it relates to the AMR situation in the country. Efforts should be made to address this issue, which may help control AMR in the country.

The weaknesses in veterinary medicine management are related to the lack of:

- Good management policy of supply and directives concerning antimicrobials
- Guidelines on surveillance and control
- Prescription, dispensing, and utilization

These key elements are critical to be considered for veterinary medicines.

6. RECOMMENDATIONS

In line with the assessment, it has been revealed that Burkina Faso is lacking an adequate regulation system in both the human and animal health sectors. In the animal health sector at the national level, only one decree is in place to regulate all systems. This is not sufficient to govern the veterinary medicines value chain, from the production to the end users. To address these issues, which are key to improving medicines issue in both the human and animal health sectors, there is a need to:

- Operationalize the One Health national multisectoral coordination platform to globally address AMR and AMS in both sectors
- Operationalize the Antimicrobial Technical Thematic Commission and its sub-commissions
- Develop a multisectoral operational plan for the appropriate use of antimicrobials to address AMS, which is crucial to improve AMR at all levels
- Strengthening antimicrobial importation surveillance in both sectors to control the quantity of medicines imported in the country
- Strengthen surveillance of antimicrobial use, prescribing, and consumption in both sectors to avoid misuse and medicines smuggling within and outside the country
- Develop and disseminate information and education and communication materials on medicines resistance and use in the human and animal health sectors
- Develop a national regulatory framework for appropriate use of affordable, quality-assured antimicrobials in humans and animals
- Develop and enact legislation and regulations on the importation, marketing authorization, production, and use of antimicrobials in both sectors
- Develop an AMS national action plan for both sectors
- Validate and implement the national multisector action plan to combat AMR
- Establish external quality control of design laboratories to detect and notify antimicrobial-resistant pathogens
- Strengthen integrated multisector surveillance of AMR
- Establish a coordinating and collaborating body for the various sectors involved in the fight against AMR, including the development and implementation of standardized operating procedures

- Establish infectious case isolation sites in tertiary hospitals

7. CONCLUSION

The regulatory aspect of AMS is a very important strategic focus for stemming the emergence of AMR. The rapid assessment of antimicrobial management policies and the regulatory framework is conducted under MTaPS using the WHO benchmarks for IHR. This assessment showed the involvement of several government departments in the management of AMR and AMS. The existence of a system of registration, surveillance, supply, and dispensing of medicines at the human health level was evidenced, as was the conduct of inspections of pharmaceutical establishments. However, it was noted that there was a deficiency in the regulation of antimicrobial management. Among these deficiencies was the absence of an appropriate policy and regulation system for the sector. The establishment of the ANRP will help transformation regulation of the sector.

According to the assessment, the animal health sector is lagging far behind and needs more improvement. The sector is very weak and in terms of regulation must be improved as soon as possible to improve AMR and AMS in Burkina Faso. Within this sector, before March 2006 the importation and trade of veterinary medicine was mainly base on the economic aspect, and proof of safety was the responsibility of traders. The country was not able to produce this proof due to the lack of technical capacity. However, putting the burden of proof on traders was problematic. The WAEMU Commission’s decision to fill this gap by providing regulations for the entire region will help avoid the nonregulation areas covered by the member states of the WEAMU zone. The authorization of marketing of veterinary medicines established by the WAEMU Commission helped to increase the safety of medicines imported, prescribed, and distributed by veterinary medicines managers. The regional AMM sets out a set of obligations for industry players to import, distribute, or deliver veterinary medicines, including antimicrobials, in a safer manner. It gives WAEMU quality control of veterinary medicines through the network of laboratories established within the region. A community inspection scheme for the manufacture and distribution of veterinary medicines was also established to carry out inspection on a regular basis of all veterinary medicines circulating within the WAEMU zone. The Animal Health Code defines the conditions under which pharmacovigilance is practiced with respect to veterinary medicines. The code set the obligations of professionals involved in the manufacturing, operating, delivering, and use of veterinary medicines.

Despite the implementation of the AMM and the various laws, regulations, directives, recommendations, and opinions that govern veterinary medicines, including antimicrobials, the gold standard remains rational prescription and utilization of veterinary medicines to avoid the surge of antimicrobial-resistant pathogens that can adversely affect human, animal, and environmental health.

8. REFERENCES

1. Cameron A, Ewen M, Auton M, Abegunde D. The world medicines situation 2011. Med Prices Availab Affordability http://www.WhoIntmedicinesareaspolicyworldmedicinessituationWMSch6wPricingv6 Pdf. 2011
2. Organization WHO, others. The world medicines situation. 2004 [cité 13 mars 2017]; Disponible sur: http://apps.who.int/iris/handle/10665/68735
3. Organisation mondiale de la Santé animale. La stratégie de l'OIE sur la résistance aux agents antimicrobiens et leur utilisation prudente 2016.
4. World Health Organization. Perspectives Politiques de l'OMS sur les médicaments: Endiguer la résistance aux antimicrobiens.
5. World Health Organization. Global antimicrobial resistance surveillance system: manual for early implementation. 2015.
6. Ndihokubwayo JB, Yahaya AA, Desta AT, Ki-Zerbo G, Odei EA, Keita B, et al. Antimicrobial resistance in the African Region: Issues, challenges and actions proposed. (16):4.
7. World Health Organization. Benchmarks for International Health Regulations (IHR) Capacities, Tools Fef 2019.
8. World Health Organization. Monitoring and Evaluation International Health Regulations (2005). Joint External Evaluation Tool Second Edition - January 2018.
9. MINISTERE DE LA SANTE. Decree No. 2018-0861/PRES/PM/MINEFID/MS establishing the National Agency for Pharmaceutical Regulation.
10. Ministère de la santé. Politique pharmaceutique au Burkina Faso. 2ème édition 2012. pp 36. [Internet]. 2012. Disponible sur: http.www.sante.gov.bf
11. Ministère de la santé. Plan d'action national multisectoriel de lutte contre la résistance aux antimicrobiens 2017-2020, Burkina Faso.
12. Ministère de la santé. Rapport de l'étude des caractéristiques de l'utilisation des médicaments de la mère et de l'enfant dans trois régions sanitaires du Burkina Faso. 2014.
13. Sana B, Kaboré A, Hien H, Zoungrana BE, Meda N. Etude de l'utilisation des médicaments chez les enfants dans un contexte de gratuité des soins. Pan Afr Med J [Internet]. 12 déc 2019;34. Disponible sur: http://www.panafrican-med-journal.com/content/article/34/194/full/
14. Office international des épizooties. Code sanitaire pour les animaux terrestres. Paris: OIE; 2010.
15. Anthony F, Acar J, Franklin A, Gupta R, Nicholls TJ, Tamura Y, et al. Antimicrobial resistance: responsible and prudent use of antimicrobial agents in veterinary medicine: -EN- -FR- -ES-. Rev Sci Tech OIE. 1 déc 2001;20(3):829-39.
16. Moulin G; Lambert C. International approach for veterinary medicinal products: OIE and Codex Alimentarius. [Communication, Power Point 36 p]. Conférence de l'OIE sur les médicaments vétérinaires en Afrique : Harmonisation et amélioration de !'enregistrement, de la distribution et du contrôle qualité. Dakar, 25 au 27 mars, 2008.
17. Jones P. International Approach for Veterinary Medicinal Products (VICH). [Communication, Power Point 30 p]. Conférence de l'OIE sur les médicaments vétérinaires en Afrique Harmonisation et amélioration de !'enregistrement, de la distribution et du contrôle qualité. Dakar. 25 au 27 mars, 2008.
18. Daré I. Harmonisation de l'enregistrement et du contrôle des médicaments vétérinaires en Afrique L'exemple de l'Union économique et monétaire ouest-africaine (UEMOA). 2007;15.

19. Ly C.; Ba M.; Coly R.; L'approvisionnèrent en médicaments et vaccins vétérinaires au Sénégal. Deuxième journée d'études de l'ordre des docteurs vétérinaires du Sénégal. Kaolack, 6 mars 2002.- 19p.
20. Abiola F.A. Le marché mondial des médicaments vétérinaires. (45). In : Actes du séminaire sur l'utilisation des médicaments vétérinaires en Afrique Subsaharienne. Dakar, EISMV, 6 au 9 Février 2001.-170p.
21. Teko-Agbo A. ; Messomo Ndjana F. ; Walbadet L. ; Niang EL.H. ; Abiola F.A. Qualité des médicaments vétérinaires en circulation au Cameroun et au Sénégal. [Communication, Power Point 32 p]. Conférence de l'OIE sur les médicaments vétérinaires en Afrique : Harmonisation et amélioration de !'enregistrement, de la distribution et du contrôle qualité. Dakar, 25 au 27 mars 2008.
22. Teko-Agbo A.; Biaou F.C.; Akoda K. ; Abiola. F.A. Contrefaçons et malfaçons de trypanocides à base de diminazene et d'anthelminthiques contenant de l'albendazole au Bénin et au Togo. RASPA, 1 (1) : 29-47.
23. Dehaumont P. ; Moulin G. Evolution du marché des médicaments vétérinaires et de leur encadrement règlementaire : conséquence sur leur disponibilité. Bull. Acad. Vet. France, 158 ; (2), 125-136.
24. World Health Organization. Perspectives Politiques de l'OMS sur les médicaments : Promouvoir l'usage rationnel des médicaments éléments principaux. 2002.
25. WHO benchmarks for International Health Regulations (IHR) capacities. Geneva: World
26. Health Organization; 2019. Licence: CC BY-NC-SA 3.0 IGO.
27. ZERBO L. Habibata., 2014. Étude préliminaire sur l'utilisation des antibiotiques dans les élevages de poules pondeuses et la présence de résidus d'antibiotiques dans les Œufs commercialisés à Ouagadougou (Burkina Faso).
