# Supplementary material for: Landscape Analysis of Existing Policies, Laws and Regulations on Antimicrobial Stewardship in 10 Low- and Middle-Income Countries
Source: Antibiotics (Basel). 2026 Aug 10;15(8):770. doi: 10.3390/antibiotics15080770 (PMC13510074; doi:10.3390/antibiotics15080770)
Supplement: Supplementary file 1 [file antibiotics-15-00770-s001.zip › S3 Country Reports/S3C_Cameroon (Human & Animal Health).pdf]

REPORT

SITUATIONAL ANALYSIS ON ANTIMICROBIAL STEWARDSHIP IN CAMEROON

August 2020

TABLE OF CONTENTS

ACKNOWLEDGEMENTS....v
ABBREVIATIONS....vi
LIST OF TABLES....i
LIST OF FIGURES....i
1. INTRODUCTION....1
2. BACKGROUND AND RATIONALE....1
3. METHODOLOGY OF THE ANALYSIS....3
4. MARKETING AUTHORIZATION, LICENSING AND INSPECTION....4
4.1. HUMAN HEALTH....4
4.1.1. Organization and Players in the Pharmaceutical Sector....4
4.1.1.1. Organization of the Pharmaceutical Sector....4
4.1.1.2. 1.2 Players in the Pharmaceutical Sector....4
4.1.2. Marketing Authorization....6
4.1.3. Pharmaceutical Inspection....7
4.2. ANIMAL HEALTH....8
4.2.1. Bodies responsible for the management of veterinary medicinal products....8
4.2.1.1. National (Central) Regulatory Authority for Veterinary Drugs....8
4.2.1.1.1. Ministry of Livestock, Fisheries and Animal Industries....8
4.2.1.1.2. Other stakeholders....9
4.2.1.2. Deconcentrated authorities (region, department, district) for the regulation of veterinary medicines 9
4.2.1.2.1. Deconcentrated services of the Ministry of Livestock, Fisheries and Animal Industries 9
4.2.1.2.2. Deconcentrated services of other sectoral administrations....10
4.2.1.2.3. Decentralized territorial authorities (regions and municipalities)....10
4.2.2. Key players, roles and responsibilities in veterinary antimicrobial use....10
4.2.2.1. International actors: FAO / OIE / WHO....11
4.2.2.2. Foreign laboratories or pharmaceutical companies....12
4.2.2.3. National actors....12
4.2.2.3.1. National public actors....12
4.2.2.3.1.1. MINEPIA....12
4.2.2.3.1.2. ONVC and ATASPAC....13
4.2.2.3.1.3. CUSTOMS and MINCOMMERCE....13
4.2.2.3.1.4. LANACOME and LANAVET....13
4.2.2.3.1.5. ANOR....14
4.2.2.3.2. Community actors: regions and municipalities....14

4.2.2.3.3. Administrative authority and law enforcement agencies (FMO) 14
4.2.2.3.4. Private sector players 14
4.2.2.3.4.1. Official circuit: Actors, roles and responsibilities 14
Importers wholesalers-distributors 14
Pharmaceutical company representative 15
retailer-distributors 15
Veterinary practices/clinics 15
Breeders' groups 15
Pet owners 16
4.2.2.3.4.2. Unofficial circuit: actors, roles and responsibilities 16
Illegal trader importer 16
Street vendor, on the run (lawn) 16
Other actors in the illicit circuit 16
4.2.3. Marketing authorisation for veterinary medicinal products 18
4.2.3.1. Marketing authorisation requirement for veterinary medicinal products 18
4.2.3.2. General measures relating to the definition of veterinary medicinal products and the regulation of their importation, manufacture, distribution, use and trade 19
4.2.3.3. Raw materials for the preparation of veterinary medicinal products, quality standards for raw materials used in the manufacture and composition of veterinary medicinal products 20
4.2.3.4. Withdrawal times and maximum residue limits 20
4.2.3.5. Requirements for substances in antimicrobials that may interfere with veterinary controls 20
5. Post-marketing surveillance and pharmacovigilance 21
5.1. HUMAN HEALTH 21
5.2. ANIMAL HEALTH 23
5.2.1. Quality of veterinary medicines 23
5.2.1.1. Verification of the characteristics announced by the manufacturer 23
5.2.1.2. Conditions for carrying out the tests and qualification of the experts involved in the tests 23
5.2.2. Notification of side effects to the competent authority 24
5.2.3. Establishment of mechanisms for product traceability, withdrawal or recall 24
5.2.4. Drug Use and AMR Policies/Regulations 24
6. PROCUREMENT POLICIES AND GUIDELINES FOR ANTIMICROBIAL STEWARDSHIP 25
6.1. HUMAN HEALTH 25
6.2. ANIMAL HEALTH 30
6.2.1. Policies for good supply management at establishments producing, storing or wholesaling.... 30
6.2.1.1. Registration and authorisation of all operators manufacturing, importing, storing, processing, wholesaling or transferring veterinary medicinal products ....

30

6.2.1.2. Organization of the distribution circuit to ensure traceability, recall, withdrawal and proper use 31

6.2.2. Definition of the responsibilities of the actors 31

6.2.3. Obligation (imposition) of rules of good practice specific to each activity 31

7. PRESCRIBING, DISPENSING AND USE 32

7.1. HUMAN HEALTH 32

7.1.1. National List of Essential Medicines 32

7.1.2. Appropriate use of antimicrobials 33

7.2. ANIMAL HEALTH 34

7.2.1. Regulation of appropriate use by prescribers, dispensers and end users 34

7.2.1.1. Prescribing and dispensing rules 34

7.2.1.2. Trade Restriction 35

7.2.1.3. Supervision of accredited organizations by a licensed professional 35

7.2.1.4. Regulation of all forms of advertising and other marketing and promotional activities 35

7.2.2. Antimicrobial use in animal health in Cameroon 37

7.2.2.1. At the national level 37

7.2.2.2. In livestock production units for production animals 38

7.2.2.2.1. Prescription, administration 38

7.2.2.2.2. Residuals and Waiting Times 39

7.2.2.3. In pets and veterinary clinics in Cameroon 39

7.2.3. Adverse effects and (in)efficacy of veterinary antimicrobials 41

8. ANALYSIS OF STRENGTHS, WEAKNESSES, OPPORTUNITIES AND THREATS 44

8.1. HUMAN HEALTH 44

8.2. ANIMAL HEALTH 47

9. CONCLUSION 52

10. REFERENCES 53

11. LIST OF CONTRIBUTORS 58

ACKNOWLEDGEMENTS

This report is the result of a collaboration between human and animal health stakeholders, with technical and financial support from the USAID/MTAPS partner. Our sincere thanks go to all stakeholders for their various contributions and their multi-faceted support in the development and finalization of this document.

ABBREVIATIONS

AM: Antimicrobials

MA: Marketing Authorization

ANRPh: National Pharmaceutical Regulatory Authority

ARVs: Antiretrovirals

AWaRe: Access Watch Reserve

ANOR: Agency for Standards and Quality

ATASPAC: Association of Technicians in Aquaculture, Health, Fisheries and Animal Productions of Cameroon, called

BNL: Non-profit organization

GP: Good Practices

CAM: Antimicrobial consumption

CENAME: National Supply Centre for Essential Medicines and Consumables

CEPCA: Council of Protestant Churches of Cameroon

CHMP: Centrale Humanitaire Médico Pharmaceutique

CIMEP: Centre d'Informations MédicoPharmaceutiques

CMA: Centre Médical d'Arrondissement

CNM: National Commission on Medicines

CEMAC: Economic and Monetary Community of Central Africa

IHC: Integrated Health Centre

DAOAH: Foodstuffs of Animal and Fishery Origin

DDEPIA: Departmental Delegate for Livestock, Fisheries and Animal Industries

DSV: Direction des Services Vétérinaires

DHIS 2: Data Health Information System 2

DLMEP: Direction de la Lutte contre la Maladie, les Epidémies et les Pandémies

DPML: Directorate of Pharmacy, Medicines and Laboratories

DROS: Direction de la Recherche Opérationnelle en Santé

FAO: Food and Agricultural Organization

FEICOM: Fonds Spécial d'Equipement et d'Intercommunale (Special Fund for Equipment and Intercommunity Intervention)

SWOT: Strengths, Weaknesses, Opportunities and Threats

FMO: Forces de Maintien de l'Ordre

FALC: Ad Lucem Medical Foundation Cameroon

FOSA: Health Training

FRPS: Regional Fund for Health Promotion

ICH: International Council for Harmonisation of Technical Requirements

IGSPL: General Inspectorate of Pharmaceutical Services and Laboratories

ISO: International Organization for Standardization

STI: Sexually Transmitted Infection HPAI:
Highly Pathogenic Avian Influenza

JP: Japanese Pharmacopoeia

LANACOME : National Laboratory of Quality Control of Medicines and Expertise

LME: WHO Model List of Essential Medicines **LNSP:**
National Public Health Laboratory **LANAVET:** National
Veterinary Laboratory

MINEPIA: Ministry of Livestock, Fisheries and Animal Industries **MTaPs:**
Medicines, Technologies, and Pharmaceutical Services **MINSANTE:**
Ministry of Public Health

MSH: Management Science for Health

OCASC: Catholic Health Organization in Cameroon

OHCEA: One Health in Central and East Africa

WHO: World Health Organization

ONPC : National Order of Pharmacists of Cameroon

OIE: World Organisation for Animal Health

NGO: Non-Governmental Organization

ONVC: National Order of Veterinarians of Cameroon

PAD: Port Autonome de Douala

SOP: Standard Operating Procedures

PG2A: Antimicrobial and Pest Management Plan

PRODEL : Livestock Development Project

PBF: Performance Based Fundings

PEC: Prise en Charge

PNLT: National Tuberculosis Control Programme

NPP: National Pharmaceutical Policy

NSP: National Strategic Plan

PTF: Technical and Financial Partner **PTN**:

National Therapeutic Protocol **RAM**:

Antimicrobial Resistance

RENAFSOM: National Network of Health Training of Muslim Obediences

RMA: Monthly Activity Report

AIDS: Acquired Immune Deficiency Syndromes

HSS: Health Sector Strategy

SYNAME: National Drug Supply System

SDPSPV: Sub-Directorate of Pharmacy and the Veterinary Private Sector

TB: Tuberculosis

USP: United States Pharmacopeia

UMA: Use of Antimicrobials

HIV: Human Immunodeficiency Virus

VCIA: Critically Important Veterinary Antimicrobials

VHIA: Very Important Veterinary Antimicrobials

VIA: Veterinary Important Antimicrobials

TABLE LIST

Table 1: Description of formal sector actors and stakeholders 26
Table 2: Antibiotic consumption (quantity) and number of prescriptions in laying hen farms in the Western region (Cameroon) between January and October 2012 (Woumbe, 2013) 38
Table 3: Dosing accuracy (UDD/ADD ratio) according to the different families of antibiotics administered and the different indications of use in laying hen farms in the Western region (Cameroon) between January and October 2012 (Woumbe, 2013) 39
Table 4: Prescription frequency of veterinary antibiotics of human health .... i mportance (Nno Nkassa, 2016) 41

LIST OF FIGURES

Figure 1: Complexity of veterinary drug(antimicrobial) distribution flows between different actors in the official (black) and unofficial (red) channels (adapted from Messomo (2006), Mazra (2014), Mouiche et al, (2018) and interviews with stakeholders................................ 17
figure 2: Criteria for prescribing antibiotics (Nno Nkassa, 2016) 40
Figure 3: Annual evolution of the frequency of use of antibiotic families (Nno Nkassa, 2016) 42
Figure 4: Annual evolution of the use of antibiotic molecules (Nno Nkassa, 2016) 43

1. INTRODUCTION

Antimicrobial resistance is the ability of a microorganism (bacteria, viruses, parasites, fungi) to grow in the presence of an antimicrobial agent. It occurs when a microorganism undergoes changes such that the drugs used to treat infections caused by these microbes become ineffective.

In Africa, it is estimated that antimicrobial resistance is one of the major causes of the increase in the prevalence of emerging and re-emerging infectious diseases. If left unchecked, this increase could lead to the death of an estimated 4.15 million people in Africa each year by 2050. It is generally accepted that self-medication, inappropriate use of plant protection products, inadequate waste management, and excessive consumption of antimicrobials in human and veterinary medicine, animal husbandry, and agriculture are the major factors contributing to the occurrence of AMR. All of these practices result in the presence of antimicrobials and/or their residues in foodstuffs and the environment (waterways, soil and subsoil), thereby significantly increasing the risk of development of resistant microbial strains.

Thus, the fourth specific objective of the national AMR plan developed by Cameroon in 2018 is to optimize the use of antimicrobials in human, animal and plant health.

2. BACKGROUND AND RATIONALE

Cameroon has legislative and regulatory texts to ensure the quality, safety and efficacy of medicines in the human, animal and environmental health sectors. However, the implementation of these texts to ensure the rational use of antimicrobials in the above sectors remains a challenge.

Indeed, none of the legal instruments specifically mention antimicrobial resistance, although some aspects of antimicrobial resistance control are addressed (OHCEA, 2017). These shortcomings have led to

the proliferation of irrational antimicrobial use and thus the development of antimicrobial resistance.

Antimicrobial use in animal health contributes significantly to the emergence of AMR. It is recognized that the existence of an informal sector for the sale of drugs and phytosanitary products, the uncontrolled prescription and dispensing of antimicrobials, self-medication and misuse of antimicrobials are among the factors that favour the development of AMR. Indeed, the joint external evaluation report of the main capacities of the International Health Regulations (IHR)-2005 (MINSANTE, 2017) of the technical area related to AMR showed that Cameroon did not have capacity (minimum score) for the detection of AMR, for the surveillance of infections caused by antimicrobial resistant pathogens, for the existence of a program for the prevention and control of health care associated infections and for the implementation of antimicrobial stewardship activities. As a result of this report, it was agreed that these capacities should be developed by 2018. Thus, Cameroon developed a three-year national action plan (2018- 2020) to combat antimicrobial resistance (MINSANTE, 2018) by aligning it with the global action plan developed by WHO with the collaboration of FAO and OIE (WHO, 2016) and tying it to the Growth and Employment Strategy document, the Health Sector Strategy 2016 - 2027 and the Rural Sector Development Strategy with the vision of making Cameroon a nation where the environment, human, animal and plant health are preserved through the responsible use of good quality antimicrobials to slow the emergence of resistant microbes and prevent their spread to other sites or locations. The strategic objectives of the AMR Action Plan are: 1) to increase awareness and understanding of the problem of antimicrobial resistance; 2) to enhance knowledge through surveillance and research; 3) to reduce the incidence of infections; 4) to optimize the use of antimicrobial agents; and 5) to ensure sustainable investments to combat antimicrobial resistance.

3. METHODOLOGY OF THE ANALYSIS

For the preparation of this report, a systematic review of legislation, regulations, administrative requirements and other instruments related to medicines management was conducted. The legal and institutional framework for antimicrobial stewardship in animal and human health and its capacity to ensure sound stewardship was assessed. The aim was to identify the strengths and weaknesses and to propose recommendations for the improvement of antimicrobial stewardship policies in Cameroon.

In sum, the analysis methodology used consisted of three points:

1. Review of legal instruments;
2. The use of the MTaPS management policy analysis tool;
3. Conducting interviews and workshops with stakeholders from both sectors;
4. Literature review of relevant studies on antimicrobial drug use

4. MARKETING AUTHORIZATION, LICENSING AND INSPECTION

4.1. HUMAN HEALTH

4.1.1. Organization and Actors of the Pharmaceutical Sector

4.1.1.1. Organization of the Pharmaceutical Sector

Law 90/035 of 10 August 1990 to regulate the practice and organization of the profession of pharmacist in Cameroon provides that all practising pharmacists must be registered with the National Order of Pharmacists. Furthermore, the practice of pharmacy in private practice is subject to prior authorization issued by the Minister of Public Health (1). However, there are persons who practice pharmacy without holding a Doctor of Pharmacy degree. Cameroon has a National Pharmaceutical Policy (NPP), the latest version of which dates back to 2013, the aim of which is to improve access to quality health care by making available to the entire population, safe, effective, good quality pharmaceutical products at an affordable price, while ensuring their rational use in complete safety. However, the document does not provide guidance on the stewardship and regulation of antimicrobials.

4.1.1.2. 1.2 Players in the Pharmaceutical Sector

All these structures are regulated by the National Pharmaceutical Regulatory Authority (NPRA). Several entities of the MINSANTE, independent of each other, make up this ANRP, namely

- The Directorate of Pharmacy, Drugs and Laboratories (DPML);
- The General Inspectorate of Pharmaceutical Services and Laboratories (IGSPL);
- The National Laboratory of Quality Control of Medicines and Expertise (LANACOME);
- the Directorate of Operational Health Research (DROS).

The operation of these entities independently and the lack of collaboration between these actors is a major weakness for the health system in Cameroon, as it does not facilitate synergy of actions in the management of quality assurance of medicines.

The DPML (by Decree N°2013/093 of 03 April 2013 on the organization of the Ministry of Public Health) ensures, among other things, the approval, import and export control of pharmaceutical products, compliance with standards, procedures and Good Practices in the field, Pharmacovigilance, approval of pharmaceutical establishments... (2). In addition, the circular letter N°D36-27 of 02 October 2019

prohibits the direct import of pharmaceutical products by dispensing chemists, on pain of suspension of their accreditation (3).

The IGSPL ensures the Inspection of Pharmacy which is governed by articles 21 to 27 of the Law on the practice and organization of the profession of pharmacist (1). In addition, Decree No. 2013/093 of April 3, 2013 on the organization of the Ministry of Public Health sets out the missions of the IGSPL, which include the control of establishments manufacturing, storing and selling pharmaceutical products as well as medical analysis laboratories, the observation of breaches of professional rules in the practice of pharmacy and medical biology, including the illegal practice of these professions (2).

The DROS by Decree N°2013/093 of 03 April 2013 on the organization of the Ministry of Public Health ensures, among other things, the monitoring of studies in clinical research (2).

LANACOME, created by Decree No. 96/055 of March 12, 1996 and amended by Decree No. 2018/764 of December 11, 2018 on the reorganization of LANACOME, is the Government's body for drug quality analysis. Its main mission is the evaluation of the conformity of the medicine to the standards in this field (its composition, dosage, degree of purity, disintegration time, absorption, hardness, ...) (5).

The National Public Health Laboratory (LNSP) which is created and organized by Order N°2964/MINSANTE of October 09, 2013, also participates in the regulatory functions of the pharmaceutical sector and laboratories. Its main vocation is to assume the mission of regulation, coordination, quality control to monitor the national capacities of public and private laboratories (6).

All these entities contribute to the quality assurance of both drugs in general and antimicrobials in particular. However, there is no specific regulation for antimicrobial stewardship in Cameroon.

The pharmaceutical establishments are divided into two sectors, namely

- Public sector establishments: CENAME, FRPS (10), health facility pharmacies (FOSA) (more than 6000);
- Private sector establishments with the following sub-sectors
  - Private for-profit: manufacturing (05), pharmaceutical wholesale (41), private pharmacies (466)
  - The private non-profit sector (BNL): is made up of the central purchasing offices for pharmaceutical products that serve the health facilities of their respective networks (FALC, OCASC, CEPCA, RENAFSOM, CBC, CBHS)

Apart from public sector establishments, private sector establishments and establishments in the BNL are subject to approval and authorisation to open signed by the

Minister of Public Health. An application to practice is also required for pharmacists practicing in private hospitals, although this is not implemented.

Drug promotion agencies exist in Cameroon and contribute to making licensed drugs known to health professionals in order to ensure their availability on the national territory. Their opening is subject to obtaining a renewable license and their activities are limited to product promotion activities (10), (11), (12). However, despite the above provisions, there is a lack of regular monitoring of the quality control of medicines (13).

4.1.2. Marketing Authorization

Article 64 of the Act on the practice and organization of the profession of pharmacist provides that "no pharmaceutical speciality or specialized medicine may be sold or given away free of charge unless it has first been approved by the authority responsible for public health under the conditions laid down by regulation".
(1). Decree No. 98/405/PM of 22 October 1998, which sets out the procedures for the approval and marketing of pharmaceutical products, organizes the approval process and establishes a National Medicines Commission (CNM) (4).

However, some drugs in certain priority health programmes (e.g. Bedaquilline PNLT) are used even though they do not have a marketing authorisation. This situation occurs when new molecules that can treat diseases within the framework of these programmes are made available to the country. Because of their importance for patient care, these molecules benefit from an import exemption before the MA is issued.

In order to ensure that pharmaceutical products in circulation in the National Drug Supply System (SYNAME) remain in conformity with the specifications contained in their marketing authorization applications and are manufactured, stored, distributed and dispensed in compliance with the standards and good practices in force in this area, a draft text on the monitoring of the national pharmaceutical market is being prepared.

An analysis of the database of registered medicines in 2019, shows that 7649 medicines are registered in Cameroon of which 36% are antimicrobials (8). However, data on the number of International Nonproprietary Names registered in Cameroon are unavailable.

Antimicrobials (antibiotics, antivirals, antiparasitics and antifungals) are used on a large scale in the fight against infectious diseases in human or animal health. For a better management of infections, the Ministry of Public Health through the Directorate of Disease Control, Epidemics and Pandemics (DLMEP) has developed management algorithms

of certain priority diseases (HIV, TB, Malaria) and certain epidemic diseases (9).

In reviewing Cameroon's pharmaceutical regulations, we were unable to find the texts governing the prescription of antimicrobials in particular and drugs in general. In fact, the law on psychotropic drugs is the only legal instrument that mentions some provisions on the prescription of drugs in Cameroon. In the field, prescriptions are extended to any health professional trained or not on the use and prescription of antimicrobials, which constitutes a factor of increase in misuse and resistance to antimicrobials. Dispensing is well regulated by law 90/05 on the practice of the profession of pharmacist in Cameroon.

4.1.3. Pharmaceutical Inspection

The IGSPL routinely conducts inspections, but due to the lack of inspectors, financial, material and human resources, it is difficult to cover all the pharmaceutical establishments in the country on a regular basis. All of this hinders the evaluation of the implementation of Good Pharmaceutical Practices. On the basis of the IGSPL's inspection reports, several shortcomings were observed in the application of regulations, namely

- Absence of some regular pharmacists in the health facilities;
- Lack of pharmacist assistants in some pharmacies ;
- Pharmacy premises sometimes unsuitable (very narrow, lack of reception area, catering, etc.);
- Cold chain not adapted or temperature of conservation of medicines not respected (absence of air conditioning);
- Stock of medicines sometimes insufficient to cover the needs of the population;
- Presence of expired products on pharmacy shelves;
- Inappropriate storage of medications ;
- Storage of drug stocks sometimes inadequate (no pallets, no particular order) ;
- Supply of pharmacies by unlicensed distributors; Supply of

unlicensed persons by wholesalers ;

- Very low local production of antimicrobials (about 03 molecules) (7)

4.2. ANIMAL HEALTH

4.2.1. Bodies responsible for the management of veterinary medicines

4.2.1.1. National (Central) Regulatory Authority for Veterinary Drugs

4.2.1.1.1. Ministry of Livestock, Fisheries and Animal Industries

The Decree n°2012/382 of 14 September 2012 on the organization of the Ministry of Livestock, Fisheries, and Animal Industries places the exercise of veterinary pharmacy under the responsibility of the Directorate of Veterinary Services through a subdirectorate and specifies the responsibilities of the subdirectorate of veterinary pharmacy and private sector which is in charge of, among others (Art 58). :

- monitoring the implementation of the government policy on pharmacy and veterinary inputs;
- the development and monitoring of the implementation of pharmacy and veterinary input projects;
- development and control of the application of the regulations on pharmacy and veterinary inputs;
- the supply of technical equipment and medicines to the technical deconcentrated services;
- of follow-up of activities of pharmacies, of laboratories and of marketing of veterinary inputs, inspection ;
- the monitoring and control of the activities of veterinarians working in private practice;
- the dissemination of scientific and technical documentation on veterinary pharmacy and veterinary inputs;
- participation in the development of quality standards for veterinary inputs;
- the promotion of the veterinary private sector ;
- assistance to veterinarians in private practice;
- of the veterinary pharmaceutical inspection ;
- the examination of applications for approval, authorisation to set up and open veterinary pharmacies and pharmaceutical laboratories;
- the development and implementation of the health mandate.

In collaboration with the DSV, the Directorate of Pasture, Animal Feed and Livestock Infrastructure, which deals with feed manufacturing establishments, also plays a central role in the issue of the use of antimicrobials as growth promoters. In addition to the DSV, this collaboration in the area of compliance with regulations on the management of veterinary drugs (and antimicrobials in particular) extends to the Direction du dDéveloppement des productions et des

Industries Animales responsible for the elaboration and the control of the application of the regulation in the field of breeding and animal industries.

4.2.1.1.2. Other stakeholders

- The administration in charge of trade ;
- The administration in charge of customs ;
- Law enforcement and security agencies.
  They operate within the framework of the ad hoc committee for the coordination of operations to combat fraud, smuggling and counterfeiting, whose mission is to clean up commercial operations with a view to ensuring healthy competition in economic activities (Decree No. 2005/0528/PM of 15 February 2005)
- This committee is in particular responsible for (Art 2):

- to review the existing legal and regulatory framework with a view to proposing possible amendments;
- initiate administrative investigations ;
- to combat the importation, possession or sale of products derived from fraud, smuggling and counterfeiting;
- to develop and monitor the implementation of related prevention and enforcement programmes;
- to propose to the government, all measures likely to stop the illicit circuits of manufacture, penetration and sale of products in order to contribute to the guarantee of the tax and customs revenues concerned;
- to monitor the application of the penalties provided for by the texts in force concerning fraud, smuggling and counterfeiting of products;
- to centralize information and intelligence on illicit commercial practices.

This committee appears to be the keystone of the fight against the illicit circuit of sale of drugs in general and antimicrobials for veterinary use in particular.

4.2.1.2. Deconcentrated authorities (region, department, district) for the regulation of veterinary medicines

4.2.1.2.1. Deconcentrated services of the Ministry of Livestock, Fisheries and Animal Industries

At the deconcentrated level, and in terms of policy and regulation of veterinary antimicrobial management, the responsibilities of the different levels are clearly established in Decree No. 2012/382 of 14 September 2012 on the organization of the Ministry of Livestock, Fisheries and Animal Industries :

- The regional level is responsible for the centralization, consolidation and exploitation of information and statistical data from the departmental delegation and the monitoring of the implementation of activities that have benefited from a delegation of competences, the implementation of regulations and standards, the monitoring of the control and application of legislation and regulations, veterinary sanitary inspection and

the preparation of periodic activity reports (Art. 85). However, these are global missions that are much more implicit and make little specific reference to the management of veterinary drugs. It should be noted that the regional veterinary services are responsible for the supply of technical equipment, drugs and products for veterinary use, for monitoring the activities of private veterinarians and for supporting and supervising public and private veterinary laboratories.

- The departmental level is responsible for monitoring the implementation of activities that have been delegated to the regional level and any other mission previously mentioned. In addition, it ensures the compilation of the departmental file of organized groups of farmers, end users of antimicrobials, the follow-up of their activities and the distribution of technical equipment and drugs (Art 92).
- The district level is responsible, among other things, for monitoring the activities of private veterinarians and for the distribution of technical equipment and medicines (Art. 96).

4.2.1.2.2. Deconcentrated services of other sectoral administrations

The missions of the deconcentrated services of other administrations are not specific to antimicrobial management. However, under the Decree setting up the ad hoc committee, no mechanism for operationalization at the deconcentrated level is provided.

4.2.1.2.3. Decentralized territorial authorities (regions and municipalities)

Law n°2019/024 of 24 December 2019 on the general code of decentralised territorial authorities specifies that specific competences and means are transferred to decentralised territorial authorities (Art 5) in various sectors, notably that of health (Art 7). To this end, the territorial authorities exercise these powers exclusively or partially according to special dispensations (Art 18). In this respect, in the field of health and social action, the competence relating to the participation, organisation and management of the supply of medicines, reagents and essential devices in accordance with the national health policy is transferred to the regions. In this respect, it is clear from this relatively recent legal instrument that the regions and municipalities will play a central role in the management policies for both human and veterinary medicines.

4.2.2. Key players, roles and responsibilities in veterinary antimicrobial use

The distribution circuit of antimicrobials in Cameroon is identical to that of other veterinary drugs in general and is composed of an official circuit called

and an informal or parallel system (Figure 1). The flows between these two channels may intersect, reflecting the complexity of the use of veterinary drugs in general and antimicrobials in particular (Figure 1). Several actors, both public and private, with varying roles and responsibilities, are involved in the different distribution channels from importation of antimicrobials to their distribution and dispensing.
Indeed in Cameroon with the exception of some vaccines produced by LANAVET, all other veterinary drugs including antimicrobials marketed come from imports (Messomo, 2006, Mazra, 2014, Mouiche et al, 2018). Officially, in addition to the administrative and customs documents (import approval, patent...) required for importation, the latter is technically conditioned by the obligation of any importing structure to have a veterinary doctor (i) registered with the National Order of Veterinarians of Cameroon and
(ii) having an authorization for installation in private clientele. The distribution and dispensing of antimicrobials are also regulated.

4.2.2.1. International actors: FAO / OIE / WHO

These are international governance bodies, generally presented as bodies that regulate policy interactions between countries on antimicrobial stewardship. As such, they formulate a "set of relevant and connected rules or guidelines (formal or informal), which prescribe or direct the responsibilities and roles of national actors and their behaviours, limit activity and shape expectations" of sovereign states.

In the area of animal health, FAO assists member countries in developing and implementing their national action plans on antimicrobial resistance based on the global action plan on antimicrobial resistance.

The 4 pillars of the action plan are

(i) Improve AMR awareness ;
(ii) Develop AMR and AMU surveillance and monitoring capabilities;
(iii) strengthen governance ;
(iv) promote good practices in food and agricultural systems, including prudent and judicious use of antimicrobials.

The OIE has developed a procedure and data quality standards to collect information on the use of antimicrobial agents in food-producing animals from Member Countries on an annual basis to create a global OIE database (OIE, 2018) . A tripartite alliance FAO
/ The OIE/WHO Global Action Plan on Antibiotic Resistance was established with the objective of building the capacity of member countries to implement OIE and Codex Alimentarius standards and guidelines to combat antibiotic resistance and support the recommendations of the WHO Global Action Plan on Antibiotic Resistance (OIE, 2015b).

The Terrestrial and Aquatic Codes cover:

- Harmonisation of national antimicrobial resistance surveillance programmes ;
- Monitoring the quantities of antimicrobial agents used in animals and determining use patterns;
- Responsible and prudent use of antimicrobial agents in veterinary medicine ;
- Assessment of the risks of antimicrobial resistance (use of antimicrobial agents in animals) ;
- The OIE list of important antimicrobial agents in veterinary medicine to address human health concerns.

4.2.2.2. Foreign laboratories or pharmaceutical companies

Contrary to western countries where the pharmaceutical industry is strongly involved in the good management of antimicrobials and the fight against antimicrobial resistance, the involvement of the pharmaceutical industry is very little perceptible in Cameroon, its action being very often limited to commercial aspects or product promotion.

4.2.2.3. National actors

4.2.2.3.1. National public actors

4.2.2.3.1.1. MINEPIA

The missions of the agents of the State veterinary services on the management of antimicrobials for veterinary use are described by the regulations at both central and deconcentrated levels.

Some projects under the supervision of MINEPIA such as the livestock development project (PRODEL) are heavily involved in supporting the supply of veterinary medicalisation services through support for the installation of several veterinarians in private practice and have an antiparasitic and antimicrobial management plan (PG2A) (MINEPIA, 2016). We can also cite various supports provided by PRODEL to young veterinarians in the context of their installation in private practice. The subsidy consists of a stock of drugs, working equipment and operation). To date, 33 veterinarians have received support and are located both around the large cities (Douala, Yaoundé, Bafoussam) and in purely rural areas (Touboro, Moulvoudaye, Djiguilao, Batouri, Garoua Boulai, Meiganga, Mbalmayo, Nkongsamba, Foumban). The objective is to support 60 young veterinarians by 2022.

4.2.2.3.1.2. CVNO and ATASPAC

The National Order of Veterinarians of Cameroon (ONVC) established by Article 1 of Law n°78/21 of 29 December 1978, includes all veterinary doctors practising in Cameroon and has as its main attributions to ensure the maintenance of the principles of morality and devotion essential to the exercise of the veterinary profession as well as the respect of the rules enacted by the Code of Veterinary Ethics.

The Association of Aquaculture, Health, Fisheries and Animal Production Technicians of Cameroon (ATASPAC) is an association of veterinary paraprofessionals that collaborates with the ONVC and whose members work in veterinary pharmacies and clinics and therefore use antimicrobials in their daily practices (Collaboration Agreement between ONVC and ATASPAC).

4.2.2.3.1.3. CUSTOMS and MINCOMMERCE

Among its many missions, Cameroonian customs have the mission of supporting other administrations. Thus, due to its location at the borders, customs, better than other administrations, are well placed to apply specific regulations issued by other ministries: prohibitions on the entry into the national territory of certain products (war material, drugs, narcotics, pesticides …); control of import and export quotas; control of compliance with regulations on cross-border movements of certain classified products (eg. Protection of the environment, protection of intellectual property, etc.); collection of duties for the benefit of certain bodies (e.g. PAD, FEICOM, phytosanitary services, etc.).

At the local market level, MINCOMMERCE's missions include monitoring the application of legislation and regulations on trade, competition, prices, metrology, subsidized products, standards, quality and after-sales service (Article 58 of Decree No. 2012/513 of November 12, 2012 on the Organization of the Ministry of Trade).

4.2.2.3.1.4. LANACOME and LANAVET

The National Veterinary Laboratory (LANAVET) and the National Laboratory for Quality Control of Medicines **(**LANACOME) are the two laboratories approved by MINEPIA for quality control of veterinary medicines.

LANAVET is a Cameroonian state-owned company whose missions are:

- Production and sale of vaccines and medicines,
- Analysis of biological samples for diagnosis,
- Study of animal diseases,
- Animal disease surveillance,
- Training of senior managers and technicians,
- Scientific collaboration with national and international organizations.

LANACOME's mission is to ensure the quality control of medicines and other health products as defined by the legislation and regulations in force, and their compliance with the standards for import, distribution and export; it also ensures the control of everyday consumer products, in strict compliance with the public health imperative.

4.2.2.3.1.5. ANOR

The Agency for Standards and Quality (ANOR), is a Public Administrative Establishment whose mission is to contribute to the development and implementation of standardization and quality in Cameroon. The standards available in ANOR's 2015 catalogue of standards, relating to the use of antibiotics and their residues, are non-mandatory and correspond to those of the Codex Alimentarius (ANOR, 2013).

4.2.2.3.2. Community actors: regions and municipalities

The local public decentralized territorial authorities that are beneficiaries of the payment of the patent, even if they do not specifically control the sale of medicines, have an indirect role on the sale of the latter because they ensure the collection of this tax (Code Général des Impôts, 2019, Section VIII of the issuance of the patent).

4.2.2.3.3. Administrative authority and law enforcement agencies (FMO)

The administrative authority and law enforcement agencies are involved in the implementation of sanctions, particularly in cases of fraud or destruction of counterfeit veterinary medicines.

4.2.2.3.4. Private sector actors

4.2.2.3.4.1. Official circuit: Actors, roles and responsibilities

The official channel is the distribution channel for veterinary drugs authorised by the State. The main actors are importers-wholesalers-distributors, retailers-distributors, representatives of pharmaceutical firms, clinicians and groups of breeders. They are obliged to follow the various provisions imposed by the regulations in force.

Importers wholesalers-distributors

Throughout the country, 23 private veterinary structures ensure the import and distribution of veterinary drugs with a very uneven distribution. In the current scheme of distribution of veterinary drugs in the formal circuit, 100% of importing distributors dispense wholesale, semi-wholesale and retail. The capillary distribution at the level of livestock markets and the countryside is done by commercial agents of these same companies, creating unfair competition to the authorized retailers. Conversely, about 34% of retailers import their products directly. The importing companies are mainly

They are located in the port city of Douala, which facilitates the reception of ordered products. Most of them have branches in regions where livestock activities are important, especially in Adamaoua, the West, the North and the Far North. Some importers do distribution beyond national borders in countries such as Chad, Nigeria, Congo and Central African Republic (Mazra, 2014).

Pharmaceutical company representative

The pharmaceutical company representative is responsible for promoting the product of the company he represents. He is the spokesperson for the pharmaceutical company that employs him. He is the link between the laboratory and the importing wholesalers, retailers and veterinary practices. Although a veterinarian by training, he or she acts as a technical sales representative and must therefore sell all the services offered by the laboratory he or she represents. One of his missions is to inform and demonstrate the effectiveness of the drug to encourage the veterinary practitioner to prescribe it. He is also supposed to demonstrate the virtues of the drug, describe its composition, contraindications, side effects and instructions for use.

retailer-distributors

These are private veterinarians who retail veterinary drugs. They are mainly located in the cities of Douala, Yaoundé and Bafoussam.

Veterinary practices/clinics

In veterinary practices, veterinary medication is most often obtained by prescription from a veterinary doctor or his technician or by presenting a prescription. Here, the clientele is generally made up of pet owners. On the other hand, in veterinary pharmacies, wholesalers and breeders' groups, a prescription is not always required. The majority of the clientele concerned are breeders. In case of need, they ask the dispensary verbally. Once in the field, the farmer himself administers the medication to the animals. Some farmers turn to the parallel market where the prices of medicines are relatively affordable although their quality has not been formally investigated (Mazra, 2014).

Breeders' groups

In addition to veterinary practices and clinics, farmers' groups hold a stock of veterinary drugs for the needs of their members. The constraints related to the supply of veterinary drugs encountered by retailers are twofold: stock-outs and transport difficulties.

Pet owners

These are the end users of veterinary antimicrobials. They are not expected to own the veterinary drugs, let alone the antimicrobials.

4.2.2.3.4.2. Unofficial circuit: actors, roles and responsibilities

This is the parallel or illicit market for the distribution of antimicrobials for veterinary use. It is animated by several actors including non-professionals (without authorization or unauthorized persons) or authorized professionals who by their activities participate in the circuit of illicit sale of products. These are mainly illegal importers, small traders or street vendors and to a certain extent clinicians, paraprofessionals and animal husbandry auxiliaries, breeders, managers of feed mills. The mass of illicit veterinary products found on the Cameroonian market originates mainly from Nigeria and Asian countries (China, India and Pakistan) (Messomo, 2006, Mazra 2014). Importing wholesalers are increasingly being described as selling veterinary drugs (including antimicrobials) to this class of actors, making life even more difficult for retail veterinarians, who are generally capital-poor (Figure 1).

Illegal trader-importer

This refers to any natural or legal person who, in the course of his or her professional activities, purchases veterinary drugs without legal authorization and in violation of regulatory provisions, in order to offer them on the local market.

Street vendor, on the run (lawn)

These are salesmen of veterinary drugs who move from one place to another for their sale, while returning periodically to a fixed point or an unauthorized sale on the public highway of veterinary drugs that are generally of small volume and / or low weight, in order to escape the authorities by "saving" in case of control.

Other actors in the illicit circuit

These are feed manufacturers, livestock auxiliaries and to some extent veterinarians who use or sell veterinary drugs from the illicit circuit or are not allowed to hold or sell products from the official circuit.

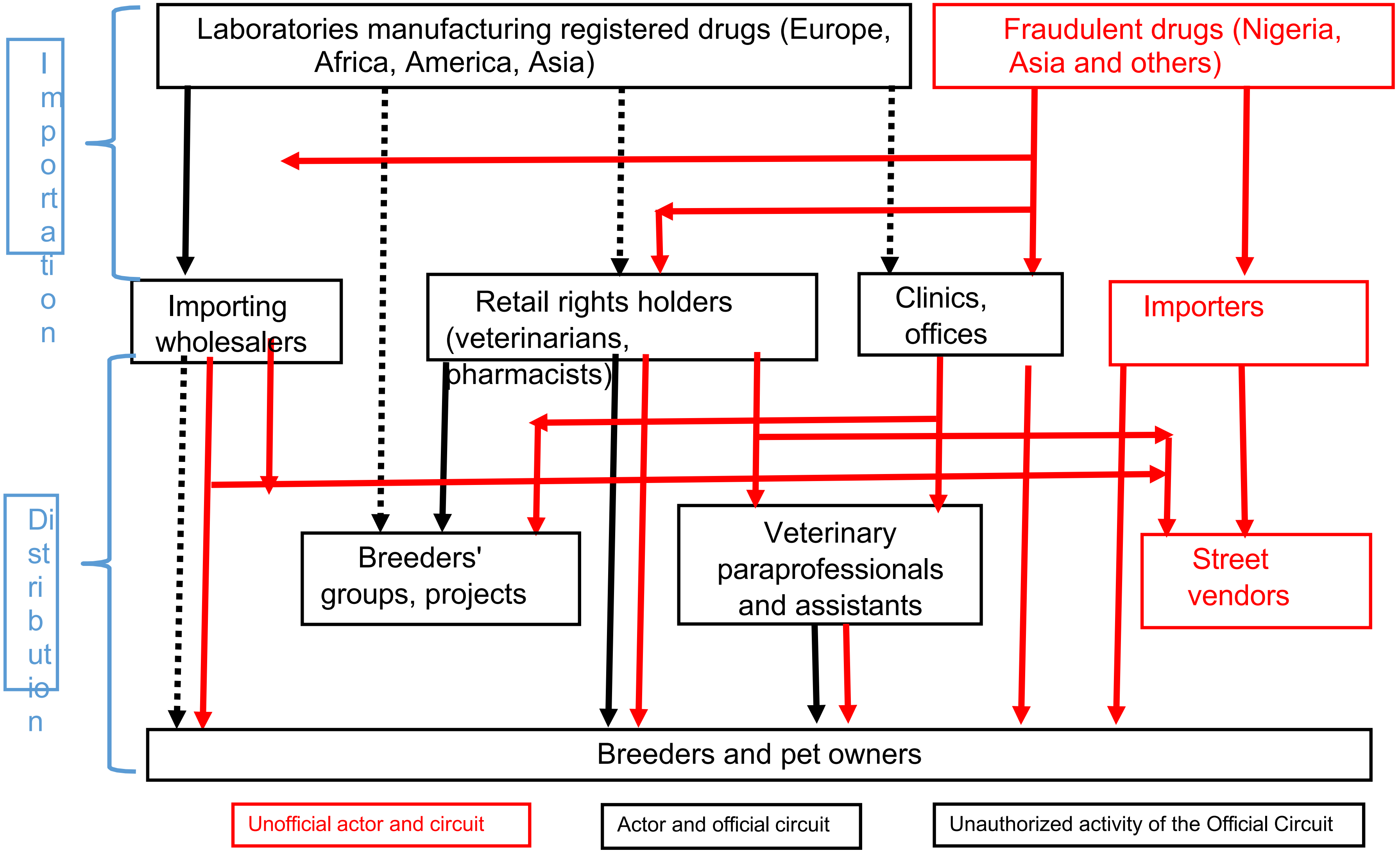

Figure 1: Complexity of veterinary drug(antimicrobial) distribution flows between different actors in the official (black) and unofficial (red) channels (adapted from Messomo (2006), Mazra (2014), Mouiche et al, (2018) and interviews with actors.

4.2.3. Marketing authorisation for veterinary medicinal products

4.2.3.1. Marketing authorisation requirement for veterinary medicinal products

Law n°2000/18 of 19 December 2000 on the regulation of veterinary pharmacy requires that a marketing authorisation be obtained, issued by the Minister in charge of veterinary services, before any veterinary drug is sold to the public (Art 3). The MA is issued for a renewable period of 5 years (Art 31).

- The MA is granted after an application addressed to the Minister in charge of veterinary services accompanied by the submission by the manufacturer or importer of an administrative and technical file (Art 4) whose composition is described by Order No. 178 CAB/PM of 5 December 2008 establishing, organisation and functioning of the commission in charge of examining the application files for marketing authorisation for veterinary medicines (except for the derogations provided for in article 18 concerning generic medicines, medicinal combinations, demonstration on the basis of scientific references of the harmlessness of the product)
) :

• documentation on the name of the medicinal product, the composition (in active substance and excipients), the pharmaceutical forms, the dosage by species, the withdrawal period (Art 12 and 13);

• documentation relating to non-clinical, pre-clinical and clinical trials, marketing authorisations obtained and decisions to refuse marketing authorisations, together with the reasons (Art 14 and 17);

• the documentation relating to the analytical tests of the veterinary medicinal products which are the subject of the marketing authorisation application, in particular the composition, description of the constituents, packaging, in-process control techniques for raw materials and stability tests (Art 15);

• documentation of safety testing and residue studies, including consumer toxicity, industrial relay or environmental toxicity risks (Art 16).

- In addition, the previous decree describes the financial conditions for granting MA (Chapter IV, Art 20 to 30), renewal, refusal (Art 35) and withdrawal or suspension of authorizations (Art 35). It should be noted that, with the exception of the financial provisions of the MA application file which differentiates the basic amount paid for the different classes of antimicrobials for veterinary use (Art 21)
The regulation concerns all veterinary medicines.

- This same law specifies the particular provisions requiring a waiver of the MA (Art. 3) in emergency or temporary situations and defines medicated feeds as any medicated mixture or premix presented for administration without processing to animals for preventive purposes. However, without explicitly mentioning antimicrobials as

The Act does not exclude the use of ingredients in medicated premixes or medicated feeds and implicitly authorizes their use by referring to the preventive use of such preparations. However, Order No. 13/MINEL of 13 October 1978 laying down the conditions for the opening of establishments for the manufacture or marketing of products intended for the feeding of domestic animals specifies, in addition to the conditions for the opening of such establishments, the packaging and labelling requirements, which must clearly indicate the name of the product, the brand name, the date of manufacture, the shelf life, the components, the net content by weight or volume of the foodstuffs and the address of the manufacturer (Art. 6). As such, any medicated food containing antimicrobials in their composition should bear this statement on the packaging. In addition, this decree provides that in case of fraud on the composition of feed intended for livestock that penalties be applied (Art 8). The previous Order, as amended and supplemented by Order No. 19/MINEL of 9 May 1979, specifies the composition of the file for the opening of such establishments (Article 2) and the requirement that they be subject to regular inspections, including the possibility of taking samples (Article 7). The conditions of application of these two important decrees are contained in the circular Note de service n°017/MINEL of 3 June 1980. Moreover, the decree n° 2008/2908 /PM of December 05, 2008 fixing the conditions of manufacture, packaging, importation, sale and distribution at the wholesale and retail level of veterinary drugs requires more information on medicated feed to the establishments authorized to import them and to the establishment authorized to distribute it (Art 19).

4.2.3.2. General measures relating to the definition of veterinary medicinal products, the regulation of their importation, manufacture, distribution, use and trade

- Law No. 90/033 of 10 August 1990 on the practice and organisation of the veterinary profession stipulates that veterinarians must comply with the regulations in force concerning the possession and management of veterinary drugs (Art. 19), defines the illegal or fraudulent exercise of the veterinary profession, in particular the facilitation of the exercise of the profession by unauthorised persons (in particular those engaged in the importation, storage, sale or distribution of veterinary drugs), thereby undermining the veterinarian's monopoly on the possession and transfer of veterinary drugs (Art 23).
- he law n°2000/18 of 19 December 2000 on the regulation of veterinary pharmacy defines the conditions for the practice of veterinary pharmacy and the manufacture, import, wholesale and retail distribution of veterinary drugs, the related offences and the penalties attached to them (Art 21, 23 and 25).

4.2.3.3. Raw materials for the preparation of veterinary medicinal products, quality standards for raw materials used in the manufacture and composition of veterinary medicinal products

The manufacturing process of veterinary drugs must be subject to compliance with the rules and quality standards determined by a specific text of the Minister in charge of veterinary services, in conjunction with the Minister in charge of standardization as stipulated by Decree No. 2008/2908 / PM of 5 December 2008 setting the conditions for the manufacture, packaging, import, sale and distribution at wholesale and retail level of veterinary drugs in its Article 26

4.2.3.4. Withdrawal times and maximum residue limits

Order No. 178 CAB / PM of 5 December 2008 on the establishment, organization and operation of the commission responsible for examining applications for marketing authorization of veterinary drugs declines the obligations of the applicant for MA to provide all documentation relating to safety testing and residue studies (Art 14 and 16). The same decree specifies that the marketing authorisation for veterinary medicinal products is suspended for a period not exceeding one year by decision of the Minister in charge of veterinary services, when it appears that for medicinal products intended to be administered to animals producing food for human consumption, the waiting time indicated is insufficient to ensure that the foodstuff from the treated animal does not contain residues that could present a danger to the health of consumers (Art 36). To this end, the provisions of Law No. 2018/020 of 11 December 2018 on the framework law on food safety sets the principles and regulatory bases relating to human food (including antimicrobial residues) to animal feed for human consumption. In addition, the Agency for Standards and Quality (ANOR) has in its catalogue of standards 2015, non-mandatory normative provisions that are related to the use of antibiotics and their residues, corresponding to those of the Codex Alimentarius.

4.2.3.5. Requirements for substances in antimicrobials that may interfere with veterinary controls

The administrative and technical file of the application for marketing authorisation must include information on the quantitative and qualitative composition of all the components of the veterinary medicinal product in usual terms and with the international non-proprietary name recommended by the World Organisation for Animal Health, if such a name exists (Art 12 of the aforementioned order).

5. POST-MARKETING SURVEILLANCE AND PHARMACOVIGILANCE

5.1. HUMAN HEALTH

At the level of the DPML, post-marketing surveillance is done through the granting of technical import visas prior to any import of pharmaceutical products. Documentary verification: invoices, GMP certificate, approval, etc. As part of the monitoring and evaluation of suppliers, the DPML carries out evaluations of good distribution and manufacturing practices during the administrative procedures for renewing approvals and certificates of good practice, but there is no data on indicators for monitoring their performance.

The regulatory texts on pharmacovigilance are currently being validated. However, a circular letter relating to pharmacovigilance guidelines has been drawn up to encourage the reporting of adverse events [14, 15]. It should be noted that therapeutic failures, ineffectiveness of drugs, and drug resistance must be reported. Under-reporting remains a problem due to insufficient information, awareness and training of health care personnel. In sum, the national system for monitoring adverse effects (pharmacovigilance) is still in its infancy in Cameroon. Nevertheless, during the last twelve months, data collection on adverse reactions and sharing of alerts have been carried out. To our knowledge, kanamycin in particular has been reported for ototoxicity effects observed in patients suffering from multi-resistant tuberculosis. The reports of this pharmacovigilance activity are not available, but we note the publication of the first (and only to date) pharmacovigilance bulletin which appeared in September 2019 (9). In particular, it mentions the alert on the use of dolutegravir in pregnant women and the recall of falsified batches of an amoxicillin-clavulanic acid.

At import points and wholesale/retail pharmacies, the IGSPL sometimes takes samples which are sent to LANACOME for control. In addition, documentary inspections are carried out at border health posts before any products enter Cameroon. However, this activity is biased because the inspectors do not have authorization to visually inspect the products. If the antimicrobial use policy is taken into account by public sector actors, the private sector is not left out.

Indeed, the implementation of Performance Based Funding (PBF), which represents an effective approach to strengthen the

The IGSPL, in its supervision guides for public and private health facilities, provides guidelines for the use of medicines [9,10]. During inspections, the IGSPL sometimes takes samples that are sent to LANACOME for controls that are carried out according to technical availability. As this activity is irregular due to a lack of financial, material and human resources, the production of data on the consumption of AM, both in value and quantity, is non-existent. However, it is noted that a first report prepared for this purpose is awaiting publication.

At the level of CENAME, the drugs acquired are part of the NEML (National List of Essential Drugs) including antimicrobials, except for some special exemptions related to new therapeutic directives. CENAME, which is the procurement body, proceeds by national or international call for tenders for the acquisition of pharmaceutical products [7]. With regard to the pre-qualification of suppliers, the public procurement code in force only provides for this provision for contracts for intellectual services [8]. 8] However, in the context of the supply of certain drugs for priority health programs, the invitation to tender is restricted to manufacturers prequalified by the WHO (World Health Organization).

The technical clauses of the invitations to tender stipulate that the suppliers selected by CENAME must comply with national pharmaceutical regulations. To this end, they have a period of three (3) months in which to submit the registration files for their products to the DPML. As for pharmaceutical manufacturers, they are required to include in their bids additional quality guarantees in the form of certificates of good manufacturing practices with ISO 15378 certification.

In terms of normative requirements on quality, the tender documents for medicines issued by CENAME refer to the American Pharmacopoeia (USP
The United States Pharmacopeia, the Japanese Pharmacopoeia and the European Pharmacopoeia are the three reference systems integrated in the international harmonization system of standards. Upon receipt of the products, CENAME visually inspects the products (organoleptic characteristics) and then takes random samples of batches which are sent to LANACOME for quality control.

At LANACOME, quality control of drug batches is carried out within the framework of inspections and imports by CENAME, health programmes and other approved distributors. It should be noted that given LANACOME's technical facilities, other foreign laboratories are called upon, in particular LANSPEX (Laboratoire National de Santé Publique et d'Expertise) in Niger and the CHMP (Centrale Humanitaire Médico Pharmaceutique) in France. With regard to ARVs (Antiretrovirals) and

Quality control of tuberculosis drugs must be carried out in WHO prequalified laboratories.

In spite of all the above-mentioned measures to guarantee the quality of medicines marketed in Cameroon, there is no report that makes it possible to assess the proportion of poor quality AM. However, alerts generally coming from the WHO are echoed at the national level by the Ministry of Public Health through the issuance of administrative documents such as circulars, press releases. Also, the IGSPL 2018-2019 activity report reveals that 15 batches of adulterated antimicrobials have been reported worldwide [11]. Furthermore, Cameroon has no independent national drug information service to date. In the past, there was a Centre d'Information Médico Pharmaceutiques (CIMEP), supervised by the National Order of Pharmacists of Cameroon (ONPC) whose role was to promote the rational use of medicines which is now obsolete.

5.2. ANIMAL HEALTH

5.2.1. Quality of veterinary medicines

5.2.1.1. Verification of the characteristics announced by the manufacturer

Any applicant for marketing authorization of veterinary drugs is required to attach to its application samples of the said product for analysis by approved laboratories as prescribed by Article 19 of Order No. 178 CAB / PM of December 5, 2008 on the establishment, organization and operation of the commission responsible for reviewing applications for marketing authorization of veterinary drugs. The objective is to verify the conformity of the characteristics announced by the applicant for the MA.

In addition, as part of the control and inspection of the veterinary establishment, the inspector or the territorially competent sworn controller is authorised to take samples to ensure the compliance of veterinary drugs (Decree No. 2008/2908 / PM of 5 December 2008 setting the conditions for the manufacture, packaging, import, sale and distribution of veterinary drugs at wholesale and retail level, Art 50). However, the modalities of this quality control are not described by the regulation.

5.2.1.2. Conditions for carrying out the tests and qualification of the experts involved in the tests

The regulations stipulate that the tests be carried out in laboratories approved by the Ministry of Veterinary Services. However, the conditions for carrying out the tests are not explicitly described, nor are the qualifications of the experts involved in the tests. However, the qualifications of the experts who are members of the committee responsible for examining applications for authorisation to market a product are not explicitly described.

on the market of veterinary drugs is described (Decree No. 2008/2908 / PM of 05 December 2008 setting the conditions of manufacture, packaging, import, sale and distribution at wholesale and retail of veterinary drugs, Art 3).

5.2.2. Notification of side effects to the competent authority

There is no regulatory requirement for adverse event reporting related to the use of veterinary drugs in general and antimicrobials in particular.

5.2.3. Implementation of mechanisms for product traceability, withdrawal or recall

The traceability mechanisms are not clearly established by the regulations. However, it should be noted that in the event of seizure or withdrawal requiring the destruction of veterinary drugs, the regulations provide for destruction procedures through Article 52 of Decree No. 2008/2908 / PM of 5 December 2008 setting the conditions for the manufacture, packaging, import, sale and distribution at wholesale and retail of veterinary drugs and Law No. 96/12 of 5 August 1996 on the framework law on environmental management.

5.2.4. Drug Use and AMR Policies/Regulations

Although several texts related to drug use exist, no formal provisions specifically related to the term antimicrobial resistance were encountered. Furthermore, most of the texts precede the development of the WHO/OIE/FAO tripartite guideline on regulation as presented in the global action plan to combat AMR. This remains furthermore true for the specific regulatory provisions for antimicrobials (Nouedjalié, 2018).

6. PROCUREMENT POLICIES AND GUIDELINES FOR ANTIMICROBIAL STEWARDSHIP

6.1. HUMAN HEALTH

1. Procurement policy

In Cameroon, the drug supply policy in general and antimicrobials in particular defined in the NPP is implemented through the National Drug Supply System (SYNAME). This system is made up of a set of actors who contribute to the quality assurance of medicines made available to the population. Antimicrobials, like all other pharmaceutical products, are imported after authorization (MA, technical visa, waivers, etc.) issued by the Ministry of Public Health to CENAME for the public sector and to approved wholesale distributors and religious purchasing centres for the private sector. These establishments ensure, in accordance with good practices, the distribution of imported products to dispensing structures. However, the implementation of the supply policy faces many difficulties:

- **At the level of CENAME**
  - Frequent shortages of antimicrobials not sufficient to meet public sector needs
  - The lack of systematic quality control of imported products

- **At the FRPS level**
  - Non-compliance with SYNAME
  - Frequent antimicrobial shortages
  - Procurement from private suppliers instead of CENAME
  - The lack of systematic quality control of imported products
  - Delivery of certain antimicrobials to health facilities not authorized to use them
  - Poor storage and preservation conditions

- **At the level of health facilities**
  - Frequent antimicrobial shortages
  - Procurement from unapproved suppliers
  - Poor antimicrobial prescribing practices
  - Poor antimicrobial dispensing practices
  - Absence or non-functioning of therapeutic committees
  - The absence of therapeutic formularies
  - Absence of pharmacists in some health facilities

- **At the level of pharmaceutical wholesale distribution establishments**
    - Poor distribution and storage practices
    - The lack of coverage of the NML
    - The lack of systematic quality control
    - Distribution of unauthorized antimicrobials
    - Frequent antimicrobial shortages
    - Delivery of products to unauthorized persons and structures

- **At the pharmacy level**
    - Dispensing antimicrobials without a prescription
    - High cost andnon-retail availability of antimicrobials leading households to seek supplies from the informal sector
    - Dispensing unauthorized antimicrobials
    - Unauthorized importation of antimicrobials
    - Dispensing of antimicrobials by non-pharmacist personnel
    - Lack of health care facilities and pharmacies in some localities

- **At the patient level**
    - Self-medication
    - Non-compliance with treatment

In addition to the formal sector described by SYNAME, there is an informal sector fuelled by the illicit trafficking of pharmaceutical products. Many households obtain their supplies through this sector, which exposes them to the use of falsified, under-dosed antimicrobials, without active ingredients and of poor quality.

Table 1: Description of formal sector actors and stakeholders

**SECTORS**<br><br>**LEVELS**	**FORMAL SECTOR**			
	**PUBLIC**	**DEVELOPMENT PARTNERS**	**PRIVATE CONFESSIONNEL**	**PRIVATE LUCRATIVE**
**Central**	- **MINSANTE**<br>**IGSPL**<br>DPML<br>CENAME<br>LANACOME<br>Pasteur Center	- **BILATERAL DONORS**<br>AFD<br>GIZ<br>Jura Susse<br>KFW	- **Health Services**<br>Ad Lucem Foundation (FALC);<br>Catholic Organization	- Private **wholesalers of** medicines and laboratory reagents<br>- Laboratorys

	Health programs<br>- HG<br>HC<br>- MINRESI<br>IMPM	- **MULTILATERAL DONORS**<br>BM<br>GAVI Global Fund<br>GDF<br>WHO<br>E U<br>UNFPA<br>UNICEF<br>UNITAID<br>USAID<br>- **NATIONAL AND INTERNATIONAL NGOS**<br>ACMS<br>Care International<br>Clinton Foundation<br>Johnson & Johnson Foundation & Johnson<br>Plan International,<br>etc.	e pour la Santé au Cameroun (OCSAC) ;<br>Supreme Islamic Council of Cameroon (SICC) ;<br>Council of Protestant Churches of Cameroon (CEPCA) : CBC, EPC, EEC, EFLC, AE, EELC, EPA, NBC, PCC, UEBC, UECC.	medical analysis
**Intermediate**	- FRPS<br>- HR			- Drugstores<br>- Analytical laboratories medical
**Peripheral**	- Health trainings<br>HD<br>CMA & CSI		- Health trainings	- Some health facilities
**Common Others**	- Households	- Households	- Households	- Households

2. Guidelines for Antimicrobial Stewardship

Good antimicrobial stewardship relies on the constant availability of reliable data to enable better decision making to improve the performance of the antimicrobial supply system.

Cameroon has National Strategic Plans (NSP) to reduce morbidity and mortality related to diseases and thus minimize their socio-economic impact on the development of the country. However, NSPs for some priority diseases such as malaria, HIV, TB are available in Cameroon and the one for hepatitis is under development. In order to operationalize the activities of the said plans, it was necessary to develop the guidelines or management guides (PEC) for certain diseases. Indeed, these define the different national therapeutic protocols. We cite, among others: Malaria ECP Guide (2019), Childhood Diseases ECP Guide (IMCI), STI Syndromic ECP Guide, TB Transmission Prevention Guide in Fosa and Communities (2016), TB ECP Guide (2020), HIV/AIDS Prevention and ECP Guidelines, Disease ECP Guide (IMCI), National Cholera Prevention and Control Guidelines (2008). (1, 2, 3, 4, 5)

The development of these guidelines is coordinated by the Ministry of Public Health. It takes place in several phases:

- Review of the literature,
- Consultation with experts and clinicians, learned societies and communities
- Development of protocols
- Validation of protocols
- National adoption of protocols

Following the validation of the various national directives, a training of national trainers involving all regions of the country is generally organized, followed by the training of health care providers at all levels of the health pyramid. These trainings include public, private secular and religious structures. In practice, a minority of providers are effectively trained, and a low rate of return from training is observed. In addition, the dissemination of these NWPs to providers in the health facilities is low.

During the training of students in the health field (medicine, pharmacy, nursing, community health workers), the protocols taught are not based on NTPs. However, the academic practical training courses that are compulsory in the health facilities allow them to become familiar with these NWPs in order to complete their theoretical training. However, the appropriation of the NWPs by the students at the end of their academic course is not always optimal. Concerning the frequency of revision of the NWP, there is no defined periodicity. However, the frequency varies according to WHO recommendations, scientific evidence, national experiences such as the

resistance observed in the field. This is the example of the 2005 STI Syndromic ECP Guide which has never been revised until today and the document on national HIV/AIDS prevention and ECP guidelines which was revised in 2013 and in 2019.

The NWPs exist for the different levels of the health pyramid, and some are adapted according to the level. This is the example of the HIV treatment protocol containing second and third line ARVs which are not prescribed at the level of the IHC and MAC, but at the level of the referral hospitals. In fact, there is a system of referral and counter-referral, and the ECP is done according to the technical platform. However, some health facilities do not always respect the implementation of the PTN. This is particularly true for the national list of essential medicines (LNME) which defines, according to the levels of the pyramid, the antimicrobials that must be accessible to patients.

The availability of TTPs in the health facilities is generally evaluated during supervisions organized by the MINSANTE. Emphasis is placed on the presence of TTPs for the management of priority diseases. Likewise, monitoring of compliance with the NWPs is done through the supervisions. AWARE classification, which should be an integral part of the NWPs, is relatively new and has not yet been instituted or implemented in Cameroon. As for surveys to evaluate compliance with NWP prescriptions at the national level, they are almost non-existent. However, some work has been done by independent researchers on a small scale, but the data were not found during this situational analysis.

Although Cameroon has to date guidelines on the prescription of antimicrobials used for the management of certain diseases, such as priority diseases (HIV, TB, Malaria,...), it does not have a national guide to good antimicrobial prescribing practices, nor guidelines on the classification of antimicrobials and their level of prescription.

As explained above, some antimicrobials are prescribed according to the level of the health pyramid. Thus, certain molecules are reserved for specific cases in certain health facilities, depending on the technical platform and the providers. For example, ^{3rd} line antiretrovirals and antimicrobials used for the management of multi-resistant or ultra-resistant tuberculosis are only reserved for hospitals and referral centers.

The NML mentions the drugs (and therefore the antimicrobials) that should be available by level of the health pyramid, not the profile or type of health care providers who should use them. To our knowledge, for infectious diseases, only the National HIV Prescribing and Management Guidelines document(7) describes (within the framework of task delegation) the prescription of antiretroviral drugs by health care providers, and by communities according to the level of the health pyramid.

The percentage of drugs (in terms of their monetary value) used in the country is provided by the private sector, that made up of antimicrobials in each sector, manufactured locally, in proportion to those imported will be given in the 2018 antimicrobial consumption report which is still not available.

Indeed, strict surveillance systems of antimicrobial consumption in the country are essential to optimize control strategies. Monitoring of veterinary antimicrobial import data could be useful for sub-Saharan African countries to effectively quantify consumption and estimate trends in antimicrobial use. (10)

6.2. ANIMAL HEALTH

6.2.1. Good supply management policies at the level of establishments producing, storing or wholesaling

6.2.1.1. Registration and authorisation of all operators manufacturing, importing, storing, processing, wholesaling or transferring veterinary medicinal products

Decree No. 2008/2908 / PM of 5 December 2008 setting the conditions for the manufacture, packaging, import, sale and distribution at wholesale and retail of veterinary drugs specifies in its Article 5 on the procedures for administrative authorization to open a veterinary pharmaceutical establishment that the rightful owners of the manufacture, packaging, import and wholesale distribution of veterinary drugs as being companies or organizations with one or more veterinary pharmaceutical establishments governed by Chapter 1 of Title IV of Law No. 2000/018 of 19 December 2000, regulating the Veterinary Pharmacy instituting the central role played by the veterinarian, pharmacist who must be the owner or a company in whose management they participate in majority.

The sale and retail distribution of veterinary drugs is also subject to the competence of the veterinarian, either directly or indirectly through supervision, as in the case of their use by the approved auxiliaries of veterinarians or groups of breeders (Art. 17). Although the status of approved auxiliary as well as that of veterinary practitioner is not clearly defined by the regulations, the status of cooperative societies is attributed to groups of breeders when they comply with Decree No. 92/455/PM of 23 November 1992 laying down the modalities for the application of Act No. 92/006 of 14 August 1992 on cooperative societies and Common Initiative Groups.

6.2.1.2. Organization of the distribution circuit to ensure traceability, recall, withdrawal and proper use

Decree No. 2008/2908 / PM of 5 December 2008 setting the conditions for the manufacture, packaging, import, sale and distribution of veterinary drugs at wholesale and retail specifies that any establishment manufacturing, packaging, selling or distributing veterinary drugs at wholesale is excluded from the sale and retail distribution of veterinary drugs (Art 45) Furthermore, these establishments must be owned by a veterinarian, a pharmacist or a company in which veterinarians have a majority share in the management (Art. 12). These are regulatory provisions that organise the distribution circuit for veterinary medicinal products while avoiding unfair competition between wholesalers and retailers.

6.2.2. Definition of the responsibilities of the actors

The Law n°2000/018 of 19 December 2000, on the Regulation of Veterinary Pharmacy describes the responsibilities of the actors for the importation of veterinary drugs (including antimicrobials), as well as the manufacturing, sale and distribution establishments in wholesale (Art 11 and 12) and retail (Article 17). As described in the previous chapters, the administrative authorisation processes for opening a veterinary pharmaceutical establishment are specified by Decree No. 2008/2908 / PM of 5 December 2008 setting the conditions for the manufacture, packaging, importation, sale and distribution at wholesale and retail of veterinary drugs. The obligations and operation of a veterinary pharmaceutical establishment are described in section II of chapter II of the obligations and operation of a veterinary pharmaceutical establishment, emphasizing the central role of the veterinarian as owner, manager. In addition, the veterinary practitioner is subject to Decree No. 84/1053 of 18 August 1984 on the code of ethics for veterinarians (or any other code of ethics adopted by the General Assembly of the National Order of Veterinarians of Cameroon) which specifies the ethical conditions for the exercise of the veterinary profession as provided for by Law No. 90/033 of 10 August 1990 on the exercise and organisation of the veterinary profession;

6.2.3. Obligation (imposition) of rules of good practice specific to each activity

The regulation provides for sanctions for violations or non-compliance with the provisions of Law No. 2000/018 of 19 December 2000, regulating veterinary pharmacy, Decree No. 2008/2908 / PM of 5 December 2008 setting the conditions for manufacture, packaging, import, of sale and distribution at wholesale and retail of veterinary drugs, especially for manufacturers of veterinary drugs, wholesale importers, retailers, veterinary practitioners, including fines and imprisonment (Title V of the above law) to the suspension and withdrawal of the authorization to open (section III of the above decree).

7. PRESCRIBING, DISPENSING AND USE

7.1. HUMAN HEALTH

7.1.1. National List of Essential Medicines

Cameroon has a National Essential Drug List (NEDL) inspired by the WHO model list of essential drugs (EML); essential antimicrobials are defined according to the levels of the health pyramid. However, this provision is not always respected for several reasons, notably

- Unavailability of antimicrobials due to stock-outs ;
- The cost, because the financial means are not the same in all the health facilities;
- The problem of access to medicines due to the remoteness of certain health facilities, the poor state of the roads and certain dilapidated health facilities;
- Lack of follow-up;
- Lack of capacity building for providers ***(1).***

The selection process for antimicrobials to be included in the NML is coordinated by the DPML. The latter collects from the health facilities the proposals to add or remove therapeutic molecules useful for the management of common diseases and sends them to the specialized commission in charge of the selection of essential drugs. However, the data collection and analysis process differs from one health facility to another ***(2).***

Information collected from the MINSANTE in Cameroon indicates that the NEML is generally revised every two years. However, we have not been able to access a normative document that clearly defines this periodicity. Normally, this list should be revised taking into account new protocols and scientific evidence. Note that the latest version of the NEML is from 2017 although it has not been widely disseminated all along the health pyramid. ***(1)***

Speaking of infectious diseases, the NTPs for priority diseases such as: HIV; TB, malaria, NML do not yet take into account certain management molecules. This is notably the case for Tenofovir + Lamivudine + Dolutegravir which were added in the last revised national guidelines and validated in 2019; Streptomycin injection present in the 2017 NML was withdrawn from the validated 2020 NTP. artesunate + Pyronaridine does not appear in the NML. ***(1), (3)***

When the NML is validated and published, health care providers who have not all been updated to the latest version will tend not to comply with its use in their current practice. ***(1)*** In addition, there is no training curriculum on the NML in the various schools, universities and faculties

that produce health care providers. Infectious disease treatment education is generally based on regional and international recommendations and does not take into account the NML.

Data on the degree of availability of the NML in the health facilities exist partially. Indeed, only those of the tracer drugs and priority diseases are available in the DHIS2 through the RMAs.

Surveys on the availability of drugs (including antimicrobials) included in the NML at the facility level are rare. However, these data are available in supervision reports.

Antimicrobials are supposed to be dispensed by prescription only. But the reality on the ground is different because in our tropical context pharmacists often find themselves dispensing antimicrobials without a prescription. Moreover, there is no text specifying this. On the other hand, in the MAs, antimicrobials are drugs dispensed only on prescription ***(1).***

7.1.2. Appropriate use of antimicrobials

The effective fight against antimicrobial resistance requires the promotion of the rational use of antimicrobials among prescribers, dispensers, patients and the general public. Awareness campaigns on the appropriate use of antimicrobials are rare in Cameroon. However, it should be noted that sporadic campaigns are organized by the pharmacy services of some health facilities. In addition, it should be said that in 2018 the Ministry of Health participated in the celebration of the World Week for the Appropriate Use of Antibiotics by organizing exchange symposia bringing together health professionals, students and the public. In addition, awareness campaigns were also conducted in health districts in the city of Yaounde as part of the said week. This was the first celebration of its kind in Cameroon [2].

In 2019, OHCEA organized a training of actors on the proper use of antimicrobials. This training was divided into two phases, a theoretical phase relating to the teachings and a practical phase relating to the effective implementation of the acquired knowledge in their professional environment through, in particular, sensitization and the realization of certain surveys [3].

In the literature, we found two related studies. The first one dates back to 2003 and shows in its main results that 62.9% (variation between 26.7% and 84%) of prescriptions in Cameroon include at least one antibiotic [4]. The second study was conducted in 2015 in the city of Douala with the objective of evaluating the knowledge, attitudes and practices of antibiotic use in Cameroon. From this study, it emerges that the bad practices related to the use of antibiotics observed are inherent to: misuse, low level of knowledge of prescribers and dispensers, and the extent

of self-medication in Cameroonian society [5]. The main recommendation of this study emphasized the importance, and even the urgency, of developing a guide to good antibiotic prescribing practices for health professionals and conducting education campaigns on the rational use of these products at the community level [5].

Infection prevention information campaigns that emphasize hand and food hygiene are only seen during the management phases of epidemics such as cholera in some parts of the country.

In addition, Cameroon also has an Expanded Programme on Immunization that regularly conducts population immunization activities and a national malaria control programme that frequently sensitizes the population on vector control, notably by distributing LLINs, while carrying out a chemoprevention campaign for seasonal malaria in the northern regions during the rainy season. Due to their scarcity, we were unable to access data evaluating the impact of awareness campaigns on antimicrobial use practices. However, Cameroon conducted in 2019 the first study of antimicrobial consumption based on import and local manufacturing data that can serve as a basis for guiding or reorienting policies on the proper use of these products [6].

7.2. ANIMAL HEALTH

7.2.1. Regulation of appropriate use by prescribers, dispensers and end users

7.2.1.1. Prescribing and dispensing rules

Documentation on the name, composition and dosage for each animal species is required at the time of application for marketing authorization. In addition, law n°2000/18 of 19 December 2000 on the regulation of veterinary pharmacy defines the conditions for the practice of veterinary pharmacy and the manufacture, import, wholesale and retail distribution of veterinary drugs, and requires veterinarians to dispense veterinary drugs only on the basis of a prescription (art 21). This obligation to dispense veterinary drugs on the basis of a prescription was already established by Articles 7, 8 and 11 of Order No. 02/MINEL of 29 January 1980 defining the technical modalities for the marketing of products for veterinary use. This old text specifically targets products that can generate chemoresistance, antibiotics and other category 2 products (OHCEA, 2017).

The veterinarian's monopoly on the possession and sale of veterinary drugs and to auxiliaries and groups of breeders under the supervision of a practising veterinarian is established by Article 17 of the above-mentioned law to ensure

the quality and safety of veterinary drugs. Thus, this management system for veterinary drugs, in which the veterinarian is the hub, is reinforced by the provisions of Law 2000/017 of 19 December 2000 on the regulation of veterinary health inspection and Decree No. 86/711 of 14 June 1986 setting out the procedures for veterinary health inspection, which must be carried out by sworn inspectors from the Ministry in charge of veterinary services. The provisions of Decree No. 2008/2908/PM of 5 December 2008 setting the conditions for the manufacture, packaging, import, wholesale and retail sale of veterinary drugs, reaffirms the veterinary monopoly (Articles 13, 16 and 23) and makes the acquisition of veterinary drugs by the farmer subject to the presentation of a prescription (Article 45) while specifying much more specifically in Chapter V the conditions for control and inspection of veterinary establishments involved in the manufacture, import and distribution, as well as the legal prohibitions. The previous decree also provides that a list of medicines for which a prescription is required is established by decision of the Minister in charge of veterinary services (Art. 45). This last provision is not yet implemented (OHCEA, 2017).

7.2.1.2. Trade Restriction

The regulations are formal in terms of restricting the trade of veterinary drugs in general and antimicrobials in particular. The provisions of Decree No. 2008/2908/PM of December 5, 2008 setting the conditions for the manufacture, packaging, import, wholesale and retail sale of veterinary drugs reaffirms the prohibition on the sale of veterinary drugs by any unapproved person, and the conditions of approval (composition of the approval file) are set by a special text of the Minister in charge of veterinary services (Art 44).

7.2.1.3. Supervision of accredited organizations by a licensed professional

The control and inspection of veterinary establishments are carried out by professionals of the Ministry in charge of veterinary services, i.e. sworn officials with special competence who are veterinary doctors or pharmacists working for the said Ministry, as stipulated in Article 49 of Decree No. 2008/2908/PM of 5 December 2008 setting the conditions for the manufacture, packaging, import, wholesale and retail sale of veterinary drugs. The modalities of this control are also described by the same law (Art 50).

7.2.1.4. Regulation of all forms of advertising and other marketing and promotional activities

Although sanctions are provided for anyone who advertises veterinary drugs without complying with the legislation in force (Law 2000/017 of 19 December 2000 on the regulation of veterinary health inspection, Art 21), there is a legal vacuum for the regulation of promotion and marketing activities.

advertising of veterinary drugs in general and antimicrobials in Cameroon.

7.2.2. Antimicrobial use in animal health in Cameroon

Analysis of antimicrobial use is carried out at different levels including:

- At the national level ;
- In livestock production units for production animals ;
- In pets, in veterinary clinics in Cameroon ;

7.2.2.1. At the national level

Nationally, the consumption of antibiotics in animal health was estimated at 217.67 tons (by weight of active substance) in a 6-year period (2014-2019), with an average of 36.28 ± 10.11 tons per year. The most used therapeutic classes were: tetracyclines 31.71% (69.02 tons), sulfonamides 23.84% (51.90 tons), quinolones 11.11% (24.18 tons) and beta-lactams 10.17% (22.13 tons) (Mouiche et al, 2020). Adjusted to the biomass by animal species, this represented a consumption of 213.32±50.26mg/kg in chicken production, 63.04±18.87mg/kg in pig production, 4.11±2.20mg/kg in cattle, 0.83±0.43mg/kg in sheep, 0.47±0.24mg/kg in goats (Mouiche et al, 2020).

Enrofloxacin (5.94%) and norfloxacin (3.80%) were the most consumed quinolones. Regarding tetracyclines, beta-lactams and sulfonamides, oxytetracycline (25.37%), penicillin (5.62%) and sulfadimidine (11.53%) were the most used molecules. Powders accounted for 60% of the total amount of active substance used during the study period.

Regarding the importance of veterinary antimicrobials for human medicine, 34.3% of the total imported antimicrobials were of critical importance while 4.6% and 25.5% were in the Reserve and Watch groups respectively classified by the WHO AWaRe categorization (Mouiche et al, 2020).

7.2.2.2. In livestock production units for production animals

7.2.2.2.1. Prescription, administration

In layers, the main route of administration of antimicrobial drugs was oral (99%). The main indications for antimicrobial use were metaphylactic (58.1%) and prophylactic (41.9%) treatment. Metaphylactic treatment was for respiratory (33.4%) and digestive (24.7%) tract symptoms. The eight (08) most used antimicrobial families were: tetracycline (43.9%), sulfonamides (26%), quinolones (12%), β-lactam (7%), polypeptides (4.3%), nitrofurans (2%),
aminoglycosides (1.3% use) (Table 2). Overall, 78.2% of the antimicrobials sold in pharmacies and 67.3% used on farms belonged to the class of antimicrobials of critical importance to human health according to the joint FAO/WHO/OIE classification. The use of antimicrobials (doxycycline, sulfonamide, ampicillin, streptomycin) that should not be administered to poultry whose eggs are intended for human consumption was observed (Woumbe, 2013). The most used antimicrobial compounds were oxytetracycline followed by colistin and sulfonamide-trimethoprim, while the least used were procaine benzylpenicillin followed by spiramycin-trimethoprim and tetracycline. The UDD/ADD ratio assesses the accuracy of dosing. The aminoglycoside (100%), macrolide (90.6%) and tetracycline (74.5%) families were the most underdosed. Trimethoprim sulfonamide (45.8%) and β-lactams (35.7%) were overdosed (Table 3). Thus, the inappropriate use of antimicrobials in laying hen farms should be highlighted. It is important to strengthen the regulation of antimicrobial use and to train poultry farmers in good practices of antimicrobial use in order to preserve their effectiveness in human and animal health.

Table 2: Antibiotic consumption (quantity) and number of prescriptions in laying hen farms in the Western region (Cameroon) between January and October 2012 (Woumbe, 2013).

Active substance	Quantity (Kg)	%	Number of prescription	%
Aminoglycosides	3.9	1.3	63	9.3
Sulphonamides - Trimethoprim	78.6	26.4	96	14.2
Macrolides	9.1	3.1	32	4.7
Nitrofurans	6	2.0	14	2.1
Polymyxins	12.8	4.3	130	19.2
Quinolones	35.7	12.0	83	12.3
Tetracyclines	130.6	43.9	231	34.1
β-lactams	20.7	7.0	28	4.1
Total	297.4	100.0	677.0	100

A total of 677 treatments (297 kg of active ingredient) were included in the analysis. *Prescription of antimicrobial combinations of different classes is considered as one prescription for each ingredient in the combination.*

Table 3: Dosing accuracy (UDD/ADD ratio) according to the different families of antibiotics administered and the different indications of use in laying hen farms in the Western region (Cameroon) between January and October 2012 (Woumbe, 2013).

		Correct dosage (%) *0.8<UDD/ADD >1.2*	Underdose (%) *UDD/ADD <0.8*	Overdosing (%) *UDD/ADD >1.2*
Antibiotic families	Aminoglycosides		63 (100)	
	Sulphonamide- Trimethoprim	14 (14.6)	38 (39.6)	44(45.8)
	Macrolides	1 (3.1)	29 (90.6)	2 (6.3)
	Nitrofurans	7 (50)	5 (35.7)	2 (14.3)
	Polymyxins	13 (10)	82 (63.1)	35 (26.9)
	Quinolones	14 (16.9)	53 (63.9)	16 (19.3)
	Tetracyclines	13 (5.6)	172 (74.5)	46 (19.9
	β-lactams		18 (64.3	10 (35.7)
Indications	Prophylactics	15 (4.5)	295 (89.4)	20 (6.1%)
	Digestive Symptoms	20 (14.4)	70 (50.4%)	49 (35.3)
	Respiratory symptoms	27 (12.9)	95 (45.5)	87 (41.6)
	Total	62 (9.3)	460 (68.9)	155 (23.2)

A total of 677 treatments (297 kg of active ingredient) were included in the analysis. *Prescription of combination antimicrobials of different classes is considered to be one prescription for each ingredient in the combination*

7.2.2.2.2. Residuals and Waiting Times

In a study on the presence of antimicrobial residues in DAOAH it was determined an overall prevalence rate of 58.23%: beef (70.55%), eggs (17.39%) and milk (69.63%) (Okah-Nnane, 2016). From a quantitative point of view, average residue concentrations in beef reach 17.58 µg / kg for penicillin G and 240 µg / kg for oxytetracycline (Vougat, 2017), which corroborates the results of other studies on milk (Edima et *al.*, 2012) and on eggs (Wafo, 2014). This shows the need to raise awareness among farmers and stakeholders in the animal sectors on biosecurity and the presence of residues in DAOAHs but also the respect of withdrawal periods. Strict regulation of veterinary drugs is important to ensure food safety and effective use.

7.2.2.3. In pets and veterinary clinics in Cameroon

When providing antibiotic therapy to pets, the choice of antibiotic to use is usually based on clinical diagnosis (in 90% of cases), while

Bacterial culture (antibiogram) is rarely used. Other criteria such as ease of administration are taken into account but to a lesser extent (Figure 2).

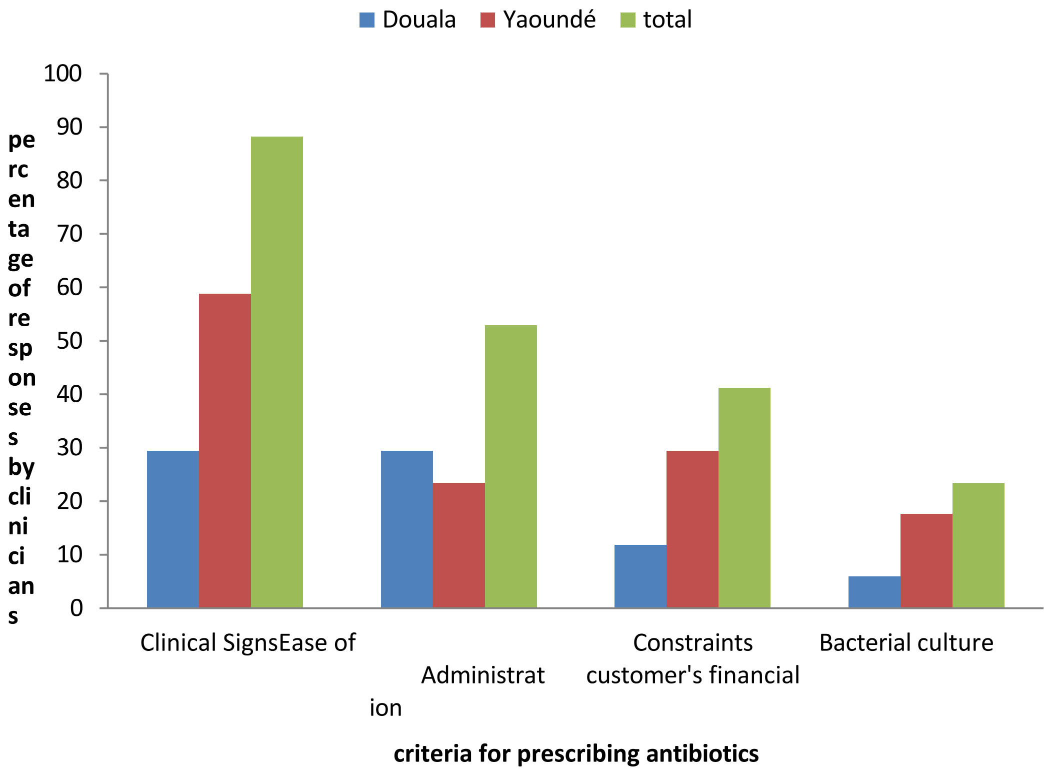

Figure 2: Criteria for prescribing antibiotics (Nno Nkassa, 2016).

Between 2012 and 2015, a total of 10 families of antibiotics were used in the clinics, with an average of 5 to 8 per year. The most used of these in terms of variable frequencies are aminoglycosides, quinolones, sulfonamides, tetracyclines and combinations; the least used being macrolides and related (just in 2014), ansamycins (2013 and 2014) and beta lactams (2013, 2014 and
2015) (Figure 3, Nno Nkassa, 2016).

It was shown that regardless of the number of years of experience of the veterinarian, quinolones (enrofloxacin and marbofloxacin) are used as first line rather than second line in 88.3% of cases. The most commonly used antibiotics during the study period (2012-2015) were sulfonamides including sulfamethoxypyridazine-trimethoprim (31%), benzylpenicillin- streptomycin combination (19.1%) and quinolones including marbofloxacin (18.1%) (Figure 4).

For the various conditions treated, the antibiotics used were within the recommendation range of the Guide to Antibiotic Therapy for Domestic Carnivores. Antibiotics critical to human medicine were used at a high percentage (42%) (Table 4). It appears that there is a use of antibiotics that does not comply with OIE/FAO/WHO recommendations, which poses a sanitary risk for both animal and human health, reinforcing the need to implement rigorous surveillance measures to avoid the misuse of antibiotics that can lead to the development of AMR.

Table 4: Prescription frequency of veterinary antibiotics of human health importance (Nno Nkassa, 2016)

**Level of importance antibiotics**	**Distribution by city** n (%)		**Total**
	Douala	Yaoundé	
Review	423(25,6)	1232(74,4)	1655(42,0)
Very important	280(12,5)	1964(87,5)	2244(57,0)
Important	27(67,5)	13(32,5)	40(1,0)
p	< 0,05	< 0,05	< 0,05

n: number of times the specific grade antibiotic was used; p: P value

7.2.3. Adverse effects and (in)efficacy of veterinary antimicrobials

Adverse effects and/or lack of efficacy of veterinary drugs have been suspected in animals. In addition, antimicrobial-related adverse reactions in humans were reported in Cameroon. Antiparasitics were the most incriminated therapeutic class in animals (61%) and humans (56%) and adverse reactions were reported in dogs (44%) and poultry (24%), while drug ineffectiveness was most observed in poultry (47%), reinforcing the need for the establishment of a national veterinary pharmacovigilance system based on solid legal foundations for a continuous evaluation of the risk-benefit effects of veterinary drugs marketed in Cameroon (Mouiche et al., 2019).

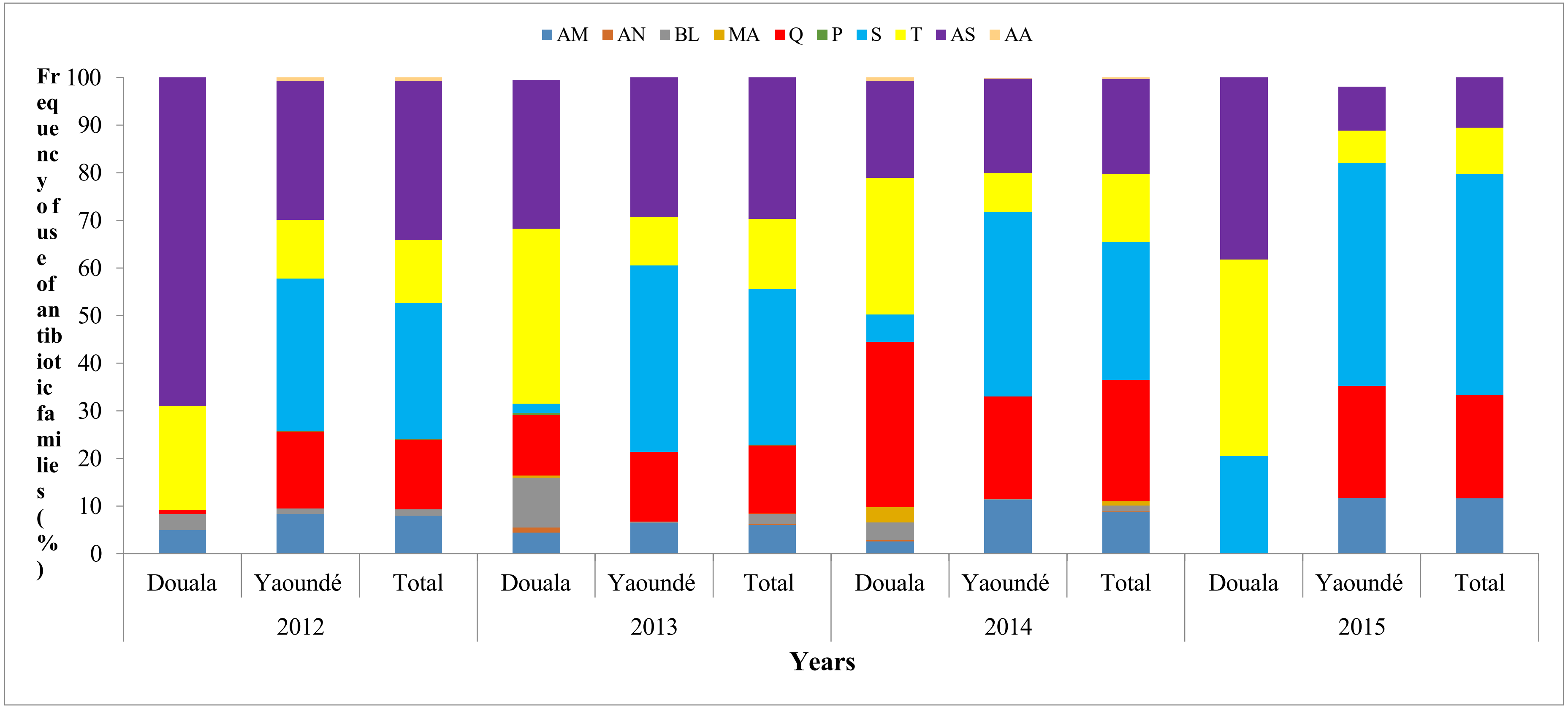

Figure 3: Annual evolution of the frequency of use of antibiotic families (Nno Nkassa, 2016).

AM: aminoglycosides, AN: ansamycins, BL: beta lactam antibiotics, MA: macrolides and related, Q: quinolones, P: polypeptides, S: sulfonamides, T: tetracyclines, AS: combinations, AA: antifungal antibiotics.

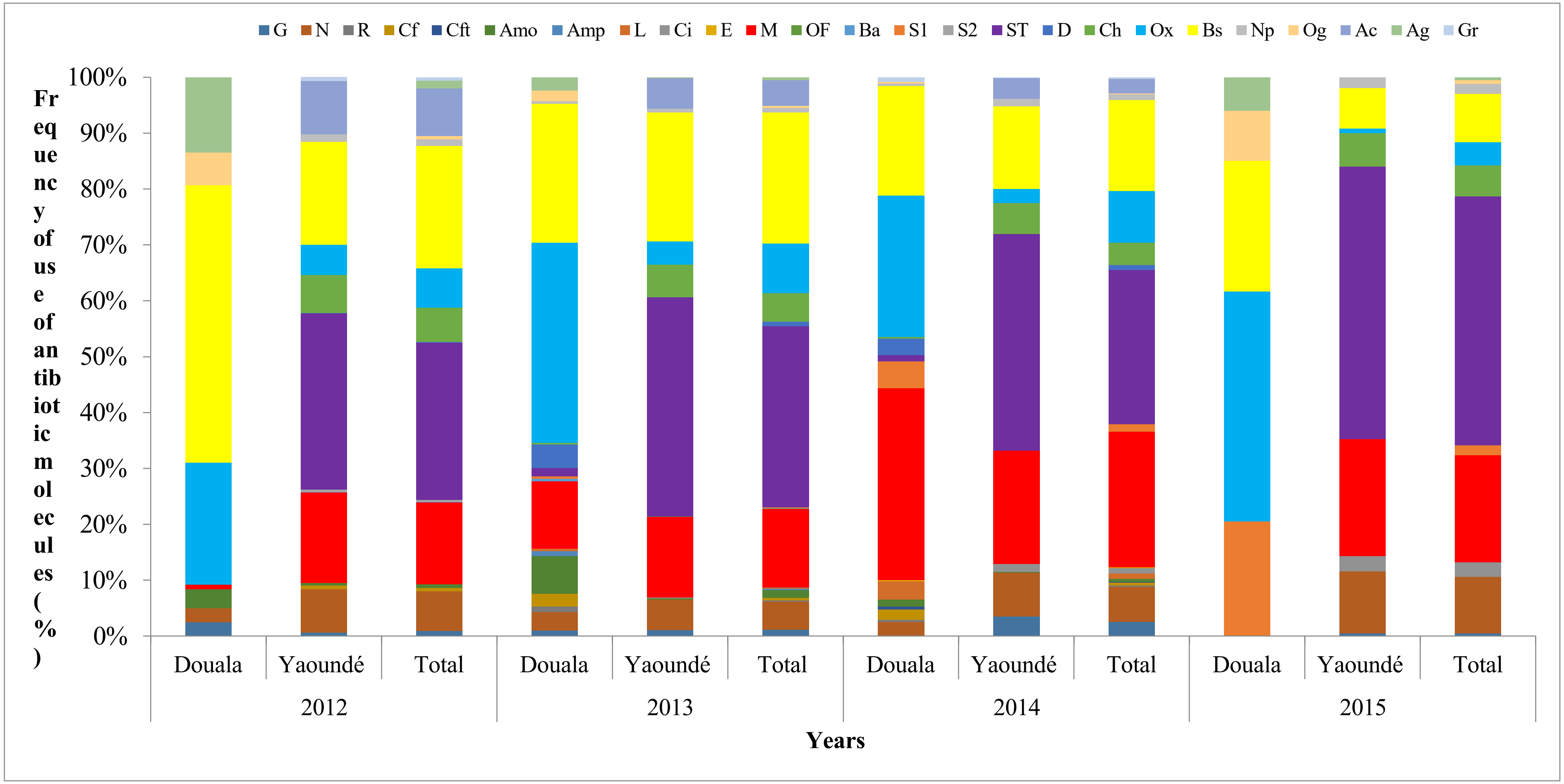

Figure 4: Annual evolution of the use of antibiotic molecules (Nno Nkassa, 2016)

G: gentamycin, N: neomycin, R: rifamycin, C: cefalexin, Cft: ceftriaxone, Amo: amoxycillin, Amp: ampicillin, L: lincomycin, Ci: ciprofloxacin, E: enrofloxacin, M: marbofloxacin, Of: ofloxacin, Ba: bacitracin, S1: sulphadimerazine, S2: sulphaguanidine, ST: sulfamethoxypyrydazine-trimethoprim, D: doxycycline, Ch: chlortetracycline, Ox: oxytetracycline, BS: benzylpenicillin-streptomycin, NP: neomycin-polymyxin, OG: oxytetracycline-gentamycin, AC: ampicillin-colistin, AG: amoxycillin-gentamycin, Gr: griseofulvin.

8. ANALYSIS OF STRENGTHS, WEAKNESSES, OPPORTUNITIES AND THREATS

8.1. HUMAN HEALTH

PHARMACEUTICAL POLICIES	
**FORCES**	**WEAKNESSES**
- Existence of a PPN<br>- Existence of laws and regulations governing the practice of pharmacy, licensing, distribution and promotion of medicines<br>- All medicines in the legal circuit have a marketing authorisation at<br>- Existence of a National Pharmaceutical Regulatory Authority<br>- carrying out inspections by the IGSPL<br>- 2018 Antimicrobial Consumption Report in Cameroon	- The NPP does not provide guidance on the proper management and regulation of drugs.<br>- Lack of guidelines and texts for prescribing and dispensing antimicrobials in particular and medicines in general.<br>- Balkanization and weak coordination between the different actors constituting the National Pharmaceutical Regulatory Authority.<br>- Dislocated operation of the NPCA<br>- Insufficient human, material and financial resources allocated ;<br>- Insufficient in-service training<br>- Practice of self-medication
**OPPORTUNITIES**	**THREATS**
- National Antimicrobial Resistance Plan<br>- Presence of TFPs	- Porous borders (bringing in counterfeit drugs that cause resistance)<br>- Antimicrobial resistance
**ANTIMICROBIAL STEWARDSHIP**	
**FORCES**	**WEAKNESSES**
- Existence of a quality assurance mechanism quality assurance mechanism of drugs set up by the DPML, the IGSPL and LANACOME	Lack of data on the proportion of poor quality antimicrobials<br>- The policy for using the NML to antimicrobials is not applied in private facilities

- Requirement for a marketing authorization for products acquired by tender<br>- Text on pharmaceutical market surveillance under preparation<br>- Inspection at points of import<br>- Visual inspection of drug samples at CENAME<br>- CENAME refers to 03 pharmacopoeias (American, Japanese and European) during calls for tender<br>- Development of alerts for the attention of<br>of the public by the MINSANTE on the publication of counterfeit AM	- Pre-qualification of suppliers is not foreseen<br>- LANACOME's limited technical resources<br>- The Special Contracts Commission, which guaranteed greater flexibility, speed and technicality, was dissolved by the last decree reorganizing CENAME
**OPPORTUNITIES**	**THREATS**
- Support of the CHMP (France) to CENAME in the facilitation of quality control of medicines	- Circulation of antimicrobials
**NATIONAL THERAPEUTIC PROTOCOLS**	
**FORCES**	**WEAKNESSES**
- Existence of some therapeutic guides<br>- Consideration of NWPs in the NML<br>- Qualified and trained NWP personnel	- Some NWPs are not updated<br>- Low production and dissemination of national documents<br>- A minority of providers trained in NWP<br>- Non-compliance with national guidelines by care providers<br>- Absence of guide national antimicrobial prescribing guidelines<br>- Availability of NTDs only for priority diseases
**OPPORTUNITIES**	**THREATS**
- Conducting AMR research<br>- Presence of TFPs	- Risk of epidemics, pandemics<br>- Antimicrobial resistance<br>- Socio-political conflicts
**NATIONAL LIST OF ESSENTIAL DRUGS**	
**FORCES**	**WEAKNESSES**

- Existence of an NML<br>- Consideration of NWPs in the NML<br>- Existence of a national multidisciplinary committee for the selection of essential medicines<br>- Partial data available<br>- NML validated and published<br>- Available at Central, Regional, CENAME, FRSP level,	- EMRL is not updated<br>- NWP of new directives not updated<br>- Lack of data on the availability of drugs on the NML<br>- Low dissemination of the NML<br>- Low availability of NML in health facilities<br>- Staff not adequately trained<br>- Pharmacovigilance system still in its infancy in Cameroon
**OPPORTUNITIES**	**THREATS**
- Presence of TFPs (USAID, MTAPS, GHSC-PSM, WHO, GEORGETOWN University,...)	- Irregularity in the holding of the sessions of the specialised commission in charge of the selection of essential medicines.<br>- Risk of epidemics, pandemics<br>- Antimicrobial resistance<br>- Socio-political conflicts

8.2. ANIMAL HEALTH

Forces	Weakness es
**National regulations, legislation or policies**	
Existence of a national pharmaceutical policy that provides guidance on the management and regulation of drugs	Lack of other policies supporting the regulation of veterinary drugs, with particular attention to antimicrobials
Existence of legislation or regulations requiring registration of medicines used in the country (MA)	Irregularity of inspection and control operations of veterinary pharmacies
Existence of legislation or regulations requiring the approval of pharmaceutical dispensing premises/points, including pharmacies. Authorization to open an establishment or to set up a private practice issued by MINEPIA for the former and ONVC for the latter	No data available on the degree of compliance with the regulations. Data are not available on the impact of the regulations.
Existence of regulations requiring registration of veterinary pharmaceutical personnel by a practitioner registration authority	No system exists to monitor antimicrobial sales throughout the country. However, veterinary drug import proformas are sent to the DSV for authorization.
The national drug regulatory authority has quality control or quality assurance and registration tools for antimicrobials in place. Through inspection, monitoring and testing of samples at the laboratory.	Lack of regulations and guidelines to regulate the promotional (advertising) activities of pharmaceutical companies
Existence of formal regulations for import, export, production, distribution, storage and use of finished antimicrobial products and active ingredients pharmaceutical	Lack of a legal framework on the use of antibiotics as a growth promoter in livestock and farms

Existence of regulations limiting veterinary antimicrobials to prescription-only status	The absence of a legal mechanism that requires the presence and intervention of a veterinary adviser in each animal production unit to ensure compliance with the standards for the prevention and treatment of infections. (charter sanitation)
	Lack of a harmonized legal framework on biosafety in livestock and farms
	Insufficient legal provisions on maximum residue limits and withdrawal periods for the administration of veterinary drugs in general and of antimicrobials in particular
Forces	Weakness es
**Good Procurement Policies**	
Quality assurance mechanisms are in place in public and private sectors to ensure the quality of antimicrobials marketed in the country	The following other quality assurance mechanisms are almost not in place:<br>• Laboratory analysis of drug samples<br>• Specific reports requested on drugs, such as bioavailability<br>• Informal information gathered from other procurement programs<br>• Certificate of Good Manufacturing Practice<br>• Monitoring of the quality of marketed products<br>• Inspection at the point of import<br>• Laboratory analysis of samples obtained at distribution points<br>• Monitoring and evaluation of supplier performance
Two public laboratories (LANAVET and LANACOME) are authorized to test the quality of antimicrobials	
The quality assurance mechanisms that are in place to ensure the quality of antimicrobials are the pre-qualification of suppliers and the submission of a profarma invoice (registration form) by the supplier for technical advice for importation. These mechanisms are regularly applied.	

	Much less regularly applied:<br>Physical inspections of veterinary drug samples (however no SOP)<br>Inspection at retail/wholesale pharmacy level (but no SOP)
Data are available on the importation of antimicrobials into value and quantity at national level to DSV	The illicit circuit escapes the data collection mechanism existing.
	Data from veterinary clinics and pharmacies or from use in production units are not systematically collected.
Research work has been carried out in previous years to take stock of the situation (see chapter 8.2)	There are no recent data on the proportion of antimicrobials below the standard over the years previous
	Documented reports of falsified veterinary antimicrobials are available in the literature and estimate a 70% rate of non-compliant antimicrobials sold on the market (Messomo, 2006; Ndottiwa, 2008)
	There is no list of veterinary antimicrobials subject to prescription to purchase

Forces	Weakness es
**Drug use and AMR policies/regulations**	
A wide range of antimicrobials belonging to different families is available on the national market	There is no policy/recommendation guiding the use of OIE guidelines for antimicrobial use
An annual report on the volume of drug imports and products for veterinary use is regularly prepared and transmitted to the OIE	There is no SOP for the treatment of infectious diseases for any animal species
	There is no list of essential medicines that includes antimicrobials (antibiotics, antifungals, antivirals and antiparasitics). However, Decree No. 2008/2908/PM of 5 December 2008 establishing the conditions for the manufacture, packaging, import, wholesale and retail sale of veterinary drugs, provides for a list of drugs for which a prescription is required to be signed by the Minister in charge of veterinary services (Art 45)
**Appropriate use of antimicrobials by prescribers, dispensers and end users**	
There are studies/reports on the degree of relevance of the appropriate prescribing and dispensing of antimicrobials (see Chapter 8.3)	Lack of surveillance and pharmacovigilance system legally regulated and operationalized appropriate use of antimicrobials (Mouiche et al., 2019)
Data are available on antimicrobial use by farmers in various production systems (including appropriate level of use) (See Chapter 8.2)	Distribution of antimicrobials by some feeders outside the regulations

	Misuse of antimicrobials in poultry and poultry products pigs
Public information campaigns on the appropriate use of veterinary antimicrobials/antibiotics and on antimicrobial resistance were conducted during the two last few years (report)	Lack of official control of residues in food of animal origin distributed on the national market
	Difficulties in accessing AMR diagnostic facilities (insufficient diagnostic laboratory)

Opportunitie s	Threats
- International commitment to fight AMR (FAO, WHO, OIE), Global Action Plan to fight AMR;<br>There is a national action plan to combat AMR;<br>The country has participated in the WHO Global Week for Appropriate Use of Antibiotics (specifically for animal health organized by LNSP) or in other global or regional awareness campaigns on antimicrobial resistance (report);<br>Training of 05 executives from MINEPIA and LANAVET on AMR management (OHCEA training 2019) ;<br>Interest of partners (FAO, OIE, USAID, MTaPS, OHCEA...) in the fight against AMR	Non-harmonized sub-regional policies
	Porosity of borders and development of illegal circuit
	Terrorism and other social, economic and political crises of external origin Weak resources for market surveillance and establishment inspection<br>Weak coverage of the national territory by private veterinary services<br>Low level of training of farmers

9. CONCLUSION

This report presents the results of a situational analysis that examined the content, coherence, relevance and effectiveness of policies related to antimicrobial stewardship in human and animal health, while identifying the strengths and weaknesses of these policies. The research focused on four themes:

- National pharmaceutical regulations, legislation or policies ;
- Good Procurement Policies ;
- Drug use and AMR policies/regulations ;
- Appropriate use by prescribers, dispensers and end users.

In human health, the NPP, whose aim is to improve access to quality health care by making safe, effective, good quality pharmaceutical products available to the entire population at an affordable price, while ensuring their rational and safe use, does not provide specific guidelines on the proper management and regulation of antimicrobials. Guidelines or guidelines for the management of certain diseases defining the different national treatment protocols are developed for certain infections but are not sufficiently appropriate for all actors in the health pyramid. Several factors are also responsible for the non-optimal use of the existing MEL throughout the health pyramid. In addition, the unofficial drug distribution circuit persists.

In animal health, although several texts relating to the use of veterinary drugs exist, specific provisions for antimicrobials are also rare. In addition, an official and an illicit circuit of veterinary drugs share the veterinary drug market with several implications. In addition, the use of antimicrobials, especially antibiotics, in production units and veterinary clinics is often not in accordance with the relevant OIE guidelines.

In the end, the improvement of antimicrobial stewardship is essential in any process of combating antimicrobial resistance (AMR). The realization of this situational analysis in the animal and human health sectors made it possible to highlight the strengths, weaknesses, opportunities and threats related to antimicrobial stewardship in Cameroon. It provides the country with priority elements and information on which to base the development of an integrated antimicrobial management action plan.

10. **REFERENCES**

Reference animal health section

1) Agency for Standards and Quality (ANOR), 2013. Who are we? www.anorcameroun.info Accessed on 09 May 2020.
2) Agency for Standards and Quality (ANOR), 2015. Catalogue of Cameroonian standards, Yaoundé, Cameroon, 76 p.
3) Edima H. C., Tofaing J. T., Ngoune I. T. and Ndjouenkeu R., 2012. Assessment of antibiotic residues in cow milk produced in Ngaoundere. International Journal of Biology, Pharmacy and Allied Sciences, 1(11), 1530-1538.
4) FAO/WHO/OIE. 2007. Report of the FAO/WHO/OIE expert meeting on critically important antimicrobial agents. Report of the FAO/WHO/OIE Expert Meeting, Rome, Italy, 68p.
5) Mazra, 2014: Veterinary drugs market in Cameroon: organization, limitations and proposals for improvement. PhD thesis in veterinary medicine, EISMV, Cheikh Anta Diop University of Dakar, 86p.
6) Messomo, 2006: Etude de la distribution et de la qualité des médicaments vétérinaire au cameroun. PhD thesis in veterinary medicine, EISMV, Cheikh Anta Diop University of Dakar, 113p.
7) MINEPIA, 2016. Antiparasitic and antimicrobial management plan (PG2A) of the Livestock Development Project (PRODEL). Final report, Yaoundé, Cameroon, 54p.
8) MINSANTE, 2018: National Action Plan to combat antimicrobial resistance, 2018-2020, Cameroon.
9) MINSANTE, 2017: Self-assessment and Action Plan for strengthening the implementation of the International Health Regulations (IHR, 2005) in Cameroon.
10) Mouiche M.M.M., Mazra M., Moffo F., Mpouam S. E., Ngoujigne A. I., Feussom K.J.M., Akoda G. K., Awah-Ndukum J., 2018: Veterinary drugs market in Cameroon: Regulation, organization and distribution channels. Bulletin of Animal Health and Production in Africa, 66 (3) 591-695.
11) M.M.M. Mouiche, B.Z.N. Njingou, F. Moffo, S.E. Mpouam, K.J. M. Feussom, and J. Awah-Ndukum, 2019. Veterinary pharmacovigilance in sub-Sahara Africa context: a pilot study of adverse reactions to veterinary medicine in Cameroon. BMC Veterinary Research,15: 301.
12) Mouiche M.M.M., Moffo F., Penda R. E., Betsama J.D., Mapiefou N. P., Achu M.A., Cleophas Kahtita Mbah, Mpouam S. E., Ciewe C.S.A., Djuikoua B.D.K., Sakativa D, Feussom K.J.M., Fotso K.Z., Awah-Ndukum J., 2020: Challenges of antimicrobial consumption surveillance in food-producing animals in Sub-Saharan African countries: Patterns of antimicrobial imported in Cameroon from 2014 to 2019. Journal of Global Antimicrobial Resistance 22, 771-778.
13) Ndottiwa M., 2008. Legislation, distribution and quality assessment of

veterinary drugs in Cameroon. PhD thesis in veterinary medicine, Rabat (I AV Hassan II), Rabat - Morocco, 155p.

14) OHCEA, 2017. Assessment of the legal and institutional framework for the fight against antimicrobial resistance in Cameroon: OHCEA Cameroon Exploratory Study.
15) ONVC and ATASPAC, 2017. Convention de collaboration entre l' Ordre National des Vétérinaires du Cameroun (ONVC) et l ' Association des Techniciens en Aquaculture, Santé, Pêches, et Productions Animales du Cameroun (ATASPAC), 4p.
16) Vougat N.B.R.R/, Garabeb R.B., Rhumbeiha K.W., Foyet H.S.,Schrunk D.E., Shao D., Zoli P.A., 2019. Penicillin-G and oxytetracycline residues in beef sold for human consumption in Maroua, Cameroon. International Journal of Food Contamination, 4:17.
17) Nno Nkassa C.M., 2016: Antibiotic use in companion animals in veterinary clinics in the cities of Douala and Yaounde (Cameroon). PhD thesis in veterinary medicine, ESMV, University of Ngaoundéré, 51p.
18) Nouedjalié K.I.U., Ngono M.R., Nguidjoe E. M., Ofodo T., Zoli P. A., 2018: Status of the regulations of the use of antimicrobials in livestock farming in Cameroon. Health Sciences and Diseases, 20 (2), 62-66.
19) Okah-Nnane N.H., 2016: Dietary exposure assessment of tetracycline residues in beef, milk and eggs marketed in Douala, Yaounde and Ngaoundere. Dissertation in Veterinary Medicine, University of Ngaoundere (ESMV), Ngaoundere - Cameroon, 85P.
20) OIE, 2015a: OIE List of Antimicrobial Agents of Veterinary Importance. (Online) http://www.oie.int/fileadmin/Home/eng/Our_scientific_expertise/docs/pdf/Eng_OIE_List_antimicrobials_May2015.pdf
21) OIE, 2015b:OIEStandards, Guidelines and Resolution on antimicrobial resistance and the use of antimicrobial agents. (Online) https://web.oie.int/delegateweb/eng/ebook/AF-book-AMR-ANG_FULL.pdf?WAHISPHPSESSID=03152ead00d06990fa9066b7b71fcabc
22) OIE, 2017: Annual report on antimicrobial agents intended for the use in animals: Better understanding of the global situation, Second report, Paris, France.
23) OIE, 2018 : OIE Annual report on antimicrobial agents intended for use in animals: better understanding global situation, Third report, Paris France, 129p
24) Wafo T. E., 2014. Evaluation of biosecurity measures in laying hen farms and investigation on the presence of antibiotic residues in table eggs in Cameroon. Dissertation in Veterinary Medicine, University of Ngaoundéré (ESMV), Ngaoundéré - Cameroon, 120 p.
25) WHO, 2016. Global Action Plan to combat antibiotic resistance. (Online) http://www.who.int/antimicrobial-resistance/global- action-plan/en/

26) Wouembé F. K., 2013. Analysis of antibiotic use in modern laying hen farms in the West Cameroon region. PhD thesis in veterinary medicine, EISMV, Cheikh Anta Diop University of Dakar, 96p.

Reference human health section

- Marketing authorisation, licence, inspection

[1] Decree N°98-405/PM OF 22 OCT 1998 fixing the modalities of approval and marketing of pharmaceutical products.

[2] Organigram of the Ministry of Public Health of Cameroon, Year 2013.

[3] Circular letter No D36-36 LC/MINSANTE/SG/DPML of 09 August 2019 on the provisions to be respected in the importation of pharmaceutical products with a marketing authorization in Cameroon

[4] Circular No. D36-52/C/MINSANTE/CAB of October 13, 2017 specifying the modalities for the procurement of pharmaceutical products for the Regional Health Promotion Funds (RHPF)

[5] Circular letter No. D36-26/LC/MINSANTE/SG/DPML of 17 July 2019 on irregularities found in the field of wholesale distribution of pharmaceutical products

[6] Decree N°2018/764 of December 11, 2018 on the reorganization of the national laboratory of quality control of medicines and expertise

[7] Decree N°2018/501 of 20 September 2018 on the reorganisation of the national supply centre for essential medicines and medical consumables

[8] Decree No. 2018/366 of 20 June 2018 on the public procurement code

[9] Quality assessment minimum activity package

[10] Grid for visiting pharmacies within the framework of the PBF

[11] IGSPL Activity Report 2018-2019

[12] Circular letter N° LC/D/36-75/MINSANTESG/DPML/SDP of 16 October 2018 on Pharmacovigilance Guidelines.

[13] Circular letter No. LC/D/36-76/MINSANTESG/DPML/SDP of October 16, 2018 designating the qualified person responsible for pharmacovigilance.

- Market surveillance and pharmacovigilance

1. Law 90/035 of 10 August 1990 to regulate the practice and organization of the profession of pharmacist in Cameroon
2. Decree N°2013/093 of 03 April 2013 on the organization of the Ministry of Public Health
3. LC N° D36-27/LC/MINSANTE/SG/DPML of 17 July 2019 relating to the prohibition of direct importation of Pharmaceutical Products by dispensing pharmacists.
4. Decree No. 98/405/PM of 22 October 1998 to lay down the procedures for the registration and marketing of pharmaceutical products
5. Decree No. 96/055 of 12 March 1996 establishing LANACOME and amended by Decree No. 2018/764 of 11 December 2018 reorganizing LANACOME
6. Order N°2964/MINSANTE of 09 October 2013 on the creation, organisation and functioning of the National Public Health Laboratory
7. IGSPL inspection reports
8. 2018 Antimicrobial Consumption Report, DPML.
9. Guide to Integrated Disease Surveillance and Response (IDSR guide)
10. LC N°D36-29/LC/MINSANTE/SG/DPML of 17 July 2019 on irregularities noted in the medical promotion/visit sector in Cameroon
11. Order N°8 of 13 July 1981 regulating the advertising of medicines.
12. No. 223/LC/MSP/DPM/SDNC/SIVAQ/BI on free medical samples
13. Grace-Ange Elong Ekambi, et al. Knowledge, practices and attitudes on antibiotics use in Cameroon: Self-medication and prescription survey among children, adolescents and adults in private pharmacies. PLoS One . 2019; 14 (2): e0212875.
14. Circular letter N° LC/D/36-75/MINSANTESG/DPML/SDP of 16 October 2018 on Pharmacovigilance Guidelines.
15. Circular letter No. LC/D/36-76/MINSANTESG/DPML/SDP of October 16, 2018 designating the qualified person responsible for pharmacovigilance.

- **Policies policies and guidelines from management Guidelines for Antimicrobial Stewardship**

1- National List of Essential Drugs and Other Pharmaceutical Products version of January 30, 2017

2- Georges Emmanuel TSAYID, Proper use of antibiotics: a week to raise awareness, Cameroon Tribune, 13 November 2018.
3- Call à application for "In-service Training on Antimicrobial Resistance (AMR) for human, animal and environmental health professionals in Cameroon," OHCEA, 2019.
4- Report of the evaluation study of the national pharmaceutical sector in Cameroon 2003
5- Elong Ekambi G-A, et al. and prescription survey among children, adolescents and adults in private pharmacies. February 2019; PLoS ONE 14(2): e0212875.
6- Draft 2018 antibiotic consumption report for Cameroon, DPML, 2020.

- **Prescription, Dispensing, and Use of Antimicrobials**

1) Malaria ECP Guide (2019)
2) Childhood Illness Care Guide (CICG)
3) Guide to syndromic STI management,
4) Guide to preventing TB transmission in health facilities and communities (2016),
5) TB ECP Guide (2020),
6) National Cholera Prevention and Control Guidelines (2008)
7) HIV/AIDS Prevention and Care Guidelines 2013
8) Disease Management Guide (CIDP)
9) Pharmacovigilance Bulletin No. 1, September 26, 2019, DPML
10) Mohamed Moctar Mouliom Mouiche ,et al. Challenges in monitoring antimicrobial consumption in food animals in sub-Saharan African countries: patterns of antimicrobials imported into Cameroon from 2014 to 2019. j.jgar.2020.06.021.

11. LIST OF CONTRIBUTORS

- Dr LOUDANG MEGUIEZE, IGSPL;
- Dr VANDI DELI, DPML;
- Dr. BOMBAH JESSICA, SDM, DPML;
- Dr SAMBA Frederique, DEX, CENAME;
- Dr MBWE MPOH Maurice, SDMT, DOSTS;
- Dr NGONO Tsala Diane, Pharmacist, DPML;
- Dr. NOGHA STEPHANIE, SV, DPML;
- Dr ELUMBA Prospère, Pharmacist, DPML ;
- Dr SHANKOU Sandra, Pharmacist, DLMEP ;
- Dr ELONG Epse EKEKE, Pharmacist, HLD ;
- Dr ABESSOLO, Infectiologist, HCY;
- Dr TOLLO TOLLO Daniel, Pharmacist, NLP;
- Dr. MOTAZE Jessica, Pharmacist, DPML;
- Dr Audrey MBOTO, Pharmacist, HCY;
- The MTaPS Team of Cameroon ;
- Dr GARGA GONNE, Director of Veterinary Services ;
- Dr. PENDA Rose Eliane, Deputy Director of Pharmacy and the Veterinary Private Sector;
- The National Veterinary Laboratory (LANAVET);
- The Regional Delegate for Livestock, Fisheries and Animal Industries of the Centre ;
- The Departmental Delegate for Livestock, Fisheries and Animal Industries of Mfoundi ;
- Dr DJUIKOUA Diane, Head of the Veterinary Pharmacy Department
- Dr. DIDIER SAKATIVA, Head of the Private Veterinary Sector Department ;
- Dr HERVE GAEL NDALLE Head of the Aquatic Species Health Department ;
- Dr. CIEWE Serge, Head of the Domestic Animal Health Service ;
- Dr DJONWE Gaston, former DSV, EPT-2/ECTAD FAO Coordinator;
- Dr. Ichakou, ONVC, MedVet;
- Dr Mazra Mahamat, Commercial Director CAPHAVET ;
- Dr Messomo Florent, LAPROVET ;
- Dr. Mohamadou, Boeringer;
- Dr Zambou Henri, EQUAVET Veterinary Clinic Douala ;
- Dr Njong, Tropical Veterinary Society ClinicYaoundé ;
- Dr Dewa Mohamadou, Managing Director MODESA S.A;
- Dr Bouopda Fotso Christelle Animal Paradise Veterinary Clinic Yaoundé ;
- Dr. Djuntu Michael, VETACTION Veterinary Clinic Ltd;
- Mrs. Ngodwe Ida, Executive at the DSV ;
- Mr. Zemeboma Hianick, Executive at the DSV.
- Ms. WEGANG Georgette, Executive, DLMEP.
