# Supplementary material for: Landscape Analysis of Existing Policies, Laws and Regulations on Antimicrobial Stewardship in 10 Low- and Middle-Income Countries
Source: Antibiotics (Basel). 2026 Aug 10;15(8):770. doi: 10.3390/antibiotics15080770 (PMC13510074; doi:10.3390/antibiotics15080770)
Supplement: Supplementary file 1 [file antibiotics-15-00770-s001.zip › S3 Country Reports/S3D_Côte d'Ivoire (Human & Animal Health).pdf]

PROGRAMME DES MÉDICAMENTS, TECHNOLOGIES ET SERVICES PHARMACEUTIQUES (MTaPS) DE L'USAID

Accès amélioré. Services améliorés. Meilleurs résultats de santé.

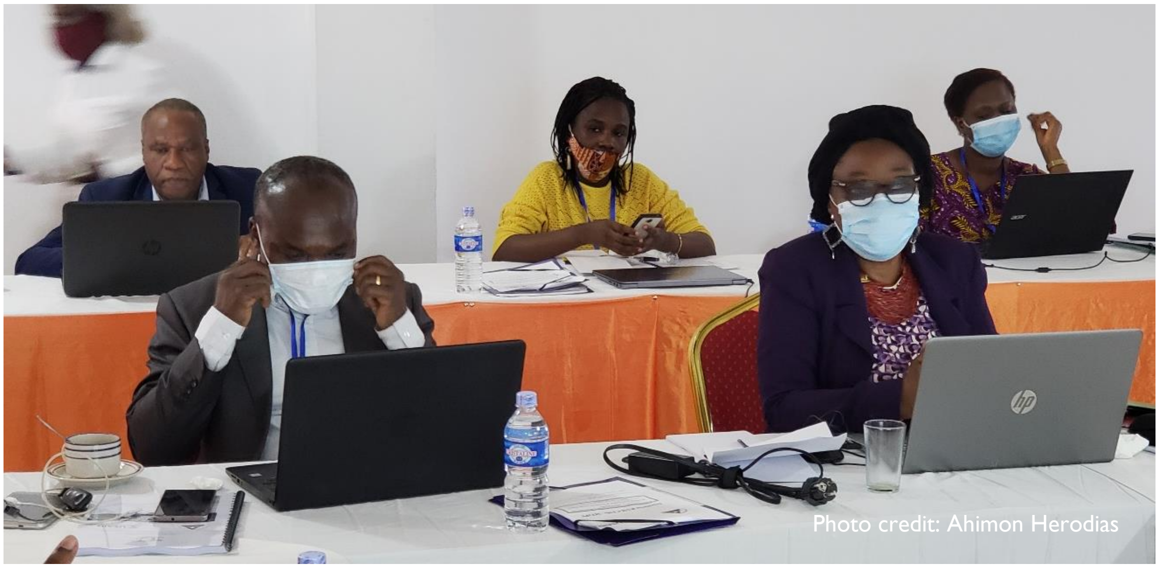

Some participants of the validation workshop of the report of the situational analysis of antimicrobial policies, regulatory framework and stewardship activities, 15-16 June, 2020, Hotel la Prunelle, Jacqueville

National RAM Secretariat - ORMICI

REPORT OF THE SITUATIONAL ANALYSIS OF ANTIMICROBIAL STEWARDSHIP IN THE HUMAN, ANIMAL, ENVIRONMENTAL AND AGRICULTURAL SECTORS IN COTE D'IVOIRE

Situational Analysis

JUNE 2020

USAID
DU PEUPLE AMERICAIN

This document was made possible by the generous support of the American people through the United States Agency for International Development (USAID) under contract number 7200AA18C00074. The contents are the responsibility of Management Sciences for Health and do not necessarily reflect the views of USAID or the United States Government.

About USAID's MTaPS Program

USAID's Medicines, Technology, and Pharmaceutical Services (MTaPS) program helps low- and middle-income countries strengthen their pharmaceutical systems, as strengthening these systems is critical to ensuring an effective health system. The MTaPS program works to increase access to essential health products and services and to promote the correct use of medicines so that people can enjoy better health. It builds on decades of expertise in developing state-of-the-art pharmaceutical systems in more than 40 countries. The MTaPS approach achieves sustainable results by including all stakeholders in the health sector: government, academia, civil society and the private sector. Led by Management Sciences for Health (MSH), a global health non-profit organization, the program is implemented by a consortium of global and local partners.

The MTaPS Consortium

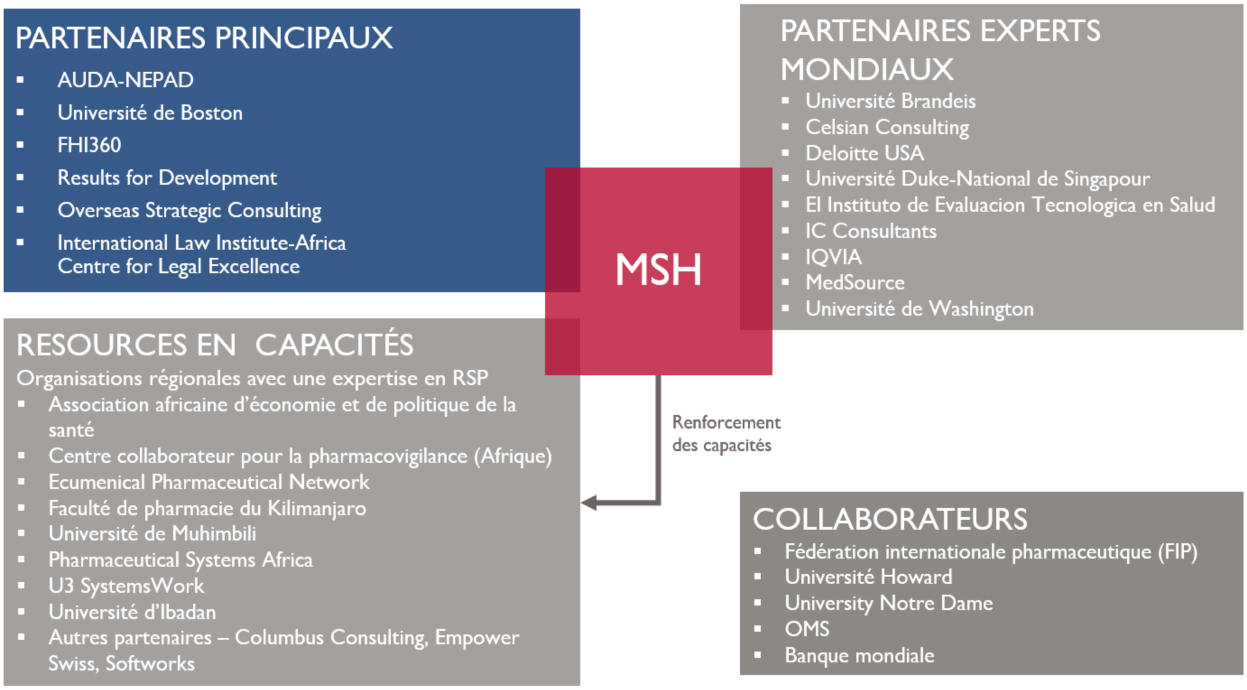

This report / document is presented by :
Herodias AHIMON
Senior Technical Advisor
Program Delivery Group
Management Sciences for Health
Ivory Coast - Abidjan Ivory Coast
Mobile phone :
+225 01 13 78 69
E-mail: hahimon@mtapsprogram.org

In collaboration with :

Professor Antoine Serge, AMARI
Pharmacist, consultant for human sector
Telephone: + 225 07 69 72 79
Email :
amariantoineserge@gmail.com

Dr. Zahoui Boris Arnaud, BITTY
Veterinary, consultant for the animal sector
Telephone: +225 59 86 42 28
Email :
bittyarnaud2004@yahoo.fr

ACRONYMS AND ABBREVIATIONS

AIRP	Ivorian Pharmaceutical Regulatory Authority
AMM	Marketing Authorization
ANDE	National Environment Agency
ALI	Adoption of Integrated Pest Management
VPA	Provisional Sales Authorizations
AVPPCI	Association of Private Veterinary Practitioners of Côte d'Ivoire
BMR	Multi-resistant bacteria
CAMMVET	Veterinary Drug Market Sanitation Campaign
CCC	Behavior Change Communication
CDC	Centers for Disease Control
ECOWAS	Economic Community of West African States
CIAPOL	Ivorian Anti-Pollution Centre
IPHC	Ivorian Pesticide Management Committee
CILSS	Permanent Inter-State Committee for Drought Control in the Sahel
CNGP	National Pesticide Management Committees
CNR	National Reference Centre
CODINORM	Côte d'Ivoire standardization
CSRS	Swiss Center for Scientific Research
CTD	Common Technical Document
MTA	Multisectoral Technical Committees
DAPL	Pharmaceutical Activity and Laboratories Department
DDD	Defined daily dose
DGE	Directorate General for the Environment
DHPSE	Directorate of Public Hygiene, Health and the Environment
DPML	Directorate of Pharmacy, Medicines and Laboratories
DMHP	Department of Hospital and Community Medicine
DSV	Direction of Veterinary Services
EBLSE	Enterobacteriaceae producing Broad Spectrum Beta-Lactamases
EEC	Joint External Evaluation
EEQ	External Quality Assessment
EPH	Public Hospital Establishment
CWSF	First Contact Health Facilities
FAO	Food and Agriculture Organization of the United Nations
FCFA	African Financial Community Franc

FSUCOM	Community-based Urban Health Training
GHSA	Global Health Security Agenda
GLASS	Global Antimicrobial Resistance Surveillance System
GMC	Multisectoral Coordination Group
GTT	Technical Working Group
GVG	Groupement des Vétérinaires Grossistes
INHP	National Institute of Public Hygiene
IPCI	Pasteur Institute of Côte d'Ivoire
STI	Sexually Transmitted Infections
LANADA	National Agricultural Development Support Laboratory
LCVB	Central Veterinary Laboratory of Bingerville
MRL	Maximum Residue Limits
LNSP	National Public Health Laboratory
MESRS	Ministry of Higher Education and Scientific Research
MIRAH	Ministry of Animal Resources and Fisheries
MSHP	Ministry of Health and Public Hygiene
MQIF	Substandard and Falsified Drugs
NPSP	New Public Health Pharmacy
OIE	World Organisation for Animal Health, formerly Office Internationale des Epizooties
OIPR	Ivorian Office of Parks and Reserves
WTO	The World Trade Organization
WHO	World Health Organization
ONVCI	National Order of Veterinarians of Côte d'Ivoire
ORMICI	Observatory of Microorganism Resistance to Antimicrobials in Côte d'Ivoire
OTC	Technical Barriers to Trade
PACE	Pan-African Programme for the Control of Epizootics
PAN-RAM	National Multisectoral Plan to Combat Antimicrobial Resistance
PANSS	National Health Security Action Plan
PCI	Infection Prevention and Control
NRTSP	National Focal Point International Health Regulations
PN-GAM	National Anti-Microbial Management Plan
NIPA 2	National Agricultural Investment Plan
POPs	Persistent Organic Pollutants
POS	Standard Operating Procedure
PPN	National Pharmaceutical Policy

PSDEPA	Strategic Plan for the Development of Livestock, Fisheries and Aquaculture
PULGA-CI	Emergency Program for the Control of Avian Influenza in Côte d'Ivoire
RAM	AntiMicrobial Resistance
RNO-CI	National Observation Network of Côte d'Ivoire
RSI	International Health Regulations
RT-PCR	Reverse Transcriptase - Polymerase Chain Reaction
SICOSAV	Veterinary Border Inspection and Control Service
SPS	Sanitary and PhytoSanitary
	(Agreement on the Application of Sanitary and Phytosanitary Measures)
VAS	Armed Forces Veterinary Service
SVM	Municipal Veterinary Services
UA-BIRA	African Union - Inter-African Bureau for Animal Resources
UEMOA	West African Economic and Monetary Union
UNICEF	United Nations Children's Fund.
USAID	United States Agency for International Development
TB	Tuberculosis
VICH	International Cooperation on Harmonisation of Technical Requirements for the Registration of Veterinary Drugs
HIV	Human Immunodeficiency Virus

SUMMARY

ACRONYMS AND ABBREVIATIONS i

PROJECT SUMMARY v

ACKNOWLEDGEMENTS v

ORIENTATION SUMMARY 6

INTRODUCTION 6

1. GENERAL CONSIDERATIONS 9
   - 1.1. GENERAL PROFILE OF COTE D'IVOIRE 9
   - 1.2. HEALTH CONTEXT 12

2. SITUATIONAL ANALYSIS 16
   - 2.1. HUMAN SECTOR 16
   - 2.2. ANIMAL SECTOR 28
   - 2.3. ENVIRONMENTAL INDUSTRY 41
   - 2.4. NATIONAL POLICY ON ANTIMICROBIAL RESISTANCE 44
   - 2.5. THE NATIONAL PLAN FOR RATIONAL ANTIMICROBIAL STEWARDSHIP 53

RECOMMENDATIONS 59

CONCLUSION 59

REFERENCES 60

LISTS OF TEXTS CONSULTED 61

PROJECT SUMMARY

**Name of the program**		USAID's Medicines, Technology and Pharmaceutical Services (MTaPS) Program
**Start and end dates of the activity :**		20 September 2018 - 19 September 2023
**Name of lead implementing partner :**		Management Sciences for Health
**Contract number :**		7200AA18C00074
**MTaPS Partners**	**Main partners**	Boston University, FHI 360, Overseas Strategic Consulting, Results for Development, International Law Institute-Africa Centre for Legal Excellence, New Partnership for Africa's Development (NEPAD)
	**Global Expert Partners**	Brandeis University, Deloitte USA, Duke National University of Singapore, El Instituto de Evaluación Tecnológica en Salud, ePath, IC Consultants, Imperial Health Sciences, MedSource, QuintilesIMS, University of Washington
	**Partners providing capacity resources**	African Association for Health Economics and Policy, Ecumenical Pharmaceutical Network, U3 SystemsWork, University of Ibadan, World Health Organization (WHO) Collaborating Centre for Pharmacovigilance at the University of Ghana, Kilimanjaro College of Pharmacy, University of Muhimbili, Pharmaceutical Systems Africa
	**Staff**	International Pharmaceutical Federation, Howard University, University of Notre Dame, WHO, World Bank

ACKNOWLEDGEMENTS

We would like to thank the ministries and national institutions for their support to the multisectoral technical working committee.

Our sincere thanks also go to the World Health Organization (WHO), the Food and Agriculture Organization of the United Nations (FAO), the World Organization for Animal Health (OIE), the Centers for Disease Control-Global Health Security Agenda (CDC-GHSA), and the United States Agency for International Development (USAID) for their technical and financial support for the preparation of this document.

Finally, our gratitude goes to ORMICI, which is a committed partner in the fight against antibiotic resistance and conducted a situation analysis in 2017.

The RAM Focal Point

Professor GUESSENND KOUADIO Nathalie

ORIENTATION SUMMARY

The National Plan for the fight against antimicrobial resistance (PAN-RAM) includes the following six strategic objectives

[i] Strengthening the organizational framework for combating antimicrobial resistance;

[ii] Increase awareness and understanding of AMR through communication, education, training; [iii] Strengthen the knowledge and evidence base on AMR through surveillance and research; [iv] Reduce the incidence of infections through effective sanitation, hygiene and prevention measures; [v] Optimize the rational use of antimicrobial drugs in human, animal and environmental health; [vi] and Promote sustainable investment based on country needs, and research and sustainable development that takes into account the needs of all countries, and increase investment in new drugs, diagnostics, vaccines and other interventions.

Prior to the evaluation of the National Plan to Combat Antimicrobial Resistance, scheduled for 2021, the AMR Technical Working Group (AMR-TWG) considered it appropriate to update the National Plan for Antimicrobial Management (PN-GAM) in the human, animal, environmental and agricultural sectors.

The working methodology was based on the capitalization of documentary resources on AMR followed by a field survey through questionnaires sent to the actors concerned.

The situation analysis describes the current situation, strengths, weaknesses, opportunities and threats (SWOT analysis) of the rational management of antimicrobials in Côte d'Ivoire. It allows to highlight the main characteristics of this policy in the human, animal, environmental and agricultural sectors.

It highlights a documented governance of the fight against AMR as well as a precise description of the actors intervening in the human, animal, environmental and agricultural sectors, which should facilitate the execution of the strategic actions and activities of the National Antimicrobial Management Plan. This plan has been enriched with some optimization axes in comparison with the original plan while maintaining the coherence with the general economy of the national plan for the fight against AMR. Thus, the optimization of the regulation of antimicrobial use, the reinforcement of inspection and control of services and practices related to antimicrobial stewardship, the active participation in the fight against the proliferation of the illicit market of substandard and adulterated antimicrobials are, among others, additional necessary strategic actions of the NAP-AMR.

In this context, it remains necessary for the development partners to support Côte d'Ivoire in the effective implementation of this PN-GAM for an effective fight against AMR and a reinforced protection of public health.

INTRODUCTION

Antimicrobials play an essential role in the control of infectious diseases in human and animal medicine. The[eme] introduction of penicillin in the 20th century significantly reduced the number of illnesses and

deaths from infectious diseases. Conversely, the miracle cure provided by antimicrobials for common infections is being eroded by the emergence of antimicrobial resistance.

Antimicrobial resistance, as defined by the World Health Organization (WHO), is the resistance of a microorganism to an antimicrobial drug to which it was previously susceptible. It results from the ability of certain microorganisms (bacteria, viruses, parasites, fungi) to withstand the attack of antimicrobial drugs such as antibiotics, antivirals and antiparasitics.

Resistance is related to the unaccountable and unsafe use of antimicrobial agents for therapeutic or non-therapeutic purposes leading to adverse effects in all sectors.

According to the ANSES report in 2014, antibiotic resistance has now obtained the status of zoonosis. Man and Animal exchange not only bacteria with, when these bacteria are pathogenic, an infectious risk, but also resistance factors that are likely to compromise the effectiveness of an antibiotic treatment.

The Global Action Plan was developed in close collaboration with the tripartite (FAO, WHO and OIE) which recognizes that the threat of AMR requires an effective approach, coordination involving many sectors at both national and international levels, and a commitment by countries reflected in a National Action Plan for AMR Control (NAP-AMR).

In this context, the government of Côte d'Ivoire adopted the agenda of the sixty-eighth World Health Assembly in May 2015 by developing its NAP-RAM through the "ONE HEALTH" approach.

PAN-RAM provides an organizational and structural matrix for inclusive and coordinated management of sectors such as human and animal health, agriculture, environment, finance, security and other stakeholders such as consumers, Non-Governmental Organizations (NGOs) and Civil Society Organizations (CSOs).

The objectives of PAN-RAM are to

1. strengthening the organizational framework for combating antimicrobial resistance ;
2. Improve awareness and understanding of antimicrobial resistance through effective communication, education and training;
3. strengthen the knowledge and evidence base through monitoring and research;
4. reduce the incidence of infection through effective sanitation, hygiene and infection control measures;
5. optimize the use of antimicrobial agents in human and animal health;
6. Promote sustainable investment based on country needs, research and sustainable development that takes into account the needs of all countries and increase investment in new medicines, diagnostics, vaccines and other interventions.

The National Plan for Rational Antimicrobial Management (NP-RAM) intends to work towards the achievement of the fifth objective of NAP-RAM. Based on a situational analysis, the NAP-MAR describes the regulation, control, supply and use of antimicrobials in the human, animal, environmental and agricultural sectors, as well as the main actors involved in antimicrobial stewardship.

The PN-GAM defines its specific objectives, its main lines of intervention, its strategic actions as well as its activities to be carried out in a logical framework in perfect coherence with the PAN-RAM. The PAN-RAM is itself developed in accordance with the principles set out in the national framework documents, in particular the National Health Development Plan (PNDS), the Strategic Development Plan for Livestock, Fisheries and Aquaculture (PSDPA), and the National Health Security Action Plan (PANSS).

Local, state and professional actors, governance structures involved in the fight against AMR and development partners have a key role to play in the effective implementation of this updated PN-GAM.

The national antimicrobial management plan defines the main strategic and operational objectives and includes a monitoring and evaluation plan. Its implementation will make it possible on the one hand to reduce the development and the propagation of AMR in the human, animal, environmental and agricultural sectors by a controlled and rational management of antimicrobial drugs and on the other hand to significantly improve the health of the populations in Côte d'Ivoire.

I. GENERAL CONSIDERATIONS

1.1. GENERAL PROFILE OF COTE D'IVOIRE

1.1.1 Presentation of Côte d'Ivoire

The 2014 General Census of Population and Housing (RGPH) gives a total population of 22,671,331 with a density of 70.3 inhabitants/km² . It is composed of 11,708,244 men or 51.7% and 10,963,087 women or 48.3%. The table below shows the main characteristics of this population.

Table I: Demographic profile of Côte d'Ivoire

	Population RGPH 2014	
	**Workforce**	**Percentages(%)**
Total population	22 671 331	100
Female population	10 963 087	48,4
Male population	11 708 244	51,6
Working population (15-64 years)	12 491 695	56
Urban population	11 370 347	50,2
Rural population	11 300 984	49,8
Foreign population	5 491 972	24,2
Population under 15 years of age	9 408 602	41,5
Average density (inhabitants/km²)	70,3	-
Dependency rate		78,6
Average annual growth rate	-	2,6
Total fertility rate (child/woman)	5	-

Source: NSI, 2014

The population living in urban areas is 11,370,347, or 50.2%, compared with 11,300,984 in rural areas, or 49.8%. Côte d'Ivoire has a high rate of immigration, the foreign population is estimated at 5 490 222 people or 24.2% of the total population.

Life expectancy at birth taking into account the impact of HIV/AIDS on mortality given by SPECTRUM in 2015 for Côte d'Ivoire is 54.3 years with respectively 53.4 years for men and 55.2 years for women[1] . The population is very young with a high number of addicts since one out of two (01) inhabitants is under 20 years old and two out of three (2/3) are under 25 years old (Figure 1). The rapid decrease in dependency ratios following the control of their fertility levels, has allowed emerging countries to open a "demographic window of opportunity" more favourable to savings, productive investments and investments in human capital, which has enabled them to benefit from the demographic dividend[2] . The demographic dividend is the benefit that countries can derive during the unique period in their history when they are undergoing their demographic transition, i.e., the transition from high levels of mortality and fertility to lower and controlled levels.

[1] NSDP2016-2020
[2] INS

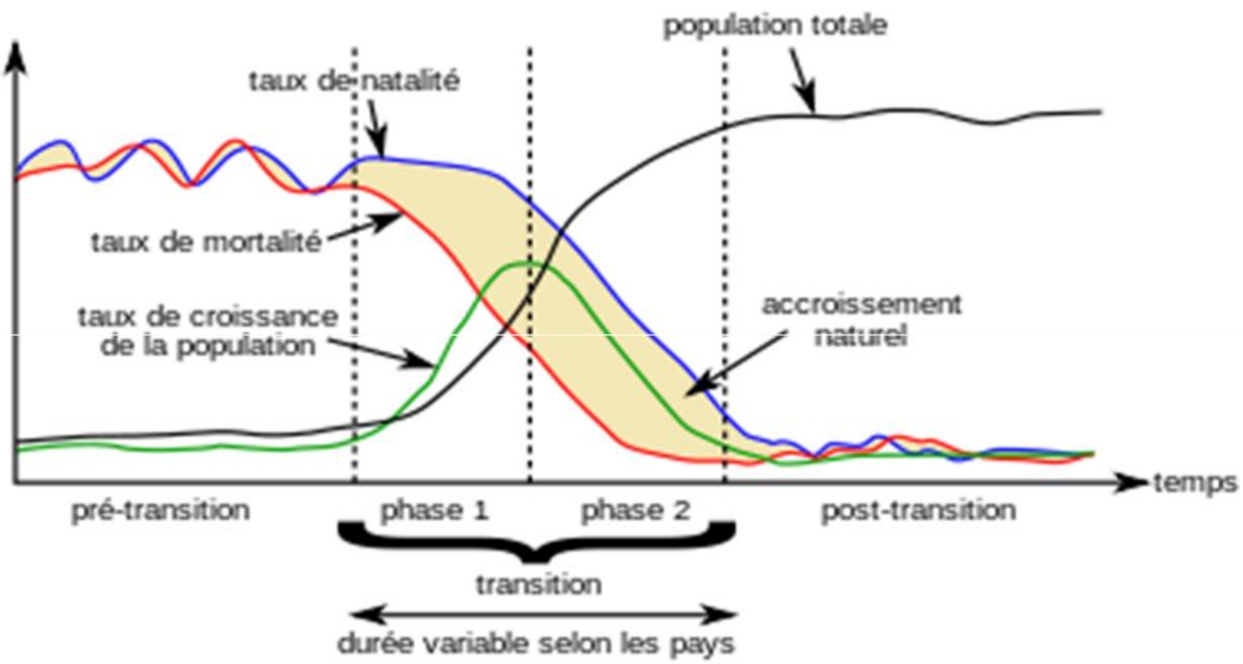

Source: SPECTRUM 2015

Figure 1: Stages of the demographic transition

The gradual control of mortality and then of fertility results in a decrease in the percentage of young people dependent on the working population, which makes it possible to better control the increase in health and education expenditures previously attributable to the continuous increase in the number of children and young people.

The realization of the demographic transition (stalled in Côte d'Ivoire since the 1990s) is one of the major transformations necessary to reach emergence and benefit from the demographic dividend. In emerging countries, population growth is now between 0.5% and 1.5% per year (compared to 2.6% in Côte d'Ivoire), women have on average no more than 3 children (compared to 5 children in Côte d'Ivoire), and 60 to 85% of them use a method of contraception, which favours their integration into the labour market (compared to only 18% in Côte d'Ivoire in 2012).

1.1.2 Administrative Context

The administrative division of the territory of March 27, 2012 includes 02 Autonomous Districts (Abidjan and Yamoussoukro), 31 regions, 107 departments, 423 sub-prefectures, 197 municipal communities and more than 8,600 villages, (www. gouv.ci).

The political capital, Yamoussoukro, is located in the centre of the country, 248 km from Abidjan, the economic capital.

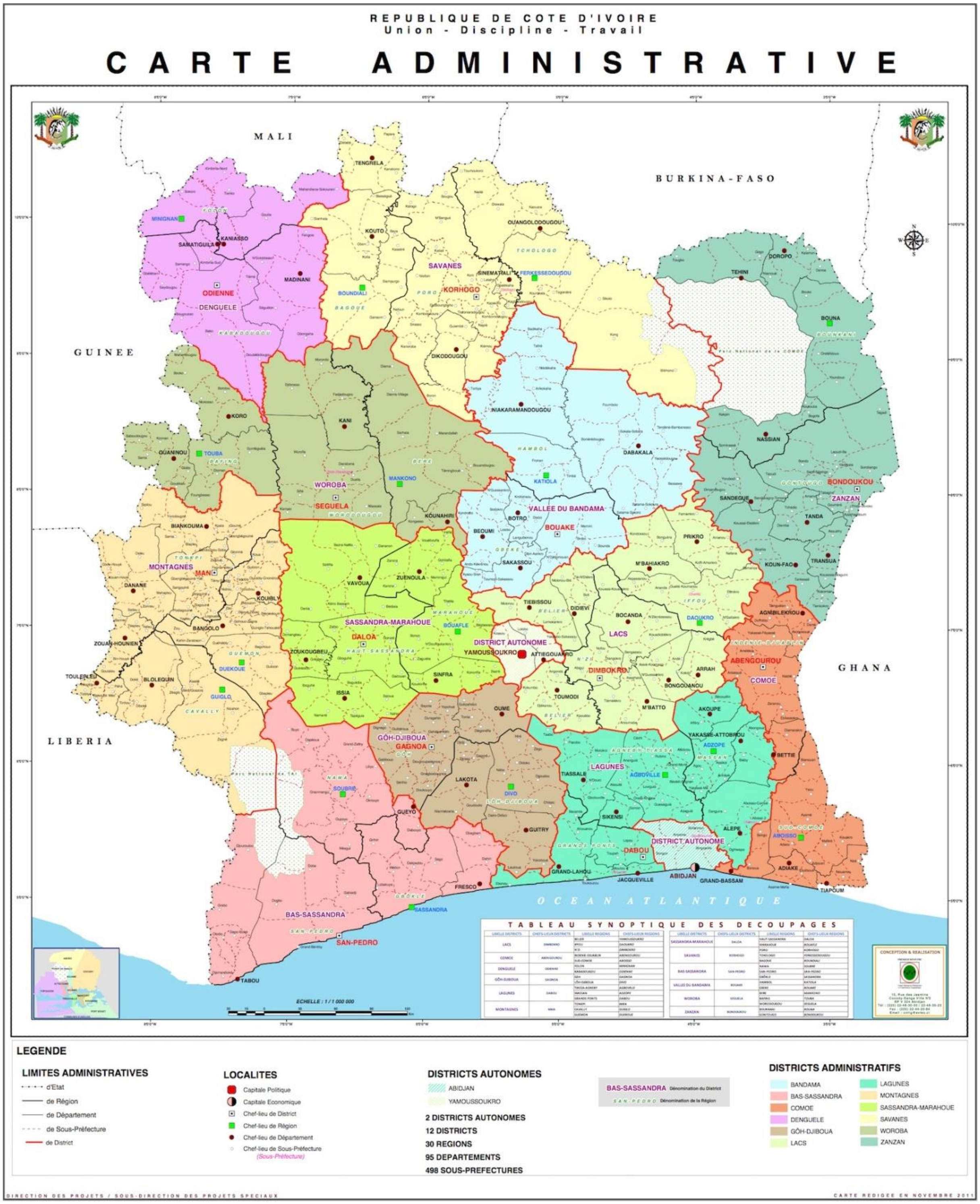

Source: National Institute of Statistics of Côte d'Ivoire

Figure 2: Administrative division of Côte d'Ivoire (BNETD, 2018)

1.2. HEALTH CONTEXT

1.2.1. HUMAN SECTOR

1.2.1.1 Health policy

The 2016-2020 NSDP places special emphasis on improving environmental sanitation and hygiene in the fight against the disease.

In recent years, the Ivory Coast has undertaken new reforms in the health sector. These are mainly (i) hospital reform, (ii) performance-based financing, (iii) health decentralisation, (iv) the organisation of community-based interventions, (v) the organisation and reorganisation of emergency services in the University Hospitals (UHC) and Regional Hospitals (RHC), and (vi) the University Health Coverage (UHC)

1.2.1.2 Organization of the Ivorian health system

The Ivorian health system includes the public health care offer, the private health care offer and the health administration. It is pyramid-shaped with three (3) levels and two sides: one managerial and the other provider (figure 3).

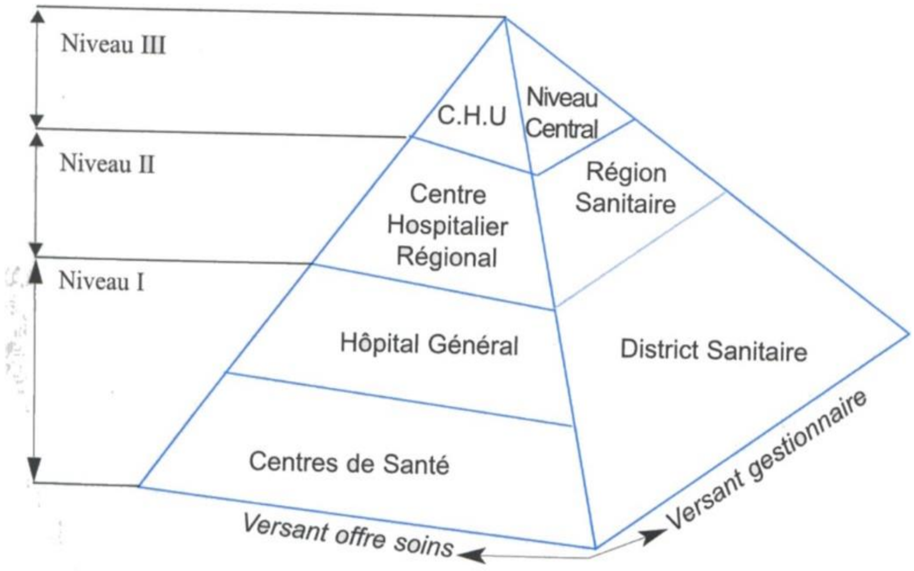

Figure 3: Health pyramid of Côte d'Ivoire (PNDS 2016-2020)

- Health authorities

The Ivorian health system has three (03) levels in its administrative structure:

- The central level, with the Minister's office, central departments and services, and health programmes, is responsible for policy definition, support and overall coordination;
- The intermediate level is composed of 33 Regional Directorates whose mission is to support the health districts in implementing health policy;

- The peripheral level is made up of 112 Departmental Directorates or Health Districts, responsible for coordinating the health activity under their jurisdiction and providing operational and logistical support to health services. The health district, the operational unit of the health system, is itself subdivided into health areas or catchment areas of the Primary Health Care Facilities (PHCF).

- Health care supply

The Ivorian health system is dominated by a predominantly public sector and a rapidly expanding private sector, alongside which traditional medicine plays a relatively important role.

- Public health sector

The public health care sector is organized into three levels:

- the primary level represented by 2330 First Contact Health Facilities (FCF);
- the secondary level made up of health establishments for first referral with 68 General Hospitals (HG), 20 Regional Hospitals (CHR) and 2 Specialized Hospitals (CHS);
- the tertiary level composed of health establishments of recourse for the second reference with 05 University Hospitals (CHU), 05 National Specialized Institutes[3] (INS) and 04 other National Public Establishments[4] (EPN) of support.

It should be noted that other ministries such as Defence, Economy and Finance, Public Service and Administrative Reform, Solidarity, Family, Women and Children, National Education and the Interior participate in the provision of care through their health infrastructures.

- Private health sector

The private health sector has developed in recent years with the emergence of private health establishments of all classes and categories (polyclinics, clinics, medical centres and practices, pharmacies and private infirmaries). It is mainly present in large urban areas or economic centres. In 2011, the country had 2036 private health establishments in this sector, of which 1482 (73%) were not authorised by the Ministry of Health and Public Hygiene (MSHP).

With 49 health establishments, the faith-based sector, associations and community-based organizations (ABC/OBC) also participate in the provision of health care, especially at the primary level[5] .

In 2014[6] , the private pharmaceutical sector occupies a very important place in the health system and covers between 80 and 90% of the drug supply. This private pharmaceutical sector mainly includes:

- four (04) wholesaler-distributors;

[3]National Institute of Public Health (INSP), National Institute of Public Hygiene (INHP), Raoul Follereau Institute (IRF), Pierre Richet Institute (IPR), Abidjan Heart Institute (ICA)
[4]National Blood Transfusion Centre (CNTS), National Public Health Laboratory (LNSP), New Public Health Pharmacy (N-PSP), Emergency Medical Service (SAMU)
[5]Ministry of Health and Public Hygiene, RCI:PNDS2016-2020
[6]Study for the Development of Local Pharmaceutical Industries in Côte d'Ivoire- UNIDO-PACIR-Project EE/IVC/010/001- 2014

- 1100 private pharmacies ;
- eight (08) drug production units, of which four (04) are in operation. Local production is estimated at 6% of the national pharmaceutical market.

- Traditional medicine

The practice and organization of traditional medicine and pharmacopoeia are regulated by *Law No. 2015-536 of July 20, 2015 and Decree No. 2016-24 of January 27, 2016 on the Code of Ethics and Deontology of practitioners of traditional medicine and pharmacopoeia.* Traditional medicine has more than 8500 Traditional Medicine Practitioners (PMT) listed by the National Program for the Promotion of Traditional Medicine (PNPMT). In 2010, 1204 TMPs were trained; some in anatomy and conventional hygiene, and others in intellectual property rights.

I.2.1.3 Laboratory network

Like the health pyramid, the laboratory network is organized in a pyramidal fashion. *Decree No. 96-876 of 25 October 1996 on the classification of public health establishments* gives the composition of the technical platform of each public health establishment and specifies the availability of medical biology analysis laboratories in each type of establishment from the rural health centre to the university hospital.

The statistical data on the distribution of laboratories is as follows:

Table II: Number of laboratories at local, intermediate/district and national levels

Level	Number
I: Laboratories of First Contact Health Facilities (FCFH)	625
II: Laboratories of General Hospitals and Regional Hospitals	100
III: Central laboratories	11
Total	736

Source: International Health Regulations (IHR) Assessment Report (WHO, 2005)

I.2.2. ANIMAL SECTOR

In Côte d'Ivoire, the animal health sector is structured around veterinary services placed under the direction and direct control of the Direction des Services Vétérinaires (DSV). The DSV is one of the 5 central operational directorates of the Ministry of Animal and Fishery Resources (MIRAH).

In its actions, the DSV is supported by the Support Project for Animal Health and Veterinary Public Hygiene (PASA-HPV). This project was born in March 2010 from the merger of the Emergency Programme for the Control of Avian Influenza in Côte d'Ivoire (PULGA-CI) and the Pan-African Programme for the Control of Epizootics in Côte d'Ivoire (PACE-CI). The PASA-HPV's mission is to improve animal health and veterinary public hygiene through inspections of foodstuffs of animal origin, live animal imports, the fight against epizootics and the fight against the sale of fraudulent medicines.

I.2.2.1 Veterinary health policy

The Strategic Plan for the Development of Livestock, Fisheries and Aquaculture (PSDEPA) 2014-2020 has as its objectives among others:

- Sustainable mitigation of animal diseases and emerging and re-emerging zoonoses

- Improving and revitalising services for the procurement, inspection, monitoring, distribution and use of veterinary products
- Strengthening sub-regional and international cooperation in the area of prudent and rational management of antimicrobials for veterinary use.

I.2.2.2 Organisation of the Ivorian veterinary health system

The Ivorian animal health system also includes public health care, private health care and the veterinary health administration run by the Côte d'Ivoire Veterinary Services.

- Veterinary health administration

There are three (03) levels in the veterinary administrative structure:

- The central level with the Cabinet of the Minister of Animal Resources and Fisheries, the central directorates and services, the animal health and veterinary public hygiene programmes, is responsible for the definition of the policy, the support and the global coordination in the field of animal health and veterinary public hygiene
- The intermediate level is composed of 19 Regional Directorates whose mission is to implement veterinary health policy
- The peripheral level is composed of 58 Departmental Directorates, 174 Posts of Livestock and Fisheries Resources (PERH) and 14 Border Inspection Posts (PIF).

- Veterinary care services
    - Public Sector

The public veterinary care sector is coordinated by the DSV and conducted in the field by the veterinary care units of the Departmental Directorates.

At the central level, the Centre Anti-Rabies Vétérinaire (CARV) housed within the DSV carries out animal health activities.

It should be noted that other ministries, notably those of Defence, the Interior and Health, are involved in the implementation of animal health and veterinary public hygiene policy.

- Private Sector

The private veterinary health sector has developed in recent years with the opening of private veterinary practices throughout the Ivorian territory. The volume of imports of veterinary drugs in the private veterinary pharmaceutical sector in 2019 amounts to the sum of Nine billion thirty-one million one hundred and eleven thousand fifty-two (9,031,111,052) CFA francs.

There are currently 12 veterinary drug importing and distributing wholesalers and 30 private veterinary practices.

I.2.2.3 Official support laboratory

The Laboratoire National d'Appui au Développement Agricole (LANADA) through the Laboratoire Central Vétérinaire de Bingerville (LCVB) is the reference laboratory for quality control of veterinary

vaccines. LANADA is under the supervision of the Ministry of Agriculture and Rural Development and is part of the national network of laboratories involved in the fight against AMR.

I.2.3. ENVIRONMENTAL SECTOR

This sector is organized on 2 levels, central and regional.

The central level includes the General Directorate of the Environment (DGE) and three central directorates: the Ivorian Anti-Pollution Centre (CIAPOL), the National Environment Agency (ANDE) and the Ivorian Office of Parks and Reserves (OIPR).

The regional level is covered by 31 Regional Directorates, of which 25 are functional, depending on the DGE and 3 regional branches depending on CIAPOL.

These antennas cover the primary level, i.e. the environmental matrices.

Wildlife monitoring is the responsibility of the OIPR in parks and reserves. For all other non-protected areas, surveillance is carried out by the Water and Forestry Service.

In Côte d'Ivoire, legal instruments have been put in place for the protection of the environment against the risks of pollution and degradation. These include *Law No. 96-766 of 3 October 1996 on the environmental code, Decree No. 97-678 of 3 December 1997 on the protection of the marine and lagoon environment against pollution, and Decree No. 98-42 of 28 January 1998 on the organisation of the emergency plan to combat accidental pollution at sea, in lagoons and in coastal areas (POLLUMAR)*. However, as in the sectors previously mentioned, there is an absence of legislative and regulatory provisions specific to AMR in the environmental sector.

I.2.4. AGRICULTURAL SECTOR

In the agricultural sector, the *law n°2015-237 of July 2015 of agricultural orientation*, defines and implements a policy aiming at ensuring the sanitary safety of food of plant and animal origin and veterinary public health by the control of zoonoses. In addition, texts, standards or regulations govern the hygienic quality of all food and feed (control and obligations for professionals). We can mention in particular *law n°63-323 of 25 July 1963 relating to the sanitary police of animals in Côte d'Ivoire; law n°63-301 of 26 June 1963 relating to the repression of fraud in the sale of goods and falsification of foodstuffs and agricultural products and decree n°79-573 of 4 July 1979 regulating restaurants.* Normative provisions have also been put in place with the creation of Côte d'Ivoire Normalisation (CODINORM) whose objectives include the elaboration of standards and the development of conformity certifications.

2. SITUATIONAL ANALYSIS

2.1. HUMAN SECTOR

The organization of the pharmaceutical sector in Côte d'Ivoire was initially governed since 1954 by provisions inherited from the French public health code made applicable in the colonies including Côte d'Ivoire. Since its independence in 1960, Côte d'Ivoire has continued to maintain a good organization of

the pharmaceutical sector based on a pharmaceutical administration represented by the Direction de la Pharmacie, du Médicament de Laboratoires (DPML).

The organization of the pharmaceutical sector in Côte d'Ivoire has been characterized since 2017 by the advent of the Ivorian Authority for Pharmaceutical Regulation (AIRP). This regulatory body of the pharmaceutical sector is instituted by the *law n°2017-541 of August 03, 2017 relating to the regulation of the pharmaceutical sector and* is operational since 2020. In addition to the AIRP, a central directorate of the Ministry in charge of Health was created, the Directorate of Pharmaceutical Activity and Laboratories (DAPL), which inherits the attributions of the Former National Program for the Development of Pharmaceutical Activity (PNDAP). The DAPL is responsible in particular for designing the National Pharmaceutical Policy and plays an important role in the selection and quantification of drugs.

National texts (laws, decrees, orders) and community standards, mainly from WAEMU (regulations, directives, decisions) govern pharmaceutical regulatory functions. Thus, the regulation *n°06/2010/CM/UEMOA relating to procedures for the approval of pharmaceutical products for human use in WAEMU Member States*, provides a framework for the registration of medicines prior to their marketing. The importation of medicines is also regulated by Decision No. 09/2010/CM/WAEMU *on the adoption of the Guide to Good Distribution and Importation Practices of Pharmaceutical Products for Human Use in WAEMU Member States*. Finally, some national provisions including the medical and pharmaceutical codes of ethics regulate the prescription and dispensing of medicines in Côte d'Ivoire.

However, apart from programmes to control tuberculosis, AIDS, malaria, hepatitis and algorithms for sexually transmitted infections (STIs), there is a lack of national guidelines for the use of antimicrobials, although the general principles of good management and use of medicines in general and antimicrobials in particular are reflected in the medical and pharmaceutical codes of ethics.

2.1.1- MARKETING AUTHORIZATION, LICENSING AND INSPECTION

2.1.1.1. Marketing authorisation and quality control

Under Article 4 of *Regulation No. 06/2010/CM/WAEMU on the approval procedures for pharmaceutical products for human use in WAEMU Member States*, "**no pharmaceutical product for human use may be placed on the market free of charge or against payment in a WAEMU Member State, if it has not first obtained a Marketing Authorization (MA)**. Apart from a few exceptions such as the continuation of a treatment not available in the list of products sold in Côte d'Ivoire, the imperative reason of public health, the needs of research or humanitarian aid, the rule of prior MA is an absolute rule and a constant of the pharmaceutical regulation systems.

2.1.1.1.1. Applicable regulations

The registration of drugs including antimicrobials in Côte d'Ivoire has always been regulated. The basic criteria for the registration of drugs (safety, therapeutic interest, novelty) have been constantly reaffirmed by successive legal provisions, namely *Law No. 65-250 of August 4, 1965 amending articles of the Public Health Code relating to the regime of drugs*, and especially *Decree No. 94-669 of December 21, 1994 on registration and dispensing of drugs in Côte d'Ivoire*. Since 2010, the registration of medicines is governed by *Regulation No. 06/2010/CM/WAEMU on the registration procedures of pharmaceutical products for human use in WAEMU Member States*. The principles and provisions of this regulation were recently

made effective in Côte d'Ivoire by three decrees relating to the establishment of a committee of experts for the technical analysis of MA dossiers (*Decree No. 2015-569 of 29 July 2015 on the establishment of the committee of experts for the technical evaluation of applications for approval of medicines)*, a national drug commission (*Decree No. 2015-568 of July 29, 2015 on the creation, organization and operation of the national drug commission*) and licensing fees (*Decree No. 2015-602 of September 2, 2015 on the establishment of fees for the marketing authorization of drugs*). The MA granted at the end of the harmonized evaluation procedures of the registration files remains a national MA issued by each WAEMU member state exclusively on its behalf.

2.1.1.1.2. Procedure for obtaining marketing authorisation

The application: The application submitted for the purpose of obtaining marketing authorization for a medicine is addressed to the National Authority for Pharmaceutical Regulation. The application is composed of five (5) modules organized as follows:

- The administrative file or module 1 ;
- The technical dossier comprising the 4 other modules, namely module 2 (summary of the technical dossier), module 3 (quality dossier), module 4 (non-clinical dossier) and module 5 (clinical dossier, bioequivalence data or comparative dissolution tests when the medicinal product is under a generic name).

The evaluation of the dossier : In accordance with Community regulations, the various bodies involved in the procedure for granting medicinal products and in particular antimicrobials are :

- the Pharmaceutical Regulatory Authority, which provides the secretariat and the administrative evaluation, i.e. module 1.
- the Committee of Experts, which carries out the technical evaluation of applications for the registration of medicinal products covering all modules 2, 3, 4 and 5;
- the National Commission for Medicines, which is responsible for validating the work of the committee of experts and proposing a final opinion on the dossier to the Minister of Health.

The Expert Committee is responsible for giving an opinion on the quality, safety, security and efficacy of pharmaceutical products for human use submitted for registration.

The national medicines commission is also responsible for giving a final opinion on applications for marketing authorisation, temporary suspensions or plans for the definitive withdrawal of marketing authorisations, variations and transfers of marketing authorisations. Variations are changes to the specifications that prevailed when the MA was obtained, during the period of validity of the MA.

2.1.1.1.3. Issuance of the MA

The MA is issued to a legal entity (generally the manufacturer laboratory through a local representative agency), for a pharmaceutical product corresponding to a speciality, a generic drug or a vaccine.

2.1.1.1.4. Validity and renewal

A MA is valid for five (5) years.

Any variation in the elements of the file of a Marketing Authorisation must be notified to the Minister responsible for health, justifying the change.

All MAs must be renewed in accordance with the regulations in force, before the expiry of their period of validity.

2.1.1.1.5. Refusal of marketing authorisation
The MA of a drug, including antimicrobials, may be refused, after a reasoned opinion from the National Commission for Medicines for Human Use.

Any medicinal product for human use presenting a danger to public health may be suspended for a period of 6 months or permanently withdrawn.

Any medicinal product which has been suspended or permanently withdrawn shall be withdrawn without delay from the distribution circuit.

2.1.1.1.6. Quality control
Quality control of medicines for registration : The *decree n°197/MSHP of October 21, 2009 fixing the conditions of the analytical expertise of drugs submitted for registration in Côte d'Ivoire* establishes, in a compulsory way, an analytical expertise of all files of drugs submitted for registration in Côte d'Ivoire. This expertise must cover both the registration application files and the analytical control of samples submitted for registration. For each drug selected, the Pharmaceutical Regulatory Authority is required to send the National Public Health Laboratory (LNSP) a copy of the technical file as well as the samples necessary for the analytical control.

The National Public Health Laboratory is in charge of quality control of incoming and circulating drugs in Côte d'Ivoire. Under *Law 2017-541 of August 03, 2017 on the regulation of the pharmaceutical sector*, the AIRP has the ability to contract with other laboratories (in case of inability of LNSP) to ensure the quality control of medicines. Thus in the absence of a systematic local quality control, the drug in circulation in Côte d'Ivoire always remains of doubtful quality.

Quality control of imported medicines: This control is regulated by *Order n°198/MSHP of 21 October 2009*. Only medicines imported by the public purchasing office (Nouvelle Pharmacie de la Santé Publique NPSP) are subject to this control by the agreement between the NPSP and the National Public Health Laboratory.

Post-marketing control or market surveillance: This control is regulated by *Order No. 198/MSHP of 21 October 2009*. However, this control is not regular and is dependent on external funding. There is no drug quality monitoring program with a sustainable funding mechanism that systematically involves the LNSP, the AIRP, the Health Programs and the New PSP.

2.1.1.2. LICENSING OF PRACTITIONERS

2.1.1.2.1 manufacturing and wholesale establishments
The manufacturing establishments or pharmaceutical industries do not benefit in Côte d'Ivoire from a sufficiently developed regulation. We have to go back to 1987 to find a specific regulation relating to the creation of pharmaceutical industries through the *decree n°173 of 18 April 1986 on the modalities of*

creation of pharmaceutical industries. In 2015, *the law n°2015-533 of July 20, 2015 relating to the practice of pharmacy* defines the industrial pharmaceutical establishment as any establishment having a manufacturing unit and carrying out the operations of manufacturing, importing, exporting and wholesaling of manufactured drugs to authorized pharmaceutical structures.

The license to practice is granted to structures that include pharmacists in accordance with the legislative and regulatory provisions in force. To date, Côte d'Ivoire has 13 authorized pharmaceutical industries (source DPML, 2020), only half of which are actually active.

Concerning wholesale distribution establishments, a distinction is made between wholesale distributors and, under a recent regulation, import and export wholesale distributors. The wholesaler-distributor establishments get their supplies from manufacturers in order to supply pharmaceutical products to retailers such as pharmacies (*decree n°015/MSHP/CAB of February 10, 2010 on the conditions of creation and operation of pharmaceutical wholesaler-distributor establishments in Côte d'Ivoire*); there are five (05) of them (LABOREX, DPCI, COPHARMED, TEDIS PHARMA for the private sector) and NPSP in charge of supplying essentially the public sector. The depository and wholesale distributor establishments for import and export also obtain their supplies from the manufacturer, but only for the supply of medicines to wholesale distributors ( ). There are three of them (AIMAS, IVOIRE SERINGUE and DEPOPHARMA) (DPML 2020).

2.1.1.2.2 Retail Drug Establishments

The establishments for the retail distribution of medicines are essentially represented by private pharmacies. Côte d'Ivoire currently has 1,150 of them, all of which are run by a pharmacist registered with the National Order of Pharmacists, who may be assisted, depending on the workload, by one or more other pharmacists and non-pharmaceutical staff commonly known as pharmacy assistants.

Hospital pharmacies are located within public and private hospitals and serve the internal drug needs of the institutions that house them.

Pharmaceutical warehouses are managed by non-pharmacists who have a minimum level of education of Grade 1 and hold a Brevet d'Etude du Premier Cycle (BEPC). These managers work under the control of the pharmacist who supplies the pharmaceutical sales depot.

These various retail outlets must be authorized by the Minister of Health.

2.1.1.2.3. Pharmaceutical representation establishments

Pharmaceutical representation establishments are establishments responsible for promoting objective medical and medico-economic information to health professionals on health products and medical equipment. They were elevated to the rank of pharmaceutical establishments by *law n°2015-533 of 20 July 2015 on the practice of pharmacy*. Commonly also called pharmaceutical representative agencies, these establishments serve as intermediaries between the manufacturing laboratory and the local pharmaceutical authorities. They follow on behalf of the manufacturing laboratory all the steps inherent to the marketing of the product and deploy in the field the marketing strategy and advertising of the drug. In this respect, the promotional establishments employ sales representatives or medical representatives and import promotional material as well as medical samples.

2.1.1.3 Inspection

The inspection activity concerns manufacturing, distribution and dispensing, import and export establishments, medical analysis laboratories, clinical trial sites and pharmaceutical promotion establishments. A procedures manual and inspection grids are available and are in line with *Decision n°08/2010/CM/WAEMU adopting the guide of good manufacturing practices for pharmaceutical products in WAEMU Member States* and *Decision n°09/2010/CM/WAEMU adopting the guide of good distribution and import practices for pharmaceutical products for human use in WAEMU Member States*. These inspections result in the issuance of good practice certificates (National Pharmaceutical Policy NPP, 2015).

In accordance with *Law No. 2017-541 of August 03, 2017 on the regulation of the pharmaceutical sector*, pharmaceutical inspection is carried out by pharmacy inspectors placed under the authority of the AIRP.

The performance of the pharmaceutical inspection is limited due to a structural deficit, lack of human, material and logistical resources and the absence of a body of sworn inspectors.

In the course of a study (Gouepo, 2019) whose objective was to assess the main characteristics of pharmaceutical inspection as practiced in pharmacy dispensaries in Côte d'Ivoire from 2009 to 2018, the indicators of inspection from the perspective of legislative and regulatory requirements applicable in Côte d'Ivoire were examined.

The study identified several deficiencies that pose a high risk of critical non-compliance to pharmacy operations, including

- The insufficient number of inspection missions by the DPML to cover all the pharmacies in the territory,
- the shortage of pharmacist inspectors (only 6 inspectors were mobilized in 2014 to cover the entire territory),
- the uneven distribution of inspections, which is almost absent in some regions,
- the constant dysfunctions in almost all the inspected pharmacies, including the absenteeism of the pharmacy owners,
- dispensing drugs in disregard of good drug dispensing practices.

2.1.2 POST-MARKETING SURVEILLANCE AND PHARMACOVIGILANCE

2.1.2.1. PHARMACOVIGILANCE

The purpose of pharmacovigilance is to ensure the safe use of medicinal products and health products placed on the market in order to contribute to their proper use in the context of public health protection and information for health professionals.

To date, there is no law in Côte d'Ivoire that is specific to pharmacovigilance.

However, at the regulatory level, pharmacovigilance is governed by *Order No. 210 of November 24, 2010 amending Order No. 16 of February 10, 2009 on the creation, organization and operation of the national system of pharmacovigilance*, and *Order No. 211* of the same day *amending Order No. 249 of November 18, 1988 on the creation of a National Commission of Pharmacovigilance*

With regard to the regulations in force, the functioning of pharmacovigilance in Côte d'Ivoire allows us to describe three categories of actors:

- a regulatory body, (
- Autorité Ivoirienne de Régulation Pharmaceutique (AIRP)),
- a shared information gathering and dissemination body, (
- a Directorate of Computer Science and Health Information)
- central bodies (national pharmacovigilance commission, national pharmacovigilance centre and their various branches.

In addition, there is a set of peripheral bodies for the recording and collection of information on pharmacovigilance, which are

- health programs,
- institutions
- the organisations producing, exploiting and distributing the products concerned by pharmacovigilance,
- health professional organizations and health professionals,
- public and private pharmacies and health facilities,
- health training institutions
- development partners.

Any holder of a marketing authorisation for a medicinal product, including an antimicrobial, must join the national pharmacovigilance system.

2.1.2.2. Antimicrobial consumption surveillance

Surveillance of antimicrobial consumption is not routinely conducted, however pilot studies have been conducted in collaboration with WHO

In 2016-2017 (October 2016 to January 2017), a national antimicrobial consumption survey was conducted among 4 private wholesalers and NPSP.

The objective of the survey was to measure the consumption of eight classes of antimicrobial drugs in Côte d'Ivoire over the period 2014-2015.

The classes of antimicrobials included in the survey are:

- antibacterials for systemic use ;
- digestive system antibiotics;
- nitro imidazole derivatives;
- antifungals;
- Antimycotics;
- anti-tuberculosis drugs;
- Antivirals;
- anti-malarial drugs.

The results of the survey showed that 1320 antimicrobial references were distributed over the period from January 1, 2014 to December 31, 2015 of which more than 90% were distributed by the private sector and 10% by the public sector (the New PSP).

Systemic antimicrobials and antimalarials accounted for 63.86% and 20.68% of the references, respectively. These two classes represent 84.5%.

In general, the results revealed that the consumption of antimicrobials increased between 2014 and 2015 from 17.65 DID/1000/inhabitants/day (daily defined doses (DDD) per 1000 inhabitants per day) to 22.46 DDD/inhabitants/day respectively. This consumption is dominated by systemic antibiotics which increased from 7.78 DDD in 2014 to 11.71 DDD in 2015 or 50.51% of overall consumption, followed by antiretrovirals with 5.78 DDD in 2014 and 6.53 DDD in 2015 or 30.6%, and antimalarials with 1.93 DDD in 2014 and 2.01 DDD in 2015 or 9.76%.

In the class of antibacterials for systemic use, amoxicillin in combination or not with inhibitors is the most used with 4.45 DIDs in 2014 or 57.2% of the consumption of antibacterials and 5.42 DIDs in 2015 or 46%.

The artemether/lumefantrine combination is the main antimalarial drug for a consumption that increased from 0.78 DID in 2014 (40.6%) to 0.98 DID in 2015 (48.7%).

Cotrimoxazole had the highest increase from 0.61 DID in 2014 to 3.28 DID in 2015 or 400% increase.

In view of the results, it appears that the consumption of antimicrobials in Côte d'Ivoire has increased over the two reference years. However, given the methodology adopted, the distribution data only give an idea without reflecting the real level of consumption at the patient level. However, factors could explain the distribution levels observed: inappropriate prescriptions, easy access to antibiotics due to the multiplicity of references and the non-existence or non-enforcement of regulations concerning them and the financial accessibility of antibiotics, especially generics *(*Survey report on antimicrobial consumption in the RCI, 2017*)*.

Table IV: Consumption by antimicrobial classes. (Source of survey on antimicrobial consumption in Côte d'Ivoire, 2017)

ATC Class	Data 2014 (DID)	Data 2015 (DID)	Changes between 2014 and 2015 (%)	TOTAL IN DID
A07AA - Digestive system antibiotics	0,003	0,012	300	0,015
D01BA - Antifungals	0,44	0,47	7	0,91
J01-Antibacterials for systemic use	7,78	11,71	50,5	19,49
J02-Antimycosis	0,58	0,56	-3,6	1,14
J04-Tuberculosis	0,52	0,41	-21,15	0,93
J05-Antivirals/ARVs	5,78	6,53	13	12,31
P01AB- Nitroimidazoles	0,62	0,76	22,6	1,38
P01B-Antipaludics	1,93	2,01	54,1	3,93

In any case, the consumption of antimicrobials remains significant since according to recent data collected from the Directorate of Pharmacy, Medicines and Laboratories (DPML) antimicrobials accounted in 2018, 37.04% of registered drugs including 19,67% for antibiotics alone, 2.85% for

antibiotics in combination and 14.52% for other anti-infectives and in 2019 34.64% including 18.5% for antibiotics alone, 2.05% for antibiotics in combination and 14.02% for other anti-infectives

(DPML Activity Report, 2018 and 2019).

2.1.2.3 National Essential Drug List (NEDL) and antimicrobials

Essential medicines are medicines that meet the priority health needs of a population. The WHO list is intended to serve as a model for the development of an essential medicines list at the national level.

In Côte d'Ivoire, the NEML is developed with the participation of local stakeholders, and can be revised every two years; however, in practice, this frequency of revision is difficult to achieve. The last NEML was signed in 2014 and the last revision completed in 2019, awaiting signature by the Minister of Health.

The choice of essential medicines depends on many factors, such as local disease prevalence, unit and total cost of treatment, available means of treatment, training and experience of available personnel, financial resources, and genetic, demographic and environmental factors.

The process of elaboration or revision of the LNME is a participatory process that involves local actors, notably the Directorate of Pharmacy, Medicines and Laboratories, the National Therapeutics Commission, the New Public Health Pharmacy, the National Program for the Development of Pharmaceutical Activities, health professionals from the public sector, pharmacists, doctors and dental surgeons from the private sector, the professional orders of pharmacists, doctors and dental surgeons as well as development partners, particularly for financing, which often constitutes a blockage in the revision process.

The revision process generally involves the following successive steps: national data collection, a workshop to revise the NML, validation of the draft list by the National Drug Commission, and signature of the draft list by the Minister in charge of Health who consecrates the NML.

The latest (2019) revised NML includes 1170 drug designations (references) of which 266 references of antimicrobials i.e. a percentage of 22.73%. These antimicrobials are divided into 201 anti-infectives (anti-amoebic, antibiotics, antifungals, anti-hepatitis B and C, antionchocercials, antimalarials, antituberculosis, schistomicides, trypanocides), 57 antiretrovirals and 18 antivirals.

For public health establishments, the use of products on the list depends on their classification by level: level A (University Hospitals and National Specialized Institutes), level B (Regional Hospitals, General Hospitals, Specialized Hospitals), level C (Urban and Specialized Health Centers, Urban Health Units), level D (Rural Health Centers), level E (Village Pharmacy).

2.1.3 GOOD SUPPLY MANAGEMENT POLICIES AND GUIDELINES FOR HUMAN ANTIMICROBIALS

2.1.3.1 Structure of the pharmaceutical market

The Ivorian pharmaceutical market is supplied by imports of medicines from various continents (90%) and by local production (10%).

According to various sources, the total pharmaceutical market, both public and private, amounted to CFAF 147.6 billion in 2006, of which CFAF 135 billion was for the private sector, i.e. more than 80%.

Two categories of structures are responsible for importing medicines, the NouvellePharmacie de la Santé Publique for the public and private wholesale distributors: LABOREX-CI (Ubipharm), DPCI, COPHARMED, TEDIS-Pharma. These structures alone do not reflètent fiable data because of direct imports by pharmacies and the existence of parallel and illicit circuits.

In terms of pharmaceutical regulation, legal and regulatory provisions exist. The various processes concerning pharmaceutical supply are governed by law n° 2015-533 of 20 July 2015. At the regulatory level, the authorized structures and products that are authorized for importation are well mentioned (Mapping of the supply and distribution systems of medicines and other health products in Côte d'Ivoire, PNDAP Dec 2016).

2.1.3.2 Institutions in the NGO sector and faith-based structures

They obtain most of their supplies of essential generic medicines either through donations that comply with WHO guidelines or through purchases from the NPSP and private pharmacies. Unfortunately, the donations received by this sector and these supply channels are often made outside the terms of these regulations.

2.1.3.3 Informal sector

It is characterized by the parallel drug market with its multiple consequences in terms of public health (therapeutic failures, intoxications, antibiotic resistance) and fiscal wastage. It has taken on a worrying scale and is estimated to account for about a third of the drug market.

The illicit pharmaceutical market is difficult to assess, given the number of clandestine transactions. Sales outlets are scattered throughout the country, but the largest market is Roxy, located in Abidjan (in the commune of Adjamé). These operators are mostly illiterate, traders, women, children, craftsmen, and paradoxically also retired health professionals, students of all profils.

Pharmaceutical products enter the informal market in Côte d'Ivoire through a number of channels, including some neighbouring countries, but also through the diversion of medicines from the official distribution channel. In fact, most of this market is made up of illicit imports, purchases from wholesalers, legal producers and pharmacies. To this must be added diversions from public and private establishments, medicines from collections of recovered medicines and medical samples. A national strategy document to combat the illicit market in pharmaceutical products was adopted by the government in 1997 with the creation of a National Committee to Combat Illicit Trafficking and Counterfeiting of Medicines, set up in 1998 following a communication to the Council of Ministers (National Pharmaceutical Policy (PPN), 2009 edition).

The need to fight against this phenomenon, among other reasons, led the State to pass *Law No. 2017-541 of August 03, 2017 on the regulation of the pharmaceutical sector,* which, in addition to creating a regulatory body whose one of the essential attributions is the fight against fake drugs, establishes very heavy criminal sanctions against the perpetrators of illicit trafficking of drugs.

The will of the State of Côte d'Ivoire to accentuate the fight against counterfeiting and the illicit market of medicines has been materialized by the adhesion to the MEDICRIME convention on July 03, 2019.

The MEDICRIME Convention is the first international criminal law instrument that condemns counterfeiting of medicines as a crime and punishes it criminally

2.1.4 PRESCRIBING, DISPENSING AND USE OF ANTIMICROBIALS FOR HUMAN USE

2.1.4.1. PRESCRIPTION

Interviews with prescribers show that the choice of antibiotic therapy is often guided by empiricism, the practitioner's experience, the clinical condition of the patient and the properties of the drug in relation to the results expected by the prescriber. This choice of drug is also influenced by the advertising done by medical sales representatives to promote their products. Despite the existence of some national guidelines for the treatment of certain diseases and apart from HIV, tuberculosis and malaria, the standardization of antibiotic therapy is problematic. Indeed, for these three diseases and especially HIV and tuberculosis, standard treatments within validated hospital protocols exist. But for other diseases or associated diseases requiring the use of antimicrobials, the therapeutic schemes and the use of antimicrobials vary according to the prescriber and the patient's compliance.

Observation shows that in health care services, probabilistic antimicrobial treatments are often favoured. In his study on the exposure profile and practical modalities of antibiotic use among patients hospitalized in the intensive care unit of the University Hospital of Yopougon in Abidjan, KACOU et al 2009, indicate that during the observation period (8 months, July 2008 to March 2009), 74.6% of patients received antibiotic therapy systematically initiated on average 1.2 days after their admission to the unit, which remained high compared to the exposure rates observed in Lyon (65%) or Paris (59%). The pressure of antibiotic use was 86.4% of the time spent in the department, which is higher than the rates observed for example at Hôpital Bichat-Claude Bernard (60%). Moreover, only 8.3% of antibiotic treatments were documented from a bacteriological point of view, compared to higher rates in Lyon (31%), Paris (54%) and Denmark (36%). In this context, the 2019 edition of the Recueil des Protocoles Thérapeutiques Normalisés, an update of the 2013 edition, should be welcomed. This book, produced by the Ministry of Health and Public Hygiene with the financial support of the World Health Organization, is intended as a practical guide to assist in the prescription and rational dispensing of drugs. It provides health professionals with the national therapeutic protocols in force. It is the result of the close cooperation between various actors of the health system and reflects the will of the Ministry of Health and Public Hygiene to achieve the rational use of drugs by publishing and distributing a collection of protocols for the therapeutic management of common pathologies. *(Compendium of Standardized Therapeutic Protocols, Editions 2019)*.

These tools for rationalizing drug use, the Compendium of Standard Therapeutic Protocols (CSTP) and the NML, should be further popularized to promote the rational use of antimicrobials.

Finally, it appears from our survey in the context of this work that prescribing practices and the quality of antimicrobial use are not monitored, even on the scale of representative samples of public or private health care institutions; this would have allowed a realistic evaluation and a consequent modification of prescribing habits, if necessary.

2.1.4. 2. DELIVERY

Generally speaking, the dispensing of medicines and antimicrobials in particular suffers from insufficient regulation. The dispensing of medicines in Côte d'Ivoire, if it is based on practices drawn from the

French public health code (compulsory prescription medicines, legislation on poisonous substances, etc.) is characterized by a lack of specific national regulations. *Decree No. 94-669 of 21 December 1994 on the conditions for the registration and dispensing of medicines was* limited, as far as dispensing was concerned, to setting out the principles of substitution and deconditioning of medicines, which had already been set out in *Decree No. 94-456 of 25 August 1994 laying down derogatory conditions relating to the rules for the dispensing of prescriptions for pharmaceutical products by pharmacists*. This emphasis on substitution and unpackaging could be explained by the need to promote generic drugs after the devaluation of the CFA franc in 1994 in order to promote access to health care for the population.

Substitution is the ability for the pharmacist to deliver a pharmaceutical product other than the one prescribed, provided that the product to be served is composed of the same active ingredients, has the same galenic form, the same dosage, the same route of administration as the prescribed medicine with a lower price; in the absence of a formal opposition from the prescriber

Deconditioning is the option offered to the pharmacist to dispense the medicinal product in fractionated quantities, out of its outer packaging, whenever the packaging used offers sufficient guarantees of conservation, hygiene and safety for the user, within the limits of an appropriate therapeutic use.

However, poorly applied substitution and deconditioning, especially in circumstances of poor compliance, can lead to antimicrobial resistance due to such misuse.

In 2015 the Ivorian legislator took legal provisions on the dispensation of medicine in Côte d'Ivoire. According to *the law n°2015-533 of July 20, 2015 on the practice of pharmacy*, *Dispensing* is understood to be the pharmaceutical act that consists of:

- The dispensing of a drug or pharmaceutical product associated with the analysis of the prescription or order concerning them;
- The provision to the patient of the information necessary for the proper use of medicines and pharmaceutical products as well as acts related to preventive advice and health education;
- The provision of information useful for the proper use of a medicinal product whose dispensing is not legally subject to the need to provide a medical prescription.

Antimicrobials are among the medicines concerned by the regulation of poisonous substances. Their delivery or dispensing is therefore subject to a prescription from a qualified person. However, in practice, there is a non-rational use of preventive microbial characterized by an insufficiency, or even an absence of the prescription, even though it is a prerequisite, and a dispensing to the detriment of good practices.

Thus, there are significant gaps in national legislation and regulations regarding the dispensing of drugs and antimicrobials in particular. Such specific regulation would optimize the rational use of antimicrobials in human medicine.

2.1.4.2. Advertising of antimicrobials for human use

Advertising for medicines and pharmaceutical establishments is now regulated by *Decree No. 2016-717 of 14 September 2016 regulating the advertising of medicines, other health products and pharmaceutical establishments*, a decree issued in application of *Decision No. 10/2010/CM/WAEMU adopting guidelines for the control of information and advertising of medicines to health professionals in WAEMU Member States.* Each

member state is obliged to bring its national provisions governing the advertising of medicines into line with the community standard.

The regulations stipulate that any advertising of a health product is subject to an administrative authorization issued by the Minister of Health.

Decree No. 2016-717 of 14 September 2016 distinguishes between two types of drug advertising: that aimed at the general public and that aimed at health professionals, in particular those called upon to prescribe or deliver drugs.

Advertising to the general public is prohibited unless a special dispensation is granted by the Minister of Health after consultation with the Conseil Supérieur de la Publicité. Antimicrobials are no exception to this rule.

The WHO has denounced the numerous practices that contravene the ethical criteria for the promotion of medicines in Africa. Studies have shown that more than 50% of medicines sold on the continent lack the information (very serious side effects) necessary for doctors to use them safely and effectively, that advertisements contain inaccuracies in indications, side effects or contraindications and that medical sales representatives are silent on side effects.

In Côte d'Ivoire, existing pharmaceutical documentation and regulatory texts governing the pharmaceutical sector are not sufficiently disseminated in the field. This is compounded by the absence of a specific public information and awareness programme.

In addition, the DPML has developed internal procedures for handling imports of promotional materials. However, in the absence of an information framework independent of the public authorities and a capacity to monitor activities in the field (prescribers and dispensers), the medical visit is accused of conveying biased, non-objective information and fuelling the illicit market for street drugs.

Finally, the information system for the rational practice of traditional medicine as well as the control and monitoring of the sector's activities is embryonic. This results in uncontrolled advertising, incentives and advertising of miracle products for diseases that are major public health concerns *(Source: National Pharmaceutical Policy, 2009).*

2.2. ANIMAL SECTOR

The legislative and regulatory texts relating to the animal health sector, although presenting provisions specific to Côte d'Ivoire, are harmonised with those of the international agreements that the country has ratified. These include sanitary and phytosanitary agreements, *Codex Alimentarius* standards, OIE guidelines on veterinary legislation, health codes for terrestrial and aquatic animals and ECOWAS and WAEMU community texts. These laws and regulations include animal health and welfare, food safety, prevention and control of zoonotic infections, epidemiological surveillance of animal diseases, registration, inspection and monitoring of veterinary drugs.

This community regulation of veterinary drugs is completed at the national level by the *law n°96-561 of July 25, 1996 relating to veterinary pharmacy and its decree of application the decree n°2001-487 of August*

09, 2001 carrying modalities of application of the law n°96-561 of July 25, 1996 and other regulatory texts relating in particular to the good pharmaceutical practices as regards veterinary drugs in particular the *decree n°35/MIPARH of August 9, 2007 fixing the rules of good practices of manufacture, importation and distribution of veterinary drugs*.

2.2.1. ORGANISATION OF VETERINARY SERVICES

The World Trade Organization (WTO) formally recognizes the responsibility of the OIE to develop standards and recommendations for international reference in the field of animal health and zoonoses, in accordance with the Agreement on the Application of Sanitary and Phytosanitary Measures.

Under this agreement, Côte d'Ivoire, as a WTO member, must align its veterinary health requirements with the recommendations contained in the Terrestrial Animal Health Code.

Chapters 6.7, 6.8, 6.9 and 6.10 of the Code provide guidance and guidelines to be implemented by the veterinary authorities of each Member State to ensure and guarantee respectively :

- Harmonisation of national surveillance and monitoring programmes for antimicrobial resistance in veterinary medicine ;
- Monitoring the quantities of antimicrobial agents used in food-producing animals ;
- Responsible and prudent use of veterinary antimicrobial agents ;
- Analysis of the risk of resistance to antimicrobial agents resulting from their use in animals.

The above-mentioned health measures must be applied by the veterinary authorities in Côte d'Ivoire.

In accordance with community texts (*regulation C/REG.21/11/10 on the harmonization of the structural framework and operational rules on plant, animal and food safety in the ECOWAS region and Regulation No. 007/2007/CM/UEMOA on plant, animal and food safety in the WAEMU.*), the veterinary authority in each Member State is the veterinary service that is competent to implement in the country the animal health measures, procedures, supervision and/or issuance of international veterinary certificate according to the forms adopted by the ECOWAS Commission and to supervise or audit their application.

In view of the above, the veterinary authority in Côte d'Ivoire is the Direction des Services Vétérinaires of the Ministère des Ressources Animales et Halieutiques.

It is in this capacity that the OIE delegate in Côte d'Ivoire is the Director of Veterinary Services. The Directorate of Veterinary Services is in charge of issues related to animal health and works closely with eight focal points recognised by the OIE, in particular the OIE focal point for products for veterinary use represented by the Deputy Director of Pharmacy and Veterinary Medicines.

At the national level, the structuring of the animal health sector is done around the veterinary services which are placed under the direction and control of the DSV. According to *Decree No. 2014-552 of 1er October 2014 on the organization of the Ministry of Animal Resources and Fisheries*, the DSV is responsible, in particular, for coordinating all public and private activities aimed at improving animal health, animal welfare, food hygiene, and the quality and safety of animal and fishery products.

The MIRAH has a Border Veterinary Inspection and Control Service (SICOSAV). This service is responsible, at the level of ports (Abidjan and San Pedro), airports and land borders, for the sanitary

control of live animals and imported or exported animal products, and for the issuance of sanitary certificates authorizing the release for consumption or export of animal products or products of animal origin. The missions and functioning of the SICOSAV are set by *the decree 069 MIPARH of July 01, 2010 fixing the practical measures of exercises of the sanitary police in borders.*

The Veterinary Services are organised into the following supervisory and support structures

2.2.1.1 National Agricultural Development Support Laboratory (LANADA)

LANADA is a public establishment with administrative character created in 1991. Its attributions, organization and functioning were revised by *Decree No. 2013-329 of 22 May 2013 on the attributions, organization and functioning of the National Laboratory for Support to Agricultural Development called LANADA.*

Within the framework of the preservation and improvement of the quality of animal and plant productions or of their production conditions, LANADA's missions are to provide logistical support to the promotion and control actions carried out by the State services, to carry out public or private research programmes or to participate in them, as well as to provide its expertise to the private sector in the form of service provision

In this capacity, LANADA is responsible in particular for :

- Study, develop and implement the necessary methods and means ;
- To provide the competent authorities with the technical elements necessary for the performance of their tasks with regard to texts relating to ;
  - Hygiene and quality of food products ;
  - The quality of agricultural products
  - Animal health, nutrition and reproduction ;
  - Veterinary pharmacy and phytosanitary products
  - Environmental protection
- Identify ways to control, monitor and improve the above areas.

LANADA has five (5) laboratories: the Central Veterinary Laboratory of Bingerville (LCVB), the Central Laboratory for Food Hygiene and Agro-Industry of Abidjan (LCHAI), the Central Laboratory of Agrochemistry and Ecotoxicology of Abidjan (LCAE), the Regional Laboratory of Bouaké (LRB) and the Regional Laboratory of Korhogo (LRK).

Regulation n°04/06/CM/UEMOA instituting a network of laboratories in charge of quality control of veterinary drugs in the UEMOA zone adopted on 23 March 2006 entrusts this network with ensuring the quality of veterinary drugs marketed in the UEMOA zone in order to guarantee their efficacy and safety

In this regard, the LCVB under LANADA has been designated by the WAEMU Commission as the laboratory in charge of quality control of veterinary vaccines in accordance with *decision n°010/2009/COM/UEMOA on the designation of laboratories of the network in charge of quality control of veterinary drugs within WAEMU*

2.2.1.2. National Agency for Support to Rural Development (ANADER)

ANADER is a mixed company created by *Decree No. 93-777 of 29 September 1993* with the mission of supporting the plant, animal and fishery production sectors, promoting the professionalism of

production and increasing quality, productivity and income. At the veterinary level, ANADER's agents are in charge of health information and sensitization of breeders. They are called upon during mass vaccination campaigns and for the management of major health crises.

2.2.1.3. Veterinary Service of the Armed Forces

The Armed Forces Veterinary Service (SVA) is in charge of food hygiene in the Ivorian army barracks and the sanitary monitoring of the animals of the Defence and Security Forces of the National Gendarmerie. The SVA collaborates closely with the MIRAH and in particular the DSV, although there is no formal framework defining the contours of this collaboration for the moment.

2.2.1.4. Municipal Veterinary Services

The Municipal Veterinary Services (MVS) are created within the framework of the transfer of certain competences from the State to the local authorities in accordance with *Decree n°2005-250 of 7 July 2005 fixing the modalities of application of law n°200-208 of 7 July 2003 on the transfer and distribution of competences from the State to the local authorities.*

They are in charge of the health inspection of animals intended for slaughterhouses, the fight against illegal slaughter, the fight against dog rabies, the inspection of cold stores with a capacity of 50 tonnes or less, the control of the hygiene of collective catering, the health inspection of markets and the conduct of epidemiological surveys on contagious diseases. The SVMs collaborate with the DSV, to which they submit an annual report.

2.2.1.5. Regional Directorates, Departmental Directorates and Breeding Stations

MIRAH is divided into 19 Regional Directorates, 58 Departmental Directorates and 174 Livestock and Fisheries Resources Posts (PERH).

These directorates and posts are called "external services of MIRAH". The missions and functioning of these services are set by *Order n°035/MIPARH/CAB of 01 April 2008 fixing the attributions and organization of the regional directorates, departmental directorates, livestock and fishery resources posts and entry posts of the Ministry of Animal Production and Fisheries Resources*. These services are in charge of coordinating all the activities of the MIRAH in the field. On the veterinary front, they are responsible for the inspection of animal products and products of animal origin, the management of anti-rabies centres, the control of livestock movements and, in some cases, the surveillance of animal diseases.

2.2.1.6. Private veterinary sector

The private veterinary sector is made up of veterinary doctors working in urban or rural areas. In accordance with Article 21 of *Law 88-684 of 22 July 1988 creating the National Order of Veterinarians,* no one may practice veterinary medicine, surgery and pharmacy unless he or she is registered with the Order of Veterinarians. In 2019, 245 veterinarians were registered with the Ordre des Vétérinaires.

The private veterinary sector is made up of veterinary medicine importers and private veterinary practitioners. In 2019, there are 30 main veterinary practices and 12 secondary veterinary practices throughout the country.

These private veterinarians are active in the distribution of veterinary drugs, animal care and the monitoring of livestock. They also carry out vaccination campaigns within the framework of a health

mandate granted by the DSV in accordance with *Decree n°95-536 of 14 July 1995 on the veterinary health mandate***.**

The instruction of their file of installation in private clientele is carried out by the DSV in accordance with the decree n° 2014-552 of 1er October 2014 relating to the organization of the Ministry of Animal and Halieutic Resources.

Furthermore, the activities of private veterinarians are governed by *law n°63-323 of 25 July 1963 on animal health in the Republic of Côte d'Ivoire, law n°88-683 of 22 July 1988 instituting a Code of Ethics for Veterinarians, law 96-561 of 25 July 1996 on veterinary pharmacy, decree n°95-536 of 14 July 1995 on the veterinary health mandate*

2.2.2. MARKETING AUTHORISATION, LICENSING AND INSPECTION

2.2.2.1 Marketing authorisations for veterinary medicinal products

According to article 1 of *law n°96-561 of 25 July 1996 on veterinary pharmacy*, "*Veterinary medicine means any substance or composition having curative or preventive properties with regard to animal diseases, as well as any product that can be administered to an animal with a view to making a medical diagnosis or to restoring, correcting or modifying its organic functions*". This definition is confirmed by *Regulation n°02/2006/CM/WAEMU laying down Community procedures for the marketing authorisation and supervision of veterinary medicinal products and establishing a Regional Committee for Veterinary Medicinal Products*.

The requirement for marketing authorisation before any veterinary medicinal product is placed on the market has always been a constant in the organisation of animal health systems in our countries. Thus, the *law n°96-561 of July 25, 1996 relating to* veterinary *pharmacy* already indicated that no veterinary drug can be delivered to the public if it did not obtain beforehand an authorization of marketing given by the WAEMU. This prerequisite is reaffirmed by article *13* of *Regulation n°02/2006/CM/UEMOA* and article *3 of Directive C/DIR.1/11/10 relating to veterinary pharmacy of UEMOA.*

2.2.2.1.1. Legislative and regulatory framework for obtaining marketing authorisations for veterinary medicinal products

The legislative and regulatory framework for the approval of veterinary drugs complies with the principles of safety and efficacy set out by international organizations (OIE, FAO, WHO) in terms of issuing MA for veterinary drugs. In Côte d'Ivoire, the approval of veterinary drugs is carried out within the overall framework of the harmonization of pharmaceutical regulations in the WAEMU community through a centralized procedure involving all member states of the union.

At the national level, the legislation provided that no veterinary drug could be made available to the public unless it had first received a marketing authorisation issued by the competent authorities after the opinion of a technical committee. Since the adoption of community texts, the provisions of these texts (WAEMU) have been implemented in the context of the registration of veterinary drugs. It is by virtue of the principle of primacy of international texts over national texts that national provisions have been replaced by community standards.

Indeed, following the *Regulation n°02/2005/CM/UEMOA relating to the harmonization of pharmaceutical regulations in WAEMU Member States* The WAEMU Commission successively issued (i) *Regulation*

n°02/2006/CM/UEMOA establishing community procedures for the marketing authorization and supervision of veterinary drugs and setting up a regional committee on veterinary drugs, (ii) *Regulation n°03/2006/CM/UEMOA establishing fees in the field of veterinary drugs within WAEMU*, (iii) *Regulation n°04/2006/CM/UEMOA instituting a network of laboratories in charge of quality control of veterinary drugs within the UEMOA zone*, *(iv) Regulation n°08/2010/CM/UEMOA amending Regulation n°02/2006/CM/UEMOA laying down Community procedures for the marketing authorisation and supervision of veterinary medicinal products and instituting a regional committee for veterinary medicinal products* and (v) *Directive n°07/2006/CM/UEMOA relating to veterinary pharmacy.*

In addition, WAEMU decisions provide more details concerning the practical modalities of MA applications:

- *Decision n°009/2009/COM/UEMOA setting out the procedures for submitting an application for a community marketing authorisation;*
- *Decision n°010/2009/COM/WAEMU on the designation of laboratories of the network in charge of quality control of veterinary drugs within WAEMU;*
- *Decision n°011/2009/COM/UEMOA establishing the list of minor and major amendments to marketing authorization dossiers.*

2.2.2.1.2. Procedure for obtaining marketing authorisations for veterinary medicinal products

This procedure will be described in the following manner:

- Application for marketing authorization

The application for marketing authorisation shall consist of the following documents:

- a letter of introduction of the application ;
- the completed marketing authorisation application form ;
- the marketing authorisation application file containing all the experimental and analytical data proving the quality, safety and efficacy of the proposed medicinal product.

The file is sent in folders or deposited at the Permanent Secretariat of the Regional Veterinary Medicines Committee in Ouagadougou (Burkina Faso).

- Evaluation of the MA application file

The administrative evaluation of the file is carried out by the Permanent Secretariat of the Regional Veterinary Medicines Committee which evaluates the corresponding fee in accordance with the provisions of *Regulation n°03/2006/CM/UEMOA instituting fees in the field of veterinary medicinal products within the UEMOA.*
After payment of the fee, the permanent secretariat shall examine the administrative admissibility of the application.

The notification to the applicant of the administrative validity of the application marks the beginning of the technical assessment procedure.

The technical evaluation of the MA application file by the Regional Committee for Veterinary Medicinal Products is done according to the modalities defined by *Regulation n°02/2006/CM/WAEMU laying down Community procedures for the marketing authorisation and supervision of veterinary medicinal products and establishing a Regional Committee for Veterinary Medicinal Products*. It cannot exceed two hundred and forty (240) days from the submission of a valid application (Article 30).

Experts are appointed in a personal capacity, based on their competence and scientific experience, particularly in the evaluation of veterinary drugs. The selection process of the experts is managed by the WAEMU Commission.

2.2.2.1.3. Granting or refusal of marketing authorisation and renewal

- Granting of the MA

The marketing authorisation shall be granted to a natural or legal person, referred to as the marketing authorisation holder, when all the regulatory requirements are met for a given quantitative and qualitative composition and pharmaceutical form.

The marketing authorization is issued to a holder established in one of the member states of the Union. If not, he appoints a local representative in charge of monitoring the procedures for obtaining marketing authorisation, pharmacovigilance, complaint management, batch monitoring and withdrawal if necessary.

- Validity and renewal of the MA

The MA is issued for a period of five (5) years and is then renewable by five-year periods according to the modalities set out in article 36 of *Regulation n°02/2006/CM/WAEMU laying down Community procedures for the marketing authorisation and supervision of veterinary medicinal products and establishing a regional committee for veterinary medicinal products*. The MA may be amended or transferred at the request of its holder in accordance with the procedures set out in Articles 34, 35 and 37 of *Regulation n°02/2006/CM/WAEMU*.

Resulting from a centralized procedure, the MA of veterinary drugs is valid from the outset in the eight (8) member states of the Union. WAEMU is responsible for notifying all member states of this approval, and they draw all the consequences, particularly with regard to its validity during the regulatory period. The list of MAs for veterinary drugs in WAEMU is available at the Directorate of Veterinary Services, with 380 veterinary drugs with MAs (DSV, 2020).

- Refusal of marketing authorization

The marketing authorisation shall be refused if it appears that either:

- the application and the accompanying file are not in conformity ;
- the veterinary medicinal product does not have the quantitative or qualitative composition declared;
- it is harmful under the conditions of use specified in the application package;
- the announced therapeutic effect is lacking on the target animal species;
- the placing on the market of a veterinary medicinal product is likely to seriously compromise the protection of human or animal health ;

- for medicinal products intended for administration to food-producing animals intended for human consumption, that the withdrawal period indicated in the dossier is insufficient to ensure that foodstuffs obtained from the treated animal do not contain residues at levels likely to be harmful to the consumer or that it is insufficiently justified ;
- the veterinary medicinal product is presented for a use prohibited on the territory of the WAEMU.

2.2.2.2 Veterinary Licensing and Inspection

Until 1984, the "medicines" sector was managed exclusively by the Société pour le Développement des Productions Animales (SODEPRA), a former company that supervised livestock farmers. Veterinary drugs were distributed by extension agents. The liberalization of this sector occurred in 1992 with the installation of importing and distributing wholesalers and private veterinary practitioners. These actors are involved in the official market for veterinary drugs. In addition to this official market, a parallel distribution circuit for veterinary drugs has developed.

2.2.2.2.1. Official distribution channel for veterinary medicinal products

Public and private actors are involved in the distribution of veterinary drugs in Côte d'Ivoire (Figure 4).

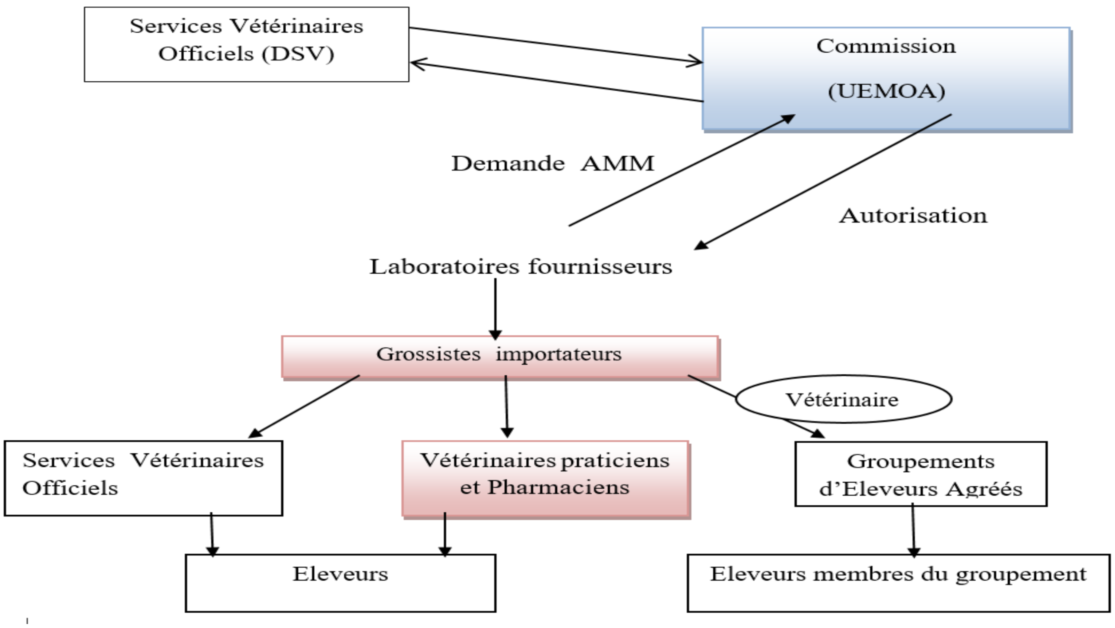

Figure 4: Official distribution channel for veterinary drugs in Côte d'Ivoire (NGOCHO, 2018)

- Public actors

The distribution of veterinary drugs on behalf of the State is carried out by the National Veterinary Pharmacy, which is a service of the Sub-Directorate of Pharmacy and Veterinary Drugs (SDPMV) of the DSV.

- Private actors

The majority of veterinary products are distributed through importing wholesalers, veterinary practices and pharmacies.

Veterinary drug wholesalers are responsible for the importation and wholesale distribution of veterinary drugs in Côte d'Ivoire. This activity is governed by *Law n°96-561 of 25 July 1996 on veterinary pharmacy*.

As of 2019, Côte d'Ivoire has registered 12 veterinary drugs wholesaler importers including 2 dispensing pharmacists (Directory of veterinary drugs wholesaler importers, DSV 2019). Eleven (11) of these wholesalers are based in Abidjan and one (01) in Agnibilékrou. These importers sourced from 37 laboratories in 14 countries on 4 continents with import volumes as follows:

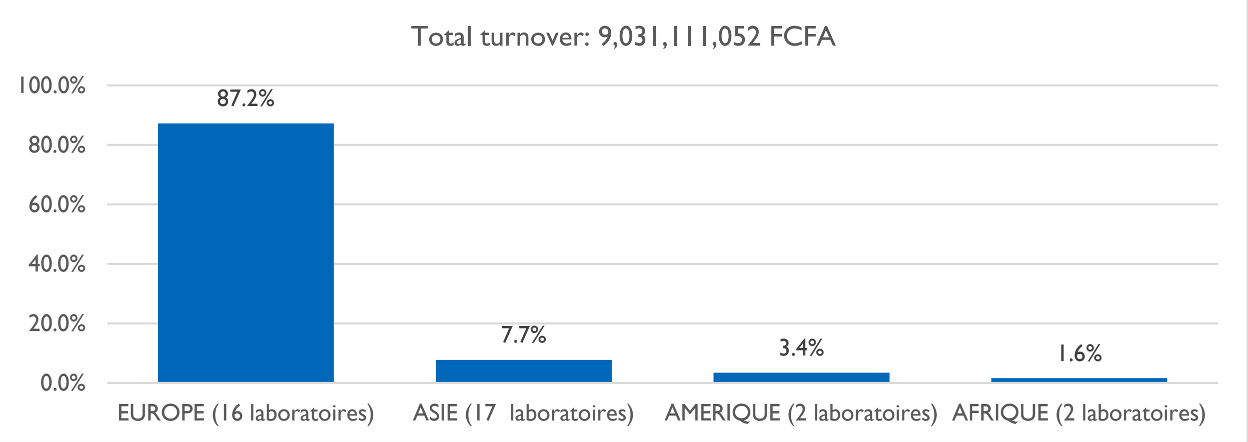

Figure 5: Import volume and source of veterinary drugs in Côte d'Ivoire in 2019

In Côte d'Ivoire, there is no manufacturing facility for veterinary drugs.

Veterinary practices are run by private veterinary practitioners who carry out, among other things, the retail distribution of veterinary medicines and the monitoring of livestock.

In Côte d'Ivoire, the establishment of livestock farms is governed by *Decree No. 98-70 of 13 February 1998 laying down the general rules for the establishment of livestock farms*. These farms are created by simple declaration to the services in charge of animal resources for farms located in the rural land area and prohibited for those to be located inside urban areas except by derogation of a commission called "municipal commission of livestock farms". In both cases, there is a regulatory deficiency related to the technical and sanitary supervision of livestock farms by veterinarians. This inadequacy could result in the inappropriate use of antibiotics.

The epidemiological surveillance system for animal diseases in Côte d'Ivoire provides for these farms to be monitored by private veterinarians and by the official services of the Ministry in charge of animal and fisheries resources. However, the regulatory mechanism that sets out the terms of application of the above-mentioned decree is inadequate, with the result that farmers do not consult private veterinarians in their breeding areas.

The lack of veterinary monitoring of these farms leads to poor biosecurity and vaccination practices. Thus, farmers use antimicrobials in an uncontrolled and abusive manner, especially since they can obtain them either without a prescription from retail outlets or directly from importing wholesalers. These bad

practices would favour the development of antimicrobial resistance and therefore negatively affect public health.

It would therefore seem appropriate to fill this regulatory gap in order to oblige farmers to contact veterinary practices and the official services of the Ministry in charge of animal health and public hygiene in the context of the implementation of animal disease epidemiosurveillance.

In pharmacies, the dispensing of veterinary medicines by pharmacists in pharmacies is governed by *law n°96-561 of 25 July 1996 relating to veterinary pharmacy.*
A survey conducted by Ngocho in 2018, showed that pharmacists are selling veterinary drugs directly to farmers without prescription.

Similarly, the technical specifications, including MA, pharmacovigilance, AMR monitoring, and withdrawal periods for veterinary drugs authorized for distribution are not known.

With regard to approved livestock groups, *Act No. 96-561 of 25 July 1996 on veterinary pharmacy* provides that, in the context of the implementation of health programmes whose execution is placed under the effective supervision and responsibility of a veterinary doctor, livestock groups may be approved for the retail sale to their members of veterinary medicines necessary for the implementation of these programmes. At present, this provision is not yet applied.

2.2.2.2.2. Unofficial distribution channel for veterinary drugs

The parallel market concerns the illegal (Figure 6) and unauthorized sale of veterinary drugs. The circulation of veterinary drugs in the parallel market is anarchic and uncontrolled. For this reason, this circuit constitutes the potential entry route for poor quality veterinary drugs on the African continent in general and in Côte d'Ivoire in particular *(ASSOUMY, 2009)*.

OULAI (2004) had also estimated that this circuit is driven by non-professionals, notably livestock traders who obtain their supplies in Burkina Faso, Ghana and Mali, sometimes from national wholesalers and distributors, and who mainly supply individual herders.

It is within this framework that WAEMU has embarked on a vast program to fight against the anarchic circulation of veterinary drugs through the implementation of the Union's Agricultural Policy (UAP).

To this end, a veterinary drug monitoring system has resulted in two Veterinary Drug Market Sanitation Campaigns (CAMMVET) carried out in 2014 and 2015. These campaigns focused on raising the awareness of actors to the phenomena of illegal distribution of medicines.

Since 2016, the enforcement phase has come into effect and covers:

the seizure of any illegal veterinary pharmaceutical product present on the national territory;

the closure of all listed outlets selling illicit veterinary pharmaceuticals;

- the denunciation, prosecution and imprisonment of all perpetrators and accomplices in the sale and distribution of fraudulent veterinary pharmaceutical drugs.

In 2019, control and repression operations carried out by the Directorate of Veterinary Services in collaboration with the Defence and Security Forces resulted in the seizure and destruction of approximately 2.7 tonnes of veterinary drugs.

Similarly, pharmacies and veterinary clinicians distribute the veterinary drug to farmers without prescription *(NGOCHO, 2018).*

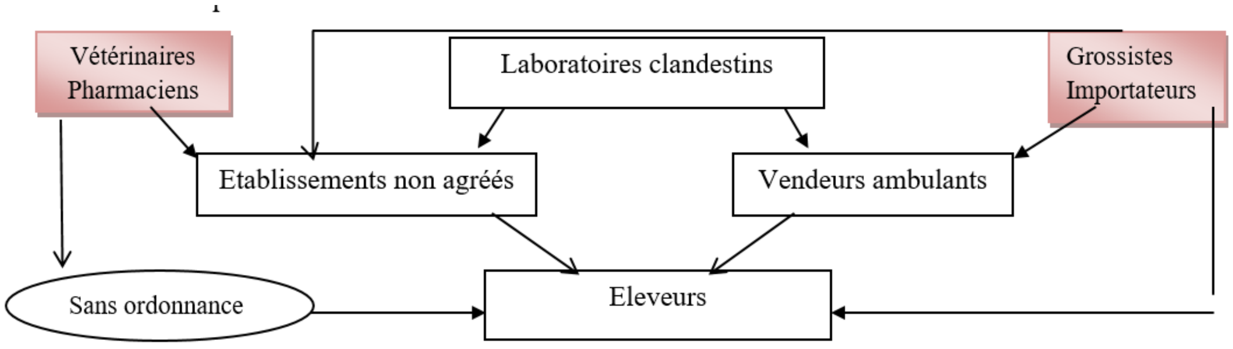

Figure 6. Illicit distribution channel of veterinary drugs in Côte d'Ivoire (NGOCHO, 2018)

2.2.3 VETERINARY SERVICES INSPECTION

The veterinary services should have an inspection body. In addition, the regulatory framework should clearly define the composition of this body, the powers of the inspectors (sampling, access to documents, administrative sanctions, unannounced inspections, inspection targets, etc.) and the obligations of the inspectors (impartiality, independence, confidentiality, integrity).

The DSV ensures, through repeated or unannounced inspections, that national and Community administrative and technical legal requirements concerning veterinary medicinal products and veterinary pharmaceutical establishments are complied with.

The veterinary authorities shall recognise the inspections carried out by other Member States and shall provide each other with relevant information on the establishments. Upon reasoned request, the veterinary authority may ask for the communication of an inspection report or the results of a control carried out by the laboratory of another Member State (*Art.* 31 and 32 of *Directive n°07/2006/CM/UEMOA relating to veterinary pharmacy*).

2.2.4 POST-MARKETING SURVEILLANCE AND PHARMACOVIGILANCE

Although UEMOA Member States encourage veterinary doctors and other animal health professionals to report to the veterinary authority any adverse effect on humans or animals that may be attributed to a veterinary drug as provided for in *Directive N°07/2006/CM/UEMOA relating to veterinary pharmacy in its article 33*, it can be said that pharmacovigilance is not really applied in Côte d'Ivoire. However, a good surveillance program includes quality control of veterinary drugs placed on the market by an official laboratory. This programme should be based on risk analysis which depends on the category of the product (antibiotics, antiparasitics, medicated feeds), the most used product (volume of sales), the existence of counterfeit products, biological products used in the control of zoonoses and regulated diseases.

There is a need to carry out activities to analyse and control the origin of medicines, obtain reliable data on imports, trace their circulation, and evaluate the quality of products in circulation.

The online sale of veterinary drugs poses a difficulty in terms of traceability insofar as it escapes control/surveillance programmes and it can be extremely difficult to identify the actors involved. There are no regulations on the e-commerce of veterinary drugs in Côte d'Ivoire.

2.2.4- GOOD SUPPLY MANAGEMENT POLICIES AND ANTIMICROBIAL GUIDELINES

According to statistics from the Directorate of Veterinary Services, a total of 115 tons of antimicrobial-containing drugs were officially imported into Côte d'Ivoire in 2019. The three most represented antimicrobial families are quinolones (33 tons), sulfonamides (25 tons) and tetracyclines (20 tons). In addition, 630,625,104 doses of veterinary vaccines, 527,765 kg of solid veterinary products and 298,157 litres of liquid veterinary products were imported in 2019.

In the same year, 2.7 tonnes of fraudulent drugs were seized by the DSV as part of the Veterinary Drug Market Sanitation Campaign (CAMMVET) and then destroyed by incineration in March 2020 at the Pasteur Institute of Côte d'Ivoire. Under these circumstances, the surveillance system for veterinary drugs placed on the market must be strengthened. The monitoring of supplies and the quality of imported drugs should therefore be a major concern for the authorities.

There are institutions at the national, regional and international levels responsible for the proper management of antimicrobial supplies in animal health.

At the international level, we can mention the OIE, the WHO and the FAO.

The regional and sub-regional level is represented by the Inter-African Bureau for Animal Resources of the African Union (AU-IBAR), WAEMU, ECOWAS.

At the national level, there is the National Order of Veterinarians of Côte d'Ivoire (ONVCI), the Group of Veterinary Wholesalers (GVG), the Association of Private Veterinary Practitioners of Côte d'Ivoire (AVPPCI), the Directorate of Veterinary Services (DSV) through the sub-directorate of pharmacy and veterinary medicine and all the laboratories involved in the AMR surveillance network (LANADA, IPCI, LNSP ...) for the quality control of imported antimicrobial agents

In Côte d'Ivoire, there are no specific regulations related to the use of antimicrobials in animal health and veterinary pharmacovigilance. In addition, the regulations related to the monitoring of supply are insufficient.

However, certain legislative and regulatory texts provide guidance on AMR, in particular *law n°96-561 of 25 July 1996 on veterinary pharmacy*.

At the international level, the Terrestrial Animal Health Code, in particular Chapters 6.7, 6.9 and 6.10, deals respectively with the harmonisation of national antimicrobial resistance surveillance and monitoring programmes; the responsible use of antimicrobial agents in veterinary medicine; and the analysis of the risks of antimicrobial resistance resulting from the use of antimicrobial agents.

However, there is no permanent, formalized monitoring by the competent authority for AMR in the animal sector.

2.2.6- PRESCRIPTION, DISPENSING AND USE OF VETERINARY ANTIMICROBIAL DRUGS

According to Chapter 3.4 of the Terrestrial Animal Health Code, veterinary legislation should provide a framework for dealing with the establishment of rules for prescribing and dispensing veterinary medicinal products and biological products.

According to Article 27 of *Law No. 96-561 of 25 July 1996 on veterinary pharmacy* in terms of retailing of veterinary drugs, there are :

- Medicinal products which do not present any real danger to the animal, the user or the consumer ;
- Veterinary medicinal products that present a danger to the animal, the user or the consumer. Their delivery free of charge or against payment is subject to the drafting, by a veterinary doctor, of a prescription to be given to a user. These medicines are the following:
  - virulent materials and products of microbial origin for use in diagnosis, prevention and treatment of animals ;
  - substances of organic origin intended for the same purposes, with the exception of those containing only chemically known principles;
  - estrogens and anabolic hormones ;
  - toxic and poisonous substances ;
  - products which may remain as toxic or dangerous residues in foodstuffs of animal origin ;
  - products whose effects are likely to cause a contravention of the legislation on fraud ;
  - products which may interfere with the sanitary control of foodstuffs from the animals to which they have been administered.

The National Order of Veterinarians has instituted a standardized and authenticated prescription pad (sticker) distributed to clinical veterinarians.

KILI (2016) showed that 64.7% of pig farmers used antibiotics as a preventive measure in the absence of pathological signs. It also showed that the main antibiotic molecules used in pig farming are penicillin, streptomycin, oxytetracycline, colistin, trimethoprim, amoxicillin, tylosin, sulfonamides, enrofloxacin, marbofloxacin and gentamicin. As for antiparasitics, the main molecules used are levamisole, albendazole, niclosamide, piperazine, amprolium, toltrazuril, ivermectin, delthamethrin, cypermethrin and amitraz.

In a survey of 95 farms on health management and medical practices in modern poultry farming in the outskirts of Abidjan conducted in 2013, BITTY (2013) observed that 95% of farmers measured powdered medicines (antibiotics, anticoccidials, disinfectants) with teaspoons. This poor practice very often leads to under or over dosing. In addition, 73% of the poultry farmers surveyed acknowledged that they did not respect the waiting period for antimicrobials used before marketing or consuming the farm by-products.

According to ABIOLA et al (2014), the prevalence rate of veterinary drug residues in food of animal origin is less than 1% in Europe, while it reaches 94% in some African countries. The presence of antibiotic residues in food of animal origin can be the cause of allergies, cancers, changes in intestinal flora, bacterial resistance and inhibition of fermentation phenomena in the dairy industry.

Harmonization of regulations in Africa in general and in Côte d'Ivoire in particular can reduce the circulation of prohibited antibiotics and allow the implementation of a control and monitoring plan for veterinary drug residues in food of animal origin.

According to JIM O'NEILL (2014), antimicrobial resistance will be the leading cause of death well before cancer from 2050.

2.3. ENVIRONMENTAL INDUSTRY

The policy, legal and institutional framework for pesticide management is relatively well developed. The policy framework covers all policy and strategy documents relating to the marketing and use of pesticides. These policy documents concern the National Agricultural Investment Plan (PNIA 2). The main actors involved in the sector are the Direction de la Protection des Végétaux, et du Contrôle Qualité (DPVCQ), the Laboratoire National d'Appui au Développement Agricole (LANADA), the Centre Ivoirien Antipollution (CIAPOL), the Laboratoire National d'Essais de Qualité Métrologique et d'Analyses des Pollutions (LANEMA), Universities and research centres, the Pesticides Committee, agricultural support and advisory companies, Professional Agricultural Organisations (PAOs), as well as professionals in the phytosanitary sector and health and environmental NGOs.

At the legal level, Côte d'Ivoire relies on international conventions, supplemented by regional and national texts.

At the international level, these include the Stockholm Convention on Persistent Organic Pollutants (POPs) of 2001, the Rotterdam Convention on the Prior Informed Consent Procedure for Certain Hazardous Chemicals and Pesticides of 1998, the Bamako Convention on the Ban of the Import into Africa of Hazardous Wastes of 1991, the Basel Convention on the Control of Transboundary Movements of 1989, the Convention on Protection against Benzene Poisoning of 1971, the International Code of Conduct on Pesticide Management (Rome 2014).

At the regional level, the "Convention to support the strengthening of the food security, locust control and pesticide management system" signed by the Permanent Inter-State Committee for Drought Control in the Sahel (CILSS) and the West African Economic and Monetary Union (UEMOA) provides a framework for the harmonization of pesticide approval rules and procedures in West Africa and the Sahel.

With regard to national legislation, these are *Law No. 2015-532 of 20 July 2015 on the new Labour Code*, *Law No. 2014-18 of 24 March 2014 on the Mining Code*, *Law No. 96-766 of 3 October 1996 on the Environment Code*, *Law No. 98-755 of 23 December 1998 on the Water Code, Law n°98-651 of July 7, 1998 on the protection of public health and the environment against the effects of industrial, toxic and nuclear waste and harmful substances, Law n°81-640 of July 31, 1981 instituting the Penal Code, Law n°64-490 of December 21, 1964 on plant protection.* Phytosanitary protection is also governed by *Decree No. 89-02 of 4 January 1989 on the approval of the manufacture, sale and use of pesticides in Côte d'Ivoire; the Inter-ministerial Order n°509/MINAGRI/MEMIS of 11 November 2014* organizing the control of pesticides, inspection and sanitary, phytosanitary and quality control of plants, products of plant origin, agricultural products and

any other material likely to carry organisms harmful to crops, human and animal health at the entry and exit gates of the national territory.

2.3.1. ORGANISATION OF THE ENVIRONMENT SECTOR AL

For the coordination of its activities, the Ministry of Environment and Sustainable Development (MINEDD) is composed of two General Directorates and three structures under supervision.

These are, on the one hand, the Directorate General of the Environment and the Directorate General of Sustainable Development and, on the other hand, the Ivorian Office of Parks and Reserves (OIPR), the National Environment Agency (ANDE) and the Ivorian Anti-Pollution Centre (CIAPOL).

In addition, MINEDD has a project for the management of obsolete pesticides called Projet de Gestion de Pesticides Obsolètes en Côte d'Ivoire (PROGEP-CI).

2.3.1.1. THE IVORIAN ANTI-POLLUTION CENTRE

CIAPOL, established by *Decree No. 91-662 of 9 October 1991*, has as its main mission the analysis of natural waters, waste and residues, as well as the evaluation of pollution and nuisances produced by industries. In accordance with articles 10 and 11 of its founding decree, CIAPOL has a central environmental laboratory which is responsible for

- to analyse, measure and follow the evolution of physico-chemical, chemical and microbiological pollution of all origins in natural waters (marine, lagoon, river, underground and meteoric), wastes (solid, liquid and gaseous) and residues within the framework of the implementation of the "National Observation Network of Côte d'Ivoire (RNO-CI)" or at the request of public or private bodies;
- assess noise nuisance ;
- to collect various national and international environmental data with the aim of improving the quality of water and the living environment;
- managing and interpreting analytical data on the state of the environment;
- formulate recommendations for quality objectives based on the various results;
- to ensure the quality and homogeneity of the results by a rigorous method at the level of the associated laboratories and by the practice of national and international intercalibration exercises;
- ensure that the national observation network of Côte d'Ivoire is integrated into the various international environmental monitoring networks;
- participate in environmental impact assessments of development projects;
- to participate in the expertise in case of accidental or chronic pollution and in the training and supervision of laboratory and service personnel in charge of environmental protection at national and regional level;
- to study at the technical level the approval files of private laboratories and services operating in the same fields of competence.

CIAPOL is an important actor in the evaluation of pollution caused by pesticides stored or produced by local industries. The recommendations formulated by its competent services make it possible to mitigate the effects of these chemicals on the environment and thus reduce the risks of intoxication of the populations.

2.3.1.2. PROJECT FOR THE MANAGEMENT OF OBSOLETE PESTICIDES IN COTE D'IVOIRE (PROGEP-CI).

- PROJECT DESCRIPTION

Côte d'Ivoire, has obtained funding from the Global Environment Facility (GEF) for the implementation of a national project to eliminate obsolete pesticide stocks. This work is part of the preparatory phase of the project whose endorsement was signed by the GEF in 2013.

- OBJECTIVES

The PROjet de GEstion des Pesticides obsolètes en Côte d'Ivoire (PROGEP-CI) aims to manage in an environmentally sound manner the stocks of obsolete pesticides and associated wastes on the Ivorian territory. Specifically, it is about:

- dispose of obsolete pesticide stocks without harming the environment and human health primarily through the safe disposal of these wastes;
- prevent the re-emergence of unwanted stocks of pesticides through the implementation of preventive measures;
- protect the health of people and the environment exposed to pesticides;
- Strengthen the capacity of Ivorian institutions for the sound management of chemicals in general and pesticides in particular.

- COMPONENTS OF THE PROJECT

PROGEP has four components:

- Component 1: Strengthening the institutional and regulatory framework ;
- Component 2: Reduction of obsolete pesticide stocks and associated waste;
- Component 3: Promotion of alternatives to pesticides ;
- Component 4: Project Management.

Component 1 aims to strengthen the government's capacity to manage, monitor and control pesticides throughout their life cycle with a particular focus on obsolete pesticides and their associated wastes.

Component 2 aims to design and implement a stockpile management system for obsolete pesticides that will include disposal and/or securing measures based on the results of the planned national inventory.

Component 3 identifies and popularizes alternatives to conventional pesticides, including the adoption of Integrated Pest Management (IPM) through a pilot intervention; it also aims to build the capacity of key stakeholders, and develop a communication and awareness strategy on the proper use of pesticides, alternatives and the impact of pesticides on human health and the environment

Component 4 aims to provide effective and efficient management support for the implementation of the project, including project management and coordination, monitoring and evaluation.

2.3.2. MARKETING AUTHORISATION, LICENSING AND INSPECTION

The Pesticides Committee of Côte d'Ivoire is an inter-ministerial committee established by *decree n°89-02 of 04 January 1989 relating to the approval, manufacture, sale and use of pesticides*. It is a framework for dealing with issues relating to pesticides. It is composed of representatives of several technical ministries in charge of Scientific Research, Health, Environment, Trade, Industry, Interior, Economy and Finance. The Pesticides Committee is set up by order of the Minister in charge of Agriculture; it meets when convened by its Chairman. The permanent Secretariat is provided by the Directorate of Plant Protection, Control and Quality (DPVCQ). The Pesticides Committee meets to examine all issues related to pesticides in Côte d'Ivoire, including

- registrations of new formulations;
- extensions of use of already registered formulations;
- Provisional Sales Authorizations (PSAs);
- renewals of approvals;
- transfers of ownership of registrations;
- changes in the composition of already registered formulations;
- changes in product names;
- approvals of plant protection professionals (plant protection companies, distributors or retailers and applicators);
- phytosanitary regulations.

The Committee gives opinions on the files presented and the Minister of Agriculture issues orders, depending on the case, to make the decisions enforceable. As part of the harmonization of pesticide registration rules and procedures in West Africa and the Sahel, the Permanent Inter-State Committee for Drought Control in the Sahel (CILSS) and the West African Economic and Monetary Union (UEMOA) signed, on November 07, 2014, a "Convention to support the reinforcement of the mechanism on food security, locust control and pesticide management". One of the major objectives of the convention is to contribute to a better management of pesticides, through the establishment of National Pesticide Management Committees (CNGP) in three 3 countries including Côte d'Ivoire. To this end, a decree on the creation, attributions, organization and functioning of the Ivorian Committee for Pesticide Management (CIGP) is being adopted

2.4. NATIONAL ANTIMICROBIAL RESISTANCE POLICY

2.4.1 GENERAL CONTEXT

Like many countries, Côte d'Ivoire has been a stakeholder in the Global Health Security Agenda (GHSA) or Global Health Security Program (GHSP) since 2015 which aims to help member states acquire the required capacities of the International Health Regulations (IHR, 2005) in order to effectively prevent, detect and respond against health threats. Since 2015, several national structures and NGOs have received funding and technical support from the U.S. government and other partners for the implementation of the global health security program for Côte d'Ivoire. According to a multisectoral collaborative approach called "one health" *(Situation Analysis Document for the development of the "one health" strategic plan of Côte d'Ivoire)*

The development of the National Health Security Action Plan (NSSAP) in 2018, supported by development partners and involving several ministries has enabled Côte d'Ivoire, to have an implementation guide for the International Health Regulations and Global Health Security. This plan takes into account the fight against AMR. From an institutional point of view, at the initiative of all the ministries involved in the Global Health Security program, *Decree No. 2019-293 of April 03, 2019 on the creation, attribution, organization and functioning of the "National One Health" platform* was taken. According to this decree, the National Platform "One Health" has the mission "to coordinate in a multisectoral and multidisciplinary approach, all health interventions to prevent, detect and respond to emerging and re-emerging diseases with pandemic potential".

In order to accomplish its mission, the national platform "One Health" is composed of the following bodies

- A watchdog committee: political and decision-making body of the platform, chaired by the Prime Minister, with the Minister in charge of health providing the secretariat and composed of ministers representing the sectors involved and international partners, notably the WHO, the OIE, the FAO and the UN-Environment;
- a coordination committee, a multi-sectoral coordination body for the activities of the "one health" platform chaired by the Minister of Health and composed of central directors, representatives of member ministries and NGOs involved in the implementation of the Global Health Security Program;
- a multi-sectoral secretariat: a body responsible for leading the "one health" platform and composed of a permanent secretary, a communication officer, a human health officer, an animal health officer and two monitoring and evaluation officers;
- Technical Working Groups (TWGs), which are consultative bodies, both proactive and reactive, to address specific issues.

In addition, the National IHR Focal Point (NFP-ISR) is appointed and the name of the institution hosting the NFP-ISR is INHP. National reference centres for the fight against communicable diseases have been created by interministerial order at the Pasteur Institute of Côte d'Ivoire (IPCI).

It should be noted that all the sectoral strategic plans related to the "one health" approach are aligned with the National Development Plan (NDP 2016-2020) which focus on the health, agriculture, animal and fishery resources, water and forestry sectors. These framework documents include:

- The National Health Development Plan (PNDS 2016-2020);
- the Strategic Plan for the Development of Livestock, Fisheries and Aquaculture (PSDEPA 2014-2020);
- the National Sustainable Development Strategy (NSSD 2012-2027);
- National Biodiversity Strategy and Action Plan (2016-2020);
- the national waste management strategy and action plan (2015-2020) ;
- Côte d'Ivoire's forest preservation, rehabilitation and expansion policy (2018-2028);
- the National Agricultural Investment Programme (PNIA-2 2016-2025).

In the animal sector, the OIE conducted an initial evaluation mission of the Veterinary Services (VS) of Côte d'Ivoire in December 2006 using the OIE Performance of Veterinary Services (PVS) tool and with

reference to the provisions of the OIE Terrestrial Animal Health Code (Chapter 1.3.3. on the evaluation of VS and Chapter 1.3.4. on the guidelines).

This expertise was renewed in November 2011 and was based on 46 critical competencies articulated around 4 pillars which are: (i) physical and financial human resources; (ii) authority and technical capacity; (iii) interaction with stakeholders and (iv) market access.

Several ministries were involved in this study, which revealed that in Côte d'Ivoire, veterinary services exercise administrative and technical control over veterinary drugs and biological products, but do not implement quality standards covering most aspects of veterinary legislation (AMR focal point, AMR surveillance, quality control of vaccines and drugs, etc.), do not monitor the adverse effects of veterinary drugs (pharmacovigilance), and do not take appropriate corrective action in the event of non-conformity

The PVS survey followed the gap analysis survey conducted in March 2012 using the OIE's *GAP Analysis* tool, which was used to develop and validate the Programme de Mise aux Normes des Services Vétérinaires (PMNSV 2015-2020).

The AMR focal point in the animal sector is represented by the Deputy Director of Pharmacy and Veterinary Medicine. The institution that houses this focal point is the Directorate of Veterinary Services. In this regard, the DSV has initiated since 2013 the development of the *draft bill on the veterinary public health code in the Republic of Côte d'Ivoire*, which takes into account all the new provisions (OIE sanitary code, WAEMU Regulation, ...) relating to animal health, pharmacy and veterinary drugs. The said draft law was adopted by the Council of Ministers on April 22, 2020 and the resulting draft law was sent to the National Assembly. About twenty applicative texts relating to veterinary pharmacy will have to be taken by regulation.

The official laboratory supporting the Veterinary Services Directorate in AMR matters is LANADA.

2.4.2- GOVERNANCE OF THE FIGHT AGAINST RAM

The governance of AMR monitoring is linked to the Multisectoral Platform, which is itself made up of technical ministries attached to the Prime Minister's Office. A coordination committee ensures the link between the AMR Technical Working Group (TWG) and the multisectoral platform. The TWG is headed by a central national multisectoral coordination body called the Multisectoral Coordination Group on AMR (GMC-RAM). The national AMR Secretariat is provided by the Observatory of Microorganism Resistance to Antimicrobials in Côte d'Ivoire (ORMICI).

In a one health approach, the National Reference Centre for Antibiotic Resistance (CNR-RAM) of the Pasteur Institute of Côte d'Ivoire (IPCI) is the national Focal Point. Six (6) Multisectoral Technical Committees (MTC) are created based on the global strategic objectives identified to achieve the PAN-RAM. The MTC includes experts in technical specialties designated by the GMC-RAM and meets on an ordinary basis every two (2) months (ordinary bimonthly meeting). However, the MTC shall meet on an extraordinary basis when necessary. Figure 7 below presents the AMR multisectoral governance organization chart.

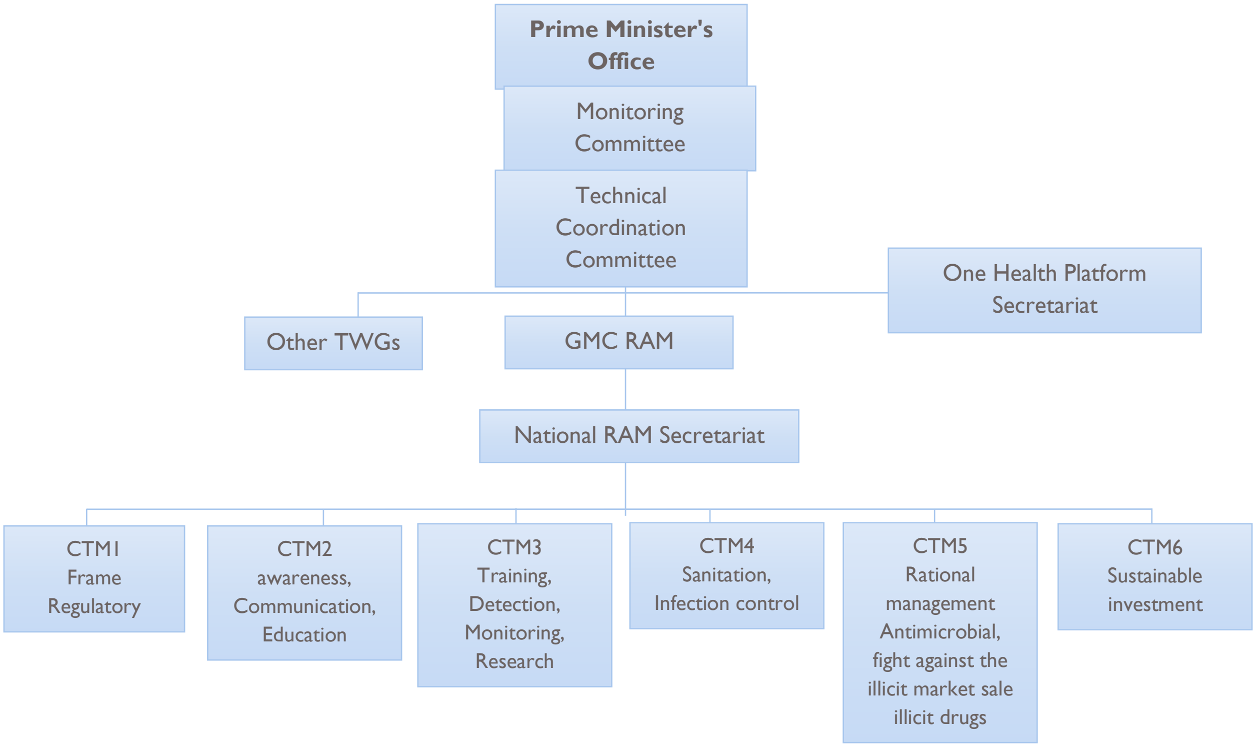

Figure 7: AMR Governance and Oversight

The roles and responsibilities of the key AMR governance bodies are outlined below.

2.4.2.1 Antimicrobial resistance focal point

The focal point for antimicrobial resistance is the Institut Pasteur of Côte d'Ivoire through the head of the National Reference Centre for Antimicrobials (CNR). The focal point of antimicrobial resistance plays the role of executive secretary of the national secretariat of AMR. The sectoral focal points appointed by their respective Ministries are part of the Observatory of Microorganism Resistance to Anti-infectives in Côte d'Ivoire (ORMICI).

The focal point is the primary contact for all AMR-related issues in the country. The focal point of the National Reference Centre for Antibiotic Resistance (NRC-AMR) is responsible for:

- Building sustainable partnerships and working nationally and internationally to combat AMR;
- Identify stakeholders and facilitate the formation of an inclusive Multisectoral Coordination Group (MCG);
- Lead and coordinate the development of a draft national action plan for AMR;
- facilitate and oversee the implementation, monitoring and evaluation of the NAP-RAM through the GMC.

2.4.2.2. Multisectoral Coordination Group (MCG)

The Multisectoral Coordination Group (MCG) on AMR is the central national coordination body. The GMC oversees and coordinates all AMR-related activities in all sectors. The members of the GMC are

representatives of different sectors and disciplines, in particular the sectors responsible for human health, animal health, environmental health, and livestock and food.

The head of the GMC is an experienced, competent and sufficiently influential resource person with authority and a strong commitment to AMR. It is also recommended that the GMC have a mechanism and appropriate credentials to ensure that its members are free of conflict of interest and that its public health activity is transparent.

The GMC includes the Head of ORMICI, representatives of the National Regulatory Bodies (medical, pharmaceutical, dental, veterinary) and hospital drug committees, the Global Partnership on Antimicrobial Resistance, Ministries of Agriculture, Livestock and Fisheries and Regulatory Authorities. GMC member organizations also include development partners and international organizations, including WHO, FAO, CDC and OIE. The GMC also includes Universities training in health and agriculture.

As part of the fight against AMR, the GMC is responsible for :

- ensure responsibility for coordinating, where appropriate, the national response to the AMR threat;
- overseeing the development of the NAP-RAM and approving it ;
- Coordinate the formulation of national programs/projects on antimicrobial resistance;
- Supervise and evaluate the implementation of heritage management activities in the country;
- provide a structure for information sharing to mutually reinforce activities between the sectors;
- Facilitate and where appropriate, agree, coordinate efforts to contain and reduce the threat of AMR at national, regional, district and community levels;
- Establish linkages and collaboration with internal and external agencies and organizations to enhance the national AMR program;
- Strengthen and promote national networking of disease-specific information systems and programmes to ensure effective and efficient use of resources and sharing of information;
- promote the sharing of expertise and resources for intersectoral surveillance and research on antimicrobial resistance;
- convene or organize scientific seminars and serve as a national scientific platform for antimicrobial resistance;
- build sustainable partnerships and work at the national and international levels to contain antimicrobial resistance;
- Identify stakeholders and facilitate the formation of a multi-disciplinary AMR GMC.

2.4.2.3. National Secretariat of the ram

The Observatory of Resistance of Microorganisms to Anti-infectives in Côte d'Ivoire (ORMICI) acts as the secretariat of the GMC.

As such, this national secretariat of the RAM is responsible for :

- prepare and disseminate the GMC's information documents, reports and advisory notes;
- to archive the proceedings and work of the GMC meetings;
- Standardize AMR detection techniques (Standard Operating Procedures);
- Coordinate the development, review, and update of a national action plan to address antimicrobial resistance;

- facilitate and oversee the implementation, monitoring and evaluation of the plan under the supervision of the GMC;
- ensure regular data collection and information sharing among all stakeholders, GMC members and their constituencies, sectors and disciplines;
- coordinate national activities for the implementation of the antimicrobial resistance surveillance system, infection control, antimicrobial stewardship
- Develop a report on AMR prevalence and trends in the Global AMR Surveillance System (GLASS) and at the national level.

2.4.2.4. Multisectoral Technical Committees (MTC)

The Multisectoral Technical Committees (MTCs) include relevant technical specialties depending on the area under consideration including experts in infectious diseases, microbiology, infection prevention and control, social health, animal health, rational use of medicines, illicit drug control, food and drug regulation, surveillance system, environmental health and health promotion.

Six (6) MTAs were validated according to the objectives:

- MTA on the Regulatory Framework ;
- MTA for awareness, communication, education ;
- MTC on training, detection, monitoring and research ;
- MTC on Sanitation, Infection Prevention and Control ;
- MTC on Antimicrobial Stewardship and Use ;
- MTA on sustainable investment in AMR.

Each MTC includes a leader designated by the GMC and at least ten (10) other members; recourse to other experts will be considered depending on the subject matter. The CTM leader is an expert in the CTM theme and has the same mandate as the GMC.

The GMC establishes Terms of Reference (ToR) for each MTA, specifying the specific scope, roles and responsibilities

In the course of their activities, the MTAs are responsible for

- collaborate with the GMC in the formulation of the NAP-RAM or other plans;
- implement the NAP-RAM or otherwise as part of their strategic objectives;
- develop the annual work plan in accordance with the NAP-RAM ;
- to inform the GMC of the progress made in the implementation of the corresponding strategic objective;
- develop strategic documents (POS, projects, ...) ;
- to transmit the bi-monthly activity reports to ORMICI;
- draw up an annual programme of MTA meetings to be submitted to ORMICI (at the latest 2 weeks before the GMC meeting);
- orient the GMC or the national AMR focal point on its theme;
- advising the GMC on medicines and treatments.

2.4.2.5. Hospital Drugs Committee (HMC)

The mandate of the MHC is to ensure the rational use of medicines, including antimicrobial stewardship. The functions of the MHC are as stipulated in the Drug and Treatment Guidelines, including:

- monitor practices to comply with standard treatment guidelines ;
- monitor the prescribing, dispensing, and use of antimicrobials against national treatment guidelines;
- facilitate operational research for rational use of antimicrobials ;
- monitor the ethical promotion of drugs and medical supplies by drug sponsors;
- provide ongoing staff training and information on antimicrobial use and therapy;
- ensure the availability of up-to-date reference material for drug information;
- advise management teams and all staff on antimicrobial issues.

The Quality Improvement and Patient Safety Teams and the CMH lead infection prevention and control activities to reduce the spread of infection in the hospital.

2.4.2.6. Infection Prevention and Control Committee (IPC)

The Infection Prevention and Control Committee (IPC) is a body for guidance, coordination, exchange and decision-making at the peripheral level, which ensures the implementation of the national policy on the Prevention and Control of Healthcare Associated Infections.

In this capacity, it is responsible for :

- To accompany the district, the EPH and the other health establishments in the implementation of the activities of Hospital Hygiene and the Fight against Nosocomial Infections;
- Coordinate the dissemination of tools (BCC materials, reference documents, data collection materials, etc.) and the mobilization of all stakeholders) and the mobilization of all actors;
- Organize capacity building for health personnel, local government agents and the community in terms of hospital hygiene and the fight against nosocomial infections;
- Work towards a sustainable sanitary waste management system;
- ensure the availability and correct use of injection safety materials (safety syringes and safety boxes), cleaning and hygiene products, personal protective equipment and separate waste collection equipment (waste bins and bags) in the care units;
- promote methods for the effective management of biological fluid exposure accidents ;
- Participate in the development or updating of the micro-plan of the health district or the EPH or the private health facility in terms of hospital hygiene and the fight against nosocomial infections or any other orientation document intended for the use of all the actors of the district or the EPH or the private health facility;
- to enforce standards and guidelines for the organization of hospital care ;
- use the tools, standards and guidelines for the development of hospital projects;
- to support public and private health establishments in the process of drawing up hospital establishment projects;
- Ensure the monitoring and evaluation of the implementation of hygiene activities of the health district or the EPH or the private health establishment.

2.3.3. Monitoring of RAM

In Côte d'Ivoire, AMR surveillance is currently based on pathogen surveillance systems through the National Reference Centres, as well as an Observatory of Micro-organism Resistance to Anti-Infectives in Côte d'Ivoire.

There is a functioning network of public health laboratories from selected first contact facilities to the National High Quality Health Laboratory and National Reference Laboratories, capable of performing priority analyses: bacteriology, virology, serology, parasitology, biochemistry, haematology and molecular biology, including RT-PCR isolation and sequencing. All these national laboratories are committed to a quality management system.

There is also an External Quality Assessment (EQA) system coordinated by IPCI for priority diseases. Despite these successes, there is a lack of a national surveillance system for antimicrobial resistance in humans, animals, agriculture and the environment. Available data on morbidity, mortality and health related to antimicrobial resistance are only partial and unreliable. Similarly, there is no consistent and sustainable investment plan for the production of new drugs, diagnostics, vaccines and other interventions and there is no national research agenda on antimicrobial resistance.

2.3.3.1. Human health

The Observatoire de la Résistance des Microorganismes aux Anti-Infectieux en Côte d'Ivoire conducted a situational analysis of antimicrobial resistance since 2011, which covered the status and drivers of resistance in animal and human diseases as well as resistance of microorganisms in the environmental setting. The report initially indicates that the group of enterobacteria including *Enterobacter spp, Serratia spp, Hafnia spp, Citrobacter spp* (except *koseri)*, *Proteus pp* (except *P. mirabilis*) showed a high level of amoxicillin resistance of 73.7%, 85.7%, 89.1%, 80.2%, 87.8% and 87.3 from 2012 to 2017 which shows a consistency in recent years. For extended-spectrum beta-lactamase enterobacteriaceae (EBLSE), the most isolated multidrug-resistant bacteria (MDR), the rates which were at 18.5% in 2012 have increased to nearly 38.4% in 2013, 38.5% in 2014, 40.1% in 2015 and 37.4% in 2016. It is therefore becoming urgent to develop surveys in community settings in order to have the rates of bacterial resistance in city medicine.

The Global Health Security Agenda (GHSA) assessment report using the Joint External Assessment (JEA) RSI tool conducted in 2016 shows that antimicrobial resistance is a major problem in Côte d'Ivoire. The report states that, antimicrobial resistance (AMR) is growing at an alarming rate to the point of outpacing the development of new control methods capable of countering infections in humans. This situation threatens patient care, economic growth, public health, agriculture, economic security and national security.

Antibiotic use in the community is indiscriminate and the recommendation to restrict it is hardly enforceable. In addition to selling antibiotics without prescription at the request of a customer, drug sellers also promote inappropriate regimens. Thus availability and rational use of medicines is a major challenge at the national level.

2.3.3.2. Animal health

Antibiotics are commonly used to treat animals. In Côte d'Ivoire, the animal diseases that frequently use antibiotics include colibacillosis in piglets, pasteurellosis, pig rouget in pig farming (KILI, 2016) and colibacillosis, avian cholera, salmonellosis in poultry farming (BITTY, 2013).

In 2017, ZOBO worked on the determination of the prevalence of antibiotic resistance and MIC of *Escherichia coli* strains isolated from droppings collected from poultry farms in Abidjan and Agnibilékro areas. This study consisted, on the one hand, in determining the prevalence of resistance of these bacterial strains to 14 antibiotics and, on the other hand, in determining the minimum inhibitory concentrations (MIC) of four antibiotics (streptomycin, tetracycline, amoxicillin-clavulanic acid and trimethoprim/sulfamethoxazole) against multidrug-resistant *E.coli* strains.

Antibiotic susceptibility testing of 43 isolates showed that *E. coli* were resistant to all 14 antibiotics. Among these antibiotics, tetracyclines (97%), sulfonamides (85%), trimethoprim-sulfamethoxazole (80%) and streptomycin (72%) had the highest resistance levels. The antibiogram also revealed a high prevalence of multidrug resistance. Thus, 96% of isolates were resistant to at least 4 antibiotics.

At the level of the animal health system, care at the community level is provided by the public sector, while animal health centres and clinics are provided by the private sector. The lack of medical personnel at the village level has led livestock keepers to visit informal sales outlets and traditional medicine practitioners to obtain medicines. In addition, farmers may administer antibiotics to animals to compensate for their poor management due to the lack of official veterinary services and the prevalence of animal diseases.

In the animal health system, private veterinary clinics and practices, veterinary anti-rabies centres and public veterinary clinics have a Veterinary Drug Management Unit (VDMC) with the acronym "VMD". The activities of the VMCs include:

- to plan the orders, the distribution of vaccines, medicines, veterinary materials to the customers;
- to manage the cold chain for the conservation of vaccines;
- report adverse events reported by clients
- check the labelling and packaging of veterinary medicines
- record withdrawal periods, antimicrobial susceptibility data, and remarks regarding the susceptibility of animals to treatment.

To date, the CGMVs are not yet formally installed or operational in private veterinary clinics and practices, veterinary anti-rabies centres and public veterinary clinics.

2.4.3.3. PREVENTION

The Directorate General of Health and Public Hygiene, in conjunction with the Directorate of Public Hygiene of Health and Environment (DHPSE) and the Directorate of Hospital Medicine and Proximity (DMHP), established in July 2017, a pilot coordination committee bringing together technicians in hygiene, sanitation and prevention of infections associated with care at the level of health region Abidjan 2. This committee through the DHPSE conducted in 2018, an analysis of the situation of infection prevention and control in 30 health facilities in the Abidjan 2 health region with financial support from UNICEF.

Another assessment conducted by DMHP covered 9 public and private tertiary facilities in August 2018 with financial support from WHO. The data analysis noted that all first contact health facilities have inadequate infection prevention and control except FSUCOM BANCO.

Nevertheless, there is a clear plan and many guidelines for infection prevention and control. At the same time, infection prevention and control committees exist in health facilities across the country, although they are not always adequately resourced, active or effective. Waste management and hygiene in community facilities need to be strengthened. In 2019 Hygiene, Infection Prevention and Control (HPCI) committees in veterinary care settings were installed in the National Anti-Rabies Veterinary Centre in Cocody and the veterinary clinic of the Gbêkè -Hambol regional directorate of animal and fishery resources. Training curricula, a learner's manual and a trainer's guide have been developed in the animal sector.

2.4.3.4. AWARENESS

In Côte d'Ivoire, public awareness of antimicrobial resistance remains a challenge, as no such program currently exists. Patients' knowledge of prescribed antimicrobials, including routes of administration, dosage, frequency of administration, duration of use, and the importance of adherence to treatment, even if they feel better, is limited. Patients generally request antibiotics even for non-infectious diseases and self-medicate with antimicrobials.

However, there are institutions such as the Community Health Directorate in the human health sector and the Livestock Education Unit within MIRAH, which have a mandate to inform the community on various topics related to human and animal health.

2.5. THE NATIONAL PLAN FOR RATIONAL ANTIMICROBIAL STEWARDSHIP

2.5.1. REMINDER OF THE GOALS AND OBJECTIVES OF THE NATIONAL MULTISECTORAL PLAN TO COMBAT ANTIMICROBIAL RESISTANCE (PAN-RAM)

II.5.1.1. Purpose of PAN-RAM

In the absence of a specific goal stated by the Côte d'Ivoire's PAN-RAM, it must be deduced that this PAN-RAM pursues the same goal as that of the Global Action Plan to combat AMR, namely "to have, for as long as possible, effective means of treatment and prevention of infectious diseases in the form of safe and effective medicines, of guaranteed quality, used responsibly and accessible to all those who need them".

2.5.1.2. Objectives of PAN-RAM

The six (06) objectives of PAN-RAM are :

1. Strengthening the organizational framework for combating antimicrobial resistance ;
2. Improve awareness and understanding of AMR through communication, education and training;
3. Strengthen the knowledge and evidence base on AMR through surveillance and research ;
4. Reduce the incidence of infections through effective sanitation, hygiene and prevention measures;
5. Optimizing the rational use of antimicrobial drugs in human, animal and environmental health ;
6. Promote sustainable investments according to the country's needs as well as research and development.

2.5.2. OBJECTIVES AND ACTIONS OF THE NATIONAL PLAN FOR RATIONAL ANTIMICROBIAL STEWARDSHIP

The objective of the PN-GAM is the strategic objective 5 of the PAN-RAM, namely to **optimize the rational use of antimicrobial drugs in human, animal, environmental and agricultural health**.

The establishment of sound antimicrobial stewardship will prevent the development of resistance associated with irrational use of antimicrobials in both humans and animals. Priority areas for addressing this issue include improving the regulatory framework for the preservation of antimicrobial agents and revising policies and guidelines for antimicrobial use in the country.

Interventions will include strengthening the regulatory authority for quality control, distribution and use of antimicrobial agents.

Other strategies include (i) increasing awareness of patients and health care providers, (ii) reducing the prevalence of substandard medicines for human and veterinary use, (iii) reducing and outlawing inappropriate and unregulated use of antimicrobial agents in agriculture and animal husbandry, (iv) strengthening the management of antimicrobial use in human and animal health care facilities by providing an evidence base for standard prescribing and delivery of care. This management will also include monitoring and evaluation of antimicrobial use and consumption at all levels.

The specific objectives and strategic actions of this plan and the activities planned to achieve them are organized as follows

- **Specific objective 5.1:** *Ensure uninterrupted access to good quality antimicrobial medicines*

Strategic action	Planned activities
5.1.1 Ensure the quality of antimicrobials marketed in the country	5.1.1.1 Strengthen pre- and post-marketing authorization (PMA) control of antimicrobials
	5.1.1.2 Supporting laboratories for the control of antimicrobial agents pre- and post-MRA

- **Specific objective 5.2:** *Improve the use of antimicrobial agents in healthcare settings.*

Strategic action	Planned activities
5.2.1 Strengthen training for prescribers of antimicrobials in human, animal, and environmental health	5.2.1.1 Strengthen training of prescribers, dispensers and other health professionals on the rational use of antimicrobial agents in human, animal and environmental health
5.2.2 Establish a national antimicrobial surveillance system	5.2.2.1 Assess antimicrobial use in the human, animal, and environmental populations (supply, distribution, prescribing, dispensing, consumption)

2.5.3. STRENGTHS, WEAKNESSES, OPPORTUNITIES, THREATS (SWOT) ANALYSIS OF THE NATIONAL PLAN FOR RATIONAL MANAGEMENT OF ANTIMICROBIALS

Analysis of the status of antimicrobial stewardship reveals strengths that can be capitalized on, opportunities that can be seized, but also weaknesses that need to be addressed and threats whose impact should be minimized.

2.5.3.1. STRENGTHS, WEAKNESSES, OPPORTUNITIES AND THREATS

2.5.3.1.1. Institutional, legal and regulatory framework for antimicrobial stewardship

FORCES

- Côte d'Ivoire has a National Multisectoral Plan to fight against antimicrobial resistance;
- The official and functional "One Health" national platform;
- The Ivory Coast has a National Pharmaceutical Policy document for human health;
- There is a political will to fight AMR according to the "One Health" approach;
- There is a policy document on the upgrading of veterinary services that takes into account the pharmacy and veterinary drugs sector;
- Legislation and regulations are available on the registration, quality control, supply and pharmacovigilance (especially in human medicine) of medicines;
- There is a legal and institutional framework for the organization of the Ivorian authority for the regulation of antimicrobial agents used in animal health (animal sector issue);
- There is governance and institutional structures dedicated to the fight against AMR;
- Existence of a laboratory network.

WEAKNESSES

- Insufficient regulation of the prescription and dispensing of drugs in general and antimicrobials in particular;
- Insufficient regulations specific to veterinary antimicrobial stewardship for pharmacovigilance, AMR surveillance, and laboratory analysis;
- Insufficient attribution of the decree instituting the Hospital Drug Committee in relation to the fight against AMR;
- Low financial accessibility of the population to medical biology laboratory services;
- Insufficient regulation of the promotional activities of medical sales representatives.

OPPORTUNITIES

- There is a political will to fight AMR according to the "One Health" approach;
- There is the IHR and Global Health Security Agenda (GHSA).

THREATS

- Political instability.

2.5.3.1.2. Marketing authorisation, licensing and inspections

FORCES

- There are regulations for obtaining marketing authorisations for human and veterinary medicines;
- There is a Laboratory (LNSP) for the quality control of drugs for which a licence is requested
- In Animal Health, there is a laboratory (LCVB) for the quality control of biological products for veterinary use.
- The technical evaluation of the registration application files is carried out by experts both for medicinal products intended for human medicine (national experts) and for those intended for animal medicine (WAEMU experts).

- Licences to practice are issued to qualified persons who are registered with mandatory professional organisations such as professional bodies, where applicable
- inspections of establishments and practices in human and veterinary medicine are provided for in the texts
- Strengthening of the institutional framework for the regulation of the pharmaceutical sector with the establishment of the Ivorian Pharmaceutical Regulatory Authority.
- There is a legal and institutional framework for the organization of the Ivorian veterinary drug regulatory authority.

WEAKNESSES

- Insufficient inspection capacity (inspection kit, rolling stock, etc.) for the sanitation of the veterinary drug market.
- Insufficient implementation of inspection activities in both human and veterinary medicine
- Insufficient technical capacity of LNSP for optimal quality control of drugs
- Insufficient quality control capacity (infrastructure, equipment, inputs and training).
- Illegal practice of pharmacy, human and veterinary medicine (not registered with professional orders, unqualified and unauthorized persons, etc.)

OPPORTUNITIES

- Strengthening of the institutional framework for the regulation of the pharmaceutical sector with the establishment of the Ivorian Pharmaceutical Regulatory Authority
- There is a community text that institutes the pharmaceutical regulatory authority in the WAEMU member states
- There is a legal, institutional and community framework for the organisation of the Ivorian veterinary medicines regulatory authority

THREATS

- Unregulated proliferation of illegal medical practices
- Difficulties in cleaning up the veterinary drug market

2.5.3.1.3. Post-marketing surveillance and pharmacovigilance

FORCES

- There are texts that organize the post-marketing control of medicines
- In human and animal health, there are regulatory and community texts that organise the pharmacovigilance of medicines.
- Existence of a national mechanism for cleaning up the veterinary drug market
- In the field of animal health, there are Community texts relating to the implementation of the control, inspection and monitoring of veterinary medicinal products.

WEAKNESSES

- Pharmacovigilance is insufficiently implemented in human medicine and almost non-existent in veterinary medicine
- Post-marketing controls are insufficiently implemented.
- Insufficient implementation of national campaigns to clean up the veterinary drug market.

OPPORTUNITIES

- Actions undertaken by the laboratory network to standardize AMR detection methods
- Implementation of the Union's Agricultural Policy (UAP) which led to national campaigns to clean up the veterinary medicine market.

THREATS

- Existence of a significant regional illicit and illegal market resulting in AMR and the persistence of adverse effects.

2.5.3.1.4. Good supply management policies and guidelines for antimicrobials

FORCES

- There is regulation of drug supplies in general and antimicrobials in particular in both the human and veterinary medicine sectors.
- There is a national committee for the quantification of drug requirements for human medicine that is essential for the public sector.
- The actors of the official circuit are known and previously authorized
- There is a network of laboratories

WEAKNESSES

- There is no national policy document for quantifying the needs and supply of veterinary drugs.
- Human and animal health professionals have limited core competencies in the "one health" approach
- The number of trained veterinarians (for the public and private sector) is insufficient in relation to current needs and planned objectives
- Low financial accessibility of populations to medical biology laboratory services
- Stockouts
- Human and animal health professionals have limited core competencies in the "one health" approach.
- Lack of national consensus protocols for the management of infectious diseases ;

OPPORTUNITIES

- Contributions of international organizations and institutions to AMR control policies (WHO, OIE, FAO, AU, etc.)
- Improvement of the legal and institutional framework for combating illicit trafficking in medicines and the proliferation of substandard and falsified medicines (signing of the MEDICRIME convention)
- Strengthening of the national system for the quantification and supply of veterinary medicines.
- Existence of some teaching on AMR in the training courses of health professionals

THREATS

- There is a significant regional illicit market for medicines in the human and animal sectors fuelled by substandard and falsified medicines
- Limited capacity/resources (financial, structural) of ministries to engage in multi-sectoral collaborative activities

2.5.4. PRIORITY ISSUES

The situational analysis and the analysis of strengths, weaknesses, opportunities, and threats identified the following priority issues for sound antimicrobial stewardship policy:

- Inadequate legislative and regulatory framework for certain aspects (prescribing, dispensing, monitoring, inspection, surveillance and pharmacovigilance) of antimicrobial stewardship;
- Insufficient human resources, especially in the animal sector, in relation to national antimicrobial stewardship objectives;
- Insufficient post-marketing surveillance and pharmacovigilance policy for antimicrobials ;
- Lack of a system for the selection and quantification of veterinary drugs ;
- Inadequate inspection, monitoring and surveillance of antimicrobial use ;
- Existence of an important regional and national illegal market of medicines in both the animal and human sectors.
- Insufficient awareness among stakeholders and the population in particular on the importance of sound antimicrobial stewardship
- Existence of stock-outs
- Lack of national consensus protocols for the management of infectious diseases

2.5.5. FACILITATION OF THE REVISION OF THE NATIONAL PLAN FOR RATIONAL MANAGEMENT OF ANTIMICROBIALS

The review of the National Antimicrobial Management Plan (PN-GAM), Strategic Objective N°5 of the National Multisectoral Plan for the fight against AMR allows us to note that certain problems identified by this situational analysis had not been taken into account in the NAP-RAM 2019-2020.

To this end, certain strategic actions can be proposed, taking into account the shortcomings identified for a greater effectiveness of the NIP-MAP. Thus, at the level of specific objective 5.2. to improve the use of antimicrobial agents in health care settings, one could envisage a strategic action concerning the optimization of the regulation of antimicrobial use. Admittedly, such a strategic action is close to strategic objective 1 of the PAN-RAM relating to the implementation of the regulatory framework for the fight against AMR, but it has the advantage of dealing specifically with the problem of the specific texts related to the prescription and dispensing of antimicrobials. The activities planned within the framework of this strategic action would be related to the development of directives and regulatory texts for the prescription and dispensing of antimicrobials including the revision of the decree instituting the Hospital Drug Committees to take into account the aspects related to the fight against AMR.

Thus, the logical framework of the National Antimicrobial Management Plan could be presented as follows

**Strategic Goal 5: Optimize the use of antimicrobial drugs in human, animal and environmental health**	
Specific objective 5.1: Ensure uninterrupted access to good quality antimicrobial medicines	
Specific objective 5.2: Improve the use of antimicrobial agents.	
	5.2.1. Strengthen the training of prescribers and dispensers of antimicrobials in human and animal health (identify training needs, develop training materials, implement training and monitoring-evaluation of training).
	**5.2.2. popularize the use of antibiograms**
	5.2.3. Establish an antimicrobial control and surveillance system.

	5.2.3.1. Assess antimicrobial use in the human, animal and environmental populations (supply, distribution, prescribing, dispensing, consumption).
	5.2.3.2. Establish the system for antimicrobial selection and quantification in veterinary medicine.
	5.2.3.3. Strengthen antimicrobial control, inspection, surveillance.
	5.2.3.4 Strengthen **controls** and **inspections** of antimicrobial drugs
	5.2.4. Strengthening specific regulations on the management of medicines, surveillance, pharmacovigilance and laboratory analysis in the animal sector.
Specific objective 5.3: Contribute to the fight against substandard and falsified antimicrobial drugs (Strengthen campaigns to clean up the veterinary drug market).	
Specific objective 5. 4: Contribute to the fight against the proliferation of illegal medical facilities	
Specific objective 5.5: Improve communication and awareness of prescribers, providers and populations on rational use of antimicrobials	

RECOMMENDATIONS

The situational analysis of the national antimicrobial drug management policy calls for the following recommendations:

1. To the Multisectoral Coordination Group for the fight against AMR
   - Revise and validate the National Policy Document on Rational Antimicrobial Stewardship;
   - Disseminate and popularize the National Policy Document on Rational Antimicrobial Stewardship;
   - Develop and validate guidelines, national texts and manuals of procedures for sound antimicrobial stewardship;
   - Disseminate and popularize guidelines, national texts and manuals of procedures for rational management of antimicrobials.
   - To work on the development and validation of consensual therapeutic protocols for the following diseases
   - Effective implementation of the national rational antimicrobial stewardship plan
   - Monitor and evaluate the national antimicrobial stewardship plan.

2. To the government
   - Provide funding for the National Antimicrobial Stewardship Plan.
   - Ensure the implementation and monitoring and evaluation of the National Antimicrobial Management Plan.

3. To the Technical and Financial Partners

Support Côte d'Ivoire in the implementation of the National Plan for Rational Antimicrobial Management.

CONCLUSION

The National Antimicrobial Management Plan (PN-GAM) is an ordered series of strategic actions and planned activities related to strategic and specific objectives for a rational management of antimicrobials

in Côte d'Ivoire. It intends to achieve the objectives of the National Multisectoral Plan for the fight against Antimicrobial Resistance (PAN-RAM) with which it is perfectly consistent. The situational analysis that founded the PN-GAM and its update highlights a detailed description of the main actors involved in the licensing, registration, supply, post-marketing control, pharmacovigilance, surveillance, control and inspection of services and modalities of use of antimicrobials in both animal and human medicine. While the situation of antimicrobial stewardship presents strengths to be capitalized on and opportunities to be seized, weaknesses and threats that could hinder the achievement of the objectives should be taken into account. Among the threats, we can mention the worrying development of illicit drug trafficking. The PN-GAM is accompanied by a monitoring and evaluation plan that will have to be budgeted for, and remains dependent on the effective involvement of all the local actors concerned and the constant support of development partners.

REFERENCES

ASSOUMY A. M., 2009. Contribution to the codification, analysis of import statistics and quality study of veterinary drugs in Côte d'Ivoire: case of Abidjan, Anyama and Bingerville. Thesis Méd. Vét : Dakar; 17

BITTY Z. B. A., 2013. Contribution to the improvement of health management and medical practices in modern poultry farming in the peri-urban area of Abidjan (Ivory Coast). Thesis Méd. Vét : Dakar; 13

GOUEPO T.E. 2019. Evaluation of the main characteristics of pharmacy dispensaries in Côte d'Ivoire from 2009 to 2018. Dissertation Master Réglémentation et Inspection pharmaceutique, UFRSP Université Félix-Houphouet Boigny, Abidjan.

KACOU A., YAPI A.D., KOUAKOU S.G., KOUAKOU L., OUATTARA L., 2009. exposure profile and practical modalities of antibiotic use in patients hospitalized in the resuscitation department of the University Hospital of Yopougon, *Rev.Int ;Sc. Med, Vol 11, n°2.*

NGBOCHO B. N., 2018. Rights and obligations of the beneficiaries of the veterinary drug in Ivory Coast Dissertation. Méd.Vét : Dakar; 22

OULAI J., 2004**.** Distribution and use of veterinary drugs in Côte d'Ivoire: case of the lagoon region. Thesis: Méd. Vét: Dakar, 12.

ZOBO A. A., 2017. Determination of the prevalence of antibiotic resistance and cmi of *Escherichia coli* strains isolated from droppings collected from poultry farms in Abidjan and Agnibilekrou areas (Ivory Coast) Thesis. Méd. Vet: Dakar; 12

OIE, 2011. Evaluation report oie pvs (performance of veterinary services) of the veterinary services of the republic of Côte d'Ivoire 14 to 24 November 2011

WEBOGRAPHY

ABIOLA et al. 2014; Antibiotic residues and food of animal origin in Africa: public health risk. Rev. Sci Tech. Off. Int. Epiz, 2014, **33** (3)

https://www.oie.int/fileadmin/home/eng/publications_&_documentation/docs/pdf/revue_plurithematique/2014/10062014-00034-fr-mensah.pdf accessed on 26/05/2020

JIM O'NEILL, 2014. Antimicrobial Resistance: Tackling a crisis for the health and wealth of nations

https://amr-review.org/sites/default/files/AMR%20Review%20Paper%20-%20Tackling%20a%20crisis%20for%20the%20health%20and%20wealth%20of%20nations_1.pdf Consulté le 26/05/2020

LISTS OF TEXTS CONSULTED

HUMAN HEALTH SECTOR

Regulation n°06/2010/CM/UEMOA of 1er October 2010 on the registration procedures for pharmaceutical products for human use in WAEMU Member States.

Decision n°009/2009/COM/WAEMU of 10 September 2009 establishing the modalities for the submission of an application for a community marketing authorization.

Decision n°011/2009/COM/UEMOA of 10 September 2009 establishing the list of minor and major changes to marketing authorisation dossiers.

Decision n°10/2010/CM/UEMOA of 1er October 2010 on the adoption of guidelines for the control of information and advertising on medicines to health professionals in WAEMU Member States.

Law n°65-250 of 4 August 1965 modifying articles of the public health code relating to the regime of medicines.

Law n°2015-533 of 20 July 2015 on the practice of pharmacy

Law n°2017-541 of August 03, 2017 on the regulation of the pharmaceutical sector

Decree No. 94-456 of 25 August 1994 establishing the derogatory conditions relating to the rules for the dispensing of prescriptions for pharmaceutical products by pharmacists.

Decree n°94-669 of December 21, 1994 on the conditions of registration and dispensing of medicines in Côte d'Ivoire.

Decree No. 96-876 of 25 October 1996 on the classification of public health establishments

Decree No. 98-473 of 13 August 1998, regulating the advertising of medicines and pharmaceutical establishments in Côte d'Ivoire.

Decree n°2015-568 of 29 July 2015 on the creation, organisation and functioning of the national drug commission.

Decree n°2015-569 of 29 July2015 creating the committee of experts for the technical evaluation of applications for drug approval.

Decree n°2015-602 of September 02, 2015 establishing fees for the Marketing Authorization of medicines.

Decree No. 2016-24 of 27 January 2016 on the Code of Ethics and Professional Conduct for practitioners of traditional medicine and pharmacy.

Decree No. 2016-717 of 14 September 2016 regulating the advertising of medicines, other health products and pharmaceutical establishments

Decree No. 2019-292 of 03 April 2019 on the creation, attribution, organization and functioning of the public health emergency operations centre.

Decree No. 2019-293 of 03 April 2019 on the creation, attribution, organization and functioning of the national platform "one health.

Order MSHP/CAB/PSPCI of 5 March 2007 on the creation, organisation and functioning of medicines committees within health structures that are not National Public Establishments (EPN) of Côte d'Ivoire

Order n°210 of 24 November 2010 modifying order n°16 of 10 February 2009 on the creation, organisation and functioning of the national pharmacovigilance system.

Order n°211 of 24 November 2010 modifying order n°249 of 18 November 1988 creating a National Pharmacovigilance Commission.

ANIMAL HEALTH SECTOR

Regulation n°02/2005/CM/UEMOA relating to the harmonization of pharmaceutical regulations in the UEMOA member states

Regulation n°02/2006/CM/WAEMU laying down community procedures for the marketing authorization and supervision of veterinary medicinal products and establishing a regional committee for veterinary medicinal products

Regulation n°02/2006/CM/WAEMU laying down community procedures for the marketing authorization and supervision of veterinary medicinal products and establishing a regional committee for veterinary medicinal products

Regulation n°03/2006/CM/UEMOA instituting fees in the field of veterinary drugs within the UEMOA

Regulation n°04/2006/CM/UEMOA instituting a network of laboratories responsible for quality control of veterinary medicines within the UEMOA zone

Regulation n°08/2010/CM/UEMOA amending Regulation n°02/2006/CM/UEMOA laying down Community procedures for the marketing authorization and supervision of veterinary medicinal products and establishing a regional committee for veterinary medicinal products

Regulation n°03/2006/CM/UEMOA instituting fees in the field of veterinary drugs within the UEMOA

Decision n°009/2009/COM/UEMOA establishing the modalities for the submission of an application for a community marketing authorization

Decision n°010/2009/COM/WAEMU on the designation of laboratories of the network in charge of quality control of veterinary drugs within WAEMU

Decision n°011/2009/COM/UEMOA establishing the list of minor and major changes to marketing authorisation dossiers.

Law n°63-323 of 25 July 1963 on animal health in the Republic of Côte d'Ivoire

Law n°88-683 of 22 July 1988 instituting a Code of Ethics for Veterinarians

Law 88-684 of 22 July 1988 creating the National Order of Veterinarians

Law n°96-561 of 25 July 1996 on veterinary pharmacy

Decree n°93-777 of September 29, 1993 creating ANADER

Decree n°95-536 of 14 July 1995 relating to the veterinary health mandate

Decree No 98-70 of 13 February 1998 laying down general rules for the installation of livestock farms

Decree n°2001-487 of August 9, 2001 on the application of law n°96-561 of July 25, 1996

Decree n°2005-250 of July 7, 2005 fixing in the field of animal production and halieutic resources, the modalities of application of the law n°200-208 of July 7, 2003 transferring and distributing competences of the State to the territorial communities.

Decree n°2013-329 of 22 May 2013 on the powers, organisation and functioning of the National Agricultural Development Support Laboratory called LANADA

Decree No. 2014-552 of 1er October 2014 on the organization of the Ministry of Animal Resources and Fisheries

Order n°35/MIPARH of 9 August 2007 establishing the rules of good practice for the manufacture, import and distribution of veterinary medicines.

Order n°035/MIPARH/CAB of 01 April 2008 to fix the attributions and organization of regional directorates, departmental directorates, livestock and fishery resources stations and entry stations of the Ministry of Animal Production and Fisheries Resources

Order 069 MIPARH of July 01, 2010 establishing practical measures for border health police exercises

ENVIRONMENTAL INDUSTRY

Law n°96-766 of October 3, 1996 on the environment code

Decree n°97-678 of 3 December 1997 on the protection of the marine and lagoon environment against pollution

Decree n°98-42 of 28 January 1998 concerning the organisation of the emergency plan to combat accidental pollution at sea, in lagoons and in coastal areas (POLLUMAR)

AGRO-FOOD INDUSTRY

Law n° 2015-237 of July 2015 on agricultural orientation

Law No. 63-301 of 26 June 1963 on the repression of fraud in the sale of goods and falsification of foodstuffs and agricultural products

Decree No. 79-573 of 4 July 1979 regulating restaurants

Professor Antoine Serge, AMARI **Doctor Zahoui Boris Arnaud, BITTY**

Pharmacist, consultant sector Veterinarian, consultant sector human medicine veterinary medicine

TECHNICAL AND ADMINISTRATIVE MANAGEMENT

Dr AHIMON DJEDJI Herodias
Senior Technical Advisor
USAID - MTaPS Program
