# Supplementary material for: Landscape Analysis of Existing Policies, Laws and Regulations on Antimicrobial Stewardship in 10 Low- and Middle-Income Countries
Source: Antibiotics (Basel). 2026 Aug 10;15(8):770. doi: 10.3390/antibiotics15080770 (PMC13510074; doi:10.3390/antibiotics15080770)
Supplement: Supplementary file 1 [file antibiotics-15-00770-s001.zip › S3 Country Reports/S3E_Mali (Human & Animal Health).pdf]

PROGRAMME DES MÉDICAMENTS, TECHNOLOGIES ET SERVICES PHARMACEUTIQUES (MTaPS) DE L'USAID

Accès amélioré. Services améliorés. Meilleurs résultats de santé.

Rapid assessment of antimicrobial stewardship and supply chain management policies and regulations in the human health and animal health sectors.

Mali
October 23, 2020

USAID
DU PEUPLE AMERICAIN

This document was made possible by the generous support of the American people through the United States Agency for International Development (USAID) under Contract No.º 7200AA18C00074. Its contents are the responsibility of

About USAID's MTaPS Program

USAID's Medicines, Technology, and Pharmaceutical Services (MTaPS) program helps low- and middle-income countries strengthen their pharmaceutical systems, as strengthening these systems is critical to ensuring an effective health system. The MTaPS program works to increase access to essential health products and services and to promote the correct use of medicines so that people can enjoy better health. It builds on decades of expertise in developing state-of-the-art pharmaceutical systems in more than 40 countries. The MTaPS approach achieves sustainable results by including all stakeholders in the health sector: government, academia, civil society and the private sector. Led by Management Sciences for Health (MSH), a global health non-profit, the program is implemented by a consortium of global and local partners.

The MTaPS Consortium

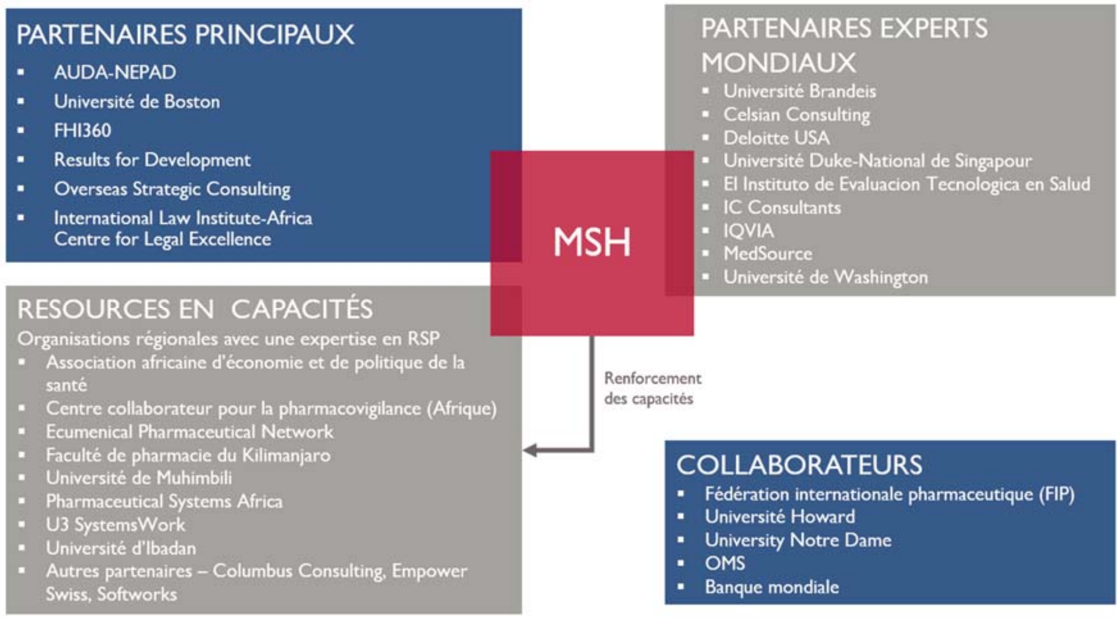

This report is presented by :
Dr. Drissa COULIBALY, National Director

National Directorate of Veterinary Services

Bamako, Hamdallaye ACI 2000 behind Immeuble ABK1, rue 209

dcoulibaly9@yahoo.fr

Telephone: Fixed line (00223) 20290777
Mobile (00223) 76023476

LIST OF ACRONYMS, ABBREVIATIONS AND/OR SPECIFIC TERMS

AMM	Marketing Authorization
AMS	Antimicrobial Stewardship
ANEH	National Hospital Evaluation Agency
AWaRe	Access Watch and Reserve
ANDVM	National Association of Veterinary Doctors of Mali
ANSSA	National Food Safety Agency
AOF	French West Africa
ATE	Technical Livestock Agent
BPC	Good Clinical Practice
GMP	Good Manufacturing Practices
CAC	Codex Alimentarius Commission
CADD	Support Unit for Decentralization and Deconcentration of the Ministry of Health
CCRVDF	Codex Committee on Residues of Veterinary Drugs in Foods
CNAM	National Centre for Disease Control Support
CNIECS	National Centre for Health Information and Education
CNOP	National Council of the Order of Pharmacists
CNPV	National Pharmacovigilance Commission
CSCom	Community Health Centre
CSRéf	Reference Health Centre
CRMV	Regional Veterinary Medicines Committee
CV	Veterinary Committee
DAOA	Food of Animal Origin
DGHSP	Directorate General of Health and Public Hygiene
DLVN	Veterinary Legislation and Standards Division
DNCC	National Directorate of Trade and Competition
DNPIA	National Directorate of Animal Production and Industry
DNSV	National Directorate of Veterinary Services
DPM	Directorate of Pharmacy and Medicines
DRSV	Direction Régionale des Services Vétérinaires
EC	Clinical Trial
EEC	Joint External Evaluation (JEE) WHO-OIE
FAO	Food and Agriculture Organization of the United Nations
FAPH	Faculty of Pharmacy
FMOS	Faculty of Medicine and Odonto-Stomatology
GCMN	National Multisectoral Coordination Group
GHSA	Global Health Security Agenda
GRA	Antimicrobial Resistance Management
GVG	Groupement des Vétérinaires Grossistes
IFAH	International Federation for Animal Health
INFSS	National Institute of Training in Health Sciences

INSP	National Institute of Public Health
RPI	Rural Polytechnic Institute
IS	Health Inspection
JECFA	Joint FAO/WHO Expert Committee on Food Additives
LCV	Central Veterinary Laboratory
LEMV	List of Veterinary Medicine Experts
NMEA	National List of Essential Medicines
MRL	Maximum Residue Limits
LNS	National Health Laboratory
MEP	Ministry of Livestock and Fisheries
MLRC	Legally Deemed Contagious Disease
MSAS	Ministry of Health and Social Affairs
MSH	Management Sciences for Health
MTaPS	Medical Technologies and Pharmaceutical Services
OECD	Organisation for Economic Co-operation and Development
OIE	World Organisation for Animal Health
WTO	World Trade Organization
WHO	World Health Organization
OH	One Health
WAHO	West African Health Organization
PALV	Veterinary Legislation Support Programme
PAN-RAM	National Action Plan against Antimicrobial Resistance
PPM	Popular Pharmacy of Mali
PPN	National Pharmaceutical Policy
PV	Pharmacovigilance
PPCB	Peri-Contagious Bovine Pneumonia
PVS	Performance of the Veterinary Services
RAM	Antimicrobial Resistance
RSI	International Health Regulations
SEC	Clinical Trial Monitoring
SDADME	Master Plan for the Supply and Distribution of Essential Medicines
SIAPS	Systems for Improved Access to Pharmaceuticals and Services
SGQ	Quality Management System
SOMAPIT	Malian Society of Infectious and Tropical Pathologies
SPC	Summary of product characteristics
SPS	Sanitary and Phytosanitary Agreements
SWEDD	Sahel Women's Empowerment and Demographic Dividend Project
TLR	Legislative and Regulatory Text
UEMOA	West African Economic and Monetary Union
USAID	United States Agency for International Development
VICH	Harmonization of Technical Requirements for Registration of Veterinary Medical Products
WAHIS	Global Zoo Health Information System

Table of Contents

List of acronyms, abbreviations and/or specific terms ii

Acknowledgements 2

Human Health Area 3

- Summary 3
- Proposed actions 4
- Introduction 4
- Methodology 5
- Results : 6
  - National regulations, legislation or policies : 6
  - Regulatory Analysis : 8
- Drug Use and PLAR Policies/Regulations 12
- National List of Essential Medicines 13
- Good Procurement Policies 16
- Appropriate use by prescribers, providers and patients 18
- Summary of variances by section or area 22

Animal Health : 22

- Operational Summary 22
- Introduction 22
- Objectives, scope and context of the evaluation 22
- Summary of findings 23
  - National antimicrobial legislation, regulations or policies 23
  - The Essential Drug List (EDL) 23
  - Good Procurement Policy 23
  - Appropriate use by prescribers, distributors and breeders 23
  - Suggestions 23
  - Conclusion 25

Introduction 26

Objectives 27

General objective 27
Specific objectives 27
Methodology 28
Work plan : 28
Methods 28
Materials 29
International approach to veterinary medicines 29
THE OIE 29
The FAO 29
THE WHO 30
VICH 30
The International Federation for Animal Health (IFAH) 30
The Codex Alimentarius Commission 30
Legislation, registration and control procedures for veterinary medicinal products in the WAEMU 30
Marketing authorisation and registration of veterinary medicinal products 32
Administrative file 32
Technical file 33
Control procedures for veterinary medicinal products 33
Findings 34
National legislation, regulations or policies 34
Marketing Authorization (MA) 35
Importation 35
Licensing 36
Distribution 36
Wholesale distribution 36
Retail distribution 36
Procedures for the distribution of veterinary drugs in Mali 37
Control and Inspection 38
Advertising control 38
Pharmacovigilance 38
Standardised Therapeutic Protocols (STP) 39
Essential Drug List 39
Good Procurement Policy 40

Appropriate use by prescribers, distributors and breeders 40
Prescription 40
Usage 42
Actors and institutions involved in antimicrobial use in animal health 42
The WAEMU Commission 42
Government structures 42
The veterinary pharmaceutical industry with respect to veterinary drugs containing antimicrobial agents 43
Wholesalers and retailers 44
Veterinarians 44
Food-producing livestock farmers 45
Feed manufacturers 46
Working with Tripartite Organizations to Address Problems 46
Strategy for action in the field of veterinary medicinal products and veterinary practice 47
In the field of veterinary medicinal products : 47
Concerning the veterinary profession : 47
Proposed strategic directions and follow-up mechanisms to address the main issues identified 47
Main problems identified 47
Proposed strategic directions 48
Multi-sectoral and "One Health" collaboration/coordination 48
Effective communication and education 49
Training and professional education on the appropriate use of antimicrobials in the veterinary sector 49
Strengthening veterinary services 50
Monitoring and Research 50
10.2.6. Strengthening the regulatory framework 51
Proposed Action Plan for Antimicrobial Stewardship in Animal Health 51
Surveillance and Research (see NAP-Mali Strategic Objective 2: Strengthen the knowledge and evidence base through surveillance and research) 52
Stewardship 53
Awareness and education (see NAP-Mali Strategic Objective 1: To improve awareness and understanding of antimicrobial resistance through effective communication, education and training). 54
Regulation and surveillance (c.f. Strategic objective 4 of PAN-Mali: Optimize the use of antimicrobial agents in human, animal, environmental and plant production health) 54

Research and innovation (c.f. NAP-Mali Strategic Objective 5: To make the economic case for sustainable investments that take into account the country's needs and increase investments in the development of new drugs, diagnostics, vaccines and other interventions) 55

Conclusion and Next Steps....59

Appendix: Human Health : 60

Appendix 1: List of Human Health Structures Interviewed : 60

Appendix 2: List of Statutory Instruments :....60

Appendix 3: Human Health Bibliography : 61

Appendix 4: Data Collection Tool : 61

Appendix 5: Human Health Interview Questionnaire : 67

Appendix: Animal Health : 68

Annex 7: Summary of the main families of antibiotics used in animal health 74

Appendix 8: Some examples of antibiotic prescriptions in animal health 75

Appendix 9: Some examples of deworming prescriptions in animal health....76

Appendix 10: Some examples of endectocide prescriptions in animal health....77

Appendix 11: Some examples of prescriptions for external pesticides in animal health....78

Appendix 12: Some examples of blood antiparasitic prescriptions in animal health 78

Appendix 13: Some examples of anti-inflammatory drug prescriptions in animal health 79

Annex 14: Main diseases encountered in Mali and the vaccines used to prevent them....79

This report would not have been possible without the generous support of the American people through the United States Agency for International Development (USAID) through the MTaPS project. The views expressed in this document are those of Management Sciences for Health and do not necessarily reflect those of USAID or the U.S. Government.

About MTaPS

The objective of the Medecines Technologies and Pharmaceutical Services (MTaPS) project is to ensure the availability of quality pharmaceuticals and effective pharmaceutical services to achieve desired health outcomes. To this end, the areas of intervention of MTaPS include improving governance, developing capacity in pharmaceutical management and services, prioritizing information for decision-making in the pharmaceutical sector, strengthening financial strategies and mechanisms to improve access to medicines and increase quality pharmaceutical services.

Recommended quote
This report may be reproduced provided that MTaPS is mentioned. Please use the following citation.

Rapid assessment of stewardship policies and regulations and a rapid assessment of antimicrobial supply chain management in the human health sector.
Support from the U.S. Agency for International Development through the Medecines Technologies and Pharmaceutical Services (MTaPS) project. Arlington, VA: Management Sciences for Health.

Keywords
Antimicrobial drugs, drug regulatory system, evaluation

Medecines Technologies and Pharmaceutical Services (MTaPS)
Management Sciences for Health
4301 North Fairfax Drive, Suite 400
Arlington, VA 22203 USA
Telephone: 703.524.6575
Fax: 703.524.7898
E-mail :
Website: www. mtapsprogram.org

ACKNOWLEDGEMENTS

The Ministry of Health and Social Affairs (MOH) of Mali, through the Directorate of Pharmacy and Medicines (DPM), would like to thank the managers of the central structures that facilitated the realization of this rapid assessment. Special thanks to the structures that allowed the collection of data, especially through interviews, such as

- The Faculty of Medicine and Odonto-Stomatology ;
- The Faculty of Pharmacy ;
- The National Institute of Training in Health Sciences ;
- The Health Inspectorate ;
- The National Health Laboratory ;
- The National Centre for Health Information and Education ;
- The National Institute of Public Health and ;
- The World Health Organization

The consultant would like to thank all the people who gave their time during the interviews and visits to establishments. In particular, he thanks the Director of the DNSV, Dr. Idrissa COULIBALY, the Head of the Division of Veterinary Legislation and Standards (DLVN), Dr. Mohamed Lamine NIARE of the National Directorate of Veterinary Services (DNSV), and Dr. Satigui SIDIBE, Head of the Diagnosis and Research Department at the Central Veterinary Laboratory (LCV), National Focal Point for Anti-Microbial Resistance (AMR) at the Ministry of Agriculture and Fisheries (MEP), for their availability, as well as the following private sector actors

- Dr Mamadou SAMASSEKOU, representing Boehringer Ingelheim Animal Health and ex MERIAL;
- Dr Boubacar KEITA CEO of SIPROVET and President of the Groupement des Vétérinaires Grossistes (GVG) ;
- Dr Oumar TOUNKARA, President of the National Order of Veterinarians of Mali (ONVM) ;
- Dr Sidiki KONATE, President of the National Association of Veterinary Doctors of Mali (ANDVM);
- Mr. Modibo KOUYATE, Representative of Interchimie and promoter of MALIVET;
- Mr. Lamine TRAORE, Representative of Van Miert Dams and Promoter of Vet-Pro Distribution SARL.

The consultant particularly thanks Dr Karim TOUNKARA, Representative of the World Organisation for Animal Health, and Dr Boubacar DIALLO, Technical Adviser for Animal Health of the MEP, for their collaboration and the information provided concerning the role of the OIE in the fight against AMR.

The consultant also thanks Dr Ibrahim Guindo of the National Institute of Public Health (INSP), National RAM Focal Point, as well as the other members of the National Multisectoral Coordination Group and the staff of the Directorate of Pharmacy and Medicines (DPM) for the information provided

We are grateful to the USAID Mali office for funding this rapid assessment and for the technical support provided by Dr. Aligui YATTARA through the guidelines he provided.

HUMAN HEALTH

SUMMARY

The commitment of the government of Mali in the fight against antimicrobial resistance has resulted in several actions including the establishment of the "One Health" platform with a thematic group for antimicrobial resistance. On the other hand, the country has developed an action plan around 06 strategic objectives including the optimization of the rational use of antimicrobials.

The country has adopted a health orientation law of which the national pharmaceutical policy is an integral part. The implementation of this pharmaceutical policy is ensured by the DPM in collaboration with other institutions such as the Pharmacie Populaire du Mali and the Laboratoire National de la Santé.

This rapid assessment identified some gaps in the regulation and supply chain for antimicrobials.

Indeed, there is no specific regulation for antimicrobials unlike some therapeutic classes such as narcotics. This is observed in the marketing authorization procedures (MA). In addition, there is no up-to-date database for the monitoring and analysis of granted MAs. Information on violations of the regulations is not centralised and requires close collaboration between the departments responsible for applying and monitoring the rules. The quality of medicines is a crucial point in pharmaceutical policy. However, there is no "product-supplier" database or use of a database for procurement of antimicrobials outside health programs.
The country has a National List of Essential Medicines which is updated every two years. This list defines the prescription authorization in the health pyramid but there are no guidelines for the prescription of antimicrobials by health care professionals (e.g. number of antimicrobials per prescription). Antimicrobials on the current list are not organized according to the AWaRe classification. There are no standard treatment protocols for antimicrobial use outside of health programs.

The guidelines for the supply of health care facilities are codified in the Master Plan for the Supply and Distribution of Essential Medicines. This scheme is applicable to all drugs, with no specific reference to antimicrobials. It should be noted that the country does not have a policy for the donation of health products. The revision of the SDADME has been noted by several evaluations. The therapeutic committees within the institutions were announced in the PPN, but the hospital law and the operating mechanisms of the hospitals do not provide for therapeutic committees, unlike the hygiene committee notified in the hospital law

Mali has codified the modalities for providing care, particularly in the private sector, and has adopted guidelines on pharmacovigilance. However, reporting rates are quite low and there is no data on compliance with the NML.

PROPOSED ACTIONS

For efficiency in the fight against antimicrobial resistance, the implementation of certain actions becomes important:

- Improvement of the collaboration of the different actors for the implementation of the National Pharmaceutical Policy;
- Development of specific regulatory framework for antimicrobials;
- Development of a health product donation policy ;
- Establishing and monitoring the functionality of the therapeutic and pharmaceutical committees;
- Development of treatment guidelines for infectious diseases not covered by a specific program;
- Revision of the Master Plan for the Supply and Distribution of Essential Medicines ;

INTRODUCTION

The Government of Mali is committed to comprehensive health management through the establishment of the "One Health" platform in accordance with Decree 2018-0369/PR-RM of April 12, 2018 on the creation, attributions, organization and functioning of the "One Health" Platform in Mali. This platform includes several sectors, the main ones being: the animal health sector, human health, agriculture and the environment.

The country's commitment to the fight against Antimicrobial Resistance has resulted in the development and technical validation of a National Action Plan to Combat Antimicrobial Resistance (PAN-RAM), the establishment of the National Multisectoral Coordination Group (GCMN) and the appointment of a National Focal Point for the fight against Antimicrobial Resistance.
In accordance with the National Action Plan against Antimicrobial Resistance (PAN-RAM), several activities must be implemented to strengthen the fight against this phenomenon. These activities include conducting studies on the use and prescription of antimicrobials including strengthening the supervision of antimicrobial management and quality control. The national plan highlighted the inadequacy of the regulation of use and especially a low level of implementation of existing guidelines and regulations on antimicrobial use.

The major challenge for the country is the adoption of a National Antimicrobial Management Plan (AMS). According to PAN-RAM, the weakness of stewardship is due to insufficient multisectoral coordination and accountability at both the central and peripheral levels, insufficient implementation of recommendations, appropriate treatment guidelines for each level of the health pyramid, and insufficient communication on the results of evaluations and audits on antibiotic use in all sectors.

It is thus under the leadership of the Directorate of Pharmacy and Medicines (DPM) and its commitment to the implementation of strategic objective 4 "Optimized use of antimicrobial drugs, including the management of antimicrobial resistance (AMR)" that a rapid assessment of the regulation and supply chain of antibiotics in the human sector has been conducted. This action will enable the country to achieve capacity level 2 on the WHO benchmark and capacity 3.4 of the International Health Regulations for the optimization of antimicrobial use

The purpose of this report is to present the results of the rapid assessment of the human health regulatory framework and supply chain.

The report summarizes the observations and findings of the country desk review while taking into account the results of previous evaluations

METHODOLOGY

This rapid assessment was carried out in several stages:

- **Orientation of the actors:** the main objective of this step was to present the data collection tool and ensure its appropriation by the people in charge of data collection.

- **Collection tool:** the tool used for this collection is the one developed by MTaPS on the basis of the experience of several countries. It includes 38 questions divided into 5 areas of assessment which are: (i) National regulations, legislation or policies, (ii) Use of medicines and AMR policies/regulations, (iii) The list of Essential Medicines, (iv) Good supply management policies and, (v) Appropriate use by prescribers, dispensers and patients

- **Transposition of the questionnaire:** The questionnaire was transposed into an Excel spreadsheet to facilitate collection and analysis. The different sections of the questionnaire were divided into different tabs

- **Data collection and analysis: Data** collection and analysis were carried out by the focal points of the DPM and the MTaPS project. In order to facilitate data collection, during the orientation stage, the documentary sources were identified on the one hand, and on the other hand the investigators undertook to carry out a pre-collection of data.

The potential documentary resources identified were: the DPM website, the Ministry of Health website and the website of the General Secretariat of the Government. The DPM website was not functional and the other two did not provide the desired information. Thus, the collection team identified the evaluations carried out on the regulations, then the identification of institutions and resource persons who could have a collection of texts on health. Thus, the reports of the main evaluations were collected from the DPM (Integrated Drug Chain in Mali, Diagnosis of the Supply Chain, SWEDD project in 2018 and Delivery to the Last Kilometer) and supply chain partners such as MSH (Diagnosis of the Supply Chain), SIAPS (Technical Report_Assessment of DPM MRS).

Collections of texts were obtained through the National Council of the Order of Pharmacists (CNOP), the Health Inspectorate (IS) and the Support Unit for Decentralization and Devolution of the Ministry of Health (CADD).

RESULTS:

The results represent the findings of the literature review and the interviews with some of the structures.

These results are structured around a few areas which are: regulation, national pharmaceutical legislation or policy, drug use and AR policy/regulations and the National Essential Drug List, Good Supply Management Policies and appropriate use by prescribers, dispensers and patients

National regulations, legislation or policies :

Structures :

Several structures are involved in either the management of medicines or information on medicines. The establishment of these structures is supported by texts of creation and their functioning is codified according to their specificity:

Central level structures :

Directorate General of Health and Public Hygiene (DGHSP)

- Decree N°2018-0673 P-RM establishing the organic framework of the DGSHP
- Decree N°2018 0671 P-RM Fixing the Organization and the modalities of functioning of the DGSHP

Directorate of Pharmacy and Medicines (DPM)

- Law N°01-040 of 7 June 2001 ratifying Ordinance N°00-039P-RM of 20 September 2000 creating the DPM.
- Decree N°2011-753 P-RM of 17 November 2011 establishing the organization and operating procedures of the DPM.

Health Inspection

- Order No. 00-058/P-RM of 28 September 2000 establishing the health inspectorate.

-

National Institute of Public Health

- Order N°2019-011 creation of the National Institute of Public Health (INSP)
- Decree N°2019-0247 fixing the organization and functioning modalities of the National Institute of Public Health (INSP).

National Food Safety Agency (ANSSA)

- Law n° 03- 043 /PRM of 30 December 2003 establishing the National Agency for Food Safety.

National Centre for Disease Control Support (CNAM)

- Order No. 01 - 036 / P-RM of 15 August 2001 establishing the National Centre for Support in the Fight against Disease.

Central Veterinary Laboratory (LCV)

- Order N°79-76CMLN of 28 June 1979 establishing the Central Veterinary Laboratory (LCV).

- Decree N°222 PG-RM of 20 August 1979 on the organisation of the Central Veterinary Laboratory (LCV).

- Law N°94-027 of 1st July 1994 establishing the LCV as an EPA.

- Decree No. 94-251/P-RM of August 8, 1994, establishing the organization and operating procedures of the LCV.

National Health Laboratory (LNS)

- Order 00-040/P-RM of 20 September 2000 establishing the National Health Laboratory (LNS).

- Decree No. 00-586/P-RM of 23 November 2000 establishing the organization and operating procedures of the National Health Laboratory (LNS).

Popular Pharmacy of Mali (PPM)

- Law N°93-032 of 11 June 1993 on the creation of a public establishment of an industrial and commercial nature called Pharmacie Populaire du Mali (PPM).

- Decree N°93-240/P-RM of 14 July 1993 fixing the organization and the modalities of functioning of the Pharmacie Populaire du Mali (PPM).

National Centre for Health Information, Education and Communication (CNIECS)

- Decree No. 01 - 120 / P-RM of March 9, 2001, Determining the organic framework of the National Centre for Health Information, Education and Communication

- Decree No. 01 - 109 / P-RM of February 26, establishing the organization and operating procedures of the National Centre for Health Information, Education and Communication

- Law n° 01 - 033 / of June 4, 2001, ratifying Ordinance n° 01 - 006 / P-RM of February 19, 2001 creating the National Centre for Health Information, Education and Communication

National Centre for Disease Control Support (CNAM)

- Order n° 01 - 036 / P-RM of August 15, 2001, establishing the National Support Centre for the fight against the disease ;

- Decree No. 01 - 487 / P-RM of 24 May 2001, establishing the organization and operating procedures of the National Centre for Support in the Fight against the Disease

One Health Platform

- Decree 2018-0369/PR-RM of 12 April 2018 on the creation, powers, organization and operation of the "One Health" Platform in Mali.

Peripheral level structures :

Regional Health Directorate "

- Decree No. 90 - 264 / P-RM, on the creation of regional and sub-regional health and social affairs departments

- Decree No. 05 - 299 / P-RM of 28 June 2005, establishing the conditions for the creation and the fundamental principles of operation of Community Health Centres (Cscom)

- Community Health Centres Collective Agreement 2006

NB: Each hospital and CSRéf (central and regional level) has its own text of creation and the decree codifying its organization and functioning

Regulatory Analysis :

The declaration of the sectoral health and population policy adopted in December 1990 and confirmed by the orientation law on health adopted in 2002 , (law n°02 - 049 / of 22 July 2002 Portant loi d'orientation sur la santé) defines the National Pharmaceutical Policy (PPN) as an integral part of the sectoral health policy. The NPP was developed and adopted in 1999 and revised in 2012 to take into account the fundamental principles of equity, justice, solidarity, and participation of the population and civil society. It takes into account the major international commitments to which Mali has subscribed with its 2012-2016 Master Plan.

The regulation of medicines, including antimicrobials, is codified in the national pharmaceutical policy. The Directorate of Pharmacy and Medicines (DPM), responsible for pharmaceutical regulation, was created by Ordinance No. 00 - 039 /P-RM of 20 September 2000 and ratified by Law No. 01 - 040 / of 7 June 2001. According to article 2 of this ordinance, the mission of the Directorate of Pharmacy and Medicines is to elaborate the elements of the national pharmaceutical policy, to ensure its execution and to ensure the coordination and control of the services that contribute to the implementation of this policy. In this respect, it is responsible for defining pharmaceutical regulations, examining and monitoring applications for authorisation to place medicines on the national market, drawing up and implementing national programmes for essential medicines and developing tools to assist in the rational use of medicines.

Unlike narcotics and poisonous substances, for which there are several policies, there are no specific provisions for prescribing, acquiring and distributing antimicrobials. These include:

1. Decree No. 199 / PG-RM of 12 July 1988, establishing the list of narcotics;
2. Decree No. 245 / PG-RM of 12 July 1988, establishing the list of poisonous substances and plants;
3. Decree No. 96-302 / PRM of 7 November 1996, establishing a National Commission to Combat Drugs;
4. Decree No. 97- 230 / PRM of August 8, 1997, establishing the modalities of importation, storage and delivery of poisonous substances and narcotics;
5. Law n° 01 - 078 / of July 18, 2001, on the control of drugs and precursors;
6. Law No. 62-56 / AN-RM of 6 August 1962, authorizing the Government to accede to the Single Convention on Narcotic Drugs;
7. Act No. 95-045 of 12 June 1995, authorizing the accession of the Republic of Mali to the United Nations Convention against Illicit Traffic in Narcotic Drugs and Psychotropic Substances, adopted in Vienna on 20 December 1988.

The import authorizations and conditions of approval or Marketing Authorization (MA) are set by the orders: Inter-ministerial Order No. 91-2776 / Mef - Maee - Mspaspf, setting the conditions for importing pharmaceutical and veterinary products and Inter-ministerial Order No. 05-2203 / MS - MEP - SG of 20 September 2005, Determining the modalities of application for marketing authorizations (MA) of drugs for human and veterinary use.

According to Article 1 of the Inter-ministerial Order No. 91-2776 / Mef - Maee - Mspaspf which stipulates that the importation of pharmaceutical and veterinary products can only be carried out by natural or legal persons authorized for this purpose. On the other hand, article 2 of the interministerial decree n° 05 - 2203 / MS - MEP - SG of 20 Sept. 2005 indicates that the marketing authorisation (MA) is issued to the manufacturer of the product or, where applicable, to the legally declared operator of the MA, after examination by the national commission for marketing authorisations of a complete file submitted by the applicant. The MA application file is sent to the Minister of Health (article 4). This decree also sets out the documents to be provided for an application for approval depending on the case.

There is no documented procedure implemented to periodically review the MAs granted. On the other hand, there is no list/database of all authorized or evaluated medicines that is regularly updated. This document is called the "National Nomenclature of medicines for human and veterinary use authorised in Mali" which is not updated annually but is often available late.

The DMP does not have a maintained database of all applications received, drugs authorised, suspended or withdrawn as well as refused MA applications. In 2018, there were 4047 registered products of which 102 were in the field of animal health.
The mission of the DMP is to improve access to quality essential health products for patients. Marketing authorization is granted according to the country's guidelines and WAMU directives.

In the work of the licensing committee, the NMEA is used as a reference. Almost all of the NMEA antimicrobials are listed in the WHO list and therefore some are not listed in the AWaRe classification (14 drugs): Cefepime injectable 1g and 2g, Ceftaroline powder 600mg, Erythromycin tablet 500mg, oral suspension 250mg/ml and 125mg/ml, Levofloxacin tablet 500mg, Ofloxacin ear drop 1.5mg/0.5ml and eye drop 0.3%, Sulfadiazine tablet 500mg and injectable 250mg/4ml, Moxifloxacin eye drop 0.5%, Tetracycline ointment 1% and 3%.

The operating licenses for pharmacies and wholesalers are granted by the Ministry of Health and monitored by the DPM. In addition to the DPM the monitoring of the provisions is carried out by the Health Inspectorate (IS) and the National Council of the Order of Pharmacists (CNOP) created by Law No. 86-36/AN-RM on the institution of the National Order of Pharmacists and amended by Law 2017-031 of 14 July 2017, on the creation of the National Council of the Order of Pharmacists Article 10 of Law 2017-031 of 14 July 2017 specifies that no pharmacist can practice his art on the national territory if he is not regularly registered in the roll of the Order of Pharmacists of Mali, including those of the active cadre of the Armed Forces and Articles 09 to 13 of the said law specify the conditions of registration in the Order of Pharmacists. The law also specifies the disciplinary sanctions and mechanisms. In accordance with article 12, the National Order of Pharmacists has sections that are specified by regulation and the conditions for registration and removal from the roll are specified by decree.

Applications for operating licenses are sent to the Order of Pharmacists by the applicants and, after analysis, the files are sent to the DMP. The DPM carries out the quality assurance of the documentation and then transmits its opinion to the Minister in charge of health for final decision. The DPM has the resources to document the licenses granted with an annual update. At the end of

2019, Mali had 604 licences for private pharmacies and 71 licences for wholesalers. The opinions of the DPM, following the analysis of the files are: the return for additional file and the rejections.

Thus, of all the license applications forwarded to the DPM on account of 2019, the following situations can be noted:

- Authorization to practice: 17 letters to complete files out of a total of 129 applications;
- Operating sites: 7 letters for file completion out of a total of 90 requests
- Opening of a private dispensary: 6 letters for completion of files and 02 rejections out of a total of 67 requests;
- Pharmaceutical wholesaler: 3 letters for completions out of a total of 8 requests;
- Opening of drug deposits: 2 letters for completions out of a total of 3 requests;

All three structures (DPM, the Health Inspectorate and the CNOP) have their own reporting and sanctioning mechanisms. The DPM addresses its recommendations to the Minister of Health, the Health Inspectorate to the President of the Republic, the Prime Minister and the Minister of Health. The sanction mechanisms are implemented in practice, however, there is no documented procedure for this purpose and data on violations are not capitalized. Consequently, it seems important to put in place a coordinated mechanism for monitoring pharmacies and wholesalers.

Recent regulatory assessments indicate that there is a policy of registration of personnel. All pharmaceutical operations (public and private) are subject to an operating license issued by the Ministry of Health on the advice of the DPM. However, this provision has shortcomings, such as the lack of information on the time required to process files (Rapport d'Evaluation du Système Pharmaceutique au Mali, PQM, Pr A. Toumi).

Order No. 91-4320 / Msp-as-pf / Cab sets out the rules for pharmaceutical manufacturing establishments without any particularity for antimicrobials. These rules are based on the WHO "good manufacturing guidelines". On the other hand, these rules constitute the annexes of the said decree.

These rules assist the operators of manufacturing units in the areas of (i) Conditions of Practice in Drug Manufacturing Establishments which deals with the private practice of the profession of drug manufacturer, Operation of Drug Manufacturing Establishments, Conditions of Operation of Drug Manufacturing Establishments, Equipment and Personnel, (ii) Manufacturing of Other Pharmaceutical Products which deals with the eligibility of practice of the operation

Mali has very little drug manufacturing industry. Thus, most medicines are imported mainly from China and India (MSH Supply Chain Diagnostic, 2017). Imports are subject to authorizations, however, there is no specific provision for the import of antimicrobials as the same verification rules including import fees apply.

Mali benefits from grants from the Global Fund to Fight Malaria, HIV and Tuberculosis. This grant represents more than 95% of the needs in antiretroviral drugs. All of these drugs are of Indian origin. This observation is the same regardless of the procurement agent (UNDP, WAMBO/VPP and PPM).

To date, there is no database at the DPM (import approval structure) on the quantities of drugs imported, including antimicrobials. Therefore, the share of the Central Purchasing Office cannot be estimated. Some studies have been done on imports. However, these studies have focused on overall volumes and amounts without differentiating between the different classes of drugs.

Decree No. 04-557/P-RM of December 01, 2004, instituting the marketing authorization of medicines for human and veterinary use does not contain regulations or guidelines on the import and export of medicines. This was identified as a major issue in the MSH/SIAPS assessment of the DPM regulatory system in 2017. However, this strengthening could not be done due to the project timeframe and available funds (according to MSH/SIAPS project officials).

However, the selection of antimicrobials for health programs subsidized by the Global Fund is made through the lists of prequalified drugs (WHO or Global Fund). On the other hand, the purchase of other antimicrobial drugs is done without taking into account the prequalification list and the PPM has not set up a "product - manufacturer pair" database. This provision had been identified as a major point in the PPM capacity building plan, however the activity has not been operationalized. The provisions of Inter-ministerial Order No. 91-2776 / Mef - Maee - Mspaspf, setting the conditions for importing pharmaceutical and veterinary products indicate in Article 5 that the complete file is submitted to the National Directorate of Economic Affairs, which, after study, submits the draft decision for approval by the Minister of Trade.

Once in the country, the supply of public health care structures is codified in the Master Plan for the Supply and Distribution of Essential Medicines (SDADME). Compliance with the said scheme is supported by Decision No. 2011-774/MS-SG of July 11, 2011, making the application of the Master Plan for the Supply and Distribution of Essential Drugs Manual mandatory.

The country developed good manufacturing practices and good distribution practices in 2007. However, no regulatory framework for their application was found.

In accordance with the mandate of the Health Inspectorate, missions are carried out to ensure compliance of practices with the regulatory framework in force. The areas of drug inspection do not focus on antimicrobials and the inspection reports are transmitted only to the Minister in charge of health, the Prime Minister and the President of the Republic. These reports provide information on (i) the agents, services and organizations inspected; (ii) the observations made; (iii) the errors and violations committed; (iv) the corrective measures taken and the desired improvements; (v) the reforms for the proper functioning of the inspected services and organizations.

However, not all these elements are systematically listed (Classification/Categorisation of non-conformities observed according to the potential risk/impact on the quality of medicines (critical, major and minor). However, there is no database of all the establishments inspected and maintained by the inspection.

Drug donations are governed by a regulatory framework. This framework is not specific to antimicrobials but no specific regulation has been found. Indeed, in the procurement policy (SDADME) revised in 2010 with decision 2011-774/MS-SG of 11 July 2011 making its application mandatory. The SDADME indicates the guidelines for drug donations. These guidelines are the WHO guidelines that have been inserted into the SDADME. It should be noted that several recent

evaluations have recommended the revision of the SDADME including that of MSH (Supply Chain Diagnostic, July 2018).

There is a mechanism for monitoring the quality of products placed on the market. This provision is implemented by the LNS. In accordance with the provisions of the national pharmaceutical policy, the National Health Laboratory is responsible for post-marketing quality control. The services for the control of the pharmaceutical practice should collaborate more in order to facilitate the sharing of information, its management and the application of measures for a better pharmaceutical service. Also, as part of the "One Health" initiative, the Central Veterinary Laboratory should be an important link in the post-market surveillance of antimicrobials.
Thus, quality controls are carried out by the quality control laboratories on samples during missions to ensure the quality of medicines distributed and on sale. Non-compliant results are shared with the DPM and the Health Inspection. In 2017, the percentage of non-conformities at import (delivery) was 1.33% and 3.67% at marketing station (Supply Chain Diagnosis, MSH, 2018 on Global Fund funding). These non-conformities were related to the dosages of products tested. The quality controls carried out involved 1269 and 491 samples in 2018 and 2019 respectively. According to the indications of the LNS, this decrease is partly related to the decrease in purchases and pre-marketing control during the applications for Marketing Authorization. The 2018 results show that 64 samples are below standards, including 3 at import and 61 at post-marketing. However, these non-compliances did not involve antibiotics. Of the 491 samples tested in 2019, no cases of non-compliance were detected.

Inter-ministerial Order N°83735/MS-MPP/SG of 3 December 2008 defines the conditions for advertising medicines and the conditions of practice of medical representatives. However, there are no legal provisions imposing the use of the information approved on products within the framework of the MA as reference materials for the control of promotion and advertising activities as well as of all advertising materials. On the other hand, there is no proactive monitoring programme to detect promotional activities or advertising campaigns in scientific journals or literature for health professionals. To date, there is no regulatory framework for the purchase of pharmaceutical products by the general public (online purchases).

The review of the regulatory framework shows that there is no specific framework for antibiotics unlike some other pharmaceutical products (drugs). In order to reflect the strong commitment of the country in the fight against antimicrobial resistance, specific provisions must govern the acquisition, distribution, use and dispensing of antimicrobials.

DRUG USE AND PLAR POLICIES/REGULATIONS

Therapeutic protocols exist for tuberculosis, malaria and HIV which are regularly updated. The closest document for the management of infectious diseases is the National Antibiotic Therapy Reference Manual developed in 2014 by the National Agency for Hospital Evaluation with the support of the Malian Society of Infectious and Tropical Pathologies (SOMAPIT).

Indeed, therapeutic guides and forms had been developed respectively in 2009 and 2006 for the main pathologies but have not been updated. On the other hand, these documents almost no longer exist in the health facilities, probably due to the very high turnover of health professionals. During the

orientation workshop on the collection tool for this rapid assessment, several participants stated that they had not seen these documents (Guide and Therapeutic Form).

Adherence to the protocols is mandatory for the pathologies of the health programs. For other infectious diseases, adherence assessment is not available.
The drug needs of health facilities should be in accordance with the provisions of the therapeutic protocols for the programs and the NML. However, it should be noted that Mali's NML takes into account the drugs of all the country's health programs.

The NML shows the drugs according to the health pyramid in the public system. However, based on the NML analysis, the availability of ACCESS drugs decreases from the central to the peripheral level. There is no formal framework for assessing compliance with national treatment protocols (NTPs). The SDADME monitoring indicators provide for the evaluation of the number of prescriptions with at least 01 antibiotics at the level of certain health facilities (CSCom and CSRef). However, there is no regulation limiting the number of antibiotics per prescription. No evaluation of the prescription in the private sector is available.

NATIONAL LIST OF ESSENTIAL MEDICINES

The National List of Essential Medicines is developed on the basis of the WHO format and is revised in several stages:

- **A phase of informing health care providers about the revision:** at this stage, correspondence is sent to the various facilities/units to identify the products that should be included in the list, while indicating additions and deletions based on the previous list. Providers are asked to indicate the reasons for the addition or deletion based on the evidence.

- **Preparatory phase:** This is done by a small technical group made up of certain managers of facilities or health care providers. The information received (additions and deletions) is compiled, analyzed and compared with the latest WHO list. Following the work, the workshop to revise the list is planned.

- **Review workshop:** the purpose of this workshop is to validate the drugs selected in the preparatory phase, validate the prescription levels of each drug according to the health pyramid.

At the end of the review workshop, the regulatory authority (DPM) initiates and submits the draft application order of the new list to the Minister in charge of health and plans the multiplication and dissemination of this new list. The list is nowadays published in poster and booklet formats. The latter are of three types, depending on the health pyramid.

Order N 2019-2521 MSAS-SG of August 26, 2019 sets the list of Essential Medicines in Mali and that according to the health pyramid. This list is revised every two (02) years. After ratification of the list by the decree, the DPM transmits it to the care structures in the public sector.

The SDADME supervisions target a certain number of medicines according to the objectives of the mission, without any particularity for antibiotics. The NEML is articulated around the health pyramid, which is composed of the central level (hospitals), districts (CSRéf) and community (CSCom) with a

particularity for the CSCom with a doctor. According to the provisions of the NEML, all health personnel are authorized to prescribe antibiotics in relation to the level of care provided. However, there is no evaluation of the implementation of the NML. The NML defines the levels of antibiotic prescribing in the health pyramid. This 2019 list includes 67 antibiotics, all of which are authorized in hospitals, 55 references in CSRéf, 49 in CSCom with physician and 39 in CSCom without physician. No other text regulates the restriction of prescribing. This is illustrated in the examples below:

- Gentamycin Injection 10mg/ml is allowed at all levels except CSCom without a doctor;
- Clindamycin 150mg tablet is not allowed in CSCom with or without a doctor;
- Injectable cotrimoxazole is only allowed in hospitals and is not allowed in the RefSCs and ComHCs.

There is no regulatory framework for the management of infectious diseases, although several formal frameworks exist to initiate such an approach, such as the health council and the surveillance committee.

In addition, the country has adopted the AWaRe classification, which could address some of the GAPs in antimicrobial use, including assessing compliance with the NML and establishing a system for reporting susceptibility and resistance data .

Antibiotics of the "ACCESS" category not authorized in CSCom without a doctor in Mali

Amoxicillin + Clavulanic Acid	Injectable	500 mg + 62.5 mg
Amoxicillin + Clavulanic Acid	Drinkable suspension	125 mg + 31.25 /5 ml
Amoxicillin + Clavulanic Acid	Drinkable suspension	250 mg + 62.5 /5 ml
Cloxacillin	Injectable	500 mg
Amikacin	Injectable	500 mg
Clindamycin	Injectable	150 mg/ml
Clindamycin	Tablet	150 mg
Cotrimoxazole	Injectable	80 mg + 16mg/ml
Gentamycin	Injectable	10 mg/ml
Gentamycin	Injectable	40 mg/ml
Metronidazole	Injectable	500 mg/100ml

Antibiotics of the "ACCESS" category not authorized in the CSCom with doctor in Mali

Amoxicillin + Clavulanic Acid	Injectable	500 mg + 62.5 mg
Amikacin	Injectable	500 mg
Clindamycin	Injectable	150 mg/ml
Clindamycin	Tablet	150 mg
Cotrimoxazole	Injectable	80 mg + 16mg/ml

Antibiotics of the "Watch" category not authorized in CSCom without a doctor in Mali

Cefotaxime	Injectable	500 mg
Ceftriaxone	Injectable	250 mg
Ceftriaxone	Injectable	1000 mg
Ceftriaxone	Injectable	500 mg
Ciprofloxacin	Injectable	200 mg/100 ml
Vancomycin	Injectable	1 G
Azithromycin	Eye drops	1.50%

Antibiotics of the "Watch" category not authorized in the CSCom with doctor in Mali

Cefotaxime	Injectable	500 mg
Ciprofloxacin	Injectable	200 mg/100 ml
Vancomycin	Injectable	1 G

In addition, a review of the National Repository of Antibiotic Therapy developed in 2014, allows us to note that several drugs of the RENAM do not exist on the LNME. These are nalidixic acid, flucoxacillin, lincomycin, tinidazole.

On the other hand, an evaluation of the performance of the supply chain carried out in 2017 within the framework of the SWEDD project showed that more than 25% of the references (drugs) of the NML were not available in the CSRéf and more than 35% in the CSCom.

The Faculty of Medicine and the Faculty of Pharmacy have adopted the WAHO training curriculum and Syllabi have been developed for the various teaching modules.
The teaching of treatment protocols for infectious pathologies is not formalized. The students are informed of the existence of these protocols, depending on the teachers' involvement profiles in the different pathologies.

Indeed, during the bacteriology or infectiology courses, treatment protocols such as HIV or immunology (Expanded Programme on Immunization) are announced to the students and more structured skills are acquired by the latter during advanced training courses in the various structures providing care.
According to some of the actors interviewed, the academic training cycles should concern general education and it is up to the state to provide training on treatment protocols when the agents take up their duties.

However, due to the turnover of health personnel and the fact that the authorization to provide care is issued by the state, it will be up to all these providers to respect the country's guidelines for the provision of care.

In light of the question on national treatment protocols, the concept of a national essential drug list is not formally taught. Students learn the concepts during their internships in the facilities.

It should be noted that the space for this teaching exists because the modules of pharmacology or therapeutics are taught in the different levels of education and this according to the profile of medicine, pharmacy and nursing.

In the Faculty of Pharmacy, according to some academic leaders, the concept of NML is recalled during the teaching of community health modules during which the National Pharmaceutical Policy is taught.

Postgraduate courses (DU) are organized e in the country. During these trainings, specific modules of national therapeutic protocols are taught.

However, these courses are not free of charge, which limits the participation of a larger number of professionals.

GOOD PROCUREMENT POLICIES

The distribution network for essential medicines (EM) in the public and community sectors was set up in Mali in 1995, as a result of the pharmaceutical supply component of the sectoral health and population policy adopted in December 1990. In order to strengthen this network, a complete mechanism called "Essential Drug Supply Master Plan (SDAME)" was adopted in 1995 and revised in 2010 to become "Essential Drug Supply and Distribution Master Plan (SDADME)", with the objective of ensuring a correct supply of quality essential drugs to the population throughout the national territory, and at costs compatible with their purchasing power.

The country has already adopted for several years, lists of essential medicines with a regular revision every 2 years. This list has the advantage of taking into account all the drugs, including those of the country's various health programs. However, there are no guidelines for the importation, prescription and dispensing of antimicrobials.

According to the provisions of Order No. 96 - 0814 / MFC-SG, the stocks of pharmaceutical and veterinary products of pharmaceutical and veterinary industrial units and importing wholesalers are declared monthly to the Minister in charge of human health and the Minister in charge of animal health respectively.

However, information on these proportions does not exist because the data are not regularly capitalized and analyzed. Nowadays it is difficult to estimate the total volume of antimicrobials used in the country including the share of imports.

DMP has not implemented a quality management system for all its regulatory processes. Activities related to the control of drugs at import are organized in accordance with the NPP for the public sector and these provisions are not available in the private sector. Thus, quality control is not performed at the time of import (delivery) on behalf of the private sector.
There are two drug quality control laboratories in the public sector, the LNS and the LCV. The LCV only performs qualitative tests and is mainly used by the animal sector, whereas the LNS performs qualitative and quantitative tests and is mainly used by human health. It should be noted that the LNS has ISO 17025 certification for microbiology but not for drugs.

Purchases by the central purchasing office are made in a different way. In fact, for program products, the products are purchased through pre-qualified manufacturers, but the central purchasing office has not set up a product-supplier pair database, either for pre-qualified products or for other drugs in the market sector

In the case of MA applications and in the procurement process by the MPC, the provision of samples is an obligation. The samples received are inspected. Also, quality controls are carried out at the time of the MA applications, during the delivery of the products and in post-marketing. However, it should be noted that marketing authorisations are often granted before the certificates of analysis of the tested samples are made available. This situation is due to the delay of the LNS in the acquisition of reference substances and this because of the difficulties of mobilization of financial resources by the LNS.

However, the interministerial decree n° 08-0345/MS-MFMEIC MEP-MA of February 13, 2008, fixing the practical modalities and tariffs of the operations of quality control of medicines, food, water and beverages stipulates in article 14 that the expenses of quality control of the aforementioned products represent 0,5% of the FOB value.

The said decree does not specify the payment mechanism, which means that the fees paid by the importers end up at the level of the Customs Services without any possibility of recovery by the LNS. On the other hand, this decree is not explicit about the samples in the case of applications for MA

In the private sector, drug imports are mostly demand driven, including unregistered drugs. Compliance of imports with the NMA is not assessed in the public or private sector.
The focus/themes of SDADME supervision in the public sector do not include checking for compliance with the NML. However, for health programs these checks are regularly performed between patient regimens and current treatment guidelines.

DMP does not have visibility on compliance with the NML by the private sector (wholesalers and private dispensaries).

The annual purchase plans of wholesalers are not made available to the DMP. To date, in addition to wholesalers, some private pharmacies import medicines. It should be noted that all imports, including those of medicines, are subject to an import authorization. This authorization is issued by the National Directorate of Trade and Competition (DNCC). In order to issue the import license, the invoices for the drugs are submitted to the DPM for human health and the DNSV for animal health for approval.
As a result, DMP and DNSV have information on the importation of drugs including antimicrobials. However, they do not have a database in place to review and compare compliance between these imports and the NML.

In Mali, there is no policy to promote local drug manufacturing units. This provision would have made it possible to reduce the list of products to be imported into the country while putting in place an adequate regulatory framework for the efficiency of the measure.

Thus, a formal consultation framework should be established for the coordination and evaluation of the quantities of antimicrobials imported by Mali.

In addition, a compilation, review and analysis mechanism should be put in place to assess quantities, compliance of imports with the NMIH

As part of the monitoring of the Good Distribution Practices Guides, the DPM informs and organizes the withdrawal of batches or drugs such as the withdrawal of chloroquine from malaria treatment protocols (Circular No. 1774 / MS-SG on the withdrawal of chloroquine, 2006).
The DMP website is not up to date and information on medicines is not made available to the public, and there is no independent system for informing the public about medicines.

Chapter 5.9, entitled "Illicit market and counterfeiting of medicines" of the National Pharmaceutical Policy provides for interventions in this regard. Thus, the issue of falsified medicines in general is transversal to the services of the Health Inspectorate, the National Health Laboratory and the Directorate of Pharmacy and Medicines.

DMP capitalizes WHO alerts related to substandard and falsified medical products including 5 alerts in 2019 and 3 in 2018. The majority of these alerts concerned vaccines. For example, in 2018, based on a WHO alert, on the non-compliance of certain batches of medicines including amoxicillin/clavulanic acid, the LNS carried out quality controls and no non-compliant batches were found.

Also, in 2017, with the support of the PQM project investigations of falsified antimalarial drugs were done by the DPM. It emerged that the batches concerned were below the standard. Thus, the DPM activated a mechanism of recall of the batches with seizure of stocks by the investigators and information to all actors for the withdrawal of the batches concerned.

For example, drug literature is available for antimalarials.

It should be noted that the LNS carries out quality controls in food microbiology for which this establishment has received a 17025 certification. As such, it was able to identify batches of adulterated milk "Pico". This investigation was done in collaboration with the National Food Safety Agency (ANSA)

APPROPRIATE USE BY PRESCRIBERS, PROVIDERS AND PATIENTS

The provision of health care in the private sector is regulated by Decree No. 91-106/P-RM of 15 March 1991, on the organization of the private exercise of health professions, and Order No. 91-4319 / Mspas-Pf-cab of 3 October 1991, which sets out the terms of organization of the private exercise of medical and paramedical professions. The criteria for the exercise of the different professions are described as doctors, dental surgeons, midwives, paramedics and the different health care establishments (medical clinic, surgical clinic and delivery clinic). This decree also indicates in chapter 8 and 9 the modalities of controls of the exercise of the medical profession and the establishments.

In the public sector, however, there is no such provision. On the other hand, in both the public and private systems, there is no framework for the renewal of the various authorizations, either for practice or for operation.

Training/refresher courses are conducted in the programs (HIV, Malaria or Tuberculosis). These trainings are generally carried out in case of changes in treatment protocols or modifications in the management of these diseases. The other infectious diseases are not set up as health programs. They are rather dispersed among different medical specialties.

There is no formal provision for post-academic training. Once granted, authorization to prescribe (public and private) or care facilities are not renewed.

Post-academic training in the country is provided through University Diplomas (DU) or Post-University Courses (EPU), seminars and conferences, colloquia and short-term practical training in services, especially public services, on treatment protocols for health programs (malaria, HIV, tuberculosis, GASPA, etc.) or in specific areas such as logistics management. These training courses within the framework of DU and EPU, conferences and seminars are not free of charge, which limits access to a larger number of practitioners. Training outside the country is also possible and is always paid for. On the other hand, the training/retraining carried out in the public system is financed by the Technical and Financial Partners (TFP), which raises the question of the sustainability of this training.

The state is committed to the establishment and operation of a pharmacovigilance system. Training has been carried out and notification forms have been made available, but the system is struggling to get off the ground. This has led to several attempts to strengthen it, including with the support of several partners. Thus, it seems necessary to review the implementation of pharmacovigilance, including the regulatory framework and the implementation structures.

Order N°08_224_/MS-SG of January 30, 2008, establishing the modalities of implementation of pharmacovigilance in Mali. However, gaps existed in the roles and responsibilities of the 02 implementing entities which are the DPM and the CNAM. This led to the decree n° 2011-4201. Despite these amendments, the pharmacovigilance system put in place but still does not function properly. However, antibiotic resistance is a reason to report. According to the CNAM, available data indicated that from 2011 to 2017, the total number of reports regarding adverse reactions amounted to 120, and mainly concerned skin disorders (Evaluation of the DPM drug regulatory system, Mali, 2017, MSH/SIAPS).
The side effects notified in 2019 are 241 for the whole country and 70.7% of these notifications concern the products of the Seasonal Malaria Chemoprevention Campaign (CPS) and of which sulphonamides were at 2.9%.

As part of the implementation of PAN-RAM activities, WHO, in collaboration with the DPM, has initiated several evaluations, including one on the use and consumption of antibiotics. The results of these studies will make it possible to establish the basis for consumption.
It should be noted that in anticipation of epidemic peaks and seasons (dry: meningitis, winter: cholera, malaria...) and themes (hygiene), public awareness and information campaigns are organized by the State, mainly through the National Centre for Information, Education and Communication on Health (CNIECS).

On the other hand, the launch of the vaccination campaign and the distribution of impregnated mosquito nets all take into account the communication component.

The NML defines the levels of antimicrobial prescribing in relation to the level of care provided. However, it does not identify the medical personnel who are authorized to prescribe them. A comparison of the NML with the AWaRe classification shows that some antibiotics, although listed in the ACCESS category, are only allowed in certain facilities; therefore, antibiotics in the Watch category are found in the level of care that often does not have physicians. Some of the NML antibiotics are not listed or have been removed from the WHO 2019 list such as Erythromycin and Ofloxacillin.

The Community Health Centres allow the country to achieve the vision of universal health coverage. The establishment of these CHCs is governed by Decree No. 05-299/P-RM of 28 June 2005, which sets out the conditions for the creation and the fundamental principles of operation of the Community Health Centres (CHCs) and the 2006 CHC collective agreement.
Rational use could lead to a reduction in the use and prescription of antibiotics and therefore potentially in the cost of prescriptions for patients.
The hospital law (Law No. 02 - 050 / of 22 July 2002), sets up the management committee and (04) advisory committees including the technical committee of hygiene and safety, the medical committee of the establishment, a technical committee of the establishment and the committee of care and obstetrics. However, there is no regulatory framework for the organization, composition, functioning, mandate and responsibilities of the therapeutic and pharmaceutical committee.
Therapeutic committees are announced in the national pharmaceutical policy without any guidance as to their implementation, unlike the health and safety committee which has a legal framework.
If the country envisages that the Hygiene Committee / IPC and AMS be coordinated by the Therapeutic Pharmaceutical Committee, the regulatory framework for their operation should be put in place quickly.

A survey on prescribing carried out by the DPM with the support of the WHO revealed that 52.5% of prescriptions in EPHs included an antibiotic compared to 72.1% for CSRéfs and 80% in CSCom (DPM 2018 evaluation report financed by the WHO). This trend was observed in the assessment of the supply chain carried out by MSH which revealed that 55% of prescriptions in the health pyramid included at least one antibiotic.
Also, during the "Diagnosis of the MSH supply chain" mission, it was found that antibiotics represent at least 50% of prescription costs. The analysis of the cost of prescriptions in the CSRéf showed the following results 2800 CFA, 3332 CFA, 1968 CFA and 1432 CFA respectively in 2017, 2015, 2013 and 2011.

In terms of communication, Mali has a National Action Plan to fight against resistance. This plan is composed of 6 strategic objectives. Strategic objective 1 "To improve awareness and understanding of antimicrobial resistance through effective communication, education and training". Thus, a communication plan has been developed by the working group in charge of this objective. This plan is linked to that of the "One Health" platform.

Mali has planned annual campaigns in connection with the World AMR Week.
Thus, in 2018, thanks to the support of WHO specific communication campaigns to antimicrobial resistance were conducted. These materials of these campaigns were produced by CNIECS and

broadcasting by national television and free radios. The television broadcasts lasted about 10 minutes and were shown before the news (prime time) for 15 days. The interventions were multidisciplinary, ranging from the Minister of Health to the directors of the central services (DPM), the consumers' association and the citizens through "micro-trottoir" programmes.

National campaigns on antimicrobial resistance, like the global campaigns for 2019, have not been carried out; however, thoughts are underway for other types of communication means on AMR (conference in schools, visits to livestock farms, etc.)
In accordance with the action plan for strategic objective 1, new campaigns are planned with a focus on health-care professionals with the support of WHO and FAO/ECTAD.
However, the DMP (2019) awareness campaigns were mainly focused on rational use of medicines and combating the illicit market of medicines without specificity for antimicrobials.

Data are not available on the impact of these campaigns. However, CNIECS has already conducted impact evaluations of awareness campaigns on malaria chemoprophylaxis. Impact evaluations of awareness campaigns have also been conducted with the KJK project to evaluate community involvement (Ouelés-Sebougou area).

SUMMARY OF VARIANCES BY SECTION OR AREA

Section or area	Force	Weakness
Institutional	Existence of a regulatory body	Structural and organisational weakness, sanctions policy in the pharmaceutical field<br>Lack of a health product donation policy, collaboration between key players in the implementation of the NNP,<br>Lack of a training directory
National regulations, legislation or policies	Existing framework for several types of products such as drugs and vaccines	Lack of specific antimicrobial framework, revision of SDADME, functionality of DMP website,<br>Absence of a Pharmaceutical and Therapeutic Committee
Drug use and PLAR policies/regulations	Existence of a regularly updated NML	Lack of treatment guidelines for infectious diseases not covered by a specific program
National List of Essential Medicines	The list is supported by an order of the Minister of Health and defines the prescription authorizations for the entire health pyramid	Lack of assessment of compliance with the NMEA
Appropriate use by prescribers, providers and patients	National pharmacovigilance system in place and antimicrobial resistance is a reason for reporting	Inadequate case reporting, DMP website functionality

ANIMAL HEALTH:

OPERATIONAL SUMMARY

INTRODUCTION

Mali, like many countries in the sub-region and in the world, is facing the emergence of AMR. This has been proven by the results of analyses carried out by the country's laboratories. Following the example of the Human Health sector which has already been the subject of a rapid assessment, the AMR Secretariat has recruited a national consultant to carry out the present study.

OBJECTIVES, SCOPE AND CONTEXT OF THE EVALUATION

The assessment focused on five key areas: national antimicrobial legislation, regulations or policies, Standard Therapeutic Protocols (STPs), Essential Medicines List (EML), supply management policy and appropriate use by prescribers, distributors and farmers.

The key issues were Marketing Authorisation (MA) and Registration of Veterinary Medicines, Licensing, Control Procedures for Veterinary Medicines, Control at the time of MA application, Import Control, Pharmacovigilance (PV) Control, Prescription, Distribution and Use,
A list of the people and institutions met with is attached.

SUMMARY OF FINDINGS

National antimicrobial legislation, regulations or policies

It was noted that there is a regulatory framework for veterinary drugs in Mali, but the provisions are often incomplete and their application is still incomplete. In addition, there are no specific regulations concerning the use of antimicrobials.

The Essential Drug List (EDL)

There is a regulation in Mali namely Decree No. 66/PG-RM of 11 March 1985 which sets the nomenclature of essential medicines for the health protection of livestock. This provision was amended in 2015.

There is no training system for the LME concept in schools specializing in the training of veterinary para-professionals in Mali.

The policy of good supply management

Law N°2016-004 of 12 February governing veterinary pharmacy in Mali stipulates that, with the exception of medicated feeds, no veterinary medicine can be issued to the public unless it has first received a MA issued by the WAEMU Commission. However, several products have no MA (local industry) or an expired MA

The management of veterinary drugs is uncontrolled: uncontrolled import and distribution channels, recourse to self-medication because of the existence of parallel markets and the low level of knowledge of farmers, weak action by the control services on fraudulent channels and counterfeit products. These observations were made during previous PVS

Appropriate use by prescribers, distributors and breeders

The distribution of veterinary drugs was a major critical point in the performance evaluation of the Veterinary Services (PVS). It is codified by a regulatory framework, but nevertheless, this distribution is done in the greatest disorder, including by people without any qualification, without any real control and without knowledge of all the technical rules in the matter.

Due to unbridled and often illegal competition, ineffective, outdated and even dangerous products are found on the market.

The products are used as a first line of defence, often against the grain, directly by the farmers.

Suggestions

The Prime Minister should :

- Engage the Government with strong political will to support the human, animal and plant health and environmental health sectors so that the authorities in these respective areas are able to rigorously monitor the implementation of the National Action Plan on AMR;

- Allocate financial resources by providing a budget line at the national level in order to encourage the different sectors and ministries to participate in the fight against AMR in a sustainable manner and to promote awareness and training in all sectors.

The Ministers of Health, Agriculture, Livestock and Environment should :

- Build technical capacity to address AMR in the areas of human, animal and plant health and the environment.

The National Directorate of Veterinary Services should :

- Strengthen regulatory frameworks to ensure access to safe, effective and quality antimicrobials;

- Take urgent measures to develop the texts of application of the law N°2016-004 of February 12, 2016 governing veterinary pharmacy in Mali by emphasizing in particular the problem of pharmacovigilance ;

- Prevent the production, distribution and consumption of substandard or adulterated veterinary products for animals and prohibit the use of antimicrobials for growth promotion in the absence of a risk analysis.

- Promote and implement good practices at each stage of the production and processing of food of animal origin;

- Increase biosecurity on farms to avoid infections by improving hygiene and animal welfare ;

- Provide an appropriate regulatory framework for animal use of critically important antimicrobials in humans;

- Phase out the use of antimicrobials as growth promoters in animals ;

- Design communication tools and interventions to change antimicrobial use behaviour in the field;

- Establish a database for monitoring and collecting quantitative data on antimicrobial use in animals;

- Adopt the concept of Standardized Therapeutic Protocols (STP) ;

- To design and implement simple and efficient control programs for the detection of antimicrobial residues in Food of Animal Origin (FAME);

- Revise the texts to adapt them to the current context.

The main actors and institutions involved in the use of antimicrobials in animal health and their roles and responsibilities are listed in the body of the report.
Also, intervention lines and follow-up mechanisms to address the main problems identified as well as an action plan for antimicrobial stewardship in the field of animal health were proposed.

Conclusion

While recent achievements and sustained government efforts are helping to prepare Mali to respond appropriately to the continuing threat of AMR, the challenge is far too complex and multi-faceted to tackle alone.

A truly national approach to AMR requires the active participation of a wide range of partners from several sectors, including key ministries such as human health, animal health, agriculture, environment and the private sector.

INTRODUCTION

Antimicrobials play a crucial role in reducing morbidity and mortality from communicable diseases worldwide. However, the emergence and spread of resistance to many of these antimicrobials is neutralizing their effectiveness. AMR threatens the effectiveness of previously used treatments for infections and presents a public health problem with local, national and global dimensions.

In May 2014, the Sixty-seventh World Health Assembly (WHA) expressed growing concern about AMR and urged governments to strengthen national action and international collaboration. Through its resolution WHA67.25, the World Health Organization (WHO) recommended a draft global action plan to combat AMR.

Mali is committed to the fight against AMR through several actions. A national expert was appointed by the Ministry of Health to draft a national action plan for the fight against AMR in 2014 (Letter N°1868 MSHP-SG of 20 August 2014). A guide to establish laboratory surveillance of AMR in Mali through a national AMR surveillance network was developed with the support of WHO in 2014. To this guide was annexed a national action plan 2014-2016.

In 2017, Mali developed a national plan inspired by the global action plan on antimicrobial resistance with six (6) strategic objectives, the fourth of which focuses on optimizing the rational use of antimicrobial drugs in human, animal and environmental health.

The country is not immune to the emergence of AMR. National data on AMR are not available at present. However, ad hoc data collected over the years show the extent of the problem

A study entitled "Antibiotic resistance of Salmonella gallinarum strains isolated from modern poultry farms in peri-urban areas in Mali" conducted by Dr Satigui SIDIBE et al of LCV in 2014-2015 in the animal sector during the period August 2014-December 2015, tested the antibiotic resistance of 52 Salmonella gallinarum isolates obtained from modern poultry farms located in peri-urban areas of the District of Bamako (27 isolates) and the cities of Ségou (16 isolates) and Sikasso (09 isolates).

The average resistance rates obtained were 98.08% to erythromycin, 94.23% to colistin, 90.38% to streptomycin, 67.31% to kanamycin, 65.38% to flumequine, 63.46% to doxycycline, 59.61% to tetracycline and 21.15% to gentamycin. All Salmonella isolates from poultry farms in Bamako District were found to be resistant to tetracycline, doxycycline and erythromycin. Similarly, resistance to erythromycin, tetracycline, colistin and streptomycin was found for all isolates from poultry farms in Sikasso. Thus, this study established the development of resistance of most of the Salmonella strains isolated to most of the usual antibiotics and, to a lesser extent, to gentamycin. These results could be due to certain insufficiencies or constraints observed in the field of animal health.

In Mali, the livestock sector faces many constraints, including those of a sanitary nature. These are generally linked to insufficient sanitary surveillance, the absence of a reliable supply circuit for veterinary drugs and the reluctance of farmers to use these products (problem of cost and receptiveness of farmers). Disease control requires good medical coverage. This medical coverage requires rational distribution and proper use of veterinary drugs, in particular by qualified personnel.

The wave of liberalisation that occurred in the livestock sector in the 1990s had as its main consequence, in the veterinary medicine sector, the multiplication of operators, the diversification of the origin of products and, in general, a decrease in their quality. This has resulted in the circulation of poor quality, poorly manufactured or counterfeit veterinary medicines, which constitute a permanent threat to animal health but also to public health.

The issue of AMR has motivated a global commitment to combat this scourge, and the health security program has adopted the "One Health" approach to achieve better results.
For example, the Joint External Evaluation (JEE) conducted in 2017 found that Mali had a capacity level of 1 (lowest capacity: absent or weak policies and regulations on appropriate use, availability and quality of antimicrobials) with respect to WHO Benchmark **3.4: Optimization of antimicrobial drug use in human and animal health**. The 2007 PVS (Performance of Veterinary Services) evaluation of the Veterinary Services of Mali and the 2017 follow-up PVS mission noted as a critical point, for public health, certain practices in the sale and administration of veterinary medicine (illicit sale, misuse, sale of medicines without MA, etc.).

To move from capacity level 1 to capacity level 2, at least five countries supported by the **Medicines Technology and Pharmaceutical Services (MTaPS) Global Health Security Agenda (**GHSA) program, including Mali, have committed to undertake an action identified in capacity level 2 of WHO reference 3.4 - namely, "Undertake an assessment of stewardship policies and activities, including the regulatory framework and stewardship of the antimicrobial supply chain, using a multi-sectoral approach.

The fight against AMR necessarily involves cleaning up the veterinary drugs (VD) sector. This issue is part of the present evaluation.

OBJECTIVES

GENERAL OBJECTIVE

The overall objective was to conduct a rapid assessment of antimicrobial policy, regulation and supply chain management in animal health.

SPECIFIC OBJECTIVES

Specifically, this will involve :

1. 1. Conduct a situational analysis of antimicrobial use in animal health ;
2. 2. Conduct a situational analysis of the regulation and control of antimicrobial use in animal health;
3. 3. Identify key actors and institutions involved in antimicrobial use in animal health, their roles and responsibilities; and incentives for behaviour change;
4. 4. Propose lines of intervention and follow-up mechanisms to address the main problems identified;

5. 5. Develop the draft Animal Health Antimicrobial Stewardship Action Plan.

METHODOLOGY

The implementation of this rapid assessment mission was entrusted to a consultant who worked in close collaboration with the country's Veterinary Services.

WORKTOP:

In the framework of the work, the plan was as follows: a documentary research phase which allowed the collection and analysis of different documents, a field survey phase in order to communicate with public and private actors likely to be involved in the field of veterinary medicines in Mali, a restitution and validation phase.

In accordance with the Rapid Situation Analysis for Rational Antimicrobial Stewardship Policy Tool, the report was developed in two stages:

- The first is a literature review from different data sources that addresses the international, sub-regional and national approach to veterinary drug legislation and regulation and antimicrobial supply chain management in animal health;

- The second step was to revise the draft report based on the results of the stakeholder interviews.

Validation of the draft report by a group of animal health experts with the involvement of the human health sector.

A final report was produced after taking into account the observations of the sponsors and those made during the validation workshop held on 27 April 2020 in the meeting room of the Inspectorate of Livestock and Fisheries.

An end-of-mission report summarizing the mission's progress, results achieved, opportunities and challenges, and recommendations for next steps will accompany this technical report.

A USAID-funded rapid assessment of the regulatory system and antimicrobial supply chain management in the field of Animal Health in Mali was conducted from March 16 to May 4, 2020 with assistance from the MTaPS program.

METHODS

The survey methodology was based on documentary research, interviews with resource persons and stakeholders in the veterinary medicine sector.
The literature review of legislative and regulatory texts and the management of the antimicrobial supply chain in the field of animal health was done through the internet (WHO and OIE etc.), the documentation of the Direction Nationale des Services Vétérinaires and the Inspection of the Ministry of Livestock and Fisheries.

MATERIALS

The Management Sciences for Health (MSH) Rapid Situation Assessment Tool for Rational Antimicrobial Stewardship Policies was used to collect the data. This tool is structured around five key areas: national antimicrobial legislation, regulations or policies, Standard Therapeutic Protocols (STPs), Essential Medicines List (EML), supply management policy, and appropriate use of antimicrobials by prescribers, distributors and farmers. Thus, the specific objectives of this rapid assessment were achieved through an analysis of data from these five (05) key areas.

To improve the accuracy of the data collected, an interview guide for resource persons and a questionnaire for stakeholders in the veterinary drug industry were developed and administered.

Several services of the ministerial departments of Livestock and Fisheries, Health, Agriculture and Environment in the district of Bamako were visited for interviews: DNSV, DNPIA, DPM, ANSSA, LNS, LCV, representatives (Council of the Order, associations of commissioned veterinarians, wholesalers, private veterinarians and breeders).

INTERNATIONAL APPROACH TO VETERINARY MEDICINES

At the international level, organisations and structures deal with veterinary medicines by developing standards, regulations and guidelines. Among these organisations, working mostly in collaboration, are the following:

- The World Organisation for Animal Health (OIE);
- World Health Organization (WHO);
- Food and Agriculture Organization of the United Nations (FAO);
- Codex Alimentarius Commission (CAC);
- International Cooperation on Harmonization of Technical Requirements for Registration of Veterinary Medical Products (VICH);
- The International Federation for Animal Health (IFAH).

THE IOE

It has developed a Veterinary Services Performance Evaluation Tool, a mechanism to evaluate veterinary services at the national level, to contribute to the planning of these services and to support them based on international standards established by the Organization. In May 2018, the latest version of the tool was completed with a new core competency in Part II-9, dedicated to AMR and the use that is made of these products; The OIE, has been actively working on the topic of veterinary products (including antimicrobial agents) for more than a decade and has developed a coherent strategy in this area.

FAO

It is currently piloting the Progressive Management Tool, which allows countries and sectors to determine where and how to progressively improve their antimicrobial use.

THE WHO

It assists national regulatory bodies in combating AMR by: (a) strengthening their capacity to oversee the development, evaluation, marketing and surveillance of medical products; (b) expediting the registration of prequalified medical products and ensuring that these products are accessible; and (c) strengthening their capacity to prevent, detect and respond to the threat of adulterated or substandard antimicrobial products.

VICH

Initially established under the auspices of the OIE, VICH was officially launched in April 1996 at the first meeting of its Steering Committee. VICH is a trilateral programme (European Union, Japan, United States of America) responsible for harmonising the technical requirements for the registration of veterinary drugs.
The main objective of VICH is to establish and implement harmonised regulatory requirements for veterinary medicinal products.

THE INTERNATIONAL FEDERATION FOR ANIMAL HEALTH (IFAH)

IFAH's mission is to foster a better understanding of animal health issues and to promote a science-based regulatory environment.

THE CODEX ALIMENTARIUS COMMISSION

The Codex Alimentarius Commission (CAC) was established in 1963 by FAO and WHO to develop food standards, guidelines and other texts, such as codes of practice, under the Joint FAO/WHO Food Standards Programme. The main purposes of this programme are the protection of consumer health, the promotion of fair practices in the food trade and the coordination of all food-related standardization work undertaken by both governmental and non-governmental organizations.

LEGISLATION, REGISTRATION AND CONTROL PROCEDURES FOR VETERINARY DRUGS IN THE WAEMU

The WAEMU, which includes eight (8) of the sixteen (16) West African countries, has developed and adopted several community texts on the harmonization of veterinary pharmaceutical legislation in a participatory manner. This approach is one of the consequences of the adoption of common agricultural policies.

The community texts drawn up and adopted by WAEMU are the following:

- Regulation N° 01/2006/CM/UEMOA of 23 March 2006 on the creation and functioning of the Veterinary Committee within WAEMU. This committee, composed of the Directors of Veterinary Services of member countries, has as its mission, among others, the examination and monitoring of the application of the texts in their respective countries;

- Regulation N° 02/2006/CM/UEMOA of 23 March 2006 as amended, establishing community procedures for the marketing authorisation and supervision of veterinary medicinal products and instituting a Regional Veterinary Medicines Committee (CRMV). The CRMV is composed of 10 experts and is responsible for: o examining MA dossiers; o preparing proposals for granting, refusing, suspending and withdrawing MA;

- Regulation N° 03/2006/CM/UEMOA of 23 March 2006 instituting fees in the field of veterinary drugs within the UEMOA ;

- Regulation No. 04/2006/CM/WAEMU of 23 March 2006 establishing a network of laboratories responsible for quality control of veterinary drugs in the WAEMU zone. This networking will allow to have a unified quality control system and to provide Member States with technical support in the field of quality control of pharmaceutical products and vaccines against animal diseases;

- Regulation N°04/2009/CM/UEMOA relating to the harmonization of rules governing the registration, marketing and control of pesticides within the UEMOA.

- Regulation N°06/10/CM/UEMOA of 1 October 2010 on the registration procedures for pharmaceutical products for human use in WAEMU Member States.

- Regulation n°08/2010/CM/UEMOA of 1st October 2010 amending article 49 of Regulation n°02/2006/CM/UEMOA of 23 March 2006 laying down Community procedures for the marketing authorisation and supervision of veterinary medicinal products and establishing a Regional Veterinary Medicines Committee;

- Directive N° 07/2006/CM/UEMOA relating to veterinary pharmacy. This Directive details the application in the field of Regulation No. 02/2006/CM/WAEMU. It describes the minimum essential rules to be applied by Member States for the production, importation, quality control and operation of veterinary pharmaceutical establishments, marketing and distribution of veterinary drugs in the WAEMU territory.

- Decision N°008/2008/COM/UEMOA on the creation of a revenue office for fees in the field of veterinary drugs within the UEMOA Commission;

- Decision N°009/2009/COM/UEMOA establishing the modalities for the submission of an application for a community marketing authorization ;

- Decision N°010/2009/COM/UEMOA on the designation of laboratories of the network in charge of quality control of veterinary drugs within UEMOA ;

- Decision N°011/2009/COM/UEMOA establishing the list of minor and major changes to marketing authorisation dossiers

In 2010, the provisions of Article 49 of Regulation N° 02/2006/CM/UEMOA were amended by Regulation N°08/2010/CM/UEMOA. Two years later, Directive N°01/2012/CM/UEMOA on the free movement and establishment of veterinary doctors who are nationals of UEMOA states was adopted in 2012. The Council of Ministers held on Wednesday, April 8, 2020 adopted the transposition of this Directive in the texts of Mali.

Member States, including Mali, must take the necessary legislative, regulatory and administrative measures for the implementation of these community texts, but this is not always the case. However, in Mali, in addition to the above-mentioned directive, Directive No. 07/2006/CM/WAEMU on veterinary pharmacy has also been transposed into the regulatory framework. It should be noted that there is no text concerning the stewardship of antimicrobials.

MARKETING AUTHORISATION AND REGISTRATION OF VETERINARY MEDICINAL PRODUCTS

The centralized system of WAEMU allows for the efficient issuance of a regional MA, which now replaces national MAs and is accompanied by an import authorization issued by one of the States (see Regulation n°02/2006/CM/WAEMU of 23 March 2006). This ensures the free movement of veterinary drugs on the territory of the Union, with exemption from customs duties and VAT. The MA application file is subject to a scientific evaluation carried out by the CRMV, involving the opinion of the Veterinary Committee and the possible intervention of experts from the List of Experts on Veterinary Medicines (LEMV) created within the WAEMU.

Once granted, the MA is valid for 05 years. Based on the list of community MAs for the year 2019 in WAEMU, 314 medicines have MAs from 2013 to 2019. However, although the modification of article 49 of the Regulation N° 02/2006/CM/UEMOA to obtain the Regulation n°08/2010/CM/UEMOA extending the transitional period which was fixed at two (2) years in 2006 by one additional year in 2010 to allow the pharmaceutical firms to comply with the new regulations, it has been noted that many products circulate without MA in Mali Also on the WAEMU list of community MAs, no MA was found for locally produced veterinary medicines.

From 2010 to date: with 38 sessions held, 626 files have been registered, 280 approved, 1 rejected and 7 abandoned (UEMOA).

The MA application file includes an administrative file and a technical file.

Administrative file

Paragraph 2 of Article 25 of Regulation No. 02/2006/CM/WAEMU of 23 March 2006, as amended, states that any application for marketing authorisation for a veterinary medicinal product shall include the following information

- The name of the medicinal product, its composition, pharmaceutical form, packaging, method and route of administration, indications, contra-indications and adverse reactions, expiry date. The species of animal to be treated, the dosage and the withdrawal period, if any, for each species shall be specified;
- The name or corporate name and address of the applicant and, where appropriate, that of the local representative ;
- The name and address of the manufacturer(s);
- The designation of the manufacturing sites, including packaging and quality control;
- Where appropriate, the list of third countries which have granted marketing authorisation for the medicinal product or in which an application is under examination ;

- The number and title of volumes of documentation submitted in support of the application.

In addition to this information, the applicant for marketing authorisation must add, as indicated in paragraph 3 of the same article, the following documents

- copies of the administrative authorisation decisions for manufacturing establishments issued to the manufacturer of the medicinal product concerned in accordance with the national legislation of the establishment and, where appropriate, a document showing that the manufacturer is inspected by the competent authorities and operates in accordance with the principles of Good Manufacturing Practice (GMP) in force at international level ;

- a copy of any marketing authorisation obtained for this veterinary medicinal product in a third country, together with a copy of the package leaflet proposed in that country, details of any decision to refuse authorisation and the reasons for that decision.

Technical file

The technical dossier, as described in paragraph 3 of Article 25 of Regulation No. 02/2006/CM/WAEMU as amended, shall include the following information and documents, presented in accordance with the analytical, safety, pre-clinical and clinical standards and protocols for the testing of veterinary medicinal products:

- description of the manufacturing method, the control methods used by the manufacturer (qualitative and quantitative analyses of components, intermediate products and finished product, specific tests for sterility, stability, toxicity, heavy metals, pyrogenic substances) and their results;

- routine methods of analysis which may be used by the competent authorities for the detection of residues ;

- where appropriate, explanations of the precautionary and safety measures to be taken when storing the medicinal product, administering it to animals and disposing of waste, as well as an indication of the potential risks which the medicinal product might pose to the environment, human and animal health and to plants ;

- expert reports on these documents and tests, samples of the sales model of the veterinary medicinal product and the package leaflet.

VETERINARY DRUG CONTROL PROCEDURES

In terms of quality control of veterinary drugs, the analysis of the situation within WAEMU has shown the almost generalized absence of organized quality control. Countries with control structures are faced with problems of insufficient financial and human resources, as well as adequate technical and scientific equipment to implement quality control programmes.

This has led WAEMU to include procedures for the control of veterinary drugs in the new community texts.

Article 47 of Regulation N° 02/2006/CM/UEMOA provides for the official control by a laboratory of the regional network of veterinary medicines quality control laboratories or by another approved laboratory, of samples of a batch of medicines on the occasion of the first import after the MA.

FINDINGS

These findings are the results of the literature review and interviews with structures and actors involved in the management of veterinary drugs in Mali.

NATIONAL LEGISLATION, REGULATIONS OR POLICIES

As a result of colonization (1895-1960), Mali was subject to a large and scattered body of legislation and regulations relating to the pharmacy sector. These include, among others, the Law of 21 April 1803, Year XI; the contents of Book V of the Public Health Code relating to the practice of pharmacy, some of whose provisions were extended to the overseas territories, Togo and Cameroon in 1953; the 1955 Order on the distribution of pharmacies in the French West Indies; and the 1960 Law establishing the Order of Pharmacists in the Federation of Mali. After the accession to international sovereignty, the new Malian State, while continuing the pharmaceutical legislation of the former guardian State which was not contrary to the new fundamental law, also introduced new texts in accordance with the political orientations of the time. As part of this evaluation, a list was made of all the legislative and regulatory texts that have been adopted in Mali in the veterinary pharmaceutical sector.

The World Organisation for Animal Health (OIE) has so far carried out an evaluation of the quality of national animal health systems, including Veterinary Services, in over 130 countries. More than 110 countries evaluated, mostly developing and emerging countries, do not yet have relevant legislation on appropriate conditions for the importation, manufacture, distribution and use of veterinary products, including antimicrobial agents. Sometimes legislation is totally absent. Where it exists, as in Mali, it is often not enforced due to lack of government capacity to carry out controls.

In Mali, antimicrobial agents are most often directly or indirectly accessible to all without constraint.

Veterinary drugs have a direct influence on the health of animals and on the sanitary quality of the products derived from them because of the significant risk posed by their residues.
Because they present a danger and are a tool of veterinary public health, veterinary drugs cannot be considered as mere commercial products but as health products. The State, which is responsible for safety, is therefore justified in regulating them.

In addition to guaranteeing safety, medicinal products are an instrument of economic support for professionals. From the moment that an efficient network of veterinary professionals is a sine qua non for the conduct of a veterinary public health policy, the public policy motivation can justify market regulation independently of competition rules.
According to Law No. 2016-004 of 12 February 2016 ensuring the transposition of Directive No. 07/2006/CM/UEMOA, which governs veterinary pharmacy in Mali, veterinary drugs require complete control of the chain (materials and processes) from manufacture to use essentially based on a principle of *a priori* control, i.e., on the prior authorization of the various activities in relation to pre-established reference systems.
Also, for use, the principle adopted is to reserve possession and prescription to qualified professionals subject to specific rules and controls.

Legislation plays a key role in AMR prevention. It is essential to sustain regulatory objectives, clarify roles and responsibilities, create concrete obligations for different stakeholders and make them enforceable. In Mali, there is already legislation in place in relevant areas that potentially allows for the required regulatory controls, but it is the implementation of this legislation on the ground that is problematic.

There is no surveillance system for antimicrobial sales in the country. Mali adopted Resolution N°26 which requested member countries to establish surveillance systems for antimicrobials but DNSV could not complete the questionnaire developed for this purpose due to lack of data.

MARKETING AUTHORIZATION (MA)

Article 6 of Law N°2016-004 of 12 February governing veterinary pharmacy in Mali stipulates that, with the exception of medicated feeds, no veterinary medicine can be delivered to the public unless it has first received a MA issued by the WAEMU Commission under the conditions mentioned in Regulation N°02/2006/CM/UEMOA of 23 March. The MA application file is subject to a scientific evaluation carried out by the CRMV, involving the opinion of the Veterinary Committee and the possible intervention of experts from the LEMV created within WAEMU.

The vast majority of products on sale in Mali have no MA, either national or community. Only one manufacturing company has been listed in Mali. However, the products of this company do not have a MA. It has several branches in different regions of Mali. It is true that the MA process is centralized within the WAEMU, but it would be important to involve the veterinary services more

IMPORTATION

Article 47 of Regulation N° 02/2006/CM/UEMOA provides for official control by a laboratory of the regional network of veterinary medicine quality control laboratories or by another approved laboratory, of samples of a batch of medicine on the occasion of the first import after the MA.

Article 7 of Law N°2016-004 of 12 February 2016 governing veterinary pharmacy subjects the importation from third countries of any veterinary medicine to prior authorization by the DNSV. Indeed, for import licenses in the veterinary field the invoices are subject to DNSV approval by the National Directorate of Trade and Competition (DNCC). However, information on the import of veterinary drugs is not often available and analyzed for decision-making.

All imported drugs are exempt from customs duties except for the Common External Tariff (CET), which consists of community levies of 2.5% on drugs imported from outside the WAEMU and ECOWAS zones, applied since 1 January 2000 for the benefit of these two institutions.

In 2016, SIPROVET is still by far the leading wholesale importer with market shares representing 57.12% of the relative value of veterinary drugs and vaccines imports in Mali.

It is followed respectively by PHARMAVET Bamako, MALIVET, MEDIVET, CANALVET, PROVETO, SAHEL VETO, with respective shares of 24.07%, 8.81%, 3.32%, 1.93%, 1.37%, 1.33%, 0.72%, 0.43%, 0.37%, 0.25%, 0.11% and 0.09%.

It should also be noted that companies such as SEDIMA, JAI LAXMI and AVIPREST CONSULTING International import poultry vaccines for their own companies.
The therapeutic families (anti-infectives, vaccines and serums) remained in the same order as last year. However, internal antiparasitics lost their third place ranking to external antiparasitics.

Anti-infectives continue to rank first with an absolute value of CFAF 778282082.6.

Compared to 2014, the anti-infectives and vaccines and serums families retain the first and second positions with decreases of 5.99% and 5.19% respectively in relative value. (Source: DNSV 2016 Annual Report)

GRANTING OF A LICENCE TO OPERATE

For Article 9 of Law No. 2016-004 of 12 February 2016 governing veterinary pharmacy, the opening of any establishment for the preparation, import, export and wholesale distribution of veterinary drugs must be subject to the granting of an operating license issued by regulation. Operating licences for wholesale importers are granted by the Ministry in charge of animal health. However, the implementing regulations for this law have not yet been drawn up, which is an obstacle to its application.
In practice, we observe sales of veterinary drugs by unauthorized actors (traders and livestock merchants) at fairs and markets.

DISTRIBUTION

Wholesale distribution

Regarding Article 10 of Law No. 2016-004 of 12 February 2016 governing veterinary pharmacy, it is mentioned that any establishment for the preparation, import, export and wholesale distribution of veterinary drugs must be owned by a veterinary doctor, a pharmacist or a company in whose management or general direction a veterinary doctor or pharmacist participates.

Retail distribution

Article 24 of Directive N°07/2006/CM/UEMOA relating to veterinary pharmacy specifies that the holding of veterinary drugs for retail distribution and their transfer free of charge or for consideration to users should be reserved for authorised persons and under the conditions defined by regulation, namely veterinary doctors holding a veterinary dispensary, doctors of pharmacy holding a pharmaceutical dispensary, and teachers at veterinary schools for the treatment of animals admitted for consultation or hospitalisation.

By way of derogation, veterinary surgeons registered with the Order to work for groups of farmers subject to an approval procedure in respect of veterinary medicinal products for current use and for the exclusive benefit of their members, and State veterinary officers in respect of medicinal products necessary for the implementation of compulsory prophylaxis directed by them and for other medicinal products insofar as no private practitioner is practising in the area.

In practice, retail distribution of veterinary drugs is carried out by veterinary clinicians, pharmacists, veterinary auxiliaries, sometimes farmers' groups, unqualified individuals and importing wholesalers in livestock or weekly markets.

Although there are about 200 veterinary practices/clinics and warehouses in Mali that have been authorized by the Ministry of Livestock and Fisheries (MEP), the distribution of medicines is poorly controlled. Thus, it is done in the greatest disorder, without any real control and without knowledge of all the technical rules in the matter. This observation was also made in previous evaluations such as the PVS.

Retailers should only supply veterinary antimicrobial drugs on the prescription of a veterinarian or other person duly authorized in accordance with legislation and all products should be properly labelled. Retailers should encourage compliance with national guidelines on the responsible use of veterinary antimicrobial drugs and should keep detailed records of all antimicrobials supplied.

Procedures for the distribution of veterinary drugs in Mali

Since the privatization of the veterinary profession, the distribution of veterinary drugs is ensured by private actors.

The veterinary drugs market is driven by legal players and illegal players share this market. The legal players are composed of importers, wholesalers, distributors and retailers who are veterinarians or pharmacists. There are also professional breeders' organisations. The illegal players are made up of traders and veterinary para-professionals. The latter are most often unqualified or poorly qualified and driven solely by the lure of profit.

- **The official market for veterinary medicines**

  The official market is the distribution channel for veterinary drugs authorized by the State. The main players are importing wholesalers, distributing wholesalers and retailers. They are obliged to follow the various provisions imposed by the regulations in force.

- The illicit market in veterinary drugs

  The size of the illicit market for veterinary drugs is not estimated. However, according to information gathered from private individuals, it is estimated that this market represents more than 40% of the total market for veterinary drugs.

Most of the players in the illicit market for veterinary drugs are traders. They are grouped in a well-structured distribution chain from wholesaler to retailer.

It has been observed on the parallel market of products of Indian and especially Chinese origin.
It should be noted that the officially installed private structures also supply the illicit market of these products with veterinary drugs.

CONTROL AND INSPECTION

Article 13 of Law N°2016-004 of 12 February 2016 governing veterinary pharmacy stipulates that the control and inspection of veterinary pharmaceutical establishments are carried out by sworn agents of the veterinary drug control services of the Ministry in charge of Livestock without prejudice to the control by other competent administrations. Control and inspection missions are carried out, but not sufficiently in relation to the ambitions. Mali has participated in several editions of the "veterinary drug sanitation campaigns in the WAEMU region".
On the other hand, at the initiative of the WAEMU, the DNSV conducted inspection visits to several establishments selling veterinary products in 2015, 2016 and 2017 (with the help of the control forces, paid thanks to WAEMU funding).

ADVERTISING CONTROL

Advertising of antimicrobial agents is in line with the principles of responsible and prudent use and is governed by Decree N°08- 3735/ MS- MEP / SG of 31 December 2008 establishing the conditions for advertising of medicines. Such advertising of antimicrobial veterinary drugs should be carried out in a manner consistent with prudent use guidelines and any other product specific regulatory recommendations. All advertising of veterinary drugs including antimicrobials should be controlled by the relevant authorities.

According to the above-mentioned decree, the authorities should verify whether advertising for veterinary drugs, including antimicrobials: is in accordance with the marketing authorization granted, especially with regard to the content of the summary of product characteristics; and is in accordance with the national legislation of the country.
The veterinary pharmaceutical industry is responsible for advertising antimicrobial veterinary medicinal products in accordance with the provisions of paragraph 35 on advertising control and for not advertising antimicrobials inappropriately directly to food animal producers.

PHARMACOVIGILANCE

For Article 29 of Law N°2016-004 of 12 February 2016 governing veterinary pharmacy, veterinary doctors and other health professionals are required to report to the DNSV any adverse reaction occurring in humans or animals that may be attributed to a veterinary drug. This information is transmitted without delay to the WAEMU Commission and the National Pharmacovigilance Commission (CNPV).

In reality, there is no pharmacovigilance data in the field of animal health. It is true that the Central Veterinary Laboratory (LCV) is a member of the national pharmacovigilance commission, but adverse

effects should be reported to the DNSV through its representatives in the field, who do not have a notification form and do not know how to fill it in. It would be important to integrate the DNSV into this national commission or to create a framework for formal collaboration between the DNSV and the LCV and to consider training DNSV field agents on pharmacovigilance, including the filling out of report forms, which should also be made available to them.

STANDARDISED THERAPEUTIC PROTOCOLS (STP)

The use of PTNs is not yet widely practiced in veterinary medicine in Mali. In practice, waiting times are not respected and animal identification is not used in most of our farms. In terms of control of diseases considered legally contagious (MRLC), we give priority to sanitary measures, administrative measures and mass prophylaxis for diseases that require vaccination. These diseases are: Contagious Bovine Pneumonia (CBPP), Rabies, Pasteurellosis (bovine, ovine, caprine), Peste des Petits Ruminants (PPR), Avian Influenza and Newcastle Disease (ND). There are specific orders to control priority diseases such as

- Order N°02/0982/MDR-SG laying down the practical measures to be taken in the fight against bovine tuberculosis;
- Order N°02/0984/MDR-SG laying down practical measures to be taken for the control of foot and mouth disease;
- Order N°97/0150/MDRE-SG laying down provisions relating to brucellosis, Order N°05/2703/MEP-SG laying down practical provisions to be taken in the fight against avian influenza (bird flu).

ESSENTIAL DRUG LIST

There is a regulation in Mali namely Decree No. 66/PG-RM of 11 March 1985 which sets the nomenclature of essential medicines for the health protection of livestock. This provision was amended in 2015.

There are no universities that train veterinarians in Mali. Most veterinary para-professionals (engineers, technicians and technical agents) have been trained as Engineers of Applied Sciences specialising in animal husbandry (ISA), Senior Animal Husbandry Technicians (TSE), Animal Husbandry Technicians (TE) or Animal Husbandry Technical Agents (ATE) in specialised educational institutions in Mali. They are mainly responsible for field activities in the DRSVs, veterinary sectors and posts, as well as in veterinary clinics and practices. The TSEs are currently trained at the IPR of Katibougou in 2 years (Baccalauréat + 2). TEs and ATEs trained at the Centre de Formation Pratique en Élevage (CFPE) in Sotuba are respectively 4 years (Diplôme d'Etude Fondamentale-DEF+4) and 2 years (DEF+2). There is also currently an agropastoral university in Ségou that trains animal health technicians and that has been approved by the competent national authorities to train veterinarians. According to the teaching staff of this university, their curricula do not currently include the LME concept.
In addition, there is no data on the availability of EMLs in veterinary clinics and practices, nor is there any survey data on listed drugs in these facilities.

GOOD PROCUREMENT MANAGEMENT POLICY

In terms of quality control of veterinary drugs, the analysis of the situation within WAEMU has shown the almost generalized absence of organized quality control. Countries with control structures are faced with problems of insufficient financial and human resources, quality and adequate technical and scientific equipment to implement control programmes.

This has led WAEMU to include procedures for the control of veterinary drugs in the new community texts.
Article 47 of Regulation N° 02/2006/CM/UEMOA provides for official control by a laboratory of the regional network of veterinary medicine quality control laboratories or by another approved laboratory, of samples of a batch of medicine on the occasion of the first import after the MA.

Article 48 of the same Regulation provides that the WAEMU Commission may mandate one or more inspectors from Member States to carry out inspections deemed necessary on or outside the territory of the Union.

As mentioned in point 5 of the chapter on "National Legislation, Regulations or Policies", the control and inspection of veterinary pharmaceutical establishments are carried out by sworn agents of the veterinary drug control services of the Ministry of Livestock without prejudice to control by other competent administrations. These agents are empowered to :

- To carry out inspections in establishments preparing, importing, exporting and selling veterinary drugs;
- Collecting samples ;
- To have access to all documents concerning their field of expertise.

These checks are carried out by the DNSV and the Livestock and Fisheries Inspectorate.
Within the framework of the Veterinary Medicines Market Sanitation Campaign (CAMMVET 2017-2018), controls were carried out by the 2 structures mentioned above. The pharmacies visited had very few medicines with MAs. Seizures were made in pharmaceutical warehouses as well as in fairs and markets for fraud, expiry, poor conservation and counterfeiting, This situation mainly concerned antibiotics, internal and external dewormers and vaccines.

The importation of veterinary drugs in Mali has been reserved exclusively for the private sector since the 1990s, which coincided with the privatisation of the veterinary profession. However, in practice, it is also done through certain traders.

APPROPRIATE USE BY PRESCRIBERS, DISTRIBUTORS AND BREEDERS PRESCRIPTION

Article 25 paragraph 1 of Directive N°07/2006/CM/UEMOA relating to veterinary pharmacy specifies that Member States shall determine prescription and labelling rules for the retail distribution of veterinary drugs according to the following categories

- Veterinary medicinal products containing one or more active ingredients which may be toxic to the animal or dangerous to the user of the medicinal product or the consumer of products of animal origin through harmful residues;

- Veterinary medicinal products which are not toxic to the animal, dangerous to the user of the medicinal product or to the consumer of products of animal origin through harmful residues.

Article 25, paragraph 2, stipulates that the retail supply, whether free of charge or against payment, of the veterinary medicinal products referred to in point 1 above shall be subject to a prescription from a veterinary surgeon, which shall be given to the user. These medicinal products may not be supplied as such to approved breeders or groups of breeders, or held or possessed by such breeders or groups, unless they are authorised and intended for agricultural or phytosanitary use.
Article 26 of Directive N°07/2006/CM/UEMOA relating to veterinary pharmacy states that Member States shall take legal measures to ensure that: any person authorised to supply retail products shall keep detailed documentation on each entry or exit of prescription drugs:

1. the date of the operation ;
2. Identification of the drug (name, pharmaceutical form, strength, target species);
3. the production lot number ;
4. the quantity received or issued ;
5. the name and address of the supplier or recipient ;
6. the name and address of the prescriber.

This information shall be kept available to the competent authorities and their inspection bodies.

For article 27 of Directive N°07/2006/CM/UEMOA relating to veterinary pharmacy, Member States shall take all legal measures to allow the prescription of appropriate veterinary drugs within the framework of article 20 of Regulation N°02/2006/CM/UEMOA laying down Community procedures for the marketing authorisation and supervision of veterinary medicinal products and establishing a Regional Committee for Veterinary Medicinal Products

Article 28 of Directive N°07/2006/CM/UEMOA relating to veterinary pharmacy stipulates that Member States shall encourage owners of food-producing animals to register the acquisition and administration of veterinary drugs subject to prescription.

Unfortunately, these provisions are rarely applied to improve the control of prescriptions, particularly article 25, paragraph 2, mentioned above, since most veterinary doctors are occupied with administrative tasks in the public sector and with managerial and commercial functions in the private sector. In practice, it is usually veterinary para-professionals (ISA, TE, ATE) who treat animals, very often without the supervision of the veterinarian. These engineers, technicians and nurses are not authorized to prescribe certain categories of veterinary drugs that could have very harmful effects on the animal or man. As a result, many veterinary drugs are sold and administered without a prescription.

DNSV and UEMOA have conducted campaigns in the fight against the illicit sale of medicines at least once a year through advertising campaigns on ORTM television and radio stations, since 2015.

Each year, the NDSU conducts information campaigns on infection prevention (hand hygiene, food hygiene, vector control, vaccinations, etc.) and on the appropriate use of antimicrobials/antibiotics, as

well as awareness campaigns on antimicrobial resistance issues. However, there has been no impact assessment of these campaigns.

Usage

In practice, the appropriate use of antimicrobial veterinary drugs is a clinical decision that should be based on the experience and local expertise of the prescribing veterinarian and on an accurate diagnosis based on appropriate procedures.

If the conditions of use on the label allow for flexibility, the veterinarian should consider a treatment regimen that is long enough to allow for effective healing of the animal, but short enough to limit the selection of resistance in foodborne and/or commensal microorganisms.

Off-label use of a veterinary medicinal product may be permitted where justified and should be in accordance with relevant national legislation, including relevant administrative withdrawal periods. It is the responsibility of the veterinarian to define the conditions for responsible use in this case with respect to the therapeutic regimen, route of administration and duration of treatment. Non-therapeutic use of antimicrobials should not be permitted.

In practice, products are used as a first line of defence, often against the grain, directly by farmers. All stakeholders agree that farmers use antimicrobials in the field, but as usual there is no data in the DNSV reports. All these products include antibiotics often used in human health. This situation was also confirmed by the experts at the validation workshop. Indeed, these experts revealed the use of antibiotics that are often critical for humans and the use of antibiotics in animal feed.

DNSV participated in the 2nd OIE Global Conference on Antibiotic Resistance and Prudent Use of Antibiotics in Marrakech, Morocco from 20-31 October 2018.

ACTORS AND INSTITUTIONS INVOLVED IN THE USE OF ANTIMICROBIALS IN ANIMAL HEALTH

THE WAEMU COMMISSION

The WAEMU Commission is responsible for issuing the marketing authorisation. The marketing authorisation is granted on the basis of data submitted by the pharmaceutical industry or the applicant, and only if the criteria of safety, quality and efficacy are met.

GOVERNMENT STRUCTURES

In Mali, the regulatory functions for veterinary drugs are performed by separate institutions, independently mandated by law to carry out the different responsibilities:

- The Direction Nationale des Services Vétérinaires (DNSV) is one of the directorates of the Ministry of Livestock and Fisheries (MEP). It is responsible for animal health; veterinary public

health (meat inspection; processing/distribution of products of animal origin and food safety); control of veterinary drugs and veterinary biological products;

- The Central Veterinary Laboratory (LCV) receives all kinds of animal samples and performs bacteriology, virology, autopsy, toxicology and food bacteriology tests (but is not accredited, unlike the LNS). It is in charge of the analysis of pesticide residues;
- The Livestock and Fisheries Inspectorate: according to the order N°09-011/P-RM of 4 March 2009 creating the inspectorate, the missions of the Livestock and Fisheries Inspectorate are to :
    - To control the operation and action of the services and bodies under the authority of the Department of Livestock and Fisheries;
    - To control the application of laws and regulations in the public and private practice of the veterinary profession;
- The National Health Laboratory (LNS) for the laboratory analysis of drug samples. Created by Order No. 00-40/PRM of 20 September 2000, the LNS has the mission of controlling the quality of medicines, food, beverages or any other substances imported or produced in the Republic of Mali and intended for therapeutic, dietetic or food purposes with a view to safeguarding the health of human and animal populations. This laboratory is accredited in food bacteriology (e.g. total coliforms, staphylococci, salmonella) and performs tests for the detection of drug residues, heavy metals;
- The National Institute of Public Health (INSA) for the implementation of technical activities related to pharmacovigilance;
- The National Animal Health Support Centre (CNASA) for the analysis of animal health risks.

THE VETERINARY PHARMACEUTICAL INDUSTRY WITH RESPECT TO VETERINARY DRUGS CONTAINING ANTIMICROBIAL AGENTS

There is only one local manufacturer (PHARMAVET-KONE). Veterinary drugs are therefore mostly imported from developed countries.

In Mali, the main manufacturers are European (MERIAL, CEVA Santé Animale, VETOQUINOL, LAPROVET, LOBS INTERNATIONAL, Boehringer Ingelheim Animal Health, Interchemie, GENEVET, VMD, and ALFASAN, SANOFI etc.). Apart from the manufacturers mentioned, suppliers of veterinary products are from Pakistan, Brazil, China and India.

However, the Central Veterinary Laboratory (CVL) is one of the main African laboratories supplying vaccines against Contagious Bovine Pneumonia (CBPP), symptomatic anthrax and bovine, ovine and caprine pasteurellosis.

According to the OIE, the veterinary pharmaceutical industry is responsible for: providing all information requested by the UEMOA Commission; ensuring the quality of this information in accordance with GMP, GLP and GCP; implementing a pharmacovigilance programme and, on request, specific surveillance to obtain data on bacterial susceptibility and resistance.

The veterinary pharmaceutical industry must comply with the principles of responsible and prudent use and established advertising codes, including: communicating information in accordance with the terms of the marketing authorization, not advertising veterinary drugs containing antimicrobial agents to food-producing animals. However, in practice, this last provision is not respected by the majority of representatives of pharmaceutical companies.

WHOLESALERS AND RETAILERS

Veterinary drugs are imported by wholesalers. Before the privatization of the veterinary profession in Mali, only the Veterinary Pharmacy, which is a state structure, played this role.

On the other hand, since the disengagement of the State from this sector of the economy, veterinary products are imported by private structures managed by veterinary doctors, livestock engineers and pharmacists. Projects and groups of approved breeders under the direction of a veterinary consultant can also import veterinary drugs.
The importation of veterinary drugs into Mali is carried out by 14 wholesale importers.

In Mali, distribution is reserved for wholesale distributors and retailers according to the provisions of the 2016 law. They codify the function of wholesale importers and distribution of veterinary drugs. However, this distribution must comply with the conditions mentioned in the penultimate paragraph of point 4.2 of the distribution. According to, the OIE, retailers must support the recommendations for the responsible and prudent use of veterinary drugs containing antimicrobial agents and keep a detailed record of the following:

- the date of issue ;
- the name of the prescriber ;
- the user's name ;
- the name of the product ;
- the batch number ;
- the expiry date ;
- the quantity supplied ;
- a copy of the prescription.

However, the lack of implementing orders has a huge impact on the application of this law.

VETERINARIANS

The promotion of public health and animal health and welfare is the responsibility of the veterinarian. This includes the prevention, identification and treatment of animal diseases. The promotion of

correct husbandry and hygiene procedures, biosecurity measures and rational vaccination strategies can contribute to limiting the use of antimicrobial agents in food-producing animals.
Veterinarians should only prescribe antimicrobial agents for animals under their care.
Veterinarians' responsibilities in the use of antimicrobial agents are to conduct an adequate clinical examination of the animal(s) and, upon completion of the examination, to :

- Administer or prescribe antimicrobial agents, only when necessary, with reference to the OIE list of important antimicrobial agents in veterinary practice;

- Careful selection of antimicrobial agents based on clinical experience and information from diagnostic laboratories where possible (isolation, identification and susceptibility testing);

- Provide a detailed treatment protocol, including precautions for use and withdrawal times, especially for off-label or off-license prescriptions.

However, the provision for a detailed treatment protocol is rarely followed by veterinarians in Mali. Professional veterinary organisations should participate in training programmes organised by the OIE

BREEDERS OF FOOD-PRODUCING ANIMALS

Food animal producers are responsible, with the advice and assistance of a veterinarian, for implementing animal health and welfare programmes on their farms to improve animal health and food safety. These farmers must :

- develop a health plan with the veterinarian in charge of the farm describing the main preventive measures (e.g. health plan for fattening units, mastitis prevention programme, endoparasite and ectoparasite control, vaccination programmes and biosecurity measures); use veterinary medicinal products containing antimicrobial agents only when prescribed by a veterinarian or other person properly trained and authorised to prescribe such veterinary medicinal products, in accordance with existing national legislation and under the supervision of a veterinarian; use veterinary medicinal products containing antimicrobial agents according to the instructions on the label, including storage conditions, or according to the instructions of the veterinarian in charge of the farm ;

- Isolate sick animals where necessary to prevent the transfer of pathogens and dispose of dead or dying animals promptly in accordance with the conditions approved by the authorities concerned; observe biosecurity measures on the farm and take all basic veterinary hygiene precautions ;

- Adhere to and document recommended withdrawal times to ensure that residue levels in food of animal origin do not pose a risk to the consumer;

- Use veterinary drugs containing antimicrobial agents before the expiry date and dispose of unused or expired products in an environmentally sound manner; retain all laboratory reports including bacteriological and susceptibility tests; these data should be made available to the veterinarian responsible for the treatment of the animals;

- Keep a proper record of veterinary drugs used containing antimicrobial agents, including the following information:

    - the name of the product and the active ingredient, the batch number and the expiry date;

- the name of the prescriber and the supplier ;
- the date of administration ;
- identification of the animal (or group of animals) to which the antimicrobial agent was administered ;
- diseases treated ;
- the doses administered ;
- Waiting times (including end dates) ;
- laboratory test results;
- treatment effectiveness;
- inform the veterinarian in charge of the cases of recurrence.

Food animal producers should participate in training programmes organised by the OIE.

FEED MANUFACTURERS

Feed manufacturers should only be permitted to supply medicated feed containing antimicrobial agents to food producing animals when prescribed by a veterinarian or other person properly trained and authorised to prescribe such veterinary drugs, in accordance with applicable national legislation and under the supervision of a veterinarian. Feed manufacturers preparing medicated feeds should follow the rules established by the Competent Authority in accordance with applicable national legislation. All medicated **feeds** and premixes must be properly labelled. **Feed** manufacturers should enforce regulations and recommendations for the responsible and prudent use of veterinary drugs containing antimicrobial agents and keep detailed records.

WORKING WITH TRIPARTITE ORGANIZATIONS TO ADDRESS ISSUES

Since the 1940s, the members of the Tripartite Organizations have worked together, with their collaboration formalized in 2010. In 2018, they signed a Memorandum of Understanding to reaffirm their commitment to achieving the five strategic objectives outlined in the Global Action Plan to Combat Antimicrobial Resistance. The Tripartite Organizations have developed a joint work plan for 2019-2020. As part of this, they have identified five areas of focus for multi-sectoral collaboration, namely: (a) implementation of national action plans; (b) awareness raising and behaviour change; (c) monitoring and surveillance of antimicrobial resistance and its use; (d) antimicrobial stewardship and optimal use; and (e) monitoring and evaluation. The Tripartite Organizations are working towards the establishment of a multi-partner trust fund to ensure regular and coordinated funding for five years. This fund will be managed by the Multipartner Trust Fund Office, which will allocate resources for the implementation of national action plans and the workplan.

STRATEGY FOR ACTION IN THE FIELD OF VETERINARY MEDICINAL PRODUCTS AND VETERINARY PRACTICE

CONCERNING THE FIELD OF VETERINARY MEDICINAL PRODUCTS :

This is a major issue for Mali. The drug market is totally disorganized with illicit practices at all levels. More than a lack of regulation, it is above all a lack of application at all levels: from the importation of medicines to their distribution and use. The main consequence is that the current competition between the various players deprives animal health professionals (in this case veterinary practitioners) of the drug margin to enable them to finance the coverage of the territory.
The key action is therefore the establishment of strict control of the veterinary drug market. This involves:

- Strengthening import controls on veterinary drugs to ensure that only duly authorized drugs enter the country. This involves stricter control of importers and greater vigilance at the borders with better coordination with the customs services;
- Tighter controls on distribution conditions to ensure that only those entitled to the drugs are distributing them.

CONCERNING THE VETERINARY PROFESSION :

The veterinary profession will be able to increase its margins on medicines and thus ensure a better presence among veterinarians. In order to strengthen the health mandate, it would be useful to reserve compulsory vaccinations (bacterial anthrax, symptomatic anthrax, Newcastle disease) for veterinary surgeons with a mandate. This operation is costless for the State, except for a communication plan to support farmers and practitioners.
As regards the statutory body of the veterinary profession, the Ordre des Vétérinaires is not very functional and no specific activities have been developed by it. This observation was also revealed by previous evaluations. For the proper functioning of this body, it is suggested to:

- To proceed with the elections and renewal of these institutions in accordance with its statutes. Failing this, the Ministry of Livestock may hold new elections;
- To respect its position as a Statutory Body ensuring, on the one hand, the quality and ethics of the services provided by veterinary practitioners, and on the other hand, a first level professional discipline;
- Create a division within the NDSV responsible for pharmacy and veterinary practice.

PROPOSALS FOR STRATEGIC DIRECTIONS AND FOLLOW-UP MECHANISMS TO ADDRESS THE MAIN ISSUES IDENTIFIED

MAIN PROBLEMS IDENTIFIED

- On the WAEMU list of community MAs, no MA has been found for locally produced veterinary drugs;
- Insufficient human resources and equipment for quality control of medicines ;

- Insufficient enforcement of regulatory texts due to the non-existence of an enforcement decree of the 2016 law governing veterinary pharmacy;
- No specific regulations on antimicrobial use
- There is no surveillance system for antimicrobial sales in the country;
- The vast majority of products on sale in Mali have no national or community MA;
- information on the import of veterinary drugs is not capitalized;
- there are no pharmacovigilance data in the field of animal health;
- The lack of a formal system in place for monitoring and collecting data on the use of antimicrobial agents in animals;
- The difficulty of estimating antimicrobial consumption ;
- No residue detection program in DAOA ;
- Insufficient skilled human resources for prescribing and dispensing antimicrobials in remote areas;
- Prescription made by unauthorized targets who are veterinary para-professionals (ISA, TE, ATE);
- Lack of impact assessment on these campaigns ;
- the products are used as a first line of defence and often against the grain directly by the farmers, but there is no data on this aspect in the various DNSV reports;
- Insufficient coordination of the different sectors using antibiotics ;
- Lack of available data on the impact of AMR awareness campaign measures.

Other factors contributing to the increase in antimicrobial resistance include:

- Illicit sale of medicines and counterfeit medicines ;
- Non-compliance with antimicrobial prescribing standards;
- Antimicrobial misuse;
- The use in animals of antibiotics of major importance in humans ;
- The use of antibiotics to promote growth in livestock ;
- Antibiotic use in food production ;

PROPOSALS FOR STRATEGIC DIRECTIONS

Multi-sectoral and One Health collaboration/coordination

In Mali, a Multisectoral Working Group on AMR has been set up under the authority of the public authorities. This multisectoral working group is operational and has a clearly defined mandate. A national action plan to combat AMR has been developed. The plan has been technically approved and is awaiting political validation. The plan is structured around six strategic objectives, of which 4ème focuses on antimicrobial stewardship. However, the assessment found that there is insufficient coordination of antimicrobial users. The technical group 4 of the national multisectoral coordination group in charge of antimicrobial stewardship needs to be operational and have more funding to scale up antimicrobial stewardship activities.

Finding: Insufficient coordination of the various sectors using antibiotics ;
Proposed strategic area: improve coordination of the different sectors using antibiotics.

Objectively verifiable indicators (OVI): number of meetings held; allocation of funds for coordination activities.

Effective communication and education

In accordance with the first strategic objective of the NAP-RAM, a communication plan has been developed.
Findings:
Difficulties in mobilizing financial resources are hampering the implementation of the plan.
No study on the impact of AMR awareness campaign measures.
Proposed strategic directions :

- Improve financial resource mobilization.
- Plan a study on the impact of AMR awareness campaign measures;

IOV :

- Number of activities carried out ;
- Number of awareness campaigns organized ;
- Number and quality of veterinary professionals and para-professionals involved in awareness campaigns ;
- Number of studies on the impact of AMR awareness campaign measures conducted ;
- Number of communication materials developed.

Training and professional education on appropriate antimicrobial use in the veterinary sector

It is true that the country has several veterinary practices and clinics but few veterinarians. They are not always available to prescribe and follow up with the breeders.

- **Observation:** There is no training structure for veterinarians. On the other hand there is little progress in the recruitment of veterinarians in the public system.

- **Proposed strategy**: provide for the in situ training of veterinarians and the mechanism for their recruitment into the public service.

- **OVI**: number of professionals trained and recruited.

Strengthening veterinary services

Mali's Veterinary Services have been evaluated and plans have been developed to strengthen capacity, using a structured approach such as the Veterinary Services Performance Evaluation process and OIE risk analysis missions. It should be noted that consumption and import data of veterinary drugs including antimicrobials are not analysed for decision making.

- **Finding:** Veterinary Services have limited capacity to exercise regulatory and administrative control over veterinary drugs and biologicals to ensure their responsible and prudent use. There is no database for the analysis of

- **Proposed strategic focus**: Develop and implement the strategic plan for the control of veterinary drugs (2019 - 2023)

- **IOV**: plan implemented by the DNSV with the support of the MEP

Monitoring and Research

Existing surveillance systems are weak enough to identify emerging threats or changes in antimicrobial resistance and antimicrobial use in animals and the use of DAOAs
Findings:

- No national plan or system for monitoring the sale/use of antimicrobial agents in animals exists in Mali;

- There is little data on the extent and impact of AMR on animal health in Mali;

- There is no mechanism to coordinate the AMR surveillance and antimicrobial use monitoring (AMU) system at the national level in the area of animal health.

Proposed strategic directions :

- Design a national monitoring system to control the quantities of antimicrobial agents sold/used in animals, based on OIE standards;

- Improving the quality of analyses with laboratory accreditation;

- Establish a mechanism that coordinates the AMR surveillance and antimicrobial use monitoring (AMU) system at the national level including an animal health database;

IOV :

- Data collected on antimicrobial agents sold/used in animals ;
- Existence of a mechanism that coordinates the AMR surveillance system and antimicrobial use monitoring (AMU) at the national level;
- LCV's accreditation certificate for the quality of its analyses is available.

STRENGTHENING THE REGULATORY FRAMEWORK

WAEMU encourages countries to transpose the directives into national regulations. Some directives have been transposed into Mali's laws, but the implementing legislation for these laws is still missing.
Observation: Absence of implementing legislation for certain laws
Proposed strategic directions :

- To elaborate the texts of application of the law 004-2016 of February 12 governing the veterinary pharmacy ;
- Establish effective enforcement procedures and monitoring to ensure compliance with the legislation;
- Adapt guidelines for the responsible and prudent use of antimicrobials based on international standards (e.g. OIE Terrestrial and Aquatic Animal Health Codes and Codex Alimentarius);
- Implement guidelines for the use of critically important antimicrobial agents, and phase out antibiotics used for growth promotion in the absence of a risk analysis.

IOV :

- Number of enforcement procedures in place ;
- Number of effective checks carried out ;
- Number of antimicrobials used for growth promotion in the absence of risk analysis removed.

PROPOSED ANIMAL HEALTH ANTIMICROBIAL STEWARDSHIP ACTION PLAN

The Government of Mali recognizes that AMR is a complex, multi-sectoral issue that needs to be addressed in a coordinated manner that takes into account the social, economic and health needs of Malians.

In order to focus on this issue, the government is committed to the fight against AMR through several actions. A national expert was appointed by the Ministry of Health to draft a national action plan for the fight against antimicrobial resistance in 2014 (Letter N°1868 MSHP-SG of 20 August 2014). A guide to establish laboratory surveillance of AMR in Mali through a national AMR surveillance network was developed with the support of WHO in 2014. To this guide was annexed a national action plan 2014-2016.

Although the government of Mali has some important levers to tackle AMR through laws and regulations, it is only by uniting the efforts of different sectors that it will be able to implement strong measures against AMR on a national scale. For this reason, the government has brought together partners and stakeholders from the human and animal health, agriculture and environment sectors to develop an inclusive national AMR strategy to encourage prudent use of antimicrobials. After extensive work with TFP partners and other stakeholders from across the human and animal health sector, the document entitled: National Action Plan (NAP) for Antimicrobial Resistance (AMR) Control "NAP-AMR 2019-2023" was developed.

The Framework document outlines action areas and targeted outcomes under the pillars of surveillance, stewardship, infection prevention and control, and research and innovation.

Mali continues to play an active role in international efforts to combat AMR. In May 2015, Member States, including Mali, endorsed the World Health Organization's (WHO) Global Action Plan to Combat AMR. During a UN General Assembly high-level meeting on AMR in September 2016, global leaders, including Mali's representative, recognized the Global Action Plan to Combat AMR as a model for AMR. These international agreements provide a significant boost to the government of Mali as it moves forward with its One Health approach to AMR.

SURVEILLANCE AND RESEARCH (SEE STRATEGIC OBJECTIVE 2 OF NAP-MALI: STRENGTHEN THE KNOWLEDGE AND EVIDENCE BASE THROUGH SURVEILLANCE AND RESEARCH)

The aim is to detect and monitor emerging trends and threats in order to guide strategies to reduce the risks and effects of antimicrobial resistance.
Evidence-based decision making is central to the development of public policy on health issues, including AMR. The data needed to formulate this evidence are obtained through rigorous and comprehensive monitoring of all agricultural, human and animal health and environmental sectors. As part of Mali's engagement with international partners, the country is a member of the Global Antimicrobial Resistance Surveillance System, initiated by the OIE, and is expected to participate in the collection, analysis and sharing of AMR data at the global level.

National AMR surveillance should be a key priority under the National Action Plan. Unfortunately in Mali, at present, animal health data are not available.

There is little data on the extent and impact of AMR in Mali, particularly those relating to

- The incidence and prevalence of AMR in humans, animals, plants, food and the environment;
- The economic impact of AMR in humans, animals, plants, food and the environment.
- No national AMR surveillance and antimicrobial use (AMU) reports are available, including AMR in human, animal, plant, food, environmental isolates and their uses in humans, animals and plant production.

Similarly, there is no mechanism that coordinates the AMR surveillance and antimicrobial use monitoring (AMU) system at the national level.

However, there are national reference laboratories, in particular: the INSP, the LCV and the LNS, which have the technical capacities required to carry out activities on AMR; unfortunately, they are not officially designated.

There is also no national AMR research programme covering a wide range of topics:

- Research to develop new treatments, diagnostic tools, vaccines or other interventions in humans, animals or plants related to infectious diseases;

- Research to find alternatives to non-therapeutic uses of antimicrobial agents in animals, including their use as growth promoters;

- Economic research, including the development of models to assess the cost of AMR and the costs and benefits of the National Action Plan to the human and animal health, food, crop production and environmental sectors.

- The lack of data in veterinary practices and clinics and within the DNSV is a major gap in the surveillance of AMR and AMU in Mali. To address these gaps, the LCV may initiate ad hoc survey projects on the incidence and prevalence of AMR in animals during this year towards the last quarter of 2020 in order to better define the burden of antimicrobial resistant organisms that are a top priority. The findings from these projects can be used to guide policy, stewardship and research initiatives.

Systems to collect antibiotic use data from veterinary practices, clinics, and livestock operations, including data on prescriptions issued by veterinarians, antimicrobials imported by wholesalers, and diagnoses for which veterinarians have recommended an antimicrobial on livestock farms, could be complemented by the AMR surveillance work that would be undertaken by LCV. For example, LCV could provide diagnostic testing services to identify and characterize existing and emerging resistant organisms from animal sources.

Monitoring AMU rates in animals is an important complement to surveillance of AMR infections and organisms. AMU data are essential to the development of the "One Health" approach to controlling the spread of AMR and to analyze trends to identify potential sources of resistance development.

The NDSU could organize a workshop on AMU surveillance in food animal farms to identify gaps and reach consensus on the way forward in the area of animal health.

The Ordre des Vétérinaires could also mobilize and support the veterinary community through the Association Malienne des Docteurs Vétérinaires (ANDVM) to develop a prototype AMU surveillance system based on prescriptions.
This work could also be complemented by a project that the DNSV will initiate with the Permanent Assembly of the Chamber of Agriculture (APCAM) to gather data on the AMU at the farm level.

The MEP could also commission the development of a "roadmap" to address data and systems gaps related to AMU surveillance in the livestock sub-sector, which can be used to provide a harmonized, value-added approach to AMU surveillance.

STEWARDSHIP

Maintain the effectiveness of existing treatments through infection prevention and control guidelines, education and awareness, regulation, and control measures.
Antimicrobial stewardship refers to coordinated actions to encourage, improve, monitor and evaluate prudent use of antimicrobials in order to safeguard the future effectiveness of antimicrobials and to promote and protect human and animal health. The stewardship activities proposed in the plan can be divided into two broad categories, namely (i) awareness and education activities for

Veterinary Services professionals and para-professionals and livestock producers and (ii) regulation and surveillance.

Awareness and education (see Strategic Objective 1 of NAP-Mali: To improve awareness and understanding of antimicrobial resistance through effective communication, education and training).

Activities in this category will focus on outreach and education of livestock producers and practising veterinarians in offices and clinics, as well as advice on care. The NIVS could play a facilitating role in antimicrobial stewardship, bringing together organizations and experts from various fields to join forces and take action.

Due to growing concerns about AMR in Mali, DNSV may organize an awareness campaign on antibiotic use in 2020-2021 for the general public, livestock farmers and veterinarians. A baseline study should be conducted before and after the campaign to evaluate the effectiveness of the activities.

2020-2021 AMR Awareness Campaign

During this campaign, a variety of tools and means should be used to raise awareness of the issue of antimicrobial misuse and its relationship to antimicrobial resistance among livestock producers, veterinarians, and the general public. A series of awareness products, including posters, brochures, and audio and video cassettes, may be developed. Dissemination of the messages in national languages through social media, local radio, national television and the press could result in widespread outreach throughout the country.

Regulation and surveillance (c.f. Strategic objective 4 of PAN-Mali: Optimize the use of antimicrobial agents in human, animal, environmental health and plant production)

The abuse and misuse of antimicrobials in animals are factors that contribute to the development and spread of AMR in human and animal populations.

Antimicrobial resistant pathogens found in animals can pose serious risks to human health when transmitted as contaminants in food or water. To address this threat, the NDSU could develop new antimicrobial rules under the OIE guidelines to protect Malians from AMR. This could include restricting the importation of certain antimicrobials used in food animals for personal use, and requiring companies to follow more stringent guidelines to ensure the quality of active ingredients in their pharmaceutical products.

A platform should also be created with regulatory authorities, the pharmaceutical industry, veterinarians, the feed industry, food animal producers, and other stakeholders to promote the prudent use of antimicrobial drugs in animals.

Within the framework of the regulations it is suggested to :

- Restrict the importation for personal use of certain antimicrobials used in food animals, and increase controls on the importation of health-relevant antimicrobials, including active pharmaceutical ingredients;

- Require companies to follow more stringent guidelines to ensure the quality of active ingredients in their pharmaceutical products;

- Require antimicrobial manufacturers and importers to report annual sales of health-significant antimicrobial drugs to the NDSB to facilitate surveillance;

- Introduce a more flexible and risk-appropriate framework to simplify the importation and sale of low-risk veterinary drugs, including those that can be used to improve animal health and welfare, and reduce the need for antimicrobials;

It would also be appropriate to draw the attention of veterinary professionals and pharmacists to the requirement that veterinary drugs containing critically important antimicrobials (refer to the OIE list) be sold by prescription only. These measures will require oversight by veterinarians and encourage the prudent use of these drugs. All such antimicrobial products administered in water or food will also require specific prudent use statements to strengthen antimicrobial stewardship. Preserving the effectiveness of antimicrobials is an important public health objective. Since antimicrobial resistant bacteria can be transmitted to humans from animals through the food chain and affect the treatment of infections in humans, these regulatory and policy initiatives are important steps in protecting the long-term health and well-being of all Malians.

To enhance and promote better stewardship of antimicrobials for animal use, the NDSB could take action by requesting pharmaceutical companies to provide information on the labelling of antimicrobial products to indicate its intention to improve the education of animal health professionals and to facilitate the prudent use and prescribing of antimicrobials for animals by requiring the inclusion of AMR warnings in the labelling of all antimicrobials for animal use. The NDSU could also request that drug sponsors update their product labelling with particular emphasis on how and when to use antimicrobials, and promote the selection of the correct antimicrobials for use in animals.

Research and innovation (c.f. NAP-Mali Strategic Objective 5: Make the economic case for sustainable investments that take into account the country's needs and increase investments in the development of new drugs, diagnostics, vaccines and other interventions)

Create new solutions through research and development activities to counteract the loss of antimicrobial efficacy.

Research and innovation are key to a comprehensive, multi-sectoral approach to AMR. Ministries and scientific agencies need to undertake in-house research, collaborate with experts from the animal industry and academia, and fund research activities to increase knowledge, develop new products and approaches to reduce AMR, and support appropriate antimicrobial use. The Government of Mali should also collaborate with international partners (OIE, FAO, WHO) to contribute to global research activities on AMR, AMU, new treatments and alternatives to antibiotics.

As part of the research it is suggested to carry out :

- Research to develop new treatments, diagnostic tools, vaccines or other interventions in humans, animals or plants related to infectious diseases;

- Research to find alternatives to non-therapeutic uses of antimicrobial agents in animals, including their use as growth promoters;

- Economic research, including the development of models to assess the cost of AMR and the costs and benefits of the National Action Plan to the human and animal health, food, crop production and environmental sectors.

- The Economic Institute for Agricultural Research (ERI) could also be tapped to undertake a genomics-based research approach to better understand how food production contributes to the development of AMR of human health concern and to explore strategies to reduce it in food production systems. The focus would be on food because of the widespread use of antibiotics in food production and the impact this has on the abundance of antimicrobial resistant bacteria in food products.

- Therefore, it would be desirable to initiate a genomic research and development project on AMR within the IER for a period of three years (2020-2023) for an amount of 30 million FCFA. This AMR project would focus on research that will guide policies, practices and products that will mitigate the development of AMR. The research under this project will answer the following questions:

    - What is the link between certain agricultural practices and the burden of AMR in food animals and their environment?

    - What is the link between exposure to agricultural AMR and the development of AMR through pathogens in humans, animals or the general environment?

    - How can genomic information be incorporated into the risk assessment models needed to guide desirable policies and practices?

As a major contributor to prevention and treatment, vaccines are low-cost, low-risk solutions that improve quality of life and reduce treatment costs in animal health. By improving the body's ability to fight harmful microorganisms, vaccines reduce the incidence and spread of disease, which in turn reduces the likelihood that AMR will develop. The LCV could initiate a project to identify a set of priorities for research and development of new and improved animal vaccines, with a particular focus on AMR. Potential animal industry collaborators will be invited to work with the LCV to develop and commercialize vaccines that will prevent infectious diseases, with an emphasis on AMR and emerging infections.

Developing the capacity to identify, characterize and diagnose endemic and emerging antimicrobial resistant organisms by developing and transferring diagnostics and laboratory approaches could also be important scientific components for the LNS.

Strategic interventions	Activities	Responsible for implementation	Identification of risks	Risk mitigation measures
Monitoring	Possibly initiate ad hoc survey projects on the incidence and prevalence of AMR in animals to better define the burden of antimicrobial resistant organisms as a top priority;<br><br>Provide diagnostic testing services to identify and characterize existing and emerging resistant organisms from animal sources;<br><br>Monitor AMU levels in animals;<br><br>Organize a workshop on AMU surveillance in food animal farms to identify gaps and reach consensus on the way forward in animal health;<br><br>Mobilize and support the veterinary community through the Malian Association of Veterinary Doctors (ANDVM) to develop a prototype AMU surveillance system based on prescriptions;<br><br>Initiate a project with the Permanent Assembly of the Chamber of Agriculture (APCAM) to collect data on the AMU at the farm level;<br><br>Commission the development of a "roadmap" to address data and systems gaps related to AMU surveillance in the livestock sub-sector.	LCV<br><br>LCV<br><br>DNSV<br><br>DNSV<br><br>Order of Veterinarians<br><br>DNSV<br><br>MEP	Insufficient financial resources.	Ensure sustainable funding for monitoring activities.
Awareness, Education and Training	To raise awareness and educate farmers and practicing veterinarians in practices and clinics, as well as on care advice;<br><br>Organize an awareness campaign on the use of antibiotics for the general public, farmers and veterinarians;<br><br>Conduct a pre- and post-campaign baseline study to evaluate the effectiveness of the activities;<br><br>Produce posters, brochures, and audio and video tapes to raise awareness of the issue of antimicrobial misuse and its relationship to antimicrobial resistance among livestock producers and veterinarians and the general public;<br><br>Disseminate messages on social media, local radio, national television and the press;<br><br>Develop training curricula ;	DNSV<br><br>DNSV<br><br>DNSV<br><br>DNSV<br><br>DNSV<br><br>DNSV<br><br>MEP<br><br>DNSV	Weak human resources involvement ;<br><br>Insufficient financial resources ;<br><br>Instability and lack of qualified personnel.	Motivating human resources ;<br><br>Ensure sustainable funding for AMR training and awareness activities;<br><br>Recruit, train and motivate qualified personnel.

Strategic interventions	Activities	Responsible for implementation	Identification of risks	Risk mitigation measures
	Initiate a University for the training of veterinarians;<br><br>Provide for continuing education of animal professionals;<br><br>Encourage the recruitment of animal health professionals into the public service.	MEP		
Regulation	Develop new antimicrobial rules under the goose guidelines to protect Malians from AMR;<br><br>Restrict the importation for personal use of certain antimicrobials used in food animals ;<br><br>Increase controls on the importation of health-relevant antimicrobials, including active pharmaceutical ingredients;<br><br>Require companies to follow more stringent guidelines to ensure the quality of active ingredients in their pharmaceutical products;<br><br>Require antimicrobial manufacturers and importers to report annual sales of health-significant antimicrobial drugs to the NDSB to facilitate surveillance;<br><br>Require the sale of veterinary drugs containing critically important antimicrobials (refer to the OIE list) only on prescription;<br><br>Require the inclusion of AMR warnings in the labelling of all animal-use antimicrobials;<br><br>Request that drug sponsors update their product labelling with particular emphasis on how and when to use antimicrobials.	DNSV<br><br>DNSV<br><br>DNSV<br><br>DNSV<br><br>DNSV<br><br>DNSV<br><br>DNSV<br><br>DNSV	Insufficient financial resources ;<br><br>Lack of adequate logistical resources ;<br><br>Non-compliance with the supply chain ;<br><br>Inadequacy and non-application of texts ;<br><br>Lack of political will.<br><br>Lack of follow-up in the application of texts.	Mobilizing financial resources<br><br>Mobilize material and financial resources ;<br><br>Strengthen legislation on supply chain compliance ;<br><br>Strengthen enforcement ;<br><br>Advocacy to build political will.<br><br>Strengthen the capacity of the Veterinary Services
Research and innovation	To develop new treatments, diagnostic tools, vaccines or other interventions in animals related to infectious diseases;<br><br>To find alternatives to non-therapeutic uses of antimicrobial agents in animals, including their use as growth promoters;<br><br>Initiate an AMR genomics research and development project;<br><br>Initiate a project to identify a set of priorities for research and development of new and improved animal vaccines, with particular emphasis on AMR.	LCV<br><br>LCV<br><br>IER<br><br>LCV	Insufficient financial resources ;<br>Instability and lack of qualified personnel	Ensuring sustainable funding for AMR research activities ;<br>Recruit, train and motivate qualified personnel;

CONCLUSION AND NEXT STEPS

The rapid assessment of the legislative and regulatory texts and the antimicrobial supply chain in the field of animal health in Mali, the identification of key actors, their roles and responsibilities, and the proposal of strategic axes and a stewardship plan to be submitted to the members of the multisectoral group of the PAN_RAM in Mali were the central elements of this consultation.

The objective of the consultation was achieved as the assessment covered the five key areas namely: national antimicrobial legislation, regulations or policies, Standard Therapeutic Protocols (STPs), Essential Medicines List (EML), supply management policy and appropriate use of antimicrobials by prescribers, distributors and farmers.

All the key elements were concerned, in particular the MA and registration of veterinary medicines, licensing, control procedures for veterinary medicines at the time of application for MA, at import, after marketing, pharmacovigilance, prescription, distribution and use. Following the findings listed, suggestions were made to address the main problems identified.

The next step will be to share the different reports with the DNSV and the GCMN and to integrate the validated Animal Health assessment report with the Human Health report in order to have a single report for this assessment in Mali;
It would also be good to improve the synergy of action and to pool efforts in the framework of the implementation of the PAN-RAM of Mali;

The work carried out could allow the DNSV to validate the strategic axes identified for the antimicrobial resistance (AMS) stewardship action plan to address the main problems identified in order to improve the animal health component included in Mali's PAN-RAM.
A truly national approach to AMR requires the active participation of a wide range of partners from several sectors, including key government departments such as human health, animal health, agriculture and environment, and the private sector. As we move forward with the development and implementation of the National Action Plan in the coming months, partners are invited to work with the government to establish and renew their commitment to strong, concrete and coordinated action to combat AMR and promote the well-being and prosperity of Malians. The National Action Plan will identify activities currently being undertaken or planned, with timelines and measurable outcomes.

APPENDIX: HUMAN HEALTH :

APPENDIX 1: LIST OF HUMAN HEALTH STRUCTURES INTERVIEWED :

First and last name	Structure	Contact
Monzon TRAORE<br>Boubacar MAIGA	Faculty of Medicine and Odontostomatology	7646 0459
Sekou BAH<br>Issa COULIBALY	Faculty of Pharmacy	7527 7527, sekoub2000@yahoo.com
Abdoulaye TRAORE	National Institute of Training in Health Sciences	7610 7774, glek10@yahoo.fr
Abou COULIBALY	National Health Laboratory	6693 5600, cheickabouc@yahoo.fr
	Health Inspection	
Ibrehima GUINDO	GCMN, RAM Focal Point	7607 8639, guindo50@gmail.com
Seydou Baba TRAORE<br>Souleymane TRAORE and	National Centre for Health Information, Education and Communication	6672 5996, sebatra2000@yahoo.fr<br>6680 3986, sstraore14@yahoo.fr
Alioune I A DIOUF	World Health Organization	7554 3201, adiouf@who.int

APPENDIX 2: LIST OF STATUTORY INSTRUMENTS :

Law No. 02 - 049 / of 22 July 2002, on the orientation law on health adopted in 2002
Law n° 01 - 078 / of July 18, 2001, on the control of drugs and precursors;
Law No. 62-56 / AN-RM of 6 August 1962, authorizing the Government to accede to the Single Convention on Narcotic Drugs;
Act No. 95-045 of 12 June 1995, authorizing the accession of the Republic of Mali to the United Nations Convention against Illicit Traffic in Narcotic Drugs and Psychotropic Substances, adopted in Vienna on 20 December 1988.
Law No. 02 - 050 / of July 22, 2002, on the hospital law sets up (04) advisory committees including the Technical Committee on Health and Safety
Law n° 01 - 040 / of June 7, 2001, creating the Directorate of Pharmacy and Medicines (DPM)
Law n°86-36/AN-RM establishing the National Order of Pharmacists
Act No. 85-41/AN-RM of 22 June 1985 on the authorization of the private practice of health professions
Decree 2018-0369/PR-RM of 12 April 2018 on the creation, powers, organization and operation of the "One Health" Platform in Mali.
Decree No. 199 / PG-RM of 12 July 1988, establishing the list of narcotics
Decree No. 245 / PG-RM of 12 July 1988, establishing the list of poisonous substances and plants;
Decree No. 96-302 / PRM of 7 November 1996, establishing a National Commission to Combat Drugs;
Decree No. 97- 230 / PRM of August 8, 1997, establishing the modalities for the importation, storage and delivery of poisonous substances and narcotics

Decree No. 2011-4201 of October 14, 2011, establishing the procedures for setting up pharmacovigilance
Decree No. 05 - 299 / P-RM of 28 June 2005, establishing the conditions for the creation and the fundamental principles of operation of Community Health Centres (Cscom)
Decree No. 91-106/P-RM of 15 March 1991, on the organization of the private practice of health professions
Order n° 00 - 039 /P-RM of September 20, 2000, creating the Directorate of Pharmacy and Medicines (DPM)
Inter-ministerial order n° 91-2776 / Mef - Maee - Mspaspf, fixing the conditions of importation of pharmaceutical and veterinary products ;
Inter-ministerial decree n° 05 - 2203 / MS - MEP - SG of 20 Sept. 2005, Determining the modalities for requesting marketing authorisations (MA) for medicines for human and veterinary use.
Inter-ministerial order n° 08-0345/MS-MFMEIC MEP-MA of February 13, 2008, fixing the practical modalities and pricing of quality control operations for medicines, food, water and beverages
Order No. 96-0814/MFC-SG establishing the list of products subject to monthly stock declaration

APPENDIX 3: HUMAN HEALTH BIBLIOGRAPHY :

CADD: Collection of Laws and Regulations on Decentralization, Health and Social Development, June 2019 Edition ;
CNOP: collection of texts from the National Council of the Order of Pharmacists
SIAPS: Evaluation of the DPM drug regulatory system, Mali, October 2017;
MSH: Mali: supply chain diagnosis, July 2018;
Cabinet McKenzy: Integrated drug supply chain in Mali, supply chain diagnostic, May 2018;
PQM: Evaluation Report of the Pharmaceutical System in Mali, Evaluation Missions of Pr A TOUMI, May - September 2017

APPENDIX 4: DATA COLLECTION TOOL :

DETAILED GUIDANCE QUESTIONS FOR A QUICK ASSESSMENT OF THE SITUATION[1]

The following guiding questions provide details on the issues to be explored during the desk review and stakeholder interviews. The guiding questions are listed by thematic area.

Instruction: Answer "Yes" or "No" to each question and add notes with facts from key documents (policies, legislation, regulations or guidelines) or data, as appropriate

National regulations, legislation or policies

1. Is there a national pharmaceutical policy that provides guidance on the proper management and regulation of medicines?

2. Are there other policies that support drug regulation, with a particular focus on antimicrobials?

[1] USAID SIAPS. 2017. Building Coalitions for Containing Antimicrobial Resistance: A Guide. Management Sciences for Health. http:/apps.who.int/medicinedocs/en/m/abstract/Js23293en/

3. Is there any legislation or regulation requiring registration of medicines used in the country? If so, to what extent is it enforced?

4. Are there any laws or regulations requiring the registration of pharmaceutical dispensing premises/points, including pharmacies? If so, who is responsible for enforcement and to what extent is it enforced?

5. Are there any regulations requiring the registration of pharmaceutical personnel by a practitioner registration authority? If so, to what extent is it enforced?

6. How many antimicrobial products are registered (including all branded antimicrobials)?

7. How many antimicrobial agents (single chemical entities, not including different brands) are registered? How many of these agents are on the WHO model list of essential medicines?

8. Does the national drug regulatory authority have quality control/quality assurance and antimicrobial registration tools in place?

9. Are pharmacies regularly inspected and monitored to ensure compliance with laws and regulations with enforcement mechanisms?

10. Are data available on the degree of enforcement of regulations? Are data available on the impact of regulations?

11. Is there a system in place to monitor antimicrobial sales across the country? If so, what guides the system?

12. Are there formal regulations for the import, export, production, distribution, storage and use of finished antimicrobial products and the corresponding active pharmaceutical ingredients?
13. Are there regulations and guidelines to govern the promotional activities of pharmaceutical companies? (If yes, ask the additional questions below starting with question 13a. If no, go to the next question).

    13a. What regulations are in place?
    13b. Are data available on the degree of enforcement of regulations?
    13c. Are data available on the impact of regulations?

14. Are there any regulations limiting antimicrobials to prescription-only status? (If yes, go to question 14a. If no, go to the next question.

    14a. Are there any exceptions to the regulations?
    14b. What processes have been adopted to monitor the implementation of these regulations?
    14c. Are data available on the degree of implementation of this policy?

Drug use and AMR policies/regulations

15. ***Standard Treatment Guidelines (STGs)*** Is there a policy/recommendation guiding the use of STGs? (Try to collect information for all levels of prescribing and for all STGs existing in the country)

16. What policies/recommendations are in place (e.g., mandatory or voluntary adherence to SDRs in a hospital facility, availability of drugs recommended in the SDR in the hospital pharmacy)?

17. Are there any SDRs for the management of infectious diseases? (Yes/No. (If yes, ask the additional questions about SDRs listed below beginning with question 17.A. If no, proceed to the questions about EMLs.)

    17.A What was the process of their development?
    17.B How often are they reviewed? When was the last review?
    17.C Are there STDs for different levels of health care practices?
    17.D Are prescribers (at different levels) trained in the use of and compliance with SDRs? If yes, what types of prescribers are trained and how often?
    17.E Do health students (medical, pharmacy, nursing, community health workers) receive training on the importance of SDRs?
    17.F Has the availability of SDRs in health facilities been assessed?

18. Is there a mechanism to monitor compliance with SDRs? If AWaRe classification is applied, is it being followed?

19. Is there any data on the level of compliance with SDRs in the public and private sectors?

20. Are there guidelines on prescribing antimicrobials, including antibiotics? What is their basis: what is the law, policy, regulations, guidelines. Is the WHO AWaRe classification for antimicrobials used? If yes, please indicate the basis and frequency of its revision (If yes, ask the additional questions below starting with question 20.A. If no, go to the next question).

    20. A What are the guidelines for classification of antimicrobials and their level of use, and authorization for prescribers? Are some antimicrobials defined and held as "reserve" (e.g., some antimicrobials are reserved for treatment of certain diseases only or can only be prescribed by a certain class or level of prescriber)?
    20.B Have antimicrobial prescribing authority levels been defined?
    20.C What levels of prescribers can prescribe antimicrobials? (Gather information on which antimicrobials nurses can prescribe [if authorized], which clinicians can prescribe, and which physicians can prescribe).
    20.D Are new policies being developed to give prescribing rights to a wider group of health professionals?
    20.E Are there other regulations on antimicrobial prescribing?
    20.F What is the level of implementation of these policies?
    20. G Is there any data on the impact of these policies?

21. Is there a national system for monitoring adverse drug reactions (pharmacovigilance)? If yes, describe its activities over the past 12 months. If no, are there any plans to establish such a

system?

22. Is there an independent national drug information service? If yes, describe its activities over the past 12 months, including the number of inquiries handled. If no, are there plans to launch such a service?

23. What percentage of medicines (in terms of their monetary value) used in the country is supplied by the private sector and what percentage by the public sector? What proportion of this percentage is antimicrobials in each sector?

24. What percentage of medicines used in the country are manufactured locally? What percentage of medicines used in the country are manufactured locally compared to those that are imported?

LME

25. Is there an EML that includes antimicrobials (antimicrobials include antibiotics, antifungals, antivirals, and antiprotozoals)? (Yes/No. (If yes, ask the follow-up questions on EMLs listed below beginning with question 25A. If no, proceed to follow-up questions).

    25.A What was the selection process for the antimicrobials included in the EML?
    25.B How often is the EML reviewed? When was it last revised?
    25.C Are antimicrobials identified and applied according to levels of health care delivery?
    25.D Do the antimicrobial drugs included in the EML match those in the SDR for infectious diseases?
    25.E Are health care providers trained in the concept of EML and rational use of medicines?
    25.F Are students in medical, pharmacy, nursing and other paramedic courses trained in the MEL concept?
    25.G Is there data on the availability of MEL in health facilities?

    25.H Is there survey data on the availability in health facilities of medicines (including antimicrobials) included in the EML?

26. Are pharmacists legally allowed to dispense certain antimicrobials without a prescription or for specific indications?

Good Procurement Policies

For the purposes of this evaluation, the suggested issues related to good supply management focus on policies or guidelines related to pharmaceutical quality and the use of the EML.

27. What quality assurance mechanisms are in place in the public and private sectors to ensure the quality of antimicrobials marketed in the country?

28. How many public and private laboratories are capable of testing the quality of antimicrobials?

29. Which of the following quality assurance mechanisms are in place to ensure the quality of antimicrobials?

- Pre-qualification of suppliers
- Supplier submits a registration form
- Physical inspection of drug samples
- Laboratory analysis of drug samples
- Specific reports requested on drugs, such as bioavailability
- Informal information gathered from other procurement programs
- The tender/order documents specify the acceptable pharmacopoeial standards
- Certificate of Good Manufacturing Practice
- Monitoring of the quality of marketed products
- Inspection at the point of import
- Inspection at retail/wholesale pharmacy level
- Laboratory analysis of samples obtained at distribution points
- Monitoring and evaluation of supplier performance
- Other (describe)

30. To what extent are the above mechanisms/regulations applied?

31. Are there data on the proportion of antimicrobials below the standard over the last two years?

32. Are documented reports of adulterated antimicrobials available?

33. Is there a policy for using the EML antimicrobial list for antimicrobial procurement? (Yes/No. (If yes, ask the additional questions listed below beginning with question 33A. If no, proceed to other questions)

    33.A Does this policy exist in the public sector?
    33.B Does this policy exist in the private sector?
    33. C Is data available on the degree of compliance with this policy?

34. Are there data on antimicrobial consumption in value and quantity at the national level? Or at the national level for hospitals?

Appropriate use by prescribers, providers and patients

35. Are there any studies/reports on the appropriateness of appropriate antimicrobial prescribing and dispensing? Indicate the main findings of any available relevant literature.

36. Have public information campaigns on the appropriate use of antimicrobials/antibiotics and antimicrobial resistance been conducted in the past two years? (Yes/No.) (If yes, ask the additional questions listed below beginning with question 36A. If no, go to the next question).

    36.A What types of campaigns have been conducted and how often?
    36.B Who conducted these campaigns?
    36.C Are there information campaigns on infection prevention (e.g., hand hygiene, food hygiene, vector control, vaccinations)?
    36.D Are there information campaigns on the appropriate use of antimicrobials/antibiotics and awareness of antimicrobial resistance issues?
    36.E Are data available on the impact of these measures?

37. Are data available on public use of antimicrobials (including the degree of appropriate use)?

38. Has the country participated in the WHO Global Week for Appropriate Use of Antibiotics or other global or regional awareness campaigns on antimicrobial resistance? If yes, briefly describe the participation or activities.

APPENDIX 5: HUMAN HEALTH INTERVIEW QUESTIONNAIRE :

Domains	Structure / Resource person
Treatment protocols	
17.E Do health students (medical, pharmacy, nursing, community health workers) receive training on the importance of NTPs?	- FPMOS-<br>FPH-<br>INFSS
LME	
25.A What was the selection process for the antimicrobials included in the EML?<br><br>What is the rate of reporting of adverse events and the proportion of antibiotics	DPM
25.F Are medical, pharmacy, nursing and other allied health students trained in the EML concept?	- FPMOS-<br>FPH-<br>INFSS
Good Management	
31. Is there any data on the proportion of antimicrobials below the standard over the last two years?<br>Total quantity of drugs tested and the proportion of antibiotics	LNS
32. Are documented reports of adulterated antimicrobials available?	- DPM-<br>IS
Antimicrobial use	
36. 36. Have public information campaigns on the appropriate use of antimicrobials/antibiotics and on drug resistance been conducted in the last two years?<br>36.A What types of campaigns have been conducted and how often?<br>36.B Who conducted these campaigns?	- WHO -<br>NFP -<br>CNIEC
36.D Are there information campaigns on the appropriate use of antimicrobials/antibiotics and awareness of antimicrobial resistance issues?<br>36.E Are data available on the impact of these measures?	- WHO -<br>NFP -<br>CNIEC

ANNEX: ANIMAL HEALTH :

Appendix 6: Categorization of antimicrobial agents of veterinary medical importance used to treat animals whose flesh or products are intended for human consumption

Antimicrobial agents (class, subclass, substance)	Species	Specific comments	AICV	ATIV	AIV
AMINOCOUMARINE<br>Novobiocin	BOV, CAP, OVI, PIS	Novobiocin is used in the local treatment of mammitis and septicaemia in fish.			X
AMINOGLYCOSIDES<br>Aminocyclitol<br>Spectinomycin	AVI, BOV, CAP, EQU, LEP, OVI, PIS, SUI API, AVI, BOV, CAP, EQU, LEP, OVI, PIS, SUI AVI, BOV,	Aminoglycosides are extremely important in veterinary medicine because of the diversity of their use and the nature of the diseases treated with them. Aminoglycosides are important in the treatment of sepsis, digestive tract diseases, respiratory diseases and urinary tract diseases.			
Dihydrostreptomycin	CAP, EQU, LEP, OVI, SUI				
Aminoglycosides + 2 deoxystreptamines<br>Kanamycin<br>Neomycin<br>Framycetin<br>Paromomycin<br>Apramycin<br>Fortimycin<br>Gentamicin<br>Tobramycin<br>Amikacin	AVI, BOV, EQU, PIS, SUI API, AVI, BOV, OVI, SUI BOV, CAP, OVI AVI, BOV, CAP, OVI, LEP, SUI AVI, BOV, LEP, OVI, SUI AVI, BOV, CAM, CAP, EQU, LEP,OVI, SUI EQU	Gentamicin is indicated for the treatment of Pseudomonas aeruginosa infections and there are few alternatives. Apramycin and fortimycin are currently only used in animals. There are few alternatives at a reasonable cost.	X		
AMPHENICOLS<br>Florphenicol<br>Thiamphenicol	AVI, BOV, CAP, EQU, LEP, OVI, PIS, SUI AVI, BOV, CAP, OVI, PIS, SUI	Phenicols are extremely important in veterinary medicine due to the diversity of their use and the nature of the diseases treated with them.<br>This class is of particular importance in the treatment of	X		

		certain fish diseases, for which there are currently very few, if any, alternative treatments.<br>This class also offers a useful alternative for the treatment of respiratory infections in cattle, swine and poultry.<br>This class, in particular florphenicol, is used to treat pasteurellosis in cattle and swine.			
ANSAMYCIN - RIFAMYCINS<br>Rifampicin<br>Rifaximin	EQU BOV, CAP, EQU, LEP, OVI, SUI	This class of antimicrobial agents is approved in only a few countries and for a very limited number of indications (mastitis). There are few alternatives.<br>Rifampicin is essential in the treatment of Rhodococcus equi infections in foals. However, it is only available in a few countries, resulting in its classification as an ATIV.	X		
ARSENICALS<br>Roxarsone<br>Nitarsone	AVI, SUI AVI, SUI	Arsenicals are used to control intestinal coccidiosis (Eimeria spp.).			X
BICYCLOMYCINE<br>Bicozamycin	AVI, BOV, PIS, SUI	Bicyclomycin is indicated for digestive and respiratory diseases in cattle and for septicaemia in fish.			X
CEPHALOSPORINS<br>First generation cephalosporins<br>Cefacetrile<br>Cefalexin<br>Cephalotin<br>Cefapyrin<br>Cefazolin<br>Cefalonium	BOV BOV, CAP, EQU, OVI, SUI EQU BOV BOV, CAP, OVI BOV, CAP, OVI	Cephalosporins are used in the treatment of septicemia, respiratory infections and mastitis.		X	
Second generation cephalosporins<br>Cefuroxime	BOV				
Third generation cephalosporins<br>Cefoperazone<br>Ceftiofur<br>Ceftriaxone	BOV, CAP, OVI AVI, BOV, CAP, EQU, LEP, OVI, SUI AVI, BOV,	Third- and fourth-generation cephalosporins are extremely important in veterinary medicine because of the diversity of their use and the nature of the			

	OVI, SUI	diseases they treat.			
Fourth generation cephalosporins<br>Cefquinome	BOV, CAP, EQU, LEP, OVI, SUI	Cephalosporins are used in the treatment of septicaemia, respiratory infections and mastitis. Alternatives have limited efficacy due to inadequate spectrum of activity or the existence of antibiotic resistance.	X		
FUSIDIC ACID<br>Fusidic acid	BOV, EQU	Fusidic acid is used in the treatment of ophthalmic diseases in cattle and horses.			X
IONOPHORES<br>Lasalocid<br>Maduramycin<br>Monensin<br>Narasin<br>Salinomycin<br>Semduramicin	AVI, BOV, LEP, OVI AVI API, AVI, BOV, CAP AVI, BOV AVI, LEP, BOV, SUI AVI	Ionophores are essential for animal health as they are used to control intestinal coccidiosis (Eimeria spp.). There are few, if any, alternatives available. Ionophores are of critical importance for poultry. This class is currently only used in animals.		X	
LINCOSAMID<br>Pirlimycin<br>Lincomycin	BOV, SUI, AVI API, AVI, BOV, CAP, OVI, PIS, SUI	Lincosamides are essential in the treatment of mycoplasma pneumonia, infectious arthritis and haemorrhagic enteritis in swine		X	
MACROLIDES (C refers to chemical structure)<br>C14 Macrolides<br>Erythromycin<br>Oleandomycin<br><br>Macrolides C15<br>Gamithromycin<br>Tulathromycin<br><br>Macrolides C16<br>Carbomycin<br>Josamycin<br>Kitasamycin<br>Spiramycin<br>Tilmicosin<br>Tylosin<br>Mirosamycin<br>Terdecamycin<br>Tildipirosine<br>Tylvalosin	API, AVI, BOV, CAP, EQU, LEP, OVI, PIS, SUI BOV<br><br>BOV<br>BOV, SUI<br><br><br><br><br>AVI<br>AVI, PIS, SUI<br>AVI, SUI, PIS<br><br>AVI, BOV, CAP, EQU, LEP, OVI, PIS, SUI AVI, BOV, CAP, LEP, OVI, SUI API,	The wide range of applications and the type of diseases treated make macrolides very important agents for veterinary medicine.<br><br>Macrolides are used to treat infections by micoplasmas in cattle and cattle feed, digestive hemorrhage in cattle (Lawsonia intracellularis) and cattle abscesses (Fusobacterium necrophorum), when there are very few alternatives. Macrolides are also used for respiratory infections in cattle.	X		

Macrolides C17<br>Sedecamycin	AVI, BOV,<br>CAP, LEP,<br>OVI, SUI API,<br>AVI, SUI, PIS<br>AVI, SUI BOV,<br>SUI AV, SUI<br><br>SUI				
ORTHOSOMYCINS<br>Avilamycin	AVI, LEP	Avilamycin is used in the treatment of enteric diseases of poultry and rabbits.<br>This class is currently only used in animals.			X
PENICILLINS<br>Natural penicillins (including esters and salts)<br>Benethamine Penicillin<br>Benzylpenicillin<br>Penethamate (hydriodide)<br>Benzylpenicillin procaine/<br>Benzathine penicillin<br>Amdinopenicillins<br>Mecillinam<br>Aminopenicillins<br>Amoxicillin<br>Ampicillin<br>Hetacillin<br>Aminopenicillin + betalactamase inhibitor<br>Amoxicillin + Clavulanic Acid<br>Ampicillin + Sulbactam<br>Carboxypenicillins<br>Ticarcillin<br>Tobicillin<br>Ureidopenicillin<br>Aspoxicillin<br>Phenoxypenicillins<br>Phenoxymethylpenicillin<br>Phenethicillin<br><br>Antistaphylococcal penicillins<br>Cloxacillin<br>Dicloxacillin<br>Nafcillin	BOV AVI,<br>BOV, CAM,<br>CAP, EQU,<br>LEP, OVI, SUI<br>BOV, CAM,<br>CAP, EQU,<br>OVI, SUI<br>BOV, SUI<br>AVI, BOV,<br>CAP, EQU,<br>OVI, PIS, SUI<br>AVI, BOV,<br>CAP, EQU,<br>OVI, PIS, SUI<br>BOV<br>AVI, BOV,<br>CAP, EQU,<br>OVI, SUI AVI,<br>BOV, SUI<br>EQU<br>PIS<br>BOV, SUI<br>AVI, SUI<br>EQU<br>BOV, CAP,<br>EQU, OVI, SUI<br>BOV, CAP,<br>OVI,<br>AVI, SUI BOV,<br>CAP, OVI<br>BOV, CAP,<br>EQU, OVI,<br>AVI, SUI	Penethamate (hydriodide) is currently only used in animals.<br>Penicillins are extremely important in veterinary medicine because of the diversity of their use and the nature of the diseases treated with them.<br>This class is used in the treatment of sepsis, respiratory infections and urinary tract infections.<br>This class is very important in the treatment of a wide variety of diseases in many animal species. There are few alternatives available at a reasonable cost.	X		

Oxacillin					
PHOSPHONIC ACID<br>Fosfomycin	AVI, BOV, PIS, SUI	Fosfomycin is essential in the treatment of certain infections in fish. There are few alternatives. It is only available in a few countries, resulting in its classification as an ATIV,		X	
PLEUROMUTILINS<br>Tiamulin<br>Valnemulin	AVI, CAP, LEP, OVI, SUI AVI, SUI	This class is also essential for treating swine dysentery (Brachyspira hyodysenteriae). However, it is only available in a few countries, thus leading to its classification as an ATIV,		X	
POLYPEPTIDES<br>Enramycin<br>Gramicidin<br>Bacitracin	AVI, SUI EQU AVI, BOV, LEP, SUI, OVI	Bacitracin is used in the treatment of necrotic enteritis in poultry. This class is used in the treatment of septicemia, colibacillosis, salmonellosis and urinary tract infections.		X	
Cyclic polypeptides<br>Colistin<br>Polymixin	AVI, BOV, CAP, EQU, LEP, OVI, SUI BOV, CAP, EQU, LEP, OVI, AVI	Cyclic polypeptides are commonly used in the treatment of gram-negative enteric infections.		X	
QUINOLONES<br>First generation quinolones<br>Fluméquine<br>Miloxacin<br>Nalidixic acid<br>Oxolinic acid	AVI, BOV, CAP, EQU, LEP, OVI, PIS, SUI PIS BOV AVI, BOV, LEP, PIS, SUI, OVI	First-generation quinolones are used in the treatment of sepsis and infections such as colibacillosis.		X	
Second generation quinolones (fluoroquinolones)<br>Ciprofloxacin<br>Danofloxacin<br>Difloxacin<br>Enrofloxacin<br>Marbofloxacin<br>Norfloxacin<br>Ofloxacin<br>Orbifloxacin<br>Sarafloxacin	AVI, BOV, SUI AVI, BOV, CAP, LEP, OVI, SUI AVI, BOV, LEP, SUI AVI, BOV, CAP, EQU, LEP, OVI, PIS, SUI AVI, BOV, EQU, LEP, SUI AVI, BOV, CAP, LEP, OVI, SUI AVI, SUI BOV, SUI	Fluoroquinolones are extremely important in veterinary medicine due to the diversity of their use and the nature of the diseases treated with them. Fluoroquinolones are critically important in the treatment of sepsis, respiratory diseases and enteric diseases.	X		

	PIS				
QUINOXALINES<br>Carbadox<br>Olaquindox	SUI SUI	Quinoxalines (carbadox) are used to treat digestive tract diseases in pigs (e.g. swine dysentery).<br>This class is currently only used in animals.			X
SULFONAMIDES<br>Sulfachlorpyridazine<br>Sulfadiazina<br>Sulfadimethoxine<br>Sulfadimidine<br>(Sulfamethazine,<br>Sulfadimerazine)<br>Sulfadoxine<br>Sulfafurazole<br>Sulfaguanidine<br>Sulfamerazine<br>Sulfadimethoxazole<br>Sulfamethoxin<br>Sulfamonomethoxin<br>Sulfanilamide<br>Sulfapyridine<br>Phthalylsulfathiazole<br>Sulfaquinoxaline	AVI, BOV, SUI<br>AVI, BOV,<br>CAP, OVI, SUI<br>AVI, BOV,<br>CAP, EQU,<br>LEP, OVI, PIS,<br>SUI AVI, BOV,<br>CAP, EQU,<br>LEP, OVI, SUI<br>BOV, EQU,<br>OVI, SUI BOV,<br>PIS AVI, CAP,<br>OVI AVI, BOV,<br>CAP, EQU,<br>LEP, OVI, PIS,<br>SUI<br>AVI, BOV, SUI<br>AVI, PIS, SUI<br>AVI, PIS, SUI<br>AVI, BOV,<br>CAP, OVI<br>BOV, SUI SUI<br>AVI, BOV,<br>CAP, LEP, OVI	Sulfonamides are extremely important in veterinary medicine because of the diversity of their use and the nature of the diseases treated with them. These classes, administered alone or in combination, are critically important in the treatment of a wide variety of diseases (bacterial, coccidial and protozoan infections) in many animal species.	X		
SULFONAMIDES +<br>DIAMINOPYRIMIDINES<br>Sulfamethoxypyridazine<br>Ormetoprim+<br>Sulfadimethoxine<br>Trimethoprim+ Sulfonamide	AVI, BOV, EQU,<br>SUI PIS AVI,<br>BOV, CAP,<br>EQU, LEP, OVI,<br>PIS, SUI				
DIAMINOPYRIMIDINES<br>Baquiloprim<br>Trimethoprim<br>Ormetoprim	BOV, SUI AVI,<br>BOV, CAP,<br>EQU, LEP, OVI,<br>SUI AVI				
STREPTOGRAMINS<br>Virginiamycin	AVI, BOV, OVI,<br>SUI	Virginiamycin is an important antimicrobial agent in the prevention of necrotic enteritis (Clostridium perfringens).			X
TRACYCLINES<br>Chlortetracycline	AVI, BOV, CAP,<br>EQU, LEP, OVI,	Tetracyclines are extremely important in veterinary medicine	X		

Doxycycline<br>Oxytetracycline<br>Tetracycline	SUI AVI, BOV, CAM, CAP, EQU, LEP, OVI, PIS, SUI<br>API, AVI, BOV, CAM, CAP, EQU, LEP, OVI, PIS, SUI API, AVI, BOV, CAM, CAP, EQU, LEP, OVI, PIS, SUI	because of the diversity of their use and the nature of the diseases treated with them. This class is critically important in the treatment of a wide variety of bacterial and chlamydial diseases in many animal species. This class is also critical in the treatment of cowdriosis (Ehrlichia ruminantium) and anaplasmosis (Anaplasma marginale) in animals due to the lack of alternatives.			
THIOSTREPTON<br>Nosiheptide	AVI, SUI	This class is currently used in the treatment of certain dermatological conditions.			X

OIE standards, guidelines and resolution on antimicrobial resistance and use of antimicrobial agents

ANNEX 7: SUMMARY OF THE MAIN FAMILIES OF ANTIBIOTICS USED IN ANIMAL HEALTH

Family	Example	Type of activity
BETA-LACTAM ANTIBIOTICS	Amoxicillin / Penicillin V / Ampicillin	Bactericide on proliferating bacteria
AMINOSIDES	Streptomycin / Gentamycin / Neomycin	Bactericide on proliferating and resting bacteria
MACROLIDES	Erythromycin / Tylosin / Spiramycin	Bacteriostatic on proliferating bacteria
LINCOSAMID	Lincomycin	Bacteriostatic on proliferating bacteria
PHENICOLES	Chloramphenicolous / Florophenicolous	Bacteriostatic on proliferating bacteria
TRACYCLINES<br><br>SULFAMIDES	Oxytetracycline / Chlortetracycline / Doxycycline<br><br>Sulfonamides / Thrimethroprime	Bacteriostatic on proliferating bacteria<br><br>Bacteriostatic on proliferating bacteria

NITROFURANES	Furazolidone / Furaldatone	Bactericide/bacteriostatic on proliferating and resting bacteria
POLYMYXINS	Colistin	Bactericide on bacteria at rest
QUINOLONES	Fluoroquinolones / Flumequine / Difloxacin	Bactericide on proliferating and resting bacteria

APPENDIX 8: SOME EXAMPLES OF ANTIBIOTIC PRESCRIPTIONS IN ANIMAL HEALTH

Name	Example	Indication	Dosage (as an indication)	Administration
TRACYCLINES	Tenalin la (Oxytetracycline 20% long acting)	Broad spectrum, sepsis, respiratory, digestive, genito-urinary infections, panariasis	1 ml / 10 kg	Intramuscular injection that can be repeated 72 hours later
	Oxytetracycline 50% virbac (Soluble oral powder for calves, pigs and poultry)	Sepsis, respiratory and digestive infections	400 mg powder / 10 kg	In milk or drinking water for 3 to 5 days
	Covunil (Oxytetracycline combined with flunixin (NSAID)	Respiratory infections with fever	1 ml / 10 kg	Deep intramuscular, single injection
PENICILLINS (alone or in combination)	Duphamox la (Amoxicillin retard)	Gram+ respiratory infections, pasteurellae	1 ml / 10 kg	Intramuscular, 2 injections 48 hours apart
	Shotapen (Benzylpenicillin and Dihydrostreptomycin)	Sepsis, pneumonia, pleuropneumonia, postpartum infections, urinary tract infections, infected wounds, panariasis	1 ml / 10 kg	Intramuscular to be repeated 72 H later if necessary

FLORFENICOL	Nuflor (300 mg or 450 mg)	Respiratory System Conditions	Cattle: 1 mL / 15 kg Cattle: 2 mL / 15 kg Sheep: 1 mL / 15 kg	Intramuscular 2 times at 48H intervals Subcutaneous 1 single injection Intramuscular 3 successive days

APPENDIX 9: SOME EXAMPLES OF DEWORMING PRESCRIPTIONS IN ANIMAL HEALTH

Name	Example	Indication	Dosage (as an indication)	Administration
ALBENDAZOLE (10% liquid or bolus form)	Liquid: Vermitan, Worminex, Albenol, Dolzaben, Valbazen	Intestinal worms Liver fluke	1 ml / 10 kg	With a dosing gun or syringe
	Bolus: Vermitan, Valbazen, Benzal, Worminex	Intestinal worms Lungworms Liver fluke	10 mg / 10 kg	With a bolus launcher or by hand
OXFENDAZOLE	Synanthic	Intestinal worms Lungworms	Drinkable suspension at 22,65mg/ml : Sheep : 2,2 mL / 10 kg or 5 mg / kg Goats : 5mL / 10 kg or 10 mg / kg Cattle : 2 mL / 10 kg or 4,5 mg / kg Drinkable suspension at 90,60 mg / mL : 5 mL / 100 kg or 4,5 mg / kg	By mouth with a syringe or a dosing gun

FENBENDAZOLE (Available as bolus, powder or oral suspension in different concentrations)	Penacur	Intestinal worms Lungworms	Goats: 10 mg / kg Sheep: 5 to 10 mg / kg Cattle: 7.5 mg / kg	By mouth with a syringe or a dosing gun By hand or with a bolus dispenser Mixed with the food for the powder form
LEVAMISOLE (Available as bolus, powder, drinkable)	Levamisole 5%, Polystrongle	Intestinal worms Lungworms	Cattle, sheep, pigs: 7.5 mg / kg Poultry: 20 mg / kg	By mouth with a syringe or a dosing gun By hand or with a bolus dispenser Mixed with the food for the powder form
NITROXINIL	Dovenix	Liver fluke and nematodes	Cattle, sheep: 10 mg / kg or 1 mL / 25 kg	Subcutaneous injection

APPENDIX 10: SOME EXAMPLES OF ENDECTOCIDE PRESCRIPTIONS IN ANIMAL HEALTH

Name	Example	Indication	Dosage (as an indication)	Administration
IVERMECTIN	Ivomec injectable and for-on Virbamec injectable and for-on	Nematodes Diptera larvae Lice Scabies	Injectable: 1 mL / 50 kg Pour-on: 1 mL / 10 kg	Strict subcutaneous injection On the line of the back
DORAMECTIN	Dectomax injection and for-on	Nematodes Diptera larvae Lice Scabies	Injectable: 1 ml / 50 kg Pour-on: 1 ml / 10 kg	Strict subcutaneous injection on the line of the back
EPRINOMECTIN	Eprinex for-on	Nematodes Diptera larvae Lice Scabies	Pour-on: 1 ml / 10 kg	On the back line

APPENDIX 11: SOME EXAMPLES OF EXTERNAL PEST CONTROL PRESCRIPTIONS IN ANIMAL HEALTH

Name	Example	Indication	Dosage (as an indication)	Administration
AMITRAZE	Taktic	Ticks & Lice (One spray) Scabies (Two sprays 9 days apart)	Cattle: Dilute 2 mL per 1 L of water Sheep, goats, pigs: Dilute 4 mL per 1 L of water	Sprayer (Do not empty into water or rivers, destroy empty containers. Do not use near people eating or drinking)

APPENDIX 12: SOME EXAMPLES OF BLOOD ANTIPARASITIC PRESCRIPTIONS IN ANIMAL HEALTH

Name	Example	Indication	Dosage (as an indication)	Administration
TRACYCLINES	Tenalin la Oxytetracycline 20% long acting	Cowdriosis	1 ml / 10 kg 20 mg / kg	Intramuscular injection that can be repeated 72 hours later
	Bolus Vermitan, Valbazen, Benzal, Worminex	Cowdriosis	400 mg powder / 10 kg	In milk or drinking water for 3 to 5 days
IMIDOCARBE	Carbesia	Babesiosis Anaplasmosis	1 ml / 100 kg 2.5 mg / kg	Deep intramuscular or subcutaneous, single injection
BUPARVAQUONE	Butaline	Theileriosis	1 ml / 20 kg 2.5 mg / kg	Intramuscular, 2 injections at 48 hours

ISOMETADINIUM	Trypamidium Veridium	Trypanosomiasis	10 ml / 100 kg 1 mg / kg	Intramuscular or strict intravenous, single injection
DIMINAZENE	Veriben Berenil	Trypanosomiasis Babesiosis Theileriosis	5 ml / 100 kg 3.5 mg / kg	Intramuscular, single injection

APPENDIX 13: SOME EXAMPLES OF ANTI-INFLAMMATORY PRESCRIPTIONS IN ANIMAL HEALTH

Name	Example	Indication	Dosage (as an indication)	Administration
CORTICOIDS	Dexadreson Dexamethasone	Fever, inflammatory state, allergy, shock, ketosis, induction of parturition	3 ml / 100 kg Calving Induction: Cattle: 10 ml Caprine: 6 to 8 ml	Intravenous Intramuscular Subcutaneous Intra-articular
MELOXICAM	Meloxidyl 20 mg / ml	Treatment of inflammatory and/or painful symptoms, usually in combination with antibiotics	Cattle: 2.5 ml / 100 kg	Subcutaneous
FLUNIXINE	Genixin	Treatment of inflammatory and/or painful symptoms, usually in combination with antibiotics	Cattle: 2 ml / 50 kg	Intramuscular

ANNEX 14: MAIN DISEASES ENCOUNTERED IN MALI AND THE VACCINES USED TO PREVENT THEM

Disease	Vaccine
Bacterial coal	ANTHRAVAC 1 ml

Symptomatic charcoal	CLOSTRIVAC 2 ml
PPCB	PERI-TI 1 ml
PPR	SR PESTEVAC 2 ml
Bovine Pasteurellosis	PASTOBOV 1 ml
Ovine Pasteurellosis	PASTOVIN 2 ml
Newcastle disease	ITA-NEW 1 ml
Avian flu	ITA-FLU 0,5 ml
