# Supplementary material for: Landscape Analysis of Existing Policies, Laws and Regulations on Antimicrobial Stewardship in 10 Low- and Middle-Income Countries
Source: Antibiotics (Basel). 2026 Aug 10;15(8):770. doi: 10.3390/antibiotics15080770 (PMC13510074; doi:10.3390/antibiotics15080770)
Supplement: Supplementary file 1 [file antibiotics-15-00770-s001.zip › S3 Country Reports/S3F-1_Mozambique Human Health).pdf]

USAID MEDICINES, TECHNOLOGIES, AND PHARMACEUTICAL SERVICES (MTaPS) PROGRAM

Improved Access. Improved Services. Better Health Outcomes.

Rapid Assessment of current antimicrobial stewardship policies, legislation, regulations, and guidelines for human health in Mozambique

Mozambique

August 2022

USAID
FROM THE AMERICAN PEOPLE

This document is made possible by the generous support of the American people through the US Agency for International Development (USAID) contract no. 7200AA18C00074. The contents are the responsibility of Management Sciences for Health and do not necessarily reflect the views of USAID or the United States Government

Recommended Citation

Tomas Zimba. *Rapid Assessment of current Antimicrobial Stewardship policies, legislation, regulations, and guidelines for Human health in Mozambique*. August 2022. Prepared for the US Agency for International Development by the USAID Medicines, Technologies, and Pharmaceutical Services (MTaPS) Program. Arlington, VA: Management Sciences for Health, Inc.

About the USAID MTaPS Program

The USAID Medicines, Technologies, and Pharmaceutical Services (MTaPS) Program enables low- and middle-income countries to strengthen their pharmaceutical systems, which is pivotal to higher-performing health systems. MTaPS focuses on improving access to essential medical products and related services and on the appropriate use of medicines to ensure better health outcomes for all populations. The program brings expertise honed over decades of seminal pharmaceutical systems experience across more than 40 countries. The MTaPS approach builds sustainable gains in countries by including all actors in health care—government, civil society, the private sector, and academia. The program is implemented by a consortium of global and local partners and led by Management Sciences for Health (MSH), a global health nonprofit.

The MTaPS Consortium

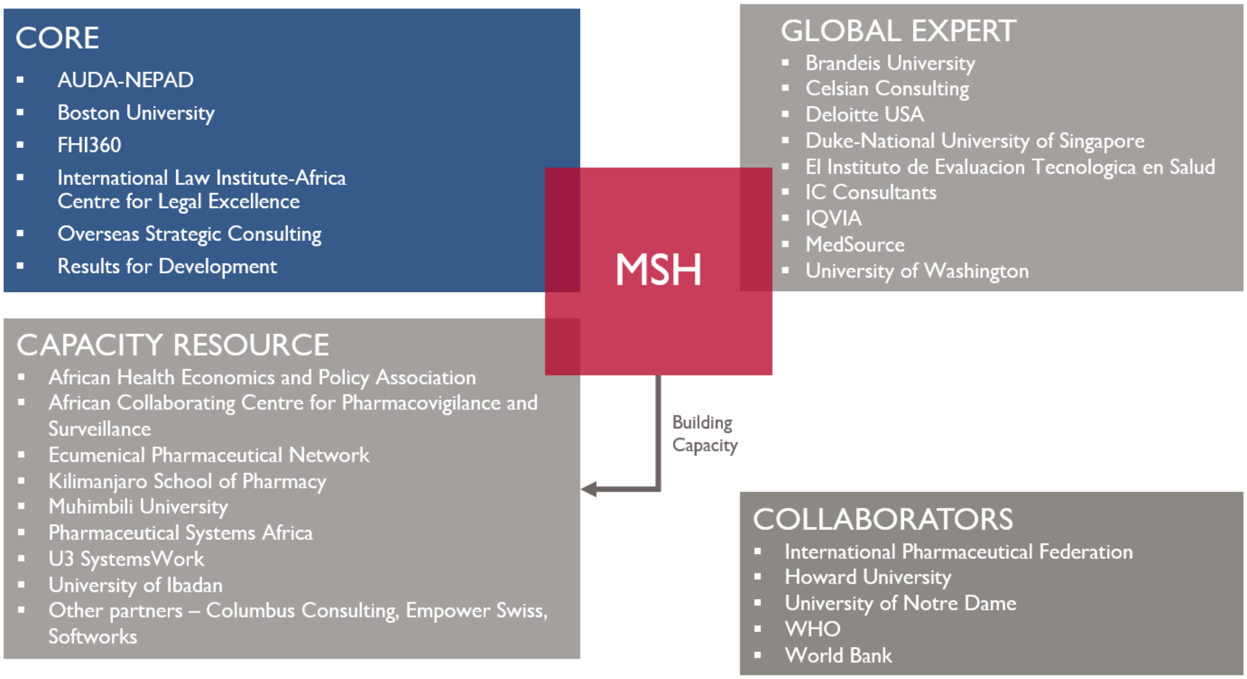

This report/document is submitted by:

Tomas Zimba,

Senior Technical Advisor - AMS,

Medicines, Technologies, and Pharmaceutical Services (MTaPS) Program,

Maputo, Mozambique.

Email: tzimba@mtapsprogram.org

ACRONYMS AND ABBREVIATIONS

AMR	antimicrobial resistance
AMS	antimicrobial stewardship
ANARME	*Autoridade Nacional Reguladora de Medicamentos de Moçambique* (national medicines regulatory authority)
AWaRe	Access, Watch, and Reserve
CMAM	*Central de Medicamentos e Artigos Médicos* (Center for Drugs and Medical Supplies)
COMED	*Conselho do Medicamentos* (Medicines Council)
CTTF	*Comissão Técnica de Terapêutica e Farmácia* (Pharmacy and Therapeutics committee)
DNAM	*Direcção Nacional de Assistência Médica* (National Directorate of Medical Assistance)
DNF	*Direcção Nacional de Farmácia* (National Directorate of Pharmacy)
DPS	*Direcção Provincial de Saúde* (Provincial Directorate of Health)
DTH	*Departamento de Terapêutica Hospitalar* (Department of Hospital Therapeutics)
FAO	Food and Agriculture Organization
FNM	*Formulário nacional de Medicamentos* (National Medicines Formulary)
GARP	Global Antibiotic Resistance Partnership
GHI	General Inspectorate of Health
GRM	Government of the Republic of Mozambique
IHR	International Health Regulations
IPC	infection prevention and control
JEE	Joint External Evaluation
LNCQM	*Laboratório Nacional de Controle de Qualidade* (National Quality Control Laboratory)
MA	marketing authorization
MASA	*Ministério da Agricultura e Segurança Alimenta* (Minister of Agriculture and Food Security)
MEDIMOC	*Empresa de Importação e Exportação de Medicamentos* (Medicines Import and Export Company)

MISAU	*Ministério da Saúde* (Ministry of Health)
MSH	Management Sciences for Health
MT	metical (Mozambican currency)
MTaPS	Medicines, Technologies, and Pharmaceutical Services
NAP	National Action Plan
NHS	National Health Service
NLEM	*Lista Nacional dos Medicamentos Essenciais* (National List of Essential Medicines)
NRA	national regulatory authority
OIE	World Organization for Animal Health
PESS	*Plano Estratégico do Sector da Saúde* (Health Sector Strategic Plan)
PMS	post-marketing surveillance
PNASS	Plano Nacional de Acção para Segurança Sanitária (National Health Security Plan)
POM	prescription-only medicine
PV	pharmacovigilance
USAID	US Agency for International Development
WHO	World Health Organization

CONTENTS

Acronyms and Abbreviations i

Project Summary iv

Executive Summary 1
- Introduction and background 1
- Objective 2
- Methodology 2
- Findings 2
- Recommendations 4

1. Introduction 4

2. Background 6

3. Objective 7

4. Methodology 7

5. Findings 7
   - 5.1 Right to Health care 8
   - 5.2 Policies and general health care system 8
   - 5.3 The Medicines Law and Regulation of Medicines 10
   - 5.4 Product registration/Marketing Authorization 12
   - 5.5 Pharmaceutical practice 13
   - 5.6 Licensing and Inspection 13
   - 5.7 Supply chain Management Policies and Guidelines relevant to AMS 14
   - 5.8 Prescribing, Dispensing, and Use of Medicines 15
   - 5.9 Post-market surveillance and Pharmacovigilance 17

6. Discussion 18

7. Recommendations 19

8. Conclusion 20

Appendix 1. List of Policies, Laws, Regulations, and Guidelines assessed 21

Appendix 2. List of Regulations and guidelines whose development/review has been supported by MTaPS 22

PROJECT SUMMARY

**Program Name:**		USAID Medicines, Technologies, and Pharmaceutical Services (MTaPS) Program
**Activity Start Date and End Date:**		September 20, 2018–September 19, 2023
**Name of Prime Implementing Partner:**		Management Sciences for Health
**Contract Number:**		7200AA18C00074
**MTaPS Partners**	**Core Partners**	Boston University, FHI 360, Overseas Strategic Consulting, Results for Development, International Law Institute-Africa Centre for Legal Excellence, NEPAD
	**Global Expert Partners**	Brandeis University, Deloitte USA, Duke-National University of Singapore, El Instituto de Evaluacion Technologica en Salud, IC Consultants, Imperial Health Sciences, MedSource, QuintilesIMS, University of Washington
	**Capacity Resource Partners**	African Health Economics and Policy Association, Ecumenical Pharmaceutical Network, U3 SystemsWork, University of Ibadan, University of Ghana's World Health Organizations (WHO) Pharmacovigilance Collaborating Center, Kilimanjaro School of Pharmacy, Muhimbili University, Pharmaceutical Systems Africa
	**Collaborators**	International Pharmaceutical Federation, Howard University, University of Notre Dame, WHO, World Bank

EXECUTIVE SUMMARY

INTRODUCTION AND BACKGROUND

Antimicrobial resistance (AMR) is a global threat to public health. Increased use of antibiotics in humans and animals has led to the development of resistance, reducing the effectiveness of the drugs against common microbial infections. The excessive and inappropriate use of antimicrobials in human and animal health and food production are the main drivers of AMR.[1]

The 2015 Global Antibiotic Resistance Partnership (GARP) situational analysis of antibiotic resistance and use in Mozambique pointed out AMR as real threat to the country's population and recommended, as a priority action, the development and implementation of a national strategic plan for AMR that would help the country preserve the effectiveness of antibiotics while ensuring their effective use against disease.[2] To align to international action against AMR, this national action plan (NAP) against AMR would need to be aligned with the Global Action Plan on AMR developed by the World Health Organization (WHO).[3] The WHO is implementing the Global Action Plan in collaboration with the Food and Agriculture Organization (FAO) and the World Organization for Animal Health (OIE) for a One Health approach. The Mozambican NAP-AMR 2019–2023 was developed and approved in 2018 by the Minister of Health and the Minister of Agriculture.[4] It contains six strategic objectives under the following three pillars: prevention of AMR, access to effective antibiotics, and rational and appropriate use of antibiotics.

In 2016 Mozambique underwent the WHO Joint External Evaluation (JEE), the results of which prompted the development of the National Health Security Plan (*Plano Nacional de Acção para Segurança Sanitária*; PNASS), which addresses all threats against human health, animal health, and the environment, while the NAP was designed to fight against AMR and promote its containment.[5] In the 2016 JEE, the country received a baseline rating of "no capacity" (score 1) for antimicrobial stewardship (AMS) and "developed capacity" (score 3) for health care–associated infection prevention and control (IPC) programs.

The 2015 WHO Global Action Plan had, as one of its strategic objectives, to optimize the use of antimicrobial medicines in human and animal health, through actions such as development and implementation of effective AMS programs in human and animal health. AMS is one of the priority actions under the NAP-AMR strategic objectives 5 (optimize the use of antibiotics in human and animal health) and 6 (change incentives that encourage the overuse of antibiotics; and enforce rational use). The WHO Benchmarks for International Health Regulations (IHR) capacities recommends the following action under benchmark 3.4 toward achieving capacity level 2: to "undertake an assessment of

---

[1] The Review on Antimicrobial Resistance, chaired by Jim O'Neill. December 2014. Antimicrobial Resistance: Tackling a crisis for the health and wealth of nations. Wellcome Trust and the United Kingdom Government.
[2] Global Antibiotic Resistance Partnership (GARP)—Mozambique Working Group. 2015. Situation Analysis and Recommendations: Antibiotic Use and Resistance in Mozambique. Washington, DC and New Delhi: Center for Disease Dynamics, Economics & Policy.
[3] World Health Organization. 2015. Global Action Plan on Antimicrobial Resistance. ISBN 978 92 4 150976 3
[4] MISAU and Minister of Agriculture and Food Security (MASA). December 2018. National Action Plan against Antimicrobial Resistance: Mozambique, 2019–2023.
[5] WHO. 2017. Joint External Evaluation of IHR Core Capacities of Republic of Mozambique. Geneva: WHO. Licence: CC BY-NC-SA 3.0 IGO

stewardship policies and activities, including regulatory framework and supply chain management of antimicrobials, using a multisectoral approach."[6]

In supporting the Government of Mozambique (GRM)'s efforts to increase national AMS capacity, the US Agency for International Development (USAID) Medicines, Technologies, and Pharmaceutical Services (MTaPS) Program, implemented by Management Sciences for Health (MSH), provided technical support in the rapid assessment of AMS policies, laws, regulations, and guidelines in the human health sector.

OBJECTIVE

The purpose of this rapid assessment was to evaluate the existing regulatory framework of AMS policies, laws, regulations, and guidelines, and the supply chain management and use of antimicrobial medicines in Mozambique; identify gaps; and make recommendations for action in the human health sector. This report presents the findings of the assessment of AMS policies and regulatory framework, supply management, and antimicrobial use in the human health sector. The assessment of the existing regulatory framework and supply chain management in the animal health sector was conducted by FAO and therefore is excluded from this report. Of note, however, it is planned that with support from the national AMS technical working group (TWG) of the AMR multisectoral coordination committee, this human health report will be merged with the animal health sector report prepared by FAO.

METHODOLOGY

A literature review of the available Mozambican legislation, regulations, and guidelines related to human medicine, AMR, antimicrobial medicines, and AMS was performed by the MTaPS Mozambique team in collaboration with the Ministry of Health (MISAU). The MISAU website was visited to download the available publications related to pharmacy and the legal framework. The downloaded documents were categorized (law, policy, regulation, and guideline), and then subjected to a desk review. To complement the internet search, MTaPS also used a previously prepared report of the technical review of the pharmaceutical legal and regulatory system in Mozambique in 2019.[7] In addition, the WHO Benchmarks for IHR capacities and the Mozambique JEE report were used as references for this assessment. Interviews of key informants selected from various stakeholders were undertaken for validation of the desk review findings, to uncover any gaps and to include their recommendations to address the gaps.

FINDINGS

The human health system of Mozambique is based on the constitution and the Law 2/77 of September 27, 1977, which ensures health care for all regardless of their financial and social status. Mozambique has a fundamental law of medicines issued for the first time on January 14, 1998 (Law 4/98 of January 14, 1998), which was updated into the medicines law 12/2017 of September 8, 2017. The citizen of Mozambique is guaranteed the right to health care in the 2004 Constitution.[8] Article 116 of the Constitution notes that the State is responsible for overseeing the production, sale and use of "chemical,

[6] WHO Benchmarks for International Health Regulations (IHR) capacities, 2019. https://apps.who.int/iris/bitstream/handle/10665/311158/9789241515429-eng.pdf?sequence=1
[7] MTaPS. May 2019. Technical Review of the Pharmaceutical Legal and Regulatory System in Mozambique.
[8] 2004 Constitution of Mozambique.

biological, and pharmaceutical products and other forms of treatment and diagnosis," and requires that health care provided by public and private entities be conducted in accordance with the law.

Mozambique does not have a consolidated health policy and documents such as the health sector strategic plan comprising its health policy framework lack mention of AMR or AMS. The country also lacks an updated national pharmaceutical policy. While the medicines Law 4/98 and its revised format 12/2017 are the backbone of medicine legislation and regulation in Mozambique, these two documents address marketing, research, registration, production, quality control, import, export, storage, distribution, transport, prescribing and dispensing, and drug pricing regime, among other items, but do not mention AMR or AMS.

Mozambique has policies, legislation, regulations, and guidelines for medicines in general since the early 1990s, but AMS and AMR were not specifically mentioned or provided for in the available and reviewed policy, legal, regulation, and guidance documents.

Although there are national standard treatment guidelines currently available for vertical programs such as malaria, tuberculosis, and sexually transmitted infections, as well as for pediatric, surgical, and obstetrics and gynecology care, their review is not regular, and standard treatment guidelines for other areas of human health, such as internal medicine and intensive care, are lacking. The existing national list of essential medicines (NLEM) and national medicines formulary (FNM) do not include the WHO Access, Watch, and Reserve (AWaRe) classification of antibiotics. Available laws address marketing and dispensing issues, but they do not mention AMS. The implementation of controls in the importation of medicines in Mozambique is performed by the national medicines regulatory authority (*Autoridade Nacional Reguladora de Medicamentos de Moçambique*; ANARME) at the country's points of entry.

Assessment of a broader range of products including antimicrobials is required for effective post-marketing surveillance (PMS). While the national pharmacovigilance (PV) system objectives address some aspects to AMS, these aspects are applicable to medicines in general and do not mention antimicrobial medicines, hence, there is a need for specific AMS related content in the PV-related legislation, regulation, and guidelines. There was no specific regulation on prescription-only sales of key antibiotics found in this desk review. There is also no regulation addressing pre-importation quality verification to deter importation of substandard and falsified or fake antimicrobials, leaving the market open to importation and marketing of non-efficacious antimicrobials.

There is poor enforcement in private pharmacies of prohibition of over-the-counter sales of antibiotics without a prescription. The country's legal instruments do not clearly state the mechanisms through which ANARME can monitor the implementation of the GRM's AMR and AMS interventions in private pharmaceutical premises.

RECOMMENDATIONS

It is recommended that a specific regulatory framework on AMS to address the identified gaps be put in place urgently. The following are additional recommendations:

- The Pharmacy and Therapeutics Committee (*Comissão Técnica de Terapêutica e Farmácia*; CTTF), ANARME, and the Department of Hospital Therapeutics (*Departamento de Terapêutica Hospitalar*; DTH) should update the NLEM and FNM to adhere to the WHO AWaRe classification.
- Beyond the guidelines for management of the vertical program for infectious diseases such as tuberculosis, HIV, and sexually transmitted diseases, the DTH should develop national treatment guidelines for the main infectious diseases, such as respiratory infections, diarrhea, and urinary tract infections.
- ANARME should develop a policy to monitor AMR and AMS interventions in the private sector.
- ANARME should develop or improve legislation to specifically address or include AMS, in line with WHO IHR benchmark 3.4 capacity level 2 action, which recommends development of legislation that regulates the use, availability, and quality of antimicrobials.
- ANARME and the DTH should develop specific regulations and guidelines for antimicrobials and AMS. Recommendations include a regulatory provision on appropriate use of antimicrobials in the existing regulatory framework for medicines and a draft regulation on prescription-only sales of key antibiotics—with the recommended capacity level 3 action of the WHO benchmark 3.4.
- ANARME, the DTH, and the national AMS TWG should develop national AMS guidelines for the AMS program, and a national action plan or strategy for AMS that includes interventions to address the identified gaps—in line with the recommended capacity level 2 action of the WHO benchmark 3.4.

1. INTRODUCTION

AMR is a global threat that requires urgent collaborative action within and among countries. AMR occurs when a microorganism develops resistance to an antimicrobial medicine to which it was previously sensitive, making standard treatments ineffective and facilitating the spread of drug-resistant infections, thus rendering the community vulnerable. Increased use of antibiotics or antimicrobial medicines in humans and animals has led to the development of resistance, reducing the effectiveness of the drugs against common microbial infections. The excessive and inappropriate use of antimicrobial agents across human, animal, and agriculture sectors are among the main drivers of AMR globally. AMR affects the management of bacterial infections in industrialized as well as in low- and middle-income countries.[9] The major driver for AMR in developing countries is the use of antibiotics in human medicine, veterinary medicine, and agriculture in addition to poor implementation of IPC.[10] AMR is associated with increased mortality, length of hospital stays, cost and readmittance to the hospital.[11] The increasing global prevalence of AMR is recognized as one of the most pressing challenges facing humanity over the coming decades, with effective treatments against infectious diseases at risk of becoming increasingly unavailable. If left unchecked, by 2050, up to 10 million people could die each year due to

[9] WHO. 2014. Antimicrobial resistance: global report on surveillance.
[10] Okeke IN, Laxminarayan R, Bhutta Z a, Duse AG, Jenkins P, O'Brien TF, et al. Antimicrobial resistance in developing countries. Part I: recent trends and current status. Lancet Infect Dis. 2005;5(August):481–93.
[11] Metlay JP, Singer DE. Outcomes in lower respiratory tract infections and the impact of antimicrobial drug resistance. Clin Microbiol Infect. 2002;8(SUPPL. 2):1–11.

AMR, 90% of them from developing countries.[1] Therefore, the WHO declared AMR to be among the top 10 global public health threats facing humanity.

A strong linkage between antibiotic consumption and the emergence of AMR has been demonstrated in European countries and United States of America.[12] The increased use of antimicrobial medicines has resulted in emergence of resistant bacterial strains and their rapid dissemination globally. Given that they are essential for treatment of bacterial infections there is a need to discourage the misuse and overuse of antimicrobial medicines. High proportions of extended spectrum beta lactamase producing *E. coli* and *K. pneumonia* as well as methicillin-resistant *Staphylococcus aureus* have been reported globally, and African countries including Mozambique are no exceptions.[13,14] Diarrheal disease, respiratory infections, and tuberculosis are most common infections in Mozambique, in addition to national HIV/AIDS prevalence of 11.5% and endemic malaria.[15] These infections are mainly treatable with antimicrobial medicines, given the lack of diagnostics for infections such as malaria and tuberculosis.[16] Antimicrobial susceptibility testing has revealed growing resistance to commonly used antibiotics for various common infectious diseases in Mozambique.[2]

In 2015, the WHO released its Global Action Plan on AMR, with one of its strategic objectives being to optimize the use of antimicrobial medicines in human and animal health, through actions such as development and implementation of effective AMS programs in the human and animal health sectors.[3] The WHO is implementing the Global Action Plan in collaboration with FAO and OIE for a One Health approach. The WHO also released its Benchmarks for IHR capacities (2019), which has suggested actions needed to improve in-country IHR capacities or health security. These actions will help countries to assess and improve their status toward sustainable capacity for each benchmark.

AMS refers to coordinated efforts and activities that seek to measure and improve the use of antimicrobials. To help map the existing situation in AMS and inform improvement measures, the WHO Benchmarks tool recommends the following action under benchmark 3.4 toward achieving capacity level 2: to "undertake an assessment of stewardship policies and activities, including regulatory framework and supply chain management of antimicrobials, using a multisectoral approach." Implementation of AMS programs have demonstrated positive public health and clinical impacts—including a reduction in costs, length of hospital stays, and the burden of AMR—while maintaining or improving patient outcomes.

To address the major component of the IHRs related to containing AMR and to guide countries' implementation of AMS programs, the WHO released recommendations to help global, regional, and national policymakers develop guidelines on which antibiotics to use and when, including the AWaRe categorization of antibiotics.[17] The AWaRe tool is meant to be applied to a country's essential medicines

---

[12] Seale AC, Gordon NC, Islam J, Peacock SJ, Scott JAG. AMR surveillance in low and middle-income settings—A roadmap for participation in the Global Antimicrobial Surveillance System (GLASS). Wellcome Open Res. 2017;2(0):1–17.
[13] Leopold SJ, van Leth F, Tarekegn H, Schultsz C. Antimicrobial drug resistance among clinically relevant bacterial isolates in sub-Saharan Africa: a systematic review. J Antimicrob Chemother. 2014;69:2337–53
[14] WHO. 2018. WHO report on surveillance of antibiotic consumption: 2016–2018 early implementation.
[15] Galatas et al., 2020. Global health. Demographic and health community–based surveys to inform a malaria elimination project in Magude district, southern Mozambique.
[16] Tadesse BT, Ashley EA, Ongarello S, Havumaki J, Wijegoonewardena M, González IJ, et al. Antimicrobial resistance in Africa: a systematic review. BMC Infect Dis. 2017;17(1):616.
[17] WHO. 2021. Access, Watch, Reserve (AWaRe) classification of antibiotics for evaluation and monitoring of use. Available from https://www.who.int/publications/i/item/2021-aware-classification

list and/or standard treatment guidelines, grouping the most effective antibiotics into three groups: Access, Watch, and Reserve. The tool aims to improve access (through the Access antibiotics), monitor important antibiotics (Watch), and preserve effectiveness of the "last resort" antibiotics (Reserve). The WHO recommends for countries to strive to have at least 60% of the national consumption of antibiotics come from the Access category by 2023 to support higher consumption of narrow-spectrum antibiotics, thereby sparing the broader-spectrum Watch and Reserve antibiotics from any emerging AMR, resulting in better use of antibiotics, mitigation of AMR, reduced health care costs, and increased access. AWaRe classification is an essential policy tool to complement interventions in hospital AMS activities.

2. BACKGROUND

The 2015 GARP situational analysis of antibiotic resistance and use in Mozambique pointed out AMR as real threat to the country's population and as a priority action, recommended the development and implementation of a national strategic plan for AMR that would help the country preserve the effectiveness of antibiotics while ensuring their effective use against disease.[2] To contribute to international action against AMR, this NAP against AMR would need to be aligned with the Global Action Plan on AMR. The Mozambican NAP-AMR 2019–2023 was developed and approved in 2018 by the Minister of Health and the Minister of Agriculture.[4] It contains six strategic objectives within three pillars: prevention of AMR, access to effective antibiotics, and rational and appropriate use of antibiotics, all using the One Health approach.

In April 2016, Mozambique underwent the WHO JEE, the results of which prompted the development of the PNASS, which addresses all threats against human health, animal health, and the environment, while the NAP was designed to fight against AMR and promote antimicrobial containment.[5] In the 2016 JEE, the country received a baseline rating of "no capacity" (score 1) for AMS and "developed capacity" (score 3) for health care–associated IPC programs.

AMS is one of the priority actions under the NAP-AMR strategic objectives 5 (optimize the use of antibiotics in human and animal health) and 6 (change incentives that encourage the overuse of antibiotics; and enforce rational use).

The MTaPS Program, implemented by MSH, has provided technical support in pharmaceutical systems strengthening, especially in the strengthening of the regulatory framework to the National Directorate of Pharmacy (*Direcção Nacional de Farmácia*; DNF), and later, to ANARME. Efforts to establish regulations to operationalize the Medicines Law 12/2017 were undertaken by the DNF with support from MTaPS, for example, a workshop on the medicines law and regulation of medicines, vaccines, and other biological products was held from November 25 to December 6, 2019, where MTaPS supported the DNF to draft 10 documents: 6 regulations, 3 guidelines, and 1 list of non-prescription/over-the-counter medicines.

In supporting the GRM's efforts to increase national AMS capacity, MTaPS provided technical support in the rapid assessment of AMS policies, laws, regulations, and guidelines in the human health sector.

3. OBJECTIVE

The purpose of this rapid assessment was to evaluate the existing regulatory framework of AMS policies, laws, regulations, and guidelines, and the supply chain management and use of antimicrobial medicines in Mozambique, and to identify gaps and make recommendations for action in the human health sector. This report presents the findings of the assessment of AMS policies and regulatory framework, supply management, and use in the human health sector.

The assessment of the existing regulatory framework and supply chain management in the animal health sector was conducted by FAO and therefore is excluded from this report. Of note, it is planned that with support from the national AMS TWG of the AMR multisectoral coordination committee, this human health report will be merged with the animal health sector report prepared by FAO.

4. METHODOLOGY

Literature review of the available Mozambican legislation, regulations, and guidelines related to human medicine, AMR, antimicrobial medicines, and AMS was performed by the MTaPS/Mozambique team in collaboration with MISAU. The team visited the MISAU website to download the available publications related to pharmacy and the legal framework. The downloaded documents were categorized (law, policy, regulation, and guideline), and then subjected to a comprehensive desk review. To complement the internet search, MTaPS also used a previously prepared report of the technical review of the pharmaceutical legal and regulatory system in Mozambique in 2019.[7] In addition, it used the WHO Benchmarks for IHR capacities and the Mozambique JEE report as references for this assessment.

In addition, MTaPS developed a questionnaire and checklist for documenting the current provisions for AMS policies and regulations. These were used to conduct interviews of key informants selected from various stakeholders for validation of the desk review findings, to uncover any gaps, and to include informants' recommendations for addressing them.

The findings and recommendations in this report will be valuable in informing the development of a national action plan for AMS, and the development or review of relevant legislation and regulatory framework to guide the use, availability, and quality of antimicrobials as recommend by the WHO Benchmarks tool under benchmark 3.4.

5. FINDINGS

A list of the documents reviewed under the rapid assessment of AMS policies and activities, including regulatory framework of the human health sector, is provided in appendix 1.

This rapid assessment defines laws as the system of rules which a particular country or community recognizes as regulating the actions of its members and which it may enforce by the imposition of penalties. A regulation is defined as the imposition of rules by government, backed using penalties that are intended specifically to modify the behavior of individuals and firms. Regulations are used to force the community to obey the laws. Laws are proposed to the country's Parliament by various regulatory

bodies, and they need its approval. The Ministries can develop legal instruments to fill in the gaps of the law and these instruments are released in the form of Ministerial Diplomas or Ministerial Orders. Laws and regulations approved by the Mozambican Parliament are published in form of decrees.

5.1 RIGHT TO HEALTH CARE

A citizen of Mozambique is guaranteed the right to health care in the 2004 Constitution of Mozambique, which states in Article 89 that "all citizens shall have the right to medical and healthcare, within the terms of the law, and shall have the duty to promote and protect public health."[8] Article 116 states, in part, that "medical and healthcare for citizens shall be organized through a national health system, which shall benefit all Mozambican people;" "the State shall promote the expansion of medical and healthcare and the equal access of all citizens to the enjoyment of this right;" "the State shall be responsible for promoting, supervising and controlling the production, the sale and the use of chemical, biological and pharmaceutical products and other forms of treatment and diagnosis;" and "the medical and healthcare activities run by collective and private entities shall be carried out in accordance with the law and be subject to the supervision of the State."

5.2 POLICIES AND GENERAL HEALTH CARE SYSTEM

Mozambique does not have a consolidated health policy.[2] The health policy framework for Mozambique is articulated in several documents, among them the Health Sector Strategic Plan (*Plano Estratégico do Sector da Saúde*; PESS).[18] The 2014–2019 PESS is the policy document intended to provide strategic guidance for the coordination of policies and programs in the health sector for the medium and long term.[19] Another guiding document is the GRM's Five-Year Program, which is a guiding tool that contains policy guidelines for all institutions, of which health falls under its strategic objective ii (expand access and improve the age of health services).[20] A third guiding document is the Action Plan for Reduction of Poverty (*Plano de Acção para Redução da Pobreza*), which focuses on strategies to overcome barriers to health care access by poor and otherwise vulnerable populations.[2]

There is lack of an updated national pharmaceutical policy that would guide medium- and long-term objectives for the pharmaceutical sector and coordination among key stakeholders with the aim of ensuring equitable access to drugs, quality of drugs, and rational drug use.[19] A draft national pharmaceutical policy is pending approval by the Council of Ministers.

Mozambique's national health system is made up of the public sector and private sector. The combined private and public sector provide health care to nearly 30% of the Mozambican population, with only an estimated 5% covered by the private sector due to widespread poverty.[18,22] The remaining part has no access to regular health care, and seeks assistance from traditional medicine practitioners, herbalists, and others.[18,22] Law 25/91 provides for the creation of the National Health Service (NHS), which is the

---

[18] https://www.afro.who.int/pt/countries/mozambique. Accessed September 2022.
[19] MISAU/*Direcção de Planificação e Cooperação*. *Plano Estratégico do Sector da Saúde* (PESS) 2014–2019. Available from https://www.misau.gov.mz/index.php/planos-estrategicos
[20] DNF. October 2020. Strategic Plan (PEAR) for the regulatory area 2021–2025. Maputo, Mozambique.

country's public health service and main provider of health care services.[18] The rapidly growing private sector has two components:

- Private not-for-profit health care providers, which are mainly international and national NGOs, and health facilities in some large public and private companies and in educational establishments, e.g., Eduardo Mondlane University[22]
- Private-for-profit providers[18]

Based on the Decree Law 5/75 of August 17, 1975, health facilities should provide health care to all citizens indiscriminately, thus implementing the principle established in the constitution.[21] After that, medical acts, medicines, and preventive measures were made free by the Decree Law 2/77 of September 27, 1977, whereby the GRM adopted a payment criterion with what it considered affordable prices for all citizens for curative health care in the 11 provinces of the country and restriction of free health care to disease prevention campaigns.[21,22] Law no. 2/77 speaks on hospitalization rates, establishing that medicines for hospitalized patients should be provided free of charge, while "basic medicines" may be provided free of charge for outpatient treatment, and that such medicines would be indicated in a list to be defined by the Ministry of Health and the Ministry of Finance.[23]

5.2.1 National Medicines Formulary and Essential List of Medicines

As part of the health policy framework, there is an FNM and an NLEM. The FNM was introduced by the Ministerial Diploma 94/81 of December 2, 1981, to regulate the use of medicines within health services. It listed medicines by generic name and reduced the number of required medicines to achieve more cost effectiveness. Its most recent edition was the 2017 one that described how to use all medicines on the NLEM. The NLEM is aligned to the Mozambican levels of health care. The FNM has recently been undergoing revision to produce the sixth edition by the national-level Pharmacy and Therapeutics Committee (*Comissão Técnica de Terapêutica e Farmácia*; CTTF) with WHO technical support; while the 2017 NLEM has been undergoing revision by the Review Committee of the List of Essential Medicines to produce the third edition.

Compliance with the NLEM and the FNM is crucial for ensuring rational use of medicine. The Ministerial Diploma 94/81 of December 2 states: "In these terms, after hearing the Technical Commission of Pharmacy and Therapeutics, and using the competence assigned to them by Decree 1/75, of July 29, the Ministers of Health, Internal Trade and Finance determine that:

- First: State stores, consumer cooperatives, and other commercial establishments based in places where there are no pharmacies may be authorized to sell the medicines and dressing items in the attached list and only these, as well as other related products that may come to be expressly authorized by the Pharmaceutical Service.

---

[21] Mitano, F. et al. 2016. Right to health: (in) congruence between the legal framework and the health system. Revista latino-americana de enfermagem, 24, e2679. Available from https://www.ncbi.nlm.nih.gov/pmc/articles/PMC4809183/
[22] Garrido, Paulo Ivo. 2020. Health, development, and institutional factors: The Mozambique case, WIDER Working Paper, No. 2020/131, ISBN 978-92-9256-888-7, The United Nations University World Institute for Development Economics Research (UNU-WIDER), Helsinki. Available from: https://doi.org/10.35188/UNU-WIDER/2020/888-7
[23] Global Legal Insights, Francisca Paulouro & Pedro Fontes, VdA. 2019. Pricing & Reimbursement: Mozambique. 2nd edition. Available from https://www.vda.pt/xms/files/05_Publicacoes/2019/Livros_e_Artigos/Pricing__Reimbursement_2019_-_Mozambique.pdf

- Second: Authorizations for the sale of these drugs will be granted by the Provincial Directorates of Health (*Direcção Provincial de Saúde*; DPS) , after consulting the respective Provincial Directorates of Internal Trade, upon payment of a fee of 1000.00 meticais (MT) to be proved by the destruction, in the corresponding application, of tax stamps of that amount.
- Third: The storage of the products referred to in the first number of this ordinance must be done in closed and properly protected cabinets.
- Fourth: Requests for the purchase of the same products by authorized establishments may be directly satisfied by the Medicines Import and Export Company (*empresa de importação e exportação de medicamentos*; MEDIMOC) or its delegations against the presentation of the respective license.
- Fifth: The prices to be charged will be those that are marked on the respective packages, and any alteration is punishable under the legal terms.
- Sixth: The DPSs must have an up-to-date register of establishments authorized to sell medicines under the terms of this ministerial diploma and communicate the licenses granted to the Pharmaceutical Service, within a maximum period of eight days.
- Seventh: The inspection of compliance with the provisions of this ministerial diploma is the responsibility, at local level, of the respective health structures, at national level, of the Pharmaceutical Service."

The FNM and NLEM include antimicrobials but are not yet aligned with the WHO AWaRe classification, which is one of the recommended WHO benchmark actions at country level.

Gaps

- Lack of an updated national pharmaceutical policy to guide the pharmaceutical sector. The draft pharmaceutical policy should include a section with a specific focus on antimicrobial medicines.
- The NLEM and FNM do not yet comply with the current WHO-recommended AWaRe categorization of antimicrobials, so they require updating.

5.3 THE MEDICINES LAW AND REGULATION OF MEDICINES

The Law 4/98 of January 14, 1998, governed the production, import, marketing, and dispensing of medicines and the establishment of the Medicines Council (*Conselho do Medicamentos*; COMED). Among the objectives of the Law 4/98 were to "govern the production, import, marketing, and dispensing of medicines, aiming to:

- Ensure that medicines in the Republic of Mozambique are safe, effective, of good quality, and correspond to the real needs of the population
- Promote the rational use of medicines
- Guarantee the regular availability of medicines, according to the needs of the population and at an affordable cost
- Promote ethics in pharmaceutical activity"

From the Law 4/98, COMED, the medicines advisory council, had the following advisory responsibilities:

- Preparing and updating the FNM
- Pronouncing itself under the terms of this law on matters pertaining to pharmacy and medicines
- Preparing therapeutic guidelines for treatment of the main diseases

- Evaluating and recommending the decision on drug registration applications and issuing the competent certificates
- Controlling the quality of medicines in circulation in the country
- Proceeding with the instruction of processes related to licensing requests from producers, importers, stockists, and pharmacies and issuing the respective registration certificates
- Approving and registering prices for the sale of pharmaceutical commodities to the public
- Promoting the rational use of medicines
- Organizing and carrying out pharmaceutical inspection

In the area of governing the regulation of medicines, this Law 4/98 was overtaken by the release of the updated medicines Law 12/2017.

The Medicines Law 12/2017 of September 8, 2017, on the regulation of medicines, vaccines, and biological products provides a solid legal framework and basis for ensuring that pharmaceutical products in Mozambique are of acceptable quality, safety, and efficacy, and specifying the responsible agency. The Medicines Law is intended to govern the production, import, marketing, and dispensing of medicines and one of its objectives is to ensure that medicines in the Republic of Mozambique are safe, effective, of good quality, and meet the needs of the population.

It also provides for the establishment of ANARME, the national regulatory authority (NRA), whose pharmaceutical regulatory functions were at first carried out by the DNF.

Article 3 of the Law 12/2017 indicates the objectives of the law as including:

- To regulate the production, distribution, and marketing of medicines, vaccines, biologicals, and other health products
- To facilitate the drug pricing system to avoid speculation
- To facilitate the drug registration system and deal with aspects related to the exercise of the pharmaceutical profession

The Law 12/2017 covers medicines, vaccines, and biological products in general; in addition, it addresses areas related to the following:

- The processes of marketing, research, registration, production, import, quality control, export, transport, prescription, dispensing, use, and the pricing regime for medicines, vaccines, and biological products for health and human use
- Control of the quality of medicines in circulation in the country
- Instruction of processes related to licensing requests in the pharmaceutical area
- Promotion of national research and production of medicines, vaccines, and biological products for human use
- Ensure the control of narcotics and psychotropic substances as established in international conventions
- Registration of professionals in the pharmaceutical area
- Ensuring PV
- Promotion of the rational use of medication
- Organization and conduct of pharmaceutical inspection

Other competences are defined in the respective organic statute.

Gap

- As noted above, the national drug legislation was first created in January 1998 and adjusted in September 2017 with a few changes. Although the Law 4/98 which established COMED as the implementing authority expected COMED to promote rational use of medicines, the adjusted Law 12/2017 created the NRA and is clearer on its responsibility for promotion of the appropriate use of medicines. Nevertheless, in both laws and the additional regulations and policies, neither AMS nor specified activities to promote appropriate use of antimicrobials are mentioned. Currently, there is no specific regulation in place to address antimicrobial use in Mozambique.

5.4 PRODUCT REGISTRATION/MARKETING AUTHORIZATION

Health product registration or marketing authorization (MA) of medicines for human health is one of the key regulatory functions that ensures access to safe, effective, and good quality medicines on the market in Mozambique, thus avoiding the use of substandard and falsified drugs and aiming to ensure provision of high quality and effective pharmaceutical products to its citizens.

The Decree 22/99 of May 4, 1999, replaced the earlier decree first issued in early 1970s (Decree 229/70 of May 20) and readjusted in 1999, whereby it was made "necessary to define the conditions, rules, and procedures for authorization of manufacture, importation, intrusion, and withdrawal of medicines from the market, their alterations and renewals". The Decree 229/70, the Decree 22/99, and the Minister of Health Order of 14 March 2001 were prepared in generic terms for all medicines. MA is also regulated by the Law 12/2017.

MA is also regulated by the Decree 93/2018 of December 31, 2018, (Regulation of Authorization for the Marketing of Medicines) as well as the draft Regulation on medicines registration issued under the Decree 93/2018, which was reviewed in May 2020 with MTaPS support.

The Medicines Registration Regulation, approved by Decree number 22/99, confers on the Minister of Health the power to issue expedited procedures for the extraordinary registration of medicines in circulation, until the establishment of a normal system of registration of medicines in the country. Under the provisions of the regulation's article 31, the Minister of Health determines for the purposes of this order medicines for which extraordinary registration is required; such registration being understood as the expedited registration of medicines in circulation in the country upon notification to COMED by the entities authorized to produce and/or import medicines.

5.4.1 Importation of medicines

Mozambique imports almost 99% of its medicines and other health products from foreign sources. The changes in the importation process had two phases. In the first phase, earlier, after Mozambican independence, the importation of all medicines was a function of the state's Drug Import and Export company MEDIMOC as per Decree Law 15/91 of August 3, 1991. As part of the restructuring process of the State's business sector, MEDIMOC was identified for privatization under article 14 of Law 15/91. Pursuant to section e) of paragraph 1 of article 8 of this same law, and the Decree no. 28/91 of November 21, 1991, a negotiation was carried out with the managers, technicians, and eligible workers,

with a view to disposal of the company's assets. Once the negotiations with the aforementioned managers, technicians, and workers were concluded, the adjudication of 70% of the assets of that unit was formalized in order to precisely define the rights and obligations of the parties, in the context of the privatization of MEDIMOC.

In the second phase, later Mozambique introduced the open market, which included the importation and exportation of medicines, and the Decree 22/99 of May 4 was approved to accommodate this new market context.

In both phases, there was no mention of pre-importation quality verification for the identification of substandard and falsified or fake pharmaceutical products. These decrees address marketing and dispensing issues but do not mention AMS. The implementation of controls in the importation of medicines in Mozambique is performed by ANARME at the country's points of entry.

5.4.2 Exportation of medicines

There is no legal provision for regulation of pharmaceutical exports from the country. For this reason, no data are available for the export or re-export of pharmaceuticals.

In 2021, MTaPS supported the development of a draft Regulation for Import, Export, and Distribution (of medical products) that also includes an annex on Guidelines for Good Distribution Practices.

Gaps

- The policy framework, medicines law, and the regulation and MAs cover pharmaceutical products in general, but these legal documents do not address AMR or AMS.
- While laws, regulations, and policies are required, there is a need for an effective monitoring of their implementation through an effective governance structure.
- There is no regulation addressing pre-importation quality verification to deter importation of substandard and falsified or fake antimicrobials, leaving the market open to importation and marketing of non-efficacious antimicrobials.

5.5 PHARMACEUTICAL PRACTICE

The Decree 21/99 of May 4, 1999, governs the exercise of the pharmaceutical profession. It establishes that the function of preparing, verifying the quality, preserving, distributing, and dispensing medicines is an activity of public interest that must be performed by the pharmaceutical profession to ensure strict compliance with technical, ethical, moral, and deontological principles. MTaPS supported the DNF in development of a draft Regulation of Exercise of the Pharmaceutical Profession to support the implementation of this decree.

5.6 LICENSING AND INSPECTION

The licensing to establish pharmacies is regulated by a specific regulation, the Ministerial Diploma 39/2003 of April 2, 2003, which establishes all the requirements to operate such establishments.

Licensing and inspection of establishments is also regulated by the Law 12/2017.

The above-mentioned regulation applies for licensing of pharmacies as well as the licensing of pharmaceutical professionals. It emphasizes the requirement for all pharmacies to employ licensed pharmaceutical professionals to dispense medicines, including antimicrobials.

The licensing for the opening of a pharmaceutical or related establishment and the practice of the pharmacy profession are done by MISAU through ANARME as stated in the Decree 229/70 of May 20, 1970.

The pharmaceutical regulatory inspection functions are carried out mainly by ANARME, in collaboration with the General Inspectorate of Health (GHI) and the DTH from the National Directorate of Medical Assistance (*Direcção Nacional de Assistência Médica*; DNAM) of MISAU, as stated in the Law 12/2017. ANARME has a mandate to inspect pharmaceutical practice in the country for public health services and the private sector. The DTH mainly focuses on pharmaceutical services in health facilities of the public health sector and does not have any influence on the private sector. The GHI performs inspections in all areas covered by MISAU as well as in the private sector. ANARME and the GHI jointly implement their activities if needed in the public and private sectors.

The Regulation for pharmaceutical inspection specifies that inspections may be conducted in private and public companies and at all stages of the pharmaceutical product distribution chain.[24] In 2020, MTaPS supported the DNF in the review of the draft 2019 Regulation on Pharmaceutical Regulatory Inspection.

In 2019 while the Regulation for Licensing Establishments approved in February 2019 by the Ministerial Council was awaiting publication, MTaPS provided technical support to its validation.

Gaps

- These legal instruments do not clearly state the mechanisms through which ANARME can monitor the implementation of the GRM's AMR and AMS interventions in private pharmaceutical premises.

5.7 SUPPLY CHAIN MANAGEMENT POLICIES AND GUIDELINES RELEVANT TO AMS

Supply chain management refers to procedures and strategies to control the distribution, sale, and use of antimicrobials from the point of manufacture to the end user. Such actions include registering human pharmaceutical premises, practice facilities, and pharmaceutical services professionals.

The Mozambican supply of pharmaceutical products for both public and private sectors are obtained from imports because local production of health products remains very low.

The management of AMR and AMS in the human health sector is being handled by ANARME in the policy, legislation, and regulatory aspects. The GHI at MISAU is the institution which reinforces the implementation of the policies, legislations, and regulations for the management of AMR and AMS through its department of pharmaceutical inspection, which works at the health facility and community levels. The management of AMR and AMS at the health facility level is implemented by the DTH. ANARME, the DTH, and the GHI are indicated as main implementers of the NAP-AMR activities of the priority actions 6.3.4 (Priority activities for the pharmaceutical supply chain and access to

[24] *Regulamento da inspeção farmaceutica* (Pharmaceutical inspection regulation), 2019 version.

antimicrobials), 6.3.5 (Activities to optimize the use of antimicrobial drugs in humans and animals), and 6.3.6 (Activities to change incentives [that encourage the overuse of antibiotics]).

Medicines can only be purchased by and used within the NHS if they are included in the FNM or in the NLEM. Both the FNM and the NLEM should be periodically revised and published by MISAU, which is also responsible for ensuring that the medicines included in these documents are in fact used. However, the FNM was last revised in 2017 and the revision process is currently incomplete.

The procurement of medicines and most medical supplies for the public health system is done by the Center for Drugs and Medical Supplies (*Central de Medicamentos e Artigos Médicos*; CMAM) created by the Decree 13/75 of September 1975. Since procurement by MISAU is undertaken by 2 institutions, along with CMAM, the Procurement Management and Execution Unit procures medical chirurgical supplies, as well as medical and non-medical equipment, furniture, non-medical supplies, and transport facilities.[25] Procurement of medicines by CMAM follows the medicines listed by the NLEM and the pharmaceutical needs of the various health facility levels. Public sector procurement of pharmaceutical products is done by CMAM with financial support of the GRM budget and donors. CMAM ensures the supply of essential generic medicines and medical consumables to all Mozambican district distribution depots via its 10 agencies in 3 regions.

In 2013, a supply management plan was developed for human pharmaceutical products. The health sector strategic plan 2014–2019 mentions that there is a Pharmacy and Logistics Strategic Plan (*Plano Estratégico de Logística Farmacêutica*), which was then undergoing revision by CMAM, but there are still gaps in mention of AMR and AMS areas.

Private sector supply is undertaken by private wholesale distributors who supply private hospital pharmacies and private dispensaries, which in turn supply private drug depots. Several laws and regulations govern the supply of health products and the implementation of regulatory functions.

When medicines are provided free of charge pursuant to the Law 2/77 and the Ministerial Order 24/85, the selection and payment of medicines are entirely handled by MISAU and the Ministry of Finance.

A draft Regulation on Price Control was developed, with MTaPS support in 2021, that aims to ensure the accessibility and availability of pharmaceutical products and guarantees the protection of consumer rights. While this regulation addresses the prices of medicines in general, it does not specifically address pricing of antimicrobial medicines.

5.8 PRESCRIBING, DISPENSING, AND USE OF MEDICINES

The rational use of medicines and other health products for better therapeutic management of the Mozambican population has been promoted through the NLEM and the FNM in general. The FNM categorizes the health facilities, and it indicates the kind of health professionals allowed to prescribe

[25] JANS team. August 2013. Joint Assessment of the Mozambican Health Sector Strategic Plan (PESS), 2014–2019. Available from https://www.uhc2030.org/fileadmin/uploads/ihp/Documents/Results___Evidence/JANS_Lessons/2013.09.02%20JANS%20English%20Report%20Final%20with%20Annexes.pdf

certain types of drugs, including antimicrobials. However, the two documents do not address AMS optimally as they lack the WHO AWaRe classification of antimicrobial drugs.

Decree 21/99 of May 4, 1999, clearly describes the dispensing procedure and who should dispense the pharmaceutical products.

The Ministerial Diploma 39/2003 of April 2, 2003, besides requiring that all pharmacies employ licensed pharmaceutical professionals to dispense medicines, also requires that medicines, including antimicrobials, only be dispensed through a prescription generated by a health professional.

There are, however, medicines that can be dispensed without a prescription as defined by law in the Ministerial Diploma 94/81 of December 2, 1981. This diploma also provides a list of these medicines, which are outside the FNM and which are drugs authorized to be sold by public stores and related commercial establishments, with an updated list from the older Law 105/77. Antibiotics are not included in this document.

Only private sector providers of health care services can prescribe medicines and other health products that are not included in the NLEM and the FNM, provided that these products are registered for use in Mozambique.

The effort to design clinical treatment guidelines was made for most of the infectious diseases of the priority parallel/vertical programs such as HIV, tuberculosis, sexually transmitted diseases, and malaria. Although there are national diagnosis and treatment guidelines for prescribers for pediatric, surgical, and obstetrics and gynecology patients, their review is not regular; and standard treatment guidelines for other areas of human health, such as internal medicine and intensive care, are lacking.

Medicines and therapeutics committees have been established in 50 out of 58 level 3 and 4 health facilities (provincial, general, and central hospitals).

Apart from the NLEM and the FNM, which describe the level at which a medicine may be prescribed, to MTaPS' knowledge there are no clinical guidelines to ensure appropriate use of antibiotics in health facilities.

Although in the public sector, prescription of antibiotics is guided by the FNM, the guideline is not often followed by the private sector. Antibiotics are classified as prescription-only medicine (POM) in Mozambique; however, one study found that POM is not fully enforced, allowing the public access to a variety of antibiotics that would otherwise be only accessible by prescription.[26] Antibiotics were available over the counter in many private pharmacies. The use of antibiotics without a prescription was mainly for cough, pain, wounds, and fever, with the most used of the non-prescribed antibiotics being amoxicillin, followed by cotrimoxazole—which are also in line with the antibiotics most frequently bought in informal markets in Maputo. The growing practice of self-medication with antibiotics exposes users to the risk of developing antibiotic resistance and antibiotic side effects and increases the health service costs and morbidity.

[26] Rodrigues, C.F. 2020. Self-medication with antibiotics in Maputo, Mozambique: practices, rationales and relationships. Palgrave Commun 6, 6 (2020). https://doi.org/10.1057/s41599-019-0385-8

Another study noted 20.9% of respondents had used non-prescribed antibiotics, nearly 90% of which were purchased in private pharmacies.[27] Those respondents who had purchased their non-prescribed antibiotics from informal markets and home stores were more likely to use them than those who purchased them from the private pharmacies. Both studies recommend a critical need to involve pharmacy workers in programs aimed at improving antibiotic use, education of the community, and interventions to reinforce adherence by pharmacies to the current legislation governing the dispensing of antibiotics.[26,27]

Gaps

- The FNM as well as the national treatment guidelines for obstetrics and gynecology, surgical, and pediatric areas require regular updating to align with the health care needs of the population.
- Standard treatment guidelines for other areas of human health, such as internal medicine and intensive care, require development and dissemination.
- There was no specific regulation on prescription-only sales of key antibiotics found in this desk review.
- Poor enforcement in private pharmacies of prohibition of over-the-counter sales of antibiotics without a prescription.
- The country's legal instruments do not clearly state the mechanisms through which ANARME can monitor the governmental AMR and AMS interventions in private pharmacies.

5.9 POST-MARKET SURVEILLANCE AND PHARMACOVIGILANCE

5.9.1 PMS

Market surveillance and control procedures set up by ANARME consist of organizing post-marketing quality controls of medicines and other health products in all pharmaceutical establishments, hospitals, and health facilities in the country every six months. The purpose of this control is to verify the conformity or nonconformity of these products to the standard specifications.

Laboratory testing of medicines is coordinated by ANARME, which carries out sampling at different levels of the pharmaceutical distribution chain and has the medicines' compliance checked at the National Quality Control Laboratory (*Laboratório Nacional de Controle de Qualidade*; LNCQM) or in certified international laboratories, depending on the sources of funding. Some donors require WHO prequalification or accreditation according to ISO:17025 for the performance of quality control on the products for which they provide funding for procurement.

The low financial resources devoted to quality control and laboratory testing, which have high operating costs, leads to very low quantities of samples being picked and analyzed. Finally, the technical skills and capacity for detecting substandard and falsified health products are insufficient at all levels of the medicines supply chain in relation to the scale of the phenomenon observed at the global level.

[27] Mate, I., et al. 2019. Knowledge, attitudes, and practices regarding antibiotic use in Maputo City, Mozambique. https://doi.org/10.1371/journal.pone.0221452

5.9.2 PV

ANARME is responsible for PV. PV is being implemented nationwide for monitoring adverse reactions to health products as per the Ministerial Diploma 94/81 of 23 of March. The objectives of the national PV system are:

1. Collect, evaluate, and disseminate information on suspected adverse reactions related to medicines subject to import and special use authorization, as well as on clinical trials carried out with medicines in human beings
2. Measure the results of responses or actions carried out by the system (e.g., risk reduction, improve drug use, improve the outcome of an adverse reaction case)
3. Without prejudice to the previous numbers, in addition to adverse drug reactions, these are relevant issues for the national system of PV:
    - deviations from pharmaceutical quality
    - medication administration errors
    - notification of loss of effectiveness
    - use of drugs for unapproved indications, which do not have adequate scientific basis
    - notification of cases of acute or chronic drug poisoning
    - assessment of drug-induced mortality
    - abuse and misuse of medications
    - interaction of drugs with adverse effects with chemical substances, other drugs or with food

The Ministerial Diploma 53/2010 provided approval for the Regulation on the National PV system. The Regulation on PV was reviewed in July 2020 with MTaPS support.

Although the PV system addresses some aspects to AMS in its purposes as mentioned above (such as medicine abuse and misuse, loss of effectiveness), these aspects are applicable to medicines in general; therefore, there is a need for specific AMS-related content in the PV-related legislation, regulations, and guidelines.

Gaps

- There is a lack of specific legislation, regulations, and guidelines dealing with AMS.
- Although the PMS is functional, only a few samples of medicines are picked, funding is limited, and there are insufficient technical skills and capacity for detecting substandard and falsified health products. There is a need to assess a broader range of products, including antimicrobials, to align with the fight against AMR.
- PV-related legislation, regulations, and guidelines do not include AMS aspects.

6. DISCUSSION

This rapid assessment provides an insight into the legal framework of Mozambique on AMS. Mozambique is a country that has gone through various sociopolitical stages after its independence from Portugal in 1975.

The current available legislation, regulations, policies, and guidelines were developed for medicines in general; and do not mention AMR or AMS. There are laws, regulations, and guidelines for the production, importation, and registration of medicines.

From this rapid assessment of the policies and legal and regulatory framework, it was observed that there have been attempts by the Mozambican government to follow international conventions and address issues of global public health interest to guarantee the safety of Mozambicans. For example, the Ministerial Diploma 160/88 of November 16, 1988, was adopted to prevent and combat the abusive use and illicit trafficking of psychotropic substances to the detriment of public health, and to pursue efforts to fulfill the obligations linked to the international body for the control of narcotics and psychotropics. Another example is the Decree 16/88 of December 27, 1988, which was adopted to guarantee as much coverage as possible with access to medicines in a context where the number of pharmacies was insufficient, and when Mozambique was in a serious economic crisis without means to purchase medicines. The purpose of the Decree 16/88 is like the Ministerial Diploma 24/85 of July 3 and highlights how Mozambique offers health and medicine to all.

The quality, safety, and efficacy of medicines have been a concern in Mozambique through the various sociopolitical contexts. There is no manufacturer of pharmaceutical products in the country, and most of the medicines are imported from other countries, thus quality and safety are not ensured through this legislation.

Mozambique has its NAP-AMR, which acknowledges that AMR is a local and global public health problem, and that antimicrobial medicine overuse and misuse are among the major drivers for the emergence, increase, and spread of AMR in hospitals and in the communities. However, there are no specific regulations, policies, or guidelines on AMR. There was no mention of AMS found in the legal documents assessed during this assessment process. There is a need to elaborate legal instruments on AMR and AMS to enable the implementation of the objectives of the NAP-AMR.

This rapid assessment concluded there is a lack of legislation, regulations, policies, and guidelines on AMR and AMS; and that the few existing documents on AMS lack essential elements, such as the WHO AWaRe classification.

7. RECOMMENDATIONS

AMS is an important component in the fight against AMR, and the implementation of AMS interventions requires clear guidelines, regulations, legislations, and policies. This rapid assessment demonstrates the lack of these guidance documents for AMS; therefore, it is recommended that:

- CTTF, ANARME, and the DTH should update the NLEM and the FNM to adhere to the WHO AWaRe classification.
- Beyond the guidelines for management of the vertical program infectious diseases such as tuberculosis, HIV, and sexually transmitted diseases, the DTH should develop national treatment guidelines for the main infectious diseases such as respiratory infections, diarrhea, and urinary tract infections.
- ANARME should develop a policy to monitor AMR and AMS interventions in the private sector
- ANARME should develop or improve legislation to specifically address or include AMS, in line with WHO IHR benchmark 3.4 capacity level 2 action that recommends development of legislation that regulates the use, availability, and quality of antimicrobials.
- ANARME and the DTH should develop specific regulations and guidelines for antimicrobials and AMS. Recommendations include a regulatory provision on appropriate use of antimicrobials in the

existing regulatory framework for medicines and a draft regulation on prescription-only sales of key antibiotics—with the recommended capacity level 3 action of the WHO benchmark 3.4.

- ANARME, the DTH, and the national AMS TWG should develop national AMS guidelines for the AMS program, and a NAP or strategy for AMS that includes interventions to address the identified gaps, in line with the recommended capacity level 2 action of the WHO benchmark 3.4.

8. CONCLUSION

The rapid assessment results will help Mozambique establish its country baseline with regards to AMS, highlight gaps and challenges, and suggest recommendations to inform efforts to revise or develop laws, policies, regulations, and guidelines to address the identified gaps.

Mozambique has had policies, legislation, regulations, and guidelines for medicines in general since the early 1990s. There is a statement on the rational use of medicines in the Law 12/2017, but it contains nothing specific to antimicrobial medicines. AMS and AMR were not specifically mentioned or provided for in the available and reviewed policy, legal, regulation, and guidance documents.

Although there are national standard treatment guidelines currently available for vertical programs such as malaria, tuberculosis, and sexually transmitted infections, as well as for pediatric, surgical, and obstetrics and gynecology care, their review is not regular; standard treatment guidelines for other areas of human health, such as internal medicine and intensive care, are lacking. The existing NLEM and FNM do not include WHO AWaRe classification of antibiotics. Available laws address marketing and dispensing issues but do not mention AMS. The implementation of controls in the importation of medicines in Mozambique is performed by ANARME at the country's points of entry.

Assessment of a broader range of products, including antimicrobials, is required for effective PMS. The national PV system objectives address some aspects to AMS, but these aspects are applicable to medicines in general and do not mention antimicrobial medicines; hence, there is a need for specific AMS-related content in the PV-related legislation, regulations, and guidelines. There was no specific regulation on prescription-only sales of key antibiotics found in this desk review. There is no regulation addressing pre-importation quality verification to deter importation of substandard and falsified or fake antimicrobials, leaving the market open to importation and marketing of nonefficacious antimicrobials.

There is poor enforcement in private pharmacies of prohibition of over-the-counter sales of antibiotics without a prescription. The country's legal instruments do not clearly state the mechanisms through which ANARME can monitor the implementation of the GRM's AMR and AMS interventions in private pharmaceutical premises.

APPENDIX 1. LIST OF POLICIES, LAWS, REGULATIONS, AND GUIDELINES ASSESSED

#	Name of the document	Document type	Description of relevant content
1	The Ministerial Diploma 94/81 of December 2, 1981	policy	Authorization of the sale of medicines listed by the CTTF; introduced the FNM listed with generic medicine names to regulate the use of medicines within health services and reduced the number of required medicines to achieve more cost-effectiveness. The FNM was accompanied by legislation that allowed the prescription of generic drugs only.
2	The Ministerial Order 24/85 of July 3	policy	After Law 2/77 established that medicines for hospitalized patients should be provided free of charge, that "basic medicines" may be provided free of charge for outpatient treatment, and that such medicines would be indicated in a list to be defined by the Ministry of Health and the Ministry of Finance; the said list was later approved by the Ministerial Order no. 24/85 of July 3 ("Ministerial Order 24/85"). The Ministerial Order 24/85 further established that all medicines distributed by community health workers would be free of charge, and that medicines dispensed by the primary care network in rural areas would have a single price of 20 MT per prescription (and should be paid by the GRM if the price exceeded that amount). Medicines provided free of charge pursuant to Law 2/77 and Ministerial Order 24/85 may only be distributed at medicine distribution points and pharmacies of the Government, which are attached to the health facilities where such medicines are prescribed. Medicines which are part of the National Medicines List and which are not included in the List of medicines provided for free pursuant to Ministerial Order 24/85 are purchased at their approved sales price.[23]
3	NLEM 2017	policy	Undergoing revision by the Review Committee of the List of Essential Medicines to produce the 3rd edition
4	FNM	guideline	Undergoing revision by the CTTF to produce the 6th edition
5	PESS 2014–2019	policy	Strategic guidance for the coordination of policies and programs in the health sector
6	Constitution of Mozambique 2004	law	Rights of citizens
7	Law 25/91 of December 31, 1991	law	Creation of the NHS
8	Decree Law 5/75 of August 17, 1975	law	Provided for the nationalization of the private clinics that existed during the colonial period, to maximize resources by offering greater access to health care services that provided indiscriminate assistance to Mozambican citizens
9	The Law 2/77 of September 27, 1977	law	Affordable prices for curative care and restriction by the GRM of free health care to disease prevention campaigns; medicines for hospitalized patients should be provided free of charge, while "basic medicines" may be provided free of charge for outpatient treatment, and that such medicines would be indicated in a list to be defined by the Ministry of Health and the Ministry of Finance.[22,23]
10	The Law 4/98 of January 14, 1998	law	Governed the production, import, marketing, and dispensing of medicines (revoked by Law 12/2017); and the establishment of the COMED. Put the regulation of the pharmaceutical sector under the then-Pharmaceutical Department.
11	The Law 105/77 of March 17, 1977	law	Lists the drugs authorized to be sold by public stores and related commercial establishments
12	The Law 3/97 of March 13, 1997	law	
13	The Law 12/2017 of September 8, 2017	law	The law on medicines, vaccines, and other biological products for human use. It also revokes Law no. 4/98 of January 14. It governs the regulation of medicines, vaccines, and biological products in Mozambique, and the establishment of the NRA named ANARME.
14	Decree 93/2018 of December 31, 2018	regulation	Regulation governing the authorization for the marketing of medicines

#	Name of the document	Document type	Description of relevant content
15	Decree 229/70 of May 20, 1970	regulation	Pharmaceutical profession; medicine
16	Decree 16/88 of December 27, 1988	regulation	This created a social fund for pediatric medicines and food supplements, whereby population groups in need of economic support because they suffer from chronic conditions or because they belong to economically deprived groups (e.g., physically handicapped, elderly, unemployed, etc.), have access, in NHS pharmacies, to medicines subsidized by the GRM (the GRM contribution to the price of medicines varies between 100%, 80% and 50% of their public sales price).[23]
17	The Decree 21/1999 of May 4, 1999	regulation	Exercise of the pharmaceutical profession. Also describes the dispensing procedure and who should dispense.
18	Decree 22/99 of May 4, 1999	regulation	Registration of medicines; expedited procedures for the extraordinary registration of medicines
19	Decree 13/75 of September 1975		Creation of CMAM
20	The Ministerial Diploma 98/2000 of August 9, 2000	regulation	Determines fees owed by applicants in relation to drug registration acts and laboratory analysis
21	The Ministerial Order of March 14, 2001	regulation	Order from MISAU providing expedited procedures for the extraordinary registration of medicines in circulation, until the establishment of the normal system of registration of medicines in the country
22	The Ministerial Diploma 39/2003 of April 2, 2003	regulation	Requirements for licensing of pharmaceutical establishments as well as the licensing of pharmaceutical professionals. Requirement for all pharmacies to employ licensed pharmaceutical professionals to dispense medicines and for all medicines to be dispensed only through a prescription generated by a health professional.
23	Ministerial Diploma 53/2010	regulation	The national PV system
24	Ministerial Order 21/2017 of March 13, 2017	regulation	Provides for a new medicine pricing regime, focusing more on simplifying price setting, and adapting it to the costs associated with importing medicines into Mozambique.[23]

APPENDIX 2. LIST OF REGULATIONS AND GUIDELINES WHOSE DEVELOPMENT/REVIEW HAS BEEN SUPPORTED BY MTAPS

#	Name of the document	Document type	Description of relevant content
1	Regulation on Regulatory Inspection	regulation	The reviewed draft regulation was prepared in April 2020.
2	Regulation of Exercise of the Pharmaceutical Profession	regulation	The draft Regulation was reviewed in April 2020.<br>Applies to manufacturers, importers, distributors, and transporters of pharmaceutical products (i.e., all those involved in the pharmaceutical business in the public and private sectors)
3	Regulation on Medicines Registration	regulation	Issued under Decree 93/2018. It provides ANARME with the ability to grant MA for a five-year period which is renewable.<br>The draft Regulation on medicines registration was reviewed in May 2020 with MTaPS support.
4	Regulation on Price Control	regulation	The draft Regulation on Price Control was developed with MTaPS support in 2021
5	Regulation on PV	regulation	The proposed revision to the Regulation on PV was reviewed in July 2020 with MTaPS support.
6	Draft Regulation for Import, Export, and Distribution	regulation	The draft Regulation was developed with MTaPS support in 2021. As per a request from ANARME, the draft has an annex of Guidelines on Good Distribution Practices. The regulation's purpose is to control activities in the distribution, import, and export of medicines and other health products.
