# Supplementary material for: Landscape Analysis of Existing Policies, Laws and Regulations on Antimicrobial Stewardship in 10 Low- and Middle-Income Countries
Source: Antibiotics (Basel). 2026 Aug 10;15(8):770. doi: 10.3390/antibiotics15080770 (PMC13510074; doi:10.3390/antibiotics15080770)
Supplement: Supplementary file 1 [file antibiotics-15-00770-s001.zip › S3 Country Reports/S3F-2_Mozambique (Animal Health).pdf]

FOOD AND AGRICULTURE ORGANIZATION OF THE UNITED NATIONS (FAO)

OSRO/GLO/507/USA

ECTAD

QUADRO JURÍDICO NACIONAL RELEVANTE PARA USO DE ANTIMICROBIANOS E RESISTÊNCIA ANTIMICROBIANA EM MOÇAMBIQUE

Por

Manuel Castiano

Consultor Nacional Legal

Novembro 2022

Maputo, Moçambique

Isenção de responsabilidade

A informação contida neste relatório é produto do perito moçambicano contratado pela FAO para analisar o quadro legal para o uso de antimicrobianos e resistência antimicrobiana em Moçambique e fornecer orientação independente à FAO e ao Ministério responsável pela Agricultura no contexto do projecto OSRO/GLO/507/USA "ECTAD" tendo em consideração o seu conhecimento e experiência. Consequentemente, as opiniões expressas neste relatório são do autor e não refletem necessariamente as opiniões ou políticas da FAO sobre certas questões, que podem ser reflectidas em documentos adicionais.

AGRADECIMENTOS

O autor agradece à equipa da FAO Roma e FAO Moçambique por fornecer todo o apoio técnico durante a elaboração do presente relatório. Igualmente, agradece os quadros técnicos do Ministério da Agricultura e Desenvolvimento Rural, todas individualidades, sector privado e comunidades que emprestaram o seu saber, que de algum modo se encontra reflectido neste documento.

ABREVIATURAS E ACRÓNIMOS

ANARME	Autoridade Nacional Reguladora de Medicamento
CATERP	Comité de Avaliação Técnica do Registo de Pesticidas
CPLP	Comunidade dos Países de Língua Portuguesa
CRM	Constituição da República de Moçambique
DNSAB	Direcção Nacional de Sanidade Agro-Pecuária e Biossegurança
DNDP	Direcação Nacional de Desenvolvimento Pecuário
FAO	Organização das Nações Unidas para a Alimentação e a Agricultura
INAE	Inspecção Nacional das Actividades Económicas
INNOQ	Instituto Nacional de Normalização da Qualidade
LMVOPBUH	Lei de Medicamentos, Vacinas e outros Produtos Biológicos para Uso Humano
MADER	Ministério da Agricultura e Desenvolvimento Rural
MIMAIP	Ministério do Mar, Águas Interiores e Pesca
MITA	Ministério da Terra e Ambiente
MISAU	Ministério da Saúde
MEF	Ministério da Economia e Finanças
MOPHRH	Ministério das Obras Públicas, Habitação e Recursos Hídricos
MINED	Ministério da Educação e Desenvolvimento Humano
OMS	Organização Mundial da Saúde
OMSA	Organização Mundial da Sanidade Animal
NM	Normas Moçambicanas
PNA	Plano Nacional de Acção contra a Resistência Antimicrobiana
PRDMPV	Proposta de Regulamento de Drogas, Medicamentos e Produtos Veterinários de 2019
RGP	Regulamento sobre a Gestão de Pesticidas
RAM	Resistência Antimicrobiana

Sumário Executivo

Na sequência do pedido formulado pelo Ministério da Agricultura e Desenvolvimento Rural (MADER) à Organização das Nações Unidas para a Alimentação e a Agricultura (FAO) através dos seus escritórios de Moçambique, no sentido de auxiliar na avaliação do quadro jurídico e institucional, e identificação de lacunas relativamente ao uso de antimicrobianos e resistência antimicrobiana iniciou-se em Março de 2022 um trabalho tendente a alcançar os seguintes objectivos:

- Recolher toda legislação relevante para resistência antimicrobiana (RAM) na sanidade animal e sectores relacionados, incluindo legislação veterinária, legislação sobre medicamentos veterinários, segurança alimentar, rações/forragem, produção animal, produção agrícola e pesticidas, qualidade da água, gestão de resíduos e proteção ambiental, seguindo a metodologia fornecida pelo Departamento Legal da FAO (LEGN).
- Apresentar um relatório jurídico nacional sobre RAM contendo propostas de recomendações para reforma legal

Para o efeito foram desenvolvidas as seguintes actividades (i) identificação e recolha toda a legislação directa ou indiretamente relevante para o uso de antimicrobianos e a relevante para a resistência antimicrobiana; (ii) análise das lacunas existentes na legislação mencionada em (i), e as possíveis soluções técnicas; (iii) preparação de um relatório descrevendo o quadro legal nacional respectivo.

Na sequência da análise efectuada, encontramos cinco quadros legais distintos e concorrentes relevantes para a redução da RAM, nomeadamente, sobre: a) medicamentos e drogas veterinárias, b) pesticidas, c) sanidade vegetal, d) ambiente, solos e residuos e, e) alimentos seguros. Estão na forja, desde 2019, dois importantes instrumentos jurídicos, nomeadamente, a Lei de Sanidade Animal e o Regulamento sobre os medicamentos, drogas e produtos veterinários.

Uma das principais conclusões deste relatório, é que em Moçambique os vários aspectos que têm impacto na RAM são abordados em diferentes actos legislativos. Isto está de acordo com as recomendações incluídas na Metodologia da FAO para a análise da legislação de RAM nos sectores alimentar e agrícola, que menciona como uma única legislação de RAM poderia criar fragmentação legal e duplicação desnecessária no quadro legal.

Moçambique não possui uma legislação sobre medicamentos e drogas veterinárias, o que representa uma enorme lacuna no desenvolvimento das actividades inerentes à sanidade animal quer terrestre quer aquática. A falta de aprovação da proposta de 2019 constitui uma grande fragilidade no sistema de prevenção da RAM.

A Sanidade Animal é governada pelo Decreto n. 26/2009, de 17 de Agosto, que aprova o Regulamento de Sanidade Animal que estabelece normas para a vigilância epidemiológica e controlo de doenças dos animais. A produção animal em é, institucionalmente, governada pelo

MADER, através da Direcção Nacional do Desenvolvimento Pecuário. No entanto, a informação sobre o uso e padrões de resistência relacionados com o uso de antibióticos em animais é muito limitada. A produção de animais aquáticos é regulada pelo Decreto n.º 99/2021 de 31 de Dezembro, que estabelece as normas e procedimentos para o exercício da actividade de aquacultura. Em relação ao uso de produtos químicos, rações e drogas veterinárias para a aquacultura, o regulamento remete a autorização pela autoridade competente para o controlo hígio-sanitário dos produtos da pesca (Inspeção de Pescado, IP através do Decreto n.º 80/2020 de 8 de Setembro).

As rações e forragens para animais terrestres encontram-se regulamentadas em instrumentos que já reivindicam sua actualização. Refirimo-nos ao a): Regulamento da Produção e Comercialização de Forragens para a Alimentação de Animais de 08 de Agosto de 1970 e al.b) Portaria n. 141/75, de 25 de Dezembro, que fixa as características que deverão possuir os alimentos simples e as rações preparadas para os animais domésticos que poderão ser comercializados para a respectiva alimentação.

Não obstante, são ainda encontradas, em alguns diplomas legais algumas disposições fazendo referência à rações. Refirimo-nos ao Regulamento de gestão de pesticidas, que proibe o transporte e armazenamento de pesticidas e rações, e ao Regulamento de Controle Higio-Sanitário dos Produtos da Pesca e Aquacultura.

A gestão de pesticidas é regulada pelo Decreto n. 6/2009 de 31 de Março de 2009, que aprova o Regulamento de Gestão de Pesticidas, que se aplica ao registo, produção, doação, comercialização, importação, exportação, embalagem, armazenamento, transporte, manuseio, uso e eliminação de pesticidas e auxiliares, por pessoas singulares ou colectivas, para agricultura, pecuária, silvicultura, protecção à saúde pública, doméstica e outros propósitos. Este decreto não inclui referências à RAM, mas poderia servir de base para regulamentar a autorização e utilização de antimicrobianos (antibióticos ou fungicidas) como pesticidas.

O quadro jurídico sobre os alimentos seguros e o ambiente, também mostram-se omissos quanto à fixação dos limites máximos de resíduos de pesticidas e medicamentos veterinários nos alimentos, e indicadores relevantes para a RAM no conjunto dos padrões de qualidade e emissão de efluentes, respectivamente.

O Decreto n. 18/2004, de 02 de Junho, que aprova o Regulamento sobre Padrões de Qualidade e Emissão de Efluentes, visando o controlo e manutenção dos níveis admissíveis de concentração de poluentes nos componentes ambientais é um importante instrumento legal. O Decreto define os parâmetros essenciais que devem caracterizar a qualidade de água, em função da sua categoria, de modo a averiguar a conformidade das águas com os padrões de qualidade e para permitir a determinação de esquemas de tratamento adequados.

Faz também menção ao destino final das descargas de efluentes líquidos industriais e efluentes domésticos. Em relação aos resíduos biomédicos, o Decreto n.º 08/2003, de 18 de Fevereiro, estabelece regras para a gestão de resíduos bio-médicos, com vista a salvaguardar a saúde e segurança dos trabalhadores das Unidades sanitárias, dos trabalhadores auxIliares e do público em geral e minimizar os impactos de tais resíduos sobre o ambiente.

Em relação à qualidade do solo, monitoramento de resíduos no solo ou prevenção de contaminação do solo e da água que abranja a contaminação com antimicrobianos é descrito no Decreto n. 18/2004, de 2 de Junho. De modo geral, os padrões de efluentes e emissões estabelecidos não integram os indicadores relevantes para a RAM, nem os mecanismos de transferência de poluentes.

A água é regulada pela Lei n. 16/91, de 31 de Agosto, que estabelece os princípios de gestão de águas, a necessidade de inventariação de todos os recursos hídricos existentes no país, o regime geral da sua utilização, as prioridades a ter em conta, os direitos gerais dos utentes e as correspondentes obrigações, entre outros.

A sanidade vegetal é regulada através do Decreto n. 05/2009, de 01 de Junho, que aprova o Regulamento de Inspecção Fitossanitária e de Quarentena Vegetal, visando entre outros aspectos assegurar a protecção e defesa fitossanitária, salvaguardando a saúde pública e o meio ambiente; certificar o processo de produção, importação e exportação de sementes e material vegetativo e colaborar com os organismos regionais e internacionais no âmbito da vigilância, prevenção e controlo zoo e fitossanitário.

Constatou-se a existência de um quadro institucional robusto para levar a cabo os mandatos sectoriais de implementação de medidas tendentes a reduzir a RAM, um mecanismo de coordenação constante do Plano Nacional Contra a Resistência Antimicrobiana, o que implica responsabilidades partilhadas entre instituições e entidades. O Grupo Interinstitucional para a prevenção e luta contra a RAM reúne-se sempre que as circunstâncias o exigirem, no entanto carece de um Regulamento Interno.

A avaliação do quadro legal sobre a resistência antimicrobiana revela-se, na sua dimensão, animal e ambiental, omissa em vários aspectos tendentes a prevenir a RAM. Outrossim, constatou-se que os medicamentos, drogas e produtos veterinários encontram-se desregulamentados em muitos dos seus aspectos, quer no que diz respeito às regras do seu ciclo de vida, registo, prescrição e monitorização dos efeitos adversos.

A robustez do quadro jurídico tendente a prevenir a RAM passa necessariamente por reforçar as legislações sectoriais, reforçando-se o quadro jurídico sobre os medicamentos, drogas e produtos veterinários, sobre as rações/forragem para os animais terrestres e aquáticos, definindo-se os

limites máximos de residuos (LMR) em alimentos, bem assim as regras sobre o exercício da profissão farmacêutica no que diz respeito às prescrições daqueles medicamentos.

Por fim, tendo em consideração que as questões conducentes à RAM incorporam diferentes componentes, e como mencionado acima, do ponto de vista de reforço legislativo, embora se reconheça a necessidade de reforço do quadro normativo sectorial, não se recomendada a adopção de legislação específica sobre RAM que procure abordar todos seus elementos num só instrumento, uma vez que tal poderia resultar ainda agudizar a fragmentação e, consequentemente, fragilização do mesmo.

Enquadramento

A RAM segundo a FAO é a capacidade dos microrganismos de persistirem ou multiplicam-se na presença de drogas projectadas para inibí-los ou matá-los, que se designam por antimicrobianos, que são utilizados no tratamento de doenças infecciosas causadas por microrganismos como bactérias, fungos, vírus e protozoários parasitas[1].

Estes medicamentos, substâncias naturais, produzidas por outros organismos, ou sintéticas que actuam inibindo ou causando a morte de micro-organismos, como fungos, bactérias e parasitas, são essenciais para preservar a saúde humana, a sanidade animal, vegetal, e o ambiente tal como preconizado pela Organização Mundial de Saúde que advoga *"One Health Approach".*

O uso excessivo ou inadequado dos antimicrobianos em humanos e animais, têm impacto sobre o ambiente e pode provocar o aparecimento de microrganismos resistentes à acção destes fármacos, tanto em bactérias comensais do intestino humano e animal, como em bactérias do meio ambiente, com a possível disseminação de genes de resistência entre diversas populações bacterianas.

O fenómeno da RAM, põe em perigo a eficácia do tratamento de uma série cada vez maior de infecções causadas por vírus, bactérias, fungos e parasitas. A RAM acontece quando os microorganismos (bactérias, fungos, vírus e parasitas) sofrem mutações ao serem expostos aos antimicrobianos e se tornam resistentes aos medicamentos utilizados para seu tratamento.

Em Moçambique, a falta de conhecimento, o uso irracional e abusivo de fármacos na sanidade animal e na produção de alimentos, são alguns dos principais factores que contribuem para o desenvolvimento da RAM, representando uma ameaça sanitária global crescente.

O uso clínico dos antimicrobianos exerce papel selecionador das cepas resistentes e, provavelmente, é a principal causa da resistência, sobretudo a observada naquele ambiente, onde o uso destas drogas é maior. Contribuição importante para a emergência da RAM, associada ao uso indevido e excessivo dos medicamentos, drogas e productos veterinários, também é dada por outros segmentos.

Na sanidade animal, pouca informação está disponível sobre o uso e padrões de RAM relacionados com o uso de antimicrobianos que são utilizados não apenas para tratar animais doentes, mas também saudáveis para prevenir doenças e para promover o crescimento[2]. No geral, embora os níveis de utilização de antimicrobianos na comunidade possam ser altos, há

[1] https://www.fao.org/antimicrobial-resistance/background/what-is-it/en/

[2] GARP - Moçambique. 2015. Análise Situacional e Recomendações: Uso e Resistência aos Antibióticos. Washington, DC e Nova Deli Centro para estudo de Dinâmica de Doenças, Economia e Política (CDDP).

pouca informação sobre os padrões de RAM, havendo indicação de que aproximadamente 54% dos utentes compram os antimicrobianos sem prescrição de um profissional[3].

Actualmente, a RAM tem sido abordada com urgência na saúde pública, na produção animal, agrícola e no meio ambiente visto que põe em risco o tratamento eficaz de infecções causadas por bactérias, parasitas, vírus e fungos, resultando em enfermidades mais prolongadas e com maior mortalidade. Embora o desenvolvimento da RAM seja um fenômeno natural dos microrganismos, actualmente verifica-se uma maior pressão selectiva e disseminação por:

i. mau uso de medicamentos antimicrobianos para a saúde humana, produção animal e vegetal;
ii. programas inadequados ou inexistentes de prevenção e controle de infecções, o que favorece a transmissão da resistência entre os microrganismos e a exposição de indivíduos a microrganismos resistentes;
iii. antimicrobianos de má qualidade;
iv. fraca capacidade laboratorial;
v. vigilância e monitorização inadequados; e
vi. insuficiente regulamentação e fiscalização do uso dos antimicrobianos.

Embora tenha havido esforços significativos em todo o mundo para aumentar a consciencialização para lidar com a emergência representada pela RAM, as partes interessadas e, em particular, os governos, devem renovar seus compromissos políticos para enfrentar essa crescente ameaça à saúde humana, sanidade animal, vegetal e ambiental.

O combate à emergência e propagação de bactérias resistentes aos antimicrobianos e ao desenvolvimento de novos mecanismos de resistência requer uma abordagem conjunta de vários segmentos governamentais e da sociedade, além de envolver a necessidade de proposição de políticas que resultem em um amplo investimento em pesquisas, na aquisição de tecnologias e no desenvolvimento de recursos humanos.

[3] Cambaco, O. et al. 2020. Community knowledge and practices regarding antibiotic use in rural Mozambique: where is the starting point for prevention of antibiotic resistance? BMC Public Health, 20:1183. BMC Public Health. 20:1183. doi.org/10.1186/s12889-020-09243-x

1. OBJECTO E OBJECTIVO DA AVALIAÇÃO

A presente avaliação incide sobre todo quadro jurídico aplicável ao uso de antimicrobiano e RAM, e tal inclui todos os instrumentos jurídicos nacionais, desde a Constituição da República de Moçambique aos Diplomas Ministeriais e procedimentos aplicáveis.

A avaliação acima referida visa identificar as principais lacunas existentes no quadro jurídico-institucional do uso de antimicrobianos e RAM, e recomendar soluções com vista a suprí-las. Para o efeito, e especificamente almeja-se:

- Analisar o quadro regulador de antimicrobiano e RAM.
- Identificar as instituições/entidades relevantes para a implementação da legislação antimicrobiana.
- Identificar e analisar lacunas na legislação relativa à antimicrobiano e RAM, e respectivas soluções legais para alcançar os objectivos político-estratégicos.
- Apresentar elementos a incorporar e modificar no actual quadro jurídico e analisar as diferentes opções para aprovação do quadro proposto.

2. ACTIVIDADES DESENVOLVIDAS

- Encontro de arranque da missão com a equipa nacional da FAO-Moçambique, no qual passamos em revista a proposta do plano de trabalho.
- Encontro de coordenação com a equipa técnica e de apoio da FAO-Roma, no qual fez-se a apresentação do plano de trabalho.
- Recolha da legislação pertinente para a compleição da avaliação do quadro jurídico de prevenção e luta contra a RAM.
- Identificação dos parceiros relevantes no combate a RAM.
- Realização de um workshop de auscultação dos principais actores intervenientes na concepção e implementação do quadro jurídico sobre o uso de antimicrobianos e RAM.

3. QUADRO INSTITUCIONAL RELEVANTE PARA A IMPLEMENTAÇÃO DA LEGISLAÇÃO SOBRE O USO ANTIMICROBIANOS E RESISTÊNCIA ANTIMICROBIANA

3.1. Papel do Ministério da Saúde

O Ministério da Saúde (MISAU) é o Órgão Central do aparelho do Estado que, de acordo com a Resolução n. 4/2017, de 26 de Maio, tem atribuições de promover os cuidados de saúde pública e o quadro normativo que governa, entre outros aspectos, o uso de antimicrobianos para o consumo humano, já que a prescrição e uso desregrado destes representa um grande desafio, porquanto associado à RAM, sobretudo nas doenças crônicas como HIV/SIDA, Tuberculose, doenças diarréicas, meningite bacteriana aguda etc. Por outro lado, a RAM arrasta consigo um aumento de custos para o MISAU, já que os pacientes tendem a permanecer por longos períodos hospitalizados e, por outro, deve-se reorientar fundos para aquisição de medicamentos de segunda ou terceira linha, em regra, mais caros. Embora as intervenções do MISAU no domínio

da RAM estejam voltadas para a componente humana, existem momentos em que o MISAU é chamado a pronunciar-se quanto ao fabrico, importação e comercialização de medicamento, drogas e produtos veterinários, sobretudo os que podem ter efeitos cancerígenos como, por exemplo, o "verde malaquita" usado no tratamento de parasitas, infecções por fungos e bactérias em peixes. As unidades orgânicas abaixo discriminadas desempenham funções orientadas para pôr cobro aos fenômenos acima descritos:

A. AUTORIDADE NACIONAL REGULADORA DE MEDICAMENTO, IP (ANARME, IP)

No que diz respeito à temática de uso de antimicrobianos e RAM, a ANARME, IP, assume grande relevância na implementação dos objetivos consagrados na Lei n. 12/2017, de 08 de Setembro, que aprova a Lei de Medicamentos, Vacinas e Outros Produtos Biológicos para o uso Humano. As competências da ANARME, IP, para além de estarem previstas no artigo 7 da supracitada Lei, também constam do respectivo Estatuto Orgânico aprovado pelo Decreto n. 115/2020, de 31 de Dezembro, e que se resume nas seguintes: a) propor ao Governo a definição ou ajustamento de políticas de medicamento, vacinas e produtos biológicos de uso humano e velar pela sua execução; b) pronunciar-se e dar parecer, nos termos da presente Lei, sobre todas as questões relativas ao medicamento, vacinas e outros produtos Biológicos para o uso humano; c) desempenhar as funções de regulação, supervisão, fiscalização e sancionamento no âmbito do medica-mento, vacinas e produtos biológicos de uso humano e de saúde; d) elaborar e manter actualizado o Formulário Nacional do Medicamento, Lista dos Medicamentos Essenciais e de Outros Insumos; e) avaliar e recomendar à decisão do Ministro que superentende a área da Saúde, sobre os pedidos de registo de medicamentos e emitir os competentes certificados; f) controlar a qualidade dos medicamentos em circulação no País; g) proceder a instrução de processos relativos aos pedidos de licenciamento na área farmacêutica; h) promover a investigação e produção nacional de medicamento, vacinas e produtos biológicos de uso humano; i) assegurar o controlo de estupefacientes e substâncias psicotrópicas conforme o estabelecido nas convenções internacionais; j) proceder ao registo dos Profissionais da Área Farmacêutica; k) garantir a farmacovigilância; l) propor a fixação de preços de medicamento, vacinas e produtos biológicos; m) promover o uso racional de medicamento; n) organizar e realizar a inspecção farmacêutica. As demais competências constam do n. 2 do Decreto supracitado.

B. Direcção Nacional de Saúde Pública

Esta Direcção assume relevância no contexto do uso de antimicrobianos, prevenção e combate da RAM, em particular para a saúde humana, e excepcionalmente para a sanidade animal e vegetal, desde que os antimicrobianos possam ter alguma interferência na saúde humana. Esta unidade orgânica encontra-se estruturada nos termos do artigo 8 da Resolução n. 4/2017, de 26 de Maio, em dois domínios importantes, nomeadamente: (a) domínio de promoção e protecção de saúde, no qual compete-lhe propor a regulamentação e controle da publicidade, da comercialização, do consumo de substâncias potencialmente nocivas à saúde ou a contaminação do ambiente em coordenação com áreas afins; e (b) domínio de prevenção e controlo de doenças, no qual compete-lhe desenvolver e implementar estratégias, normas e programas de

prevenção e controlo das epidemias, endemias e outras doenças transmissíveis e não transmissíveis; estabelecer e manter um sistema de informação epidemiológica para detecção de doenças de notificação obrigatória e outras.

3.2. Papel do Ministério da Agricultura e Desenvolvimento Rural

A Resolução n. 3/2020, de 13 de Março, estabelece no seu artigo 3 as seguintes competências do Ministério da Agricultura e Desenvolvimento Rural (MADER): (a) na área da Agricultura, nomeadamente, garantir a defesa sanitária vegetal, controlo fitossanitário e biossegurança; (b) na área da Pecuária, nomeadamente, garantir a defesa da sanidade animal, incluindo os aquáticos, controle zoossanitário e saúde pública. Estas competências são exercidas, no MADER, pela Direcção Nacional de Sanidade Agropecuária e Biossegurança (DNSAB), e pela Direcação Nacional de Desenvolvimento Pecuário (DNDP), de acordo com a estrutura do MADER estabelecida no artigo 4 da supracitada resolução. Compulsadas competências destas duas Direcções, constata-se que a Resolução é omissa no que diz respeito a entidade encarregue de executar tarefas atinentes aos medicamentos, drogas e produtos veterinários. É possível constatar que, nos termos do artigo 12 da Resolução citada, são funções da DNSAB garantir o controlo higio-sanitário dos produtos de origem animal, estabelecer mecanismos de vigilância de doenças de animais com impacto na economia e na saúde pública, velar pelo cumprimento da legislação sobre produção pecuária e sanidade animal, e exercer as competências atribuídas à Autoridade Veterinária, assegurar a protecção e defesa fitossanitária, salvaguardando a saúde pública e o meio ambiente, estabelecer mecanismos de vigilância, prevenção, controlo e erradicação de doenças e agentes de doenças fitossanitárias, com impacto da economia e na saúde pública, de onde resulta a pertinência de coordenação com as unidades orgânicas do MISAU. Por outro lado, nas competencias da DNDP assume relevância para a RAM, nos termos do artigo 8 da Resolução acima, a garantia da defesa de sanidade animal, e establecer mecanismos de prevenção, controlo e erradicação de doenças e agentes de doença dos animais.

3.3. Papel do Ministério do Mar, Águas Interiores e Pesca

O Decreto Presidencial n. 17/2015, de 25 de Março estabelece no seu artigo 3 as competências do Ministério do Mar, Águas Interiores e Pesca (MIMAIP) designadamente na área de inspeção e certificação higio-sanitária dos produtos de origem aquática as seguintes: propor a definição de políticas, estratégias e planos no que respeita à qualidade higio-sanitária dos produtos da pesca; proceder ao licenciamento das unidades produtivas, a inspecção e certificação sanitária dos produtos de origem aquática destinados ao mercado interno e a exportação, assim como dos produtos importados. Estas competências são exercidas pela Inspecção do Pescado (IP), criada através do Decreto n. 71/2019, de 26 de Agosto, que estabelece no artigo 5, as competências de proceder ao licenciamento sanitário e à inspecção de unidades produtivas de manuseamento e processamento de produtos da pesca e de rações para animais aquáticos; inspeccionar e proceder à certificação sanitária das rações para animais aquáticos, em coordenação com a Autoridade Veterinária Competente; promover a monitorização e certificação da sanidade dos organismos aquáticos, em coordenação com a Autoridade Veterinária Competente; realizar programas de pesquisa e avaliação relacionados com os perigos e riscos nos organismos

aquáticos, produtos da pesca, e rações para animais aquáticos; realizar a verificação e auditoria do cumprimento e da conformidade dos requisitos hígio-sanitários das unidades produtivas, em todas as fases da cadeia produtiva, incluindo a distribuição e comércio; e assegurar o processo de certificação do corpo de inspecção do pescado e manter o respectivo nível de certificação.

3.4. Papel do Ministério da Terra e Ambiente

A Resolução n. 10/2020, de 05 de Maio estabelece no seu artigo 3 as competências do Ministério da Terra e Ambiente (MITA) designadamente área do Ambiente as seguintes (a): propor a aprovação de legislação, políticas, estratégias e normas para as acções de preservação da qualidade ambiental; (b) estabelecer medidas de prevenção da degradação e controlo da qualidade ambiental; (c) promover iniciativas de gestão adequada de resíduos sólidos e efluentes. Estas competências são exercidas, no MITA pela Direcção Nacional do Ambiente, nos termos do artigo 10 do supracitado diploma. A implementação destas competências passam necessariamente por uma maior coordenação, regulamentação e promoção de acções tendentes a assegurar a qualidade do ambiente, que pode ser posto em causa devido a má gestão, uso e descarte indiscriminado dos antimicrobianos.

4. Quadro Político de uso de antimicrobianos

A Constituição da República de Moçambique (CRM) de 2004, alterada pela Lei n. 1/2018, de 12 de Junho, consagra, no artigo 89, o direito à saúde, nos seguintes termos: “todos os cidadãos têm o direito à assistência médica e sanitária, nos termos da lei, bem como o dever de promover e defender a saúde pública” e define as linhas mestras ao legislador ordinário e às instituições do Estado, para dar primazia a um dos grandes constrangimentos identificados, que é a importação, distribuição e circulação descontrolada e venda informal de antimicrobianos, quando no artigo 116, n. 5 atribui ao Estado a competência de “promover, disciplinar e controlar a produção, a comercialização e o uso de produtos químicos, biológicos, farmacêuticos e outros meios de tratamento e de diagnóstico”. No plano internacional, Moçambique é membro da OMS, FAO e OMSA, que adoptaram directrizes que orientam os Estados Membros a desenvolver planos nacionais e implementar medidas tendentes reduzir a RAM.

Não obstante, a orientação constitucional e das directrizes internacionais o país ainda não tem uma política específica para o uso de antimicrobianos e prevenção da RAM. Porém, em 2019, Moçambique adoptou um Plano Nacional de Acção Contra a Resistência Antimicrobiana, que contém três pilares, seis intervenções estratégicas, e com acções prioritárias para a saúde pública e sanidade animal e vegetal.

O Plano reconhece a necessidade de rever, actualizar ou desenvolver regulamentos relacionados com a gestão de medicamentos, tanto para a saúde humana como animal, a fim de encorajar a utilização apropriada e racional dos antimicrobianos. Para este efeito, são indicadas acções concretas, tais como o controlo e registo da qualidade dos antimicrobianos na saúde humana e animal, a análise de normas do uso de antimicrobianos em saúde animal, incluíndo a revisão de quem está autorizado a prescrever ou administrar um antibiótico em animais, o fortalecemento

do controlo sobre a importação ilegal dos antimicrobianos nas fronteiras, ou a elaboração de egulamentos que estabeleçam os critérios para o licenciamento de importadores, vendedores por atacado, fabricantes e moinhos de aditivos alimentares, entre otras.

5. Análise legal das áreas relevantes e com impacto na RAM em Moçambique

Através da Lei n. 12/2017, de 08 de Setembro, foi aprovada a Lei de Medicamento, Vacinas e Outros Produtos Biológicos para o Uso Humano, visando ajustar ao estágio de desenvolvimento sócio-económico e do mercado, marcado à data por contrafacção e o contrabando de produtos farmacêuticos. A lei em referência foi concebida para ser aplicada aos processos de produção, distribuição, uso e disponibilização eficiente e segura, bem como a garantia da qualidade de medicamentos, vacinas, produtos biológicos e de saúde para os cidadãos.

Relativamente aos animais, existe uma proposta de regulamento de 2019, sobre os medicamentos, drogas, e produtos veterinários cujo objecto é estabelecer as normas e procedimentos aplicáveis para a autorização de introdução no mercado, suas alterações, a produção, importação, exportação, armazenamento, comercialização, rotulagem, informação, publicidade, farmacovigilância e a utilização dos mesmos para o uso animal e a respectiva inspecção. Actualmente, é no Regulamento de Sanidade Animal aprovado pelo Decreto n. 26/2009, de 17 de Agosto, onde encontramos algumas disposições sobre o uso de medicamentos, drogas e produtos veterinários, que será objecto de análise neste relatório.

Finalmente, relativamente às substâncias aplicadas em produtos agrícolas, o Decreto n. 6/2009, 31 de Março, aprova o regulamento sobre a gestão de pesticidas. A este propósito, este instrumento aplica-se a qualquer substância ou mistura de substâncias destinada à prevenção, destruição ou controlo de qualquer peste, incluindo vectores de doenças humanas e animais, espécies indesejáveis e maléficas de plantas ou animais com efeitos perniciosos sobre a produção ou que de algum modo interferem na produção, processamento, armazenagem, transporte e colocação no mercado de alimentos, produtos agrícolas, rações para animais ou substâncias que possam ser administradas em animais para o controlo de insectos ou outras pestes, com vista a protecção dos mesmos contra a deterioração durante a armazenagem e transporte.

Estes três instrumentos preenchem o quadro jurídico nacional de regulação dos medicamentos, essenciais para regular o uso adequado de antimicrobianos e reverter o quadro de RAM. A seguir passamos a analisar de forma transversal os principais aspectos que podem afectar o quadro de esforços nacionais tendentes a prevenir, combater e reverter o cenário de RAM.

ÁREAS JURÍDICAS RELEVANTES PARA RAM EM MOÇAMBIQUE

1) MEDICAMENTOS, DROGAS E PRODUTOS VETERINÁRIOS

A. ENQUADRAMENTO

A regulamentação dos medicamentos e drogas veterinárias é uma das áreas mais críticas no combate ao desenvolvimento e disseminação da RAM na alimentação e na agricultura. A regulamentação trata principalmente da autorização, produção/fabrico, importação, distribuição

de exportação, venda e uso de medicamentos e drogas veterinárias. É importante que a regulamentação em todas as fases seja suficientemente robusta. As deficiências na regulamentação podem contribuir para o aumento do desenvolvimento e disseminação da RAM.

B. ANÁLISE DA LEGISLAÇÃO NACIONAL SOBRE MEDICAMENTOS, DROGAS E PRODUTOS VETERINÁRIOS

Moçambique não possui uma legislação específica sobre medicamentos, drogas e productos veterinários, o que representa uma enorme lacuna no desenvolvimento das actividades inerentes à sanidade animal quer terrestre quer aquática. Neste contexto, e em linha com os esforços do governo liderado pelo MADER através da Autoridade Veterinária, a Direcção Nacional de Sanidade Animal e Biosegurança, (DNSAB) como a autoridade competente, foi esboçada a Proposta de Regulamento de Drogas e Produtos Veterinários no ano de 2019. No entanto, desde então, esta proposta não mereceu quaisquer desenvolvimentos dignos de realce e permanece no estágio da sua concepção. Os medicamentos, produtos biológicos, hormonas e drogas destinados a animais encontram-se regulados de forma muito superficial no Decreto n. 26/2009, de 17 de Agosto que aprova o Regulamento de Sanidade Animal (RSA). O Regulamento de Controlo Higio-Sanitário dos productos da pesca e aquacultura, aprovado pelo Decreto n. 80/2020, de 08 de Setembro, contempla referências sobre os LMR nestes produtos, e remete a sua definição ao plano nacional de controlo de residuos.

É sobre estes instrumentos que fazemos as nossas análises. A falta de aprovação da proposta de 2019 constitui uma grande fragilidade no sistema de prevenção da RAM. Importa referir que a análise procurará sempre confrontar o regime neles consagrado.

I. Definição

O artigo 2 do RSA contém uma longa lista de definições-chave (animal, certificado sanitário internacional, incidência, quarentena, vigilância epidemiológica, etc. para citar apenas alguns). No entanto, nem todas estas definições incluídas no glossário estão alinhadas com as da OMSA. O RSA, embora não defina o que sejam medicamentos veterinários, ele contém no seu glossário a definição de *Produtos biológicos*, qualificando-os como sendo "*reactivos biológicos, soros, vacinas e material genético de origem microbiana, utilizados na investigação, diagnóstico, tratamento e prevenção de doenças*". Esta definição é tão ampla que incorpora em si mesma as drogas, medicamentos e produtos veterinários. O Regulamento de Contriolo Higio Sanitário dos Produtos da Pesca e Aquacultura, embora faça referência aos medicamentos veterinários, quando se refere a necessidade de adopção de um plano nacional de controle de residuos, mas ambos não contemplam a sua definição em seu glossário. A proposta de Regulamento (2019), para além de definir medicamentos como sendo "*substância, drogas, vacinas e biológicos disponibilizados para a prevenção, tratamento, controlo ou erradicação de doenças*", também define *medicamento veterinário de referência ou original*, *medicamento veterinário genérico* e *medicamento veterinário imunológico*.

II. Autoridade competente

O Decreto n. 26/2009, de 17 de Agosto que aprova o RSA define esta autoridade como sendo o Ministério que superintende a área da agricultura, através da Direcção Nacional dos Serviços de Veterinária, o que é depois retomado no artigo 4. Para cumprir este mandato, as competências da autoridade veterinária estão estabelecidas no artigo 7, e, são estabelecidos como organismos de execução, no artigo 6, a Direcção Nacional dos Serviços de Veterinária e as entidades do Ministério que superintende a área da agricultura a quem forem delegadas competências. Os Governos Provinciais e Distritais devem prestar à Autoridade Veterinária toda a colaboração e apoio necessários e comunicar qualquer alteração do estado de saúde dos animais da respectiva área de jurisdição (artigo 8).

A autoridade veterinária pode delegar competências a entidades a nível local, mas o Director Nacional dos Serviços de Veterinária tem o poder de revogar ou suspender a eficácia de uma decisão tomada ou instrução transmitida pela entidade a nível local a quem foi delegada competência (artigo 6). Relativamente aos animais aquáticos, o Regulamento de Controlo Higio-Sanitário define, no artigo 6, a Inspecção do Pescado, IP como a Autoridade competente, para implementar o controlo oficial para avaliação dos níveis de residuos de drogas e medicamentos veterinários nos productos da aquacultura.

III. Registo/autorização

Enquanto o RSA 17roducto pelo Decreto n. 26/2009, refere-se ao registo de animais (artigo 38), e de explorações (artigo 39), em relação ao registo de medicamentos veterinários, apenas foi encontrada uma 17roductos17 geral, no artigo 104 que preconiza *"A importação, registo, preparação e venda de medicamentos, 17productos biológicos e químicos destinados a animais, com a excepção do registo de drogas acaricidas e carracicidas, ficam sujeitos ao licenciamento",* o que pressupõe que o licenciamento seja o pré-requisito para o registo. Portanto, é possível 17roduct que existe uma obrigação de autorização e registo de medicamentos, 17roductos biológicos e químicos destinados a animais neste RSA supra.

A Proposta de Regulamento de Drogas, Medicamentos e Produtos Veterinários 2019 (PRDMPV, 2019) pretende ser um instrumento que engloba todas as questões e de forma transversal, relativas a esta matéria evitando-se a assim o seu tratamento fragmentários, e é neste contexto que prevê a consagração, no artigo 8 n.1 (2019), da obrigatoriedade de registo junto da Autoridade Veterinária, de todas as drogas, medicamentos e outros produtos de uso veterinário produzidos, importados e comercializados em território nacional. O artigo 23 da Proposta (2019), obriga a manutenção de registos por parte dos estabelecimentos de venda de medicamentos veterinários à disposição das autoridades para efeitos de controlo e fiscalização, de todas as transacções de medicamentos veterinários fornecidos mediante receita médico -veterinária que inclua, para cada transacção de entrada ou saída. O artigo 25 prevê ainda a existência de um livro de registo para efeitos de controlo e fiscalização de todas as transações de medicamentos veterinários que conterão: *data da transação, nome, número do lote de produção, quantidade recebida ou fornecida, nome e endereço do fornecedor ou do destinatária e cópia da receita médico-veterinária*, sendo considerada infracção a venda, por pessoa ou entidade não autorizada, de produto veterinário sujeito a prescrição médica. O artigo 72 da citada Proposta,

realça que as informações obrigatórias sobre o registo de utilização e detenção ou posse de drogas, medicamentos e produtos veterinários encontram-se descritas nos números 1 a 7.

IV. Lista de medicamentos essenciais

Apesar do RSA não fazer referência a uma lista de medicamentos, drogas e produtos veterinários, através do Diploma Ministerial n. 57/86, de 29 de Outubro, foi aprovado o Formulário Nacional para Veterinária visando pôr termo à má gestão de medicamentos e disciplinar o seu uso e criar meios que facilitem o trabalho dos veterinários e outros trabalhadores. Já o regulamento de controlo higio-sanitário dos productos da pesca e aquacultura, por referência ao plano nacional de controlo de residuos e contaminantes, embora não consagre expresamente uma lista de medicamentos veterinários aprovados para aplicação em productos de pesca, adopta a lista do Estado de Mercado.

A Proposta (2019), na sua al. e), do artigo 7, obriga a Autoridade Veterinária tornar públicas as determinações sobre a lista de medicamentos essenciais, formulário nacional de medicamentos, a farmácia padrão e os medicamentos proibidos, ouvida a entidade que superintende a saúde humana.

V. Controlo de qualidade

O controlo de qualidade é tratado no capítulo VI do RSA. O artigo 103 prevê que o controlo dos produtos de origem animal visa garantir que os mesmos tenham as características e qualidade adequadas ao fim mencionado no certificado sanitário. O controlo dos produtos referidos é realizado pela Autoridade Veterinária, que pode recorrer a laboratórios de referência para a tomada de decisão.

VI. Rotulagem, embalagem e publicidade

O RSA aprovado pelo Decreto n. 26/2009, estabelece no seu artigo 29 que as embalagens devem ser rotuladas com indicação expressa da respectiva origem, tipo e quantidade do produto, data de fabrico, data de expiração e período de validade, e tratando-se de importação de medicamentos, produtos biológicos e patológicos considerados infectantes, tais devem ser acondicionados de modo a evitar qualquer possibilidade de contaminação exterior e embalados de acordo com as regras da OMS. No entanto, o artigo 29 do RSA é omisso quanto às questões de reembalagens, a língua utilizada em embalagens, obrigatoriedade de colocação no rótulo a informação como números de série ou marcas de autenticação, para evitar que os medicamento, drogas e produtos veterinários não registados ou falsificados entrem no mercado.

Na Proposta (2019), verifica-se, em particular no artigo 35 n. 2, a previsão de proibição de fornecer ao público medicamentos e drogas veterinárias em embalagens que não estejam rotuladas e/ou que não incluam prospecto de acordo com as suas disposições. A proposta prevê ainda consagrar algumas normas sobre as embalagens e rotulagem. Os artigos 36 a 42 abordam questões relacionadas à língua a ser usada na rotulagem e prospecto, sinais ou imagens nas rotulagem e prospecto, e informações contidas nas embalagens.

A publicidade só é autorizada para os medicamentos veterinários sujeitos a receita médico-veterinária desde que seja fundamentada em trabalhos de índole científica de reconhecida idoneidade, devendo mencionar as respectivas referências bibliográficas. Os princípios e requisitos de publicidade estão previstos nos artigos 46 e 47 da Proposta (2019).

VII. Prescrição venda ou dispensa de medicamentos veterinários

O RSA e o Regulamento para o Controlo Hígio-Sanitário dos Produtos da Pesca são omissos quanto a esta matéria bem como quanto à categorização dos prescritores de medicamentos, drogas e produtos veterinários.

A Proposta de (2019) não prevê a proibição de venda de drogas e produtos veterinários sem prescrição, nem menciona locais específicos para a venda desses produtos. No artigo 15 n. 8 da referida Proposta prevê-se que o importador seja o responsável jurídico pelas drogas, medicamentos e produtos de uso veterinário por si importados e deve possuir um responsável técnico, ou seja, um médico veterinário que assegure a qualidade das actividades desenvolvidas. A Proposta de 2019 vai ainda no sentido de proibir a venda ou dispensa de antimicrobianos sem prescrição médica (artigo 74 al. g), mas é omissa relativamente às categorias autorizadas de prescritores que podem prescrever antimicrobianos no sector de saúde animal e também quanto à obrigatoriedade de manter e relatar registos de prescrições relacionadas aos medicamentos, drogas e produtos veterinários.

VIII. Venda

Em relação a venda, o RSA, especificamente no artigo 104, estabelece que a venda de medicamentos, produtos biológicos e químicos destinados a animais estão sujeitos a licenciamento ou parecer técnico prévio da Autoridade Veterinária, excepto tratando-se de drogas acaricidas e carracicidas, e mais não se consagra à semelhança da Proposta (2019), que prevê que os estabelecimentos de produção e/ou distribuição e venda de drogas, medicamentos e outros produtos de uso veterinário possuam as características e os equipamentos adequados à actividade, tendo em conta o articulado nº1 do artigo 2 do Decreto 34/2013 de 2 de Agosto (Regulamento de Licenciamento Comercial). Os ns. 3 e 4 do artigo 20 da Proposta (2019), realçam que os estabelecimentos de destino das drogas, medicamentos, produtos veterinários, e princípios activos devem estar licenciados/aprovados pela autoridade que detiver a competência para o efeito e a Autoridade Veterinária é responsável pela verificação das condições aplicáveis aos estabelecimentos, antes do início da actividade e periodicamente de acordo com uma análise do risco. O exercício de distribuição a grosso e a retalho de drogas, medicamentos e produtos veterinários obriga a manutenção, em serviço, de um responsável técnico (médicos veterinários, bioquímicos, químicos ou farmacêuticos) e também os demais membros do pessoal com conhecimentos técnicos para garantir a qualidade das actividades desenvolvidas.

Os nrs 4 e 5 do artigo 15 da Proposta (2019), prevêem a proibição da importação, venda e aquisição de drogas, medicamentos e produtos veterinários de qualidade desconhecida, falsificados (contrafeitos) e fora do prazo ou com prazo inferior a 3 meses, e de produtos perigosos se as medidas de controlo tais como restrição para certificação de usuários ou medidas

do género sejam insuficientes para assegurar que tal produto possa ser manuseado com risco aceitável para o utilizador. Em relação às penalidades, a Proposta (2019), prevê multas e outras penas aplicáveis às diversas infracções cometidas no decurso da venda de medicamentos veterinários não registrados, de produtos banidos ou fora de prazo e de produto veterinário sujeito a prescrição médica por uma pessoa ou entidade não autorizada. Esta proposta devia fazer referência à proibição de venda de producto veterinário sem prescrição quando tal for exigida bem como a proibição de venda do mesmo por pessoa não autorizada.

IX. Uso

Não existe uma legislação específica sobre o uso de medicamentos e productos veterinários de modo a asegurar que os níveis de residuos de antimicrobianos em productos de origen animal não represente um risco ao consumidor. No entanto, o artigo 104 do RSA, prevê a proibição do uso de hormonas e de promotores de crescimento na produção animal, ficando a sua utilização para fins terapêuticos sujeita a autorização, fiscalização e controlo da Autoridade Veterinária, podendo a sua administração ser autorizada com base em normas estabelecidas pela mesma. Cabe ainda à Autoridade Veterinária determinar o uso de tripanocidas, sua alternância, bem como aprovar o regime de tratamentos de acordo com as necessidades de defesa sanitária impostas pelas condições específicas na área.

Outrossim, o artigo 114 do RSA contempla casos que carecem de autorização escrita da Autoridade veterinária, como é o caso do uso de vacina, soro, toxina, antitoxina, antígeno com a prevenção, tratamento ou cura de qualquer doença animal, ou ectoparasita, ou para manutenção ou melhoramento da saúde, crescimento, produção ou capacidade de trabalho de qualquer animal.

X. Fabrico

Tanto a RSA quanto a Proposta (2019), são omissos em relação a registos de produção e quanto ao cumprimento de regras de produção de medicamentos, drogas e produtos veterinários tendentes a minimizar a contaminação ambiental com antimicrobianos resultantes do processo de produção. A legislação também é omissa em relação à proibição ou restrição à reembalagem dos medicamentos ou drogas veterinárias e o tratamento dos resíduos resultantes da produção. A PRDMPV (2019), prevê no artigo 20 que os estabelecimentos de produção e/ou distribuição e venda de drogas, medicamentos e outros produtos de uso veterinário devem possuir características e equipamentos adequados à actividade, tendo em conta o articulado nº1 do artigo 02 do Decreto 34/2013 de 2 de Agosto (Regulamento de Licenciamento Comercial). ademais, os estabelecimentos de destino das drogas, medicamentos, produtos veterinários, e princípios activos devem estar licenciados/aprovados pela Autoridade Veterinária.

2) SANIDADE ANIMAL E PRÁTICAS DE PRODUÇÃO PARA PREVENIR DOENÇAS EM ANIMAIS TERRESTRES E AQUÁTICOS

A. ENQUADRAMENTO

O maneio adequado da saúde animal, com foco na prevenção de doenças, pode contribuir para reduzir a necessidade de antimicrobianos e consequentemente minimizar o desenvolvimento e disseminação da RAM. Uma boa legislação veterinária, alinhada com os padrões internacionais de referência aprovados pela OIE, pode ter um impacto positivo na prevenção de doenças. A legislação veterinária também é necessária para reconhecer o mandato e as funções da autoridade veterinária e para implementar a "cadeia de comando", onde as responsabilidades e poderes da autoridade, desde o nível central até aos implementadores da legislação a nível provincial, distrital e da localidade, são claramente definidos.

B. ANÁLISE DA LEGISLAÇÃO VETERINÁRIA

I. Sanidade Animal

A Sanidade Animal em Moçambique é governada pelo Decreto n.o 26/2009 de 17 de Agosto que aprova o Regulamento de Sanidade Animal. O regulamento estabelece normas para a vigilância epidemiológica e controlo de doenças dos animais em Moçambique e tem como objectivos: a) proteger a saúde pública; b) proteger o mercado nacional e de exportação de animais, produtos de origem animal e outros que possam ser afectados directa ou indirectamente por doenças dos animais; c) servir de base para levar a cabo a vigilância epidemiológica, controlo e erradicação de doenças de grande importância económica e/ou para a saúde pública; d) servir de base para a compensação por perdas causadas por doença dos animais, e, e) servir de base para a observância das condições do bem-estar animal.

A Autoridade Veterinária responsável pela aplicação das normas do Regulamento é o Ministério que superintende a área da agricultura, através da Direcção Nacional de Veterinária. São competências da Autoridade Veterinária através do artigo 7: a) Delegar competências a entidades do Ministério que superintende a área da agricultura a nível local; b) Garantir a salubridade dos produtos de origem animal e coordenar o funcionamento da inspecção hígio sanitária e controlo veterinário na produção e processamento dos produtos de origem animal; c) Elaborar os programas e adoptar normas com vista a levar a cabo a vigilância, controlo e erradicação das doenças infecto-contagiosas e parasitárias dos animais; d) Definir, coordenar e avaliar a aplicação das normas inerentes aos programas de vigilância, controlo e erradicação das doenças infecto-contagiosas e parasitárias dos animais; e) Manter e desenvolver o sistema de informação epidemiológica; e) Tornar públicas as determinações relativas às doenças da Lista de doenças de declaração obrigatória.

A nível provincial e distrital, os governos têm a responsabilidade de prestar à Autoridade Veterinária toda a colaboração e apoio necessários para o cumprimento do Regulamento e comunicar à Autoridade Veterinária qualquer alteração do estado de saúde dos animais da respectiva área de jurisdição.

A importação, circulação e trânsito de animais, seus produtos, subprodutos, despojos, forragens, produtos vegetais, veículos e contentores para o transporte de animais ou produtos é descrita no capítulo II. As providências aplicáveis à defesa sanitária, relativas ao registo e concentração dos animais, registo de alterações, vacinações e outros programas sanitários de cumprimento obrigatório, banhos carracicidas, quarentena, locais de abate, matança e inspeção de animais e carne, e beneficiações são descritas no capítulo III.

O capítulo IV trata de questões relacionadas a medidas aplicáveis às doenças de declaração obrigatória. As medidas sanitárias e locais de confinamento para animais selvagens estão descritas no capítulo V e por fim o controle dos produtos de origem animal e para uso veterinário, nomeadamente a qualidade de produtos, utilização de medicamentos, produtos biológicos, hormonais e drogas são descritas no capítulo VI.

II. Produção Animal

A produção animal em Moçambique é governada pelo Ministério da Agricultura e Desenvolvimento Rural (MADER), através da DNDP através da resolução nr 3/2020 de 13 de Março que aprova o estatuto orgânico do MADER. As funções da DNDP estão descritas no artigo 8 da Resolução supracitada.

Por outro lado, a informação sobre o uso e padrões de resistência relacionados com o uso de antibióticos em animais em Moçambique é muito limitada. Sabe-se, no entanto, que antibióticos são geralmente usados em animais para alimento, em particular na criação de frangos, para prevenção de doenças e tratamento para além da promoção de crescimento, mas nenhuma fármaco-vigilância é realizada de momento[4].

A produção de animais aquáticos é regulada pelo Decreto n.º 99/2021 de 31 de Dezembro, que estabelece as normas e procedimentos para o exercício da actividade de aquacultura. No âmbito de aplicação do presente Regulamento é extensivo às actividades de manuseio de reprodutores, da produção de ração e sementes aquícolas, o processamento e comercialização de produtos da aquacultura, construção de infraestruturas de produção e outros acessórios bem como todos os serviços de apoio à actividade da aquacultura e no âmbito de aplicação, não abrange animais de ornamentação, organismos geneticamente modificados e anfíbios.

Em relação ao uso de produtos químicos, rações e drogas veterinárias para a aquacultura, o regulamento remete a autorização pela autoridade competente para o controlo hígio-sanitário dos produtos da pesca (Inspeção de Pescado, IP através do Decreto n.º 80/2020 de 8 de Setembro). Na descarga de efluentes são aplicadas as normas relativas às águas residuais de acordo com o plano de gestão ambiental.

---

[4] Grupo de Trabalho da Parceria Global da Resistência a Antibióticos - GARP - Moçambique. 2015. Análise Situacional e Recomendações: Uso e Resistência aos Antibióticos. Washington, DC e Nova Deli Centro para estudo de Dinâmica de Doenças, Economia e Política (CDDEP).

A prevenção e controlo de doenças e espécimes infectados da aquacultura obedecem aos regimes jurídicos relativos à sanidade dos animais aquáticos e de plantas aquáticas. As doenças de comunicação obrigatória estão previstas em disposições específicas sobre Sanidade de Animais e Plantas Aquáticas.

3) LEGISLAÇÃO SOBRE RAÇÕES/FORRAGENS

A. ENQUADRAMENTO

O Regulamento da Produção e Comercialização de Forragens para a alimentação de Animais de 08 de Agosto de 1970, define forragem *toda a substância destinada à alimentação dos animais, qualquer que seja a sua origen ou composição,* o que sugere que o mesmo aplica-se quer para animais terrestres quer aquáticos. No entanto, o Regulamento apenas acaba detalhando forragens para alimentação do gado (artigo 2). No mesmo regulamento, o termo ração *é utilizado, apenas, para os alimentos compostos capazes de fornecerem os principios necessários à nutrição racional dos animais, satisfazendo as necessidades de conservação e/ou de produção das espécies a que se destinam (artigo 2, n. 2).*

Não existe outro instrumento jurídico específico que trate de rações, sendo apenas encontradas algumas disposições jurídicas que fazem referência às rações em alguns diplomas legais tais como o Regulamento de Gestão de Pesticidas e o regulamento de control higio-sanitário dos produtos da pesca e aquacultura. O Regulamento de Gestão de Pesticidas, na verdade, não regula as rações, apenas proibe que pesticidas sejam transportadas e armazenadas juntamente às rações. Também estabelece que que os locais onde são produzidos, consumidos ou armazenados rações devem estar a 100 metros dos armazéns de pesticidas (artigo 42).

Através da Portaria n. 145/75, de 25 de Dezembro, foram fixadas as características que deverão possuir os alimentos simples e as rações preparadas para os animais domésticos que poderão ser comercializados para a respectiva alimentação. Neste contexto, foram fixadas as características de rações para: Bovinos de carne; Bovinos leiteiros; Cabalinos; Arietinos; Suínos; Cães e Gatos; Coelhos; e Aves, nos seus parámetros máximos de proteína, fibra, cálcio (incluido mínimo) e fósforo. No entanto, a evolução tecnológica na produção actual de rações determinou o desajustamento e desactualização dos referidos parâmetros, havendo necessidade urgente da actualização deste instrumento jurídico.

Por seu turno, o Regulamento para o Controlo Higio Sanitário dos Produtos da Pesca, aprovado pelo Decreto n. 80/2020, de 08 de Setembro, aplicável às rações para animais aquáticos (art.1 e art. 2 al. a)), que para além de consagrar o princípio da rastreabilidade, atribui a autoridade competente a responsabilidade de realizar programas de pesquisa, análise e avaliação relacionados com os perigos e riscos em rações para animais aquáticos (art. 6, al.k)), prestar serviços de análises laboratoriais relativas à segurança de rações para animais aquáticos (art. 6, al.s)), publicar a lista de laboratórios oficiais e de referência para análise e controles oficiais e auto control das unidades produtivas de rações para os animais aquáticos, competindo ao

inspector do pescado, fiscalizar em toda cadeia produtiva, incluindo a documentação relativa às rações para animais aquáticos (art. 10, al.f)). Importa referir que o regulamento começa por definir ração *como sendo qualquer alimento para animais aquáticos de aquacultura*. Este conceito é distinto do constante do Regulamento da Produção e Comercialização de Forragens para a alimentação de Animais de 08 de Agosto de 1970. Não obstante, a autoridade competente não estar ainda capacitada em termos de recursos humanos, materiais e financeiros para executar os pertinentes comandos relativamente às rações, pode-se dizer que o regime das rações para animais aquáticos está suficientemente abordado no regulamento em análise, enquanto para animais terrestres a matéria encontra-se ainda regulamentada no Regulamento da Produção e Comercialização de Forragens para a alimentação de Animais de 08 de Agosto de 1970. As referências regulamentares sobre rações, para além de fragmentadas e dispersas, mostram-se também insuficientes para regular toda a cadeia de rações e desempenhar o seu papel na prevenção da RAM, mas o Regulamento da Produção e Comercialização de Forragens para a alimentação de Animais de 08 de Agosto de 1970, contém dispositivos sobre o acondicionamento, licenciamento, comercialização, importação/exportação e fiscalização da actividade forrageira.

I. Aditivos de alimentos

O quadro jurídico sobre os aditivos alimentares vem consagrado no Diploma Ministerial n. 100/87, de 23 de Setembro, que aprova o Regulamento sobre Aditivos Alimentares e complementado pelo Diploma Ministerial n. 80/87, de 01 de Julho, que aprova o Regulamento sobre Alimentos importados. Estes instrumentos assumem relevância no que diz respeito aos alimentos, na medida em que regulam a importação, produção, comercialização e utilização de aditivos alimentares (artigo 3). Com efeito, para além de aprovar uma lista dos aditivos alimentares autorizados e a sua dose máxima de emprego nos alimentos, o presente diploma consagra regras relativas a todos os aditivos e adjuvantes tecnológicos na preparação de produtos alimentares, obrigatoriedade de comercialização de aditivos alimentares armazenados em embalagem identificada com um rótulo. No entanto, estes diplomas não são aplicáveis às rações para animais. É no Regulamento sobre gestão de pesticidas onde encontramos uma norma aplicável aos alimentos quando o legislador estabelece a proibição do uso de substâncias reguladoras do crescimento de plantas.

Relativamente aos animais, sobretudo terrestres, o Regulamento da Produção e Comercialização de Forragens para a alimentação de Animais de 08 de Agosto de 1970, considera como fazendo parte de forragens, os suplementos alimentares (proteicos, minerais, vitamínicos e antibióticos) e entende que os aditivos alimentares são *qualquer substância que pode incluir os suplementos vitamínicos e antibióticos e que, adicionada a otros alimentos, se destina a melhorar a sua eficiência ou a atingir determinada finalidade profilática..*

O regime constante do Regulamento da Produção e Comercialização de Forragens para a alimentação de Animais de 08 de Agosto de 1970, é suficientemente amplo para cobrir toda a cadeira de valores das forragens. Há que revisitá-lo com vista a actualizá-lo às modernas

dinámicas da indústria forrageira bem como o regime sancionatório. Concomitatantemente, urge rever a Portaria n.141/75, de 25 de Dezembro, por consagrar um regime complementar àquele instrumento.

4) PESTICIDAS

A. ENQUADRAMENTO

A gestão de pesticidas é regulada pelo Decreto n. 6/2009 de 31 de Março de 2009, que aprova o Regulamento de Gestão de Pesticidas que garante que todos os processos que envolvam trabalhar com ou manuseio de pesticidas não sejam realizados em detrimento da saúde pública, animal e ambiental. O regulamento aplica-se ao registo, produção, doação, comercialização, importação, exportação, embalagem, armazenamento, transporte, manuseio, uso e eliminação de pesticidas e auxiliares, por pessoas singulares ou colectivas, para agricultura, pecuária, silvicultura, protecção à saúde pública, doméstica e outros propósitos.

O regulamento identifica as instituições envolvidas na gestão de pesticidas e, suas tarefas, nomeadamente (i) Repartição de Registo e Controlo de , (ii)Comissão Técnica de Avaliação de Registo de Pesticidas; e o (iii) Comitê Técnico Consultivo para Agroquímicos. Por outro lado, mandata que a lista dos pesticidas seja publicada 15 dias após a avaliação dos pedidos de registo de pesticida (artigo 17). Este quadro de gestão de pesticidas visava complementar o Regulamento de Pesticidas aprovado pelo Diploma Ministerial n. 153/2002, de 11 de Setembro, que também salientava no seu artigo 30, que as empresas ou entidades que empregam pessoas nas áreas de armazenamento, comercialização, transporte, aplicação e eliminação de pesticidas devem assegurar a formação contínua e actualizada do seu pessoal, incluindo regras de combate a incêndios, intoxicações, primeiros socorros, derrames e outros perigos. As empresas têm a responsabilidade de formar os seus funcionários junto das entidades governamentais no Ministério da Agricultura sendo este último responsável pela preparação e administração dos cursos.

B. ANÁLISE DA LEGISLAÇÃO RELEVANTE PARA A GESTÃO DE PESTICIDAS

A gestão de pesticidas é regulada pelo Decreto n. 6/2009 de 31 de Março de 2009 (Regulamento de Gestão de Pesticidas) que recomenda que todos os processos que envolvam o manuseio de pesticidas não sejam realizados em detrimento da saúde pública, animal e ambiental. O regulamento aplica-se ao registo, produção, doação, comercialização, importação, exportação, embalagem, armazenamento, transporte, manuseio, uso e eliminação de pesticidas e auxiliares, por pessoas singulares ou colectivas, para agricultura, pecuária, silvicultura, protecção à saúde pública, doméstica e outros propósitos. O Regulamento de Pesticidas, aprovado pelo Decreto n. 6/2009, estabelece a obrigatoriedade ao médico veterinário reportar qualquer suspeita da existência de doenças constantes dos seus anexos 1 e 2. Por outro lado, mandata que a lista dos pesticidas seja publicada 15 dias após a avaliação dos pedidos de registo de pesticida (artigo 17).

O Regulamento de Gestão de Pesticidas (RGP) inclui no seu artigo 1 uma extensa lista de termos-chave para a correcta interpretação da lei, tais como pesticida, resíduo de pesticidas, limite máximo de resíduos, toxicidade, entre outros. No entanto, compulsando o Código de Conduta

Internacional para a Gestão de Pesticidas, constata-se que o Regulamento de Gestão de Pesticidas é omisso na definição de muitos conceitos tais como os de *pesticidas altamente perigosos, gestão integrada de pesticidas, gestão de vector integrado, ciclo de vida, equipamento de protecção pessoal, peste, operador de controlo de peste e grupos vulneráveis*. Há então necessidade de ajustar a secção das definições do Regulamento.

Relativamente ao registo e autorização de pesticidas, o RGP estabelece (artigo 8, n. 1), que a produção[5], doação, comercialização[6], importação e utilização de qualquer substância com acção pesticida está sujeita a um registo prévio, junto do Comité de Avaliação Técnica do Registo de Pesticidas (CATERP), a favor de empresas ou instituições, devidamente estabelecidas no País e autorizadas pelos ministérios de tutela, e que assumam a inteira responsabilidade técnica e ambiental da utilização dos pesticidas por ela registados.

O artigo 17 obriga a que a lista dos pesticidas registados seja publicada quinze dias após a avaliação dos pedidos de registo de pesticida, e sempre que haja quaisquer alterações significativas dos dados técnico-científicos que estiveram na base do registo desse pesticida, fundamentalmente os relativos ao impacto do produto no âmbito toxicológico, ambiental e biológico, deve o registador ser informado. O registo de pesticida contêm: *"a) A marca comercial; b) A substância activa e o seu teor; c) O nome do titular de registo; d) A validade do registo; e) O número de registo; f) A categoria dos pesticidas; g) A respectiva classe toxicológica".* Esta lista deve ser enviada às Alfândegas, IIAM e às demais unidades orgânicas relevantes para disponibilizar a todos os interessados.

Para a venda de pesticidas agrários e os utilizados na saúde pública contra vectores de doenças, o RGP consagra a obrigatoriedade dos estabelecimento comerciais serem autorizados pelo Ministério da Indústria e Comércio, mediante parecer favorável da DNSAB (artigo 28), e somente os maiores de idade podem distribuir pesticidas, e desde que tenham nível de escolaridade básico maior (artigos 17 a 20 do Regulamento de Pesticidas, aprovado pelo Ministerial n. 153/2002, de 11 de Setembro). Importa referir que só podem ser colocados à venda pesticidas que contenham o rótulo, que aliás, é condição para a aprovação do registo de um pesticida. O rótulo deve identificar o *produto, o titular do registo, providências, advertências e precauções, bem como directrizes de uso e procedimentos para os primeiros socorros.* O RGP recomenda aos ministros que superintendem as áreas de agricultura, ambiente e saúde a aprovação das especificações e o formato dos rótulos dos pesticidas. Não há registo deste Diploma Ministerial conjunto ter sido aprovado, o que representa uma omissão.

---

[5] O artigo 38 n. 7, do RGP estabelece que os Ministérios que superintendem as áreas da agricultura, ambiente e saúde, ouvidos o Ministério do Trabalho e o Instituto Nacional de Normalização da Qualidade (INNOQ), definem as condições e as características dos locais de produção de pesticidas.

[6] O artigo 43 do RGP obriga a que o vendedor explique ao comprador as características e finalidades do pesticida, bem como as medidas de precaução e de segurança a serem observadas no seu manuseamento. O RGP protege os menores, indivíduos portadores de anomalias psíquica, cegueira, ou outra anomalia física evidente, ou lesões cutâneas visíveis que possam dificultar ou tornar perigosa a manipulação de pesticidas, proibindo-se que os mesmos comprem e vendam pesticidas (artigo 43 n.5, al.b).

De qualquer modo, os comerciantes de pesticidas devem fornecer, trimestralmente, ao Registador as quantidades dos pesticidas adquiridos, vendidos e os respectivos *stocks*, devendo, caso tenha sucursais em diferentes cidades ou locais, fornecer estes dados de forma separada por estabelecimento (artigo 44 n. 3, RGP).

Para a materialização deste propósito o RGP obriga a que o comerciante faça constar na factura, recibo e/ou venda a dinheiro de cada venda o nome do comprador, a marca do produto número de lote, respectivas quantidades e datas de vendas, mas tais informações não são relatadas, como por exemplo, a um centro que recolhe e estude esse tipo de informação, sobretudo quando haja reacções adversas. Relativamente aos utilizadores dos pesticidas, o RGP consagra, no artigo 37, o dever de os aplicar de modo a não prejudicar as culturas, animais, consumidores dos produtos vegetais e animais, organismos não visados, bem como minimizar o risco para o aplicador, para a população em geral e para o meio ambiente.

A este propósito, impõe-se também que o lote de pesticidas cujo prazo de validade tenha expirado seja imediatamente retirado do mercado, passando a ser considerado um pesticida obsoleto (artigo 43.7), que igualmente, não devem ser importados, doados e comercializados (artigo 45). No entanto, é possível a revalidação da data do lote de pesticida (artigo 46). Caso não seja possível, deve o pesticida ser eliminado (artigo 47, n.1) mediante autorização da Direcção Nacional de Gestão Ambiental (DNGA), ouvido o Registador.

Finalmente, o regulamento também se refere à eliminação de embalagens vazias, que "devem ser descontaminadas através da tripla lavagem, tomando-se os devidos cuidados para que não se contamine rios, lagos ou quaisquer cursos de águas superficiais ou subterrâneos". Uma vez descontaminados, podem ser reutilizados para novos pesticidas, mas não para qualquer tipo de alimento humano ou animal, ou água. Se tiverem de ser destruídos, devem ser tomadas todas as precauções necessárias para evitar a contaminação do meio ambiente (artigo 49).

Não obstante a análise e o controlo do LMR em pesticidas ser efectuado pelo Laboratório Nacional de Higiene de Alimentos e Águas do MISAU (artigo 56 n. 5) com base nos dados submetidos, e sempre que possível em obediência aos valores da Comissão Codex Alimentarius da OMS/FAO, e publicados no Boletim da República, mas não há evidência de publicação destes valores.

Independentemente do que se faz menção, importa referir que este regulamento, para além de não definir os LMR em pesticidas, também não inclui referências à RAM, o que seria de todo útil, porquanto,poderia servir de base para regulamentar a autorização e utilização de antimicrobianos (antibióticos ou fungicidas) como pesticidas. De qualquer modo, e em linha a metodologia da FAO é na legislação sobre os alimentso onde encontramos algunas referencias aos LMR em productos alimentares, mas sempre com referência aos parâmetros definidos pelo Codex Alimentarius da FAO.

5) ALIMENTOS SEGUROS

A. ENQUADRAMENTO

O uso intensivo e desregrado de medicamentos, drogas e produtos veterinários em produtos animais e vegetais pode conduzir a que estes passem na cadeia de valores e sejam consumidos pelos humanos e esses medicamentos passem para os humanos, constituindo assim os alimentos num veículo para o aumento da AMR dependendo dos cuidados na sua cadeia produtiva.

No ordenamento jurídico moçambicano não existe uma legislação específica sobre a segurança dos alimentos ou alimentos seguros. Na ausência de uma legislação específica, as questões sobre os alimentos seguros podem ser encontradas ainda que de forma superficial e fragmentada, na lei do direito do consumidor e na lei de saúde pública.

Não obstante, do ponto de vista institucional, o país possui um conjunto de instituições tal como o INNOQ, habilitado para (a) promover o desenvolvimento de um sistema nacional da qualidade de forma a integrar todas as componentes relevantes para a melhoria da qualidade de produtos e (b) elaborar, homologar e divulgar Normas Moçambicanas (NM). Por outro lado, o quadro fiscalizador é realizado nos termos do artigo 5 do Decreto n. 43/2017, de 11 de Agosto, pela Inspecção Nacional das Actividades Económicas (INAE) responsável pela promoção de acções de natureza preventiva assegurando a qualidade, genuinidade, composição, aditivos alimentares, outras substâncias e de rotulagem dos géneros alimentícios para consumo humano e dos alimentos para animais.

B. ANÁLISE DA LEGISLAÇÃO SOBRE ALIMENTOS SEGUROS

I. Sistema de Segurança Alimentar

Através do Decreto n. 19/2021, de 09 de Abril, foi aprovado o Regulamento da Lei que cria o Sistema Nacional de Qualidade, que consagra diversos subsistemas de qualidade, em particular o de normalização, medidas sanitárias e fitossanitárias e avaliação da qualidade Ambiental. De particular importância é o subsistema sanitária e fitossanitária que têm como objectivo a protecção da vida humana, animal e vegetal. Este subsistema de medidas sanitárias e fitossanitárias é implementado pelos Ministérios que superintendem a área da agricultura, saúde, pescas, ciência e tecnologia.

Este subsistema visa garantir que o país adopte medidas sanitárias e fitossanitárias necessárias para assegurar o nível adequado de protecção sanitária ou fitossanitária por forma a que essas medidas não sejam incompatíveis com o preconizado no Acordo sobre Medidas Sanitárias e Fitossanitárias da OMC e da Organização Internacional de Epizootias-OIE. No entanto, a adopção de medidas específicas de compatibilização internacional ainda não se efectivou no ordenamento jurídico pátrio.

O sistema de segurança alimentar é também assegurado pela Lei n. 22/2009, de 28 de Setembro de 2009, que aprova a Lei da Defesa do Consumidor, e regulamentada pelo Decreto n. 27/2016, de 18 de Julho, que protege o consumidor contra alimentos suspeitos de serem impróprios para

consumo humano. O artigo 15, n. 4 al.b) define como sendo impróprios ao uso e ao consumo os bens deteriorados, alterados, adulterados, avariados, falsificados, corrompidos, fraudados, nocivos à vida ou à saúde, perigosos ou ainda, aqueles em desacordo com as normas regulamentares de fabricação, distribuição ou apresentação. Incluímos nesta categoria os produtos animais ou vegetais que apresentam níveis de resíduos acima dos tecnicamente recomendados, o que pressupõem desde logo a existência de uma lei que determine os tais limites máximos de resíduos admissíveis.

Ainda relativamente aos alimentos seguros, é imprescindível referenciar os medicamentos e drogas veterinárias para os animais aquáticos, previstos no Decreto n. 80/2020, de 08 de Setembro que aprova o Regulamento para o Controlo Higio Sanitário dos Produtos da Pesca e aquacultura, que estabelece dos artigos 35 a 39, medidas de gestão de crises de natureza sanitária, que devem ser adoptadas no domínio da segurança dos produtos da pesca e de rações para animais aquáticos, e os planos de emergência, de contingência e de controlo de resíduos de droga veterinários para produtos de aquacultura. Embora estes planos sejam adoptados em coordenação com a Autoridade Veterinária (artigo 40 n. 1), âmbito da biossegurança, a Autoridade Competente estabelece as regras específicas e os mecanismos voltados para prevenção, monitoria e controlo para erradicar ou minimizar os riscos resultantes da exposição e manipulação de organismos aquáticos vivos susceptíveis de causar efeitos adversos ao homem e ao meio ambiente (artigo 40 n. 3).

II. Limites Máximos de Resíduos de drogas veterinárias em alimentos

O Regulamento sobre o Controlo Higio Sanitário dos Produtos da Pesca, aprovado pelo Decreto n. 80/2020, faz algumas referências ao cumprimento dos requisitos sobre resíduos, contaminantes e substâncias proibidas como uma das obrigações a aplicar pelos operadores e a verificar aquando da execução dos controlos oficiais (artigo 29). Para efeito, a Autoridade competente elabora e implementa anualmente o plano de controlo de resíduos de medicamentos e drogas veterinários para a avaliação dos respectivos níveis nos produtos de aquacultura (artigo 38) e, similarmente, para os produtos da pesca, a Autoridade Competente elabora e implementa o plano nacional e control de contaminantes ambientais (artigo 39). O plano visa verificar a conformidade dos resíduos de medicamentos veterinários com os limites máximos de residuos fixados, controlar a quantidade dos residuos de pesticidas com os níveis fixados, e determinar a concentração dos contaminantes ambientais. O plano define ainda quais são os elementos químicos que devem ser monitorados nos productos da pesca e, embora estabeleça que os limites máximos de residuos são os definidos pelo Codex Alimentarius e dos mercados do destino, o plano consagra os LMR das substâncias, compostos nos productos da pesca bem como de metais pesados em espécies de pescado. Paradoxalmente, o plano nacional de controlo de residuos e contaminantes e os LMR nele constantes, não é um instrumento público, sendo apenas do conhecimento da autoridade competente que faz uso dele nos seus controlos oficiais. Não obstante, é carácter obrigatório a todas as unidades de produção de productos da pesca e de aquacultura.

O Regulamento de Pesticidas aprovado pelo Decreto n. 6/2006, de 31 de Março, estabelece no seu artigo 52 a obrigatoriedade dos agricultores respeitarem os intervalos de segurança relativos ao período entre a última aplicação de um pesticida e a colheita do produto, e remete ao Comité Nacional do Codex Alimentarius a fixação dos valores do LMR em productos alimentares. Já o RSA proíbe a importação, processamento, comercialização de carnes e vísceras de animais que possuem resíduos de hormonas ou antibióticos ou qualquer outra substância que possa constituir perigo para a saúde pública. Tal proibição já era consagrada pelo Diploma Ministerial n. 80/87, que no seu artigo 16 refere-se que os *níveis de contaminantes inorgânicos, micotoxinas e microbiológicos deverão estar em conformidade com os limites máximos permitidos pela legislação nacional ou que vierem a ser fixados pelo Ponto de Contacto, respeitando os valores constantes dos padrões de identidade e qualidade da Comissão do Codex Alimentarius FAO/OMS, ou de outras instituições de reconhecida capacidade, a critério do Ponto de Contacto.*

Apesar destes regulamentos fazerem referências à resíduos, não encontramos no quadro jurídico normas de fixação dos respectivos limites máximos (LMR) em alimentos, e indicadores relevantes para a RAM no conjunto dos padrões de alimentos, padrões de qualidade e emissão de efluentes, respectivamente, remetendo-se para outras normas ou directivas a respectiva fixação.

A referida fixação é fundamental para a eficácia não apenas do quadro jurídico acima mencionado, como também da Lei n. 3/2022, de 10 de Fevereiro, que aprova a Lei da Saúde Pública, que classifica os resíduos em perigosos e sólidos. Esta lei consagra quanto à protecção de alimentos (artigo 29), que as autoridades que superintendem a área de saúde e outras autoridades relacionadas devem zelar, proteger e evitar a contaminação, falsificação e outras acções que alterem a integridade dos alimentos e atentem contra a Saúde Pública, competindo à Inspecção-Geral de Saúde, em coordenação com outras autoridades competentes relacionadas, proceder à vigilância e controlo de qualidade de alimentos para o consumo humano. À semelhança da demais legislação nacional, esta lei é omissa relativamente à fixação de LMR de medicamentos veterinários e pesticidas permissíveis em alimentos.

O Regulamento de gestão de pesticidas remete a fixação dos LMR de um pesticida em productos alimentares ao Comité Nacional do *codex alimentarius,* o Regulamento sobre o control higio-sanitário remete ao Plano de controlo de resíduos de drogas veterinários para produtos de aquacultura. É, pois, necessário a domesticação e consagração em um instrumento legal dos LMR em productos alimentares de modo a tornar eficazes as disposições normativas dos instrumentos jurídicos acima mencionados e conferir maior segurança jurídica aos particulares e certeza na actuação das autoridades inspectivas.

6) AMBIENTE, SOLO E RESÍDUOS

A. ENQUADRAMENTO

A Constituição da República de Moçambique, confere a todos os cidadãos o direito de viver num ambiente equilibrado. A materialização deste direito passa necessariamente por uma gestão correcta do ambiente e dos seus componentes, pela criação de condições propícias à saúde e

bem estar das pessoas e a preservação dos recursos naturais que a sustentam, aspectos abordados pela lei-quadro do ambiente, a Lei n. 20/97, de 01 de Outubro.

O ambiente constitui um veículo de disseminação da RAM, factor que tem vindo a ser reconhecido no plano global. É sabido que os antibióticos podem chegar ao meio ambiente através de três canais principais: a) resíduos animais, b) resíduos humanos e c) resíduos industriais, podendo contaminar o solo, culturas e fontes de água e estimular o desenvolvimento de resistência a medicamentos entre os patógenos com os quais interagem. Por outro lado, é difícil prever a rapidez com que se degradam, pois são muito diversos quimicamente.[7]

B. ANÁLISE DA LEGISLAÇÃO RELEVANTE PARA O AMBIENTE, SOLO E RESÍDUOS

O governo de Moçambique, através do seu quadro regulador adoptou através do Decreto n. 18/2004, de 2 de Junho, o Regulamento sobre Padrões de Qualidade e Emissão de Efluentes, visando o controlo e manutenção dos níveis admissíveis de concentração de poluentes nos componentes ambientais. Tendo em consideração que os componentes do ambiente, de acordo com a Lei do Ambiente, Lei n. 20/97, de 01 de Outubro, são entre outros o ar, a luz, a terra e a água, podemos inferir que as acções de monitorização e vigilância incluem todos os componentes.

O Decreto n. 18/2004, de 02 de Junho, no seu artigo 12, define os parâmetros essenciais que devem caracterizar a qualidade de água, em função da sua categoria, de modo a averiguar a conformidade das águas com os padrões de qualidade e para permitir a determinação de esquemas de tratamento adequados.

O Decreto no seu artigo 16 realça que o destino final das descargas de efluentes líquidos industriais no meio receptor, deverá ser feita através de emissão apropriada para o efeito, devendo no entanto, o efluente final descarregado, obedecer aos padrões de emissão ou descarga estabelecidos no no presente regulamento. A localização do ponto de emissão ou descarga dos efluentes deverá ser determinada no âmbito do licenciamento ambiental, de forma a que não haja alteração da qualidade das águas do meio receptor, impossibilitando a utilização das suas águas para outros fins.

Sem prejuízo da legislação específica, a descarga de efluentes domésticos no meio receptor deverá obedecer aos padrões fixados no regulamento, e se o meio receptor for oceano, há que garantir que os efluentes emitidos obedeçam aos padrões estabelecidos no regulamento supracitado.

Em relação aos resíduos biomédicos, a lei moçambicana os define como os resíduos resultantes de actividades de medicina humana e veterinária de diagnóstico, tratamento e investigação. O Decreto n.º 8/2003, de 18 de Fevereiro, aprovou o Regulamento sobre Gestão de Resíduos Biomédicos, que estabelece regras para a gestão de resíduos bio-médicos, com vista a salvaguardar a saúde e segurança dos trabalhadores das Unidades sanitárias, dos trabalhadores

[7] O'Neills, J. The Review on Antimicrobial Resistance: tackling drug-resistant infections globally. 2016. 84p.

auxIliares e do público em geral e minimizar os impactos de tais resíduos sobre o ambiente. Este decreto indica de que forma os resíduos biomédicos devem ser geridos, separados, identificados, armazenados, depositados, removidos, transportados e eliminados/tratados.

No que diz respeito ao descarte de resíduos de farmas, mercados de animais, matadouros, instalações de quarentena e outros estabelecimentos onde os animais podem ser produzidos ou mantidos estes são legislados pelo Decreto n. 18/2004, de 2 de Junho, o Regulamento sobre Padrões de Qualidade e Emissão de Efluentes. Um outro instrumento de controle ambiental é o processo de Avaliação do Impacto Ambiental (AIA), aprovado pelo Regulamento Sobre o Processo de AIA, o Decreto n. 54/2015, de 31 de Dezembro, que prevê a ocorrência de auditoria ambiental (artigo 25 n. 3). No seu artigo 4, o Decreto categoriza as actividades em diferentes categorias que estão sujeitas ou não a realização de EIA antes da sua implementação.

Em relação à qualidade do solo, monitoramento de resíduos no solo ou prevenção de contaminação do solo e da água que abranja a contaminação com antimicrobianos, o artigo 18 Decreto n. 18/2004, de 2 de Junho, define os parâmetros para a manutenção da qualidade dos solos e o artigo 19 afirma que é proibido o depósito no solo, foram dos limites legalmente estabelecidos de substâncias nocivas, que possam determinar ou contribuir para a sua degradação. De qualquer modo, os padrões de efluentes e emissões estabelecidos não integram os indicadores relevantes para a RAM, nem os mecanismos de transferência de poluentes.

O quadro de gestão ambiental é implementado a nível central e no âmbito da governação descentralizada, competindo aos Governo Provinciais e Secretarias de Estado Provinciais, através das respectivas Direcções da Terra e Ambiente e Serviços, respectivamente levar a cabo programas inerentes a assegurar um ambiente são. Ainda no quadro da monitorização e vigilância ambiental, o Governo, através de Regulamento sobre a Inspecção Ambiental, aprovou pelo Decreto n. 11/2006, de 15 de Junho, um importante instrumento de execução das competências da Agência Nacional para o Controlo da Qualidade Ambiental (AQUA), criada pelo Decreto n.º 80/2010, de 31 de Dezembro, e alterado pelo Decreto n.º 2/2016, de 10 de Fevereiro.

É então da competência da AQUA, realizar actividades no domínio da Investigação para o controlo da Qualidade Ambiental, tais como pesquisa e monitoramento da qualidade ambiental para o desenvolvimento sustentável dos recursos naturais, terrestres e marinhos-costeiros; elaborar os indicadores ambientais para avaliação de riscos associados a substâncias poluidoras; e no domínio do controlo da qualidade ambiental, realizar auditorias ambientais públicas e, monitorar e controlar a privadas, propondo as medidas de prevenção e mitigação dos impactos ambientais. No domínio da fiscalização ambiental, pode levantar autos de notícia em caso de infracções (artigo 9 do Regulamento Sobre Inspecção Ambiental).

O quadro acima descrito é ainda reforçado pelo Diploma Ministerial n. 74/2016, de 28 de Outubro, que aprova os procedimentos para Eliminação de Produtos Farmacêuticos. O referido diploma assume sua relevância, porquanto, aplica-se as unidades sanitárias públicas e privadas, aos fabricantes de produtos farmacêuticos, as empresas importadoras e distribuidoras de

medicamentos, farmácias, Postos de medicamentos, lojas de comércio geral, Organizações não-governamentais e pessoas singulares, e consagra os seguintes métodos de eliminação e formas de tratamento e productos farmecêuticos antes do aterro: aterro sanitário aberto, aterro sanitário de alta tecnologia, esgotos, queima em recipientes abertos, incineração a temperatura média, incineração a alta temperatura e decomposição química. O regime aquí consagrado, na ausência, de um regime específico, pode ser adaptado para os medicamentos, drogas e productos veterinários.

7) QUALIDADE DA ÁGUA

A. ENQUADRAMENTO

A importância dos recursos hídricos em todos os sectores da vida tem originado um aumento cada vez maior de necessidades da sua utilização. De entre os seus vários usos, destacam-se o consumo humano (abastecimento), aquacultura (cultivo dos organismos aquáticos), agricultura (a irrigação), uso industrial (produção de energia eléctrica, outros usos), navegação marítima (inclui exploração pesqueira e turismo). Consoante as necessidades e as quantidades que cada utente entender e se estiver contaminada com resíduos de antimicrobianos ou materiais/organismos resistentes, consequentemente irá contribuir para o desenvolvimento e disseminação da RAM.

B. ANÁLISE DA LEGISLAÇÃO RELEVANTE PARA A QUALIDADE DA ÁGUA

Em Moçambique, a água e legislada pela Lei n. 16/91 de 31 de Agosto, que estabelece os princípios de gestão de águas, a necessidade de inventariação de todos os recursos hídricos existentes no país, o regime geral da sua utilização, as prioridades a ter em conta, os direitos gerais dos utentes e as correspondentes obrigações, entre outros.

O direito de uso das águas do domínio público é reconhecido em regime de uso livre, em determinados casos por meio de autorizações de uso ou de concessões de aproveitamento, em casos especialmente regulados. Aos usos e aproveitamentos privativos resultantes de licença ou concessão terão acesso quaisquer pessoas, singulares ou colectivas, públicas ou privadas, nacionais ou estrangeiras devidamente autorizadas a actuar em território nacional, nos termos desta lei e desde que não ponham em causa o equilíbrio ecológico e o meio ambiente. O abastecimento de água à população, para consumo humano e para satisfação das necessidades sanitárias, têm prioridade sobre os demais usos privativos.

O artigo 29 da Lei n. 16/91 de 31 de Agosto, destaca que as águas concedidas para fins agrícolas ou industriais transmitem-se juntamente com o direito ao uso e aproveitamento da terra onde essas explorações se acham implantadas e nas mesmas condições, garantindo sempre a minimização do impacto ambiental e, em especial, zelar pela qualidade da água.

No que diz respeito a actividades de aquacultura, o Decreto n.º 99/2021 de 31 de Dezembro que regulamenta o exercício de actividade da aquacultura, no seu artigo 13 remete ao uso e aproveitamento da terra e das águas, que integram o domínio público para fins da actividade da aquacultura, estão sujeitos ao regime de licenciamento definido nas Leis da Terra e das Águas

respectivamente. A importação e uso de produtos químicos, rações e drogas veterinárias para a aquacultura em qualquer sistema de cultivo (aberto ou fechado) ficam sujeitos a autorização pela autoridade competente para o controlo hígio-sanitário dos produtos da pesca de acordo com artigo 41. Em relação à gestão de efluentes, o artigo 41 nr 3, refere que na descarga de efluentes são aplicadas as normas relativas às águas residuais de acordo com o plano de gestão ambiental.

A descarga de águas residuais é legislada pelo Regulamento sobre Padrões de Qualidade Ambiental e de Emissão de Poluentes (Decreto-Lei nº 18/2004, de 2 de Junho). Este decreto tem como principal objetivo, a proteção do meio ambiente das descargas das águas residuais urbanas e industriais, através do estabelecimento de critérios de recolha, tratamento e descarga das mesmas, porém é omisso em relação à reutilização de águas residuais para fins agrícolas.

A Lei 16/91 de 31 de Agosto no seu artigo 23 afirma que o aproveitamento privado da água depende o licenciamento, estando sujeitos a esse processo actividades de: a) prospecção, captação e o aproveitamento de águas subterrâneas incluídas nas zonas de protecção; b) instalação de depósitos, a implantação de culturas ou plantações e o abate de árvores nos leitos e margens das correntes naturais contínuas ou descontínuas e dos lagos, lagoas e pântanos; c) a extracção de materiais inertes, designadamente areia e cascalho, dos leitos e margens das correntes naturais contínuas ou descontínuas e dos lagos, lagoas e pântanos.

8) SANIDADE VEGETAL

A. ENQUADRAMENTO

Moçambique possui condições edafo-climáticas que propiciam a ocorrência de diferentes tipos de pragas/doenças durante o desenvolvimento das culturas. De acordo com o MADER, as perdas de produção devido ao ataque de pragas/doenças estimam-se em cerca de 40% quando medidas de controlo não forem implementadas atempadamente. A vigilância adequada de pragas pode ajudar o país a conter a ocorrência e disseminação de pragas de plantas e, assim, reduzir a necessidade de uso de antimicrobianos na produção agrícola, bem como conter o desenvolvimento de resistência a pesticidas de plantas.

B. ANÁLISE DA LEGISLAÇÃO RELEVANTE PARA A SANIDADE VEGETAL

Moçambique é signatário da Convenção Fitossanitária Internacional (*International Plant Protection Convention*) e as Normas Internacionais de Medidas Fitossanitárias (*International Standards for Phytosanitary Measures*) são legisladas pelo Decreto 5/2009 de 1 de Junho que aprova o Regulamento de Inspecção Fitossanitária e de Quarentena Vegetal.

A Autoridade Nacional Fitossanitária (ANF) no país é a Direção Nacional de Sanidade Agropecuária e Biossegurança (DNSAB), subordinada ao MADER, que tem entre outras competências: a) assegurar a protecção e defesa fitossanitária salvaguardando a saúde pública e o meio ambiente; b) certificar o processo de producao, importacao e exportacao de sementes e material vegetativo; c) fiscalizar e inspecionar as redes comerciais de sementes, fertilizantes e pesticidas, d) estabelecer mecanismos de vigilância, prevenção controlo e erradicação de doenças e agentes de doenças fitossanitárias, com impacto na economia e na saúde pública e e)

colaborar com os organismos regionais e internacionais no âmbito da vigilancia, prevencao e controlo zoo e fitossanitário.

A DNSAB cumpre também no seu mandato a emissão de Licenças de Importação de Plantas e certificados fitossanitários. Em relação aos produtos sujeitos a controlo fitossanitário, estes devem ser acompanhados do Certificado Fitossanitário do país de origem ou de procedência, de acordo com as normas de Convenção Fitossanitária Internacional. A importação de produtos sujeitos a controlo não especificados no Decreto n 5/2009 está sujeita à obtenção prévia de uma autorização de importação concedida pela ANF que é emitida após uma análise de risco de pragas.

Existe no Regulamento de Inspecção Fitossanitária e de Quarentena Vegetal uma Lista de Pragas de Quarentena de declaração obrigatória, constituindo dever de qualquer cidadão participar à ANF ou qualquer entidade administrativa local a ocorrência das mesmas.

O Regulamento de Inspecção Fitossanitária e de Quarentena Vegetal é omisso em relação aos planos de contingência, porém no artigo 25 afirma que a ANF pode impor o regime de quarentena em determinada recinto quando verifique: a) existirem razões e/ou evidências para suspeitar que os produtos sujeitos a controlo estejam infectadas/ infestadas por qualquer praga; b) a presença de produtos sujeitos ao controlo infectados ou infestados por pragas da lista de organismos de quarentena no país; c) a existência de produtos sujeitos ao controlo, que tenham permanecido ou transitado em áreas infectadas/ infestadas ou suspeitas; d) existir perigo de disseminação da praga para áreas livres.

O Regulamento faz referência em relação a mandato para designar laboratórios de referência, oficiais e autorizados para a testagem de sementes usando métodos recomendados pelo ISTA (*International Seed Testing Association*) porém é omisso em relação a obrigação destes em compartilharem dados de vigilância com outros laboratórios de referência, oficiais e autorizados ou entidades governamentais.

Embora não haja estipulação específica de que a ANF deva delegar ou coordenar com outras instituições para cumprir suas funções no regulamento, na prática o faz. por exemplo, no seu funcionamento, possui um Comité de Aconselhamento Técnico, que é o órgão de consulta do Ministério que superintende a agricultura em matéria de protecção de plantas, constituído por várias entidades públicas e privadas, entre elas representante do Ensino Superior, Ministério da Saúde, Ministério da Terra e Ambiente, Ministério da Indústria e Comércio, Ministério do Turismo, Ministério do Interior, da Autoridade Tributária de Moçambique, da Confederação das Associações Económicas de Moçambique (CTA) e do Centro de Produção de Inspecção Botânica.

9) COORDENAÇÃO INSTITUCIONAL

A. ENQUADRAMENTO

A Organização Mundial da Saúde (OMS), a Organização das Nações Unidas para a Alimentação e Agricultura (FAO) e a Organização Mundial da Sanidade Animal (OMSA, fundada como OIE)

reconhecem a RAM como uma ameaça à saúde global. O Plano de Acção Global sobre RAM recomenda que os países criem um organismo interministerial para coordenar e promover a colaboração na governação da RAM.

Os mecanismos de articulação e coordenação inter-institucional, a nível nacional, na promoção do uso de antimicrobianos e redução da RAM, encontram-se espelhados no Plano Nacional de Acção contra a Resistência Anti-Microbiana (PNA 2019-2023), que preconiza a existência de uma liderança firme na coordenação e implementação do plano.

Para o efeito, uma estrutura de governação ou gestão nacional foi estabelecida. A mesma é composta pelas seguintes instituições: MISAU, MADER, MITA, MIMAIP, Ministério da Economia e Finanças (MEF), Ministério das Obras Públicas, Habitação e Recursos Hídricos (MOPHRH) e Ministério da Educação e Desenvolvimento Humano (MINED), e apoiado por um grupo técnico.

Com o mecanismo de coordenação inter-institucional pretende-se garantir a manutenção de uma comunicação regular e eficaz, em matéria de uso de antimicrobianos e combate à resistência antimicrobiana, o que implica responsabilidades partilhadas entre instituições e entidades[8]. Apesar do PNA (2019-2023) estabelecer uma estrutura de coordenação inter-institucional, o seu funcionamento e operacionalização não foi estabelecido por um instrumento legal, que conferisse ao grupo interministerial um mandato específico, competências, periodicidade de reunião, etc, o que faz com que os esforços tendentes a reduzir a RAM continuem fragmentados carecendo, no geral, de financiamento adequado.

Apesar de haver progressos e resultados positivos nos esforços de redução da resistência antimicrobiana, com a adopção do plano respectivo, deve-se reconhecer que ainda persistem grandes constrangimentos e ameaças ao processo. Tais constrangimentos já tinham sido identificados em vários documentos técnicos em 2015[9] e 2019[10], e estes incluem:

- Insuficientes recursos humanos, a diversos níveis, para assegurar uma maior cobertura da vigilância epidemiológica e controle dos antimicrobianos que entram e circulam no país;
- Fraca coordenação e capacidade de comunicação a vários níveis, sobretudo provincial e local;
- Deficiências nas infraestruturas de gestão de dados o que prejudica a monitorização regular e a recolha de dados fiáveis para medir o nível da RAM;
- Escassez de laboratórios competentes para apoiar na identificação exacta de microrganismos resistentes;

[8] Para mais informações, por favor consulte o Plano Nacional de Acção Contra a Resistência Antimicrobiana 2019-2023, 1ª Edição Maputo, 2019, disponível em: https://cdn.who.int/media/docs/default-source/antimicrobial-resistance/amr-spc-npm/nap-library/nap_20_11_2018---mozambique---2019-2023.pdf?sfvrsn=1349207d_4&download=true

[9] Análise situacional e recomendações: Uso e Resistência aos Antibióticos em Moçambique, disponível em https://www.cddep.org/wp-content/uploads/2017/06/executive_summary_portugues_language_1.pdf , acesso a 01 de Março de 2022.

[10] Ver Plano Nacional de Acção Contra a Resistência Antimicrobiana 2019-2023, 1ª Edição Maputo, 2019.

B. ANÁLISE DA LEGISLAÇÃO

De um **ponto de vista jurídico**, a principal restrição é a **falta de um instrumento jurídico que consagre a estrutura inter-institucional para a gestão da RAM delineada no Plano Nacional de Acção**. O estabelecimento desta estrutura através de um instrumento jurídico permitiria uma clara atribuição de funções a cada uma das instituições designadas, bem como a definição de mecanismos para operacionalizar o seu funcionamento, e facilitaria a responsabilização dos seus membros.

Por outro lado, o quadro jurídico sobre a RAM em Moçambique, ora é omisso é varios domínios como havemos de demonstrar adiante, ora é remissivo para outros diplomas legais ou instrumentos internacionais orientadores da actividade, o que se esbarra muitas das vezes com a inércia do legislador em aprovar os referidos diplomas legais gerando-se assim um vazio, ou fica ultrapassado com as constantes alterações dos instrumentos internacionais que nem chegam a ser domesticados.

10) CONCLUSÕES E RECOMENDAÇÕES

Da análise efectuada ao quadro jurídico-político da RAM constatou-se que o País não possui uma política específica para o uso de antimicrobianos e RAM, não obstante em 2019 ter sido aprovado o Plano Nacional de Acção Contra a Resistência Antimicrobiana (2019-2023), que contém três pilares, seis intervenções estratégicas, e com acções prioritárias para a saúde pública, sanidade animal e vegetal.

A implementação de do Plano acima mencionado apresenta desafios não só institucionais que se prendem com a falta de um instrumento regulador da desejada coordenação inter-institucional, com impactos na monitorização das acções previstas no plano cuja validade vai até 2023.

Da avaliação do quadro legal visando prevenir a RAM chegou-se às seguintes conclusões, que tomam em consideração as diversas áreas objecto de análise. Assim:

No domínio dos Medicamentos e Drogas e Produtos Veterinários

- Moçambique não possui uma legislação específica sobre medicamentos, drogas e outros produtos veterinários, o que representa uma enorme lacuna nos esforços de redução da RAM. Constatou-se também existir uma proposta de regulamento de medicamentos, drogas e produtos veterinários datada de 2019, cuja versão final ainda não foi levada à aprovação, pelo facto de ter sido considerado contra-producente aprovar o Regulamento antes da Lei de Sanidade Animal.

- A matéria sobre os medicamentos, drogas e produtos veterinários encontra-se regulamentada de forma fragmentária, dispersa e superficial nos Regulamentos de Gestão de Pesticidas, de Sanidade Animal e de Controlo Higio Sanitário dos Produtos da Pesca.
- Ainda no que diz respeito aos LMR constatou-se haver algumas referências quer no RGP, no regulamento de controlo higio-sanitário dos produtos da pesca e aquacultura, no regulamento de sanidade animal e na lei de saúde pública. No entanto, nenhum destes instrumentos fixa os LMR, e indicadores relevantes para a RAM no conjunto dos padrões de alimentos, padrões de qualidade e emissão de efluentes.

- Da análise aos diversos instrumentos jurídicos constatou-se que a eficácia de muitos dispositivos normativos dependem de normas complementares, que ainda não foram aprovadas tornando-as ineficazes. Tal é o caso, por exemplo, já citado do LMR e das especificações e o formato dos rótulos cuja aprovação depende dos Ministros responsáveis pela agricultura, ambiente e saúde. Há assim, necessidade de fazer-se o levantamento das várias disposições normativas remissivas e complementá-las. A título de exemplo a lei dos crimes contra a saúde pública preconiza vários subsistemas de qualidade visando garantir que o país adopte medidas sanitárias e fitossanitárias necessárias para assegurar o nível adequado de protecção sanitária ou fitossanitária, por

forma a que essas medidas não sejam compatíveis com o preconizado no Acordo sobre Medidas Sanitárias e Fitossanitárias da OMC e da Organização Internacional de Epizootias-OIE. No entanto, a adopção de medidas específicas de compatibilização internacional ainda não foram adoptadas no ordenamento jurídico pátrio.

- O Regulamento de Sanidade Animal não contém disposições específicas sobre os requisitos para a autorização de medicamentos veterinários. O mesmo diploma legal, é ainda omisso quanto às *actividades no ciclo de vida dos medicamentos, drogas e produtos veterinários, à lista, classificação, monitorização, reembalagens, língua utilizada em embalagens, obrigatoriedade de colocação, no rótulo, de informação como números de série ou marcas de autenticação, procedimentos de produção e requisitos para garantir padrões de qualidade, obrigatoriedade das farmácias, outros estabelecimentos ou profissionais que vendem medicamentos, drogas e produtos veterinários, de manterem e relatarem registos de venda, regras de produção de medicamentos, drogas e produtos veterinários tendentes a minimizar a contaminação ambiental com antimicrobianos resultantes do processo de produção, Sistema de autorização para fabricantes de medicamentos veterinários, regras sobre descarte de medicamentos veterinários não utilizados ou obsoletos, sobre armazenamento de medicamentos, Drogas e produtos veterinários, sobre registo de receitas médicas veterinárias efectuadas.*
- A robustez do quadro jurídico tendente a prevenir a resistência antimicrobiana passa necessariamente por reforçar as legislações sectoriais. Por outro lado, não é recomendada a adopção de legislação específica sobre RAM que aborde todos estes elementos, uma vez que tal poderia resultar numa fragmentação do quadro jurídico.

No domínio dos pesticidas

- O RGP recomenda aos ministros que superintendem as áreas de agricultura, ambiente e saúde a aprovação das especificações e o formato dos rótulos dos pesticidas, o que eventualmente estaria de acordo com o Sistema Globalmente Harmonizado de Classificação e Rotulagem de Produtos Químicos. Não há registo deste Diploma Ministerial conjunto ter sido aprovado, o que representa uma omissão a ser colmatada.

- Ainda relativamente aos pesticidas, vale referir que o respectivo procedimento de registo impõe às empresas a responsabilidade técnica e ambiental da utilização dos pesticidas, e o resgisto do pesticida não é aprovado se o pesticida puder causar impacto negativo inaceitável ao ambiente, saúde humana e/ou animal ou mesmo o nivel dos residuos nos alimentos estiverem acima dos LMR ou as suas características físico-químicas, toxilógias e ecotoxicológicas não estiverem dentro dos padres internacionalmente aceites.

No dominio das rações/forragens

- O Regulamento da Produção e Comercialização de Forragens para a alimentação de Animais de 08 de Agosto de 1970, embora abarque toda a cadeia de valor das forragens, ele restringe-se aos alimentos para o gado, embora possa ser usado com as necessárias

adaptações para outros animais. Torna-se assim necessário revisitar este instrumento e actualizá-lo para responder a demanda neste dominio.

- A Portaria n. 141/75, de 25 de Dezembro, que , que fixa as características dos alimentos simples e as rações preparadas para os animais domésticos que poderão ser comercializados para a respectiva alimentação, deve ser sofrer uma revisão nos parâmetros máximos e mínimos dos componentes das rações, em particular proteína, fibra, cálcio e fósforo.

No domínio dos alimentos seguros:

- Embora Moçambique não possua uma legislação específica sobre os alimentos seguros é no Regulamento sobre o Controlo Higio Sanitário dos Produtos da Pesca, aprovado pelo Decreto n. 80/2020, onde encontramos algumas referências ao cumprimento dos requisitos sobre resíduos, contaminantes e substâncias proibidas como uma das obrigações a aplicar pelos operadores e a verificar aquando da execução dos controlos oficiais (artigo 29). A implementação dos planos de controlo de resíduos de medicamentos e drogas veterinários para a avaliação dos respectivos níveis nos produtos de aquacultura (artigo 38) constitui a pedra angular, mas estes instrumentos não se encontram no domínio público.

- Apesar dos instrumentos jurídicos fazerem referências à resíduos de medicamentos veterinarios e pesticidas nos alimentos, não encontramos no quadro jurídico normas de fixação dos respectivos limites máximos (LMR), e indicadores relevantes para a RAM no conjunto dos padrões de alimentos, padrões de qualidade e emissão de efluentes, respectivamente., limitando-se o regulamento supra mencionado e o regulamento de pesticidas a remeter, para o Plano Nacional de Controlo de Resíduos, para o Codex Alimentarius da FAO e regras do mercado de destino.

No domínio institucional

- Os mecanismos de prevenção e combate a RAM continuam sectorizados e fragmentados, e o funcionamento e operacionalização do sistema de coordenação inter-institucional instituido pelo PNA (2019-2023) carece de regulamentação própria em instrumento legal com termos de referência claros, e mandatos especificados, competências e periodicidade de reuniões definidas.
- É necessário assegurar a partilha de dados e informação em tempo real através da concepção de uma base de dados sobre registo de importação e acções adversas de antimicrobianos garantindo-se assim uma correcta monitoria e acompanhamento

Referências:

- Análise situacional e recomendações: Uso e Resistência aos Antibióticos em Moçambique, disponível em https://www.cddep.org/wp-content/uploads/2017/06/executive_summary_portugues_language_1.pdf , acesso a 01 de Março de 2022.
- Chadd, Camila: Resistência Antimicrobiana e segurança de alimentos. Disponível em https://foodsafetybrazil.org/resistencia-antimicrobiana-e-seguranca-de-alimentos/ acesso a 14 de Março de 2022.
- Ministério da Saúde, Plano Nacional de Acção Contra a Resistência Antimicrobiana 2019-2023, 1ª Edição Maputo, 2019
- O'Neill, J., and The Review on Antimicrobial Resistance (2015). Antimicrobials in Agriculture and the Environment: Reducing Unnecessary Use and Waste. Available at: http://amrreview.org/sites/default/files/Antimicrobials%20in%20agriculture%20and%20the%20environment%20-%20Reducing%20unnecessary%20use%20and%20waste.pdf

LEGISLAÇÃO RELEVANTE

LEIS

- Lei n. 1/2018, de 12 de Junho que aprova a Lei de Revisão da Constituição da República de Moçambique
- Lei n. 12/2017, de 08 de Setembro, que aprova a Lei de Medicamentos, Vacinas e Outros Produtos Biológicos para o uso Humano
- Lei n. 20/97, de 01 de Outubro, que aprova a Lei do Ambiente
- Lei n. 22/2009, de 28 de Setembro, que aprova a Lei de defesa do consumidor
- Lei 3/2022, 10 de Fevereiro, Lei dos Crimes contra a Saúde Pública
- Lei n. 34/2014, de 31 de Dezembro, que aprova a Lei do Direito à Informação

DECRETOS e DECRETOS PRESIDENCIAIS

- Decreto n. 05/2009, de 01 de Junho, que aprova o Regulamento de Inspecção Fitossanitária e de Quarentena Vegetal
- Decreto n. 26/2009, de 17 de Agosto, Regulamento de Sanidade Animal
- Decreto n. 6/2009, 31 de Março, que aprova o regulamento sobre a gestão de pesticidas
- Decreto n. 29/2019, de 18 de Abril, que aprova as Boas Práticas para o Fabrico de Medicamento para Uso Humano
- Decreto n.º 27/2016, de 18 de Julho, que aprova o Regulamento da Lei de Defesa do Consumidor
- Decreto n. 43/2017, de 11 de Agosto, que cria o Inspecção Nacional de Actividades Económicas,
- Decreto n. 18/2004, de 02 de Junho, que aprova o Regulamento sobre Padrões de Qualidade e Emissão de Efluentes

- Decreto n. 19/2021, de 09 de Abril, que aprova o Regulamento da Lei que cria o Sistema Nacional de Qualidade.
- Decreto 15/2006, de 22 de Junho que aprova o Regulamento sobre os Requisitos Higiênicos-Sanitários de Produção, Transporte, Comercialização e Inspecção e Fiscalização de Géneros Alimentícios
- Decreto n. 80/2020, de 8 de Setembro, que aprova o Regulamento para o Controlo Higio-Sanitário dos Produtos da Pesca
- Decreto n. 81/2020, de 09 de Setembro, foi aprovado o Estatuto Orgânico do Instituto Nacional de Normalização da Qualidade
- Decreto n. 100/87, de 23 de Setembro, que aprova o Regulamento sobre aditivos alimentares
- Decreto n. 9/2016, de 18 de Abril, que aprova o Regulamento de Fortificação de Alimentos com Micronutrientes Industrialmente Processados
- Decreto n.º 34/2013 de 2 de Agosto, que aprova o Regulamento do Licenciamento da Actividade Comercial
- Decreto Presidencial n. 17/2015, de 25 de Março, cria o Ministério do Mar, Águas Interiores e Pescas
- O Regulamento da Produção e Comercialização de Forragens para a alimentação de Animais de 08 de Agosto de 1970.
- Portaria n. 141/75, de 25 de Dezembro, que , que fixa as características dos alimentos simples e as rações preparadas para os animais domésticos que poderão ser comercializados para a respectiva alimentação.

DIPLOMAS MINISTERIAIS

- Diploma Ministerial n. 51/84, de 03 de Outubro que aprova, o Regulamento sobre os Requisitos Higiénicos dos Estabelecimentos Alimentares
- Diploma Ministerial n. 80/87, de 01 de Julho,
- Diploma Ministerial n. 51/84, de 03 de Outubro
- Diploma Ministerial n.º 58/2016, de 09 de Setembro, foi aprovada a lista de medicamentos essenciais para o consumo humano

RESOLUÇÕES

- Resolução n. 3/2020, de 13 de Março- Estatuto do MADER
- Resolução n. 10/2020, de 05 de Maio Estatuto do MITA
