# Supplementary material for: Landscape Analysis of Existing Policies, Laws and Regulations on Antimicrobial Stewardship in 10 Low- and Middle-Income Countries
Source: Antibiotics (Basel). 2026 Aug 10;15(8):770. doi: 10.3390/antibiotics15080770 (PMC13510074; doi:10.3390/antibiotics15080770)
Supplement: Supplementary file 1 [file antibiotics-15-00770-s001.zip › S3 Country Reports/S3G-1_Nigeria (Human Health).pdf]

USAID MEDICINES, TECHNOLOGIES, AND PHARMACEUTICAL SERVICES (MTaPS) PROGRAM

Improved Access. Improved Services. Better Health Outcomes.

REPORT OF RAPID ASSESSMENT OF ANTIMICROBIAL STEWARDSHIP POLICIES AND ACTIVITIES IN HUMAN HEALTH IN NIGERIA

Nigeria

October 2021

This document is made possible by the generous support of the American people through the US Agency for International Development (USAID) contract no. 7200AA18C00074. The contents are the responsibility of Management Sciences for Health and do not necessarily reflect the views of USAID or the United States Government.

About the USAID MTaPS Program

The USAID Medicines, Technologies, and Pharmaceutical Services (MTaPS) Program enables low- and middle-income countries to strengthen their pharmaceutical systems, which is pivotal to higher-performing health systems. MTaPS focuses on improving access to essential medical products and related services and on the appropriate use of medicines to ensure better health outcomes for all populations. The program brings expertise honed over decades of seminal pharmaceutical systems experience across more than 40 countries. The MTaPS approach builds sustainable gains in countries by including all actors in health care—government, civil society, the private sector, and academia. The program is implemented by a consortium of global and local partners and led by Management Sciences for Health (MSH), a global health nonprofit.

The MTaPS Consortium

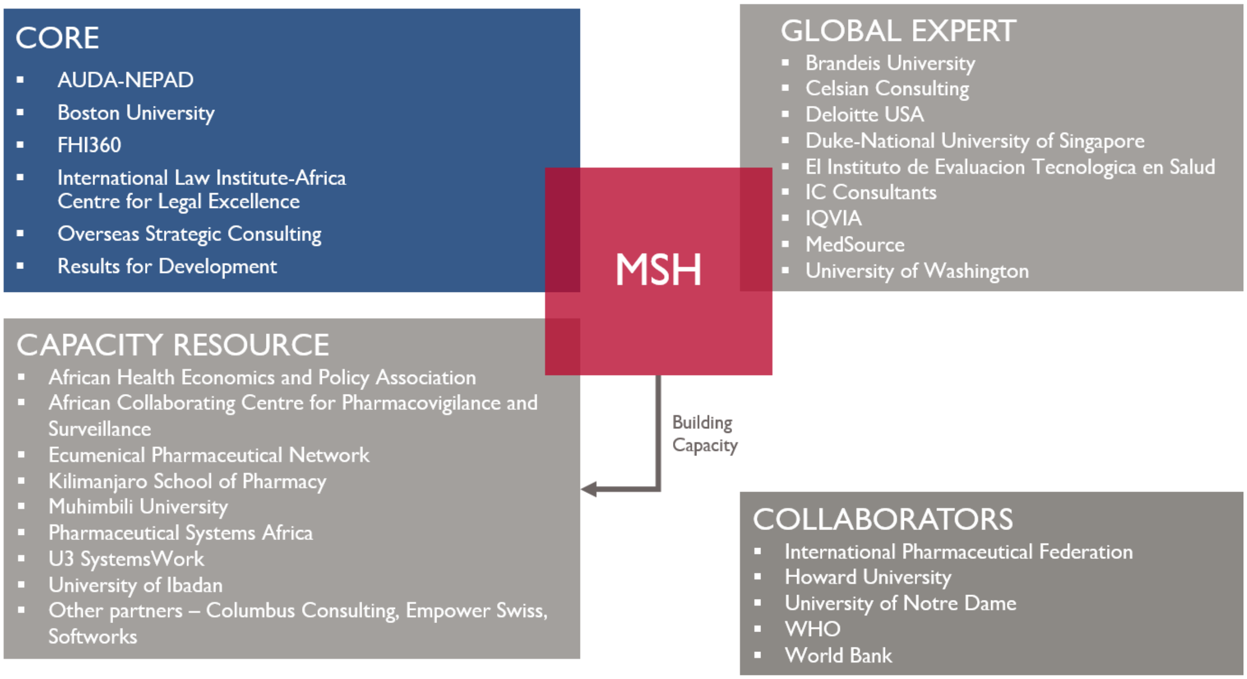

This report/document is submitted by:

Pharm. Moshood Oluku Lawal
AMS Consultant-Human Health
USAID/MTaPS Nigeria
Abuja
Email: molawal60@gmail.com

ACRONYMS AND ABBREVIATIONS

ADR	adverse drug reaction
AIDS	acquired immunodeficiency syndrome
AMR	antimicrobial resistance
AMS	antimicrobial stewardship
API	active pharmaceutical ingredient
COP	Committee of Practice
DTC	drug and therapeutics committee
DRF	Drug Revolving Fund Scheme
EML	Essential Medicines List
EQA	external quality assessment
FCT	Federal Capital Territory
FEC	Federal Executive Council
FMOH	Federal Ministry of Health
GLASS	Global AMR Surveillance System
GMPs	Good Manufacturing Practices
HF	health facility
HIV	human immunodeficiency virus
IHR	International Health Regulation
IPC	infection prevention and control
MAH	marketing authorization holder
MSLCN	Medical Laboratory Science Council of Nigeria
MTaPS	Medicines, Technologies, and Pharmaceutical Services program
MTC	medicines and therapeutics committee
NAFDAC	National Agency for Food and Drug Administration and Control
NCDC	National Centre for Disease Control
NCSC	NAFDAC Consumer Safety Club
NCSPs	NAFDAC Consumer Safety Publications
NDDG	National Drug Distribution Guidelines
NDSAC	National Drug Safety Advisory Committee
NHIS	National Health Insurance Scheme
NPC	National Pharmacovigilance Centre
NRL	National Reference Laboratory
NVRI	National Veterinary Research Institute
OTC	over-the-counter
PCN	Pharmacist Council of Nigeria
PMS	post-marketing surveillance
POM	prescription-only medicine
PV	pharmacovigilance
RMP	risk management plan
STG	standard treatment guideline
TB	tuberculosis
TWG	technical working group
USAID	US Agency for International Development
WASH	water, sanitation, and hygiene

DEFINITIONS

Antibiotic: An antibiotic is an agent or substance that is produced by or derived from a microorganism that kills or inhibits the growth of another living microorganism. Antibiotic substances that are synthetic, semi-synthetic, or derived from plants or animals are, strictly speaking, not antibiotics. However, they have been classified as such; therefore, an "antibiotic" refers to an antimicrobial agent with the ability to kill or inhibit bacterial growth.

Antimicrobial: An antimicrobial is an agent or substance derived from any source (microorganisms, plants, animals, synthetic, or semi-synthetic) that acts against any type of microorganism, such as bacteria (antibacterial), mycobacteria (anti-mycobacterial), fungi (antifungal), parasite (anti-parasitic), or viruses (antiviral). All antibiotics are antimicrobials, but not all antimicrobials are antibiotics.

Antimicrobial resistance: Antimicrobial resistance (AMR) occurs when bacteria, viruses, fungi, and parasites change over time and no longer respond to medicines, making infections harder to treat and increasing the risk of disease spread, severe illness, and death. Microorganisms such as bacteria, fungi, viruses, and parasites change when exposed to antimicrobial drugs such as antibiotics (antibacterials), antifungals, antivirals, antimalarials, and anthelmintics. As a result, the medicines become ineffective.

Antimicrobial stewardship: Antimicrobial stewardship (AMS) consists of "the optimal selection, dosage, and duration of antimicrobial treatment that results in the best clinical outcome for the treatment or prevention of infection, with minimal toxicity to the patient and minimal impact on subsequent resistance."

AMS program: An AMS program is an organizational or system-wide health care strategy to promote appropriate use of antimicrobials through the implementation of evidence-based interventions.

CONTENTS

Acronyms and Abbreviations i

Definitions ii

Project Summary iv

1. Introduction 1

2. Overview 2

3. Situational Analysis 5
   - 3.1. Methodology of the Analysis 5
   - 3.2. Health Care Delivery System in Nigeria 5
   - 3.3. Antimicrobials Use in Nigeria 7
   - 3.4. Knowledge and Practice of Antimicrobial Stewardship 7
   - 3.5. Current Status of AMR/AMS Implementation in Nigeria 9
   - 3.6. Governance and Institutional Framework 12
     - 3.6.1 Governance and Coordination 12
   - 3.7. Policy, Legislative, and Regulatory Framework 12
     - 3.7.1 National Drug Policy 12
     - 3.7.2 Essential Medicines List, MTCs, and Standard Treatment Guideline 14
     - 3.7.3 Pharmacovigilance and Post-Marketing Surveillance 14
     - 3.7.4 Legislations 16
   - 3.8. Manufacturers and Products Registration 16
   - 3.9. Medicines Classification and Control 17
   - 3.10. Medicines Supply Chain 17
   - 3.11. Regulation on Prescribing and Dispensing of Antibiotics 18
   - 3.12. Infection Prevention and Control 18

4. Analysis of Strengths, Weaknesses, Opportunities, and Threats 21
   - 4.1. Governance and Institutional Framework 21

5. Recommendations 24
   - 5.1. Immediate and Administrative Efforts 24

References 26

PROJECT SUMMARY

**Program Name:**		USAID Medicines, Technologies, and Pharmaceutical Services (MTaPS) Program
**Activity Start Date And End Date:**		September 20, 2018–September 19, 2023
**Name of Prime Implementing Partner:**		Management Sciences for Health
**Contract Number:**		7200AA18C00074
**MTaPS Partners**	**Core Partners**	Boston University, FHI 360, Overseas Strategic Consulting, Results for Development, International Law Institute-Africa Centre for Legal Excellence, NEPAD
	**Global Expert Partners**	Brandeis University, Deloitte USA, Duke-National University of Singapore, El Instituto de Evaluacion Technologica en Salud, IC Consultants, Imperial Health Sciences, MedSource, QuintilesIMS, University of Washington
	**Capacity Resource Partners**	African Health Economics and Policy Association, Ecumenical Pharmaceutical Network, U3 SystemsWork, University of Ibadan, University of Ghana's World Health Organizations (WHO) Pharmacovigilance Collaborating Center, Kilimanjaro School of Pharmacy, Muhimbili University, Pharmaceutical Systems Africa
	**Collaborators**	International Pharmaceutical Federation, Howard University, University of Notre Dame, WHO, World Bank

1. INTRODUCTION

The rise of antimicrobial resistance (AMR), if not stopped, threatens to plunge humanity back into an era of health uncertainty few people alive today can remember. AMR does not follow national borders; its consequences affect the lives of everyone on the planet and blight the prospects of future generations. AMR is not only a health problem, but also a development problem; yet with the right approach and intelligent investment, the AMR tide can be turned.

According to the World Bank 2017 report titled *Drug-Resistant Infections: A Threat to Our Economic Future,* 700,000 people die each year due to AMR. Without action, the death toll could rise even higher, reaching as many as 10 million deaths annually by 2050, and causing a 3.8 percent reduction in annual gross domestic product (GDP) across the globe. The world's poorest people—those living in low- and middle-income countries (LMICs)—are disproportionately vulnerable.

AMR also stands to have a significant impact on gender equity and countries' human capital because it affects people's lives through loss of time at school or at work, increased health care spending, and a reduction in health facilities' ability to quickly manage a patient's health condition. Given the important role of health, agriculture, and food systems, antimicrobials are critical to ensuring that countries can fully realize their human capital and that we can build more equitable societies.[1]

The emergence of the COVID-19 pandemic resulted in the subsequent rampant use of antibiotics in the treatment of a viral infection: an estimated 75% of COVID-19 patients were given antibiotics against co-infections and secondary infections, even though the occurrence of these is estimated at below 10% and 15%, respectively. AMR could thus become a sequela of Covid-19.

The need to exercise restraint in the use and handling of antimicrobials has long been recognised, yet this has become imperative in the face of emerging "superbugs" and the drying up of the pipeline of new antibacterials. A careful and responsible approach is important for reducing overuse and misuse of antibiotics by promoting rational use of antimicrobials through the concept of AMS. Optimizing the use of antimicrobial agents in human and animal health requires strengthening the sound management of antimicrobial use in health care settings by providing evidence for prescribing and dispensing according to standards.

Nigeria's National Action Plan for AMR (2017–2020) has a detailed operational component that includes well-defined strategic objectives for coordinated and reinforced management of AMR involving human and veterinary medicine, the environment, and the agro-food sector.

Noticeable progress has been made in the implementation of these strategies and associated activities, as reported by Country-Led Midterm Joint External Evaluation implementation progress since JEE 2019. It is further necessary to carry out an assessment to identify specific gaps in policies, legislation, regulations, and guidelines related to AMS in the human health sector. Thus, the need was identified for this rapid situational analysis of stewardship policies and activities, including the regulatory framework and supply chain management of antimicrobials; and the development of a national AMS plan for Nigeria that addresses the identified gaps and proffers required interventions in the human and animal health sectors.

2. OVERVIEW

Antimicrobial drugs are chemical substances of natural or synthetic origin that suppress the growth of, or destroy, microorganisms, including bacteria, fungi, helminths, protozoa, and viruses. They are medicines used to prevent and treat infections in humans, animals, and plants.

Effective antimicrobial drugs are prerequisites for both preventive and curative measures, protecting patients from potentially fatal diseases and ensuring that complex procedures, such as surgery and chemotherapy, can be provided at low risk. Yet systematic misuse and overuse of these drugs in human medicine and food production have put every nation at risk due to the aggravation of the natural phenomenon of AMR. AMR "threatens the very core of modern medicine and the sustainability of an effective, global public health response to the enduring threat from infectious diseases.[2]

AMR is the resistance of a microorganism to an antimicrobial drug that was originally effective for treatment of infections caused by it. Resistant microorganisms (including bacteria, fungi, viruses, and parasites) are able to withstand attack by antimicrobial drugs—such as antibacterial drugs (e.g., antibiotics), antifungals, antivirals, and antimalarials—so that standard treatments become ineffective and infections persist, increasing the risk of spread to others, cost of treatment, severity of illness, and the risk of death. Especially alarming is the rapid global spread of multi- and pan-resistant bacteria (also known as "superbugs") that cause infections that are not treatable with existing antimicrobial medicines such as antibiotics.

Drug-resistant infections were previously associated predominantly with hospitals and acute care settings, but over the last decade resistant infections have been seen in the wider community too, including long-term care facilities for elderly people.

The evolution of resistant strains is a natural phenomenon that occurs when microorganisms replicate themselves erroneously or when resistant traits are exchanged between them. The pathways to synthesize antibiotics have been around for millions of years. For example, some microorganisms in the environment produce antibiotics naturally and they are used for communication and to compete with other organisms in their environment for space and resources. The organisms that produce natural antibiotics of course have to be able to protect themselves against the action of that antibiotic, and often carry resistance mechanisms. Other organisms in the same environment will also evolve over time, and resistant variants may be selected since they can survive nearby the antibiotic-producing organisms. Therefore, even before the introduction of antibiotics as medicines, antibiotic resistance mechanisms could be found.

For these reasons, the world has always been aware of AMR. Sir Alexander Fleming, who is credited with creating the first antibiotic, penicillin, in 1928, cautioned of the impending crisis while accepting his Nobel prize in 1945: "There is the danger that the ignorant man may easily under dose himself and by exposing his microbes to non-lethal quantities of the drug make them resistant." Since then, antibiotics have proved one of the most effective interventions in human medicine. Unfortunately, the overuse and misuse of this precious resource have brought us to a global crisis of AMR.[3]

The World Economic Forum has identified antibiotic resistance as a global risk beyond the capacity of any organization or nation to manage or mitigate alone, but in general there is little awareness of the

potential social, economic, and financial impacts of drug resistance. In developed economies, these include higher health care costs and decreases in the labor supply, productivity, household incomes, national income, and tax revenues. In the European Union alone, a subset of drug-resistant bacteria is responsible annually for some 25,000 deaths, with extra health care costs and lost productivity due to AMR amounting to at least EUR 1500 million. Similar analyses are needed for LMICs. Resistance to common veterinary antimicrobial medicines also results in food production losses, poor animal welfare, and extra costs.[3]

The threat of AMR is amplified by the poor clinical pipeline of new antimicrobials; in 2019, WHO identified 32 antibiotics in clinical development that address the WHO list of priority pathogens, of which only six were classified as innovative. Furthermore, a lack of access to quality antimicrobials remains a major issue. Antibiotic shortages are affecting countries of all levels of development and especially their health care systems.

To address this crisis, nearly seven decades after Fleming's lecture, in May 2015, the 68th World Health Assembly (WHA) passed WHA Resolution 68.7 and adopted the Global Action Plan on AMR. The resolution requested that member states develop or adopt country-specific action plans in line with the global plan. The objective was to have multisectoral national action plans in place by the 2017 World Health Assembly. The Global AMR Surveillance System (GLASS) was also instituted in September 2015. The overall goal of the action plan is to ensure, for as long as possible, continuity of the ability to treat and prevent infectious diseases with effective and safe medicines that are quality-assured, used in a responsible way, and accessible to all who need them.[2]

The Global Action Plan on AMR outlines five objectives:

- To improve awareness and understanding of AMR through effective communication, education, and training
- To strengthen the knowledge and evidence base through surveillance and research
- To reduce the incidence of infection through effective sanitation, hygiene, and infection prevention measures
- To optimize the use of antimicrobial medicines in human and animal health
- To develop the economic case for sustainable investment, taking account of the needs of all countries and to increase investment in new medicines, diagnostic tools, vaccines, and other interventions

To attain the above objectives, WHO recognized that "reducing antimicrobial resistance will require the political will to adopt new policies, including controlling the use of antimicrobial medicines in human health and animal and food production. In most countries, antibiotics can be purchased in markets, shops, pharmacies or over the Internet without prescription or involvement of a health professional or veterinarian. Poor quality medical and veterinary products are widespread, and often contain low concentrations of active ingredients, encouraging emergence of resistant microbes. Laws to ensure that medicines are of assured quality, safe, effective and accessible to those who need them need to be enacted and enforced."[2]

Nigeria, on November 26, 2016, approved the establishment of Nigeria's National AMR Coordinating Body at the NCDC. A National AMR Technical Working Group (AMR-TWG) was created and tasked

with conducting a situation analysis of AMR in Nigeria and designing a National Action Plan for AMR containment in the country; the group comprises stakeholders from human health, animal health, food animal production, and environment sectors. At the end of its activities and subject to the required procedures and approvals the National Action Plan (NAP) for Antimicrobial Resistance (2017–2022) was produced. The Nigeria plan identified the following priority gaps:

- Poor public awareness and weak coordination of AMR awareness activities by government and partners such as vertical disease control programs
- Poor "One Health" coordination of animal and human national disease surveillance systems, nonexistence of a national AMR laboratory surveillance system, and no dedicated funding for AMR control activities
- Nonexistence of a national infection prevention and control (IPC) coordinating body or guidelines, and poor budgetary support for IPC activities in health facilities (HFs)
- Lack of AMS in both private and public sectors
- A complete absence of studies on the health and economic impact of AMR in Nigeria and poor coordination of research on antibiotic use[5]

The goal of the action plan is to reduce, prevent, and slow the evolution of resistant organisms and their impact on health care while ensuring optimal use and improved access to effective, safe, and quality-assured antimicrobials for continued successful management of infections. The plan incorporates the One Health Approach and aims at implementing proposed actions by strengthening and using existing national systems or by creating new structures where they do not exist. The focus areas are essential the same with the objectives of the global plan. These are:

- Increasing awareness and knowledge on AMR and related topics
- One Health AMR surveillance and research
- IPC in the tripartite sector
- Promote rational access to antibiotics and AMS
- Invest in research to quantify the cost of resistance and develop new antimicrobials and diagnostics[4]

A careful and responsible approach is important to reducing overuse and misuse of antibiotics by promoting rational use of antimicrobials through the concept of AMS. In the human population, ensuring prudent prescribing across different communities and settings, in different patient populations, in diverse geographies, resource levels, and cultures requires a truly innovative, flexible, collaborative, and cross-disciplinary approach.

Both the global and Nigeria plans have the objective to optimize the use of antimicrobial medicines in human and animal health, and focus on promoting rational access to antibiotics and AMS. This requires the implementation of a well-coordinated program and activities taking advantage of and strengthening existing structures, policies, legislations, and regulations, as well as the filling of identified gaps. However, it is important to stress that this objective cannot be achieved in isolation; its realization is equally dependent on the implementation of the other four objectives, all of which are interwoven.

AMS has been defined as "the optimal selection, dosage, and duration of antimicrobial treatment that results in the best clinical outcome for the treatment or prevention of infection, with minimal toxicity to the patient and minimal impact on subsequent resistance." It also can be defined as an "organisational or

health care system-wide approach to promoting and monitoring judicious use of antimicrobials to preserve their future effectiveness." A more clinical definition that, perhaps, is practical and an engaging message for clinicians is "The Right Antibiotic for the Right Patient, at the Right Time, with the Right Dose, and the Right Route, Causing the Least Harm to the Patient and Future Patients."[3]

AMS is one of three "pillars" of an integrated approach to health systems strengthening. The other two are (1) IPC and (2) medicine and patient safety.

AMS helps to control AMR by optimizing the use of antimicrobials. Linking all three pillars to other key components of infection management and health systems strengthening, such as AMR surveillance and adequate supply of quality-assured medicines, promotes equitable and quality health care toward the goal of achieving universal health coverage. AMS principles also apply to the use of antimicrobials in the animal and agriculture sectors, typically with an emphasis on the responsible and prudent use of these agents.[4]

3. SITUATION ANALYSIS

A situation analysis is the process of gathering and evaluating information on the internal and external environments of a program/project to assess its current strengths, weaknesses, opportunities, and threats (SWOT), and to guide its goals and objectives. This in turn helps with devising a strategy to move forward from the current situation to the desired situation. This report will therefore make the attempt to:

- Identify and strengthen achievements made so far in the implementation of the AMR NAP
- Identify the gaps in the implementation of the AMR plan and factors responsible
- Proffer recommendations to sustain and strengthen the implementation of the AMR NAP

3.1. METHODOLOGY OF THE ANALYSIS

The preparation for this report involved a systematic review of legislation, regulations, administrative requirements, and other instruments related to drug management. The legal and institutional framework for the management of antimicrobials in human health as well as its capacity to ensure their rational management, was assessed. The aim is to identify the strength, weaknesses, opportunities, and threats; and to propose recommendations for improving antimicrobial management policies in Nigeria. The analysis methodology consisted of four points:

- Review of local and global publications on AMR
- Review of legal and regulatory instruments
- Use of the MTaPS management policy analysis tool
- Conducting interviews with key actors

3.2. HEALTH CARE DELIVERY SYSTEM IN NIGERIA

Nigeria is the most densely populated country in Africa. It has an estimated population of 212,234,999 as of September 13, 2021, based on Worldometer elaboration of the latest United Nations data.[6] It is a

federation, made up of 36 states and a Federal Capital Territory at Abuja, and 774 Local Government Areas, operating a three-tiered government structure.

3.2.1 Orthodox health care services in Nigeria are provided by both private and public sectors, with the public health service organized into primary, secondary, and tertiary levels. Community health workers (CHWs) provide the majority of the services at primary care centers. Secondary care facilities provide both general medical services as well as some specialized health services. Tertiary facilities, which provide the highest level of health care in the country, are also training centers for medical students; they include specialist/teaching hospitals and federal medical centers. There is a tertiary facility in every state, with a few having more than one. The National Health Act 2014 (Section 1[2d]) and the National Health Policy ascribes responsibilities for primary health care to the local governments, secondary care to the states, and tertiary care to the federal government.

In 2020, it was estimated that 20,226 HFs existed in Nigeria, with 85.2% being primary health care facilities, 14.4% secondary facilities, and 0.4% tertiary care facilities. By sector, 73.1% of facilities are public and 26.9% are private.[7]

In addition to the pharmacies in all tertiary, secondary, and primary health care facilities, there are 4,444 licensed pharmacies and 194,634 patent medicines stores as of 2020.[8] Only a few pharmacies in public hospitals are licensed.

According to the Medical Laboratory Science Council of Nigeria (MSLCN), the laboratory of the General Hospital Bwari in Federal Capital Territory (FCT) is the only registered and accredited (ISO 15189) laboratory; and there 20 accredited private medical laboratories.[9] The failure of public HFs to register or obtain practicing licenses is discouraging, as the quality of services being rendered is suspect.

A 2017 report showed that an estimated 77.5% of current health expenditure (CHE) in the country was financed out-of-pocket (OOP). Approximately 66.6% of the health expenditure was spent on infectious and parasitic diseases. Curative care services were the dominant function on which health expenditures were spent, accounting for 36.2% of current health spending. Pharmaceuticals and other medical goods not specified by function accounted for 31.6%. Preventive care services were the third major health care function by value, accounting for 12.1% of CHE.[9]

High OOP expenses and curative care service, along with low levels of preventive care services, are factors that in one way or the other indicate or may lead to improper use of antibiotics, and subsequently may promote AMR.

3.2.2 Traditional medicine still enjoys tremendous patronage from the populace, in both the rural and urban areas. Several efforts have been made by the country to develop the field, culminating in the establishment of a Traditional Medicine Division in 2007 and its upgrading to a full-fledged department in 2018.

In 2007, a bill for the establishment of a Traditional Medicine Council of Nigeria was forwarded to the National Assembly by the Federal Executive Council (FEC); the bill was not passed by the assembly. However, with the passage of time it became necessary to review the bill; this has been done and it was

approved in October 2020 by the FEC specifically to establish a Council for Traditional, Alternative and Complementary Medicine Practice in Nigeria. It is still awaiting transmission to the National Assembly.

It is gladdening to know that some states have, however, established Traditional Medicine Boards as envisaged by the national bill.

3.3. ANTIMICROBIALS USE IN NIGERIA

High levels of use and misuse of antibiotics are still prevalent in Nigeria. A study titled "Antibiotic Use among Hospitalized Patients in Northern Nigeria: A Multi-Centre Point-Prevalence Survey" revealed that the prevalence of antibiotic use was high, with one in three prescriptions including a broad-spectrum antibiotic. Prolonged use of surgical antibiotic prophylaxis and redundant antibiotic combination were observed. Overall, 80.1% (257/321) of patients in the study used at least one antibiotic on the day of the survey. The prevalence of antibiotic use ranged from 72.9% in obstetrics and gynecology to 94.6% in pediatric medical specialty. Community-acquired infections (38.7%) and surgical antibiotic prophylaxis (22.5%) were the most common indications. Surgical antibiotic prophylaxis was used or scheduled to be used for more than a day in all cases. Metronidazole (30.5%), ciprofloxacin (17.1%), ceftriaxone (16.8%), amoxicillin-clavulanate (12.5%), and gentamicin (11.8%) were the most commonly prescribed antibiotics. Overall, broad-spectrum antibiotics represented one-third of all the prescriptions. The change of initial antibiotic prescription was reported in one-third of the patients; the reasons include a switch to oral antibiotic (28.5%), escalation (4.5%), and de-escalation (3.6%). Of the 257 patients with an antibiotic prescription, 6.2% had redundant antibiotic combinations. The authors advised that AMS interventions are recommended to reduce the use of antibiotics and promote appropriate antibiotics prescribing.[10]

Among the general populace, the use of antibiotics is still very high and unsatisfactory. It was observed that among university students in Nigeria the use of antibiotics was not only high but also irrational. Irrational use included use of antibiotics for wrong indications, incomplete course of treatment, and self-medication. The reasons given for use of antibiotics revealed that respondents do not know the difference between bacterial and viral infections and therefore use antibiotics for both kinds of infections.[11] Breastfeeding mothers were found to have a lack of knowledge about the use of antibiotics and antibiotic resistance, which results in bad habits and inappropriate attitudes when it comes to using antibiotics; 64.07% declared that they obtained their drugs from a pharmacy or medical store, with the most commonly used being penicillins (65.53%), nitroimidazoles (14.04%), quinolones (7.23%) and co-trimoxazole (4.68%).[12]

3.4. KNOWLEDGE AND PRACTICE OF ANTIMICROBIAL STEWARDSHIP

A 2017 report to WHO, by students and staff of the AMR Centre at the London School of Hygiene & Tropical Medicine on antibiotic prescribing and resistance from low- and middle-income countries (including Nigeria) prescribing and dispensing professionals fund across these studies echo the calls from multilateral agencies for a holistic approach to addressing AMR. Contrary to the expectation of the need for more education to improve knowledge of AMR, this report finds that awareness of resistance among these health professionals—who were qualified prescribers and dispensers —was high. An important

distinction can be made between *knowledge* of AMR and prudent use of antibiotics, and *information* about versus information needed to *address* AMR, which was lacking in terms of local patterns. Health professionals believed they could be more targeted about antibiotic treatment if they had access to this information. However, from our respondents' perspectives, the concerns over risks of nontreatment will remain while the infrastructure to allow follow-up, provide confidence in hygiene standards, and ensure adequate time and investigation of each patient remains poor."[13]

Another study on drug use patterns in south Nigeria showed good prescribing indices regarding injections and antibiotics but a higher index of polypharmacy, poor utilization of the Essential Medicines List (EML), and lack of adherence regarding generic prescribing compared with previously obtained regional recommended optimal values.[14]

Furthermore, a similar study, "Drug Prescription Pattern in a Nigerian Teaching Hospital," also revealed poor compliance to the WHO core prescribing indicators: polypharmacy, poor antibiotics and injection use, and lack of compliance to EML prescriptions.[15] These findings are reinforced by those of another report, "A Review in the General Outpatient Clinic of a Nigerian Tertiary Hospital," which again revealed poor compliance to the WHO core prescribing indicators in the form of polypharmacy, poor antibiotics and injection use, and lack of compliance to EML prescriptions. The report advised that "there is need for adequate monitoring of prescription writing in the health facility in order to ensure rational drug use."[16]

The authors of a 2019 report titled "Antimicrobial Resistance and Stewardship Knowledge and Perception among Medical and Pharmacy Students in Nigeria" observed that the practice of AMS in Nigerian hospitals is very limited and the subject is rarely included in undergraduate medical and pharmacy curriculums. The study evaluated the knowledge and perceptions of a cohort of Nigerian medical and pharmacy students in concepts of AMR and ASP, observed that more pharmacy students had formal training on ASP compared with medical students (41.3% vs. 27.5%, $p < 0.05$). Pharmacy students (n = 84.3% and 90.5%) were significantly more knowledgeable of factors that promote the spread of AMR and interventions to combat resistance than medical students (n = 73.9% and 82.3%), respectively. Interestingly, 23.3% of medical students thought pharmacists should lead ASP teams, while 5.8% of pharmacy students thought doctors should lead ASP. However, both held poor perceptions of each other's roles in the ASP team. The authors concluded that knowledge of AMR and ASP among medical and pharmacy students in Nigeria is lacking. Interprofessional collaboration to change perceptions and drive ASP in urgently needed.[17]

Gaps and challenges to the implementation of the AMS among the hospitals were identified in a cross-sectional survey of 20 federal, state and private tertiary hospitals randomly selected from the six geopolitical zones of Nigeria, involving the use of an adapted WHO tool on AMS to collect data in each hospital on the following: the existence of AMS committee, accountability and responsibility, AMS actions, education and training, M&E, IPC practice, facilities to support AMS, and challenges to AMS implementation. The report indicated that only 6 (30%) of the 20 hospitals had AMS committees, while only 2 (10%) had any evidence of leadership commitment to AMS. There was no regular AMS-related education or training, monitoring, evaluation, or reporting activities in the hospitals, except in 7 (25%) that had participated in the global point prevalence survey (Global-PPS) of antimicrobial use and resistance hosted by the University of Antwerp, Belgium. Challenges impeding AMS activities were found

to include lack of human and financial resources, prescribers' opposition, lack of awareness, and absence of AMS committees. Most of the gaps and challenges bordered on seeming lack of knowledge and inadequate communication among prescribers and other stakeholders.

The report concluded that AMS, a proven strategy in curbing AMR, is established in only a few tertiary HFs in Nigeria, and that there is a need for intense education and training activities for prescribers and other stakeholders, including but not limited to hospital administrators.[18]

In another survey, which assessed the perception, attitudes, and practices of the AMS program among health care workers, and also sought to determine the factors that influence the attitude and practices of the AMS program among research participants at the Nnamdi Azikiwe University Teaching Hospital, Nnewi, Nigeria, it emerged that only 6 (3.75%) of the respondents considered their knowledge and skills of AMS program as excellent while 54 (33.5%) of them did not consider AMR to be a serious health care problem. The study showed overall poor knowledge and poor attitudes toward the practice of the AMS program, and further that there is a need to create awareness through regular training and retraining of health care personnel as well as the development of functional AMS programs within our teaching hospitals to align with global trends and best practices.[19]

3.5. CURRENT STATUS OF AMR/AMS IMPLEMENTATION IN NIGERIA

Focus area 4 of the National Action Plan to combat AMR identified a few strategic interventions in AMS. The operational plan lists several high-level activities on stewardship:

- Improve access to quality antimicrobial agents for infections in humans and animals
- Promote optimal procurement and distribution of quality antimicrobials and diagnostics for human and animal use
- Enhance local production of quality antimicrobial agents and diagnostics for human and animal use
- Expand National Health Insurance Scheme (NHIS) coverage to include more enrollees
- Promote AMS in human and animals
- Promote the use of up-to-date treatment guidelines and ensure prudent use in humans and animals
- Promote optimal prescribing and dispensing of antimicrobials in humans and animals
- Strengthen regulatory agencies across all sectors (humans, animals, and environment) to enable them to perform their mandate with regard to antimicrobials
- Strengthen the capacity of regulatory agencies across One Health sectors (human, animals, food products, and environment)
- Enhance intersectoral coordination and collaboration between/among regulatory agencies

The above is strengthened by WHO Benchmark 2019 in section 3.4 (Optimize use of antimicrobial medicines in human and animal health and agriculture), urging members with no or weak policies and regulations to:

- Undertake an assessment of stewardship policies and activities, including regulatory framework and supply chain management of antimicrobials, using a multisectoral approach.
- Review the essential medicines list and clinical guidelines that promote appropriate use
- Assess existing monitoring of antimicrobial use and consumption

- Develop a draft national AMS plan or strategy and national legislation that regulates use, availability, and quality of antimicrobials[12]

The Country-Led Midterm Joint External Evaluation of IHR Core Capacities, 2019, recorded progress on the AMR program in the country as follows:

- Passage of the Act establishing the Nigeria Centre for Disease Control (2018) by the President of the Federal Republic of Nigeria in 2018, establishing NCDC as a statutory government agency with the mandate for communicable disease control; it is mandated to serve as the AMR Coordinating Body for the country.
- The One Health AMR Technical Working Group was formally inaugurated; it conducted a situation analysis and developed a National Action Plan for AMR (now due for review).
- The AMR Coordinating Committee (AMRCC) coordinates regular meetings with the AMR-TWG (at least three times a year),
- The NCDC National Reference Laboratory (NRL) and National Veterinary Research Institute (NVRI) have been established and designated as the AMR National Reference Laboratory, for human health and animal health, respectively,
- Nine human health and six animal health sentinel laboratories have recently been assessed by the AMRCC as part of the AMR surveillance network; human health laboratories have commenced generation of data on six priority pathogens from blood cultures (*Staphylococcus aureus, Streptococcus pneumoniae, Klebsiella pneumoniae, Acinetobacter* spp., *E. coli,* and *Pseudomonas aeuroginosa*).
- The capacity and capability of laboratories on AMR surveillance are being built with support from partners.
- AMR surveillance data across human health laboratories are collected using WHONET software, with reports routinely shared and collated with NCDC and subsequently reported to GLASS.
- Collection, collation, and reporting of animal health AMR surveillance leverages the existing National Animal Disease Information Service (NADIS) surveillance structure.
- National IPC guidelines have been developed.
- The Turn Nigeria Orange (TNO) initiative, to optimize 30 HFs to become IPC centers of excellence, has been implemented. The focus is on increasing access to water, sanitation, and hand hygiene. Training on behavioral change to improve uptake of IPC measures (participatory approach) has been ongoing since 2017. Training has been completed in 15 (of 23) targeted hospitals with facilitators from NCDC, organized by the FMOH through support from the Robert Koch Institute (RKI),
- A biosecurity plan for avian influenza and African swine fever is available and has been implemented and applied within the animal health AMR NRL and in sentinel surveillance laboratories. The plan includes safe transportation of samples to the different identified laboratories for relevant tests.
- Review of the Animal Disease Control Act (2006) has commenced and awaits presidential assent.
- The following steps have been takin to optimize use of antimicrobial medicines in human and animal health and agriculture:

    - Retrieval of import data on current animal health sources of antimicrobial usage from the National Regulatory Authority and National Agency for Food and Drug Administration and Control (NAFDAC).

- Reporting of antimicrobial usage data to the World Organisation for Animal Health (OIE) Global database.
- Commencement at four tertiary hospitals of human health sentinel sites for the conduct of AMS activities. These include Lagos University Teaching Hospital; Obafemi Awolowo University Teaching Hospital, Ile Ife; National Hospital Abuja and University College Hospital, Ibadan.
- Commencement of a national point prevalence survey on antibiotic use to be completed by January 17, 2020
- An increase in the number of community pharmacies reporting data on antibiotic consumption (humans) from 71 to 123 over a 2-year period (2017 to date) across Nigeria in a total of 23 states.

However, the report also reported some implementation challenges, including:

- Delay in assent to the amended Animal Disease Control Act by the President of the Federal Republic of Nigeria. This is despite the Animal and Disease and Control Act (Repeal and Reenactment) Bill, 2019 (H.B. 1268) being passed into law by the Senate on April 9, 2019.
- Nonexistence of specific regulation for AMS in both human and animal health component.
- Nonexistence of surveillance protocols/ guidelines in animal health.

The report further noted that the animal health sector does not currently participate in external quality assurance (EQA) assessments. While IPC guidelines exist for human health, none exist for the animal health sector. It advised that to optimize the use of antimicrobial medicines in human and animal health, there is a need for the publication of documents addressing appropriate use, availability, and quality of antimicrobials.[19]

Though Nigeria is not listed in the "Country, territories, and areas profiles" section of the GLASS Report 2021, which summarizes surveillance activities participation for AMR, AMC, HIV infection, TB, malaria, and focused surveillance (Tricycle and EGASP); data nevertheless show progress in the development and strengthening of national AMR surveillance systems over the past three years.

However, high rates of resistance among antimicrobials frequently used to treat common bacterial infections have been observed. The new AMR indicators in the Sustainable Development Goal monitoring framework, the frequency of bloodstream infections due to methicillin-resistant *S. aureus*- and *E. coli*-resistant to third generation cephalosporins, were reported by 25 and 49 countries, territories, and areas, respectively.

The reported high rates of resistance in common human infections are also disquieting. If confirmed, the reported very high rate of resistance in common pathogens causing urinary tract infections (UTIs) to a first-line drug such as co-trimoxazole (around 50%) may preclude empirical treatment with this agent and hamper efforts to rationalize the use of antimicrobial medicines toward as narrow a spectrum of activity as possible. This problem highlights the importance of obtaining specific AMR information to inform national treatment guidelines. Crucially, emergence of a carbapenem-resistant organism has also being reported.[20]

3.6. GOVERNANCE AND INSTITUTIONAL FRAMEWORK

3.6.1 Governance and Coordination

In 2017, Nigeria produced and launched a National Action Plan on AMR (2017–2020); this was consequent upon the passage WHA resolution 68.7 and also adopted the Global Action Plan on AMR by the 68th World Health Assembly in May 2015. The resolution requested that member states develop or adopt country-specific action plans in line with the global plan.

The plan, in line with the One Health Approach, was endorsed by the Honorable Ministers of Agriculture and Rural Development, Environment, and Health. However, considering the importance of water in the water, sanitation, and hygiene (WASH) program, and the strategic place of WASH in IPC, it is important to work closely with the Federal Ministry of Water Resources.

AMR activities in Nigeria are being coordinated by the NCDC, which serves as the secretariat. A National AMR Technical Working Group was created, comprising stakeholders from the human health, animal health, food animal production, and environment sectors. The AMR-TWG, which meets quarterly, carried out a situation analysis of AMR in Nigeria and designed the National Action Plan for AMR containment in the country. The AMR Coordinating Committee also coordinates regular meetings with the AMR-TWG.

A Technical Working Group on AMS has been established, and it is functional. This committee also meets quarterly; its meetings are held just before the main AMR-TWG meetings.

There is also a Committee of Practice group (COP), which meets regularly to review relevant topics on the use of antibiotics.

The AMR National Action Plan governing structure provides for a steering committee at its apex; this committee comprise the Honorable Ministers of Agriculture and Rural Development (FMA&RD), FMOH, and the Federal Ministry of Environment (FMEvr). The steering committee's role is to provide the overall policy direction for the containment of AMR in Nigeria. However, this crucial committee has not yet been launched. Additionally, the NCDC does not periodically send feedback reports to any of the three ministers on the committee.

3.7. POLICY, LEGISLATIVE, AND REGULATORY FRAMEWORK

3.7.1 National Drug Policy

Nigeria has a National Drug Policy (NDP), which was first published in 1990 with the goals "to make available at all times to the Nigerian populace adequate supplies of drugs that are effective, affordable, safe and of good quality; to ensure the rational use of such drugs; and to stimulate increased local production of essential drugs." The policy has been reviewed twice, in 2005 and 2021, however, with a modified goal of making adequate supplies of drugs that are effective, affordable, safe, and of good quality available at all times to the Nigerian populace.

The NDP 2021 edition, however, has adequate objectives to ensure careful and responsible use of antimicrobials, as follows:

- To ensure efficient and drug management in the public and private sectors
- To ensure equitable access to safe, efficacious, good, quality, and affordable drugs at all levels of health care delivery
- To promote rational use of by prescribers, dispensers, and consumers
- To promote local production of drugs and pharmaceutical raw materials through favorable policies and advocacy for local manufacturing
- To promote the export of locally produced drugs
- To establish a well-ordered system in Nigeria
- To strengthen institutions responsible for the administration, legislation, and regulation of the NDP
- To promote pharmaceutical research and development in support of the NDP
- To strengthen postmarketing surveillance and pharmacovigilance (PV) for all drugs, including traditional medicines
- To reduce the burden of AMR
- To reduce and prevent the menace of drug and substance abuse
- To strengthen the use of data for evidence-based decision making in the implementation of the NDP
- To enlist government commitment at all levels in the implementation of the NDP
- To harmonize and optimize international collaborations and support to achieve the objectives of the NDP

The policy set various targets to be attained towards the realization of these objectives. These targets include:

- 80% adherence to the Essential Medicines List in the public sector by August 31, 2020
- 50% implementation of the National Drug Distribution Guidelines by December 31, 2022
- 100% compliance with National Guidelines on Drug Donations by June 30, 2022
- 80% awareness of the concept of rational drug use among prescribers, dispensers, consumers by June 30, 2022, and 60% adherence to rational drug dispensing and use by June 30, 2023
- Institutionalization of functional Medicines and Therapeutic Committees (MTCs) and drug information centers in all secondary and tertiary HFs by December 31, 2025

One of the key strategies in the policy for the achievement of the above goals and objective is AMS, it hopes, in Section 4.16.7 AMS) to do this by:

- Development of a national policy on AMS
- Creating awareness on the use of antimicrobials
- Strengthening IPC
- Strengthening of laboratory services
- Strengthening of the surveillance system
- Promotion of rational use of antimicrobials
- Research and development[21]

Other policies and guidelines deriving from the NDP include:

- Nigerian National Pharmacovigilance Policy and Implementation Framework, 2020
- Nigeria Supply Chain Policy for Pharmaceuticals and other Healthcare Products, 2016;
- National Quality Assurance Policy for Medicines and Other Health Products, 2015

- National Drug Distribution Guidelines, 2012
- Guidelines for Donation of Medicines and Health Care Equipment in Nigeria, 2007
- Standard Treatment Guidelines, 2016

There is a need to closely work with the FMOH and other stakeholders and partners in the implementation of these policies and guidelines; this can be done by incorporating elements of this policy in the AMS National Plan.

3.7.2 Essential Medicines List, MTCs, and Standard Treatment Guideline

These key strategies of the NDP to ensure the availability of safe, efficacious, good, quality drugs and their rational.

The EML is backed by the National Drug Formulary and Essential Drugs List Act No 43 of 1989; the act was passed with a first list and formulary. The EML has been reviewed six times and is now in its seventh edition (2020); the formulary is currently being reviewed by the review committee established by the act. A standard treatment guideline (STG) was produced in 2008 and reviewed in 2016.

The AwaRe classification of antibiotics in line with WHO recommendations is not reflected in the latest edition of the EML; the process for integrating the classification has commenced with the ministry and it is hoped that the next edition will reflect this or that an addendum to current one will be issued.

The review committee is a multidisciplinary one and its work is guided by relevant WHO publications, which are considered and adopted to make appropriate recommendations to the Honorable Minister of Health for approval. The committee in doing its work also receives and reviews applications from stakeholders for inclusion or removal of medicines from the list.

Survey reports and projects on the EML and STG, while showing high awareness of these policy documents, revealed low compliance with their application in procurement, prescribing, and dispensing of medicines in the country as specified by the NDP. This is despite the provision of the National Drug Policy that specifies that "the Essential Drugs List shall be used for:

- Procuring drugs and their use in the public sector
- Prescribing drugs in the public sector
- Drug information to health care providers
- Producing STGs and a National Formulary
- Reimbursing for drugs in the National Health Insurance Scheme

It is reported (by the NCDC) that only 25% of the nation's tertiary hospitals have MTCs, while these barely exist at secondary health care facilities.

3.7.3 Pharmacovigilance and Post-Marketing Surveillance

PV is one of the strategies of the NDP, officially called the Nigerian National Pharmacovigilance Policy, first launched in 2012 and reviewed 2020. The implementation of the policy is domiciled in NAFDAC, where a National Pharmacovigilance Centre (NPC) is located in the Directorate of Pharmacovigilance and Post-Market Survey. There are also six zonal centers, hosted by tertiary health institutions; each

secondary and tertiary HF is encouraged to set up a PV committee. The vision of the policy is to ensure rational use of medicines in Nigeria

The NPC was created in 2004 with the following responsibilities:

1. Implementation of national policy on PV and provision of science-based advice and information on safe use of medicines
2. Post-marketing surveillance of all regulated products in collaboration with marketing authorization holders (MAHs)
3. Coordination of PV activities nationwide
4. Implementation of risk management plans (RMPs) in collaboration with the pharmaceutical industry and MAHs as appropriate
5. PV inspections
6. Supporting the establishment of active PV centers in health care institutions in the country
7. Creation of awareness on PV among health professionals, health care providers, marketing authorization holders, and the general public
8. Promoting rational and safe use of medicines in Nigeria
9. Medicine safety communication
10. Coordinating the activities of the National Drug Safety Advisory Committee (NDSAC)
11. Sourcing, collating, preserving, storing, retrieving, and dissemination of information on food, drugs, cosmetics, chemicals, and other NAFDAC-regulated products
12. Establishing and maintaining a functional national database on adverse drug reactions (ADRs) and other medicine-related problems.
13. Conducting regular surveys on regulated products for required regulatory action
14. Coordinating the activities of NAFDAC Consumer Safety Club (NCSC) in secondary schools and NAFDAC Consumer Safety Publications (NCSP)

The PV/PMS Directorate receives, sources, and alert notifications on recalls and safety information on regulated products. On review of the safety information received, timely alert notices and safety communication ("Dear Health Care Provider" letter) are provided to the public and health care providers, respectively. The NPC has received 23,993 reports since its inception' causality assessment for over 80% of these reports have been concluded and uploaded to WHO database (Vigiflow).

To further ensure the safety of medicinal products that it regulates, NAFDAC in 2016 first published NAFDAC Good Pharmacovigilance Practice Regulations, and these were reviewed 2021. The regulations describe the obligations of the Certificate of Registration Holder to set up a system for PV to collect, collate, and evaluate information about suspected adverse reactions to products it puts into the Nigerian market. The ultimate goal is to ensure that medicinal products entering the Nigerian market are safe and effective and to continue to provide a satisfactory balance between their benefits and risks.

Not much is known to have been done on PV in the states, and some of our tertiary hospitals. There is a need to advocate for the entrenchment of PV across the health sector.

3.7.4 Legislations

There are several pieces of legislations related to drugs; while some preceded the formulation of the National Drug Policy in 1990, many of them emanated from the policy. Key legal instruments relevant to AMS are the following:

1. Pharmacists Council of Nigeria Decree, 91, 1992 (Cap P17 LFN 2004)—now being reviewed, awaiting the assent of the president
2. Poison and Pharmacy Acts, Cap 535, 1946
3. National Food and Drug Administration and Control Decree 19, 1993 (Act Cap N.1 LFN 2004)
4. Drugs and Related Products (Registration, etc.) Decree, 1993 (Act Cap F.33 LFN 2004)
5. Drugs and Related Products (Registration, etc.) Amendment Decree 20, 1999
6. Food and Drugs Act, 1976
7. Food and Drugs (Amendment Decree 1999[22]

The above acts, among others, regulate the practice of the pharmacy profession—including registration of pharmacists and pharmacy technicians; registration of premises for food and drugs manufacturing and handling; inspection of these facilities; prescription and dispensing of certain class of drugs (including antibacterials) only on the order of a signed prescription given by a registered qualified medical, dental, or veterinary surgeon. They also specify offences and penalties. Some of these acts are currently being reviewed to engage with emerging issues.

The implementation of these acts is being faced with some challenges regarding interpretation, and the roles and responsibilities of relevant health care professionals and their respective regulatory bodies. In some instances, the challenge borders on disregard of the law despite court pronouncements.

There is therefore a need to harmonize these laws in accordance with relevant organs such as the Nigeria Law Reform Commission and the Office of the Attorney General of the Federation in order to ensure their effective and efficient implementation.

3.8. MANUFACTURERS AND PRODUCTS REGISTRATION

The act establishing the National Agency for Food and Drug Administration, specifically Section 5(f), and others acts cited above, as well as the Drug and Other Products Registrations 2019, stipulate that the registration of food, drugs and other regulated products is a mandate of the agency. This is supported by additional regulations on labeling and advertisement. However, there are no specific regulations on antimicrobials, except the provisions of Good Manufacturing Practices (GMPs) for a certain class of antibiotics. The registration of drugs is being enforced through routine a monitoring and enforcement exercise.

In addition, under the provisions of the above acts the Pharmacist Council of Nigeria (PCN) is mandated to register all premises in which pharmaceutical businesses are to take place: be they manufacturing, importation, wholesale, distributor, retailing, or veterinary or hospital pharmacy. The PCN either alone or as part of various task forces carries out inspection and enforcement exercises. In 2020, the PCN visited a total of 1,104 pharmacies and 5,028 patent medicine stores; as a result, 353 pharmacies and 3,866 patent medicine stores were shut down.[22]

3.9. MEDICINES CLASSIFICATION AND CONTROL

NAFDAC is tasked with the classification of drugs as controlled, prescription-only medicines (POM) or over-the-counter (OTC) medicines. The first two categories may be handled only by registered prescribers and dispensers, whereas proprietary patent medicine vendors are permitted to handle OTC products. Controlled medicines require additional regulatory control in line with the provisions of the Dangerous Drug Act, 1935, and NAFDAC regulations. As part of the regulation of controlled medicines, manufacturers, importers, wholesalers, distributors, retailers, and hospital pharmacies are to provide return of utilization through the "Disposal of Poison Book" (Form B). There may be need to introduce a similar procedure for certain antibiotics to be supervised by the MTCs in each health care facility, without its criminal components.

3.10. MEDICINES SUPPLY CHAIN

Contrary to the goal and objective of the NDP, 2nd Edition (2005), to stimulate and increase local production, the nation still depends largely on imported medicines, mainly from China and India, for its health care system. There is no local production of active pharmaceutical ingredient (API). The advent of the COVID-19 pandemic, when exporting nations had to introduce restrictions on exportation of both finished products and APIs, demonstrated the vulnerability of the nation and the need to step up local production of medicines. Available records from NAFDAC indicate that there are 1,643 registered imported products and 330 registered locally produced products, accounting for 80% and 20%, respectively, of the market. Only 10% of registered antibiotics are produced locally.[23] This situation also makes it a bit more difficult for NAFDAC to ascertain the qualities of the imported products, despite the measures put in place by the agency.

This situation is compounded by the unsatisfactory distribution of drugs in the country. The parallel importation of unregistered products and the proliferation of unlicensed patent medicines stores are of great concern; it is estimated that for everyone licensed patent medicines store there are close to 100 unlicensed ones. Furthermore, the larger chunk of drugs distribution in Nigeria is through the unregulated open markets across the nation.

In response to this situation, the country launched its National Drug Distribution Guidelines (NDDG) in 2009; however, without being implemented the guidelines were reviewed in 2012. The NDDG observed that "One of the major challenges of the pharmaceutical sector and the health care delivery system of Nigeria is the uncoordinated drug distribution system which is not in line with the good drug supply management that the National Drug Policy envisaged."

The goal of the NDDG is to establish a well-ordered drug distribution system for Nigeria. Its objectives are as follows:

- To ensure efficient and effective drug supply management in the public and private sectors
- To ensure that all drugs in the national drug distribution system are safe, efficacious, effective, affordable, and of good quality
- To ensure access to good quality and affordable drugs at all levels

The guidelines were reviewed and revised to accommodate the open markets with the creation of Coordinated Wholesale Centres (CWCs); seven of these (Aba, Ibadan, Kaduna, Kano, Lagos, Oba, and Owerri) have been approved by the PCN, but construction has only commenced on two (Kano and Lagos). The creation of the CWCs is intended to ensure the availability of a controlled and regulated environment for drug distribution in Nigeria. However, after 12 years and several deadlines the NDDG remain largely unimplemented.

There are two federal government–owned central medical warehouses, in Lagos and Abuja; they are supported by six zonal warehouses across the country. These warehouses handle only disease program drugs. Available records also indicate that two of 36+1 states of Nigeria do not have a central medical warehouse.[24]

The tertiary HFs are responsible for procurement and management of drugs and related health commodities use in their facilities. The approved management system is the Drug Revolving Fund Scheme (DRF), which is one of the strategies for the implementation of the NDP. DRF is to be coordinated in each facility via a multidisciplinary committee headed by the head of pharmacy of each facility. This process, however has not gone smoothly; the DRF has been grounded in almost all facilities due to heavy indebtedness to the suppliers of drugs.[21]

3.11. REGULATION ON PRESCRIBING AND DISPENSING OF ANTIBIOTICS

There are no specific regulations or legislation on the prescribing of antibiotics. Access to this class of medicines is provided for by the Poison and Pharmacist Act (1946 and 1947), which categorized them as prescription-only medicines. They can be obtained only by a signed order of licensed medical, dental, or veterinary surgeon, and dispensed only by a licensed pharmacist. Exception to this is the availability of dispersible amoxycillin tablets for pneumonia at the primary health care level.

Antibiotics are readily accessed without prescription by citizens, however, through the regulated pharmacies and patent medicines stores as well as from the medicine hawkers.

Attempts in the past by the Federal Ministry of Health to produce a legally binding prescription policy has not been successful.

3.12. INFECTION PREVENTION AND CONTROL

WHO describes IPC as a practical, evidence-based approach that prevents patients and health workers from being harmed by avoidable infection and as a result of AMR. Every infection prevented is one that needs no treatment. Prevention of infection can be cost-effective and implemented in all settings and sectors, even where resources are limited. Good sanitation, hygiene, and other infection prevention measures that can slow the development and restrict the spread of difficult-to-treat antibiotic-resistant infections are a "best buy." Effective IPC requires constant action at all levels of the health system, including by policy makers, facility managers, health workers, and those who access health services. IPC is unique in the field of patient safety and quality of health care, as it is universally relevant to every health worker and patient, at every health care interaction. Defective IPC causes harm and can kill. Without effective IPC, it is impossible to achieve quality health care delivery.

WASH is very central to effective IPC: The 2019 report *Water, Sanitation, Hygiene National Outcome Routine Mapping Nigeria,* released by the Federal Ministry of Water Resources revealed that "up to 171 million people are off the SDG target for access to safely managed drinking water supply services with only 14% of the population having access to safely managed drinking water supply services."

Two-thirds of the population (66%) use drinking water supplies contaminated by thermotolerant coliform at the point of collection, and a similar proportion (63%) ingest contaminated water at the point of consumption within the household.

National indicators for access to sanitation show a slight improvement, though rates of open defecation are still very high; 44% of the population were found to have access to basic sanitation services in 2019, compared with 42% in 2018. Despite this progress, the number of people practicing open defecation barely changed since 2018, as about one-quarter of the population (23%), or 46 million people, still defecate in the open. Only 17% of the population use improved sanitation facilities that have a proper handwashing facilities and soap.

The survey showed a decrease in access to hygiene services between 2018 and 2019; 16% of the population were found to use basic hygiene services in 2019 compared with 21% in 2018. Despite high awareness of handwashing times, only 12% of household heads consider handwashing after cleaning up a child's feces or changing a child's diaper and only 1 in 10 household heads were able to demonstrate proper handwashing techniques with soap under running water.

More than half (55%) of HFs had basic water supply services while 17% of hospitals did not have any water source at all. Only 1 in 10 HFs (10%) have basic sanitation services and slightly more than one-quarter of HFs (26%) do not have toilets/latrines on premises. As little as 15% of HFs have improved toilets, which are separated for females and allow for menstrual hygiene management, while one in five HFs (21%) have improved toilets which are accessible to those with limited mobility.

Basic hand hygiene services are available in only 20% of HFs. About 1 in 3 HFs (32%) have hand hygiene stations with soap and water at points of care, while one-quarter (26%) have hand hygiene stations with soap and water at toilets. One-third of HFs (33%) safely separate their garbage and waste into at least three labeled bins in consultation areas and safely dispose of garbage/waste. Composite analysis of WASH services in HFs showed that 14% of HFs have combined access to basic water and hygiene services, 5% have access to basic sanitation and hygiene services, and just 4% have access to all three WASH services. Management and sustainability of WASH services in HFs is also a concern, as only 28% of HFs with WASH facilities keep a dedicated budget for cleaning and maintaining WASH facilities. Hospital records showed that the average diarrhea cases per month in the prior 12 months is 43%, while that among under 5 children within the same period is 47%.[25]

The above findings are also supported by the 2020 Surveillance Report of Water Quality, Sanitation and Hygiene (Wash) Services in Healthcare Facilities (HCFs) in South East Zone (Abia, Imo And Anambra States), by the Water Safety Branch in the Department of Food and Drug Service of the FMOH, which revealed the following:

- Almost all the HFs depend on borehole water within the facility except for a few that depend on a nearby private borehole or on tanker water supply for their services.

- No cleaning protocols/standard operating procedure (SOPs) are in place across majority of the HFs.
- Most HFs have no standing committee on WASH and lack of formal training of staff on WASH activities.
- The proportion of HFs that satisfy the basic WASH services standards (according to WHO/UNICEF) in the monitoring exercise was as follows:
- Basic water supply services: 95.5%
- Basic sanitation services: 59.5%
- Basic hygiene services: 85.7%
- Basic health care waste management services: 67.7%
- Basic environmental cleaning services: 29.8%
- Toilet with facilities for menstrual hygiene management: 7.1%
- Toilet accessible for people with limited mobility: 3.6%
- Water samples that passed PH test: 3.5%
- Water samples that passed microbiological test: 51.7%

The report's recommendations included:

- There is a need to develop a national guideline on WASH services in HFs in Nigeria. (This has already been approved and work has started on it.)
- There is a need for the state governments and the management of the health care facilities to establish a standard water treatment process for their water sources to improve the PH, chemical, and microbiological qualities of the water.
- There a is need for training and retraining of staff on WASH activities based on the latest trend in WASH practices.
- Continuous monitoring and surveillance of WASH services is needed in HFs to ensure compliance in the provision of quality WASH in the HFs across the country.
- The data on Water Quality Monitoring should be shared with Federal Ministry of Water Resources for further monitoring by their regional laboratory.[26]

As reported in the Country-Led Midterm Joint External Evaluation of IHR Core Capacities, 2019, there is a need to intensify the implementation of the Turn Nigeria Orange initiative to optimize 30 HFs to become IPC centers of excellence. The initiative should be extended to the states and with the engagement of the Ministry of Water Resources.

The IPC within an HF requires a multidisciplinary trained group with interested stakeholders across the facility that interact with and advise the IPC team. For example, the IPC committee could include senior facility leadership, senior clinical staff, and leads of other relevant complementary areas, such as biosafety, pharmacy, microbiology or clinical laboratory, waste management, water, sanitation and hygiene services, and quality and safety.

4. ANALYSIS OF STRENGTHS, WEAKNESSES, OPPORTUNITIES, AND THREATS

4.1. GOVERNANCE AND INSTITUTIONAL FRAMEWORK

**Governance and Coordination**	
**Strengths**	**Weaknesses**
▪ Adoption of the "One Health" on AMR<br>▪ Institution of a functional AMR-TWG<br>▪ Presence of a functional AMR-CC<br>▪ Establishment of a functional AMS-TWG<br>▪ Establishment of a functional COP<br>▪ Production and launching of AMR NAP<br>▪ Passage of NCDC Establishment Act, 2018	▪ Absence of the AMR steering committee<br>▪ Low involvement of Federal Ministry of Water Resources<br>▪ Inadequate attention on AMR at FMOH<br>▪ Lack regular briefing of FMOH by NCDC<br>▪ Low involvement/participation of states
**Opportunities**	**Threats**
▪ Availability of a large number of public HFs<br>▪ Presence of several relevant agencies for collaboration<br>▪ Availability of supportive partners/donors<br>▪ Availability of a dedicated NCDC workforce and committed leadership<br>▪ Involvement of National Council on Health	▪ Low involvement of Federal Ministry of Water Resources<br>▪ Low level of AMR activities at FMOH<br>▪ Low communication with and utilization of the power of the office of the Honorable Minister of Health
**Policy, Legislative, and Regulatory Framework**	
**Policies**	
**Strengths**	**Weaknesses**
▪ Availability of National Drug Policy, 3rd edition (2020)<br>▪ Presence of other relevant policies such as on supply chain	▪ Poor implementation of policies<br>▪ Noncompliance with the provisions of the policies<br>▪ Unrealistic targets
**Opportunities**	**Threats**
▪ Large vibrant population of Nigeria<br>▪ Large external markets in Africa	▪ Interprofessional rivalry,<br>▪ Absence of a Pharmaceutical Development Plan<br>▪ Low-level development of the petrochemical industry
**Legislations and Regulations**	
**Strengths**	**Weaknesses**
▪ Availability of adequate laws on drugs<br>▪ Availability of strong regulatory bodies	▪ Poor coordination of programs and activities between agencies,<br>▪ Absence of specific regulations on antibiotics to reflect the public health concerns on AMR
**Opportunities**	**Threats**
▪ Collaboration between regulatory bodies<br>▪ Supervision of regulatory bodies by same ministry (FMOH).	▪ Multiplicity of enforcing authorities that sometimes constitute a burden on the regulated
**Essential Medicines List, MTCs, and STGs**	
**Strengths**	**Weaknesses**
▪ Existence of a national EML that is reviewed fairly regularly<br>▪ Existence of a National STGs<br>▪ Existence of law on essential medicines<br>▪ Existence of EML/NDF review committee	▪ No inclusion of AWaRe classification of antibiotics in the current edition<br>▪ Also, STG did not apply AWaRe classification
**Opportunities**	**Threats**
▪ Availability of large number of federal tertiary hospitals<br>▪ Availability of large number of secondary HFs	▪ Absence of MTCs and AMS committees in most tertiary facilities<br>▪ Almost complete absence of MTCs and AMS committees at secondary health care facilities<br>▪ Lack of institutional treatment guidelines in HFs<br>▪ Low staffing levels, especially for pharmacists and medical doctors in secondary HFs

	▪ Depleted numbers of pharmacists in the Department of Food and Drug Services of FMOH
**Pharmacovigilance and Post-Marketing Surveillance**	
**Strengths**	**Weaknesses**
▪ Existence of the NDF<br>▪ Existence of a specific PV policy<br>▪ Existence of the National Drug Advisory Committee<br>▪ Existence of a functional national PV center<br>▪ Existence of six zonal PV centers<br>▪ Existence of a reporting system<br>▪ Reporting to the global platform	▪ Low focus on PV at the state level<br>▪ Poor communication with FMOH
**Opportunities**	**Threats**
▪ Existence of large network of HFs<br>▪ Collaboration with various professional associations<br>▪ Exploration of the NCH platform	▪ High parallel (undocumented) importation<br>▪ Large number of unlicensed medicines stores<br>▪ Involvement of untrained personnel in drug distribution<br>▪ High levels of self-medication
**Facilities Registration and Accreditation of Laboratories and Pharmacies and Other Diagnostic Units**	
**Strengths**	**Weaknesses**
▪ Existence of laboratories in all tertiary HFs<br>▪ Existence of pharmacies in all HFs<br>▪ Existence of radiology diagnostic facilities<br>▪ Availability of pharmacists and medical laboratory scientists	▪ Poorly managed pharmacies in most of the tertiary hospitals, resulting in indebtedness<br>▪ Lack of electronic record keeping
**Opportunities**	**Threats**
▪ Existence of regulatory agencies for registration and accreditation<br>▪ Establishment of the National Tertiary Health Institutions Standard Committee	▪ Lack of standardized laboratories<br>▪ Lack of licensed and standardized pharmacies<br>▪ Incessant disruption electricity supply
**Regulations on Prescribing and Dispensing Antibiotics**	
**Strengths**	**Weaknesses**
▪ Existence of the Poison and Pharmacist Act (Cap 535) 1990 LFN<br>▪ Classification of antibiotics as POM	▪ Inadequacy of current legislation to address emerging public health issues such as AMR/AMS<br>▪ Inadequate human resources<br>▪ Poor communication from NCDC to FMOH for the directive directives
**Opportunities**	**Threats**
▪ Existence of agencies and committees to respond to AMR/AMS requirements<br>▪ Supervision of virtually all agencies by FMOH	▪ Slow response to emerging public health issue of AMR/AMS
**Knowledge and Practice of AMR/AMS in Nigeria**	
**Strengths**	**Weaknesses**
▪ High global and national focus on AMR/AMS<br>▪ Availability of large volume of literature on AMR/AMS	▪ Poor knowledge of AMR/AMS global dimension amongst health care professionals<br>▪ Lack of increased focus on AMR/AMS in the training curriculum of health care professional
**Opportunities**	**Threats**
▪ Availability of appropriate human resources<br>▪ Existence of institutions for the dissemination and entrenchment of AMR/AMS	▪ Poor learning culture<br>▪ Hesitancy to embrace new concepts<br>▪ Inadequate institutional framework for monitoring compliance with policies, regulations, and guidelines
**Medicine Supply Chain**	
**Strengths**	**Weaknesses**
▪ Existence of adequate policies, guidelines, and legislation<br>▪ Existence of relevant regulatory bodies	▪ Inadequate commitment to policy implementation<br>▪ Poor monitoring of policy implementation
**Opportunities**	**Threats**
▪ Existence of National Economic Council for intervention in the implementation of the NDDG (2012)<br>▪ Existence of NCH	▪ Disregard for policies, regulations, guidelines, and legislation<br>▪ Continue existence of unlicensed open market operators

Health Care System	
**Strengths**	**Weaknesses**
▪ Well-spread health care facilities<br>▪ Adequate policies, legislation, and regulation<br>▪ Large number of health care personnel training institute	▪ Poor budgetary funding (4.16% in 2020)<br>▪ Poor management<br>▪ Multiplicity of management bodies, especially at the state level<br>▪ Lopsided availability of experts in THs
**Opportunities**	**Threats**
▪ Availability of large pool of trained, but unemployed health care professional	▪ Unregulated traditional medicine practice<br>▪ Incessant industrial strikes<br>▪ Interprofessional rivalry<br>▪ Brain drain
**Infection Prevention and Control**	
**Strengths**	**Weaknesses**
▪ Availability of adequate global resources (literature)<br>▪ Availability of human resources<br>▪ Existence guidelines	▪ Poor collaboration and integration of national efforts<br>▪ Low focus on IPC by FMOH
**Opportunities**	**Threats**
▪ Large number of HFs<br>▪ Existence of global support	▪ Poor water public supply across the nation<br>▪ Poor state of governance, especially at the Local Government level<br>▪ Lack of water supply in some HFs<br>▪ Lack of treated water supply in most HFs<br>▪ Open defecation (OD) (only 61 out of 744 LGAs are OD free)

5. RECOMMENDATIONS

A quick review of the above situation and the associated SWOT analysis will reveal that issues to be addressed in the development and implementation of an efficient and effective AMS in Nigeria can be immediate, medium term, and long term. Alternatively, such efforts can also be classified as immediate/administrative and developmental.

The immediate and administrative as well as the short-term issues are those the FMOH, implementing agencies/institutions, and supporting partners can take measures or actions to address, and which can bring about significant improvement in AMS and containment of AMR in Nigeria.

On the other hand, the medium- and long-term, or developmental, issues will require the intervention or involvement of other ministries and approval of higher authorities. For these reasons, the following efforts are recommended:

5.1. IMMEDIATE AND ADMINISTRATIVE EFFORTS

- Governance and Institutional Framework
    - The AMR Steering Committee should be constituted, with the inclusion of the Federal Ministry of Water Resources.
    - It is necessary to improve communication and ensure regular briefing of the steering committee members on the progress in and obstacles to the implementation of the AMR and AMS National Action Plans.
    - The AMR/AMS desk at the FMOH should be strengthened.
    - Existing collaboration between implementing agencies should be sustained and strengthened.
    - Effective coordination of partners/donors' interventions should be improved and supported.
- Policy, Legislative, and Regulatory Framework
    - An NDP implementation and monitoring desk should be created in the Department of Food and Drug Services of the FMOH.
    - The EML should be revised to reflect AWaRe classification of antibiotics.
    - The Office of the Honourable Minister of Health should issue directives for the establishment of MTCs, PV, IPC, and AMS Committees.
    - Specific regulations on antibiotics need to be developed to integrate and reflect AMS.
    - Health facility pharmacies and laboratories must be legally registered and licensed.
    - Review, resuscitation, and harmonization of Revolving Fund Scheme in Health Institution.
- Knowledge and Practice of AMS
    - Advocacy and engagement of stakeholders should be increased.
    - Health care professionals and administrators should be thoroughly trained in AMR and AMS.
- Infection Prevention and Control
    - The IPC Desk in the FMOH should be established or strengthened.

- Coordination IPC programs and activities in the country should be strengthened.
- Water treatment plants should be installed in health facilities, especially in the laboratory.

Medium- and Long-Term Efforts

- Policy implementation by the FMOH needs to be improved.
- The involvement of states and secondary health facilities in AMR/AMS should be strengthened.
- Budgetary provision for AMR/AMS activities needs to be made across the board.
- The modules/content on AMR/AMS in the training curricula of health care professionals should be increased.
- Pharmacy human resources capacity at FMOH should be improved.
- A prescription policy should be developed.
- The National Drug Distribution Guideline (NDDG) 2012 should be implemented.
- Relevant regulatory agencies need to be strengthened.
- The provision of water supply to health facilities needs to be ensured.

REFERENCES

1. Barış E. Antimicrobial resistance: another long-term consequence of COVID-19. World Bank blog. April 29, 2021. https://blogs.worldbank.org/team/enis-baris
2. WHO. Global Action Plan on Antimicrobial Resistance 2015. Geneva: WHO; 2015.
3. British Society for Antimicrobial Chemotherapy (BSAC). Antimicrobial stewardship: from principles to practice. Birmingham, UK: BSAC; 2018.
4. Nigeria Centre for Disease Control. National Action Plan for Antimicrobial Resistance, 2017–2022. Abuja: NCDC; 2017.
5. WHO. Antimicrobial Stewardship Programmes in Health Care Facilities in Low- and Middle-Income Countries: A WHO Practical Toolkit. Geneva: WHO; 2019.
6. National Population Commission (website). September 13, 2021. https://nationalpopulation.gov.ng.
7. Federal Ministry of Health. Nigeria Health Registry (NFR) 2020. Abuja: FMOH; 2020.
8. Pharmacist Council of Nigeria. Licensing data. Abuja: PCN; 2021.
9. Federal Ministry of Health. National Health Accounts 2017. Abuja: FMOH; 2017.
10. Abubakar U. Antibiotic use among hospitalized patients in northern Nigeria: a multicenter point-prevalence survey. BMC Infect Dis. 20: article number 86; 2020.
11. Abimbola IO. Knowledge and practices in the use of antibiotics among a group of Nigerian university students. Int J Infect Contr. ISSN 1996-9783. www.ijic.info doi: 10.3396/ijic.v9i1.007.13.
12. Salihu Dadari HI. Antibiotics use, knowledge and practices on antibiotic resistance among breastfeeding mothers in Kaduna state (Nigeria). J Infect Pub Health. 3(12): 2072-79; 2020.
13. Antimicrobial Resistance Centre at the London School of Hygiene & Tropical Medicine. Antibiotic prescribing and resistance: Views from low-and middle-income prescribing and dispensing professionals. Report to the World Health Organization. London: LSHTM; 2017.
14. Opadeyi AO. Drug utilisation pattern in South-South Nigeria using the WHO core drug prescribing indicators. Africa J Medicine Med Sci. 49(2); 2020.
15. Sodipo OO, Adedokun A, Adejumo O. Drug prescription pattern in a Nigerian Teaching Hospital. Nigeria J Fam Pract. 8(4); 2017.
16. Chijoke-Nwauche IN, Chukwumezie CA, Udezi TW. Prescribing indicators: a review in the general outpatient clinic of a Nigerian tertiary hospital. J Health Sci Res. 2; 2018.
17. Abimbola IO. Knowledge and practices in the use of antibiotics among a group of Nigerian university students. Int J Infect Contr. ISSN 1996-9783. www.ijic.info doi: 10.3396/ijic.v9i1.007.13.
18. Iregbu KC, Nwajiobi-Princewill PI, Medugu N, et al. Antimicrobial stewardship implementation in Nigerian hospitals: gaps and challenges African J Clin Exp Microbiol. 2021.
19. Okechukwu R, Eli-Ilo J, Onyejiaka C, et al. Survey of perception, attitudes and practices of antimicrobial stewardship among Health workers in a Nigerian university teaching hospital: Nnamdi Azikiwe University Teaching Hospital, Nnewi, Nigeria. BMJ. 10.1136/bmjebm-2019-EBMLive
20. WHO. Global antimicrobial resistance and use surveillance system (GLASS) Report 2021. Geneva: WHO; 2021.
21. FMOH. National Drug Policy. 3rd ed. Abuja: FMOH; 2021.
22. Pharmacists Council of Nigeria. Compilation of pharmacy, drug and related laws and rules in Nigeria. Abuja: PCN; nd.
23. National Agency for Food and Drug Administration and Control (NAFDAC). Lists of registered locally manufactured and imported drugs (2016–2018). Abuja: NAFDAC; 2019.

24. FMOH. National product chain management programme. Abuja: FMOH; nd.
25. Federal Ministry of Water Resources. Water, sanitation, hygiene national outcome routine mapping Nigeria: a report of findings 2019. Abuja: FMOWR; 2019.
26. FMOH. Report of the monitoring and surveillance of water quality, sanitation and hygiene (WASH) services in healthcare facilities in South East zone (Abia, Imo and Anambra States). Abuja: FMOH; 2020.
