# Supplementary material for: Landscape Analysis of Existing Policies, Laws and Regulations on Antimicrobial Stewardship in 10 Low- and Middle-Income Countries
Source: Antibiotics (Basel). 2026 Aug 10;15(8):770. doi: 10.3390/antibiotics15080770 (PMC13510074; doi:10.3390/antibiotics15080770)
Supplement: Supplementary file 1 [file antibiotics-15-00770-s001.zip › S3 Country Reports/S3G-2_Nigeria (Animal Health).pdf]

USAID MEDICINES, TECHNOLOGIES, AND PHARMACEUTICAL SERVICES (MTaPS) PROGRAM

*Improved Access. Improved Services. Better Health Outcomes.*

# RAPID SITUATION ANALYSIS OF ANTIMICROBIAL USE AND STEWARDSHIP IN THE ANIMAL SECTOR

**Nigeria**

**December 2021**

USAID FROM THE AMERICAN PEOPLE

*This document is made possible by the generous support of the American people through the US Agency for International Development (USAID) contract no. 7200AA18C00074. The contents are the responsibility of Management Sciences for Health and do not necessarily reflect the views of USAID or the United States Government.*

### About the USAID MTaPS Program

The USAID Medicines, Technologies, and Pharmaceutical Services (MTaPS) Program enables low- and middle-income countries to strengthen their pharmaceutical systems, which is pivotal to higher-performing health systems. MTaPS focuses on improving access to essential medical products and related services and on the appropriate use of medicines to ensure better health outcomes for all populations. The program brings expertise honed over decades of seminal pharmaceutical systems experience across more than 40 countries. The MTaPS approach builds sustainable gains in countries by including all actors in health care—government, civil society, the private sector, and academia. The program is implemented by a consortium of global and local partners and led by Management Sciences for Health (MSH), a global health nonprofit.

### The MTaPS Consortium

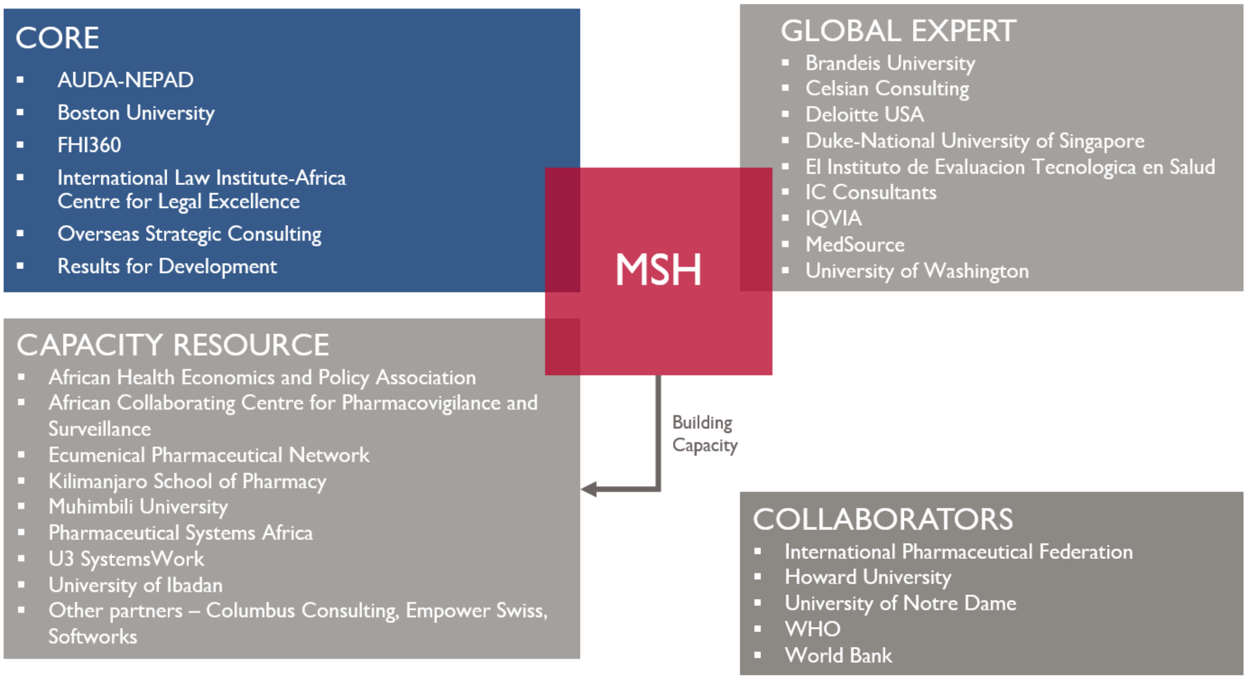


### This report/document is submitted by:

Dr. Sati Ngulukun
AMS Consultant-Animal Health
USAID/MTaPS Nigeria
Abuja
Email: sngulukun@gmail.com

# ACRONYMS AND ABBREVIATIONS

| | |
|---|---|
| AMR | antimicrobial resistance |
| AMRCC | Antimicrobial Resistance Coordinating Committee |
| AMS | antimicrobial surveillance |
| AMU | antimicrobial use |
| AST | antimicrobial susceptibility testing |
| DVPCS | Department of Veterinary and Pest Control Services |
| FAO | Food and Agricultural Organization of the United Nations |
| FMARD | Federal Ministry of Agriculture and Rural Development |
| GAP | Global Action Plan |
| LFN | Laws of the Federation of Nigeria |
| MSH | Management Sciences for Health |
| MTaPS | Medicines, Technologies, and Pharmaceutical Services |
| NAFDAC | National Agency for Food and Drug Administration and Control |
| NAMR-TWG | National Antimicrobial Technical Working Group |
| NCDC | Nigeria Centre for Disease Control |
| NOHSC | National One Health Steering Committee |
| NVRI | National Veterinary Research Institute |
| OIE | World Organisation for Animal Health |
| STG | standard treatment guideline |
| SWOT | strengths, weakness, opportunities, and threats |
| TWG | technical working group |
| USAID | US Agency for International Development |
| VCN | Veterinary Council of Nigeria |
| VMAP | Veterinary Medicines and Allied Products |
| WHA | World Health Assembly |
| WHO | World Health Organization |

# DEFINITIONS

**Antimicrobial:** An antimicrobial agent is a chemical substance, a drug, used for the treatment of a disease caused by a microorganism, which kills the microorganisms or inhibits their growth.

**Antimicrobial resistance:** Antimicrobial resistance, as defined by the World Health Organization, is the resistance of a microorganism to an antimicrobial drug to which it was previously susceptible. It results from the ability of certain microorganisms (bacteria, viruses, parasites, fungi) to withstand the attack of antimicrobial drugs such as antibiotics, antivirals, and antiparasitics.

**Antimicrobial stewardship:** Antimicrobial stewardship consists of a series of interventions aimed at optimizing antimicrobial prescribing (selection, dosage, route of administration, and duration of antimicrobial therapy), delivery, and use to reduce antimicrobial resistance.

**Rational use:** Rational use is the set of individual and collective means, principles, and practices related to medicines that aim to preserve or promote health.

# CONTENTS


Acronyms and Abbreviations ..... i

Definitions ..... ii

Project Summary ..... iv

1. Introduction ..... 1

2. Rapid Analysis ..... 3
   - 2.1. Institutional Framework and Governance Structure ..... 3
   - 2.2. Animal Health Services ..... 4
     - 2.2.1 Workforce in the Animal Health Sector ..... 4
     - 2.2.2 Veterinary Services System ..... 4
     - 2.2.3 Veterinary Laboratory Services ..... 5
     - 2.2.4 Access to Health Care in the Terrestrial and Aquatic Animal Sector ..... 5
   - 2.3. Bacterial Resistance in Animals ..... 5
   - 2.4. Policies and Legislation on Antimicrobial Use in the Animal Health Sector ..... 6
     - 2.4.1 Laws/Regulations Governing Medicine Control/Use in Animals in Nigeria ..... 7
   - 2.5. Antimicrobial Management And Supply Chain In The Animal Health Sector ..... 8
     - 2.5.1 Policy and Guidelines for the Good Management of Antimicrobial Supply ..... 9
     - 2.5.2 Marketing Authorization for Antimicrobials and Quality Control ..... 9
     - 2.5.3 Licensing Exercise and Inspection Activities ..... 9
     - 2.5.4 Prescribing, Dispensing, and Using Antimicrobials ..... 9
     - 2.5.5 Postmarketing Surveillance and Pharmacovigilance ..... 10
   - 2.6. Antimicrobial Use Data in Animals In Nigeria ..... 10
   - 2.7. Antimicrobial Use and the Drivers in Animal Health Sector ..... 11

3. SWOT Analysis of the Rapid Assessment ..... 12
   - 3.1. Strengths ..... 12
   - 3.2. Weaknesses ..... 12
   - 3.3. Opportunities ..... 13
   - 3.4. Threats ..... 13

4. Key Priority Gaps Identified ..... 14

5. Recommendations ..... 14

References ..... 16

# PROJECT SUMMARY

| | | |
|---|---|---|
| **Program Name:** | | USAID Medicines, Technologies, and Pharmaceutical Services (MTaPS) Program |
| **Activity Start Date And End Date:** | | September 20, 2018–September 19, 2023 |
| **Name of Prime Implementing Partner:** | | Management Sciences for Health |
| **Contract Number:** | | 7200AA18C00074 |
| **MTaPS Partners** | **Core Partners** | Boston University, FHI 360, Overseas Strategic Consulting, Results for Development, International Law Institute-Africa Centre for Legal Excellence, NEPAD |
| | **Global Expert Partners** | Brandeis University, Deloitte USA, Duke-National University of Singapore, El Instituto de Evaluacion Technologica en Salud, IC Consultants, Imperial Health Sciences, MedSource, QuintilesIMS, University of Washington |
| | **Capacity Resource Partners** | African Health Economics and Policy Association, Ecumenical Pharmaceutical Network, U3 SystemsWork, University of Ibadan, University of Ghana's World Health Organizations (WHO) Pharmacovigilance Collaborating Center, Kilimanjaro School of Pharmacy, Muhimbili University, Pharmaceutical Systems Africa |
| | **Collaborators** | International Pharmaceutical Federation, Howard University, University of Notre Dame, WHO, World Bank |

# 1. INTRODUCTION

Antimicrobials are drugs widely used around the world and which play an essential role in the fight against infectious diseases in human, animal health, agriculture, and the environment. However, their inappropriate use can lead to the emergence of resistance. According to the World Health Organization (WHO), antimicrobials are among the most misused of all drugs, because of their wide availability, familiarity, generally low cost, and relative safety. WHO advocates for strengthening appropriate use, focuses on monitoring, and reducing their inappropriate use in humans and animals (WHO 2017).

Antimicrobial resistance (AMR) is a global threat that causes 700,000 deaths annually. It is estimated that AMR will account for 10 million deaths and annual economic losses of USD 100 by 2050 if no urgent actions are taken (O'Neil 2016). Tackling AMR requires multisectoral and cross-disciplinary efforts, and thus demands urgent and concerted actions as embedded in the "One Health Approach" (OIE 2016). The One Health Approach embraces efforts to redress the inappropriate use of antimicrobials in humans, animals, and the environment.

Antimicrobials are widely used in animals to treat infections, prevent diseases, and promote growth (Kimera et al, 2020). Although the use of antimicrobials is imperative in treating animals, most antimicrobial use (AMU) in animals is actually directed to prevent infections and promote growth. In the United States, around 70% of antimicrobials that are used to treat human infections are also sold for use in food animals (USDA 2017). Data from low- and middle-income countries are limited, but empirical estimates suggest the rampant use of antimicrobials in food animals, a serious concern (O'Neil 2016).

Over the years, evidence has suggested that use of antimicrobials in animals has contributed to the development of AMR in humans. In addition, the use of antimicrobials in low (subtherapeutic) doses in food animals as growth promoters creates a perfect environment for the emergence of AMR. Prudent use principles recognize that antimicrobials are essential for animal health and welfare *and* must be used in a responsible, appropriate, and targeted manner to prevent overuse and misuse of antimicrobials in agriculture, and reduce the development of resistance, particularly against medically important antimicrobials.

In May 2015, the World Health Assembly (WHA) adopted resolution WHA68.7 and endorsed the Global Action Plan (GAP) on AMR. The WHO GAP aims to optimize the use of antimicrobials in animal health, urging its member countries to develop their own national action plans to tackle AMR. In the same year, the 83rd General Session of the World Organization for Animal Health (OIE) World Assembly of Delegates adopted a resolution, "Combating Antimicrobial Resistance and Promoting the Prudent Use of Antimicrobial Agents in Animals," in support of the GAP. WHO recommends reducing and restricting the use of medically important antimicrobials in food animals for disease prevention and growth promotion (WHO 2017). The UN's Food and Agriculture Organization of the (FAO) Action Plan on AMR focuses on surveillance and monitoring, strengthening governance, and promoting good practices of optimal use of antimicrobials in food and agriculture (FAO 2016). OIE shares a similar commitment and collaborates with the WHO and the FAO to address these challenges (OIE 2016).

In many countries, antimicrobial agents remain widely available, with virtually no restrictions or controls on their use. Of the 136 OIE members assessed through an initial OIE Performance of Veterinary

Services (PVS) Evaluation covering up to December 2019, almost three-quarters could not regulate veterinary medicinal products (VMPs) (assessed as "Level 1"), or had only limited capability to exercise regulatory and administrative control over the import, manufacture, and market authorization (registration) of these products to ensure their safety and quality. The countries were unable to ensure VMPs' responsible and prudent use in the field ("Level 2"). The absence or low levels of control of VMPs overall leads to the limited control of veterinary products that contain antimicrobial agents. These antimicrobial agents potentially circulate freely in the market and, like ordinary goods, they may be falsified or substandard, and/or they may be provided without clinical or laboratory diagnosis. This variable quality and unrestricted use of antimicrobial products creates conditions of high risk for the development and spread of resistance.

In most sub-Saharan African countries, Nigeria inclusive, surveillance programs for antibiotic use and AMR in humans and animals are either lacking or low. Furthermore, in Nigeria, the lack of regulation of existing veterinary drug markets and low involvement of pharmacists and veterinarians in the formal and informal drug distribution market may contribute to the issue of substandard drugs in the marketplace.

Responsible antibiotic use is essential to prevent emergence of antibiotic-resistant bacteria, protect patients from adverse drug effects, and avoid antibiotic residue in **animal** products. The irrational use of antimicrobial agents has been well identified in animals, wherein noncompliance to the withdrawal-period concept causes drug resistance to develop. Development of drug resistance to antimicrobials used in humans is increasing due to irrational use of antimicrobials in animals through food of animal origin such as meat, milk, and eggs. To address this problem, there is a need for developing antimicrobial stewardship (AMS) programs. AMS includes not only limiting inappropriate use but also optimizing antimicrobial selection, dosing, route, and duration of treatment to maximize clinical cure or prevention of infection.

It is essential to strengthen stewardship for AMU in the animal health sector. The stewardship will also encompass monitoring and evaluating the use and consumption of antibiotic at all levels. In addition, priorities to support optimizing AMU include the improvement of the regulatory framework for preservation of antimicrobial agents and revisiting the policies, regulations, guidelines, and directives on the use of antimicrobials in the country. Other interventions will include strengthening the regulatory system in controlling quality, distribution, and use of antimicrobial agents, including inappropriate or unregulated use of antimicrobial agents in the animal health sector.

# 2. RAPID ANALYSIS

## 2.1. INSTITUTIONAL FRAMEWORK AND GOVERNANCE STRUCTURE

The Nigeria National AMR Coordinating Body was established in 2016 at the Nigerian Centre for Disease Control (NCDC). Subsequently, a National AMR technical working group was formed, comprising stakeholders from human health, animal health, food animal production, and environmental sectors. A situation analysis of AMR was conducted by this working group. The result of the situation analysis was the development of the National Action Plan (NAP) for Antimicrobial Resistance (2017–2022).

Nigeria's AMR governance structure is based on the One Health Approach and consists of the National One Health Steering Committee (NOHSC), National One Health Technical Committee (NOHTC), Antimicrobial Resistance Coordinating Committee (AMRCC), and National AMR Technical Working Group (NAMR-TWG), together focused on five strategic areas (Awareness and Education, Surveillance, Stewardship, Infection Prevention, and Control/Biosecurity and Research) (figure 1). The NAMR-TWG oversees policy in the human, animal, and environment sectors (figure 2). The coordinating center is at the NCDC. The NOHSC is the highest-level policy and governance body responsible for all activities related to One Health and AMR in Nigeria. The purpose of the AMRCC is to guide, oversee, and monitor AMR-related activities in all sectors to ensure a systematic and comprehensive implementation of AMS.

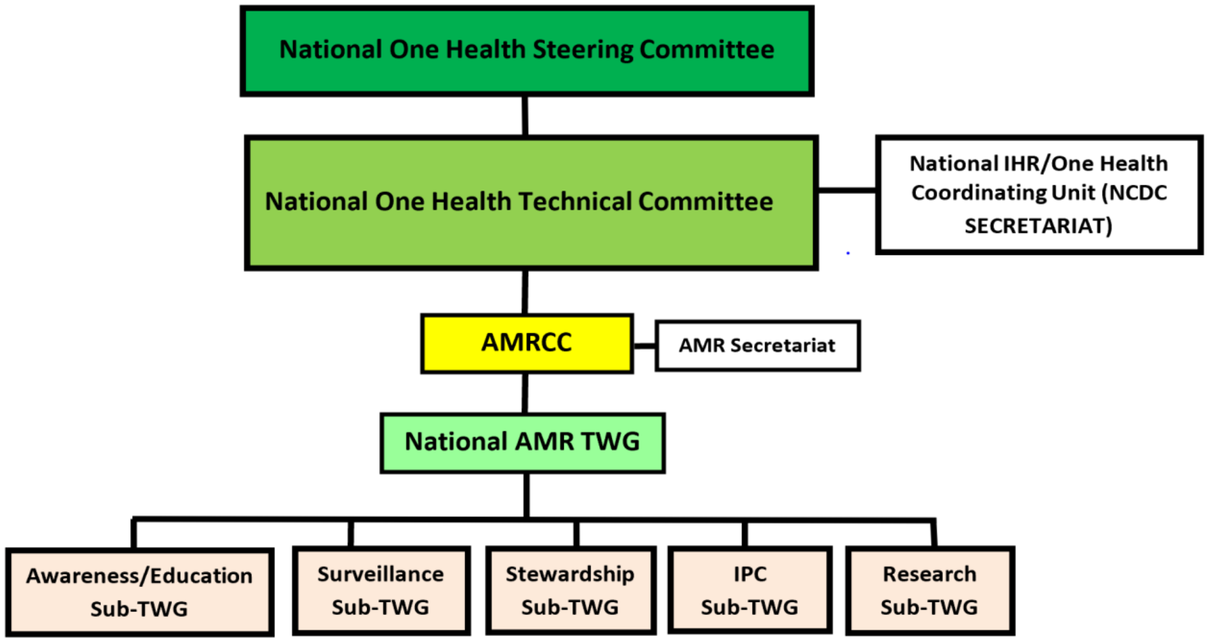


**Figure 1. Nigeria's One Health/AMR governance structure**

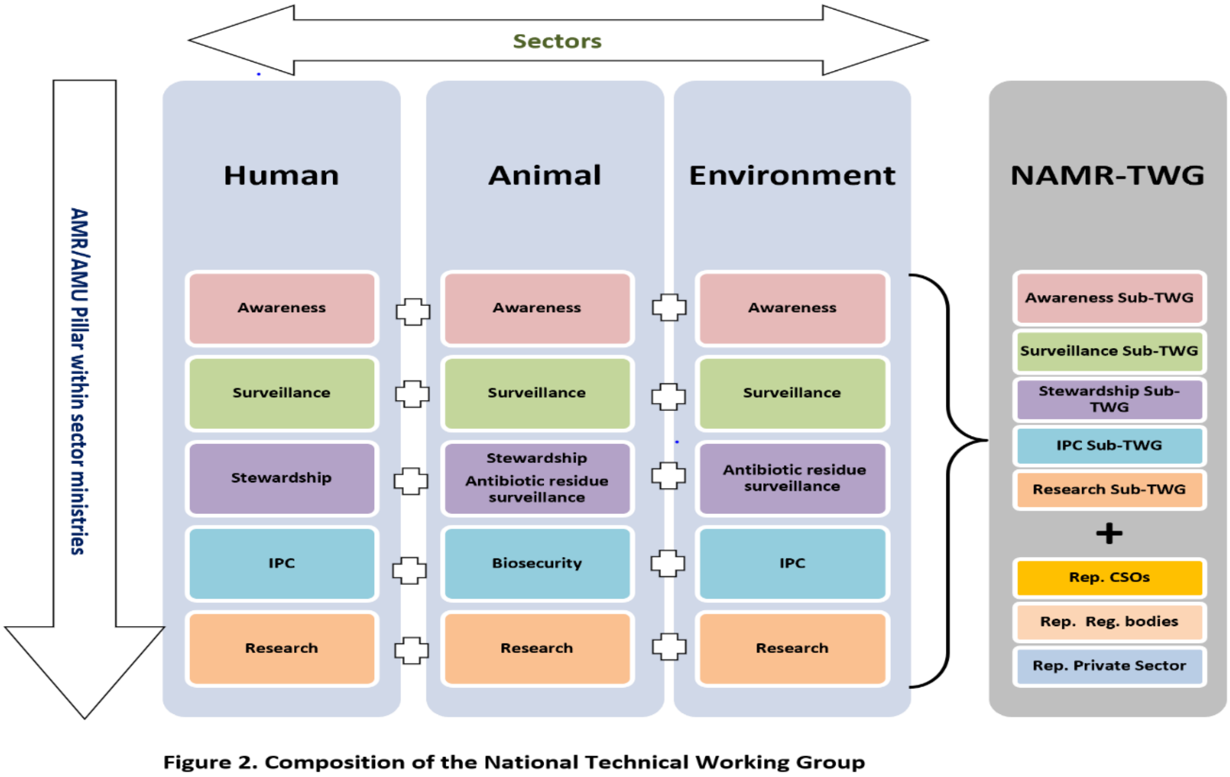


**Figure 2. Composition of the National Technical Working Group**

## 2.2. ANIMAL HEALTH SERVICES

### *2.2.1 Workforce in the Animal Health Sector*

Nigeria has eleven (11) universities with veterinary faculties across the country that are accredited for the training of veterinarians. The Veterinary Council of Nigeria (VCN) has also established the College of Veterinary Surgeons, which offers membership and fellowship diplomas. The VCN also conducts continuing education programs. Three colleges of animal health and production located in Ibadan, Kaduna, and Vom, respectively, also provide training for veterinary paraprofessionals. In addition, there are faculties and colleges of agriculture as well as schools of veterinary medical laboratory for training of terrestrial and aquatic animal practitioners. About 8,500 veterinarians are registered with the VCN. About 2,000 of these are employed in the public sector, with the remaining working in the private sector. Veterinary laboratory practitioners are employed mainly at the National Veterinary Research Institute (NVRI) in Vom; at laboratories within the veterinary faculties; and at state and private veterinary laboratories. Other categories within the workforce are agricultural scientists and support staff within other agricultural sub-sectors.

### *2.2.2 Veterinary Services System*

The Department of Veterinary and Pest Control Services (DVPCS) of the Federal Ministry of Agriculture and Rural Development (FMARD) functions as the policy adviser to the national government on all animal health, safety, and wholesomeness of food of animal origin for human consumption, as well as pest control services. DVPCS draws its mandate from the Animal Diseases Control Act No. 10 of

1988 and the OIE standards, recommendations, and guidelines for animal health and welfare. The DVPCS has offices in each of the 36 states and the Federal Capital Territory (FCT). The department is involved in the prevention, control, and eradication of transboundary animal diseases and pests, control of vectors and vector-borne diseases, zoo-sanitary certification services, provision of veterinary public health services, food safety services, and zoonotic diseases. The main goal of the DVPCS is to guarantee a healthy national herd, wholesomeness of foods of animal origin, the country's participation in international trade in livestock and livestock products, and the general economic well-being of the populace. It also coordinates the national animal disease control programs. In carrying out its mandate, the DVPCS works with other departments, agencies, and the private sector in provision of animal health services at the national, state, and local government levels.

The National Veterinary Research Institute is responsible for the diagnosis of and research into all animal diseases and zoonoses; the Veterinary Services departments at the federal and state levels are responsible for implementation of disease control programs; the Veterinary Public Health Division is responsible for food safety and zoonotic disease control. Veterinary services are provided by both government and private animal health facilities. However, the majority of these are situated in urban areas, with very limited provision of services to rural areas.

### *2.2.3 Veterinary Laboratory Services*

Laboratory diagnostics for terrestrial and aquatic animal diseases in Nigeria are carried out by the NVRI, university veterinary teaching hospitals, state veterinary laboratories, and some private laboratories, such as Animal Care Consult, Olam, and Zoetis. The NVRI is the designated national reference laboratory for animal health and has 6 zonal and 17 other diagnostic laboratories located in 23 states. The Central Diagnostic Laboratory at Vom coordinates the activities of all these laboratories. It also carries out referral diagnosis for states and for private veterinarians. The NVRI is also the AMR national reference laboratory for animal health.

### *2.2.4 Access to Health Care in the Terrestrial and Aquatic Animal Sector*

In Nigeria, veterinary services are carried out in both the public and private sectors. Public sector veterinarians are located at the federal/state levels, universities, research institutes, and colleges of agriculture. Private sector veterinarians are in various service areas, including aquatic and terrestrial animals, pharmaceutical, laboratory and consultancy services provision, and animal production and input supplies. There is an interface between the public and private, especially in the areas of surveillance, disease diagnosis, and vaccination. The sanitary mandate ensures veterinarians provide veterinary services, especially in the country's rural areas. However, because of the low number of veterinarians and a large animal population in the country, access to veterinary care in the rural areas remains low. Veterinary health care services are available at the primary, secondary, and tertiary levels, in addition to the private facilities.

## 2.3. BACTERIAL RESISTANCE IN ANIMALS

A review of the Nigerian literature revealed that resistant bacteria are commonly recovered from livestock (cattle, sheep, goats, camels, pigs), poultry, and other animals. High levels of resistant organisms have also been isolated from foods such as meats, eggs, dairy, and vegetables. With the aid of

diverse phenotypic or genotypic methods, studies have been carried out on a wide range of isolates using different classes of antimicrobials. In a study by Igbinosa et al. (2016), 67% of vibrio from aquaculture samples were multidrug-resistant; this is attributable to AMU in aquaculture. In almost all instances, resistant organisms were recovered from livestock, poultry, aquaculture, and from foods (dairy, vegetables, and meats). While it is documented that resistant organisms in animals could have been acquired from human and other sources, the high levels of antimicrobial residues in Nigerian meats and eggs and the low recovery of resistant organisms from wildlife point to antimicrobial use in agricultural and veterinary practices as the principal driver of resistance (Kabir et al. 2004). Drugs are the most frequently detected chemical residues in food of animal origin, and the majority of these are antimicrobials commonly used in veterinary practice in Nigeria (Aliu 2004).

## 2.4. POLICIES AND LEGISLATION ON ANTIMICROBIAL USE IN THE ANIMAL HEALTH SECTOR

In Nigeria, the regulation of all veterinary and human dugs (administration and control) is under the National Agency for Food and drug Administration and Control (NAFDAC). Veterinary administration and control are under the department of Veterinary Medicine and Allied Products (VMAP) within NAFDAC. The Federal Ministry of Health provides policy direction for the human health sector, whereas the FMARD gives policy direction for the animal health sector. NAFDAC was established by Decree No.15 of 1993 (now Act Cap N1 Laws of the Federal Republic of Nigeria 2004) with the mandate to register, regulate, and control the use, sale, importation, exportation, manufacture, and distribution of food, drugs, cosmetics, medical devices, packaged water, and chemicals. The NAFDAC Act provides legal backing for the agency to conduct its regulatory functions, including the registration/licensing of all regulated products prior to marketing of such products in Nigeria; inspection and investigation of relevant manufacturing facilities within and outside the country to ascertain their Good Manufacturing Practice (GMP) status toward certification of the sites and their products; quality control of products meant for import and export; regulation of products; labeling; promotion and public education; and post-marketing surveillance of registered products to ensure maintenance of quality and safety, and compilation of standard specifications.

The main policy, legal, and institutional issues involved in control of veterinary drugs, in particular, involve regulating the manufacture and import of veterinary drugs, their distribution around the country, and their provision to the buying public. The goals of veterinary drugs control are to be met within a wider framework of international obligations, and a national framework of a country's domestic laws on animal health, animal production, and human health. With respect to the drugs themselves, it is widely recognized that the goal of regulating the supply of veterinary drugs is to guarantee their quality, safety, and efficacy at the time of their administration to the animal.

Nigeria currently does not have specific legislation addressing AMU and AMR, but has acts of parliament, policies, and regulations pertaining to drugs, especially in the human health sector. Nigeria is signatory to various international agreements and standards that have a bearing on AMU. The OIE (now called the World Organisation for Animal Health) is an intergovernmental organization that collects and distributes information on animal diseases to its 182 member states (Nigeria inclusive), and assists them with their disease-control activities (OIE 2016). It also sets the internationally recognized standards for animal health and trade.

In undertaking its role as a standard-setting organization for animal health, including zoonoses, the OIE has developed a wide range of international standards on antimicrobial agents, in particular on responsible and prudent use. These standards are regularly reviewed and updated through the transparent and inclusive process of expert advice and member consultation before presentation for adoption to the World Assembly of Delegates from the 182 member countries each year (Nigeria is a member). The Codex Alimentarius Commission (Codex), a subsidiary organ of FAO and WHO, develops the internationally recognized standards for food safety, including those pertaining to veterinary drug and pesticide residues in food.

#### *2.4.1 Laws/Regulations Governing Medicine Control/Use in Animals in Nigeria*

Ten major laws and regulations are relevant to medicine use in animals:

1. The National Agency for Food and Drugs Administration and Control Law: Decree 15 of 1993, established the National Agency for Food and Drugs Administration and Control (NAFDAC), as amended by Decree 20 of 1999. Under this law, NAFDAC is empowered to enact regulations and guidelines that govern the importation, licensing, labeling, and usage of drugs (including antimicrobials for humans and animals) by sections 5 and 30 of the National Agency for Food and Drug Administration and Control Act Cap NI 35, Laws of the Federation of Nigeria (LFN) 2004. Within the agency, a Directorate of Veterinary and Allied Products has been created to cater for veterinary medicinal products.
2. Animal Disease Control Act 1988 (as amended). This act provides regulation for the control and prevention of animal diseases, with the objective of preventing the introduction and spread of infectious and contagious diseases among animals, hatcheries, and poultries in Nigeria. It is currently being reviewed to specifically address emerging issues and areas that are obsolete.
3. The Veterinary Surgeons Act Cap V3 LFN, 2004, as amended. This law established the Veterinary Council of Nigeria to regulate the standard of training and practice of the veterinary profession in the country. It also set standards for registration of veterinarians and to regulate their conduct. It issues the annual practicing license to registered veterinarians as well as registers and licenses (annually) veterinary practicing premises. This currently does not cover registration of veterinary paraprofessionals; however, this coverage is being pursued in a proposed amendment, in line with World Organisation for Animal Health (OIE) requirements.
4. The Veterinary Practicing Premises Registration Regulations, (2018) published in the official gazette in January 2019.
5. Counterfeit and Fake Drugs (miscellaneous provisions) Act, Cap 73, of 1990: This act prohibits the production, importation, manufacture, sale and distribution of any counterfeit, adulterated, banned, or fake drugs. It also prohibits the unauthorized sale of drugs in an open market.
6. Meat Law (1968). Under this law, all slaughterhouses must be registered with the Director of Veterinary Services of each state. Slaughtering except in a registered abattoir is illegal. Animals to be slaughtered are supposed to be rested in the lairage at least 24 hours before slaughtering, during which antemortem inspection, such as disease screening and residue screening, is done. Postmortem inspection is compulsory.
7. NAFDAC drug and related products registration Regulations 2019. This regulation deals with the registration of drugs (human and veterinary) and related products manufactured, imported, exported, advertised, sold, distributed, or used in Nigeria.

8. NAFDAC drug and related product labeling Regulations 2019. These regulations apply to all labeling of drugs (human and veterinary) and related products manufactured, imported, exported, sold, distributed, or used in Nigeria.
9. Nigerian Veterinary Formulary. This instrument was first published in 2007 and is the compendium of essential veterinary drugs (including antimicrobials), biologics, and chemical pesticides. The current edition was published in 2018. The veterinary drugs and biologics included are for the protection of the health and welfare of animals.
10. OIE list of antimicrobials of veterinary importance (OIE 2007). In developing the list, OIE member countries (Nigeria inclusive) at the 75th Session by Resolution No. XXVIII agreed that any antimicrobial authorized for use in veterinary medicine according to the criteria of quality, safety, and efficacy as defined in the Terrestrial Animal Health Code (Appendix 3.9.3. Guidelines for the Responsible and Prudent Use of Antimicrobial Agents in Veterinary Medicine) is important.

## 2.5. ANTIMICROBIAL MANAGEMENT AND SUPPLY CHAIN IN THE ANIMAL HEALTH SECTOR

NAFDAC is the main government institution responsible for regulating different aspects of the pharmaceutical supply chain in Nigeria. NAFDAC was established by Decree No.15 of 1993 (now Act Cap N1 Laws of the Federal Republic of Nigeria 2004) with the mandate to regulate and control the use, sale, importation, exportation, manufacture, and distribution of food, drugs, cosmetics, medical devices, packaged water, and chemicals. Veterinary products (antimicrobials and biologics) are mainly imported, with some produced locally. The National Veterinary Research Institute produces animal vaccines (bacterial and viral vaccines) locally. However, the production of NVRI does not meet the total requirement for the livestock sector; this is therefore, supplemented through importation. Most of the veterinary drugs (including antimicrobials) are imported. The federal government does not purchase veterinary drugs for supply, although state veterinary services that run the revolving drug scheme have stores where drugs are bought and kept for supply to their veterinary hospitals and clinics.

Most manufacturers and importers of veterinary drugs have distributors who sell to retailers, who in turn sell to animal health care providers and farmers/producers. The supply and distribution network is not well organized owing to involvement of unqualified persons in the procurement and distribution of veterinary drugs, inadequate storage facilities, and poor transportation. For vaccines, NVRI sell only to registered veterinary outlets and veterinarians. Manufacturers and importers have their own distribution channels for distributing drugs in the private sector; they sell to wholesalers, retailers, hospitals, clinics and farms. This results in medicines being sold in unregistered and unlicensed premises and, in many cases, by non-pharmacists/veterinarians.

While NAFDAC regulates the product-related aspects of the pharmaceutical sector, the VCN, established by the veterinary Surgeons Act Cap V3 LFN, 2004, is responsible for regulating the practice aspects, including registration and training of veterinary surgeons and development of basic veterinary curricula for degree programs and mandatory continuing education programs. The VCN also regulates all premises where veterinary services are rendered. This includes inspection and registration of veterinary practices, including hospitals/clinics and veterinary pharmacies, to ensure compliance with standards. In reality, any end user can purchase from a retail outlet. The implication of this is that nonprofessionals as well as farmers have unfettered access to veterinary drugs (including antimicrobials)

and vaccines, often not following the regulations for use; this invariably leads to abuses that encompass inappropriate use.

### *2.5.1 Policy and Guidelines for the Good Management of Antimicrobial Supply*

VMAP regulates and controls the importation, exportation, manufacture, advertisement, distribution, sale and use of veterinary medicines, other veterinary products, pesticides, and agro-chemicals. The DVPCS has the Quality Assurance and Standards Division and the Public Health Division, which regulate food safety, as well. The Quality Assurance and Standards Division addresses commodity certification, veterinary drugs, and food, ensuring adequate supplies of quality-assured essential antimicrobials at various levels of the human health and animal health delivery systems

### *2.5.2 Marketing Authorization for Antimicrobials and Quality Control*

NAFDAC (VMAP) regulates and controls the importation, exportation, manufacture, advertisement, distribution, sale and use of veterinary medicines, veterinary products, pesticides, and agro-chemicals.

Veterinary medicines and biologicals are sold through veterinary retail outlets (agro-vets) made up of agro-veterinary shops, pharmacies, general shops, and open-market veterinary shops. The agro-veterinary shops stock most classes of drugs, including trypanocidals, anthelmintics, antibiotics, acaricides, agro-chemicals such as fertilizers, vaccines, and surgical materials. Many of the veterinary shop owners and salespersons in rural areas do not have professional qualification although, according to the Veterinary Practicing Premises Registration Regulation (2018), they should have it.

### *2.5.3 Licensing Exercise and Inspection Activities*

NAFDAC Drug and Related Products Registration Regulations 2019 deals with the registration of drugs (human and veterinary) and related products manufactured, imported, exported, advertised, sold, distributed, or used in Nigeria. The VCN also has responsibility for registering veterinary practicing premises. The Veterinary Practicing Premises Registration Regulations (2018) was published in the official gazette in January 2019. In 2018, the VCN registered the following: veterinary medical or veterinary pharmacy, 85; import of veterinary drugs, 20; wholesalers 5; manufacturer of two product lines.

Regulatory agencies should effectively play their roles in controlling standards of practice; they must take more decisive actions regarding over-the-counter sale of antimicrobials for both human health and animal health.

### *2.5.4 Prescribing, Dispensing, and Using Antimicrobials*

In the veterinary practice in Nigeria, the drug is an input for the veterinarian. The veterinarian consults, makes the diagnosis, prescribes the drug, dispenses the drug, and uses the drug. This is a safety principle specific to science and veterinary medicine recognized by the OIE and applied by the Veterinary Authorities of Member States.

In Nigeria, veterinary antimicrobials are prescription-only drugs (Nigerian Veterinary Formulary 2018). However, in practice and research work in Nigeria (Adekanye et al., 2020) revealed that nonprofessionals, including farmers, are involved in prescribing and dispensing veterinary drugs, including

antimicrobials. Regulatory agencies should effectively play their roles in controlling standards of practice; they must take more decisive actions regarding over-the-counter sale of antimicrobials for both human health and animal health.

### *2.5.5 Post-Marketing Surveillance and Pharmacovigilance*

In Nigeria, pharmacovigilance in the animal health sector is almost nonexistent. Likewise, there are no national regulations specific to pharmacovigilance in the animal health sector. However, monitoring for the quality control of veterinary drugs is done by NAFDAC. Therefore, it would be appropriate to establish/update and promote a national list of essential veterinary drugs.

Activities to analyze and control the origin of drugs, obtain reliable data on imports, trace their circulation, and assess the quality of products in circulation are insufficiently carried out.

The online sale of veterinary drugs constitutes a traceability difficulty, as it is difficult to identify the actors involved. There are no regulations on the e-commerce of veterinary drugs in Nigeria.

There should be regulation of veterinary pharmacovigilance aimed at establishing an effective mechanism for monitoring the use of veterinary medicinal products within Nigeria.

## 2.6. ANTIMICROBIAL USE DATA IN ANIMALS IN NIGERIA

One of the missions of the World Organisation for Animal Health (OIE) is to ensure prudent use of antimicrobials in animals through application of the OIE standards set in the Terrestrial Animal Health Code (Chapter 6.9) and the Aquatic Animal Health Code (Chapter 6.2). In pursuance of this, the OIE in 2013 had a global conference themed "Responsible and Prudent Use of Antimicrobial Agents for Animals: International Solidarity to Fight Against Antimicrobial Resistance." Recommendations were made by participants to the OIE and member countries for implementation. One of the recommendations was to establish a global database with the aim of collecting and harmonizing quantitative data on the use of antimicrobials in animals. This was to start from 2013, and includes the baseline data and reporting. Thus, Nigeria through FMARD submitted the baseline data and reported on data for 2014 to 2019. The various classes of antimicrobials used in food animals include tetracyclines, fluoroquinolones, macrolides, sulfonamides, polypeptides, amphenicol, penicillins, aminoglycosides, glycopeptides, pleuromutilins, and nitrofurantoin. During this period, an average of 300,000kg of active ingredients of these antimicrobial agents were imported for use in animal health in Nigeria.

FMARD, therefore, reports data on AMU to the World Organisation for Animal Health (OIE) using the option 1. According to the FMARD report, on average, about 300,000 kg of antimicrobial agents are imported into the country on an annual basis for use within the animal health sector, some of which are in the list of critical antimicrobials under the OIE classification. These quantities are from what has been imported through the authorization agency and do not include other procurement sources. There is a need to know the quantities used in specific species of food animals and the possible route of administration. Activities to analyze and control the origin of drugs, obtain reliable data on imports, trace their circulation, and assess the quality of products in circulation are insufficiently carried out. Improvements in the availability of antibiotic use/consumption data are needed so that progress in

reducing the overall quantities of antibiotics can be demonstrated. Availability of data ensures policy is evidence-based and individual farmers and veterinary practitioners can benchmark their antibiotic use.

This will entail advocacy and training of the stakeholders in the drug use chain (pharmaceutical manufacturers, wholesalers/retailers, veterinarians and veterinary paraprofessionals, and farmers).

The online sale of veterinary drugs constitutes a traceability difficulty insofar as it escapes control/surveillance programs, and it can be extremely difficult to identify the actors involved. There are no regulations on the e-commerce of veterinary drugs in Nigeria.

## 2.7. ANTIMICROBIAL USE AND THE DRIVERS IN ANIMAL HEALTH SECTOR

Literature search in Nigeria shows that antimicrobial agents are widely distributed, readily available, and commonly used in animal production (Ojo et al. 2016). Fluoroquinolones and other critically important antimicrobials for human medicine are widely used in animals as prophylaxis. In a study in northcentral Nigeria (Alhaji and Isola 2018), antimicrobials frequently used by pastoralists in their animals are tetracyclines (96.6%), tylosin (95.6%), penicillins (94%), streptomyins (93%), sulfonamides (92.4%), gentamycin (75%), neomycin (67.2%), and ciprofloxacin (39.6%). They said they used some of the drugs for treatment of infections and for growth promotion.

In a study carried out to assess antibiotic usage in commercial poultry farms in Ogun state, Nigeria, antibiotics used among farmers included a wide range of different classes (Adebowale et al. 2016). Gentamycin (76.7%) was the most frequently used antibiotic, followed by tetracycline (64.1%), enrofloxacin and ciprofloxacin (57.3%), furazolidone (49.5%), streptomycin (46.6%), furaltadone (43.7%), chloramphenicol (41.7%), penicillin (25.2%), erythromycin (24.3%), and nalidixic acid (11.7%). Seventy-five percent (75%) of the farmers believed that veterinary prescriptions were not necessary because of their long years of experience in the poultry business. The purpose for use of antibiotics was mainly prophylactic (> 60%), and less frequently therapeutic (< 15%). It was also observed that 65% agreed it was always advisable to administer antibiotics regularly to prevent disease outbreaks. On the other hand, 70.9% were not aware that misuse of antibiotics in animals poses a serious risk to human health while 70% of the farmers lacked awareness of antibiotic residue.

Antimicrobial stewardship appears to be low among farmers, as some do not understand the need to observe the withdrawal periods of medicines before the slaughter of the animals, or where they do, sometimes fail to observe them.

Antimicrobials such as furazolidones and chloramphenicol, already banned by NAFDAC for use in animals, are still found to be freely marketed and used in livestock production in some parts of Nigeria (Adebowale et al. 2016; Ojo et al. 2016).

Some of the drivers of AMU identified in the animal sector are:

1. The need to prevent the occurrence of diseases or infections within flocks and herds is a major reason for the high use of antimicrobials within the animal health sector (Adebowale et al. 2016).
2. Poor adherence to treatment guidelines.

3. Many farmers routinely incorporate minute doses of antimicrobials into the animal feed or water to enhance growth and, in the case of poultry production, to increase egg size in order to guarantee more income from sales.
4. Feed manufacturers incorporate antimicrobials to improve the animals' feed efficiency, thus maintaining patronage from farmers.
5. Farmers treating their animals without consulting experts and sharing medication with other farmers.
6. Antibiotics usually used for treatment in many viral infections such as Newcastle disease, parvovirus, and lumpy skin disease, and in parasitic blood infections such as trypanasomosis.
7. The fear factor has been reported as a driver of AMU in the animal health sector (Achi et al. 2021). Farmers' fear of losing their animals and their investments is one major factor that drives AMU, and hence AMR. Animal market traders easily have access to antimicrobials and administer them to their animals while awaiting buyers. They do this out of fear of losing their animals before sale, and for fear of a diseased animal spreading an infection to other animals meant for sale. Veterinarians and other animal health workers also administer and prescribe antimicrobials as a "blind" treatment to get rid of any infection and for fear of losing clients who might question their competency and skills.

# 3. SWOT ANALYSIS OF THE RAPID ASSESSMENT

## 3.1. STRENGTHS

1. Existence of an AMR National Action Plan
2. Existence of Regulatory Council (Veterinary Council of Nigeria) for animal health
3. Existence of a One Health Technical Committee with an AMR National Technical Working Group (NTWG) coordinated by NCDC
4. Availability of a national drug policy
5. Existence of a national AMR reference laboratory and other sentinel laboratories for surveillance and antimicrobial susceptibility testing (AST)
6. Presence of indigenous veterinary pharmaceutical manufacturers
7. Established animal disease surveillance structure at the Federal Ministry of Agriculture and Rural Development
8. Availability of National Veterinary Formulary
9. Control of veterinary and human drugs is under one regulatory body

## 3.2. WEAKNESSES

1. Absence of a national veterinary drug policy
2. Lack of veterinary drug storage and distribution centers in the country
3. Weak animal health systems, especially at the state and local government levels
4. Inadequate policies and regulations on AMU in animals
5. No specific legislation addressing AMR/AMU in the animal sector
6. Lack of standard treatment guidelines (STGs) for animal health
7. Lack of an updated veterinary essential drugs list

8. Low antimicrobial stewardship in both the public and private veterinary sectors
9. Weak AMR surveillance in the animal health sector
10. Inadequate animal health laboratories to carry out microbial identification and AST
11. Lack of adequate data on AMU and consumption in animal production and health
12. Inadequate number of indigenous veterinary pharmaceutical manufacturers and overreliance on imported veterinary pharmaceuticals
13. Inadequate numbers of animal health professionals, especially at the local levels
14. Involvement of unqualified persons in the procurement, distribution, and sales of veterinary drugs, including antimicrobials
15. Lack of enforcement of laws and regulations on AMU
16. Unfettered access of farmers and nonprofessionals to antimicrobials
17. Involvement of nonprofessionals, including farmers, in the prescribing and dispensing of veterinary antimicrobials
18. Inadequate pharmacovigilance in the animal health sector
19. Lack of adherence to withdrawal periods before slaughter and sale of products

## 3.3. OPPORTUNITIES

1. One Health Approach to AMR/AMU
2. Leveraging existing health policies and surveillance systems in the One Health tripartite sectors to build AMR/AMU surveillance system
3. Possibility of using the AMR Community of Practice to institute antimicrobial education and stewardship among animal health providers and the community
4. Availability of a national AMR animal health reference laboratory and increase in the number of private veterinary laboratories for bacterial identification and AST to inform rational prescription of antimicrobials by veterinarians
5. Establishment of more indigenous pharmaceutical manufacturing companies for development of new antimicrobials
6. Targeting professional annual conferences, national forums and farmers' associations (Poultry Association of Nigeria, Miyyeti Allah Cattle Breeders Association of Nigeria, etc.) for advocacy activities
7. Leveraging the existing communication and social mobilization structures (social media platforms) at all levels for to carry out public awareness creation activities

## 3.4. THREATS

1. Insecurity in Nigeria especially, in the rural communities, where the farmers are located
2. Poor enforcement of regulations and policies on AMR/AMU control and prevention
3. Funding gap—no dedicated budget line for AMR/AMU control and surveillance
4. Chaotic veterinary drug distribution system in the private sector, high level of counterfeit medicines in circulation, and smuggling-in of unregistered medicines
5. Unregistered practitioners and prescription of antimicrobials by unlicensed persons
6. Illegal importation/smuggling of veterinary drugs in to the country

7. Lack of community veterinary pharmacies in rural areas and sale of prescription medicines by unlicensed persons
8. Poor coordination of research on antibiotic use
9. Non-recruitment of veterinary doctors and paraveterinary professionals, especially at the state and local government levels

## 4. KEY PRIORITY GAPS IDENTIFIED

1. Inadequate policies/regulations and enforcement in the supply chain, distribution, control, and use of antimicrobials in the animal health sector
2. Inadequate public awareness and engagement of stakeholders on prudent antimicrobial use and stewardship in the animal health sector
3. Poor antimicrobial use/consumption surveillance systems in the animal health and production sector
4. Inadequate veterinary laboratories for identification and AST to inform prescription and dispensing of antimicrobials for treatment, especially at the local levels

## 5. RECOMMENDATIONS

This situation analysis was essential to obtain a baseline of where the animal health sector is regarding the policies, use, and challenges for achieving optimal AMU and stewardship in Nigeria. Next, it will be used to develop a National Action Plan on antimicrobial stewardship. The recommendations below are focused on a One Health Approach for achieving the goals.

1. Give urgent priority to developing and establishing a national policy on AMR. Nigeria also needs to better implement and regulate policies related to animal health care delivery. These include but are not limited to the national veterinary drug policy and STGs for terrestrial and aquatic animals.

2. Ensure that Nigerian veterinary medicines and diagnostics supply chains are strengthened and secured. The introduction of a robust track-and-trace technology system should be explored as a solution to the gap in knowledge of the drug distribution system for monitoring access and use of antimicrobials. A robust regulatory framework needs to be established for supply chain control of all antimicrobials. A sound regulatory framework will ensure responsible supply and use of antimicrobials along the entire supply chain. This will be achieved by veterinary drug registration and supply chain control covering import, manufacture, distribution, and sale.

3. Strengthen the system to ensure prudent use of antimicrobials in veterinary medicine and reduce inappropriate use of antimicrobials in food-producing animals. This can be achieved through:

    - Establishing a roadmap to progress toward veterinary prescription for antimicrobials used in all animal sectors
    - Establishing national guidelines for prudent use of antimicrobials in terrestrial and aquatic animals (such as restricting the use of critically important antimicrobials for animal health)

- Designing targeted surveillance systems for AMU in terrestrial and aquatic animals and strengthening the system to assess antibiotics at the level of importation, distribution, and use in the veterinary sector
- Increasing awareness among stakeholders and the public on AMU in aquatic environments and antimicrobial resistance impacts
- Strengthening the legislation to prevent residues in food of animal origin
- Establishing national standards for food of animal origin to ensure food safety
- Phasing out growth-promoting antibiotics in animal husbandry
- Redrafting existing legislation to harmonize regulations, align with OIE guidelines, and clarify roles and responsibilities of regulators in monitoring and enforcement of regulations
- Enacting legislation that requires veterinary prescription for all veterinary antimicrobials, including those used in livestock and aquaculture.
- Strengthening/expanding regulations related to veterinary drug control to ensure prudent use of antimicrobials in the veterinary sector
- Preparing guidelines for antimicrobial usage in the veterinary sector as done in human health sector
- Expanding regulations related to use of drugs in aquatic environment, in relation to fisheries and aquaculture
- Establishing antibiotic stewardship program and monitoring system in the veterinary sector
- Strengthening control of antimicrobials and their use in animal health by farmers and para veterinarians without limiting access
- Ensuring adequate supplies of quality-assured essential antimicrobials at various levels of the animal health delivery and production system
- Promoting stewardship and pharmacovigilance in the use of antimicrobials in animals.
- Establishing an AMU monitoring system for use of antimicrobials in the animal sector
- Improving human resources and strengthening diagnostic laboratory capacity to improve the ability of animal health care professionals to make informed antimicrobial use decisions
- Developing STGs and an Essential Medicine List to reflect the current diseases and pathogens in animals

## REFERENCES


Achi CR, Ayobami O, Mark G, et al. Operationalizing One Health in Nigeria: reflections from a high-level expert panel discussion commemorating the 2020 World Antibiotics Awareness Week. Front Public Health. 2021;9:673504. doi: 10.3389/fpubh.2021.673504

Adebowale OO, Adeyemo OK, Awoyomi O, et al. Antibiotic use and practices in commercial poultry laying hens in Ogun State Nigeria. Revue d'élevage et de Médecine Vétérinaire des Pays Tropicaux. 2016;69(1):41-5.

Adekanye UO, Ekiri AB, Galipó E, et al. (2020). Knowledge, attitudes and practices of veterinarians towards antimicrobial resistance and stewardship in Nigeria. Antibiotics. 9:453. https://www.mdpi.com/2079-6382/9/8/453/htm

Alhaji NB, Isola TO. Antimicrobial usage by pastoralists in food animals in North-central Nigeria: the associated socio-cultural drivers for antimicrobials misuse and public health implications. One Health. 2018;6:41-7. https://www.ncbi.nlm.nih.gov/pmc/articles/PMC6249405/

Aliu YO. Veterinary drug residues in Nigeria's food. Paper presented at National Awareness Training Programme on Food Contaminants and Residues; May 6–7, 2004; Kaduna Nigeria.

Food and Agriculture Organization of the United Nations (FAO). The FAO action plan on antimicrobial resistance 2016–2020. Rome: FAO; 2016. https://www.fao.org/3/i5996e/i5996e.pdf

Igbinosa EO. Detection and antimicrobial resistance of vibrio isolates in aquaculture environments: implications for public health. Microbial Drug Resist. 2016;22(3):38-45. https://pubmed.ncbi.nlm.nih.gov/26540391/

Kabir J, Umoh V, Audu-Okoh E, et al. Veterinary drug use in poultry farms and determination of antimicrobial drug residues in commercial eggs and slaughtered chicken in Kaduna State, Nigeria. Food Control. 2004;15(2):99-105. https://www.researchgate.net/publication/223521790_Veterinary_drug_use_in_poultry_farms_and_determination_of_antimicrobial_drug_residues_in_commercial_eggs_and_slaughtered_chicken_in_Kaduna_State_Nigeria

Kimera ZI, Mshana SE, Rweyemamu MM, et al. Antimicrobial use and resistance in food-producing animals and the environment: an African perspective. Antimicrob Resist Infect Control. 2020;9:37. https://doi.org/10.1186/s13756-020-0697-x%0A

OIE World Organisation for Animal Health. The OIE strategy on antimicrobial resistance and the prudent use of antimicrobials. Paris: OIE; 2016. https://www.oie.int/app/uploads/2021/03/en-oie-amrstrategy.pdf

Ojo OE, Fabusoro E, Majasan AA, et al. Antimicrobials in animal production: usage and practices among livestock farmers in Oyo and Kaduna States of Nigeria. Trop Anim Health Prod. 2016; 48(1):189-97. https://link.springer.com/article/10.1007%2Fs11250-015-0939-8

O'Neill J. Tackling drug-resistant infections globally: final report and recommendations. Review on Antimicrobial Resistance. London: Wellcome Trust/HM Government; 2016. https://amr-review.org/sites/default/files/160518_Final%20paper_with%20cover.pdf

US Food and Drug Administration (FDA). 2015 Summary report on antimicrobials sold or distributed for use in food-producing animals. Washington, DC: FDA; 2016. https://www.fda.gov/downloads/ForIndustry/UserFees/AnimalDrugUserFeeActADUFA/UCM588085.pdf

World Health Organization (WHO). WHO guidelines on use of medically important antimicrobials in food-producing animals. Geneva: WHO; 2017. https://apps.who.int/iris/bitstream/10665/258970/1/9789241550130-eng.pdf?ua
