# Supplementary material for: Landscape Analysis of Existing Policies, Laws and Regulations on Antimicrobial Stewardship in 10 Low- and Middle-Income Countries
Source: Antibiotics (Basel). 2026 Aug 10;15(8):770. doi: 10.3390/antibiotics15080770 (PMC13510074; doi:10.3390/antibiotics15080770)
Supplement: Supplementary file 1 [file antibiotics-15-00770-s001.zip › S3 Country Reports/S3H_Senegal (Human & Animal Health).pdf]

IP3 Conseil
Vecteur de transformation

USAID

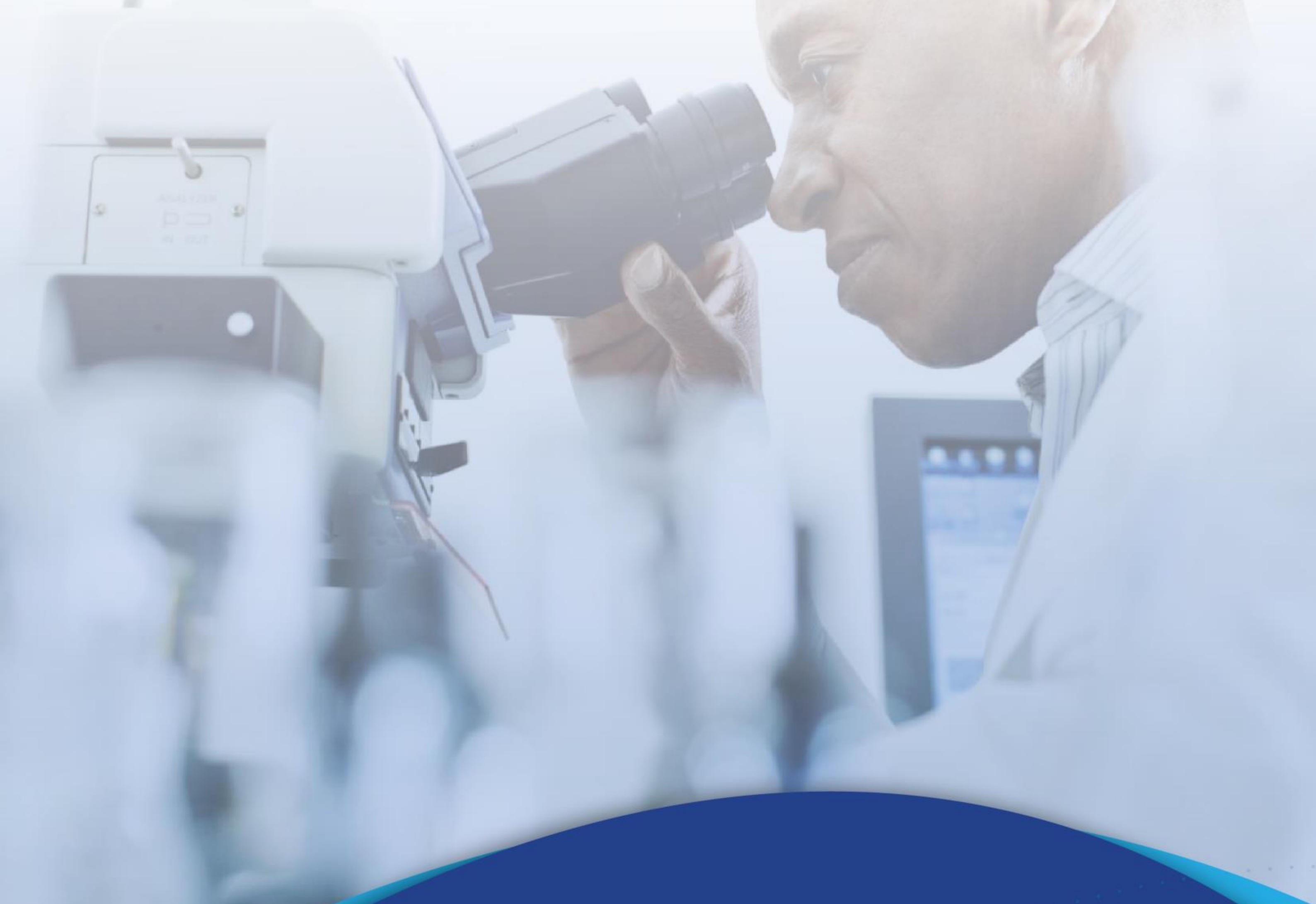

RAPPORT

ANALYSE SITUATIONNELLE RAPIDE DE LA REGULATION, DU CONTRÔLE ET DE L'UTILISATION DES ANTIMICROBIENS DANS LES SECTEURS DE LA SANTE HUMAINE ANIMALE ET VEGETALE

Sise Zone B N°38

+221 33 867 39 49
+221 77 650 45 47

contact@ip3conseil.com
www.ip3conseil.com

BP 11425 CP 13000
Dakar-Peytavin

SUMMARY

1. INTRODUCTION 11
1.1 Background 11
1.2. Antimicrobial stewardship issues - CAM 11
1.3. Relevance of rapid situational analysis 12
1.4. Purpose of the rapid situational analysis 12
1.5. Methodology for rapid situational analysis 13
1.5.1. Documentary review 14
1.5.2. Data collection 14
1.6. Outline of the situational analysis report 16
2. ORGANIZATION OF HEALTH CARE SYSTEMS 18
2.1. Organization of the human health system 18
2.1.1. General organization of the health system 18
2.1.2. Organization of the pharmaceutical sector 19
2.2 Organization of the animal and plant health system and regulatory framework 22
2.2.1. Organisation of the animal health system 22
2.2.1.1. The health mandate 25
2.2.1.2. Approval of veterinary medicinal products 25
2.2.1.3. Quality control of veterinary medicines 26
2.2.1.4 Pharmaceutical inspection and supply chain control 26
2.2.2. Organization of the plant health system and regulatory framework 26
3. STATUS OF RAM IN HUMAN AND ANIMAL HEALTH 29
3.1 STATE OF THE ART OF HUMAN HEALTH RAM 29
3.1.1. Mapping of actors and their roles 29
3.1.2. Surveillance of priority diseases and diseases with epidemic potential 31
3.1.3. Diagnosis of resistance profiles 33
3.1.4. Antimicrobial consumption pattern 35
3.2 STATE OF PLAY OF THE RAM IN ANIMAL HEALTH 48

3.2.1. Mapping of actors and their roles 48

3.2.2. Epidemiological surveillance of animal diseases 48

3.2.3. The annual livestock vaccination campaign 48

3.2.4. Antimicrobial resistance patterns in animal health 49

4. ANALYSIS OF THE REGULATORY FRAMEWORK 52

4.1. Mapping of actors and their roles 52

4.1.1. Mapping the actors and their roles in human health 52

4.1.2. Mapping of actors and their roles in animal health 54

4.1.3. Mapping of stakeholders and their roles in plant health 57

4.2 Status of antimicrobial regulation 59

4.2.1. Status of antimicrobial regulation in human health 59

4.2.2. Status of antimicrobial regulation in animal health 62

4.2.3. Status of antimicrobial regulation in plant health 64

5. QUALITY CONTROL 68

5.1. Quality control actors and their roles 68

5.1.1. Quality control actors and their roles in human health 68

5.1.2. Quality control actors and their roles in animal health 68

5.1.3. Quality control actors and their roles in plant health 69

5.2 Status of antimicrobial quality control 71

5.2.1. Status of antimicrobial quality control in human health 71

5.2.2. Status of antimicrobial quality control in animal health 74

5.2.3. Status of antimicrobial quality control in plant health 74

6. ANTIMICROBIAL USE 78

6.1. SUPPLY CHAIN ANALYSIS 78

6.1.1. Mapping of actors and their roles 78

6.1.1.1. Mapping the actors and their roles in human health 78

6.1.1.2. Mapping of actors and their roles in animal health 81

6.1.1.3. Mapping of stakeholders and their roles in plant health 83

6.1.2. State of play of the supply chain 83

6.1.2.1. Status of the antimicrobial supply chain in human health 83

6.1.2.2. Status of the antimicrobial supply chain in animal health 87

6.1.2.3. Status of the antimicrobial supply chain in plant health 88

6.2. ANALYSIS OF ACTUAL PRESCRIBING AND DISPENSING PRACTICES 91

6.2.1. Mapping of actors 91

6.2.1.1. Mapping of antimicrobial prescribers and dispensers in the human health sector 91

6.2.1.2. Mapping of antimicrobial prescribers and dispensers in the animal health sector 94

6.2.2. State of play of actual practices 95

6.2.2.1. Status of actual antimicrobial prescribing and dispensing practices in human health 95

6.2.2.2. Review of actual antimicrobial prescribing and dispensing practices in animal health 101

6.2.2.3. Overview of actual plant health practices 103

7. CONCLUSION AND RECOMMENDATIONS 105

7.1. Conclusion 105

7.2. Recommendations 105

7.2.1. Recommendations regulatory framework and quality control of antimicrobials... 105

7.2.2. Recommendations on rational use of antimicrobials... 105

8. BIBLIOGRAPHY 107

9. APPENDIX 110

GLOSSARY

ANCAR: National Agency for Agricultural and **Rural** Consultancy

MA: Marketing Authorization

APV: Autorisation Provisoire de Vente

ASN : Association Sénégalaise de Normalisation

ATC: Anatomical Therapeutic Chemical Classification

ATE : Technical Livestock Agent

ANSM : National Agency for the Safety of Medicines and Health Products

ESBL: Extended Spectrum Beta-Lactamase

BRH: Regional Hygiene Brigade

CAM : Antimicrobial Drug Consumption

CAP: Poison Control Centre

Case: Cephalosporinase

Casei: Inducible Cephalosporinase

CBN: Carbapenemase

CHN: High Level Cephalosporinase

CILSS: Inter-State Committee for Drought Control in the Sahel

IPPC: International Plant Protection Convention

CLIN: Committee for the Fight against Nosocomial Infections

CNCR: National Council for Rural Cooperation

NPC: National Pesticide Committee

CPRS: Centre de Promotion et de Réinsertion Sociale

CSP: Sahelian Pesticide Committee

DDD: Defined Daily Doses

DDG: Dispensation with Global Delivery

DDN: Dispensation á Délivrance Nominative

DLSI: Division for the Control of AIDS and Sexually Transmitted Infections

DMPV: Division of Veterinary Medicine and Pharmacy

DOPM: Direction de l'Océanographie et des Pêches Maritimes

DPC: Directorate of Civil Protection

DPCA: Direction de la Pêche Continentale et de l'Aquaculture

DPM: Directorate of Pharmacy and Medicines

DPV: Direction de la Protection des Végétaux (Plant Protection Directorate)

DRDR: Direction Régionale du Développement Rural

DSR/SE: Directorate of Reproductive Health/Child Survival

DTSS: Directorate of Labour and Social Security

DVRM: Deposit and Sale with Margin Return

EISMV: Interstate School of Veterinary Science and Medicine

EPS: Public Health Establishment

FAO: Food and Agriculture Organization of the United Nations

ISMED: Institute of Drug Sciences

ISRA: Senegalese Institute of Agricultural Research

ITE : Livestock Engineer

LCAB: Laboratory of Analytical Chemistry and Bromatology

LACOMEV: Veterinary Drug Control Laboratory

NMLC: National Drug Control Laboratory

LNERV: National Laboratory for Livestock and Veterinary Research

NDSL: National List of Essential Drugs and Products

MAER: Ministry of Agriculture and Rural Equipment

MEDD: Ministry of the Environment and Sustainable Development

MEPA: Ministry of Livestock and Animal Production

MLSB: Macrolides Lincosamides Streprogramine B

MSAS: Ministry of Health and Social Action

MSH: Management Sciences for Health

NTD: Neglected Tropical Diseases

OIE: World Organisation for Animal Health

WHO: World Health Organization

Pase: Penicillinase

PDIS: Programme de Développement Intégré de la Santé

PHN: High Level Penicillinase

NAP: National Supply Pharmacy

PNDSS: National Health and Social Development Plan

PNGA: National Antimicrobial Management Plan

NMCP: National Malaria Control Programme

NTP: National Tuberculosis Control Programme

PRA: Regional Supply Pharmacy

PRAPS: Regional Support Programme for Pastoralism in the Sahel

PRONALIN: National Program for the Fight against Nosocomial Infections

PS : Wild Phenotype

PSE: Plan Sénégal Emergent

NSP: National Strategic Plan

PUI : Internal use pharmacy

AMR: Antimicrobial Resistance

SAED : Société d'Aménagement des Terres du Delta

MSSA: Methicillin-susceptible Staphylococcus Aureus

SNH: National Hygiene Service

SNPV: National Pharmacovigilance System

SODAGRI: Agricultural and Industrial Development Company

SODEFITEX: Company for the Development of Textile Fibres

SARS: Regional Social Action Service

USAID: United States Agency for International Development

UEMOA: West African Economic and Monetary Union

LISTS OF TABLES

Table 1: List of interview guides related to the regulatory framework, quality control and use of antimicrobials in the human health, animal health and plant health sectors 15

Table 2: List of medical laboratories that perform antibiotic susceptibility testing 30

Table 3: Surveillance results for priority diseases and diseases with epidemic potential 2014 to 2017 31

Table 4: Type of Pathogens Isolated from Pathological Products 32

Table 5: The Bacteria Watch List 33

Table 6: Summary of structures involved in monitoring 36

Table 7: List of antimicrobials in the study 37

Table 8: Number of pharmaceutical references by data source 37

Table 9: AWaRe classification of antibiotics registered by DMP 60

Table 10: Sample of vegetable crops in the study 76

Table 11: Market shares of importing wholesalers of medicines and vaccines 87

Table 12: Consumption of active ingredients used in the formulation of plant protection products 89

Table 13: Imports of plant protection products in 2018 89

Table 14: Prevalence results and diagnostic data for antibiotic prescribing 95

Table 15: Distribution of 257 prescriptions by antibiotic family 96

Table 16: Distribution of appropriate antibiotic prescriptions by evaluation criteria 96

Table 17: Distribution of controlled molecules according to their quantity per batch unit in 2017 110

Table 18: Distribution of controlled molecules according to their quantity per batch unit in 2018 110

Table 19: Distribution of controlled molecules according to their quantity per batch unit in 2019 110

Table 20: Summary of standards on MRL1 (mg a.i./kg) by pair of vegetable speculation and active ingredient of pesticide for an assessment of the sanitary risk 111

Table 21: Pesticide residue levels found by vegetable crop and by locality 112

Table 22: Collection sheet 114

LIST OF CHARTS

Figure 1: Organization of the health system in Senegal 18

Chart 2: Distribution by sector ... 39
Graph 3: Distribution by structure ... 39
Figure 4: Distribution of references by ATC code ... 40
Figure 5; Distribution of references in the antibacterial class ATC code J01 ... 41
Graph 6: Distribution of penicillins and beta-lactams ATC code J01C ... 43
Figure 7: Distribution of other beta-lactams ATC code J01D ... 43
Figure 8: Distribution of macrolides ATC code J01F ... 44
Figure 9: Distribution of Fluoroquinolones J01MA ... 44
Graph 10; Distribution of references in the antimalarial class ... 45
Graph 11: Distribution of references by volume units and by year ... 45
Chart 12: Summary of antimicrobial testing activities over the past 3 years ... 72
Graph 13: Distribution of controlled molecules according to their quantity per batch unit ... 72
Figure 14: Pesticides Searched in the Study ... 76
Graph 15: Number of occurrences of an active ingredient in samples of vegetable crops ... 77
Figure 16: ANP Supply Chain ... 80
Figure 17: Evolution of antimicrobial drug imports by value ... 83
Figure 18: Value of imports (millions F.CFA) of antimicrobial drugs by therapeutic family over the last 5 years ... 84
Figure 19: Number of antimicrobial molecules imported in the last five years ... 84
Figure 20: Composition of drug imports ... 85
Graph 21: Total imports of phytosanitary products in Kg/Litre ... 88
Figure 23: Distribution of antimicrobials by health facility ... 100

LISTS OF FIGURES

Figure 1: MEPA / DSV PARTIAL ORGANIZATION ... 24
Figure 2: Map of medical laboratories performing antibiotic susceptibility testing ... 29
Figure 3: Monitoring system for plant protection products ... 70
Figure 4: distribution circuit for veterinary drugs in Senegal ... 82

Figure 5: Pesticide Marketing Flows 83

Figure 6; Mapping of pharmacies 93

1. INTRODUCTION

1.1. Context

Antimicrobial stewardship is defined as a series of interventions to optimize antimicrobial use and includes the selection, dosing, route of administration, and duration of antimicrobial therapy to optimize clinical patient outcomes and reduce related adverse events.

In other words, it can be considered as coordinated interventions to promote, monitor, improve, and evaluate the judicious use of antimicrobials in order to safeguard their future efficacy and protect and promote human and animal health.

Over the past decade, antimicrobial stewardship has become a global concern on the agendas of international organizations including the World Health Organization, the World Organization for Animal Health (OIE), the Food and Agriculture Organization of the United Nations (FAO), USAID etc.

For example, since 2015, WHO, FAO and OIE have recommended that all member countries in these organizations put in place coordinated antimicrobial stewardship plans in the human, animal, plant and environmental health sectors in a "One Health" spirit.

It is in this context that Senegal has received funding from USAID to develop a national antimicrobial stewardship plan. Thus, this rapid situational analysis study of the regulation and control of antimicrobials is part of this perspective.

1.2. Antimicrobial stewardship issues - CAM

Antimicrobial drugs are natural, synthetic or semi-synthetic substances with the ability to kill microorganisms and/or inhibit their growth.

They have long been used for the treatment, control and prevention of infectious diseases in human and animal medicine.

Since their discovery, they have proven to be very effective in fighting infections that were sometimes serious or fatal for humans and animals.

Medicines have always proved to be effective solutions to many of the world's health problems that seriously threaten human and animal health.

As a result, antimicrobial drugs have always played an important role in global and national health systems. Their appropriate use has helped to address several human and animal health threats.

However, their excessive and inappropriate use in human and animal medicine, as well as their use for non-therapeutic purposes in the agri-food chain, has led to the development of resistance in microbial agents.

1.3. Relevance of rapid situational analysis

The rapid situational analysis of the regulation, control and use of antimicrobials aims to collect, analyze, synthesize and share data on the organization and supervision of antimicrobial use in Senegal.

It provides an opportunity to review the shortcomings of the strategies in place to control the consumption of antimicrobial drugs and to influence policy directions to better manage and control their circulation and consumption.

It is a good strategy to demonstrate the extent of the problem of antimicrobial drug consumption and the deviations from the rules of prescribing, rational dispensing and proper use.

Indeed, the rapid situational analysis makes it possible to assess the legislative framework in place for sound management by highlighting its strengths (best practices) and weaknesses (challenges or areas to be strengthened).

It also allows the identification of recommendations to be taken into account in the elaboration of the NGP. In addition, the rapid situational analysis makes it possible to identify or anticipate the main indicators for monitoring the PNGA. Finally, it will allow the various strategic axes of the NGAP to be put into perspective.

1.4. Objective of the rapid situational analysis

The rapid situational analysis of the regulation and control of antimicrobial drugs will provide a comprehensive overview of the strengths and weaknesses of the regulatory framework for the antimicrobial drug value chain.

The overall objective of this study is to review the regulatory and quality control framework for antimicrobial drugs, including production, importation, distribution, prescribing, dispensing and rational use.

Specifically, it is about :

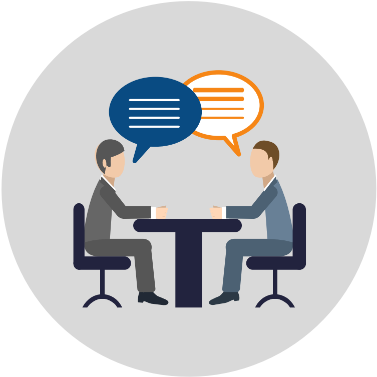

01 **Faire l'état des lieux du cadre ou dispositif institutionnel mis en place dans le cadre du suivi de l'usage rationnel des antimicrobiens ;**

02 **Faire un aperçu des politiques et réglementations en vigueur pour optimiser l'usage rationnel des médicaments ainsi que de leur applicabilité ;**

03 **Faire un diagnostic des politiques de contrôle de la qualité des antimicrobiens ainsi que de leur applicabilité.**

1.5. Methodology of rapid situational analysis

Strategies were deployed to conduct the rapid situational analysis. The overall methodology implemented in the framework of the mission was multisectoral and participatory. This inclusive approach with the participation of the various stakeholders is one of the four fundamental points of the multisectoral methodological approach implemented by the team of experts.

Within the framework of this rapid situational analysis, this participatory dimension was carried out through a meeting to harmonize the methodological approach with the sponsor, the USAID MTaPS (Medicines, Technologies and Pharmaceutical Services) program implemented by Management Sciences for Health (MSH), and a strategic orientation workshop attended by the main actors involved. The workshop was held at the Directorate of Pharmacy and Medicines (DPM) and was attended by representatives of the Directorate of Pharmacy and Medicines, the National Drug Control Laboratory, the USAID MTaPS program and the consultant IP3-Conseil.

The various stages of this process were interactive and iterative. They allowed for the harmonization of the multisectoral methodological approach and the validation of the schedule of activities and deliverables, the taking into account of the expectations of the various stakeholders in the rapid situational analysis phase, the setting up of a monitoring mechanism for the mission or technical committee and the identification of the stakeholders to be involved.

1.5.1. The document review

Senegal already has a great deal of experience in developing strategic plans as it developed, in 2017, a multisectoral national action plan for the management and control of antimicrobial resistance - AMR and which was validated at the end of that same year. This experience deserves to be capitalized.

Thus, the document review is an important step of the mission. This documentation phase made it possible to take stock of the situation and to document the strategy implemented to date in this area. More specifically, on the one hand, the exploitation of the documentation relating to the action plans implemented. On the other hand, it is the study of :

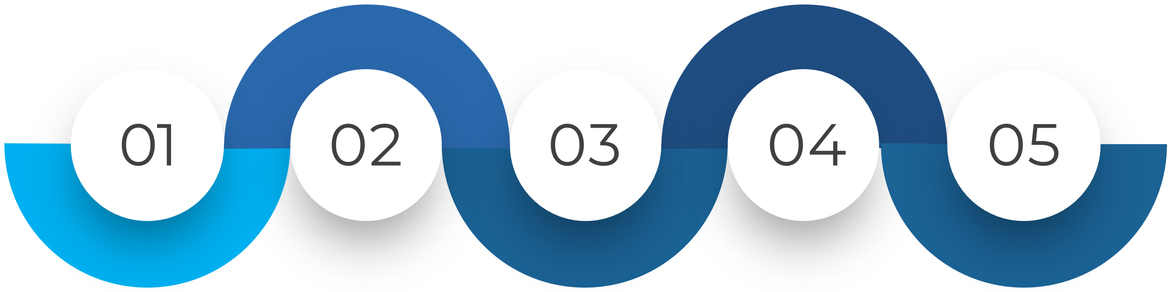

La situation de l'utilisation des antimicrobiens dans les secteurs de la santé humaine et animale ;

La situation de la régulation et du contrôle de l'utilisation des antimicrobiens dans les secteurs de la santé humaine et animale ;

Les principaux acteurs intervenant dans l'utilisation des antimicrobiens en santé humaine et animale ainsi que leurs rôles et responsabilités ;

Les principaux axes d'intervention et de suivi pour faire face aux principaux problèmes identifiés lors de l'évaluation.

Les principaux acteurs intervenant dans l'utilisation des antimicrobiens en santé humaine et animale ainsi que leurs rôles et responsabilités ;

1.5.2. Data collection

The data collection methodology for the rapid situational analysis of the regulatory framework, quality control and antimicrobial use in the human, animal and plant health sectors embraces the multisectoral nature of the "One Health" concept.

It takes into account all the dimensions of rational management (regulation, production or importation, marketing, consumption, quality control and use, etc.) and all the actors involved in the management of antimicrobials in human health, animal health and plant health.

Thus, in this rapid situational analysis of antimicrobial regulation, quality control, and use, **15 interview guides** were developed to collect all the information needed to complete the regulatory, quality control, and antimicrobial use scan.

It also includes data from the survey conducted by the CERES LOCUSTOX Foundation laboratory among 90 producers of market garden crops in the Niayes area.

The following table presents the different interview guides and the actors involved in the interviews.

Table 1: List of interview guides related to the regulatory framework, quality control and use of antimicrobials in the human health, animal health and plant health sectors

DOMAINS	REGULATORY FRAMEWORK MAINTENANCE GUIDES	RELEVANT STAKEHOLDERS	SAMPLE SIZES
**Human health**	Maintenance guide for the regulatory framework	• Directorate of Pharmacy and Medicines<br>• National Drug Control Laboratory	3
	Supply Chain Management Interview Guide	Wholesalers Distributors	3
	Provider Interview Guide	Pharmacists	29
	Prescriber Interview Guide	Caregivers	53
**Animal health**	Regulatory Framework Interview Guide	• Order of Veterinary Doctors of Senegal<br>• Direction of Veterinary Services	2
	Interview guides for veterinary microbiological laboratories	• LACOMEV - Interstate School of Veterinary Science and Medicine<br>• National Laboratory for Livestock and Veterinary Research - LNERV	2
	Interview Guide for Veterinary Drug Control Laboratories	LACOMEV - Interstate School of Veterinary Science and Medicine	1
	Supply Chain Management Interview Guide	Importing wholesalers	6
	Maintenance Guide for Food Manufacturers	Animal feed industry	4
	Interview Guide for Veterinary Practices	Livestock Engineers and Livestock Technicians	10
	Interview Guide for Veterinary Practices	Veterinary doctors	35
	Interview guide for pharmacies	Pharmacists	9
	Interview guide for public service workers and NGOs	Public service agents and NGOs	12
	Interview Guide for Livestock Producers	Breeders	34
**Plant health**	Interview guide for institutional stakeholders	Direction de la Protection des Végétaux	1
	Surveys of producers in the Niayes area carried out by the CERES Foundation laboratory LOCUSTOX	Farmers	90

1.6. Outline of the situational analysis report

This rapid situational analysis document on the regulation, control and use of antimicrobials in the human health, animal health and plant health sectors is structured in six (6) parts described below.

In each section, the rapid situation analysis is done separately for the human health, animal health and plant health sectors, except in the case of the AMR status report, which specifically concerns human and animal health.

The structure of this report is as follows:

- Organization of health systems
    - Organization of the human health system
    - Organization of the animal health system and regulatory framework
    - Organization of the plant health system and regulatory framework
- State of the art on AMR in human health
    - Mapping of actors and their roles
    - Surveillance of priority diseases and diseases with epidemic potential
    - Diagnosis of resistance profiles
    - Antimicrobial consumption profile
- State of the art on AMR in animal health
    - Mapping of actors and their roles
    - Epidemiological surveillance
    - Annual livestock vaccination campaign
    - Resistance profiles
- Rapid situational analysis of the regulatory framework for antimicrobial use
    - Mapping of actors and their roles in human, animal and plant health
    - Status of antimicrobial regulation in human, animal and plant health
- Rapid situational analysis of antimicrobial quality control
    - Mapping of actors and their roles in human, animal and plant health
    - Status of antimicrobial quality control in human, animal and plant health
- Rapid situational analysis of antimicrobial use
    - Supply chain analysis in human, animal and plant health
    - Analysis of actual prescription practices, dispensing in human, animal and plant health and use in animal and plant production
- Conclusion and recommendations

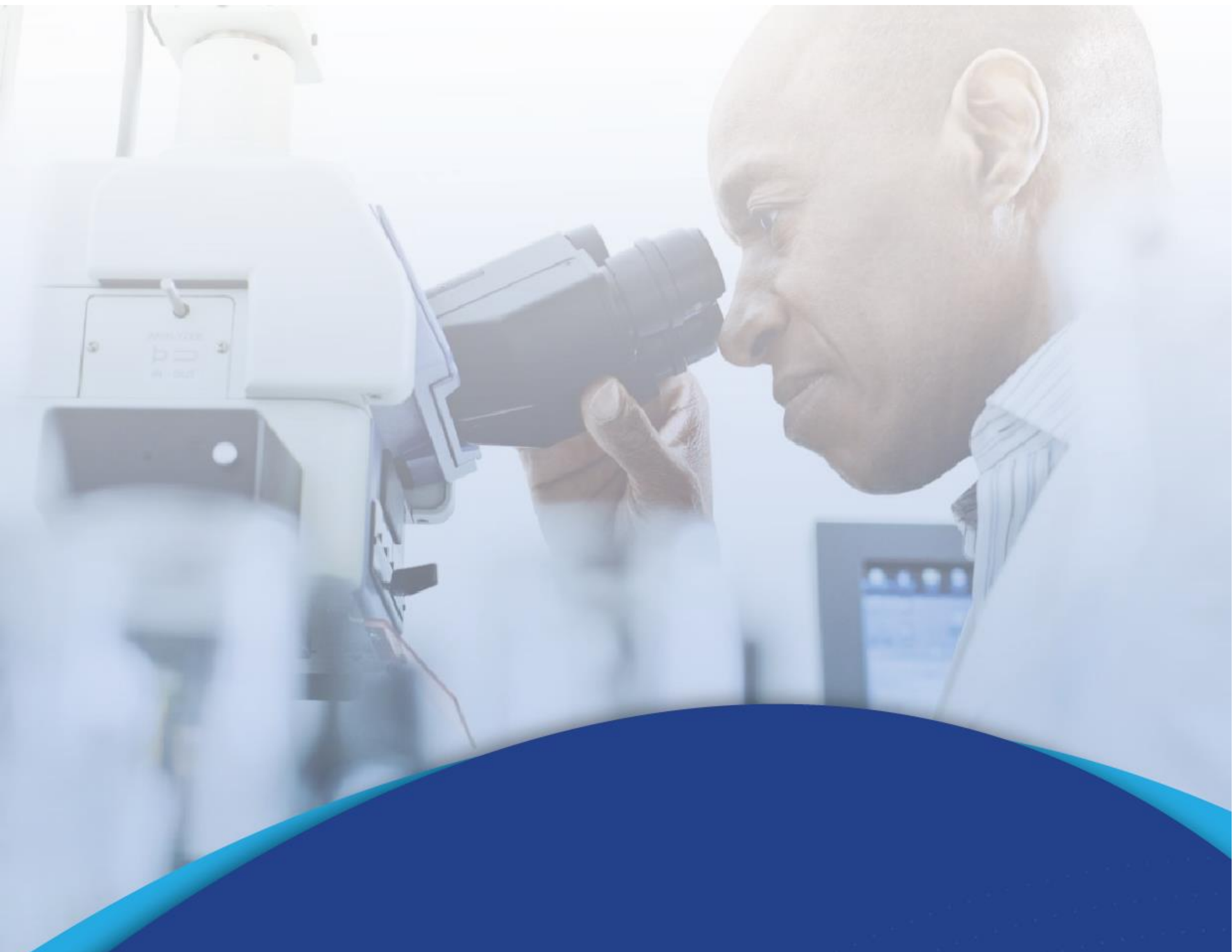

ORGANISATION DES SYSTEMES DE SANTE HUMAINE, SANTE ANIMALE ET SANTE VEGETALE

2. ORGANIZATION OF HEALTH CARE SYSTEMS

2.1. Organization of the human health system

2.1.1. General organization of the health system

The human health sector in Senegal is under the supervision of the Ministry of Health and Social Action, which is responsible for implementing the National Health and Social Action Policy, in collaboration with other ministries, partners, local authorities, programmes, agencies and institutions.

For the effective implementation of the National Health and Social Action Policy, the Ministry of Health and Social Action has adopted a multi-year planning document called the Integrated Health Development Programme (PDIS), which has evolved into the National Health and Social Development Plan (PNDS).

These multi-year planning documents have made it possible to carry out major reforms in the organization and operation of the health and social welfare sector in order to bring it into line with international standards, to comply with Community directives and to integrate the strategic guidelines of line II "*Human capital development, social protection and sustainable development*" of the Emerging Senegal Plan.

The organization of the health and social action sector is pyramidal. The positioning of the various operational structures has been integrated into the administrative division of the country in the interests of territorial equity.

Thus we find :

- A central level which includes the Minister's Office, the General Secretariat, the General Directorates, the National Directorates, the Central Services attached, the National Centres for Social Reinsertion and the Level 3 Public Health Establishments;
- A strategic intermediate level that includes the Medical Regions, the Regional Hygiene Brigades (BRH), the Regional Social Action Services (SRAS) and the Level 2 Public Health Establishments;
- An operational peripheral level with the Health Districts, the Hygiene Sub-Brigades, the Departmental Services of Social Action, the Centres for Promotion and Social Reinsertion (CPRS) and the Level 1 Public Health Establishments.

The health status of the Senegalese population is marked by :

- Still high maternal and infant mortality and morbidity rates;
- A persistent burden of communicable diseases, despite significant progress over several decades ;
- A rapid increase in the burden of non-communicable diseases, most of which are chronic, high-cost diseases.

The graph below illustrates the organization of the health system in Senegal

Figure 1: Organization of the health system in Senegal

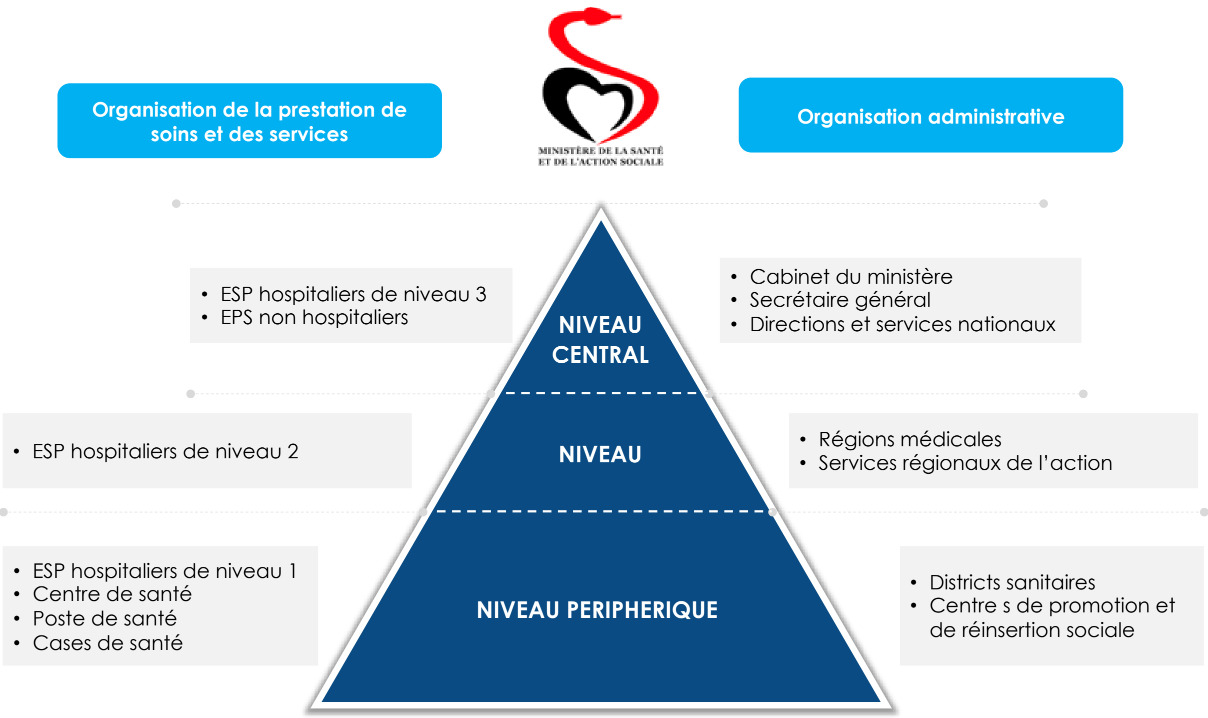

Source: PNDSS 2019 - 2028

2.1.2. Organization of the pharmaceutical sector

The pharmaceutical sector in Senegal comprises several institutional and operational actors who work towards a common goal, namely to make medicines available, financially and geographically accessible, of guaranteed quality, efficacy and safety, at the lowest cost and used rationally.

The regulatory function of the pharmaceutical sector is entrusted to the Directorate of Pharmacy and Medicines - DPM, whose mission is to develop, implement and monitor policy and programs in the field of pharmacy and medicines.

As such, it is responsible for drafting and ensuring the application of legislative and regulatory texts relating to pharmacy, medicines and medical devices.

Regulations require a prescription for any use of antibiotics in human and animal health. However, difficulties are noted in the respect of their application.

The Pharmacy and Drug Directorate provides the following regulatory functions:

- **Drug Licensing**

The approval of medicines is subject to a regulatory procedure for obtaining marketing authorisations (MA).

All applicants for drug approval must design and submit an administrative and technical file commonly referred to as a "technical file for marketing authorization application" to the Directorate of Pharmacy and Medicine.

The format and content of the technical file must be in accordance with the annex to Regulation No. 06/2010/CM/WAEMU and Circular No. 00310 of February 7, 2011 on the approval procedures for pharmaceutical products for human use in WAEMU Member States.

The technical part of the marketing authorisation applications is entrusted to groups of experts who are responsible for evaluating the quality, efficacy and safety of the candidate medicines. After evaluation, the experts submit their evaluation reports.

These marketing authorisations (MA) are issued by order of the Minister of Health and Social Action after a reasoned opinion from the national visa commission. They are renewed every five years.

- The DPM has drawn up and posted on its website a list of medicines with valid marketing authorisations. This database is periodically updated according to the issuance of new MAs or the deferment of existing MAs. This list is available (see Excel file "Antimicrobial list with MA" attached to the situational analysis report). An extract of this list composed of antibiotics registered from 2015 to 2017 classified according to the WHO AWaRe classification is presented in the Human Health Antimicrobial Regulatory Update (see Table 9)

Technical quality control of medicines for human use is carried out by the National Laboratory for the Control of Medicines (LNCM).

The mission of the LNCM is to ensure the technical control of medicines in relation with the Directorate of Pharmacy and Medicines (DPM) and the National Supply Pharmacy (PNA).

For the quality control of antibiotics, the NMLC has a microbiological control laboratory, with all the necessary facilities and processes, but which struggles to function properly due to a lack of logistics. We have presented a balance sheet of their activities during the last years 2017-2019 (see graph 12 and 13).

The LNCM has established and implemented a technical collaboration with the malaria (PNLP), tuberculosis (PNT), HIV/AIDS and the Directorate of Reproductive Health and Child Survival (DSR/SE) programs to monitor the quality of antimalarials, antituberculosis drugs, antiretrovirals and reproductive health drugs at the dispensing sites

This partnership allows for the monitoring of the quality of medicines of these programmes (PNLP, PNT, DLSI, DSR/SE, etc.) at the peripheral level with the MINILAB programme. The MINILAB programme only concerns antimalarials, anti-tuberculosis drugs, antiretroviral drugs and reproductive health/child survival drugs. Antibiotics are not included in this surveillance.

- **National Pharmacovigilance System**

The National Pharmacovigilance System (SNPV) of Senegal was established in 1998 by ministerial order (Order No.[0] 4012 of February 6, 1998 and Order No.[0] 05036 of April 22, 2009 on the national pharmacovigilance system). The whole circuit from notification, to data processing, to decision making, to feedback... has been codified and put in place.

This system is coordinated by the Directorate of Pharmacy and Medicines. The SNPV includes all the actors of the health system: the Health Programs, the Anti-Poison Center, the National Drug Control Laboratory, the Expanded Program of Immunization, the Public and Private Health Establishments, the Medical Regions, the Health Professionals.

In Senegal, since 2009, the National Pharmacovigilance System (SNPV) set up has made 132 reports of medicines with adverse effects, including 42 antimicrobials, i.e. 32% of medicines.

A total of 3,816 reactions were reported, of which 3,449 reactions (90% of the reactions) were from antimicrobials.

The following table shows the distribution of antimicrobials and the number of reactions recorded by therapeutic class in the period 2009-2019. (More detailed table see annex)

Table 2: Number of reactions recorded by therapeutic class

Therapeutic class	Antimicrobials		Reactions	
	Number	Percentage	Number	Percentage
Antibiotic	17	40%	64	2%
Antifungal	2	5%	4	0%
Anti-malarial	12	29%	3280	95%
Pest control	7	17%	97	3%
Antituberculosis	4	10%	4	0%
**Grand total**	**42**	**100%**	**3449**	**100%**

Source: Pharmacy and Drugs Directorate

Senegal developed a National Pharmacovigilance Guide in 2010 with support from the Global Fund, USAID, WHO and the AIDS and Sexually Transmitted Infections Control Division (DLSI) of the Ministry of Health and Social Action.

- **Pharmaceutical inspection**

The DMP has a pharmaceutical inspection service, which is responsible for carrying out regulatory inspections of pharmaceutical establishments.

Cheikh Anta Diop University has created an Institute of Drug Sciences (ISMED) in charge of training pharmacy inspectors from WAEMU countries.

A pharmaceutical inspection guide has just been developed and posted on the DMP website.

Regulatory inspections are used to monitor and evaluate the regulatory compliance of pharmaceutical establishments involved in the manufacture, distribution, dispensing and quality control of medicines. No fines or penalties have ever been imposed on pharmaceutical establishments in Senegal.

Indeed, only legislative aspects are taken into account in regulatory inspections. Therefore, due to the lack of regulatory provisions, the DMP does not yet have a legal basis to impose fines or penalties on pharmaceutical establishments.

They may be scheduled, unannounced, or following problems of non-compliance with regulatory requirements of good manufacturing, distribution, dispensing and laboratory practices.

- **Licensing of pharmaceutical establishments**

In Senegal, all pharmaceutical establishments engaged in the manufacture, importation, distribution and dispensing of medicines are subject to an administrative procedure for obtaining authorizations to open and operate a pharmaceutical establishment.

Opening authorizations are obtained after evaluation of the application files according to criteria defined by order of the Minister of Health and Social Action.

The operating permits are obtained after all the required investments have been made and certified as complying with the standards in force, after the regulatory inspection of the installations by the DPM inspectors.

- **Control of the supply chain**

The DPM has authorized four drug distributors that supply private dispensing structures. The National Supply Pharmacy is the public drug purchasing centre which has the status of a non-hospital public health establishment. It is responsible for supplying public health facilities.

2.2. Organization of the animal and plant health system and regulatory framework

2.2.1. Organisation of the animal health system

Until 1992, the Senegalese government had a virtual monopoly on the provision of veterinary services, providing most animal care and veterinary drugs to farmers at subsidised rates. Only a small number of private animal health facilities existed at that time.

After 1992, a new context emerged with the implementation by the State of a policy of privatisation of veterinary services, which led to a distinction between a public sector (in charge of State missions) and a private sector (in charge of the private practice of the veterinary profession).

- **The public animal health sector**

At the public level, the central level in charge of animal health missions is represented by the Ministry of Livestock and Animal Production (MEPA) through its Directorate of Veterinary Services (DSV).

The Veterinary Services Directorate comprises :

- Zoo Sanitation Division;
- Veterinary Public Health Division;
- the Division of Veterinary Medicine and Pharmacy.

At the peripheral level, its branches are the Regional and Departmental Livestock Services and the Veterinary Stations under the respective responsibility of veterinary doctors, ITEs (Livestock Work Engineers) and ATEs (Livestock Technical Agents) who must devote their professional time to the service of the State (Law 2008-07 of 24 January 2008).

Figure 1: MEPA / DSV PARTIAL ORGANIZATION

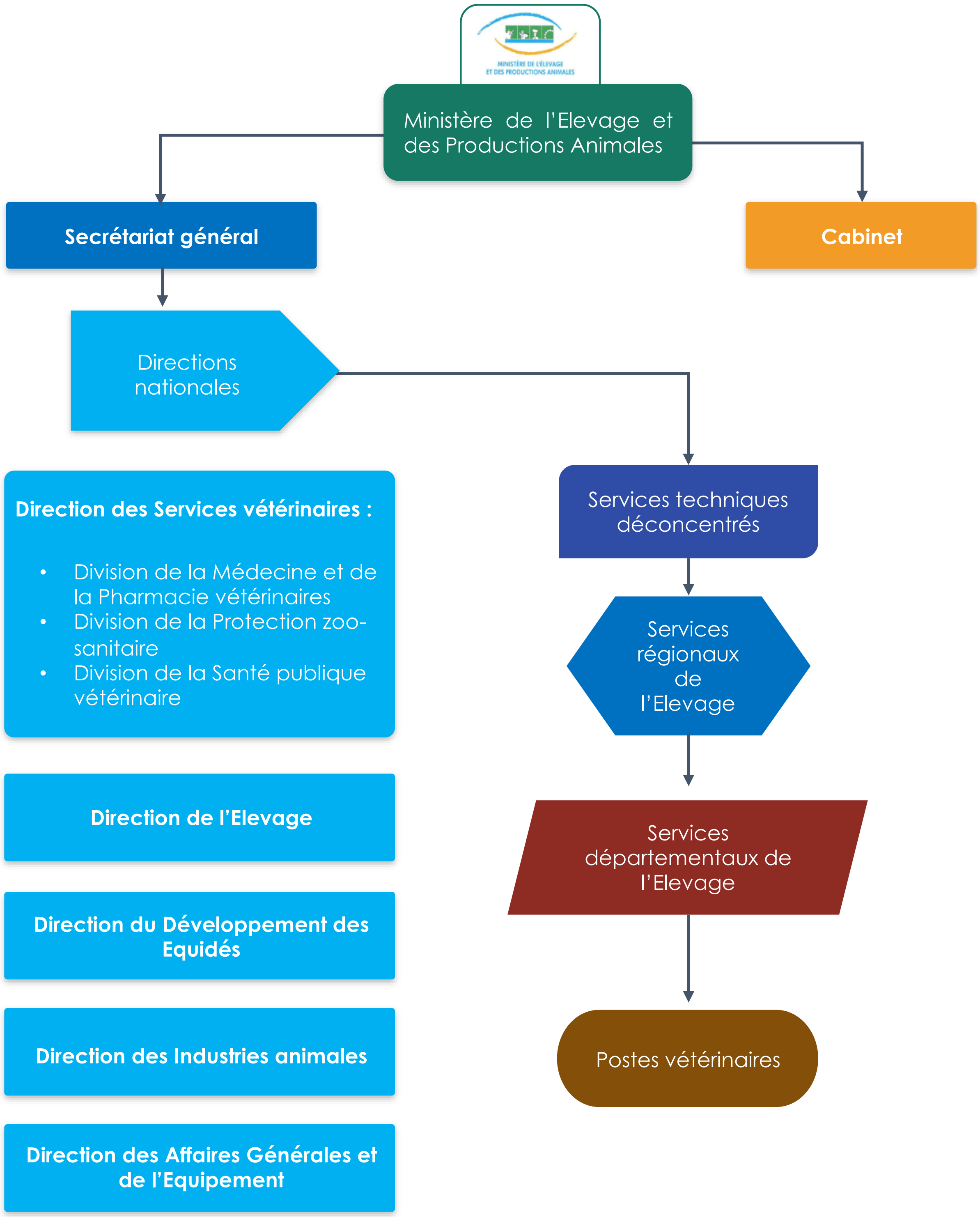

Source: Ministry of Livestock and Animal Production (MEPA)

- **The private animal health sector**

Animal health services in the private sector are provided by the following private structures

- Import and wholesale distribution structures for veterinary drugs ;
- Veterinary practices, clinics and pharmacies owned by veterinary doctors;
- Veterinary practices owned by ITEs or ATEs.

It is important to note that livestock auxiliaries (helpers) trained in the application of primary care are used as relays for these practices in order to reach remote rural areas. Public services such as NGOs (non-governmental organizations) can also call on them in the course of their activities.

Private practice remains under the supervision and control of the MEPA and the Order of Veterinary Doctors of Senegal (ODVS), as is the case for the health mandate.

2.2.1.1. The health mandate

In order to accompany the process of privatisation of veterinary medicine and to strengthen the existing sanitary system, the Senegalese State, by Decree No. 95-645 of 6 July 1995, grants the execution of the sanitary mandate to private veterinary doctors who request it. This mandate is intended for the execution of collective prophylaxis operations against certain animal diseases.

2.2.1.2. Approval of veterinary medicinal products

Any veterinary drug circulating in Senegal must obtain a marketing authorization (MA). This MA was previously issued by the Minister in charge of livestock following the approval of a commission that studied the files. However, since 2006, as registration has become community-based, no veterinary medicine should be administered to animals or delivered to the public unless it has first received a marketing authorisation issued by the UEMOA (West African Economic and Monetary Union) Commission under the conditions set out in Regulation N°02/2006/CM/UEMOA of 23 March 2006. Thus, the medicine covered by this MA can circulate freely in the various member states of the Union as long as this document is valid.

2.2.1.3. Quality control of veterinary medicines

This control is carried out by the DSV through its Division of Veterinary Medicine and Pharmacy (DMPV). Samples are taken along the distribution chain and analyses are carried out at the LACOMEV of the EISMV in Dakar.

2.2.1.4. Pharmaceutical inspection and supply chain control

These inspections and controls are also the responsibility of the DMPV (DSV/MEPA).

It is a Division composed of two Offices that process import requests for veterinary drugs and nutrition products and ensure the inspection and control of drugs in collaboration with the Veterinary Services of the Port and Airport. For the surveillance of the parallel market, the central team is supported by the deconcentrated services.

The main operations are carried out during the CAMVET (Campaigns for the Sanitation of the Veterinary Medicine Market). A national campaign to clean up the veterinary drug market is conducted every year. The actors are the Veterinary Services Directorate, the Deconcentrated Livestock Services, the ODVS, the PTFs and the Law Enforcement and Security Forces.

2.2.2. Organization of the plant health system and regulatory framework

The regulation of pesticides is based on the law n°84-14 of February 2, 1984 relating to the control of agro pharmaceutical specialities and similar specialities and the decree n°84- 503 of May 2, 1984 carrying application of the aforementioned law.

It should also be noted that Order No. 47-47 of 22 April 1971 regulates the packaging used for agricultural pesticides formulated in Senegal. Other decrees have been issued in the context of pesticide management in Senegal. These are as follows

- The Order n° 05381 of May 20, 1985 which fixes the composition and the rules of organization of the National Commission of Approval of the Agro pharmaceutical Specialities and the Assimilated Specialities;
- Ministerial Order n° 10777 of August 4, 1992 creating an intermediary of revenues at the Ministry of Rural Development and Hydraulics relating to the control of agro pharmaceutical specialities and Assimilated Specialities
- Order n° 000149 of January 11, 1994 appointing the manager of the revenue fund entitled Control of Agro-Pharmaceutical Specialities and Similar Specialities
- The interministerial decree n° 10390 of December 2, 1994 fixing the amount of the royalty relating to the approval of the Agro pharmaceutical Specialities and the Assimilated Specialities.

Along with the decree that set up the National Approval Commission, the three other decrees should ensure and complete the proper functioning of the structure in charge of approving pesticides in Senegal.

Other texts deal in part with pesticides:

- Law No. 2001-01 of 15 January 2001 on the Environment Code;
- Prime Ministerial Order No. 005161 of 26 May 1995 (National Commission for Sustainable Development) ;
- Ministerial Order N°000852 of February 8, 2002 (National Chemicals Management Commission) ;
- Catalogue of Senegalese standards, 1996 edition (standard on pesticide residues);
- Ministerial Order N°3504/MEA dated 09 May 2001 establishing a "National Committee to monitor the programme to promote the intrinsic quality (pesticide residues) of fruit and vegetables for export", chaired by the Director of Cabinet of the Ministry of Agriculture.

A draft decree regulating the use of biological control agents and bio-pesticides was presented and introduced in the circuit of visas and signatures.

At the international level, Senegal,

- adopted the FAO "International Code of Conduct on the Distribution and Use of Pesticides" and ratified the 1999 revised version of the International Plant Protection Convention (IPPC);
- is a stakeholder in the FAO's International Standards for Phytosanitary Measures (ISPM).

Other international agreements signed, ratified or adopted by Senegal are

- Code of Ethics on International Trade in Chemicals of April 1994;
- International Code of Conduct on the Distribution and Use of Pesticides ;
- Basel Convention on the Transboundary Movement of Hazardous Wastes of 22 March 1989 ;
- African Convention on the Prohibition of the Import into Africa of Hazardous Wastes in all Forms and the Transboundary Control of such Wastes generated in Africa;
- Rotterdam Convention on the Principle of Prior Informed Consent (PIC) ;
- Stockholm Convention on Persistent Organic Pollutants (POPs) ;
- Agreement on "Common regulations on the registration of pesticides in CILSS member countries";
- London Guidelines for the Exchange of Information on Chemicals in International Trade UNEP/GC/17.

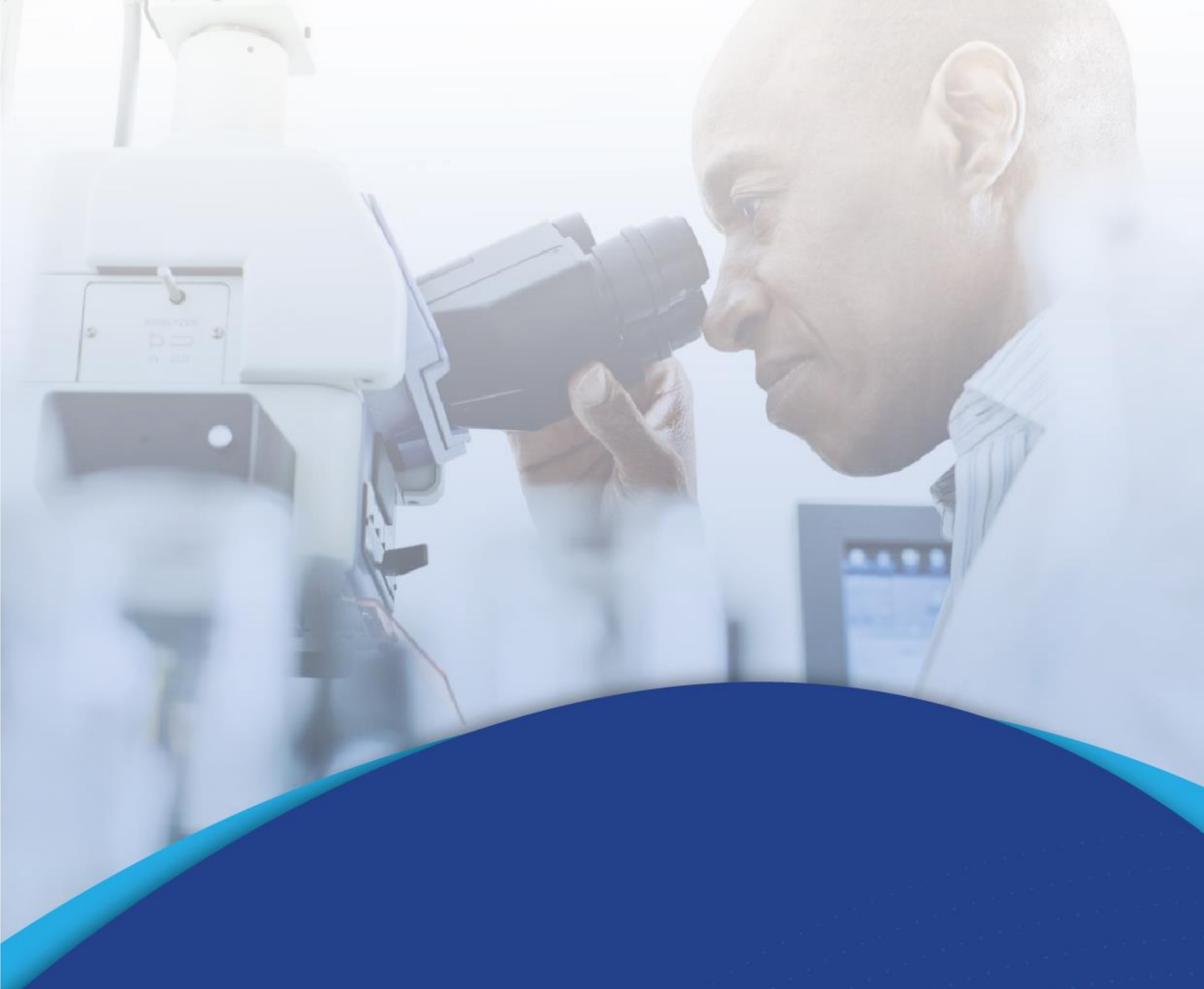

ETAT DES LIEUX DE LA RESISTANCE AUX ANTIMICROBIENS (RAM) EN SANTE HUMAINE ET SANTE ANIMALE

3. STATE OF THE ART OF RAM IN HUMAN AND ANIMAL HEALTH

3.1. STATE OF THE ART IN HUMAN HEALTH RAM

3.1.1. Mapping of actors and their roles

The Directorate of Laboratories of the Ministry of Health and Social Action (MSAS) is the institutional actor in charge of coordinating the implementation of national AMR surveillance strategies.

As part of its missions, the Laboratories Directorate is responsible for supervising the activities of public and private medical biology laboratories concerning the monitoring of antimicrobial resistance (AMR).

The Laboratories Directorate also ensures the implementation of the National Multisectoral Plan for Surveillance and Control of Antimicrobial Resistance, developed in 2017 and covering the period 2018-2022.

The National Public Health Laboratory of Thiès (human health) and the National Laboratory of Livestock and Veterinary Research (animal health) are the reference laboratories for AMR.

The map of medical laboratories that perform antibiotic susceptibility testing and therefore AMR monitoring is presented in Figure 1.

Figure 2: Map of medical laboratories performing antibiotic susceptibility testing

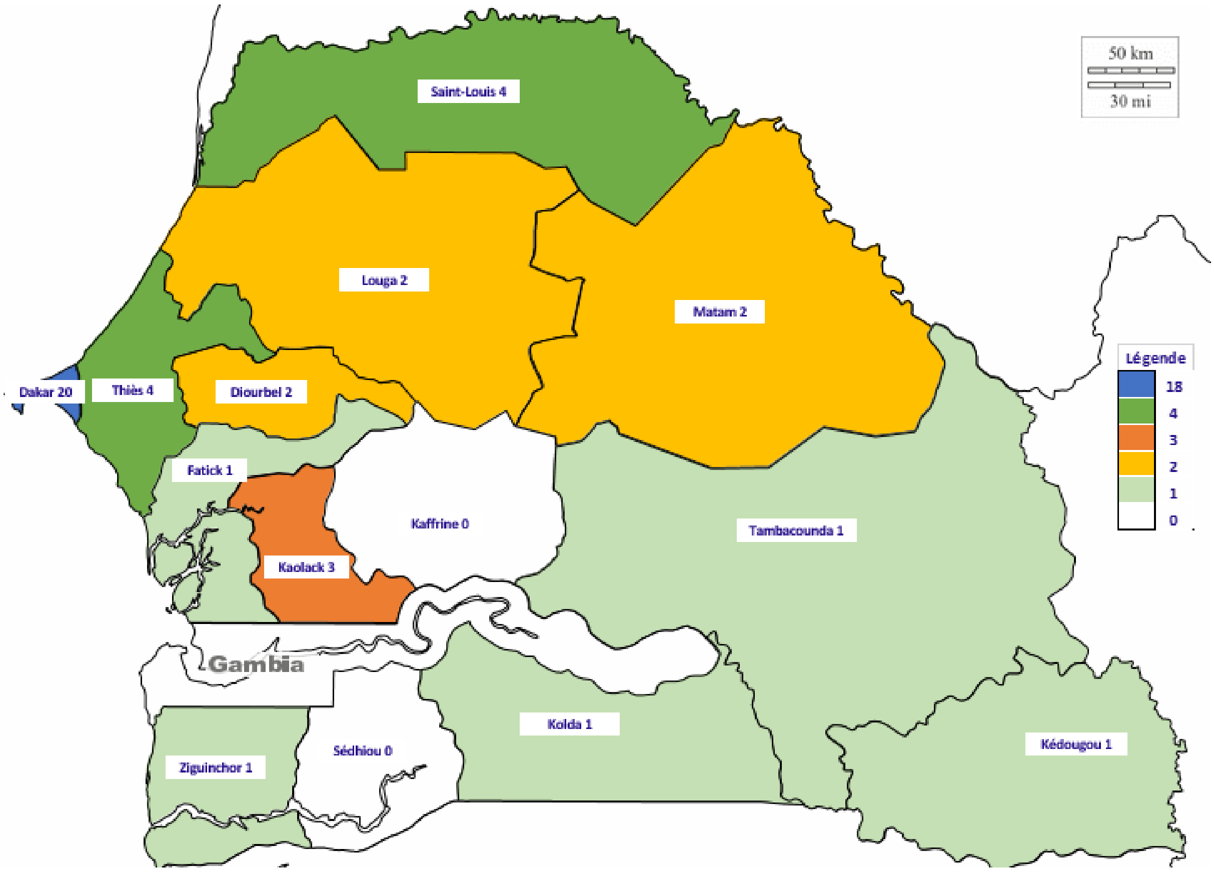

Source: Laboratories Department - MSAS

Table 3: List of medical laboratories that perform antibiotic susceptibility testing

N°	Region	District	Laboratory
1	DAKAR	CENTRE	Laboratory of the Centre for Diagnosis and Research in Molecular Medicine (CDRMM)
2			Medical Biology Laboratory ANALAB
3			Bacteriology Laboratory of the CHNU of Fann
4			Bacteriology Laboratory of the PES level 3 Albert Royer
5		DIAMNADIO	Laboratory of the EPS level 2 of Diamniadio
6		GUEDIAWAYE	King Baudouin Level 1 PES Laboratory
7			PHE Level 3 Laboratory DALAL DIAM
8		MBAO	Bacteriology laboratory of the EPS level 3 Psychiatric Hospital of Thiaroye
9			Laboratory of the EPS level 3 Pikine
10		NORTH	HOGGY Level 3 PES Laboratory
11		RUFISQUE	Laboratory of PES level 1 Youssou Mbargane Diop of Rufisque
12		SUD	Medical Biology Laboratory of the INSTITUT PASTEUR of Dakar
13			Medical Biology Laboratory BIO 24
14			ROKHAYA NIANG Medical Biology Laboratory
15			Medical Biology Laboratory SOLABSEN
16			Bacteriology Laboratory of the CAP Clinic
17			Bacteriology Laboratory of the CHU Aristide LEDANTEC
18			Bacteriology Laboratory of the CHU PRINCIPAL
19			IHS Level 1 PHE Laboratory (Medina Polyclinic)
20			PES Level 3 Laboratory ABASS NDAO
21	DIOURBEL	DIOURBEL	Heinrich Lubke PES Level 3 Laboratory
22		TOUBA	Level 3 PES Laboratory Matlaboul Fawzaini
23	FATICK	FATICK	PES level 3 laboratory Fatick
24	KAOLACK	KAOLACK	Medical Biology Laboratory BIOAMARIE
25			PES Level 2 Laboratory Elhadj Abdoulaye Niass
26			Kaolack Medical Region Laboratory
27	KEDOUGOU	KEDOUGOU	Laboratory of the Kédougou Health Centre
28	KOLDA	KOLDA	Laboratory of the PES level 2 of Kolda
29	LOUGA	LINGUERE	Level 1 PES Laboratory Magatte LO
30		LOUGA	Level 2 PES Laboratory Amadou Sakhir MBAYE
31	MATAM	MATAM	Laboratory of the PES level 2 of Matam
32			Laboratory of the EPS level 2 of Ourossogui
33	SAINT-LOUIS	PODOR	Laboratory of the PES level 2 of Ndioum
34		SAINT-LOUIS	BIONDAR Medical Biology Laboratory
35			St. Louis PHE Level 2 Laboratory
36			Saint Louis Medical Region Laboratory
37	TAMBACOUNDA	TAMBACOUNDA	Laboratory of the PES level 2 of Tambacounda
38	THIES	MBOUR	Level 1 PES laboratory in Mbour
39		THIES	Level 2 PES Laboratory St. John of God
40			Level 2 PES Laboratory Amadou Sakhir NDIEGUENE
41			National Public Health Laboratory
42	ZIGUINCHOR	ZIGUINCHOR	Laboratory of the PES level 2 of Ziguinchor

Source: Directorate of Laboratories - Ministry of Health and Social Action

3.1.2. Surveillance of priority diseases and diseases with epidemic potential

In Senegal, a surveillance system for diseases with epidemic potential and priority diseases based on data from medical biology laboratories has been set up by the National Network of Laboratories to monitor the profile of bacteria at risk.

Since November 2008, coinciding with the launch of the first issue of the National Laboratory Network's quarterly newsletter, laboratory data for the surveillance of diseases with epidemic potential and priority diseases are collected monthly and transmitted to the NLN.

The collection of the first laboratory data concerned the fourth quarter of 2008 and was disseminated on the February 2009 liaison bulletin and the collection of the last data dates back to September 2017 (National Laboratory Network Liaison Bulletin - NLN).

This surveillance began to be centralized in 2012 following the creation of the Directorate of Laboratories (Decree n0 2012-543 of 24 May 2012 fixing the distribution of State services according to Ministries), attached to the General Directorate of Health at the level of the Ministry of Health and Social Action.

The table below summarizes the compilation of data from medical laboratories from 2014 to 2017.

These data relate to the types of bacterial strains isolated from pathological products in the context of surveillance of priority diseases and diseases with epidemic potential.

Table 4: Surveillance results for priority diseases and diseases with epidemic potential 2014 to 2017

Diseases	Statistics	2014	2015[1] *	2016	2017[2] *
**MENINGITES**	Total sample	847	375	3695	579
	Number of pathogens	18	21	92	19
	Percentage of pathogens	2,1%	5,6%	2,5%	3,3%
**CHOLERA**	Total sample	855	1763	281	240
	Number of pathogens	0	0	0	0
	Percentage of pathogens	0,0%	0,0%	0,0%	0,0%
**SHIGELLOSES**	Total sample	853	1763	10158	992
	Number of pathogens	11	23	66	8
	Percentage of pathogens	1,3%	1,3%	0,6%	0,8%
**TUBERCULOSIS**	Total sample	2222	7508	51197	22218
	Number of pathogens	685	1366	6446	3243
	Percentage of pathogens	30,8%	18,2%	12,6%	14,6%
**MALARIA**	Total sample	5140	7035	74826	13796
	Number of pathogens	420	965	8708	1017

[1] For the year **2015**, the data collected covers two quarters **(January to June 2015)**
[2] For the year **2017**, the data collected covers two quarters **(January to June 2015)**

	Percentage of pathogens	8,2%	13,7%	11,6%	7,4%
**SHISTOSOMIASIS**	Total sample	4962	6556	51533	13661
	Number of pathogens	339	268	813	328
	Percentage of pathogens	6,8%	4,1%	1,6%	2,4%
**HIV**	Total sample	5078	10739	109153	43047
	Number of pathogens	177	473	3003	1314
	Percentage of pathogens	3,5%	4,4%	2,8%	3,1%

Source: Directorate of Laboratories - Ministry of Health and Social Action

Table 5: Type of Pathogens Isolated from Pathological Products

DISEASE	PATHOGENES	2014	2015	2016	2017
**MENINGITES**	N. meningitidis A	0	0	9	0
	N. meningitidis B	0	0	1	0
	N. meningitidis W135:	1	5	32	7
	S. pneumoniae	15	4	19	9
	H. influenzae	2	3	4	0
	Gram-positive diplococci (+)	0	7	15	2
	Gram-positive diplococci (-)	0	2	6	0
	Gram (-) polymorphic bacilli.	0	0	6	1
	**Total CSF samples**	**847**	**375**	**3695**	**579**
**CHOLERA**	V. cholerae O1:	0	0	0	0
	**Total stool samples**	**855**	**1763**	**281**	**240**
**SHIGELLOSES**	Shigella dysenteriae	0	0	2	1
	Other shigella	11	23	64	7
	**Total stool samples**	**853**	**1763**	**10158**	**992**
**TUBERCULOSIS**	Blades to + :	283	637	2547	1519
	Blades at ++.	183	385	1602	687
	Blades at +++.	219	344	2297	1037
	**Total samples**	**2222**	**7508**	**51197**	**22218**
**MALARIA**	Pl. falciparum	416	959	8452	1014
	Other Plasmodia	4	6	256	3
	**Total samples**	**5140**	**7035**	**74826**	**13796**
**STI**	Syphilis (Serology)	223	190	1499	1241
	Candida albicans	654	552	3230	1484
	N. gonorrhoeae	28	4	29	30
	Trichomonas vaginalis	103	65	269	128
	Chl. trachomatis (Direct)	104	35	331	190
	Chl. trachomatis (Serol)	68	99	518	124
**SHISTOSOMIASIS**	Sch. haematobium	311	250	749	299
	Sch. Mansoni	28	18	64	29
	**Total samples**	**4962**	**6556**	**51533**	**13661**

**HIV**	HIV-1	156	423	2648	1148
	HIV-2	19	33	276	110
	HIV-1+2	2	17	79	56
	**Total samples**	**5078**	**10739**	**109153**	**43047**

Source: Directorate of Laboratories - Ministry of Health and Social Action

3.1.3. Diagnosis of resistance profiles

As part of the Bacterial Antibiotic Resistance Surveillance Project, the Laboratories Directorate conducted a study of the prevalence of bacterial agents in pathological products and their antibiotic susceptibility patterns from January 1 to December 31, 2012.

The results of this study were compiled in a progress report developed on October 10, 2014.

- **Study population**

The sample studied consists of all bacterial species in pathogenic situations, isolated from diagnostic samples in medical laboratories.

- **Inclusion criteria**

All pathogenic bacterial species isolated from diagnostic samples in medical laboratories that have been formally identified and subjected to an antibiotic susceptibility test, regardless of the technique used, were taken into account.

- **Results**

The analysis of the collected data allowed to assess the prevalence of the different pathogenic species in each region and to have the status of resistance at the level of each pathological product.

The results were used to draw up a list of pathogens to be monitored, which is presented in the following table:

Table 6: The Bacteria Watch List

Bacteria causing diseases with epidemic potential	Neisseria miningitidis
	Vibrio cholerae
	Shigella spp
Bacteria of public health importance	Salmonella spp

	Acinetobacter spp
	Pseudomonas aeruginosa
	Streptococcus pneumoniae
	Hemophilus influenzae
Predominant bacteria	Escherichia coli
	Staphylococcus aureus
	Klebsiella spp
	Enterobacter spp

The majority of the strains (71.8%) were isolated in the Dakar region, followed by the Saint Louis region with 6.2% of the strains essentially isolated and the Thiès region with 5.2% of the strains isolated.

In addition, the study identified eighteen (18) resistance phenotypes including ESBL, PBN, SASM, PS, PHN, Pase, CHN, MRSA, Case, Casei, TRI, MLSb, and CBN from interpreted susceptibility test results.

Among the main phenotypes identified, ESBLs represent 24.8%.

Resistance phenotypes	Bacterial species	Percentage
ESBL	Klebsiella pneumoniae	36,9%
	E. coli	39,3%
	Enterobacter spp	11%
	Klebsiella oxytoca	4,9%
	Other species	11%
SASM	Staphylococci	2,9%

Finally, the results of the study were used to identify the main bacterial strains isolated.

Bacterial strains isolated	Bacterial species	Percentage
Enterobacteriaceae	Escherichia coli	54,8%
	Klebsiella pneumoniae	25,2%
	Enterobacter spp	8,31%
Staphylococci	Staphylococcus aureus	83,6%
	Staphylococcus saprophyticus	9,5%
Non-fermentative gram-negative bacilli	Pseudomonas aeruginosa	65%

	Acinetobacter spp	18,9%
Streptococci	Group B Streptococcus	28,6%
	Pneumococcus	13%
	Group D Streptococcus	

A first rapid situational analysis report was published in 2016 on the national antimicrobial resistance surveillance system based on laboratory data (***National Antibiotic Resistance Surveillance System: Year 1: Situational Report***)

3.1.4. Antimicrobial consumption profile

As part of the fight against AMR, Senegal conducted a pilot antimicrobial consumption surveillance survey in the Dakar region in 2018. The overall objective of the antimicrobial consumption profile survey is to obtain data on national antimicrobial consumption in Senegal over the period 2015-2017, in accordance with WHO methodology.

- **Methodology of the study**

The main steps in conducting this study were:

- Development of a national surveillance protocol for data collection

  This protocol consisted of developing a directory of antimicrobials. Thus, from the drug database of the Directorate of Pharmacy and Medicines, an exhaustive list of all antimicrobial drugs that have been granted marketing authorization was established.

- Training of investigators

  Several investigators from various backgrounds were trained in the use of the data collection form as well as the WHO Excel tool for surveillance of antimicrobial consumption

- Data collection in the field.

  Five (5) teams of three investigators, i.e. fifteen investigators in total (see attached list) were responsible for collecting consumption data from private drug importers, stock supply managers of public sector establishments and hospital pharmacies.

- **Establishments included in the monitoring**

For the private sector: these are the four private wholesale distributors, namely:

- LABOREX Senegal ;
- UBIPHARM Senegal ;

- SODIPHARM ;
- DUOPHARM ;

- **For the public sector**: from the NAP and the NTP.

The National Tuberculosis Control Programme was included in the study because purchases/supplies are made directly from it

- **For hospital structures:** Level 3 public health establishments located in Dakar were chosen. These are:

  - Grand-Yoff General Hospital (HOGGY);
  - Centre Hospitalier National de Fann ;
  - Abass Ndao National Hospital ;
  - Aristide le Dantec National University Hospital ;
  - Hôpital Principal de Dakar ;
  - National Hospital of Pikine

Table 7: Summary of structures involved in monitoring

ORDER NUMBER	HEALTH FACILITIES	NUMBERS
1	Approved drug importing establishments (private wholesalers)	04
2	National Procurement Pharmacy (NPP)	01
3	National Tuberculosis Control Programme (NTP)	01
4	National Hospitals (CHN)	06

Source: Antimicrobial Consumption Profile Survey Mission Summary Report: Directorate of Pharmacy and Medicines: May 2018

- **Evaluation criteria:**

Antimicrobial consumption is estimated through the supply of antimicrobial drugs to pharmacies by the private sector; and for the public sector, through the supply and distribution of drugs at the level of public health facilities by the ANP and the distribution of anti-tuberculosis drugs by the NTP for the management of tuberculosis. For the public sector, consumption was evaluated using data from the internal pharmacy of the targeted hospitals.

- **Antimicrobials include**

Eight classes of systemic antimicrobials described in the table below and identified by ATC code were included in the surveillance. Topical antimicrobials were excluded:

Table 8: List of antimicrobials in the study

ANTIMICROBIALS	ATC CODE
Antibacterial	J01
Antibiotics for the digestive system	A07AA
Nitroimidazole derivatives against protozoan diseases	P01AB
Antifungals	D01BA
Antimycotics	J02
Anti-tuberculosis drugs	J04A
Antivirals	J05
Anti-malarial drugs	P01B

Source: Antimicrobial Consumption Profile Survey Mission Summary Report: Directorate of Pharmacy and Medicines: May 2018

- **Data collection tools**

A data collection form was developed following the WHO model with simplified headings for data on antimicrobial consumption (in annex). This form allowed the following information to be filled in:

- Product data
- Consumption data
- Data on the antimicrobial substance

Results

- **Data by sector**

The data below shows the number of global references used at the country level between 2015 and 2017. Unfortunately not all data was available at the same time, so one of the private wholesalers did not make their consumption data available.

Table 9: Number of pharmaceutical references by data source

STRUCTURES	NUMBER OF REFERENCES	YEARS OF CONSUMPTION
**National Supply Pharmacy**	**116**	**2016,2017**
**National Tuberculosis Control Programme**	**15**	**2015, 2016, 2017**
**Laborex**	**251**	**2015, 2016, 2017**
**Ubipharm Senegal**	**198**	**2015, 2016, 2017**

Sodipharm	217	2016,2017
Grand Yoff General Hospital	38	2015, 2016, 2017
Dakar Main Hospital	44	2015, 2016, 2017
Dantec Hospital	39	2015, 2016, 2017
Fann Hospital (Central Pharmacy, CTA, Cuomo, Crcf)	111	2015, 2016, 2017
Albert Royer Children's Hospital	16	2015, 2016, 2017
Order of Malta	29	2015, 2016, 2017
Abass Ndao Hospital	28	2015, 2016, 2017
National Hospital of Pikine	41	2015, 2016, 2017
GLOBAL	549	2015, 2016, 2017

Chart 2: Distribution by sector

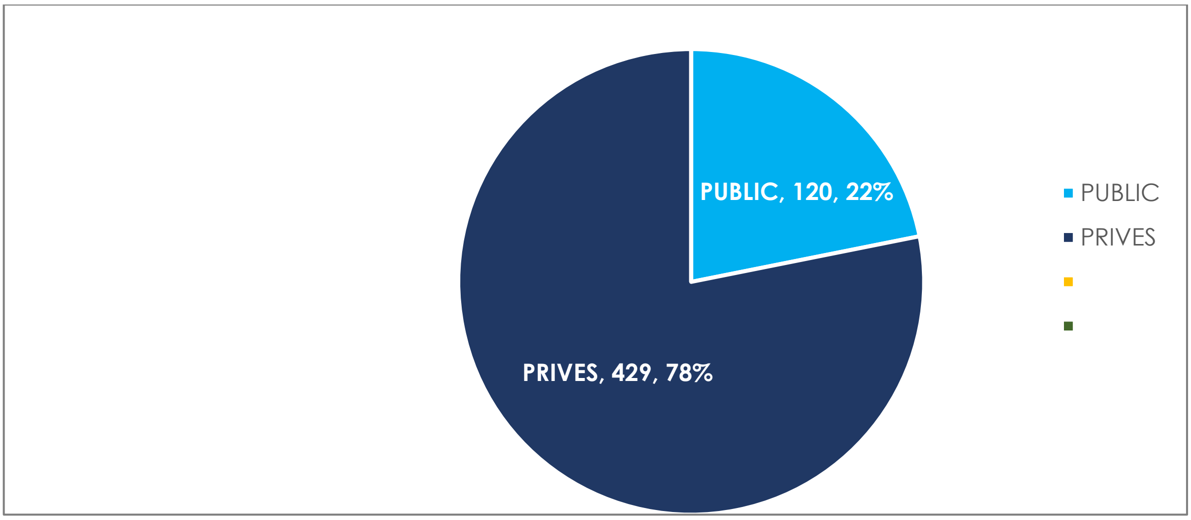

Source: Antimicrobial Consumption Profile Survey Mission Summary Report: Directorate of Pharmacy and Medicines: May 2018

Nearly 549 references were distributed between 1er January 2015 and 31 December 2017, all sectors combined (see Table 8). 78% (about 429) of the antimicrobials are distributed by private wholesalers and the remaining 22% by the National Supply Pharmacy (PNA) and the National Tuberculosis Control Program (PNT) (See chart 2).

At the hospital level, pharmacies are supplied by the National Supply Pharmacy (PNA) for most antibacterial drugs. On the other hand, wholesale distributors supply them with specialties that are not available at the NPP.

The distribution of antimicrobial and TB drug consumption by hospital (community level) gives us the following profile:

Chart 3: Distribution by structure

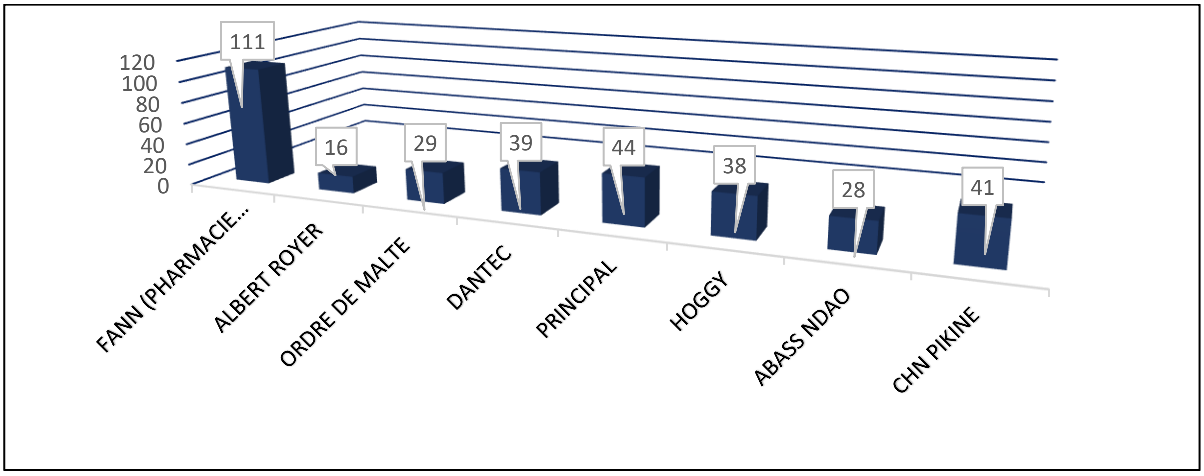

Source: Antimicrobial Consumption Profile Survey Mission Summary Report: Directorate of Pharmacy and Medicines: May 2018

- **Data by therapeutic class**

From the results obtained, a consumption profile of the different therapeutic classes could be determined. Antibacterials for systemic use are among the most consumed molecules according to the results collected from the collection sites and during the period 2015-2017.

Figure 4: Distribution of references by ATC code

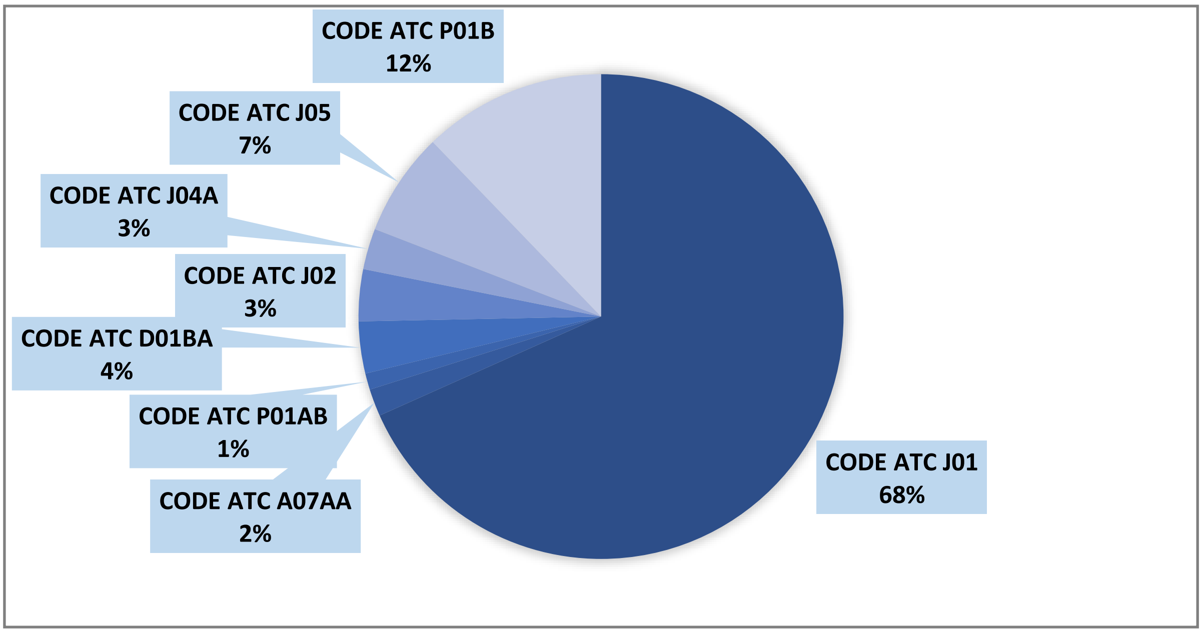

Source: Antimicrobial Consumption Profile Survey Mission Summary Report: Directorate of Pharmacy and Medicines: May 2018

Antibacterials for systemic use (ATC code J01) alone account for 68% of references. Antimalarials account for 12% of references. These two classes account for a total of 80% (see graph 4).

Antibiotics for the digestive system (ATC code A07AA), as well as Nitroimidazole derivatives for protozoal diseases (ATC code P01AB) account for only 3% of the references.

Antifungals (ATC code D01BA), antimycotics (ATC code J02) and antituberculosis drugs (ATC code J04A) each account for 3% of references. Antivirals (ATC code J05) account for 7% of these same references.

All of this data was collected during the period 2015-2017.

Reference distribution of the class of antibacterials for systemic use ATC code J01.

After analysis of antibacterials for systemic use ATC code J01, this is what we get:

Figure 5; Distribution of references in the antibacterial class ATC code J01

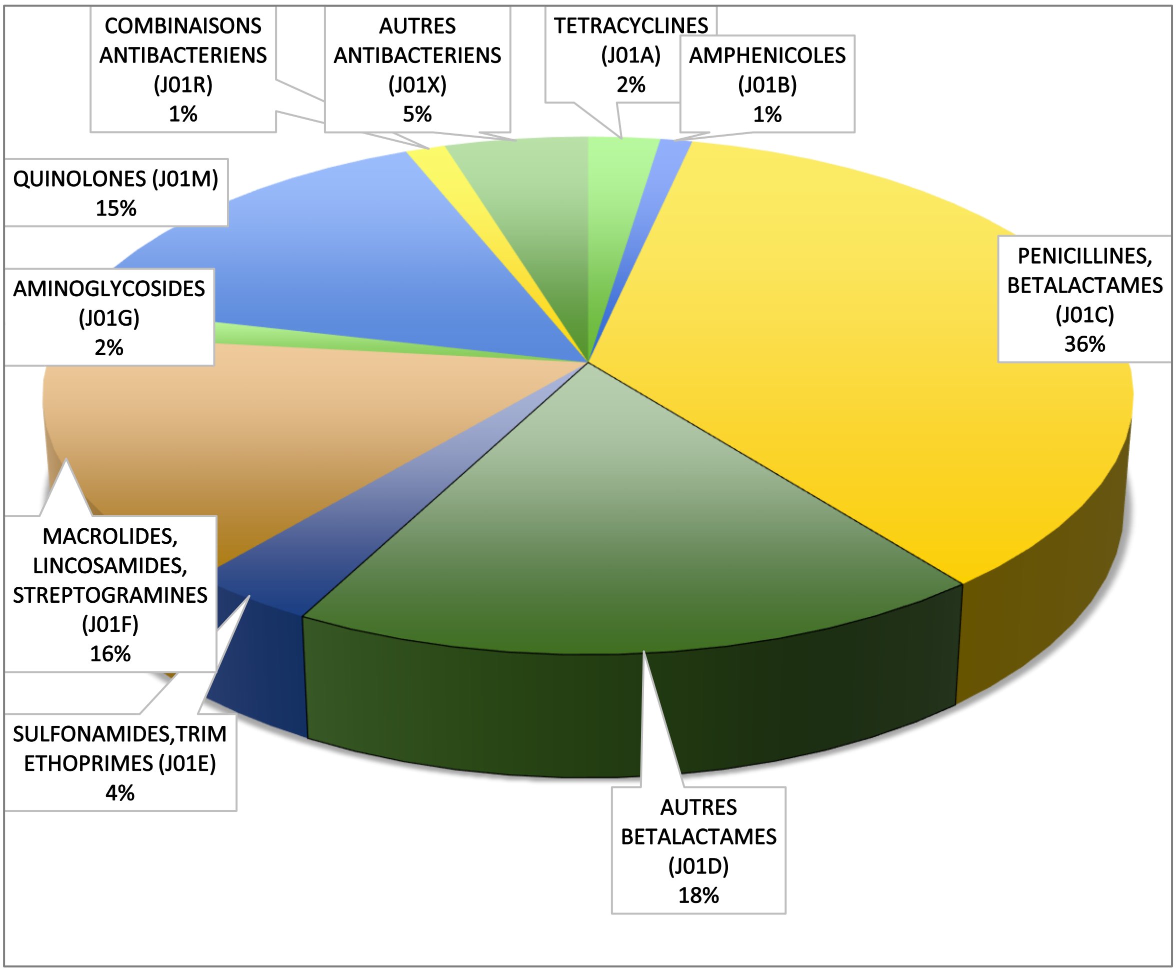

Source: Antimicrobial Consumption Profile Survey Mission Summary Report: Directorate of Pharmacy and Medicines: May 2018

Data collected over the period 2015 to 2017 show that J01C penicillins account for 36% of the antibiotic class, which places them at the top of human health antibiotic consumption. They are followed by the cephalosporins, monobactams and penems (J01D) class with 18%. The group of macrolides, lincosamides and stretogramins (J01F) is in third place with 16% of antibiotic references.

The other groups aminoglycosides (J01G), tetracyclines (J01A), amphenicoles (J01B), sulfonamides/trimetoprimes (J01E) and combinations (J01R and J01X) constitute 15% of the antibacterial class.

ANTIBACTERIALS	ATC CODE J01	NUMBER OF REFERENCES
TETRACYCLINES	J01A	9
AMPHENICOLES	J01B	4
PENICILLINS AND BETALACTAMS	J01C	134
OTHER BETA-LACTAM ANTIBACTERIALS	J01D	69
SULFONAMIDES AND TRIMETHOPRIMES	J01E	13
MACROLIDES, LINCOSAMIDES, STRPTOGRAMS	J01F	58
AMINOGLYCOSIDES	J01G	7
QUINOLONES	J01M	57
COMBINATIONS OF ANTIBACTERIALS	J01R	5
OTHER ANTIBACTERIALS	J01X	18

Source: Antimicrobial Consumption Profile Survey Mission Summary Report: Directorate of Pharmacy and Medicines: May 2018

- **Distribution of Penicillins and Beta lactams ATC code J01C:**

In Dakar, Amoxicillin accounts for 30.6% of antibiotic consumption, Amoxicillin-Clavulanic acid combination 43.28% and Beta lactam resistant penicillins 13.43%. These are Flucloxacillin, Cloxacillin and Oxacillin.

The share of Beta-lactam sensitive penicillins (Phenoxymethylpenicillin, Benzylpenicillin and Benzathine benzylpenicillin) represents 8.9%. The remaining 3.79% is divided between Ampicillin and Penicillin combinations.

Figure 6: Distribution of penicillins and beta-lactams ATC code J01C

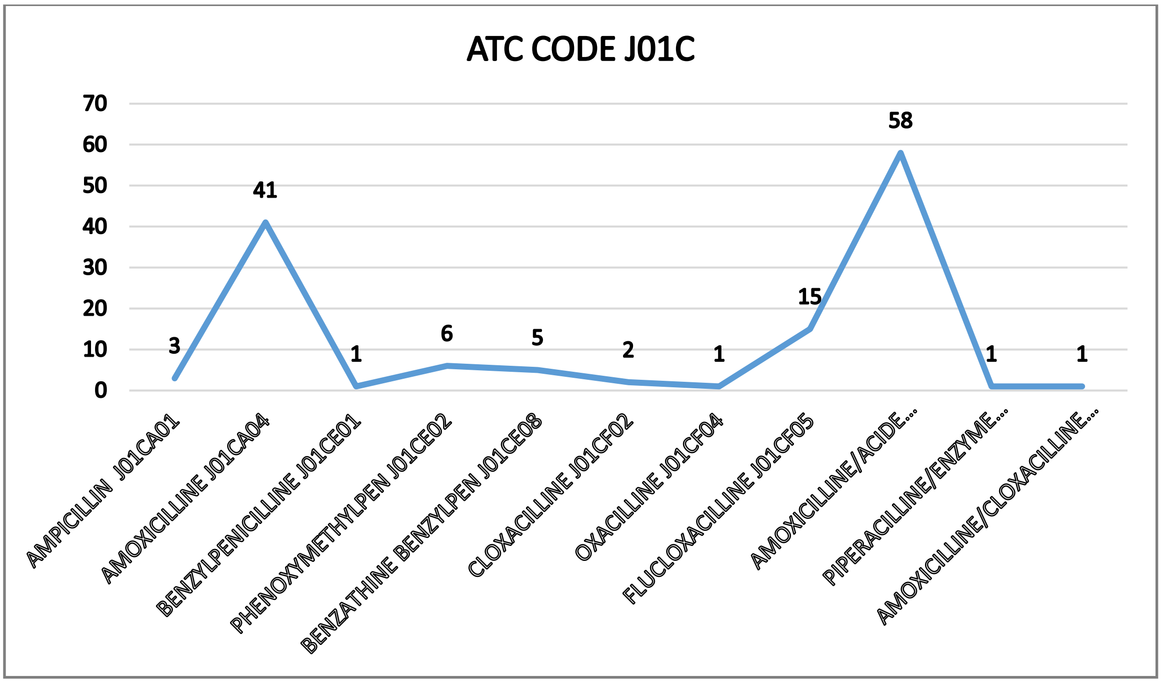

- **Distribution of other antibacterials Beta lactams ATC code J0D**

Figure 7: Distribution of other beta-lactams ATC code J01D

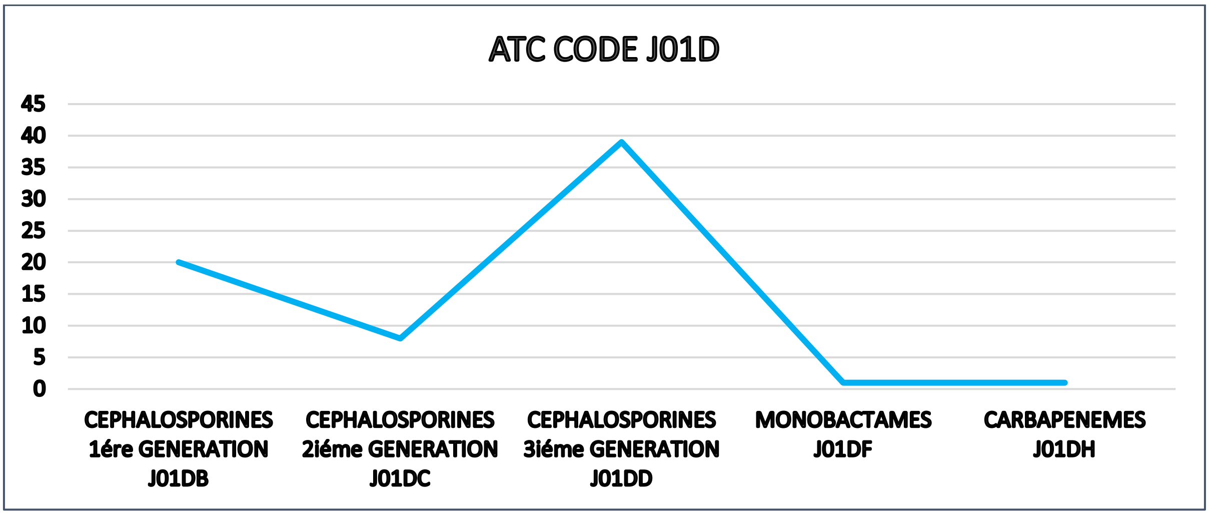

Source: Antimicrobial Consumption Profile Survey Mission Summary Report: Directorate of Pharmacy and Medicines: May 2018

The graph above shows the consumption of cephalosporins by the public and private wholesalers. First, second and third generation cephalosporins represent 28.98%, 11.59% and 56.52% respectively.

Monobactams and Carbapenems each represent 1.45%.

Distribution of Macrolides, Lincosamides and Streptogramins ATC code J0F

Figure 8: Distribution of macrolides ATC code J01F

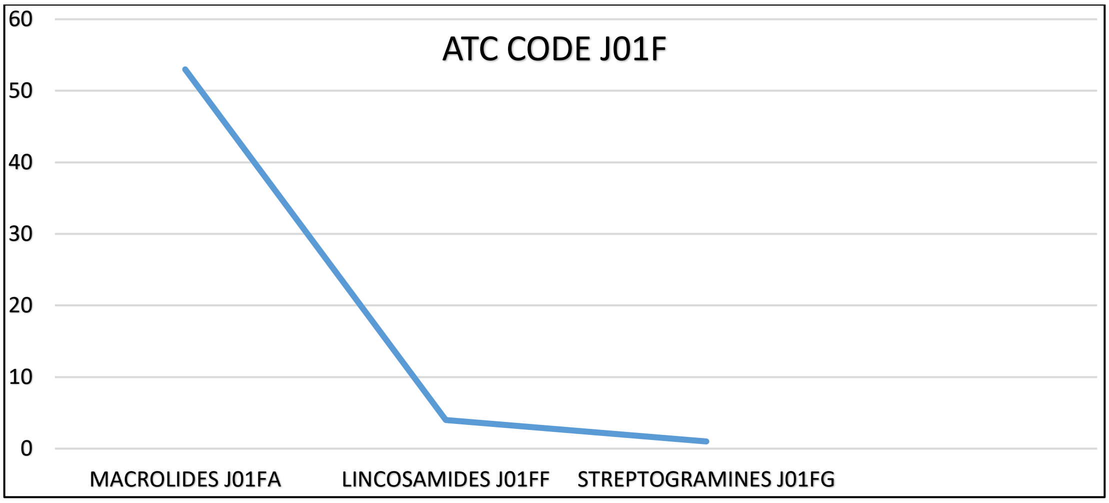

Macrolides J01FA represent 91.38% of this class against 8.62% totalled by Lincosamides (and Streptogramins).

Among these macrolides, the Azithromycin group (ATC code J01FA10) is the most used in terms of consumption with 56.6%. The Erythromycins (J01FA01) and Spiramycins (J01FA02) groups represent 13.2% and 11.32% of macrolides respectively.

Distribution of Quinolones ATC code J01M

The only Quinolones available on the market are represented by the Fluoroquinolones ATC code J01MA whose distribution is the following:

Figure 9: Distribution of Fluoroquinolones J01MA

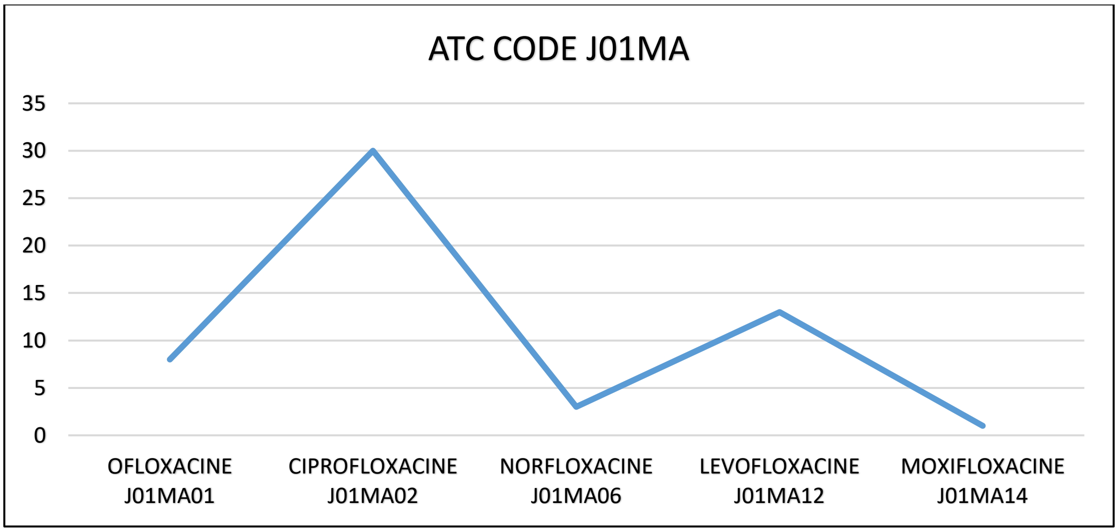

Source: Antimicrobial Consumption Profile Survey Mission Summary Report: Directorate of Pharmacy and Medicines: May 2018

Ciprofloxacins are the most consumed Fluoroquinolones with 54.54%, followed by Levofloxacins with 23.63%, Ofloxacins with 14.54%. Norfloxacin and Moxifloxacin account for 7.27% of the consumption of this class.

- **Distribution of references in the class of antimalarial drugs ATC code P01B**

Graph 10; Distribution of references in the antimalarial class

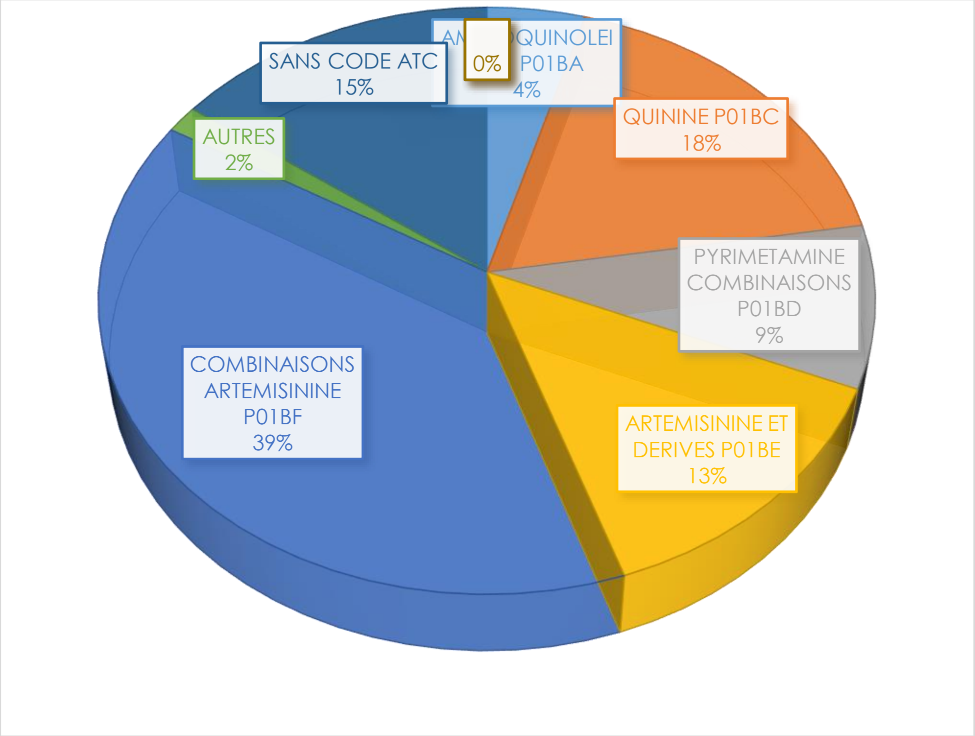

Source: Antimicrobial Consumption Profile Survey Mission Summary Report: Directorate of Pharmacy and Medicines: May 2018

In terms of antimalarials, Artemisinin-based combination therapies (P01BF) account for 39% (26/67) of the references in this class, with 73.07% (19/26) of Artemether+Lumefantrine combinations (P01BF01) and 15.38% (4/26) of Artesunate+Amodiaquine combinations. Artemisinins (P01BE) represent 13% (9/67) with Artesunate consumption estimated at 44.4% (4/9).

Quinine (P01BC) is still well consumed, especially in the public sector with 18% (12/67).

- **Antimicrobial consumption**

The volume of antimicrobials distributed by public and private wholesalers to health facilities (hospital pharmacies and pharmacy dispensaries) from 2015 to 2017 shows the consumption expressed in volume units of all pharmaceutical forms combined:

Graph 11: Distribution of references by volume units and by year

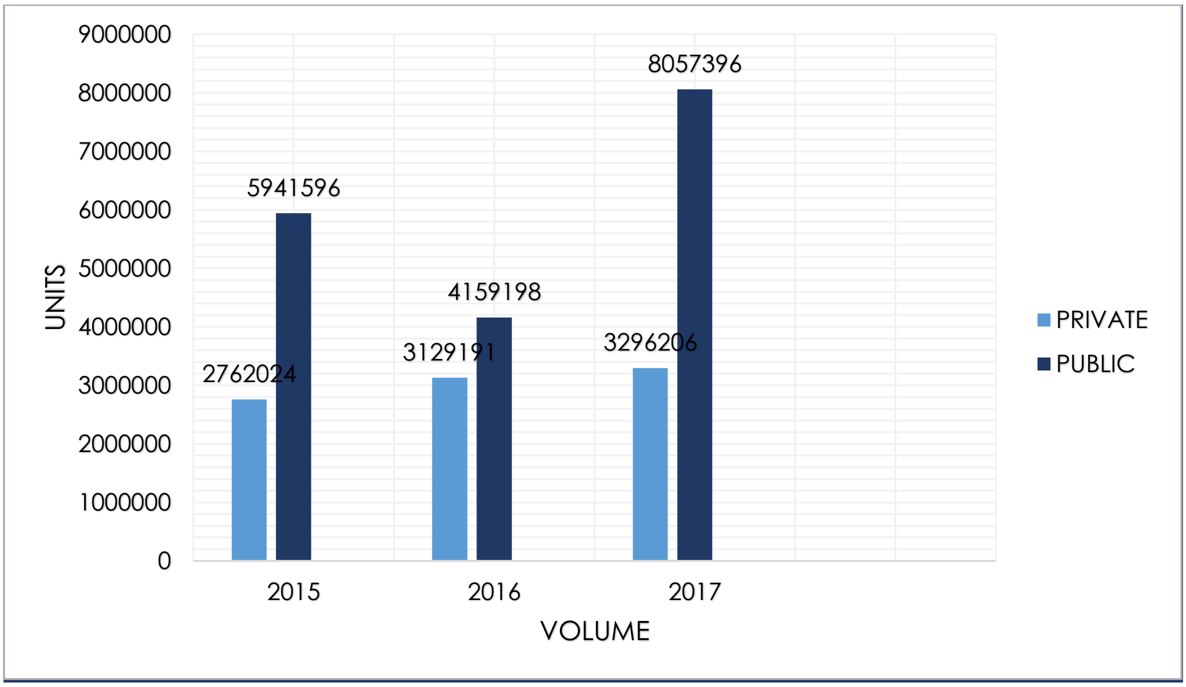

Source: Antimicrobial Consumption Profile Survey Mission Summary Report: Directorate of Pharmacy and Medicines: May 2018

Unfortunately, of the four private wholesale distributors identified in Senegal, consumption data were collected from three wholesalers, namely UBIPHARM, LABOREX and SODIPHARM. And out of the three wholesalers, two provided consumption data for 2015.

8703620 units of antimicrobials were distributed in 2015, 7288389 in 2016 and 11353602 in 2017.

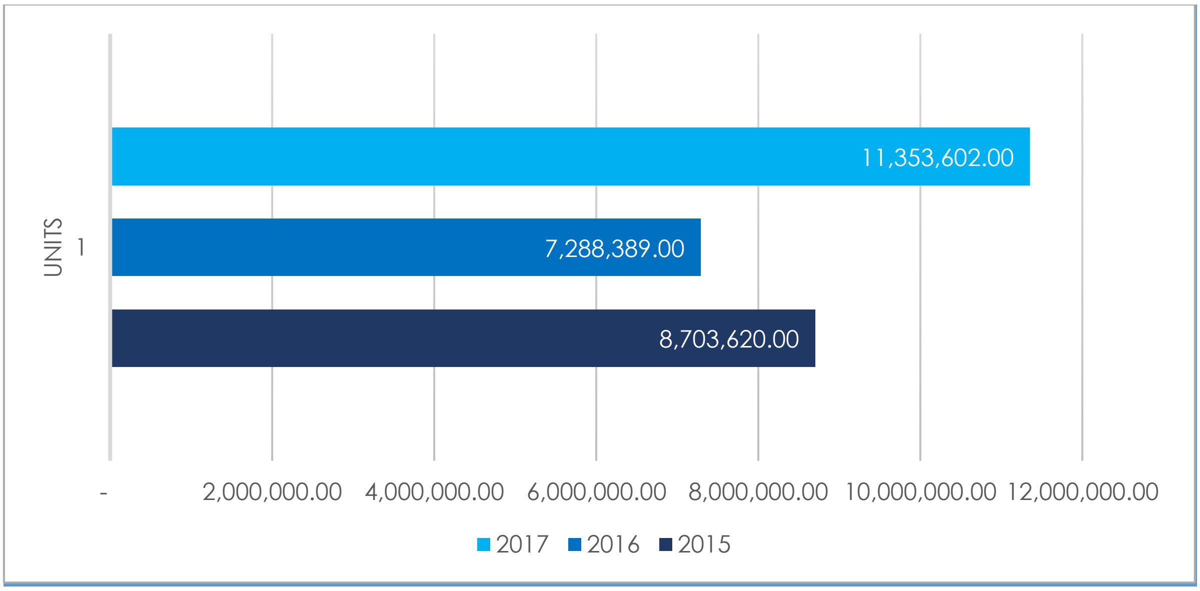

Findings

- The management of medicines in general, and of antimicrobials in particular, is not well ensured (lack of traceability, absence of management software for some, poorly completed stock sheets, etc.);
- Many of the data collected are incomplete (data for some periods are not available);
- Some information about the drug (dosage, packaging...) is not available;
- Delay in the transmission of electronic data which is one of the causes of the delay in the delivery of results;
- The electronic data is not well sorted (all routes of administration were given when only oral and parenteral routes are concerned by this collection);
- Staff working conditions must be improved for good antimicrobial stewardship (adapted premises, logistics...).

Recommendations :

This was a pilot phase in the Dakar region, and other surveys are planned within the framework of AMR. Thus, in the future, with the aim of continuous improvement of antimicrobial collection surveys, it is necessary to :

- Better coordination between DMP and structures and timely delivery of mail;
- Motivation of the actors involved;
- RU training for health professionals in antimicrobial stewardship;
- Management of medicines at hospital level to be improved and centralized ;
- Computerization of drug inventory management for better traceability and visibility;
- Tightening of regulations on the prescription and dispensing of antibiotics

3.2. STATUS OF THE RAM IN ANIMAL HEALTH

3.2.1. Mapping of actors and their roles

The main actors are the laboratory of Microbiology, Immunology and Infectious Pathology (MIPI) of the EISMV in Dakar and more particularly the National Laboratory of Livestock and Veterinary Research of ISRA.

In addition to their roles in research and development and teaching, these laboratories are generally solicited for microbiological analyses by various applicants such as :

- The Directorate of Veterinary Services (DSV),
- Private vets and para vets,
- Poultry and dairy farm managers,
- Hatcheries
- Poultry farmers
- Livestock projects and programmes...

3.2.2. Epidemiological surveillance of animal diseases

Epidemiological surveillance targets the main diseases, including rinderpest, contagious bovine pleuropneumonia, peste des petits ruminants, pasteurellosis (bovine and small ruminants), African horse sickness, Newcastle disease, avian influenza, Rift Valley fever, contagious lumpy skin disease, foot-and-mouth disease, African swine fever and rabies. These diseases are monitored under the National Animal Disease Surveillance System (NADSS).

The overall objective of the NESS is to safeguard animal and public health and to adapt animal disease control to the new context of trade globalisation and the risks associated with trade in animals and animal products. Its specific objectives are:

- to detect any outbreak of animal diseases, including zoonoses;
- to determine the real importance of the diseases monitored in livestock and wildlife by studying their prevalence and economic impact;
- evaluate the results of animal disease control plans;
- to provide the Veterinary Services with epidemiological information to assist in decision-making on animal disease control (Source: DSV/MEPA).

3.2.3. The annual livestock vaccination campaign

The Senegalese government, through the MEPA, organizes an annual subsidized livestock vaccination campaign. This campaign is carried out in the framework of a public-private partnership with the participation of veterinary surgeons (through the execution of the health mandate). It targets five priority diseases:

- peste des petits ruminants (PPR);
- African horse sickness (AHS);
- Newcastle disease (ND);
- Lumpy skin disease in cattle (LSD)

- contagious bovine pleuropneumonia (CBPP).

Apart from these five diseases, other targeted vaccinations carried out on the basis of the realities of the country's different agro-ecological zones include haemorrhagic septicaemia, pasteurellosis of small ruminants, botulism, symptomatic anthrax, anthrax, foot-and-mouth disease, enterotoxaemia, Rift Valley fever, sheep pox, swine pasteurellosis and rabies, in order to improve the health coverage of the herd (Source: DSV/MEPA).

3.2.4. Antimicrobial resistance patterns in animal health

We were not able to obtain data from the LNERV and the MIPI laboratory of EISMV, the main actors of microbiological studies in animal health. However, our bibliographic research allowed us to synthesize the following information concerning AMR profiles in animal health in Senegal:

- Identification of a new multi-resistant salmonella in broiler meat in Senegal in 2000. The salmonella strain was identified from a muscle sample. The susceptibility test showed resistance to most of the antibiotics commonly used in human medicine (ampicillin, tobramycin, gentamicin, sulfamethoxazole-trimethoprim, tetracycline, chloramphenicole), except quinolones. The strain also exhibited extended spectrum beta-lactamase (ESBL). Molecular analysis showed that the strain harboured two resistance genes (source: Cardinale E. et al. REMVT, 2000, 53 (1), 5-8).

- Epidemiological analysis of Salmonella enterica ssp. enterica serovars Hadar, Brancaster and Enteritidis from humans and broilers in Senegal: out of 142 salmonellae isolated, 79 from Senegalese patients with sporadic diarrhoea and 63 from poultry, antibiosensitivity testing showed multidrug resistance (to more than two drugs) in 14.5% of Salmonella Hadar isolates and in 5% of Salmonella Enteritidis isolates Resistance to quinolones was considered of particular importance: 14.5% of Salmonella Hadar isolates were resistant to nalidixic acid. The similarity of profiles between human and poultry isolates provided indirect evidence of transmission of Salmonella from contaminated poultry meat. However, most Salmonella isolates remained drug-susceptible (source: Cardinale E. et al., Journal of Applied Microbiology, 2005;99(4):968-977).

- Comparative study between urban effluents representing the population of E. coli of human origin and slaughterhouse effluents representing the population of E. coli of animal origin: the study showed a resistance to at least one tested antibiotic of 17.5% of the isolates of E. coli of animal origin (cattle) against 57.5% for the isolates of human origin (source: DIALLO A. A. et al, African Journal of Microbiology Research 2017 July: 11(25):1035-1042

- Predominance and antimicrobial resistance of Salmonella isolated from broiler farms, chicken carcasses and street restaurants in Casamance, Senegal: This study was undertaken to determine the predominance and distribution of Salmonella in

57 randomly selected broiler farms, towards the end of the rearing period, and in chicken products in urban and periurban areas in Casamance, Senegal, and to assess the antimicrobial resistance patterns of Salmonella serovars. High levels of resistance were found to sulfamethoxazole-trimethoprim (75.9%), tetracycline (74.7%), trimethoprim (76.2%), streptomycin (73.9%) and sulfonamides (72%). All Salmonella serovars were susceptible to fluoroquinolones and third-generation cephalosporins. A large proportion of isolates belonging to 11 serovars were resistant to two or more antibiotics (source: DIONE M. M. et al., Journal of Food Protection (2009) 72 (11): 2423-2427.

- Antimicrobial resistance profile of Salmonella isolates from chicken carcasses in Dakar, Senegal: Between July 2012 and July 2013, three types of chicken carcasses (chaitr chickens, layers, and local chickens) obtained from retailers in the markets of Dakar and its suburbs were examined. Salmonella was isolated from 300 chicken carcasses. In these samples, 273 isolates were obtained, belonging to 22 serovars. The three most frequently identified serovars were Salmonella Istanbul, Salmonella Brancaster and Salmonella Kentucky. Overall, 21% of isolates were resistant to quinolones and fluoroquinolones. Serovar Istanbul was resistant to tetracycline (TE) and sulfamethoxazole-trimethoprim (SXT). Serovars Brancaster and Kentucky were resistant to betalactams and quinolones or fluoroquinolones. The rare serovar Senftenberg had the strongest resistance pattern, showing resistance to betalactams including imipenem (IMP). A large number of isolates were resistant to TE (66%) and SXT (47%). Resistance to cephalosporins (5%), chloramphenicol (2%), gentamicin (8%) and IMP (1%) was less frequent (source: FALL-NIANG N. K. et al, Foodborne Pathogens and Disease (2019) Vol. 16, No. 2

- **Findings**
  - Antimicrobial resistance in animal health, particularly in poultry, dates back a few decades and has a growing trend
  - The transmission of resistant germs via human food is a proven fact
- **Recommendations**
  - Regulate the health monitoring of livestock farms to reduce the use of antimicrobials.

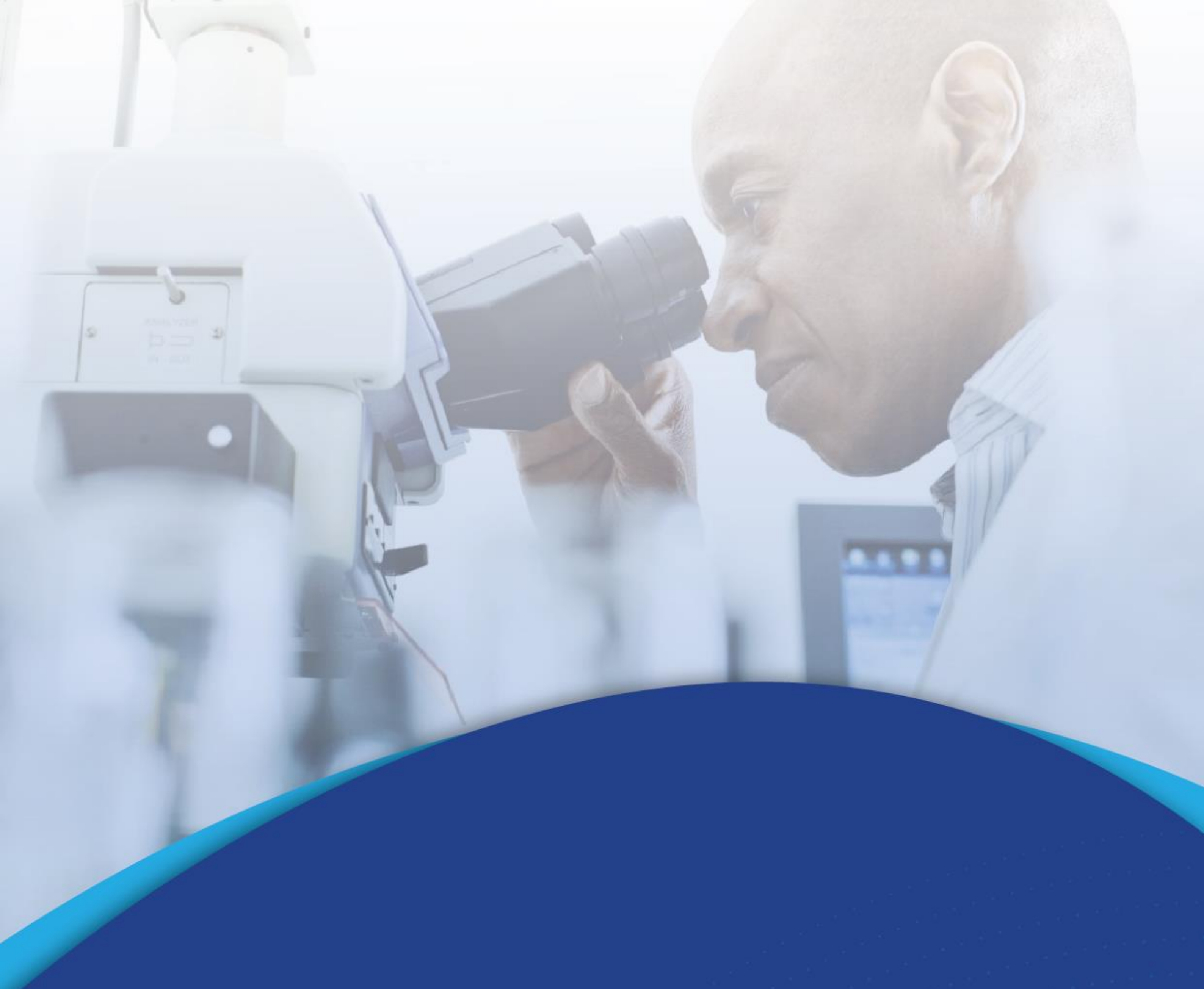

ANALYSE SITUATIONNELLE DU CADRE REGLEMENTAIRE DES ANTIMICROBIENS

4. REGULATORY ANALYSIS

4.1. Mapping of actors and their roles

4.1.1. Mapping of actors and their roles in human health

The institutional players in the pharmaceutical sector are as follows:

Directorate of Pharmacy and Medicines

The Directorate of Pharmacy and Medicines was created as the National Authority for Pharmaceutical Regulation by Decree No.⁰ 2004-1404 of November 4, 2004, organizing the Ministry of Health and Medical Prevention.

It is responsible, by the Ministry of Health and Social Action, for implementing the following regulatory missions:

- the licensing of establishments for the manufacture, distribution and importation of drugs
- the registration of medicinal products for the purpose of issuing marketing authorisations ;
- control of drug imports
- pharmaceutical inspection ;
- Pharmacovigilance ;
- monitoring of clinical trials ;
- control of the promotion and advertising of medicinal products ;
- pharmaceutical information management and rational use of medicines.
- the development, implementation and monitoring of policy and programmes in the field of pharmacy and medicines
- the development and monitoring of the application of legislative and regulatory texts relating to pharmacy, medicines and medical devices.

The Laboratories Department

Created in 2012, the Laboratories Department is responsible for developing, implementing and monitoring policy and programs in the field of medical biology.

In this capacity, it is responsible for drawing up and ensuring the application of legislative and regulatory texts relating to medical biology laboratories, reagents and other laboratory products.

It is responsible, in particular, for :

- the promotion and control of public and private medical laboratories;
- authorization for the import of reagents ;
- supervision of all public and private medical laboratories;

- the organisation of the national system of external evaluation of the quality of laboratory analyses, in relation to disease control programmes and reference laboratories;
- support and guidance for laboratories in their quality approach.

The Poison Control Centre

The Poison Control Centre (CAP) was established by Decree No. 2004-1404 of 4 November 2004 on the organization of the Ministry of Health and Medical Prevention. Its mission is to ensure the prevention and management of intoxications and diseases caused by food and xenobiotics, including health products, pesticides, household products, industrial products and plants. It is also responsible for :

- the follow-up of intoxication cases recorded within the framework of toxicovigilance;
- to make the accountability of adverse reactions due to health products.

The National Procurement Pharmacy (NPP)

The ANP, created in 1954 to meet the medico-pharmaceutical needs of French West Africa (AOF), became a public service attached to the Ministry of Health after independence and then a non-hospital public health establishment according to law 98-12 of March 2, 1998 on hospital reform.

The mission of the ANP is to ensure the availability, financial and geographical accessibility of quality essential medicines and products to the population. The ANP is the wholesale distributor for the public and parapublic sector. It has 11 fixed Regional Supply Pharmacies (PRA) and 3 mobile PRA.

Private wholesale distributors of medicines

By virtue of their mission, private wholesale distributors act as an interface between pharmaceutical laboratories and pharmacies. Through their daily deliveries to pharmacies, they guarantee the accessibility and availability of medicines to all, under optimal safety conditions.

They buy from pharmaceutical laboratories, manage and store in their establishments throughout the country, deliver and make available to pharmacies references of medicines with a marketing authorisation and, in rare cases, specialities without a visa.

In Senegal, the commercial distribution system is extremely efficient in ensuring the timely delivery of high quality products. The DPM has authorized ***four (4)*** wholesale distributors to work in the country. These are ***LABOREX, UBIPHARM*** (formerly Cophase), ***SODIPHARM*** and ***DUOPHARM.*** They supply the private commercial pharmaceutical sector, particularly pharmacies.

Drug Manufacturing Establishments ;

There are ***five (5)*** local drug manufacturing units authorized by the DPM. They are: *CANONNE SA (VALDAFRIQUE), WEST AFRIC PHARMA, MEDIS, INSTITUT PASTEUR* which produces the yellow fever vaccine under the control of the DPM and the LNCM and *PARENTERUS* which has recently entered the market.

Despite the creation of production units (*SOCAFI PHARMA and TERANGA PHARMA*), the industrial fabric remains weak. Senegal imports 85 to 90 per cent of its drug needs.

- **Drug Dispensing Structures**

Approval for dispensing facilities is granted by the Ministry of Health, on the proposal of the Directorate of Pharmacy and Medicines, after consulting the Senegalese Order of Pharmacists.

According to the WHO report, Senegal has 1186 pharmacies in 2018. However, they are heavily concentrated in the most urban areas. Indeed, the Dakar region alone is home to 604 pharmacies in the country, or 51 percent of the total. It is followed respectively by the regions of Thiès (152), Diourbel (75), Saint Louis (69) and Kaolack (60).

- **The Order of Pharmacists of Senegal**

The Order of Pharmacists of Senegal was established by Law No. 73-62 of 19 December 1973. It is a public institution of a professional nature, with civil status and financial autonomy. It is headed by a National Council of the Order of Pharmacists, whose headquarters are in Dakar.

The purpose of the Order of Pharmacists is to ensure that professional duties are respected and to defend the honour and independence of the profession. To this end, it has a code of ethics and jurisdictional powers (disciplinary chambers).

The Order has, among other missions :

- the regulation of the profession by the registration of pharmacists practising their art in Senegal in the roll of the Order, with the exception of pharmacists belonging to the active health service of the Senegalese Army and foreign military pharmacists serving under military assistance;
- representation of the profession before administrative and judicial authorities and the community.

4.1.2. Mapping of actors and their roles in animal health

The Senegalese State has accompanied the development of the privatisation process of the veterinary profession with :

- Law n° 92-52 of 10 July 1992 creating the Order of Veterinary Doctors of Senegal,
- Decree No. 93-514 of 27 April 1993 on the Code of Ethics for Veterinary Medicine,

- Decree No. 95-645 of 6 July 1995 instituting the health mandate granted to private veterinarians who apply for it and intended for the execution of collective prophylaxis operations against certain animal diseases,
- Law 2008-07 of 24 January 2008 organizing the veterinary profession and pharmacy.

The various players in the animal health sector are as follows:

The Directorate of Veterinary Services (DSV)

It was created in June 2008, following the restructuring of the Ministry of Livestock (Senegal, MEPA, DSV Annual Report 2015). It is responsible in particular for:

- zoo sanitary protection through the implementation of the national system of epidemiological surveillance, medical and sanitary prophylaxis and animal disease control;
- the development and implementation of animal health and veterinary public health strategies;
- the elaboration and application of regulations in the field of veterinary profession and pharmacy, in the field of food safety of animal origin and, in relation with the Livestock Directorate, of the quality of food intended for animals;
- liaison with national, regional and global specialist bodies in the field of animal health and veterinary public health;
- the management of import and veterinary certification for export of animals and animal products (seeds, eggs, meat, embryos, etc.).

The Order of Veterinary Doctors of Senegal (ODVS) :

The order was created by law 92-52 of 10 July 1992, the missions of the ODVS are :

- the guarantee of professional competence and morality of the veterinary doctors registered in the roll of the Order with respect to the public authorities and private clients;
- the defence of the practice of veterinary medicine against any falsification or abuse;
- fixing the fees for veterinary consultations, care and interventions ;
- the arbitration of conflicts of a professional nature which arise between members registered on the roll of the Order or between the latter and their clients insofar as these conflicts are directly related to the practice of veterinary medicine;
- the application of the veterinary code of ethics ;
- the formulation of opinions to the public authorities on all matters of national interest relating to the practice of veterinary medicine.

The National Laboratory for Livestock and Veterinary Research (LNERV)

Housed within ISRA (Institut sénégalais de Recherches agricoles), although dependent on the Ministry of Agriculture, is involved in the animal health system through research and vaccine production.

- **The Interstate School of Veterinary Science and Medicine (ISVM)**

The EISMV intervenes through its laboratory of control of veterinary drugs (LACOMEV), the laboratory of Microbiology Immunology and Infectious Pathologies (MIPI) as well as the laboratory of parasitology, for the aspects relating to antimicrobials.

- **Importing and distributing wholesalers of veterinary drugs**

According to the provisions of Law 2008-07 of 24 January 2008, in particular Article 37, the manufacture, preparation, import, packaging, wholesale and wholesale distribution of veterinary drugs are the responsibility of approved structures whose technical management is ensured by a veterinary doctor or a pharmacist.

- **Veterinary doctors, ITEs and ATEs in private practice**

According to the provisions of Law 2008-07 organizing the veterinary profession and pharmacy, particularly in its Article 2, the practice of the said profession in Senegal is reserved for veterinary doctors, livestock engineers (ITE) and technical livestock agents (ATE).

Private practice is subject to authorisation by the Minister in charge of Livestock after a reasoned opinion from the Council of the Order of Veterinary Doctors of Senegal (Law 2008-07, article 9); it can be done :

- Or, as a full-time or part-time employee of an approved enterprise or organisation of an associative nature with activities in the field of animal husbandry: in this case, their scope of work is limited to the corporate purpose of the enterprise or organisation,
- Or, on their own account or within a professional veterinary organisation of an associative nature, at the client's premises, in a practice, in a clinic, within the territorial limits where they have been authorised to practise or, in case of force majeure, in any other place.

The veterinary doctor is authorised to practise veterinary medicine and pharmacy, while the ITEs and ATEs are authorised to practise veterinary nursing under the responsibility of a veterinary doctor in private practice or, failing that, of the chief veterinary officer of the locality where he practises or of the nearest administrative district to which his area of practice belongs, in particular as regards the supply and holding of medicines in connection with his authorised activities.

It is however necessary to specify that since 2012, any veterinary doctor from a WAEMU Member State can be authorized to practice freely in another Member State of the Union under the conditions defined by Directive N° 01-2012-CM-UEMOA of 10 May 2012.

- **Holding, prescribing and dispensing of veterinary medicines by the different actors :**

Still according to the provisions of the law 2008-07, only the following persons may hold veterinary medicines for sale to users and for retail distribution, whether free of charge or for a fee

- veterinary doctors registered with the Order of Veterinary Doctors of Senegal;
- pharmacists who own a pharmacy.

Moreover, only the veterinary doctor or veterinarian is entitled to prescribe a prescription.

4.1.3. Mapping of actors and their roles in plant health

The institutional framework for pesticide management is based mainly on five ministries: Ministry of Agriculture and Rural Equipment, Ministry of Livestock and Animal Production, Ministry of Fisheries and Maritime Economy, Ministry of Health and Social Action, and Ministry of Environment and Sustainable Development.

The structure of the Ministry of Agriculture and Rural Equipment is organized in two levels of intervention:

- A national intervention structure: the Direction de la Protection des Végétaux (DPV) ;
- Intervention structures at the decentralized level: the Regional Directorates for Rural Development (DRDR).

The Plant Protection Directorate is responsible for the general protection of crops throughout the national territory. It provides technical and material support to the Regional Directorates of Rural Development.

The main mission of the DPV is first to prevent the introduction of new pests into the national territory in accordance with the provisions of the IPPC, by setting up at the level of land, sea and air borders twelve phytosanitary control posts and eight (08) agricultural warning bases (AWBs) in charge of inspecting and controlling all the entries of plants and plant parts coming from the other States

The mission consists, then, in combating the pests which are already present in the national territory by using effective and safe chemical means which preserve as much as possible the environment and the health of the populations, but also biological and natural means.

At the regional level, the DRDRs are divided into departments including the plant protection department. The plant protection services are linked to village pest control committees set up in each village.

- **Subsidiary bodies**

Other actors are members of the National Chemicals Management Commission. They are :

- Laboratory of Analytical Chemistry and Bromatology,
- Directorate of Pharmacy and Medicines,
- Senegalese Association of Standardization,
- National Council for Rural Cooperation (CNCR),
- Laboratory of the CERES-LOCUSTOX foundation,
- Directorate of Civil Protection,
- National Hygiene Service,
- Inter-State School of Veterinary Science and Medicine (EISMV),
- Customs,
- Consumer associations,
- Institut Sénégalaise de Recherche Agricole (ISRA),
- Industry Branch,
- NGOs (CONGAD, Plan International AFRICA),
- Directorate of Labour and Social Security,
- CONACILSS,
- Directorate of Oceanography and Marine Fisheries,
- Directorate of Inland Fisheries and Aquaculture,
- Association of formulators and distributors of pesticides (Crop Life Senegal).

4.2. Status of antimicrobial regulation

4.2.1. Status of antimicrobial regulation in human health

Findings

- There is no specific antimicrobial stewardship strategy in the national pharmaceutical policy document. They are well taken into account in the strategies and tools deployed for rational use of medicines.
- Senegal does not have regulations or policies specifically dedicated to antimicrobial drugs. The existing legal arsenal and policy documents and operational guides fully integrate antimicrobial drugs.
- A database specifically dedicated to antimicrobial drugs and their use has not been developed. Antimicrobial drugs are well documented in the general drug database.
- Senegal has a national list of essential medicines and products - LNMPE version 2018 (Order n0 016917 of 20/07/2018), inspired by the model list of essential medicines of the World Health Organization (WHO). To date, one hundred and forty-seven (147) antimicrobial molecules are referenced in the National List of Essential Medicines and Products - LNMPE.
- Senegal does not have a special quality assurance and quality control system for antimicrobial drugs. The monitoring of the quality of antimicrobial drugs is well taken into account in the existing system.
- A mechanism is in place to monitor the sale of drugs in Senegal, including antimicrobials. There is no mechanism for monitoring the consumption of antimicrobial drugs.
- The national strategic plan - PSN 2019-2023 of the Directorate of Pharmacy and Medicines and the National Laboratory for the Control of Medicines foresees, as foreseen by **the strategic orientation n° 4: reinforcement of communication for the good use of medicines and other health products**, the reinforcement of communication actions for a rational use of medicines to fight against the abusive use of antimicrobials and the development of antimicrobial resistance. This problem is well taken into account in strategic axis n°7 (strategic orientation n°4), more precisely in specific objective n°7.2: to eradicate the irrational use of antimicrobials by prescribers, dispensers and consumers.
- There is no specific surveillance system for antimicrobial drugs. Adverse drug reaction surveillance for antimicrobial drugs is well covered by the existing national system.
- The AWaRe classification is not used for antimicrobial drugs on the national essential drug list.
- There are no formal guidelines on the prescribing levels for antimicrobial drugs at this time.
- There is a list of antimicrobials with MA at the Directorate of Pharmacy and Medicines. The following table is the list of registered antibiotics from 2015 to 2017

present in the DPM antimicrobial directory and classified according to the WHO AWaRe classification.

Table 10: AWaRe classification of antibiotics registered by DMP

Antibiotics	Classes	ATC code	Categories
Amoxicillin	Penicillins	J01CA04	Access
Amoxicillin/clavulanic Acid	Beta lactam - beta lactamase inhibitor	J01CR02	Access
Azithromycin	Macrolides	J01FA10	Watch
Benzathine benzylpenicillin	Penicillins	J01CE08	Access
Cefalexin	First-generation cephalosporins	J01DB01	Access
Cefepime	Fourth-generation cephalosporins	J01DE01	Watch
Cefixime	Third-generation cephalosporins	J01DD08	Watch
Cefotaxime	Third-generation cephalosporins	J01DD01	Watch
Cefpodoxime proxetil	Third-generation cephalosporins	J01DD13	Watch
Ceftazidime	Third-generation cephalosporins	J01DD02	Watch
Ceftriaxone	Third-generation cephalosporins	J01DD04	Watch
Cefuroxime	Second-generation cephalosporins	J01DC02	Watch
Chlortetracycline	Tetracyclines	J01AA03	Watch
Ciprofloxacin	Fluoroquinolones	J01MA02	Watch
Clarithromycin	Macrolides	J01FA09	Watch
Doxycycline	Tetracyclines	J01AA02	Access
Erythromycin	Macrolides	J01FA01	Watch
Flucloxacillin	Penicillins	J01CF05	Access
Fosfomycin (oral)	Phosphonics	J01XX01	Watch
Gatifloxacin	Fluoroquinolones	J01MA16	Watch
Gentamicin	Aminoglycosides	J01GB03	Access
Imipenem/cilastatin	Carbapenems	J01DH51	Watch
Levofloxacin	Fluoroquinolones	J01MA12	Watch
Lincomycin	Macrolides	J01FF02	Watch
Meropenem	Carbapenems	J01DH02	Watch
Moxifloxacin	Fluoroquinolones	J01MA14	Watch
Norfloxacin	Fluoroquinolones	J01MA06	Watch
Ofloxacin	Fluoroquinolones	J01MA01	Watch
Spiramycin/metronidazole	Combination of antibiotics	J01RA04	Watch

Sulfamethizole/trimethoprim	Trimethoprim - sulfonamide combinations	J01EB02	Access
Sulfamethoxazole/trimethoprim	Trimethoprim - sulfonamide combinations	J01EE01	Access
Sulfametrole/trimethoprim	Trimethoprim - sulfonamide combinations	J01EE03	Access
Sulfamoxole/trimethoprim	Trimethoprim - sulfonamide combinations	J01EE04	Access
Thiamphenicol	Amphenicols	J01BA02	Access
Tobramycin	Aminoglycosides	J01GA01	Watch

Assets

- Existence of a national pharmaceutical policy that fully integrates the promotion of rational use of medicines.
- Existence of a regulatory framework for the manufacture, import, distribution, dispensing and licensing of pharmaceutical establishments.
- Existence of an antibiotics commission at the level of the Committees for the Fight against Nosocomial Infections -CLIN in the Public Health Establishments - EPS, established by ministerial decree n° 005711 of 07 July 2004.
- Existence of a National Committee on Antibiotic Therapy, established by a ministerial order n° 05808 of July 3, 2008.
- Existence of national recommendations on the proper use of antibiotics developed by the National Programme for the Fight against Nosocomial Infections.

Deficiencies

- Lack of a national program and methodology for collecting and processing data on antimicrobial drug consumption.
- Lack of a system for recording and centralizing antimicrobial drug consumption data (surveillance team).
- Lack of a database of antimicrobial drugs marketed in the country
- No systematic use of the DHIS2 platform to report antimicrobial consumption data.
- Lack of a monitoring system for the sale of antimicrobial drugs.
- Non-use of the WHO AWaRe classification in the National Essential Drug List - NEDL.
- Lack of formal guidelines on authorization levels for antimicrobial drugs.
- Lack of coordination between the national antibiotic therapy committee, the AMR surveillance system, and antimicrobial consumption surveillance.
- No multisectoral and integrated coordination of surveillance of antimicrobial drug consumption in the human health, animal health, environmental, and agriculture and agri-food sectors.

- The WHO ATC (Anatomical Therapeutic Chemical Classification) is not used for antimicrobial drugs on the National Drug and Essential Products List - NDSL.
- Lack of use of Defined Daily Doses (DDDs) to quantify antimicrobial drug use.
- Existence of an unofficial, illegal, unregulated and uncontrolled channel for the sale of medicines, the data of which cannot be collected or evaluated.
- Porosity of land borders to illegal imports and re-exports.
- Opportunities for direct importation of antimicrobial drugs by prescribers or patients.
- Possibility of direct procurement of antimicrobial drugs from neighbouring countries.
- Antimicrobial drug consumption data from donations can be difficult to compile.

Recommendations

- Set up a national multidisciplinary team to monitor the consumption of antimicrobial drugs (Pharmacists, Data Manager, Logisticians, Qualiticians...).
- Establish a national antimicrobial drug use surveillance program.
- Establish a partnership agreement between the public and private sectors to facilitate the collection and transmission of antimicrobial drug consumption data.
- Develop protocols and clinical and therapeutic guidelines for the use of antimicrobial drugs.
- Implement the ATC/DDD methodology for collecting antimicrobial drug consumption data.
- Develop a national database for antimicrobial drugs included in the consumer surveillance program with a valid marketing authorization.
- Establish a regulatory provision that mandates the provision of data on the use and consumption of antimicrobial drugs by the relevant sectors.
- Implement training modules on health data collection for health staff and data providers.

4.2.2. Status of antimicrobial regulation in animal health

Findings

- Existence in Senegal of collaboration between MEPA and ODVS; however, the levels of information in terms of regulations on veterinary pharmacy and on the availability or not of certain data (particularly for antimicrobials) are not the same;
- There are laws organizing the veterinary profession and pharmacy, but the levels of authorization to open veterinary practices and clinics as well as veterinary care practices differ according to the level of education of the professional bodies authorized to practice the veterinary profession;
- Senegal does not have specific regulations or policies on antimicrobial drugs;
- Senegal has a quality control and assurance system for veterinary drugs (DMPV); however, this system does not include specific provisions for antimicrobials, although they are generally taken into account;

- Data on efficacy, compliance, and/or adverse events related to antimicrobial use exist but are not shared
- Although taking into account the OIE list of antimicrobials of veterinary importance, Senegal does not have its own list of essential antimicrobials;
- The quality assurance system for veterinary drugs takes antimicrobials into account but not in a specific way;
- Antimicrobial guidelines exist but are sometimes not well known or used;
- No monitoring of antimicrobial use by farmers ;
- An annual sanitation (CAMVET) and awareness campaign on veterinary drugs is held annually, but it is not directed solely at antimicrobials;
- Existence of networks of livestock auxiliaries that can be used to raise awareness of rabies but are not channelled;
- Existence of community radio stations that can be used as a means of raising awareness but are not sufficiently exploited in the fight against AMR;
- Most of the agents of the wholesale distribution structures of veterinary drugs have already received capacity building on AMR but few of them participate in public awareness campaigns;
- There is extensive use of antimicrobial agents in poultry production, particularly in the form of anti-stress agents (often antimicrobials combined with vitamins);
- The non-compliance rates of antimicrobials for veterinary use analyzed by LACOMEV are very high: more than 50% overall, with an average of 45% for the legal circuit and 55% for the illegal circuit.

Assets

- Existence of a legal framework regulating the manufacture, importation, possession, distribution and dispensing of veterinary drugs;
- Existence of regulations on prescription levels for veterinary drugs including antimicrobials (Law 2008-07);
- Existence of a Division of Veterinary Medicine and Pharmacy at the DSV (MEPA);
- Presence in Senegal of a laboratory for the control of veterinary drugs (LACOMEV);
- Privatisation of veterinary medicine, which has led to the occupation of the field, even if not homogeneous, by various actors in animal health (veterinary doctors, ITE, ATE), making the care of animal health much more available;
- The use of antimicrobials as growth promoters in animal feed is not permitted in Senegal;
- Existence of data on the level of antimicrobial use (IPMV/DVDS/MEPA reports) ;
- Existence of a High National Council for Global Health Security / One Health (HCNSSM/GHSAS) housed in the General Secretariat of the Government (SGG).

Deficiencies

- Lack of rigour in the application of certain provisions regulating the animal health sector ;

- Non-compliance with licensing provisions by some actors ;
- Presence of antimicrobials without marketing authorisation justified by the slowness of the Community authorisation procedures making the available therapeutic arsenal limited;
- Lack of an independent system to monitor the sale of antimicrobial drugs for veterinary use ;
- No multisectoral and integrated coordination of antimicrobial drug consumption monitoring in the human health, animal health, environmental and agriculture and agri-food sectors: One Health often remains at the conceptual stage;
- Existence of an unofficial, illegal, unregulated and uncontrolled channel for the sale of medicines, the data for which can neither be collected nor evaluated with certainty;
- Insufficient personnel and logistical means at the DMPV / DSV level, making its activities irregular;
- Porosity of land borders to illegal imports and re-exports ;
- Possibility of direct purchase of antimicrobial drugs by farmers, from veterinary practices and pharmacies, without prior prescription or even from some wholesalers.

Recommendations

- To rigorously implement and set up a national multidisciplinary team to monitor antimicrobial drug consumption in human, animal and environmental health (One Health);
- Establish a partnership agreement between the public and private sectors to facilitate the collection and transmission of antimicrobial drug consumption data;
- Develop protocols and clinical and treatment guidelines for antimicrobial drug use;
- To exploit the LACOMEV data on AMR in order to better define the problem in the veterinary environment in Senegal and to help in decision making;
- Strengthen the UEMOA community registration system in order to speed up the procedure for granting marketing authorizations;
- Make a regulatory provision that mandates the provision of data on the use and consumption of antimicrobial drugs by the relevant sectors;
- Implement training modules on health data collection for health staff and data providers.

4.2.3. Status of antimicrobial regulation in plant health

Findings

At the sub-regional level, the countries rely on the CILSS regulation on "Common regulation on the registration of pesticides in CILSS member countries".

The Sahelian Pesticides Committee (SPC), based in Bamako (Mali), is the linchpin of the common regulations. In practice, it replaces the national registrations. It is a very important instrument in the national and concerted management of pesticides.

At the community level, there are two regulations on the harmonization of rules governing the registration, marketing and control of pesticides within WAEMU and ECOWAS ("Regulation N004/2009/CM/UEMOA, and Regulation N003/2008/ECOWAS").

Pesticides officially authorized in the country are those that have obtained the Authorisation Provisoire de Vente or the homologation of the Comité Sahélien des Pesticides (CSP).

At the national level, the Direction de la Protection des Végétaux (DPV) is the body in charge of the extension and application of pesticide regulations.

The use of unregistered pesticides or pesticides without a provisional authorisation for sale is exceptionally accepted in the following cases

- In the case of a phytosanitary, veterinary or sanitary emergency such as the unexpected invasion of a pest or the unexpected appearance of a disease vector
- If no other pest management alternatives are available.

Senegal has a phytosanitary regulation materialized by the phytosanitary booklet whose last revision dates back to 2017.

Assets

- Existence of a National Directorate (DPV) in charge of the regulation and control of phytosanitary products.
- National Chemicals Management Commission and National Pesticides Management Subcommission,
- Adoption, dissemination and application of ECOWAS and UEMOA regulatory texts on pesticides.
- Plurality of actors involved in the regulation and control of pesticides
- Existence of a system of monitoring and follow-up of manufacturers, distributors and retailers of pesticides with the DPV as the central entity and the phytosanitary control posts and agricultural warning bases as deconcentrated entities.

Deficiencies

- Difficulties in implementation and inadequate monitoring and follow-up ;
- Weak collaboration between different institutions ;
- Lack of effective implementation of the texts governing the activity ;
- Weak capacity of state services to intervene
- Absence of national texts to regulate the use of plant protection products in plant health

Recommendations

- Strengthen the logistical capacities of institutional actors, in particular the DPV, phytosanitary control posts and agricultural warning bases.
- Establish the national legislative and regulatory framework on the rational use of plant protection products in plant health.
- Establish nationwide approved sales outlets for plant protection products and incentive policies for the formalization of informal dealers.
- Organize national conferences on the regulation and control of plant protection products.

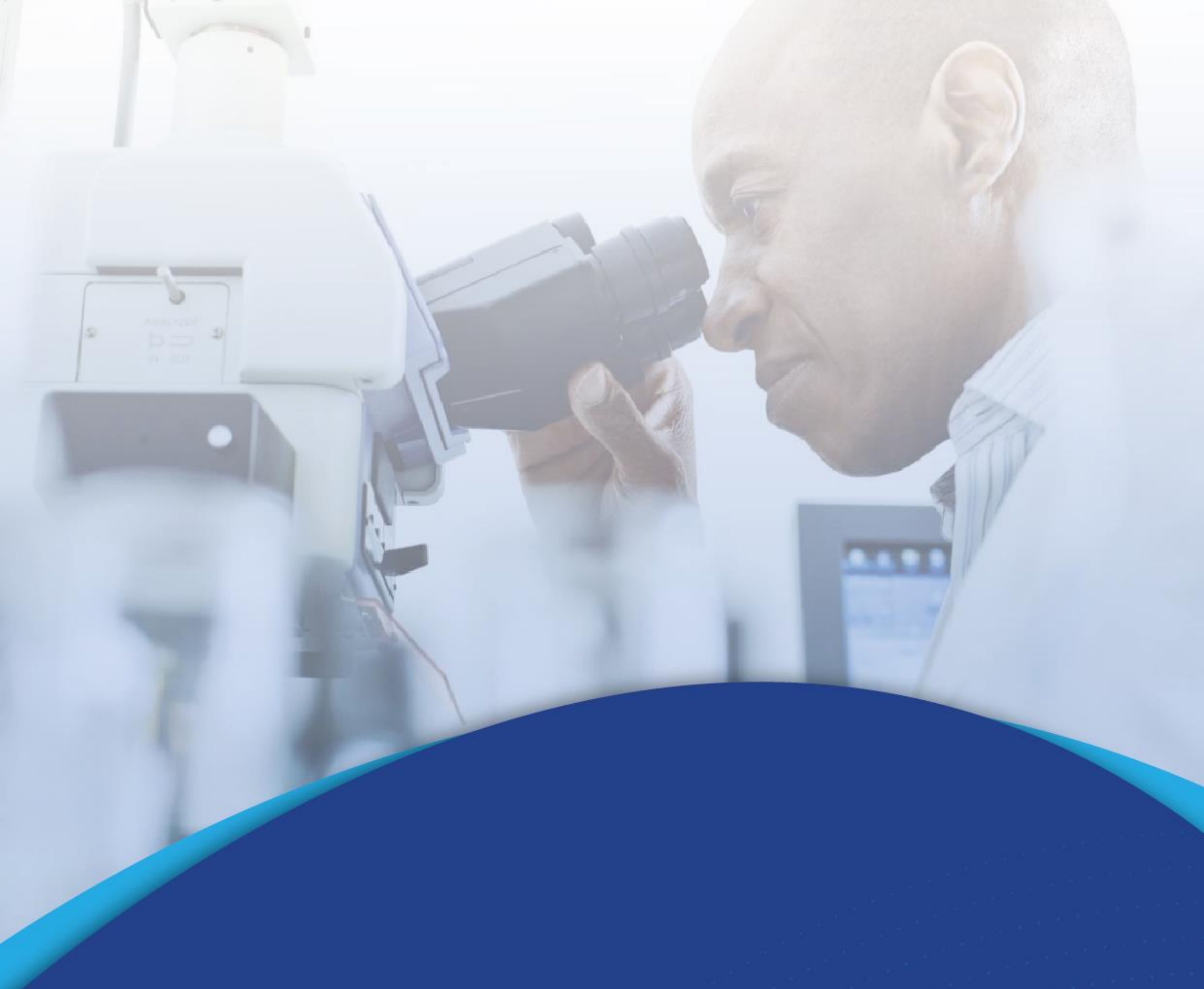

ANALYSE SITUATIONNELLE DU CONTRÔLE DE LA QUALITE DES ANTIMICROBIENS

5. QUALITY CONTROL

5.1. Quality control actors and their roles

5.1.1. Quality control actors and their roles in human health

- **The National Drug Control Laboratory (NDCL)**

Under Decree No. 2004 1404 of 4 November 2004, the LNCM is responsible for the technical control of medicines, reagents and other health products.

To this end, the LNCM carries out the following activities

- the control of samples of medicinal products subject to registration procedures ;
- control of samples of medicines from public and private pharmaceutical establishments, taken by the Inspectors of the Directorate of Pharmacy and Medicines - DPM;
- the control of batches of yellow fever vaccine manufactured by the Pasteur Institute of Dakar, within the framework of the release of batches by the Directorate of Pharmacy and Medicines - DPM;
- The evaluation of manufacturers' files received from the Directorate of Pharmacy and Medicines - DPM ;
- the control of drug samples monitored in the framework of collaborative studies with other laboratories in the world, organized by the WHO and/or ANSM (Agence Nationale de Sécurité des Médicaments) and the USP PQM (Promotion of the Quality of Medicines of the American Pharmacopoeia)
- the control of products whose use requires guarantees similar to those of medicines for public health; the monitoring of the quality of medicines in these programmes (National Malaria Control Programme - PNLP, National Tuberculosis Control Programme - PNT, Division for the Control of AIDS and Sexually Transmitted Infections - DLSI, Directorate for Reproductive Health and Child Survival - DSR/SE, etc.) at the peripheral level with the Minilab programme ;
- the control of drugs from the National Supply Pharmacy (PNA) and those used in the fight against Neglected Tropical Diseases (NTD).

5.1.2. Quality control actors and their roles in animal health

The main actor of the quality control of veterinary drugs is the LACOMEV **(Laboratory of Control of Veterinary Drugs).** The LACOMEV is a laboratory :

- of the World Organisation for Animal Health (OIE),
- specialized in the analytical expertise of drugs at the Inter-State School of Veterinary Sciences and Medicine in Dakar.
- official partner of the Livestock Directorate of several West and Central African countries for the MA (Marketing Authorization) and registration files, as well as the monitoring of the quality of veterinary drugs. It is therefore solicited by the pharmaceutical firms of the UEMOA, particularly in the framework of community

MAs, but also by the DMPV of Senegal (MEPA/DSV) and the PRAPS (Regional Programme of Support to Pastoralism in the Sahel / Senegal).

The LACOMEV has as activities :

- quality control of veterinary drugs (finished products),
- identification of active ingredients,
- the dosage of active ingredients,
- control of galenic characteristics,
- the determination of antibacterial and antiparasitic residues,
- the analytical and statistical study of the quality of veterinary medicines,
- training and research.

5.1.3. Quality control actors and their roles in plant health

In the agricultural sector, the quality control of phytosanitary products is ensured by the Direction de la Protection des Végétaux **(DPV) and the laboratory of the CERES LOCUSTOX Foundation.**

PLANT PROTECTION DEPARTMENT

The activities of control of phytosanitary products carried out by the DPV and its deconcentrated bodies are summarized as follows

- Monitor and supervise manufacturers, distributors and resellers
- Checking residue levels before issuing a phytosanitary certificate for pesticides intended for export
- Monitoring of farms and farming practices to link farming practices to exceedance of maximum residue limits (MRLs)
- Controlling packaging centres and sales outlets
- Inspect pesticide storage facilities for applicants for approval
- Inspecting sites for pest control, rodent control, disinfection and fumigation,

To this end, the DPV has set up a monitoring system composed of several actors.

Figure 3: Monitoring system for plant protection products

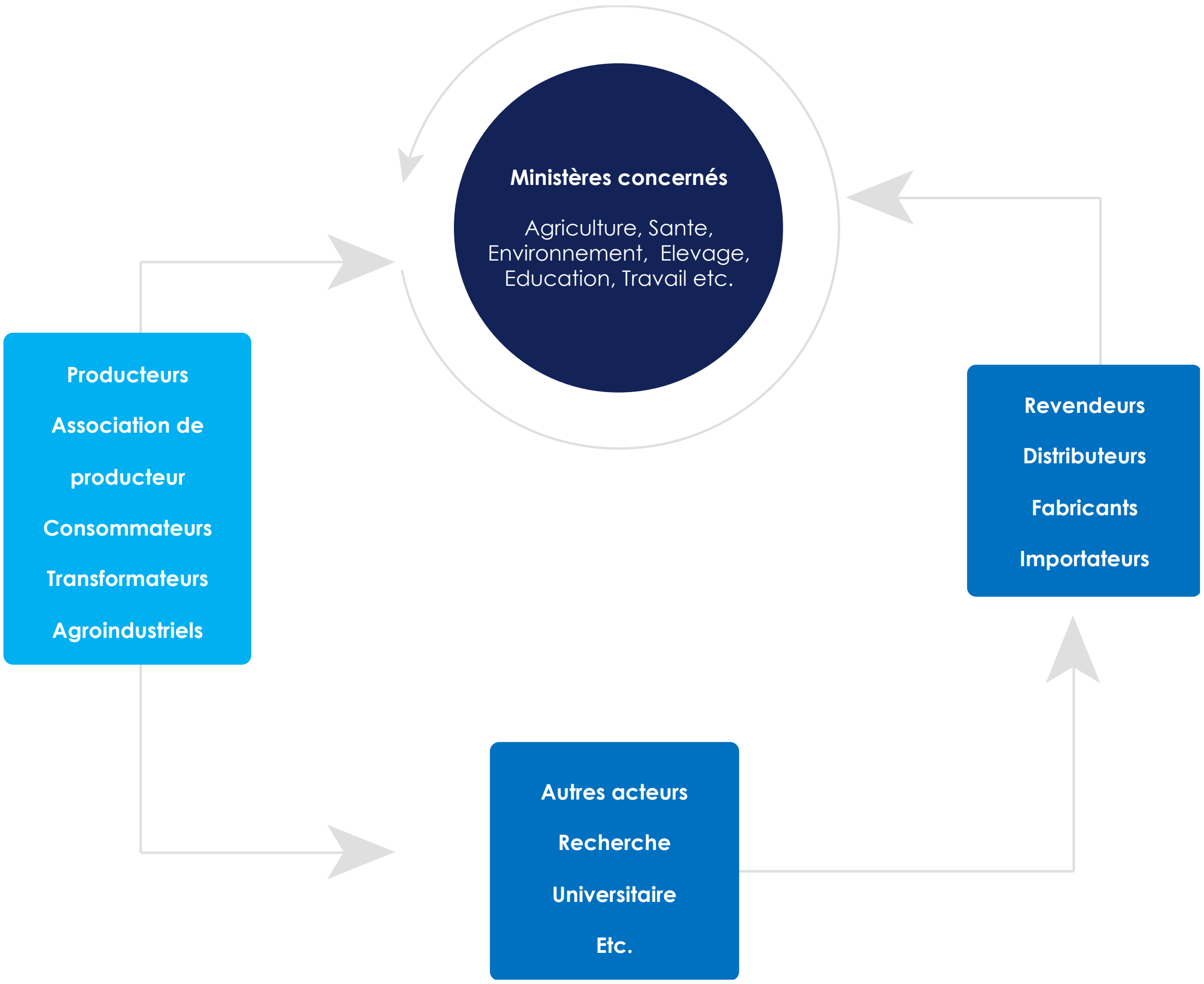

Source: Annual Report of the Plant Protection Legislation Office of the VPD 2017

the CERES Foundation laboratory LOCUSTOX

The control activities of the CERES LOCUSTOX Foundation's laboratory are focused on :

- The study of the impacts of pesticides on the environment, animal and human health
- Analysis of residues of pesticides, heavy metals, PCBs and other pollutants in agricultural and fishery products;
- Advice to decision makers, distributors, producers and exporters on the rational and safe use of pesticides;
- Training of producers on alternative methods to conventional chemical control;
- Promotion of the sanitary quality (Maximum Residue Limits of Pesticides) of agricultural products;
- The control of the negative impacts of chemicals through the implementation of its scientific investigation tools in terms of control:

- the quality of phytosanitary treatments and the monitoring of their possible harmful effects on the environment and the health of the population;
- of industrial waste in accordance with the Environmental Code.

5.2. Status of antimicrobial quality control

5.2.1. Status of antimicrobial quality control in human health

Findings

The National Drug Control Laboratory - NDCL is an essential link in the quality monitoring of antimicrobial drugs.

To operationalize the drug control mission, the NMLC deploys two control devices:

- Monitoring the quality of programme drugs (Antiretrovirals, Anti-malarials, Anti-tuberculosis and Reproductive health drugs);
- Antimicrobial control through compliance testing (MA, NAP, inspections, post-marketing follow-up, etc.).

The balance of quality control activities for antimicrobial drugs and health program drugs (Antiretrovirals-Anti-malarials-Antituberculosis) for the last three years presented in graph 1 below gives the following figures:

In the last three years, the NMLC has controlled a total of 33 antimicrobial molecules with an estimated quantity of 496 lots. Control activity has decreased over the last two years compared to 2017. Indeed, the NMLC controlled 12 antimicrobial molecules evaluated at 273 lots in 2017 against 9 molecules of 34 lots in 2018 and 12 molecules of 189 lots.

Figure 12: Summary of antimicrobial testing activities over the last 3 years

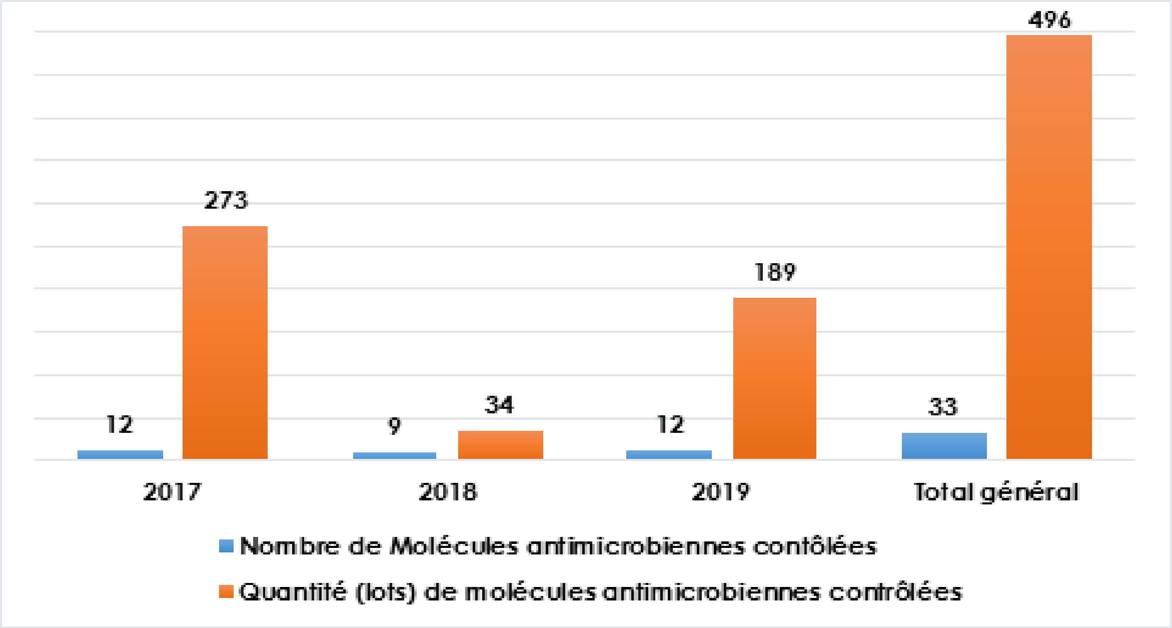

Source: National Drug Control Laboratory (NDCL)

The distribution of the controlled molecules according to their quantity in unit of batch is presented in the following graph 2. Analysis of the data in this graph shows that the molecules CCM Antiretrovirals (94 batches), Lamivudine/Zidovudine/Nevirapine (80 batches), Lopinavir/ritonavir (77 batches) and CCM Anti-malarial (62 batches) have been the most frequently tested by the NML over the last three years.

Graph 13: Distribution of controlled molecules according to their quantity per batch unit

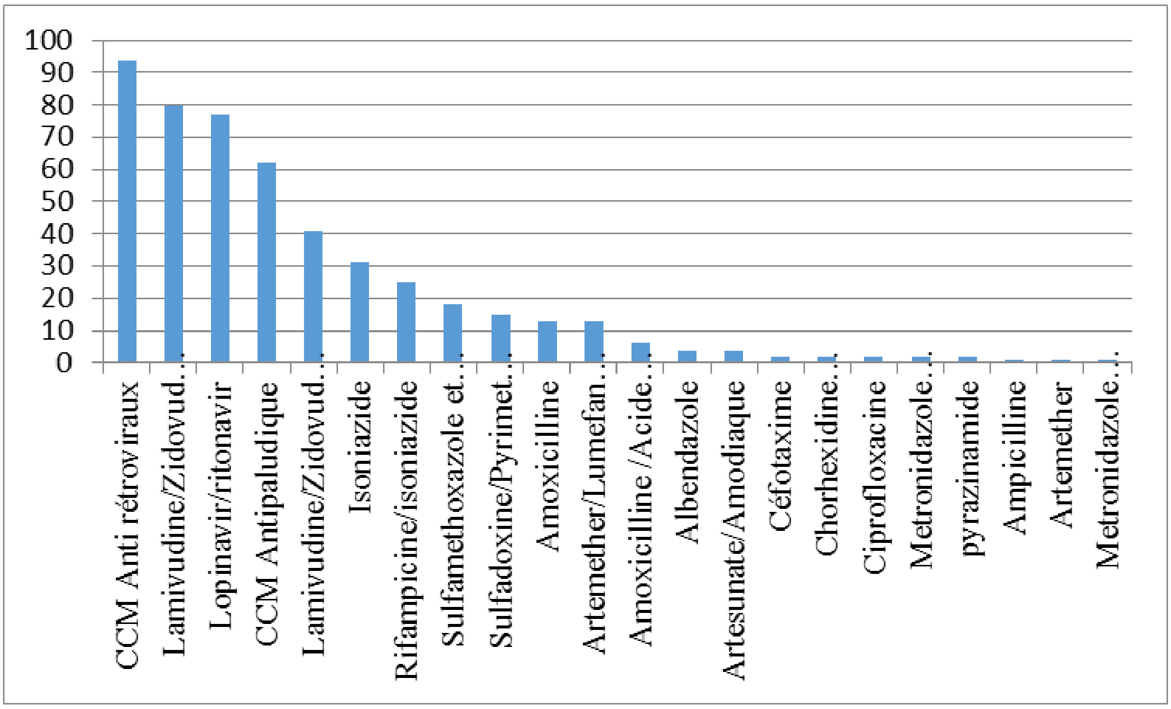

Source: National Drug Control Laboratory (NDCL)

In addition, in 2019, sixty (60) dispensation sites were supervised in the 14 regions of Senegal with 420 samples taken

The LNCM is also engaged in a quality process with the ISO 17025 v 2017 standard, the aim of which is to move towards accreditation, with the technical and financial support of USP-PQM+-USAID (Promoting Quality of Medicines).

Antimicrobial drug and antimicrobial resistance surveillance - AMR is one of the perspectives of the NMLC for 2020.

Assets

- Adequate technical facilities for the quality control of medicines;
- Operational quality management system and ISO 17025 v 2017 accreditation project;
- Recipient of a 75% grant from USP-PQM (United States Pharmacopeia Drug Quality Promotion Program) for the acquisition of reference materials;
- Development of a strategic development plan 2019 - 2023;
- Reference laboratory for the control of the yellow fever vaccine produced by the Pasteur Institute of Dakar.

Deficiencies

- Lack of legal status and administrative and financial autonomy to recover the costs of carrying out quality controls of medicines;
- Insufficient technical staff ;
- Lack of equipment maintenance contracts with external service providers;
- Weakness of the resources allocated by the guardianship ;
- Absence of a partnership agreement with the Directorate of Pharmacy and Medicines and the National Supply Pharmacy;
- Lack of a mechanism to monitor the quality of antimicrobial drugs in the context of the fight against antimicrobial resistance at the national level ;
- Difficult access to continuing education for technical staff.

Recommendations

- Institutional change towards administrative and financial autonomy, with a clear legal status;
- Position the NMLC as a national reference laboratory for monitoring the quality of antimicrobial drugs, as part of the national antimicrobial consumption monitoring program;
- Strengthen the human, material and financial resources for a great autonomy of operation;
- Consolidate partnerships for the continuity of technical support to enhance the technical platform;
- Implement the creation of the two new control units foreseen in the strategic development plan.

5.2.2. Status of antimicrobial quality control in animal health

Findings

- The control of veterinary drugs is frequently carried out by the LACOMEV;
- The main clients for quality control of veterinary drugs are the Directorate of Veterinary Services (DSV), the PRAPS (Regional Support Programme for Pastoralism in the Sahel) and pharmaceutical companies;
- Samples of veterinary drugs come from both the formal and informal channels;
- Of the samples received and analysed by LACOMEV, the rate of non-compliance is 50% (samples from the sub-region) and 60% for samples from Senegal;
- All samples received and analysed are the subject of a double archived test report in electronic and paper version.

Strengths:

- LACOMEV is a reference laboratory for the quality control of veterinary drugs in the sub-region;
- Carrying out quality control of veterinary drugs from the informal system.

Disadvantages:

- High rate of non-compliance of controlled drugs.

Recommendations :

- To position LACOMEV as a reference laboratory for the quality control of veterinary drugs within the framework of the rational management of antimicrobial drugs programme;
- Strengthen the technical platform of LACOMEV ;
- Develop partnerships with other reference laboratories in human and environmental health, agriculture and agri-food.

5.2.3. Status of antimicrobial quality control in plant health

Findings

- The existence of a system of regulation and control of imports of phytosanitary products, including the obtaining of an approval before import and the setting up of phytosanitary control posts and agricultural warning bases
- The existence of health and environmental risks related to obsolete plant protection products and empty packaging not disposed of in accordance with current recommendations
- Carrying out a national inventory of plant protection products within the framework of the project for the management of obsolete products;

- The existence of a laboratory for the control of pesticide residues and the promotion of sanitary quality (MRL) in agricultural products;
- The existence of a monitoring system for antimicrobial sales in the agricultural sector;
- The existence of a national antimicrobial resistance program that is multisectoral;
- Existence of an inspection system for agricultural practices.
- Programme for Monitoring Health Quality and Developing Draft National Health Standards for Maximum Residue Limits (MRLs) for Pesticides
- Research and development project on the development of warning systems on health risks linked to pesticides (SARIS-pesticides.) and health monitoring linked to exposure to pesticides of producers/applicators in the Niayes area
- WALLONIE BRUSSELS Project "Better protection of the health of the Senegalese consumer by improving the quality control of foodstuffs
- The CERES Foundation laboratory LOCUSTOX has conducted several studies on the levels of contamination of soil, water and market garden crops by pesticide residues in the Niayes zone over the past five years. The results of the most recent study (published in March 2018) on the "monitoring of the quality of geo-referenced market garden produce in the Niayes zone in 2017" are presented below:

Figure 14: Pesticides Searched in the Study

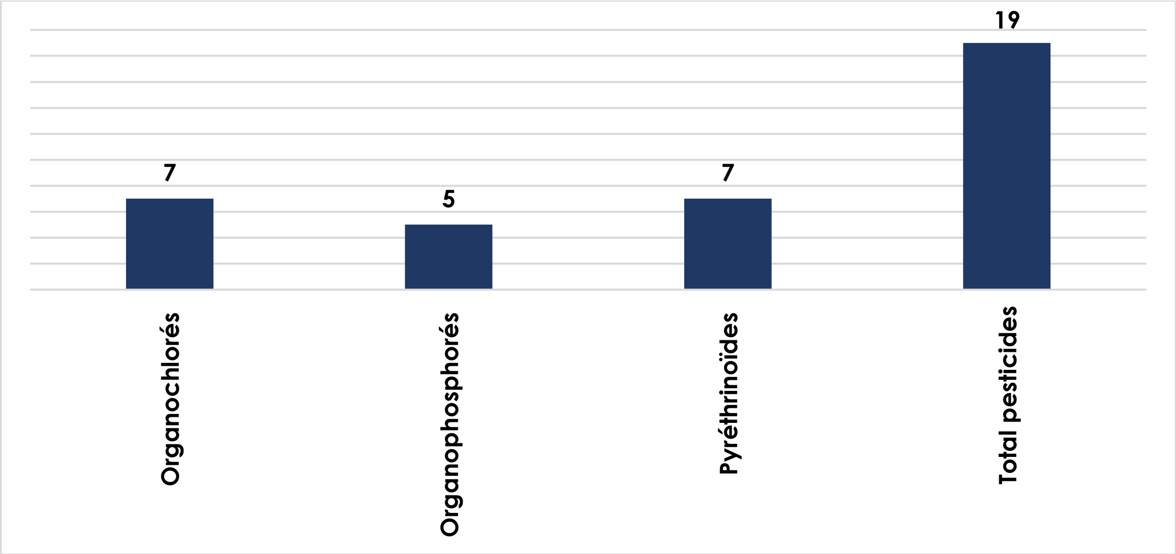

Source: Report on the monitoring of the quality of geo-referenced market garden produce in the Niayes area in 2017

Table 11: Sample of vegetable crops in the study

Vegetable speculation	A Bitter	Eggplant	Carrot	Cabbage	Sheet of mint	Green Bean	Turnip	Onion	Potato	Leek	Pepper	Tomato
**Number**	7	3	5	5	**1**	4	1	6	2	2	4	5
**Percentage (%)**	16	7	11	11	2	9	2	13	4	4	9	11

Source: Report on the monitoring of the quality of geo-referenced market garden produce in the Niayes area in 2017

The results of the analysis of pesticide residues in the vegetable crops are recorded in the appendix.

The results of pesticide residue analyses on samples of vegetable crops contaminated by pesticides from forty-four (44) localities (Table 3) show that, of the 19 pesticides targeted, only six (06) are present in the 10 targeted vegetable crops, with a frequency of occurrence of :

- 27 and 22 times, respectively, for dimethoate and dicofol;
- 11 and 1 times, respectively, for Lambda-cyhalothrin and bifenthrin;
- 7 times each for Trifluralin and chlorpyrifos Ethyl.

Figure 15: Number of occurrences of an active ingredient in samples of vegetable crops

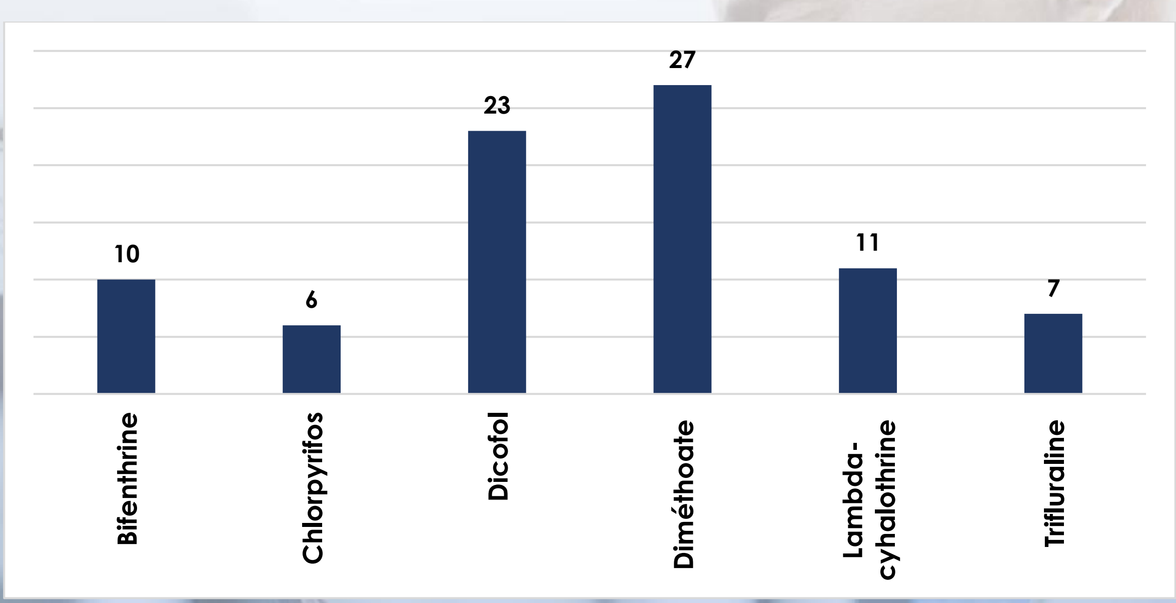

Source: Report on the monitoring of the quality of geo-referenced market garden produce in the Niayes area in 2017

- **Strengths:**
  - Existence of a functional regulation and control system for plant protection products
  - Project for the management of obsolete phytosanitary products, realization of a national inventory of obsolete products and recovery of empty packaging from small producers
  - Existence of a laboratory for the control of pesticide residues in agricultural products
  - Existence of a national monitoring system for sales of plant protection products in the agricultural sector.

ANALYSE SITUATIONNELLE DE L'UTILISATION DES ANTIMICROBIENS

- **Deficiencies**
  - Non perpetuation of pesticide monitoring and control structures
  - Lack of human and logistical resources to verify pesticide compliance
  - Inadequate system for monitoring the use of plant protection products in agricultural practices

- **Recommendations**
  - Strengthening quality control of active ingredients used in the formulation of pesticides and imported plant protection products
  - Strengthening of the system for monitoring plant protection products at national level
  - To position the CERES LOCUSTOX Foundation laboratory as a reference laboratory for quality control of pesticides and residues in agricultural products

6. ANTIMICROBIAL USE

6.1. SUPPLY CHAIN ANALYSIS

6.1.1. Mapping of actors and their roles

6.1.1.1. Mapping of actors and their roles in human health

The players in the supply chain are as follows:

Directorate of Pharmacy and Medicines

The Directorate of Pharmacy and Medicines was created as the National Authority for Pharmaceutical Regulation by Decree No. 2004-1404 of November 4, 2004, organizing the Ministry of Health and Medical Prevention.

It is charged by the Ministry of Health and Social Action to implement the following regulatory missions:

- licensing of establishments for the manufacture, distribution and importation of drugs;
- the registration of medicinal products for the purpose of issuing marketing authorisations ;
- control of drug imports ;
- pharmaceutical inspection ;
- pharmacovigilance ;
- monitoring of clinical trials ;
- control of the promotion and advertising of medicinal products ;
- pharmaceutical information management and rational use of medicines ;
- the development, implementation and monitoring of policy and programmes in the field of pharmacy and medicines;
- the development and monitoring of the application of legislative and regulatory texts relating to pharmacy, medicines and medical devices.

The National Procurement Pharmacy (NPP)

The **ANP** is the technical arm of the Ministry of Health and Social Action for the supply of medicines at the national level. It ensures the supply through an international call for tender and the distribution of essential medicines for the public sector.

The supply chain includes, among other components, (i) selection and quantification of medicines and other health products, (ii) procurement and importation of medicines, (iii) distribution and storage, and (iv) use/consumption.

For selection, there is a national list of essential medicines which is regularly revised. However, there is a problem with its availability at the various service delivery points.

For quantification, there are mechanisms for quantifying medicines and other health products. For the acquisition of medicines, the procedures of the code of markets for

the purchase of essential medicines, the low coverage of the needs for medicines by local production and the difficulties linked to the process of obtaining marketing authorisations slow down the process.

For distribution, the ANP has implemented several strategies in recent years to make essential medicines and products available and accessible.

This mainly involves reinforcing the territorial coverage by setting up eleven Regional Supply Pharmacies (PRA), setting up sales depots with margin repayment (DVRM) in health facilities, and implementing innovative initiatives (PRA Mobile, "Jegesinaa", "Yeksina").

It has a two-year replenishment cycle. Whenever possible, ANP contracts for a range of quantities and staggered deliveries to allow for adjustments to increases and decreases in product demand.

Ninety percent of the products it supplies come from abroad, and ***ten percent*** come from local manufacturers.

In addition to the international call for tender launched every two years, the ANP carries out a national call for tender intended exclusively for drug producers based in the WAEMU region.

Figure 16: ANP Supply Chain

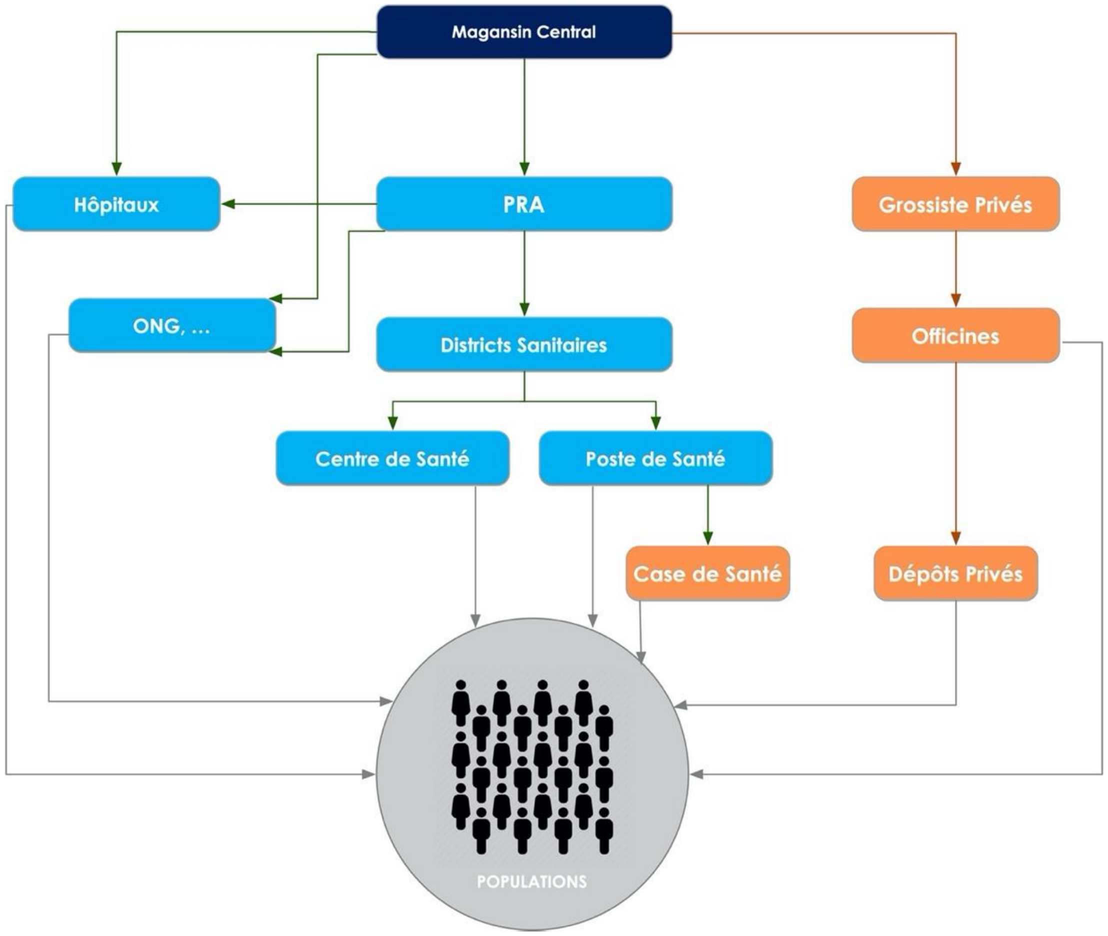

- **Private wholesale distributors of medicines**

By virtue of their mission, private wholesale distributors act as an interface between pharmaceutical companies and pharmacies. Through their daily deliveries to pharmacies, they guarantee the accessibility and availability of medicines to all, under optimal safety conditions.

They purchase from pharmaceutical companies, manage and store in their establishments throughout the country, deliver and make available to pharmacies references of medicines with a marketing authorisation and in rare cases of specialities without a visa.

In Senegal, the commercial distribution system is extremely efficient in ensuring the timely delivery of high quality products. The DPM has authorized ***four (4)*** wholesale distributors to work in the country. These are ***LABOREX, UBIPHARM*** (formerly Cophase), ***SODIPHARM*** and ***DUOPHARM.*** They supply the private commercial pharmaceutical sector, particularly pharmacies.

6.1.1.2. Mapping of actors and their roles in animal health

The private sector animal health actors are:

- Wholesalers importers distributors ;
- Veterinary Doctors in private practice ;
- ETIs and ETAs in veterinary nursing practices;
- Pharmacists.

According to the BMPV (DSV/MEPA) report for the year 2019, wholesalers, veterinary doctors and veterinary para-professionals (ITEs and ATEs) total three hundred and fourteen (314), including 194 ITEs and ATEs, 120 veterinary doctors installed as wholesalers (8) or as practitioners (112). Others are employees of private structures.

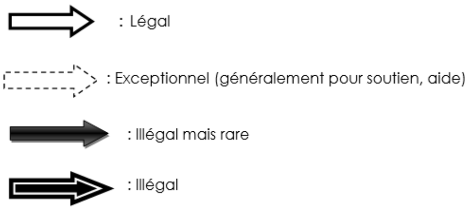

Figure 4 : distribution circuit of veterinary drugs in Senegal

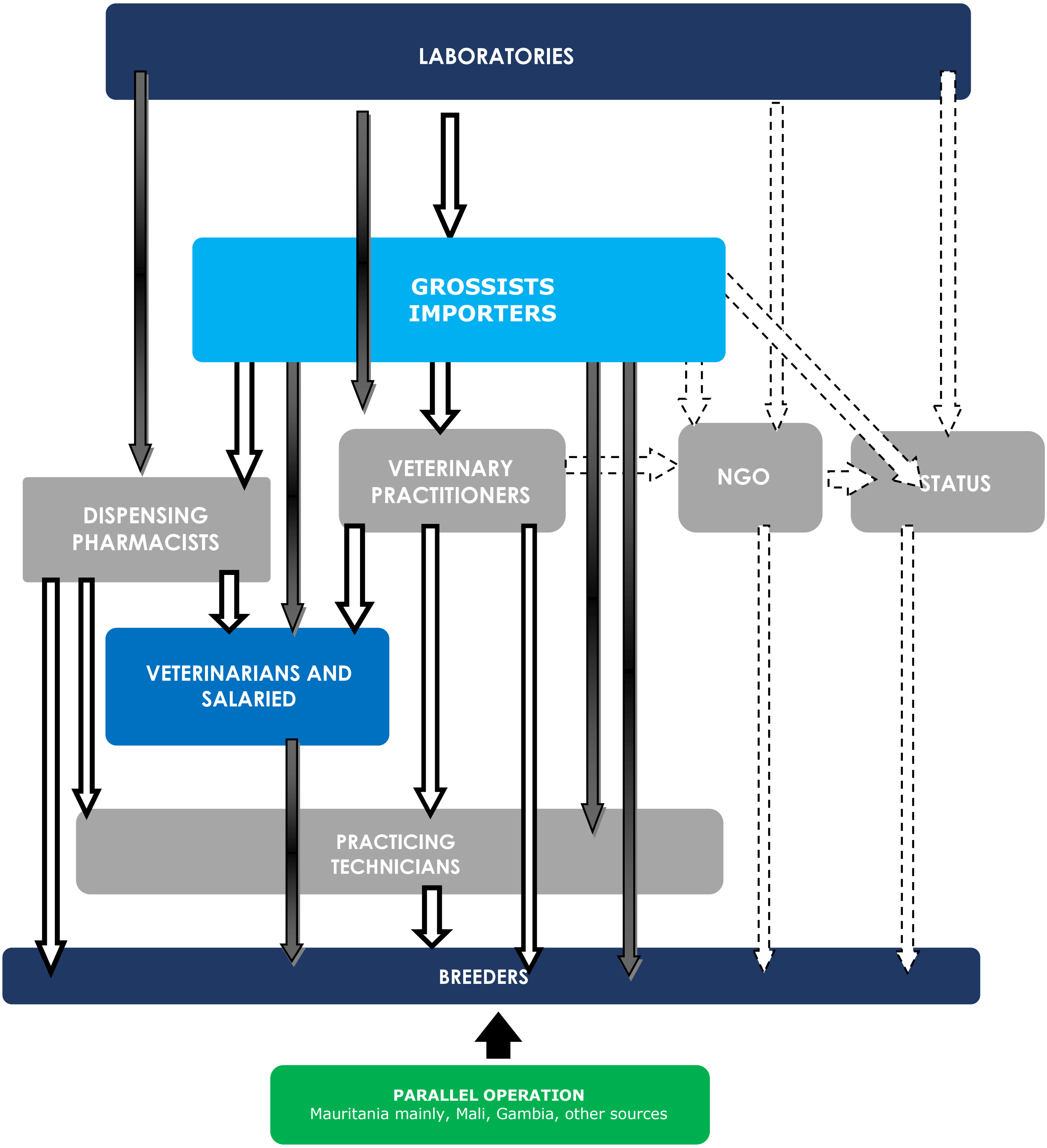

6.1.1.3. Mapping of actors and their roles in plant health

The pesticide supply chain is ensured by private companies authorized to import and sell phytosanitary products for agricultural, domestic and sanitary use. The main manufacturers of pesticides are SENCHIM and SPIA, mainly for agricultural use, SOCHIM and VALDAFRIQUE which mainly make public hygiene pesticides *(pest and pesticide management plan, May 2013)*.

The pesticide import system is well controlled. Any request for the import of phytosanitary products is subject to obtaining prior import approval.

The marketing flow of agro-pharmaceutical products is described in the figure below:

Figure 5: Pesticide Marketing Flows

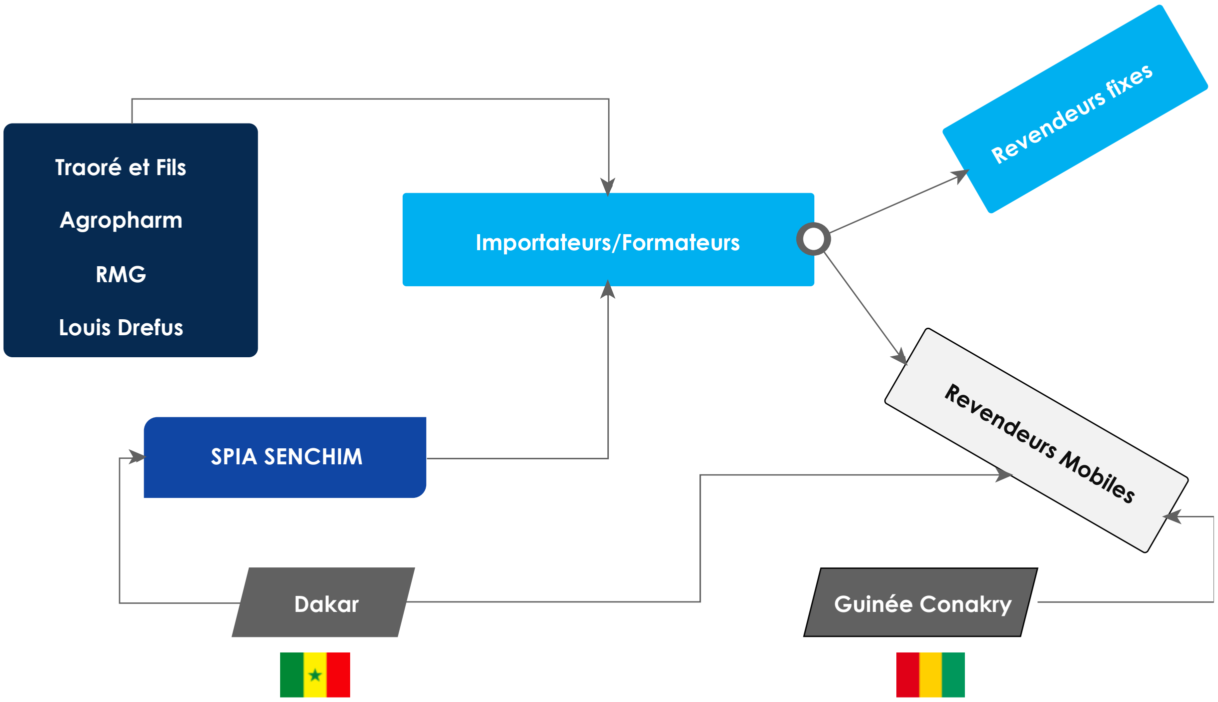

Source: Direction de la Protection des Végétaux

6.1.2. State of play of the supply chain

6.1.2.1. Status of the antimicrobial supply chain in human health

Findings

- **On imports of antimicrobials**

Drug imports over the past five years have varied in value. They rose in 2016 to reach a peak of 48,724 million CFA francs, followed by a considerable drop in 2017 to 37,276 million CFA francs, and then gradually rose to 48,359 million CFA francs in 2019.

Figure 17: Evolution of antimicrobial drug imports in value

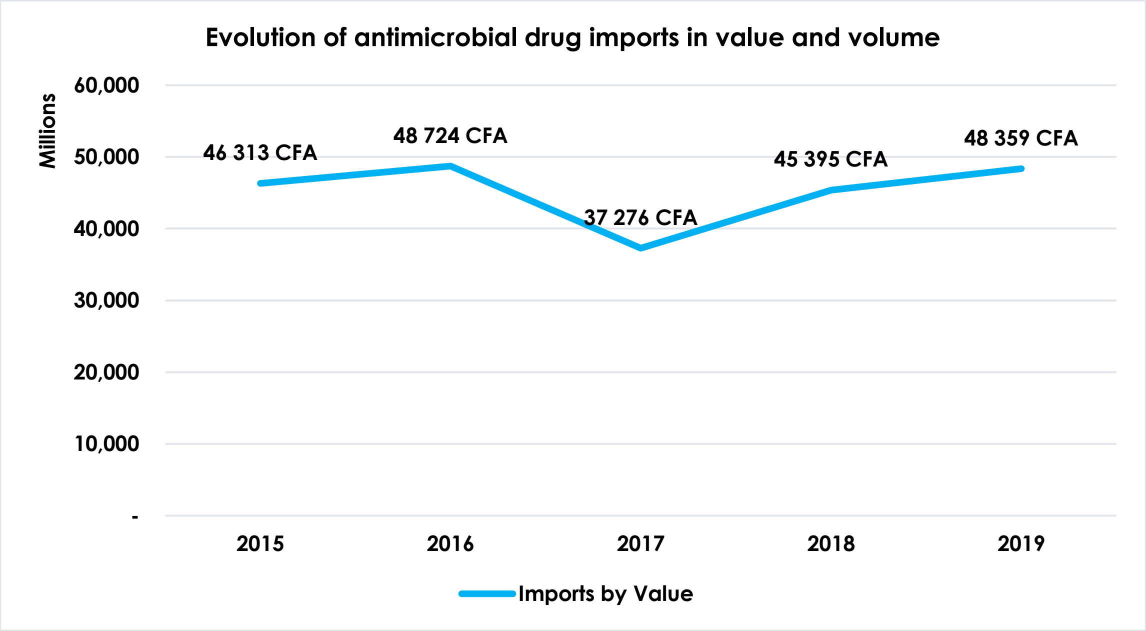

Source: IMS HEALTH

Overall, Senegal's imports of antimicrobial drugs over the last 5 years amount to 226,067 million CFA francs. These figures show the importance and the need to pay more attention to quality control of drug imports, especially antimicrobials.

Figure 18: Value of imports (millions F.CFA) of antimicrobial drugs by therapeutic family over the last 5 years

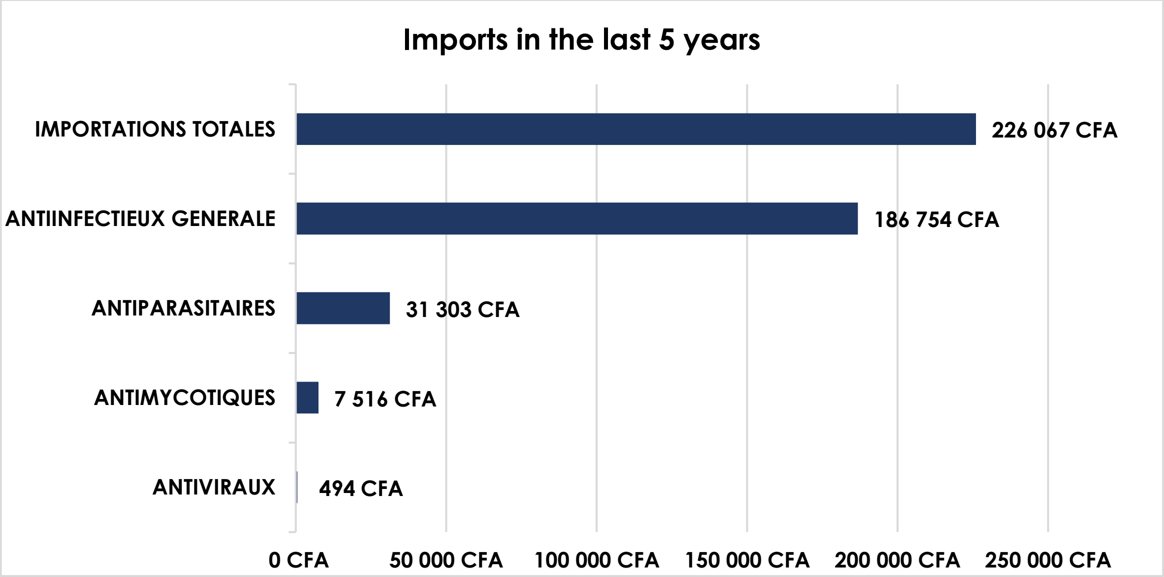

Source: IMS HEALTH

In the last 5 years, 196 antimicrobial molecules have been imported into Senegal.

Figure 19: Number of antimicrobial molecules imported in the last five years

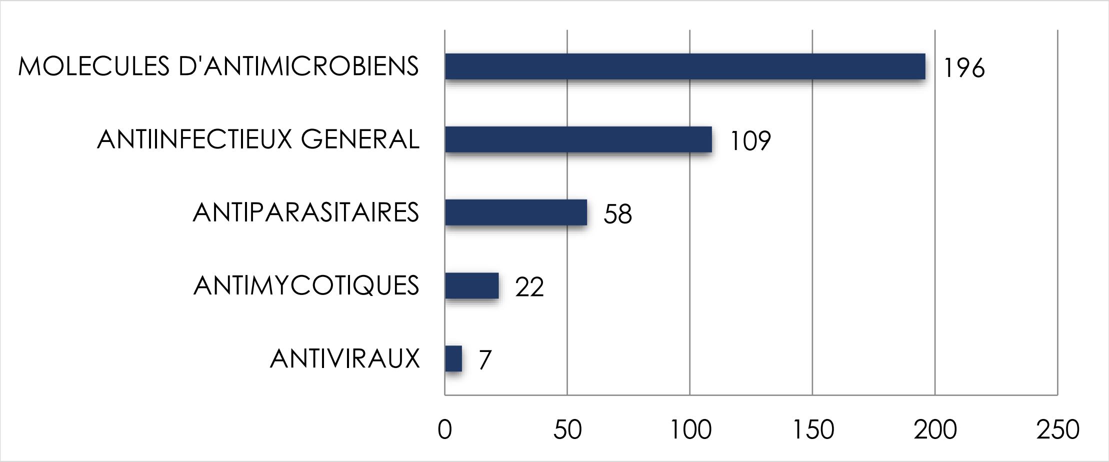

Source: IMS HEALTH

On average, antimicrobial drugs on the national essential drugs and products list account for 83% of annual imports over the past 5 years

Figure 20: Composition of drug imports

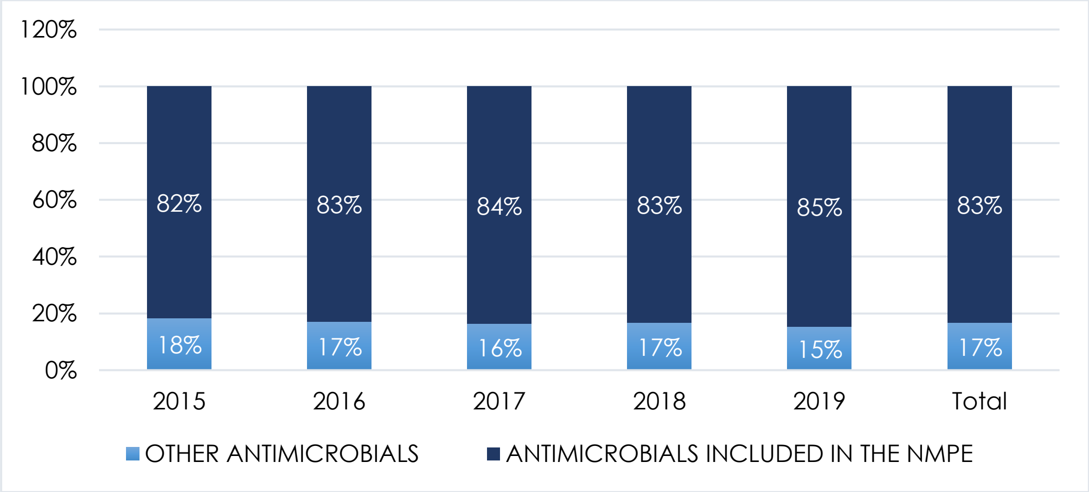

Source: IMS HEALTH

A detailed analysis of imports is presented in the Excel file "Total antimicrobial import data" attached to the rapid situational analysis report.

- **Findings on actual practices**

The existing quality assurance system is operational and includes the following checks:

- pre-qualification of suppliers,
- integration of antimicrobial test reports and GMP certificates on purchasing documentation,
- physical inspection of samples,
- monitoring the quality of imported antimicrobials,
- inspection at the point of import,
- monitoring and evaluation of supplier performance,
- control of the ambient parameters.

- Compilation and reporting of fake, falsely labeled, counterfeit, adulterated, and substandard antimicrobial drugs is not always systematic and is insufficient (1 in 3 wholesalers);
- Antimicrobial drugs on the national list of essential medicines and products are imported and distributed by private wholesalers-distributors (100% of wholesalers);
- The National Supply Pharmacy uses only the national list of essential drugs and products for the procurement of antimicrobial drugs;
- The import and distribution data of antimicrobial drugs are compiled in a single database for all references (100% of wholesalers);
- Private supply chain professionals have not received any capacity building on antimicrobial resistance and promotion of rational use of antimicrobial drugs.

Assets

- The organization of the supply chain allows antimicrobial drugs to be available and accessible in multi-source generics, branded generics and originator drugs;
- The traceability system for drug imports and sales. This positions supply chain actors as providers of antimicrobial use and consumption data;
- Logistics information on drug purchases provides comprehensive data such as product type (multi-source generic, branded generic, Princeps), volumes, manufacturers, lot numbers, expiration dates...).

Deficiencies

- Lack of coordination of antimicrobial drug supplies between the public and private sectors ;
- Administrative burdens in the procurement of medicines for the public sector ;
- Data from supply chain actors that may not reflect actual drug consumption, but rather import cycles;
- Private supply chain actors who may be reluctant to provide their consumption data, in the absence of a binding regulatory provision;
- Import files that are often a compilation of administrative and technical information that must be sorted and analyzed. This can be tedious;
- Data from the public purchasing office, which do not reflect the total consumption of medicines in the public sector, due to purchases made by some public health establishments without going through it.

Recommendations

- Develop a national procurement strategy to improve the availability and accessibility of quality antimicrobial drugs;
- Establish a partnership agreement with the reference laboratory (NML) for the import control of antimicrobial drugs;
- To assist the actors in the supply chain to comply with the requirements of the guide to good practice in the wholesale distribution of medicines;

- Create a framework for consultation between the actors in the supply chain for better coordination of antimicrobial drug orders;
- Establish a national logistics information management system for the importation and distribution of antimicrobial drugs;
- Train the staff of the supply chain actors on the issue of antimicrobial resistance, rational use of antimicrobial drugs and health data collection;
- Encourage supply chain actors to hire data managers.

6.1.2.2. Status of the antimicrobial supply chain in animal health

- **Imports**

The share of imports of veterinary drugs and vaccines amounts to 5,377,372,152 FCFA (five billion three hundred and seventy-seven million three hundred and seventy-two thousand one hundred and fifty-two FCFA) in 2019. The total quantity of antibiotic imported in 2019 is estimated at 14,461 kg and represents 36% of the value of imports.

Tetracyclines, with 7046 kg, are the main therapeutic class imported into Senegal. They are followed by sulfonamides (4,420 kg), macrolides (916 kg) and fluoroquinolones (665 kg).

- **Importing wholesalers**

In 2019, only 9 of the licensed veterinary import and wholesale distribution establishments imported. SEDIMA (hatchery) is licensed to import vaccines on behalf of its farms.

Table 12: Market shares of importing wholesalers of drugs and vaccines

Importing wholesalers	Values in CFA	Percentage share
Distrivet	72 030 638	1,4
General Vet	225 694 469	4,2
Global vet	635 067 393	11,8
SEDIMA	353 058 360	6,57
Senevet	2 720 710 225	50,59
Soprasen	19 016 193	0,32
Soprodel	1 295 256 628	24,08
Sosedel	45 274 808	0,84

Source: BMPV/DSV/MEPA

Findings on actual practices

- Only 15% of wholesale importers distribute medicines without complying with the legislation in force;

- Only 15% of importing wholesalers distribute antimicrobial drugs without complying with the legislation in force;
- 100% of importing wholesalers report distributing antimicrobials without market authorization;
- 57% of the sales of importing wholesalers are antimicrobial drugs;
- The sales of antimicrobials by importing wholesalers are around an average of 600 million CFA per year, which is on average 30% of their turnover;
- No importing wholesaler carries out quality control either at import or at storage, let alone at distribution;
- 85% of importing wholesalers receive feedback on potential efficacy, compliance and/or adverse reaction findings related to antimicrobial use;
- 57% of importing wholesalers report that they do not maintain an antimicrobial database;
- 85% of importing wholesalers report being trained in the rational use of antimicrobials.

Recommendations

- Supervision of the distribution circuit for veterinary medicinal products ;
- To monitor and supervise the facilities of para-veterinarians;
- Enforce the Pharmacy and Veterinary Clinic Act;
- Regulate the holding and use of antimicrobials;
- Require practitioners to report and report to DSV on antimicrobial use;
- Make a short list of first-line antibiotics;
- To provide an annual update on antibiotic entries into the country;
- Continue to raise awareness at all levels;
- To help outgoing veterinarians to settle in strategic areas of the country to avoid that the veterinary medicine is in the hands of a non professional;
- To assist the competent services in monitoring and controlling the distribution of veterinary medicines;
- Increase the dialogue between the actors and reinforce the control on the ground (laboratory analyses, importers' follow-up, etc.).

6.1.2.3. Review of the antimicrobial supply chain in plant health

Findings

- **On imports of plant protection products**

Imports of plant protection products were estimated at 738,197 Kg/Litre in 2013, reaching a peak of 814,648 Kg/Litre in 2015 and a significant decrease in the following years to 700,669 Kg/Litre in 2018.

In 2017, pesticide imports were estimated at 563,120 Kg/Litre (report from the DPV's phytosanitary legislation office).

Graph 21: Total imports of plant protection products in Kg/Litre

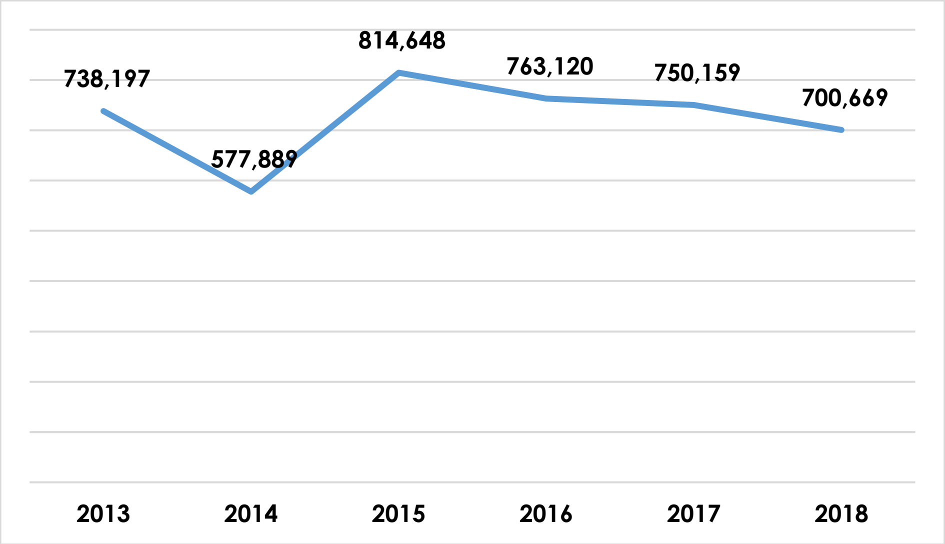

Source: Direction de la Protection des Végétaux (DPV)

Table 3 summarizes the use of active ingredients in the formulation of crop protection products from 2012 to 2014.

Table 13: Consumption of active ingredients used in the formulation of plant protection products

Main product types and chemical groups	Quantity of active ingredient consumed (metric tons)		
	2012	2013	2014
INSECTICIDES, (including acaricides, molluscicides and nematicides)	345,3	441,54	249,74
HERBICIDES, (including defoliants and desiccants)	375,1	169,57	270,1
FUNGICIDES AND BACTERICIDES	61,6	68,93	16
FUNGICIDES SEED TREATMENT	0,35	38,07	35,64
INSECTICIDES SEED TREATMENT	0,66	1,92	37,68
GROWTH REGULATORS	0,68	2,1	
RODENTICIDES	0,54	0,4	0,63

Source: Direction de la Protection des Végétaux (DPV)

Table 4 summarizes the imports of plant protection products and their use in agricultural practices recorded at the VPD level.

Table 14: Imports of plant protection products in 2018

Trade name of crop protection product	Product quantity	Unit	Usage
Titan 25 EC	2250	Liters	Field food crops
Pyrical 5	117	Tons	Food crops in aquatic environments
Azox	700	Liters	Food crops in aquatic environments
Sultan 480UL	6000	Liters	Field food crops
Pyrical 240UL	800	Liters	Field food crops
Pyrical 480 UL	2000	Liters	Food crops in aquatic environments

Queletox 640 UL			
Chlorocal 20	28000	Liters	Food crops in aquatic environments
Fenical 400UL	19800	Liters	

Source: Direction de la Protection des Végétaux (DPV)

In 2017 the status of applications for approval to import pesticides is as follows:

- 75 new applicants for accreditation
- 102 applications for renewal of approval for the sale and distribution of pesticides
- 10 applications for renewal of approval for disinsectisation, disinfection, rodent control and fumigation
- 248 permits issued
- 48 biological licences issued

The existence of a list of pesticides banned from sale and circulation throughout the country: Monocrotophos, Triazophos, Metamidophos, Fipronil, Paraquat, Furadan, Carbosulfan.

Significant quantities of counterfeit pesticides have been seized throughout the country.

Assets

- Existence of a system for monitoring and compiling import data on plant protection products
- Existence of a procedure for prior approval of importers of plant protection products (import approval)
- Compliance with import approval procedures for distribution and sale by formal actors
- Control of the sale of pesticides by obtaining prior registration from the CSP or a provisional authorization for sale issued by the DPV;
- Existence of local formulators of phytosanitary products

Deficiencies

- Well-developed informal channels (weekly markets, high border permeability)
- Easier access to fraudulent pesticides than registered ones
- Non-availability of registered pesticides at all locations
- Lack of legal information (information on the active substance, method of use and precautions for use)
- Irregular control of the marketing of plant protection products by the national plant protection services.
- Insufficient human and logistical resources to monitor the actors.
- Insufficient personal protective equipment by growers when using pesticides
- Reuse of empty packaging by producers or disposal in farms and landfills or burning and burning on site.

Recommendations

- Regular production of activity reports on formulations, imports, distributions and sales of plant protection products
- Extension of the list of pesticides banned from sale and circulation to formal actors
- Formalization of informal dealers and nationwide coverage of registered pesticide sales outlets
- Establishment of incentive-based trade policies to improve the affordability (price) of registered pesticides
- Strengthening the human and logistical resources of phytosanitary control posts and agricultural warning bases

6.2. ANALYSIS OF ACTUAL PRESCRIBING AND DISPENSING PRACTICES

6.2.1. Mapping of actors

6.2.1.1. Mapping antimicrobial prescribers and dispensers in the human health sector

The Prescribers

Antimicrobial prescribing facilities are present at all levels of the health pyramid. They are public and private hospitals, public and private clinics and peripheral health structures.

The main prescribers are:

- Doctors: medical prescriptions are governed by law 66-069 of 4 July 1966 on the practice of medicine and the medical association
- Dental surgeons: medical prescription is governed by law 1981-70 of 10 December 1981 on the practice of dental surgery and the order of dental surgeons
- Midwives: prescription is governed by Law No. 2017-14 of 20 January 2017 relating to the exercise of the Profession of Midwife in Senegal and establishing the Order of Midwives and Maieuticians
- Nurses

Antimicrobial dispensing facilities

- **Retail pharmacies**

According to the WHO report, Senegal has 1186 pharmacies in 2018. However, they are heavily concentrated in the most urban areas. Indeed, the Dakar region alone is home to 604 pharmacies in the country, or 51 percent of the total. It is followed respectively by the regions of Thiès (152), Diourbel (75), Saint Louis (69) and Kaolack (60).

The table below shows the number of pharmacies in each region, which illustrates the density in urban areas of the country.

Figure 6: Mapping of pharmacies

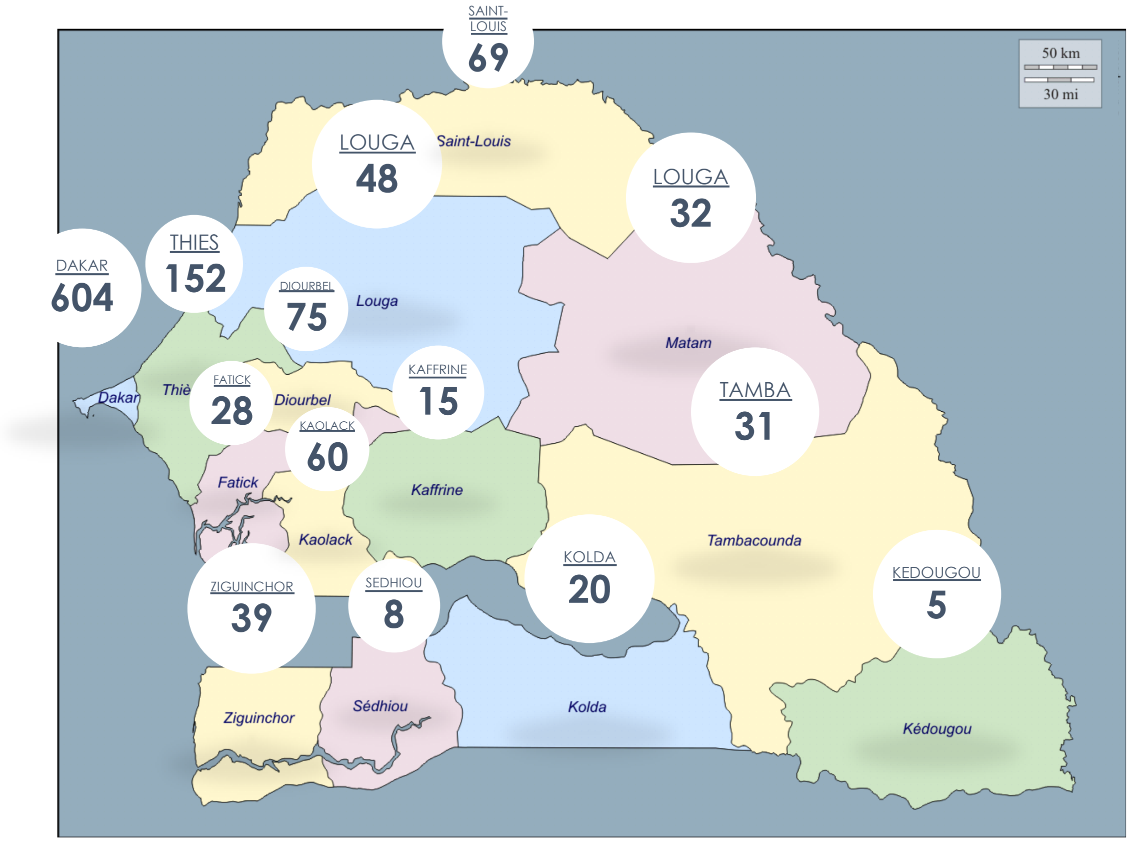

Source: Order of Pharmacists of Senegal

Internal use pharmacies (PUI)

The management and use of medicines in the hospital setting poses several challenges. The distribution of medicines varies between hospitals, either as soon as the patient is admitted, or with a prescription that must be filled before care is provided.

The route taken by medicines, from prescription to administration to the patient, is a process that most often involves the physician, pharmacist and nursing staff; it includes the following steps

- the receipt of the request from the care units,
- preparation of the order,
- delivery and dispensing,
- the safety of the drug circuit.

When this circuit is not controlled, it increases the risk related to the use of medicines and the risk related to the non-observance of treatments by patients.

Dispensing can be done in two ways depending on the nature of the drug and the associated risks.

- Nominative Delivery Dispensing (NDD) for medicines with a critical benefit/risk ratio and/or a risk of misuse, medicines with a narrow therapeutic index and medicines for which specific traceability and economic monitoring is required;
- Global Dispensing (DDG) for drugs that do not present a critical benefit/risk ratio and/or a risk of misuse, drugs with a broad therapeutic index and drugs with a stable and defined prescription.

Source: Guide to Good Hospital Practice - BPH

6.2.1.2. Mapping antimicrobial prescribers and dispensers in the animal health sector

The Prescribers

In actual practice, prescribers of veterinary drugs are :

- Veterinary doctors working in private practice (veterinary practices, clinics and pharmacies) as authorized by the regulations;
- Veterinary doctors and veterinary para-professionals not authorized by the regulations in force:
    - certain officials,
    - some employees of private structures outside the practices owned by veterinary doctors,
    - some NGO employees,
    - Paraprofessionals in private practice (veterinary nursing practices).

Dispensing structures

In actual practice, the dispensing of veterinary drugs is done by :

- Practices owned by veterinary doctors (licensed),
- Pharmacists (authorized),
- Veterinary nursing practices owned by ITEs and ATEs (not licensed).

6.2.2. State of the art of real practices

6.2.2.1. Review of actual prescribing and dispensing practices of antimicrobials in human health

Findings in antimicrobial prescribing practices (Evaluation of the quality of antibiotic prescribing in the Emergency Department of CHNU Fann by the Infectious Diseases Department: 2012)

In 2012, a study on the evaluation of the quality of antibiotic prescribing was conducted at the emergency department (SAU) of the CHNU de Fann by the infectious diseases department.

- **Evaluation criteria**
  - choice of molecule
  - dosage,
  - duration,
  - rhythm and
  - combination of antibiotics.
- **Methodology**
  - Collection of epidemiological, clinical, bacteriological and therapeutic data;
  - Assessment of the quality of antibiotic prescribing in accordance with national recommendations (see attached Antibiotic Guide, 2010 version)
  - Data analysis with epi-info 2000 software.
- **Results**

Table 15: Prevalence results and diagnostic data for antibiotic prescribing

Prevalence of antibiotic prescribing		
Number of patients consulted		1683
Number of patients who received antibiotic therapy		211
Number of antibiotic prescriptions		257
Frequency of antibiotic prescriptions		12,50%
Average age of patients		35,4 ±15
Pathological history	HTA	37,50%
	Asthma	33,30%
**Diagnostic data**		
Respiratory infections		128 cases / 60.7%.
Digestive pathologies		49 cases / 23.3%.
Urogenital pathology		15 cases / 7%.
Community-acquired pneumonia		21,80%
salmonellosis		15,20%
sore throats		11,40%
pharyngitis		9%

Source: Report on the evaluation of the quality of antibiotics at CNHU Fann

The evaluation of the quality of antibiotic prescribing gave the results presented in the tables below:

Table 16: Distribution of 257 prescriptions by antibiotic family

Antibiotic families	Workforce	Percentage (%)
β-lactams	115	45
Macrolides	72	28
Quinolones	54	21
5 nitro - imidazoles	15	6
Sulfonamides	1	0,4
**Total**	**257**	**100**

Source: Report on the evaluation of the quality of antibiotics at CNHU Fann

Table 17: Distribution of Appropriate Antibiotic Prescriptions by Assessment Criteria

Criteria for evaluating the prescription	Workforce	Percentage (%)
Choice of drug and indication (n=211)	114	54
Dosage (n=257)	175	68
Duration (n=257)	145	56
Rate of administration (n=257)	186	72
Route of Administration (n=257)	250	97
Antibiotic combination (n=45)	30	67

Source: Report on the evaluation of the quality of antibiotics at CNHU Fann

- **Discussions**
  - The most commonly prescribed antibiotics were amoxicillin (22.6%), azithromycin (19.8%), ciprofloxacin (16.7%) and amoxicillin-clavulanic acid (14.8%).
  - Prescriptions were appropriate in 54% of cases.
  - Inappropriate prescribed treatments included arthritis, bronchitis, gastritis, osteitis, pharyngitis and PID.
  - The percentage of underdosing noted is 32%.
  - Inappropriate dosing was most common for flucloxacillin, spiramycin, amoxicillin and amoxicillin-clavulanic acid.

Findings from actual practices

- Unfortunately, these rational use tools are not used enough. Indeed, the results of the interview showed that 67.7% of prescribers stated that they do not use rational use guidelines for antimicrobials on patients.
- Training sessions for health professionals are regularly organized but in an insufficient manner. Indeed, only one (1) prescriber out of ten (10) declared having benefited from or being aware of capacity building on the use of antimicrobials during the last three years.
- Information campaigns and campaigns to combat the dangers of using street drugs are also conducted by the DPM in collaboration with partners. Similarly,

information and awareness-raising campaigns have also been carried out, but have not reached many health professionals. In fact, only 16% of health professionals have benefited from them.

- When asked: Have you ever participated in a Global Antimicrobial Stewardship Week? All prescribers' answers were negative.
- Therapeutic education is far from being systematic. Because when asked "Have you implemented therapeutic education programs for patients on the use of antimicrobials, the dangers of misuse and the development of antimicrobial resistance?", only one (1) health professional out of four (4) declared that he/she practiced therapeutic education on patients.
- The use of susceptibility testing to better target the pathogen is a neglected practice among prescribers. The results show that 25% of antimicrobial prescribers use confirmatory laboratory tests for pathology before prescribing antimicrobials.
- The monitoring of the rational use of medicines is ensured in the framework of inspections. However, data reporting is almost non-existent despite the progress noted with the appearance of the high-performance health information system platform (DHIS2). The results of the study show that only 13% of health professionals report on antimicrobial prescribing.

Recommendations

- Establish antimicrobial use protocols at all levels;
- Build capacity and training on antimicrobial use ;
- Participate in training seminars on antimicrobials and treatment failures;
- Train all providers on antimicrobial standards and protocols;
- Have training guides for better knowledge of antimicrobials ;
- Provide districts with therapeutic protocols on antimicrobial prescribing;
- Increase public awareness of self-medication ;
- Establish an information channel for antimicrobial prescription updates;
- Update therapeutic protocols, especially in rural areas;
- Involve prescribers in all antimicrobial stewardship programs.

Findings from antimicrobial drug dispensing

Antimicrobial drugs are presented in the pharmacy under the names of specialties, generics, brands and hospital packaging (in boxes of 10 to 50 blister packs).

They are among the therapeutic families with the most generics (more than 75% of presentations are generics). Among the molecules presented in hospital packaging, we have: Ampicillin, Amoxicillin, Cotrimoxazole, Metronidazole, Ciprofloxacin, Erythromycin, Griseofulvin, the combination Sulfadoxine-Pyrimethamine.

Antimicrobial dispensing is done on a prescription, advisory and client request basis.

Prescriptions are for specialties, branded generics and hospital packaging, depending on the prescriber's qualifications.

The majority of prescriptions that come from private clinics and medical offices are for brand names. For public sector hospitals and health centres, all presentations are encountered.

The prescriptions of the health posts are mainly based on the International Non-proprietary Names (INN), with the particularity that they usually only bear the stamp of the structure (the name and the qualification of the prescriber are not mentioned).

Patient information is also sometimes not mentioned, especially when prescriptions come from health posts, which causes some difficulties at the time of dispensing.

Pharmacists dispense many antimicrobials on a consultative basis:

- Tooth pain with decay and inflammation of the gums,
- a sore throat, a superinfected wound,
- a cough with purulent sputum,
- a suspected urinary tract infection,
- an abscess,
- some forms of diarrhea, etc.

The choice of an antimicrobial depends on its spectrum of activity and the site of infection. It must be recognized that this is a rather random approach, without any control over the germ involved and the proposed treatment. Such an attitude is debated and contributes to the misuse of antimicrobials.

Antimicrobials such as Amoxicillin, Cotrimoxazole commonly known as Bactrim and to a lesser extent Griseofulvin and Sulfadoxine-Pyrimethamine are requested spontaneously all day long.

While some pharmacists take the trouble to check whether the request is justified, others systematically give the product. Added to the very low prices (from 250 FCFA to 1,000 CFA) of the blister packs, this behaviour of pharmacists encourages self-medication and misuse.

This is of particular concern because clients are not interested in the warnings of the drug professional whenever he or she tries to educate them about the importance of rational use of antimicrobials.

Observation of actual practices

- From the results of the interview guides, it appears that only 20% of the pharmacists who responded to the guide have implemented an antimicrobial dispensing traceability system.
- 85% of pharmacists responding to the interview guide offered an alternative treatment when antibiotics were not necessary.
- 65% of pharmacists do not provide therapeutic patient education on the rational use of antimicrobials.
- Only 30% of the pharmacists who responded to the interview guide have been trained in AMR.
- Nine out of 10 pharmacists responding to the interview guide stated that the blister pack format has a negative effect on rational use of antimicrobials.

In the hospital setting, the most commonly prescribed antibiotics are:

1 Amoxicilline/Acide Clavulanique,

2 Ceftriaxone,

3 Amoxicilline,

4 Métronidazole,

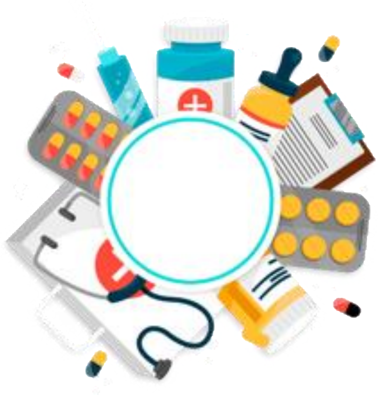

5 Ofloxacine,

6 Cotrimoxazole,

7 Ciprofloxacine.

Figure 22: Distribution of antimicrobials by health facility

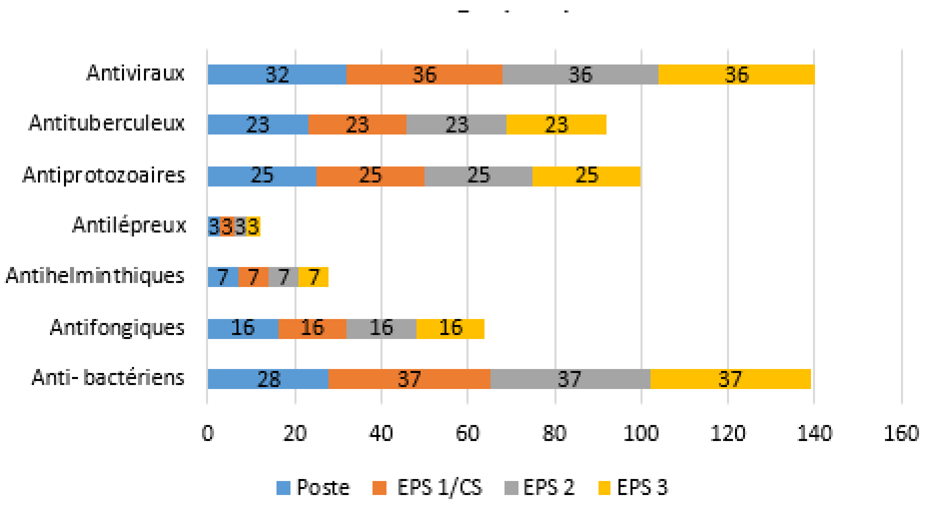

Source: National List of Essential Drugs and Products

Different situations of misuse may exist in the hospital environment:

- Unnecessary requirements ;
- Delayed antibiotic administration for patients with severe infections ;
- Broad-spectrum antibiotics used too often ;
- Narrow-spectrum antibiotics used inappropriately ;
- Antibiotic dosage below or above the appropriate dosage for a given patient;
- Treatment duration too short or too long.

Recommendations

- Undertake awareness-raising activities among prescribers and dispensers on the rational use of antimicrobials;
- Encourage prescribers and providers to educate patients about antimicrobial use and the importance of adherence;
- Inform all groups of prescribers and dispensers of factors that may have a strong influence on their prescribing habits, such as economic benefits, promotional activities and incentives from the pharmaceutical industry;
- Encourage the development and use of treatment guidelines and protocols for the proper use of antimicrobials;
- Establish hospital therapeutics committees to oversee antimicrobial use in hospitals;
- Develop and regularly update guidelines on antimicrobial treatment and prophylaxis;
- Controlling antimicrobial use and usage patterns ;
- Provide informed advice and appropriate written information when dispensing antimicrobials;
- Encourage patients to complete their treatment and, if this is not possible, to dispose of any unused antimicrobial drugs appropriately;

- Work with prescribers to ensure that there is sufficient stock to complete or continue any ongoing treatment;
- Recommend treatments other than antimicrobials for minor ailments;
- Integrate an educational component into antimicrobial drug promotion and marketing activities;
- Monitoring and controlling economic incentives for misuse of antimicrobial drugs ;
- Establish hospital therapeutics committees to oversee antimicrobial use in hospitals.

6.2.2.2. Review of actual antimicrobial prescribing and dispensing practices in animal health

The following practices emerged from the interview guides:

Prescribing practices of veterinary doctors (veterinary practices)

- 37.5% of veterinary doctors dispense antimicrobials (in any form) to farmers and animal care workers without a prescription and without consulting their animals first;
- 60% of veterinary doctors do not use laboratory analyses, including antibiograms, before applying antibiotics;
- 50% of veterinary doctors report prescribing human antimicrobials for the purpose of treating animals;
- 80% of veterinary doctors inform the veterinary authority or suppliers of any efficacy, compliance and/or adverse reaction findings related to antimicrobial use;
- 50% of veterinary doctors have received capacity building on antimicrobial stewardship;
- 80% of veterinary doctors report being aware of the OIE AMR strategy;
- 53% of veterinary doctors report using animal health antimicrobial guidelines.

Prescription practices of para-professionals (health care offices)

- 54% of veterinary para-professionals say they do not use laboratory tests, including antibiotic susceptibility tests, before starting antibiotic treatment
- 55% of para-professionals report using human antimicrobials to treat animals when veterinary specialties are not available
- 80% of paraprofessionals report informing the veterinary authority about potential efficacy, compliance, and/or adverse effects findings related to antimicrobial use in the field
- 45% of paraprofessionals reported not having received capacity building on antimicrobial stewardship and use
- 36% of paraprofessionals report not using antimicrobial guidelines

Antimicrobial use practices in animal feed

- The authorization of the use of antimicrobials in feed as growth promoters in animal production is not unanimously supported by the feed industry. In fact, 75% of the feed industry is aware that the use of antimicrobials in feed as growth promoters in animal production is not permitted.
- The authorization of the use of antimicrobials in feed as growth promoters in animal production is not unanimously supported by the feed industry. In fact, 75% of the feed industry is aware that the use of antimicrobials in feed as growth promoters in animal production is not permitted.
- 25% of poultry feed manufacturers report incorporating anticoccidial antimicrobials in some feeds as a growth promoter except for finishing. However, they do not label the types of antimicrobials incorporated or their levels

The practices of public service agents

- All the public service agents declare that they are solicited by breeders for therapeutic procedures on their animals. They all accept to provide services to breeders
- 90% of the public service agents solicited for the administration of antimicrobials declare that they always take the precaution of verifying whether this medication was prescribed by an authorized person or structure
- Nearly 20% of public servants report using human antimicrobials for the purpose of treating animals
- 80% of public officials report informing their supervisors about potential efficacy, compliance, and/or adverse events related to antimicrobial use in the field
- Only 10% of these public officials reported receiving capacity building on antimicrobial stewardship and use

Recommendations

- Collaboration between veterinary doctors and animal health technicians
- Strengthening the fight against fraudulent practices in animal health
- Strict regulations on antimicrobial prescribing and dispensing practices
- Establish functional bodies to control and monitor the sale of antimicrobials
- Increase awareness of the harm of self-medication in animal production
- Implement regulation (licensing) of veterinary drug prices

6.2.2.3. Inventory of actual practices in plant health

Findings of actual practices (survey of 90 producers in the Niayes area in the Thiès region conducted in November 2019)

- 90 do not measure product efficacy over time to monitor for resistance
- 72 treat as needed without following a treatment protocol
- 1only uses organic products
- 90 use registered products
- 90 use the same treatment products for several campaigns without diversifying because of limited supply
- 90 know the product for each plant disease
- 86 do not adhere to treatment schedule (evening), target is not met, or receive treatment at low dose
- 90 noted that some products are no longer effective on targets, however no study has shown whether this is due to resistance or product quality
- 46 say they have increased the treatment dose to get the same result as 4 years ago (3.5 caps instead of 2 caps)
- 89 do not know the active ingredient of plant protection products or their toxicity, they trust the sellers.
- The level of schooling is very low with only 7 having reached secondary school.

Assets

- All producers surveyed use registered products
- All the producers surveyed know the product for each plant disease

Deficiencies

- All the producers surveyed do not evaluate possible resistance due to the use of phytosanitary products
- The vast majority of producers surveyed do not use treatment protocols
- Almost all the producers surveyed do not use alternative methods to plant protection products
- Most of the producers surveyed do not have a good understanding of good plant treatment practices (doses, targets, schedules, diversification, composition, toxicity)
- Low progressive effectiveness of some products

Recommendations

- Training of actors (producers) on good practices
- Promotion of the use of alternative methods
- Periodic evaluation of farming practices and the impact of the use of plant protection products

7. CONCLUSION AND RECOMMENDATIONS

7.1. Conclusion

The rapid situational analysis of the regulation and control of antimicrobials in the human, animal, plant and environmental health sectors showed overall the strengths and weaknesses of the legislative and regulatory corpus and the quality control system for antimicrobial drugs in Senegal.

In the human and animal health sectors, the regulatory and control mechanisms for antimicrobials are functional and operational. On the other hand, in the plant and environmental health sectors, there is a lack of a regulatory framework and a lack of operational quality control of antimicrobials.

A good rationalization of prescriptions and dispensing of antimicrobial drugs, the implementation of therapeutic education programs for patients, and the eradication of unlicensed drug outlets could help to secure, control and optimize the consumption of antimicrobial drugs.

7.2. Recommendations

In view of the strengths and weaknesses identified in the rapid situational analysis, we make the following general recommendations common to the various sectors concerned by the study.

7.2.1. Recommendations regulatory framework and quality control of antimicrobials

- Institutional transformation of the LNCM to achieve administrative and financial autonomy, with a clear legal status
- Position the laboratories (NMLC, LACOMEV and CERES LOCUSTOX) as national reference laboratories for monitoring antimicrobial quality, as part of the national antimicrobial consumption monitoring program
- Develop the partnership between reference laboratories in human and animal health and agriculture
- Establish operational teams to monitor antimicrobial consumption

7.2.2. Recommendations on the rational use of antimicrobials

- Establish a partnership agreement with reference laboratories (NMLC, LACOMEV, CERES) for the quality control of antimicrobial drugs
- Supporting supply chain actors in complying with good practice guidelines for wholesale distribution of medicines and agricultural practices
- Create a framework for consultation between the actors in the supply chain for better coordination in ordering antimicrobial drugs
- Encourage all stakeholders involved in rational use of antimicrobials to recruit data managers
- Make collection of antimicrobial consumption data mandatory at all levels
- Regulate the holding and use of antimicrobials
- Making a short list of first-line antibiotics in animal health

- Increasing consultation between actors and strengthening control on the ground
- Establish therapeutic protocols and guidelines for rational use of antimicrobials in the sectors concerned
- Build capacity and train all providers on rational use of antimicrobials
- Increase public awareness of self-medication
- Establish an information channel for antimicrobial prescription updates
- Involve prescribers in all antimicrobial stewardship programs

8. BIBLIOGRAPHY

[1] .	Agreement on "Common Regulations on the Registration of Pesticides in CILSS Member Countries
[2] .	International Plant Protection Convention (IPPC)
[3] .	Basel Convention on the Transboundary Movement of Hazardous Wastes of 22 March 1989
[4] .	African Convention on the Prohibition of the Import into Africa of Hazardous Wastes in all Forms and the Transboundary Control of such Wastes Generated in Africa;
[5] .	Rotterdam Convention on the Principle of Prior Informed Consent (PIC);
[6] .	Stockholm Convention on Persistent Organic Pollutants (POPs);
[7] .	FAO International Code of Conduct on the Distribution and Use of Pesticides
[8] .	Code of Ethics on International Trade in Chemicals of April 1994
[9] .	International Code of Conduct on the Distribution and Use of Pesticides
[10] .	London Guidelines for the Exchange of Information on Chemicals in International Trade UNEP/GC/17
[11] .	Regulation No.⁰ 06/2010/CM/UEMOA on procedures for the approval of medicinal products for human use in WAEMU Member States
[12] .	Law 73-62 of 19 December 1973 on the Order of Pharmacists of Senegal
[13] .	Law n° 54-418 of 15 April 1954 applying the Public Health Code (Book V) in the Overseas Territories
[14] .	Law No. 98-12 of 2 March 1998 on hospital reform
[15] .	Law 2008-07 of 24 January 2008 on the organisation of the public animal health sector
[16] .	Law n°84-14 of February 2, 1984 relating to the control of agri-pharmaceutical specialities and similar specialities
[17] .	Law No. 2001-01 of 15 January 2001 on the Environment Code;
[18] .	Law n° 92-52 of 10 July 1992 creating the Order of Veterinary Doctors of Senegal
[19] .	Decree n⁰ 2004-1404 of 04 November 2004 on the organization of the Ministry of Health and Medical Prevention
[20] .	Decree n° 95-645 of 6 July 1995, grants the execution of the health mandate to private veterinary doctors who request it

[21] .	Decree n°84- 503 of May 2, 1984 on the application of the law n°84-14 of February 2, 1984 relating to the control of agro pharmaceutical specialities and similar specialities
[22] .	Decree No. 93-514 of 27 April 1993 on the Code of Ethics for Veterinary Medicine,
[23] .	Decree No. 95-645 of 6 July 1995 instituting the health mandate granted to private veterinary doctors who apply for it and intended for the execution of collective prophylaxis operations against certain animal diseases
[24] .	Order No. 47-47 of 22 April 1971 on the regulation of packaging used for the packaging of agricultural pesticides formulated in Senegal
[25] .	Order n° 05381 of 20 May 1985 which sets the composition and the rules of organization of the National Commission for the Approval of Agro-Pharmaceutical Specialities and Similar Specialities
[26] .	Ministerial order n° 10777 of August 4, 1992 creating an intermediary of receipts in the Ministry of Rural Development and Hydraulics relating to the control of the Agro pharmaceutical specialities and the Assimilated Specialities
[27] .	Order No. 000149 of January 11, 1994 appointing the manager of the revenue fund entitled Control of Agro-Pharmaceutical Specialties and Similar Specialties
[28] .	Inter-ministerial decree n° 10390 of December 2, 1994 fixing the amount of the royalty relating to the approval of the Agro pharmaceutical Specialities and the Assimilated Specialities.
[29] .	Prime Ministerial Order No. 005161 of 26 May 1995 (National Commission for Sustainable Development) ;
[30] .	Ministerial Order N°000852 of 08 February 2002 (National Chemicals Management Commission) ;
[31] .	Ministerial Order N°3504/MEA dated 09 May 2001 establishing a "*National Committee to monitor the programme to promote the intrinsic quality (pesticide residues) of fruit and vegetables for export*", chaired by the Director of Cabinet of the Ministry of Agriculture.
[32] .	Order No.⁰ 005711 of July 7, 2004 creating the Antibiotics Commission
[33] .	Arrêté n⁰ 05808 du 03 Juillet 2008 portant création du comité national d'antibiothérapie
[34] .	Order No.⁰ 16917 of July 20, 2018 on the National List of Essential Medicines and Products
[35] .	Order n⁰ 4012 of February 6, 1998 on the National Pharmacovigilance System
[36] .	Order n0 5711 of July 7, 2004 on the creation of Committees for the Fight against Nosocomial Infections
[37] .	Circular n0 00310 of February 10, 2011 on the procedures for the approval of medicines for human use

[38] .	Collection of legislative and regulatory texts on pharmacy in Senegal
[39] .	http://www.fao.org
[40] .	https://www.memoireonline.com/12/09/3065/Evaluation-des-methodes-de-controle-phytosanitaire-et-qualite-des-produits.html
[41] .	Implementation of Regional Pesticide Policies in West Africa: Report of the Case Study in Senegal
[42] .	FAO Action Plan on Antimicrobial Resistance 2016-2020
[43] .	Global Action Plan to Combat Antimicrobial Resistance WHO
[44] .	Global Action Plan to combat antimicrobial resistance : Report of the Secretariat
[45] .	Pest and Pesticide Management Plan of the Regional Support Project for the Sahel Irrigation Initiative (PARIIS)
[46] .	Integrated Vector and Pesticide Management Plan for the REDISSE SENEGAL Project: Interim Report
[47] .	Senegal Agricultural Market Development Program: Pest and Pesticide Management Plan
[48] .	Programme de Renforcement Des Systèmes Régionaux De Surveillance de Maladies (PRSRSM)
[49] .	Integrated Water Resources Management and Multiple Use Development Project in the Senegal River Basin (PGIRE)
[50]	Summary report of the May 22-31, 2018 MPD antimicrobial consumption profile survey mission.
[51]	Report on the monitoring of the quality of geo-referenced vegetable products in the Niayes zone in 2017
[52]	Report on the establishment of the reference situation on the environmental chemical risk (water and soil) and on the sanitary quality related to synthetic chemical pesticides of vegetable products (cabbage, bitter eggplant, onion, pepper, lettuce, carrot, potato, leek, and tomato) in the Niayes area
[53]	Report on the analysis of pesticide residues in water and imported, exported and locally produced horticultural products
[54]	Annual report office of plant protection legislation of the DPV in 2017

9. ANNEX

Table 18: Distribution of controlled molecules according to their quantity per batch unit in 2017

Molecules	Molecular batches	
	Number	Proportion(%)
Lamivudine/Zidovudine/Nevirapine	80	29,3
Lopinavir/ritonavir	68	24,9
Lamivudine/Zidovudine	41	15,0
Rifampicin/isoniazide	25	9,2
Isoniazid	17	6,2
Sulfamethoxazole and Trimethoprim	16	5,9
Sulfadoxine/Pyrimethamine	11	4,0
Artemether/Lumefantrine	10	3,7
Amoxicillin	3	1,1
Chorhexidine Digluconate	2	0,7
**Grand total 2017**	**273**	**100**

Source: National Drug Control Laboratory (NDCL)

Table 19: Distribution of controlled molecules according to their quantity per batch unit in 2018

Molecules	Molecular batches	
	Number	Proportion(%)
Lopinavir/ritonavir	9	26,5
Cefotaxime	2	5,9
Ciprofloxacin	2	5,9
pyrazinamide	2	5,9
Sulfamethoxazole and Trimethoprim	2	5,9
Amoxicillin	1	2,9
Ampicillin	1	2,9
Artemether	1	2,9
**Grand Total 2018**	**34**	**100**

Source: National Drug Control Laboratory (NDCL)

Table 20: Distribution of controlled molecules according to their quantity per batch unit in 2019

Molecules	Molecular batches	
	Number	Proportion(%)
CCM Anti-malarial	62	32,8
Amoxicillin	9	4,8
Amoxicillin/clavulanate suspension buv	6	3,2
Albendazole	4	2,1
Artesunate/Amodiaque	4	2,1
Sulfadoxine/Pyrimethamine	4	2,1
Artemether/Lumefantrine	3	1,6
Metronidazole Tablet	2	1,1
Metronidazole Infusion	1	0,5
**Grand Total 2019**	**189**	**100**

Source: National Drug Control Laboratory (NDCL)

Table 21: Summary of standards on MRL1 (mg a.i./kg) by couple of vegetable speculation and active ingredient of pesticide for an assessment of the sanitary risk

Speculation / Market gardeners / Active ingredient	Bitter eggplant	Eggplant	Carrot	Cabbage	Mint leaf	Green Bean	Turnip	Onion	Potato	Leek	Pepper	Tomato
Aldrin	0,01	0,01	0,01	0,01	0,01	0,01	0,01	0,01	0,01	0,01	0,01	0,01
Bifenthrin	0,01	0,01	0,01	0,4	0,01	0,01	0,01	0,01	0,01	0,01	0,01	0,3
Chlorpyrifos	0,01	0,01	0,1	0,01	0,01	0,01	0,01	0,2	2	0,01	0,01	0,01
Cypermethrin	0,03	0,03	0,05	1	0,01	0,05	0,05	0,01	0,05	0,05	0,5	0,2
DDT	0,01	0,01	0,01	0,01	0,01	0,01	0,01	0,01	0,01	0,01	0,01	0,01
Deltamethrin	0,01	0,01	0,02	0,01	0,01	0,01	0,01	0,05	0,01	0,2	0,01	0,3
Dicofol	0,01	0,01	0,01	0,01	0,01	0,01	0,01	0,01	0,01	0,01	0,01	0,01
Dieldrine	0,01	0,01	0,01	0,01	0,01	0,01	0,01	0,01	0,01	0,01	0,01	0,01
Endrin	0,01	0,01	0,01	0,01	0,01	0,01	0,01	0,01	0,01	0,01	0,01	0,01
Esfenvalerate	0,01	0,01	0,01	0,01	0,01	0,01	0,01	0,01	0,01	0,01	0,01	0,01
Fenvalerate	0,01	0,01	0,01	0,01	0,01	0,01	0,01	0,01	0,01	0,01	0,01	0,01
Heptachlor	0,01	0,01	0,01	0,01	0,01	0,01	0,01	0,01	0,01	0,01	0,01	0,01
Lambda-Cyhalothrin	0,5	0,5	0,02	0,5	0,01	0,05	0,01	0,2	0,02	0,1	0,3	0,1
Lindane	0,01	0,01	0,01	0,01	0,01	0,01	0,01	0,01	0,01	0,01	0,01	0,01
Malathion	0,02	0,02	0,02	0,02	0,01	1	0,2	1	0,02	0,02	0,02	0,1
Parathion	0,05	0,05	0,05	0,05	0,01	0,05	0,05	0,05	0,05	0,05	0,05	0,05
Profenofos	0,01	0,01	0,01	0,01	0,01	0,01	0,01	0,01	0,01	0,01	0,01	10
Dimethoate	0,02	0,02	0,02	0,2	0,01	0,02	0,1	0,02	0,05	0,02	0,5	0,02
Trifluralin	0,01	0,01	0,01	0,01	0,01	0,01	0,01	0,01	0,01	0,01	0,01	0,01

[1]**MRL:** Colourless: (Codex Alimentarius MRL), Yellow: (EU MRL), Green (default MRL)

Source: Report on the monitoring of the quality of geo-referenced market garden produce in the Niayes area in 2017

Table 22: Pesticide residue levels found by vegetable crop and by locality

Location	Speculation<br>Sampled market garden	Pesticides	CILSS approval status[1]	Results (mg/kg)	MRL (mg/kg)	Number of overruns of the MRL
1. Keur A Ndoye 1	Leek	Chlorpyrifos	YES	0,4	0,01	40
		Dicofol	NO	3,6	0,01	360
		Lambda-cyhalothrin	YES	0,1	0,1	-
		Dimethoate	YES	0,03	0,02	1,5
		Trifluralin	NO	0,1	0,01	10
2. Keur A Ndoye	Leek	Dicofol	NO	0,05	0,01	5
		Trifluralin	NO	0,01	0,01	-
3. Kayar	Bitter eggplant	Bifenthrin	YES	0,1	0,01	10
		Lambda-cyhalothrin	YES	0,15	0,5	0,3
		Dimethoate	YES	0,4	0,2	2
4. Bayar/Beer	Tomato	Dicofol	NO	0,4	0,3	1,33
		Lambda-cyhalothrin	YES	0,16	0,1	1,6
		Dimethoate	YES	0,16	0,02	8
5. Gorom 1	Cabbage	Bifenthrin	YES	0,2	0,4	0,5
		Chlorpyrifos	YES	0,07	0,01	7
		Dicofol	NO	0,2	0,01	20
		Dimethoate	YES	0,1	0,2	0,5
6. Gorom 2	Onion	Dicofol	NO	0,24	0,01	24
		Lambda-cyhalothrin	YES	0,29	0,2	1,45
		Dimethoate	YES	0,29	0,02	14,5
7. Bambilor/Dene 1	Mint leaf	Dimethoate	YES	0,05	0,05	-
		Dicofol	NO	0,03	0,01	3
		Dimethoate	YES	0,15	0,01	15
8. Bambilor/Dene 2	Green bean	Dicofol	NO	0,03	0,01	3
		Dimethoate	YES	0,15	0,02	7,5
9. Kounoune	Green bean	Chlorpyrifos	YES	0,08	0,01	8
		Dicofol	NO	0,4	0,01	40
		Dimethoate	YES	1	0,02	50
10. Kounoune	Green bean	Bifenthrin	YES	0,16	0,01	16
		Lambda-cyhalothrin	YES	0,16	0,05	3,2
11. Sangakam 1	Green bean	Lambda-cyhalothrin	YES	0,5	0,05	10
		Dimethoate	YES	1	0,02	50
12. Dara	Cabbage	Dimethoate	YES	1,5	0,05	30
13. Mbidiem	Pepper	Dicofol	NO	0,04	0,01	4

		Dimethoate	YES	1	0,5	2
14. Ndiender	Onion	Bifenthrin	YES	0,07	0,01	7
		Chlorpyrifos	YES	0,1	0,2	0,5
		Dicofol	NO	0,2	0,01	20
		Lambda-cyhalothrin	YES	0,09	0,2	0,45
15. Keur MbirNdao	Onion	Bifenthrin	YES	0,2	0,01	20
		Dicofol	NO	0,07	0,01	7
		Dimethoate	YES	1	0,02	50
		Trifluralin	NO	0,05	0,01	5
16. SanthiouNdong 1	Bitter Eggplant	Dicofol	NO	0,07	0,01	7
		Dimethoate	YES	1	0,02	50
17. SanthiouNdong	Pepper	Dicofol	NO	0,03	0,01	3
		Dimethoate	YES	1,4	0,5	2,8
18. Ndiop Sao	Tomato	Dimethoate	YES	0,15	0,02	7,5
19. Mboro	Turnip	Dimethoate	YES	0,17	0,1	1,7
		Trifluralin	NO	0,03	0,01	3
20. Notto	Cabbage	Bifenthrin	YES	0,3	0,04	7,5
		Chlorpyrifos	YES	0,1	0,01	10
21. Keri	Onion	Bifenthrin	YES	0,06	0,01	6
		Chlorpyrifos	YES	0,03	0,2	0,15
		Dicofol	NO	2	0,01	200
22. Gueyenne	Bitter Eggplant	Dicofol	NO	0,06	0,01	6
23. Diamballo	Pepper	Dicofol	NO	0,03	0,01	3
24. Darou Lhoudoss 1	Onion	Dicofol	NO	0,08	0,01	8
		Lambda-cyhalothrin	YES	0,3	0,2	1,5
25. Darou Khoudoss 2	Onion	Trifluralin	NO	0,7	0,01	70
26. KhonkheYoye 1	Bitter Eggplant	Dicofol	NO	0,3	0,01	30
		Trifluralin	NO	0,13	0,01	13
27. Teureul 1	Bitter Eggplant	Dicofol	NO	0,03	0,01	3
		Dimethoate	YES	0,14	0,01	14
		Trifluralin	NO	0,02	0,01	2
28. Burial 2	Tomato	Dicofol	NO	0,06	0,01	6
		Dimethoate	YES	1,1	0,02	55
29. Thiouougne	Cabbage	Dimethoate	YES	1,4	0,2	7
30. Potou 1	Carrot	Dimethoate	YES	1,1	0,02	55
31. Mouril	Tomato	Dimethoate	YES	0,93	0,02	46,5
32. Ring 1	Tomato	Dimethoate	YES	1,01	0,02	50,5
33. Sole	Bitter Eggplant	Dimethoate	YES	1	0,02	50
	Carrot	Bifenthrin	YES	0,04	0,01	4

34. Rao Peulh		Lambda-cyhalothrin	YES	0,09	0,02	4,5
		Dimethoate	YES	1,4	0,02	70
35. FassNgom 2	Bitter Eggplant	Bifenthrin	YES	0,12	0,01	12
		Dicofol	NO	0,1	0,01	10
		Lambda-cyhalothrin	YES	0,06	0,5	0,12
36. FassNgom 3	Carrot	Dicofol	NO	0,09	0,01	9
		Dimethoate	YES	0,08	0,02	4
37. Tebene	Carrot	Bifenthrin	NO	0,07	0,01	7
		Lambda-cyhalothrin	YES	0,04	0,02	2
		Dicofol	NO	0,11	0,01	11
		Dimethoate	YES	0,3	0,02	15
[1]CILSS registration of Bifenthrin only on cotton, Chlorpyrifos on various crops, Dimethoate only on tomato, Lambda-Cyhalothrin only on cotton, tomato, in public health and against the desert locust						

Source: Report on the monitoring of the quality of geo-referenced market garden produce in the Niayes area in 2017

Table 23: Collection sheet

ANTIMICROBIAL CONSUMPTION DATA COLLECTION FORM									
ATC Code	DCI	Name of the presentation	Pharmaceutical form	Dosage	Route of administration	Package size	Number of boxes sold in 2015	Number of boxes sold in 2016	Number of boxes sold in 2017

Table 24: List of antimicrobials with adverse reactions

Therapeutic class	Antimicrobials	Number of reactions
Anti-malarial	Amodiaquine	1579
Anti-malarial	Sulfadoxine pyrim	1338
Anti-malarial	Amodiaquine;Pyrimethamine;Sulfadoxine	266
Pest control	Praziquantel	47
Antibiotic	Cotrimoxazole	28
Anti-malarial	Amodiaquina	20
Pest control	Albendazole	20
Anti-malarial	Amodiaquine;Artesunate	20
Anti-malarial	Sulfadoxine	19
Pest control	Albendazol	18
Anti-malarial	Sulfadoxine pyrimethamine	16
Anti-malarial	Pyrimethamine;Sulfadoxine	12
Pest control	Alben	7

Anti-malarial	Sulfadoxina w/ Pirimetamina	6
Antibiotic	Amoxicillin	6
Antibiotic	Cotrimoxazol	5
Antibiotic	Augmentin	4
Antibiotic	Metronidazole	3
Antibiotic	Ciprofloxacin	3
Antifungal	Grisefuline	3
Pest control	Mebendazole	2
Pest control	Meben	2
Antibiotic	Bactrim forte	2
Antibiotic	Cotrimaxazole	2
Anti-malarial	Fansidar	2
Antibiotic	Ampicillin	2
Antibiotic	Erythromycin	2
Antibiotic	Augmentin paediatric	1
Antituberculosis	Ethambutol	1
Antibiotic	Ospen	1
Antibiotic	Metronidazol	1
Anti-malarial	Sulfamethoxazole;Trimethoprim	1
Pest control	Combivir	1
Antibiotic	Rivamox	1
Antibiotic	Azithromycin	1
Antituberculosis	Isoniazid;Rifampicin	1
Antituberculosis	Isoniazid	1
Antibiotic	Erythromycin	1
Antifungal	Nystatin	1
Antibiotic	Streptomycin	1
Anti-malarial	Sulfamethoprim	1
Antituberculosis	Rifampicin	1

Source: Directorate of Pharmacy and Medicines
