# Supplementary material for: Landscape Analysis of Existing Policies, Laws and Regulations on Antimicrobial Stewardship in 10 Low- and Middle-Income Countries
Source: Antibiotics (Basel). 2026 Aug 10;15(8):770. doi: 10.3390/antibiotics15080770 (PMC13510074; doi:10.3390/antibiotics15080770)

USAID MEDICINES, TECHNOLOGIES, AND PHARMACEUTICAL SERVICES (MTaPS) PROGRAM

Improved Access. Improved Services. Better Health Outcomes.

Rapid Situational Analysis of AMS Policies, Legislation, Regulations, and Guidelines in Human and Animal Health

Tanzania

September 2022

USAID
FROM THE AMERICAN PEOPLE

This document is made possible by the generous support of the American people through the US Agency for International Development (USAID) contract no. 7200AA18C00074. The contents are the responsibility of Management Sciences for Health and do not necessarily reflect the views of USAID or the United States Government

Recommended Citation

Romuald Mbwasi and Augustine B. Matondo. *Rapid Situational Analysis Report of current AMS Policies, Legislation, Regulations, and Guidelines for Animal and Human Health in Tanzania Mainland*. September 2022. Prepared for the US Agency for International Development by the USAID Medicines, Technologies, and Pharmaceutical Services (MTaPS) Program. Arlington, VA: Management Sciences for Health, Inc.

ACKNOWLEDGEMENTS

The US Agency for International Development (USAID) Medicines, Technologies, and Pharmaceutical Services (MTaPS) Program would like to acknowledge the support of the Ministry of Livestock and Fisheries and the secretariat of the multisectoral technical group for the National Action Plan on Antimicrobial Resistance (NAP-AMR) strategic objective 4 (optimize the use of antimicrobial agents in human and animal Health) for the provision of reports and relevant documents and exchange of information to support the review. MTaPS also recognizes the readiness and cooperation received from the Tanzania Medicines and Medical Devices Authority (TMDA), the Pharmacy Council, the Veterinary Council of Tanzania, the Tanzania Dairy Board, the Tanzania Meat Board, the Directorate of Grazing Land and Animal Feed Resources and the Legal Service Unit of the Ministry of Livestock and Fisheries, and the Directorate of Veterinary Services, as well as all stakeholders from the Government and private entities listed in annexes 1A–C for their support and contribution to this report.

Special acknowledgement goes to Mrs. Siana Mapunju, former AMR focal person, Mrs. Emiliana Francis, the current AMR focal person at the Ministry of Health, and Dr. Gibonce Kayuni, AMR focal person at the Ministry of Livestock and Fisheries, for their support. Lastly, the financial support offered from USAID through its implementing partner, MTaPS, and the immediate technical and logistical support received from Dr. Talhiya Yahya, Senior Technical Advisor–AMS; Dr. Edgar Lusaya, USAID MTaPS Country Project Director; Kate Kikule, Principal Technical Advisor–Pharmaceutical Regulatory Systems; and other MTaPS country, regional, and head office staff are highly appreciated.

About the USAID MTaPS Program

The USAID Medicines, Technologies, and Pharmaceutical Services (MTaPS) Program enables low- and middle-income countries to strengthen their pharmaceutical systems, which is pivotal to higher-performing health systems. MTaPS focuses on improving access to essential medical products and related services and on the appropriate use of medicines to ensure better health outcomes for all populations. The program brings expertise honed over decades of seminal pharmaceutical systems experience across more than 40 countries. The MTaPS approach builds sustainable gains in countries by including all actors in health care—government, civil society, the private sector, and academia. The program is implemented by a consortium of global and local partners and led by Management Sciences for Health (MSH), a global health nonprofit.

The MTaPS Consortium

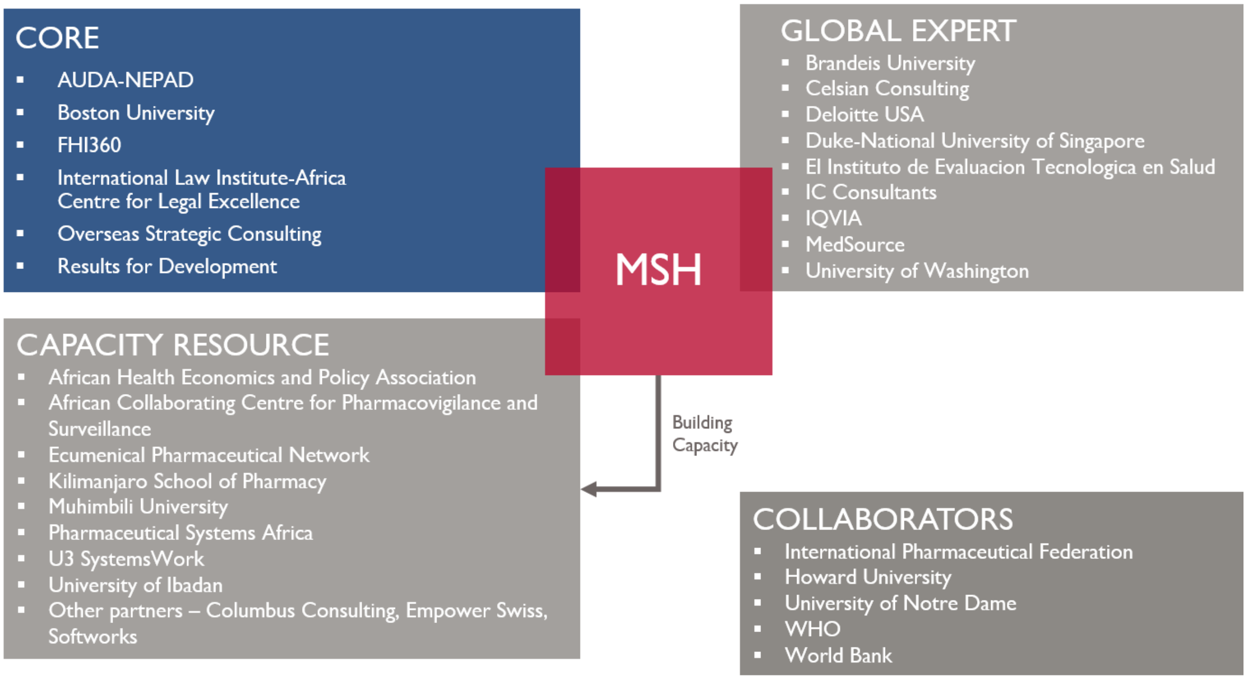

This report/document is submitted by:

Romuald Mbwasi, Consultant,

and Augustine B. Matondo (PhD), Consultant,

Medicines, Technologies, and Pharmaceutical Services (MTaPS) Program,

P.O. Box 2,

Dar es Salaam, Tanzania.

Email: rmbwasi@gmail.com; matondo@sua.ac.tz

ACRONYMS AND ABBREVIATIONS

ADDO	accredited drug dispensing outlets
ADR	adverse drug reaction
AMC	antimicrobial consumption
AMR	antimicrobial resistance
AMS	antimicrobial stewardship
AST	antimicrobial susceptibility testing
AWaRe	Access, Watch, and Reserve
Cap.	chapter
CEM	cohort event monitoring
CHMT	council health management team
DDD	Defined Daily Dose
DDD/1000/D	Defined Daily Dose/1000 inhabitants/day
DVS	Directorate of Veterinary Services
EDCTP	European and Developing Countries Clinical Trials Partnership
FAO	Food and Agriculture Organization
GHSA	Global Health Security Agenda
GN	Government Notice
HSSP V	Fifth Health Sector Strategic Plan
IHR	International Health Regulations
IPC	infection prevention and control
JEE	Joint External Evaluation
LGA	local government authority
MAB	Ministerial Advisory Board
MAH	market authorization holder
MCC	multisectoral coordination committee
MLF	Ministry of Livestock and Fisheries
MOH	Ministry of Health
MPG	Medicines Policy Guidelines
MSD	Medical Stores Department
MSH	Management Sciences for Health
MTaPS	Medicines, Technologies, and Pharmaceutical Services
MTC	medicines and therapeutics committee

MTWG	multisectoral technical working group
NAP	National Action Plan
NEMC	National Environmental Management Council
NEMLIT	national essential medicines list
NEVML	national essential veterinary medicines list
NHIF	National Health Insurance Fund
NHP	National Health Policy
OIE	World Organization for Animal Health
PAVIA	PhArmacoVIgilance Africa
PC	Pharmacy Council
PMS	post-marketing surveillance
POM	prescription-only medicine
PPS	point prevalence survey
PROFORMA	Pharmacovigilance infrastructure and post-marketing surveillance system capacity building for regional medicine regulatory harmonization in East Africa
PV	pharmacovigilance
PVS	pharmacovigilance system
RHMT	regional health management team
RUM	rational use of medicines
SCM	supply chain management
SF	substandard and falsified
STG	standard treatment guidelines
TB	tuberculosis
TBS	Tanzania Bureau of Standards
TDB	Tanzania Dairy Board
TFDA	Tanzania Food and Drug Regulatory Authority
TLDMP	Tanzania Livestock Development Master Plan
TMDA	Tanzania Medicines and Medical Devices Authority
TPRI	Tropical Pesticides Research Institute
TVLA	Tanzania Veterinary Laboratory Agency
TWG	technical working group
USAID	US Agency for International Development
VCT	Veterinary Council of Tanzania
WHO	World Health Organization

CONTENTS

Acronyms and Abbreviations i

Project Summary v

1. Executive Summary 1
   - Objective 2
   - Methodology 2
   - Findings on the primary policies and institutions relevant to AMS 2
   - Critical gaps identified in policy, market authorization, licensing, inspection, supply, and distribution of antimicrobial medicines 3
   - General Conclusion 6
2. Introduction 6
3. Background 7
4. Objective 8
5. Methodology 8
   - Method 9
   - Limitations 10
6. Overarching National Policies 10
   - National Health Policy of Tanzania 10
   - Fifth Health Sector Strategic Plan (HSSP V) 13
   - National Livestock Policy 13
   - National Fisheries Policy 14
   - Tanzania Livestock Development Master Plan 15
7. Marketing Authorization, Licensing, and Inspection 16
   - Market Authorization for Human and Animal health sectors 16
8. Post-marketing surveillance and Pharmacovigilance in Human and Animal Health 21
   - Human health 22
   - Animal health 24
9. Supply chain management 25
   - Human health 25
   - Animal health 27
10. Prescribing, Dispensing, and Use 28
    - Human health 28
    - Animal Health 32
11. Discusssion and Recommendations for the Human Health Sector 36

Policy and legal framework to support AMS in the human health sector 36
Purchasing and distribution of antimicrobials 36
Prescribing and dispensing 37
Irrational antimicrobial use 39

12. Discusssion and Recommendations for the Animal Health Sector 39

13. Identified gaps in the current policies and regulatory framework, and Recommendations for action 42
Human health 42
Animal health 45

14. Conclusion 50

Annex 1. List of Policies, Laws, Regulations, and Guidelines Relevant to AMS in Human and Animal Health 51

Annex 2. List of Key informants in the stakeholder interviews (Human Health) 52

Annex 3. List of Key informants in the stakeholder interviews (Animal Health) 53

Annex 4. List of MCC/TWG members who participated in the dissemination/feedback meeting (Situational Analysis Report presentation) 54

Annex 5. Implementation Status of Some of the Activities Under Strategic Objective 4 of the NAP-AMR 2017–2022 58

Annex 6. Processed requests for registration of medicinal products in 2020/21 61

Annex 7. Other requests for product registration 61

Annex 8. Requests for registration of premises for medicinal products 61

Annex 9. Historical data on registration of Veterinary practice facilities 61

Annex 10. Inspections conducted on registered Veterinary premises 62

Annex 11. Samples Collected and Analyzed under Post-marketing Surveillance (2009–2013) 62

Annex 12. Analysis of Samples of Products by the TMDA from Different Sources 62

Annex 13. Post-Market Surveillance of Veterinary Pharmaceutical Products (2016–2019) 63

Annex 14. Historical trend of Registration of Veterinary Skilled Personnel 63

Annex 15. TMDA Organogram 64

PROJECT SUMMARY

**Program Name:**		USAID Medicines, Technologies, and Pharmaceutical Services (MTaPS) Program
**Activity Start Date and End Date:**		September 20, 2018–September 19, 2023
**Name of Prime Implementing Partner:**		Management Sciences for Health
**Contract Number:**		7200AA18C00074
**MTaPS Partners**	**Core Partners**	Boston University, FHI 360, Overseas Strategic Consulting, Results for Development, International Law Institute-Africa Centre for Legal Excellence, NEPAD
	**Global Expert Partners**	Brandeis University, Deloitte USA, Duke-National University of Singapore, El Instituto de Evaluacion Technologica en Salud, IC Consultants, Imperial Health Sciences, MedSource, QuintilesIMS, University of Washington
	**Capacity Resource Partners**	African Health Economics and Policy Association, Ecumenical Pharmaceutical Network, U3 SystemsWork, University of Ibadan, University of Ghana's World Health Organizations (WHO) Pharmacovigilance Collaborating Center, Kilimanjaro School of Pharmacy, Muhimbili University, Pharmaceutical Systems Africa
	**Collaborators**	International Pharmaceutical Federation, Howard University, University of Notre Dame, WHO, World Bank

1. EXECUTIVE SUMMARY

Antimicrobial resistance (AMR) is a global threat to public health. The increasing global prevalence of AMR is recognized as one of the most pressing challenges facing humanity over the coming decades, with effective treatments against infectious diseases at risk of becoming increasingly unavailable. If left unchecked, by 2050, 10 million people could die each year due to AMR, 90% from developing countries.[1] One of the driving factors for the rise of AMR includes the use of counterfeit and substandard and falsified (SF) medicines, and misuse of legitimate antimicrobial agents in different sectors, notably in human health and animal health sectors.

One of the initiatives to address the AMR challenge is to initiate and implement antimicrobial stewardship (AMS) which is a systematic multisectoral approach for optimizing therapy through various structures and interventions guided by World Health Organization (WHO) Benchmarks for International Health Regulations (IHR) capacities.[2] However, it was noted that, despite significant developments in the implementation of other areas of IHR, Tanzania Mainland was identified to have no capacity (score 1) on AMS following the Joint External Evaluation (JEE) conducted in 2016.[3] Since then, efforts have been made to raise the score through implementation of the National Action Plan on Antimicrobial Resistance (NAP-AMR) 2017–2022, in which AMS is one of the priority actions under its strategic objective 4 (optimize the use of antimicrobial agents in human, animal, and plant health).[4] To optimize the prudent use of antimicrobials, the following priority actions are listed in the NAP: development of a regulatory framework for preservation of antimicrobial agents through revisiting the policies, regulations, guidelines, and directives on the use of antimicrobials; and establishment of AMS programs to ensure appropriate use of antimicrobial agents, all using the One Health approach.

In supporting the Government efforts to raise the national AMS capacity, the US Agency for International Development (USAID) Medicines, Technologies, and Pharmaceutical Services (MTaPS) Program, implemented by Management Sciences for Health (MSH), provided technical support in the rapid assessment/situational analysis of AMS policies, laws, regulations, and guidelines in human and animal health sectors. This analysis helped to highlight specific areas in the regulatory framework and supply chain management of antimicrobials that can be improved in line with priority action 8 (regulatory framework for preservation of antimicrobial agents) of the NAP-AMR. The situational analysis results are narrated in this document and include recommendations for action to address the identified gaps.

---

[1] The Review on Antimicrobial Resistance, chaired by Jim O'Neill. December 2014. Antimicrobial Resistance: Tackling a crisis for the health and wealth of nations. Wellcome Trust and the United Kingdom Government.
[2] WHO Benchmarks for International Health Regulations (IHR) capacities, 2019. https://apps.who.int/iris/bitstream/handle/10665/311158/9789241515429-eng.pdf?sequence=1
[3] WHO. 2017. Joint External Evaluation of IHR Core Capacities of the United Republic of Tanzania. Geneva: World Health Organization. License: CC BY-NC-SA 3.0 IGO.
[4] Ministry of Health, Community Development, Gender, Elderly and Children, United Republic of Tanzania. 2017. The National Action Plan on Antimicrobial Resistance 2017–2022.

OBJECTIVE

This rapid analysis sought to review the various policies, legislation, regulations, and guidelines impacting AMS in Tanzania Mainland and identify relevant gaps that must be addressed in the human health and animal health sectors.

METHODOLOGY

The analysis focused on four thematic areas:

1. Market authorization, licensing, and inspection
2. Post-marketing surveillance and pharmacovigilance (PV)
3. Supply management policies and guidelines
4. Prescribing, dispensing, and use relevant to antimicrobials.

A rapid comprehensive desk documentary analysis on Tanzania mainland's current AMS policies, legislation, regulations, and guidelines was conducted, which included the regulatory framework and supply chain management related to the supply and use of antimicrobials. This was supplemented by interviews with key informants along the antimicrobial supply chain. The initial findings were shared with stakeholders, and the stakeholder feedback was used to consolidate the findings and recommendations presented in this report.

FINDINGS ON THE PRIMARY POLICIES AND INSTITUTIONS RELEVANT TO AMS

Human health

Tanzania has the necessary policies and a robust regulatory framework, with essential regulations and guidelines upon which AMS can be built in the human health sector. These include the National Health Policy (NHP), Medicines Policy Guidelines (MPG), principal Acts relating to the Tanzania Medicines and Medical Devices Authority (TMDA) and the Pharmacy Council (PC), their regulations and guidelines, the Private Hospitals (Regulation) Act, Cap. 151, the National Essential Medicines List (NEMLIT), and national Standard treatment guidelines (STG) with Access, Watch, and Reserve (AWaRe) categorization of antibiotics. The MPGs address AMS by implementing a strategic objective "to improve therapeutic outcomes in patients at all levels" and setting various interventions to achieve the goal. Nevertheless, most of the principal Acts were enacted when AMR was not observed as a global health problem; therefore, they lack specific clauses that would address AMR and AMS directly. Future reviews will be necessary to cover the existing legal gaps.

Animal health

Among the strengths relevant to AMS in the animal health sector is the presence of specific sections in the National Livestock Policy (2006) promoting AMS. Section 3.14 of the policy encourages minimal use of chemicals and veterinary drugs in livestock production; section 3.18 recognizes collaboration with institutions regulating veterinary drugs and supports the manufacture, import, and distribution of quality drugs; and section 3.20 contain statements on the regulation of veterinary services. Therefore, the available legal instruments are supported by the National Livestock Policy.

CRITICAL GAPS IDENTIFIED IN POLICY, MARKET AUTHORIZATION, LICENSING, INSPECTION, SUPPLY, AND DISTRIBUTION OF ANTIMICROBIAL MEDICINES

Human health

- Policy implementation level
  - At the policy level, the government recognizes increasing AMR affecting the most common antimicrobials, irrational use of antimicrobials, inadequate awareness and understanding of AMR, limited evidence on AMR, weak infection prevention and control (IPC) integration programs, and an unsustainable fragmented approach to curbing AMR.
  - There is no medicine policy as a standalone document since the Ministry of Health (MOH) decided to have only one health policy which includes elements of drug-related issues. There is, therefore, a document called the Medicines Policy Guidelines for implementing the National Health Policy. The MPGs acknowledge that the formulation of the NEMLIT is well-coordinated and comprehensively evidence based. However, the use of local data is limited to support decision making during selection.
  - Though established, many hospitals' medicine and therapeutics committees (MTC) are not functional, and the hospitals lack hospital formularies to address the issue of AMR by setting up AMS programs.

- Legal level
  - Market authorization
    - Market authorization has not yet included specific issues to address antimicrobials as a particular group but takes a general approach for all products. In the scheduling, antimicrobials are included among the list of prescription-only medicines (POM) but do not have their own specific schedule.
    - Existence of unregistered and SF medicines in the market
    - Fragmented laws to enforce and control the promotion and advertisement of medicines
    - Low reporting rate of adverse drug reactions
    - Although the post-marketing surveillance (PMS) system is functional, the range of sampling seems to be minor.
    - The PV reporting level as per the WHO reporting rates per population is still low: only 2.5% of the required number of reports; therefore, it cannot be used conclusively to determine the safety of the medicine being reported.

  - Licensing and Inspection
    - Existence of unregistered premises
    - Unauthorized pharmaceutical personnel dealing with medication management and dispensation
    - A regulatory framework to control pharmaceutical services licensing covers business and professional licensing, premises inspection and registration, pre- and routine inspections, and the respective regulatory authorities. However, the existing laws and regulations are silent on antimicrobials, particularly acquisition limitations based on service level and comprehensive requirements for professional staffing to provide pharmaceutical services.

- Supply and Distribution
  - Weak control on dispensation, prescribing, and use of prescription medicines
  - Uncontrolled distribution/sale of antimicrobials, regardless of facility level of service, which in a way affects the success of AWaRe categorization and AMS programs
  - There are reports of a broad violation of the existing laws and regulations, characterized by stocking medicines not approved for that level, dispensing POMs without prescription, and nonprofessionals providing dispensing services and indiscriminate distribution of POMs to premises not allowed to store and distribute such drugs.

Recommendations for the human health sector

- In collaboration with its regulatory authorities, the MOH should review the legal system to include specific sections in the principal laws and regulations to address antimicrobials and set them apart from other general prescription medicines. Matters of categorization linked to distribution, prescribing, and dispensing must come out vividly in the legal framework.
- The two authorities, the TMDA and PC, should effectively enforce adherence to all laws, regulations, and guidelines equally to all players in the provision of pharmaceutical services (public and private).
- It is now high time for the TMDA and PC to institute a system that will allow thorough antimicrobial distribution and consumption audits at all levels of health services, with the possibility of data extraction so that the actual national antimicrobial consumption (AMC) trend should be available annually and analyzed for informed decision making.
- The MOH should strengthen public health facilities' laboratory diagnostic capacity to allow efficient antimicrobial susceptibility testing (AST), and it should build a database of easily accessible antimicrobial sensitivity testing results that can be used primarily for lower health care–level facilities during empirical prescribing.
- Similarly, higher health care–level private health facilities should be required to have laboratory diagnostic capacity or use other laboratory facilities for AST when prescribing medicines in the Watch and Reserve categories.

Animal health

- Livestock and Fisheries Policies
  - The Livestock Policy of 2006 and Fisheries Policy of 2015 do not explicitly address AMS, and they lack a strategy for AMS.

- Institutional and Legal implementation
  - Administration of the TMDA
    - The current Ministerial Advisory Board (MAB) does not reflect the prescribed list of statutory Board members in the TMDA Act, Cap. 219; for example, the Director of Veterinary Services and a representative from the Fisheries sector are not part of the current MAB.

  - Marketing authorization
    - Despite the mandates of animal health being under the Ministry of Livestock and Fisheries (MLF) through the Directorate of Veterinary Services (DVS), the DVS only participates in the initial stages of evaluating medicinal product application dossiers and does not have any

influence on decisions to authorize or not to authorize any medicinal products destined for use in the animal health sector.

- PMS and PV
  - PMS of veterinary medicinal products is limited in terms of product and geographical coverage
  - The existing pharmacovigilance system (PVS) completely excludes the animal health sectors
  - Acaricide importers are not involved in PMS for their products. Furthermore, there is no system for dealing with withdrawing acaricides that are ineffective due to resistance.

- Supply management, prescription, and use
  - Critical shortages of extension staff undermine regulatory functions. For example, 3,642 of the required 14,025 livestock extension staff are available as of June 2022, and only "40% of mandatory inspections under the MLF are done."[5,6]
  - The lack of a real-time data-sharing mechanism for inspections and other regulatory functions between the MLF regulatory bodies and the TMDA undermines the effective regulation of antimicrobials.
  - There is a contradiction between the TMDA (Scheduling of Medicines) (Amendment) Regulations, 2018, and the Veterinary Act (Veterinary Practice by Paraprofessionals and Paraprofessional Assistant Regulation), 2005, on the role of Veterinary Paraprofessionals in providing prescriptions.
  - There are contradictions between the Regulation for registration and movement of animal feed resources (Government Notice [GN] 327) and the Standards of Animal Feeds Regulation of 2013, GN 58.
  - There are no clearly defined roles in the certification of animal feeds between the Tanzania Bureau of Standards (TBS) and Tanzania Veterinary Laboratory Agency (TVLA) concerning antimicrobial residues or growth promotors in animal feeds.
  - The TMDA and the National Environmental Management Council (NEMC) have no system to track and audit compliance with waste disposal at registered health facilities.

Recommendations for the animal health sector

- The MLF should consider streamlining AMS in the respective policies during the next reviews by aligning the respective policies with the NAP-AMR and the Policy Guidelines for implementing AMS.
- The MOH should consider appointing the Director of Veterinary Services and a representative from the Fisheries section to the MAB within the framework of the TMDA Act, Cap. 219.
- The TMDA should integrate the DVS of the MLF throughout the decision-making process of authorizing or not authorizing medicinal products destined for use in the animal health sector. The DVS's role should not be limited to an advisory one in the initial stages of the process, such as evaluation of product application dossiers.
- The TMDA should consider improving PMS by widening geographical and product coverage.
- The TMDA (MOH) should undertake review of the current regulations and improve collaboration with the DVS (MLF) to establish and implement PV in the animal health sector.
- The MLF should compel market authorization holders (MAH) of acaricides to conduct PMS and submit their PMS reports to the DVS at specified periods.

[5] MLF Budget Speech 2022/2023.
[6] Tanzania Livestock Inspection Framework 2021.

- The DVS should develop a mechanism for dealing with acaricides that are no longer effective.
- The MLF should consider employing health extension officers in at least every ward in under-served areas as part of public interventions in areas that are less attractive for the private sector to offer veterinary services.
- The TMDA Regulations on Scheduling of Medicines (Amendment) 2018 and its associated guidelines should be reviewed.
- The Standards of Animal Feeds Regulation of 2013, GN 58 should be repealed, and the role of TBS and TVLA in the certification of animal feeds should be clarified.
- The TMDA and the NEMC should consider having an efficient system for auditing waste disposal at registered health facilities.

GENERAL CONCLUSION

The situational analysis has revealed the existence of institutions, policies, strategies, laws, regulations, and guidelines governing the regulation of antimicrobials. However, there are still some institutional policies and legal gaps that limit the regulatory system's efficiency. Some institutional gaps can be addressed without needing to change the existing policies and laws, although others may require a review of existing laws, regulations, and guidelines. In all cases, the implementation of legal instruments requires human and financial resources to enforce the terms and conditions attached to the issued licenses through voluntary compliance or sanctions.

In addition, it is essential to strengthen interinstitutional cooperation and coordination between the regulatory institutions responsible for enforcing and monitoring antimicrobial distribution, consumption, and disposal of waste generated at formal (registered) and informal premises in both human and animal health sectors.

2. INTRODUCTION

AMR is a global threat that requires urgent collaborative action within and among countries. AMR occurs when a microorganism develops resistance to an antimicrobial medicine to which it was previously sensitive, making standard treatments ineffective and facilitating the spread of drug-resistant infections, thus rendering the community vulnerable. The increasing global prevalence of AMR is recognized as one of the most pressing challenges facing humanity over the coming decades, with effective treatments against infectious diseases at risk of becoming increasingly unavailable. If left unchecked, 10 million people could die each year due to AMR, 90% being from developing countries.[1] One of the driving factors for the rise of AMR includes the use of counterfeit and SF medicines, and misuse of legitimate antimicrobial agents in different sectors, notably in human health and animal health sectors. Inappropriate use of antimicrobial agents across human, animal, and agriculture sectors is among the main drivers of AMR globally.

In 2015, the WHO released its Global Action Plan on AMR, with one of its strategic objectives being to optimize the use of antimicrobial medicines in human and animal health, through actions such as

development and implementation of effective AMS programs in human and animal health.[7] The WHO also released its Benchmarks for IHR capacities (2019), which has suggested actions needed to improve in-country IHR capacities or health security, helping countries to assess and improve their status on sustainable capacity for each benchmark.[2]

AMS refers to coordinated efforts and activities that seek to measure and improve the use of antimicrobials. It is a systematic multisectoral approach for optimizing therapy through various structures and interventions guided by the WHO Benchmarks. To help map the existing situation in AMS and to inform improvement measures, the WHO Benchmarks tool recommends the following action under benchmark 3.4 for achieving capacity level 2 (undertake an assessment of stewardship policies and activities, including regulatory framework and supply chain management of antimicrobials, using a multisectoral approach). Implementation of AMS programs have demonstrated positive public health and clinical impacts—including a reduction in costs, length of hospital stays, and the burden of AMR—while maintaining or improving patient outcomes.

3. BACKGROUND

In February 2016, Tanzania was the first to undergo the WHO JEE.[3] Despite significant developments in the implementation of other IHR areas, Tanzania Mainland was rated as score 1; i.e., no capacity in AMS. Tanzania is implementing its NAP-AMR 2017–2022, in which AMS is one of the priority actions under its strategic objective 4 (optimize the use of antimicrobial agents in human, animal, and plant health).[8] The challenge is to raise the AMS score to meet Global Health Security Agenda (GHSA) priorities. To optimize the prudent use of antimicrobials, the following priority actions are listed in the NAP-AMR: development of a regulatory framework for preservation of antimicrobial agents through revisiting the policies, regulations, guidelines, and directives on the use of antimicrobials; and establishment of AMS programs to ensure appropriate use of antimicrobial agents, all using the One Health approach.

The USAID MTaPS Program provided technical support in developing the multisectoral AMS policy guidelines aimed at controlling antimicrobial use in the human and animal health sectors, in which the strategies for implementing AMS programs are delineated.[9] To safeguard appropriate selection of antimicrobial agents for specific disease conditions, the AMS policy guidelines note that the Ministries responsible for human, animal, and plant health should ensure that empirical treatment guidelines are developed based on evidence in line with guidance from the WHO/World Organization for Animal Health (OIE)/Food and Agriculture Organization (FAO). The developed policy guidelines consider the global framework for implementing AMS developed by WHO, FAO, and OIE.[10] The framework contains several components: developing a national research agenda on AMS and supporting research and development on antimicrobial use; enforcing regulations on antimicrobial manufacturing, handling, and distribution; and prescribing, dispensing, use, and disposal in all responsible sectors. Other components include establishing AMS programs, adherence to STGs for empirical treatment, strengthening

[7] World Health Organization. 2015. Global Action Plan on Antimicrobial Resistance. ISBN 978 92 4 150976 3.
[8] Ministry of Health, Community Development, Gender, Elderly and Children, United Republic of Tanzania. 2017. The National Action Plan on Antimicrobial Resistance 2017–2022.
[9] Ministry of Health, Ministry of Agriculture, Ministry of Livestock, United Republic of Tanzania. June 2020. Policy Guidelines for Implementing Antimicrobial Stewardship: Tanzania Mainland.
[10] Global Framework for Development & Stewardship to Combat Antimicrobial Resistance.

laboratory diagnostics by performing AST, and collecting and analyzing data on antimicrobial use. Outside of the regulatory framework, there are already significant advances in Tanzania mainland in other components of AMS described above.

In addition, MTaPS provided inputs into the strategies to operationalize the policy guidelines and provided technical support to formulate the AWaRe list of antibiotics and the review of the combined NEMLIT and STGs.[11]

In further supporting the Government's efforts to raise the national AMS capacity, MTaPS provided technical support in the rapid assessment/situational analysis of AMS policies, laws, regulations, and guidelines in the human and animal health sectors to highlight specific areas to be improved in the regulatory framework and supply chain management of antimicrobials. This is in line with priority action 8 (regulatory framework for preservation of antimicrobial agents) of the NAP-AMR, which aims to improve the current regulatory framework on AMR through the formulation and review of the existing policies, laws, and guidelines on the use of antimicrobial agents in the human, animal, and agriculture sectors. The situational analysis results are chronicled in this document and include recommendations for action to address the identified gaps. The implementation of this activity addresses the one remaining action needed to progress toward achieving WHO IHR benchmark 3.4 capacity level 2, thus contributing to 100% achievement for that level for Tanzania.

4. OBJECTIVE

The main objective of the assessment was to contribute to the improvement of the current regulatory framework through rapid assessment of Tanzania's current stewardship policies on regulation, supply, and use of antimicrobials in the human and animal health sectors to highlight gaps and recommendations on areas that may be improved under four specific thematic areas listed below:

- Market authorization, licensing, and inspection
- PMS and PV
- Supply chain management (SCM) policies and guidelines relevant to antimicrobials
- Prescribing, dispensing, and use, as appropriate to antimicrobials

5. METHODOLOGY

A comprehensive desk review of documents on Tanzania Mainland's current stewardship policies on regulation, supply, and use of antimicrobials in human and animal health was undertaken to develop a first draft of the rapid situational analysis report. The documents' data sources included available policies, legislation, regulations, and guidelines in the four thematic areas specified in the main objective of the assessment.

[11] Ministry of Health, Community Development, Gender, Elderly and Children, United Republic of Tanzania. 2021. Standard treatment guidelines and National Essential Medicines List for Tanzania Mainland. Sixth edition.

Excluded under this assessment were the agriculture and environmental sectors.

The first draft situational analysis report was expected to include itemized information gaps and areas that needed further clarification, which could be obtained through subsequent in-person stakeholder interviews. The interviews aimed at gathering more information from key informants among the stakeholders, clarifying the previously assembled evidence of the relevant policies, legislation, regulations, and guidelines. A summary of findings was developed before engaging stakeholders in dissemination of the results using a One Health approach. The information gathered through stakeholder engagement was used for enriching the first draft report on situational analysis and preparing the second draft and presentations of findings to the central-level AMS technical working group (TWG) and the AMR multisectoral coordination committee (MCC). These presentations also included recommendations for the development of the AMS section of the future NAP, which will replace the current NAP-AMR after 2022. The final report on situational analysis in Tanzania Mainland was then prepared.

Note: Analysis of policies, legislation, regulations, and guidelines in the agriculture and environment sectors is critical for a comprehensive analysis of AMS policies, but it was not part of the scope of work that guided the analysis and preparation of this report. However, the environmental sector is mentioned only where it is directly linked to the existing human and veterinary medicine regulations and guidelines.

METHOD

MTaPS engaged human and animal health experts as consultants to conduct the assessment. Independent preliminary desktop reviews and analyses of existing policies, legislation, regulations, and guidelines in the human and animal health sectors were carried out, and an initial draft report was prepared.

The desk review was followed by stakeholder consultations for the respective human health and animal health sectors to gather more information and to clarify issues identified during the desktop documentary review. The selection of key informants for interviews from among the stakeholders was based on their organizations' involvement; the participants' roles in developing and implementing AMS-related policies, laws, regulations, and guidelines; and their experiences and knowledge of the current regulatory framework. The participants can be arbitrarily categorized into three types: regulatory; those involved in pharmaceutical management and businesses; and either users of, or those engaged in, animal/human health services. MTaPS recruited 7 key informants from the human health sector and 20 from the animal health sector for an interview to enrich the first draft report (annex 3). The stakeholder views and recommendations were compiled and included in the separate stakeholder recommendations report for the human and animal health sectors.

The information gathered from the stakeholder consultation was used to refine the first draft of the report, which generated the second draft. The findings in the second draft were compiled and presented to the national AMS TWG and the AMR MCC (annex 4) to provide inputs to the ongoing review of NAP-AMR (2017–2022), to feed into the development of the next NAP-AMR 2023–2028, and to gather more feedback to improve the second draft report.[8]

The two draft reports on human and animal health sectors were merged to produce one final report which detailed findings of the rapid situational analysis of AMS policies, legislation, regulations, and guidelines in Tanzania's Mainland, and which provided recommendations for action.

LIMITATIONS

Consultations within the human health sector were challenging because targeted persons were involved in other commitments within the short time required to complete the assignment. Nevertheless, this was compensated for by the stakeholder workshop, where most of the targeted persons were available. In addition, because of the limited time available for preparing the reports and dissemination, it was not possible to have more than one dissemination meeting.

It is expected that dissemination to relevant audiences, including regulators and users of antimicrobials, will be supported and will continue beyond the publication of this report.

6. OVERARCHING NATIONAL POLICIES

NATIONAL HEALTH POLICY OF TANZANIA

The health sector is guided by the NHP 2007, while a new policy is being drafted.[12]

Policy issues

The increasing unavailability of medicines and other health products is critical for any health care delivery system. Despite the efforts by the government to improve the pharmaceutical sector, there are still shortages of medicines and health products, thereby affecting the provision of quality health services. Furthermore, AMR is an emerging public health problem. Likewise, there is the use of ineffective medicine selections methods, irrational prescribing and dispensing, the decline in the number and production capacity of domestic pharmaceutical industries, uncontrollable medicine, the high price of drugs, low capability of regulatory agencies, inadequate human resources for pharmaceutical services, and overdependence on imported health commodities.

The NHP acknowledges the private sector contribution to the health sector and sets building partnerships between public sector ministries, departments, and agencies, the private sector actors, religious institutions, civil society, and community-based organizations in the provision of health services as one of its principal goals.

Policy statements of the NHP

The Government will carry out the following:

- Ensure that the medicines and health commodities supply system is efficient, transparent, sustainable, and adhering to national and international quality standards
- Put in place a system to enable local manufacturing and to supply medicines and health commodities
- Establish measures to combat antimicrobial resistance

[12] National Health Policy 2007.

- Establish a mechanism of price control for medicine and health commodities
- Enhance capacity building of organs for quality control of medicine and health products

Medicines Policy Guidelines

Policy issues

The implementation of the NHP is guided by the MPGs for performance of the NHP.[12,13] The MPGs acknowledge that, although the NEMLIT is available, the selection of medicines partially relies on local evidence-based information.[11] However, for most of the medicines included in the NEMLIT, their selection is based on evidence mainly from international information or therapeutic data. The NEMLIT, besides other functions, guides the selection of antimicrobials for the human health sector. Subsequent editions of NEMLIT/STG shall be according to the existing AMR profile in the human health sector. The current NEMLIT/STG (6th Edition 2021) has included the WHO-recommended AWaRe categorization of antibiotics. Once evidence is gathered on the existing AMR country profile, the AWaRe categorization will be improved for the next edition of the NEMLIT/STG.

The MPGs also outline some constraints as listed below:

- There is still a lack of evidence or data at the country level that guides the inclusion/exclusion of antimicrobial agents in the NEMLIT/STG. The selection of antimicrobials is based on clinical experience and international evidence-based data.
- Limited infrastructure and capacity to enforce the existing laws, regulations, and guidelines, which are expressed in the form of:
  - Existence of unregistered and SF medicines in the market
  - Presence of unregistered premises
  - Unauthorized pharmaceutical personnel dealing with medication management and dispensation
  - Weak control on prescribing, dispensation, and use of prescription medicines
  - Low reporting rate of adverse drug reactions (ADR)

Other notable problems though not stated in the MPGs, include:

- AMR and the absence of or weak AMS programs to address the AMR problem
- Weakly controlled distribution/sale of antimicrobials regardless of health facility level of service, which in a way affects the success of implementation of AWaRe categorization and AMS programs in the country

Further in their implementation matrix (2022–2032), the MPGs have included an objective "to improve therapeutic outcomes in patients at all levels," to address AMR through the implementation of AMS programs at different health facility levels, as envisioned to be achieved through the following set of strategies:

- Creating awareness among the community members on appropriate medicines use through advocacy on rational use of medicines (RUM) and implementing strategies for RUM at all levels, such that 80% of prescribed antibiotics are as per AWaRe categorization by 2032,
- Conducting annual point prevalence surveys (PPS) to ascertain the level of adherence at facility levels under AMS
- Rollout of AMR surveillance system to all health facility levels

[13] Medicines Policy Guidelines for implementation of NHP.

- Conducting medicine utilization review to inform decision making in the health commodity supply chain
- Developing RUM modules and incorporating them into a national e-Learning platform for continuous professional development.

It could be possible to achieve what the MPGs intend to do, but this can only be possible if the implementation of AWaRe and STGs requires both public and private providers to abide by the existing MOH guidelines.

Current practice is that guidelines such as these are enforced only in the public sector. To implement the AMS program in the private and public sectors, the existing laws, regulations, and guidelines need to be reviewed to accommodate AMR, and therefore AMS, in the legal framework to make it binding for both public and private health facilities.

In efforts to address the deficiencies in the NHP, the government, through the One Health approach, developed the National Action Plan on Antimicrobial Resistance (NAP-AMR) 2017–2022, which is currently being implemented.[8] The NAP addresses the strategy to fight AMR by establishing structures to implement and monitor it. To implement the plan, the Tanzanian government has established the following structures:

- A national AMR Secretariat with two AMR focal persons, one from the MOH and the other from the MLF, and the secretariat that includes other members appointed by the MCC. The MCC is a steering body that oversees the coordination of all AMR-related activities.
- In addition, the MCC has under it multisectoral technical working groups (MTWGs) that are established and mandated to oversee the implementation of specific responsibilities in select AMR areas such as providing technical inputs, conducting a situation analysis, and collaborating with MCC to develop action plans. Four MTWGs have been established, covering different areas related to AMR control. These groups are:
    - MTWG for awareness, effective communication, and education
    - MTWG for AMR knowledge, surveillance, research, and sustainable investment
    - MTWG for IPC, sanitation, and hygiene
    - MTWG for antimicrobial use and stewardship.

Each of these groups has one strategic objective to implement. In the case of this assignment, the MTWG for antimicrobial use and stewardship (AMS TWG) is relevant. This TWG addresses the NAP-AMR strategic objective 4 (optimize the use of antimicrobial agents in human and animal health). In optimizing the prudent use of antimicrobials, two priority areas have been identified under strategic objective 4: regulatory framework for the preservation of antimicrobial agents, and AMS. Specific goals have been identified under each priority area to implement strategic objective 4. For example, for the priority area of the regulatory framework for the preservation of antimicrobial agents, the group is tasked to ensure the appropriate use of antimicrobial agents through 2 strategic interventions: developing and reviewing policies, laws, regulations, and guidelines on the use of antimicrobial agents in humans, animals, fish, and agriculture, and monitoring proper use of preserved antimicrobial agents. For the priority area of AMS, the group is tasked with 2 strategic interventions: to establish AMS programs, and to monitor AMS programs. For each strategic intervention, several activities have been identified for implementation. The implementation status is presented in annex 5.

FIFTH HEALTH SECTOR STRATEGIC PLAN (HSSP V)

AMS issues are incorporated in the fifth health sector strategic plan (HSSP V) (2021–2026) in section 5.1.6 on infectious disease control.[14] The strategy is to reduce morbidity and mortality due to infectious diseases due to preventive measures, early detection, and early treatment for infectious diseases of public health importance and broaden the perspective of contagious disease control beyond malaria, HIV, and tuberculosis (TB).

Under section 5.4.3 on Medicines and Supply Systems, the government strategy is to improve the PMS system (quality of drugs in the market), intervene to reduce AMR, including optimal prescription of medicines, and expand the coverage and range of antimicrobials monitored. RUM of medicines will be mainstreamed into supply chain interventions. All institutions will have (electronic) versions of guidelines, and health workers will have the skills to apply these guidelines, protocols, and standard operating procedures correctly. The MTCs in health facilities will oversee the realization of these strategies while the Government will train and deploy health staff for pharmaceutical services.

Furthermore, HSSP V highlights the threat of zoonoses and AMR and the need to take a One Health approach, including engaging veterinary professionals and animal scientists to jointly control zoonoses and addressing AMR, food safety and other health threats, animals, and the environment. The MOH aims to enhance the use of TMDA apps and tools to improve the monitoring of both the quality and the safety of medicines. In addition, the MOH will review and disseminate the STG/NEMLIT based on evidence.

NATIONAL LIVESTOCK POLICY

The current livestock policy developed in 2006 does not contain specific statements on AMR or AMS; however, the policy has supportive AMS statements in its different sections.[15] Section 3.14 promotes minimal use of agrochemicals, including veterinary drugs, through the concept of organic farming. The idea came out following the increasing use and misuse of such chemicals resulting in health hazards. The policy's objective is to promote organically produced livestock products to exploit particular market demands, to promote and create awareness of organic livestock farming in collaboration with other stakeholders, and to encourage and promote investment in organic livestock farming while strengthening technical support services and practices.

Furthermore, section 3.18 describes veterinary medicines and vaccines as including pharmaceuticals, chemicals, and biologicals used for treating, preventing, and diagnosing diseases of animals and for promoting productivity. The policy recognizes both the role of the private sector in the supply of medicines and vaccines and the role of the MLF in regulatory and monitoring functions. The policy specifies that the MLF will collaborate with the former Tanzania Food and Drug Authority (TFDA), currently renamed TMDA, in regulating veterinary medicines and vaccines. The policy further identifies the existing constraints concerning the use of veterinary medicines, including their availability, high costs, poor quality, low awareness of stakeholders, and poor distribution network. The policy set out strategic

[14] Ministry of Health, Community Development, Gender, Elderly and Children. Health Sector Strategic Plan July 2021–June 2026. (HSSP V).
[15] Ministry of Livestock Development, United Republic of Tanzania. December 2006. National Livestock Policy. Available from https://www.tnrf.org/files/E-INFO_National_Livetock_Policy_Final_as_per_Cabinet_Dec-2006.pdf

objectives to ensure adequate supply, accessibility, and affordability of safe, quality, and productive veterinary medicines through the following policy statements:

1. The Government will encourage and support the manufacturing, importation, and distribution of quality veterinary medicines.
2. The Government will strengthen technical support services in veterinary medicine.

Additional relevant statements are provided under section 3.20, which emphasizes the need for regulating veterinary services to facilitate the compliance of paraprofessionals and professionals to the guidelines set by the OIE regarding the delivery of veterinary services and observation of ethics as stipulated in their respective codes of conduct.[16,17] In this respect, the policy recognizes that the delivery of veterinary services is still in transition, whereby the private sector works alongside the public veterinary service providers. It highlights existing constraints, including inadequate capacity to monitor and advise, insufficient expertise, inadequate infrastructure and facilities, low awareness of stakeholders, and poor coordination of different stakeholders engaged in the delivery of veterinary services. Given these deficiencies, the policy supports strengthening technical support services for the regulation of the veterinary profession, strengthening enforcement of compliance to standards and ethics governing the practice of veterinary medicine, and promoting educational advancement and communication concerning the veterinary profession.

Acaricides are also addressed in the National Livestock Policy on page 25, Section 3.11.3(a) under vector and vector-borne diseases and tick and tick-borne diseases control. Tick resistance is among the identified constraints in the policy, and the policy sets an objective of controlling ticks and eradicating tick-borne diseases to increase livestock production and productivity through the following action policy statements:

1. The Government will strengthen technical support services on tick and tick-borne disease control in collaboration with other stakeholders.
2. The Government will strengthen infrastructure for control of ticks and tick-borne diseases.
3. The Government will collaborate with other stakeholders to encourage and promote investment in the production of acaricides, anti-protozoan drugs, and other livestock inputs.

Lastly, section 4.3.3 of the policy emphasizes the involvement of the public and private sectors to facilitate policy implementation. Stakeholders include regulatory agencies such as the TFDA (currently TMDA), the Tropical Pesticides Research Institute (TPRI), the NEMC, the TBS, the Tanzania Dairy Board (TDB), and the Veterinary Council of Tanzania (VCT), among others.

NATIONAL FISHERIES POLICY

The current National Fisheries Policy of 2015 does not address AMR and AMS; however, the policy acknowledges the rise of intensive aquaculture production and the emerging challenges in fish disease surveillance, diagnosis, control, and treatment, and therefore includes disease prevention programs as a priority that can indirectly contribute to AMS.[18]

---

[16] The Veterinary Act (Code of Professional Conduct and Ethics for Veterinarians and Veterinary Specialists), 2005.
[17] The Veterinary Act (Code of Conduct for Veterinary Paraprofessionals and Paraprofessional Assistants) Regulations, 2005.
[18] Ministry of Livestock and Fisheries Development, The United Republic of Tanzania. 2015. National Fisheries Policy.

TANZANIA LIVESTOCK DEVELOPMENT MASTER PLAN

The Tanzania Livestock Development Master Plan (TLDMP) 2017/2018–2021/2022 sets out livestock-sector investment interventions on improved genetics, feed and water resources, and health services, among others.[19] Complementary policy support could help meet the sectorwide targets of improving productivity and the key livestock value chains. It is important to note that the government stopped providing animal health services in 1992, and the private sector was expected to assume the role of providing veterinary services. However, the inability of some livestock keepers to pay veterinary service fees continues to undermine the service delivery and sometimes leads to breakdowns of services. The TDLMP emphasizes that the supply of private veterinary services will be determined by profitability and several other factors arising from economies of scale, such as the size of the livestock enterprises in the locality, the nature of potential and actual animal diseases, and the types of animals. Therefore, according to the TLDMP, where private veterinary work is unprofitable, some public intervention is warranted. Thus, within the TLDMP framework, veterinary or animal health services will be done in a modified way through selective privatization and support policy. In this mode, the public sector will continue to offer veterinary services through general veterinarians and paraveterinary staff and assume all roles of delivering quality animal health services that the private sector cannot provide. The TLDMP outlines key priorities to continue supporting the growth of the private sector, including:

- Offering incentives for the private sector involvement in veterinary service provision in rural areas, including cost sharing for the prevention and control of diseases of economic importance
- Establishing a reporting system for the collection of reports on veterinary drugs and vaccines performance at all levels
- Strengthening enforcement of the Animal Disease Act, Cap. 156 and the Grazing Land and Animal Feed Resources Act, Cap. 180[20, 21]
- Building the capacity of animal feed and meat inspectors and formulating and enforcing poultry feed inspection, biosecurity, and other relevant disease control guidelines

It is generally worth noting that TLDMP does not have direct statements on AMS. However, it does address AMS indirectly by strengthening veterinary services, improving reporting of the performance of veterinary drugs, and strengthening enforcement of the existing laws.

The review of the Livestock Policy and the TLDMP creates a window of opportunity that can be used to consolidate AMR and AMS strategies in both documents.

[19] Ministry of Livestock and Fisheries, The United Republic of Tanzania. December 2017. Tanzania Livestock Master Plan (2017/2018–2021/2022).
[20] Animal Disease Act, Cap.156.
[21] The Grazing-land and Animal Feed Resources Act, 2010. Cap. 180.

7. MARKETING AUTHORIZATION, LICENSING, AND INSPECTION

MARKET AUTHORIZATION FOR HUMAN AND ANIMAL HEALTH SECTORS

A functional medicines registration system has been established, which gives the TMDA the mandate to issue market authorization to registered products. The medicines registration requirement is a legal requirement under section 53 of the TMDA Act, Cap. 219 which require all medicines to receive market authorization through mandatory registration of products by the TMDA.[22] A specific Expert Committee appointed by the Director General of the TMDA reviews medicines registration applications. The committee advises the Director General on the outcome of their evaluation. The system for medicine registration application is well elaborated in the regulation for registration of medicinal products and the TMDA compendium guidelines for market authorization of medicinal products.[23,24] The compendium requires the applicant to submit a detailed scientific (dossier) file. The dossier includes information about the chemistry, formulation, manufacturing, quality control and nonclinical and clinical studies demonstrating the quality, safety, and efficacy of active pharmaceutical ingredient(s) and the corresponding finished pharmaceutical product. Registered products are given unique registration numbers for each product and are issued with a registration certificate as evidence of marketing authorization. All medicines legally circulating in the Tanzania market are registered and therefore have marketing authorization for five years, after which the registration will require renewal or will automatically expire if not renewed. Registration is based on the following principal criteria:

- Availability of the medicine for public interest
- The medicines should be safe, efficacious, and of acceptable quality
- Effects on human health
- Effects on animal health, on consumers of food of animal organs, on the environment, and on users

Besides the documentary review and laboratory testing, TMDA inspectors conduct manufacturing site inspections to ascertain adherence to current Good Manufacturing Practices.[25] A medicinal product can be registered by the product manufacturer or through the agent. In the latter case, the agent becomes the sole distributor of the product in the country, and all other importers of the product must go through the agent. In the other case where the manufacturer registers the product, the registered product can be imported by any approved pharmaceutical importer.

All registered products are scheduled as per the TMDA Medicine Scheduling Regulations 2018.[26] A Medicines Scheduling Advisory Committee is appointed to provide technical advice and

[22] The Tanzania Medicines and Medical Devices Act, Cap. 219. Revised Edition of 2021.
[23] The Tanzania Medicines and Medical Devices (Registration of Medicinal Products) Regulations, 2015.
[24] TMDA. July 2020. Compendium Guidelines for Marketing Authorization of Medicinal Products. Doc. No. TMDA/DMC/MRE/G/001, Rev. No. 01.
[25] TMDA. March 2020. Guidelines on Submission of Documentation for Renewal of Registration of Human and Veterinary Pharmaceutical Products. Doc. No. TMDA/DMC/MRE/G/003, Revision No. 1.
[26] The Tanzania Food, Drugs and Cosmetics (Scheduling of Medicines) Regulations, 2015. GN. No. 63. Available from https://www.tmda.go.tz/uploads/publications/en1663930705-en1545384426-GN.%20No%2063.pdf

recommendations on scheduling human and veterinary medicines and other restrictions on the accessibility and availability of particular medications.

Human health

There are six schedules of human medicines; the interesting categories are the POM to be prescribed by an authorized medical practitioner and pharmacy, or "behind the counter." Most antimicrobials fall under the POM category, with very few antimicrobial formulations under the pharmacy-only medicines category. The Authority may cancel or suspend the marketing authorization of any product if there are deficiencies in the safety, quality, or efficacy of the product, if there is failure to comply with the marketing authorization requirements, or if there are changes in national policies. A deregistered product cannot be imported or marketed, and the registration certificate must be surrendered to the Authority immediately.

Licensing and Inspection

There are six schedules depending on the type of pharmaceutical service; business and premises licensing and are controlled by the TMDA Act, Cap. 219 and the Pharmacy Act, 2011.[22,27] Both Acts prohibit the provision of pharmaceutical services without pre-inspection and approval through issuing a license or permit to provide such services by the respective authority.[27,28] According to the TMDA Act, Cap. 219, the Authority has the mandate to carry out pre-inspection and routine inspection of premises and issue licenses/permits to the following categories of businesses:

- Pharmaceutical manufacturing plants, including compounding units
- Wholesale distributors (these are the importers who supply local wholesalers in bulk)
- Importers and exporters, through the issuance of import/export permits
- Pharmaceutical warehouses for the above categories

As per Pharmacy Act 2011, the PC has the mandate to carry out pre- and routine inspections of premises and issue licenses/permits to the following category of businesses:

- Community pharmacies
- Hospital/institutional pharmacies
- Accredited drug-dispensing outlets (ADDO)
- Any other premises that stores and dispenses medicines to clients

The PC is mandated to register all premises issued with a license, and all professional practitioners (pharmaceutical cadres) and issue registration/enrollment/enlisting certificates depending on the level of professional qualification, as listed below:[29]

- Pharmacist—registration certificate with annual renewal
- Pharmaceutical technician—enrollment certificate with annual renewal
- Pharmaceutical assistant—enlisting certificate with annual renewal

[27] Pharmacy Act 2011
[28] The Tanzania Food, Drugs and Cosmetics (Registration of Premises, Importation and Exportation of Pharmaceutical Products and Raw Materials) Regulations, 2015. GN 312.
[29] The Pharmacy (Premises Registration) Regulations, 2020

- ADDO and pharmaceutical dispensers are given recognition certificates. This group is not yet included in the principal Act, but some regulations control their practices.

In some cases, the roles of the two Authorities (the TMDA and the PC) interact, especially under inspection. For example, TMDA must enter and inspect any premises that store and distribute/dispense medicines since the TMDA is responsible for all pharmaceutical products. On the other hand, the Council can also enter and inspect any premise where pharmaceutical professional services are provided and where one of the registered professionals practices. The Council is responsible for professional practice adherence.

The other area of interaction is adherence to the level of service provided. The TMDA controls the type of products a given service level can store and distribute/dispense. At the same time, the Council defines the professional level of that person allowed to provide a given level of services. Examples include the TMDA-controlled list of medicines for ADDOs and the Council-defined roles and types of drugs the registered professional can deliver on their own. The licenses for both Authorities are issued once; however, permits are given annually based on regulatory adherence in the previous year, which shows the importance of annual inspection before permit issuance. Both Authorities are mandated to refuse permit issuance if the applicant has failed to comply with the law/regulations.

The TMDA claims to have sufficient human and financial capacity to carry out routine inspections of all premises before permit issuance.[30,31] The PC has 2,124 community pharmacies, 14,965 ADDOs, and several hospital-based dispensing outlets, far above the financial and human resource capacity the Council can afford. The Council has delegated the inspection activities to the Council Health Management Team (CHMT) and regional pharmacists. They expected to carry out a pre-licensing inspection for new premises and routine inspections before issuing new permits for the old premises and submit reports to the PC to approve the issuance of licenses or permits. The delegation of this part of the PC's authority to the local government authority (LGA) was expected to improve the inspection system performance.

Gaps

Marketing authorization has not yet included specific issues to address antimicrobials as a particular group but takes a general approach for all products. Antimicrobials are included in the POM part of the Regulation on scheduling of medicines, but they do not have their own specific schedule. *Could a separate antimicrobial schedule based on AWaRe categorization be possible with the increase in AMR?* That could make control of antimicrobial distribution possible and more manageable and monitor their consumption.

According to several reports, the inspection system is weak, characterized by broad violations of the existing laws, regulations, and guidelines.[32,33,34] Permits are issued without routine inspections and there is inadequate enforcement of meeting regulatory requirements for premises licensing, for example,

---

30 The Pharmacy (Pharmacy Practice and the Conduct of Business of a Pharmacy) Regulations, 2020.
31 TMDA. Annual Report 2019/20.
32 Horumpende et al. 2020. Point prevalence survey of antimicrobial use in three hospitals in North-Eastern Tanzania
33 TFDA. Ten (10) Years of Regulating Food, Medicines, Cosmetics and Medical Devices: Milestones Attained. 2003–2013. Available from https://www.tmda.go.tz/uploads/publications/en1546244565-10%20Years%20Book%20english-2.pdf
34 The Tanzania Medicines & Medical Devices (Appointment of Inspectors) Order, 2020.

professional staffing levels, type of service and products acquired and distributed, provision of services without license or permit, and vast violation of dispensing practices.

A regulatory framework controls licensing of pharmaceutical services provision, professional licensing, pre- and routine inspection, and registration of premises. However, the existing laws and regulations are silent on antimicrobials, particularly acquisition limitations based on service level and the required professional staffing. Different studies observe a broad violation of the existing laws and regulations, characterized by stocking medicines not approved for that level, dispensing POM without prescription, and nonprofessionals providing dispensing services.[35] Furthermore, there is an indiscriminate distribution of POM to premises not allowed to store and distribute such medicines, there are clinicians dispensing medicines, and there are pharmaceutical personnel dispensing POM without a prescription. All these weaknesses negatively affect the fight against AMR and the building of effective AMS programs in the country.

Animal health

In addition to the TMDA, which regulates market authorization for both human and veterinary medicines, the VCT and the DVS jointly regulate market authorization for acaricides.[36]

The VCT is also accountable for regulating animal health facilities, veterinarians, veterinary paraprofessionals, and paraprofessional assistants.[37] The DVS is also charged with regulating, preventing, and controlling animal diseases and acaricide quality and use.[20] The TDB, Tanzania Meat Board, and the TBS all hold standards for food products concerning antimicrobial residues.[38,39,40] In contrast, the NEMC governs the disposal of dangerous wastes, including those from pharmaceuticals and health facilities.[41] The LGAs provide multisectoral linkages to all the competent authorities under different legislations. The various sources mainly support inspections in their respective jurisdictions.

Registration of Animal health Antimicrobials

The TMDA registers veterinary medicines under the TMDA Act, Cap. 219. Available data (annexes 6 and 7) indicate a functioning registration system and a high level of compliance. Information required when submitting a request for registration of medicinal products for Veterinary use is provided in the TMDA guidelines, pages 43–118.[42] The registration of veterinary medicinal products is based on brand, formulation, and packaging. For example, for Oxytetracycline with similar ingredients and from the same manufacturer or importer packed in 100mL and 500mL; the different pack sizes are registered as different products, each attracting registration fees. This charging system applies to all applications for registration of medicinal products for parenteral use. Oral formulations are exempted from this rule, and single registration of the same product can apply to different packaging. Procedures for registration

[35] Seni et al. 2020. Antimicrobial use across six referral hospitals in Tanzania: a point prevalence survey.
[36] The Animal Diseases (Acaricide Application and Management Regulation), 2019. GN. 955.
[37] The Veterinary Act, Cap. 319.
[38] The Dairy Industry Act, Cap 262.
[39] The Meat Industry Act 2006, Cap. 421.
[40] The Standards Act, Cap. 130 (No. 2 of 2009).
[41] The Environmental Management Act, 2004, Cap. 191: The Environmental Management (Hazardous waste control and Management) Regulations, 2019. GN. 676.
[42] TMDA. Guidelines on Submission of Documentation for Registration of Veterinary Medicinal Products, March 2020.

of acaricides are outlined in the Animal Diseases (Acaricide Application and Management Regulations), 2019, where the VCT is responsible for registration.[36]

Licensing of premises, animal health facilities, and personnel dealing with antimicrobials

Manufacturing, wholesale, and logistical facilities are registered by the TMDA, whereas the VCT registers retail outlets and other animal health facilities registered under the Veterinary Act, Cap. 319.[37]

The registration and licensing of premises dealing with veterinary medicinal products are generally categorized into three main categories: manufacturing, wholesale outlets, and retail outlets. The separation of regulatory functions between the VCT and the TMDA is prescribed in the respective regulations under the Veterinary Act, Cap. 319 and the TMDA Act, Cap. 219.[43]

Other facilities include those used for logistics, such as transport and storage (annex 7). The registration of retail shops by the VCT is due to the nature of services provided by these premises, including the provision of veterinary consultations and ambulatory services (annex 8). The number of VCT-registered veterinary centers by June 2021 was 1,786, an increase from 1,709 recorded in April of the preceding year (annex 9).

As with other types of licenses provided for pharmaceuticals for human health, the licenses are issued once, but permits are given annually, based on regulatory adherence. Although the VCT registers veterinary retail pharmacies and other animal health facilities, the TMDA remains with the mandate for quality assurance of all antimicrobials, and therefore it is responsible for inspecting antimicrobials. At the same time, the VCT monitors the compliance of professionals and other requirements for providing veterinary services and the respective code of conduct for professionals and paraprofessionals.[16,17]

According to the Application and Management Regulation of 2019 for Acaricides, licensing of persons/companies dealing with acaricides is under the VCT and the DVS.[36] The DVS approve acaricides for use in Tanzania after the product passes quality tests at the point of entry. Noncompliant importers must return the unfit acaricide to the country of origin. The testing for acaricide quality control is conducted by authorized laboratories approved by the DVS. The VCT registers the acaricides, whereas the respective LGAs register facilities for acaricide applications through the District Veterinary Officer. Under the MLF, the DVS is responsible for advising on policy setting and regulating the quality of acaricides imported and distributed to the market.

Inspection of antimicrobials and premises

The inspection aims to monitor compliance and is conducted by the TMDA (annex 10) in collaboration with the MLF and LGAs through appointed inspectors. Activities related to inspection are occasionally conducted jointly, but in most cases, each authority conducts independent inspections. The TMDA inspects and monitors pharmaceutical quality, whereas the VCT inspects premises and facilities used to apply acaricides, other veterinary services and personnel.

---

[43] TMDA. The Tanzania Food, Drugs and Cosmetics (Registration and Licensing of Premises for Veterinary Products) Regulations, 2018.

Gaps

- Although the TMDA Act, Cap. 219 includes different stakeholders in the MAB, the current MAB does not reflect the prescribed list of 14 statutory Board members found in the TMDA Act. For example, although the DVS and representatives from the fisheries sector are expected to be members of the MAB as per the TMDA Act, neither the DVS nor a representative from the fisheries sector is appointed to the MAB.
- The activities of the Human and Veterinary Medicine Directorate of the TMDA are skewed toward the human health sector (annex 15) based on the reports and guidelines on PV, surveillance, and clinical trials. Combining human and veterinary medicinal products under one Directorate overshadows matters of the livestock sector, as human health always takes priority over animal health.
- There is weak integration of MLF in the decision-making process. For example, the representative of the DVS only participates in the initial stages of evaluating applications (in the Veterinary Medicines Registration Technical Committee at the TMDA) and does not have any influence on decisions to register or not to register any medicinal products destined for use in the animal health sector.
- Requests for imports (new antimicrobials not previously registered) are advised to pass through the MLF, but this is not mandatory, and the recommendations of the MLF can be overruled. The overruling of the advice of the MLF is incompatible with the TMDA Act, Cap. 219, Part IV, sections 51 and 60, which prescribe conditions for refusal of registration and deregistration, including adverse effects on animal health and with the considerations that animal health matters are administered by the MLF through the DVS as specified in the Animal Disease Act, Cap. 156. It is recommended that the DVS be provided with more power on the registration of pharmaceuticals destined for use in animal health within the framework of the TMDA Act, Cap. 219 and the Animal Disease Act, Cap. 156.
- Although both the TMDA and the VCT conduct regulatory functions, the two do not have real-time data-sharing mechanisms, thus affecting the timely and informed formulation of antimicrobial regulatory policies by the MLF and the MOH.

8. POST-MARKETING SURVEILLANCE AND PHARMACOVIGILANCE IN HUMAN AND ANIMAL HEALTH

The available PMS system covers the human and animal health sectors.[44] It refers to the procedures to monitor quality, safety, and efficacy after products have been registered and distributed for use.[45] PV is science and activities relating to detecting, assessing, understanding, and preventing adverse effects or other possible medicine-related problems.[46] It is vital to have fully functional PMS and PV systems to ensure that safe products are available in the market and that timely steps are taken to identify and minimize adverse effects caused by approved pharmaceutical products. These PMS and PV activities are conducted and/or coordinated by the TMDA.

[44] TMDA. Annual post-marketing surveillance (PMS) report for selected human and veterinary medicines circulating in Tanzania.
[45] TMDA. July 2019. Guidelines for Developing and Implementing Medicines Post Marketing Surveillance Program. Doc. No.: TMDA/DMC/CTP/G/008 Rev #. 0. Available from https://www.tmda.go.tz/uploads/files/PMS%20GUIDELINES%20FINAL%20OG.pdf
[46] The Tanzania Food, Drugs and Cosmetics (Pharmacovigilance) Regulations, 2018.

Section 18 of the TMDA Registration and Scheduling of Medicines Regulations requires every MAH to conduct periodic PMS and safety studies and submit a report to the Authority every two years for an evaluation.[26] According to the TMDA, the incidence of SF or poor-quality products is very low, and necessary actions is taken once an unregistered or SF product is spotted in the market.

HUMAN HEALTH

PMS

The TMDA has a PMS (2009) strategy/plan to monitor the quality of medicines in the Tanzanian market systematically. According to reports, PMS is active and functional. The 10th anniversary book shows that over 2009 to 2013, the system collected 423 samples and subjected them to quality control tests.[33] Annex 11 shows the samples and the outcome of the quality testing. The authority took various measures, including the withdrawal of the products that failed quality tests in the market. Based on the results in annex 11, the PMS exercises positively impact the quality of products circulating in the Tanzania market. In the TMDA PMS annual report 2018/2019, the authority reports that 158 samples were collected. Product Information Review indicates some of the samples collected had deficiencies not related to active ingredient contents, and all 158 samples passed confirmatory tests.

In discussions with the TMDA, two issues became apparent:

1. The TMDA does not have a specific category for antimicrobials in the schedule of medicinal products but would consider one if it were found to have an additional advantage in the control of AMR and in strengthening the AMS programs. Nevertheless, the authority needs to see such changes go together with a system that will monitor antimicrobial distribution and use.
2. The authority also acknowledged that although the MAHs are obliged to provide annual reports on PMS issues related to their products, only a few have a PMS system in place. The rest do not have such a system and are not adhering to the reporting requirement. However, the authority showed some letters addressed to all MAHs reminding them to adhere to PMS reporting requirements without fail.

Pharmacovigilance and inspection

A PV system (PVS) has been in place since 1993 and is functionally supported by the National Guidelines for Monitoring Medicines Safety 2018 to detect, assess and understand how to implement the PVS.[47] The TMDA has established a Pharmacovigilance Risk Assessment Committee within the PVS. The primary role of the PVS is to detect, assess, understand, and prevent ADRs. The guidelines guide on how PV and PMS activities can be carried out at the national level and at lower levels.

Two ADR monitoring systems are used:

- A passive surveillance or "spontaneous PV system" involves the use of an ADR reporting form, commonly known as the Yellow Form, for voluntary reporting of ADRs by health care workers. The

[47] TFDA. 2018. National Guidelines for Monitoring Medicines Safety. Third edition, June 2018. Doc. No. TFDA/DMC/CTP/G/002, 2018. Available from https://www.tmda.go.tz/uploads/documents/en1628143300-en1554375724-16.%20REVISED%20%20NATIONAL%20PV%20GUIDELINES%20%20%20%20JUNE%202018(1)-2.pdf

forms have been widely distributed nationwide to hospitals, health centers, dispensaries, and pharmacies. The TMDA prepays the cost of submitting the filled form.

- The second system is the Cohort Event Monitoring (CEM). The TMDA adopted this system in 2009 to collect more ADR reports. The technique involves a close follow-up (via active surveillance) of specified medicines used at health facilities. Medications used in treating illnesses that affect many people (diseases of public health importance), such as malaria, TB, and HIV/AIDS, are subjected to a CEM approach. According to the TMDA report, from 1993 to 2013, 8,040 reports were collected through CEM.[31] The affected products were antimalarials, namely, Artemether + Lumefantrine and Dihydroartemisinin + Piperaquine, and some antiretroviral medicines.

To facilitate the implementation of PVS, the TMDA has established seven PV centers mainly hosted at National, Zonal and some Regional Referral hospitals countrywide. However, the ADR reporting level is still low as per the WHO recommendation, with only 2.5% of the required number of reports based on the country's population; therefore, it cannot be used conclusively to determine the safety of the reported medicines. Nevertheless, the TMDA is determined to obtain the reports using this passive system using proactive approaches.

The guidelines also list several responsibilities of the MAH manufacturers/agents who should ensure that all the sections described in the Pharmacovigilance Regulations are observed.[46] Other specific issues to be addressed include having an appropriate PV and risk management system to assure responsibility and liability for the MAHs' products on the market. MAHs should submit PV reports every two years or as deemed necessary by the TMDA.

A multiprofessional/multisectoral National PV Technical Committee was established in 2008. It meets quarterly. Its primary obligations are to review, evaluate, and analyze adverse event reports, assess pharmaceutical risks, and provide recommendations to the MOH.

The TMDA organizes and carries out training on PV for health professionals, MAHs, and the public. To get them to understand the technical reporting and importance of PV and therefore improve the reporting rate, the TMDA also conducts seminars and awareness campaigns on PV (TMDA awareness campaign leaflets).

The PV guidelines define the roles of each stakeholder who may contribute to the improved performance of the PVS.[47] Functions are defined for the following:

- Patients and consumers
- Health facilities (public and private)
- Health management teams (CHMTs and Regional Health Management Teams [RHMT])
- TMDA offices
- Development partners and vertical programs
- The MOH

The guidelines require the CHMTs and RHMTs to appoint focal persons (pharmacists) at the council and regional levels to coordinate PVS-related issues.

The TMDA is currently implementing two projects: the pharmacovigilance infrastructure and post-marketing surveillance system capacity building for regional medicine regulatory harmonization in East

Africa (PROFORMA), and PhArmacoVIgilance Africa (PAVIA). PROFORMA is funded by the European Union's Framework Program for Research and Innovation Horizon 2020 via the European and Developing Countries Clinical Trials Partnership (EDCTP2).[48] PROFORMA is a consortium between academia and the national medicine regulatory authorities from four African countries: Ethiopia, Kenya, Tanzania, and Rwanda, as well as other international collaborators, including the Karolinska Institute (Sweden) and the WHO Collaborating Centre in Pharmacovigilance Lab, Netherlands. PAVIA is a consortium that is funded by the European and Developing countries Clinical Trials Partnership (EDCTP).[49] The PAVIA project aims to strengthen PV in four African countries: Ethiopia, Nigeria, Eswatini, and Tanzania.

Annexes 11 and 12 provide information on the level of TMDA PVS and PMS system implementation outcome in terms of products either picked through PMS activities of the TMDA or submitted by clients who had suspicion about the quality of the products or primary analysis.

Gaps

The PMS system is functional; however, the range of sampling seems to be limited. There is a need to see a broader range of products covered, specifically antimicrobials in line with the fight against AMR, especially those in the Watch and Reserve categories. The results in annexes 11–12 show that many products in the market have the desired quality standards, as the level of suspected effects that failed quality tests were only 3.8%. However, this combination of PMS and PVS looked at only a few products.

Responding to the observed gap, the TMDA noted that it targets products for specific diseases of public concern, such as malaria, TB, and HIV/AIDS, and that is why they monitor only a few medical products. Some of these products fall under the antimicrobial/antibiotic categories. Nevertheless, with the increasing threat of AMR, it is recommended that the TMDA readdress its PMS strategy to include antimicrobial products under the Watch and Reserve categories to follow up on their effectiveness. The TMDA is more than ready to remove any antimicrobial from the market if sufficient evidence shows that the product has high resistance and is no longer effective. However, the Authority observed a lack of adequate data on antimicrobial effectiveness and a lack of inflow of information on the level of resistance of various products currently in the market to the Authority to support decision making.

The Authority recommends strengthening the national antibiogram database to notify the Authority to make evidence-based decisions when necessary.

ANIMAL HEALTH

PMS

Available data show a functional post-market surveillance system.[50] PMS is conducted regularly, including antimicrobials for the animal health sector. The data indicate a limited number of samples of veterinary medicines that failed the confirmation test for quality, even though all tested models passed the

---

[48] https://proforma.ki.se/om-oss/
[49] https://pavia-africa.net/about
[50] Fimbo, AM, Maganda, BA, Mwamwitwa, KW, et al. Post marketing surveillance of selected veterinary medicines in Tanzania mainland. BMC Vet Res 18, 216 (2022). https://doi.org/10.1186/s12917-022-03329-x

screening tests (annex 13). There are guidelines for developing and implementing a PMS program (2019) that will apply to therapeutic antimicrobials.[51] PMS for acaricides is mandated to laboratories approved by the DVS, as stipulated in the Acaricide Application and Management Regulations of 2019.[36]

Pharmacovigilance

There are no mechanisms for PV or a national PV technical committee for the animal health sector.

Gaps

- Although there is PMS in place, the PMS is limited in terms of products and geographical coverage. For example, in 2019/2020, only one product was tested. The number of regions reached ranged from 2 to 6 out of 26 regions of mainland Tanzania (annex 13).
- The existing PVS completely excludes the animal health sectors, as evidenced in the current Pharmacovigilance Regulations (2018), National Guidelines for Monitoring Medicines Safety (2010), and the National Pharmacovigilance Roadmap (2019–2023).[52]
- There is an inadequate PMS mechanism for monitoring the quality of acaricides and tick resistance to acaricides in the market.

9. SUPPLY CHAIN MANAGEMENT

HUMAN HEALTH

The AMS policy guidelines note the achievement the human health sector has made in the supply chain management of antimicrobials, including the establishment of the Medical Stores Department (MSD), the TMDA, and the TPRI, which enable the monitoring of AMC in the country.[9]

However, the guidelines identify several constraints, such as weaknesses in laws, regulations, and policies enforced by the authorities, particularly in distributing antimicrobials to various premises, community pharmacies, ADDOs, and health facilities of different categories.

Existence of NEMLIT, STGs, and AWaRe categorization of antimicrobials

The country has a STG/NEMLIT (2021) in which it has adopted the WHO AWaRe classification of antimicrobials. All these documents are available physically or on the internet and can be acquired freely. Hospitals have been instructed to revive or establish their MTCs, and MTC guidelines (2020) have been developed and distributed.[53] Medicines in the STG/NEMLIT are categorized per level of service and professional level; for example:

- A = Medicines used at the dispensary level
- B = Medicines used at the health center level
- C = Medicines used at the council hospital level

[51] TMDA. July 2019. Guidelines for Developing and Implementing Medicines Post Marketing Surveillance Programme. Doc. No.: TMDA/DMC/CTP/G/008 Rev #. 0.
[52] TMDA. January 2021. The National Pharmacovigilance Roadmap 2019–2023.
[53] Ministry of Health, Community Development, Gender, Elderly and Children, United Republic of Tanzania. December 2020. Medicines and Therapeutics Committee Guidelines: Tanzania Mainland.

- D = Medicines used at regional referral hospital level
- S = Medicines used at zonal referral, national, and special hospital level

AMS programs have been initiated in 10 regional and zonal referral hospitals.

Procurement and distribution in the public sector

All public health facilities abide by the STG/NEMLIT when procuring and distributing essential medicines, which should be generic. The MSD is the principal supplier for public health facilities and strictly adheres to generic procurement as per the NEMLIT. The distribution of medicines in the public sector follows the STG/NEMLIT, whereby medications are categorized per level of service. Public health facilities can procure medicines from private suppliers under the Prime Vendor System or when required products are unavailable at the MSD.[54, 55]

The Prime Vendors selection strictly follows procurement procedures. The system has a comprehensive section that looks at the prospective supplier's quality system during the prequalification phase (Prime Vendor guidelines 2022). A special committee, the Health Commodities Receiving Committee, has been formed at each facility level to receive incoming health commodities. The committee checks physical quality characteristics and quantity supplied against the delivery documents.

Regarding AWaRe, there is a current lack of Regulations that would eventually strictly be followed by all those involved in the supply chain. Now, the guidance on the distribution of antimicrobials by AWaRe categorization is implemented only in the public sector but optionally followed by private providers. The lack of such regulations is a critical gap for the full implementation of AMS in the fight against AMR and establishing an effective AMS countrywide.

Procurement and distribution in the private sector

Private suppliers are categorized as importers, primarily supplying in bulk to local wholesale distributors, who usually sell small quantities to retailers and health facilities. The TMDA registers importers, while the PC registers local wholesalers. The TMDA controls medicine importation under the Regulations for importation and exportation of Pharmaceutical Products, 2015.[28] The TMDA must issue an importation permit after being satisfied that the intended products are registered in Tanzania.

Supply and distribution of special antimicrobials

The government, through the TMDA, restricts the importation and distribution of all antimicrobials that cover special disease programs of public health concern. These include antimicrobials for treating TB, leprosy, and HIV/AIDS. Private health service providers under special contracts/memorandum of understanding or arrangements with the government can receive these products under the program to provide free services to clients who prefer to get such services from private providers. If such restrictions hold, it would be necessary to review the regulations so that similar conditions can be applied in the distribution and prescribing of other antimicrobials. This would be one way to support the AMS programs.

---

[54] https://www.jaziaprimevendor.or.tz/

[55] Widemayer et al. 2019. Jazia prime vendor system - a public-private partnership to improve medicine availability in Tanzania: from pilot to scale. https://joppp.biomedcentral.com/articles/10.1186/s40545-019-0163-4

Gaps

For in-market distribution, wholesalers must follow the existing laws and regulations on the distribution of medicines to retailers and health facilities based on their level of services and the qualification of providers at that level. But because there is no control system in place yet, wholesalers sell antimicrobials of any category to any retailer or health facility, irrespective of its level of service. Unsurprisingly, antimicrobials not approved for facilities such as ADDOs, private dispensaries, medical clinics, and health centers are found at these levels and dispensed, in many cases, without prescription. The unrestricted acquisition and distribution, especially for antimicrobials in the private sector, defeats the categorization per level of service, professional competence, and AWaRe enforcement in public health facilities. Regarding the One Health approach, if restrictions are applied to only one sector (the public sector), then little impact in the fight against AMR will be achieved. No system regulates or monitors antimicrobials' distribution once they are in the market, making it challenging to determine the country's AMC status and other AMS-related indicators. Distributors are not obliged to keep up-to-date data on antimicrobial distribution or sales.

ANIMAL HEALTH

Most regulations governing antimicrobials in the human health supply chain apply to animal health with some notable differences. The supply chain of veterinary pharmaceuticals can be conveniently divided into six nodes or categories. These include importers who either import finished or semi-finished products, manufacturers, repackaging companies, storage, wholesale distributors, retailers, and consumers, including veterinary health facilities, livestock farms, hatcheries, and other forms of animal health services.[56] Imports, manufacturing and wholesale, storage, and transport vehicles (distribution) of veterinary pharmaceuticals are all exclusively regulated by the TMDA, except for acaricides, which the DVS and the VCT regulate.

The TMDA Act, Cap. 219, Veterinary Act, Cap. 319, and Animal Disease Act, 2003 are the principal legislations governing the veterinary antimicrobial supply chain. Key regulations include the TMDA (Good Storage and Distribution Practices) Regulations, 2021.[57] The Regulations control storage and distribution practices of regulated products, including antimicrobials, in Mainland Tanzania. The TMDA (Registration and Licensing of Premises for Veterinary Products) Regulations, 2018 deal with the registration of manufacturing and wholesale premises.[43] The Veterinary Procedures for inspection of veterinary facilities, 2005 regulate the registration of retail premises, other types of animal health facilities, and personnel in the respective facilities, and the Animal Diseases (Acaricide Application and Management) Regulation 2019 regulates the import, distribution, and monitoring quality and use of acaricides. The VCT registers retail outlets of medicinal products written by the TMDA, in most cases falling under the category of veterinary centers (annex 9). The TMDA remains the sole authority for ensuring the quality of veterinary pharmaceuticals throughout the supply chain, except for antimicrobials regulated under the Animal Diseases (Acaricide Application and Management) Regulation 2019.

---

[56] Mdegela et al. 2021. Antimicrobial Use, Residues, Resistance and Governance in the Food and Agriculture Sectors, Tanzania. Antibiotics 10, no. 4: 454. https://doi.org/10.3390/antibiotics10040454

[57] Draft regulations of the Tanzania Medicines and Medical Devices (Good Storage and Distribution Practices) Regulations, 2021.

Procurement and distribution

Procurement and distribution of antimicrobials used in animal health services are 100% done by the private sector. Therefore, the supply and availability of veterinary antimicrobials will be determined by market demand and profitability in the respective locality.

Gaps

- The mode of delivery of veterinary services makes it an area less attractive to private entrepreneurs, and therefore the vacuum is filled by an unregulated informal supply chain.
- The existence of an informal supply chain undermines the efficiency of antimicrobial supply chain control and the potential outcome of AMS programs.
- Regulatory inefficiency is also contributed to by other factors, including inadequate financial and human resources and uncoordinated regulatory activities done by respective regulatory authorities.
- The MLF also appoints inspectors and conducts inspections of facilities registered by the VCT. Despite overlaps in some regulatory activities, there is inadequate coordinated inspection between regulatory authorities and there is no real-time data-sharing mechanism.
- The MLF, through the livestock inspection framework, acknowledges that only 40% of mandatory inspections are accomplished due to limited resources.

10. PRESCRIBING, DISPENSING, AND USE

HUMAN HEALTH

Prescribing, dispensing, and use of antimicrobials in Tanzania are regulated through the TMDA Act and the Pharmacy Act.

To implement the laws, the authorities have developed regulations requiring service providers to abide by them. For example, both Acts prohibit self-prescribing or dispensing prescription medicines (under which most antimicrobials fall) without a valid legal prescription.

Despite the regulations, the control of prescribing and dispensing and use of antimicrobials remains a challenge. A description of some of the findings on prescribing and dispensing patterns follows below.

Prescribing patterns in public health facilities

The prescribing pattern in public health facilities is guided by the STG/NEMLIT. In addition, various disease-specific procedures and protocols exist at the national or program level, to which prescribers must adhere. Adherence to prescribing as described by the STG and other specific guidelines and protocols is one of the ways to implement AMS programs. However, studies show that adherence and compliance to dosage schemes are low.[32,35]

For the public sector, antimicrobials are categorized per level of service as described above and per the WHO AWaRe concept. Accordingly, antimicrobials in the Access group are found in many health facilities and can be prescribed empirically, whereby a prescriber may decide to name the medicines after physical examination, not knowing precisely the type of infection and the targeted antimicrobial

susceptibility level. According to the STG/NEMLIT and AWaRe categorization, antimicrobials listed in the Watch group need clinical justification. It requires a clinician to request some laboratory tests, including a sensitivity test against this group's antimicrobials so that only antimicrobials sensitive to that microorganism should be prescribed to treat that infection. Antimicrobials in the Reserve category can only be prescribed with a justification as those under the Watch group but only by a prescriber who is a specialist in that medical field.

According to the STGs and NEMLIT, antimicrobials under Watch and Reserve can only be available at a higher level of services where laboratory services (diagnostic services) are available, and additionally for the Reserve antimicrobials, where the specialist is available.

The guidelines also provide a guide on prophylactic prescribing, such as pre-surgery, with limitations on the dose duration. Still, according to studies, there is poor adherence to that at the hospital level.[35]

In general, antimicrobial prescribing and dispensing in many public health facilities at different service levels are empirical, with low adherence to the existing STGs and protocols and no clinical justification. Prescribers rarely request laboratory tests for clinical confirmation and microbial resistance. Similarly, antiobiograms (antimicrobial sensitivity testing profiles on various microorganisms) are not widely available for prescribers' reference when prescribing.

Another PPS in tertiary hospitals concluded that there was a high utilization rate of broad-spectrum antibiotics in Tanzanian hospitals and prolonged surgical antibiotic prophylaxis as standard practice. It recommended using PPS as an essential tool to improve future antibiotic use in Tanzania hospitals.

Prescribing patterns in private health facilities

According to existing laws and regulations on private health facility practice, service registration is strictly per level of service (medical clinics, dispensaries, health centers, and hospitals) with required professional personnel for each class of service. For example, section 1.11 of the Private Hospital (Regulations) Act states clearly that "only those drugs allowed at that level of the health facility should be available," and section 1.20 of the same Act says that "those running the private health facilities should always use the guidelines issued by the MOH from time to time," while section 2.6 forbids medical clinics to "store medicines or dispense them except for an emergency purpose."[58] Finally, section 1.25 says, "independent pharmacies (community pharmacies) and laboratories are not allowed to examine and prescribe to patients."

Prescribers tend to overprescribe, prescribing too many and multiple medications for the same treatment (antibiotic combinations). They often prescribe unnecessary medications that are not part of the standard treatment guidelines.

Patients do not always use medications as prescribed; some take partial doses due to medical costs. Further, patients pressure the health care providers to prescribe many drugs because they think that is associated with good care.[59]

---

[58] The Private Hospital (Regulations) Act, Cap. 151

[59] Baraka P. Poyongo and Raphael Zozimus Sangeda. Pharmacists' Knowledge, Attitude and Practice regarding the Dispensing of Antibiotics without Prescription in Tanzania: An Explorative Cross-Sectional Study.

To try to drive the private sector to adhere to the STG/NEMLIT, the MOH has been using the National Health Insurance Fund (NHIF) to reinforce prescribing as per the STGs by tying reimbursement to adherence to the guidelines. A health facility would not be reimbursed if the product prescribed does not follow the current STGs, in respect of the level of facility, AWaRe category requirements, and professional qualification of the prescriber. This step supports the AMS programs, but because there is less authority control, non-NHIF members are prescribing without necessarily adhering to STGs.

The TMDA and the MOH unit for public education have intensified consumer education through media and visual leaflets to reduce self-medication tendencies and overuse of antimicrobials. This educational material is another option for lowering the practice of over-prescribing, self-prescribing, and dispensing without prescription, thus providing direct support to AMS.[60]

Comment: Although the legal framework is very clear, the private sector is freely doing the contrary, and therefore the STG/NEMLIT and AWaRe categorization are not adhered to in many cases. The private sector is also substantially not adhering to the prescription regulation.[61]

In general, although the private health facilities' registration is strictly per level of service and the required professional human resources, enforcement of the establishing Act and Regulations is very weak. Private health facilities can acquire medicines/antimicrobials of all levels regardless of the level and professional availability and do not observe the guidelines provided by the MOH.

Medicine dispensing in the public sector

Dispensing medicines, especially antimicrobials, generally follows a well-regulated system at all public health facilities. In many public health facilities, especially at the primary health care level, dispensers have low professional qualifications; pharmacists are underemployed, and therefore rarely dispense or directly supervise dispensing; nurses and nurse assistants take most of the dispensing practice. The lowly trained dispenser has little authority to question a medical doctor's prescription and may go through with dispensing without critical evaluation and seeking justification for the prescription. This is critical for an effective AMS regarding adherence to AWaRe categorization. Prescribers may prescribe medicines that require clinical explanation, and the professionally low-trained dispenser may fear facing the prescriber if they ask for clinical justification.

Comment: The pharmacy should have a hospital formulary in which all medicines are described, and the prescribing justification is clearly stated. In this case, a current activity under MTaPS to support the development of hospital formularies is a step in the right direction to strengthen the dispensing practice. NHIF will not reimburse public health facilities as private facilities if the prescribing has not followed the current STGs. The reimbursement conditions imposed by NHIF are another step to support AMS at the facility level.

The use of legal prescriptions in all public facilities is mandatory, and prescribing antimicrobials is based on the STGs: drugs under Watch and Reserve require clinical justification, and for Reserve, a specialist.

---

[60] Matumizi Sahihi ya Dawa:Vijiuasumu (Antibiotics).

[61] The Pharmacy (Prescription Handling and Control) Regulations, 2020. Government Notice No. 266. https://www.pc.go.tz/files/SUPP%20GN%20267%2017%20APRIL%202020%20-The%20Pharmacy%20(Pharmacy%20Practice%20and%20Conduct%20of%20Business%20of%20a%20Pharmacy)%20Regulations,%202020%20-%20Final.pdf

If this is strictly implemented at all levels, control of antimicrobial prescribing and dispensing in the public health sector will likely improve, providing essential support to AMS.

However, studies have observed high use of antimicrobials at some referral hospitals, from 40% to 63%, where ceftriaxone and metronidazole were the most commonly used antibiotics, administered in 40% and 24% of courses, respectively. Indication-based antibiotic selection was concordant with national guidelines in 63% of the courses, but this fell to 15% when course duration was considered. Antibiotic courses were completed as prescribed 28% of the time among evaluable courses. 17% of the over 200 patients obtained a microbiologic culture. It was concluded that antibiotic therapy was often incomplete, was generally guideline discordant, exhibited limited diversity of selection, and frequently lacked diagnostic confirmation.[62]

Medicine dispensing in the private sector

Medicine dispensing, as in the public sector, is controlled by the two Acts—the TMDA and Pharmacy Acts—whereby all prescription medicines must be dispensed upon presentation of a legal prescription. A legal prescription is described under the PC Regulations 2021, last formulated as a response to AMR and to prevent the broad violation of the Pharmacy Act 2011. Medicine dispensing in the private sector occurs at multiple service provision outlets. It can arise in care homes (usually owned by nurses), private medical clinics, dispensaries, health centers, polyclinics, and hospitals. Private health facilities also include the for-profit and faith-based facilities, which usually fall under not-for-profit.

Dispensing at private for-profit facilities

According to TMDA Regulations, a particular schedule of prescription medicines has been established known as ADDO prescription-only medicines.[26] The ADDO prescription-only medicines are based on government-approved medicines for dispensaries and health centers, mainly serving the rural population. ADDOs are restricted from expanding the list of POM and dispensing medicines without a prescription. ADDO Regulations under the Pharmacy Council also control ADDOs.

The dispensing practice at ADDOs is concerning. First, they have expanded the list of antimicrobial prescription medicines freely supplied by the wholesalers. In most cases, they dispense these medicines without prescriptions. Even more critical is that often they dispense incomplete courses of treatment depending on the client's financial ability or as the client has requested. Unfortunately, most wholesalers' sale of antimicrobials to ADDOs is indiscriminate, meaning even the AWaRe categorization is not observed. One can easily find antimicrobials far beyond the ADDO level stored and dispensed indiscriminately. Mystery clients asked for and received amoxicillin with no prescription, commonly ranging from 86.32–89.9% in all three study regions. Furthermore, most outlets (98–99%) sold half-courses of antibiotics without asking the client any questions.[63]

Medicine dispensing and use practices at community pharmacies

Just like for the ADDOs, community pharmacies are controlled by similar laws and regulations, but they are allowed to store and legally dispense a more comprehensive range of prescription medicines, except for

[62] Matthew S. Haldeman et al. 2019. Evaluation of Antimicrobial Utilization and Concordance with National Guidelines at a Tertiary Hospital in the Southern Highlands Zone of Tanzania.
[63] Pendo M. Ndaki et al. 2021. Dispensing Antibiotics without Prescription at Community Pharmacies and Accredited Drug Dispensing Outlets in Tanzania: A Cross-Sectional Study.

unique restricted products under special public health programs. Although community pharmacies are registered under a pharmacist, either under the owner as a pharmacist or a superintendent pharmacist, pharmacists rarely work full-time at community pharmacies. Instead, they are staffed with lower cadres who usually have no training in pharmacy or are not allowed to provide services at these outlets. ADDO dispensers, nurse assistants, or nurses often provide complete services without a pharmacist's direct supervision. In most cases, community pharmacies behave similarly to the ADDOs; they dispense antimicrobials with or without a prescription, dispense half-courses, and sometimes ask the client no questions. For example, a study conducted in Dar es Salaam across community pharmacies revealed pharmacists' excellent knowledge of AMR.[63] However, even with this knowledge, 72.6% admitted to dispensing antimicrobials without prescriptions in their daily practice. Notably, 84.1% of pharmacists with master's or PhD education in pharmacy were likely to dispense without a prescription, compared with 69.3% among those with a bachelor's degree. The reasons for dispersing antimicrobials without a prescription included seeking more profit for the pharmacy business, patient failure to obtain a prescription, and the lack of stringent inspection of pharmacies by regulatory authorities. Penicillins, macrolides, and fluoroquinolones were the classes of antibiotics most commonly dispensed without a prescription.

In addition, the dispensing without prescription at community and ADDO outlets is also fueled by the fact that regulatory bodies do not have strict follow-up measures to control medical practitioners who prescribe and dispense by themselves in private health facilities for outpatients, especially in clinics, dispensaries, and health centers that store prescription medicines above their level of services. There should be stringent inspection and control for both dispensing outlets and prescribing health facilities with a limited mandate to dispense prescription medicines. Restrictions on prescribing and dispensing would allow a free flow of clients from private health facilities as sources of prescriptions to community pharmacies and ADDOs for dispensing. If authorities do not handle this, the fight against AMR is likely to fail, and the AMS programs may not achieve substantial success.

Medicine dispensing and use at private health facilities

Dispensing medicines in most lower health service levels, such as at medical clinics, dispensaries, health centers, and some secondary health facilities (hospitals), is mainly done by dispensing staff who are not trained in pharmacy. The majority are assistant nurses (a one-year trained nursing cadre), trained nurses, pharmaceutical and ADDO dispensers, or staff with experience working in hospitals. Dispensing and use of medications at private health facilities do not necessarily abide by the existing MOH laws, guidelines, and regulations. There is a broad violation in terms of dispensing practices and types of medicines stored and dispensed, disregarding the level of health facility and the qualifications of the prescriber. The private sector is weakly controlled in terms of dispensing and use. There are hardly any visible data that can be extracted for evaluation of the level of consumption of antimicrobials at the user point.

ANIMAL HEALTH

Key legislations regulating prescribing, dispensing, and use relevant for antimicrobials in the animal health sector include the TMDA Act, Cap. 219, and the respective regulation under the Veterinary Act, Cap. 319, including the code of conduct for professionals and paraprofessionals. Another legislation is the Acaricide Application and Management Regulation under the Animal Disease Act, Cap. 156. The persons responsible for the delivery of animal health services, including prescribing and dispensing medicines, are

holders of a veterinary degree or are paraprofessionals (diploma and certificate). The VCT is the only institution with the mandate to certify the animal health workforce through registration, enrollment, and enlisting of veterinarians, paraprofessionals, and paraprofessional assistants, respectively (annex 8).

Prescribing antimicrobials

Criteria for prescribing antimicrobials for veterinary use are described in the TMDA Medicine Scheduling Regulations 2018.[26] The following four categories are used for controlling prescriptions:

1. Controlled medicines, including narcotics and psychotropics: these are types of medicines under the International Drug Control Treaties, which the United Republic of Tanzania has signed.
2. Prescription-only medicines (i.e., the section for prescription-only veterinary medicines): Prescription-only veterinary medicines are identified under the following conditions:
   - Where a diagnosis of the condition for which the drug is intended would be beyond the competence of the livestock owner and therefore requires the service of a registered professional or enrolled/enlisted paraprofessional
   - Where the drug administration must be done by a professional or paraprofessional, i.e., when the route of administration is parenteral, and the animal needs to be monitored by the professional or paraprofessional in person
   - Where the drug is classified as having potential for misuse by farmers (e.g., indiscriminate use of oxytetracycline)
   - Where the drug administered changes/alters the animal physiology significantly, causes severe adverse reactions and requires monitoring of the patient by a veterinarian (e.g., the use of anticoagulants)
   - Where the drug is highly toxic (narrow therapeutic index), and the dose/toxicity level is finely balanced
   - All biologicals (e.g., vaccines to balance treatment regimens)
3. Pharmacy-only medicine: for veterinary use, these are medicines not suitable for general sale for animals but which do not meet the criteria for classification as prescription drugs; substances that may be used for food-producing animals but need no particular precaution against the potential risk to the person administering the product; drugs that require advice on the probability of undesirable effects and interaction with widely veterinary drugs; and drugs or compounds prepared at a pharmacy after a prescription from a veterinarian.
4. General sales veterinary medicines: drugs that require no specific technical advice concerning their method of use; they pose no significant risk to the animal being treated, the person administering the substance, the consumer, or the environment and the purchasing person having an animal in their charge.

Dispensing and use of antimicrobials

Dispensing of veterinary antimicrobials is considered a veterinary practice and is regulated by the Veterinary Act, Cap. 319 and Animal Disease Act, Cap. 156. The dispensing outlets are veterinary

centers, also known as Agrovets, ADDO, or *duka la dawa muhimu la mifugo* (DLDM-Vet).[64] The veterinary center provides retail and veterinary pharmacy services.

Other facilities include animal hospitals, veterinary clinics, laboratories, quarantine centers, ambulatory services, home/farm veterinary services, and training. There is no clear distinction between retail pharmacies, veterinary centers, and accredited veterinary dispensing outlets. All these are considered veterinary practice facilities registered by Veterinary Councils as Veterinary Centers (annex 8). The Regulations of the Veterinary Act, Cap. 319 allows veterinarians and veterinary paraprofessionals to dispense antimicrobials in these facilities.[16] The latter are required to work under the supervision of a veterinarian. Paraprofessional assistants are expected to play a supportive role and are not authorized to dispense veterinary antimicrobials. This rule is not in harmony with the TMDA Regulations regarding the part of paraprofessional assistants.[30] In most cases, prescription and dispensing services are done in the same consultation session.

The National Livestock Policy recognizes the need to reduce the unnecessary use of antimicrobials in the livestock sector and the need to regulate the use of antimicrobials as animal feed additives and growth promoters. Subsequently, the Grazing–land and Animal Feed Resources Act, Cap. 180 and its regulations limit antimicrobial agents' use in animal feeds.[20,65,66] In addition, to the Dairy Industry Act, 2004, Cap. 262, the Dairy industry (raw milk grading and minimum quality and safety requirements) Regulations 2020 prohibits the sale of milk with veterinary drug residues, and it is mandatory to sample and test raw milk regarding veterinary drugs and pesticides before shipment.[38,67] The maximum levels of veterinary drug residues are defined under the Standards Act 2009.[40]

Use of antimicrobials in aquaculture

There are no antimicrobials authorized for use in aquaculture. The increase in intensive aquaculture production systems for food and ornamental and the absence of proper preventive measures for diseases could contribute to the illegal use of antimicrobial agents meant for poultry in treating fish diseases.[59] Other antimicrobials in fish could come from animal manure used for pond fertilization or disposal of antimicrobial waste to the environment that can later reach the aquatic environment.[68]

Standard Treatment Guidelines and National Essential Veterinary Medicine List

According to the information available from the DVS, the STG for use in animal health has been developed and is currently in final preparation for dissemination. However, the STG does not cover aquaculture, as there are no antimicrobials authorized for use in aquaculture in Tanzania.

The National Essential Veterinary Medicine List (NEVML) is not available.

---

[64] *Orodha ya dawa zinazoruhusiwa katika Duka La Dawa Muhimu* (ADDO) (*Kanuni ya* "The Tanzania Food, Drugs and Cosmetics [Scheduling of Medicines] Regulations, 2015" [GN.No.63 of 2015]).
[65] The Grazing-land and animal feed resources (Registration and Movement of animal feed resources and products) regulations, 2021, GN. 327.
[66] Standards for Animal Feeds Regulations, 2013, GN. 58.
[67] Dairy industry (raw milk grading and minimum quality and safety requirements) (amendment) regulations, 2020. GN 534.
[68] Shah, S.Q.A.; Colquhoun, D.J.; Nikuli, H.L.; Sørum, H. Prevalence of Antibiotic Resistance Genes in the Bacterial Flora of Integrated Fish Farming Environments of Pakistan and Tanzania. Environ. Sci. Technol. 2012, 46, 8672–8679.

Antimicrobials in the environment

Registered premises such as shops and animal health facilities are required to dispose of expired, spoiled antimicrobials based on the TMDA (recall, handling, and disposal of unfit medicines) Regulations and the Environmental Management (Hazardous Waste Control and Management) Regulations.[69,41]

Gaps

- Based on the schedule for ADDO veterinary medicines, all oral antimicrobials are categorized as General Sales Veterinary Medicines.[26] These include chlortetracycline powder, oxytetracycline powder, sulfaclozine powder, and others. Only antimicrobials that require parenteral administration are classified as POM.
- Some antimicrobials are misused for prophylaxis.[70]
- Less attractive and profitable business environments, especially in rural areas, create critical shortages of the formal workforce, such as veterinarians, veterinary paraprofessionals, and paraprofessional assistants. Such deficiencies lead to weak enforcement and compliance with the regulations and guidelines. It is not uncommon for unskilled and nonprofessionals to purchase and use prescription-only antimicrobials on their farms.
- The third schedule (List of registrable and nonregistrable animal feed resources) of the Regulation for registration and movement of animal feed resources lists antibiotics and growth promoters as prohibited substances but excludes other antimicrobials.[65]
- The sixth schedule, serial number 2 of the Standards for animal feed regulations set a maximum limit of antibiotics allowed in animal feeds that is considered limited in scope, too high, and contradictory to the regulation on registration and movement of animal feed resources.[66,65]
- The STG for aquaculture is unavailable.
- The lack of general NEVML may affect AMS programs.
- Antimicrobial waste generated at home from backyard farms is not covered by the existing Environmental Management (Hazardous Waste Control and Management) Regulations, 2021 and the TMDA (Recall, Handling and Disposal of Unfit Medicines) Regulations. The disposal of these wastes may be through traditional methods since most LGAs lack efficient systems and the capacity to handle such types of garbage.
- The Environmental Management (Hazardous Waste Control and Management) Regulations, 2021 and the TMDA (Recall, Handling and Disposal of Unfit Medicines) Regulations, 2015 cover waste generated at registered premises, but there is no efficient mechanism for tracking and monitoring compliance with waste management and disposal at these facilities.

[69] Tanzania Food, Drugs and Cosmetics Act, 2003. Cap.219: Recall, handling and disposal of unfit medicines and cosmetics regulations, 2015. GN. 313.
[70] Kimera, ZI et al. 2020. Antibiotics, 9(12), 838; https://doi.org/10.3390/antibiotics9120838

11. DISCUSSSION AND RECOMMENDATIONS FOR THE HUMAN HEALTH SECTOR

POLICY AND LEGAL FRAMEWORK TO SUPPORT AMS IN THE HUMAN HEALTH SECTOR

Tanzania's NHP, MPGs, and HSSP V all recognize the threat to public health due to increased AMR nationally and globally, and therefore have set policies, laws, regulations, guidelines, strategies, and action plans to improve the availability, rational prescribing, dispensing, and use of antimicrobials. In line with One Health approach, Tanzania developed and is implementing its NAP-AMR 2017–2022.

The legal framework controls imports, distribution, service provision, and use of medicines. Two regulatory bodies have been established (the TMDA and the PC) to oversee legal adherence to existing laws, regulations, and guidelines in the provision of related pharmaceutical services.

The situational analysis has shown that although basic legal requirements such as product marketing authorization, import control, legal business and premises licensing, and registration are in place, there are substantial gaps in controlling provider practices or ensuring adherence to the established legal requirements. The existing legal framework is not very clear on the distribution of antimicrobials by distributors. However, the level of service of the registered premises and the related restrictions are well established in the principal laws and subsequent regulations. In other words, the regulating mechanism is weak to enforce total adherence to the existing regulatory framework.

Several incidences show some regulatory enforcement weaknesses or gaps. For example, the sale of antimicrobials by distributors to retailers and health facilities in the private sector is not monitored, and there is no system to do that. This gap leads to the indiscriminate distribution of antimicrobials to some facilities restricted to acquiring and dispensing such antimicrobials according to the legal requirements. Outlets such as ADDOs, and primary private health facilities (dispensaries, clinics, and health centers) have antimicrobials far above their legal level of service. Other examples include the broad violations experienced in antimicrobial prescribing and dispensing without prescription and limited enforcement of the use of the STG/NEMLIT and AWaRe categorization in the private sector.

Furthermore, use of national STGs is more often enforced in public health facilities, leaving the private sector at liberty to provide services while disregarding such guidelines. For example, the STG/NEMLIT and AWaRe categorization are only followed and adhered to by public health facilities while at the same time, the private providers have the liberty to follow them or do otherwise. However, legal requirements require public health facilities to follow national guidelines issued by the MOH. This gap makes it challenging to implement AMS programs nationwide, defeating the One Health approach concept in the fight against AMR.

PURCHASING AND DISTRIBUTION OF ANTIMICROBIALS

In Tanzania, the marketing, importation, or production of antimicrobials, as for other pharmaceuticals, is well controlled by the TMDA. However, the distribution of antimicrobials in the country is still a challenge for the following reasons: although the MOH issues guidelines, including categorizing

antimicrobials as a strategy to fight AMR, these guidelines do not directly affect the whole health supply system. Adherence is obligatory for public health care providers but optional to private providers.

Once the antimicrobials are in the country, their distribution is not strictly regulated, resulting in the indiscriminate acquisition of antimicrobials of different categories, even for facilities which are legally prohibited from acquiring and dispensing such antimicrobials. Furthermore, there is no system in place that can follow antimicrobial distribution (distributors are not bonded to provide that data) and AMC at the user level (providers are not required by any law to provide consumption data on antimicrobials). With the strategies under the NAP-AMR set, revisiting the existing laws, regulations, and guidelines to include sections on the acquisition of antimicrobials per categorization and strictly on the level of service and professional capability should be undertaken.

PRESCRIBING AND DISPENSING

Laws, regulations, and guidelines control prescribing and dispensing practices in the country. There are a proportionally high number of private health facilities in Tanzania, with about 42% of health facilities in the country belonging to this sector. These facilities are therefore a vital component in the fight against AMR; and if they are seriously involved in the AMS programs, they can make a great impact in the containment of AMR in the country.

Given the current scenario of weak regulatory control of the private sector, including lack of strict requirements to employ the right pharmaceutical personnel, unlimited access to any antimicrobial regardless of their level of service registered for, and lack of regular inspections, AMS programs will not succeed in the private sector. As the fight against AMR should take a multisectoral approach, Tanzania might not win the war against this global critical public health threat if variable liberal compliance to regulatory requirements in the private sector is not controlled.

Although the prescribing and dispensing practices in the private sector are unsatisfactory, very few studies have been done on the private sector. Several studies have been done on ADDOs, community pharmacies, and public sector health facilities, leaving the private health facilities broadly not well researched.

However, in a systematic analysis study in Africa, the authors observed that prescribing indicators were generally worse in private facilities compared with public facilities.[71] Analysis of prescribing across two time periods, 1995–2005 and 2006–2015, showed no consistent trends. The researchers concluded that prescribing indicators for the African region deviate significantly from the WHO reference targets. They urged for increased collaborative efforts to improve medicine prescribing practices in Africa to enhance the optimal utilization of scarce resources and avert negative health consequences.[71]

Despite strict regulations formulated under the Pharmacy Council and the TMDA concerning which types of stores are legally allowed to sell antibiotics and which require prescriptions, in practice,

[71] Richard Ofori-Asenso et al. Prescribing indicators at primary health care centres within the WHO African region: a systematic analysis (1995–2015).

antibiotics can commonly be purchased without prescriptions from both authorized and unauthorized drug stores.[72,73,74]

In the National AMC Review for 2017–2019,[75] the average defined daily dose (DDD) per 1,000 inhabitants per day (DDD/1,000/D) for all antimicrobials was 80.8 ± 39.35. The DDD/1,000/D declined from 136.41 in 2017 to 54.98 in 2018 and 51.02 in 2019. Doxycycline, amoxicillin, and trimethoprim-sulfamethoxazole were the most frequently consumed antibiotics during these years, accounting for 20.01, 16.75, and 12.42 DDD/1,000/D, respectively.

Most of the AMC in Tanzania occurred in the private sector, with the proportion of private sector antibiotic consumption increasing annually from 2017 to 2019. Based on AWaRe classification, >90% of AMC was for Access class medications, with Watch and Reserve class medications accounting for <10% and <1%, respectively. The reviewers concluded that the private sector use of antimicrobials is significantly increasing and should be carefully monitored following national policies. Future work is necessary to improve reporting of AMC patterns in sub-Saharan Africa.[75]

A study that sought to determine patient and drug seller attitudes and behaviors toward antibiotics and resistance found that 24% of patients were sold antibiotics for ailments such as cough, stomachache, and diarrhea.[76] In-depth interviews with patients whose medical officers sold antibiotics found that 49% of those prescriptions were inappropriate[77].

In addition to selling antibiotics without a prescription at a client's request, drug sellers also promoted inappropriate regimens. Several studies in Tanzania have confirmed irrational prescriptions of antibiotics in hospitals. A study in Moshi hospitals found that 80% of children under five years old presenting with diarrhea and cough received antibiotics inappropriately.[73] The most prescribed antibiotics were penicillins, sulphonamides, aminoglycosides, and macrolides. Clinical officers and assistant medical officers were the providers most likely to prescribe antibiotics, and diarrhea was the condition most likely to trigger an antibiotic prescription.[73]

Nevertheless, as described previously, these legal requirements are widely violated in both sectors (public and private). Still, the situation is aggravated in the private sector because of weak regulatory follow-up (insufficient and less effective inspections) and limiting the implementation of most MOH guidelines to the public sector only, leaving the private sector at liberty.

There is also a double standard practiced in enforcing the law and regulations on dispensing that goes without drawing the attention of the MOH or the regulatory bodies. As shown in many studies, the MOH and the Authorities always see the dispensing outlets (ADDOs, community pharmacies) as the

---

72 Poyongo et al. https://www.mdpi.com/2226-4787/8/4/238
73 Gwimile et al. 2012. Antibiotic prescribing practice in management of cough and/or diarrhea in Moshi Municipality, Northern Tanzania: cross-sectional descriptive study. The Pan African medical journal, 12, 103.
74 Harumpende et al. https://journals.plos.org/plosone/article?id=10.1371/journal.pone.0207465
75 Mbwasi, Romuald et al. 2020. National Consumption of Antimicrobials in Tanzania: 2017–2019. Frontiers in Pharmacology, 11, 585553. https://doi.org/10.3389/fphar.2020.585553
76 Minzi, O., and Manyilizu, V. 2013. Application of basic pharmacology and dispensing practice of antibiotics in accredited drug-dispensing outlets in Tanzania. Drug, healthcare and patient safety, 5, 5–11. https://doi.org/10.2147/DHPS.S36409
77 Kahabuka et al. 2013; https://journals.plos.org/plosone/article?id=10.1371/journal.pone.0058789

only violators in medicine dispensing practices, yet they are silent on the broad violations practiced by most prescribers, especially at private health facilities.

The law prohibits prescribers from dispensing, but the practice is widespread.[61] Authorities and MOH pay no attention to this practice, which has serious consequences for adherence to the rule of dispensing only with a prescription at dispensing outlets. Since the dispensing outlets know that sending clients to medical practitioners does not assure that the client will come back with a prescription (as the prescriber will, in many cases, dispense the prescription), the only way to survive in the business is to dispense without a prescription. Therefore, authorities must closely address these problems to bring the two sides in line with the existing laws and regulations. Fair play will take the nation a long way toward controlling AMR.

IRRATIONAL ANTIMICROBIAL USE

Several causes lead to irrational antimicrobial use problems, including weak regulatory oversight, high costs when seeking services at private health facilities, or long waiting times for services at public health facilities. The empirical nature of prescribing antimicrobials practiced by most prescribers makes clients see no difference between seeking services at a health facility or going directly to a dispensing outlet to get the antimicrobial. Other issues include an attitude of putting business before service provision by some providers, and a lack of sufficient knowledge of providers and users on the subsequent consequences of irrational antimicrobial use at the individual and national levels. All these issues must be critically analyzed, and strategies must be set to implement AMS programs to fight AMR effectively.

12. DISCUSSSION AND RECOMMENDATIONS FOR THE ANIMAL HEALTH SECTOR

The assessment results on policies, laws, regulations and guidelines presented in this report show the existence of a regulatory framework supported by the Livestock policy, the Fisheries policy, and the TLDMP 2017/2018–2021/2022.[15,18,19]

Another piece of information published elsewhere has observed the coherence between the existing policy and legal framework.[78] However, the respective policies do not adequately and directly address AMS. Fortunately, the Livestock policy and TLDMP are due for review. This could be an opportunity to align the new approaches and livestock development strategies with the recently developed guiding documents, including the NAP-AMR, the National Antimicrobial Resistance Surveillance Framework[79], and the policy guidelines for implementing AMS.[9] The MLF, in collaboration with stakeholders, should consider addressing AMS directly when the respective policies are reviewed, including the integration of AMR and AMS in the livestock and fisheries development master plans. This will strengthen the existing

[78] Ndomondo-Sigonda, M., Miot, J., Naidoo, S., Dodoo, A., & Kaale, E. 2017. Medicines Regulation in Africa: Current State and Opportunities. Pharmaceutical medicine, 31(6), 383–397. https://doi.org/10.1007/s40290-017-0210-x
[79] Ministry of Health, Community Development, Gender, Elderly and Children, and Ministry of Livestock and Fisheries, the United Republic of Tanzania. August 2018. National Antimicrobial Resistance Surveillance Framework. Available from https://www.mifugouvuvi.go.tz/uploads/publications/sw1602246063-NATIONAL%20ANTIMICROBIAL%20RESISTANCE%20SURVEILLANCE%20FRAMEWORK.pdf

policy support on the legal system regulating veterinary antimicrobials, including acaricides and other categories of pesticides.

The privatization of veterinary services has seen a proliferation of operators and diversification of medicinal products and origins, increasing the need for more robust control and monitoring of product quality. Although institutional gaps exist, as highlighted in the respective thematic areas, there is a relatively sufficient institutional framework for regulating veterinary pharmaceuticals in terms of antimicrobials. The framework provides an opportunity to close existing gaps and monitor antimicrobials' quality, safety, and efficacy. The weak linkages between the prominent regulatory institutions, namely the TMDA, the VCT, and the DVS, may limit the efficiency and benefits that could potentially be achieved through stronger cooperation and real-time data sharing. Fortunately, some improvements in linkages between these institutions can be achieved without the need to change the TMDA Act, Cap. 219 or the respective Veterinary Act, Cap. 319 and the Animal Disease Act, Cap.156. In addition, interinstitutional cooperation is supported by existing policies guiding the human and animal health sectors.

The increase in interoperability and coordination is likely to minimize the negative impact of inadequate human resources, unsatisfactory performance in critical functions such as PV, and other limitations as previously reported.[78] The already-established partnership through various memoranda of understanding signed between the respective regulatory institutions can be used as a milestone toward stronger cooperation and demonstrate that it is possible to collaborate and achieve a common goal. Possible improvements for consideration could include inserting the Director of Veterinary Services and one representative of the Fisheries sector relevant to antimicrobial control from the MLF into the MAB, as per the TMDA Act, Cap. 219. The addition of these representatives will increase the linkage between the MLF and the MOH and facilitate the timely development of informed policies in the respective Ministries.

The regulatory authorities stand to gain from improved relations through more consolidated and complementary efforts when discharging their regulatory roles. Internal administrative changes, such as splitting the Human and Veterinary Medicine Directorate of the TMDA (annex 15), can create an excellent linkage, giving the two sections equal decision-making and consolidated focus on related product registration. The TMDA can have a closer working relationship with the MLF, including having a collaborative real-time data-sharing platform that can feed information from either direction between the TMDA, the VCT, and the DVS.

Further improvements can be achieved by transforming the VCT from a department within the MLF to a semi-autonomous council to reduce the potential of political influence in its regulatory functions. This can be achieved by amending the Veterinary Act, Cap. 319.

The quality of registered veterinary medicinal products can be affected at any point along the supply chain, depending on various factors such as transportation and storage conditions. Therefore, continuous surveillance and PV are essential to ensure the safety of animals and animal products. The analysis identified that existing laws support PMS, and the system for PMS, which is supported by the presence of quality control laboratories and surveillance reports, is relatively advanced compared with other countries in the region and Africa.[50] However, there is room for further improvement to increase geographical and product coverage.

Despite having a relatively more robust regulatory system in the region covering all core thematic areas, including market authorization, registration, and surveillance, there is a significant gap in PV for the animal health sector. This assessment is based on the existing regulations and guidelines for PV. The weakness in PV is generally not unique to Tanzania, which might be attributed to the fact that its PVS is still being developed, as is also the case in other African countries.[80] However, the situation requires urgent interventions and may require cooperation between the TMDA, the MLF, the VCT, LGAs, and farmers through their respective organizations, and other stakeholders such as professional associations, due to the nature and complexity of veterinary services.

Furthermore, deliberate actions are needed to promote compliance with the current antimicrobial legal system by encouraging the prudent use of antimicrobials in the animal health sector. For example, the MLF should consider expediting the dissemination of the animal health STG and developing the NEVML, which will include pharmaceuticals for the aquaculture sector. The inclusion of aquaculture is justified by the current trend of rising aquaculture production coupled with the illegal use of antimicrobials.[68] The absence of authorized antimicrobials for aquaculture could potentially undermine AMS, as unauthorized and unguided use of antimicrobials in aquaculture production systems will be inevitable.

The increasing peri-urban livestock productions call for evaluating the existing policies and support available on the use and handling of veterinary drugs and antimicrobial waste at livestock farms and backyard production systems. Inadequate guidance and lack of a supportive environment for managing veterinary drug waste generated at both large farms and backyard farms may increase the likelihood of environmental contamination and the potential of antimicrobial waste to be recycled back to humans and animals through drinking water, through water used to irrigate short-cycle vegetables, and through animal manure used for agriculture.

The absence of designated antimicrobial waste collection centers at LGA and the lack of a tracking system for the waste degenerated from registered premises may result in antimicrobial waste mixed with regular household waste for disposal at the designated areas by the respective LGAs.

Additional gaps and the respective recommendations are summarized in the relevant section in table 2 below.

[80] AFCC2/RI-African Medicines Regulatory Harmonization Project (P128332). Report No: ICR127371: Implementation Completion and Results Report, June 29, 2018. Available from https://documents1.worldbank.org/curated/en/118891534182537235/pdf/ICR-for-the-African-Medicines-Regulatory-Harmonization-Project-P128332-Final-08082018.pdf

13. IDENTIFIED GAPS IN THE CURRENT POLICIES AND REGULATORY FRAMEWORK, AND RECOMMENDATIONS FOR ACTION

Tables 2 and 3 provide additional details on the identified gaps and recommendations in the animal health and human health sectors.

HUMAN HEALTH

Table 2. Gaps identified in current policies and regulatory framework and recommendations for action (human health sector)

Policy/Laws/Regulations/Guidelines/ Institutions	Identified Gaps and Issues	Recommendations
Current TMDA Act (2003) and Pharmacy Act (2011)	■ The two Acts, though exhaustive in many respects, lack sections that specifically address issues of AMR and AMS	In collaboration with its two regulatory bodies, the MOH should review the existing laws to address the gap.
MPGs for the implementation of the NHP	■ There is no National Medicine Policy as a unique document, but some elements are included in the NHP.	Through the MOH, responsible for policy issues, the government must reconsider its decision as this action excludes Tanzania from all other countries following WHO guidance.
1. Marketing Authorization, Licensing, and Inspection		
Market authorization, licensing, and inspection of pharmaceuticals (TMDA Act 2003 and Pharmacy Act 2011)	■ The market authorization has not yet included specific issues to address antimicrobials as a particular group but takes a general approach for all products.<br>■ Antimicrobials are included in the prescription-only medicines in the scheduling, but do not have their own specific schedule.	■ Review the existing sections of the TMDA Act addressing market authorization to have antimicrobials as a particular category with more restrictive sections on acquisition and level of use.<br>■ A similar review should be undertaken for the Pharmacy Act to add sections restricting dispensing specific antimicrobials at facilities that do not meet set legal requirements.<br>■ A legal requirement that enforces distributors and retailers to keep AMC data and report in line with a given schedule electronically should be included as a section in the reviewed Acts.<br>■ A national antimicrobial electronic distribution system should be developed to facilitate data visibility and enable the country to monitor its antimicrobial use and consumption profile.<br>■ The TMDA should design a mechanism that will monitor the adherence of MAH to PMS requirements of reporting on their products registered in the Tanzania market.
	■ The existing laws and regulations are silent on antimicrobials, particularly acquisition limitations based on service level and the required professional staffing.	■ The TMDA, PC, and Private Health facility establishment Acts should be reviewed to include explicit sections specifying antimicrobial acquisition, limitations, facility capacity to carry

Policy/Laws/Regulations/Guidelines/ Institutions	Identified Gaps and Issues	Recommendations
	■ Different studies observe a vast violation of the existing laws and regulations characterized by stocking medicines not approved for that level, dispensing prescription-only medicines without prescription, and nonprofessionals providing dispensing services.<br>■ Other violations include indiscriminate distribution of POM to premises not allowed to store and distribute such drugs, clinicians prescribing and dispensing, etc.	out susceptibility tests, and staffing required to store, prescribe, and dispense the relevant antimicrobials.<br>■ The TMDA and the PC should work in close collaboration to set up outlet inspection strategies to utilize the two regulatory bodies' financial and human resources available to carry out routine and unscheduled exhaustive inspections, and to take punitive actions against violators.
2. PMS and PV		
PMS and PV (TMDA Act 2003)	■ The PMS is functional; nevertheless, the sampling range seems small; it would be better to see a broader range of products being assessed, covering specifically antimicrobials in line with the fight against AMR, especially those in the Watch and Reserve categories.	■ The TMDA will have to readdress their PMS strategy to include antimicrobial products under the Watch and Reserve categories to follow up on their effectiveness.<br>■ In collaboration with the TMDA and the PC, the MOH should set strategies to develop a national data system on AMR use and consumption to create a national antibiogram that would allow visibility of the information on the level of resistance of antimicrobials in the market. Such a system would enable the MOH, the authorities, and service providers to make informed therapeutic and market authorization decisions.
3. SCM Policies, Laws, Regulations, and Guidelines Relevant to AMS		
SCM policies, laws, regulations, and guidelines relevant to AMS (TMDA Act 2003, Pharmacy Act 2011, STG/NEMLIT)	■ Wholesalers are not following the existing laws and regulations on the distribution of medicines to retailers and health facilities based on the level of services and the qualification of providers at that level.<br>■ Antimicrobials not approved for facilities such as ADDOs, private dispensaries, medical clinics, and health centers are found at these levels and dispensed, in many cases, without legal prescriptions.<br>■ There is unrestricted acquisition and distribution of antimicrobials in the private sector, defeating the effectiveness of the categorization per level of service, professional competence, and AWaRe enforcement in public health facilities.<br>■ The TMDA and MSD data on antimicrobials is mixed with that of other pharmaceuticals, making it more challenging to extract antimicrobial data for consumption assessment.	■ The TMDA, the PC, and the Registrar of private health facilities should include legal phrases in their existing laws and regulations that would make it mandatory for distributors and providers to have electronic systems in their business. The electronic system would show the flow of antimicrobials and allow regulatory inspectors to scan through the system, extract data when necessary, and obligate distributors to provide reports on distribution, specifically for Watch and Reserve categories.<br>■ Both authorities (TMDA and PC) should develop national systems that would follow the distribution of pharmaceuticals from the distributors to the end users.<br>■ The current Acts should be reviewed to see if a clear section regarding pharmaceutical distribution, especially of antimicrobials from suppliers to the end user, can be inserted. For the control to be effective, a supply chain monitoring system (possibly electronic) should be put in place for data visibility at all levels, from distributors to end-user facilities.<br>■ On the public side, the MSD, for the public sector distribution system, should establish an electronic system or a link with the existing systems being used at the zonal and facility levels to

Policy/Laws/Regulations/Guidelines/ Institutions	Identified Gaps and Issues	Recommendations
		monitor antimicrobial distribution, use, and consumption to have complete data visibility on AMC in the public sector.<br>▪ The TMDA and the MSD should develop a separate data system for imported and distributed antimicrobials, respectively, so that the system can monitor the antimicrobial distribution and use at the national level.
4. Prescribing and Dispensing Patterns in Private Health Facilities		
Prescribing and dispensing patterns in Private health facilities (Pharmacy Act 2011, STG/NEMLT, NHIF)	▪ Many private providers do not adhere to the STG/NEMLIT and other STGs and the AWaRe categorization. Private sector adherence to the prescription regulation 2021 is weak.[61]<br>▪ Although the private health facilities' registration is strictly per level of service and the required professional human resource, enforcement of the establishing Act and regulations is very weak.[58]<br>▪ Private health facilities can acquire medicines/antimicrobials of all levels regardless of the status and availability of professionals at that facility and do not observe the guidelines provided by the MOH.<br>▪ A mushrooming tendency of polyclinics has been observed, leading to broad violations in prescribing and use of antimicrobials. Furthermore, these polyclinics are being established under the old Act, which recognizes only regular clinics, with limitations including prohibiting storage of medicines and dispensing in a medical clinic except for emergency or diagnostic purposes, when specialist drug administration is required, or where dispensing authority has been granted by the MOH.[58] Unfortunately, the legal and operational functionality of the so-called polyclinics is unavailable and their compliance to the above limitations not present.	▪ The MOH should demand adherence to critical regulations and guidelines by the private sector, especially regarding prescribing criteria that follow requirements under the AWaRe categorization, and it should require private hospitals to establish MTCs and develop hospital formularies.<br>▪ There are reimbursement conditions set by the NHIF for public and private health facility members. The MOH should extend this requirement to be mandatory for all health insurance organizations in Tanzania. Furthermore, a special authority regulating health insurance schemes (private and public) should be established that will be able to centrally control and monitor adherence to the prescribing requirements and reimbursement conditions.<br>▪ It is recommended that the regulation allowing the establishment of the polyclinics be quickly reviewed to address, among other issues, the pharmaceutical services that are allowed to be provided at such facilities and the requirements of pharmaceutical human resources. Such a review is critical because polyclinics' establishment is still based on the provisions for medical clinics in the Private Health Facilities Act and regulations, prohibiting storing and dispensing medicines in such clinics.[58]
	▪ Community pharmacies such as the ADDOs dispense antimicrobials with or without a prescription, half-courses of antimicrobials, and sometimes ask the client no questions before dispensing. The dispensing without prescription at community pharmacies and ADDOs is also fueled by the fact that the regulatory bodies do not have strict follow-ups to control the medical practitioners. They prescribe and dispense in private health facilities for outpatients, especially in	▪ The MOH should review the Act establishing medical clinics, dispensaries, health centers, and private hospitals. The Act should be reexamined to insert specific sections with regulations to make prescribers aware of their limitations. Currently, clinicians in the private sector take it for granted that they can do what they are doing and do not see it as a legal violation.<br>▪ The TMDA, the PC, and the MOH quality department should institute stringent inspections and control for dispensing outlets

Policy/Laws/Regulations/Guidelines/ Institutions	Identified Gaps and Issues	Recommendations
	clinics, dispensaries, and health centers that store prescription medicines above their service levels.	and prescribing health facilities with a limited mandate to dispense prescription medicines. Restricting prescribing and dispensing by the private health facility clinicians would allow a free flow of clients with prescriptions from private health facilities as sources to community pharmacies for dispensing, creating fair play between the providers.
TMDA Act, 2003 Cap. 219; TMDA (Scheduling of Medicines) (Amendment) Regulations, 2018	■ In many public health facilities, especially at the primary health care level, dispensers have a lower level of professional training; pharmacists are underemployed and therefore are rarely dispensing or directly supervising the dispensing process.<br>■ The insufficiently trained staff have little authority to question a medical doctor's prescription, and therefore the antimicrobial prescription may go through without critical evaluation and seeking justification for the prescription.	■ In collaboration with its regulatory authorities, the MOH should review and establish stringent pharmaceutical cadre establishment at each level of service (public and private) and vigorously monitor adherence.<br>■ The PC should review the conditions for the pharmacist superintendent, making their availability change from part-time to full-time now that not having enough pharmacists in the country is no longer a problem. Pharmacists should be employed full-time at all pharmacies.<br>■ Since the number of pharmaceutical technicians, pharmacy assistants, and one-year dispensers has increased substantially, the PC needs to move further to require pharmaceutical services at ADDOs, dispensaries, and health centers to be provided by these cadres and be stringently monitored and implemented.

ANIMAL HEALTH

Table 3. Gaps identified in current policies and regulatory framework and recommendations for action (animal health sector)

Policy/Laws/Regulations/ Guidelines/Institutions	Identified Gaps and Issues	Recommendations
Livestock Policy 2006 and Fisheries Policy 2015	■ The two policies do not explicitly address AMS and lack a strategy for AMS.	■ The MLF should consider including AMS in the review of the policies.
Livestock Development Master Plan; Fisheries Development Master Plan	■ The TLDMP indirectly addresses AMS.<br>■ The documents are not aligned with the NAP-AMR.	■ Consider aligning the documents by including specific goals related to AMR, and particularly AMS
Marketing Authorization, Licensing, and Inspection		
TMDA Act, 2003, Cap. 219; TMDA (Registration of Medicinal Products) Regulations, 2015**Error! Bookmark not defined.**	■ Weak integration of MLF in the current decision-making process on the regulation of antimicrobials<br>■ Weak data-sharing mechanism between the MLF and the TMDA	■ Integrate the DVS, the VCT, and the Directorate of Fisheries in the MAB to properly advise on regulating antimicrobials for use in the animal health sector and strengthen the linkage between the MLF and the MOH.<br>■ Consider integrating the DVS in decisions regarding import and registration beyond the advisory role.

Policy/Laws/Regulations/ Guidelines/Institutions	Identified Gaps and Issues	Recommendations
		▪ The MLF should set policies that will guide the TMDA when evaluating proposals for antimicrobial registration, imports, and/or manufacture.<br>▪ The TMDA and the MLF should consider creating a system of real-time data exchange between the DVS, the VCT, and the TMDA to facilitate coordinated regulatory activities and timely use of the collected data.
TMDA Act, 2003, Cap. 219	▪ The administrative structure of the TMDA, especially the Human and Veterinary Medicine Directorate, may contribute to inadequate attention to some matters related to animal health.	▪ The TMDA should restructure the Human and Veterinary Medicine Directorate to create two separate Directorates that will be more focused and effective in each sector.
Veterinary Act, 2003	▪ The VCT registers retail drug outlets, regulates the conduct of veterinary professionals and paraprofessionals, and market authorization for acaricides. Decisions on these critical functions will likely be influenced if the Council has less autonomy.<br>▪ The VCT is currently functioning as a department at the MLF. This may create undue influence and inefficiency in its regulatory function.	▪ MLF should consider restructuring to make VCT function as a semiautonomous entity rather than its current status where it operates as a department within the MLF.
The Tanzania Medicines and Medical Devices (Fees and Charges) Regulations, 2021[81]	▪ The high cost of product registration caused by the package-based charging system for antimicrobials administered through the parenteral route may be one of the reasons for noncompliance.	▪ The TMDA should strengthen awareness among stakeholders on different requirements for quality control evaluation between products meant for parenteral use and nonparenteral use.
Tanzania Livestock Inspection Framework<br>The Veterinary Act (Procedures for Inspection of Veterinary facilities Regulations), 2005	▪ Only 40% of mandatory inspection required to be conducted by the MLF is done due to financial and human resources constraints.	▪ The MLF should consider employing veterinarians in all LGAs, at least up to the district level.
PMS and PV		
PMS	▪ Currently, PMS is functional but limited in terms of products and geographical coverage. For example, in 2019/2020, only one product was tested. The number of regions reached ranged from 2 to 6 out of 26 regions in mainland Tanzania.	▪ Consider increasing the range of products and geographical coverage when conducting independent PMS
TMDA PV Regulations (2018);<br>National Guidelines for Monitoring Medicines Safety (2010);	▪ The existing PVS completely excludes the animal health sector.	▪ The TMDA (MOH) is considering collaborating with the DVS (MLF) to establish and implement PV in the animal health sector.

[81] Tanzania Medicines and Medical Devices (Fees and Charges) Regulations, 2021.

Policy/Laws/Regulations/ Guidelines/Institutions	Identified Gaps and Issues	Recommendations
The National PV Roadmap (2019–2023)		
Supply Management Policies and Guidelines Relevant to Antimicrobials		
Livestock Policy 2006; TLDMP (2017–2022)<br><br>MLF Budget Speech, 2022/2023	■ The mode of delivery of veterinary services disadvantages areas that are less attractive to private entrepreneurs, such as rural locations; therefore, the vacuum is filled by an unregulated informal supply chain.<br>■ Although the Government continues to complement where there is a deficit, as stipulated in the TLDMP, a critical shortage of extension staff undermines this strategy. For example, 3,642 of the required 14,025 livestock extension staff were available as of June 2022 (MLF Budget Speech 2022/2023).	■ The MLF should collaborate with LGAs to increase the number of animal health extension officers as part of the implementation of the TLDMP.<br>■ Priority should be given to underserved areas as part of public interventions in areas that are less attractive for the private sector to offer veterinary services.
Prescription, Dispensing, and Use		
Guideline for ADDOs, annex 6 "*Mwongozo wa usajili wa maduka ya dawa muhimu za mifugo*" 2015 (Guidelines for registration of essential veterinary drugs)	■ All antimicrobials that are administered orally are categorized as General Sales Medicines.<br>■ Only antimicrobials for the parenteral route are categorized as POM.<br>■ However, some oral formulations categorized as General Sales in the guidelines are registered as POM.	■ The TMDA should revise the guidelines to update it to exclude antimicrobials from the list of General Sales Medicines.
The TMDA (Scheduling of Medicines) (Amendment) Regulation 2018;<br>The Veterinary Act (Veterinary Practice by Paraprofessionals and Paraprofessional Assistant) Regulation 2005	■ The TMDA (Scheduling of Medicines) (Amendment) Regulation 2018 allows veterinary paraprofessional assistants to prescribe POM.<br>■ The Veterinary Act (Veterinary Practice by Paraprofessionals and Paraprofessional Assistant) Regulation 2005 only allows veterinarians and veterinary paraprofessionals to prescribe and dispense medicines. The latter work under the supervision of the veterinarians.<br>■ There is a critical shortage of the formal workforce (veterinarians, veterinary paraprofessionals, and vet paraprofessional assistants).<br>■ The STG for the animal health sector is not available to stakeholders.<br>■ There are no authorized antimicrobials for use in aquaculture.	■ The TMDA (Scheduling of Medicines) (Amendment) Regulation 2018 and The Veterinary Act (Veterinary Practice by Paraprofessionals and Paraprofessional Assistant) Regulation 2005 should both be harmonized.<br>■ The MLF should consider employing veterinarians in all LGAs, at least up to the district level.<br>■ In some areas, the lack of skilled vet professionals and paraprofessionals limits compliance. Considerations should be made to employ more paraprofessionals, at least up to the ward level.<br>■ The MLF should expedite the dissemination of animal health STG and consider widening its scope to include aquaculture animals.<br>■ Evaluate the current situation on the need for antimicrobials in the aquaculture sector to minimize illegal and imprudent use of antimicrobials not certified for use in aquaculture.
The Veterinary Act (Code and professional conduct for Veterinarians and Veterinary	■ Veterinarians, veterinary specialists, veterinary paraprofessionals, and paraprofessional assistants are obliged to keep all medical records. However, there is no	■ Consider enforcing a centralized case management system that records cases and treatments, starting with registered facilities as a pilot. The system can be monitored remotely and can serve as

Policy/Laws/Regulations/ Guidelines/Institutions	Identified Gaps and Issues	Recommendations
Specialists) Regulations, 2005;<br>The Veterinary Act (code of conduct for Veterinary Paraprofessionals and paraprofessional assistants) Regulations, 2005	efficient audit mechanisms to enforce this, especially in ambulatory services.<br>▪ All Veterinarians are obliged to report any adverse drug effect to the local state. This requirement is difficult to enforce.	dispensing register for veterinary centers that can be audited against the original stock of pharmaceuticals.<br>▪ Consider collaboration with the TMDA to develop a system that can accommodate farmers, paraprofessionals, and paraprofessional assistants for reporting adverse drug effects as part of the proposed PVS for the Animal health sector.
The Grazing-Land and Animal Feed Resources Act, 2010, Cap. 180;<br>The Grazing-Land and Animal Feed Resources (Registration and Movement of animal feed resources and products) Regulations, 2021, GN. 327;<br>The Standards for Animal Feeds Regulations, 2013, GN. 58	▪ In the regulation for registration and movement of animal feed resources and products (GN. 327), only growth promotors and antibiotics are non-registrable animal feed resources. In contrast, the regulation is silent on other antimicrobials.<br>▪ GN 58 sets the maximum limit of antibiotics in animal feeds to 0.22% but it is not clear if the limitation applies to individual components of antibiotics or if the limit is too high and contradicts the regulation of registration and movement of animal feed resources.<br>▪ Certification of animal feeds is not centralized. No clear mandate between MLF, TVLA, and TBS.	▪ The MLF should consider repealing the Standards of Animal Feeds Regulation of 2013, GN 58.<br>▪ Review the GN 327 to include other types of antimicrobials as nonregistrable feed substances<br>▪ The MLF and the Ministry of Industry and Trade should consider harmonizing procedures for animal feed certification, which should be done by one institution.<br>▪ The TVLA can be certified by the TBS to perform all animal feed certifications on its behalf under the Standards Act, Cap. 130.
Dairy industry Act, Cap. 262;<br>Dairy industry (raw milk grading and minimum quality and safety requirements) (amendment) regulations, 2020	▪ The Regulations prohibit the sale of milk with veterinary drug residues. However, enforcement of this is challenging, as most of the dairy sector operates under an informal supply chain.<br>▪ There is a limited number of milk collection centers.<br>▪ There are 107 dairy milk processing enterprises countrywide.	▪ The MLF, through the TDB, prioritizes and promotes investment in milk collection centers through which antimicrobial residue checks can be implemented along with the conventional checks.<br>▪ The Government should consider providing incentives to milk processors to attract more investment and support more dairy producers to be integrated into a formal milk supply chain that can easily be regulated.
The Meat Industry Act, 2006<br><br>Standards Act, Cap. 130<br><br>The Livestock Identification, Registration and Traceability Act No.12 of 2010	▪ The legislation lacks provisions that control the use of antimicrobials in food animals, particularly for meat production.<br>▪ However, antimicrobial residues in animal products are covered under the Standards Act.<br>▪ Enforcement of the standards in domestically produced animal products is deficient.<br>▪ Informal meat and egg production makes it difficult to regulate standards concerning antimicrobial residues.	▪ The Livestock Identification, Registration, and Traceability Act, 2010, Cap. 184 makes the reports on the history of treatment to be mandatory for all large animals brought for slaughter, to support compliance with the Standards Act, Cap. 130.[82]<br>▪ The MLF should consider using an electronic tracking system for auditing information provided in the movement permits on treatment history against ad-hoc surveillance meat samples collected and tested for antimicrobial residues.
Animal Diseases (Acaricide Application and Management) Regulation, 2019	▪ The DVS must check and monitor tick resistance and recommend it accordingly. However, there is no prior plan for withdrawal of acaricides that encountered resistance.	▪ The MLF should develop a mechanism (regulations) for dealing with acaricides that are no longer effective.<br>▪ Acaricide suppliers should be responsible for regularly conducting PMS and submitting the report to the DVS.

[82] The Livestock Identification, Registration and Traceability Act, Cap. 184. 2010. Available from https://www.mifugouvuvi.go.tz/uploads/publications/sw1619685333-IDENTIFICATION%20ACT%202010.pdf

Policy/Laws/Regulations/ Guidelines/Institutions	Identified Gaps and Issues	Recommendations
	▪ AMS interventions exclude the link between acaricide resistance and irrational use of antimicrobials.<br>▪ Ward and village livestock field officers are expected to manage acaricide application centers, but only 50% of the needed officers are available.	▪ The DVS, through authorized laboratories, should conduct regular PMS of acaricides to monitor quality.<br>▪ The MLF should consider employing more extension officers, at least up to ward level.
Environmental Management (Hazardous Waste Control and Management) Regulations, 2021;<br>TMDA (Recall, Handling and Disposal of Unfit Medicines) Regulations, 2015	▪ Antimicrobial waste generated at homes, farms and backyard farming systems are not covered by the regulations; instead, this is left to LGAs.<br>▪ LGAs do not have a system in place to handle such waste.<br>▪ No system to track compliance with waste disposal at health facilities	▪ The MLF, the NEMC, the TMDA, and LGAs should consider developing a mechanism to handle domestic antimicrobial waste.<br>▪ The NEMC and the MLF should consider developing an electronic log for tracking the disposal of antimicrobial and antimicrobial-contaminated waste in large farms and health facilities.

14. CONCLUSION

The review has revealed that despite Tanzania Mainland having relevant policies, strategies, laws, regulations, and guidelines in place, there are gaps in the implementation characterized by weak regulatory control and the inexistence of systems in place that can monitor, audit, and evaluate antimicrobial distribution, consumption, and waste disposal in both human and animal health sectors. There is also a weak strategy to fully include private human health providers in the fight against AMR and the establishment of AMS programs. This weakness is characterized by the current trend of government and implementing partners primarily targeting the public health system to implement AMS programs, while leaving the private sector at liberty.

The MAB is a vital organ that can significantly impact the overall regulatory functions performed by the TMDA. As defined in the current TMDA Act, Cap. 219, a broader and inclusive MAB may bring novel and complementary ideas, covering all core areas of TMDA regulatory functions that may benefit the MOH. It is also recommended to review the current structure and operations of the TMDA directorate responsible for human and veterinary medicine registration. A splitting of the directorate into two sections/directorates is suggested. This process would create more focused decision making and better interinstitutional collaboration between the TMDA and the respective Ministries without affecting the TMDA mandate.

Furthermore, the MLF should revise the current administrative structure where the VCT operates as a department within the MLF. This mode of operation makes the VCT vulnerable to undue influence that could undermine the objectives of the Council.

The TMDA and the MLF, through the DVS and the VCT, should consider improving data-sharing mechanisms and coordination to promote synergy between regulatory authorities in the respective Ministries responsible for human and animal health. This initiative should not exclude the PC and other relevant professional councils that could support the regulatory functions.

Lastly, the respective Ministries should urgently consider reviewing policies and legislations that are not consistent with the current NAP-AMR and the AMS policy implementation guidelines. They should also consider a strategy on how to improve waste handling and disposal at different levels, including having a robust monitoring mechanism to track and monitor compliance on antimicrobial waste disposal.

ANNEX 1. LIST OF POLICIES, LAWS, REGULATIONS, AND GUIDELINES RELEVANT TO AMS IN HUMAN AND ANIMAL HEALTH

POLICIES

1. National Health Policy (NHP), 2003
2. Medicines Policy Guidelines for implementation of the NHP
3. National Livestock Policy, December 2006
4. National Fisheries Policy, 2015

ACTS OF PARLIAMENT

1. Tanzania Medicine and Medical Devices Authority Act, Cap. 219
2. Finance Act, No. 8, 2019
3. Veterinary Act, Cap. 319
4. Animal Disease Act, Cap. 156
5. The Dairy Industry Act, Cap. 262
6. The Meat Industry Act, Cap. 421
7. The Standards Act, Cap. 130
8. The Environmental Management Act, Cap. 191
9. The Grazing-Land and Animal Feed Resources Act, Cap. 180
10. Private Hospital (Regulations) Act, Cap. 151
11. The Livestock Identification, Registration, and Traceability Act, Cap. 184
12. Pharmacy Act, Cap. 311

STATUTORY INSTRUMENTS: REGULATIONS, ORDERS

1. The Environmental Management (Hazardous Waste Control and Management) Regulations, 2021
2. TMDA-Pharmacovigilance Regulations (2018)
3. The Grazing-Land and Animal Feed Resources (Registration and Movement of Animal Feed Resources and Products) Regulations, 2021
4. Standards of Animal Feed Resources Regulations, 2013
5. Dairy Industry (Raw Milk Grading and Minimum Quality and Safety Requirements) (Amendment) Regulations, 2020
6. The Tanzania Food, Drugs, and Cosmetics (Registration of Medicinal Products) Regulations, 2015
7. The Tanzania Medicines and Medical Devices (Good Manufacturing Practices Enforcement) Regulations, 2018
8. TMDA Medicine Scheduling Regulations, 2018
9. The Tanzania Food, Drugs, and Cosmetics (Registration of Premises, Importation, and Exportation of Pharmaceutical Products and Raw Materials) Regulations, 2015
10. The Pharmacy (Premises Registration) Regulations, 2020
11. The Pharmacy (Pharmacy Practice and the Conduct of Business of a Pharmacy) Regulations, 2020
12. Animal Diseases (Acaricide Application and Management) Regulation, 2019
13. TMDA (Registration and Licensing of Premises for Veterinary Products) Regulations, 2018
14. The Veterinary (Procedures for the Inspection of Veterinary Facilities) Regulations, 2005

15. The Veterinary Act (Code of Professional Conduct and Ethics for Veterinarians and Veterinary Specialists) Regulations, 2005
16. The Veterinary Act (Code of Conduct for Veterinary Paraprofessionals and Paraprofessional Assistants) Regulations, 2005
17. The Pharmacy (Prescription Handling and Control) Regulations, 2020
18. Orodha ya dawa zinazoruhusiwa katika Duka La Dawa Muhimu (ADDO) (Kanuni ya "The Tanzania Food, Drugs, and Cosmetics (Scheduling of Medicines) Regulations, 2015") (GN. No. 63 of 2015)
19. TMDA (Recall, Handling, and Disposal of Unfit Medicines) Regulations, 2015
20. The Veterinary Act (Veterinary Practice by Paraprofessionals and Paraprofessional Assistant Regulation), 2005

GUIDELINES AND CIRCULARS

1. Policy Guidelines for Implementing Antimicrobial Stewardship. June 2020
2. Medicines Policy Guidelines for Implementation of NHP
3. Standard Treatment Guidelines & National Essential Medicines List, Tanzania Mainland, 2021
4. Standard Treatment Guidelines for Animal Diseases in Tanzania
5. National Guidelines for Monitoring Medicines Safety (2010)
6. Tanzania Livestock Development Master Plan (2017/2018, 2021–2022)
7. TMDA Compendium Guidelines for Market Authorization of Medicinal Products (2020)
8. Guidelines on Submission of Documentation for Renewal of Registration of Human and Veterinary Pharmaceutical Products
9. TMDA Guidelines on Submission of Documentation for Registration of Veterinary Medicinal Products (2020)
10. Mwongozo wa usajili wa maduka ya dawa muhimu za mifugo, 2015, TFDA/DMC/MI&E/G/011
11. TMDA Compendium Guidelines for Market Authorization of Medicinal Products (2020)
12. The National Action Plan on Antimicrobial Resistance (NAP-AMR) 2017–2022, United Republic of Tanzania

ANNEX 2. LIST OF KEY INFORMANTS IN THE STAKEHOLDER INTERVIEWS (HUMAN HEALTH)

#	Participant's Position*	Designation/Responsibilities	Name of Organization
1	Mrs. Elizabeth Shekalaghe	Registrar, Pharmacy Council	Pharmacy Council of Tanzania
2	Dr. Raphael E. Maltaba	Acting Quality Assessment and Claim Verification Manager	NHIF
3	Mr. Emmanuel Alphonse	Manager, Responsible for Drug Registration and Control	TMDA
4	Mr. Noel Muhadu	Ag. Procurement Manager, Pharmaceuticals	MSD
5	Mr. Richard Makala	Procurement officer, Pharmaceuticals	MSD
6	Dr. Ibadi Kaondo	Clients demand analysis	MSD
7	Ms. Grace Butoboro	Pharmacist, Quality assurance officer	MSD

ANNEX 3. LIST OF KEY INFORMANTS IN THE STAKEHOLDER INTERVIEWS (ANIMAL HEALTH)

#	Participant's Position*	Designation/Responsibilities	Name of Organization
1	Mr. Alex Juma Ismail	Drug Registration Officer/Evaluation of Medicinal Products	TMDA
2	Mr. Felchism Apolnary Oisso	Ag. Manager, Medicine Registration; oversees Medicines Registration Activities	TMDA
3	Dr. Athanas Richard Mseki	Drug Registration Officer/Review of Product application dossiers	TMDA
4	Dr. Obed Malangu Nyasebwa	Acting Registrar/Regulation of Veterinary Professionals and Facilities	VCT, MLF
5	Ms. Mariam Juma Mgendwa	Legal Officer/handles all legal matters at MLF	MLF
6	Dr. Michael Japhet Madege	Assistant Director, Transboundary Disease Control/Disease Surveillance and Control	MFL
7	Dr. George Mutani Msalya	Registrar of the Board	TDB, MLF
8	Mrs. Stella Sebastian Balili	Dairy Production Officer/Registration of stakeholders, farmers' education on good milk management (from farm to the table)	TDB, MLF
9	Mr. Geofrey Kakuru Sosthenes	Livestock Officer/Inspection of Meat Industry Stakeholders	Tanzania Meat Board, MLF
10	Mr. Rogers Michael Shengoto	Assistant Director, Directorate of Grazing Land and Animal Feed Resources (DGLF)/ Head of Livestock Feed Resources Section	MLF
11	Mr. Alfred Elisa Msokwa	Principal Environmental Management Officer/Environmental Impact Evaluation Standards	NEMC
12	Mrs. Gladness Gilliard Malisa	Environmental Impact Assessment Officer/Environmental Impact Assessment	NEMC
13	Dr. Nassoro Athuman Mopei	Veterinary Officer, Dar es Salaam City Council/Supervise Veterinary Services, Appointed Inspector by MLF	Dar es Salaam City Council, Ilala, Dar es Salaam
14	Dr. Betila Aloyce Badanga	Veterinary Doctor/Offer Veterinary Services (Private Veterinary Services)	VET-FARM
15	Dr. Pius Daniel Ntanga	District Veterinary Officer/Supervise/Inspection of Veterinary services	Kinondoni Municipal Council
16	Mr. Boaz Magito Ndamo	Chief Executive Officer; oversees operations	Magito Store Co. Ltd (Dealers of Veterinary Medicinal Products)
17	Ms. Mwajuma Iddi Dukulai	Standards Officer/Development of National Standards in the Food and Agricultural Sector	TBS
18	Mr. Mataba Ikumbo Kalelema	Agricultural Officer (Plant Health)/Plant Health and Pest Control	Ministry of Agriculture
19	Dr. Gibonce Andrew Kayuni	Assistant Director/AMR Focal Person, Directorate of Veterinary Services	MLF
20	Dr. Hamisi L. Nikuli	Assistant Director/Disease Control in Aquatic Animals	MLF

ANNEX 4. LIST OF MCC/TWG MEMBERS WHO PARTICIPATED IN THE DISSEMINATION/FEEDBACK MEETING (SITUATIONAL ANALYSIS REPORT PRESENTATION)

Day II

Sections/cells that are not colored can be updated with data
Sections/cells colored in Green are filled in with formulas and will update automatically
*You may use as many log sheets as needed for complete number of participants or attendees.

PATH

PARTICIPANT ATTENDANCE LOG

EVENT OR ACTIVITY TITLE	AMR NATIONAL ACTION PLAN REVIEW WORKSHOP
EVENT CORDINATOR	BACHANA RUBEGWA
EVENT OR ACTIVITY DATE(S)	7-12 SEPTEMBER 2022
EVENT LOCATION	GOLDCREST HOTEL

Day II - 8/9/2022

S/n	Participant Name	Position held	Salary scale	Institution	Region	Phone number	Email address	Signature
1.	Talhiya Yahya	STA		MSH MTaPS	Dsm	0717224461	eyahya@mtapsprogram.org	[signature]
2.	VIVIAN J. WONANJI	PDRO	LSSE3	TMDA	[illegible]	0769553613	vtwonanji@[illegible]	[signature]
3.	ABUBAKAR HOZA	S/Lecturer	PUTS 4	SUA	Morogoro	0674970072	abshoza@gmail.com	[signature]
4	ALPHALODGN KYELUZA	DRIVER	TGS-D	MOH	DODOMA	0755-884319	[illegible]@gmail.com	[signature]
~~5~~	~~SIMBA KASANE~~	~~DRIVER~~						
6.	ALFRED CHACHA	Deputy Manager	TGSD	NPHL	DAR	0767431721	alfred_chacha@yahoo.com	[signature]
7.	STANFORD NDIBALEMA	Ass. DIRECTOR	LSSE2	MLF	Dodoma	0757597022	stanford.ndibalema@mlf.go.tz	[signature]
8	Erick V. Komba	Consultant	N/A	ASM	DODOMA	0713584054	ekomba@sua.ac.tz	[signature]
9.	SIGIFRID MTEY	IEO	TMDA S.S.1	TMDA	DSM	0653649019	sigimtey@gmail.com	[signature]
10	DR. BALTAZAR G. LEBA	DMD COORDINATOR	TGS-G	PMO	DOM	0710899642	baltazar.leba@pmo.go.tz	[signature]
11	EDWIN MKWAMA	CAE Coordinator	TGHS G	CDI	MOROGORO	0766903246	mkwama@gmail.com	[signature]
12.	DAVID MBAGA	DRIVER	TGSD	NPHL	DAR	0716020125	—	[signature]
13	ALBERTO MWAKIPESILE	DRIVER	PGSS 3.3	SUA	MORO	0718797278	albertomwakipesile55@gmail.com	[signature]

Prepared by: Mary Bukombe
Title: Senior Program Assistant
Signature & Date [signature] 8/9/2022

Verified by: Bachana Rubegwa
Title: AMR Advisor
Signature & Date [signature] 8/9/2022

Day II

Sections/cells that are not colored can be updated with data
Sections/cells colored in Green are filled in with formulas and will update automatically
*You may use as many log sheets as needed for complete number of participants or attendees.

PATH

PARTICIPANT ATTENDANCE LOG

EVENT OR ACTIVITY TITLE	AMR NATIONAL ACTION PLAN REVIEW WORKSHOP
EVENT CORDINATOR	BACHANA RUBEGWA
EVENT OR ACTIVITY DATE(S)	7-12 SEPTEMBER 2022
EVENT LOCATION	GOLDCREST HOTEL

Day II – 8/9/2022 (day II)

S/n	Participant Name	Position held	Salary scale	Institution	Region	Phone number	Email address	Signature
14 ✓	FIONA CHILUNDA	ADVISOR	MA	T/AFYA	DSM	0784459055	fiona.chilunda@[illegible]	[illegible]
15 ✓	ZUBERI ABDALLAH	PHARMACIST	TGHSG	PORALG	DSM	0789992228	zubeabdi@yahoo.com	[illegible]
16 ✓	SAMIR SAIFUTI	CL P'GIST	TGHSG	UDOM	DOM	0767-250800	chamicheka@gmail.com	[illegible]
17 ✓	DR. JUSTINE ASSENGA	PVO	TGSJ	MOLF	Dodoma	0753860704	asseagola@yahoo.com	[illegible]
18 ✓	Ambele Mwafulango	MANAGER	TGHSE	NPAL	DSM	0716078422	ambele.mwafulango@afya.go.tz	[illegible]
19 ✓	MWAJUMA I. DUKULAI	STANDARDS OFFICER	TBSS8	TBS	DSM	0784931003	mwajuma.dukulai@tbs.go.tz	[illegible]
20 ✓	ALEXANDER MZULA	LECTURER	PUTS 3.3	SUA	MOROGORO	0767022411	amzula@sua.ac.tz	[illegible]
21 ✓	SIANA MAPUNJO	MCC Secretary		NIMR	DOM	0713566720	[illegible]	[illegible]
22 ✓	ROBINSON MDEGELA	Prof	PUTS 6.3	SUA	MORO	07824378[illegible]	mdegela@sua.ac.tz	[illegible]
23 ✓	Emiliana Francis	AMR COORDINATOR	TGHSG	MOH	DOM	0757063503	emmyjra@yahoo.com	[illegible]
24 ✓	~~GODWIN KULWA~~	~~LSC~~	~~TGHS-G~~	~~MUH~~	~~DSM~~	~~0788982076~~	godwg@gmail	[illegible]
25 ✓	AMOSI LAZARO	Serena program	NRF	DSM	0784650585	—	[illegible]
26 ✓	SIMBA KASAMBA	DRIVER	Program	MLF	DOM	0713710095	—	[illegible]

Prepared by: Mary Bukombe
Title: Senior Program Assistant
Signature & Date [illegible]
8/9/2022

Verified by: Bachana Rubegwa
Title: AMR Advisor
Signature & Date [illegible]
8/9/2022

Day II

Sections/cells that are not colored can be updated with data
Sections/cells colored in Green are filled in with formulas and will update automatically
*You may use as many log sheets as needed for complete number of participants or attendees.

PATH

PARTICIPANT ATTENDANCE LOG

EVENT OR ACTIVITY TITLE	AMR NATIONAL ACTION PLAN REVIEW WORKSHOP
EVENT CORDINATOR	BACHANA RUBEGWA
EVENT OR ACTIVITY DATE(S)	7-12 SEPTEMBER 2022
EVENT LOCATION	GOLDCREST HOTEL

Day II - 8/9/2022

	S/n	Participant Name	Position held	Salary scale	Institution	Region	Phone number	Email address	Signature
27	1	DEODATUS KAKOKO	ASS PROF.	PUTS 5.1	MUHAS	DAR	0716-538030	deodatus kakoko @ gmail.com	[illegible]
28	2	Grace Mwasyela	PS	TGS D	MLF	Dom	0657 483155	grace mwasyela @ gmail.com	[illegible]
	3	Gladness Malisa	EMO	PGSS 6	NEMC	DSM	0752364553	gladness.malisa2@nemc.or.tz	[illegible]
30	4	Obed Nyasebwa	DRVCT	LSSE	MLF	DOM	0689825862	obedmalangue gmail.com	[illegible]
31	5	JOSEPH E. CHILONGANI	PRIN. Res. SCIENTIST	PRSS/7/1	NIMR	DOM	0756449218	jchilongani@ gmail.com	[illegible]
32	6.	REHEMA MBULALINA	HGCU	LSSE	MLF	DOM	0713405091	rehemah @ hotmail.com	[illegible]
33	7	Rose Shija	EDM		WHO	DSM	0754222263	shijar@who.int	[illegible]
34	8.	Gibonce Kayuni	MCC Secretariat	TGS J	MLF	Dom	0655421010	kayunigg@gmail.com	[illegible]
35	9	Reuben Abednego	Lab. Scientist	TGHS G	NPHL	DSM	0717469616	reubenabednego@gmail.com	[illegible]
36	10	ZACHARIAH MAKONDO	MBAU	TVLA 12	TVLA	DSM	0762968829	zmakondo@gmail.com	[illegible]
37	11	Doreen Mloka	S/Lecture	PUTS 4.5	MUHAS	DSM	0712459528	dmloka@yahoo.com	[illegible]
38	12	MTEBE MAJIGO	S/LECTURER	PUTS 4	MUHAS	DSM	0754265561	mmajigo@gmail.com	[illegible]
39	13	AKILI MAWAZO	Microbiologist	TGHS G	MOA	DSM	0784919809	akilimawazo@gmail.com	[illegible]

Prepared by: Mary Bukombe
Title: Senior Program Assistant
Signature & Date [illegible] 8/9/2022

Verified by: Bachana Rubegwa
Title: AMR Advisor
Signature & Date [illegible] 8/9/2022

Day II

Sections/cells that are not colored can be updated with data
Sections/cells colored in Green are filled in with formulas and will update automatically
*You may use as many log sheets as needed for complete number of participants or attendees.

PATH

PARTICIPANT ATTENDANCE LOG

EVENT OR ACTIVITY TITLE	AMR NATIONAL ACTION PLAN REVIEW WORKSHOP
EVENT CORDINATOR	BACHANA RUBEGWA
EVENT OR ACTIVITY DATE(S)	7-12 SEPTEMBER 2022
EVENT LOCATION	GOLDCREST HOTEL

Day II - 8/9/2022

	S/n	Participant Name	Position held	Salary scale	Institution	Region	Phone number	Email address	Signature
40	1	AUGUSTINE MATONDO	S/LEG.	PUT 4	SUA	MORO	0743450361	matondo@sua.ac.tz	[signature]
41	2	D. Romuald Mbwasi	S/L	-	SJUT	DODOMA	0767 202235	rmbwasi@gmail.com	[signature]
42	3	Longinus Tegulirwa	PEO	TGS H	MLF- FLSU	DARESSALAAM	0787026173	tegulirwa@yahoo.com	[signature]
43	4	Kaklima matabo	AO	TGSD	MOA	DODOMA	0769 224740	ikalelema@gmail.com	[signature]
				STAFF					
44		Mary Bukombe	SN Progm	N/A	IDDS	DSM	0767020904	[illegible]	[signature]
45		Bachana Rubegwa	AMRADV	—	IDDS	DSM	0769337332	BRubegwa@Path.org	[signature]

Prepared by: Mary Bukombe
Title: Senior Program Assistant
Signature & Date [signature] 8/9/2022

Verified by: Bachana Rubegwa
Title: AMR Advisor
Signature & Date [signature] 8/9/2022

ANNEX 5. IMPLEMENTATION STATUS OF SOME OF THE ACTIVITIES UNDER STRATEGIC OBJECTIVE 4 OF THE NAP-AMR 2017–2022

Strategic Intervention	Activities	Indicator	Comments
	Develop guidelines for handling and preservation of antimicrobial agents		
Develop and review policies, laws, and guidelines on the use of antimicrobial agents in human	Prepare a draft of the guidelines<br>Conduct stakeholders' validation meeting<br>Printing and distribution	Guidelines for handling and preservation of antimicrobial agents developed	Policy Guidelines for Implementation of Antimicrobial Stewardship developed, approved, and disseminated
	Develop/review National Medicines and Policies to address AMR		
	Review a draft of the policy<br>Conduct stakeholders meeting<br>Prepare cabinet paper<br>Approval of the policy	National policy developed/reviewed medicines	The National Policy 1991 is not reviewed due to Government policy directives; i.e., we should have only one policy in the sector (the NHP). However, an MPG has been developed for the implementation of the Medicines Policy, which is in the process of approval.
	Develop/strengthen regulations on the use and control of prescriptions		
	Prepare draft regulations under the Pharmacy Act, Cap. 311 and Animal disease Act, No.16 of 2003<br>Conduct stakeholders' validation meetings<br>Printing and dissemination of regulations	Regulations on the use and control of prescriptions in place	The Pharmacy (Prescription Handling and Control) Regulations, 2020 are available, but enforcement, especially in the private sector, is weak.
	Review STG/NEMLIT for human medicine, including antimicrobials		
	Review the guidelines<br>Conduct stakeholders validation meeting<br>Printing and distribution	STG/NEMLIT for human medicines, including antimicrobials, reviewed and in place	The STG/NEMLIT 2017 edition was reviewed, and the STG/NEMLIT 2021 edition is available. The antibiotics are categorized into AWaRe groups.
Monitor proper use of preserved antimicrobial agents	Conduct regular inspections of pharmaceutical and agrochemical outlets		
	Establish joint inspection teams<br>Develop inspection tools including checklist<br>Conduct training to inspectors<br>Procure vehicles and motorcycles for inspection<br>Conduct joint inspection	# of inspections carried out jointly	The survey on the use of antibiotics is conducted in 16 public hospitals. Also, training materials for RUM, including antimicrobial developed. The training of health care workers was conducted in 10 hospitals.<br><br>Also, supportive supervision and mentorship on RUM were conducted in a few hospitals.
	Conduct refresher training on professional ethics and enforcement on the use of antimicrobial agents		
	Conduct training needs assessments<br>Develop training programs<br>Conduct training of trainers<br>Conduct training for professionals	# Training sessions and # of trainees	The material was developed, and the training was conducted in all hospitals under the MTaPS project.
	Establish a virtual networking platform for the use of preserved antimicrobials agents		
	Procure relevantly virtual networking equipment<br>Establish a central database on use and resistance of antimicrobial agents	Virtual networking platform on the use of preserved antimicrobial agents developed	The establishment of the virtual network is in progress. Several consultative meetings have been conducted.

Strategic Intervention	Activities	Indicator	Comments
	Conduct training to designated officers on the use of virtual equipment		
	Operationalize and monitor virtual platform		
	Develop health facility and other AMR policies		
Establish AMS programs	Hospitals to develop formulary based on STG/NEMLIT	Hospital formulary and other AMR policies formulated	The Ministry provided a statement requesting hospitals to develop hospital formularies. All hospitals under the MTaPS project are progressing well with the activity. Also, at the national level, a Hospital Formulary template has been developed.
	Hospitals to develop/adopt AMR policies		All hospitals under the MTaPS project have adopted the AMR policy. AMS committees have been established with clear terms of reference. All hospitals have developed a costed AMS work plan. Further, capacity building on AMR and AMS was done in all hospitals under the project.
	Monitor the use of the hospital formulary and other AMR policies		Supportive supervision to monitor the formulation of the Hospital Formulary and implementation of AMS activities was conducted, and scoring was done using a monitoring and evaluation tool adopted from the WHO with additional indicators on the performance of STG/NEMLIT and the functionality of MTCs.
	Develop a system for the use of standard prescription		
	Design/review a standard prescription	Standard prescription available and used in health facilities	Done
	Print and distribute prescriptions to all practitioners		
	Audit antimicrobial prescribing		
	MTC/stewardship team to collect data on antimicrobial agent consumption	The use of antimicrobial agents in health facilities	The tool has been designed, and the activity will be implemented during the next phase.
	Audit if the data audited complies to stipulated standards	Audit if the data audited	To be implemented in the next phase of the project.
	Provide feedback to prescribers		To be implemented in the next phase of the project
	Prepare and share the report on health facility antimicrobial use and susceptibility test results with health care workers		
	Prepare a report on the trend of antimicrobial use in health facilities	Reports on AMR shared with facility health workers	Hospitals prepared the report and shared it during Extension for Community Healthcare Outcomes (ECHO) meetings.
	Prepare the report on AMR susceptibility test results		Hospitals prepare the report and share it during ECHO meeting.
	Share the report to core workers		The report is shared through ECHO platform meetings and hospital clinical meetings
	Submit a report to responsible authorities		??
	Educate prescribers, pharmacists, nurses, and lab personnel about good antimicrobial prescribing practices and AMR		
	Prepare training materials		Done

Strategic Intervention	Activities	Indicator	Comments
	Conduct training	Number of trainings on good antimicrobial prescribing practices and AMR conducted	Done to selected hospitals
Intervention 9.2 Monitor AMS programs	Establish and operationalize MTCs and other related committees		
	Facilitate meetings of MTCs		Done
	Conduct regular supervision to MTCs		Supportive supervision conducted
	Conduct joint inspection		
	Provide capacity building on AMS programs for MTCs and other related committees		
	Prepare training materials	Number of trainings on good antimicrobial prescribing practices and AMR conducted	Done
	Conduct training		The training was conducted for the hospital MTCs and established AMS committees.
	Conduct supportive supervision to monitor AMS programs		
	Develop a supervision checklist	Functional MTCs	The checklist developed
	Carry out regular supervision		Supervision was conducted using the developed checklist

ANNEX 6. PROCESSED REQUESTS FOR REGISTRATION OF MEDICINAL PRODUCTS IN 2020/21

Products	Remarks	By June 2020	Received	Total	Evaluated	Approved	Rejected	Remained
Veterinary Drugs (Dawa za Mifugo)	New	0	119	119	110	46	0	9
	Renewal	1	43	44	38	37	0	6
	Subtotal	1	162	163	148	83	0	15

Source: Extracted from the TMDA Annual Report 2020/21 entitled Taarifa ya Utendaji kazi kwa mwaka 2020/21. Available at: https://www.tmda.go.tz/publications/50

ANNEX 7. OTHER REQUESTS FOR PRODUCT REGISTRATION

Products	Remarks	By June 2019	Received	Total	Evaluated	Approved	Rejected	Remained
Veterinary Drugs (Dawa za Mifugo)	Changes	0	23	23	22	15	0	1
	Requests (Hoja)	2	85	87	77	NA	NA	10
	Subtotal	2	108	110	99	15	0	11

Source: Extracted from the TMDA Annual Report 2020/21 entitled Taarifa ya Utendaji kazi kwa mwaka 2020/21. Available at: https://www.tmda.go.tz/publications/50

ANNEX 8. REQUESTS FOR REGISTRATION OF PREMISES FOR MEDICINAL PRODUCTS

Type of premise		Requests		Total	Evaluated	Registered	Rejected
		New	Renewal				
Medicines (Dawa)	Local manufacturer (Viwanda vya ndani)	23	7	30	33	24	4
	Wholesale pharmacy (Veterinary)	10	49	59	60	60	0
	Storage (Maghala)	14	30	44	42	39	3
	Retail pharmacy (Veterinary)	2	0	2	2	2	0
	ADDOs for veterinary drugs (human & veterinary)	-	-	-	-	-	-
	Vehicles for transporting medicines (Magari ya kubebea dawa)	23	41	64	64	64	0
	Subtotal	72	127	199	201	189	7

Source: Extracted from the TMDA Annual Report 2020/21 entitled Taarifa ya Utendaji kazi kwa mwaka 2020/21. Available at: https://www.tmda.go.tz/publications/50

ANNEX 9. HISTORICAL DATA ON REGISTRATION OF VETERINARY PRACTICE FACILITIES

List of registered veterinary practice facilities as of April 2020	
**Category**	**Registered**
Veterinary hospital	1
Veterinary clinics	18
Veterinary centers	1,709
Veterinary service companies	12

Data source: Veterinary Council of Tanzania

ANNEX 10. INSPECTIONS CONDUCTED ON REGISTERED VETERINARY PREMISES

Type of business	Total	Complied	%
Wholesale pharmacy (veterinary)	427	359	84
ADDOs for veterinary drugs (veterinary)	751	601	80
Storage (Maghala)	2	2	100
Retail pharmacy (veterinary)	-	-	0
Vehicles for transporting medicines (Magari ya kubebea dawa)	3	2	67
Subtotal	1,183	964	81

Source: Extracted from the TMDA Annual Report 2020/2021 entitled: Taarifa ya Utendaji kazi kwa mwaka 2020/2021. Available at: https://www.tmda.go.tz/publications/50

ANNEX 11. SAMPLES COLLECTED AND ANALYZED UNDER POST-MARKETING SURVEILLANCE (2009–2013)

Year	Medicine Type	Number of Samples			
		Collected	Tested	Passed	Failed
2009/10	Cloxacillin	138	60	30 (50%)	30 (50%)
	Quinine	143	70	70 (100%)	0 (0%)
2010/11	Artemether + Lumefantrine	59	9	9 (100%)	0 (0%)
2011/12	Stavudine + Lamivudine + Nevirapine	58	22	20 (90.9%)	2 (9.1%)
2012/13	Zidovudine + Lamivudine + Nevirapine	25	12	12 (100%)	0 (0%)
	Total	423	173	141 (81%)	32 (18%)

Source: TFDA. 10 Years book (2003–2013)

ANNEX 12. ANALYSIS OF SAMPLES OF PRODUCTS BY THE TMDA FROM DIFFERENT SOURCES

Source of Product	Carried out (2018/19)	Received (2019/20)	Total	Investigated	Carried over (2020/21)	Passed (number)	Passed (%)
TMDA Lab, Dar es Salaam							
Inspection—human products	25	46	71	70	0	60	84.5
Submitted by clients	6	32	40	40	0	28	95
Preliminary investigation	28	1,466	1,494	1,128	366	1,126	99.8
PMS	0	30	30	30	0	29	96.7
PV	0	1	1	1	0	1	100
Subtotal/average (%)	59	1,577	1,636	1,270	366	1,254	98.4
TMDA Lab, Mwanza							
Inspection—human products	6	32	38	38	0	37	97.4
PMS	0	357	357	357	0	343	96.1
Subtotal/average (%)	6	389	395	395	0	380	96.2
Grand total/average (%)	65	1,966	2,031	1,665	366	1,635	98.2

Source: TMDA. Annual report 2019/20

ANNEX 13. POST-MARKET SURVEILLANCE OF VETERINARY PHARMACEUTICAL PRODUCTS (2016–2019)

Year	Type of drugs	Samples	Regions	Screened	Passed screening	Failed conf. test
2016/17	Oxytetracycline (inj. 10%)	58	5	58	58	0
	Oxytetracycline (inj. 20%)	72	5	71	71	7 (71)
2017/18	Ivermectin injection	48	4	48	48	0
	Trimethoprim/sulfamethoxazole	3	2	3	3	0
	Sulfadiazine/Trimethoprim	17	5	17	17	0
2018/19	Albendazole 10% suspension	50	6	50	50	1(5)

Source: PMS Reports 2016/17, 2017/18, 2018/19. Available at: https://www.tmda.go.tz/pages/post-marketing-surveillance-of-medicines

ANNEX 14. HISTORICAL TREND OF REGISTRATION OF VETERINARY SKILLED PERSONNEL

Registered Veterinary Skilled Personnel (2022)	
**Category**	**Registered**
Veterinarians	1,078
Veterinary paraprofessionals	2,299
Veterinary paraprofessional assistants	2,550
Meat inspectors	904
Laboratory technicians	70
Artificial insemination technicians	78

Data Source: Veterinary Council of Tanzania

ANNEX 15. TMDA ORGANOGRAM

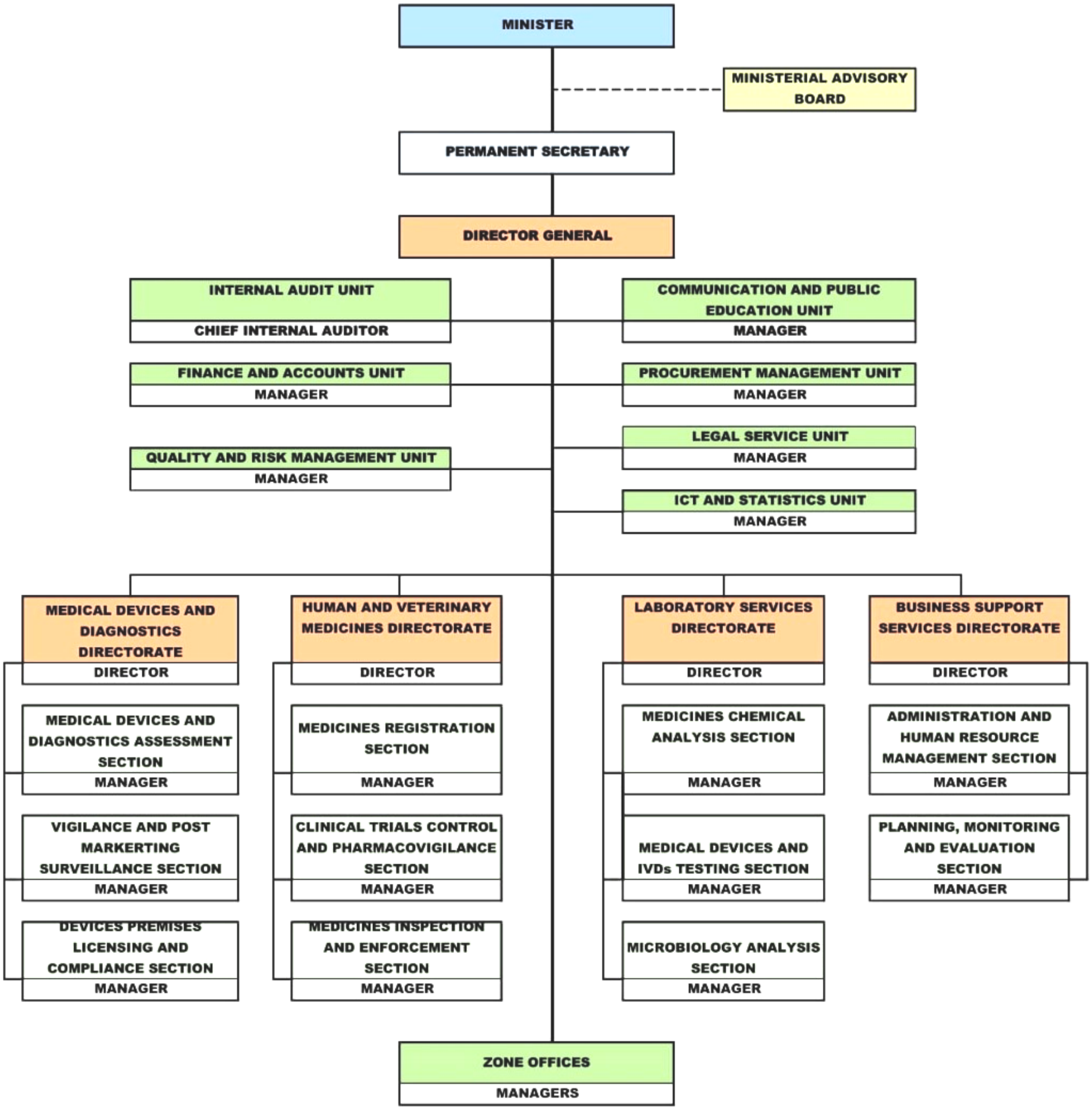

Supplement: Supplementary file 1 [file antibiotics-15-00770-s001.zip › S3 Country Reports/S3I_Tanzania (Human & Animal Health).pdf]
