# Supplementary material for: Landscape Analysis of Existing Policies, Laws and Regulations on Antimicrobial Stewardship in 10 Low- and Middle-Income Countries
Source: Antibiotics (Basel). 2026 Aug 10;15(8):770. doi: 10.3390/antibiotics15080770 (PMC13510074; doi:10.3390/antibiotics15080770)
Supplement: Supplementary file 1 [file antibiotics-15-00770-s001.zip › S3 Country Reports/S3J_Uganda (Human & Animal Health).pdf]

Uganda's Current Policies and Regulations on Antimicrobial Stewardship for Human Health, Animal Health, and Agriculture

October 2021

USAID FROM THE AMERICAN PEOPLE

USAID MEDICINES, TECHNOLOGIES, AND PHARMACEUTICAL SERVICES (MTaPS) PROGRAM

Improved Access. Improved Services. Better Health Outcomes.

The USAID Medicines, Technologies, and Pharmaceutical Services (MTaPS) Program is funded under USAID Contract No. 7200AA18C00074. MTaPS enables low- and middle-income countries to strengthen their pharmaceutical systems, which is pivotal to higher-performing health systems. USAID MTaPS' goal is to enable low- and middle-income countries to strengthen their pharmaceutical systems to ensure sustainable access to and appropriate use of essential medicines and related pharmaceutical services. The program brings expertise honed over decades of seminal pharmaceutical systems experience across more than 40 countries. The MTaPS approach builds sustainable gains in countries by including all actors in health care—government, civil society, the private sector, and academia.

MTaPS is implemented by a consortium of global and local partners and led by Management Sciences for Health (MSH), a global health nonprofit. To learn more, visit https://www.mtapsprogram.org/

DISCLAIMER:

The views expressed in this publication do not necessarily reflect the views of the U.S Agency for International Development or the U.S Government.

The MTaPS Consortium

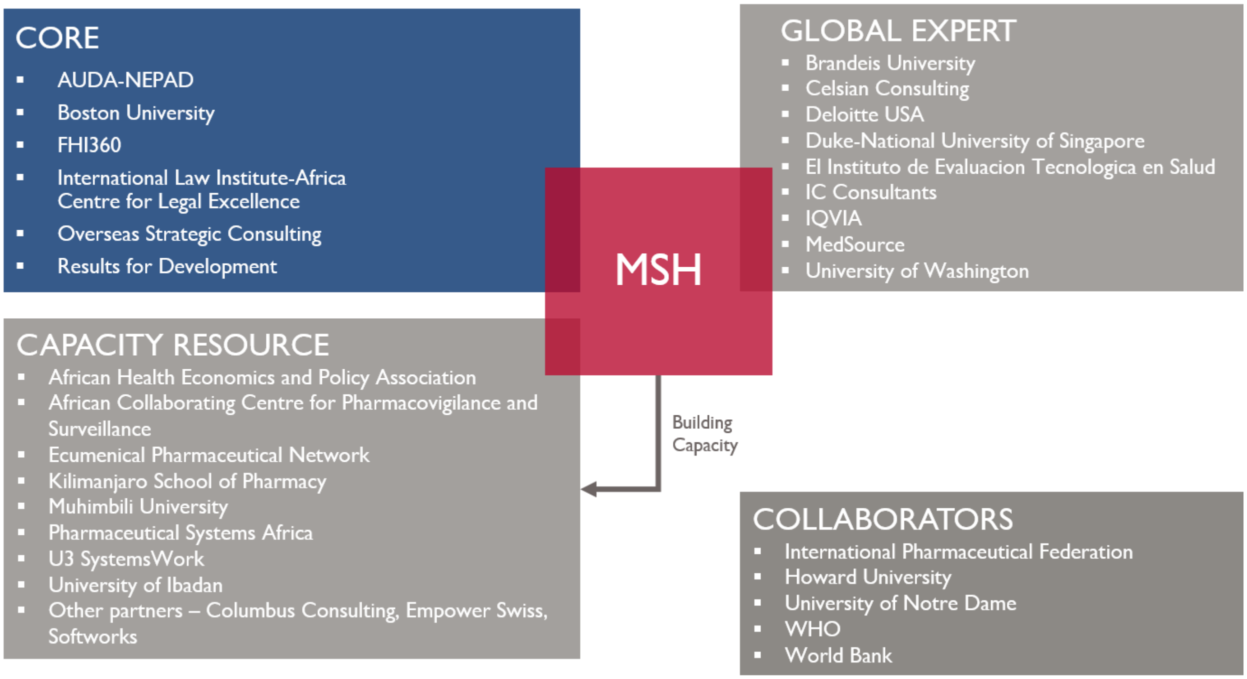

DISCLAIMER: The authors' views expressed in this publication do not necessarily reflect the views of the United States Agency for International Development (USAID) or the United States Government.

ACKNOWLEDGMENT

The assessment of Uganda's Current Policies and Regulations on Antimicrobial Stewardship for Human Health, Animal Health, and Agriculture was conducted in close collaboration with the Uganda Ministry of Health, Ministry of Agriculture Animal Industry and Fisheries (MAAIF), and the United States Agency for International Development (USAID) Medicines Technologies and Pharmaceutical Services Program implemented by a consortium led by Management Sciences for Health (MSH) and funded by USAID. Kate Kikule, Marion Murungi, JP Waswa, and Reuben Kiggundu were especially instrumental in providing technical input and guidance, before, and during the assessment.

Special thanks to the Government of Uganda leadership, especially at the MoH and MAAIF for allowing us access to personnel and relevant documents during the assessment. We thank Dr. Patrick Vudriko from Makerere University and Gordon Sematiko Katende who led the data collection and analysis efforts, and for their commitment and dedication during the assessment. Thank you to Dr. Henry Kajumbula for providing support on behalf of the National AMR Sub-committee. Thanks also to the National Drug Authority, National Medical Stores, Joint Medical Stores, Food and Agricultural Organization, and the USAID Strengthening Supply Chain Activity implemented by MSH for your technical input. Sincere thanks to all those individuals that provided technical expertise, contributed information, and participated in the document review and validation process. (For the complete list of contributors, see Annex 2 and 3).

ABOUT THE PROGRAM UNDER WHICH THE ASSESSMENT WAS ORGANIZED

Support for this assessment was provided through the USAID MTaPS program, Contract Number: 7200AA18C00074, which supports low- and middle-income countries to strengthen their pharmaceutical systems, with the aim of higher-performing health systems. USAID's mandate for MTaPS is to provide technical assistance to Uganda to strengthen systems and practices for IPC and the optimal use of antimicrobial medicines, including promoting and strengthening AMS and strengthening multisectoral coordination (MSC) to contain AMR. MTaPS' sub-objective 5.4 is dedicated to AMR containment. MTaPS has supported the GOU in implementing strategic activities in the NAP and responding to disease outbreaks. The challenge is now to implement the NAP nationwide, down to the facility level, and to raise the JEE-2 scores for AMR to meet the priorities of the GHSA and obtain a higher score on the WHO benchmarks for IHR capacities.

At the request of the Ministry of Health, Ministry of Agriculture Animal Industry and Fisheries, MTaPS committed to supporting a comprehensive assessment of Uganda's Current Policies and Regulations on Antimicrobial Stewardship for Human Health, Animal Health, and Agriculture with the goal of contributing to AMR control.

DISCLAIMER: The authors' views expressed in this publication do not necessarily reflect the views of the United States Agency for International Development (USAID) or the United States Government.

RECOMMENDED CITATION

USAID MTaPS (2021). Uganda's Current Policies and Regulations on Antimicrobial Stewardship for Human Health, Animal Health, and Agriculture. Submitted to the United States Agency for International Development by the USAID Medicines Technologies and Pharmaceutical Services Program (MTaPS), Under USAID Contract Number: 7200AA18C00074.

USAID Medicines, Technologies, and Pharmaceutical Services (MTaPS) Program
Management Sciences for Health
4301 North Fairfax Drive, Suite 400
Arlington, VA 22203 USA
Telephone: 703.524.6575
Fax: 703.524.789
Website: https://www.mtapsprogram.org

DISCLAIMER: The authors' views expressed in this publication do not necessarily reflect the views of the United States Agency for International Development (USAID) or the United States Government.

ACRONYMS AND ABBREVIATIONS

ACCA	Agricultural Chemical Control Act
ACCB	Agricultural Chemical Control Board
ACCTC	Agricultural Chemical Control Technical Committee
ADE	adverse drug event
ADR	adverse drug reaction
AHPC	Allied Health Professionals Council
AMR	antimicrobial resistance
AMS	antimicrobial stewardship
AMU	antimicrobial use
AU-IBAR	African Union Inter-African Bureau for Animal Resources
AWaRe	access, watch, reserve
BUBU	Buy Uganda Build Uganda
CED	committee on essential drugs
CNF	Committee on the national formulary
COVAB	College of Veterinary Medicine, Animal Resources and Biosecurity (Makerere University)
CPB	Crop Protection Board
CPD	continuing professional development
DAR	Directorate of Animal Resources
DCR	Directorate of Crop Resources
DFR	Directorate of Fisheries Resources
DPP	Director For Public Prosecution
DVO	district veterinary officer
EAC	East African Community
EAC-RPMOA	East African Community Regional Pharmaceutical Manufacturing Plan of Action
EDLU	Essential Drugs List for Uganda
EMHS	essential medicines and health supplies
EMHSLU	essential medicines and health supplies list for Uganda
EVML	essential veterinary medicines list
FAO	UN Food and Agriculture Organization
FPP	finished pharmaceutical product
GDP	Good Distribution Practices
GMP	Good Manufacturing Practices
HC2, HC3	health center at level 2 or 3
HCWM	health care waste management
JMS	Joint Medical Store
M&DPC	Medical and Dental Practitioners Council
MAAIF	Ministry of Agriculture, Animal Industry, and Fisheries
MAH	marketing authorization holder
MOFPED	Ministry of Finance, Planning, and Economic Development
MOH	Ministry of Health
MOH-PD	Ministry of Health-Pharmacy Department
MSH	Management Sciences for Health
MTaPS	Medicines, Technologies, and Pharmaceutical Services
MTC	medicines and therapeutics committee
NAP	National Action Plan

DISCLAIMER: The authors' views expressed in this publication do not necessarily reflect the views of the United States Agency for International Development (USAID) or the United States Government.

NCHE	National Council for Higher Education
NDA	National Drug Authority
NDPA	National Drug Policy and Authority
NMS	National Medical Stores
NVDP	National Veterinary Drug Policy
NVF	national veterinary formulary
OIE	World Organization for Animal Health
PD	Pharmacy Department
POM	prescription-only medicine
PPDA	Public Procurement and Disposal of Public Assets Authority
PSU	Pharmaceutical Society of Uganda
PV	pharmacovigilance
SI	statutory instrument
STG	standard treatment guideline
SVTG	standard veterinary treatment guide
TCM	traditional and complementary medicine
UCG	Uganda Clinical Guidelines
UGX	Uganda shilling
UNBS	Uganda Bureau of Standards
USD	US dollar
UVB	Uganda Veterinary Board
VEN	vital, essential, necessary
VMP	veterinary medicinal product
NRM	National Resistance Movement
FAO	Food and Agriculture Organization

DISCLAIMER: The authors' views expressed in this publication do not necessarily reflect the views of the United States Agency for International Development (USAID) or the United States Government.

CONTENTS

Acronyms and Abbreviations i

FOREWORD v

Executive summary vi

Introduction 1

Scope of work outline 3

Methodology 4

Findings 5
- National Policies 5
  - Uganda National Health Policy 5
  - Health Sector Development Plan III 2015/16–2019/20 5
- Marketing Authorization, Licensing, and Inspection 5
  - Human Health 5
  - Policies and Regulations for Medicines with Specific Focus on Antimicrobials 5
  - Animal Health 7
  - Schedules of VMPs Permitted in Pharmacies and Drug Shops 10
- Post-Marketing Surveillance and PV 10
  - Human Health 10
  - Animal Health 12
  - NDA Veterinary PV 12
- Supply Management Policies and Guidelines for Antimicrobials 12
  - Human Health 12
  - Animal Health 16
- Prescription, Dispensing, and Use 18
  - Human Health 18
  - Animal Health 21
- Antimicrobial Regulation and Use in Crop Protection 29
  - Policy, Laws, and Competent Authority to Regulate Agrochemicals 29
  - Supply Management Policies and Guidelines for Crop Antimicrobials 29
  - Post-Marketing Surveillance and PV for Agrochemicals 29
  - Prescribing, Dispensing, and Using Agrochemicals 30
- Antimicrobial Regulation and Use in Aquaculture 30
  - Policy, Laws, and Competent Authority to Regulate Antimicrobials Used in Fish 30
  - Marketing Authorization, Licensing, and Inspection of Fish Antimicrobials 30
  - Supply Management Policies and Guidelines for Fish Antimicrobials 31
  - Post-Marketing Surveillance and PV for Fish Antimicrobials 31
  - Prescribing, Dispensing, and Using Antimicrobials in Aquaculture 31

DISCLAIMER: The authors' views expressed in this publication do not necessarily reflect the views of the United States Agency for International Development (USAID) or the United States Government.

Proposed next steps and recommendations....32
Human....32
Animal, Fish, and Crop Sectors....32
Short Term....33
Medium to long term....33

Conclusion....34

AMS Policies, Regulations, and Guidelines analysis; Gaps identified; and Recommendations....36
Human Health Sector....36
Animal, fish, and crop sectors....44

Appendix i: Policies, Laws, Guidelines, and Circulars relevant to AMS in the Human health, Animal, fish, and Crop Sectors....52

APPENDIX ii: FIGURES & GRAPHS....54

ANNEX i: List of key respondents (human Health)....56

ANNEX ii: List of Key Informants from the Animal, Fisheries and Crop sector interviewed....58

DISCLAIMER: The authors' views expressed in this publication do not necessarily reflect the views of the United States Agency for International Development (USAID) or the United States Government.

FOREWORD

Uganda has made great strides toward implementing plans and strategies to contain antimicrobial resistance. Notably, through the One Health platform, ministries and implementing partners have made progress on implementing the National Action Plan on Antimicrobial Resistance, 2018. Additionally, Uganda participated in a Joint External Evaluation (JEE) for International Health Regulations (IHR) in 2017, in which it scored a capacity level 3 under the capacity requirements for antimicrobial stewardship (AMS).

Use and misuse of antimicrobials in humans, animals, crops, and fish production is the major modifiable driver of antimicrobial resistance. As such, AMS interventions at the policy, regulatory, and implementation levels are imperative to improving how well antimicrobials are used to prevent use, misuse, and overuse practices that contribute to the rapid development of resistant strains.

To tailor impactful AMS interventions, it is important to understand the enabling environment for both use and misuse in Uganda. The Medicines, Therapeutics, and Pharmaceutical Services Program (MTaPS), with funding from USAID, supported the Ministries of Health and Agriculture, Animal Industry, and Fisheries to assess stewardship policies and activities, including a regulatory framework and supply chain management of antimicrobials by using a multisectoral approach (a JEE capacity 2 benchmark activity).

This report details the findings of the assessment. The objective is to use these findings and recommendations to inform next steps on implementation of AMS activities in Uganda using a One Health approach.

Dr. Neville Oteba
Commissioner of Health Services
Pharmaceuticals and Natural Medicine
Ministry of Health

Dr. Ademun Rose Okurut
Commissioner, Department of Animal Health
Ministry of Agriculture, Animal Industries, and Fisheries

DISCLAIMER: The authors' views expressed in this publication do not necessarily reflect the views of the United States Agency for International Development (USAID) or the United States Government.

EXECUTIVE SUMMARY

Antimicrobial resistance (AMR) is one of the serious threats to humanity in the 21st century. If not properly addressed, it will cause the death of an estimated 10 million people every year soon. Inappropriate use of antimicrobial agents across human, animal, and agriculture sectors is among the main drivers of AMR globally. In December 2018, Uganda launched the One Health National Action Plan (NAP) on AMR, in line with the tripartite (WHO-FAO-OIE) AMR action plan. Antimicrobial stewardship (AMS) is one of the key pillars of the NAP. The USAID Medicines, Technologies, and Pharmaceutical Services (MTaPS) program, implemented by Management Sciences for Health, is supporting Uganda in assessing AMS policies and regulations in the human health, animal health, and agriculture sectors by using a multisectoral approach.

This review sought to identify the various legislation, policies, and regulations that impact AMS and identify salient gaps that need to be addressed in the human, animal, fish, and crop sectors.

A comprehensive desk review of documents on Uganda's current AMS policies and regulations on the supply and use of antimicrobials was conducted. Additional data were obtained through interviews with key stakeholders along the antimicrobial supply chain. The data sources identified the available policies, legislation, regulations, and guidelines in the following thematic areas: market authorization, licensing, and inspection; post-marketing surveillance and pharmacovigilance (PV); supply management policies and guidelines; and prescribing, dispensing, and use.

The major policies that govern AMS in the human and animal sectors include the National Medicines Policy for the human sector, the NVDP for the animal sector, and the National Drug Policy and Authority (NDPA) Act Cap 206 for both human and animal sectors. The National Drug Authority (NDA), created by an act of Parliament in 1993, has since grown to internationally recognized status in the quality of its services. To achieve this status, NDA developed 9 statutory regulations and over 22 guidelines from the primary law to guide drug registration, licensing, registration of personnel and premises, the conduct of clinical trials, control of publication and adverts of drugs, import and export of drugs, and PV in the human and animal sectors. No conventional antimicrobials used in human and animal health have been registered for use in crop protection and aquaculture under the Agriculture Chemical Control Act (ACCA) and the Fish (Aquaculture) Rules, respectively. However, animal antimicrobials are used in aquaculture establishments without extra-label prescription by qualified veterinarians, posing a great challenge to AMS in the sector. The laws with direct impact on antimicrobial consumption/use include laws governing professionals who handle antimicrobials in human and animal health, the Plant Protection and Health Act, ACCA, and the Fish Act.

Key AMS Gaps Identified and Recommendations

In the human health sector, Uganda has several laws, but none were identified to specifically address AMR. The Constitution provides a right for only basic medical care; other treatment must be paid out of pocket. Drug prices are not regulated; thus, people cannot afford them, leading to procuring partial doses of antibiotics, one of the factors leading to AMR. There is inadequate funding for the health sector

DISCLAIMER: The authors' views expressed in this publication do not necessarily reflect the views of the United States Agency for International Development (USAID) or the United States Government.

to procure medicines and the ruling party National Resistance Movement (NRM) manifesto does not include strategies for fighting AMR.

Several existing laws are very old and do not address the current challenges of AMR. There is sparse availability of quality prescribers in public health facilities, leading to drug misuse. There are conflicting mandates within laws; for example, national quantification for pharmaceutical products is shared by the Ministry of Health-Pharmacy Department (MOH-PD), NDA, and National Medical Store (NMS) under different acts. Irregular antimicrobial supply practices include directives from the Ministry of Finance, Planning, and Economic Development (MOFPED) to NMS, bending procurement conditions outside PPDA. Parliament passed the traditional and complementary medicine (TCM) bill, which incorporates TCM into the national health system. The East African Community (EAC) countries developed a joint action plan (EAC-RPMPOA) to address pharmaceutical development in the region, but Uganda has not domesticated most of the strategies in this plan. The current essential medicines and health supplies list for Uganda (EMHSLU) classifies antimicrobials only according to vital, essential, and necessary (VEN), not considering access, watch, and reserve (AWaRE) classification. There is no law to specifically regulate health care waste management (HCWM) in both human and animal sectors; even the National Environment Act does not make specific provisions for this.

NDA licensing guidelines dictate distance between pharmacies as a measure to promote equitable distribution of pharmaceutical services and permits one pharmacist to supervise two pharmacies; this encourages non-pharmacists to dispense restricted antimicrobials without the immediate supervision of a pharmacist. There is rampant use of unregistered drugs and unauthorized prescribers across the whole country. The government is upgrading health centers at level 2 (HC2s) to HC3s, introducing qualified prescribers at HC3s, but NDA drug schedules do not recognize them.[1] The guidelines for preventing and treating HIV/AIDS do not mention challenges and mitigating factors to avoid AMR, irrespective of using many antimicrobials in HIV/AIDS treatment. Different departments within MOH organize individual education campaigns on AMR issues, but these are not harmonized.

It is recommended that the constitutional requirement of providing basic health care be amended to comprehensive health care. The government should be lobbied to increase funding for the health sector, moving toward the Abuja Declaration commitment of 15% of the country's annual budget, plus expanding funding options to include health insurance. The AMR NAP task force should ensure that the AMR program is part of national political agenda for political and financial support. MOH should spearhead the amendment of outdated acts, regulations, and guidelines to meet current challenges in AMS. New acts, regulations, and guidelines should be developed to address the identified gaps in AMS, such as a drug pricing policy, price regulatory framework, and pharmaceutical waste management. The Ministry of Agriculture, Animal Industry, and Fisheries (MAAIF)/NDA should adopt mandatory traceability for critical products like fish, cattle, chicken, eggs, pork, and milk. Training institutions should emphasize AMR and AMS during training and post-graduate on-the-job training.

[1] MOH. Guidelines for Designation, Establishment and Upgrading of Health Units. http://library.health.go.ug/publications/health-infrastructure/guidelines-designation-establishment-and-upgrading-health-units

DISCLAIMER: The authors' views expressed in this publication do not necessarily reflect the views of the United States Agency for International Development (USAID) or the United States Government.

The National Chemotherapeutics Laboratory and NDA should be supported in identifying, recognizing, and integrating the process of TCM as alternative affordable and accessible antimicrobials. Research should be supported in AMS, for example, the effect of using hand sanitizers on the pressure to use antimicrobials and its impact on AMR. The EAC Regional Pharmaceutical Manufacturing Plan of Action[2] (EAC-RPMOA) should be aligned to the Ugandan context to implement Buy Uganda Build Uganda (BUBU). The EMHSLU must be updated to adopt the WHO AWaRe classification of antimicrobial drugs per level of care. A national medical countermeasures supply chain plan should be incorporated in the health system for better management of antimicrobial use (AMU) in emergencies and epidemics. Health care institutions and farms under MAAIF should be supported with infrastructure and capacity to handle antimicrobial waste, such as the construction of pits, dumping sites, and incinerators. The NDA licensing guidelines should be amended to provide for one pharmacist per pharmacy and bar any dispensing of restricted antimicrobials in his/her absence. The NDA policy of equitable distribution of pharmaceutical services should be amended to population served, rather than a policy based on absolute distance between pharmacies. Professional bodies should improve their regulatory supervision of their members/prescribers. NDA should amend drug schedules to recognize new prescribers.

In the animal and agriculture sectors, the key gaps in antimicrobial market authorization, registration, and inspection are:

- Lack of specific policies to guide AMS strategy
- Absence of a separate, competent department responsible for veterinary drug regulation, in line with OIE terrestrial code and African Union Inter-African Bureau for Animal Resources (AU-IBAR)-EAC recommendations
- Weak guidelines for antimicrobial waste disposal
- Merger of human and veterinary antimicrobial schedules in NDPA, which affects restrictions
- Conflicting jurisdictional mandates between MAAIF and the NDA over developing the essential veterinary medicines list (EVML) and formulary
- Fish drugs registered by NDA not listed in the NDA drug register
- Lack of specific guidelines for licensing antimicrobials used in fish and crop protection

Post-marketing surveillance for veterinary antimicrobials remains low, and unlicensed antimicrobials circulate at border districts due to weak enforcement. Overall, antimicrobial post-market quality assurance remains low. A weak PV system for animal antimicrobials due to low levels of veterinary staffing at NDA, intermittent community outreach and lax adverse drug event (ADE) reporting by veterinarians and farmers are widespread. Irregular antimicrobial supply practices that affect AMS include lack of guidelines for antimicrobial donations to farmers, poor antimicrobial dispensing practices, and lack of AMC data.

[2] 2nd EAC Regional Pharmaceutical Manufacturing Plan of Action 2017–2027; https://www.eahealth.org/policy-publications/2nd-eac-regional-pharmaceutical-manufacturing-plan-of-action-2017%E2%80%932027

DISCLAIMER: The authors' views expressed in this publication do not necessarily reflect the views of the United States Agency for International Development (USAID) or the United States Government.

To strengthen AMS in the animal, fish, and crop sectors, it is recommended that MAAIF:

- Develop AMS specific policies
- Incorporate AMS into the laws for regulating animal health workers and disease control in animals, fish, and crops
- Strengthen structures for regulating antimicrobials, in line with OIE and AU-IBAR-EAC recommendations
- Strengthen compliance enforcement for good pharmacy practices, Good Distribution Practices (GDP), and good dispensing practices
- Review and amend the current NDA antimicrobial drug schedule to include separate schedules for human and animal drugs
- Strengthen post-marketing surveillance and PV for antimicrobials across all agricultural sectors
- Incorporate AMS in curricula for training human resources in animal, fisheries, and crop sectors
- Invest in laboratory diagnostic infrastructure to enhance good clinical practices and promote rational antimicrobial prescription
- In conjunction with local government, align the AMR NAP to the local context in their routine or annual activity plans and allocate financial resources for implementation, monitoring, and evaluation.

DISCLAIMER: The authors' views expressed in this publication do not necessarily reflect the views of the United States Agency for International Development (USAID) or the United States Government.

INTRODUCTION

AMR is estimated to cause annual mortality of 700,000 people worldwide, and every country is potentially affected. If not properly addressed, the number could grow to 10 million per year globally by 2050.[3] "Inappropriate use of antimicrobial agents across human, animal, and environmental sectors is among the main drivers of antimicrobial resistance."[4] Therefore, the WHO 13th General Programme of Work (GPW 13, 20190) identified AMR as a priority with significant relevance to its Triple Billion targets of achieving universal health coverage, addressing health emergencies, and promoting healthier populations.[5] The WHO regional office for Africa released a report in 2013 indicating that AMR was already a threat to Africa with the appearance of drug resistance, including multidrug resistance to TB,[6] and recommended several strategies, one of which was NAPs.

The animal sector is equally affected by AMR. Various antimicrobials, some of which are used in human health, are also used to control bacterial diseases and as feed additives to promote the growth of livestock and are responsible for AMR.[7] In Uganda, the veterinary drug industry evolved from donor-based centralized supply and distribution in 1970s to being led by the private sector in 1993. However, even though privatization of the animal drug supply (including antimicrobials) has been commended for increasing access to drugs, it has negatively affected both quality and stewardship under a weak regulatory framework.

AMR is a complex multisectoral phenomenon that requires multisectoral approach. The international community, led by WHO, OIE, and FAO, have identified weak antimicrobial medicinal product regulation as one of the major drivers of AMR. Uganda is signatory to the May 2015 WHO resolution for a Global Action Plan for combating AMR (WHO 2015), and it adapted and launched an NAP on AMR in December 2018.[8] The NAP acknowledges the importance of AMS in preserving the effectiveness and efficacy of antimicrobial agents and lays it out as Strategic Objective 3 (Promote Optimal Access and Use of Antimicrobials). The WHO International Health Regulation benchmark 3.4 (Optimizing use of antimicrobial medicines in human and animal health) details a stepwise roadmap of actions for building a country's capacity; one of the actions expected of the signatories was developing their national AMS plan or strategy.

---

[3] UN Ad Hoc Interagency Coordination Group on Antimicrobial Resistance. 2019. No Time to Wait: Securing the future from drug-resistant infections; https://www.who.int/publications/i/item/no-time-to-wait-securing-the-future-from-drug-resistant-infections

[4] Holmes AH, Moore LS, Sandford A, et al. Understanding the mechanisms and drivers of antimicrobial resistance. Lancet. 2016 Jan 9;387(10014):176–87 (https://pubmed.ncbi.nlm.nih.gov/26603922/, accessed June 20, 2021).

[5] WHO. 13th general programme of work of WHO 2019–2023. Geneva: WHO; 2019 (https://apps.who.int/iris/bitstream/handle/10665/324775/WHO-PRP-18.1-eng.pdf, accessed June 20, 2021)

[6] WHO Regional Office for Africa. Antimicrobial resistance in the African region: Issues, actions, and challenges proposed. Geneva: WHO; 2013. https://www.afro.who.int/sites/default/files/2017-06/amr-paper-march-2013-jbn-and-all.pdf. Accessed January 3, 2022.

[7] Liu K, Han J, Li S, et al. Insight into the diversity of antibiotic resistance genes in the intestinal bacteria of shrimp Penaeus vannamei by culture-dependent and independent approaches. Ecotoxicol Environ Saf. 2019;172:451–9

[8] MOH. Antimicrobial Resistance National Action Plan 2018–2023. Kampala: MOH; 2018. https://cddep.org/wp-content/uploads/2018/12/GoU_AMR-NAP.pdf

DISCLAIMER: The authors' views expressed in this publication do not necessarily reflect the views of the United States Agency for International Development (USAID) or the United States Government.

To inform the stewardship plan, the USAID Medicines, Technologies, and Pharmaceutical Services (MTaPS) program, implemented by MSH, is supporting Uganda in assessing AMS policies and regulations in the human and animal health sectors in Uganda by using a multisectoral approach. This review sought to identify the various policies, legislation, and regulations that impact AMS and identify salient gaps that need to be addressed.

DISCLAIMER: The authors' views expressed in this publication do not necessarily reflect the views of the United States Agency for International Development (USAID) or the United States Government.

SCOPE OF WORK OUTLINE

The main purposes were to:

1) Undertake a comprehensive desk review of documents on Uganda's current stewardship policies on regulation, supply, and use of antimicrobials in human health, animal health and agriculture to develop a first draft of the assessment report. The documents' data sources were to detail available policies, legislation, regulations, and/or guidelines in the following areas:

   - Market authorization, licensing, and inspection
   - Post-marketing surveillance and PV
   - Supply management policies and guidelines relevant for antimicrobials
   - Prescribing, dispensing, and use, as relevant to antimicrobials

   Information gaps and areas that need further clarification that may be determined through subsequent in-person stakeholder interviews.

2) Conduct key informant interviews with selected stakeholders (that will be determined according to item 1 above) to gather detail and clarify the previously assembled evidence of the available policies, legislation, regulations, and/or guidelines relevant to this activity. This information was to be used to update the first draft and produce a second draft of the assessment report.
3) Collect additional documents and consolidating findings from items 1 and 2 to produce a final draft assessment report for review

As per the contract between MTaPS and the consultants (Gordon Sematiko Katende and Dr. Patrick Vudriko), the following were agreed to as deliverables:

- Report on findings of the assessment of policies and regulations on use of antibiotics in Uganda
- A summary of findings presented to the national AMR subcommittee

DISCLAIMER: The authors' views expressed in this publication do not necessarily reflect the views of the United States Agency for International Development (USAID) or the United States Government.

METHODOLOGY

MTaPS worked with government ministries and the regulatory authorities in both the human health and animal health sectors to assess stewardship policies, the regulatory framework, and supply chain management of antimicrobials by using a multisectoral approach.

The first phase of the exercise involved reviewing documents on Uganda's current stewardship policies on regulation, supply, and use of antimicrobials in human health and animal health by two experts. Acts of Parliament were downloaded from publicly available repositories. The NDA, MOH, and MAAIF websites were used to download the laws, statutory instruments (SIs), guidelines, circulars, published reports, and articles in peer-reviewed journals on AMR/AMU guidelines for drug regulation. When critical data on AMR/AMU could not be retrieved online, a request was made to the relevant government departments or agencies. The key thematic areas for the review of antimicrobial regulations and use focused on market authorization, licensing, and inspection; post-marketing surveillance and PV; supply management policies and guidelines for antimicrobials; and prescribing, dispensing, and use, of antimicrobials.

The first draft report was shared with key stakeholders to validate the information and identify information gaps. This was followed by phase 2 of interviews with purposeful, identified stakeholders to fill the gaps. The second draft report was then generated, reflecting the status with recommendations to address identified gaps. Finally, additional documents were reviewed, and findings consolidated to produce the final assessment report. The final report will inform the development of the NAP on AMS.

DISCLAIMER: The authors' views expressed in this publication do not necessarily reflect the views of the United States Agency for International Development (USAID) or the United States Government.

FINDINGS

NATIONAL POLICIES

Uganda National Health Policy

It is from the provisions of the 1995 Constitution that Uganda's Parliament passed the National Health Policy with a theme of Promoting People's Health to Enhance Socioeconomic Development. It highlighted that, on average, 28% of health facilities in Uganda had a constant supply of medicines and health supplies throughout the year, of which only 30% of the essential medicines and health supplies (EMHS) required for the basic package were provided for in the framework for medium expenditure, requiring adequate action. This indicates that even antimicrobials are under-supplied.

Health Sector Development Plan III 2015/16–2019/20

By 2015, the pharmaceutical sector had improved the availability of and access to EMHS from 43% in 2009/2010 to 63.8% in 2014/2015. During this period, both donors and government increased their funding for drugs from USD 92 million to USD 410 million (including ring-fenced USD 85 million for long-lasting insecticide nets). This improved public confidence in the health system.[9] Development partners financed the plan by over 80%, primarily directed to malaria, HIV/AIDS, and TB, leaving the other areas to government and out of pocket to users. It is worth noting that per capita government expenditure on EMHS of USD 2.4 million (FY 2013/14) stood far below the earlier projection of USD 12 million (Health Sector Development Plan III MOH September 2015).

MARKETING AUTHORIZATION, LICENSING, AND INSPECTION

Human Health

The **Food and Drugs Act 1959**[10] is an old law but is still in force. Some components of its mandate on drugs were taken over by the NDA act. It is implemented by two regulations: the Control of Quality (Iodized Salt) and the Food Fortification Regulations. The act provides for registration of food products and food premises, but this is not being done. Adulteration of food with antimicrobial agents, especially antibiotics and formalin (milk, fish) as preservatives, is rampant, thereby finding their way into humans. This misuse can lead to AMR and is dangerous to consumers.

Policies and Regulations for Medicines with Specific Focus on Antimicrobials

The **National Drug Policy** enacted in 1993 had policy and authority fused into a single act; it was updated to separate policy from authority and included new changes and strategies to improve access to medicines. The revised policy revealed that the quality of pharmaceutical products imported into the country had been improving considerably as evidenced by the percentage of products failing quality tests at NDA dropping from 11% in 2010/11 to 4% in 2013/14. Uganda's heavy dependence on donor funds

---

[9] MOH. Health Sector Development Plan 2015/16-2019/20

[10] Government of Uganda (GOU). Food and Drugs Act 1959 (Cap. 278). Kampala: GOU; 1959. https://www.ecolex.org/details/legislation/food-and-drugs-act-1959-cap-278-lex-faoc096144/

DISCLAIMER: The authors' views expressed in this publication do not necessarily reflect the views of the United States Agency for International Development (USAID) or the United States Government.

was highlighted and valued at USD 6 per capita per year for medicines and vaccines, which puts Uganda in a vulnerable position. The drug use indicator showed that only 15–20% of health workers could correctly diagnose and treat, which can lead to drug misuse. However, the policy does not address drug pricing, yet high out-of-pocket expenditures are recognized. Quality prescribers are very sparse in public health facilities. The policy created some ambiguity when it mandated that MOH-PD quantify national requirements for pharmaceutical products,[11] which is a statutory requirement for NDA (NDA Act 1993 Part III, art 10, sec 1, 2, and 3) and for NMS under the NMS Act 1993 Part II sec 5. The policy is silent about TCM and its practitioners who make a lot of unregulated claims about TCMs.

The National Pharmaceutical Sector Strategic Plan III 2015–2020[12] addresses issues such as the medicines supply chain, pharmaceutical financing, appropriate use, and accessibility with focus on good governance, quality of care, equity, and efficiency. The plan highlighted that over 75% of the disease burden can be prevented through health promotion and prevention.

From the **National Drug Policy and Authority Act**, NDA developed 9 regulations and more than 22 guidelines. Some provisions of the act created conflicting and sometimes duplicated roles with other ministries, departments, and agencies; for example, with NMS, they share statutory quantification of medicines; with MAAIF, they share regulation of drugs for animals, fish, and birds. With the Uganda Bureau of Standards (UNBS), they share regulation of products with food supplements and cosmetics. With the Ministry of Local Government, they share licensing of premises from which medicines are sold; and with the Allied Health Professionals Council (AHPC), they share regulating the practice of allied health professions. The NDA board is fully constituted, but it is a large board of 20 members, some of whom have conflicts of interest; for example, members of the Joint Medical Store (JMS) and NMS sit on the board. This indicates that regulation of veterinary drug outlets and sellers is not collaborative.

The **NDA Registration of Products** regulation covers registration of products, raw materials, finished products, parking materials, and surgical instruments. It requires that products first be registered in the country of origin. There is no separate provision for antimicrobial agents; all are treated the same stringent way.

Through the **TCM Bill**, MOH elevated the use of TCMs by proposing the position of principal pharmacist at MOH-PD. The officer's role would be to oversee TCM incorporation into public sector health services. Parliament passed the TCM bill,[13] which establishes a council to regulate, register, license, inspect, and discipline TCM practitioners. It prohibits one person from practicing both modern medicine and TCM. The bill does not state how spiking antibiotics in herbal concoctions will be fought.

[11] National Medicines Policy 2015, p. 21; https://www.health.go.ug/cause/national-medicines-policy-2015/
[12] MOH. National Pharmaceutical Sector Strategic Plan (NPSSP) III 2015–2020. Kampala: MOH; 2015. http://library.health.go.ug/publications/pharmaceuticals-and-drugs/national-pharmaceutical-sector-strategic-plan-iii-2015-2020
[13] GOU. Traditional and Complementary Medicine Act 2019. Kampala: GOH; 2019. https://ncri.go.ug/document/the-traditional-and-complementary-medicine-act-2019/

DISCLAIMER: The authors' views expressed in this publication do not necessarily reflect the views of the United States Agency for International Development (USAID) or the United States Government.

The **Guidelines on Submission of Documentation for Marketing Authorization of a Pharmaceutical Product for Human Use**[14] recommends the common technical document format for submissions, which involve assembling all the quality, safety, and efficacy information into a single document. Antimicrobial products are reviewed stringently. Herbal antimicrobials follow a different submission process.

Guidelines on Hand Sanitizers for use during the COVID-19 Pandemic[15] enables NDA to ensure quality, efficacy, and safety of sanitizers. NDA tests products, inspects facilities, grants market authorization, and updates the public. Sanitizers are mainly promoted for use against coronavirus, however, they play a vital role in preventing infections due to other microorganisms.

Animal Health

Since 1993, the mandate for market authorization, licensing, and inspection of veterinary drugs, including antimicrobials, was vested in NDA by NDPA Cap 206. NDA regulates both human and veterinary medicines, although it is under MOH. The administrative structure, board composition, and representation of animal sector specialists on NDA committees influences prioritization and effective regulation of antimicrobials and AMS, as explained below.

NDA Board (Part I, Section 3 NDPA Cap 206) and Statutory Committees

Representation of the animal sector on the NDA board is provided for by section 3 of NDPA Cap 206. The board consists of 19 members drawn from the health sector (11), animal sector (2), herbalist sector (1), Ministry of Trade (1), police (1), Ministry of Defense (1), and the public (2). The lack of a directorate responsible for veterinary medicines and an imbalance in board representation has been reported by AU-IBAR and EAC[16] to impact negatively on effective regulation of veterinary medicinal products (VMPs), including antimicrobials. NDPA Cap 206 section 6 mandates that the NDA board establish a committee on essential drugs (CED) and a committee on the national formulary (CNF). These two committees are key in determining which medicines, including antimicrobials, will be legally registered in Uganda. The membership of the two committees is listed in NDPA Cap 206. All 11 CED members are from the human health sector with no representation from the animal sector. Similarly, only one member from the Faculty of Veterinary Science is included on the CNF. Although NDA initiated development of a national veterinary formulary (NVF), the draft document has not been endorsed by MAAIF and other animal sector stakeholders because of a conflict over mandates and reports of inadequate consultation of MAAIF by NDA. Currently, the draft formulary is under review by MAAIF.

---

[14]NDA. Guidelines on Submission of Documentation for Marketing Authorization of a Pharmaceutical Product for Human Use. Kampala: NDA; 2018.
[15] Guidelines on hand sanitizers for use during the Covid-19 pandemic. April 2020
[16] AU-IBAR-EAC Report on critical review and assessment of the status of policies, laws and regulations and procedures for veterinary medicinal products and biologicals in EAC (Report: 7/7/2017)

DISCLAIMER: The authors' views expressed in this publication do not necessarily reflect the views of the United States Agency for International Development (USAID) or the United States Government.

Human Resource for Antimicrobial and Other VMP Regulations

NDA has five directorates, each of which plays a key role in regulating antimicrobials and other VMPs. Currently, NDA has 17 veterinarians distributed in the 5 directorates. Although there is a head of veterinary medicine in NDA, the position is non-statutory, therefore veterinary medicine is not represented in the top management of NDA. On average, each region has one NDA veterinary regulatory officer whose area of operation spans over 15 districts. The gross underemployment of veterinarians and other veterinary medicine practitioners and its potential impact on the appropriate use of antimicrobials has been reported by AU-IBAR and EAC.

Registration of Antimicrobials

The registration of veterinary medicines is in line with the National Veterinary Drug Policy (NVDP) 2002 and the National Policy on Delivery of Veterinary Services. The goal of the NVDP is to promote and ensure the availability and use of quality veterinary drugs, including antimicrobials, in a professional manner. NVDP 2002 recognizes NDA as the authority that regulates veterinary drugs, notwithstanding the loopholes it identified in NDPA Cap 206. Section 3.5.3 of NVDP 2002 states that "NDA shall establish an appropriate registration and inspection system for veterinary drugs." Accordingly, NDA developed SI number 24 in 2014 for regulation of veterinary drugs registration. SI 24 provides definitions, a procedure for applying for registration, details of VMPs, antimicrobials, vaccines, and biologicals. The relevant application forms for antimicrobial registration are readily accessible on the NDA website. By 2020, NDA registered 470 VMPs and one veterinary herbal product, accounting for only 10% of the total medicinal products (4,540) registered by the authority.[17] According to NDA, more than 86 tons of veterinary antimicrobials were legally imported for use in the animal sector in FY2009/2020. Among the antimicrobials imported, tetracycline accounted for almost half, and the rest included sulfonamides, aminoglycosides, penicillins, macrolides, fluoroquinolones, and polypeptides (appendix 2, figure A1). Most of these antimicrobials are classified by WHO as either critically or highly important for treatment of human diseases.[18] However, there are reports of unregistered drugs, including antimicrobials, on the Ugandan market.[19]

Licensing Personnel to Operate Veterinary Drug Shops and Pharmacies

Licensing of both premises and human resources aims to ensure protection of the quality of antimicrobials and technical services offered in the drug outlets to support AMS.

Section 3.11 of the NVDP highlights that correct handling and safe use of classified veterinary drugs, including antimicrobials, requires professionally competent, licensed persons. The Veterinary Surgeons Act Cap 277 mandates the Uganda Veterinary Board (UVB) as the body responsible for issuing practice

---

[17] Intergovernmental Authority on Development. Uganda NDA drug registration statistics; http://mrh.igad.int/production/index_ug

[18] WHO. Critically Important Antimicrobials for Human Medicine: Ranking of medically important antimicrobials for risk management of antimicrobial resistance due to non-human use. Geneva: WHO; 2018. https://apps.who.int/iris/bitstream/handle/10665/312266/9789241515528-eng.pdf

[19] Luseba D, Rwambo P. Review of the policy, regulatory and administrative framework for delivery of livestock health products and services in Eastern and Southern Africa. London: Global Alliance for Livestock Veterinary Medicines; 2015; galvmed.org.

DISCLAIMER: The authors' views expressed in this publication do not necessarily reflect the views of the United States Agency for International Development (USAID) or the United States Government.

licenses to qualified veterinary surgeons.[20] The NDPA Cap 206 (SI 35 section 3) prohibits anyone from importing, exporting, manufacturing, or supplying wholesale or retail drugs, including antimicrobials, without a license from NDA. The regulatory instrument for licensing (SI 35) and accompanying guidelines are available on NDA's website.

Veterinary Drug Retailers (Shops)

Until recently, UVB recommended to the NDA that duly registered veterinary surgeons and diploma holders in animal health and production (from accredited institutions) be eligible for licensure to operate class C drug shops. However, in 2018, holders of a bachelor's degree in veterinary medicine (BVM) were stopped by NDA from operating class C drug shops.[21] The BVM holders were advised to open veterinary clinics to improve on the rational use of classified drugs amid resistance from the veterinary fraternity. The proposed paradigm will only work if NDA strictly enforces withdrawal of restricted drugs, such as antimicrobials, from drug shops if clinics are to be viable.

Licensing of Drug Outlets and Manufacturing Facilities

NDPA Cap 206 SI 36 mandates that NDA license veterinary drug outlets and manufacturing facilities before they operate in Uganda. For licensure of a veterinary pharmacy, NDPA Cap 206 requires that a pharmacist is a co-director.[22] After applying for a license, NDA carries out the inspection and, if the application requirements are satisfied, it issues a certificate of suitability of premise. By May 2021, NDA licensed 1,234 veterinary drug outlets (1,141 drug shops and 93 wholesale and retail pharmacies) (appendix 2, figure A2), 2 veterinary drug manufacturing factories, and 1 veterinary drug repackaging factory. A total of 66% of the pharmacies licensed in Uganda are located in the central region and another 30% in the western and southwestern regions. West Nile and the northern region have over half of the ruminant population in the country but does not have a single veterinary pharmacy. Drug shops accounted for 92% of all drug outlets registered by NDA, with the majority clustered in the western and southwestern (48%) and central (19%) regions. The uneven distribution of veterinary drug outlets suggests inequality in access, contrary to the strategic focus of the NVDP 2002 that aims to increase access to essential VMPs and their equitable distribution in the country. In the absence of retail and wholesale pharmacies in the northern and West Nile regions, antimicrobials are stocked and dispensed by drug shops against NDPA Cap 206. What requires more interrogation is whether NDPA Cap 206, section 14 (the requirement that a pharmacist be a codirector) is among the major impediments to opening new pharmacies in areas such as the West Nile and northern Uganda and whether section 14 has an adverse impact on AMS.

---

[20] GOU. Veterinary Surgeons Act 1958 Cap 277 sec 6; https://www.ecolex.org/details/legislation/veterinary-surgeons-act-1958-cap-277-lex-faoc096738/

[21] NDA. Professional licensing Guideline 2018, section 3.4 issued on 20/12/2017

[22] National Drug Policy and Authority Act Cap 206, part II, section (14) on license of person to conduct business in restricted drugs; 14(c) in the case of a body corporate, that at least one of the directors is a pharmacist resident in Uganda; and 14 (d) in the case of a partnership, that at least one of the partners is a pharmacist resident in Uganda.

DISCLAIMER: The authors' views expressed in this publication do not necessarily reflect the views of the United States Agency for International Development (USAID) or the United States Government.

Schedules of VMPs Permitted in Pharmacies and Drug Shops

NDPA Cap 206 categorizes both human and veterinary drugs into eight schedules. The most important schedules relevant to AMU, AMS, and AMR include the following: the second schedule, which contains class B drugs (or controlled) drugs that are further divided into two. Group 1 contains the most critical and highly important antimicrobials based on OIE classification.[23] According to NDPA Cap 206, group 1 drugs may be supplied by retail pharmacies only on prescription of a duly qualified veterinary surgeon. The schedule does not separate antimicrobials for use in humans from those intended for use in animals, thus failing to categorize antimicrobials based on OIE criteria. Group 2 contains important veterinary antimicrobials, such as sulfonamides specifically formulated or in feed approved for the prevention and treatment of diseases in poultry. The schedule is not explicit on multicombination antimicrobial formulations sold on the market. Such multiple combinations are prone to over-prescription because of broad spectrum of activity, thus negatively impacting AMU and AMS.

The third schedule contains class C licensed drugs, which may be sold retail by licensed pharmacies or drug shops. With respect to AMU and AMS, group 1 of the schedule includes antibiotics contained in preparations or concentrates for feeding animals. It also specifically lists sulfadimidine contained in preparations formulated and labeled for treatment of poultry diseases, containing 16% w/v sulfonamide. To prevent misuse of antimicrobials in food animals, MAAIF has currently imposed a ban on importation of concentrates containing antimicrobials, and a law for regulating animal feeds has been drafted and its principles approved by the Cabinet.[24] The seventh schedule requires that antimicrobials in class A, class B (group 1), or class C (group 2) must be kept in a place that cannot be accessed by the public. However, it is common to encounter drug shops stocking and displaying antimicrobials, including injectable formulations.[25] Such practice is common where NDA supervisory oversight is limited. This predisposes antimicrobials to irrational dispensing and use.

POST-MARKETING SURVEILLANCE AND PV

Both post-marketing surveillance and PV are regulatory functions and part of the mandate of NDA. NVDP 2002 also recognizes the importance of monitoring the quality of veterinary drugs in Uganda.

Human Health

NDPA Cap 206 part VII gives powers to NDA inspectors to access premises (licensed or not) and vehicles used to carry drugs. NDA inspectors pick samples from the field and submit to the Directorate of Laboratory Services for quality analysis. NDA developed the Uganda trainer and trainee handbooks for training health workers on PV. However, NDA does not effectively regulate the medical technologies (technovigilance) that can impact rational drug use.

[23] OIE List of Antimicrobials of Veterinary Importance (adopted May 2007, Resolution XXVIII); https://www.oie.int/fileadmin/Home/eng/Internationa_Standard_Setting/docs/pdf/OIE_list_antimicrobials.pdf
[24] Kungu AA. Government Introduces Legislation to Curb Substandard Animal Feeds. January 2019. https://www.softpower.ug/govt-introduces-legislation-to-curb-substandard-animal-feeds/
[25] OIE, 2018. OIE PVS Follow-up Report for Uganda. P107

DISCLAIMER: The authors' views expressed in this publication do not necessarily reflect the views of the United States Agency for International Development (USAID) or the United States Government.

Guidelines for Reporting Adverse Drug Reactions

These PV regulations do not apply to the safety reports for new investigational drugs. Drugs, vaccines, surgical instruments, test kits, and diagnostic devices are covered. NDA provides an online adverse drug reaction (ADR) reporting platform (table 1), including a toll-free phone line, WhatsApp, and a VigiFlow-web-based database.

Table 1. Adverse drug reactions reports received by NDA[26]

**FY**	2019/2020	2018/19
**ADR reports**	1,824	538
**Drug-related complaints**	26	40
**Recalls**		22

Regulation on Conducting Clinical Trials[27]

Clinical trials, including for antimicrobials, can only be conducted with authority from NDA, which ensures prior clearance from the Uganda National Council for Science and Technology. Sponsors of a clinical trial are limited to product owner, patent owner, manufacturer, or their agent. Importation of clinical trial drugs must be granted clearance separately. Table 2 tracks clinical trial application processing from 2016 to 2020.

Table 2. Clinical trial applications received evaluated and approved by NDA[26]

**Financial year**	Received	Evaluated	Evaluated with in service delivery time
**2016/17**	26	26	18
**2017/18**	154	150	103
**2018/19**	151	161	131
**2019/20**	164	151	137

Guidelines on Submitting Periodic Drug Safety Update Reports[28]

NDA requires marketing authorization holders (MAHs) to submit ongoing safety data to enable continual decisions on risk-benefit of registered products. MAHs should have in place local structures with qualified personnel and processes for collecting, assembling, and reporting ADRs and other safety and use data to NDA.

[26] NDA. Report to the Nation 2020, page 36
[27] NDPA. ClinRegs/Uganda. 2022. https://clinregs.niaid.nih.gov/country/uganda#_top
[28] https://www.nda.or.ug

DISCLAIMER: The authors' views expressed in this publication do not necessarily reflect the views of the United States Agency for International Development (USAID) or the United States Government.

Animal Health

NDPA Cap 206 mandates that NDA carry out routine post-marketing surveillance for veterinary drugs to ensure that only authorized, quality, and safe antimicrobials and VMPs circulate on the market. In FY 2019/20, the NDA laboratory analyzed 2,100 human and animal medicine and medical device samples (appendix 2, figure A3). Only 11% of the samples analyzed were animal medicines compared to 89% for human health care products. Among the veterinary medicinal products analyzed, only 26 (11%) were veterinary drugs and vaccines combined; the rest were acaricides (89%). Of major concern is that the few (26) veterinary medicines and vaccine samples analyzed in FY 2019/20 had a very high failure rate of 46.2% (12/26). The available data on the NDA website does not show what proportion of the failed veterinary drugs were antimicrobials.

NDA Veterinary PV

NDPA Cap 206 SI 37 mandates that NDA carry out routine PV on veterinary antimicrobials and other animal drugs and make the information available to the public. It is a legal requirement for veterinary practitioners to report any ADRs and suspicions of fake products to the regulator. NDA requires that manufacturers of veterinary antimicrobials carry out PV in collaboration with their local technical representatives on their registered products and make such records available to the regulator. NDA also creates awareness on PV among veterinarians and farmers through community outreach. In FY 2019/20, NDA sensitized about 760 participants across the country.[29] The key concerns reported by NDA in its veterinary medicines PV bulletin of 2020 that require regulatory action include cross-over use of ARVs in animals; aliquoting of veterinary drugs, including antimicrobials; class B drugs (antimicrobials) stocked in drug shops; importers directly supplying farmers with drugs, including antimicrobials; unqualified personnel working in drug outlets; stocking expired drugs; and counterfeiting. However, in the absence of formal collaboration between NDA and district veterinary officers (DVOs), it is difficult to effectively enforce veterinary PV at the district level.

SUPPLY MANAGEMENT POLICIES AND GUIDELINES FOR ANTIMICROBIALS

NDA is mandated to regulate import, export, production, distribution, storage, and use of finished human and animal antimicrobial products and their corresponding active pharmaceutical ingredients. To effectively implement its role, NDA developed the Importation and Exportation of Drugs Regulations.[30]

Human Health

Regional and international collaboration requires that the agency have acceptable international standards. The WHO Regulatory Systems Strengthening Division assessed NDA and rated the agency at maturity level 4, which is the highest level of performance according to the Global Benchmarking Tool

[29] NDA. Veterinary Medicines Updates Bulletin Issue 5, June 2020. https://www.nda.or.ug/directorate-of-product-safety/#DPSBulletins
[30] NDPA. National Drug Policy and Authority (Importation and Exportation of Drugs) Regulations, 2013. https://extranet.who.int/sph/sites/default/files/document-library/document/final%20narrative%2011_09_19.pdf

DISCLAIMER: The authors' views expressed in this publication do not necessarily reflect the views of the United States Agency for International Development (USAID) or the United States Government.

Revision VI 2018. It was audited by SGS United Kingdom Ltd., found to be compliant, and certified to ISO 9001:2015for Quality Management System.

NAP for Health Security 2019–2023[31] helps Uganda champion the fight against AMR through surveillance and research in both the human and animal sectors by building capacities for an integrated national laboratory system for quick detection of priority infectious agents. A memorandum of understanding was signed between government ministries and agencies to form the national One Health platform.

East African Community Regional Pharmaceutical Manufacturing Plan of Action[2] sets four high-level targets:

1. Decrease dependency on pharmaceutical imports from outside EAC from more than 70% to less than 50%
2. Expand product portfolios of EAC firms to cater to more than 90% of disease conditions
3. Expand the percentage of EAC national medicine procurement agencies' purchases sourced from EAC pharmaceutical manufacturers to at least 50%
4. Within ten years, have at least five companies producing advanced pharmaceutical formulations that meet international standards by domesticating public health-related World Trade Organization's Trade-Related Aspects of Intellectual Property Rights flexibilities within the national laws of partner states and putting in place incentives to promote R&D in the pharmaceutical industry. (However, little has been done to achieve this target.)

NDA Importation and Exportation of Drugs Regulations authorizes only licensed persons to import any drug, including antimicrobials. More attention is given to narcotic drugs and psychotropic substances but NOT antimicrobial agents. Therefore, importers of narcotic drugs or psychotropic substances are required to make returns to NDA, but it is not a requirement for antimicrobial agents. For donated drugs, guidelines are not clear on how recipients of donations should treat any surplus antimicrobials received, which have the potential of being misused. Table 3 summarizes drug import clearance by the NDA from 2018 to 2020.

Table 3. Drug imports received and cleared by NDA

**FY**	2018/2019	2019/2020
**Received**	8,787	8,696
**% Cleared within 2 days***	97.71	95.3

*The remaining 2.29% and 4.7%, respectively, were either queried or rejected, indicating a high percentage of quality products coming into the country.

[31] National Action Plan for Health Security (NAPHS) 2019–2023

DISCLAIMER: The authors' views expressed in this publication do not necessarily reflect the views of the United States Agency for International Development (USAID) or the United States Government.

NDA Guidelines for Introducing a Locally Manufactured New Pharmaceutical Product on the Uganda Market,[32] SI No. 35; Licensing Regulations, 2014: Section 19(2) ensures, in terms of quality, that imported and locally manufactured antimicrobials are treated the same, following NDA Good Manufacturing Practices (GMP) guidelines.

Regulation on Control of Publication and Advertisement Relating to Drugs[33] applies to human, veterinary, and herbal of both. These apply to all activities and materials for the publication or advertisement of drugs including promotions, promotion materials and packaging materials including their quality and accuracy. The guidelines categorize advertisements[33] and publication materials as: category A that targets prescribers; category B that targets medical or veterinary establishments; and category C that targets over-the-counter drugs for the public.

The **National Independent Drug Information Service** is part of MOH. It collects information on all parameters of ministry interest, including drug components in the Supervision, Performance Assessment, and Recognition Strategy (SPARS), where data on AMR under pillar 3 (dispensing quality information) can be found.

Percentage of Medicines Manufactured Locally is a deliberate policy implemented by NDA to support local industries by imposing a 12% tax on 37 selected pharmaceuticals being imported, even though equivalent drugs are being locally produced, in line with BUBU. This helped develop local industries from only 10 in 2010 to 30 in 2020, with 10 more under construction and opening soon to raise the cumulative total to 40 facilities.[34]

The **Essential Drugs List for Uganda (EDLU)** was first developed in 2003 and is reviewed every five years. The current version includes health supplies and is thus called the EMHSLU.[35] All items are categorized by level of care according to VEN classification. The current EMHSLU incorporated recommendations from all stakeholders at all levels of health care. It includes care for all common conditions in the Uganda Clinical Guidelines (UCG). The laboratory supplies were developed by the Uganda National Health Laboratory Services team. The current UCH was due for review in 2020.

Health Care Providers Trained on the Concept of EMLS and Appropriate Medicine Use allows MOH-PD to follow up on the training of health care providers on the concept of EML through continuing medical education by hospital medicines and therapeutics committees (MTCs) and by using SPARS tools. Professional councils do the follow-up to ensure the course unit on essential medicines management is taught by training institutions under their jurisdiction.

---

32 NDA. Guidelines for Introducing a Locally Manufactured New Pharmaceutical Product on Uganda Market; 2018; https://www.nda.or.ug/nda/files/downloads/Guidelines_Introducing%20new%20products%20on%20market_INS_GDL_004_R1.pdf

33 Regulations on Control of Publication and Advertisement Relating to Drugs, July 2017; www.nda.org.ug

34 NDA, Report to the Nation 2020

35 MOH. Essential Medicines and Health Supplies List for Uganda 2016. https://www.health.go.ug/cause/essential-medicines-and-health-supplies-list-2016/

DISCLAIMER: The authors' views expressed in this publication do not necessarily reflect the views of the United States Agency for International Development (USAID) or the United States Government.

Ensuring the Quality of Antimicrobials

Public Sector

The NMS Act created the NMS in 1993 and mandates that it procure, store, and distribute to all public health facilities and advise NDA, MOH, MOFPED, and Ministry of Local Government on issues of medicine estimates, distribution, and use. The PPDA regulations and NMS acts guide NMS' procurement procedures, but they were recently deviated from because of MOFPED's circulars directing NMS to use local currency for public procurement/contracting (which precluded international market participation in tendering processes), resulting in lengthening supply chains, increasing the complexity of the supply chain, and risking introduction of substandard and falsified medicines into the lengthened supply chain. NMS administration calls for action to address the weak legal framework and current enforcement mechanism meant to deter providers of falsified medicines, wherein prosecutions (working with stakeholders, such as the Uganda Police Force and the Director for Public Prosecution [DPP]) are pursued through litigation in the court/judicial system characterized by delays and low success rates. Although NMS relies on NDA for product quality checks, it runs its own internal quality control laboratory for quick spot quality checks on suspected products.

Private Sector

Private sector operators of health facilities do not release prescriptions, as private pharmacies initiate the entire range of providing drugs to patients. This illegality needs to stop; stringent enforcement of adherence to the NDA act must be done. Professional associations should be empowered to enforce ethics and discipline among their membership.

JMS is the most prominent private pharmaceutical warehouse in Uganda, serving a clientele of more than 3,000 local health facilities and several others from neighboring countries. It was established as a joint venture between the Uganda Catholic Medical Bureau and Uganda Protestant Medical Bureau. Its website[36] reveals that it is licensed by NDA to import, export, and wholesale medicines and related health care supplies; repair, service, and maintain medical equipment; and build capacity. It has ultramodern warehousing facilities, conforming to Good Storage Practices, ISO 9001:2015. It operates a minilab for quality assurance in addition to writing field reports on the historical performance of suppliers. JMS works with Makerere University to develop comparative studies on product quality.[37] JMS procures products from the EDLU only when using funds from the government; funds secured elsewhere are used for other products outside the EDLU. JMS prequalifies suppliers who are requested to prove accreditation from stringent regulatory authorities and accreditation with other quality management systems. The institution keeps supplier records, evaluates annually, and organizes a conference for feedback once every two years. JMS does not treat antimicrobials differently.

[36] www.jms.co.ug
[37] Emmanuel Higenyi, JMS, Director Technical Services, personal communication.

DISCLAIMER: The authors' views expressed in this publication do not necessarily reflect the views of the United States Agency for International Development (USAID) or the United States Government.

Public Procurement and Disposal of Public Assets (Procurement of Medicines and Medical Supplies) Regulations[38] is a special regulation that was passed by the Government of Uganda outside the general PPDA procurement of other goods and services to reduce delivery times, select suppliers, etc. Use of past and user information, such as PV, is taken into consideration, although use of generic names is encouraged while brands with special consideration will be allowed, thus protecting patients. This works well for AMR considerations during procurement.

The draft **National Medical Countermeasures Supply Chain Plan** was tested in the wake of the COVID-19 emergency response by Wasswa.[39] This tested tool provided good guidance for proper management of antimicrobial agents under emergencies to avoid misuse and ease access. The government is encouraged to expeditiously ratify it for official use.

The **Uganda Cancer Institute**[40] **and Uganda Heart Institute**[41] are the only self-regulating bodies purposely given statutory mandate to procure specialized EMHS[40] to ensure high-quality services.

NDA Guidelines for Variation of Registered Medicinal Products[42] stipulate that a registered finished pharmaceutical product (FPP) MAH is responsible for the registered FPP throughout its life cycle, irrespective of the regular reviews by NDA. The MAH may also wish to alter or improve the FPP all subject to NDA approval. The guideline review date was March 12, 2021.

NDA Guidelines for the Recall or Withdrawal of a Medicinal Product[43] covers medicines, medical devices, and in vitro devices, which, for reasons related to their safety, quality, and efficacy, are to be withdrawn from the market.

Uganda does not have a comprehensive policy/law on managing expired, spoiled, and used containers of antimicrobial agents. MOH did have a National Health Care Waste Management Plan 2009/10–2011/12,[44] but it was not implemented; as a result, HCWM in Uganda continues to be below minimum acceptable safety standards.

Animal Health

The veterinary supply chain for antimicrobials and other VMPs has multiple facets. The FAO legal paper on legislation for veterinary drugs control[45] states that the law must ensure that drugs are of good quality, safe, and efficacious. A careful balance must be made between legal controls and ensuring access to essential antimicrobials to promote animal health, welfare, and production.

---

38 Public Procurement and Disposal of Public Assets (Procurement of Medicines and Medical Supplies) Regulations. Uganda Gazette #9, Vol CVII, February 14, 2014
39 International Journal of Science and Research (IJSR) ISSN: 2319-7064 / SJIF (2019): 7.583.
40 Uganda Cancer Institute Act, 2016
41 Uganda Heart Institute Act 2016
42 NDA. Guidelines for Variation of Registered Medicinal Products. 2018.
43 NDA. Guidelines for the Recall or Withdrawal of a Medicinal Product – 2017
44 National Health Care Waste Management Plan 2009/10- 2011/12
45 FAO. Legislation for veterinary drugs control. 2004. http://www.fao.org/3/bb071e/BB071E.pdf

DISCLAIMER: The authors' views expressed in this publication do not necessarily reflect the views of the United States Agency for International Development (USAID) or the United States Government.

Manufacturing and Importation of Veterinary Antimicrobials

Most veterinary antimicrobials sold in Uganda are imported as the country has only one veterinary manufacturing plant licensed to produce antimicrobials. NDPA Cap 206 (SI 29 section 13) requires that anyone intending to register antimicrobials and other VMPs manufactured outside Uganda provide evidence that the product is registered in the country of origin. NDPA Cap 206 (SI 34) regulates import of antimicrobials. Under this regulation, no one is allowed to import VMPs unless provided with an import license by NDA. Prior to issuance of an import license, NDA is legally required to carry out GMP to ascertain whether the facility that manufactured the antimicrobial complied with internationally accepted GMP guidelines used by NDA. Importers of veterinary antimicrobials are required to apply for and obtain a certificate of verification from NDA for each drug before importation.

The details of product description and packaging requirements are provided in NDPA Cap 206 SI 34 (www.nda.or.ug). A drug must meet strict labelling requirements; upon arrival, all requirements must be verified at the port of entry into Uganda for regulatory compliance. Similarly, all drug donations are supposed to be approved by NDA after fulfilling application requirements.[46] The export of antimicrobials and other VMPs out of Uganda is granted by NDA upon fulfilling its regulatory requirements[47] (annex figure A1 in appendix 2) for quantities of antimicrobials cleared for import by NDA.

Distribution of Veterinary Antimicrobials

Distributors are the link between manufacturers or importers and retailers to the community. NDPA Cap 206 regulation 16 mandates that NDA control distribution of drugs. Distributors (wholesalers), brokers, suppliers, logistics providers, traders, transport companies, and forwarding agents and their employees are required to comply with the NDA guideline on GDP for pharmaceutical products. NDA is mandated to monitor/inspect drug outlets licensed to distribute veterinary drugs for compliance with GDP on various aspects of the distribution chain. Irrespective of the existence of the above legal provisions, stakeholders have raised many concerns that affect AMS. The irregular inspection of distributors by NDA (on average, four times a year) leads to violation of GDP guidelines; some distributors supply antimicrobials directly to farmers (end users) against the GDPs. This leads to unnecessary stocking of large quantities of antimicrobials at farm level, resulting in their misuse. Products nearing their shelf-life are dumped to retailers by distributors at half price to avoid losses due to expiry and incurring costs for destruction. The unsuspecting end user is likely to get products that are of poor quality due to the above practices.

Sale of Veterinary Antimicrobials

At the point of sale, veterinary antimicrobials and other VMPs are expected to retain their quality attributes to ensure that their efficacy and safety are preserved. The choice of who sells which type of antimicrobials and other VMPs is provided for in the NDPA Cap 206 drug schedules. Antimicrobials are listed in the second schedule and categorized as class B, or controlled drugs. They are only permitted in

[46] NDA. Procedure for importing drug donations in Uganda. 24/10/2018
[47] National Drug Policy and Authority (Importation and Exportation of Drugs) Regulations, SI #34, 2014; https://www.fao.org/faolex/results/details/en/c/LEX-FAOC171264/

DISCLAIMER: The authors' views expressed in this publication do not necessarily reflect the views of the United States Agency for International Development (USAID) or the United States Government.

pharmacies, supplied by retail only on prescription by a duly qualified veterinary surgeon for animal treatment. However, antibiotics contained in concentrates for animal feeds fall under class C and may be sold by pharmacy and drug shops to farmers against the current position of the OIE ban on using antimicrobials in feeds by member states. Whereas the law adequately provides controls for the sale of antimicrobials, the major challenge accrues from enforcement and uniqueness of veterinary practice. The animal sector is categorized based on production systems and agro-ecological zones. For example, the pastoral livestock production region in Karamoja is underdeveloped, with less than 25 veterinarians employed in the public sector. As such, over 90% of drugs, including antimicrobials, are in the hands of farmers and community animal health workers who may not be able to read instructions on drug bottles. The region has no single pharmacy, and the few veterinary drug shops are either unregistered or struggle to get qualified personnel to register as a supervisor of the shop. This grossly affects AMU and AMS with the likely consequence of accelerating emergence of AMR.

PRESCRIPTION, DISPENSING, AND USE

Human Health

The objective of the **National Medicines Policy** was to ensure that end users receive maximum therapeutic benefits. Among its strategies that cover the entire medicine supply chain is enforcing good dispensing and practice in public and private sectors.[48] The policy does not address medicine pricing and financing AMR activities and is silent on animal medicines.

NDA registration of pharmaceutical premises/outlets is routinely updated; the current version's scope covers renewal and new licenses for class C drug shops, pharmacies, and manufacturing facilities.

Registration of personnel handling pharmaceutical products: All professional bodies annually maintain a register of their members recognized as prescribers and authorized to prescribe and/or dispense classified medicines, which includes many antimicrobial agents. However, reports of unauthorized, unqualified[49] dispensers, and self-medication are rampant.[50]

NDA regulation limiting antimicrobials to prescription only: The National Medicines Policy limits antibiotics as prescription-only medicines (POMs). Exceptions to POMs are made because of limited availability of relevant professionals at health facilities. Government delegates some prescribing powers to lower cadres; this ungazetted practice is done on behalf of the director general of Health Services, which makes it the norm in public health facilities. Projects like reproductive health recommend use of antibiotics by the lower cadres (tetracycline ointment). Community drug distributors are empowered to handle antimicrobial agents but not to prescribe. Unfortunately, the exception has led to unauthorized prescribing. It has been reported that senior clinicians rarely follow the set

[48] National Medicines Policy 2015, p. 25; https://www.health.go.ug/cause/national-medicines-policy-2015/
[49] Bbosa GS, Wong G, Kyegombe DB, Ogwal-Okeng J. Effects of intervention measures on irrational antibiotics/antibacterial drug use in developing countries: A systematic review. Health 2014 6(2): 171-87; https://www.scirp.org/journal/paperinformation.aspx?paperid=42481
[50] Twinomujuni E. Factors Associated with Irrational Use of Antibiotics among Community Members in Kishabya Parish, Shuuku Sub-County in Sheema District [dissertation]. Kampala: International Health Sciences University; 2016.

DISCLAIMER: The authors' views expressed in this publication do not necessarily reflect the views of the United States Agency for International Development (USAID) or the United States Government.

limitations, especially regarding reserving certain drugs. (e.g., rifampicin for TB is often prescribed by senior clinicians for other conditions.)

Standard treatment guidelines (STGs),[51] commonly referred to as UCG, and the EMHSLU work together and must be used together. EMHSLU has all the essential drugs prescribed in the UCG, with guidance on level of care where they can be prescribed; specialty drugs are reserved for referral facilities, where specialists are found. Both UCG and EMHSLU were due for review in 2020.

Policies recommending adherence to STGs includes using and maintaining the MTC Manual,[52] which helps guide and encourages adherance to using STGs; the manual's scope includes selecting products, ordering and procurement, storage, distribution, and tracking use and accountability.

Consolidated Guidelines for the Prevention and Treatment of HIV and AIDs in Uganda[53]: This is revised every two years; it covers prevention, screening, management of co-infections and non-communicable diseases, prophylaxis and pre-emptive treatment, and when to use antimicrobial medicines in TB and HIV.

Studies/reports on the level of appropriate antimicrobial prescribing and dispensing: A research paper by Nayiga[54] concluded that antibiotics are used differently in treating humans and animals, irrespective of urban centers or rural areas across Uganda. Another study[55] found resistance to ciprofloxacin to be very high at 99.6%, confirming that AMR is a threat. Another research paper[56] revealed that over 82% of infections caused by enterobacteriaceae produced extended-spectrum β-lactamase. This study also reaffirms that AMR is a threat, and that ciprofloxacin is no longer useful. The Uganda National Academy of Sciences (UNAS), together with the Global Antibiotic Resistance Partnership produced a country situational analysis on microbials,[57] which revealed rampant antimicrobial resistance in Uganda and that microbial infections contributed to 37% of all hospital admissions in the country. The most widespread antimicrobial-resistant illnesses identified by UNAS were pneumonia and tuberculosis, among others.

Confirming that AMR is a threat, including watch and reserve drugs, the study by Foster[58] also discovered that the AMR level in the country is high and concluded that "misuse of drugs was the major

[51] MOH. Uganda Clinical Guidelines 2016; https://health.go.ug/sites/default/files/Uganda%20Clinical%20Guidelines%202016_FINAL.pdf

[52] MOH. Medicines and Therapeutics Committee Manual 2018

[53] MOH. Consolidated Guidelines for the Prevention and Treatment of HIV and AIDS in Uganda 2020

[54] Nayiga S, Kayendeke M, Nabirye C, et al. Use of antibiotics to treat humans and animals in Uganda: a cross-sectional survey of households and farmers in rural, urban and peri-urban settings, JAC Antimicrob Resist. 2020;2(4). https://doi.org/10.1093/jacamr/dlaa082

[55] Kakooza F, Musinguzi P, Workneh M, et al. Implementation of a Standardized and Quality-Assured Enhanced Gonococcal Antimicrobial Surveillance Programme in Accordance with WHO Protocols in Kampala, Uganda. Sex Transm Infect. 2021 97(4): 312-6.

[56] Bebell LM, et al. Antimicrobial-resistant infections among postpartum women at a Ugandan referral hospital. 2017 Apr 13;12(4):e0175456. doi: 10.1371/journal.pone.0175456

[57] UNAS, CDDEP, GARP-Uganda, Mpairwe Y, Wamala S. Antibiotic Resistance in Uganda: Situation Analysis and Recommendations. Kampala, Uganda: Uganda National Academy of Sciences, Center for Disease Dynamics, Economics, and Policy. 2015. p. 107. https://www.cddep.org/wp-content/uploads/2017/06/uganda_antibiotic_resistance_situation_reportgarp_uganda_0-1.pdf

[58] Foster SD, Sosa A, Najjuka CF, et al. Drivers of Antibiotic Resistance in Uganda and Zambia. [ppt] June 14, 2011

DISCLAIMER: The authors' views expressed in this publication do not necessarily reflect the views of the United States Agency for International Development (USAID) or the United States Government.

contributor to antibiotic resistance in the country." Laxness in regulating the sale of drugs contributed to misuse.[59] Odoi and Odoi reviewed AMR in Uganda and the attempts being made to contain its spread.[60] They concluded that there is already a high level of drug resistance to commonly prescribed antibiotics in Uganda, including multidrug resistance. Musoke implemented a project to strengthen AMS in Wakiso district in Uganda[61] by using a One Health approach. Results demonstrated that AMS interventions using a One Health approach can promote understanding of the rational use of antimicrobials and improve practices in health facilities and communities. These revelations can inform the design of large-scale national AMS interventions in support of implementation of the Uganda AMR NAP.

The **Uganda AMR NAP**[62] was launched in 2018 to "prevent, slow down, and control the spread of resistant organisms." MOH handles the human sector, and MAAIF takes the lead in the animal sector implementation of the NAP. The action plan sets out a coordinated and collaborative One Health approach, involving key stakeholders in government and other sectors, coordinated by the Uganda National Antimicrobials Resistance Committee. MAAIF has developed EVDL and guidelines for using antibiotics on the farm. Double reporting is practiced as monthly routine reports are delivered to the National Animal Disease Diagnostic and Epidemiology Center regarding AMR (Joint External Evaluation 2017).

Political goodwill from the government:[63] The NRM is the political party in power; in its manifesto, it pledged to institute controls at all levels of the medicine supply chain to ensure that only good and cost-effective medicines are on the market. However, it excluded strategies for AMR. MOH, MAAIF, and NDA should advise on including and prioritizing AMS. There is limited awareness on AMR among the public and policy makers, yet political commitment is key for securing financial commitment and support. One of the assignments for the One Health team is to maintain national and international political support for action.

Public education campaigns on appropriate AMU and drug resistance: MOH has been organizing appropriate medicine use workshops since 2018. This annual event is organized jointly by universities, development partners, MOH, and professional bodies. Different departments within MOH individually organize education campaigns on infection control, rational use of drugs, food hygiene, vector control, and immunization; all these require harmonization rather than stand-alone efforts.

---

[59] Asiimwe J. Antibiotic resistance: AMR a health threat in Uganda. Down to Earth Jan 2020. https://www.downtoearth.org.in/news/health/antibiotic-resistance-amr-a-health-threat-in-uganda-68932
[60] Odoi R, Odoi MJ. Anti-Microbial Resistance in Uganda. Africa Health January 2019
[61] Mukose D, Kitutu FE, Mugisha L. A One Health Approach to Strengthening Antimicrobial Stewardship in Wakiso District, Uganda. Antibiotics (Basel). 2020 9(11): 764. https://pubmed.ncbi.nlm.nih.gov/33142711/
[62] Government of Uganda. Antimicrobial Resistance National Action Plan 2018-2023
[63] NRM Manifesto 2021-2026: Securing your Future; pages 233-238

DISCLAIMER: The authors' views expressed in this publication do not necessarily reflect the views of the United States Agency for International Development (USAID) or the United States Government.

Animal Health

Prescription of Veterinary Antimicrobials

Veterinary antimicrobials are scheduled as prescription drugs, and the NDPA Cap 206 requires that they are dispensed upon prescription by a duly qualified veterinary surgeon. The Veterinary Surgeons Act Cap 277 gives the power to determine who qualifies to practice and prescribe drugs to the UVB. Anyone who intends to practice veterinary medicine in Uganda must apply to the UVB. Upon satisfying the requirements for registration and paying the registration fee prescribed by the board, the board then issues a certificate of registration. Licensed veterinary surgeons or practitioners are required to observe the veterinary code of practice and ethics and pay allegiance to the veterinary oath. The Veterinary Surgeons Act gives powers to UVB to investigate any complaint against a licensed veterinary surgeon and to discipline and/or cancel the holder's license if found guilty of violating codes of conduct by a disciplinary hearing presided over by a magistrate. Any person who practices without a UVB license purporting to be a veterinary surgeon commits an offense and is liable to a less punitive conviction of a fine of only UGX 3,000. The weak punishment prescribed by the Veterinary Surgeons Act to unqualified/unlicensed individuals purporting to be veterinarians has encouraged widespread quackery[64] and abuse of antimicrobials. Although UVB was established by an act of Parliament, it does not get sufficient financial support from MAAIF to carry out its regulatory functions. Lack of continuing professional development (CPD) opportunities in AMR and AMS for veterinarians is another weakness identified.

Dispensing Antimicrobials in Veterinary Drug Outlets

According to the NDPA Cap 206, class B drugs, which includes veterinary antimicrobials, must be dispensed by a pharmacist or a licensed person upon receiving a signed prescription. The law requires that prescription records against which restricted drugs have been dispensed be kept and presented to NDA for inspection. NDA also issued further instruction through Circular No. 004/2018, directing suppliers (pharmacies) to keep detailed information on products dispensed and that such information be kept for two years. What remains unclear is whether NDA enforces the circular in the absence of any publicly available report regarding compliance to the instructions given to suppliers.

The dispensing of veterinary antimicrobials requires technical expertise in matters of animal health. Although Part III article 24 section 3 of the NDPA Cap 206 and NDA Circular 004/2018 provides a general guide on good dispensing practices for controlled drugs, it falls short of international best practices for dispensing restricted VMPs, including antimicrobials. For example, the Australian Veterinary Association (AVA) requires that a licensed veterinarian dispense restricted drugs, such as antimicrobials under his or her care but cannot delegate this to a subordinate. As a good practice, AVA requires that the dispensing veterinarian attach a label to the container indicating the following information: name of owner or custodian of the animal(s); name or species of the animal; name (including business name), address, and telephone number of the veterinarian; name of the drug; the words (in capital letters) "KEEP OUT OF REACH OF CHILDREN", and "FOR ANIMAL TREATMENT ONLY"; and directions for

---

[64] Bukamba NF. Understanding veterinary quackery in Uganda. J Dairy Vet Anim Res. 2019 8(4):153. https://medcraveonline.com/JDVAR/JDVAR-08-00259.pdf

DISCLAIMER: The authors' views expressed in this publication do not necessarily reflect the views of the United States Agency for International Development (USAID) or the United States Government.

use, including safe handling instructions. In Uganda, the general practice under the current regulatory regime is that even licensed veterinary drug outlets do not prioritize prescriptions before dispensing antimicrobials and other VMPs. Similarly, some licensed veterinary drug outlets employ unqualified personnel who dispense antimicrobials and VMPs without prescription and instructions. A study by Dion in the Lira and Mukono districts found that 90% of the veterinary drug shop owners employ unqualified personnel.[65] This calls for UVB and NDA to inspect and censure licensed drug outlet operators who do not comply with the code of conduct as provided in the Veterinary Surgeons Act Cap 277 and NDPA Cap 206, respectively.

AMU Practices in the Animal Sector

The Veterinary Surgeons Act Cap 277 vests the mandate of treatment of animals in the hands of veterinary practitioners. The act defines the practice of veterinary surgery to mean the performance of any operation and the giving of any treatment, advice, diagnosis, or attendance in respect of an animal for gain or reward. The Animal Disease Act Cap 38 (section 9) gives powers to a veterinary practitioner or officer to take samples to detect or diagnose animal disease. In so doing, a veterinary practitioner establishes a relationship with the owner of the animal, also termed a "bona fide" veterinarian-client relationship. In a bona fide relationship, the veterinarian only gives treatment when he/she has seen the animal patient where it is kept, examined clinically, made diagnosis, and then initiating treatment; the individual continues to monitor the animal for any unwanted drug effects or failure in the therapeutic regimen. This is what constitutes ethical use of antimicrobials and other VMP based on the code of conduct and ethics of the veterinary profession. However, this code of conduct is largely not followed because many unqualified actors are currently involved in dispensing and treating animals with antimicrobial drugs and other VMPs. Such practice predisposes to misuse of AM and AMR.

Standard Veterinary Treatment Guide

This document contains the common disease conditions, causes, differentials, clinical manifestations, and diagnosis and treatment options (including first and second line). It is the mandate of MAAIF to develop the standard veterinary treatment guide (SVTG). Currently, there is no treatment guideline for the animal sector in Uganda.

National Veterinary Formulary

This book contains the list of all approved medicines and information about their pharmacology and toxicology. The NVF developed by NDA has not been approved even though it is required by NDPA Cap 206, part II section 2-4, which says that veterinary drugs can only be registered if listed in the formulary.

Essential Veterinary Medicines

The EVML contains the essential medicines that satisfy the therapeutic needs of the country based on disease burden, and it guides appropriate AMU. The list forms a basis for prioritizing registration of

[65] Dione MM, Amia WC, Ejobi F, et al. Supply Chain and Delivery of Antimicrobial Drugs in Smallholder Livestock Production Systems in Uganda. Front Vet Sci 2021 p. 954.

DISCLAIMER: The authors' views expressed in this publication do not necessarily reflect the views of the United States Agency for International Development (USAID) or the United States Government.

antimicrobials and other VMPs as enshrined in the NDPA Cap 206, part II, sections 1 and 2. The NDPA law requires that EVML is revised from time to time. The first edition of EVML was published by NDA in 2001 and was not updated for 19 years. The EVML 2001 was not used by veterinarians because it was not accessible to most of them. In 2020, MAAIF, with support from MTaPS, reviewed the 2001 edition and published it with major changes. The major changes with respect to antimicrobials in EVML 2020 included the introduction of restriction to access and use in compliance with the OIE antimicrobial classification. A special section was also created for listing medicines used in wildlife, aquaculture, and apiary (some of the drugs are antimicrobials).

Guidelines for Appropriate Use of Antimicrobials

The NVDP (2002) emphasizes the need to promote rationale use of antimicrobials and other VMPs. In so doing, animals, the public, and the environment will be protected against harmful effects of veterinary drugs. On the other hand, the NDPA Cap 206, part IX section 59 also mandates that NDA ensure appropriate use of antimicrobials and other VMPs through public awareness and enhancing knowledge on proper use of drugs. Currently, various stakeholders are implementing AMR projects in partnership with MAAIF. Awareness messages on AMR and guidelines for infection prevention and appropriate AMU for cattle, small ruminants, poultry, piggery, and fish farming were developed by MAAIF in 2020. The NDPA Act also mandates that NDA develop basic and postgraduate training in the health sector [Article 59 section 2 subsection (a)]. However, it is not clear why the NDPA law excluded development of such a course for the animal sector, given NDA's statutory role in veterinary drug regulation and AMS.

Laboratory Diagnostic Services in the Animal Sector

The animal sector does not have a laboratory policy that guides strategy for development of animal health laboratory infrastructure and regulation. There are about 40 functional veterinary diagnostic laboratories[66] in the country, of which only 6 carry out bacterial culture and antibiotic susceptibility tests. Aside from the Mbarara and Mbale regional veterinary labs that offer culture and sensitivity services, the other four are all located in Kampala and Wakiso, away from most of the farmers and veterinarians who need their services. These laboratory centers are also constrained by equipment/consumables, personnel, and financial resources.[67] The weak national animal diagnostic laboratory capacity implies that antimicrobial drugs are mainly administered wrongly because animal health workers base their diagnosis mostly on clinical signs.[68] Where bacterial infection is diagnosed through clinical examination, antibiograms are not routinely done prior to treatment because of the lack of functional labs in most of the districts in Uganda.

Training Veterinary Human Resources for AMS/Diploma and Degree Programs

Training institutions play an important role in determining the quality of human resources that handle antimicrobials and other VMPs. Currently, more than 10 universities and tertiary institutions are

---

[66] FAO. Veterinary Laboratory Mapping Report; key informant interview. 2018
[67] Nakayima J, Nerima B, Sebikali C. et al. An assessment of veterinary diagnostic services needs in Uganda. J Vet Med Anim Health 2016 8(7), pp.50-55.
[68] OIE. OIE PVS Evaluation Follow-Up Mission Report: Uganda. 2019

DISCLAIMER: The authors' views expressed in this publication do not necessarily reflect the views of the United States Agency for International Development (USAID) or the United States Government.

accredited by the National Council for Higher Education (NCHE) to train veterinary and veterinary paraprofessionals.[69] NCHE was established by the Universities and Other Tertiary Institutions Act 2001 to regulate public and private higher institutions of learning. UVB recognizes and licenses only veterinarians from training institutions and programs accredited by NCHE. It is presumed that an accredited program has undergone rigorous stakeholder consultation and meets Day One competencies for veterinary[70] and veterinary paraprofessionals prescribed by OIE.[71] The Day One competence prescribed by OIE for graduating veterinarians on antimicrobials includes knowing common drugs, appropriate use, withdrawal periods in food animals, mechanisms of action, mechanisms of development of AMR in zoonotic pathogens, appropriate use of vaccines, and responsible disposal of antimicrobials and vaccines to protect the environment. The Day One competencies on medicinal products should be used as a benchmark to set standards for development and review of curricula for any veterinary and veterinary paraprofessional cadres intending to practice or dispense veterinary antimicrobials.

Veterinary CPD in AMS/AMU/AMR

MAAIF, Uganda Veterinary Association, and UVB must ensure that veterinarians and veterinary paraprofessionals maintain high standards of practice through CPD. NVDP 2002 gives the responsibility for ensuring that registered veterinarians undertake periodic refresher training on safe and correct use of veterinary drugs to UVB. One of UVB's mandates is to monitor and enforce the standards of CPD education programs. UVB developed guidelines for conducting CPD are available on its website (http://www.ugandavetboard.org/). A survey conducted in 2019 jointly by UVB; Makerere University College of Veterinary Medicine, Animal Resources and Biosecurity (COVAB); and the University of Surrey on the training needs of veterinarians found that AMR/AMS ranked among the top five CPD training needs for veterinarian and veterinary paraprofessionals in Uganda.[72] To this point, no AMR/AMU CPD follow-up training has been done based on the findings of this study.

Disposal of Veterinary Pharmaceutical Wastes

Safe disposal of antimicrobials and other VMPs is an important component of the antimicrobial drug cycle. NVDP 2002 emphasizes the need to regulate disposal of VMPs and gives the mandate to NDA. Disposal of pharmaceutical wastes must be done in accordance with the National Environmental (Waste Management) regulation SI 52 (1999). Poor disposal of antimicrobials in the environment can precipitate emergence of AMR by environmental bacteria, which can potentially cause infection in humans and animals. The NDA guide encourages drug outlets and importers to submit expired products for safe disposal by the regulator or accredited service providers, depending on the weight of the waste. In the absence of an SI for pharmaceutical waste management and incentives for disposal, it remains unclear how NDA effectively implements the guidelines. A study in Tirana, Albania, found that pharmacies were usually reluctant to dispose of pharmaceutical wastes through the regulator because the proper way of

---

69 NCHE accredited course; available at https://www.unche.or.ug/webpages/programlist.aspx

70 OIE. Recommendations on the Competencies of Graduating Veterinarians ('Day 1 Graduates') to Assure National Veterinary Services of Quality. 2012.

71 OIE. OIE Competency Guidelines for Veterinary Paraprofessionals. 2018.https://www.oie.int/fileadmin/Home/eng/Support_to_OIE_Members/docs/pdf/A_Competence.pdf

72 Endacott IC, Ekiri AB, Alafiatayo R, et al. Continuing Education of Animal Health Professionals in Uganda: A Training Needs Assessment. *J Vet Med Educ* 2021: e20200161.

DISCLAIMER: The authors' views expressed in this publication do not necessarily reflect the views of the United States Agency for International Development (USAID) or the United States Government.

disposal is costly, considering they have already made losses on expired products. The same study found that the lack of compliance to appropriate disposal was highly likely when pharmacies were not frequently supervised. This finding indicates that, in the absence of regulatory incentives and regular enforcement supervision, the guideline issued by NDA is poorly adhered to. A key informant with knowledge of poor disposal of pharmaceutical waste, including antibiotics, listed common practices, such as dumping nearly expired drugs by distributors to drug shops and other markets, then directly to farmers at half price to avoid the cost of disposal.

Veterinary Community Engagement by NDA and MAAIF

Both NVDP 2002 and NDPA Cap 206 recognize the need to promote public awareness and knowledge on the proper use of drugs and sensitization of stakeholders on drug regulations. Currently, most veterinary drug-related outreaches at livestock farms are carried out by NDA. Available information from NDA shows that, in FY2019/20, NDA carried out 755 community outreaches, of which only 33 (4%) were specifically for engagement of veterinary stakeholders (appendix 2, figure A5). The low level of engagement could be attributed to the few numbers of veterinarians employed in NDA, among other reasons. MAAIF, on the other hand, does not have a regular outreach program specifically for VMPs. This mainly arises from the fact that, since NDA was formed, MAAIF has never had a unit or division specifically designated for veterinary pharmaceuticals. This has created a big gap in developing appropriate policies, legal regimes, and strategies for strengthening the veterinary pharmaceutical sector. Recently, the commissioner of animal health established a drug desk at MAAIF, manned by two staff who have other job duties in addition to the drug desk. The oversight role on rational use of veterinary antimicrobials has been devolved from MAAIF to the district veterinary department, following the decentralization policy[73] and enactment of the Local Government Act, 1997.[74] However, weak coordination between MAAIF and DLG in promoting rational use of antimicrobials negatively affect AMS. Thus, in the absence of a full-fledged unit or department in MAAIF with legal mandate over veterinary medicines, the regulation of animal drugs and specifically antimicrobials will remain challenging and exacerbate the already alarming trends in AMS and AMR burden in the animal sector.

High Risk of Antimicrobial Misuse and Residues in Poultry and Cattle Products

Several poultry diseases occur in Uganda, including Newcastle disease, fowl pox, Marek's disease, fowl typhoid, fowl cholera, and coccidiosis. Other poultry diseases caused by viral, bacterial, protozoan, chlamydial, and fungal and helminths, among others, occur frequently. In cattle, endemic diseases, such as tick-borne diseases, and viral and bacterial diseases occur in various proportions and causes losses to farmers. The booming commercial poultry sector, especially in urban and peri-urban areas, has increased the demand for antimicrobials for both treatment and prophylaxis. With weak diagnostic and extension services, drug shops are the most immediate point of care where antimicrobial drugs are handed over to mostly unqualified persons to carry out treatments irrationally. The liberalization of veterinary drugs

---

[73] Wesonga WS, Madasi B, Nambo E. Factors Associated with a Low Veterinary Regulatory Compliance in Uganda, Their Impact and Quality Management Approaches to Improve Performance. Open Journal of Veterinary Medicine, 2018, 8(12), p. 207.

[74] The Local Governments Act, Cap 243. https://www.ec.or.ug/docs/LOCAL%20GOVERMENTS%20ACT.pdf

DISCLAIMER: The authors' views expressed in this publication do not necessarily reflect the views of the United States Agency for International Development (USAID) or the United States Government.

that made the private sector take the lead in the animal drug supply chain, coupled with minimum government control, has resulted in easy access to antimicrobials and misuse of antimicrobials.[75] Unqualified persons are likely to use antimicrobials for treating almost all illnesses, even when it is not necessary. With lack of knowledge or poor adherence to the withdrawal period, the burden of antimicrobial residues is increasing in poultry (meat and eggs) and cattle (milk and meat) products.

A previous study showed that 13% of 284 milk samples obtained from milk-cooling centers in the Mbarara and Masaka districts and 18% of 453 samples of edible cattle tissues had violable levels of penicillin G (4 µg/L) as set by the European Union.[76] It was also found that 96% of farmers and their managers did not adhere to drug withdrawal periods after treating animals with antibiotics, and only 14% were aware of the dangers associated with exposing consumers to cattle product that contain offending levels of antibiotics. This finding agrees with a similar study done by Byaruhanga[77] in 2020. The presence of residues in animal products affects Uganda's compliance with the World Trade Organization's sanitary and phytosanitary measures.[78] Consumption of animal products contaminated with antimicrobial residues is a public health threat responsible for emergence of AMR in human pathogens. The AMR NAP recognizes the threat posed by irrational use of antimicrobials in food animal production and has proposed a One Health strategy for mitigating its impact. A One Health national technical committee has been formed to guide and accelerate intersectoral implementation of the NAP.

Regulation of Antimicrobials in Animal Feeds

The Directorate of Animal Resources (DAR; part of MAAIF) is the authority responsible for regulating animal feeds in Uganda. The animal feed policy[79] provides the strategic policy environment for guiding the development and regulation of the animal feed sector. Feeds are commonly used as a medium for delivering antimicrobials in intensive livestock production for prophylaxis and growth promotion. The realization that using medicated feed increases the risk for emerging AMR has compelled the commissioner of animal health at MAAIF to ban importation of premixes and concentrates that contain antimicrobials. However, in the absence of a law that regulates the feed sector, adulteration of feeds with antimicrobials remains a major concern. The OIE Performance of Veterinary Services report[80] also scores Uganda at level 1, indicating that the country cannot regulate animal feeds. An unpublished 2020 study carried out at COVAB on adulteration of pig feeds with antimicrobials revealed that 100% of the

---

[75] Ilukor J, Birner R, Rwamigisa P, Nantima N. Analysis of veterinary service delivery in Uganda: An application of the Process Net-Map Tool. Hohenheim and Kampala: University of Hohenheim, Institute of Agricultural Economics and Social Sciences in the Tropics and Subtropics, Germany, and Ministry of Agriculture Animal Industry and Fisheries Department of Livestock Health and Entomology. [Google Scholar] (2013).

[76] Sasanya et al. 2008. Public Health Perspectives of Penicillin G Residues in Cow Milk and Edible Bovine Tissues Collected from Mbarara and Masaka Districts, Uganda. https://www.researchgate.net/publication/216198198_Public_Health_Perspectives_of_Penicillin_G_Residues_in_Cow_Milk_and_Edible_Bovine_Tissues_Collected_from_Mbarara_and_Masaka_Districts_Uganda

[77] Byaruhanga J, et al. Comparison of Tick Control and Antibiotic Use Practices at Farm Level in Regions of High and Low Acaricide Resistance in Uganda. 2020. Available from https://pubmed.ncbi.nlm.nih.gov/32908661/

[78] World Trade Organization. WTO Agreements Series: Sanitary and Phytosanitary Measures. https://www.wto.org/english/res_e/booksp_e/agrmntseries4_sps_e.pdf

[79] MAAIF. National Animal Feeds Policy. Kampala: MAAIF; 2005.

[80] OIE. PVS Evaluation Follow-up Report for Uganda, p. 113; 2018.

DISCLAIMER: The authors' views expressed in this publication do not necessarily reflect the views of the United States Agency for International Development (USAID) or the United States Government.

30 pig feeds sampled at swine farms contained at least one detectable level of antimicrobials.[81] The detection level of antimicrobials in poultry feed was 80% in a similar study conducted on 30 poultry farms in another study.[82] The most detected antimicrobials in both pig and poultry feeds included tetracycline, sulfonamides, and erythromycin at various concentrations. Considering that both studies found that most pig and poultry farmers rarely observe the withdrawal period when selling their animals for slaughter and eggs for consumption underpins the risk of exposing humans to residues of antimicrobials through consumption of animal food sources. Stakeholders in the animal sector have urged MAAIF to introduce legislation to regulate the animal feed sector. MAAIF has drafted the Animal Feed Bill.[83] The principles of the bill were approved by the Cabinet in 2019, but to this point the bill has not been enacted. The proposed animal feed law is expected to regulate the import/export, manufacture, licensing of personnel and premises, registration of feeds (including premixes and concentrates), distribution and enforcement for compliance, and guarding against adulteration of animal feeds with unauthorized chemicals and drugs, such as antimicrobials, to protect animal and public health.

Regulation and Appropriate Use of Animal Vaccines

Animal vaccines are central to animal disease prevention and control. The OIE prioritized vaccination as one of the key strategies against AMR.[84] The Government of Uganda has also identified vaccination as one of the key strategies against AMR in its NAP. This is based on the principle that most of the diseases that are treated using antimicrobials can be prevented through vaccination especially in food animals such as poultry and ruminants. The Animal Diseases Control Act gives the mandate of control of notifiable diseases (rabies, anthrax, foot and mouth disease [FMD], contagious bovine pleuropneumonia [CBPP], and lumpy skin disease [LSD]) to the central government. The rest of the diseases are categorized as private good, and their control is the responsibility of the animal owner and local animal health officials in local government and private practice. MAAIF procures limited doses of vaccines against FMD, rabies, LSD, and peste des petits ruminants virus annually, but not enough to cover more than 5% of the susceptible animal population. The supply of vaccines against notifiable diseases is state-controlled. Vaccines are centrally procured by MAAIF and distributed to district veterinary departments in local government. Supply of vaccines against "private" diseases is done by the private sector.

NDA is mandated by NDPA Cap 206 to regulate vaccine registration and importation. However, the commissioner of animal health approves the vaccines that NDA registers. Currently, the country imports up to 58 different types of animal vaccines, mostly through the private sector. Over 50% of these are for poultry,[85] an indication of the indispensable role of vaccination in prevention of poultry diseases. The major challenges affecting vaccine regulation and uptake include lack of national capacity for vaccine quality assurance, low funding for the national animal vaccine store to procure and distribute

---

81 Nabanda, Vudriko. Assessing antibiotic use in pig feeds and production in Kampala and Mukono district. 2020 [unpublished]

82 Tayebwa, Vudriko, 2020. Assessing antibiotic use in poultry feeds and production in Kampala and Wakiso districts. 2020 [unpublished]

83 Draft animal feed bill, June 19, 2019. https://budget.go.ug/sites/default/files/Sector%20Spending%20Agency%20Budgets%20and%20Performance/2019-2020_VoteMPS_010_MinistryofAgriculture_Animal_Fisheries_4_2_201911_56_40AM.pdf

84 OIE. OIE Strategy on Antimicrobial Resistance and the Prudent Use of Antimicrobials. 2016

85 MAAIF. Animal Vaccine Importation Data for 2018–2020; data obtained from INOVAC project, COVAB.

DISCLAIMER: The authors' views expressed in this publication do not necessarily reflect the views of the United States Agency for International Development (USAID) or the United States Government.

animal vaccines for notifiable diseases, weak regulation of animal vaccines, a poor cold chain management system affecting the viability of vaccines, presence of unregistered vaccines, unconfirmed reports of fake vaccines, and weak post-vaccination monitoring. Outbreaks of diseases among vaccinated flocks affects farmers' confidence on the value of vaccines. Makerere University currently supports a research team working closely with MAAIF to roll out post-vaccination monitoring services offered by the RTC Laboratory (COVAB) to poultry farmers to optimize vaccination and reduce overdependence on antimicrobials for prophylaxis and growth promotion.

Acaricide and Insecticide Regulations and Impact on AMS

Acaricides and insecticides are a class of chemicals called ectoparasiticides used for controlling external parasites and vectors, such as ticks, tsetse flies, mites, and lice. Although ectoparasiticide regulation has no direct impact on AMS, it should be noted that failure to regulate them appropriately predisposes emergence of acaricide and insecticide resistance. Currently, Uganda is experiencing one of the worst acaricide resistance crises, especially in the southwestern and central cattle corridors. Most farms have run out of available chemical options for controlling ticks, resulting in an outbreak of tick-borne diseases such as East Coast fever, babesiosis, and anaplasmosis. High incidence of these three diseases has led to exponential consumption of antimicrobials, such as oxytetracycline, a first-line drug used by farmers and herdsmen to treat almost every illness in cattle. This perhaps explains why the import of tetracycline accounts for almost half of the total quantity of antimicrobials recorded by NDA in Uganda in 2019. NDPA Cap 206 SI 30 provides a guideline for conducting trials on ectoparasiticides. The guidelines notwithstanding, NDA and MAAIF have not implemented a centralized acaricide rotation plan in licensing of ectoparasiticides so that one or two reservoir molecules are kept out of the market, which has made the country vulnerable to acaricide resistance, with collateral damage to AMS and contamination of animal products, as reported by Byaruhanga.[76]

Antibiotic Use in Wildlife

Various antibiotics are being used to treat sick animals and for infection control after surgery either in the wild or zoos. A broad range of antibiotics used for wildlife include fluoroquinolones, tetracyclines, trimethoprim, sulfonamides, and cephalosporins. Although there are guidelines for appropriate use of antimicrobials in domestic animals, there is no publicly available guideline for AMU in wildlife. It is important to note that all antibiotics used on wildlife animals are categorized as either highly critical or important based on OIE classification criteria. Although AMR in wildlife is occasionally reported, there are growing concerns that close interaction between humans and wild animals are responsible for cross-transmission of AMR between humans and wildlife. In captive animal establishments such as zoos, antimicrobial-resistant pathogens can also be transferred to animals through meat they are fed on, especially carcasses contaminated with antibiotic-resistant organisms. This presents emerging challenges in AMR/AMS policies and regulations at the wildlife–domestic interface and zoos.

DISCLAIMER: The authors' views expressed in this publication do not necessarily reflect the views of the United States Agency for International Development (USAID) or the United States Government.

ANTIMICROBIAL REGULATION AND USE IN CROP PROTECTION

Policy, Laws, and Competent Authority to Regulate Agrochemicals

The agriculture policy gives strategic direction on the need for MAAIF to ensure that farmers have access to inputs and control the quality of agrochemicals.[86] The Plant Protection and Health Act (2015)[87] recognizes the importance of agricultural chemicals in treating plant diseases caused by bacteria and fungi. The regulation of crop protection chemicals is governed by the ACCA.[88] The Department of Crop Inspection and Registration and the Crop Protection Board (CPB) are responsible for implementing the ACCA, and UNBS ensures compliance with the required standards during importation.[89] Bactericide and fungicide are among the agricultural chemicals regulated by ACCA.

Marketing Authorization, Licensing, and Inspection of Crop Protection Antimicrobials

The ACCA mandates that the Agricultural Chemical Control Board (ACCB) regulate agrochemicals in Uganda. The board is currently fully constituted,[90] and its regulatory function is supported by the Agricultural Chemicals Control Technical Committee (ACCTC), inspectors, and analysts for agrochemicals. There is a clear process for registering agrochemicals, including bactericide and fungicides, and this process includes registration of the company and product, laboratory analysis of the agrochemical sample, field trial for efficacy, review of the trial report by ACCTC, decision on registration by CPB, import verification, and obtaining a certificate of conformity to allow importation.

Supply Management Policies and Guidelines for Crop Antimicrobials

MAAIF does not have a specific policy that governs supply of antibiotics and fungicides for crop protection. However, enforcement of regulatory compliance to the ACCA is jointly carried out by agrochemical inspectors and the agricultural police. The list of registered agrochemicals for crop protection includes bactericides and fungicides, such as cooper oxychloride, bacterimycin, Nugro bioactivator (chitin-based product), and Bio Cure-B (biological fungicide). No synthetic antimicrobials used either in livestock or humans have been licensed for use in crop protection by the ACCB.

Post-Marketing Surveillance and PV for Agrochemicals

Post-marketing surveillance is carried out by the agrochemical inspectors in the department of crop inspection and certification, and enforcement is supported by the agricultural police. The ACCA law permits inspectors, with the aid of the police, to close unlicensed agrochemical shops. The agrochemical analysis laboratory established under the ACCA supports quality audits of agrochemicals. The departments of crop protection, crop inspection, and certification work closely with the Uganda

---

[86] MAAIF. National Agriculture Policy. 2013. https://www.agriculture.go.ug/wp-content/uploads/2019/04/National-Agriculture-Policy.pdf

[87] Republic of Uganda. Plant Protection and Health Act. 2015. http://extwprlegs1.fao.org/docs/pdf/uga158615.pdf

[88] Republic of Uganda. Agricultural Chemicals (Control) Act 2007. Uganda Gazette No. 17, Vol. C, 5th April.

[89] de Boef W. Counterfeiting in African Agriculture Inputs: Challenges & Solutions. 2014. https://agrilinks.org/sites/default/files/resource/files/BMGF_Addressing%20Counterfeit%20Ag%20%20Inputs_Research%20Read-out%20(2)%20(1).pdf

[90] MAAIF. 2019. New Boards for Seed and Agricultural Chemical Control Inaugurated by Agriculture Minister. 2019. https://www.agriculture.go.ug/new-boards-for-seed-and-agricultural-chemical-control-inaugurated-by-agriculture-minister/

DISCLAIMER: The authors' views expressed in this publication do not necessarily reflect the views of the United States Agency for International Development (USAID) or the United States Government.

National Association of Community and Occupational Health to support monitoring agrochemical safety among users.

Prescribing, Dispensing, and Using Agrochemicals

The Department of Crop Protection is mandated to ensure that agrochemicals, including antibiotics, are correctly and safely used. MAAIF has developed a manual for responsible use of pesticides, which helps guide extension workers and farmers on appropriate and safe use of chemicals. However, the guide does not include a section on antimicrobials because the register for pesticides, at the time of publication, did not contain any conventional antibiotics. On the other hand, fungicides are widely used and would necessitate guideline for their appropriate use and prevention of fungicide resistance.

ANTIMICROBIAL REGULATION AND USE IN AQUACULTURE

Policy, Laws, and Competent Authority to Regulate Antimicrobials Used in Fish

The Directorate of Fisheries Resources (DFR) is the competent authority responsible for development, review, and implementation of policies, laws, regulations, standards, and guidelines for the fish sector.[91] The strategic development and regulation of the aquaculture sector is guided by the Uganda Agriculture Policy (2013) and the 2018 National Fisheries and Aquaculture Policy.[92] The Fish Act Chap 197[93] is the main law that governs the capture fish industry, but it is now considered obsolete. A new draft bill on fisheries and aquaculture[94] was drafted to repeal the Fish Act and create a new law that addresses the needs of the aquaculture industry.

Marketing Authorization, Licensing, and Inspection of Fish Antimicrobials

The use of chemicals and antibiotics for controlling and treatment of fish diseases is provided for in the SI on Fish (Aquaculture) Rules.[95] At the operational level, the Fisheries Regulation and Control Division and Fisheries Quality Assurance and Safety Division are responsible for regulatory enforcement of aquaculture rules. The rules define an antibiotic as "a chemical or biological agent or a combination of both that can destroy or prevent the growth of bacteria and other microorganism." The rules also require that any person who intends to produce inputs, such as antibiotics, feeds, and hormones, for use in aquaculture must certify these products with a competent authority. NDA[96] is currently the authority that licenses aquaculture drugs under special arrangement with MAAIF. NDA licenses aquaculture products upon clearance by the commissioner for animal health-MAAIF.

---

[91] MAAIF. Directorate of Fisheries Resources. http://www.agriculture.go.ug/directorate-of-fisheries-resources/
[92] National Fisheries and Aquaculture Policy. 2017. http://extwprlegs1.fao.org/docs/pdf/uga201565.pdf
[93] Fish Act Chap 197. https://businesslicences.go.ug/kcfinder/upload/files/The%20Fish%20Act%20Cap%20197.pdf
[94] Stop Illegal Fishing. FAO, Ministry of Agriculture in joint effort to regulate fishing in Uganda as stakeholders validate Fisheries and Aquaculture Bill 2018. https://stopillegalfishing.com/press-links/fao-ministry-of-agriculture-in-joint-effort-to-regulate-fishing-in-uganda-as-stakeholders-validate-fisheries-and-aquaculture-bill-2018/
[95] Fish (Aquaculture) Rules 2003 No. 81
[96] Key informant interview respondent from NDA

DISCLAIMER: The authors' views expressed in this publication do not necessarily reflect the views of the United States Agency for International Development (USAID) or the United States Government.

Supply Management Policies and Guidelines for Fish Antimicrobials

The EVML of 2020[97] contains seven antibiotics: chlortetracycline, enrofloxacin, erythromycin, oxytetracycline, sulfadimethoxine, sulfadimetoprim, and sulfamerazine. Aside from the fluoroquinolone-enrofloxacine that is designated as restricted, the others are not. Currently, NDA does not include fish drugs and antimicrobials in its veterinary drug register. No antibiotic has been specifically registered for use in aquaculture by NDA.[98] However, some farmers use livestock (poultry) antibiotics, especially oxytetracycline, in hatcheries, nurseries, and grow-out systems without veterinary advice.[99]

Post-Marketing Surveillance and PV for Fish Antimicrobials

The aquaculture rules give the power to aquaculture inspectors to enter any premise where aquaculture is practiced and cease the use of illicit drugs, chemicals, and feeds that can harm both fish and the public. However, the Fish Act is weakly enforced because of the lack of appropriate SIs.

Prescribing, Dispensing, and Using Antimicrobials in Aquaculture

There is one guideline on infection prevention and appropriate AMU[100] which is not widely disseminated to inform practice. There is no fish disease treatment guideline to support fish health workers in prudent drug prescription and case management using antimicrobials. One study investigated the susceptibility of bacteria isolated from tilapia and catfish and found that bacteria isolates from the two fish species were susceptible to common antibiotics.[101] However, the authors warned that with intensification of aquaculture and use of animal manure to fertilize fishponds, AMR is likely to emerge in fish bacteria.

---

[97] MAAIF. Essential Veterinary Drug List for Uganda 2020, p. 88
[98] Key informant interview from NDA
[99] Key informant interview from NARO
[100] MAAIF. Guidelines for Infection Prevention and Appropriate Antimicrobial Use in the Animal Sector: Fish Farming. 2020
[101] Wamala SP, Mugimba KK, Mutoloki S, et al. Occurrence and Antibiotic Susceptibility of Fish Bacteria Isolated from Oreochromis niloticus (Nile Tilapia) and Clarias Gariepinus (African catfish) in Uganda. Fish Aquat Sci 2018 21(1), 1-10. https://fas.biomedcentral.com/track/pdf/10.1186/s41240-017-0080-x.pdf

DISCLAIMER: The authors' views expressed in this publication do not necessarily reflect the views of the United States Agency for International Development (USAID) or the United States Government.

PROPOSED NEXT STEPS AND RECOMMENDATIONS

HUMAN

Uganda has several laws developed from the Constitution as the supreme law to regulate pharmaceutical products. The National Drug Policy provides guidance on national commitments and health aspirations. The main regulatory actor is NDA, created in 1993, which has evolved to a level of being recognized regionally and internationally because of the quality of its regulatory practices. NDA developed 9 SIs and more than 20 guidelines (regularly revised) to implement the NDA act that covers both human and animal drugs. There is no specific law to address AMS, but some aspects are addressed in several existing laws. Some laws are outdated and do not address current AMS challenges, and conflicts of mandates also exist. It has been revealed that some areas of AMS have no existing legislation and requires development, like drug pricing and pharmaceutical waste management.

The existing laws and guidelines on supply management are adequate, however, implementation and regulatory supervision are the major challenges. The public sector has a human resource gap of qualified personnel to implement whereas the private sector generally does not adhere to the existing supply chain guidelines, especially at the end of the supply chain. However, import and export regulations are adhered to with strict monitoring by NDA, on top of in-house quality measures, like minilabs, by the major importers. NDA does effective post-marketing surveillance for quality and takes regulatory actions; however, this stops at pharmaceuticals, and medical technologies are not well regulated. NMS has challenges in meeting acceptable procurement standards due to restrictive directives from MOFPED.

Outdated laws, such as the food and drugs act, should be amended to address current challenges and conflicting mandates. New legislation must be developed to address areas of AMS with no specific law, such as pharmaceutical waste management and drug pricing, that impact rational use in a country with high out-of-pocket expenditures on drugs. Government should be lobbied to increase health sector funding and prioritize AMS in its political agenda. MOFPED should be advised to desist from giving directives to NMS that compromise good supply management and procurement principals. Training institutions and professional bodies should emphasize and enforce adherence to good distribution and prescribing practices. NDA should amend the drug schedules to recognize the new prescribers that government is introducing at the various service levels. NDA should include regulating medical technologies (technovigilance) among its regulatory priorities. EMHSLU be amended to introduce the AWaRE classification. Finally, if these interventions are implemented and the population is sensitized on AMS, Uganda will achieve effective AMS.

ANIMAL, FISH, AND CROP SECTORS

To achieve the AMR NAP strategic goals for AMS, in line with the International Health Regulations benchmark 3.4 and the tripartite (FAO, OIE, and WHO) international instruments on the use of antimicrobials in the animal and plant sectors, the following is recommended:

DISCLAIMER: The authors' views expressed in this publication do not necessarily reflect the views of the United States Agency for International Development (USAID) or the United States Government.

Short Term

Strengthen AMS along the supply chain: The NDA needs to strengthen compliance enforcement for GDP, good pharmacy practices, and good dispensing practices; apprehend unqualified personnel in veterinary drug outlets; have the statutory body (UVB) strictly enforce professional standards; and ensure that antimicrobial consumption data are captured at all levels (imports, sales, and use).

Fully adapt the AMR NAP on AMS into MAAIF activity plans and track implementation progress: Although the human sector has made significant progress, the animal, fish, and crop sectors are lagging. MAAIF and the Ministry of Local Government (DVOs, district fisheries officers, and district agricultural officers) should be supported to accelerate domestication of the AMR NAP into their activity plans and monitor progress. The participation of the animal, fish, and crop sector actors in the various One Health intersectoral committees on AMR and AMS should be strengthened.

Strengthen post-marketing surveillance and PV: MAAIF and NDA need to strengthen enforcement of post-marketing surveillance to ensure that only quality antimicrobials and vaccines circulate in the market. More efforts need to be put on the animal, aquaculture (fish), and crop sectors since the human sector has made more progress. This should be done in parallel with building national capacity for PV across the above sectors.

Promote appropriate use of antimicrobials: The government needs to support development of diagnostic laboratory infrastructure in the animal sector to enhance good clinical practices; strengthen AMS content in the curriculum for training veterinarians and fish health specialists; advocate for mass sensitization of the population on AMR to promote behavioral change against self-medication; build sustainable capacity for appropriate disposal of antimicrobials at various nodes (importers, pharmacies and drug shops, DVO, and farms) of the antimicrobial supply chain to protect the environment.

Medium to long term

Register and regulate antimicrobials: MAAIF needs to strengthen the structural gaps that affect effective antimicrobial regulation in the animal sector, in line with the OIE veterinary medicinal product legislation and AU-IBAR-EAC recommendations.

Develop AMS specific policies: MAAIF, NDA, and stakeholders should develop AMS-specific policies and guidelines for the animal, fish, and crop sectors to support AMS.

Review existing laws to incorporate AMS: The laws for professional regulation and disease control in the animal, fish, and crop sectors need to be reviewed by MAAIF to include AMS, taking advantage of the polices and laws that are under revision, such as the veterinary medicines, vaccines, and devices policy (2020 draft); the Uganda veterinary and paraveterinary practitioners' bill; animal feed bill, and fisheries and aquaculture bill.

DISCLAIMER: The authors' views expressed in this publication do not necessarily reflect the views of the United States Agency for International Development (USAID) or the United States Government.

CONCLUSION

In the human health sector, the existing laws and guidelines on supply management are adequate, however, implementation and regulatory supervision are the major challenges. The public sector has a human resource gap of qualified personnel to implement AMS, and the private sector generally does not adhere to the existing supply chain guidelines, especially at the end of the supply chain. However, import and export regulations are adhered to with strict monitoring by NDA, on top of in-house quality measures, like minilabs, by major importers. NDA does effective post-marketing surveillance for quality and takes regulatory actions; however, this stops at pharmaceuticals only as medical technologies are not well regulated. NMS has challenges in meeting acceptable procurement standards because of restrictive directives from MOFPED.

In the animal sector, the policy on veterinary drugs and laws for animal disease control and veterinary profession regulation needs to be reviewed and amended because they are very old and do not sufficiently address AMS. Although NDA has developed several SIs and guidelines for regulating veterinary drugs, structural challenges and weak enforcement affects efficient regulation and monitoring the quality of antimicrobials on the market. This is further compounded by lack of a full-fledged division or department responsible for providing policy direction on VMPs, antimicrobials, and strategic AMS interventions at MAAIF. MAAIF should consider establishing a full-fledged division for veterinary pharmaceutical service under the Department of Animal Health to support AMS policy development and strategic guidance. It is recommended that outdated laws, like the food and drugs act, be amended to address AMS and conflicts in mandates. New legislation needs to be developed to address areas of AMS with no specific laws addressing them, such as pharmaceutical waste management and drug pricing, that impact rational use in a country with high out-of-pocket expenditures on drugs. Government should be lobbied to increase health sector funding and prioritize AMS in its political agenda. MOFPED should be advised to desist from giving directives to NMS that compromise good supply management and procurement principles. Training institutions and professional bodies should emphasize and enforce adherence to good distribution and prescribing practices. NDA should amend the drug schedules to recognize the new prescribers that the government is introducing at various service levels. NDA should include regulating medical technologies (technovigilance) among its regulatory priorities. EMHSLU should be amended to introduce the AWaRE classification. Finally, if the above interventions are implemented and the population is sensitized on AMS, Uganda will achieve effective AMS.

The challenges affecting appropriate antimicrobial prescription and use in the animal sector include lack of access to guidelines for AM prescription for veterinarians; lack of an STG; dispensing of antimicrobials mostly without prescription, exacerbated by weak supportive supervision to ensure adherence to good pharmacy practices; illegal stocking of drug shops with antimicrobials; excessive use of antimicrobials in intensive poultry production for prophylaxis and growth promotion; weak pre-service and CPD trainings on AMS; widespread misuse of antimicrobials by unqualified persons because of weak professional regulation; lack of therapeutic committees at national and DVO levels to guide rational use of antimicrobials; weak post-market quality assessment of veterinary antimicrobials; poor regulation of animal vaccine; and lack of guidelines for good disposal of antimicrobials on farms. The few infection prevention and control materials that have been developed by MAAIF and its partners have not been disseminated widely. The structural weakness in NDA warrants creation of a competent authority

within or independent of NDA for effective regulation of VMPs and antimicrobials, in line with Uganda's Cabinet directive on establishing a separate veterinary drug regulatory authority (2019) and OIE's and AU-IBAR/EAC's recommendations.

In the aquaculture sector, the situation is far worse because there are no legal instruments that govern appropriate antimicrobial registration, licensing, registration of personnel and premise, conduct of clinical trial, control of publication and adverts of drugs, import and export of drugs, suitability of premise, PV, and disposal of antimicrobials used in aquaculture. Thus, there is need to fast-track both policy and laws for regulating aquaculture health products, including antimicrobials by MAAIF.

In the crop sector, conventional antimicrobials used in animal and human health have not been registered for use in crop protection in Uganda. However, there are glaring policy gaps for AMS because of the lack of AMS-specific guidelines for crop production.

Overall, data on implementing the AMR NAP on AMS in the animal, crop, and aquaculture sectors are lacking. It is therefore recommended that both MAAIF and the Ministry of Local Government need to adapt the key AMS strategic activities in its sectoral annual plan and allocate resources for implementation, monitoring, and evaluation.

AMS POLICIES, REGULATIONS, AND GUIDELINES ANALYSIS; GAPS IDENTIFIED; AND RECOMMENDATIONS

HUMAN HEALTH SECTOR

Policy/regulation/guideline	Identified gaps	Recommendations
**Constitution of Uganda 1995**	Under the 20th Objective, the state is mandated to provide citizens with only basic medical services.	Government should be lobbied to raise the constitutional requirement of providing basic health care to providing comprehensive health care.
**Second National Health Policy 2010**	Inadequate funding for the health sector for procuring medicines	Policy should be revised, specifically the strategy for resource allocation for health care, including innovative strategies like health insurance
**Health Sector Development Plan III, 2015/16–2019/20**	■ Inadequate funding for the health sector, government is not making adequate investments to fight AMR danger<br>■ High out-of-pocket medical expenditure by citizens<br>■ It was planned to ensure that the ruling party manifesto be fully aligned to the plan BUT the 2021–2026 NRM manifesto did not include strategies to fight AMR.	■ Advocate for government funding to the health sector to be increased and expanding the funding options to include health insurance options<br>■ Because the government is a signatory to the Abuja declaration, it should be reminded to contribute 15% of its annual budget to health care improvement<br>■ AMR taskforce should always ensure that the AMR agenda is in the national political agenda.
**Marketing authorization, licensing, and inspection**		
**Chapter 278 - The Food and Drugs Act. 18 June 1959**	■ The act is not explicit on mechanisms and guidelines for accreditation of certification bodies or persons engaged in food businesses and procedure and guidelines for accrediting food safety laboratories and notification of the accredited laboratories.<br>■ Outdated penalties for breach of this act are very low and not prohibitive<br>■ Ministry has not developed regulations and infrastructure to operationalize the act.	■ MAAIF should develop regulations to implement provisions of the act.<br>■ The penalties for breach should be revised to be tougher, up to life imprisonment like in India.[102]<br>■ Country should start adopting mandatory traceability for critical products, such as fish, cattle, chicken, eggs, pork, and milk.<br>■ Government should support investment in food safety infrastructure, i.e., laboratories, slaughterhouses.<br>■ NGOs, community-based organizations, and civil society organizations should be supported to run advocacy campaigns and educate the public/farmers on dangers of food adulteration.
**National Drug Policy 2002**	■ Gross underfunding of the drug budget<br>■ Policy does not address drug pricing<br>■ Sparse availability of quality prescribers in health facilities and no strategy to alleviate this in the policy	■ Advocate for better funding to implement the policy objectives<br>■ Drug pricing policy and price regulatory framework should be developed.<br>■ Support should be given to relevant bodies to train or retrain the different prescribers

[102] Prevention of Food Adulteration (Uttar Pradesh Amendment) Act, 1976, India

Policy/regulation/guideline	Identified gaps	Recommendations
	■ Although mention is made of the challenges of antibiotic availability, even without prescription, which is one of the drivers of AMR, the policy provided no remedy.<br>■ MOH-PD is mandated to quantify national requirements for pharmaceutical products. (National Medicine Policy 2015, page 21), but this is also a statutory requirement for NDA (NDA Act 1993 Part III art. 10 sec1, 2 and 3; same mandate to NMS under NMS Act 1993 Part II sec 5).<br>■ Policy does not mention systems for licensing or tracking traditional healers or their products, and as such, a lot of unsubstantiated claims are made about TCMs	■ Training institutions should be supported to emphasize AMR/AMS during training or on-the-job training.<br>■ Responsibility for quantification must be completely removed from NMS and NDA and given to MOH because quantification is part of medicine management and should be carried out by the PD so that NMS focuses on medicine supply based on the quantification outcome to avoid conflict of interest; amend NDA and NMS acts.<br>■ Deliberate effort made to support local manufacturers of priority antimicrobial agents; National Chemotherapeutic Laboratory and NDA should be supported to identify, recognize, and process approval/integration of TCM products that can be used as alternative, affordable, and accessible antimicrobials
**National Pharmaceutical Sector Strategic Plan III 2015–2020**	Over 75% of disease burden can be prevented through health promotion and prevention; more effort and strategies should be targeted to preventive rather than curative measures, but there are no measures to achieve this.	Research in preventive medicine to identify fundable health preventive measures that reduce use of antimicrobials and thus reduce AMR; this will provide relief to the curative drug financing burden and AMR (vaccination, sanitization, laboratory services should be supported).<br><br>Development of IT-based communication system for data collection, analysis, and feedback using the countrywide telephone network by both health workers and the public and for PV
**NDA Act 1993**	■ Law does not clearly specify regulation of medical devices.<br>■ Lack of harmonization of different institutional mandates and limited collaboration with other agencies; conflicting and duplicative roles with other ministries, departments, and agencies (NMS, MAAIF, UNBS)<br>■ Weak veterinary medicine regulation in collaboration with players in veterinary sector<br>■ Large board membership with conflicts of interest in representation (major stakeholders regulated, e.g., JMS, NMS, sit on the board)<br>■ Several government policies and bills that would complement NDA act are still at the draft stage stagnating regulatory progress	■ Amend the act to meet current global and national challenges (to include identified poorly or unregulated products, review board composition and size)<br>■ Develop other laws to separate roles and responsibilities<br>■ Strengthen veterinary component in regulations<br>■ Expedite enactment of outstanding bills (pharmacy profession and practice; national health insurance scheme; TCM; national food and medicines; animal feed; veterinary medicines, vaccines, and technology; para-vet professionals)
**Statutory instruments no. 29. The NDA (registration) regulations, 2014.**	No distinctive provision for antimicrobial agents as is the case for vaccine	Regulations should be amended to specify measures to ensure appropriate registration and monitoring of antimicrobial agents.

Policy/regulation/guideline	Identified gaps	Recommendations
**NDA Guidelines on Submission of Documentation for Marketing Authorization of a Pharmaceutical Product for Human Use – 2018**	The guidelines do not apply to vaccines, biosimilars, biotherapeutics and herbal, preparations. The guidelines are due for review in March 2021.	Guidelines be amended to include antimicrobial products, vaccines, biosimilars, biotherapeutics, and herbal preparations
**NDA guidelines on hand sanitizers for use during the COVID-19 pandemic-April 2020**		Use of sanitizers should be promoted in places where availability of soap and water is limited. It is an effective preventive measure in controlling overuse of antimicrobial agents, especially when used as a prophylactic agent (post-surgery, etc.). The culture of sanitizing has developed, and its promotion should be sustained to reduce the burden on AMU. Research into the effect of sanitizer use on the pressure to use antimicrobials and subsequent impact on AMR should be initiated and supported.
**TCM Bill**	Issuing licenses to manage TC drugs is proposed to be given to the council, which will conflict with NDA mandate<br>President has taken too long to assent to the bill	▪ Find out the underlying reasons the president, who has always been very keen on herbals and TCMs, has not yet assented to the bill.<br>▪ There is a lot of excitement with herbals, and herbalists are difficult to tame without an enabling law; lobby to expedite approval with highlighted amendments.<br>▪ Having alternative effective herbal preparations will reduce pressure on conventional antimicrobial agents.<br>▪ Quickly develop infrastructure to address adulterated antimicrobials sold in herbal concoctions, one of the causes of antimicrobial abuse/resistance.
**5. Post-marketing surveillance and PV**		
**NDA PV regulations, 2014**	▪ Medical technologies, e.g., needles and syringes, are not monitored for quality during use in the field, but if they are of poor quality, they can contribute to irrational use or misuse of medicines.<br>▪ NDA produced both a trainer and trainee manual for health workers on PV, but they are not widely disseminated or in use.	NDA should develop regulations and guidelines to start regulating medical technologies (technovigilance)<br>NDA's Medicines Information Directorate should be facilitated to disseminate and promote use of the manuals.
**NDA Conduct of Clinical Trials Regulations, 2013**	The regulation is silent on how or what the process is for handling traditional and complimentary drugs under trial.	Amend regulation to include provisions of managing TCM clinical trials
**NDA Regulation 37: Guideline on submitting periodic drug safety update report, April 2018**	The guideline does not provide for how and when NDA should provide feedback and or respond.<br>Guidelines were due for review April 2021.	NDA should be supported to provide routine reports to the public (feedback to health professionals) on reports received under this guideline to answer questions, such as what is the % adherence to provisions herein by product license holders?

Policy/regulation/guideline	Identified gaps	Recommendations
		Review guideline
**Supply management policies and guidelines for antimicrobials**		
**East African Community Regional Pharmaceutical Plan of Action (EAC-RPMPOA) 2017-2027**	■ The action plan has not been domesticated to fit in the BUBU.<br>■ Harmonization process, including HR plans, stalled because of pressures from professional bodies<br>■ Policies favoring local manufacturing, like tax exemptions, are slowly/selectively being implemented on high tariff goods and services.<br>■ Unit cost for utilities (water and electricity), building costs for cement, etc., not competitive, nor uniform in the community countries<br>■ Strategy emphasizes production and neglects proper use where AMR should have been addressed<br>■ Percentage of medicines used and manufactured in the country stands at around 10%	■ The action plan must be domesticated to fit in the BUBU.<br>■ The harmonization process, including HR plans, should be expedited.<br>■ Develop national policies favoring local manufacturing, like tax exemptions and high tariffs on goods and services produced and available in Uganda<br>■ Pricing structure for major industrial inputs (unit cost for utilities, [water, electricity, building costs for cement, etc.) be revisited and adjusted<br>■ Review strategy and address product use issues, including AMR/AMS
**NDA Importation and Exportation of Drugs Regulations**		Guidelines should be amended to insert provisions to demand user accountability for antimicrobial agents, which are similar to narcotics to avoid mismanagement; demand that drugs are returned to NDA
**Regulations and guidelines to regulate the promotional activities of pharmaceutical products, 2017**	Although medications for specified diseases are listed in the regulations and guidelines to regulate the promotional activities of pharmaceutical products, 2017, for which advertisement is prohibited, no mention is made of antimicrobials.	■ Ensure that NDA is supported in monitoring the many radio stations and other media for compliance<br>■ Revise drug schedules for each category of prescriber, emphasizing antimicrobial agents<br>■ Develop and widely publish/broadcast messages discouraging self-medication as this is a major cause of AMR<br>■ Update guideline (review date is August 4, 2020) to include current issues, e.g., COVID-19
**Essential Medicines and Health Supplies List for Uganda (EMHSLU), 2016**	List still classifies antimicrobial products according to VEN rather than AWaRe<br><br>Current edition was due for review in 2020	Uganda EMHSLU should be reviewed and updated to include new developments, including adopting the new AWaRe categorization as opposed to the VEN system (in current edition); guidance on availability of drugs per level of care should be maintained.
**NAP for Health Security (NAPHS) 2019–2023**	■ The country assessment under NAPHS identified that there is no financing mechanism or funds for a timely response to public health emergencies.<br>■ There is no effective public health response at points of entry.	Government, through the One Health secretariat, should be supported to create a financing mechanism to implement the plan.
**National Medical Stores Act 1993, Chapter 207**	■ NMS mandated to procure basic drugs, but the act does not reference drugs needed when treating drug-resistant cases	■ Quantification should be the responsibility of MOH and the Ministry of Local Government (consumers, not the procuring

Policy/regulation/guideline	Identified gaps	Recommendations
	■ Procuring agency is given the statutory mandate to quantify need and advise NDA, MOH, MOFPED, and the Ministry of Local Government, as per MOFPED; circular on use of local currency for public procurement/contracting precluded international market participation in tendering processes; resulted in lengthening supply chains through use of local agents and increased complexity of supply chain; poses risk of substandard and falsified medicines being introduced into the supply chain<br>■ Weak legal framework and current enforcement mechanism to deter providers of falsified medicines, wherein prosecutions (working with stakeholders like Uganda Police Force and DPP) are through litigation process in court/judicial system with delays and low success rates.	agency to avoid conflict of interest, leading to over procurement and dumping.<br>■ Review NMS and transfer duplicated roles to MOH (see identified gaps).<br>■ The act should be amended to provide for reserve drugs specifically reserve antibiotics.<br>■ Existing law should take precedence over directives/ circulars, in keeping with the principle of supremacy of the law; harmonize with PPDA law that currently allows for use of internationally freely convertible currencies (e.g., USD) to facilitate sourcing of medicines directly from international markets to shorten supply chain and improve access and availability of quality, safe, and efficacious EMHS<br>■ Review and fine-tune current laws/regulations to be more prohibitive and serve as a deterrent to suppliers/ manufacturers of poor-quality products, including, but not limited to, significant financial penalties; institute product recalls and financial compensation for poor-quality products supplied
**PPDA (Procurement of Medicines and Medical Supplies) Regulations, 2014**	Use of generic names is good, but in cases where certain generic brands are not effective, the law is not favorable. The regulation's sec. 12 3 states that guidance for decision making is reference data from WHO and not data locally generated (from users)	The regulation should be amended to provide for use of locally generated evidence in deciding procurement of drugs, especially antibiotics, where resistance to some molecules might be detected locally by users.
**Draft National Medical Countermeasures Supply Chain Plan 2019**		This comprehensive multisectoral tool needs to be incorporated into the health system with lessons learned from the pilot done by MTaPS for better management of future epidemics.
**Uganda Cancer Institute Act, 2016**[40]<br><br>**Uganda Heart Institute Act, 2016**[41]	■ Among the functions of the institutes is procurement of super specialized drugs and equipment<br>■ Cancer and cardiac patients are very vulnerable, hence the country must avoid substandard, fake, falsified or falsely labelled products, because the upward trend of AMR has not been matched by development of new antimicrobial agents to treat emerging resistant pathogens.	Cancer and heart institutes should be given extra support in procurement and quality control to ensure they get value for money and safety for patients.
**Guidelines for Variation of Registered Medicinal Products 2018**		NDA should build strong relationships with other regulatory agencies to effectively implement provisions of the guidelines. Programs to sensitize users/public should be supported so that they know that change in most cases is in their favor.

Policy/regulation/guideline	Identified gaps	Recommendations
**NDA. Guideline for the recall or withdrawal of a medicinal product 2017**	▪ Guideline was authorized by the Directorate of Inspectorate, not the executive secretary or board chair, as is the case with other guidelines.<br>▪ Its review date is August 21, 2020.<br>▪ NDA enforcement measures highlighted are ambiguous. It does not mention any action toward the affected population; responsibility of the importer hinges on recall and paying NDA recall costs (e.g., if animals died after using a bad product, who compensates the farmer?)	The guidelines on recall and withdrawal should be amended; review date is long overdue and gaps should be addressed
**National Health Care Waste Management Plan 2009/10-2011/12**	▪ No HCWM guidelines; National Environment Act does not make specific and detailed provisions for HCWM<br>▪ Poor segregation, handling, and disposal practices at health facilities; this includes antimicrobial agents, which poses serious health hazards to people living in the vicinity of health care institutions because expired, spoiled/broken unused capsules, tablets, syrups, ampoules, and vials are often dumped around and filter into the soil, water system, or are illicitly recycled for human and animal use<br>▪ Health care workers, patients, and communities are exposed to nosocomial infections both within health care facilities and the surrounding communities.	▪ HCWM national policy and guidelines should be developed to protect the existing antimicrobials from mismanagement<br>▪ Developed tools should be disseminated and health workers sensitized as a priority activity in the fight against AMR<br>▪ Institutions should be supported with infrastructure and capacity to handle HCWM, e.g., construction of pits, dumping sites, procurement installation and management of incinerators, etc.; this must be comprehensive for both health facilities and farms under MAAIF
**7.0: Prescription, dispensing, and use**		
**Registration of pharmaceutical premises/outlets - NDA licensing guidelines 2019**	NDA unilaterally decides distance between pharmacies as a measure to promote equitable distribution of pharmaceutical services. The guideline allows one pharmacist to supervise more than one pharmacy, which encourages non-pharmacists to dispense restricted antimicrobials without any immediate supervision from the pharmacist who might be at other premises, which may lead to irrational use, a contributing factor to AMR.	▪ Regulations should be amended to restrict the pharmacist to one pharmacy and bar any dispensing of restricted antimicrobials in his/her absence.<br>▪ The policy of equitable distribution of pharmaceutical services should be promoted in the reviewed guidelines, but the factor of population served should be upheld to decide how many pharmacies serve an area rather than absolute distance policy.
**Registration of pharmaceutical personnel**	Rampant use of unregistered drugs and unauthorized prescribers, especially in the reserve category	Effort should be made to support NDA and those in the drug supply chain (JMS/NMS/MOH/MAAIF) to avail products through correct channels to avoid falsified/substandard supplies, which lead to increased AMR
**Chapter 280 of the Pharmacy and Drugs Act**	▪ Rampant use of unregistered drugs in private pharmacies	▪ PSU should be empowered to regulate all pharmacists on ethical dispensing and rational drug use; weed out unauthorized operators/quacks

**Policy/regulation/guideline**	Identified gaps	Recommendations
	▪ Many unauthorized persons operating pharmacies are dispensing POMs without prescriptions (pharmacy initiated); AWaRe not respected	▪ PSU should ensure that training institutions relevant to their profession align AMS in their training curricula.
**Medical and Dental Practitioners Act 1998**[103]	▪ Rampant use of unregistered drugs and unauthorized prescribers especially in the reserve category<br>▪ Many doctors do not observe AWaRe in prescribing and dispensing drugs.	▪ M&DPC should be empowered to regulate all its members on ethical prescribing and rational drug use; weed out unauthorized operators/quacks<br>▪ M&DPC should ensure that the medical schools align AMS in their training curriculum.
**Nurses and Midwives Act of 2000. SI No.274 - 1**	▪ Rampant use of unauthorized personnel<br>▪ Prescribing and dispensing drugs beyond level authorized in the NDA act especially in the reserve category on the market.<br>▪ The professional members do not observe AWaRe in prescribing and dispensing drugs	▪ Nurses and Midwives Council (NMC) and Private Midwives Association (PMA) should be empowered to regulate all prescribers on ethics, rational drug use, and AMR/AMS; weed out unauthorized operators/quacks<br>▪ NMC/PMA should ensure that training institutions relevant to their profession align AMS in their training curricula.
**Allied Health Professionals Act 1996**	▪ Rampant use of unauthorized personnel<br>▪ Prescribing and dispensing drugs beyond level authorized in the NDA act, especially in the reserve category<br>▪ Professional members do not observe AWaRe in prescribing and dispensing drugs	▪ AHPC should be empowered to regulate all prescribers on ethics and rational drug use; weed out unauthorized operators/quacks<br>▪ AHPC should ensure that training institutions relevant to their profession align AMS in their training curricula
**Regulation limiting antimicrobials to POM status**	▪ Government delegates some prescribing powers to lower cadre; done "on behalf of the director general health services" which makes it legal practice<br>▪ Government has upgraded health center level 2 (HC2) to HC3, introducing senior clinical officers, highly trained degreed nurses, and a dispenser at that level to improve the qualifications of prescribers and their technical capacity[104]	▪ Government should recruit the right cadres to prescribe the controlled drugs because doctors, pharmacists, nurses, and midwives are available on the labor market.<br>▪ NDA should develop guidelines to streamline prescribing rights and amend schedules accordingly in collaboration with the relevant professional bodies; should coordinate the best way forward to protect not only the drug molecules but also patients<br>▪ Senior professionals, through their respective MTCs and MOH-PD, should be empowered to develop institutional working protocols to strengthen adherence to AWaRe and encourage research.
**UCG 2016**		Revision and update of both UCG and EMHSLU should immediately and concurrently be done.
**MTC Manual 2018**[105]		▪ Support should be rendered to health facilities countrywide to establish MTCs because a functional and active MTC has many

103 Medical and Dental Practitioners Act 1998
104 Guidelines for Designation, Establishment and Upgrading of Health Units 2011
105 Ministry of Health Medicines and Therapeutics Committee Manual 2018

**Policy/regulation/guideline**	Identified gaps	Recommendations
		benefits, which includes prevention and improved management of AMR.<br>■ The MTC manual needs to be available and disseminated to all facilities (facilitation needed), both public and private.<br>■ Measures to combat dispensing over the counter, in unlicensed drug stores, and in open vans in markets should be supported.<br>■ Programs to discourage self-medication among the population should be promoted and supported as this is one major way of abusing antibiotics and antifungals, leading to AMR.
**Consolidated Guidelines for Prevention and Treatment of HIV and AIDS in Uganda, 2020**	Guidelines do not mention challenges and mitigating factors to avoid AMR, knowing that many antimicrobial agents are used in HIV/AIDS treatment and care. The opportunity to sensitize users/prescribers on proper use of antimicrobial agents is missing in the guidelines, yet these vulnerable patients use many antimicrobial agents to manage opportunistic infections, including multidrug resistant bacteria in TB.	■ Guideline's next amendment should include AMS strategies to emphasize prevention and treatment; it has no mention of AMR, yet antimicrobial agents are prescribed frequently and widely to manage HIV/AIDS opportunistic infections.<br>■ Mention is made of monitoring co-trimoxazole for ADRs and that TB treatment should include all relevant antimicrobial agents.<br>■ The next edition should include an advocacy section on AMS/rational use of antimicrobials.
**Uganda AMR NAP 2018–2023**		■ Measures equivalent to those developed for coronavirus sensitization and sanitation have proved effective and should be adopted among the several strategies; AMU control measures should be promoted to avoid misuse, especially emphasizing AWaRe classification in prescribing and use<br>■ Political leadership must be interested in investing in research and innovation. For example, the public's recent uptake in sanitizing, either washing with soap and water or using hand sanitizers, which the community well received because of COVID-19. There is the need to study the impact this has on AMR and sustain its advantages.
**NRM Manifesto 2021-2026 "securing your future"**	The manifesto emphasizes availability, control, pilferage/drug theft and less effort on proper rational use, especially for antimicrobials. Being a policy document by the government in power, excluding strategies for AMR, is a significant oversight.	■ MOH, MAAIF and NDA should advise/ lobby on inclusion and prioritization of AMS by the ruling party because there is limited awareness on AMR among the public and policy makers, political commitment leads to financial support<br>■ One Health team should be reminded that part of its mandate is to maintain national and international political support for action.

ANIMAL, FISH, AND CROP SECTORS

Policy/regulation/guideline	Identified gaps	Recommendations
**Veterinary drug policy (2002)**	■ MAAIF has no full-fledged division of veterinary medicines and vaccines to implement the policy and give strategic policy direction on AMC, AMS, and AMR<br>■ The policy is old and does not adequately address AMS in livestock and companion animals<br>■ Lack of strategic policy statement on the regulation of antimicrobials used in aquaculture	■ MAAIF needs to establish a full-fledged division or department for veterinary medicines and vaccines to provide strategic policy guidance on antimicrobial licensing and stewardship.<br>■ DAR/MAAIF should expedite the review and/or approval of the revised veterinary medicines and devices policy (2020 draft).<br>■ The revised veterinary medicines policy should address policy gaps in regulation of antimicrobials in aquaculture
**National Agriculture Policy (2013)**	■ Lacks strategy for AMS for bactericides and fungicides used in crop protection	■ DCR/MAAIF should review the Agricultural Policy to include strategy for AMS in crop protection
**National Fisheries and Aquaculture policy (2018)**	■ Policy does not explicitly emphasize strategies for antimicrobial regulation and stewardship in the aquaculture sector	■ DFR/MAAIF should review the policy to include strategies for regulation of antibiotics and medicated aquaculture premixes.
**NDPA Cap 206: NDA Board composition in relational to its function in guiding antimicrobial regulation and stewardship**	■ Gross underrepresentation of the veterinary sector and various specialties in the composition of the board (10%) to effectively guide antimicrobial registration and AMS<br>■ No veterinary representation in NDA management in absence of directorate of veterinary medicine<br>■ At structural level, NDA does not have specific department for veterinary medicines that works closely with MAAIF on matters of veterinary medicines and antimicrobials regulation and stewardship.	■ MOH should review NDPA Cap 206 to create a separate directorate for veterinary medicines that shall have various committees whose membership is drawn from the various animal health specialties to guide AMS.<br>■ A separate directorate will permit representation of veterinary medicines in NDA management to provide guidance on matters of AMS, AMC, and AMR through the head of the directorate OR<br>■ MAAIF should implement the directive of the Cabinet (2019) to establish a separate agency to regulate veterinary medicinal products in line with AU-IBAR and EAC recommendations on AMS
**NDPA Cap 206: Essential medicines and formulary committees that guide antimicrobial registration**	■ Conflict of mandate on who should be responsible for development of EVML and NVF; although NDPA Cap 206 gives the mandate to NDA, NVDP 2002, in section 3.5.3, gives the mandate to MAAIF and UVB.<br>■ No veterinary representation on the CED<br>■ Underrepresentation of the various specialties and technical domains in the animal sector in the NFC	■ NDA needs to relinquish the responsibility of developing EVML to MAAIF in line with the veterinary drug policy and best practices.<br>■ NDA needs to ensure that veterinary specialists from ruminant, companion animals, wildlife, and aquaculture are represented in CED<br>■ Review NDPA Cap 206 to streamline representation of the various specialties on the CNF
**NDA's veterinary human resources to support antimicrobial regulation and stewardship**	■ NDA is noncompliant with provisions of the OIE terrestrial code chapter 3.4 in the absence of a specialty body responsible for veterinary medicines, which	MAAIF should establish a competent authority to comply with OIE chapter 3.4.

**Policy/regulation/guideline**	Identified gaps	Recommendations
	compromises both the quantity and quality of veterinary human resources required for effective regulation of antimicrobials.<br>▪ An out-of-court settlement between NDA and PSU that blocks veterinarians from inspecting pharmacies undermines their relevance and contributions to NDA (consent judgement of 08/12/2019 before Judge Kabiito).	MAAIF and NDA need to seek judicial review to reverse the out-of-court settlement that impedes the roles and contribution of veterinarians toward effective VMPs and antimicrobial regulation in NDA.
**Registration of veterinary antimicrobials by NDA in line with NDPA Cap 206, SI 2014 No. 24**	▪ The definitions/interpretation of key terminologies used with respect to registration of veterinary antimicrobials and other medicinal products (VMPs) are not included in SI No. 24 of 2014.<br>▪ SI. No. 24 sub-section 16-18 does not guide (discourage) registration of antimicrobial products that contain more than 3 classes of antimicrobials (risk factor for multidrug-resistant AMR).<br>▪ Presence of unlicensed and illicit antimicrobials in some districts due to weak enforcement by NDA.<br>▪ Evaluation for registration of antimicrobial focuses more on pharmaceutical quality than bioequivalence and therapeutic efficacy	▪ MOH should amend SI No.24 of 2014 to include key definitions regarding VMPs<br>▪ Review NVDP 2002 and NDPA Cap 206 SI No. 24 to address concerns over registration of multi-ingredient veterinary antimicrobial products used in food animals.<br>▪ NDA should hasten enforcement of mandatory registration of antimicrobials and other VMPs to eliminate unlicensed products sold in Uganda.<br>▪ NDA should require bioequivalence tests for product registration for generics to ensure that only quality antimicrobials are licensed
**NDPA Cap 206: Registration of veterinary vaccines**	▪ There is lack of capacity for quality assurance of animal vaccines by NDA<br>▪ Lack of post vaccination strategy and weak capacity for post vaccination monitoring	▪ MAAIF and NDA need to build laboratory capacity to assess the quality of animal vaccines before and after registration.<br>▪ MAAIF should develop post-vaccination strategy and strengthen post-vaccination monitoring in livestock and poultry.
**Licensing personnel to operate veterinary drug shops and vet pharmacy (Veterinary Surgeons Act, 1958)**	▪ Veterinary Surgeons Act (1958) does not mandate UVB to regulate para veterinary professionals.<br>▪ Veterinary Surgeon's Act is too old with lenient punishment for offenders.<br>▪ Weak enforcement of the Veterinary Surgeons Act because of limited support to the veterinary board by MAAIF.<br>▪ Unqualified persons operate veterinary drug outlets because of weakness in enforcement by NDA, thus affecting good dispensing practices for antimicrobials.	▪ MAAIF should expedite the enactment of the Veterinary and Veterinary Paraprofessional bill to give UVB a greater mandate to regulate veterinary paraprofessionals.<br>▪ The revised Veterinary and Veterinary Paraprofessional law should have prohibitive penalties to deter malpractices.<br>▪ MAAIF needs to provide financial support needed by UVB to execute its legal mandate.<br>▪ NDA needs to improve regulatory enforcement using NDPA Cap 206, SI 35 section to ensure AMS.
**NDPA Cap 206: Licensing of personnel to operate veterinary drug retail (shops)**	▪ NDA guidelines are not very specific on the qualifications of personnel that handle veterinary medicinal products at all levels of the supply chain.	▪ Amend NDPA Cap 206 to specify qualifications of prescribers of veterinary medicinal products to allow prescription ONLY by qualified veterinary professionals.

Policy/regulation/guideline	Identified gaps	Recommendations
**Licensing of drug outlets and manufacturing facilities in line with NDPA Cap 206**	■ Mandatory requirement refor a pharmacist to be a co-director when opening veterinary pharmacy, which affects equitable distribution of veterinary pharmacies in areas such as West Nile, Karamoja, Acholi, and Lango sub-regions.<br>■ Unequitable distribution of veterinary drug outlets (especially pharmacies) often creates a vacuum in the supply of antimicrobials, which is filled illegally by drug shops and irrational cross-over use of human antimicrobials in animals.	■ MOH should review NDPA Cap 206 section 14 to create the space for opening up pharmacies to the market, with pharmacists being part of the human resources required to run pharmacy outlets.<br>■ NDA should provide incentives to promote licensing of veterinary drug outlets in poorly served regions such as West Nile, Karamoja region and northern Uganda.
**Marketing authorization, licensing and Inspection of antimicrobials used in Aquaculture (NDPA Cap 206 and Fish Rules)**	■ Whereas NDA currently licenses fish health drugs, both the NDPA Cap 206 and Fish (Aquaculture) Rules do not have specific clauses regulating fish antibiotics and other drugs.<br>■ The Fish Rules do not give clear guidance on licensing individuals and premises for selling fish drugs and chemicals.<br>■ There is no specific guideline for licensing and listing of antimicrobials for fish health in MAAIF and NDA.	■ MAAIF and NDA should develop guidelines for licensing, importation, and distribution of fish antibiotics.<br>■ Revise fish rules to address the concerns above<br>■ DFR and NDA should develop guidelines for licensing antimicrobials for aquaculture and publish the list of officially registered fish health drugs as part of the veterinary drug register.
**Marketing authorization, licensing, and inspection of antimicrobials used in crop protection (ACCA)**	■ Lack of SIs for the ACCA impedes effective implementation of the law.<br>■ Lack of guidelines on restricting registration of synthetic antimicrobials (used in animal and human health) for use in crop protection	■ MAAIF needs to develop SIs for ACCA to ensure effective implementation to guarantee AMS<br>■ CPB and the ACCB should develop a guideline that restricts registration of synthetic antimicrobials used in animal and human health.
**B: Post-marketing surveillance and PV**		
**NDPA Cap 206: NDA veterinary antimicrobial post-marketing surveillance: regulation and performance**	■ Weak implementation of post-marketing surveillance by NDA, especially in Karamoja and West Nile region.<br>■ High failure rate for veterinary drugs based on NDA quality analysis report for FY 2019/2020<br>■ Antibiotics sold in drug shops contrary to the NDPA Cap 206<br>■ Unlicensed antimicrobials sold in drug outlets in hard-to-reach areas	■ NDA needs to strengthen antimicrobial post-marketing surveillance to deter illicit products from entering the market.<br>■ The high quality-failure rate of veterinary drugs, illicit sale of unlicensed veterinary drugs, and sale of antimicrobials in drug shops contrary to the NDPA Cap 206 require internal policy shift for prioritization of veterinary drug regulatory mechanisms. These may include reforms in the structure, human resources, and increasing frequency of surveillance to deter inflow of substandard veterinary medicines and antimicrobials.
**Post-marketing surveillance for animal vaccines to ensure quality of vaccines are preserved (NDPA Cap 206)**	■ Lack of legal prescribing standards for cold chain system for vaccine storage in both private and government<br>■ Drug shops store and sell animal vaccine with doubtful quality because of the lack of capacity to maintain cold chain system	■ MAAIF and NDA need to develop guidelines for good vaccine storage and distribution practices for both private sector and government vaccine stores

Policy/regulation/guideline	Identified gaps	Recommendations
		■ NDA should strengthen inspection and compliance enforcement for good vaccine storage and distribution practices
**NDA veterinary antimicrobial PV (NDPA Cap 206)**	■ The SI on PV lacks a provision for good vigilance in inspection and enforcement.<br>■ Lack of guidelines to deter extra-label use of drugs (cross-overs) between human and animals<br>■ No formal collaboration between NDA and veterinary department affects ADE reporting by vets<br>■ Lack of guidelines to deter dispensing injectable antimicrobials in small volumes to farmers by drug shops<br>■ Lack of awareness among farmers and animal health workers on importance of PV affects reporting level<br>■ No user-friendly digital system for capturing ADEs	■ NDA needs to develop and implement good vigilance practice for inspection and enforcement.<br>■ MAAIF and NDA need to develop guidelines on extra-label use of drugs.<br>■ NDA needs to collaborate with district veterinary department and MAAIF regional veterinary inspectors to support PV.<br>■ NDA needs to strengthen its post-marketing surveillance to deter aliquoting of antimicrobials in drug shops<br>■ NDA needs to improve community outreach on antimicrobial PV.<br>■ MAAIF and NDA need to develop digital system for capturing ADEs for the animal sector.
**Post-marketing surveillance and PV in aquaculture (NDPA Cap 206)**	■ At operational level, there is weak institutional linkage between NDA, DFR, and farmers in guiding appropriate regulation and use of antibiotics in fish health.<br>■ No clear PV system for aquaculture products, including antimicrobials by NDA and DFR<br>■ Limited human resource capacity in fish disease diagnosis and prudent antimicrobial prescription.<br>■ Antimicrobial consumption in fish health is not monitored.	■ MAAIF/DFR/DAR and NDA need to strengthen coordination mechanism for regulation of aquaculture drugs and antimicrobials.<br>■ MAAIF/DFS and NDA need to develop guideline for post-marketing surveillance and PV targeting the aquaculture sector.<br>■ MAAIF/DFR/DAR needs to build comprehensive capacity for fish health, including diagnostic infrastructure.<br>■ MAAIF and NDA should start to monitor AMC in the aquaculture industry.
**Post-marketing surveillance and PV in crop protection (NDPA Cap 206)**	■ Weak laboratory capacity for quality assurance of agrochemicals<br>■ Weak post-marketing surveillance, leading to infiltration of the market with falsified and substandard agrochemicals<br>■ The agricultural police are poorly funded to support enforcement.	■ MAAIF should build capacity for agrochemical quality control to allow post-marketing surveillance against falsified and substandard chemicals.<br>■ MAAIF/DCR needs to employ more inspectors to support enforcement.<br>■ MAAIF needs to allocate more resources to the agricultural police to support compliance enforcement.
**C: Supply management policies and guidelines relevant for antimicrobials**		
**Manufacturing and importation of antimicrobials and other VMPs**	■ Import of fluoroquinolones for food animal production needs regulatory guideline in line with OIE.	■ NDA and MAAIF should develop a guideline on importation and use of highly critical antimicrobials, such as fluoroquinolones.

**Policy/regulation/guideline**	Identified gaps	Recommendations
**(NDPA Cap 206)**	■ NDPA Cap 206 SI 34 part III does not provide rules for internal donations, which in most cases are given directly to communities by NGOs.<br>■ Underutilization of AMC data for AMS strategy by both NDA and MAAIF<br>■ The antimicrobial consumption data at drug outlets and farm levels are not readily available.	■ NDA and MAAIF need to develop guidelines for donation of antimicrobials to communities to avoid NGOs giving large quantities of antimicrobials directly to farmers without due consideration of their stewardship.<br>■ MAAIF and NDA should utilize the AMC data collected, in addition to existing AMR surveillance data, to develop regulatory guidelines on AMS for the various classes of antimicrobial drugs imported and used in Uganda.<br>■ MAAIF and NDA need to strengthen AMC data collection at farm level and drug outlets, respectively.
**Distribution of veterinary antimicrobials and VMPs (NDPA Cap 206)**	■ The NDA GDP guideline is due for revision; its due date was April 5, 2021.<br>■ GDP guideline does not clarify sanctions for failure to adhere to the guideline.	■ Review the NDA GDP guidelines to include penalties for distributors who supply antimicrobial products directly farmers or dump near-expiration products to retailers and farmers.<br>■ The revised GDP should include stringent penalties for noncompliance and increase regulatory inspection to ensure adherence to GDP rules.
**Sale of animal antimicrobials (NDPA Cap 206)**	■ Weak enforcement of provision of NDPA Cap 206 that requires dispensing of antimicrobials on prescription by a veterinary surgeon.<br>■ Lack of guideline for GDP specifically for animal antimicrobials in the era of AMR<br>■ Merger of schedules and drug classification for human and animal drugs against FAO recommendations	■ Strict enforcement of prescription before dispensing to promote AMS<br>■ NDA and MAAIF should develop a guideline for dispensing animal antimicrobials and ensure it's enforced.<br>■ MOH should review the NDPA to reschedule VMPs.
**NDPA Cap 206 and Fish Rules: Supply management with policies and guidelines for aquaculture antimicrobials**	Fish rules do not provide for GMP, good distribution and dispensing practices for aquaculture antibiotics and other drugs	MAAIF should develop an SI that supports implementation of GMP prior to product licensing and GDPs for licensed products.
**Supply management policies and guidelines relevant for Crop protection antimicrobials (ACCA)**	Lack of SI for enforcement of good manufacturing, distribution, and dispensing practices for crop protection antimicrobials	MAAIF should develop SIs for ACCA to enable effective enforcement of the law.
**C: Prescription, dispensing, and use**		
**Prescription of antimicrobials in the animal sector (Veterinary Surgeons Act)**	■ Unlicensed personnel prescribing antimicrobials<br>■ Guidelines for prescription of antimicrobials and other VMPs<br>■ Lack of policy on renewal of annual practicing license based on CPD points	■ MAAIF should fast-track enacting the veterinary and para-veterinary professionals bill.<br>■ Develop guidelines for prescription of veterinary antimicrobials and VMPs.

Policy/regulation/guideline	Identified gaps	Recommendations
	■ Poor funding of extension service providers to offer clinical support to farming communities	■ UVB should partner with Makerere University's COVAB to develop CPD programs for veterinarians in AMS and AMR.<br>■ MAAIF should advocate for more funding of veterinary clinical services by government to allow professional use of antimicrobials and other VMPs.
**NDPA Cap 206 and Veterinary Surgeon's Act: Dispensing of antimicrobials and VMPs in drug outlets**	■ NDPA Cap 206 does not provide specific guidelines on good antimicrobial dispensing practices in veterinary drug outlets.<br>■ Licensed veterinarians are technically well placed to dispense antimicrobials and offer technical guidance on AMU; they are considered auxiliary staff by NDPA Cap 206.<br>■ Unlicensed personnel dispensing antimicrobials in drug shops because of weak enforcement	■ NDA and MAAIF need to develop guidelines on good antimicrobial dispensing practices in veterinary drug outlets.<br>■ Review NDPA Cap to recognize veterinarians as essential staff in veterinary pharmacy<br>■ UVB and NDA needs to regularly carry out regulatory inspection of licensed veterinary drug outlets and discipline errant veterinarians or owners of drug outlets that employ unqualified persons.
**Veterinary Drug Policy 2002: SVTG for appropriate use of antimicrobials**	Uganda does not currently have a SVTG for the various animal species of interest	MAAIF should develop an SVTG for cattle, poultry, sheep and goats, swine, and fish to guide veterinary practitioners.<br><br>MAAIF should develop SVTG; companion animal guide should also be developed by MAAIF.
**NDPA Cap 206 and NVDP 2002: NVF**	■ No approved NVF since establishment of NDA in 1993.<br>■ MAAIF decries inadequate consultation by NDA in the development of the current draft NVF, thus affecting approval process.	■ MAAIF should expedite review and approval of the draft NVF.<br>■ A full-fledged veterinary medicines and devices unit or division needs to be established at MAAIF to coordinate and guide policies and coordinate with NDA.
**NDPA Cap 206: EVML**	■ The EVML 2020 is not widely disseminated.<br>■ Veterinary practitioners not adequately sensitized about the technical implication of restriction of certain antimicrobial classes in the EVML.	■ MAAIF needs to disseminate the EVML 2020 to veterinary practitioners<br>■ Sensitize veterinary practitioners and stakeholders on EVML.
**National Veterinary Service Delivery Policy 2002: Availability of laboratory diagnostic services for diagnosis of animal diseases and antibiotic sensitivity tests before prescription**	■ Very few vet labs have the capacity to carry out culture and antibiogram (mostly located in Kampala)<br>■ Available vet labs are poorly funded and offer services erratically without quality management systems.<br>■ Qualified lab technologists are not employed at local government vet labs because of budget constraints.<br>■ Lack of policy and strategy for the development of the animal sector labs<br>■ Few veterinarians actively use labs for diagnosis because the services are far from them.	■ MAAIF should develop a laboratory policy and strategy for developing animal sector labs.<br>■ Government should provide technical and financial support to capacitate existing labs to carry out bacterial culture for diagnosis and antibiogram to inform the choice of antimicrobial therapy.<br>■ MAAIF should advocate for reform of public service and local government to include the positions of laboratory technologists and technicians.<br>■ MAAIF needs to develop both policy and strategy for the animal sector labs.

Policy/regulation/guideline	Identified gaps	Recommendations
		▪ Develop district lab infrastructure and ensure both private and government veterinarians use labs for diagnosis before prescription.
**Training competent veterinary human resource by degree and diploma awarding in AMS (curricula/NCHE)**	▪ No standardized AMU and AMS module for training veterinary and veterinary paraprofessionals<br>▪ No criteria for determining minimum weight of pharmacology, toxicology, or therapeutics modules attended as a basis for licensing cadres to operate veterinary drug outlets.	▪ UVB should develop criteria for assessing weight of veterinary pharmacological content as a basis for issuance of certificate to practice in veterinary drug outlets.<br>▪ Training institutions should incorporate AMS in training curriculum, UVB should guide institutions to review their curriculum to include various aspects of AMR, AMU, and AMS prior to submission of the curriculum to NCHE for accreditation.<br>▪ NDA, MAIF, UVB, and COVAB should jointly develop standard AMU and AMS curriculum content for veterinary and veterinary paraprofessional curriculum for adoption by the various training institutions.
**Veterinary Surgeons Act: CPD in AMR/AMU/AMR**	▪ CPD is not mandatory for renewal of practicing license by UVB.<br>▪ Currently, no AMR/AMU/AMS specific CPD is offered for veterinarians in Uganda.<br>▪ Limited collaboration between UVB, COVAB and other training institutions in accelerating CPD initiatives for AMR/AMU/AMS.	▪ UVB needs to make CPD a mandatory requirement for renewal of practicing license for veterinarians and veterinary paraprofessionals.<br>▪ Strengthen collaboration between UVB, COVAB, and other training institutions in developing and implementing CPD in various areas of VMPs, including AMR.
**NDPA Cap 206 and National Environment Management Act; Disposal of veterinary pharmaceutical/ antimicrobial wastes**	▪ NDPA Cap 206 does not have a specific SI for regulation of pharmaceutical wastes.<br>▪ NDA only has a brief guide on disposal of pharmaceutical waste, which does not provide details on how farm antimicrobial wastes should be disposed appropriately.<br>▪ Weak institutional capacity for pharmaceutical waste management at district local government	▪ MOH and MAAIF should develop a specific SI for regulation of pharmaceutical (antimicrobial) wastes along the supply chain.<br>▪ NDA and MAAIF needs to develop guideline for safe disposal of antimicrobial wastes on farms.<br>▪ MAAIF and NDA need to build capacity and incentives for veterinary/pharmaceutical/ antimicrobial waste management across the country.
**High risk of antimicrobial misuse and residues in poultry and cattle products and implementation of the NAP**	▪ Lack of specific policy on use of antimicrobials in food animal products<br>▪ The residue mentoring programs targets selective food animal species, such as beef and milk.<br>▪ Widespread irrational use of multi-ingredient antimicrobial powders for poultry care<br>▪ Lack of systematic tracking and tracer of implementation of the NAP	▪ MAAIF needs to develop AMS policy specific to food animals.<br>▪ MAAIF should develop and implement comprehensive antimicrobial residue monitoring in food of animal origin.<br>▪ MAAIF and the Ministry of Local Government need to strengthen sanitary and phytosanitary measures at farm level.

Policy/regulation/guideline	Identified gaps	Recommendations
		■ Implement NAP and carry out quarterly tracer reports on progress made on optimizing AMU and AMS.
**Fish Rules: Prescription and use of antimicrobials in Aquaculture**	■ Weak diagnostic capacity for fish diseases<br>■ No STG for fish diseases<br>■ Veterinary antimicrobials are used as extra label without certification by the chief fisheries officer<br>■ Guideline for appropriate use of antimicrobials in fish farming is not widely disseminated<br>■ Infection prevention and control materials developed by MAAIF is not disseminated widely.	■ DFR/MAAIF should strengthen technical capacity for diagnosis of fish disease.<br>■ DFR/DAR-MAAIF should develop standard fish treatment guideline to assist in appropriate use of antimicrobials in management of fish bacterial diseases.<br>■ DAR/DFR-MAAIF should develop guideline for extra label use of veterinary antimicrobials in fish.<br>■ DFR/MAAIF should disseminate the guideline on appropriate use of antimicrobials in fish farming.
**Antibiotic use in wildlife**	Lack of AMS policy and guideline in captive and wild animals	■ UWA, UWEC, and MAAIF should develop guidelines for AMU in wildlife.<br>■ UWA and MAAIF need to monitor antimicrobial residues in meat given to carnivores to avoid emergence of antibiotic-resistant organisms to those animals.
**NDPA Cap 206: Acaricide and insecticide regulations and impact on AMS**	■ Absence of centralized control in registering ectoparasiticides to allow for a reservoir molecule for acaricide resistance intervention<br>■ Lack of plan for withdrawal of ectoparasiticides against which stable resistance has emerged<br>■ Lack of regulation for control of multi-ingredient ectoparasiticide formulations in an endemic acaricide resistance situation<br>■ AMS interventions exclude link between acaricide resistance and irrational use of antimicrobials.	■ DAR/MAAIF and NDA need to develop a mechanism (regulations) for centralized rotation of ectoparasiticides to allow creation of a reservoir molecule, withdrawal of resisted chemicals, and regulation of registration of coformulated acaricides in resistance areas.<br>■ AMS campaigns should target areas currently experiencing high levels of acaricide resistance due to increased prophylactic and therapeutic use of antimicrobials against tick-borne diseases

APPENDIX I: POLICIES, LAWS, GUIDELINES, AND CIRCULARS RELEVANT TO AMS IN THE HUMAN HEALTH, ANIMAL, FISH, AND CROP SECTORS

Acts of Parliament

- Animal Diseases Act Cap 38 (1964): Sets rules for animal disease control in Uganda
- National Drug Policy & Authority (NDPA) Act Cap 206 (1993): NDPA established NDA, a regulatory body responsible for regulating human and animal drugs.
- Local Government Act (1997): Provides for the establishment of authorities for local government.
- National Medicines Policy July 2015
- National Policy on Delivery of Veterinary Services (2001): Provides MAAIF with strategic vision for provision of veterinary clinical services, vector (ticks and tsetse) control, pharmaceutical supply management, animal welfare, and veterinary public health
- Pharmacy and Drugs Act (1971): Established council for PSU and Pharmacy Board to regulate the practice of pharmacy in Uganda
- The Fish Act 1951. Provides for the control of fishing; the conservation of fish; the purchase, sale, marketing, and processing of fish; and matters connected therewith.
- National Environment Act Cap 153: Established the National Environment Management Authority and regulates disposal of pharmaceutical wastes
- National Veterinary Drug Policy (2002): Provides MAAIF strategic vision and plan for VMP regulations
- Universities and Other Tertiary Institutions Act of Parliament Act, 2001: Established NCHE to assure quality and accredit training institutions and programs
- Veterinary Surgeons Act (1958): Established the Veterinary Board that regulates veterinary practitioners

Statutory instruments – regulations

- National Drug Policy & Authority (Registration) regulation 2014:29
- National Drug Policy & Authority (Conduct of Ectoparasiticides Field Trial) regulation 2014:30
- National Drug Policy & Authority (Fees) regulation 2014:31
- National Drug Policy & Authority (Conduct of Clinical Trials) regulation 2014:32
- National Drug Policy & Authority (Control of Publication & Advertisement relating to Drugs) regulation 2014:33
- National Drug Policy & Authority (Importation & Exportation of Drugs) regulation 2014:34
- National Drug Policy & Authority (Licensing) regulation 2014:35
- National Drug Policy & Authority (Certificate of Suitability of Premises) regulation 2014:36
- National Drug Policy & Authority (Pharmacovigilance) regulation 2014:37
- National Environment (Waste Management) regulation 1999: 52
- Fish Act 1951, Statutory Instrument on Fish Rules Regulation 2003:81
- Food and Drugs (Control of Quality) (Iodated Salt) regulations 1997: 59
- Food and Drugs (Food Fortification) regulation 2005:2.

Guidelines and circulars

- NDA guidelines for Introducing a Locally Manufactured New Pharmaceutical Product on the Ugandan Market (09/02/2018)
- NDA guidelines on submission of documentation for marketing authorization of an immunological product for veterinary use (25/10/2018)
- NDA guideline on veterinary pharmacovigilance in Uganda (21/12/2020)
- NDA guidelines on good manufacturing practice for medicinal products (30/05/2017)
- NDA guidelines on Submission of Documentation for Marketing
- Authorization of a Pharmaceutical Product for Human Use
- NDA Professional Guidelines 2018-Licensing Renewal of License for Pharmacies (20/12/2017)
- Addendum No.1 to the professional guideline 2018 - Licensing: Renewal of License for Pharmacies
- NDA Professional Guidelines 2018 - Licensing: Renewal of License for Pharmaceutical Manufacturing Facilities (5/10/2017)
- NDA Professional Guidelines 2018 – Licensing: Renewal and New Licenses for Class C Drug Shops
- NDA Guidelines on Good Distribution Practice for Pharmaceutical Products (05/04/2018)
- NDA Guidelines for the Recall or Withdrawal of a Medical Product (21/08/2017)
- Uganda Essential Veterinary Medicines List (2020) [MAAIF]

Professional bodies/prescribers

- Pharmacy and Drugs Act 1970
- Medical and Dental Practitioners Act 1998
- Nurses and Midwives Act. 2000. Statutory Instrument 274-1
- Allied Health Professionals Act. May 1996
- Veterinary Surgeons Act (1958)

APPENDIX II: FIGURES & GRAPHS

Figure A1. Quantity of animal antimicrobials imported (in kilograms) in 2019 (Source: NDA)

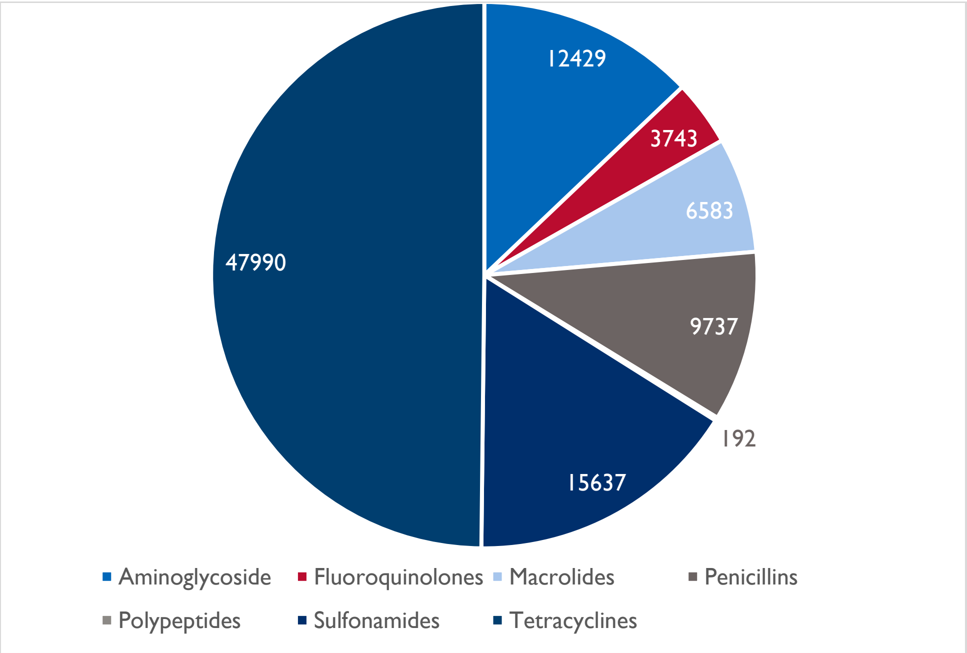

Figure A2: Number of veterinary pharmacies and drug shops licensed by NDA (Source: NDA, May 2021)

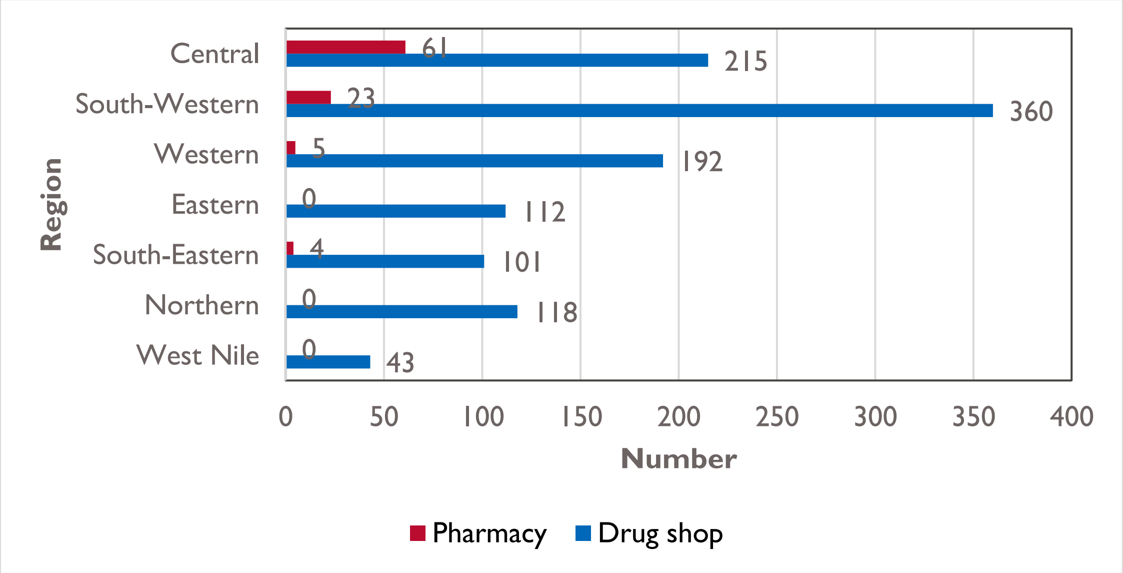

Figure A3: NDA drug laboratory quality tests performance in FY 2019/20 (Source: NDA website)

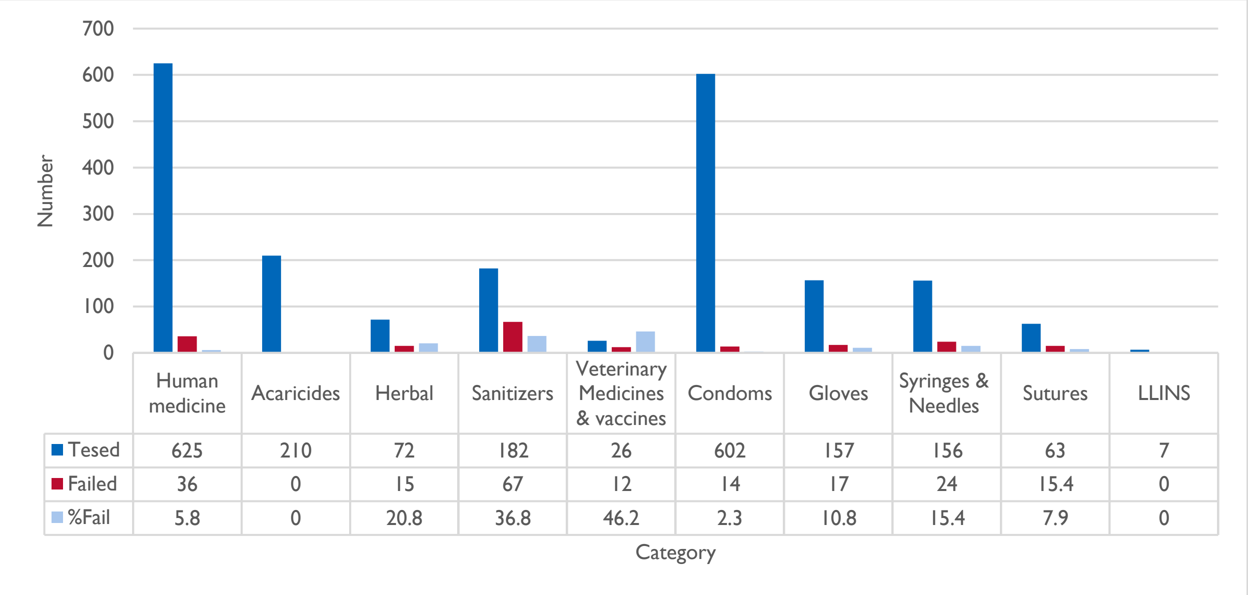

	Human medicine	Acaricides	Herbal	Sanitizers	Veterinary Medicines & vaccines	Condoms	Gloves	Syringes & Needles	Sutures	LLINS
Tesed	625	210	72	182	26	602	157	156	63	7
Failed	36	0	15	67	12	14	17	24	15.4	0
%Fail	5.8	0	20.8	36.8	46.2	2.3	10.8	15.4	7.9	0

Figure A5. Level of engagement of veterinary stakeholders by NDA in FY 2019/2020 compared to other engagements (Source: NDA website)

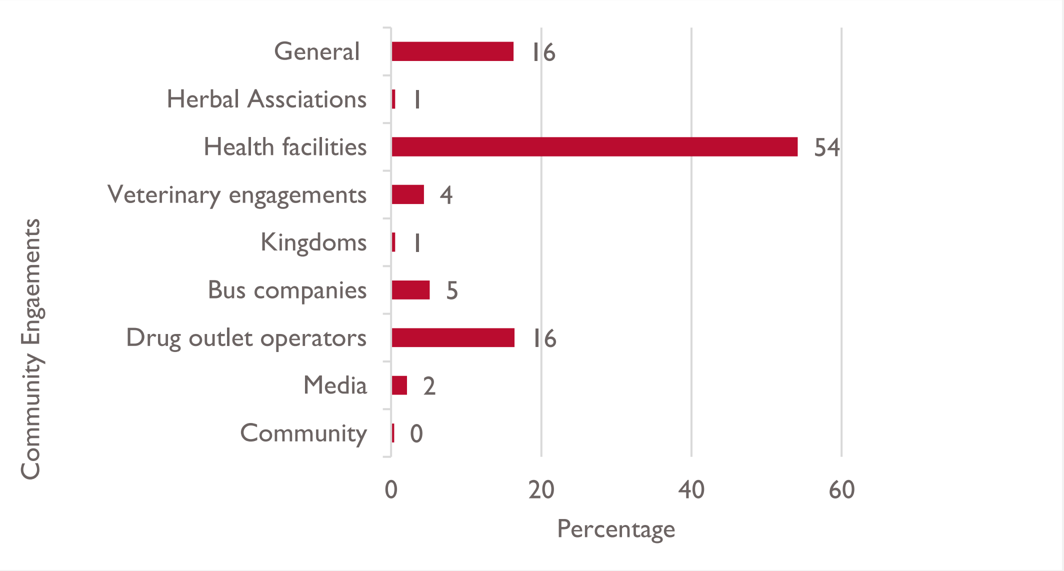

ANNEX I: LIST OF KEY RESPONDENTS (HUMAN HEALTH)

Name	Organization/designation
**Dr. Neville Oteba**	Commissioner of Health Services Pharmaceuticals & Natural Medicine Ministry of Health
**Dr. Sebisubi Fred**	Assistant Commissioner Health Services, Quality Assurance, MOH
**Dr. David Nahamya**	Executive Secretary, NDA Board
**Dr. Mwesigwa Dennis**	Director, Imports–Exports, NDA
**Mr. Fred Sekyana**	Public Relations Manager, NDA
**Dr. Obua Thomus**	Senior Pharmacist, MOH Supply Chain
**Dr. Mildad Baguma**	Executive Director, JMS
**Dr. Higenyi Emmanuel**	Director Technical Services, JMS
**Dr. Okiror**	Pharmacist, Rene Industries Ltd
**Dr. Martha Ajolong**	Principal Pharmacist, MOH
**Mr. Pater Ssali**	Quality Assurance Officer, NDA
**Dr. Tusubira Evan**	Former Regulatory Officer Clinical Trials, NDA
**Dr. Julius Mayengo**	Regulatory Officer - NDA
**Dr. Ismail Ntale**	Regulatory Officer - NDA
**Dr. Robin Kajekye**	Regulatory Officer - NDA
**Dr. Allan Ssemwanga**	Regulatory Officer - NDA
**Dr. Moses Kamabale**	General Manager, NMS
**Mr. Sekamatte Musa**	MOH-NAP, One Health
**Dr. Jaenne Muhindo**	Head Veterinary Drugs, NDA
**Dr. Henry Kajumbula**	MOH, One Health
**Dr. Sheilah Nabukeera**	Procurement Officer, NMS
**Dr. Lamwaka Alice V**	Gulu University
**Mr. Anguyo Dralega**	Head Schools of Allied Health Professionals, Makerere University
**Dr. Annet Kutesa**	Head, Department of Dentistry, Makerere University
**Dr. Mugerwa Ibrahimm**	NHLDS Ministry of Health
**Dr. Kalyango Peter**	CEO, House and McGeorge (private pharmacy)
**Dr. Francis Otim Etura**	Technical Director Phillips/Star Pharmaceuticals Ltd
**Dr. Patience Muwanguzi**	Head, School of Nursing, Makerere University
**Dr. Winnie Nambatya**	Makerere School of Pharmacy/Mulago Hospital
**Dr. Fadhiru Pakoyo Kamba**	Head, School of Pharmacy, Makerere University
**Dr. Tapan Kumar Sarkar**	Production Pharmacist, Kampala Pharmaceutical Industries Ltd
**Dr. Meera Vadodaria**	Rene Industries Ltd
**Dr. Parminder Singh**	Chairman, Uganda Pharmaceutical Manufacturers Association
**Dr. Pamela Achii**	President, PSU
**Prof. Richard Odoi Adome**	Professor, School of Pharmacy Makerere University

**Prof. Ogwang Patrick Engeu**	Head, School of Pharmacy, Mbarara University of Science and Technology
**Dr. Marion Murungi**	Senior Technical Advisor, USAID MTaPS
**Dr. JP Waswa**	Technical Advisor, USAID MTaPS
**Dr. Reuben Kiggundu**	Country Project Director, USAID MTaPS

ANNEX II: LIST OF KEY INFORMANTS FROM THE ANIMAL, FISHERIES AND CROP SECTOR INTERVIEWED

Name	Designation	Organization
Dr. Rose Ann Ademun	Commissioner Animal Health	MAAIF
Dr. Emmanuel Isingoma	Veterinary Inspector	MAAIF
Mr. Byantwale Tibeijuka Stephen	Commissioner Crop Protection	MAAIF
Ms. Ephrance Tumubine	Assistant Commissioner Crop Protection	MAAIF
Mr. Alex Twebaze	Assistant Commissioner	MAAIF
Dr. Ben Ssenkera	Senior Veterinary Officer	MAAIF
Dr. Stella Atim	Head NADDEC	MAAIF
Mr. Emmanuel Apiku	CEO of Apiviva Agrotechno Consultant and Supplies Ltd	Moyo
Dr. John Walakira	Fish Expert and Director Abi Zardi	NARO
Dr. Jaenne Muhindo	Head Veterinary Drugs, NDA	NDA
Dr. Vincent Kayizi	Regulatory Officer	NDA
Dr. Edward Sekawoja	Regulatory Officer	NDA
Dr. William Tamale	Head Regional Offices	NDA
Dr. Ponsiano Samuel Wamala	Fish Bacterial Researcher	COVAB
Dr. Benedicto Byamukama	Assistant Lecturer/Pharmacology	COVAB
Denis Kitosi Kabasa	Veterinary Officer	Kiboga
Dr. John Bosco Tingira	Principal Veterinary Officer	Kiboga
Dr. Florance Kasirye	Registrar	UVB
Dr. Dominic Lali	Board Member	UVB
Dr. Nicholas Kauta	Former Director Animal Resources	Consultant
Dr. Isaac Kikozza	Importer/Distributor	Vet Center Pharmacy
Dr. Brian Arinaitwe	Country Lead, ALPHA	Zoetis
Dr. Gerald Nizeyimana	Animal Health Expert	FAO
Dr. Nguma Willy	District Veterinary Officer	Arua district
Dr. David Mugabi	District Veterinary Officer	Kayunga district
Mrs. Joyce Ikwaput Nyeko	Commissioner Fisheries	MAAIF
Dr. James Watuwa	Zoo Wildlife Veterinarian	UWEC
Dr. Deo Ndumu	Assistant Commissioner & Disease Control & Chair AMR TWC-MAAIF	DAR, MAAIF
Dr. Emmanuel Isingoma	Senior Veterinary Officer	DAR, MAAIF
Dr. Merab Acham	Focal Person AMR/Senior Veterinary Officer	DAR, MAAIF
Dr. Micheal Kimanga	Senior Vet Inspector	DAR, MAAIF
Dr. Christine Nabaale	Vet Inspector	DAR, MAAIF
Dr. Isreal Magazei	Vet Inspector	DAR, MAAIF
Dr. Yvette Ssebunya	Vet Inspector	DAR, MAAIF
Dr. Okuyo Bosco	Principal Vet Inspector	DAR, MAAIF
Dr. Drake Ayebare	Veterinary Inspector	DAR, MAAIF
Dr. Freddy E. Kitutu	Senior Lecturer	Department of Pharmacy, CHS-MAK
Dr. John Kateregga	Lecturer	COVAB-MAK
Dr. Samuel George Okech	Lecturer	COVAB-MAK
Dr. Benedicto Byamukama	Lecturer	COVAB-MAK
Dr. Dan Kasibule	President	Uganda Veterinary Association (UVA)
Dr. Fluggy Kandox Mukasa	Member	UVA
Dr. Hanington Katumba	General Secretary	VEDAS Net
Dr. Joseph Byaruhanga	Director	VEDAS Net
