# Supplementary material for: Landscape Analysis of Existing Policies, Laws and Regulations on Antimicrobial Stewardship in 10 Low- and Middle-Income Countries
Source: Antibiotics (Basel). 2026 Aug 10;15(8):770. doi: 10.3390/antibiotics15080770 (PMC13510074; doi:10.3390/antibiotics15080770)
Supplement: Supplementary file 1 [file antibiotics-15-00770-s001.zip › S1 Baseline AMS_regulation status per country JEEs.pdf]

Supplementary material S1:

Table S1: Status of AMS, including AMS-related regulatory measures at baseline Joint External Evaluations (JEE) in 10 partner countries

Country	Date of baseline JEE	Baseline JEE score* (P.3.4 AMS)	Status of AMS-related regulatory measures at baseline JEE (as stated in the JEE baseline report)**	
			**Strengths/best practices**	**Areas that need strengthening and challenges**
Bangladesh [1]	March 2016	2	In the livestock sector, including animal and aquaculture, the use of growth promoters was prohibited. Additionally, efforts were underway to develop standard treatment guidelines (STG) for infectious diseases and an antibiotic policy. Furthermore, strict enforcement of good manufacturing practices for antibiotic production was being implemented.	Rampant self-medication and over-the-counter use of antimicrobials; questionable quality of locally-manufactured drugs; use of growth promoters in poultry and fish production; and weak enforcement of policies in the animal sector.
Burkina Faso [2]	December 2017	1	Some antimicrobial market control measures, such as inspections, were implemented. Regulatory documents related to the approval of medicines for human and animal health were in existence. Additionally, a system was in place for the registration and re-registration of antimicrobials.	Antimicrobial control systems not sufficiently operational
Cameroon [3]	September 2017	1	There was a law in place regarding the organization and exercise of the pharmacy profession, along with implementing plans. Additionally, there was a law regulating veterinary pharmacy.	Raising awareness among health professionals and populations on the judicious use of antimicrobials in human, animal, and environmental health
Côte d'Ivoire [4]	December 2016	1	Coordination mechanism for AMS activities was operational at national level including antimicrobial use and consumption (AMU/C) surveillance.	National plan for AMS activities

Mali [5]	June 2017	1	Guidelines for antibiotic therapy (2015) and AMU/C surveillance existed	National plan for AMS; development and application of provisions regulating antibiotic prescriptions in human and animal health; and development of national guidelines on the proper use of antibiotics in human health and Animal health and agriculture.
Mozambique [6]	April 2016	1	Drug laws regulating prescriptions and sales of antibiotics existed. Furthermore, the National Drug Formulary guided health professionals on prescribing all medicines.	Inconsistent and weak implementation and enforcement of existing drug law resulting in self-medication and uncontrolled community use of antimicrobials; inadequate laws regulating use of antimicrobials in the animal sector (especially poultry); and overuse of antibiotics for growth promotion.
Nigeria [7]	June 2017	2	There was a legal requirement for prescription-only antimicrobial use for human health.	Inadequate enforcement of prescription-only antimicrobial use in human; regulation of antimicrobial use in the animal sector
Senegal [8]	November 2016	1	Not presented during the external evaluation	National plan for AMS activities; apply regulations on prescribing antimicrobial agents; monitoring quality and pharmacovigilance of antimicrobials; streamline the illicit antimicrobial market; strengthen regulations on AMU/C.
Tanzania [9]	February 2016	1	National regulatory authority showed interest in AMR and AMU/C	Weaker regulations to control AMU/C in the animal health sector; and need to streamline and roles and responsibilities of the professional councils in control of AMU/C

Uganda [10]	June 2017	3	Policy documents to support AMS regulation including the national AMR action plan (2017-2022); national drugs policy (2015); Uganda clinical guidelines (2016); among others.	Inadequate enforcement of regulations on AMS

** Based on the 2016 JEE tool (JEE-1)*[11]
*** There was no associated IHR benchmarks tool when these baseline JEEs were conducted*

References

[1] World Health Organization (WHO), "World Health Organization (WHO). Joint external evaluation of IHR core capacities of the People's Republic of Bangladesh," 2016, *WHO, Geneva*. [Online]. Available: https://extranet.who.int/sph/sites/default/files/document-library/document/JEE Report Bangladesh 2016.pdf (accessed December 17, 2024)

[2] World Health Organization (WHO), "Évaluation externe conjointe des principales capacités RSI du Burkina Faso," 2017, *WHO, Geneva*. [Online]. Available: https://iris.who.int/bitstream/handle/10665/272453/WHO-WHE-CPI-REP-2018.12-fre.pdf?sequence=1 (accessed January 7, 2025)

[3] World Health Organization (WHO), "Évaluation externe conjointe des principales capacités RSI de la République du Cameroun," 2017, *WHO, Geneva*. [Online]. Available: https://iris.who.int/bitstream/handle/10665/259676/WHO-WHE-CPI-REP-2017.60-fre.pdf?sequence=1 (accessed January 7, 2025)

[4] World Health Organization (WHO), "Joint external evaluation of IHR core capacities of the Republic of Côte d'Ivoire," 2017, *WHO, Geneva*. [Online]. Available: https://extranet.who.int/sph/sites/default/files/document-library/document/JEE Report Cote D%27ivoire 2016.pdf (accessed January 7, 2025)

[5] World Health Organization (WHO), "Évaluation externe conjointe des principales capacités RSI de la République du Mali," 2017, *WHO, Geneva*. [Online]. Available: https://iris.who.int/bitstream/handle/10665/259455/WHO-WHE-CPI-REP-2017.58-fre.pdf?sequence=1 (accessed January 7, 2025)

[6] World Health Organization (WHO), "Joint External Evaluation of IHR Core Capacities of Republic of Mozambique," 2017, *WHO, Geneva*. [Online]. Available: https://extranet.who.int/sph/sites/default/files/document-library/document/JEE Report Mozambique 2016.pdf (accessed August 7, 2025)

[7] World Health Organization (WHO), "Joint External Evaluation of IHR Core Capacities of the Federal Republic of Nigeria," 2017, *WHO, Geneva*. [Online]. Available: https://iris.who.int/bitstream/handle/10665/259382/WHO-WHE-CPI-REP-2017.46-eng.pdf?sequence=1 (accessed January 7, 2025)

[8] World Health Organization (WHO), “Joint external evaluation of IHR core capacities of the Republic of Senegal,” 2017, *WHO, Geneva*. [Online]. Available: https://iris.who.int/bitstream/handle/10665/259849/WHO-WHE-CPI-REP-2017.31-eng.pdf?sequence=1 (accessed January 7, 2025)

[9] World Health Organization (WHO), “Joint external evaluation of IHR core capacities of the United Republic of Tanzania,” 2017, *WHO, Geneva*. [Online]. Available: https://iris.who.int/bitstream/handle/10665/254510/WHO-WHE-CPI-2017.7-eng.pdf?sequence=1 (accessed January 7, 2025)

[10] World Health Organization (WHO), “Joint external evaluation of IHR core capacities of the Republic of Uganda,” 2017, *WHO, Geneva*. [Online]. Available: https://extranet.who.int/sph/sites/default/files/document-library/document/JEE Report Uganda 2017.pdf (accessed January 7, 2025)

[11] World Health Organization (WHO), “Joint external evaluation tool: International Health Regulations (2005) - first edition,” 2016, *WHO, Geneva*. [Online]. Available: https://iris.who.int/bitstream/handle/10665/204368/9789241510172_eng.pdf?sequence=1 (accessed January 7, 2025)
