# Supplementary material for: Landscape Analysis of Existing Policies, Laws and Regulations on Antimicrobial Stewardship in 10 Low- and Middle-Income Countries
Source: Antibiotics (Basel). 2026 Aug 10;15(8):770. doi: 10.3390/antibiotics15080770 (PMC13510074; doi:10.3390/antibiotics15080770)
Supplement: Supplementary file 1 [file antibiotics-15-00770-s001.zip › S2 Assessment tool.pdf]

Supplementary material S2: Assessment tool

DETAILED GUIDING QUESTIONS for RAPID SITUATIONAL ASSESSMENT[1]

The following guiding questions provide some details on what to look for as part of both desktop assessment and through stakeholder interviews. The guiding questions are listed by topical areas. Instruction: For each question, mark whether a "Yes" or a "No" and provide notes with evidence linked to key documents – i.e. policies, legislation, regulations or guidelines – or data where relevant

<u>National regulations, legislation or policies</u>

1. Is there a national medicines policy that offers guidance on pharmaceutical management and regulation?
   Are there other policies that support the regulation of medicines with specific focus to antimicrobials?

2. Is there legislation/ regulation requiring registration of medicines used in the country? If yes, what is the level of enforcement?

3. Is there legislation/ regulation requiring registration of pharmaceutical premises/outlets including pharmacies? If yes, who is responsible for enforcement and what is the level of enforcement?

4. Is there a regulation requiring registration of pharmaceutical personnel by the Practitioners Registration Authority? If yes, what is the level of enforcement?

5. How many antimicrobial products are registered (including all branded antimicrobials)?

6. How many antimicrobial agents (unique chemical entities, not counting the different brands) are registered? How many of these are listed on the WHO model list of essential medicines?

7. Does the national medicines regulatory authority have tools for quality control/quality assurance and registration of antimicrobials in place?

8. Are pharmacies routinely being inspected and monitored to ensure compliance to laws and regulations with enforcement mechanisms in place?

9. Are any data available on the level of enforcement of the regulations? Are any data available on the impact of the regulations?

10. Is there a system of monitoring antimicrobial sales throughout the country? if yes what guides the system?

11. Are there official regulations on import, export, production, distribution, storage and use of finished antimicrobial products and their corresponding active pharmaceutical ingredients?

12. Are there any regulations and guidelines to regulate the promotional activities of pharmaceutical companies? (If yes, ask the follow-up questions listed below starting with question 13a. If no, go to the next question.)

---

[1] USAID SIAPS. 2017. Building Coalitions for Containing Antimicrobial Resistance: A Guide. Management Sciences for Health. http:/apps.who.int/medicinedocs/en/m/abstract/Js23293en/

13a. What regulations are in place?
13b. Are any data available on the level of enforcement of the regulations?
13c. Are any data available on the impact of the regulations?

13. Is there a regulation limiting antimicrobials to prescription-only medicines status? (If yes, go to question 14a, If no, go to the next question.)

14a. Are there exceptions to the regulation?
14b. What processes have been adopted to oversee enforcement of this regulation?
14c. Are there any data on the level of enforcement of this policy?

<u>Medicine Use and AMR-related Policies/Regulation</u>

Standard Treatment Guidelines (STGs)

14. Is there a policy/recommendation guiding the use of STGs? (Try to gather information for all levels of prescribing and for all STGs that exist in the country.)

15. What policies/recommendations are in place (e.g., mandatory or voluntary adherence to STGs in a hospital setup, availability of those medicines recommended in the STG in the hospital pharmacy)?

16. Do STGs exist for the management of infectious diseases? (Yes/No. If yes, go to the STG follow-up questions listed below starting at 17.A. If no, go to questions about EMLs).

17.A What was the process of their development?
17.B How often are they revised? When were they last revised?
17.C Do STGs exist for different levels of health care practice?
17.D Are prescribers (at different levels) trained on the use of and adherence to the STGs? If yes, what kinds of prescribers are trained and how often?
17.E Are health students (medical, pharmacy, nursing, community health workers) trained on the importance of STGs?
17.F Has the availability of STGs in health facilities been evaluated?

17. Is there a mechanism to monitor adherence to STGs? If AWaRE categorization is in force, is this adhered to?

18. Are there any data on the level of prescribing adherence to STGs in the public and private sectors?

19. Is there guidance on prescription of antimicrobials including antibiotics? what is its basis; law, policy, regulations, guidelines; Is WHO's AWaRE classification of antimicrobials utilized? If yes please provide the basis; how often is it reviewed (If yes, ask the follow-up questions listed below starting with question 20.A. If no, go to the next question.)

20. A What is the guidance on classification of antimicrobials and level of use, authorization for prescribers? Are certain antimicrobials defined and kept as "reserve" agents (e.g., some antibiotics reserved for treating only certain diseases or restricted to be prescribed only by a certain category or level of prescribers)?
20.B Have levels of antimicrobial prescribing authority been defined?
20.C Which levels of prescribers can prescribe antimicrobials? (Gather information on which antimicrobials nurses can prescribe [if authorized], which clinical officers can prescribe, and which medical officers can prescribe.)
20.D Are any new policies being developed to allow prescribing rights to a wider group of health professionals?
20.E Are there any other regulations on prescribing of antimicrobials?

20.F What is the level of implementation of these policies?
20.G Are any data available on the impact of these policies?

20. Is there a national adverse drug reaction monitoring (pharmacovigilance) system? If yes, describe its activities over the past 12 months. If no, is there any plan to start such a system?

21. Is there a national independent drug information service? If yes, describe its activities over the past 12 months, including the number of enquiries answered. If no, is there any plan to start such a service?

22. What percentage of medicines (in terms of monetary value) used in the country is contributed by the private sector and what percentage by the public sector? What proportion of this is for antimicrobials in each sector?

23. What percentage of medicines used in the country is manufactured locally? What percentage of antimicrobials used in the country is manufactured locally in proportion to those imported?

EMLs

24. Is there an EML that includes antimicrobials (antimicrobials include antibiotics, antifungals, antivirals, and antiprotozoals)? (Yes/No. If yes, go to the EML follow-up questions listed below starting at 25.A. If no, go to any additional questions.)

25.A What was the process of selecting the antimicrobials included in the EML?
25.B How often is the EML revised? When was it last revised?
25.C Are the antimicrobials listed and applied according to the levels of health care delivery?
25.D Are the antimicrobial medicines included in the EML consistent with those in the STG for infectious diseases?
25.E Are health care providers trained on the concept of EMLs and rational medicine use?
25.F Are students of medical, pharmacy, nursing, and other paramedic courses trained on the concept of EMLs?
25.G Are there any data on the extent of availability of EML in health facilities?

25.H Any there any survey data on the availability of drugs (including antimicrobials) included in the EML in health facilities?

25. Are pharmacists legally allowed to dispense certain antimicrobials without a prescription or for specific indications?

<u>Supply Management Policies</u>

For the purposes of this assessment, suggested questions regarding supply chain management focus primarily on policies or guidelines related to pharmaceutical product quality and the utilization of the EML.

26. What quality assurance mechanisms are in place in the public and private sectors to ensure the quality of antimicrobials marketed in the country?

27. How many public and private laboratories are capable of testing antimicrobial quality?

28. Which of the following quality assurance mechanisms are in place to ensure antimicrobial quality?

- ☐ Prequalification of suppliers
- ☐ Supplier submits registration form
- ☐ Physical inspection of drug samples
- ☐ Laboratory analysis of drug samples
- ☐ Specific drug reports requested, such as bioavailability
- ☐ Informal information gathered from other procurement programs
- ☐ Tender/order documents specify which pharmacopeia standards are acceptable

- ☐ Good Manufacturing Practice certification
- ☐ Monitoring of quality of the marketed product
- ☐ Inspection at the point of importation
- ☐ Inspection at retail/wholesale pharmacies
- ☐ Lab testing of the samples obtained from drug outlets
- ☐ Supplier performance M&E
- ☐ Other (describe)

29. To what extent are the above mechanisms/regulations enforced?

30. Are there any data on the proportion of antimicrobials that were substandard in the last two years?

31. Are documented reports of falsified antimicrobials available?

32. Is there a policy of using the EML antimicrobial list for procurement of antimicrobials? (Yes/No. If yes, ask the follow-up questions listed below starting at 33.A. If no, go to any additional questions)

    33.A Does this policy exist in the public sector?
    33.B Does this policy exist in the private sector?
    33.C Are there any data on the level of adherence to the policies?

33. Is there data on antimicrobial consumption by value and volume at country level? Or in a national hospital level?

<u>Appropriate Use by Prescribers, Dispensers, and Patients</u>

34. Do any studies/reports exist on the level of appropriateness of antimicrobial prescribing and dispensing? Record key findings of all relevant documents that are available.

35. Have public education campaigns on appropriate antimicrobial/antibiotic use and drug resistance been carried out in the last two years? (Yes/No. If yes, ask the follow-up questions listed below starting with 36.A. If no, go to the next question.)

    36.A What kind of campaigns have been conducted and how frequently?
    36.B Who conducted these campaigns?
    36.C Are there education campaigns relating to infection prevention (e.g., hand hygiene, food hygiene, vector control, immunizations)?
    36.D Are there education campaigns relating to appropriate use of antimicrobials/antibiotics and sensitization of AMR issues?
    36.E Are there any data on the impact of these measures?

36. Are there any data on the use of antimicrobials by the public (including level of appropriateness of use)?

37. Has the country participated in WHO World Antibiotic Awareness Week or other world/regional AMR awareness campaigns? If yes, briefly describe participation/activities.
