# Supplementary material for: Landscape Analysis of Existing Policies, Laws and Regulations on Antimicrobial Stewardship in 10 Low- and Middle-Income Countries
Source: Antibiotics (Basel). 2026 Aug 10;15(8):770. doi: 10.3390/antibiotics15080770 (PMC13510074; doi:10.3390/antibiotics15080770)
Supplement: Supplementary file 1 [file antibiotics-15-00770-s001.zip › S4 SCM regulations.pdf]

Supplementary material S4: SCM regulations

S4A: Existing regulation on supply chain management (SCM) in human health and animal health sectors in the ten surveyed countries

		Bangladesh		Burkina Faso		Cameroon		Cote d'Ivoire		Mali		Mozambique		Nigeria		Senegal		Tanzania		Uganda	
		Human	Animal	Human	Animal	Human	Animal	Human	Animal	Human	Animal	Human	Animal	Human	Animal	Human	Animal	Human	Animal	Human	Animal
SCM	Supply chain management policy/strategy	1	1	1	1	1	1	1	1	1	1	1	1	1	1	1	1	1	1	1	1
	Government-owned central suppliers	1	0	1	0	1	0	1	0	1	0	1	0	1	1	1	0	1	0	1	0
	Price control policy/measures	0	0	0	0	0	0	0	0	0	0	1	0	0	0	0	0	0	0	0	0
	Regulation of promotional activities	1	1	1	1	0	0	0	0	1	1	0	1	1	1	0	0	0	0	1	1
	Informal markets	1	1	1	1	1	1	1	1	1	1	1	1	1	1	1	1	1	1	1	1

1 = yes
0 = no

S4B: Existing regulation/guidelines on supply chain management and antimicrobial prescribing, dispensing, and use in the human health and animal health sectors in the ten surveyed countries

		Bangladesh		Burkina Faso		Cameroon		Cote d'Ivoire		Mali		Mozambique		Nigeria		Senegal		Tanzania		Uganda	
		Human	Animal	Human	Animal	Human	Animal	Human	Animal	Human	Animal	Human	Animal	Human	Animal	Human	Animal	Human	Animal	Human	Animal
Antimicrobial prescribing, dispensing, and use	National AMS governance body	0	0	1	1	0	0	1	1	1	1	1	1	1	1	1	0	1	1	1	1
	National AMR plan/strategy	1	1	1	1	1	1	1	1	1	1	1	1	1	1	1	1	1	1	1	1
	National AMS plan/strategy	1	0	0	0	0	1	1	1	1	0	0	0	0	0	0	0	0	0	0	0
	National AMC/U surveillance guidelines	0	1	0	1	0	0	0	0	0	0	0	0	0	0	0	0	1	1	1	1
	National AMS guidelines	1	1	0	0	0	0	0	0	0	0	0	0	1	0	1	1	1	1	0	1
	National essential medicines list	1	1	1	0	1	0	1	1	1	1	1	1	1	1	1	0	1	0	1	1
	National medicines formulary	1	1	1	0	0	0	0	0	0	0	1	1	1	1	0	0	0	0	0	0
	AWaRe categorization of antibiotics	1	0	0	0	0	0	0	0	1	0	0	0	0	0	0	0	1	0	0	0
	Standard treatment guidelines	1	0	1	0	0	0	1	0	1	0	0	0	1	0	0	0	1	1	1	0
	Regulation on use of FDC antimicrobials	1	0	0	0	0	0	0	0	0	0	0	0	0	0	0	0	0	0	0	0
	Legalized prescribing	1	1	1	1	1	1	1	1	1	1	1	0	1	1	1	1	1	1	1	1
	Legalized dispensing	1	1	1	1	1	1	1	1	1	1	1	0	1	1	1	1	1	1	1	1
	Pre-service AMS/AMR training curriculum	0	0	0	0	0	0	0	0	1	0	0	0	0	0	0	0	0	0	0	0
	Re-licensure linked CPD	0	1	0	0	0	0	0	0	0	0	0	0	0	0	0	0	0	0	1	0
	Policy on Hospital DTCs	1	0	0	0	1	0	1	0	1	0	1	0	0	0	0	0	1	0	1	0
	Regulation AMU for growth promotion	0	1	0	0	0	0	0	0	0	0	0	1	0	0	0	1	0	1	0	0

1 = yes
0 = no
